# Supplementary material for: Use of community engagement interventions to improve child immunisation in low-income and middle-income countries: a systematic review and meta-analysis
Source: BMJ Open. 2022 Nov 8;12(11):e061568. doi: 10.1136/bmjopen-2022-061568 (PMC9644342; doi:10.1136/bmjopen-2022-061568)
Supplement: Author's manuscript [file bmjopen-2022-061568.draft_revisions.pdf]

BMJ Open is committed to open peer review. As part of this commitment we make the peer review history of every article we publish publicly available.

When an article is published we post the peer reviewers' comments and the authors' responses online. We also post the versions of the paper that were used during peer review. These are the versions that the peer review comments apply to.

The versions of the paper that follow are the versions that were submitted during the peer review process. They are not the versions of record or the final published versions. They should not be cited or distributed as the published version of this manuscript.

BMJ Open is an open access journal and the full, final, typeset and author-corrected version of record of the manuscript is available on our site with no access controls, subscription charges or pay-per-view fees (http://bmjopen.bmj.com).

If you have any questions on BMJ Open's open peer review process please email info.bmjopen@bmj.com

BMJ Open

Use of community engagement interventions to improve child immunisation in low- and middle-income countries: a systematic review and meta-analysis

Journal:	*BMJ Open*
Manuscript ID	bmjopen-2022-061568
Article Type:	Original research
Date Submitted by the Author:	09-Feb-2022

Complete List of Authors:	Jain, Monica; International Initiative for Impact Evaluation Dehli, Evaluation Office<br>Shisler, Shannon; International Initiative for Impact Evaluation<br>Lane, Charlotte ; International Initiative for Impact Evaluation<br>Bagai, Avantika; International Initiative for Impact Evaluation, Evaluation Office<br>Brown, Elizabeth; University of California Berkeley, Center for Effective Global Action<br>Engelbert, Mark; International Initiative for Impact Evaluation, Synthesis and Reviews

Keywords:	Public health < INFECTIOUS DISEASES, Community child health < PAEDIATRICS, PUBLIC HEALTH

SCHOLARONE™
Manuscripts

I, the Submitting Author has the right to grant and does grant on behalf of all authors of the Work (as defined in the below author licence), an exclusive licence and/or a non-exclusive licence for contributions from authors who are: i) UK Crown employees; ii) where BMJ has agreed a CC-BY licence shall apply, and/or iii) in accordance with the terms applicable for US Federal Government officers or employees acting as part of their official duties; on a worldwide, perpetual, irrevocable, royalty-free basis to BMJ Publishing Group Ltd ("BMJ") its licensees and where the relevant Journal is co-owned by BMJ to the co-owners of the Journal, to publish the Work in this journal and any other BMJ products and to exploit all rights, as set out in our licence.

The Submitting Author accepts and understands that any supply made under these terms is made by BMJ to the Submitting Author unless you are acting as an employee on behalf of your employer or a postgraduate student of an affiliated institution which is paying any applicable article publishing charge ("APC") for Open Access articles. Where the Submitting Author wishes to make the Work available on an Open Access basis (and intends to pay the relevant APC), the terms of reuse of such Open Access shall be governed by a Creative Commons licence – details of these licences and which Creative Commons licence will apply to this Work are set out in our licence referred to above.

Other than as permitted in any relevant BMJ Author's Self Archiving Policies, I confirm this Work has not been accepted for publication elsewhere, is not being considered for publication elsewhere and does not duplicate material already published. I confirm all authors consent to publication of this Work and authorise the granting of this licence.

For peer review only

Use of community engagement interventions to improve child immunisation in low- and middle-income countries: a systematic review and meta-analysis

Monica Jain, Shannon Shisler, Charlotte Lane, Avantika Bagai, Elizabeth Brown, Mark Engelbert

International Initiative for Impact Evaluation (Monica Jain PhD, Shannon Shisler PhD, Charlotte Lane PhD, Avantika Bagai M.Sc and Mark Engelbert PhD) and **Center for Effective Global Action, University of Berkeley, California, USA** (Elizabeth Brown PhD)

Correspondence to:

Dr. Monica Jain

Evaluation Office, International Initiative for Impact Evaluation (3ie), New Delhi, 110017, India

Email: mjain@3ieimpact.org

Keywords:

Immunization

Immunization Programs

Mass Vaccination

Poverty Areas

Public Health Administration

Word count:

Body: 4,474

Abstract: 299

For peer review only

Abstract

Objective

To support evidence informed decision making, we systematically examine the effectiveness and cost-effectiveness of community engagement interventions on routine childhood immunisation outcomes in low and middle-income countries (L&MICs) and identify contextual, design, and implementation features associated with effectiveness.

Methods

In May 2019 and May 2020, we searched academic and grey literature databases and websites for experimental and quasi-experimental impact evaluations of community engagement interventions considering outcomes related to routine child immunisation in L&MICs. Interventions were mostly compared with the status quo. No language, publication type, or date restrictions were imposed. We extracted summary data from published reports and appraised quantitative risk of bias in duplicate. We conducted additional searches for economic and qualitative evidence on the included impact evaluations. We used random effects meta-analysis to examine effects on the primary outcome, full immunisation coverage.

Results

Our search identified over 43000 studies and 61 were eligible for analysis. The average pooled effect of community engagement interventions on full immunisation coverage was SMD 0.14 (95% CI: 0.06 to 0.23, $I^2 = 94.46$). The most common source of risk to the quality of evidence (risk of bias) was outcome reporting bias: most studies used caregiver-reported measures of vaccinations received by a child in the absence or incompleteness of immunisation cards. Reasons consistently cited for intervention success include appropriate intervention design, including building in community engagement features; addressing common contextual barriers of immunisation and leveraging facilitators; and accounting for existing implementation constraints. The median intervention cost per treated child per vaccine dose (excluding the cost of vaccines) to increase absolute immunisation coverage by one percent was USD 2.30.

Conclusion

Community engagement interventions are successful in improving outcomes related to routine child immunisation. The findings are robust to exclusion of studies assessed as high risk of bias.

For peer review only - http://bmjopen.bmj.com/site/about/guidelines.xhtml

Protocol registration: Campbell Collaboration, DOI: 10.1002/cl2.1119.

Strengths and limitations of this study

- Thorough literature search of twenty-one major electronic databases and reporting as per Preferred Reporting Items for Systematic Reviews and Meta-Analysis guidelines.
- Presents a nuanced framework of community engagement with a typology that differentiates three types of interventions: interventions in which community engagement is embedded, and those interventions that engage community in their design or implementation.
- The effects of community engagement interventions are robust to exclusion of studies assessed as high risk of bias for almost all the primary outcomes. The effects are also uniform across geographies and baseline immunisation rates.
- For some immunisation outcomes the evidence base for drawing conclusions is adequate, for others it is limited.
- Evidence base is skewed across the three engagement types with a relatively large evidence base for those interventions in which community engagement is embedded and limited for those interventions using engagement in implementation autonomy

For peer review only - http://bmjopen.bmj.com/site/about/guidelines.xhtml

Introduction

Immunisation is one of the most cost-effective interventions to prevent and control life-threatening infectious diseases. From 2001 to 2020, projects that introduced or increased coverage of vaccines averted an estimated 14 million deaths, 350 million cases of illness, 8 million cases of long-term disability and 700 million disability-adjusted life-years.[1] Nonetheless, rates of routine vaccination of children in low- and middle-income countries (L&MICs) are low or stagnant. In 2019, an estimated 19.7 million infants did not receive routine immunisations. Around 60% of these children live in ten L&MICs, including Ethiopia, India, Nigeria, and Pakistan as of 2019.[2]

Community engagement approaches feature prominently in global immunisation strategies.[3] Their role has become increasingly important in preparing for and responding to COVID-19.[4,5] However, there is a dearth of rigorous and systematic evidence about whether and how community engagement interventions work to increase routine childhood immunisations, for whom, or at what cost. There is, therefore, a need to make such evidence available to guide policymakers and public health practitioners in making informed decisions about these interventions. To address this knowledge gap, we conducted a systematic review examining the effects of community engagement interventions on outcomes related to childhood immunisation in L&MICs, determining their cost-effectiveness, and identifying contextual, design and implementation features that may be associated with intervention effectiveness.

WHO 2020 defines community engagement as "*a process of developing relationships that enable stakeholders to work together to address health-related issues and promote well-being to achieve positive health impact and outcomes*."[6] For this review we developed a framework that classified community engagement interventions based on *process* of engagement as in the WHO definition. It also corresponds to the "utilitarian perspective" of community engagement captured and articulated in Brunton et al. (2017)[7]: "*In utilitarian perspectives, health (and other) services reach out to engage particular communities that they have identified require assistance and the intervention is devised within existing policy, practice, and resource frameworks*." In addition, our framework goes beyond one-way communication to include some consultation or dialogue with the community or some decision making by them. We considered three points within an

For peer review only - http://bmjopen.bmj.com/site/about/guidelines.xhtml

intervention during which engagement could occur as elaborated below and in Appendix 1 pp. 3-8 (also see Supplementary Figure 1).

Engagement in the design of interventions: community input or feedback was sought before implementing an intervention (e.g., pilot, needs assessment, formative evaluation, and outreach).

Engagement in implementation autonomy of interventions: community was utilized in intervention implementation as health care workers, facilitators, or problem solvers and only if they had some opportunity to affect or influence its implementation.

Engagement as the intervention (engagement is embedded): a serious attempt was made to gain community buy-in for activities or new cadres of community-based structures were established (e.g., village health committees or community health volunteers).

Methods

Overview

The protocol of this systematic review with meta-analysis is registered with The Campbell Collaboration.[8] We followed the Campbell and Cochrane Collaborations' guidelines for systematic reviewing[9-12] and drew on theory-based mixed methods impact evaluation[13] and systematic review[14,15] concepts. We followed the PRISMA reporting guidelines. The amendments to the information provided in the protocol is reported in Appendix 2 pp.9-10.

Search strategy and selection criteria

We implemented a systematic and comprehensive search strategy, in consultation with an information specialist. In May 2019 and May 2020, we searched 21 databases of academic and grey literature and 12 further websites for experimental and quasi-experimental impact evaluations of community engagement interventions considering outcomes related to routine child immunisation in L&MICs (using the World Bank country classification to determine L&MIC status). No language, publication type or date restrictions were imposed. The complete search strategy and terms are detailed in Appendix 3 pp. 11-15. We complemented this with citation tracking and contacting experts. The grey literature search was conducted by study authors and external consultant reviewers. We only included studies evaluating an intervention meeting our definition of community engagement. We defined "community" as a group of people who were

For peer review only - http://bmjopen.bmj.com/site/about/guidelines.xhtml

served by a particular primary health facility providing routine immunisation services. Therefore, we excluded interventions targeting higher levels of the health system (e.g., state-level officials). We also excluded studies for which the reported quantitative data could not be meaningfully converted to an effect size (Appendix 4 pp. 16-27). In cases of relevant missing or incomplete data, we contacted study authors to obtain the required information. If we were unable to obtain the necessary data, we reported the characteristics of the study but did not include these studies in the meta-analysis. We conducted additional searches for economic and qualitative evidence on the included impact evaluations (Appendix 5 pp. 28-36).

At both the title and abstract and full-text screening stages, all papers were double screened. Reconciliation meetings were held to resolve disagreements and a study author made final decisions on unresolved cases. Qualitative papers and project documents were screened as they were identified in the search and reviewed again before they were fully coded.

Outcomes

The primary outcomes considered in this review are coverage rates for (a) full immunisation, (b) third dose of diphtheria, pertussis and tetanus (DPT) or pentavalent, (c) measles, or (d) the timeliness of any of these doses. Additional antigen-specific immunisation coverage outcomes and secondary outcomes reflecting upstream conditions (e.g., attitudes about vaccination and access to immunisation services) and downstream effects (e.g., morbidity and mortality) of the primary outcomes were also included. Official health records and parent recall were considered acceptable sources of measures of immunisation coverage. The former was used when both measures were reported separately.

Data analysis

We used Microsoft Excel to extract descriptive information and effect sizes from included studies using double coding. Coders reconciled their answers and a study author made final decisions in case of disagreements. For qualitative analysis, all impact evaluations and additional documentation identified in the search were coded in Nvivo. Cost data was single coded and checked by a study author (Appendix 6 pp. 37-64).

To avoid double-counting of evidence from different papers focusing on the same study, we linked these papers prior to analysis. We extracted data from the most recent publication. When data was

For peer review only - http://bmjopen.bmj.com/site/about/guidelines.xhtml

reported over multiple time periods, we extracted data for each period. Where authors reported the same outcome using more than one analytical model, we extracted data from the authors' preferred model specification. When the preference was not specified, we used the model with the most controls. Where studies reported outcomes related to multiple treatment arms and only one comparison group, we chose the treatment arm that was identified as the arm that would be scaled up.

To assess quantitative risk of bias, we created an adapted version of the Cochrane guidelines for assessing RCTs and non-randomised studies.[16,17] These assessments were conducted by two independent reviewers. Coders reconciled their answers and a study author made final decisions in case of disagreements. For testing the sensitivity of the results to low-quality studies, we ran each analysis with and without studies scoring high risk of bias.

We critically appraised qualitative and mixed-methods studies using an adapted version of the nine-item framework developed by the Critical Appraisal Skills Programme.[18] In addition, we carried out a sensitivity analysis in which we considered only the high-quality qualitative studies that had a risk of bias assessment score of 20 or higher, indicating low risk of bias. For cost evidence, we assessed risk of bias along six primary dimensions adapted from a combination of tools, including: Doocy and Tappis (2017);[19] Campbell Collaboration Economic Methods Policy Brief;[20] and Methods for the Economic Evaluation of Health Care Programmes[21] (Appendix 7 pp. 65-89).

We calculated the standardised mean difference (SMD), or Cohen's *d*, its variance and standard error for each effect, converting effects reported in other metrics as necessary, using formulae provided in Borenstein and colleagues (2009).[22] In all cases we then adjusted Cohen's *d* to Hedges' *g* as defined in Ellis (2010).[23] For studies reporting regression results, we followed the approach of Keef and Roberts (2004)[24] using the regression coefficient and the pooled standard deviation of the outcome.

The amount of heterogeneity (i.e.,τ^2) was estimated using the DerSimonian-Laird estimator.[25] The Q-test for heterogeneity[26] and the I^2 statistic[27] are reported. We complement this with an assessment of heterogeneity of effect sizes graphically using forest plots. Whenever feasible, we conducted moderator analyses using meta-regression to investigate sources of heterogeneity. The

For peer review only - http://bmjopen.bmj.com/site/about/guidelines.xhtml

analysis was carried out using R (version 4.0.4)[28] and the **metafor** package (version 2.4.0).[29] All analyses used a random effects model.

Qualitative analysis followed a mix of inductive and deductive coding approaches to identify themes related to barriers and facilitators, reasons for intervention success or failure, and uptake and fidelity challenges.

Role of the funding source

The funder of the study had no role in study design, data collection, data analysis, data interpretation, or writing of the report.

Patient and public involvement in research

There was no patient or public involvement in this research.

Ethics statement

Ethical consent from an Ethics Committee or Institutional Board was not required as this study does not involve human subjects.

Results

Our search identified over 43,000 records, which were reduced to 29,481 unique abstracts after de-duplication (Figure 1). After title and abstract screening, we considered 1,285 studies for full-text screening and could not locate an additional 44, published mostly before 2000. We excluded articles at full-text for not satisfying the inclusion criteria by country (129), study type (evaluation study) (304), evaluation method (213), outcome (235) and community engagement type (172). We ultimately identified 61 impact evaluations (Table 1)[30-91] that assessed the effects of community engagement interventions on outcomes related to routine child immunisation in L&MICs. Five studies did not include sufficient data to calculate an effect size, thus 56 studies were included in the meta-analysis.

[Place figure 1 here]

For peer review only - http://bmjopen.bmj.com/site/about/guidelines.xhtml

Table 1 describing characteristics of the included studies

Author	Country	Summary of intervention	Engagement type	Study design	Duration (in months)	Outcomes
Admassie 2009[30]	Ethiopia	Formation of a cadre of community-based health extension workers and using community resources for construction of health posts.	Multiple (EII + EAI)	PSM	48	FIC, BCG, DPT3, OPV1, OPV2, OPV3, measles, morbidity
Adamu 2019[31]	Nigeria	Quality improvement programme where health workers use iterative processes to develop localised and contextually relevant plans to resolve health service delivery and demand bottlenecks.	Multiple (EID + EII)	ITS	1	Dropouts
Alhassan 2019[32]	Ghana	Using a bottom-up approach, the intervention first recruited and trained community groups to identify service delivery gaps in healthcare facilities.	Multiple (EII + EAI)	RCT	10	FIC
Andersson 2009[33]	Pakistan	Community dialogues to address barriers to vaccination. The guidelines for the dialogue were created after consultation with the intended beneficiaries.	Multiple (EID + EAI)	RCT	8	Knowledge about immunisation, attitude about immunisation, community norms, readiness to vaccinate, household norms & decision-making measles, DPT3

For peer review only - http://bmjopen.bmj.com/site/about/guidelines.xhtml

Arifeen 2009[34]	Bangladesh	Formation of a cadre of village health volunteers and enlisting support of local religious leaders to convey messages about child health.	EAI	RCT	71	Measles, mortality
Assegaai 2018[35]	South Africa	Lay community-based workers were formalised as community health workers and served as a part of the outreach teams.	EAI	DID	36	FIC, measles, morbidity
Banerjee 2010[36]	India	Provision of immunisation services and incentives to caregivers. A trusted community-based organisation was a key stakeholder in design and delivery of the intervention.	Treatment 1: EAI<br>Treatment 2: Multiple (EID + EAI)	RCT	18	FIC, BCG, partial immunisation
Banerjee 2020[37]	India	This evaluation tested two different interventions: 1. Incentives to caregivers. The community's feedback was solicited on the kind of incentive. 2. Community influencers were identified to spread information about immunisation.	EID<br>EAI	RCT	14	Knowledge about immunisation, attitude about immunisation, FIC, DPT1, DPT2, DPT3, measles
Banwat 2015[38]	Nigeria	Female members of the community whose children are fully immunised were nominated in each community to serve as peer educators.	EAI	CBA	-	FIC, knowledge about immunisation, attitude about immunisation, readiness to vaccinate
Biemba 2016[39]	Zambia	A national policy to create a cadre of well-trained and motivated community-based health workers.	EAI	DID	23	FIC, morbidity

For peer review only - http://bmjopen.bmj.com/site/about/guidelines.xhtml

Björkman 2009[40]	Uganda	Communities were involved in monitoring the quality of health services and the performance of health service providers.	EII	RCT	0.16	FIC, BCG, OPV0, OPV3, DPT1, DPT3, measles, Partial routine immunisation, mortality
Bolam 1998[41]	Nepal	Training for community health workers and midwives which was developed in collaboration with health workers and experts.	EID	RCT	3	FIC
Borkum 2014[42] Carmichael 2019[43] (linked study)	India	Performance-based incentives to frontline workers. The nature of incentives was decided upon in consultation with the frontline workers.	EID	RCT	12	Health card availability, CHW capacity, FIC, BCG, DPT1, DPT2, DPT3, OPV1, OPV2, OPV3, measles, partial immunisation, timeliness
Calderón-Ortiz & Mejía-Mejía 1996[44]	Mexico	Creation of a community-based cadre of volunteers to register and track children in the community for immunisation.	EAI	CBA	4	FIC, BCG, DPT3, OPV3, measles
Carnell 2014[45]	Ethiopia	Formation of a cadre of community health workers to mobilize the community and encourage uptake of health services.	EAI	DID	60	DPT3, measles

For peer review only - http://bmjopen.bmj.com/site/about/guidelines.xhtml

Costa-Font 2017[46]	India	Establishment of the village health and sanitation committees to monitor health service provision at the community level.	EAI	IV	-	BCG, DPT1, OPV0
Demilew 2020[47]	Ethiopia	A poster/stamp system that reminded health workers of the child's immunisation status and simultaneously encouraged caregivers to immunise their children. The intervention was designed in consultation with health workers.	Multiple (EID + EAI)	RCT	17	FIC, BCG, DPT1, DPT2, DPT3, partial immunisation
Dipeolu 2017[48]	Nigeria	Text message reminders to mothers regarding immunisation schedule. The messages were field tested with mothers to get the content right.	EID	DID	9	Knowledge about immunisation, attitude about immunisation, timeliness
Domek 2019[49]	Guatemala	SMS text messages to caregivers. A prior feasibility and acceptability study was conducted for the intervention.	EID	RCT	2	Timeliness
Engineer 2016[50]	Afghanistan	Pay-for-performance bonuses paid quarterly to health workers. The bonus amount was revised after receiving health worker feedback.	EID	RCT	24	Experience & satisfaction with health services, Formal HW motivation, capacity & performance, DPT3

For peer review only - http://bmjopen.bmj.com/site/about/guidelines.xhtml

Findley 2013[51]	Nigeria	Formation of a cadre of community volunteers to facilitate group discussions on health and track/register women/children for health services.	EAI	DID	24	FIC
Gibson 2017[52]	Kenya	SMS reminders and monetary incentives to caregivers. A feasibility study was conducted in 2013 for this intervention.	EID	RCT	12	Community norms, FIC, BCG, DPT1, DPT2, DPT3, OPV0, OPV1, OPV2, measles, timeliness
Goel 2012[53]	India	A multi-component campaign which involved women groups in awareness generation to improve health service uptake.	EAI	DID	48	FIC
Gurley 2020[54]	India	Community members were trained to design and produce culturally appropriate, 'hyper-local' videos to promote health seeking behaviours.	Multiple (EID + EAI)	RCT	11	Knowledge about immunisation, attitude about immunisation, FIC, DPT3, partial immunisation, timeliness, dropouts
Herrera-Almanza 2018[55]	Madagascar	Community-based primary health care services intervention that included the deployment of volunteer community health workers in remote areas.	Multiple (EII + EAI)	DID	26	Health card availability, OPV3, DPT3, measles, mortality, partial immunisation

For peer review only - http://bmjopen.bmj.com/site/about/guidelines.xhtml

Igarashi 2010[56]	Zambia	The GMP+ sessions were conducted by medical personnel from Public Health Centers. During these sessions, community volunteers provided some operational and managerial support to ensure the effective implementation of the sessions.	EII	CBA	43	FIC, timeliness, attitude about immunisation, community norms
Janssens 2011[57]	India	Dissemination of health promoting messages to women in the community who are encouraged to further spread the awareness.	EAI	IV	56.4	DPT3, measles
Johri 2020[58]	India	Interventions, designed through formative research, to increase caregiver knowledge and adherence to childhood immunisation.	EID	RCT	3	Knowledge about immunisation, Awareness of place, time, schedule for vacc., attitude about immunisation
Lee 2015[59]	Zambia	Creation of a new cadre of frontline workers from the community, called community health assistants, to provide primary health care services.	EAU	RCT	3	Formal HW motivation, capacity & performance, BCG, OPV3, measles, timeliness, CHW capacity, morbidity
Mayumana 2017[60]	Tanzania	Payment-for-performance scheme for health facilities. Health workers and health facility governing committees decided the allocation of funds.	EII	DID	30	Stockouts

For peer review only - http://bmjopen.bmj.com/site/about/guidelines.xhtml

Memon 2015[61]	Pakistan	Formation of community health committees to promote perinatal and new-born care. Formative research informed the intervention design.	Multiple (EID + EAI)	DID	16	FIC
Modi 2019[62]	India	The mHealth intervention package consisting of mobile phone-based job aids for community health workers. The intervention was piloted in 2015.	EID	RCT	12	DPT3, morbidity
Mohanan 2020[63]	India	Social accountability interventions to promote community-based collective action to improve delivery of health and nutrition services to children.	EAI	RCT	12	Experience & satisfaction with health services, attitudes about health providers, formal health worker supply, FIC, BCG, DPT3, OPV1, OPV3, IPV, measles, morbidity, mortality
More 2012[64]	India	Urban slum-dweller women's groups used community dialogues to address barriers to improving perinatal health.	EAI	RCT	36	Mortality
More 2017[65]	India	The intervention comprised multiple activities like home visits to caregivers, groups meetings, community events and other supportive services.	EAI	RCT	24	Health card availability, attitudes about health providers, FIC, BCG, measles, partial immunisation

For peer review only - http://bmjopen.bmj.com/site/about/guidelines.xhtml

Morris 2004[66]	Honduras	Monetary vouchers to women in the communities and setting up of community-based committees to oversee health service quality and access.	EAI	RCT	24	DPT1, measles
Murthy 2019[67]	India	Voice call reminders to pregnant women and caregivers. The message content was tested for appropriateness through community focused groups.	EID	RCT	21	Knowledge about immunisation, FIC
Nagar 2018[68]	India	A digital pendant-based health record of the child and a voice call reminder system. A formative study was conducted in 2016 and communities were consulted on the design of the pendant.	EID	RCT	3	Timeliness
Nagar 2020[69]	India	A digital pendant-based health record of the child. Health providers used a mobile application to scan the pendant to update the child's medical history. Prior formative research informed the intervention design.	EID	RCT	20	FIC, DPT1, DPT2, DPT3
Nzioki 2017[70]	Kenya	Formation of a cadre of community health workers.	EAI	CBA	0	FIC
Oche 2011[71]	Nigeria	Group meetings with caregivers and dialogues with community leaders to improve uptake of routine immunization services.	EAI	DID	9	Knowledge about immunisation, DPT1, DPT3, dropouts
Okeke 2017[72]	Nigeria	A national scheme to create, train and deploy a cadre of midwives to serve underserved rural and remote populations in Nigeria.	Multiple (EII + EAI)	DID	40	BCG, DPT3, OPV3, measles, mortality

For peer review only - http://bmjopen.bmj.com/site/about/guidelines.xhtml

Okoli 2014[73]	Nigeria	A conditional cash transfer program to encourage uptake of health services. Community groups were consulted while deciding the cash amount.	EID	ITS	7 to 18	OPV0
Olayo 2014[74]	Kenya	Formation of a cadre of community health workers who then facilitated dialogue at the community level and supported other community-based workers.	Multiple (EII + EAI)	DID	24	Health card availability, DPT1, DPT3, measles
Olken 2014[75]	Indonesia	Block grants for maternal and child health that incorporated relative performance incentives were implemented in villages through creation of village-level health committees.	Multiple (EII + EAI)	RCT	18 to 30	FIC, morbidity, mortality
Oyo-Ita 2020[76]	Nigeria	A multi-component intervention involving traditional and religious leaders for engaging communities in planning and delivery of immunisation services.	Multiple (EII + EAI)	RCT	18	FIC, partial immunisation, Timeliness
Pramanik 2020[77]	India	Trained facilitators from local NGOs interacted with the communities to enable communities to leverage their own strengths for addressing their concerns related to child health.	EAI	RCT	13	Knowledge about immunisation, attitude about immunisation, attitudes about health providers, health card availability, FIC, DPT1, DPT2, DPT3, timeliness, dropouts

For peer review only - http://bmjopen.bmj.com/site/about/guidelines.xhtml

Rahman 2008[78]	Pakistan	Mental health support program with counselling sessions for pregnant and post-partum women. Prior intervention pilots informed the intervention design.	EID	RCT	11	FIC, morbidity
Rahman 2016[79]	Bangladesh	New cadre of community health workers delivered essential maternal, neonatal, and child healthcare and nutrition services.	EAI	DID	48	FIC, morbidity
Rao 2014[80]	India	Creation of a cadre of community health workers to improve basic health outcomes through community engagement.	EAI	DID	60	BCG, DPT3, OPV3, measles, FIC, partial immunisation, supply of CHWs
Robertson 2013[81]	Zimbabwe	Use of cash transfers for behaviour change. Local NGO and community leaders were involved in beneficiary targeting and compliance monitoring. The intervention was also tested for feasibility during a prior study.	Multiple (EID + EII)	RCT	12	FIC, community norms, OPV0
Roy 2008[82]	Bangladesh	Rural maintenance programme recruited and trained women for road maintenance, health awareness, numeracy, human rights, gender equity, health and nutrition, and business management.	EAI	DID	11	FIC, BCG, DPT1, DPT2, DPT3, OPV0, OPV1, OPV2, OPV3, measles, partial immunisation
Saggurti 2018[83]	India	Formation of health-focused self-help groups with women of reproductive age coming from the most marginalized communities.	EAI	DID	2	Timeliness

For peer review only - http://bmjopen.bmj.com/site/about/guidelines.xhtml

Sankar 2013[84]	India	Formation of committees with representatives of the community, local government and service providers to ensure better convergence and coordination of service delivery.	Multiple (EII +EAI)	DID	30	FIC, BCG, DPT1, DPT2, DPT3, OPV0, OPV1, OPV2, OPV3, measles, partial immunisation, timeliness
Seth 2018[85]	India	The study evaluated two different interventions: role of compliance-linked incentives versus text messaging to improve childhood immunisations. Incentive amount was determined after input was received from the local investigators as well as the community workers.	EID	RCT	9.7	Partial immunisation, timeliness, attitude about immunisation, attitudes about health providers
Shukla 2018[86]	Afghanistan	Community representatives along with health officials identify the health needs of the communities and communicate those to the service providers.	EII	DID	6	Supply of CHWs, DPT3
Siddiqi 2020[87]	Pakistan	Visual reminders to caregivers in the form of wearable bracelets for the child. The bracelets were designed in consultation with the caregivers.	EID	RCT	12	DPT3, measles
Tandon 1988[88]	India	Enlisting community-based volunteers to motivate and encourage family members to utilise maternal and child health services.	EAI	CBA	120	FIC, BCG, DPT2, DPT3, OPV2
USAID 2008[89]	Ethiopia	Creation of a cadre of community health promoters to carry out behaviour change communication activities in the communities.	EAI	CBA	48	FIC, BCG, DPT1, DPT3, OPV3, morbidity, health

For peer review only - http://bmjopen.bmj.com/site/about/guidelines.xhtml

						card availability, measles, dropouts
Webster 2019[90]	Uganda	Community-based outreach and follow-up with caregivers to improve immunisation uptake and reduce defaulters.	EII	RCT	12	Health card availability, BCG, DPT1, DPT2, DPT3, OPV0, OPV1, OPV2, OPV3, IPV, measles, partial immunisation, timeliness, attitude about immunisation, dropouts, morbidity, mortality
Younes 2014[91]	Bangladesh	The intervention involved 162 women's groups who used participatory approaches to discuss maternal and neonatal health issues.	EAI	DID	20	FIC, morbidity

Note: EAI – Engagement in design, EID = Engagement in intervention, EII = Engagement in implementation autonomy

For peer review only - http://bmjopen.bmj.com/site/about/guidelines.xhtml

Of the 31 included studies with experimental designs, only two had a low risk of bias, six had some concerns and 23 had a high risk of bias. Of the 30 included quasi-experimental studies, only two were assessed as low risk of bias, one as some concerns and 27 as high risk of bias. Biases arising from outcome measurement and deviations from intended interventions were the most common across both the study designs (Appendix 8 pp. 90-91, Supplementary Figures 2-3).

Although only five qualitative studies scored strong on all key elements, most studies received strong scores on most key elements and had quality appraisal scores greater than 20, indicating low risk of bias (Appendix 8, pp.91-92, Supplementary Table 1, Supplementary Figures 4-5). The most common elements found to be missing were sample characteristics and analytic methods. The quality of the cost evidence in the 22 studies that included such evidence was mixed (Appendix 8 pp. 92-95, Supplementary Figure 6).

Community Engagement Interventions

Below we present the findings related to the primary outcomes of interest. Supplementary Table 2 summarises the quantitative findings both primary and secondary outcomes across all intervention types (Appendix 9, pp. 120-121).

Full Immunisation

A total of $k = 28$ studies examined the effect of community engagement interventions on full childhood immunisation and found $\hat{\mu} = 0.14$ ([95% CI: 0.06 to 0.23], $z = 3.28$, $p = 0.001$), indicating a small but significant benefit for the treated group of .14 standard deviation units (Figure 2). The rank correlation test indicated funnel plot asymmetry ($p = 0.03$) but the regression test did not ($p = 0.57$; see Supplementary Figures 7-7b in Appendix 9 pp. 96-97). The true effects appear to be heterogeneous ($I^2 = 94.5\%$). Outlier analyses revealed that Banerjee (2010) may be a potential outlier, and sensitivity analyses removing Banerjee (2010) reduced the overall average effect ($\mu = 0.08$ [95% CI: 0.04 to 0.12]), but it was still positive and significant ($z = 4.12$, $p < .001$).[1]

Sensitivity analyses were also conducted to examine the robustness of the results to the exclusion of low-quality studies. When studies assessed as high risk of bias were removed, the resulting

[1] For all other outcomes, outlier analyses will be presented in Appendix 9.

For peer review only - http://bmjopen.bmj.com/site/about/guidelines.xhtml

effect was slightly larger and still statistically significant ($\hat{\mu} = 0.18$ [95% CI: 0.08 to 0.27]), $k = 4$, $z = 3.67$, $p < 0.001$). We examined several potential sources of heterogeneity, including exposure to the intervention, evaluation period, study design, year, geographical region, data source, whether the intervention was implemented by a government agency (either alone or in combination with another agency), whether new cadres of health workers were established, presence of vaccine hesitancy, and baseline vaccine coverage rates. There were no significant moderators in the context of this model (see Appendix 9, Supplementary Table 3, pp 122-127).

[Place figure 2 here]

DPT 3

A total of $k = 22$ studies examined the effect on DPT3 vaccination coverage and found a small but significant benefit to the treated group compared to the untreated group ($\hat{\mu} = 0.10$ [95% CI: 0.06 to 0.14], $z = 4.75$, $p < 0.001$; Figure 3). The true outcomes appear to be heterogeneous ($I^2 = 76.8\%$). When low quality studies were removed, the average effect increased slightly ($\hat{\mu} = 0.11$ [95% CI: 0.05 to 0.17], $k = 4$), and was still statistically significant ($z = 3.70$, $p < 0.001$; see Appendix 9, Supplementary Figures 8 – 10, pp. 97 – 98). Publication year was a significant source of heterogeneity; each additional year reduced the size of the effect by .014 standard deviation units (see Appendix 9, Supplementary Table 3, pp. 123-124).

[Place figure 3 here]

Measles

A total of $k = 20$ studies examined the effect on measles vaccination coverage and found a very small but significant benefit for the treated group compared to the untreated group ($\hat{\mu} = 0.07$ ([95% CI: 0.03 to 0.11], $z = 3.21$, $p < 0.01$; *see Figure 4*). When low quality studies were removed, the average effect increased ($\hat{\mu} = 0.09$, $k = 6$, [95% CI: 0.03 to 0.15]) and was still statistically significant ($z = 2.98$ $p = 0.003$; see Appendix 9, Supplementary Figures 11 – 13, pp. 99 – 100). The true outcomes appear to be heterogeneous ($I^2 = 73.6\%$). None of the moderators were significant sources of heterogeneity (see Appendix 9, Supplementary Table 3, pp. 122-127).

[Place figure 4 here]

For peer review only - http://bmjopen.bmj.com/site/about/guidelines.xhtml

Vaccination Timeliness

We found a small but significant effect on all three timeliness outcomes: full immunisation timeliness ($\hat{\mu} = 0.15$ [95% CI: 0.07 to 0.24], $z = 3.41$, $p < 0.001$); DPT3 timeliness ($\hat{\mu} = 0.09$ [95% CI: 0.03 to 0.14], $z = 3.00$, $p < 0.01$); and measles timeliness ($\hat{\mu} = 0.23$ [95% CI: 0.14 to 0.32], $z = 5.06$, $p < 0.001$). For all timeliness outcomes, tests of heterogeneity were not significant (p > .05). For full immunisation and measles timeliness outcomes, the sensitivity analysis could not be conducted due to an inadequate number of studies. For DPT3, the average effect increased but became non-significant when low quality studies were removed. (See Appendix 9: pp.100-103 including Supplementary Figures 14-16 & Supplementary Tables 2 & 3 for detailed analysis).

Analyses of secondary outcomes are presented in Appendix 9, Supplementary Figures 17-32, pp. 103-119.

Subgroups of community engagement interventions

Table 2 presents an overview of primary outcomes by engagement type. Studies that used engagement as the intervention had a significant positive effect on full childhood immunisation, DPT3 vaccination and measles vaccination, but evidence was insufficient to synthesize measures of vaccination timeliness. See Appendix 10: pp. 128-140 including Supplementary Figures 33-44 for a detailed analysis of primary and secondary outcomes.

When studies used community engagement in the design, there was a significant positive effect on full childhood immunisation and measles vaccination but not on DPT3 vaccination. Additionally, there was a positive significant effect on timeliness of full childhood immunisation and DPT3 vaccination. No studies using engagement in the design reported on timeliness of measles vaccinations. See Appendix 11: pp. 141-154 including Supplementary Figures 45-59 for a detailed analysis of primary and secondary outcomes.

For engagement in implementation autonomy, the analysis is based on a limited number of studies and we found no significant effect on either coverage or timeliness outcomes. There were no studies reporting on the timeliness of measles vaccination. See Appendix 12: pp. 157-161 including Supplementary Figures 60-66 for a detailed analysis of primary and secondary outcomes.

For peer review only - http://bmjopen.bmj.com/site/about/guidelines.xhtml

Finally, some studies combined multiple engagement types in their interventions. These interventions had a significant effect on DPT3 vaccination but not on measles vaccination or full childhood vaccination. Evidence was insufficient to synthesise measures of vaccination timeliness. See Appendix 13: pp. 162-176 including Supplementary Figures 67-81 for a detailed analysis of primary and secondary outcomes.

Table 2. Overview of the primary outcomes by engagement type

	**Random effects summary estimate for all interventions**	**Engagement as the intervention**	**Engagement in the design**	**Engagement in the implementation autonomy**	**Multiple engagement types**
	g, [95% CI], I^2, (k)	g, [95% CI], I^2, (k)	g, [95% CI], I^2, (k)	g, [95% CI], I^2, (k)	g, [95% CI], I^2, (k)
**Full immunisation**	**0.14**, [0.06, 0.23],** 94.46, (28)	**0.08**, [0.03, 0.13],** 70.00, (12)	**010*, [0.02, 0.19],** 23.83, (5)	0.23, [-0.001, 0.47], 73.07, (2)	0.22, [-0.12, 0.56], 97.94, (9)
**Measles**	**0.07**, [0.03, 0.11],** 73.64, (20)	**0.10***, [0.05, 0.15],** 60.29, (10)	**0.11*, [0.02, 0.21],** 0.00, (2)	0.03, [-0.09, 0.15], 54.84, (2)	0.03, [-0.10, 0.16], 86.76, (6)
**DPT3**	**0.10***, [0.06, 0.14],** 76.78, (22)	**0.09**, [0.03, 0.15],** 73.17, (6)	0.04, [-0.01, 0.08], 0.00, (6)	0.11, [-0.05, 0.28], 80.12, (3)	**0.20**, [0.06, 0.34],** 87.93, (7)
**Timeliness (DTP3)**	**0.09**, [0.03, 0.14],** 0.00, (7)	N/A	**0.12**, [0.03, 0.21],** 0.00, (4)	0.04, [-0.06, 0.13], 0.00, (2)	N/A
**Timeliness (Measles)**	**0.23***, [0.14, 0.32],** 0.00, (3)	N/A	N/A	N/A	N/A

For peer review only - http://bmjopen.bmj.com/site/about/guidelines.xhtml

**Timeliness (full immunisations)**	**0.15***, [0.07, 0.24],** 9.66 (5)	N/A	**0.15*, [0.004, 0.29], 7.202, (2)**	0.38, [-0.28, 1.03], 0.00, (2)	N/A

Qualitative findings

Programme design characteristics were associated with intervention success or failure across all engagement types. Certain aspects of community engagement itself, such as conducting stakeholder consultations, holding community dialogues or involving community leaders were associated with better immunisation outcomes. Non-engagement intervention design features also affected intervention success. These design features include incentives given to caregivers and leadership and supportive supervision, which improved overall health service delivery and health worker performance. Among the studies that attributed intervention failure to programme design, inadequate duration, frequency or exposure to the intervention were the most notable themes.

The importance of accounting for *contextual* barriers to or facilitators of immunisation emerged consistently. Limited availability of services, especially insufficient staff and vaccine supply, were dominant barriers to immunisation, affecting outcomes in the early portion of the causal chain. Other common barriers to immunisation included practical barriers faced by caregivers such as costs, largely indirect, and logistics (wait time and language barriers) or distance. There was more variation in barriers related to social norms, fear, and an understanding of the importance of immunisation by type of engagement. Poor quality of services, including uninviting attitudes of health workers, posed a barrier to immunisation in communities that received engagement as the intervention or were engaged in the design of the intervention.

However, we also found that certain contextual factors could become facilitators of immunisation outcomes, provided an intervention has adequately situated itself to leverage them. Across all engagement types, studies associated caregivers' awareness and perception of the benefits of vaccination with improved immunisation outcomes. Similarly, availability of health infrastructure and good quality of services were also associated with improved intervention uptake and its impact.

For peer review only - http://bmjopen.bmj.com/site/about/guidelines.xhtml

Implementation failures, such as low fidelity, were a common reason for intervention failure. Across all engagement types, interventions did not properly account for existing implementation constraints and practicalities on the ground and were forced to change their implementation plans. Many of these issues were encountered due to uncontrollable factors or invalid theory of change assumptions. For instance, programme design may not have accounted for the unavailability of intended participants due to competing priorities, thereby potentially invalidating the assumption of beneficiary exposure in the casual pathway. Administrative challenges were cited consistently though their nature varied across engagement types ranging from technical limitations (such as limited cellphone service) to political constraints to insufficient staffing levels.

Results were broadly consistent when only qualitative studies with a quality appraisal score greater than 20 were considered. The full qualitative synthesis and sensitivity analysis is available in Appendix 14(pp. 177-225: including Supplementary Tables 4-20 & Supplementary Figures 82-106)

Cost effectiveness findings

Among the 14 studies for which we could calculate cost-effectiveness, we found that the median intervention cost per treated child per vaccine dose ￼ (excluding the cost of vaccines) to increase absolute immunisation coverage by one percent was USD 2.30 (all costs are reported in 2019 US dollars) and the average cost was USD 43.45. There were three outlier observations that drove up this average cost and without them cost per vaccine dose to increase absolute immunisation coverage by one percent averaged USD 3.18 (See Appendix 15: pp. 226-231 including Supplementary Tables 21-22 & Supplementary Figures 107-108).

Discussion

Our systematic review uses a detailed framework of community engagement interventions to assess their effectiveness for improving outcomes related to routine child immunisation in L&MICs. As far as we are aware, ours is the first systematic review to do this. We found that community engagement interventions had a small, but significant, positive effect on all primary immunisation outcomes related to coverage and their timeliness. Sensitivity analyses excluding high risk of bias studies showed that the effect was slightly larger and still statistically significant

For peer review only

For peer review only - http://bmjopen.bmj.com/site/about/guidelines.xhtml

for almost all the primary outcomes for which we had sufficient data. The effects were also uniform across geographies and baseline immunisation rates.

We also found that certain features of interventions may contribute to their success. These include (a) appropriate intervention design, including building in community engagement features; (b) addressing common contextual barriers of immunisation and leveraging facilitators; and (c) accounting for existing implementation constraints and practicalities on the ground. The median intervention cost per treated child per vaccine dose (excluding the cost of vaccines) to increase absolute immunisation coverage by one percent was USD 2.30.

Among the four types of community engagement interventions, we found that engagement as the intervention (embedded community engagement), which involves creation of community buy-in or development of new community-based structures or cadres, had consistent positive effects on more primary vaccination coverage outcomes than the others. We also found engaging the community in the design of the intervention had a positive effect on most primary outcomes related to coverage. We found no ubiquitous patterns of heterogeneity among the primary outcomes.

We drew on 61 studies for meta-analysis, comprising 31 RCTs and 30 quasi experimental studies. For full immunisation, DPT3 and measles coverage, we could draw upon 28, 22 and 20 studies respectively for pooled effects. However, for the timeliness of these coverage outcomes we had only 0 to 7 studies to assess the pooled effects. Thus, while for some outcomes the evidence base for drawing conclusions is adequate, for others it is limited. Among the four kinds of community engagement interventions, there was a relatively large evidence base for those with engagement as the intervention and those with multiple engagement types, while for interventions using engagement in implementation autonomy, the evidence was quite limited.

We identified additional documentation comprising qualitative studies, project reports, formative/process evaluations and observation studies for 39 of the 61 included impact evaluations. However, the crucial qualitative papers which help us gain a deeper understanding of overall intervention mechanisms of change were found for only 17 of the 61 IEs. Likewise, only 14 of the primary studies included in this review both estimated the intervention cost and reported it with sufficient detail for the review team to calculate the cost-effectiveness of the treatment. Low quality cost data and the unavailability of underlying cost data contributed to the small number of primary studies included in the cost-effectiveness analysis.

For peer review only - http://bmjopen.bmj.com/site/about/guidelines.xhtml

The quantitative evidence was mostly low quality, though the randomised studies were generally of higher quality than quasi-experimental studies. The quality of qualitative studies was generally high. The quality of the cost evidence was mixed. Despite the quality concerns about quantitative evidence, the sensitivity analysis conducted by excluding low-quality studies corroborated the overall findings. Despite a comprehensive search strategy and the inclusion of grey literature, publication bias was detected for the three primary coverage outcomes (full immunisation, DPT3, and measles). While bias correction analyses indicated an identical effect size for full immunisation and DPT3, the effect for measles was reduced when publication bias was corrected for. Timeliness outcomes had an insufficient number of studies to test for publication bias, which limits our ability to interpret heterogeneity.

COVID-19 has impacted routine child immunisation negatively in some countries, and community engagement interventions could be an effective way to counteract this decline. The positive effects of community engagement interventions can be expected across a variety of settings, although some engagement approaches appear to be more effective than others. Positive design features should be integrated into these interventions, including features such as holding community dialogues or involving community leaders, and non-community engagement features such as local supportive supervision and incentives to healthcare workers or caregivers. Wherever possible, binding contextual barriers to immunisation, such as weak health systems and social norms, should be accounted for in the design of interventions. Existing contextual facilitators for immunisation, such as good existing health systems or high maternal education, could be leveraged for increasing intervention impacts. Important implementation pre-conditions, such as regular internet service or sufficient staffing, should be assessed and established before the implementation or addressed through the design itself. Close monitoring of intervention implementation along with good understanding of context is important to help make necessary modifications in case of unexpected challenges, such as political instability.

For better-quality evidence and deeper mechanistic understanding, policy makers and practitioners should consider prioritising funding or commissioning research in the following areas: (a) ways of ameliorating outcome measurement bias due to self-reported immunisation coverage outcomes, as this was a principal source of bias; (b) better reporting of interventions, more rounded analysis of why the interventions worked through mixed-methods evaluations and greater focus on

For peer review only - http://bmjopen.bmj.com/site/about/guidelines.xhtml

intermediate outcomes for improved understanding of causal mechanisms; and (c) collection and reporting of high quality cost data to enable cost-effectiveness analysis, which is important for decision making within budget constraints.

Contributors

MJ, ME and AB conceived the review and wrote the initial protocol. ME and AB did the systematic search. ME, MJ and AB screened and identified studies and MJ and AB made final decisions regarding study inclusion. SS did the statistical analysis. CL and AB did the qualitative analysis. EB synthesised the cost evidence. MJ provided critical inputs on the whole analysis, checked data, coordinated the review and had full access to all materials and results. External consultants supported the authors in search, screening, data extraction and critical appraisal of quantitative and qualitative evidence base. All authors critically reviewed and revised the manuscript and approved the final document for submission. MJ is responsible for the overall content and is the guarantor.

Funding and competing interests

This research was funded through a grant from the Bill & Melinda Gates Foundation (OPP1115129) to the International Initiative for Impact Evaluation (3ie). Through this grant, 3ie provided funding and technical assistance for seven impact evaluations of community engagement interventions for immunisation as a part of its immunisation evidence programme. This technical assistance included, but was not limited to: reviewing study designs, analysis plans, and data collection instruments; advising research teams on how to improve study components and address challenges that arise during the course of the evaluation; and supporting grantees in engaging with stakeholders to promote uptake and use of evidence generated by the evaluations.

As members of 3ie staff, authors MJ, AB and ME have all had varying levels of involvement in reviewing deliverables for these evaluations and providing research teams with technical assistance. Several procedural safeguards and transparency measures were put in place to mitigate the risk this conflict of interest imposed. First, all candidate studies, including those funded by 3ie, underwent a rigorous multi-step screening process, including review at the title, abstract, and full-text levels. To qualify for inclusion in the SR, a study was judged to meet the inclusion criteria related to study design, outcomes and population by two independent screeners who have reviewed the full text of the study. The 3ie study authors were responsible for assessing whether the studies met the inclusion criteria for community engagement because of the complexity of the framework.

For peer review only - http://bmjopen.bmj.com/site/about/guidelines.xhtml

However, these authors have no financial interest in this area and have not published any prior reviews on the topic. The remaining study authors have no conflicts of interest to declare.

Data availability statement

The impact evaluations and other documents included in this systematic review are public and available. The quantitative, qualitative and cost-related data extracted from these studies are available from the corresponding author upon request.

Acknowledgments

This research has been undertaken as a part of 3ie's immunisation evidence programme, supported by the Bill and Melinda Gates Foundation, Seattle, USA. We would like to thank Sohail Agha, Senior Program Officer, Gates Foundation, Seattle, for his continued support and engagement on the evidence programme and the review. Special thanks to Molly Abbruzzese whose guidance helped shape the scope of our evidence programme and aided the conceptualisation of this review. We thank Lisa Menning, Danielle Pedi, Jennifer (Fluder) Siler and Pedja Stocjicic for their valuable feedback on the review.

We wish to thank John Eyers, Yoav Vardy, Daniela Anda, Shradha Parsekar, Sejal Luthra, Yue Zhan, Aditi Hombali, Lalitha Vadrevu, Reva Datar, Pankhuri Jha, Beáta Berkovics, Ashton Baafi, Meital Kupfer David Atika, Himani Aggarwal, Agrima Sahore, Mansi Wadhwa, and Harini Narayanan for excellent support and research assistance. We would further like to thank Marie Gaarder, Executive Director, Birte Snilstevit, Director of Synthesis and Reviews Office and Sebastian Martinez, Director of Evaluation Office, 3ie, for their guidance on the review process.

References

1. Ozawa S, Clark S, Portnoy A, et al. Estimated economic impact of vaccinations in 73 low-and middle-income countries, 2001–2020. Bulletin of the World Health Organization. 2017;95(9):629. doi: 10.2471/BLT.16.178475.
2. World Health Organization. Immunization Coverage. World Health Organisation. 2019 Available from: https://www.who.int/news-room/fact-sheets/detail/immunization-coverage.
3. World health Organization. Immunization Agenda 2030: A Global Strategy to Leave No One Behind. World Health Organization, Geneva. 2020 Available from: https://www.who.int/teams/immunization-vaccines-and-biologicals/strategies/ia2030

For peer review only - http://bmjopen.bmj.com/site/about/guidelines.xhtml

4. World Health Organization. WHO and UNICEF warn of a decline in vaccinations during COVID-19. Geneva/New York: World Health Organization, 2020. Available from: https://www.who.int/news/item/15-07-2020-who-and-unicef-warn-of-a-decline-in-vaccinations-during-covid-19
5. UNICEF. Maintaining routine immunization services vital during the COVID-19 pandemic – WHO and UNICEF. Geneva/ Copenhagen: UNICEF, 2020. Available from: https://www.unicef.org/eca/node/5936
6. World Health Organization. Community engagement: a health promotion guide for universal health coverage in the hands of the people. Geneva: World Health Organization; 2020. Licence: CC BY-NC-SA 3.0 IGO. Available from https://www.who.int/publications/i/item/9789240010529
7. Brunton G, Thomas J, O'Mara-Eves A, Jamal F, Oliver S, Kavanagh J. Narratives of community engagement: a systematic review-derived conceptual framework for public health interventions. BMC Public Health 2017;17(1):944. doi: 10.1186/s12889-017-4958-4.
8. Jain M, Engelbert M, Gaarder M, Bagai A, Eyers J. PROTOCOL: Use of community participation interventions to improve child immunisation in low-and middle-income countries: A systematic review and meta-analysis. Campbell Systematic Reviews. 2020;16(4). doi: 10.1002/cl2.1119.
9. The Steering Group of the Campbell Collaboration. Campbell Systematic Reviews: Policies and Guidelines. The Campbell Collaboration. 2017.
10. Hammerstrøm K, Wade A, Jørgensen A.-MK. Searching for studies: A guide to information retrieval for Campbell Systematic Reviews. Campbell Systematic Reviews 2010;Supplement 1. Available at: www.campbellcollaboration.org/lib/download/969/
11. Higgins J, Green S. Cochrane handbook for systematic reviews of interventions. (version 5.0.2, updated September 2009). The Cochrane Collaboration. 2011.
12. Shadish W, Myers D. Research design policy brief. Oslo: Campbell Collaboration 2014. Available from: http://www.campbellcollaboration.org/artman2/uploads/1/C2_Research_Design_Policy_Brief-2.pdf
13. White H. Theory-based Impact Evaluation: Principles and Practice. Working paper series No. 3. New Delhi: 3ie. 2009.

For peer review only - http://bmjopen.bmj.com/site/about/guidelines.xhtml

14. Snilstveit B. Systematic reviews: from 'bare bones' reviews to policy relevance. Journal of development effectiveness. 2012;4(3):388-408. doi: 10.1080/19439342.2012.709875.
15. Waddington H, Aloe AM, Becker BJ, et al. Quasi-experimental study designs series—paper 6: risk of bias assessment. Journal of Clinical Epidemiology. 2017;89:43-52. doi: 10.1016/j.jclinepi.2017.02.015.
16. Sterne JAC, Savović J, Page MJ, et al. RoB 2: a revised tool for assessing risk of bias in randomised trials. BMJ 2019;366:l4898. doi: 10.1136/bmj.l4898.
17. Sterne JAC, Hernán MA, Reeves BC, et al. ROBINS-I: a tool for assessing risk of bias in non-randomized studies of interventions. BMJ 2016; 355; i4919; doi: 10.1136/bmj.i4919.
18. Critical Appraisal Skills Programme. CASP Qualitative Checklist. 2018. Available at: https://casp-uk.b-cdn.net/wp-content/uploads/2018/03/CASP-Qualitative-Checklist-2018_fillable_form.pdf.
19. Doocy S, Tappis H. Cash-based approaches in humanitarian emergencies: a systematic review. Campbell Systematic Reviews. 2017;13(1):1-200. doi: 10.4073/csr.2017.17.
20. Shemilt I, Mugford M, Byford S, et al. The Campbell Collaboration Economics Methods Policy Brief. Oslo: Campbell Collaboration. 2008. http://www.campbellcollaboration.org/artman2/uploads/1/Economic_Methods_Policy_Brief.pdf
21. Drummond MF, Sculpher MJ, Claxton K, Stoddart GL, Torrance GW. Methods for the economic evaluation of health care programmes. Oxford: Oxford university press, 2015.
22. Borenstein M, Hedges LV, Higgins JPT, Rothstein H. Introduction to Meta Analysis (Statistics in Practice). Chichester: John Wiley & Sons, 2009.
23. Ellis PD. The essential guide to effect sizes: Statistical power, meta-analysis, and the interpretation of research results. Cambridge: Cambridge University Press, 2010.
24. Keef SP, Roberts LA. The meta-analysis of partial effect sizes. British Journal of Mathematical and Statistical Psychology. 2004;57(1):97-129. doi: 10.1348/000711004849303.
25. DerSimonian R, Laird N. Meta-analysis in clinical trials. Controlled clinical trials 1986;7(3):177-88. doi:10.1016/0197-2456(86)90046-2
26. Cochran WG. The combination of estimates from different experiments. Biometrics 1954;10:101-129. doi: 10.2307/3001666.

For peer review only - http://bmjopen.bmj.com/site/about/guidelines.xhtml

27. Higgins JPT, Thompson SG. 2002. Quantifying heterogeneity in a meta-analysis. Statistics in medicine 2002;21(11):1539-58. doi:10.1002/sim.1186.
28. R Core Team. R: A language and environment for statistical computing. Vienna, Austria: R Foundation for Statistical Computing, 2018. Available from https://www.R-project.org/
29. Viechtbauer W. Conducting meta-analyses in r with the metafor package. Journal of Statistical Software 2010;36(3):1–48. doi: 10.18637/jss.v036.i03
30. Admassie A, Abebaw D, Woldemichael AD. Impact evaluation of the Ethiopian health services extension programme. Journal of Development Effectiveness. 2009;1(4):430-49. doi: 10.1080/19439340903375724.
31. Adamu AA, Uthman OA, Gadanya MA, Wiysonge CS. Implementation and evaluation of a collaborative quality improvement program to improve immunization rate and reduce missed opportunities for vaccination in primary health-care facilities: a time series study in Kano, Nigeria. Expert review of vaccines. 2019;18(9):969-91. doi: 10.1080/14760584.2019.1647782
32. Alhassan RK, Nketiah-Amponsah E, Ayanore MA, Afaya A, Salia SM, Milipaak J, Ansah EK, Owusu-Agyei S. Impact of a bottom-up community engagement intervention on maternal and child health services utilization in Ghana: a cluster randomised trial. BMC public health. 2019;19(1):1-1. doi: 10.1186/s12889-019-7180-8.
33. Andersson N, Cockcroft A, Ansari NM, et al. Evidence-based discussion increases childhood vaccination uptake: a randomised cluster controlled trial of knowledge translation in Pakistan. BMC International Health and Human Rights. 2009;9(1):1-9. doi: 10.1186/1472-698X-9-S1-S8
34. Arifeen SE, Hoque DE, Akter T, et al. Effect of the Integrated Management of Childhood Illness strategy on childhood mortality and nutrition in a rural area in Bangladesh: a cluster randomised trial. The Lancet. 2009;374(9687):393-403. doi: 10.1016/S0140-6736(09)60828-X.
35. Assegaai T, Reagon G, Schneider H. Evaluating the effect of ward-based outreach teams on primary healthcare performance in North West Province, South Africa: A plausibility design using routine data. South African Medical Journal. 2018;108(4):329-35. doi: 10.7196/SAMJ.2017.v108i4.12755.

For peer review only - http://bmjopen.bmj.com/site/about/guidelines.xhtml

36. Banerjee AV, Duflo E, Glennerster R, Kothari D. Improving immunisation coverage in rural India: clustered randomised controlled evaluation of immunisation campaigns with and without incentives. The BMJ. 2010;340:c2220. doi: 10.1136/bmj.c2220.
37. Banerjee A, Chandrasekhar A, Duflo E, et al. Evaluating the Impact of Interventions to Improve Full Immunisation Rates in Haryana, India. Impact Evaluation Report 126. New Delhi: International Initiative for Impact Evaluation (3ie) 2020. https://doi.org/10.23846/TW10IE126.
38. Banwat ME, Lar LA, Abok IA, Yiltok ES. Effect of peer education on knowledge, attitude and completeness of childhood routine immunization in a rural community of Plateau State. Research Journal of Health Sciences. 2015;3(4):264-74.
39. Biemba G, Yeboah-Antwi K, Vosburg KB, et al. Effect of deploying community health assistants on appropriate treatment for diarrhoea, malaria and pneumonia: quasi-experimental study in two districts of Zambia. Tropical Medicine & International Health. 2016;21(8):985-94. doi: 10.1111/tmi.12730.
40. Björkman M, Svensson J. Power to the people: evidence from a randomized field experiment on community-based monitoring in Uganda. The Quarterly Journal of Economics. 2009;124(2):735-69. doi: 10.1162/qjec.2009.124.2.735
41. Bolam A, Manandhar DS, Shrestha P, Ellis M, Anthony MD. The effects of postnatal health education for mothers on infant care and family planning practices in Nepal: a randomised controlled trial. Bmj. 1998;316(7134):805-11. doi: 10.1136/bmj.316.7134.805.
42. Borkum E, Rangarajan A, Rotz D, Sridharan S, Sethi S, Manorajini M. Evaluation of the Team-Based Goals and Performance-Based Incentives (TBGI) Innovation in Bihar. Mathematica Policy Research; 2014. https://ideas.repec.org/p/mpr/mprres/d8e1097122ff47a6bf42580c82677834.html.
43. Carmichael S, Mehta K, Raheel H, et al. Effects of team-based goals and non-monetary incentives on front-line health worker performance and maternal health behaviours: a cluster randomised controlled trial in Bihar, India. BMJ Global Health. 2019;4(4):e001146. doi: :10.1136/bmjgh-2018-001146
44. Calderón-Ortiz, R, Jesus M. Estrategia de contratacion permanente dentro del Programa de Vacunacion Universa. Salud Pública de México 1996;38(4):243-48. https://www.redalyc.org/articulo.oa?id=10638404

For peer review only - http://bmjopen.bmj.com/site/about/guidelines.xhtml

45. Carnell MA, Dougherty L, Pomeroy AM, Karim AM, Mekonnen YM, Mulligan BE. Effectiveness of scaling up the 'Three Pillars' approach to accelerating MDG 4 progress in Ethiopia. Journal of health, population, and nutrition. 2014;32(4):549.

46. Costa-Font J, Parmar D. Political agency and public healthcare: Evidence from India. WIDER Working Paper; 2016;number 6640. Munich: UNU-WIDER. https://doi.org/10.35188/UNU-WIDER/2016/179-6.

47. Demilew A, Girma M, McElwee E, Datta S, Barofsky J, Disasa T. Improving Immunisation through Supportive Feedback and Nonmonetary Incentives: Impact Evaluation in Ethiopia. Impact Evaluation Report. New Delhi: International Initiative for Impact Evaluation (3ie) 2020.

48. Dipeolu IO. Effect of Mobile-Phone Reminder Test Messages on Mothers' Knowledge and Completion of Routine Immunisation in Rural Areas of Oyo State, Nigeria. PhD Thesis, Ibadan, Nigeria: University of Ibadan 2017. https://library.adhl.africa/handle/123456789/11865.

49. Domek GJ, Contreras-Roldan IL, Bull S, et al. Text message reminders to improve infant immunization in Guatemala: A randomized clinical trial. Vaccine. 2019;37(42):6192-200. doi: 10.1016/j.vaccine.2019.08.046.

50. Engineer CY, Dale E, Agarwal A, et al. Effectiveness of a pay-for-performance intervention to improve maternal and child health services in Afghanistan: a cluster-randomized trial. International journal of epidemiology. 2016;45(2):451-9. doi: 10.1093/ije/dyv362.

51. Findley SE, Uwemedimo OT, Doctor HV, Green C, Adamu F, Afenyadu GY. Early results of an integrated maternal, newborn, and child health program, Northern Nigeria, 2009 to 2011. BMC Public Health. 2013;13(1):1-2. doi: 10.1186/1471-2458-13-1034

52. Gibson DG, Ochieng B, Kagucia EW, et al. Mobile phone-delivered reminders and incentives to improve childhood immunisation coverage and timeliness in Kenya (M-SIMU): a cluster randomised controlled trial. The Lancet Global Health. 2017;5(4):e428-38. doi: 10.1016/S2214-109X(17)30072-4.

53. Goel S, Dogra V, Gupta SK, et al. Effectiveness of Muskaan Ek Abhiyan (the smile campaign) for strengthening routine immunization in Bihar, India. Indian pediatrics. 2012;49(2):103-8. doi: 10.1007/s13312-012-0023-7.

For peer review only

For peer review only - http://bmjopen.bmj.com/site/about/guidelines.xhtml

54. Gurley N, Shearer J, Srivastava Y, Mahapatra S, Desmond M. Impacts of community-led video education to increase vaccination coverage in Uttar Pradesh, India: A mixed methods randomised controlled trial. Impact Evaluation Report 125. 2020th ed. New Delhi: International Initiative for Impact Evaluation (3ie). https://doi.org/10.23846/TW10IE125.

55. Herrera-Almanza C, Rosales-Rueda MF. Reducing the Cost of Remoteness: Community-Based Health Interventions and Fertility Choices. Journal of Health Economics. 2020;73:102365. doi: 10.1016/j.jhealeco.2020.102365.

56. Igarashi K, Sasaki S, Fujino Y, et al. The impact of an immunization programme administered through the Growth Monitoring Programme Plus as an alternative way of implementing Integrated Management of Childhood Illnesses in urban-slum areas of Lusaka, Zambia. Transactions of the Royal Society of Tropical Medicine and Hygiene. 2010;104(9):577-82. doi:10.1016/j.trstmh.2010.05.008

57. Janssens W. Externalities in program evaluation: the impact of a women's empowerment program on immunization. Journal of the European Economic Association. 2011;9(6):1082-113. doi: 10.1111/j.1542-4774.2011.01041.x.

58. Johri M, Chandra D, Kone KG, et al. Social and Behavior Change Communication Interventions Delivered Face-to-Face and by a Mobile Phone to Strengthen Vaccination Uptake and Improve Child Health in Rural India: Randomized Pilot Study. JMIR mHealth and uHealth. 2020;8(9):e20356. doi: 10.2196/20356.

59. Lee SS. Three field experiments on incentives for health workers. PhD Thesis, Cambridge: Harvard University; 2015. https://dash.harvard.edu/handle/1/17467500.

60. Mayumana I, Borghi J, Anselmi L, Mamdani M, Lange S. Effects of Payment for Performance on accountability mechanisms: Evidence from Pwani, Tanzania. Social Science & Medicine. 2017;179:61-73. doi: 10.1016/j.socscimed.2017.02.022.

61. Memon ZA, Khan GN, Soofi SB, Baig IY, Bhutta ZA. Impact of a community-based perinatal and newborn preventive care package on perinatal and neonatal mortality in a remote mountainous district in Northern Pakistan. BMC pregnancy and childbirth. 2015;15(1):1-9. doi: 10.1186/s12884-015-0538-8.

62. Modi D, Dholakia N, Gopalan R, et al. mHealth intervention “ImTeCHO” to improve delivery of maternal, neonatal, and child care services—A cluster-randomized trial in tribal areas of Gujarat, India. PLoS medicine. 2019;16(10):e1002939. doi: 10.1371/journal.pmed.1002939.

For peer review only - http://bmjopen.bmj.com/site/about/guidelines.xhtml

63. Mohanan M, Rajan VS, Swanson K, Thirumurthy H. Information and Facilitation Interventions for Accountability in Health and Nutrition: Evidence from a Randomized Trial in India. Economic Research Initiatives at Duke (ERID) Working Paper. Durham: Duke University; 2020(295). https://www.ssrn.com/abstract=3544786.

64. More NS, Bapat U, Das S, et al. Community mobilization in Mumbai slums to improve perinatal care and outcomes: a cluster randomized controlled trial. PLoS medicine. 2012;9(7):e1001257. doi: 10.1371/journal.pmed.1001257.

65. More NS, Das S, Bapat U, et al. Community resource centres to improve the health of women and children in informal settlements in Mumbai: a cluster-randomised, controlled trial. The Lancet Global Health. 2017;5(3):e335-49. doi: 10.1016/S2214-109X(16)30363-1.

66. Morris SS, Flores R, Olinto P, Medina JM. Monetary incentives in primary health care and effects on use and coverage of preventive health care interventions in rural Honduras: cluster randomised trial. The Lancet. 2004;364(9450):2030-7 doi: 10.1016/S0140-6736(04)17515-6.

67. Murthy N, Chandrasekharan S, Prakash MP, et al. The Impact of an mHealth voice message service (mMitra) on infant care knowledge, and practices among low-income women in India: findings from a pseudo-randomized controlled trial. Maternal and child health journal. 2019;23(12):1658-69. doi: 10.1007/s10995-019-02805-5.

68. Nagar R, Venkat P, Stone LD, Engel KA, Sadda P, Shahnawaz M. A cluster randomized trial to determine the effectiveness of a novel, digital pendant and voice reminder platform on increasing infant immunization adherence in rural Udaipur, India. Vaccine. 2018;36(44):6567-77. doi: 10.1016/j.vaccine.2017.11.023.

69. Nagar R, Ambiya MS, Singh P, et al. A Mixed-Methods Evaluation to Determine the Effects of a Novel MHealth Platform for Maternal Child Health Tracking in Rural Udaipur, India." 3ie Series Report 129. New Delhi: International Initiative for Impact Evaluation (3ie); 2020. https://developmentevidence.3ieimpact.org/search-result-details/impact-evaluation-repository/a-mixed-methods-evaluation-to-determine-the-effects-of-a-novel-mhealth-platform-for-maternal-child-health-tracking-in-rural-udaipur-india/8984.

70. Nzioki JM, Ouma J, Ombaka JH, Onyango RO. Community health worker interventions are key to optimal infant immunization coverage, evidence from a pretest-posttest experiment in Mwingi, Kenya. Pan African Medical Journal. 2017;28(1). doi: 10.11604/pamj.2017.28.21.11255.

For peer review only - http://bmjopen.bmj.com/site/about/guidelines.xhtml

71. Oche MO, Umar AS, Ibrahim MT, Sabitu K. An assessment of the impact of health education on maternal knowledge and practice of childhood immunization in Kware, Sokoto State. Journal of Public Health and Epidemiology. 2011;3(10):440-7. https://academicjournals.org/article/article1379497483_Oche%20et%20al.pdf.

72. Okeke E, Glick P, Abubakar IS, et al. Better Obstetrics in Rural Nigeria: Evaluating the Midwives Service Scheme. 3ie Impact Evaluation Report 56. International Initiative for Impact Evaluation (3ie); 2017. https://www.3ieimpact.org/evidence-hub/publications/impact-evaluations/better-obstetrics-rural-nigeria-evaluating-midwives.

73. Okoli U, Morris L, Oshin A, Pate MA, Aigbe C, Muhammad A. Conditional Cash Transfer Schemes in Nigeria: Potential Gains for Maternal and Child Health Service Uptake in a National Pilot Programme. BMC Pregnancy and Childbirth 2014;14(1):408. doi: 10.1186/s12884-014-0408-9.

74. Olayo R, Wafula C, Aseyo E, Loum C, Kaseje D. A quasi-experimental assessment of the effectiveness of the Community Health Strategy on health outcomes in Kenya. BMC health services research. 2014;14(1):1-3. doi: 10.1186/1472-6963-14-S1-S3.

75. Olken BA, Onishi J, Wong S. Should aid reward performance? Evidence from a field experiment on health and education in Indonesia. American Economic Journal: Applied Economics. 2014;6(4):1-34. doi: 10.1257/app.6.4.1.

76. Oyo-Ita, A, Bosch-Capblanch X, Ross A, et al. Impacts of Engaging Communities through Traditional and Religious Leaders on Vaccination Coverage in Cross River State, Nigeria. Impact Evaluation Report 127. 2020th ed. New Delhi: International Initiative for Impact Evaluation (3ie); 2020. https://doi.org/10.23846/TW10IE127.

77. Pramanik S, Ghosh A, Goswami A, et al. Impacts of the Stimulate, Appreciate, Learn, and Transfer community engagement approach to increase immunisation coverage in Assam, India, 3ie Grantee Final Report. New Delhi: International Initiative for Impact Evaluation (3ie); 2020.

78. Rahman A, Malik A, Sikander S, Roberts C, Creed F. Cognitive behaviour therapy-based intervention by community health workers for mothers with depression and their infants in rural Pakistan: a cluster-randomised controlled trial. The Lancet. 2008;372(9642):902-9. doi: 10.1016/S0140-6736(08)61400-2.

For peer review only - http://bmjopen.bmj.com/site/about/guidelines.xhtml

79. Rahman M, Yunus FM, Shah R, et al. A controlled before-and-after perspective on the improving maternal, neonatal, and child survival program in rural Bangladesh: an impact analysis. PLoS One. 2016;11(9):e0161647. doi: 10.1371/journal.pone.0161647.
80. Rao T. The Impact of a Community Health Worker Program on Childhood Immunization: Evidence from India's 'ASHA' Workers. Available at SSRN 2444391. 2014. http://www.ssrn.com/abstract=2444391.
81. Robertson L, Mushati P, Eaton JW, et al. Effects of unconditional and conditional cash transfers on child health and development in Zimbabwe: a cluster-randomised trial. The Lancet. 2013;381(9874):1283-92. doi: 10.1016/S0140-6736(12)62168-0.
82. Roy SK, Bilkes F, Islam K, et al. Impact of pilot project of Rural Maintenance Programme (RMP) on destitute women: CARE, Bangladesh. Food and nutrition bulletin. 2008;29(1):67-75. doi: 10.1177/156482650802900108.
83. Saggurti N, Atmavilas Y, Porwal A, et al. Effect of health intervention integration within women's self-help groups on collectivization and healthy practices around reproductive, maternal, neonatal and child health in rural India. PLoS One. 2018;13(8):e0202562. doi: 10.1371/journal.pone.0202562.
84. Sankar D. Improving Early Childhood Development through Community Mobilization and Integrated Planning for Children. Results from the Evaluation of Bachpan Program, Ratlam District, Madhya Pradesh, India. Discussion Paper Series 59. Washington, D.C.: The World Bank; 2013. http://crossasia-repository.ub.uni-heidelberg.de/3467/.
85. Seth R, Akinboyo I, Chhabra A, et al. Mobile phone incentives for childhood immunizations in rural India. Pediatrics. 2018;141(4):e20173455. doi: 10.1542/peds.2017-3455.
86. Shukla M. Impact of a health governance intervention on provincial health system performance in Afghanistan: a quasi-experimental study. Health systems & reform. 2018;4(3):249-66. doi: 10.1080/23288604.2018.1477536.
87. Siddiqi DA, Ali RF, Munir M, Shah MT, Khan AJ, Chandir S. Effect of vaccine reminder and tracker bracelets on routine childhood immunization coverage and timeliness in urban Pakistan (2017-18): a randomized controlled trial. BMC public health. 2020;20(1):1086. doi: 10.1186/s12889-020-09088-4.

For peer review only - http://bmjopen.bmj.com/site/about/guidelines.xhtml

88. Tandon BN, Sahai A. Immunization in India: contribution of Integrated Child Development Services scheme to expanded programme of immunization. Journal of tropical pediatrics. 1988;34(6):309-12. doi: 10.1093/tropej/34.6.309.

89. USAID. Essential Services for Health in Ethiopia: Final Report. Washington, DC: USAID; 2008. https://publications.jsi.com/JSIInternet/Inc/Common/_download_pub.cfm?id=10111&lid=3.

90. Webster J, Landegger J, Bruce J, et al. Impacts of IRC's Fifth Child Community Engagement Strategy to Increase Immunisation in Northern Uganda. 3ie Grantee Final Report. New Delhi: International Initiative for Impact Evaluation (3ie); 2019. https://www.3ieimpact.org/sites/default/files/2019-02/gfr-TW10.1018-IRC-Imunization-Program-Uganda.pdf.

91. Younes L, Houweling TA, Azad K, et al. The effect of participatory women's groups on infant feeding and child health knowledge, behaviour and outcomes in rural Bangladesh: a controlled before-and-after study. J Epidemiol Community Health. 2015 Apr 1;69(4):374-81. doi: 10.1136/jech-2014-204271.

92. Full list of included qualitative studies and excluded impact evaluations are provided in Appendix 15 pp. 216-231.

Supplementary material

Supplementary material includes

1. Appendix 1: Overview of community engagement framework
2. Appendix 2: Full search terms for one database.
3. Appendix 3: Qualitative and cost-evidence search strategy
4. Appendix 4: Data extraction tools
5. Appendix 5: Risk of bias assessment tools
6. Appendix 6: Risk of bias in the included studies
7. Appendix 7: Quantitative results – all community engagement interventions
8. Appendix 8: Quantitative results – Engagement as intervention
9. Appendix 9: Quantitative results - community engagement in intervention design
10. Appendix 10: Community engagement in implementation autonomy of interventions
11. Appendix 11: Multiple engagement types
12. Appendix 12: Qualitative synthesis of results

For peer review only - http://bmjopen.bmj.com/site/about/guidelines.xhtml

13. Appendix 13: Synthesis of cost evidence
14. Appendix 14: Bibliography (including List of studies excluded at full-text screening stage, with brief reasons)
15. Appendix 15: Amendments to information provided in the protocol

Figure legends

Figure 1. PRISMA flow diagram

Figure 2. Forest plot showing the observed outcomes and the estimate of the random-effects model for the impact of community engagement interventions on full childhood immunisation. Note that # (number of) Participants is specific to each effect and thus may not reflect the sample size for the full study.

Figure 3. Forest plot showing the observed outcomes and the estimate of the random-effects model for the impact of community engagement interventions on DPT3 vaccination. Note that # Participants is specific to each effect and thus may not reflect the sample size for the full study.

Figure 4. Forest plot showing the observed outcomes and the estimate of the random-effects model for the impact of community engagement interventions on measles vaccination. Note that # Participants is specific to each effect and thus may not reflect the sample size for the full study.

For peer review only - http://bmjopen.bmj.com/site/about/guidelines.xhtml

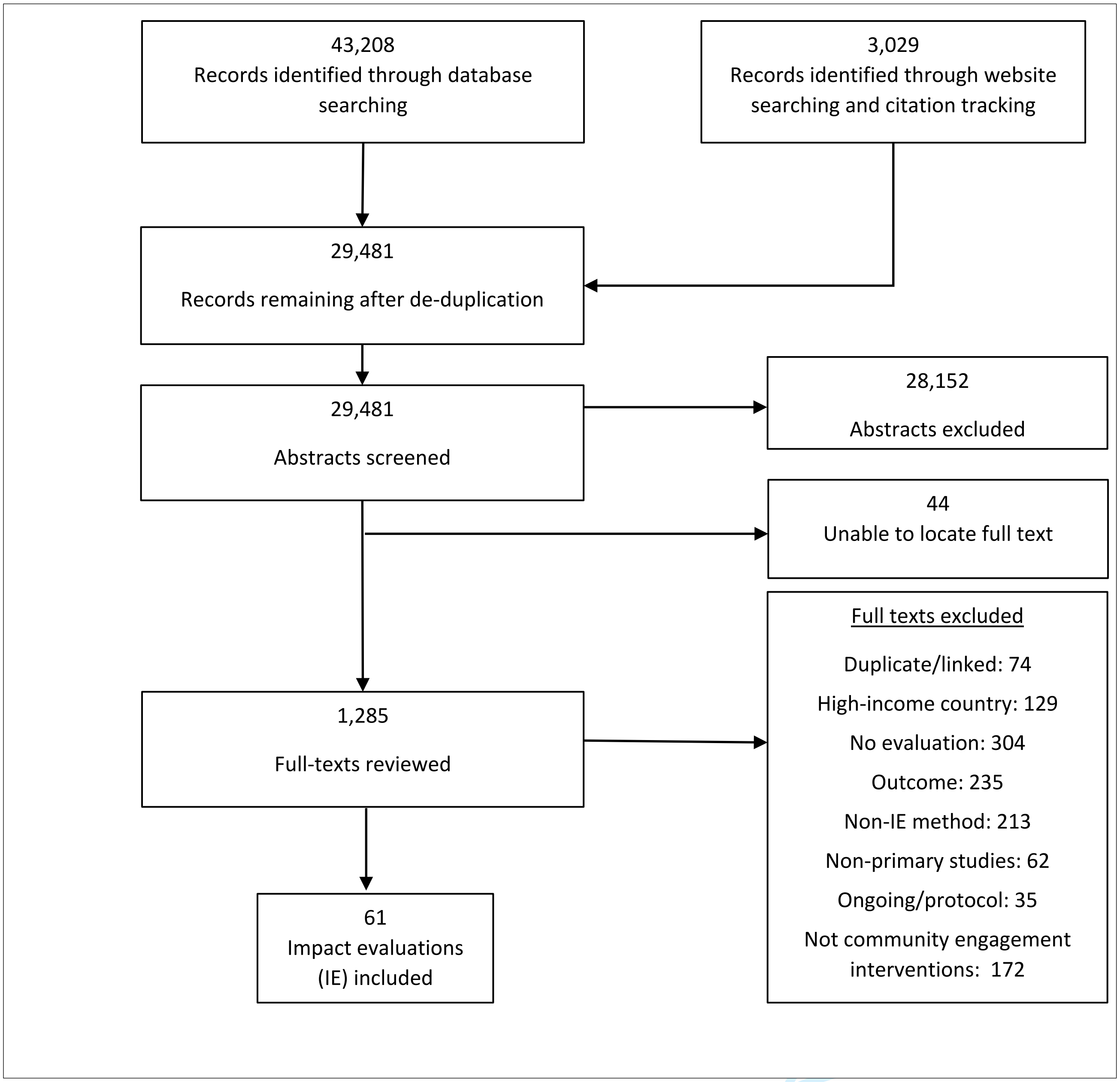
43,208
Records identified through database searching
3,029
Records identified through website searching and citation tracking
29,481
Records remaining after de-duplication
29,481
Abstracts screened
28,152
Abstracts excluded
44
Unable to locate full text
1,285
Full-texts reviewed
Full texts excluded
Duplicate/linked: 74
High-income country: 129
No evaluation: 304
Outcome: 235
Non-IE method: 213
Non-primary studies: 62
Ongoing/protocol: 35
Not community engagement interventions: 172
61
Impact evaluations (IE) included

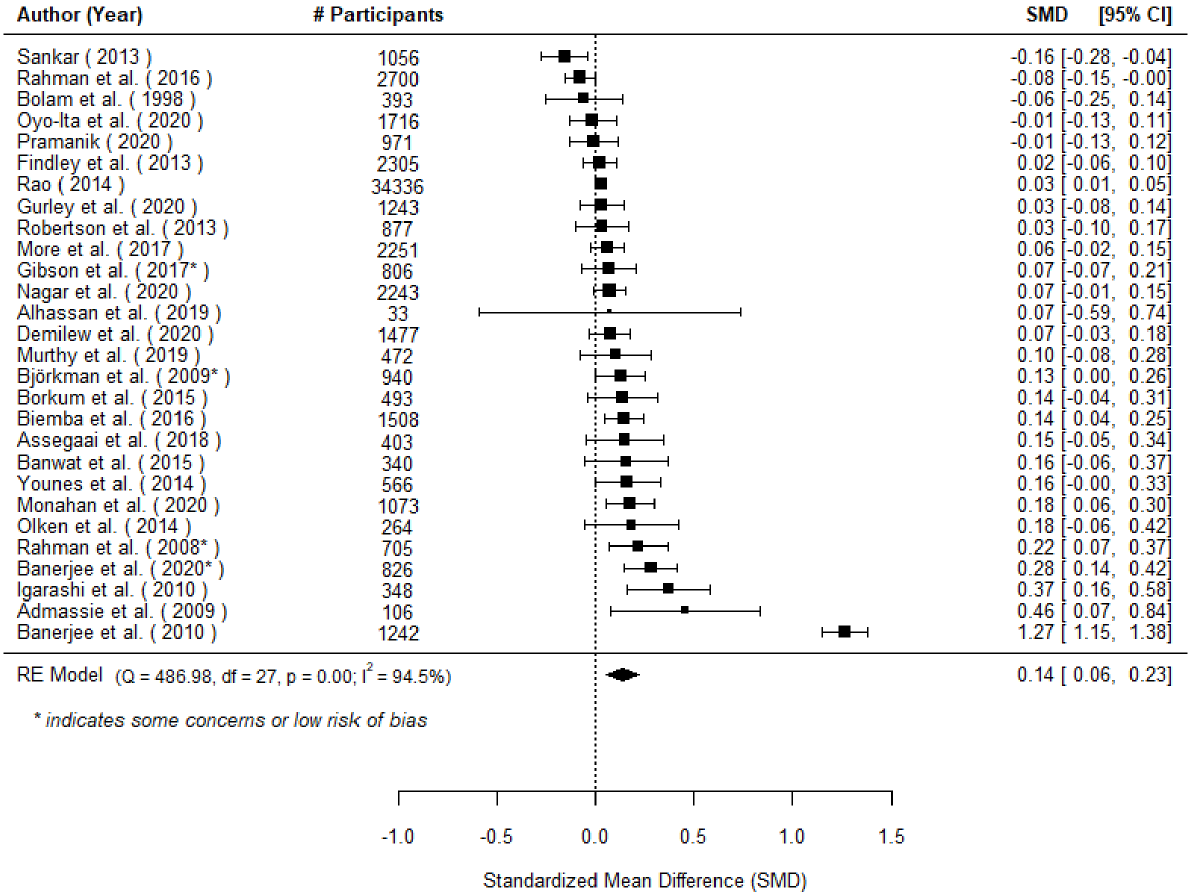
Author (Year)
Participants
SMD [95% CI]
Sankar (2013) 1056 -0.16 [-0.28, -0.04]
Rahman et al. (2016) 2700 -0.08 [-0.15, -0.00]
Bolam et al. (1998) 393 -0.06 [-0.25, 0.14]
Oyo-Ita et al. (2020) 1716 -0.01 [-0.13, 0.11]
Pramanik (2020) 971 -0.01 [-0.13, 0.12]
Findley et al. (2013) 2305 0.02 [-0.06, 0.10]
Rao (2014) 34336 0.03 [0.01, 0.05]
Gurley et al. (2020) 1243 0.03 [-0.08, 0.14]
Robertson et al. (2013) 877 0.03 [-0.10, 0.17]
More et al. (2017) 2251 0.06 [-0.02, 0.15]
Gibson et al. (2017*) 806 0.07 [-0.07, 0.21]
Nagar et al. (2020) 2243 0.07 [-0.01, 0.15]
Alhassan et al. (2019) 33 0.07 [-0.59, 0.74]
Demilew et al. (2020) 1477 0.07 [-0.03, 0.18]
Murthy et al. (2019) 472 0.10 [-0.08, 0.28]
Björkman et al. (2009*) 940 0.13 [0.00, 0.26]
Borkum et al. (2015) 493 0.14 [-0.04, 0.31]
Biemba et al. (2016) 1508 0.14 [0.04, 0.25]
Assegaai et al. (2018) 403 0.15 [-0.05, 0.34]
Banwat et al. (2015) 340 0.16 [-0.06, 0.37]
Younes et al. (2014) 566 0.16 [-0.00, 0.33]
Monahan et al. (2020) 1073 0.18 [0.06, 0.30]
Olken et al. (2014) 264 0.18 [-0.06, 0.42]
Rahman et al. (2008*) 705 0.22 [0.07, 0.37]
Banerjee et al. (2020*) 826 0.28 [0.14, 0.42]
Igarashi et al. (2010) 348 0.37 [0.16, 0.58]
Admassie et al. (2009) 106 0.46 [0.07, 0.84]
Banerjee et al. (2010) 1242 1.27 [1.15, 1.38]
RE Model (Q = 486.98, df = 27, p = 0.00; I2 = 94.5%) 0.14 [0.06, 0.23]
* indicates some concerns or low risk of bias
-1.0 -0.5 0.0 0.5 1.0 1.5
Standardized Mean Difference (SMD)

review only

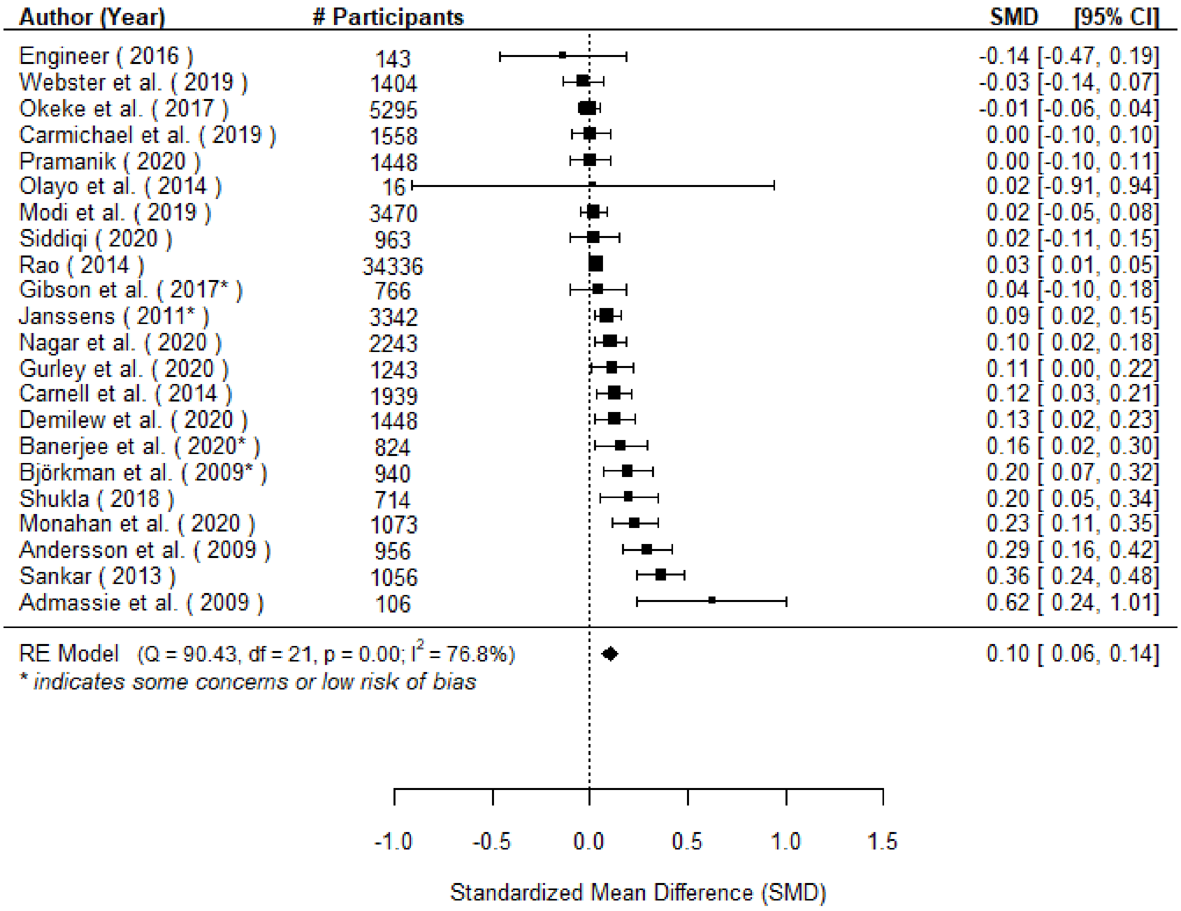
Author (Year)
Participants
SMD [95% CI]
Engineer (2016) 143 -0.14 [-0.47, 0.19]
Webster et al. (2019) 1404 -0.03 [-0.14, 0.07]
Okeke et al. (2017) 5295 -0.01 [-0.06, 0.04]
Carmichael et al. (2019) 1558 0.00 [-0.10, 0.10]
Pramanik (2020) 1448 0.00 [-0.10, 0.11]
Olayo et al. (2014) 16 0.02 [-0.91, 0.94]
Modi et al. (2019) 3470 0.02 [-0.05, 0.08]
Siddiqi (2020) 963 0.02 [-0.11, 0.15]
Rao (2014) 34336 0.03 [0.01, 0.05]
Gibson et al. (2017*) 766 0.04 [-0.10, 0.18]
Janssens (2011*) 3342 0.09 [0.02, 0.15]
Nagar et al. (2020) 2243 0.10 [0.02, 0.18]
Gurley et al. (2020) 1243 0.11 [0.00, 0.22]
Carnell et al. (2014) 1939 0.12 [0.03, 0.21]
Demilew et al. (2020) 1448 0.13 [0.02, 0.23]
Banerjee et al. (2020*) 824 0.16 [0.02, 0.30]
Björkman et al. (2009*) 940 0.20 [0.07, 0.32]
Shukla (2018) 714 0.20 [0.05, 0.34]
Monahan et al. (2020) 1073 0.23 [0.11, 0.35]
Andersson et al. (2009) 956 0.29 [0.16, 0.42]
Sankar (2013) 1056 0.36 [0.24, 0.48]
Admassie et al. (2009) 106 0.62 [0.24, 1.01]
RE Model (Q = 90.43, df = 21, p = 0.00; I² = 76.8%) 0.10 [0.06, 0.14]
* indicates some concerns or low risk of bias
-1.0
-0.5
0.0
0.5
1.0
1.5
Standardized Mean Difference (SMD)

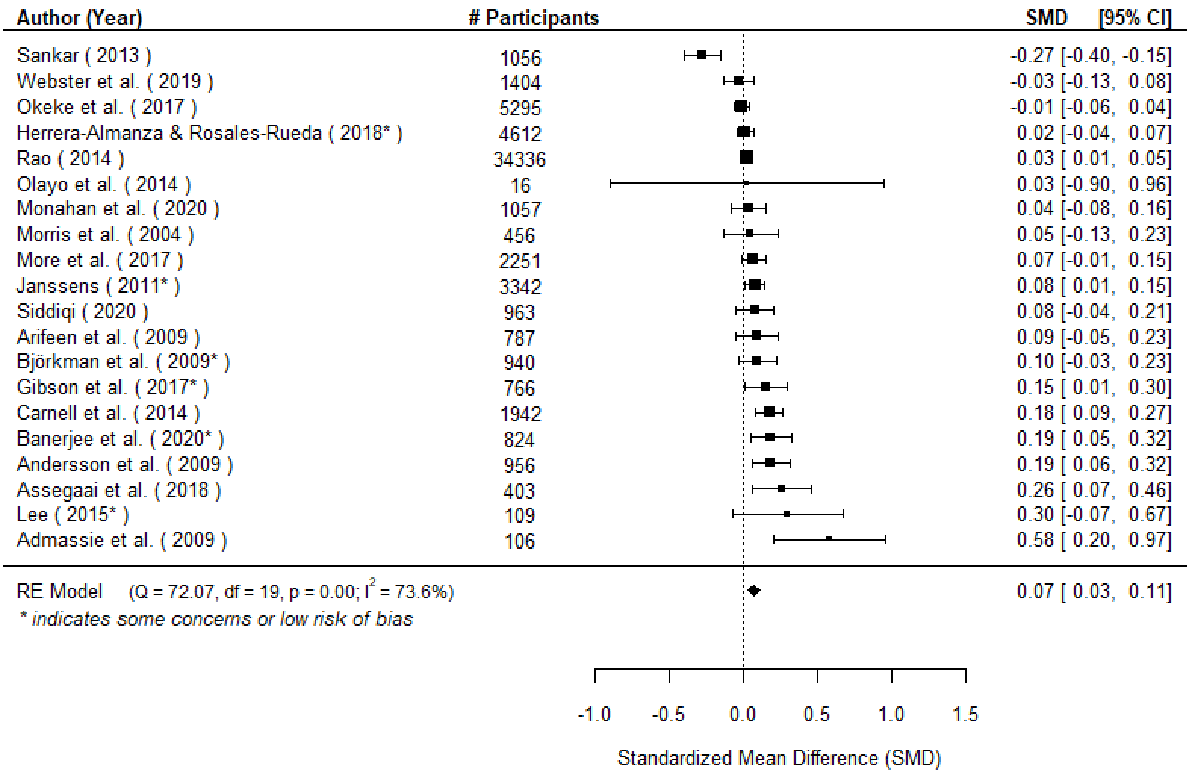
Author (Year)
Participants
SMD [95% CI]
Sankar (2013) 1056 -0.27 [-0.40, -0.15]
Webster et al. (2019) 1404 -0.03 [-0.13, 0.08]
Okeke et al. (2017) 5295 -0.01 [-0.06, 0.04]
Herrera-Almanza & Rosales-Rueda (2018*) 4612 0.02 [-0.04, 0.07]
Rao (2014) 34336 0.03 [0.01, 0.05]
Olayo et al. (2014) 16 0.03 [-0.90, 0.96]
Monahan et al. (2020) 1057 0.04 [-0.08, 0.16]
Morris et al. (2004) 456 0.05 [-0.13, 0.23]
More et al. (2017) 2251 0.07 [-0.01, 0.15]
Janssens (2011*) 3342 0.08 [0.01, 0.15]
Siddiqi (2020) 963 0.08 [-0.04, 0.21]
Arifeen et al. (2009) 787 0.09 [-0.05, 0.23]
Björkman et al. (2009*) 940 0.10 [-0.03, 0.23]
Gibson et al. (2017*) 766 0.15 [0.01, 0.30]
Carnell et al. (2014) 1942 0.18 [0.09, 0.27]
Banerjee et al. (2020*) 824 0.19 [0.05, 0.32]
Andersson et al. (2009) 956 0.19 [0.06, 0.32]
Assegaai et al. (2018) 403 0.26 [0.07, 0.46]
Lee (2015*) 109 0.30 [-0.07, 0.67]
Admassie et al. (2009) 106 0.58 [0.20, 0.97]
RE Model (Q = 72.07, df = 19, p = 0.00; I² = 73.6%) 0.07 [0.03, 0.11]
* indicates some concerns or low risk of bias
-1.0
-0.5
0.0
0.5
1.0
1.5
Standardized Mean Difference (SMD)

review only

Use of community engagement interventions to improve child immunisation in low- and middle-income countries: a systematic review and meta-analysis

Supplementary material

Table of Contents

APPENDIX 1: OVERVIEW OF COMMUNITY ENGAGEMENT FRAMEWORK 3

APPENDIX 2: AMENDMENTS TO INFORMATION PROVIDED IN THE PROTOCOL 9

APPENDIX 3: SEARCH STRATEGY AND SEARCH TERMS FOR IMPACT EVALUATIONS 11

APPENDIX 4: LIST OF EXCLUDED IMPACT EVALUATION STUDIES 16

APPENDIX 5: QUALITATIVE AND COST-EVIDENCE SEARCH STRATEGY 28

Qualitative search protocol 28

Cost evidence search protocol 30

List of included qualitative studies 30

List of included other studies 33

APPENDIX 6: DATA EXTRACTION TOOLS 37

Quantitative data extraction tool 37

Qualitative data extraction tool 42

Cost data extraction tool 57

APPENDIX 7: RISK OF BIAS ASSESSMENT TOOLS 65

Quantitative risk of bias tool 65

Qualitative risk of bias tool 81

Cost evidence risk of bias tool 89

For peer review only

For peer review only - http://bmjopen.bmj.com/site/about/guidelines.xhtml

APPENDIX 8: RISK OF BIAS IN THE INCLUDED STUDIES 90

Quantitative risk of bias 90

Qualitative risk of bias 91

Risk of bias in cost effectiveness estimates 92

APPENDIX 9: QUANTITATIVE RESULTS – ALL COMMUNITY ENGAGEMENT INTERVENTIONS 96

APPENDIX 10: QUANTITATIVE RESULTS – ENGAGEMENT AS INTERVENTION 128

APPENDIX 11: QUANTITATIVE RESULTS - COMMUNITY ENGAGEMENT IN INTERVENTION DESIGN 141

APPENDIX 12: COMMUNITY ENGAGEMENT IN IMPLEMENTATION AUTONOMY OF INTERVENTIONS 155

APPENDIX 13: MULTIPLE ENGAGEMENT TYPES 162

APPENDIX 14: QUALITATIVE SYNTHESIS OF RESULTS 177

Barriers to immunisation 177

Facilitators of immunisation 187

Reasons for intervention success 193

Reasons for intervention failure 203

Reasons for heterogenous impacts 213

Uptake and fidelity challenges 213

APPENDIX 15: SYNTHESIS OF COST EVIDENCE 226

For peer review only

For peer review only - http://bmjopen.bmj.com/site/about/guidelines.xhtml

Appendix 1: Overview of community engagement framework

The focus of our review is community engagement interventions, which are increasingly being emphasised in international and national policy frameworks as a means to improve immunisation coverage and reach marginalised communities (UNICEF 2018, WHO 2015, WHO 2017b; WHO 2017a).

The most common approach for categorizing community engagement interventions is probably the International Association for Public Participation (IAP2) framework (iap2.org), which identifies five levels of engagement ranging from inform to empower corresponding to increasing community influence over the decisions. This framework corresponds to the “social justice perspective” in Brunton et al. (2017) in which the community engagement is rooted in concerns about social justice, which requires that the health needs are identified by communities themselves and they mobilise themselves into action to make changes within the community. However, after pilot testing of the IAP2 framework on a range of community engagement interventions, we determined that most interventions are based on a more “utilitarian perspective.” These two perspectives of community engagement are very well captured and articulated in Brunton et al. (2017), who in their systematic review of community engagement narratives in public health point out that:

“Historically, interventions to promote health were driven by professionals, with little or no input from the targeted populations; more recently, community engagement has become central to national strategy and guidance for promoting public health, because, from a ‘utilitarian’ point of view, it is thought that more acceptable and appropriate interventions will result, which may result in improved service use and outcomes. Interventions that are based on a utilitarian perspective seek to involve communities in order to improve the effectiveness of the intervention. The intervention itself may be decided upon before the community is invited for its views; or, while the intervention itself is not designed by community members they may be involved in other ways, such as priority setting, or in its delivery. In utilitarian perspectives, health (and other) services reach out to engage particular communities that they have identified require assistance and the intervention is devised within existing policy, practice, and resource frameworks.”

Due to the difference in how the IAP2 framework approaches community engagement and how interventions are actually implemented, it was difficult to systematically categorize interventions using the IAP2 framework with a high degree of consistency among coders. In addition, even for the interventions rooted in “social justice” perspective, their description in the studies was too limited to identify them and map their intensity of community engagement without introducing a lot of subjectivity and non-systematicness.

To avoid this misclassification as far as possible, we experimented with different frameworks and ultimately settled on the one which focuses on process of engagement rather than its intensity. We found that focusing on when and how the community is engaged is a more practical framework for the kind of community engagement interventions that have been evaluated in real world settings. Our approach corresponds to some degree to the “extent of engagement” part of the conceptual framework developed in Brunton et al. (2017) and we also kept the spirit of IAP2 framework by including interventions in which engagement goes beyond one-way communication, i.e. beyond the inform level of IAP2, to include some consultation or dialogue with the community or some decision making by the community members. We found this approach to be relatively easier to apply, less subjective, less prone to classification error and potentially useful to practitioners. The development of this process-oriented framework took a period of around one month and involved three of our core team members

For peer review only - http://bmjopen.bmj.com/site/about/guidelines.xhtml

We consider three points within an intervention during which engagement can occur: engagement can occur in the design of the intervention, engagement can occur in the implementation of the intervention, or the intervention may be engagement. We break these categories into further sub-groups representing specific ways through which engagement can occur; for example, the development of new cadres of health workers, pilot studies, and the involvement of the community in governance and decision making.

This framework adds a new dimension to the discussion of types of engagement. As practitioners consider the design of their programs, they can use the evidence provided through this framework to determine when and how best to engage with the community.

Engagement as the intervention (engagement is embedded): In these interventions a serious attempt was made to gain community buy-in for activities or new cadres of community-based structures were established, such as village health committees or community health volunteers. While, in a few cases, the development of new cadres can be solely for the purpose of didactic teaching and one-way communication, it generally results in dynamic discussion and two-way communication. As such, we chose to include these interventions. Interventions which are themselves community engagement can motivate communities to take ownership of service delivery and address local problems with local solutions.

Engagement in the design of interventions: In these interventions community input or feedback was sought before the implementation of an intervention. Such feedback can take the form of a pilot, needs assessment, formative evaluation, or other outreach effort. This form of engagement must occur before the implementer undertakes an action. These interventions allow the community to influence the form of the ultimate action taken. Depending on the weight that is given to the feedback, these engagement activities can align to any of the IAP2 categories other than inform, which was not considered.

Engagement in implementation autonomy of interventions: In many interventions the community is not asked for input in their design, but is utilized in their implementation as health care workers, facilitators, or problem solvers. In the spirit of IAP2 framework to go beyond inform level interventions, we only included those interventions in this category where the community members involved in the implementation of the intervention had some opportunity to affect or influence its implementation. This broadly aligns with the involve, collaborate and empower levels of engagement. Due to the inclusion criteria of some autonomy in implementation, the interventions under this category generally involved an existing community led governance structure which weighed in on implementation decisions or the community providing resources without which the intervention could not be implemented. We excluded interventions in which community members, like community health workers or frontline health workers, were involved in implementation, but no new cadres of health workers were created and they could not influence its implementation. For example, community health workers supplying hygiene kits or doing home visits. In addition, interventions which built capacity of existing cadres of community members or provided supportive supervision were excluded. For example, m-health apps for community workers and training of peer facilitators or community health workers or frontline health workers that only allowed for a one-way transfer of knowledge.

For peer review only - http://bmjopen.bmj.com/site/about/guidelines.xhtml

Examples of engagement types

ENGAGEMENT AS THE INTERVENTION (ENGAGEMENT IS EMBEDDED)

Effect of health intervention integration within women's self-help groups on collectivization and healthy practices around reproductive, maternal, neonatal and child health in rural India

Health-focused self-help groups were created for women of reproductive age in marginalized communities.

Effect of peer education on knowledge, attitude and completeness of childhood routine immunization in a rural community of Plateau State.

A new cadre of peer educators was created. Women were trained to provide women with information about routine childhood immunisation. This does not qualify as engagement in implementation because the intervention was the training of peer educators and not the subsequent actions of the peer educators.

Impacts of engaging communities through traditional and religious leaders on vaccination coverage in Cross River State, Nigeria

Traditional and religious leaders were trained to utilize their leadership role to support immunisation. After the training, these leaders then presented data at ward development committee meetings and engaged with the community to encourage immunsation.

ENGAGEMENT IN THE DESIGN OF AN INTERVENTION

Cognitive behaviour therapy-based intervention by community health workers for mothers with depression and their infants in rural Pakistan: a cluster-randomised controlled trial

Many pilots directly seek the feedback of community members. In this study, health workers and depressed mothers were asked about the relevance and usefulness of the intervention before scaling.

Mobile Phone Incentives for Childhood Immunizations in Rural India

Caregivers were given mobile phone credit incentives for completing imminizations. The amount of the incentive was decided upon through conversations that involved community members.

ENGAGEMENT IN THE IMPLEMENTATION AUTONOMY OF AN INTERVENTION

The impact of an immunization programme administered through the Growth Monitoring Programme Plus as an alternative way of implementing Integrated Management of Childhood Illnesses in urban-slum areas of Lusaka, Zambia

The GMP+ sessions were conducted by medical personnel from Public Health Centers. During these session, community volunteers provided some operational and managerial support to ensure the effective implementation of the sessions.

Effects of payment for performance on accountability mechanisms: Evidence from Pwani, Tanzania

The intervention is a performance-based financing mechanism. The community health committee was involved in decisions about how to spend the funds gained through the pay for performance mechanism.

Interventions are defined based on the actions of the external actors, ie implementers, not the community itself. If an intervention spurred the community to take action, the categorization of the intervention is based on the intervention which spurred the action and not the actions that the community took as a result of the intervention. This is because another community might take different actions as a result of the same intervention. The distinction is especially important for interventions to empower communities to improve their own systems. In these cases, the intervention is the activity that empowered the community; this intervention is engagement. For example, interventions which develop village committees to identify local challenges and solutions are themselves engagement interventions. This is because the intervention is the development of the committee and not the actions of the committee. A similar committee in a different area may choose to take different actions, even if the implementing agency does the same development process. Although the community may have engaged in the design of the committee, they likely were not engaged in the design of development process which brought about the committee. For interventions to be qualified as engagement in the design or implementation, the intervention must be an action by an implementer which the community influenced. For example, community members could be consulted in the design of materials used in

For peer review only - http://bmjopen.bmj.com/site/about/guidelines.xhtml

outreach activities (Murthy 2019, Nagar 2020). In the example of the development of a new committee, the implementer influenced the action of the community, the community did not influence the action of the implementer.

We defined “communities” in reference to the lowest level of the health service delivery system (or whatever level provides routine immunisation services in the local context). A community is a group of people who are served by a particular primary health facility. Thus, communities encompass a wide range of stakeholders, including caregivers, health service providers, and influential community members such as religious or other traditional leaders. Therefore, our review included any intervention that was directed towards any of these types of community members. Interventions that targeted higher levels of the health system, such as state-level officials, were excluded.

How the interventions might work

A 2015 3ie scoping paper (Sabarwal et al. 2015) systematically mapped the literature on immunisation interventions involving community engagement. Several programme managers and policy experts provided insight regarding why community engagement could be the key to improving immunisation outcomes for children in areas where the coverage has stagnated or declined or that are hard to reach. The findings from the scoping study indicate that working with or engaging communities could help develop a better understanding of the context, target population, problems and barriers, and lead to identification of contextually relevant solutions and desired outcomes, and mobilising community support for them. Because individuals usually function under the influence of social norms, efforts to change these norms can be effective in changing behaviour (Bicchieri and Xiao 2009; Reynolds Subašić and Tindall 2015). People respond to their peers and community and, while activities such as information and education campaigns might have some influence, individuals might feel bound by collective decisions, preventing sustained change (Riedy et al. 2012). The role of peers and of social norms in shaping attitudes towards vaccination is particularly important given that vaccine hesitancy has been documented in countries of all income levels (although it takes different forms in different countries; Dubé, Gagnon, Nickels, Jeram, and Schuster 2014). Community engagement may be effective at overcoming these barriers to immunisation. In both high-income countries (HICs; O’Mara-Eves et al. 2013) and L&MICs (De Buck et al. 2017), community engagement has been an effective model of modifying health behaviours in particular. Hence, community engagement could be an important determinant of success or failure of an intervention aimed at improving immunisation coverage.

Due to the variability in contexts, activities to address the barriers to immunisation through community engagement also vary. Therefore, no single theory of change can capture all the different ways that community engagement will affect immunisation outcomes. Furthermore, there is no strict correspondence between types of engagement and intervention activities. For the most part, each activity can be structured such that community engagement is the intervention (engagement is embedded) or the community is engaged in the design or implementation of the intervention. For example, consider an intervention involving village health committees in which community members are adequately represented. These committees can be created by a sponsoring agency with little or no input from the actual community. In this case, the intervention is engagement. On the other hand, community members may develop a village health committee during the design phase of major clinic infrastructure projects to ensure the efficient management of the intervention. Hence, these committees could arise from community involvement in the design of the intervention. In addition, an existing village health committee could be leveraged for the implementation of community meetings at which committee members lead a dialogue on immunisation.

Broadly, we expect that community engagement activities will increase awareness of vaccine-preventable diseases, knowledge of where and when to get vaccinations, and motivation to get vaccinations among caregivers (Figure 1). Community engagement may also increase skill, motivation, and accountability of health workers. These ought to lead to improved demand for and delivery of services, which will increase the number of vaccinated children and could potentially reduce child morbidity and mortality. It is important to note that child mortality and morbidity are affected by critical factors beyond immunisation, such as access to safe food and adequate nutrition, safe water and

For peer review only - http://bmjopen.bmj.com/site/about/guidelines.xhtml

quality care by a trained health provider when needed. Without these critical enabling factors immunisation alone may not be effective in reduction of child mortality and morbidity.

For peer review only

For peer review only - http://bmjopen.bmj.com/site/about/guidelines.xhtml

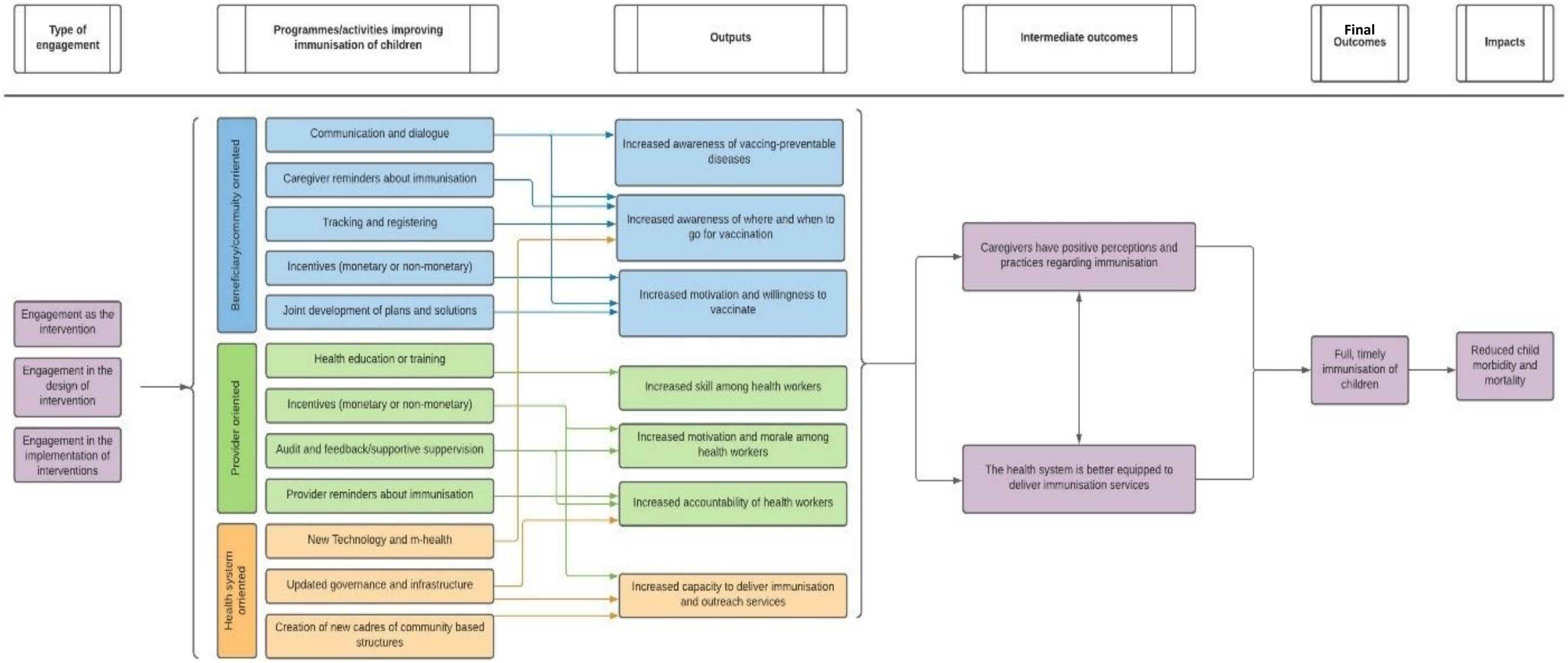

Supplementary Figure 1: Intervention theory of change

Only

Appendix 2: Amendments to information provided in the protocol

1. In the report, we have replaced the word ‘participation’ for ‘engagement’, a change that is also reflected in our review title and objectives. Though the IAP2 framework uses the terms "public participation" and "community engagement" interchangeably, we realise that this may lead to confusion as there may be inter researcher differences in how these terms are perceived and used. Therefore, for maintaining consistency, we have used community engagement throughout the report and avoided any references to community participation. From a linguistic perspective, these words are used interchangeability in our review, as is evident from our protocol (please refer to pp. 1-3). Therefore, this change does not alter the scope of our review in any way. In the protocol we had proposed to use the IAP2 spectrum of community engagement for determination of interventions to be included in the review. However, after pilot testing of the IAP2 framework on a range of community engagement interventions, we found that most interventions are based on a more “utilitarian perspective” put forth in Brunton et al. (2017). Thus, for this review we ultimately settled on the community engagement framework which focuses on process of engagement rather than its intensity. Our approach corresponds to some degree to the “extent of engagement” part of the conceptual framework developed in Brunton et al. (2017) and we also kept the spirit of IAP2 framework by including interventions in which engagement goes beyond one-way communication, i.e. beyond the inform level of IAP2, to include some consultation or dialogue with the community or some decision making by the community members. We found this approach to be relatively easier to apply, less subjective, less prone to classification error and potentially useful to practitioners
2. In the section on objectives, we have clubbed what were originally review questions 3 and 4 in the protocol (p. 5). These questions related to understanding how implementation features and contextual factors such as barriers and facilitators to immunisation, are associated with relative success or failure of community engagement interventions. When we set out to answer these questions through our qualitative synthesis, we realised that all these factors are interlinked and, in various combinations, may influence the outcomes along the causal chain. Therefore, we combined the two objectives and have presented our analysis and results accordingly. This modification to the review objectives does not alter our study’s scope.
3. We had intended to include the relevant Chinese literature in the review. However, because of political disruptions in Hong Kong, it did not materialize.
4. We have added many more secondary outcomes as per our evidence gap map framework and also their categorization has changed. But data was not available in primary studies for most of them, so they could not be analysed in this review.
5. In the protocol we stated that ‘When multiple papers report different results on an identical outcome, we will contact the authors to enquire about the differences and choose the results that more accurately reflect the impact of the intervention as relevant to our research questions. If contacting the author does not yield a clear decision, we will use results from the paper with the latest publication date.’ However, due to the large scope and limited resources of our review, we instead used results from the most recent paper.
6. In the protocol, we stated that ‘Where multiple outcomes are reported from different specifications, we will select the specification with the lowest risk of bias in attributing impact, for example, the most appropriately specified outcomes equation.’ However, given that risk of bias could not be assessed until after inclusion decisions were made, we instead used the authors preferred specification. If this was not explicitly stated, but the authors reported one specification as the “main results” and other specifications as “robustness checks” we used the “main results” specification. If neither of these applied, we used the specification with the most controls.

For peer review only - http://bmjopen.bmj.com/site/about/guidelines.xhtml

7. In the protocol we stated that 'When multiple treatment arms, we will divide the control group by the number of treatment arms.' Instead, we chose independent effects, choosing either a) the treatment arm with the most components, b) the treatment arm that was identified by the authors as the one that would be scaled up, or c) when number of components was equal, the arm that was the most cost effective (e.g. choosing unconditional cash transfers (UCTs) over conditional cash transfers as the administrative costs related to UCTs are significantly lower)

For peer review only

Appendix 3: Search strategy and search terms for impact evaluations

We searched the academic databases and websites listed below on 17 May 2019 and updated the search on 5 May, 2020. A full record of the applied search terms is provided in Appendix C.

1. MEDLINE
2. CAB Global Health
3. EMBASE
4. Cochrane Controlled Trials Register (CENTRAL)
5. CINAHL
6. PsycINFO
7. Popline
8. Africa-wide information
9. Academic search complete
10. Scopus
11. Campbell Library
12. Google Scholar
13. EconLit
14. IDEAS/RePEc
15. WHO Global Index Medicus
16. Pascal-Francis
17. Open-Grey
18. Grey Literature Report
19. Social Science Research Network (SSRN)
20. Eldis
21. GAVI
22. Epistemonikos
23. Innovations for Poverty Action (IPA)
24. Abdul Latif Jameel Poverty Action Lab (J-PAL)
25. 3ie Impact Evaluation Repository
26. 3ie Systematic Review Repository
27. Registry of International Development Impact Evaluations (RIDIE)
28. Global Development Network
29. World Bank Development Impact Evaluation (DIME) and Impact Evaluation Policy Papers
30. Inter-American Development Bank
31. Center for Global Development
32. Center for Effective Global Action (CEGA)
33. DFID Research for Development (R4D)
34. USAID

Website searches for capturing grey literature were carried out from January to May 2020

Search terms

L&MICs

2. (Afghanistan or Albania or Algeria or Angola or Argentina or Armenia or Armenian or Azerbaijan or Bangladesh or Benin or Byelarus or Byelorussian or Belarus or Belorussian or Belorussia or Belize or Bhutan or Bolivia or Bosnia or Herzegovina or Hercegovina or Botswana or Brazil or Bulgaria or Burkina Faso or Burkina Fasso or Upper Volta or Burundi or Urundi or Cambodia or Khmer Republic or Kampuchea or

For peer review only - http://bmjopen.bmj.com/site/about/guidelines.xhtml

Cameroon or Cameroons or Cameron or Camerons or Cape Verde or Cabo Verde or Central African Republic or Chad or Tchad or China or Colombia or Comoros or Comoro Islands or Comores or Mayotte or Congo or Zaire or Costa Rica or Cote d'Ivoire or Ivory Coast or Cuba or Djibouti or French Somaliland or Dominica or Dominican Republic or East Timor or East Timur or Timor Leste or Ecuador or Egypt or United Arab Republic or El Salvador or Eritrea or Ethiopia or Fiji or Gabon or Gabonese Republic or Gambia or Gaza or Georgia Republic or Georgian Republic or Ghana or Grenada or Guatemala or Guinea or Guiana or Guyana or Haiti or Honduras or India or Maldives or Indonesia or Iran or Iraq or Jamaica or Jordan or Kazakhstan or Kazakh or Kenya or Kiribati or Korea or Kosovo or Kyrgyzstan or Kirghizia or Kyrgyz Republic or Kirghiz or Kirgizstan or Lao PDR or Laos or Lebanon or Lesotho or Basutoland or Liberia or Libya or Macedonia or Madagascar or Malagasy Republic or Malaysia or Malaya or Malay or Sabah or Sarawak or Malawi or Mali or Marshall Islands or Mauritania or Mauritius or Agalega Islands or Mexico or Micronesia or Middle East or Moldova or Moldovia or Moldovian or Mongolia or Montenegro or Morocco or Ifni or Mozambique or Myanmar or Myanma or Burma or Namibia or Nepal or Netherlands Antilles or Nicaragua or Niger or Nigeria or Muscat or Pakistan or Palau or Palestine or Panama or Paraguay or Peru or Philippines or Philipines or Phillipines or Phillippines or Papua New Guinea or Romania or Rumania or Roumania or Rwanda or Ruanda or Saint Lucia or St Lucia or Saint Vincent or St Vincent or Grenadines or Samoa or Samoan Islands or Navigator Island or Navigator Islands or Sao Tome or Senegal or Serbia or Montenegro or Seychelles or Sierra Leone or Sri Lanka or Solomon Islands or Somalia or Sudan or Suriname or Surinam or Swaziland or Eswatini or South Africa or Syria or Tajikistan or Tadzhikistan or Tadjikistan or Tadzhik or Tanzania or Thailand or Togo or Togolese Republic or Tonga or Tunisia or Turkey or Turkmenistan or Turkmen or Uganda or Ukraine or Uzbekistan or Uzbek or Vanuatu or New Hebrides or Venezuela or Vietnam or Viet Nam or West Bank or Yemen or Zambia or Zimbabwe or Rhodesia)

3. (Africa or Asia or Caribbean or West Indies or South America or Latin America or Central America)
4. ((developing or less* developed or least developed or under developed or underdeveloped or middle income or low* income or underserved or under served or deprived or poor* or resource limited or resource constrained) adj (countr* or nation? or population? or world or state* or emerging econom* or global south))
5. ((developing or less* developed or least developed or under developed or underdeveloped or middle income or low* income or resource limited or resource constrained) adj (economy or economies))
6. (low* adj (gdp or gnp or gross domestic or gross national))
7. (low adj3 middle adj3 countr*)
8. (lmic or lmics or third world or lami countr*)
9. transitional countr*
10. 1 OR 2 OR 3 OR 4 OR 5 OR 6 OR 7

Immunisation

1. (immuniz* or immunis* or vaccin* or inoculat* or innoculat* or immunotherap* or prophyla*)

Young children and caregivers

1. (child* or infant* or newborn* or neonat* or prenatal or pre natal or antenatal or ante natal or baby or babies or toddler* or preschool* or parent* or mother* or father* or maternal or paternal or caregiver* or grandparent* or grandmother* or grandfather* or family member*)

Impact evaluation methods

For peer review only - http://bmjopen.bmj.com/site/about/guidelines.xhtml

1. (random* or experiment* or (match* adj2 (propensity or coarsened or covariate)) or "propensity score" or "difference in difference*" or "difference-in-difference*" or "differences in difference*" or "differences-in-difference*" or "double difference*" or "quasi-experimental" or "quasi experimental" or "quasi-experiment" or "quasi experiment" or ((estimator or counterfactual) and evaluation*) or "instrumental variable*" or (IV adj2 (estimation or approach)) or regression discontinuity or time series or segment* regression)

- **Example full search strategy**

Below we present a draft of the full search strategy used to search MEDLINE. Note that in MEDLINE's syntax, terms with strokes (e.g., Immunization/) denote Medical Subject Heading (MeSH) terms, while strings appended with "ti,ab,kw" are searched in the title, abstract, and keyword fields of records in the database.

Ovid MEDLINE(R) and Epub Ahead of Print, In-Process & Other Non-Indexed Citations, Daily and Versions(R) <1946 to May 15, 2019> Searched 16th May 2019

1 (immuniz* or immunis* or vaccin* or inoculat* or innoculat* or immunotherap* or prophyla*).ti,ab,kw. (672732)

2 immunization/ or immunization, passive/ or immunization schedule/ or immunization, secondary/ or immunotherapy, active/ or vaccination/ or Immunization Programs/ or mass vaccination/ (158163)

3 Tuberculosis Vaccines/ or BCG Vaccine/ or Diphtheria-Tetanus Vaccine/ or Meningococcal Vaccines/ or Pertussis Vaccine/ or Diphtheria-Tetanus-acellular Pertussis Vaccines/ or Diphtheria-Tetanus-Pertussis Vaccine/ or Diphtheria-Tetanus Vaccine/ or Measles Vaccine/ or Mumps Vaccine/ or Rubella Vaccine/ or Measles-Mumps-Rubella Vaccine/ or Poliovirus Vaccines/ or Poliovirus Vaccine, Inactivated/ or Poliovirus Vaccine, Oral/ or Japanese Encephalitis Vaccines/ or Rotavirus Vaccine/ (49639)

4 or/1-3 (720583)

5 developing countries.sh,kf. (83271)

6 (Africa or Asia or Caribbean or West Indies or South America or Latin America or Central America).ti,ab,kw. (200981)

7 Africa/ or Asia/ or Caribbean/ or West Indies/ or South America/ or Latin America/ or Central America/ (73389)

8 (Africa or Central America or South America or Caribbean or Central Asia or Afghanistan or Albania or Algeria or Angola or Argentina or Armenia or Armenian or Azerbaijan or Bangladesh or Benin or Byelarus or Byelorussian or Belarus or Belorussian or Belorussia or Belize or Bhutan or Bolivia or Bosnia or Herzegovina or Hercegovina or Botswana or Brazil or Bulgaria or Burkina Faso or Burkina Fasso or Upper Volta or Burundi or Urundi or Cambodia or Khmer Republic or Kampuchea or Cameroon or Cameroons or Cameron or Camerons or Cape Verde or Cabo Verde or Central African Republic or Chad or Tchad or China or Colombia or Comoros or Comoro Islands or Comores or Mayotte or Congo or Zaire or Costa Rica or Cote d'Ivoire or Ivory Coast or Cuba or Djibouti or French Somaliland or Dominica or Dominican Republic or East Timor or East Timur or Timor Leste or Ecuador or Egypt or United Arab Republic or El Salvador or Eritrea or Ethiopia or Fiji or Gabon or Gabonese Republic or Gambia or Gaza or Georgia Republic or Georgian Republic or Ghana or Grenada or Guatemala or Guinea or Guiana or Guyana or Haiti or Honduras or India or Maldives or Indonesia or Iran or Iraq or Jamaica or Jordan or Kazakhstan or Kazakh or Kenya or Kiribati or Korea or Kosovo or Kyrgyzstan or Kirghizia or Kyrgyz Republic or Kirghiz or Kirgizstan or Lao PDR or Laos or Lebanon or Lesotho or Basutoland or Liberia or Libya or Macedonia or Madagascar or Malagasy Republic or Malaysia or Malaya or Malay or Sabah or Sarawak or Malawi or Mali or Marshall Islands or Mauritania

For peer review only - http://bmjopen.bmj.com/site/about/guidelines.xhtml
For peer review only

or Mauritius or Agalega Islands or Mexico or Micronesia or Middle East or Moldova or Moldovia or Moldovian or Mongolia or Montenegro or Morocco or Ifni or Mozambique or Myanmar or Myanma or Burma or Namibia or Nepal or Netherlands Antilles or Nicaragua or Niger or Nigeria or Muscat or Pakistan or Palau or Palestine or Panama or Paraguay or Peru or Philippines or Philipines or Phillipines or Phillippines or Papua New Guinea or Romania or Rumania or Roumania or Rwanda or Ruanda or Saint Lucia or St Lucia or Saint Vincent or St Vincent or Grenadines or Samoa or Samoan Islands or Navigator Island or Navigator Islands or Sao Tome or Senegal or Serbia or Montenegro or Seychelles or Sierra Leone or Sri Lanka or Solomon Islands or Somalia or Sudan or Suriname or Surinam or Swaziland or Eswatini or South Africa or Syria or Tajikistan or Tadzhikistan or Tadjikistan or Tadzhik or Tanzania or Thailand or Togo or Togolese Republic or Tonga or Tunisia or Turkey or Turkmenistan or Turkmen or Uganda or Ukraine or Uzbekistan or Uzbek or Vanuatu or New Hebrides or Venezuela or Vietnam or Viet Nam or West Bank or Yemen or Zambia or Zimbabwe or Rhodesia).ti,ab,kw,sh. (1387951)

9 ((developing or less* developed or least developed or under developed or underdeveloped or middle income or low* income or underserved or under served or deprived or poor* or resource limited or resource constrained) adj (countr* or nation? or population? or world or state*)).ti,ab,kw. (91955)

10 ((developing or less* developed or least developed or under developed or underdeveloped or middle income or low* income or resource limited or resource constrained) adj (economy or economies)).ti,ab,kw. (504)

11 (low* adj (gdp or gnp or gross domestic or gross national)).ti,ab,kw. (234)

12 (low adj3 middle adj3 countr*).ti,ab,kw. (12819)

13 (lmic or lmics or third world or lami countr*).ti,ab,kw. (6486)

14 (transitional countr* or emerging econom* or global south).ti,ab,kw. (873)

15 or/5-14 (1487811)

16 4 and 15 (80257)

17 Parents/ or Fathers/ or Mothers/ or Grandparents/ or Caregivers/ or Single Parent/ or Pregnant Women/ or Child, Preschool/ or Infant/ or Infant, Newborn/ or Infant, Low Birth Weight/ or Infant, Small for Gestational Age/ or Infant, Very Low Birth Weight/ or Infant, Extremely Low Birth Weight/ or Infant, Postmature/ or Infant, Premature/ or Infant, Extremely Premature/ (1597959)

18 (child* or infant* or newborn* or neonat* or neo nat* or prenatal or pre natal or ante natal or antenatal or baby or babies or toddler* or preschool* or parent* or mother* or father* or maternal or paternal).ti,ab,kw. (2284298)

19 or/17-18 (2893768)

20 16 and 19 (28515)

21 (random* or experiment* or (match* adj2 (propensity or coarsened or covariate)) or "propensity score" or ("difference in difference*" or "difference-in-difference*" or "differences in difference*" or "differences-in-difference*" or "double difference*") or ("quasi-experimental" or "quasi experimental" or "quasi-experiment" or "quasi experiment") or ((estimator or counterfactual) and evaluation*) or "instrumental variable*" or (IV adj2 (estimation or approach)) or regression discontinuity or time series or segment* regression).ti,ab,kw. (2983909)

For peer review only - http://bmjopen.bmj.com/site/about/guidelines.xhtml

22 Randomized Controlled Trial/ or Random Allocation/ or Evaluation Studies/ or Propensity Score/ or Interrupted Time Series Analysis/ or Controlled Before-After Studies/ or Controlled Clinical Trial/ or Non-Randomized Controlled Trials as Topic/ (896249)

23 or/21-22 (3415351)

24 Cost Analysis/ or Cost-Benefit Analysis/ or Quality-Adjusted Life Years/ or Economics, Medical/ or Cost of Illness/ or Health Care Costs/ or Direct Service Costs/ or Budgets/ or Health Care Sector/ or Public Expenditures/ (193420)

25 (cost-effective* or cost-benefit).ti,ab,kw. (130711)

26 ("life year" or "life years" or qaly* or daly*).ti,ab,kw. (18749)

27 ((economic* or cost*) adj6 (mortality or death* or markov)).ti,ab,kw. (17246)

28 ("cost minimi*" or "cost-utilit*" or "economic evaluation*" or "economic review*" or "cost outcome" or "cost analys*" or "economic analys*" or "budget* impact analys*").ti,ab,kw. (27594)

29 or/24-28 (301485)

30 (review or meta-analysis).pt. (2557796)

31 meta-analysis/ or "systematic review"/ (163880)

32 cochrane database of systematic reviews.jn. (14162)

33 (systematic review or literature review).ti. (130870)

34 or/30-33 (2590211)

35 23 or 29 or 34 (5946083)

36 20 and 35 (8309)

37 exp Animals/ (22312530)

38 Humans/ (17732234)

39 37 not (37 and 38) (4580296)

40 36 not 39 (7984)

For peer review only - http://bmjopen.bmj.com/site/about/guidelines.xhtml

Appendix 4: List of excluded impact evaluation studies

We excluded the following studies at full-text screening for not meeting our community engagement criteria.

Reference list

Aaby, Peter, Adam Roth, Henrik Ravn, Bitiguida Mutna Napirna, Amabelia Rodrigues, Ida Maria Lisse, Lone Stensballe, et al. 2011. "Randomized Trial of BCG Vaccination at Birth to Low-Birth-Weight Children: Beneficial Nonspecific Effects in the Neonatal Period?" The Journal of Infectious Diseases 204 (2): 245–52. https://doi.org/10/ds6pbx.

Aggarwal, Shilpa. 2018. "The Long Road to Health: Healthcare Utilization Impacts of a Road Pavement Policy in Rural India." Working paper. Hyderabad: Indian School of Business. http://eprints.exchange.isb.edu/884/.

Ahmed, Tashrik, Aneesa Arur, Damien de Walque, and Gil Shapira. 2019. "Incentivizing Quantity and Quality of Care: Evidence from an Impact Evaluation of Performance-Based Financing in the Health Sector in Tajikistan." Policy Research Working Papers 8951. Washington, DC: The World Bank. https://doi.org/10.1596/1813-9450-8951.

Alatas, Vivi, Arun Chandrasekhar, Markus Mobius, Benjamin Olken, and Cindy Paladines. 2019. "When Celebrities Speak: A Nationwide Twitter Experiment Promoting Vaccination in Indonesia." NBER Working Paper Series 25589. Cambridge, MA: National Bureau of Economic Research. https://doi.org/10.3386/w25589.

Amaral, João, Eleanor Gouws, Jennifer Bryce, Álvaro Jorge Madeiro Leite, Antonio Ledo Alves da Cunha, and Cesar G. Victora. 2004. "Effect of Integrated Management of Childhood Illness (IMCI) on Health Worker Performance in Northeast-Brazil." Cadernos de Saúde Pública 20 (suppl 2): S209–19. https://doi.org/10/dbvvrz.

Andrade, Mônica Viegas, Flávia Chein, Laetícia Rodrigues de Souza, and Jaume Puig-Junoy. 2012. "Income Transfer Policies and the Impacts on the Immunization of Children: The Bolsa Família Program." Cadernos de Saúde Pública 28 (7): 1347–58. https://doi.org/10/gghf2x.

Andreoni, James, Michael Callen, Muhammad Yasir Khan, Karrar Jaffar, and Charles Sprenger. 2016. "Using Preference Estimates to Customize Incentives: An Application to Polio Vaccination Drives in Pakistan." NBER Working Paper Series 22019. Cambridge, MA: National Bureau of Economic Research. https://doi.org/10.3386/w22019.

Anjum, Qudsia, Aamir Omair, S. N. Bazmi Inam, Yousuf Ahmed, Yaseen Usman, and Shazia Shaikh. 2004. "Improving Vaccination Status of Children under Five through Health Education." JPMA. The Journal of the Pakistan Medical Association 54 (12): 610–13.

Aquino, Rosana, Nelson F. de Oliveira, and Mauricio L. Barreto. 2009. "Impact of the Family Health Program on Infant Mortality in Brazilian Municipalities." American Journal of Public Health 99 (1): 87–93. https://doi.org/10/btntq8.

Arraiz, Irani, and Sandra Rozo. 2011. "Same Bureaucracy, Different Outcomes in Human Capital? How Indigenous and Rural Non-Indigenous Areas in Panama Responded to the CCT." OVE Working Papers 03/11. Washington, D.C.: Inter-American Development Bank. http://www.ssrn.com/abstract=1847124.

Atnafu, Asfaw, Kate Otto, and Christopher H. Herbst. 2017. "The Role of MHealth Intervention on Maternal and Child Health Service Delivery: Findings from a Randomized Controlled Field Trial in Rural Ethiopia." MHealth 3 (September). https://doi.org/10/gcmhjc.

Attanasio, Orazio, Luis Carlos Gómez, Patricia Heredia, and Marcos Vera-Hernández. n.d. "The Short-Term Impact of a Conditional Cash Subsidy on Child Health and Nutrition in Colombia," 15.

Ayieko, Philip, Stephen Ntoburi, John Wagai, Charles Opondo, Newton Opiyo, Santau Migiro, Annah Wamae, et al. 2011. "A Multifaceted Intervention to Implement Guidelines and Improve Admission Paediatric Care in Kenyan District Hospitals: A Cluster Randomised Trial." Edited by Igor Rudan. PLoS Medicine 8 (4). https://doi.org/10/dtj73r.

Balasubramaniam, Sudharsanam, Somesh Kumar, Reena Sethi, Elaine Charurat, Kamlesh Lalchandani, Anne Schuster, and Bulbul Sood. 2018. "Quasi-Experimental Study of Systematic Screening for Family Planning

For peer review only - http://bmjopen.bmj.com/site/about/guidelines.xhtml

Services among Postpartum Women Attending Village Health and Nutrition Days in Jharkhand, India." International Journal of Integrated Care 18 (1). https://doi.org/10/gg9pgr.

Banerjee, Rakesh, and Ashish Sachdeva. 2015. "Pathways to Preventive Health, Evidence from India's Rural Road Program." USC-INET Research Paper 15–19. Los Angeles, CA: USC Dornsife Institute for New Economic Thinking. http://www.ssrn.com/abstract=2636999.

Bangure, Donewell, Daniel Chirundu, Notion Gombe, Tawanda Marufu, Gibson Mandozana, Mufuta Tshimanga, and Lucia Takundwa. 2015. "Effectiveness of Short Message Services Reminder on Childhood Immunization Programme in Kadoma, Zimbabwe - a Randomized Controlled Trial, 2013." BMC Public Health 15 (1). https://doi.org/10/f63zrg.

Barham, Tania. 2005. "The Impact of the Mexican Conditional Cash Transfer Program on Immunization Rates." Boulder: University of Colorado. https://ibs.colorado.edu/barham/wp/CCTimmunWBfin.pdf.

Barham, Tania, Logan E. Brenzel, and John A. Maluccio. 2007. "Beyond 80%: Are There New Ways of Increasing Vaccination Coverage? Evaluation of CCT Programs in Mexico and Nicaragua." HNP Discussion Paper 41537. Washington: World Bank. http://www.ssrn.com/abstract=993760.

Barham, Tania, and John A. Maluccio. 2009. "Eradicating Diseases: The Effect of Conditional Cash Transfers on Vaccination Coverage in Rural Nicaragua." Journal of Health Economics 28 (3): 611–21. https://doi.org/10/czhvx3.

Basinga, Paulin, P. J. Gertler, Agnes Binagwaho, A. L. B. Soucat, J. R. Sturdy, and C. M. J. Vermeersch. 2009. "Impact of Performance Based Financing in Rwanda: Health Facility Level Analysis." Working Paper 32. New Delhi: Global Development Network. http://www.gdn.int/impact-performance-based-financing-rwanda-health-facility-level-analysis.

Basinga, Paulin, Paul J Gertler, Agnes Binagwaho, Agnes LB Soucat, Jennifer Sturdy, and Christel MJ Vermeersch. 2011. "Effect on Maternal and Child Health Services in Rwanda of Payment to Primary Health-Care Providers for Performance: An Impact Evaluation." The Lancet 377 (9775): 1421–28. https://doi.org/10/b2f469.

Beck, Simon, Anni-Maria Pulkki-Brännström, and Miguel San Sebastián. 2015. "Basic Income – Healthy Outcome? Effects on Health of an Indian Basic Income Pilot Project: A Cluster Randomised Trial." Journal of Development Effectiveness 7 (1): 111–26. https://doi.org/10/f3n3kb.

Berhane, Yemane, and Joyce Pickering. 1993. "Are Reminder Stickers Effective in Reducing Immunization Dropout Rates in Addis Ababa, Ethiopia?" Journal of Tropical Medicine and Hygiene 96 (3): 139–45. https://europepmc.org/article/med/8505766.

Bernal, Pedro, and Sebastian Martinez. 2020. "In-Kind Incentives and Health Worker Performance: Experimental Evidence from El Salvador." Journal of Health Economics 70 (March). https://doi.org/10/ghpbkj.

Binyaruka, Peter, Edith Patouillard, Timothy Powell-Jackson, Giulia Greco, Ottar Maestad, and Josephine Borghi. 2015. "Effect of Paying for Performance on Utilisation, Quality, and User Costs of Health Services in Tanzania: A Controlled before and after Study." Edited by Jan Ostermann. PLOS ONE 10 (8). https://doi.org/10/gghf22.

Bonfrer, Igna, Lyn Breebaart, and Ellen Van de Poel. 2016. "The Effects of Ghana's National Health Insurance Scheme on Maternal and Infant Health Care Utilization." Edited by Osman Alimamy Sankoh. PLOS ONE 11 (11). https://doi.org/10/f9q46z.

Bonfrer, Igna, Ellen Van de Poel, and Eddy Van Doorslaer. 2014. "The Effects of Performance Incentives on the Utilization and Quality of Maternal and Child Care in Burundi." Social Science & Medicine 123 (December): 96–104. https://doi.org/10/f6vdkg.

Bossio, Juan Carlos, Ivan Sanchis, Gustavo Ariel Armando, Sergio Javier Arias, and Humberto Jure. 2019. "Resultado de una estrategia de recordatorios previos y posteriores a la fecha de vacunación para mejorar la oportunidad de la vacunación a los seis meses." Cadernos de Saúde Pública 35 (12). https://doi.org/10/ghpbkt.

Bradley, Janet, and Susan Igras. 2005. "Improving the Quality of Child Health Services: Participatory Action by Providers." International Journal for Quality in Health Care 17 (5): 391–99. https://doi.org/10/bdjp6m.

Brenner, Stephan, Rachel P. Chase, Shannon A. McMahon, Julia Lohmann, Christopher J. Makwero, Adamson S. Muula, and Manuela De Allegri. 2020. "Effect Heterogeneity in Responding to Performance-Based Incentives:

For peer review only - http://bmjopen.bmj.com/site/about/guidelines.xhtml

A Quasi-Experimental Comparison of Impacts on Health Service Indicators between Hospitals and Health Centers in Malawi.” Health Systems & Reform 6 (1). https://doi.org/10/gg2njg.

Briere, E. C., T. K. Ryman, E. Cartwright, E. T. Russo, K. A. Wannemuehler, B. L. Nygren, S. Kola, et al. 2012. “Impact of Integration of Hygiene Kit Distribution with Routine Immunizations on Infant Vaccine Coverage and Water Treatment and Handwashing Practices of Kenyan Mothers.” Journal of Infectious Diseases 205 (suppl 1): S56–64. https://doi.org/10/f3wknp.

Brown, V. B., O. A. Oluwatosin, J. O. Akinyemi, and A. A. Adeyemo. 2016. “Effects of Community Health Nurse-Led Intervention on Childhood Routine Immunization Completion in Primary Health Care Centers in Ibadan, Nigeria.” Journal of Community Health 41 (2): 265–73. https://doi.org/10/f8czr8.

Brown, Victoria Bolanle, Oyeninhun Abimbola Oluwatosin, and Martins Olusola Ogundeji. 2017. “Impact of Training Intervention on Immunization Providers’ Knowledge and Practice of Routine Immunization in Ibadan, South-Western Nigeria: A Primary Health Care Experience.” Pan African Medical Journal 26. https://doi.org/10/ghpbkv.

Brughal, R F, and J P Kevany. 1996. “Maximizing Immunization Coverage through Home Visits: A Controlled Trial in an Urban Area of Ghana.” Bulletin of the World Health Organization 74 (5): 517–24. https://www.ncbi.nlm.nih.gov/pmc/articles/PMC2486871/.

Busso, Matias, Julian Cristia, and Sarah Humpage. 2015. “Did You Get Your Shots? Experimental Evidence on the Role of Reminders.” Journal of Health Economics 44 (December): 226–37. https://doi.org/10/f75n4m.

Cahyadi, Nur, Rema Hanna, Benjamin A. Olken, Rizal Adi Prima, Elan Satriawan, Ekki Syamsulhakim, and Rema Hanna. 2018. “Cumulative Impacts of Conditional Cash Transfer Programs: Experimental Evidence from Indonesia.” NBER Working Paper Series 24670. Cambridge: National Bureau of Economic Research. https://www.nber.org/papers/w24670.

Carrillo, Bladimir, Wilman J Iglesias, and Juan C Trujillo. 2015. “Attainments and Limitations of an Early Childhood Programme in Colombia.” Health Policy and Planning 30 (7): 906–16. https://doi.org/10/f7vmx5.

Carvalho, Natalie, Naveen Thacker, Subodh S. Gupta, and Joshua A. Salomon. 2014. “More Evidence on the Impact of India’s Conditional Cash Transfer Program, Janani Suraksha Yojana: Quasi-Experimental Evaluation of the Effects on Childhood Immunization and Other Reproductive and Child Health Outcomes.” Edited by Jeremy D. Goldhaber-Fiebert. PLoS ONE 9 (10). https://doi.org/10/gghf24.

Chakrabarti, Averi, Karen A. Grépin, and Stéphane Helleringer. 2019. “The Impact of Supplementary Immunization Activities on Routine Vaccination Coverage: An Instrumental Variable Analysis in Five Low-Income Countries.” Edited by Vijayaprasad Gopichandran. PLOS ONE 14 (2). https://doi.org/10/gghf25.

Chandir, S., A.J. Khan, H. Hussain, H.R. Usman, S. Khowaja, N.A. Halsey, and S.B. Omer. 2010. “Effect of Food Coupon Incentives on Timely Completion of DTP Immunization Series in Children from a Low-Income Area in Karachi, Pakistan: A Longitudinal Intervention Study.” Vaccine 28 (19): 3473–78. https://doi.org/10/cz4mk3.

Chansa, Collins, Ashis Das, Jumana Qamruddin, Jed Friedman, Akafwilangachi Mkandawire, and Monique Vledder. 2015. “Linking Results to Performance: Evidence from a Results Based Financing Pre-Pilot Project in Katete District, Zambia.” Discussion Paper 98265. Washington, DC: The World Bank. https://openknowledge.worldbank.org/handle/10986/22390?show=full.

Chelagat, T., G. Kokwaro, J. Onyango, and J. Rice. 2020. “Effect of Project-Based Experiential Learning on the Health Service Delivery Indicators: A Quasi-Experiment Study.” BMC Health Services Research 20 (1). https://doi.org/10/gg9pgv.

Chen, Li, Xiaozhen Du, Lin Zhang, Michelle Helena van Velthoven, Qiong Wu, Ruikan Yang, Ying Cao, et al. 2016. “Effectiveness of a Smartphone App on Improving Immunization of Children in Rural Sichuan Province, China: A Cluster Randomized Controlled Trial.” BMC Public Health 16 (1). https://doi.org/10/f82vfx.

Chen, Yvonne Jie, Namrata Chindarkar, and Yun Xiao. 2019. “Effect of Reliable Electricity on Health Facilities, Health Information, and Child and Maternal Health Services Utilization: Evidence from Rural Gujarat, India.” Journal of Health, Population and Nutrition 38 (1). https://doi.org/10/ghpbk6.

For peer review only - http://bmjopen.bmj.com/site/about/guidelines.xhtml

Cristia, Julian, William N. Evans, and Beomsoo Kim. 2015. “Improving the Health Coverage of the Rural Poor: Does Contracting-out Mobile Medical Teams Work?” The Journal of Development Studies 51 (3). https://doi.org/10/ghpwp8.

Cristia, Julián P, William N Evans, and Beomsoo Kim. 2011. “Does Contracting-out Primary Care Services Work? The Case of Rural Guatemala.” IDB Working Paper Series IDB-WP-273. Washington. https://www.econstor.eu/handle/10419/88975.

Cristia, Julian, Ariadna García Prado, and Cecilia Peluffo. 2015. “The Impact of Contracting in and Contracting out Basic Health Services: The Guatemalan Experience.” World Development 70 (June): 215–27. https://doi.org/10/gghf27.

Cruzado de la Vega, Viviana. 2017. “Pagos por desempeño para mejorar estado nutricional infantil: impacto de los convenios de apoyo presupuestario en tres regiones peruanas con alta prevalencia de desnutrición cronica infantil, 2010-2014.” Revista Peruana de Medicina Experimental y Salud Pública 34 (3): 365–76. https://doi.org/10/gghf28.

Daoud, Adel, and Bernhard Reinsberg. 2019. “Structural Adjustment, State Capacity and Child Health: Evidence from IMF Programmes.” International Journal of Epidemiology 48 (2): 445–54. https://doi.org/10/gfqr5z.

Dicko, Alassane, Sidy O Toure, Mariam Traore, Issaka Sagara, Ousmane B Toure, Mahamadou S Sissoko, Alpha T Diallo, et al. 2011. “Increase in EPI Vaccines Coverage after Implementation of Intermittent Preventive Treatment of Malaria in Infant with Sulfadoxine -Pyrimethamine in the District of Kolokani, Mali: Results from a Cluster Randomized Control Trial.” BMC Public Health 11 (1). https://doi.org/10/cvr2cj.

Dissieka, Romance, Marissa Soohoo, Amynah Janmohamed, and David Doledec. 2019. “Providing Mothers with Mobile Phone Message Reminders Increases Childhood Immunisation and Vitamin A Supplementation Coverage in Côte d’Ivoire: A Randomised Controlled Trial.” Journal of Public Health in Africa 10 (1): 56–60. https://doi.org/10/ghpbk9.

Djibuti, Mamuka, George Gotsadze, Akaki Zoidze, George Mataradze, Laura C Esmail, and Jillian Clare Kohler. 2009. “The Role of Supportive Supervision on Immunization Program Outcome - a Randomized Field Trial from Georgia.” BMC International Health and Human Rights 9 (S1). https://doi.org/10/dxd2rk.

Domek, Gretchen J., Ingrid L. Contreras-Roldan, Sheana Bull, Sean T. O’Leary, Guillermo Antonio Bolaños Ventura, Michael Bronsert, Allison Kempe, and Edwin J. Asturias. 2019. “Text Message Reminders to Improve Infant Immunization in Guatemala: A Randomized Clinical Trial.” Vaccine 37 (42): 6192–6200. https://doi.org/10/ghpbmb.

Drain, Paul K, Josoa S Ralaivao, Alexander Rakotonandrasana, and Mary A Carnell. 2003. “Introducing Auto-Disable Syringes to the National Immunization Programme in Madagascar.” Bulletin of the World Health Organization, 8.

D’Souza, Prathibha V, and J Umarani. 2014. “Teaching Package Improves Mothers Knowledge on Vaccine Preventable Diseases and Vaccination: A Quasi Experimental Study.” International Journal of Research in Medical Sciences 2 (3): 976–82. https://doi.org/10/ghpv3h.

Dykstra, Sarah, Amanda Glassman, Charles Kenny, and Justin Sandefur. 2019. “Regression Discontinuity Analysis of Gavi’s Impact on Vaccination Rates.” Journal of Development Economics 140 (September): 12–25. https://doi.org/10/gg9pgw.

Edoka, Ijeoma, Tim Ensor, Barbara McPake, Rogers Amara, Fu-Min Tseng, and Joseph Edem-Hotah. 2016. “Free Health Care for Under-Fives, Expectant and Recent Mothers? Evaluating the Impact of Sierra Leone’s Free Health Care Initiative.” Health Economics Review 6 (1). https://doi.org/10/gghf29.

Eichler, Rena, Paul Auxila, Uder Antoine, and Bernateau Desmangles. 2007. “Performance-Based Incentives for Health: Six Years of Results from Supply-Side Programs in Haiti.” CGD Working Paper 121. Washington, DC: Center for Global Development. http://www.ssrn.com/abstract=1003249.

Ekhaguere, Osayame A, Rosena O Oluwafemi, Bolaji Badejoko, Lawal O Oyeneyin, Azeez Butali, Elizabeth D Lowenthal, and Andrew P Steenhoff. 2019. “Automated Phone Call and Text Reminders for Childhood Immunisations (PRIMM): A Randomised Controlled Trial in Nigeria.” BMJ Global Health 4 (2). https://doi.org/10/gghf3b.

For peer review only - http://bmjopen.bmj.com/site/about/guidelines.xhtml

Eze, G U, and A O Adeleye. 2015. “Enhancing Routine Immunization Performance Using Innovative Technology in an Urban Area of Nigeria.” West African Journal of Medicine 34 (1): 3–10. https://pubmed.ncbi.nlm.nih.gov/26902809/.

Gajate-Garrido, Gissele, and Clement Ahiadeke. 2012. “The Effect of Parents’ Insurance Enrollment on Health Care Utilization: Evidence from Ghana.” SSRN Scholarly Paper ID 2158824. Rochester, NY: Social Science Research Network. https://doi.org/10.2139/ssrn.2158824.

Garly, M. L., C. L. Martins, C. Bale, F. da Costa, F. Dias, H. Whittle, and P. Aaby. 1999. “Early Two-Dose Measles Vaccination Schedule in Guinea-Bissau: Good Protection and Coverage in Infancy.” International Journal of Epidemiology 28 (2): 347–52. https://doi.org/10/cgqfgz.

Gauri, Varun. 2009. “Do International Treaties Promote Development? The Convention on the Rights of the Child and Basic Immunization.” Policy Research Working Paper 4964. Washington: The World Bank. https://doi.org/10.1596/1813-9450-4964.

Gauri, Varun, and Peyvand Khaleghian. 2002. “Immunization in Developing Countries: Its Political and Organizational Determinants.” Policy Research Working Paper 2769. Washington: The World Bank. https://www.infona.pl/resource/bwmeta1.element.elsevier-4a3b393c-c1da-3dff-9b7c-91c05767fb9d.

Gilbert, Sarah Skye, Ngwegwe Bulula, Emmanuel Yohana, Jenny Thompson, Emily Beylerian, Laurie Werner, and Jessica C. Shearer. 2020. “The Impact of an Integrated Electronic Immunization Registry and Logistics Management Information System (EIR-ELMIS) on Vaccine Availability in Three Regions in Tanzania: A Pre-Post and Time-Series Analysis.” Vaccine 38 (3): 562–69. https://doi.org/10/ghpbsr.

Goodson, James L., Manisha A. Kulkarni, Jodi L. Vanden Eng, Kathleen A. Wannemuehler, Annett H. Cotte, Rachelle E. Desrochers, Bakolalao Randriamanalina, and Elizabeth T. Luman. 2012. “Improved Equity in Measles Vaccination from Integrating Insecticide-Treated Bednets in a Vaccination Campaign, Madagascar: Equity in Measles Vaccination in Madagascar.” Tropical Medicine & International Health 17 (4): 430–37. https://doi.org/10/fxsrbw.

Guindon, G. Emmanuel. 2014. “The Impact of Health Insurance on Health Services Utilization and Health Outcomes in Vietnam.” Health Economics, Policy and Law 9 (4): 359–82. https://doi.org/10/f6g47g.

Gultiano, Soccoro A., and Elizabeth M. King. 2006. “A Better Start in Life: Evaluation Results from an Early Childhood Development Program.” Philippine Journal of Development 33 (1/2): 101–28. https://ideas.repec.org/p/phd/pjdevt/pjd_2006_vol__xxxiii_nos__1and2-d.html.

Habib, Muhammad Atif, Sajid Soofi, Simon Cousens, Saeed Anwar, Najib ul Haque, Imran Ahmed, Noshad Ali, Rehman Tahir, and Zulfiqar A Bhutta. 2017. “Community Engagement and Integrated Health and Polio Immunisation Campaigns in Conflict-Affected Areas of Pakistan: A Cluster Randomised Controlled Trial.” The Lancet Global Health 5 (6): e593–603. https://doi.org/10/f962m2.

Hagiwara, Akiko, Mika Ueyama, Asad Ramlawi, and Yasuyuki Sawada. 2013. “Is the Maternal and Child Health (MCH) Handbook Effective in Improving Health-Related Behavior? Evidence from Palestine.” Journal of Public Health Policy 34 (1): 31–45. https://doi.org/10/ghpbss.

Haji, Adam Hassan. 2017. “Evaluation of SMS and Sticker Reminders in Reducing Dropout Rates in Routine Child Immunization in Selected Districts in Kenya.” Master Thesis, Juja: Jomo Kenyatta University of Agriculture and Technology. http://ir.jkuat.ac.ke/handle/123456789/3069.

Hajizadeh, Mohammad, Jody Heymann, Erin Strumpf, Sam Harper, and Arijit Nandi. 2015. “Paid Maternity Leave and Childhood Vaccination Uptake: Longitudinal Evidence from 20 Low-and-Middle-Income Countries.” Social Science & Medicine 140 (September): 104–17. https://doi.org/10/f7ppdx.

Hategeka, Celestin, Hinda Ruton, and Michael R. Law. 2019. “Effect of a Community Health Worker MHealth Monitoring System on Uptake of Maternal and Newborn Health Services in Rwanda.” Global Health Research and Policy 4 (1). https://doi.org/10/ghpbst.

Heinrich, Carolyn J., and Matthew T. Knowles. 2020. “A Fine Predicament: Conditioning, Compliance and Consequences in a Labeled Cash Transfer Program.” World Development 129 (May). https://doi.org/10/ghpbsv.

For peer review only - http://bmjopen.bmj.com/site/about/guidelines.xhtml

Helleringer, Stephane, Patrick O. Asuming, and Jalaa Abdelwahab. 2016. "The Effect of Mass Vaccination Campaigns against Polio on the Utilization of Routine Immunization Services: A Regression Discontinuity Design." Vaccine 34 (33): 3817–22. https://doi.org/10/f8w56m.

Hu, Yu, Yaping Chen, Ying Wang, Quanwei Song, and Qian Li. 2017. "Prenatal Vaccination Education Intervention Improves Both the Mothers' Knowledge and Children's Vaccination Coverage: Evidence from Randomized Controlled Trial from Eastern China." Human Vaccines & Immunotherapeutics 13 (6): 1477–84. https://doi.org/10/gghf3f.

Huillery, Elise, and Juliette Seban. 2019. "Financial Incentives, Efforts, and Performances in the Health Sector: Experimental Evidence from the Democratic Republic of Congo." Economic Development and Cultural Change, March. https://doi.org/10/ghpbsq.

Hutchinson, Paul, Peter Lance, David K. Guilkey, Mohammad Shahjahan, and Shahida Haque. 2006. "Measuring the Cost-Effectiveness of a National Health Communication Program in Rural Bangladesh." Journal of Health Communication 11 (sup2): 91–121. https://doi.org/10/b7wdkt.

Ikilezi, Gloria, Orvalho J. Augusto, Joseph L. Dieleman, Kenneth Sherr, and Stephen S. Lim. 2020. "Effect of Donor Funding for Immunization from GAVI and Other Development Assistance Channels on Vaccine Coverage: Evidence from 120 Low and Middle Income Recipient Countries." Vaccine 38 (3): 588–96. https://doi.org/10/ghpwdv.

Ikilezi, Gloria, Orvalho J Augusto, Alyssa Sbarra, Kenneth Sherr, Joseph L Dieleman, and Stephen S Lim. 2020. "Determinants of Geographical Inequalities for DTP3 Vaccine Coverage in Sub-Saharan Africa." Vaccine 38 (18): 3447–54. https://doi.org/10/ghpwdw.

Jaupart, Pascal, Lizzie Dipple, and Stefan Dercon. 2019. "Has Gavi Lived up to Its Promise? Quasi-Experimental Evidence on Country Immunisation Rates and Child Mortality." BMJ Global Health 4 (6). https://doi.org/10/ghpwdx.

Kagucia, E Wangeci. 2018. "MHealth Interventions to Improve Measles Vaccination Coverage and Timeliness: An Assessment of the Immediate and Long-Term Impact on Vaccine-Seeking in Rural Kenya." PhD Thesis, Baltimore, MD: Johns Hopkins University. https://jscholarship.library.jhu.edu/handle/1774.2/61130.

Kamatsuchi, Mahoko, Adrian Gheorghe, and Dina Balabanova. 2019. "The Global Scale and Implications of Delivering Multiple Interventions through Integrated Child Health Events." BMJ Global Health 4 (4). https://doi.org/10/ghpwd2.

Kandpal, Eeshani, Harold Alderman, Jed Friedman, Deon Filmer, Junko Onishi, and Jorge Avalos. 2016. "A Conditional Cash Transfer Program in the Philippines Reduces Severe Stunting." The Journal of Nutrition 146 (9): 1793–1800. https://doi.org/10/f8zrr6.

Karing, Anne. 2018. "Social Signaling and Childhood Immunization: A Field Experiment in Sierra Leone." Berkeley Working Paper. Berkeley: University of California. https://economics.yale.edu/sites/default/files/jmp_socialsignaling.pdf.

Kawakatsu, Yoshito, Tomohiko Sugishita, Kennedy Oruenjo, Stephen Wakhule, Kennedy Kibosia, Eric Were, and Sumihisa Honda. 2015. "Effectiveness of and Factors Related to Possession of a Mother and Child Health Handbook: An Analysis Using Propensity Score Matching." Health Education Research 30 (October): 935–46. https://doi.org/10/f749wb.

Kazi, Abdul Momin, Murtaza Ali, Khurram Zubair, Hussain Kalimuddin, Abdul Nafey Kazi, Saleem Perwaiz Iqbal, Jean-Paul Collet, and Syed Asad Ali. 2018. "Effect of Mobile Phone Text Message Reminders on Routine Immunization Uptake in Pakistan: Randomized Controlled Trial." JMIR Public Health and Surveillance 4 (1). https://doi.org/10/ghpwd3.

Kern, Ana Paula, Marcel de Toledo Vieira, and Ricardo da Silva Freguglia. 2018. "Impactos Do Programa Bolsa Família Na Imunização Das Crianças." Working paper. https://www.anpec.org.br/encontro/2018/submissao/files_I/i12-cb6dfc0f070c8ca9e77abc8563c3801f.pdf.

Khaleghian, Peyvand. 2003. "Decentralization and Public Services: The Case of Immunization." Policy Research Working Paper 2989. Washington: The World Bank. https://openknowledge.worldbank.org/handle/10986/19159?show=full.

For peer review only - http://bmjopen.bmj.com/site/about/guidelines.xhtml

Kusuma, Dian, Hasbullah Thabrany, Budi Hidayat, Margaret McConnell, Peter Berman, and Jessica Cohen. 2017. “New Evidence on the Impact of Large-Scale Conditional Cash Transfers on Child Vaccination Rates: The Case of a Clustered-Randomized Trial in Indonesia.” World Development 98 (October): 497–505. https://doi.org/10/ghpwd4.

Lamanna, Camillo, and Lauren Byrne. 2019. “A Pilot Study of a Novel, Incentivised MHealth Technology to Monitor the Vaccine Supply Chain in Rural Zambia.” Pan African Medical Journal 33. https://doi.org/10/ghpwp5.

Levy, Dan, and Jim Ohls. 2010. “Evaluation of Jamaica’s PATH Conditional Cash Transfer Programme.” Journal of Development Effectiveness 2 (4): 421–41. https://doi.org/10/dtkp26.

Lin, Meng Ann. 2016. “Stimulating Demand: An Assessment of the Conditional Cash Transfer Project in Afghanistan.” PhD Thesis, Baltimore: Johns Hopkins University. https://jscholarship.library.jhu.edu/handle/1774.2/39740.

Liu, Yong, Zhaokang Yuan, Yuxi Liu, Upali W Jayasinghe, and Mark F Harris. 2014. “Changing Community Health Service Delivery in Economically Less-Developed Rural Areas in China: Impact on Service Use and Satisfaction.” BMJ Open 4 (2). https://doi.org/10/f6mwb9.

Loevinsohn, B P, and E Gareaballah. 1992. “Missed Opportunities for Immunization during Visits for Curative Care: A Randomized Cross-over Trial in Sudan.” Bulletin of the World Health Organization 70 (3): 335–39. https://www.ncbi.nlm.nih.gov/pmc/articles/PMC2393287/.

Loevinsohn, Benjamin P., and Michael E. Loevinsohn. 1986. “Improvement in Coverage of Primary Health Care in a Developing Country through Use of Food Incentives.” The Lancet 327 (8493): 1314–16. https://doi.org/10/bjgw7d.

Looij, Frank van de, Dudzai Mureyi, Chenjerai Sisimayi, Jaap Koot, Portia Manangazira, and Nyasha Musuka. 2015. “Early Evidence from Results-Based Financing in Rural Zimbabwe.” African Health Monitor 6: 32–36. https://collaboration.worldbank.org/content/sites/collaboration-for-development/en/groups/results-based-financing/groups/oba-rbf-newsroom/documents.entry.html/2016/01/14/early_evidence_from-Kgih.html.

Maluccio, John A., and Rafael Flores. 2005. “Impact Evaluation of a Conditional Cash Transfer Program the Nicaraguan Red de Protección Social.” Research report 141. 0 ed. Washington, DC: International Food Policy Research Institute. https://doi.org/10.2499/0896291464RR141.

Mampe, Tumelo, Helen Schneider, and Gavin Reagon. 2016. “Effectiveness of Ward Based Outreach Teams in the North West Province: An Evaluation.” Cape Town: University of the Western Cape. http://rgdoi.net/10.13140/RG.2.2.36191.66725.

Masuda, Kazuya, and Chikako Yamauchi. 2020. “How Does Female Education Reduce Adolescent Pregnancy and Improve Child Health? Evidence from Uganda’s Universal Primary Education for Fully Treated Cohorts.” The Journal of Development Studies 56 (1): 63–86. https://doi.org/10/ggv6vq.

Mathanga, Don P., Elizabeth T. Luman, Carl H. Campbell, Chimwemwe Silwimba, and Grace Malenga. 2009. “Integration of Insecticide-Treated Net Distribution into Routine Immunization Services in Malawi: A Pilot Study.” Tropical Medicine & International Health 14 (7): 792–801. https://doi.org/10/b44rkh.

Mazumder, S., S. Taneja, R. Bahl, P. Mohan, T. A. Strand, H. Sommerfelt, B. R. Kirkwood, et al. 2014. “Effect of Implementation of Integrated Management of Neonatal and Childhood Illness Programme on Treatment Seeking Practices for Morbidities in Infants: Cluster Randomised Trial.” BMJ 349 (August). https://doi.org/10/gb3snq.

McMahon, Shannon A., Stephan Brenner, Julia Lohmann, Christopher Makwero, Aleksandra Torbica, Don P. Mathanga, Adamson S. Muula, and Manuela De Allegri. 2016. “Evaluating Complex Health Financing Interventions: Using Mixed Methods to Inform Further Implementation of a Novel PBI Intervention in Rural Malawi.” BMC Health Services Research 16 (1). https://doi.org/10/f8zjkr.

Mehta, Kala M., Francois Rerolle, Sonali V. Rammohan, Davis C. Albohm, George Muwowo, Heidi Moseson, Lesley Sept, Hau L. Lee, and Eran Bendavid. 2016. “Systematic Motorcycle Management and Health Care Delivery: A Field Trial.” American Journal of Public Health 106 (1): 87–94. https://doi.org/10/f8hd4m.

For peer review only - http://bmjopen.bmj.com/site/about/guidelines.xhtml

Mensah, Joseph, Joseph R Oppong, and Christoph M Schmidt. 2009. “Ghana’s National Health Insurance Scheme in the Context of the Health MDGs: An Empirical Evaluation Using Propensity Score Matching.” Ruhr Economic Papers 157. https://papers.ssrn.com/sol3/papers.cfm?abstract_id=1532169.

Mohan, Pavitrra, Baya Kishore, Sharad Singh, Rajiv Bahl, Anju Puri, and Rajesh Kumar. 2012. “Assessment of Implementation of Integrated Management of Neonatal and Childhood Illness in India.” Journal of Health, Population and Nutrition 29 (6): 629–38. https://doi.org/10/ghpwft.

Musa, O I, D B Parakoyi, and A A Akanbi. 2006. “Evaluation of Health Education Intervention on Safe Immunization Injection among Health Workers in Ilorin, Nigeria.” Annals of African Medicine 5 (3): 122–28. https://www.semanticscholar.org/paper/Evaluation-of-Health-Education-Intervention-on-Safe-Musa-Parakoyi/5f3eaa0090f82149c7de44c442ec094d31a04591?p2df.

Nanyunja, Miriam, Rosamund F. Lewis, Issa Makumbi, Rachel Seruyange, Eva Kabwongera, Possy Mugyenyi, and Ambrose Talisuna. 2003. “Impact of Mass Measles Campaigns among Children Less than 5 Years Old in Uganda.” The Journal of Infectious Diseases 187 (s1): S63–68. https://doi.org/10/bzksr6.

Nasir, Narila Mutia, Yuli Amran, and Yasuhide Nakamura. 2017. “Changing Knowledge and Practices of Mothers on Newborn Care through Mother Class: An Intervention Study in Indonesia.” Journal of Tropical Pediatrics 63 (6): 440–46. https://doi.org/10/gghf3q.

Onishi, Junko. 2014. “Philippines Conditional Cash Transfer Program Impact Evaluation 2012.” World Bank Report 75533-PH. Washington, DC: The World Bank. https://assessments.hpc.tools/sites/default/files/assessments/Philippines%20Conditional%20Cash%20Transfer%20Program%2C%20Impact%20Evaluation%202012.pdf.

Owais, Aatekah, Beenish Hanif, Amna R Siddiqui, Ajmal Agha, and Anita KM Zaidi. 2011. “Does Improving Maternal Knowledge of Vaccines Impact Infant Immunization Rates? A Community-Based Randomized-Controlled Trial in Karachi, Pakistan.” BMC Public Health 11 (1). https://doi.org/10/bpswbk.

Özer, Mustafa, Jan Fidrmuc, and Mehmet Ali Eryurt. 2018. “Maternal Education and Childhood Immunization in Turkey.” Health Economics 27 (8): 1218–29. https://doi.org/10/gdkgd3.

Padilla, Alcides de J., and Juan C. Trujillo. 2015. “An Impact Assessment of the Child Growth, Development and Care Program in the Caribbean Region of Colombia.” Cadernos de Saúde Pública 31 (10): 2099–2109. https://doi.org/10/gghf3w.

Pandey, Priyanka, Ashwini R. Sehgal, Michelle Riboud, David Levine, and Madhav Goyal. 2007. “Informing Resource-Poor Populations and the Delivery of Entitled Health and Social Services in Rural India: A Cluster Randomized Controlled Trial.” JAMA 298 (16): 1867–75. https://doi.org/10/ddxdhz.

Pathak, Yuvraj, and Karen Macours. 2017. “Women’s Political Reservation, Early Childhood Development, and Learning in India.” Economic Development and Cultural Change 65 (4): 741–66. https://doi.org/10/ghpwf6.

Powell-Jackson, Timothy, Camilla Fabbri, Varun Dutt, Sarah Tougher, and Kultar Singh. 2018. “Effect and Cost-Effectiveness of Educating Mothers about Childhood DPT Vaccination on Immunisation Uptake, Knowledge, and Perceptions in Uttar Pradesh, India: A Randomised Controlled Trial.” Edited by James K. Tumwine. PLOS Medicine 15 (3). https://doi.org/10/gc4pqg.

Pramanik, Santanu, Arpita Ghosh, Rituu B. Nanda, Marlou de Rouw, Philip Forth, and Sandra Albert. 2018. “Impact Evaluation of a Community Engagement Intervention in Improving Childhood Immunization Coverage: A Cluster Randomized Controlled Trial in Assam, India.” BMC Public Health 18 (1). https://doi.org/10/gdhbjf.

Prinja, Shankar, Ruby Nimesh, Aditi Gupta, Pankaj Bahuguna, Madhu Gupta, and Jarnail Singh Thakur. 2017. “Impact of M-Health Application Used by Community Health Volunteers on Improving Utilisation of Maternal, New-Born and Child Health Care Services in a Rural Area of Uttar Pradesh, India.” Tropical Medicine & International Health 22 (7): 895–907. https://doi.org/10/gghf35.

Prosser, Wendy, Philippe Jaillard, Emmanuelle Assy, Shawn T. Brown, Graça Matsinhe, Mawutondji Dekoun, and Bruce Y. Lee. 2017. “System Redesign of the Immunization Supply Chain: Experiences from Benin and Mozambique.” Vaccine 35 (17): 2162–66. https://doi.org/10/f96jsd.

For peer review only - http://bmjopen.bmj.com/site/about/guidelines.xhtml

Rahman, Mohammad Mahbubur, and Saseendran Pallikadavath. 2019. “Maternal and Child Health Care Services’ Utilization Data from the Fourth Round of District Level Household Survey in India.” Data in Brief 23 (April). https://doi.org/10/ghpwgm.

Rajkotia, Yogesh, Omer Zang, Pierre Nguimkeu, Jessica Gergen, Iva Djurovic, Paula Vaz, Franscisco Mbofana, and Kebba Jobarteh. 2017. “The Effect of a Performance-Based Financing Program on HIV and Maternal/Child Health Services in Mozambique—an Impact Evaluation.” Health Policy and Planning 32 (10): 1386–96. https://doi.org/10/gcfv5b.

Rasella, Davide, Rosana Aquino, Carlos AT Santos, Rômulo Paes-Sousa, and Mauricio L Barreto. 2013. “Effect of a Conditional Cash Transfer Programme on Childhood Mortality: A Nationwide Analysis of Brazilian Municipalities.” The Lancet 382 (9886): 57–64. https://doi.org/10/f2kfx4.

Reinbold, Gary W. 2019. “Effects of the Convention on the Rights of the Child on Child Mortality and Vaccination Rates: A Synthetic Control Analysis.” BMC International Health and Human Rights 19 (1). https://doi.org/10/ghgdqs.

Robinson, J Stephen, Barton R Burkhalter, Barbie Rasmussen, and Ristianto Sugiono. 2001. “Low-Cost on-the-Job Peer Training of Nurses Improved Immunization Coverage in Indonesia.” Bulletin of the World Health Organization 79 (2): 150–58. https://www.scielosp.org/article/bwho/2001.v79n2/150-158/en/.

Roth, A. E., C. Stabell Benn, H. Ravn, A. Rodrigues, I. M. Lisse, M. Yazdanbakhsh, H. Whittle, and P. Aaby. 2010. “Effect of Revaccination with BCG in Early Childhood on Mortality: Randomised Trial in Guinea-Bissau.” BMJ 340 (mar15 1). https://doi.org/10/c8q74b.

Rusa, Louis, Jean de Dieu Ngirabega, Willy Janssen, Stefaan Van Bastelaere, Denis Porignon, and Werner Vandenbulcke. 2009. “Performance-Based Financing for Better Quality of Services in Rwandan Health Centres: 3-Year Experience.” Tropical Medicine & International Health 14 (7): 830–37. https://doi.org/10/cc5zvh.

Ryman, Tove K., Elizabeth C. Briere, Emily Cartwright, Karen Schlanger, Kathleen A. Wannemuehler, Elizabeth T. Russo, Steve Kola, et al. 2012. “Integration of Routine Vaccination and Hygiene Interventions: A Comparison of 2 Strategies in Kenya.” The Journal of Infectious Diseases 205 (suppl_1): S65–76. https://doi.org/10/f3wkpb.

Ryman, Tove K., Ajay Trakroo, Aaron Wallace, Satish Kumar Gupta, Karen Wilkins, Pankaj Mehta, and Vance Dietz. 2011. “Implementation and Evaluation of the Reaching Every District (RED) Strategy in Assam, India, 2005–2008.” Vaccine 29 (14): 2555–60. https://doi.org/10/bjqf85.

Salami, Lamidhi, Edgard-Marius Dona Ouendo, and Benjamin Fayomi. 2016. “Effects of Results Based Financing Models on Data Quality Improvement in Benin on 2014.” Universal Journal of Public Health 4 (6): 324–31. https://doi.org/10/ghpwgc.

Salami, Lamidhi, Edgard-Marius Ouendo, and Benjamin Fayomi. 2018. “Effects of Results Based Financing Models on the Performance of Exposed Health Zones in Benin.” International Journal Of Community Medicine And Public Health 5 (10): 4188–99. https://doi.org/10/ghpwgd.

Sangwan, Santosh, and Anju Manocha. 2009. “Maternal Knowledge and Child Health.” Journal of Human Ecology 25 (1): 51–54. https://doi.org/10/gghf33.

Sato, Ryoko, and Abdullahi Belel. 2020. “The Effect of Performance-Based Financing on Child Vaccinations in Northern Nigeria.” Vaccine 38 (9): 2209–15. https://doi.org/10/ghpwgf.

Schlumberger, M., A. Bamoko, T.M. Yaméogo, F. Rouvet, R. Ouedraogo, B. Traoré, M. Tinto, et al. 2015. “Impact positif sur le Programme élargi de vaccinations de l’envoi de SMS de rappel à partir d’un registre informatisé, Bobo-Dioulasso (Burkina Faso).” Bulletin de la Société de pathologie exotique 108 (5): 349–54. https://doi.org/10/gghf34.

Sengupta, Nandana, and Aakanksha Sinha. 2018. “Is India’s Safe Motherhood Scheme Leading to Better Child Health Care Practices?” Global Social Welfare 5 (1): 49–58. https://doi.org/10/ghpwgg.

Shei, Amie, Federico Costa, Mitermayer G Reis, and Albert I Ko. 2014. “The Impact of Brazil’s Bolsa Família Conditional Cash Transfer Program on Children’s Health Care Utilization and Health Outcomes.” BMC International Health and Human Rights 14 (1). https://doi.org/10/gbf36j.

For peer review only - http://bmjopen.bmj.com/site/about/guidelines.xhtml

Sherry, Tisamarie B., Sebastian Bauhoff, and Manoj Mohanan. 2017. “Multitasking and Heterogeneous Treatment Effects in Pay-for-Performance in Health Care: Evidence from Rwanda.” American Journal of Health Economics 3 (2): 192–226. https://doi.org/10/ghpwgh.

Shuaib, Waqas, Julia Marielly Suarez, Juan David Romero, Carlos Dillon Pamello, Richard Alweis, Aizaaz Ali Khan, Syed Raza Shah, Hassan Shahid, Serge B. PierreCharles, and Laura Rosemary Sanchez. 2016. “Transforming Patient Care by Introducing an Electronic Medical Records Initiative in a Developing Country.” Health Informatics Journal 22 (4): 975–83. https://doi.org/10/gghf36.

Sinha, Nistha, and Joanne K Yoong. 2009. “Long-Term Financial Incentives and Investment in Daughters: Evidence from Conditional Cash Transfers in North India.” RAND Labor and Population working paper series WR-667. Santa Monica: RAND Corporation. https://doi.org/10.1596/1813-9450-4860.

Soeters, Robert, Peter Bob Peerenboom, Pacifique Mushagalusa, and Célestin Kimanuka. 2011. “Performance-Based Financing Experiment Improved Health Care in the Democratic Republic of Congo.” Health Affairs 30 (8): 1518–27. https://doi.org/10/c6w2rq.

Steinhardt, L. C., I. Aman, I. Pakzad, B. Kumar, L. P. Singh, and D. H. Peters. 2011. “Removing User Fees for Basic Health Services: A Pilot Study and National Roll-out in Afghanistan.” Health Policy and Planning 26 (Suppl. 2): ii92–103. https://doi.org/10/drchbp.

Subedi, Sudarshan, Bishnu Prasad Sharma, and Chiranjivi Adhikari. 2018. “Cost and Consequences of Integrated Public Health Campaign in Baglung District, Nepal.” Economic Journal of Development Issues 23 & 24 (January): 113–22. https://doi.org/10/ghpwgk.

Talukder, Md., Ubaidur Rob, Syed Abu Musa, Ashish Bajracharya, Kaji Keya, Forhana Noor, Eshita Jahan, Md. Hossain, Jyotirmoy Saha, and Benjamin Bellows. 2014. “Evaluation of the Impact of the Voucher Program for Improving Maternal Health Behavior and Status in Bangladesh.” Dhaka: Population Council. https://doi.org/10.31899/rh10.1000.

Tanner, Jeffery, Ryotaro Hayashi, and Yunsun Li. 2015. “Improving Coverage and Utilization of Maternal and Child Health Services in Lao PDR: Impact Evaluation of the Community Nutrition Project.” IEG Working Paper 99929. Washington: The World Bank Group. https://documents.worldbank.org/en/publication/documents-reports/documentdetail/441841468187767237/improving-coverage-and-utilization-of-maternal-and-child-health-services-in-lao-pdr-impact-evaluation-of-the-community-nutrition-project.

Thomas, Ranjeeta Alice. 2011. “Essays on Ex Ante Evaluations of Cash Transfer Programs.” PhD Thesis, York: University of York. http://etheses.whiterose.ac.uk/2004/.

Thomson, Dana R, Cheryl Amoroso, Sidney Atwood, Matthew H Bonds, Felix Cyamatare Rwabukwisi, Peter Drobac, Karen E Finnegan, et al. 2018. “Impact of a Health System Strengthening Intervention on Maternal and Child Health Outputs and Outcomes in Rural Rwanda 2005–2010.” BMJ Global Health 3 (2). https://doi.org/10/gdcrw5.

Thysen, Sanne Marie, Stine Byberg, Marie Pedersen, Amabelia Rodrigues, Henrik Ravn, Cesario Martins, Christine Stabell Benn, Peter Aaby, and Ane Bærent Fisker. 2014. “BCG Coverage and Barriers to BCG Vaccination in Guinea-Bissau: An Observational Study.” BMC Public Health 14 (1). https://doi.org/10/f6k4xv.

Uddin, Md. Jasim, Md. Shamsuzzaman, Lily Horng, Alain Labrique, Lavanya Vasudevan, Kelsey Zeller, Mridul Chowdhury, Charles P. Larson, David Bishai, and Nurul Alam. 2016. “Use of Mobile Phones for Improving Vaccination Coverage among Children Living in Rural Hard-to-Reach Areas and Urban Streets of Bangladesh.” Vaccine 34 (2): 276–83. https://doi.org/10/f766t2.

Unger, Jean-Pierre. 1991. “Can Intensive Campaigns Dynamize Front Line Health Services? The Evaluation of an Immunization Campaign in Thiès Health District, Senegal.” Social Science & Medicine 32 (3): 249–59. https://doi.org/10/bc5fn2.

Usman, Hussain R., Saeed Akhtar, Faiza Habib, and Imtiaz Jehan. 2009. “Redesigned Immunization Card and Center-Based Education to Reduce Childhood Immunization Dropouts in Urban Pakistan: A Randomized Controlled Trial.” Vaccine 27 (3): 467–72. https://doi.org/10/bcv8fd.

For peer review only - http://bmjopen.bmj.com/site/about/guidelines.xhtml

Usman, Hussain R., Mohammad H. Rahbar, Sibylle Kristensen, Sten H. Vermund, Russell S. Kirby, Faiza Habib, and Eric Chamot. 2011. “Randomized Controlled Trial to Improve Childhood Immunization Adherence in Rural Pakistan: Redesigned Immunization Card and Maternal Education.” Tropical Medicine & International Health 16 (3): 334–42. https://doi.org/10/dm9tm7.

Vaidyanathan, Radha. 2019. “Immunization Coverage among Under-Five Children Living along a School Student through Child-to-Child and Child-to-Parent Information, Education and Communication Strategy.” Indian Journal of Public Health 63 (4): 334–40. https://doi.org/10/ghpwgq.

Van de Poel, Ellen, Gabriela Flores, Por Ir, and Owen O’Donnell. 2016. “Impact of Performance-Based Financing in a Low-Resource Setting: A Decade of Experience in Cambodia.” Health Economics 25 (6): 688–705. https://doi.org/10/f8kzhk.

Varghese, Beena, Reetabrata Roy, Somen Saha, and Sidsel Roalkvam. 2014. “Fostering Maternal and Newborn Care in India the Yashoda Way: Does This Improve Maternal and Newborn Care Practices during Institutional Delivery?” Edited by Hamid Reza Baradaran. PLoS ONE 9 (1). https://doi.org/10/f5s7zr.

Verma, M, J Chhatwal, and P V Varughese. 1995. “Antenatal Period: An Educational Opportunity.” Indian Pediatrics 32: 171–77. https://www.semanticscholar.org/paper/Antenatal-period%3A-an-educational-opportunity.-Verma-Chhatwal/b44c9c679879ada229d8a2719b5637155190f4d1?p2df.

Wadhwa, Sagar. 2019. “Conditional Cash Transfers and Parental Investment in Daughters: Evidence from India.” Paper presented at the Population Association of America 2019 Annual Meeting, Austin, TX. http://paa2019.populationassociation.org/abstracts/193315.

Wagstaff, Adam, and Shengchao Yu. 2007. “Do Health Sector Reforms Have Their Intended Impacts ? The World Bank’S Health VIII Project in Gansu Province, China.” Journal of Health Economics 26: 505–35. https://doi.org/10/cx4kc6.

Wallace, Aaron S., Kenny Peetosutan, Andi Untung, Marisa Ricardo, Prima Yosephine, Kathleen Wannemuehler, David W. Brown, et al. 2019. “Home-Based Records and Vaccination Appointment Stickers as Parental Reminders to Reduce Vaccination Dropout in Indonesia: A Cluster-Randomized Controlled Trial.” Vaccine 37 (45): 6814–23. https://doi.org/10/ghpwgw.

Walque, Damien de, Paul Jacob Robyn, Hamadou Saidou, Gaston Sorgho, and Maria Steenland. 2017. “Looking into the Performance-Based Financing Black Box: Evidence from an Impact Evaluation in the Health Sector in Cameroon.” Policy Research Working Paper 8162. Washington, DC: The World Bank. https://doi.org/10.1596/1813-9450-8162.

Wang, Paul C., Albert Mwango, Sarah Moberley, Benjamin J. Brockman, Alison L. Connor, Penelope Kalesha-Masumbu, Simon Mutembo, et al. 2015. “A Cluster Randomised Trial on the Impact of Integrating Early Infant HIV Diagnosis with the Expanded Programme on Immunization on Immunization and HIV Testing Rates in Rural Health Facilities in Southern Zambia.” Edited by David Joseph Diemert. PLOS ONE 10 (10). https://doi.org/10/f779tz.

Weldemariam, Mesele. 2010. “The Impact of Fiscal Decentralization on Education and Health Outcomes in Ethiopia: A Regional Panel Data Analysis.” Master Thesis, Addis Ababa: Addis Ababa University. http://etd.aau.edu.et/handle/123456789/14217?show=full.

Wong, Benjamin KC, Shaza A Fadel, Shally Awasthi, Ajay Khera, Rajesh Kumar, Geetha Menon, and Prabhat Jha. 2019. “The Impact of Measles Immunization Campaigns in India Using a Nationally Representative Sample of 27,000 Child Deaths.” ELife 8 (March). https://doi.org/10/ghpwgx.

Yoong, Joanne. 2007. “Does Decentralization Hurt Childhood Immunization?” Stanford: Stanford University. http://citeseerx.ist.psu.edu/viewdoc/summary?doi=10.1.1.370.4021.

Yoosuf, Abdul-Sattar. 1993. “Group Learning by Mothers about Primary Health Care.” World Health Forum 14 (1): 20–22. https://pubmed.ncbi.nlm.nih.gov/8439363/.

Zang, Omer, Sebastien Djienouassi, Gaston Sorgho, and Jean Claude Taptue. 2015. “Impact of Performance-Based Financing on Health-Care Quality and Utilization in Urban Areas of Cameroon.” African Health Monitor 7 (21): 10–14. https://indexmedicus.afro.who.int/aim/opac_css/doc_num.php?explnum_id=72209.

For peer review only - http://bmjopen.bmj.com/site/about/guidelines.xhtml

Zeng, Wu, Donald S Shepard, Jean de Dieu Rusatira, Aaron P Blaakman, and Bernice M Nsitou. 2018. "Evaluation of Results-Based Financing in the Republic of the Congo: A Comparison Group Pre–Post Study." Health Policy and Planning 33 (3): 392–400. https://doi.org/10/gcvmpw.

Zeng, Wu, Daxin Sun, Henry Mphwanthe, Tianwen Huan, Jae Eun Nam, Pascal Saint-Firmin, Gerald Manthalu, Suneeta Sharma, and Arin Dutta. 2019. "The Impact and Cost-Effectiveness of User Fee Exemption by Contracting out Essential Health Package Services in Malawi." BMJ Global Health 4 (2). https://doi.org/10/gghf4b.

Zizien, Zawora Rita, Catherine Korachais, Philippe Compaoré, Valéry Ridde, and Vincent De Brouwere. 2019. "Contribution of the Results-based Financing Strategy to Improving Maternal and Child Health Indicators in Burkina Faso." The International Journal of Health Planning and Management 34 (1): 111–29. https://doi.org/10/gd2zrp.

Zombré, David, Manuela De Allegri, and Valéry Ridde. 2020. "No Effects of Pilot Performance-Based Intervention Implementation and Withdrawal on the Coverage of Maternal and Child Health Services in the Koulikoro Region, Mali: An Interrupted Time Series Analysis." Health Policy and Planning 35 (4): 379–87. https://doi.org/10/ggnfpf.

For peer review only

For peer review only - http://bmjopen.bmj.com/site/about/guidelines.xhtml

Appendix 5: Qualitative and cost-evidence search strategy

Qualitative search protocol

Purpose

The purpose of this review is to inform policy recommendations related to community engagement interventions to improve immunisation outcomes in low- and middle-income countries. Specifically, we would like to provide insights related to (1) what to do / not do and (2) where to do it / not do it. We want to know not only what works, but why and how.

Our primary, qualitative research question is: What factors relating to programme design, implementation, context, and mechanism are associated with better or worse outcomes along the causal chain? Do these vary by the level of community engagement?

Sub questions are:

1. What pre-intervention activities and characteristics of local context facilitate or inhibit the effectiveness of an intervention?
    a. What characteristics of local context facilitate or inhibit the effectiveness of an intervention? These include but are not limited to feasibility, clinic readiness, acceptability, need, barriers, and demographic composition.
    b. What is the evidence regarding the likelihood that pre-intervention activities, including diagnostics, formative research, and community engagement, affect the effectiveness of an intervention?
2. What is the evidence supporting or refuting the existence of certain steps along the ToC?
    a. Which steps in the causal chain tend to be barriers to or facilitators of impact? Where does the causal chain tend to be interrupted?
    b. What time varying contextual factors affect the relationships seen in the ToC?

In order to respond to these questions, 3ie seeks to identify qualitative papers related to the quantitative papers included in the broader systematic review on effectiveness of community engagement interventions for improving immunisation outcomes. This information will contextualise the results of the quantitative papers. The qualitative papers will be instrumental in expanding our understanding of why and how certain interventions were successful (or not).

This document outlines the suggested approach for identifying these qualitative papers.

Required steps

1. Create a unique dropbox folder for each study using the first author's last name and year as the folder title
2. For each step outlined below, save all potentially relevant documents to this file as they are identified.

    a. Consider this stage to be equivalent to title and abstract screening. If you identify an article that can be quickly discarded as irrelevant (such as budgetary information), there is no need to include. However, if you think that there may be any information related to our research questions, please include at this stage.

    b. If relevant websites are identified that cannot be easily converted to PDF format, create a word document. Write the title of the webpage and provide the link below it.

If a trial registration number for clinicaltrials.gov is provided

For peer review only - http://bmjopen.bmj.com/site/about/guidelines.xhtml

Use this approach if a trial registration number for clinicaltrials.gov is provided. This will be referenced in the abstract and / methods section and appear in a form similar to that found in Robertson et al 2013:

This trial is registered with ClinicalTrials.gov, number NCT00966849.

If no trial registration number is indicated, proceed to the following section (Section 2: If the project has a unique name).

1.1 Go to the website clinialTrials.gov

1.2 Under "find a study" in the field "other terms," enter the trial registration number.

1.3 There should only be one hit. Click on the title under "study title"

1.4 Three tabs should be available under the basic study information. They read "study details," "tabular view," and "study results." Select "study results"

1.5 Scroll to the bottom of the page. Under "more information" there are sections titled "Publication of results" and "other publications"

a. In most (but not all) cases, there should be a list of publications here. Save the relevant files to the folder

After this, proceed to section 2.

If the project has an unique name

Use this approach if the intervention is named. If a project name is not given in the paper, but the project is registered on clinicaltrials.gov, use the project title provided there.

2.1 Search the project name on google scholar.

a. Review each search result until you find 5 in a row that are irrelevant.

b. At this point, stop and proceed to step 2.2.

2.2 If the funder's name is not provided, proceed directly to step 2.3. If the funder's name is provided, search the project name on the funder's website.

a. Review each search result until you find 5 in a row that are irrelevant.

b. At this point, stop and proceed to step 2.3.

2.3 If the implementer's name is not provided, proceed directly to section 3. If the implementer's name is provided, search the project name on the implementer's website.

a. Review each search result until you find 5 in a row that are irrelevant.

Other options

If the trial is not registered and does not have a formal name, the following approaches can be used as a last resort:

For peer review only - http://bmjopen.bmj.com/site/about/guidelines.xhtml

3.1 If funder and / or implementer names are provided, go to their websites. Search for the article on their website. It may link to a program page.

a. If no program page is identified, try to manually search through their website.

3.2 Search the full article name on google and google scholar. Review the top 10 hits on each to see if they provide more information about the program.

If during either of these steps, a program name and / or trial registration number are identified, return to the relevant steps above.

Searching funder and implementer websites

Some of these websites will have good, built-in search functions. If these are useful, use them. However, if the provided search functionality is not adequate, type site:HomepageOfFunderWebsite SearchTerm. For example, if I wanted to search the World Bank website for information about COVID, I would search "site:www.worldbank.org COVID" in google (Note: no space between "site:" and the url). This will provide me hits from the World Bank website related to COVID.

If no linked articles are identified

At the end of each day, email Avantika (abagai@3ieimpact.org) and Charlotte (clane@3ieimpact.org) the title of all papers for which you were unable to identify linked articles.

Cost evidence search protocol

The evaluations included in the cost and cost-effectiveness evidence were drawn from the 61 evaluations that were identified for the systematic review. Since cost reporting and analysis is often not required by donors or included in impact evaluations of global development interventions, we undertook structured outreach to the authors of the 61 included studies to request any additional cost evidence. In case the lead or corresponding author email addresses were nonfunctional, we contacted one of the co-authors.

We successfully contacted 58 out of 61 study authors that reported intervention effectiveness only or intervention effectiveness and partial cost information and requested estimates of the intervention costs as well as any additional raw data files, summary tables, sensitivity analyses, cost evidence, published reports or notes, and non-published documentation, methods or analyses relating to cost-effectiveness. In the first two weeks of this request, our team obtained responses from a total of 25 study authors, of which 12 produced or pointed us in the direction of economic evidence for their respective interventions and 7 authors confirmed that any such costing analysis was not undertaken as part of their study. We excluded any studies that did not report estimates of both costs and effectiveness and identified 22 evaluations with both cost and effectiveness estimates.

List of included qualitative studies

Ministry of Public Health and Sanitation. 2012. National Communication strategy for community health services. Nairobi, Kenya: Ministry of Public Health and Sanitation, Republic of Kenya. https://www.slideshare.net/chskenya/chs-kenya-national-communicationstrategyforcommunityhealthservices20122017

Adamu, Abdu A., O. A. Uthman, M. A. Gadanya, and C. S. Wiysonge. 2020. "Using the consolidated framework for implementation research (CFIR) to assess the implementation context of a quality improvement program to reduce missed opportunities for vaccination in Kano, Nigeria: a mixed methods study." *Hum Vaccin Immunother* 16 (2):465-475. doi: 10.1080/21645515.2019.1654798.

For peer review only - http://bmjopen.bmj.com/site/about/guidelines.xhtml

Anwari, Z., M. Shukla, B. A. Maseed, G. F. Wardak, S. Sardar, J. Matin, G. S. Rashed, S. A. Hamedi, H. Sahak, A. H. Aziz, M. Boyd-Boffa, and R. Trasi. 2015. "Implementing people-centred health systems governance in 3 provinces and 11 districts of Afghanistan: a case study." *Confl Health* 9:2. doi: 10.1186/1752-1505-9-2.

Baba-Ari, F., E. A. Eboreime, and M. Hossain. 2018. "Conditional Cash Transfers for Maternal Health Interventions: Factors Influencing Uptake in North-Central Nigeria." *Int J Health Policy Manag* 7 (10):934-942. doi: 10.15171/ijhpm.2018.56.

Balarajan, Yarlini, and Michael R. Reich. 2016. "Political economy of child nutrition policy: A qualitative study of India's Integrated Child Development Services (ICDS) scheme." *Food Policy* 62:88-98. doi: https://doi.org/10.1016/j.foodpol.2016.05.001.

Billah, S. M., D. E. Hoque, M. Rahman, A. Christou, N. S. Mugo, K. Begum, T. Tahsina, Q. S. Rahman, E. K. Chowdhury, T. M. Haque, R. Khan, A. Siddik, J. Bryce, R. E. Black, and S. El Arifeen. 2018. "Feasibility of engaging "Village Doctors" in the Community-based Integrated Management of Childhood Illness (C-IMCI): experience from rural Bangladesh." *J Glob Health* 8 (2):020413. doi: 10.7189/jogh.08.020413.

Chimhutu, Victor. 2011. "Pay for Performance in Maternal Health in Tanzania: Perceptions, Expectations and Experiences in Mvomero district."

Chimhutu, Victor, Nils Gunnar Songstad, Marit Tjomsland, Mwifadhi Mrisho, and Karen Marie Moland. 2016. "The inescapable question of fairness in Pay-for-performance bonus distribution: a qualitative study of health workers' experiences in Tanzania." *Globalization and Health* 12 (1):77. doi: 10.1186/s12992-016-0213-5.

Chimhutu, Victor, Marit Tjomsland, and Mwifadhi Mrisho. 2019. "Experiences of care in the context of payment for performance (P4P) in Tanzania." *Globalization and Health* 15 (1):59. doi: 10.1186/s12992-019-0503-9.

Chimhutu, Victor, Marit Tjomsland, Nils Gunnar Songstad, Mwifadhi Mrisho, and Karen Marie Moland. 2015. "Introducing payment for performance in the health sector of Tanzania- the policy process." *Globalization and Health* 11 (1):38. doi: 10.1186/s12992-015-0125-9.

Doctor, H. V., S. E. Findley, A. Ager, G. Cometto, G. Y. Afenyadu, F. Adamu, and C. Green. 2012. "Using community-based research to shape the design and delivery of maternal health services in Northern Nigeria." *Reprod Health Matters* 20 (39):104-12. doi: 10.1016/s0968-8080(12)39615-8.

Grayman, Jesse Hession, Siti Ruhanawati, and Nelti Anggraini. 2014. "Opportunities and approaches for better nutrition outcomes through PNPM generasi : a qualitative study."

Henning, M. J., J. M. Zulu, C. Michelo, S. Simmons Zuilkowski, and C. Hubner. 2020. "Adolescent Mothers' Experiences With Community Health Assistants in Rural Zambia." *Int Q Community Health Educ* 40 (4):353-361. doi: 10.1177/0272684x19896737.

Khuzwayo, L. S., and M. Moshabela. 2017. "The perceived role of ward-based primary healthcare outreach teams in rural KwaZulu-Natal, South Africa." *Afr J Prim Health Care Fam Med* 9 (1):e1-e5. doi: 10.4102/phcfm.v9i1.1388.

Lignou, Sapfo, Sushmita Das, Jigna Mistry, Glyn Alcock, Neena Shah More, David Osrin, and Sarah J. L. Edwards. 2016. "Reconstructing communities in cluster trials?" *Trials* 17 (1):166. doi: 10.1186/s13063-016-1284-6.

Marcus, Tessa S., Jannie Hugo, and Champak C. Jinabhai. 2017. "Which primary care model? A qualitative analysis of ward-based outreach teams in South Africa." *African journal of primary health care & family medicine* 9 (1):e1-e8. doi: 10.4102/phcfm.v9i1.1252.

Moosa, S., A. Derese, and W. Peersman. 2017. "Insights of health district managers on the implementation of primary health care outreach teams in Johannesburg, South Africa: a descriptive study with focus group discussions." *Hum Resour Health* 15 (1):7. doi: 10.1186/s12960-017-0183-6.

Okeke, Edward N., Emma Pitchforth, Josephine Exley, Peter Glick, Isa Sadeeq Abubakar, Amalavoyal V. Chari, Usman Bashir, Kun Gu, and Obinna Onwujekwe. 2017. "Going to scale: design and implementation challenges of a program to increase access to skilled birth attendants in Nigeria." *BMC Health Services Research* 17 (1):356. doi: 10.1186/s12913-017-2284-2.

Oladepo, O., I. O. Dipeolu, and O. Oladunni. 2019. "Nigerian rural mothers' knowledge of routine childhood immunizations and attitudes about use of reminder text messages for promoting timely completion." *J Public Health Policy* 40 (4):459-477. doi: 10.1057/s41271-019-00180-7.

For peer review only - http://bmjopen.bmj.com/site/about/guidelines.xhtml

Oladepo, Oladimeji, Isaac Oluwafemi Dipeolu, and Opeyemi Oladunni. 2020. "Outcome of reminder text messages intervention on completion of routine immunization in rural areas, Nigeria." *Health Promotion International*. doi: 10.1093/heapro/daaa092.

Olafsdottir, Anna Elisabet, Iddy Mayumana, Irene Mashasi, Ikunda Njau, Masuma Mamdani, Edith Patouillard, Peter Binyaruka, Salim Abdulla, and Josephine Borghi. 2014. "Pay for performance: an analysis of the context of implementation in a pilot project in Tanzania." *BMC Health Services Research* 14 (1):392. doi: 10.1186/1472-6963-14-392.

Pérez, Myriam Cielo, Dinesh Chandra, Georges Koné, Rohit Singh, Valery Ridde, Marie-Pierre Sylvestre, Aaditeshwar Seth, and Mira Johri. 2020. "Implementation fidelity and acceptability of an intervention to improve vaccination uptake and child health in rural India: a mixed methods evaluation of a pilot cluster randomized controlled trial." *Implementation Science Communications* 1 (1):88. doi: 10.1186/s43058-020-00077-7.

Phiri, S. C., M. L. Prust, C. P. Chibawe, R. Misapa, J. W. van den Broek, and N. Wilmink. 2017. "An exploration of facilitators and challenges in the scale-up of a national, public sector community health worker cadre in Zambia: a qualitative study." *Hum Resour Health* 15 (1):40. doi: 10.1186/s12960-017-0214-3.

Rahayu;, Sri Kusumastuti, Nina Toyamah;, Stella Aleida Hutagalung;, Meuthia Rosfadhila; &, and Muhammad Syukri. 2008. Qualitative Baseline Study for PNPM Generasi and PKH : The Availability and Use of the Maternal and Child Health Services and Basic Education Services in the Provinces of West Java and East Nusa Tenggara. In *Development Economics Working Papers 22536*: East Asian Bureau of Economic Research.

Rahman, A. 2007. "Challenges and opportunities in developing a psychological intervention for perinatal depression in rural Pakistan--a multi-method study." *Archives of women's mental health* 10 (5):211-219. doi: 10.1007/s00737-007-0193-9.

Rahman, Atiya, Margaret Leppard, Sarawat Rashid, Nauruj Jahan, and Hashima E. Nasreen. 2016. "Community perceptions of behaviour change communication interventions of the maternal neonatal and child health programme in rural Bangladesh: an exploratory study." *BMC Health Services Research* 16 (1):389. doi: 10.1186/s12913-016-1632-y.

Robertson, Laura, Phyllis Mushati, Morten Skovdal, Jeffrey W. Eaton, Jeremiah C. Makoni, Tom Crea, Gideon Mavise, Lovemore Dumba, Christina Schumacher, Lorraine Sherr, Constance Nyamukapa, and Simon Gregson. 2014. "Involving Communities in the Targeting of Cash Transfer Programs for Vulnerable Children: Opportunities and Challenges." *World Development* 54:325-337. doi: https://doi.org/10.1016/j.worlddev.2013.09.002.

Sacks, Audrey and Grayman, Jesse Hession. 2018. Indonesia - Long-Term Generasi Qualitative Study (English)

Shah, P., N. Madhiwala, S. Shah, G. Desai, K. Dave, N. Dholakia, S. Patel, S. Desai, and D. Modi. 2019. "High uptake of an innovative mobile phone application among community health workers in rural India: An implementation study." *Natl Med J India* 32 (5):262-269. doi: 10.4103/0970-258x.295956.

Shelley, K. D., Y. W. Belete, S. C. Phiri, M. Musonda, E. C. Kawesha, E. M. Muleya, C. P. Chibawe, J. W. van den Broek, and K. B. Vosburg. 2016. "Implementation of the Community Health Assistant (CHA) Cadre in Zambia: A Process Evaluation to Guide Future Scale-Up Decisions." *J Community Health* 41 (2):398-408. doi: 10.1007/s10900-015-0110-5.

Skovdal, Morten, Phyllis Mushati, Laura Robertson, Shungu Munyati, Lorraine Sherr, Constance Nyamukapa, and Simon Gregson. 2013. "Social acceptability and perceived impact of a community-led cash transfer programme in Zimbabwe." *BMC Public Health* 13 (1):342. doi: 10.1186/1471-2458-13-342.

Skovdal, Morten, Laura Robertson, Phyllis Mushati, Lovemore Dumba, Lorraine Sherr, Constance Nyamukapa, and Simon Gregson. 2013. "Acceptability of conditions in a community-led cash transfer programme for orphaned and vulnerable children in Zimbabwe." *Health Policy and Planning* 29 (7):809-817. doi: 10.1093/heapol/czt060.

Vita, Febriany, Febriany Vita, Toyamah Nina, Toyamah Nina, Sodo Robert Justin, Sodo Robert Justin, Budiyati Sri, and Budiyati Sri. 2011. Qualitative Impact Study for PNPM Generasi and Pkh on the Provision and the

For peer review only - http://bmjopen.bmj.com/site/about/guidelines.xhtml

Utilization of Maternal and Child Health Services and Basic Education Services in the Provinces of West Java and East Nusa Tenggara. In *SMERU Research Institute*: SMERU Research Institute.

Zulu, J. M., J. Kinsman, C. Michelo, and A. K. Hurtig. 2013. "Developing the national community health assistant strategy in Zambia: a policy analysis." *Health Res Policy Syst* 11:24. doi: 10.1186/1478-4505-11-24.

Zulu, Joseph Mumba, John Kinsman, Charles Michelo, and Anna-Karin Hurtig. 2014. "Hope and despair: community health assistants' experiences of working in a rural district in Zambia." *Human Resources for Health* 12 (1):30. doi: 10.1186/1478-4491-12-30.

List of included other studies

Adamu, A. A., O. A. Uthman, M. A. Gadanya, O. O. Adetokunboh, and C. S. Wiysonge. 2019. "A multilevel analysis of the determinants of missed opportunities for vaccination among children attending primary healthcare facilities in Kano, Nigeria: Findings from the pre-implementation phase of a collaborative quality improvement programme." *PLoS One* 14 (7):e0218572. doi: 10.1371/journal.pone.0218572.

Adamu, Abdu Abdullahi. 2019. "Using quality improvement approach to address missed opportunities for vaccination in Kano Metropolis, Nigeria." PhD, Faculty of Medicine and Health Sciences, Stellenbosch University.

Alhassan, Robert Kaba. 2017. "Healthcare quality in Ghana: Improving healthcare quality and health worker motivation to promote sustainable health insurance." PhD, Faculty of Medicine (AMC-UvA), University of Amsterdam.

Banwat, ME., PW; Bupwatd, LA; Lar, AA; Apagu, and AI. Zoakah. 2014. "Effect of peer education on timeliness and completeness of routine immunization: An assessment in rural communities in North-Central Nigeria." *International Journal of Community Research* 3 (3):60-67.

Bekele, Abebe, Mengistu Kefale, and Mekonnen Tadesse. 2016. "Preliminary Assessment of the Implementation of the Health Services Extension Program: The case of Southern Ethiopia." *The Ethiopian Journal of Health Development* 22 (3).

Binyaruka, P., and J. Borghi. 2017. "Improving quality of care through payment for performance: examining effects on the availability and stock-out of essential medical commodities in Tanzania." *Trop Med Int Health* 22 (1):92-102. doi: 10.1111/tmi.12809.

Binyaruka, P., E. Patouillard, T. Powell-Jackson, G. Greco, O. Maestad, and J. Borghi. 2015. "Effect of Paying for Performance on Utilisation, Quality, and User Costs of Health Services in Tanzania: A Controlled Before and After Study." *PLoS One* 10 (8):e0135013. doi: 10.1371/journal.pone.0135013.

Binyaruka, P., B. Robberstad, G. Torsvik, and J. Borghi. 2018a. "Does payment for performance increase performance inequalities across health providers? A case study of Tanzania." *Health Policy Plan* 33 (9):1026-1036. doi: 10.1093/heapol/czy084.

Binyaruka, Peter, and Laura Anselmi. 2020. "Understanding efficiency and the effect of pay-for-performance across health facilities in Tanzania." *BMJ Global Health* 5 (5):e002326. doi: 10.1136/bmjgh-2020-002326.

Binyaruka, Peter, Bjarne Robberstad, Gaute Torsvik, and Josephine Borghi. 2018b. "Who benefits from increased service utilisation? Examining the distributional effects of payment for performance in Tanzania." *International Journal for Equity in Health* 17 (1):14. doi: 10.1186/s12939-018-0728-x.

Borghi, J., R. Little, P. Binyaruka, E. Patouillard, and A. Kuwawenaruwa. 2015. "In Tanzania, the many costs of pay-for-performance leave open to debate whether the strategy is cost-effective." *Health Aff (Millwood)* 34 (3):406-14. doi: 10.1377/hlthaff.2014.0608.

Borkum, Evan;, Anitha; Sivasankaran, Divya; Vohra, Swetha; Sridharan, Dana; Rotz, Lakshmi; Ramnakrishnan, Mercy; Manoranjini, Sukhmani; Sethi, and Anu. Rangarajan. 2015. "Evaluation of the Team-Based Goals and Performance-Based Incentives (TBGI) Intervention in Bihar: Findings from the 30-month Follow-up." *Mathematica Policy Research*.

Carmichael, Suzan L, Kala Mehta, Hina Raheel, Sridhar Srikantiah, Indrajit Chaudhuri, Shamik Trehan, Sunil Mohanty, Evan Borkum, Tanmay Mahapatra, Yingjie Weng, Rajani Kaimal, Anita Sivasankaran, Swetha Sridharan, Dana Rotz, Usha Kiran Tarigopula, Debarshi Bhattacharya, Yamini Atmavilas, Wolfgang Munar, Anu Rangarajan, and Gary L Darmstadt. 2019. "Effects of team-based goals and non-monetary incentives on front-

For peer review only - http://bmjopen.bmj.com/site/about/guidelines.xhtml

line health worker performance and maternal health behaviours: a cluster randomised controlled trial in Bihar, India." *BMJ Global Health* 4 (4):e001146. doi: 10.1136/bmjgh-2018-001146.

Cockcroft, A., N. Andersson, K. Omer, N. M. Ansari, A. Khan, U. U. Chaudhry, and U. Ansari. 2009. "One size does not fit all: local determinants of measles vaccination in four districts of Pakistan." *BMC Int Health Hum Rights* 9 Suppl 1 (Suppl 1):S4. doi: 10.1186/1472-698x-9-s1-s4.

Costa-i-Font, Joan and Parmar, Divya. 2017. Political Agency and Public Health Care: Evidence from India. In *CESifo Working Paper Series*. India: United Nationa University- UNU-WIDER.

Crea, Thomas M., Andrew D. Reynolds, Aakanksha Sinha, Jeffrey W. Eaton, Laura A. Robertson, Phyllis Mushati, Lovemore Dumba, Gideon Mavise, J. C. Makoni, Christina M. Schumacher, Constance A. Nyamukapa, and Simon Gregson. 2015. "Effects of cash transfers on Children's health and social protection in Sub-Saharan Africa: differences in outcomes based on orphan status and household assets." *BMC Public Health* 15 (1):511. doi: 10.1186/s12889-015-1857-4.

Doctor, H. V., and T. Dahiru. 2010. "Utilization of non-skilled birth attendants in northern Nigeria: a rough terrain to the health-related MDGs." *Afr J Reprod Health* 14 (2):37-45.

Doctor, H. V., S. E. Findley, G. Cometto, and G. Y. Afenyadu. 2013. "Awareness of critical danger signs of pregnancy and delivery, preparations for delivery, and utilization of skilled birth attendants in Nigeria." *J Health Care Poor Underserved* 24 (1):152-70. doi: 10.1353/hpu.2013.0032.

Doctor;, Henry V., Sally E. Findley;, Radheshyam Bairagi; and, and Tukur Dahiru. 2011. "Northern Nigeria Maternal, Newborn and Child Health Programme: Selected Analyses from Population-Based Baseline Survey." *The Open Demography Journal* 4:11-21.

Domek, G. J., I. L. Contreras-Roldan, E. J. Asturias, M. Bronsert, G. A. Bolaños Ventura, S. T. O'Leary, A. Kempe, and S. Bull. 2018. "Characteristics of mobile phone access and usage in rural and urban Guatemala: assessing feasibility of text message reminders to increase childhood immunizations." *Mhealth* 4:9. doi: 10.21037/mhealth.2018.03.05.

Domek, G. J., I. L. Contreras-Roldan, S. T. O'Leary, S. Bull, A. Furniss, A. Kempe, and E. J. Asturias. 2016. "SMS text message reminders to improve infant vaccination coverage in Guatemala: A pilot randomized controlled trial." *Vaccine* 34 (21):2437-2443. doi: 10.1016/j.vaccine.2016.03.065.

Domek, G. J., S. T. O'Leary, S. Bull, M. Bronsert, I. L. Contreras-Roldan, G. A. Bolaños Ventura, A. Kempe, and E. J. Asturias. 2018. "Measuring vaccine hesitancy: Field testing the WHO SAGE Working Group on Vaccine Hesitancy survey tool in Guatemala." *Vaccine* 36 (35):5273-5281. doi: 10.1016/j.vaccine.2018.07.046.

Findley, S. E., O. T. Uwemedimo, H. V. Doctor, C. Green, F. Adamu, and G. Y. Afenyadu. 2013. "Comparison of high- versus low-intensity community health worker intervention to promote newborn and child health in Northern Nigeria." *Int J Womens Health* 5:717-28. doi: 10.2147/ijwh.s49785.

Gibson, D. G., B. Ochieng, E. W. Kagucia, D. Obor, F. Odhiambo, K. L. O'Brien, and D. R. Feikin. 2015. "Individual level determinants for not receiving immunization, receiving immunization with delay, and being severely underimmunized among rural western Kenyan children." *Vaccine* 33 (48):6778-85. doi: 10.1016/j.vaccine.2015.10.021.

Grant, Carolyn, Dipty Nawal, Sai Mala Guntur, Manish Kumar, Indrajit Chaudhuri, Christine Galavotti, Tanmay Mahapatra, Kunal Ranjan, Gangesh Kumar, Sunil Mohanty, Mohammed Aftab Alam, Aritra Das, and Safia Jiwani. 2018. "'We pledge to improve the health of our entire community': Improving health worker motivation and performance in Bihar, India through teamwork, recognition, and non-financial incentives." *PLOS ONE* 13 (8):e0203265. doi: 10.1371/journal.pone.0203265.

Mirkuzie Woldie, Sudhakar Narayan Morankar, Garumma T. Feyissa, and Ronald Labonte, and David Sanders. 2015. "Coverage of child health services in rural districts of Ethiopia with the health services extension program." *Journal of Public Health and Epidemiology* 7 (7):223-231. doi: https://doi.org/10.5897/JPHE2015.0733.

Mitchell, Steven, Neil Andersson, Noor Mohammad Ansari, Khalid Omer, José Legorreta Soberanis, and Anne Cockcroft. 2009. "Equity and vaccine uptake: a cross-sectional study of measles vaccination in Lasbela District,

For peer review only - http://bmjopen.bmj.com/site/about/guidelines.xhtml

Pakistan." *BMC international health and human rights* 9 Suppl 1 (Suppl 1):S7-S7. doi: 10.1186/1472-698X-9-S1-S7.

Modi, D., R. Gopalan, S. Shah, S. Venkatraman, G. Desai, S. Desai, and P. Shah. 2015. "Development and formative evaluation of an innovative mHealth intervention for improving coverage of community-based maternal, newborn and child health services in rural areas of India." *Glob Health Action* 8:26769. doi: 10.3402/gha.v8.26769.

Modi, D., J. Patel, S. Desai, and P. Shah. 2016. "Accessing completeness of pregnancy, delivery, and death registration by Accredited Social Health Activists [ASHA] in an innovative mHealth project in the tribal areas of Gujarat: A cross-sectional study." *J Postgrad Med* 62 (3):170-2. doi: 10.4103/0022-3859.183168.

Modi, Dhiren, Somen Saha, Prakash Vaghela, Kapilkumar Dave, Ankit Anand, Shrey Desai, and Pankaj Shah. 2020. "Costing and Cost-Effectiveness of a Mobile Health Intervention (ImTeCHO) in Improving Infant Mortality in Tribal Areas of Gujarat, India: Cluster Randomized Controlled Trial." *JMIR Mhealth Uhealth* 8 (10):e17066. doi: 10.2196/17066.

Moore., Charity. 2008. Accessing Honduras' CCT Programme PRAF, Programa de Asignacion familiar: Expected and unexpected realities. Brazil: International Poverty Centre.

Nagar, Ruchit B. 2016. "Tying Community Engagement With Appropriate Technology At The Last Mile: A Cluster Randomized Trial To Determine The Effectiveness Of A Novel, Digital Pendant And Voice Reminder Platform On Increasing Infant Immunization Adherence Among Mothers In Rural Udaipur, India." School of Public Health, Yale University.

Negusse, H., E. McAuliffe, and M. MacLachlan. 2007. "Initial community perspectives on the Health Service Extension Programme in Welkait, Ethiopia." *Hum Resour Health* 5:21. doi: 10.1186/1478-4491-5-21.

Oduenyi, Chioma, Victor Ordu, and Ugo Okoli. 2019. "Assessing the operational effectiveness of a maternal and child health (MCH) conditional cash transfer pilot programme in Nigeria." *BMC Pregnancy and Childbirth* 19 (1):298. doi: 10.1186/s12884-019-2418-0.

Okeke, Edward, Peter Glick, Amalavoyal Chari, Isa Sadeeq Abubakar, Emma Pitchforth, Josephine Exley, Usman Bashir, Kun Gu, and Obinna Onwujekwe. 2016. "The effect of increasing the supply of skilled health providers on pregnancy and birth outcomes: evidence from the midwives service scheme in Nigeria." *BMC Health Services Research* 16 (1):425. doi: 10.1186/s12913-016-1688-8.

Onwujekwe, Obinna, Tim Ensor, Pamela Ogbozor, Chinyere Okeke, Uche Ezenwaka, Joseph P. Hicks, Enyi Etiaba, Benjamin Uzochukwu, Bassey Ebenso, and Tolib Mirzoev. 2020. "Was the Maternal Health Cash Transfer Programme in Nigeria Sustainable and Cost-Effective?" *Frontiers in public health* 8:582072-582072. doi: 10.3389/fpubh.2020.582072.

Padayachee, Thesandree;, Natasha; Chetty, Muzi; Matse, Tumelo; Mampe, and Helen Schneider. 2013. Progress in the establishment of ward based outreach teams: experiences in the North West Province. University of Western Cape.

Quayyum, Z., M. N. Khan, T. Quayyum, H. E. Nasreen, M. Chowdhury, and T. Ensor. 2013. ""Can community level interventions have an impact on equity and utilization of maternal health care" - evidence from rural Bangladesh." *Int J Equity Health* 12:22. doi: 10.1186/1475-9276-12-22.

Rahman, Mahfuzar, Fatema Tuz Jhohura, Sabuj Kanti Mistry, Tridib Roy Chowdhury, Tanveen Ishaque, Rasheduzzaman Shah, and Kaosar Afsana. 2015. "Assessing Community Based Improved Maternal Neonatal Child Survival (IMNCS) Program in Rural Bangladesh." *PLOS ONE* 10 (9):e0136898. doi: 10.1371/journal.pone.0136898.

Rangarajan;, Anu, Evan Borkum;, Swetha Sridharan;, Dana Rotz;, Mercy Manoranjini;, Seth Morgan;, Lalit Dandona;, Rakhi Dandona;, Priyanka Chaman;, and G. Anil Kumar. 2013. "Baseline Findings from the Ananya Evaluation." *Mathematica Policy Research Reports* 3e3c44fb11b84a07a3063c242.

Robertson, Laura, Phyllis Mushati, Jeffrey W. Eaton, Lorraine Sherr, Jeremiah C. Makoni, Morten Skovdal, Tom Crea, Gideon Mavise, Lovemore Dumba, Christina Schumacher, Shungu Munyati, Constance Nyamukapa, and Simon Gregson. 2012. "Household-based cash transfer targeting strategies in Zimbabwe: are we reaching the

For peer review only - http://bmjopen.bmj.com/site/about/guidelines.xhtml

most vulnerable children?" *Social science & medicine (1982)* 75 (12):2503-2508. doi: 10.1016/j.socscimed.2012.09.031.

Robinson, Victor C. 2006. Summary Report Women's TheRuralMaintenance Program: ACashforWorkProgram.

USAID. 2008. Essential Services for Health in Ethiopia: Final report November 2003- September 2008. Ethiopia: USAID.

UNICEF. 2010. Evaluation report of the community health strategy implementation in Kenya. Division of Community Health Services

Venkat, Preethi. 2016. "Intermediate Assessment Of The Khushi Baby Crct: Implementation Of A Novel Mhealth Solution For Vaccination Record Keeping In Rural Udaipur, Rajasthan, India."

Wakadha, H., S. Chandir, E. V. Were, A. Rubin, D. Obor, O. S. Levine, D. G. Gibson, F. Odhiambo, K. F. Laserson, and D. R. Feikin. 2013. "The feasibility of using mobile-phone based SMS reminders and conditional cash transfers to improve timely immunization in rural Kenya." *Vaccine* 31 (6):987-93. doi: 10.1016/j.vaccine.2012.11.093.

For peer review only

Appendix 6: Data extraction tools

Quantitative data extraction tool

1. VARIABLE LABEL	EXPLANATION
Study ID	This is the study ID - it should match the study ID from the Outcome Mapping Sheet (e.g., SC-SR_1)
Estimate ID	The estimate ID will provide a specific number for each effect size extracted and should include the original study number, underscore, then the unique ID number (e.g., SC-SR1_1, SC-SR1_2 and so on)
Author	For 1 author: leading author last name (e.g., Gomez) For 2 authors: both author last names with ampersand in between (e.g., Smith & Bahn) For 3 or more authors: leading author last name followed by et al. (e.g., Gupta et al.)
Year	Year published
Design	0=Experimental Design (e.g., RCT), 1=Quasi-Experimental Design
How Counterfactual is Chosen?	Free text (e.g., random control trial, propensity score matching, etc) - Multiple codes are ok
Analysis type for this effect size	Free text, what type of analysis was used (Regression, 2SLS, ANCOVA, etc.)- Multiple codes are ok
Country	Country of intervention
Region	Region/continent of intervention
Estimate Type	Type of data for this effect size: 1 = Continuous - means and SDs, 2 = Continuous - mean difference and SD, 3 = Dichotomous outcome - proportions, 4 = Regression data - dichotomous outcome (e.g., logistic regression) 5 = Regression data - continuous outcome (e.g., linear regression)
Comparison	1=No intervention (service delivery as usual), 2=Other intervention, 3=Pipeline (wait-list) control (still service delivery as usual)
Describe Comparison Group	If answer above is (1) no intervention, type N/A, if (2) Other Intervention, list what intervention the control group is receiving, if (3) Pipeline control, report when the control group will receive the intervention in relation to the treatment group (e.g., one year later)
Subgroup	Is this analysis of a subgroup? 0=no, 1=yes

For peer review only

For peer review only - http://bmjopen.bmj.com/site/about/guidelines.xhtml

If yes to subgroup, describe	Free text, describe the subgoup if applicable (e.g., boys, girls). If no subgroup, type N/A
Source	Note the page number, table number, column, and row you used to extract the data
Treatment Effect	1=Intention to Treat (ITT), 2=Average Treatment Effect on the Treated (ATET), 3=Average Treatment Effect (ATE) 4 = Local Average Treatment Effect (LATE)
Intervention	Free text, what is the intervention
Engagement as intervention	1=yes, 0=No
Engagement as intervention: developing community buy-in	1=yes, 0=No
Engagement as intervention: creation of new cadres or health committees	1=yes, 0=No
Engagement as intervention: both	1=yes, 0=No
Engagement in design	1=yes, 0=No
Engagement in design: community decision makinh	1=yes, 0=No
Engagement in design: community feedback	1=yes, 0=No
Engagement in implementation	1=yes, 0=No
Engagement in implementation: governance and decisions	1=yes, 0=No
Engagement in implementation: provision of resources	1=yes, 0=No
Multiple engagement types	1=yes, 0=No
Engagement in design and engagement as intervention	1=yes, 0=No
Engagement in design and implementation	1=yes, 0=No
Engagement in implementation and engagement as intervention	1=yes, 0=No
Exposure to intervention (in months)	How long is the intervention exposure itself?
Evaluation period (in months)	The total number of months elapsed between offering an intervention and the point at which an outcome measure is taken post intervention, or as a follow-up measurement. If less than one month, use decimals (e.g., one week would be .25)

For peer review only - http://bmjopen.bmj.com/site/about/guidelines.xhtml

Post-intervention or change from baseline?	0 = Post-intervention, 1 = Change from baseline
Source of the Outcome data (only use for outcomes in category JAA)	1=Immuniation Card, 2=Recall, 3=Combination of both immunisation card and recall, 4=Health Admin Data, 5=N/A
Author definition of outcome	Free text - How does the author define the outcome?
**OUTCOME CODES**	
**Thinking and Feeling**	Code 1 under any applicable columns. See OUTCOME GUIDANCE document for further explanations.
**Social Processes**	Code 1 under any applicable columns. See OUTCOME GUIDANCE document for further explanations.
**Readiness to Vaccinate**	Code 1 under any applicable columns. See OUTCOME GUIDANCE document for further explanations.
**Practical Factors**	Code 1 under any applicable columns. See OUTCOME GUIDANCE document for further explanations.
**Community Health Workers**	Code 1 under any applicable columns. See OUTCOME GUIDANCE document for further explanations.
**Vaccinators**	Code 1 under any applicable columns. See OUTCOME GUIDANCE document for further explanations.
**IAC Admins**	Code 1 under any applicable columns. See OUTCOME GUIDANCE document for further explanations.
**Health information systems**	Code 1 under any applicable columns. See OUTCOME GUIDANCE document for further explanations.
**Vaccine Availability**	Code 1 under any applicable columns. See OUTCOME GUIDANCE document for further explanations.
**Resources**	Code 1 under any applicable columns. See OUTCOME GUIDANCE document for further explanations.
**Vaccination coverage**	Code 1 under any applicable columns. See OUTCOME GUIDANCE document for further explanations.
**Health Outcomes**	Code 1 under any applicable columns. See OUTCOME GUIDANCE document for further explanations.
**EFFECT SIZE DATA EXTRACTION**	
Reverse Sign (i.e., decrease is good)	Record 0='no' if an increase is good, record 1='yes' if a decrease is good and the sign needs to be reversed.
Unit of analysis	What is the unit of analysis? UOA for this effect size: 1= Individual, 2= Household, 3= Group (e.g. community

For peer review only - http://bmjopen.bmj.com/site/about/guidelines.xhtml

	organisation), 4= Health Center, 5 = Village, 6 = Other, 7 = Not clear
mean_t	Outcome mean for the treatment group
sd_t	Outcome standard deviation for treatment group
mean_c	Outcome mean for the comparison group
sd_c	Outcome standard deviation for control group
mean_overall_diff	Overall mean difference (treatment - control)
diff se	Standard error of the overall mean difference
Diff _t	t-statistic of mean difference
Odds ratio	Odds ratio reported in the study
OR_se	Odds ratio standard error reported in the study
Risk ratio	Risk ratio reported in study
RR_se	Risk ratio standard error
reg_coeff	Report the regression coefficient of the treatment effect
reg_SE	Report the associated standard error of the regression coefficient.
reg_t	Report the associated t statistic of the effect size (coefficient/SE)
Exact p value	Exact p value if given, if not, record as written in the manuscript (e.g., p < .001, or p > .05)
clust_t	Number of clusters - treatment group
clust_c	Number of clusters - control group
clust_T	Number of clusters - total sample
n_t	Sample size - treatment group
n_c	Sample size - control group
n_T	Sample size - total sample
Does the sample size need to be adjusted (if so complete column CL)?	Code as 'yes' or no'
periods (1 if cross sectional)	Record how many periods of evaluation there are (e.g., cross section is 1, panel data with 3 measurements is 3)
Treatment Variable	Record the treatment variable as written in the model (e.g., the variable name the author uses, such as ("Intervention x Time")
dataset	Record if data comes from an identified dataset

For peer review only - http://bmjopen.bmj.com/site/about/guidelines.xhtml

coder	Record your name
Notes	Record any notes important for the team
n_T_revised	THIS IS FOR SENIOR QUANT LEAD TO FILL OUT
sp	THIS IS FOR SENIOR QUANT LEAD TO FILL OUT
d	THIS IS FOR SENIOR QUANT LEAD TO FILL OUT
g	THIS IS FOR SENIOR QUANT LEAD TO FILL OUT
var(d)	THIS IS FOR SENIOR QUANT LEAD TO FILL OUT
se(d)	THIS IS FOR SENIOR QUANT LEAD TO FILL OUT
CI_l	THIS IS FOR SENIOR QUANT LEAD TO FILL OUT
CI_u	THIS IS FOR SENIOR QUANT LEAD TO FILL OUT
remove	THIS IS FOR PROJECT MANAGER TO FILL OUT
Formula Used	THIS IS FOR SENIOR QUANT LEAD TO FILL OUT
yi_1	THIS IS FOR SENIOR QUANT LEAD TO FILL OUT
yi_rev	THIS IS FOR SENIOR QUANT LEAD TO FILL OUT
yi	THIS IS FOR SENIOR QUANT LEAD TO FILL OUT
vi	THIS IS FOR SENIOR QUANT LEAD TO FILL OUT
wi	THIS IS FOR SENIOR QUANT LEAD TO FILL OUT
ywi	THIS IS FOR SENIOR QUANT LEAD TO FILL OUT
95ci_lower	THIS IS FOR SENIOR QUANT LEAD TO FILL OUT
95ci_upper	THIS IS FOR SENIOR QUANT LEAD TO FILL OUT
cilow_3sf	THIS IS FOR SENIOR QUANT LEAD TO FILL OUT
cihigh_3sf	THIS IS FOR SENIOR QUANT LEAD TO FILL OUT
ci	THIS IS FOR SENIOR QUANT LEAD TO FILL OUT
wb_yi	THIS IS FOR SENIOR QUANT LEAD TO FILL OUT
Checked	THIS IS FOR EFFECT SIZE RELIABILITY CHECKER TO FILL OUT
ROB Category	THIS IS FOR SENIOR QUANT LEAD OR PM TO FILL OUT

For peer review only

Qualitative data extraction tool

Name	Description
Causal mechanism	This set of codes describes the causal mechanisms observed or hypothesized
Barriers to immunisation	Select any state reasons that people were not immunised. Try to use previously added codes to reduce possible redundancies, but add codes if needed.
Demand side barriers	Barriers related to low demand for immunisations
Fear	Low demand for immunisation due to fear of side effects or other factors
Financial	Select the financial reason that people were not immunised Add sub-codes as needed.
Cost of immunisation	People were not immunised due to the cost of the immunisation Do not add sub-codes
Cost of transport	People were not immunised due to the cost of transport.
Opportunity costs	People were not immunised due to opportunity costs
Knowledge of Immunisation Schedule	Low demand for immunisation due to limited knowledge of its importance. This includes instances when the person simply does not feel that immunisations are important (regardless of "book knowledge")
Logistics or distance	Barriers related to logistics or distance. For example, it is too far away, I need to arrange for XXX to happen while I am gone...
Self-efficacy	Issues related to not feeling empowered to get vaccinated. This includes not knowing how to go about getting vaccinations. For example, not knowing when or where to get vaccinated
Social norms	Low demand for immunisation due to social pressures and social norms
Understanding of importance	Low demand for immunisation due to limited knowledge of its importance. This includes instances when the person simply does not feel that immunisations are important (regardless of "book knowledge")
Supply side barriers	Limitations to supply that cause problems for immunisation
Availability of services	Services may not be physically present. This includes clinics being closed.
Human resources	Limited human resources may reduce the availability of immunisations. Ex: Appropriate staff were not present to provide the injection
Lack of Infrastructure or supplies	Use code if the lack of immunisation was because there were no drugs, vaccines, cold chain or other items related to physical infrastructure of the health facility

For peer review only - http://bmjopen.bmj.com/site/about/guidelines.xhtml

Poor quality services	Immunisations are not common because there is low quality of service. Providers may not be cooperative, slow, unprofessional/rude, unempathetic, abusive, or inconsistent.
Lack of accountability	Use code for text referring to issues related to accountability, corruption, governance and transparency in providing services.
Lack of Motivation	Use if for text indicating that the health workers are not motivated to perform their duties due to any reason like lack of monetary incentives, recognition, low job satisfaction and other issues.
Baseline conditions and descriptive characteristics	Discussions of the baseline conditions and descriptive characteristics of the region or population in which the intervention took place. This does not necessarily come from a baseline survey, but can be through other sources. Do not add sub-codes
Health systems context	Discussion of the health system functioning and context Example: The clinic served a population of 1,000 people Do not add sub-codes
Immunisation rates	Discussion of baseline immunisation rates. Example: Immunisation rates were low at baseline. Do not add sub-codes
Political context	Discussion of the political context Example: Political unrest made the intervention difficult to implement. Do not add sub-codes
Socioeconomic status and demographics	Discussions of SES and demographics at baseline. Example: The community was mostly farmers. Population size Do not add sub-codes
Facilitators of immunisation	Select any state reasons that people were immunised. Try to use previously added codes to reduce possible redundancies, but add codes if needed.
Demand Side Facilitators	
Favourable population SES characteristics	For instance those from a higher wealth quantile, urban areas or mothers with higher educational qualifications are more likely to get their children immunised. Do not add sub-codes.
Knowledge of immunisation schedule	Use this code if caregivers are aware of all immunisations in the schedule and know which vaccination is due when. Do not add sub-codes.
Lack of fear	Text indicating that there was no fear or vaccine hesitancy. Do not add sub-codes
Lack of financial constraints	Caregivers can afford the cost of immunisation, cost of transportation to visit healthcare facilities, taking children for immunisation does not come at a high opportunity cost for the caregiver. Do not add sub-codes.

For peer review only - http://bmjopen.bmj.com/site/about/guidelines.xhtml

	Lack of logistics or distance related challenges	For instance, caregivers were able to access health services because they lived within a 5km radius of a healthcare facility or they received outreach services regularly. Do not add sub-codes.
	Self-efficacy	Caregivers feel empowered to get their children vaccinated. For example, they know when and where to get vaccinated. Do not add sub-codes.
	Social norms	The social environment owing to prevailing social pressures and norms is encouraging towards child immunisation. For instance, the mother in law in the household or the traditional/religious leaders in the community support immunisation of children. Do not add sub-codes.
	Understanding of importance	Caregivers are aware about the importance of immunisation and are willing to get their children vaccinated. For example, caregivers are able to correctly identify vaccine preventable illness and therefore, more likely to get their children immunised. Do not add sub-codes.
	Supply-side facilitators	
	Availability of infrastructure or supplies	
	Good quality of services	
	High accountability	
	High motivation	
	Human resources	
	Availability of services	
Impacts		What impacts were ultimately achieved? Use this code for general impacts evaluated through the IE that do not fall into one of the other groups. Do not add sub-codes
	Descriptive impacts	Include authors' descriptions of impacts that were not directly measured through the IE. Ex: There was generally an increase over time in most sites
	Equity considerations	If impacts on marginalized groups are considered, select the type of marginalized group considered. Do not add sub-codes
	Ethnic minority	Impacts on ethnic minorities Do not add sub-codes
	Other	Impacts on other marginalized groups, like scheduled castes in India. Do not add sub-codes
	Religious minority	Impacts on religious minorities Do not add sub-codes
	Women	Impacts on women Do not add sub-codes

For peer review only - http://bmjopen.bmj.com/site/about/guidelines.xhtml

Long term impacts	Impact measured over a timeframe of more than 5 years from the end of the intervention Do not add additional sub-codes
Unintended impacts	Only use this sub-code if the impact is stated as unintended. Do not add sub-codes
Reasons for project success or failure	Select text in which authors discuss the reasons for the success or failure of the project. This could be related to intervention design, implementation, or the research approach. Example: The intervention was not successful due to a lack of political will. Do not add sub-codes
Failure	
Implementation or scale-up challenges	
Competing priorities of health workers	
Other delays, disruptions or implementation variability	
Payment delays	
Intervention	
Design - Engagement	
Design - other	
Duration, frequency and exposure or reach	
Not accounting for existing constraints or uncontrollable contextual trends	
Caregiver competing priorities	
Civil unrest or political instability or natural calamity	
Fear of AEFI	
Health system issues	
Health service access or availability	
Poor quality of service or infrastructure	Also includes issues related to health worker demotivation, recruitment or retention.

For peer review only - http://bmjopen.bmj.com/site/about/guidelines.xhtml

High baseline coverage	The interventions are not geared to/designed to achieve last mile coverage or breakthrough stagnating rates.
Lack of awareness or understanding	
Migration	
Resource constraints or scarcity	Infrastructure, electricity, cellular network, etc.
Social norms	
Wider socio-economic or health-related progress	
Study design	
Contamination	
Other methodological shortcomings	
Mixed results	
Success	
Existing or changing favourable factors or contextual trends	
Health system enablers	
Good quality services and infrastructure	Includes enablers like good outreach, service monitoring, etc.
Health service access and availability	
Self efficacy, social norms and awareness	
Socio-economic factors	
Implementation improvements	
Intervention features	
Community engagement	
Behaviour change communication	
Community dialogues	

For peer review only - http://bmjopen.bmj.com/site/about/guidelines.xhtml

Community involvement in planning and implementation	
Incentives	
Needs assessments, pilots or stakeholder consultations	
Other types of engagement	
Customisation to local context	
Health system integration and organisational structure	
Health worker training	
Intervention duration, dose and exposure	
Leadership and supportive supervision	
Other design features	
Positive participant or beneficiary views of intervention	
Theory of change	Information related to the ToC. Do not code here, but use sub-codes. Do not add sub-codes.
Assumptions	when discussing a causal chain mechanism, we often make assumptions about what will happen. These assumptions are often implicit. We may print materials with the assumption that people will be able to read them. We may train people with the assumption they speak our language. When these assumptions are stated, use the sub-codes to indicate if these assumptions were validated or not. Do not add sub-codes
Incorrect assumption	This indicates that there was an assumption in the causal chain mechanism and the assumption was not correct. For example, a study assumed that increasing supply would increase immunisations. Here, we are assuming that people want immunisations and that low supply is the problem. If instead, we find that people did not want the immunisations to begin with, the assumption was incorrect and would be indicated here. Do not add sub-codes

For peer review only - http://bmjopen.bmj.com/site/about/guidelines.xhtml

Valid assumption	this indicates that there was an assumption in the causal chain mechanism and the assumption was valid. For example, a study assumed that increasing supply would increase immunisations. Here, we are assuming that people want immunisations and that low supply is the problem. If this proves to be true, the text should be selected here. Do not add sub-codes
Causal chain mechanisms	Descriptions of how change is expected to occur. These causal chains are often represented as flow charts, or at least they can be. Text that reflects a causal chain mechanism will discuss expected actions and reactions. "We did this and we expected that." Do not add sub-codes
Conclusions	Authors conclusions. Any summary of the take-home message of the article. Tends to be "this worked," "this did not work," "in conclusion." Do not add sub-codes
Cost	Discussions of costs, including the costs of the intervention and any form of cost-analysis. Only select key "take-home message." Do not add sub-codes
Data source	For anything related to causal mechanisms, uptake, and uptake and fidelity challenges, indicate what data source provided this information. Do not add additional sub-codes
Author notes or experiences	
FGDs	
Implementer interpretations or experiences	
Individual interviews	
Literature Review	Use this code if the text alludes to existing evidence on the subject
Observations	If the data comes from direct observations. This must be enumerators looking at and noting the state of something. If it is simply "general impressions," use the notes/interpretations or experience codes. Do not add sub-codes
Intervention description	This set of codes describes the intervention
Beneficiary selection	Select relevant information regarding how beneficiaries were selected for the intervention. Note: This is how beneficiaries of the intervention were selected, NOT a research sampling design or how research participants were selected. Do not add sub-codes
Community involvement	Description of how the community was involved in beneficiary selection. Example: A community council decided who was eligible. Do not add sub-codes
Equity considerations	Descriptions of efforts to ensure beneficiary selection was fair and equitable. Example: There was a quota to ensure adequate representation for ethnic minorities Do not add sub-groups

For peer review only - http://bmjopen.bmj.com/site/about/guidelines.xhtml

Other selection	Other descriptions of the selection process and eligibility criteria Do not add sub-codes
Researcher involvement	Description of the researcher involvement in selection, including selection criteria designed for academic analysis Example: If only those who were involved in and RCT received the intervention, then the sampling frame for the RCT would also be how beneficiaries for the intervention were selected. Do not add sub-codes
Community engagement	This set of codes describes community engagement in the interventions
Community mobilization	Select the relevant reference to community mobilization, regardless of how this phrase is used Do not add sub-codes
Absent	Statements that there was no effort at community mobilisation Example: A challenge was that community members were not aware of the intervention activities. Do not add sub-codes
Definitions of community mobilization	The author's definition for community mobilisation Do not add sub-codes
Present - with trust building	Descriptions of community engagement that was done in such a way as to establish trust and buy-in. Example: A local organisation was recruited to inform community members of the intervention and explain its importance. Note: expect significant double coding with "Developing community buy-in, Present" Do not add sub-codes
Present - without trust building	Descriptions of community mobilization that was done without establishing trust and buy-in. Example: Simply informing the community of an intervention Do not add sub-codes
Inclusion criteria	Select the relevant reason this intervention / activity was included in the SR. Inclusion criteria into the research project should fall under "sampling frame." Do not use main code, only use sub-codes Do not add sub-codes
Community engagement as the intervention	Select the way in which community engagement was used as the intervention itself Do not add sub-codes
Developing community buy-in	Descriptions of activities whose primary purpose was the establishment of community buy-in / trust Do not add sub codes
Absent	Explicit statements that efforts to establish community buy-in / trust were not made should be assigned this code Example: Authors state that the intervention proceeded without engaging the community Do not add sub-codes
Present	If there were activities whose primary purpose was to establish community buy in or trust, select this code Example: Meetings with local leaders to get their support Note: expect significant double coding with "community mobilization, present - with trust building" Do not add sub-codes
New cadres	Description of the development of new cadres of community based structures or systems for health outreach Examples: Developing community health workers, health volunteers, or the establishment of committees Do not add sub-codes

For peer review only

For peer review only - http://bmjopen.bmj.com/site/about/guidelines.xhtml

Community engagement in designing the intervention	Select the type of community engagement that was sought before on the design of the intervention Do not add sub-codes
Absent	The community was not given the opportunity to provide feedback or make decisions on the design of the intervention Example: Explicit statements that the community was not consulted on the design of the intervention Do not add sub-codes
Community decision making	The community was given the opportunity to make decisions regarding the design of an intervention. This could vary from taking iterative feedback from the community to consensus building on the design of the intervention to the design being community led
Absent	The community did not have decision making power. Example: The intervention design was established a priori and not subject to change Do not add sub-codes
Community led	The community had the ultimate decision-making power and / or the intervention design was community led. Example: The community identified which barriers would be targeted and developed an action plan Do not add sub-codes
Partial	The community had some decision-making power Example: Decisions were made through collaboration between the community, implementers, and / or researchers Do not add sub-codes
Community feedback	The community was given the opportunity to provide feedback on the design of the intervention
Formative evaluation or stakeholder consultation	
Needs assessment	
Pilot	
There was a previous pilot	There was a pilot in which it is explicitly stated that community feedback was taken Do not add sub-codes
This was a pilot	The present study is a pilot in which community feedback is taken
Community engagement in implementation	Communities had some opportunity to affect the implementation of the intervention. For example, community is required to spend resources on building health infrastructure and decides whether the intervention will be implemented in their community or those where community members come together to form governance structures such as health committees . OR The community has some responsibility for making decisions regarding the delivery of an intervention. Do not add sub-codes

For peer review only - http://bmjopen.bmj.com/site/about/guidelines.xhtml

For peer review only

	Governance and decisions	
	Absent	Explicit statements that the community did not have decision making power. Example: Statements that the community was excluded from implementation. This may be stated as a problem during the discussion. Do not add sub-codes
	Present	The community has some form of decision making in the implementation of the intervention. This could include a community led governance structure. Example: There was a community group monitoring the implementation of the intervention. Do not add-sub codes
	Provision of resources	The community provides resources during the implementation Examples: Community provides building materials Do not add sub-codes
Component 1		Each unique component should be coded separately. A unique component is defined as the set of activates that are dependent on one another. Activities within separate components are not dependent on one another. Select the depth of engagement for the initial description of the component but code all subsequent activities to the main code. Example: A sticker based reminder and a digital reminder could each be implemented independently and would be separate components Do not add sub-codes
	Does not include engagement	Descriptions of interventions that involved no community engagement Example: The production of cold chain transport of vaccines Do not add sub-codes
	Includes some engagement	Description of activities that involved some community engagement Example: Health clinics holding vaccination days and posting fliers Do not add sub-codes
	Primary focus is engagement	Description of activities whose primary focus was community engagement Example: The establishment of community health councils Do not add sub-codes
Component 10		
	Does not include engagement	
	Includes some engagement	
	Primary focus is engagement	
Component 2		
	Does not include engagement	
	Includes some engagement	
	Primary focus is engagement	

For peer review only - http://bmjopen.bmj.com/site/about/guidelines.xhtml

Component 3

Does not include engagement

Includes some engagement

Primary focus is engagement

Component 4

Does not include engagement

Includes some engagement

Primary focus is engagement

Component 5

Does not include engagement

Includes some engagement

Primary focus is engagement

Component 6

Does not include engagement

Includes some engagement

Primary focus is engagement

Component 7

Does not include engagement

Includes some engagement

Primary focus is engagement

Component 8

Does not include engagement

Includes some engagement

Primary focus is engagement

Component 9

Does not include engagement

For peer review only - http://bmjopen.bmj.com/site/about/guidelines.xhtml

For peer review only

Includes some engagement	
Primary focus is engagement	
Future directions for implementation	Discussion of next steps, how the program could be re-designed, or suggestions for the future Do not add sub-codes
Intervention exposure or reach	Discussions of the proportion of the eligible population that the intervention tried to reach. For example, the number of people who received the video (regardless of whether it was actually watched). Do not add sub-codes
Objective	Statement of the goal, objective, or target of the intervention Do not add sub-codes
Participant views of the intervention	Text related to how participants perceived the intervention
Negative views	Participants did not view the intervention positively
Positive views	Participants viewed the intervention positively
Personnel implementing the program	Description of who implemented the program Do not add sub-codes
Target group	Description of the target group for this program Note: This could be different from beneficiary selection if (for example) fathers were provided with information to get them to have their wives immunise their children. Do not add additional sub-codes
Uptake	Program uptake is defined as initial engagement with the intervention. This could be attending the first meeting, or expressing interest. Use sub-codes when appropriate and only primary code when the text does not fall into the other two. Do not add additional sub-codes
Assessment of uptake	Description of how uptake was assessed Example: Attendance sheets were used to collect information on attendance at the first meeting. Do not add sub-codes Do not add sub-codes
Description of uptake	Description of uptake Example: Uptake was high, with 80% of eligible women participating in the intervention Do not add sub-codes
Research design	This set of codes describes the research design and provides information on internal and external validity. Do not add sub-codes
Author discussion of external validity	Any explicit discussion by authors Do not add sub-codes
Conflict of interest	Discussion of conflicts of interests. This could include discussions related to the independence of evaluators and data collectors from the implementers and donors. Select the relevant code indicating presence / absence of a conflict Do not add sub-codes Do not add sub-codes

For peer review only - http://bmjopen.bmj.com/site/about/guidelines.xhtml

No	Select any statement directly indicating that there was no conflict of interest Example: The data collection team was hired as external contractors Do not add sub-codes
Unclear	Select any text that makes a potential conflict of interest unclear Do not add sub-codes
Yes	Select any text that indicates a clear conflict of interest Example: The implementers were the data collectors Do not add sub-codes
Data collection	Discussion of data collection. Use main code for general discussions and only use sub-codes as appropriate Do not add sub-codes
Response accuracy	Any measure or discussion of response accuracy Example: We expect that some of our findings may be related to social desirability bias Do not add sub-codes
Retrospective	Text indicating that data collection was retrospective. Include discussion of challenges this may have caused Do not add additional sub-codes
Sampling frame	Descriptions of the sampling frame. Only select text related to how people were ultimately enrolled / selected. Text related to geographic distribution of villages, the method of randomisation (e.g. computer generated vs. paper based), and other details about the preparation for selection is not needed. Example: Women of child bearing age were selected when they presented at the clinic. Do not add sub-codes
Data limitations	Discussions of the limitations to the data that pose challenges (eg. we could not analyse X because of Y) Do not add additional sub-codes
Ethical approval	Statement of ethical approval Do not add sub-codes
Future directions for research	Descriptions of future directions for research Do not add sub-codes
Other limitations	Text related to limitations in the research design that are not data related. Example: Failure in randomisation Example: Failure in randomisation Do not add sub-codes
Uptake and fidelity challenges	This set of codes describes challenges with uptake and fidelity. Most include sub-codes titled "present" and "absent." In each case, present reflects a statement that this challenge occurred and absent reflects a statement that this challenge was not encountered. Add sub-codes if needed
Adherence	Discussion of people (not) completing the intervention activities (eg. stopped attending training sessions). Could involve discussions of variability in engagement with the intervention or discussions of continued engagement with the intervention. Do not add sub-codes
Absent	There were not challenges related to adherence. ie adherence was high

For peer review only - http://bmjopen.bmj.com/site/about/guidelines.xhtml

For peer review only

Present	There were problems with adherence. ie adherence was low
Administrative	Challenges related to record keeping, monitoring, and other administrative activities Do not add sub-codes
Absent	There were not administrative challenges. Example: All paperwork was conducted quickly and on time
Present	There were administrative challenges Ex: Paperwork was finished late and this delayed implementation
Attrition in the research	People did not complete the research study (eg. did not respond to all rounds of data collection Do not add sub-codes
Absent	There was not attrition in the research study. ie attrition was low
Present	There was significant attrition in the research study. ie attrition was high
Budget	Challenges due to budget limitations Do not add sub-codes
Budget limitations in the intervention	The intervention could not be implemented in a desired way due to budget limitations Do not add sub-codes
Absent	There were not budgetary limitations to the intervention. Ex: Due to the generous funding of our donor, we were able to.....
Present	There were budgetary limitations to the intervention Ex: We had planned X, but due to budget limitations has to Y
Budget limitations in the research study	The research study could not be implemented as desired due to budget limitations Do not add sub-codes
Absent	There were not budgetary limitations to the research study Ex: Due to the generous funding of our donor, we were able to ......
Present	There were budgetary constraints in the research study Ex: We were not able to collect X data due to limited budget
Contamination	Select the appropriate type of contamination described Do not add sub-codes
Contamination between intervention and control	The control group was incidentally exposed to the intervention. Example: Nearby villages were aware of education campaigns conducted.
Absent	Explicit statements that this contamination did not occur Do not add sub-codes
Present	Contamination between intervention and control occurred
Contamination by other programs	Other ongoing programs may have affected results Do not add additional sub-codes
Absent	There was not contamination by other programs Ex: No other similar programs were functioning in the area
Present	There was contamination by other programs Ex: The Red Cross was conducting a similar program in control villages at the time of our intervention

For peer review only - http://bmjopen.bmj.com/site/about/guidelines.xhtml

Hawthorn effects	Any description of the change in behaviour (real or reported) that is the result of people knowing they are being observed, without any real intent to adopt or maintain these behaviours. Example: Respondents in the UCT group may have been affected by the evaluation process: community awareness about the aims of the project could have affected actual or reported behaviours. Do not add sub-codes
Absent	Hawthorn effects were not observed or expected Ex: By using an enumeration team that was unaffiliated with the intervention, we expect to have reduced the chances that people adjusted their behaviour simply due to the enumerator's presence
Present	Hawthorn effects were observed or expected Ex: Due to significant marketing around the desirability of certain behaviours, people may have changed how they acted in front of enumerators without actually adopting certain behaviours when they were not observed.
Low implementation fidelity	Deviations in implementation of the intervention from what was planned. Note: this must be from what was planned, not simply what would be considered desirable Do not add sub-codes
Absent	Fidelity challenges were not present. The intervention was implemented as intended. Ex: The intervention was implemented as intended
Present	The intervention was not implemented as intended. Ex: Due to confusion among facilitators, some changed the order in which material was presented.
Mobilization	Challenges related to mobilizing people to participate Do not add sub-codes
Mobilizing in the intervention	People were not willing to engage with the intervention Do not add sub-codes
Absent	Low participation was not a challenge observed. ie participation was high.
Present	The challenge of low participation was encountered. ie participation was low
Mobilizing in the research	Challenges getting those who participated in the intervention to join the research project. (eg. refuse to be interviewed) Do not add sub-codes
Absent	Low participation was not a challenge observed. ie participation was high.
Present	The challenge of low participation was encountered. ie participation was low
Other Challenges	

For peer review only - http://bmjopen.bmj.com/site/about/guidelines.xhtml

For peer review only

Cost data extraction tool

Study Characteristics	Variable Label	Explanation	Source
**Coder**			
Study ID	Study ID	This is the study ID - it should match the study ID from the Outcome Mapping Sheet (e.g., SC-SR_1)	Data extraction tool_immunisation
	Study ID Component		
Year	Year	Year published	Data extraction tool_immunisation
Design	Design	0=Experimental Design (e.g., RCT), 1=Quasi-Experimental Design	Data extraction tool_immunisation
Country	Country	Country of intervention	Data extraction tool_immunisation
Study Link			
Which Vaccines were studied?			
How many vaccine doses were delivered?			
Source of info about vaccines			
Outcomes	[Source from CEA Inventory]		
Target population			
Implementing partners		What organization(s) carried out the intervention? [Please list names of organization(s) that implemented the program, e.g. IRC, Government of Uganda. Etc)]	Studies
Analytical perspective		Was the perspective of the costing stated explicitly? [Yes, No]. Analytical perspective is the choice of who has standing in the costing and determines whose costs and benefits will be counted. Perspective should be stated explicitly in the costing, so here it is ok to do a kw search for "perspective" to check	Studies
Analytical perspective		What is the analytical perspective of the costing (i.e. donor, financial cost, economic or social perspective).	Studies
Treatment arms		How many treatment arms were included in the study [# of treatment arms]	
Comparison	Comparison	1=No intervention (service delivery as usual), 2=Other intervention, 3=Pipeline (wait-list) control (still service delivery as usual)	Data extraction tool_immunisation
Describe Comparison Group	Describe Comparison Group	If answer above is (1) no intervention, type N/A, if (2) Other Intervention, list what intervention the control group is receiving, if (3) Pipeline control, report when the control group will receive the intervention in relation to the treatment group (e.g., one year later)	Data extraction tool_immunisation
Subgroup	Subgroup	Is this analysis of a subgroup? 0=no, 1=yes	Data extraction tool_immunisation

For peer review only - http://bmjopen.bmj.com/site/about/guidelines.xhtml

If yes to subgroup, describe	If yes to subgroup, describe	Free text, describe the subgoup if applicable (e.g., boys, girls). If no subgroup, type N/A	Data extraction tool_immunisation
Source	Source	Note the page number, table number, column, and row you used to extract the data	Data extraction tool_immunisation
Treatment Effect	Treatment Effect	1=Intention to Treat (ITT), 2=Average Treatment Effect on the Treated (ATET), 3=Average Treatment Effect (ATE) 4 = Local Average Treatment Effect (LATE)	Data extraction tool_immunisation
Summary of intervention	Summary of intervention	Free text, what is the intervention	Intervention_community engagement_dataset [col E]
Component #	Component #	List in numbers 1-6	Intervention_community engagement_dataset [col H]
Description	Description	Description [Col I in Intervention_community engagement_dataset]	Intervention_community engagement_dataset [col I]
Notes	Notes	Description [Col S in Intervention_community engagement_dataset]	Intervention_community engagement_dataset [col S]
Exposure to intervention (in months)	Exposure to intervention (in months)	How long is the intervention exposure itself?	Data extraction tool_immunisation
Evaluation period (in months)	Evaluation period (in months)	The total number of months elapsed between offering an intervention and the point at which an outcome measure is taken post intervention, or as a follow-up measurement. If less than one month, use decimals (e.g., one week would be .25)	Data extraction tool_immunisation
Post-intervention or change from baseline?	Post-intervention or change from baseline?	0 = Post-intervention, 1 = Change from baseline	Data extraction tool_immunisation
Source of the Outcome data (only use for outcomes in category JAA)	Source of the Outcome data (only use for outcomes in category JAA)	1=Immunisation Card, 2=Recall, 3=Combination of both immunisation card and recall, 4=Health Admin Data, 5=N/A	Data extraction tool_immunisation
Author definition of outcome	Author definition of outcome	Free text - How does the author define the outcome?	Data extraction tool_immunisation
Activities		What are the main (non-evaluation) activities undertaken by the project? (e.g. program design, targeting, community outreach, training of trainers, training of beneficiaries, M&E, etc.). Describe	Studies
Activities Data Source		Was the source of descriptive information about program activities taken from a table or taken from text? [Text, Table, Other(describe)]	Studies
Timeline		Is an intervention timeline included with the report? [Yes, No, If yes, page #]	Studies
Ingredients (unit cost of ingredients)		Is a list of cost ingredients or inputs for CEA, CBA, or CUA provided? [Yes, No]	Studies

For peer review only - http://bmjopen.bmj.com/site/about/guidelines.xhtml

		Ingredients will be items like "personnel", "staff", "travel", "office support", "program materials", etc. Note that searches for the unit costs of activities should include text searches as well as searches of tables.	
Are unit costs reported?		Look for a table with units or unit costs reported. Unit costs give the value of individual items that were used during the intervention, for example the unit cost of a staff person, e.g. a "health specialist" would be given in terms of monthly wages, or compensation or salary. Note that searches for the unit costs of activities should include text searches as well as searches of tables. [Yes, No, If Yes Page #]	Studies
Cost data sources		Was a source of cost data reported? (i.e. financial reports or accounting systems of implementing NGOs or donors (expenditures), NGO or donor (budgets), (market prices) Describe (or copy from text). Clarify - expenditure is what's actually spent, budget is what's planned for spending / not audited, often incorrect and not updated.	Studies
Costing methodology		Was a method of costing described? (i.e. key works: gross costing, micro-costing, ingredients method, activity-based costing method) [if yes, what was the method].	
Type of efficiency analysis (see glossary)		Indicate (CBA/CEA/CUA/cost-minimization, or innovative approaches: Social Return on Investment (SROI), Multi-Criteria Appraisal (MCA). Response options [CBA, CEA, CUA, SROI, MCA, or N/A if only "total cost", also ok to insert descriptive results that do not conform, e.g. "cost transfer ratio"]	Studies
Any cost-effectiveness analysis?	Source [from CEA Inventory]		
Any quant discussion of costs? (Full program)	Source [from CEA Inventory]		
If yes, what type? [Page number]	Source [from CEA Inventory]		
Currency of cost reporting		What is the currency in which costs were reported, i.e. USD, Rwandan Francs, etc.	Studies
Exchange rate		What exchange rate was used for currency adjustments? [e.g. 1.2 USD to 1 Euro]	
Exch rate year (estimated)			
Exchange rate date		What was the date, month, year of the exchange rate used? [If the exchange rate was calculated as an average over the implementation period, copy verbatim from text the definition of average exchange rate used]	

For peer review only - http://bmjopen.bmj.com/site/about/guidelines.xhtml

Was there discussion of a discount rate?		[Yes, No]	Studies
Discount rate		Which is the discount rate used in the analysis [this will be a % , please give the value]	Studies
If no discount rate is applied, was any justification provided?		Paste explanation provided in text. We consider any acknowledgement of the discount rate to be justification. For example, ok to report" "we did not discount costs or benefits" as justification	Studies
Inflation adjustment		Does the report mention if costs are given in "real" or "nominal" terms? Look also for "inflation"	Studies
Inflation adjustment of cost reported		If costs are reported in real terms, what inflation index was used to adjust for inflation?	Studies
Base year of the costing	base_year	Was a "base year" (or "start year") of the costing reported? [Yes, No] [If yes, What year?]	Studies
Average cost per number of participants		Was the average cost of the intervention reported per participant? [Yes, No] [If Yes, $ Amount per participant, and currency]	Studies
What is the denominator used in average intervention cost?	ac_ denominator	[Number, Description (please copy description verbatim from report)]	Studies
Average cost per DALY or QALY?		Is the average cost per DALY or QALY reported? [Yes, No, If Yes Page #], Also, add note if cost 'per capita' was given rather than average cost per number of participants.	Studies
Total intervention cost (excluding vaccine cost)	total_cost	Report total cost of the intervention, and page.	Studies
Did the total intervention cost reported above include the cost of vaccines?	vac_incl	A "No" indicates that cost ingredients were reported, but there is no reporting of vaccine costs. "Unclear" means we cannot tell from the information given if vaccines were included in total cost [Yes, No, Unclear]	Studies
Total cost description	total_cost_ desc	If there is a description of what is included in total cost, copy description from text here	Studies
Government contribution included in total cost?	tc_gov_ contb	[Yes, No]	
Is more than one total cost given in the report?		[Yes, No, If Yes, please describe additional "total cost" that is reported	
Number of immunisations			
Cost per vaccine delivered, cost per additional immunisation administered or cost per dose (of vaccine). Type of vaccine is given in col I			

For peer review only - http://bmjopen.bmj.com/site/about/guidelines.xhtml

Description and page number of cost per additional vaccine delivered.		Note if average costs per additional vaccine or marginal costs per additional vaccine is reported, where marginal costs should exclude fixed costs.	
Cost per additional child immunised_ All	cpc_all	[Yes, No, If Yes, please report cost per additional child immunised_all]	
Description and page number of cost per additional CHILD Immunised			
Cost per additional child immunised_DPT3	cpc_dpt3	[Yes, No, If Yes, please report cost per additional child immunised with DPT3]. Please make a note if only DPT1 was reported.	
Cost per additional child immunised_Measles	cpc_measles	[Yes, No, If Yes, please report cost per additional child immunised with measles]	
Baseline immunisation coverage of the target population_All		What was the reported baseline rate of immunisation (all immunisations) in the target population? [Percentage, or range]	Studies
Was baseline immunisation coverage of All immunisations reported for the target population, general population, or sample?		Was baseline immunisation coverage of All immunisations reported for the target population, general population, or sample? [target population, general population, sample]	Studies
Baseline immunisation coverage of the target population_DPT3		What was the reported baseline rate of immunisation (DPT3) in the target population? [Percentage, or range]	Studies
Was baseline immunisation coverage of DPT3 reported for the target population, general population, or sample?		Was baseline immunisation coverage of DPT3 reported for the target population, general population, or sample? [target population, general population, sample]	Studies
Baseline immunisation coverage of the target population_Measles		What was the reported baseline rate of immunisation (Measles) in the target population? [Percentage, or range]	Studies
Final immunisation coverage of the target population_All		What was the reported final rate of immunisation (all immunisations) in the target population? [Percentage, or range]	Studies
Final immunisation coverage of the target population_DPT3		What was the reported final rate of immunisation (DPT3) in the target population? [Percentage, or range]	Studies
Final immunisation coverage of the target population_Measles		What was the reported final rate of immunisation (Measles) in the target population? [Percentage, or range]	Studies
Non-compliance		Was there any evidence of non-compliance with treatment assignment. Non-compliance is where individuals assigned to treatment do not take the treatment, or the case where individuals assigned to control DO take the treatment. Ok to keyword search on "non-compliance", "non compliance", "noncompliance" [Yes, No] [If Yes, copy related text on non-compliance]	Studies

For peer review only - http://bmjopen.bmj.com/site/about/guidelines.xhtml

Spillover		Was there any evidence of spillover where individuals assigned to control receive a benefit or incur a 'cost' that results from the treatment intervention? [Yes, No] [If Yes, copy related text on spillovers detected]	Studies
Attrition or drop out		Was there evidence that participants dropped out of the study before follow-up could be completed? [Yes, No] [If Yes, copy related text on drop outs]	Studies
Vaccination coverage_All	JAA01. Full routine immunisation for children	Binary measure of whether or not children have received all routine vaccinations for the relevant country or region.	Data extraction tool_immunisation
Vaccination coverage_DPT3	JAA05. DPT3	Binary measure of whether or not children have received the third dose of the DPT or pentavalent vaccine. If the study does not specifically say "DPT3" (or "pentavalent 3"), but refers to "complete DPT/penta vaccination" or something similar, then use this code.	Data extraction tool_immunisation
Vaccination coverage_Measles	JAA11. Measles	Binary measure of whether or not children have received the measles vaccine	Data extraction tool_immunisation
Costs for significant outcomes only?		Does the report mention that costs will be reported only where significant outcomes are observed? [Yes, No]	

List of coded variables

VARIABLE LABEL	EXPLANATION
Study ID	This is the study ID - it should match the study ID from the Outcome Mapping Sheet (e.g., SC-SR_1)
Estimate ID	The estimate ID will provide a specific number for each effect size extracted and should include the original study number, underscore, then the unique ID number (e.g., SC-SR1_1, SC-SR1_2 and so on)
Author	For 1 author: leading author last name (e.g., Gomez)<br>For 2 authors: both author last names with ampersand in between (e.g., Smith & Bahn)<br>For 3 or more authors: leading author last name followed by et al. (e.g., Gupta et al.)
Year	Year published
Design	0=Experimental Design (e.g., RCT), 1=Quasi-Experimental Design
How Counterfactual is Chosen	Free text (e.g., random control trial, propensity score matching, etc) - Multiple codes are ok
Analysis type for this effect size	Free text, what type of analysis was used (Regression, 2SLS, ANCOVA, etc.)- Multiple codes are ok
Country	Country of intervention
Estimate Type	Type of data for this effect size: 1 = Continuous - means and SDs, 2 = Continuous - mean difference and SD, 3 = Dichotomous outcome - proportions, 4 = Regression data - dichotomous outcome (e.g., logistic regression)

For peer review only - http://bmjopen.bmj.com/site/about/guidelines.xhtml

	5 = Regression data - continuous outcome (e.g., linear regression)
Comparison	1=No intervention (service delivery as usual), 2=Other intervention, 3=Pipeline (wait-list) control (still service delivery as usual)
Describe Comparison Group	If answer above is (1) no intervention, type N/A, if (2) Other Intervention, list what intervention the control group is receiving, if (3) Pipeline control, report when the control group will receive the intervention in relation to the treatment group (e.g., one year later)
Subgroup	Is this analysis of a subgroup? 0=no, 1=yes
If yes to subgroup, describe	Free text, describe the subgoup if applicable (e.g., boys, girls). If no subgroup, type N/A
Source	Note the page number, table number, column, and row you used to extract the data
Treatment Effect	1=Intention to Treat (ITT), 2=Average Treatment Effect on the Treated (ATET), 3=Average Treatment Effect (ATE) 4 = Local Average Treatment Effect (LATE)
Intervention	Free text, what is the intervention
Exposure to intervention (in months)	How long is the intervention exposure itself?
Evaluation period (in months)	The total number of months elapsed between offering an intervention and the point at which an outcome measure is taken post intervention, or as a follow-up measurement. If less than one month, use decimals (e.g., one week would be .25)
Post-intervention or change from baseline?	0 = Post-intervention, 1 = Change from baseline
Source of the Outcome data (only use for outcomes in category JAA)	1=Immunization Card, 2=Recall, 3=Combination of both immunization card and recall, 4=Health Admin Data, 5=N/A, 6 = unclear
Author definition of outcome	Free text - How does the author define the outcome?
Intervention	Coded as engagement as intervention, engagement in the intervention design, engagement in implementation autonomy, or multiple engagement types (place a 1 in the appropriate column
If yes to multiple engagement types	Identify which engagement types are used.
Implemented by the government	0 = no, 1 = yes
Were new cadres of health workers formed?	0 = no, 1 = yes
Was vaccination hesitency identified as a barrier?	0 = no, 1 = yes
Baseline rates of full immunisation	record percentage vaccinated
Baseline rates of DPT3	record percentage vaccinated
**OUTCOME CODES**	
	Code 1 under any applicable columns.
**EFFECT SIZE DATA EXTRACTION**	
Reverse Sign (i.e., decrease is good)	Record 0='no' if an increase is good, record 1='yes' if a decrease is good and the sign needs to be reversed.
Unit of analysis	What is the unit of analysis? UOA for this effect size: 1= Individual, 2= Household, 3= Group (e.g.

For peer review only - http://bmjopen.bmj.com/site/about/guidelines.xhtml

	community organisation), 4= Health Center, 5 = Village, 6 = Other, 7 = Not clear
mean_t	Outcome mean for the treatment group
sd_t	Outcome standard deviation for treatment group
mean_c	Outcome mean for the comparison group
sd_c	Outcome standard deviation for control group
mean_overall_diff	Overall mean difference (treatment - control)
diff se	Standard error of the overall mean difference
Diff _t	t-statistic of mean difference
Odds ratio	Odds ratio reported in the study
OR_se	Odds ratio standard error reported in the study
Risk ratio	Risk ratio reported in study
RR_se	Risk ratio standard error
reg_coeff	Report the regression coefficient of the treatment effect
reg_SE	Report the associated standard error of the regression coefficient.
reg_t	Report the associated t statistic of the effect size (coefficient/SE)
Exact p value	Exact p value if given, if not, record as written in the manuscript (e.g., p < .001, or p > .05)
clust_t	Number of clusters - treatment group
clust_c	Number of clusters - control group
clust_T	Number of clusters - total sample
n_t	Sample size - treatment group
n_c	Sample size - control group
n_T	Sample size - total sample
periods (1 if cross sectional)	Record how many periods of evaluation there are (e.g., cross section is 1, panel data with 3 measurements is 3)
Treatment Variable	Record the treatment variable as written in the model (e.g., the variable name the author uses, such as ("Intervention x Time")
dataset	Record if data comes from an identified dataset
coder	Record your name
Notes	Record any notes important for the team
ROB Category	0 = low risk of bias, 1 = some concerns, 2 = high risk of bias

For peer review only

Appendix 7: Risk of bias assessment tools

Quantitative risk of bias tool

Code	Question	Coding format	Criteria
General	ID	EPPI ID	
Genera	Study first author	Open answer	
General	Time taken to complete assessment	Minutes	
General	Design type: What type of study design is used?	1= Randomised controlled trial (RCT) (random assignment to households/individuals) or quasi-RCT<br>2 = Cluster-RCT (quasi-RCT)<br>3 = Pseudo-RCT	-
General	Methods used for analysis: Which methods are used to control for selection bias and confounding?	1 = Statistical matching (PSM, CEM, covariate matching)<br>2 = Difference in differences (DID) estimation methods<br>3 = IV-regression (2-stage least squares or bivariate probit)<br>4 = Heckman selection model<br>5 = Fixed effects or random effects regression<br>6 = Covariate adjusted estimation<br>7 = Propensity weighted regression<br>8 = Comparison of means<br>9 = Other (please state)	-
General	Design and analysis method description	Open answer	Briefly describe the study design and analysis method undertaken by the authors.
General	Study population	Open answer	Provide any details in the paper that describe how the study population was selected, answering the question: what is the sampling strategy to recruit participants from that population into the evaluation?
General	Type of comparison group	1=No intervention (service delivery as usual)<br>2=Other intervention<br>3=Pipeline (wait-list) control (still service delivery as usual)	Indicate type of comparison group
General	Type of comparison group (if other)	Open answer	

For peer review only - http://bmjopen.bmj.com/site/about/guidelines.xhtml

General	Ethical clearance	Open answer	Provide any details of ethical research clearances granted. Report unclear if this information is not available.
General	Study registration	Open answer	Provide any details of study registration, including registry IDs, etc.
1: Bias arising from randomisation process	1.1 Was the allocation sequence random?	(1) Yes;<br>(2) Probably yes;<br>(3) Probably no;<br>(4) No;<br>(5) No information;	1.1 Was the allocation sequence random?<br>Answer 'Yes' if a random component was used in the sequence generation process. Examples include computer-generated random numbers; reference to a random number table; coin tossing; shuffling cards or envelopes; throwing dice; or drawing lots. Minimization is generally implemented with a random element (at least when the scores are equal), so an allocation sequence that is generated using minimization should generally be considered to be random.<br><br>Answer 'No' if no random element was used in generating the allocation sequence or the sequence is predictable. Examples include alternation; methods based on dates (of birth or admission); patient record numbers; allocation decisions made by clinicians or participants; allocation based on the availability of the intervention; or any other systematic or haphazard method.<br><br>Answer 'No information' if the only information about randomization methods is a statement that the study is randomized.<br><br>Note: In some situations a judgement may be made to answer 'Probably no' or 'Probably yes'. For example, if the study was large, conducted by an independent trials unit or carried out for regulatory purposes, it may be reasonable to assume that the sequence was random. Alternatively, if other (contemporary) trials by the same investigator team have clearly used non-random sequences, it might be reasonable to assume that the current study was done using similar methods.
1: Bias arising from randomisation process	Question 1.1 answer justification	Open answer	Justification for coding decision<br>(Include a brief summary of justification for rating, mentioning your response to all sub questions, cite relevant pages).
1: Bias arising from randomisation process	1.2 Did baseline differences between intervention groups suggest a problem with the randomisation process?	(1) Yes;<br>(2) Probably yes;<br>(3) Probably no;<br>(4) No;<br>(5) No information;	1.2 Did baseline differences between intervention groups suggest a problem with the randomization process?<br><br>Note that differences that are compatible with chance do not lead to a risk of bias.

For peer review only - http://bmjopen.bmj.com/site/about/guidelines.xhtml

			Answer 'No' if no imbalances are apparent or if any observed imbalances are compatible with chance.<br><br>Answer 'Yes' if there are imbalances that indicate problems with the randomization process, including:<br>(1) substantial differences between intervention group sizes, compared with the intended allocation ratio;<br>or<br>(2) a substantial excess in statistically significant differences in baseline characteristics between intervention groups, beyond that expected by chance (i.e., on more than 10% of the characteristics reported); or<br>(3) imbalance in one or more key prognostic factors, or baseline measures of outcome variables, that is very unlikely to be due to chance and for which the between-group difference is big enough to result in bias in the intervention effect estimate.<br>Also answer 'Yes' if there are other reasons to suspect that the randomization process was problematic, such as:<br>(4) excessive similarity in baseline characteristics that is not compatible with chance; or<br>(5) surprising absence of one or more key baseline characteristics that would be expected to be reported.<br><br>Answer 'No information' when there is no useful baseline information available (e.g. abstracts, or studies that reported only baseline characteristics of participants in the final analysis).<br><br>Note: Trialists may undertake analyses that attempt to deal with flawed randomization by controlling for imbalances in prognostic factors at baseline. To remove the risk of bias caused by problems in the randomization process, it would be necessary to know, and measure, all the prognostic factors that were imbalanced at baseline. It is unlikely that all important prognostic factors are known and measured, so such analyses will at best reduce the risk of bias.
1: Bias arising from randomisation process	Question 1.2 answer justification	Open answer	Justification for coding decision<br>(Include a brief summary of justification for rating, mentioning your response to all sub questions, cite relevant pages).
1: Bias arising from	Assignment mechanism: Was the allocation or identification	(0) Low risk of bias<br>(1) Medium risk of bias/Some concerns	Utliize your answers for questions 1.1 and 1.2 to determine the overall score.

For peer review only

randomisation process	mechanism random or as good as random?	(2) High risk of bias	
1: Assignment mechanism - Justification	Assignment justification	Open answer	Justification for coding decision<br>(Include a brief summary of justification for rating, mentioning your response to all sub questions, cite relevant pages).
2: Unit of analysis - Assessment	Unit of analysis: Is unit of analysis in cluster allocation addressed in standard error calculation?	1=Yes<br>2=No<br>3=Not reported/unclear<br>4=Not applicable	Score "Yes" if Unit of Analysis = Unit of Randomization OR if UoA ≠ UoR and standard errors are clustered at the UoR level OR data is collapsed to the UoR level<br><br>Score "Not reported/unclear" if not enough information is provided on the way the standard errors were calculated or what the unit of analysis is.<br><br>Score "Not applicable" if it is not a cluster RCT.<br><br>Score "No" otherwise.Score "Yes" if Unit of Analysis = Unit of Randomization OR if UoA ≠ UoR and standard errors are clustered at the UoR level OR data is collapsed to the UoR level<br><br>Score "Not reported/unclear" if not enough information is provided on the way the standard errors were calculated or what the unit of analysis is.<br><br>Score "Not applicable" if it is not a cluster RCT.<br><br>Score "No" otherwise.
2: Unit of analysis - Justification	Question 2 answer justification (provide page numbers)	Open answer	-
3. Bias due to missing outcome data (panel data)	3.1. Was there attrition, if yes, then what was the rate in treatment and control/comparison groups?	(1) Yes;<br>(2) Probably yes;<br>(3) Probably no;<br>(4) No;<br>(5) No information;	3.1. Was there attrition, if yes, then what was the rate in treatment and control/comparison groups?<br><br>In case of attrition, answer 'yes' or 'probably yes', otherwise answer 'no' or 'probably no'.<br><br>If there is no discussion on attrition then score 'no information'.<br><br>Specify the rate of attrition by the study arms.<br>Specify the attrition rate assumed for power calculations.

For peer review only - http://bmjopen.bmj.com/site/about/guidelines.xhtml

			Has the study discussed the ability of the follow-up study to detect the hypothesised outcome effect with the sample size attained?
3. Bias due to missing outcome data – Justification (panel data)	Question 3.1 answer justification	Open answer	Justification for coding decision<br>(Include a brief summary of justification for rating, mentioning your response to all sub questions, cite relevant pages).
3. Bias due to missing outcome data (panel data)	3.2. Does the study establish that attrition is randomly distributed (e.g. by examining correlation with determinants of outcomes, in both treatment and comparison groups or by presenting data showing balance on key characteristics across treatment and control)?	(1) Yes;<br>(2) Probably yes;<br>(3) Probably no;<br>(4) No;<br>(5) No information;	3.2. Does the study establish that attrition is randomly distributed (e.g. by examining correlation with determinants of outcomes, in both treatment and comparison groups or by presenting data showing balance on key characteristics across treatment and control)?<br><br>Score 'yes' or 'probably yes' if balance on key characteristics between attritors and non-attritors for each intervention group (including control) using baseline data has been presented and finds that there is no systematic difference on at least observable dimensions.<br><br>Score 'no' or 'probably no' if the study finds that there is a systematic difference on observables between attritors and non-attritors in any of the intervention groups.<br><br>If the baseline balance table between atritors and non-attritors is not presented or discussed then score 'no information'.
3. Bias due to missing outcome data – Justification (panel data)	Question 3.2 answer justification	Open answer	Justification for coding decision<br>(Include a brief summary of justification for rating, mentioning your response to all sub questions, cite relevant pages).
3. Bias due to missing outcome data – (panel data)	3.3. If there is non-random attrition, have authors used convincing statistical techniques to identify and adjust for the attrition bias?	(1) Yes;<br>(2) Probably yes;<br>(3) Probably no;<br>(4) No;<br>(5) No information;<br>(6) Not applicable	3.3. If there is non-random attrition, have authors used convicing statistical techniques to identify and adjust for the attrition bias?<br><br>If the study has used convincing parametric or non-parametric techniques (like ignorable maximum likelihood, Manski-Lee bounds) to identify and adjust for attrition bias, then score 'yes' or 'probably yes'. If the techniques used are not convincing then score 'no' or 'probably no'. If the study does not discuss using any statiscal techniques for identifying and adjusting for attrition bias then score 'no information'.

For peer review only - http://bmjopen.bmj.com/site/about/guidelines.xhtml

			If there is no non-random attrition, then this question does not apply and the score should be 'not applicable'
3. Bias due to missing outcome data – (panel data)	Question 3.3 answer justification	Open answer	Justification for coding decision<br>(Include a brief summary of justification for rating, mentioning your response to all sub questions, cite relevant pages).
3. Bias due to missing outcome data – Justification (panel data)	Bias due to missing outcome data justification	Open answer	Justification for coding decision<br>(Include a brief summary of justification for rating, mentioning your response to all sub questions, cite relevant pages).
3. Bias due to missing outcome data - Assessment		(0) Low risk of bias<br>(1) Medium risk of bias/Some concerns<br>(2) High risk of bias	Utliize your answers for questions 3.1 to 3.3 to determine the overall score.
3. Bias due to missing outcome data (repeated cross section)	3.1. Was there difference in sample size across baseline and follow-up surveys among treatment and control groups? If yes, what was the rate of difference among treatment and control/comparison groups? Was it lower or higher than baseline?	(1) Yes;<br>(2) Probably yes;<br>(3) Probably no;<br>(4) No;<br>(5) No information;	3.1. Was there difference in sample size across baseline and follow-up surveys among treatment and control groups? If yes, what was the rate of difference among treatment and control/comparison groups? Was it lower or higher than baseline?<br><br>In case of difference, answer 'yes' or 'probably yes', otherwise answer 'no' or 'probably no'.<br><br>If there is no information to assess the differences, then score 'no information'.<br><br>Specify the rate of difference by the study arms.<br>Specify the non-compliance and/or attrition rate assumed for power calculations.<br>Has the study discussed the ability of the follow-up study to detect the hypothesised outcome effect with the sample size attained?
3. Bias due to missing outcome data – Justification (repeated cross section)	Question 3.1 answer justification	Open answer	Justification for coding decision<br>(Include a brief summary of justification for rating, mentioning your response to all sub questions, cite relevant pages).
3. Bias due to missing outcome	3.2. Does the study establish that the sampling frame for baseline and follow-up	(1) Yes;<br>(2) Probably yes;<br>(3) Probably no;	3.2. Does the study establish that the sampling frame for baseline and follow-up surveys is same? Are the clusters (eg health center) and sub-clusters (eg. villages) the same across baseline and follow-up surveys?

For peer review only - http://bmjopen.bmj.com/site/about/guidelines.xhtml

data (repeated cross section)	surveys is same? Are the clusters (eg health center) and sub-clusters (eg. villages) the same across baseline and follow-up surveys?	(4) No;<br>(5) No information;	Score 'yes' or 'probably yes' if sampling frame (clusters and sub-clusters) are the same for baseline and follow-up surveys<br><br>Score 'no' or 'probably no' if the clusters or sub-clusters were different across baseline and follow-up surveys.<br><br>If there is no information on sampling frame across baseline or follow-up surveys, then score 'no information'.
3. Bias due to missing outcome data – Justification (repeated cross section)	Question 3.2 answer justification	Open answer	Justification for coding decision<br>(Include a brief summary of justification for rating, mentioning your response to all sub questions, cite relevant pages).
3. Bias due to missing outcome data –(repeated cross section)	3.3. Was the sample selection of respondents carried out in the same way as in the baseline? Was the intended number of respondents in the clusters/sub-clusters same across baseline and follow-up surveys?	(1) Yes;<br>(2) Probably yes;<br>(3) Probably no;<br>(4) No;<br>(5) No information;	3.3. Was the sample selection of respondents carried out in the same way as in the baseline? Was the intended number of respondents in the sub-clusters same across baseline and follow-up surveys?<br><br>Score 'yes' or 'probably yes' if the study used same listing process for selection of respondents and their intended number was same across baseline and follow-up surveys,<br><br>Score 'no' or 'probably no' if the study used different listing process for selection of respondents or their intended number was different across baseline and follow-up surveys.<br><br>If there is no information on the selection process of respondents then score 'no information'.
3. Bias due to missing outcome data - Justification (repeated cross section)	Question 3.3 answer justification	Open answer	Justification for coding decision<br>(Include a brief summary of justification for rating, mentioning your response to all sub questions, cite relevant pages).
3. Bias due to missing outcome data – Assessment	Bias due to missing outcome data	(0) Low risk of bias<br>(1) Medium risk of bias/Some concerns<br>(2) High risk of bias	Utliize your answers for questions 3.1 to 3.3 to determine the overall score using the criteria below

For peer review only - http://bmjopen.bmj.com/site/about/guidelines.xhtml

(repeated cross section)			
Bias due to missing outcome data – Justification (repeated cross section)	Bias due to missing outcome data justification	Open answer	Justification for coding decision<br>(Include a brief summary of justification for rating, mentioning your response to all sub questions, cite relevant pages).
3. Bias due to missing outcome data - Assessment	(0) Low risk of bias<br>(1) Medium risk of bias/Some concerns<br>(2) High risk of bias		Utliize your answers for questions 3.1 to 3.3 to determine the overall score.
3.Bias due to missing outcome data - Justification	Bias due to missing outcome data justification	Open answer	Justification for coding decision<br>(Include a brief summary of justification for rating, mentioning your response to all sub questions, cite relevant pages).
4: Bias due to deviations from intended interventions - Assessment	4.1. Was the study adequately protected against spill-overs/ contamination?	(1) Yes;<br>(2) Probably yes;<br>(3) Probably no;<br>(4) No;<br>(5) No information;	4.1. Was the study adequately protected against spill-overs/ contamination?<br><br>Score "Yes" if the intervention is unlikely to spill-over to comparisons (e.g. participants and non-participants are geographically and/or socially separated from one another and general equilibrium effects are not likely) and that the treatment and comparisons are isolated from other interventions which might explain changes in outcomes.<br><br>Score "No" if allocation was at the individual or classroom level and there are likely spill-overs within households and communities which are not controlled for (such as using trasferable vouchers), or other interventions likely to affect outcomes operating at the same time in either group.<br><br>Score "no information" if spill-overs and/or contamination are not discussed
4: Bias due to deviations from intended interventions - Justification	Question 4.1 answer justification	Open answer	Justification for coding decision<br>(Include a brief summary of justification for rating, mentioning your response to all sub questions, cite relevant pages).

For peer review only - http://bmjopen.bmj.com/site/about/guidelines.xhtml

4: Bias due to deviations from intended interventions - Assessment	4.2. Was the process of being monitored free from bias?	(1) Yes;<br>(2) Probably yes;<br>(3) Probably no;<br>(4) No;<br>(5) No information;	4.2. Was the process of being monitored free from bias?<br><br>Score "Yes" if the authors state explicitly that the process of monitoring the intervention is blinded, or argue convincingly why it is not likely that intervention delivery could affect the performance of participants in treatment and comparison groups in different ways (such as resulting in Hawthorne or John Henry effects). or there is nothing in the surveys that might have given the control participants an idea of what the other group might receive (e.g. provide cash transfer to the intervention area participants or reveal informmation that they did not have before or they did but there is no risk that this has changed their behaviours;<br><br>Score "No" if the the authors do not use an appropriate method to prevent Hawthorne and John Henry Effects (e.g. blinding of monitoring or other methods to ensure consistent monitoring across groups) and there is a risk that the intervention delivery or survey process could have changed the behaviours of treatment and comparison groups in different ways.<br><br>Score "no information" if the authors do not discuss potential bias due to monitoring of intervention.<br><br>Hawthorne effects may result where participants know that they are being observed and John Henry Effects may result from participant knowledge of being compared.
4: Bias due to deviations from intended interventions - Justification	Question 4.2 answer justification	Open answer	Justification for coding decision<br>(Include a brief summary of justification for rating, mentioning your response to all sub questions, cite relevant pages).
4: Bias due to deviations from intended interventions - Assessment	4.3. Was an appropriate analysis used to estimate the effect of assignment to intervention?	(1) Yes;<br>(2) Probably yes;<br>(3) Probably no;<br>(4) No;<br>(5) No information;	44.3. Was an appropriate analysis used to estimate the effect of assignment to intervention?<br><br>Both intention-to-treat (ITT) analyses and modified intention to treat (mITT) analyses excluding participants with missing outcome data should be considered appropriate. (Missing outcome data are addressed in a separate domain). Both 'as treated' analyses (in which trial participants are grouped according to the intervention that they received, rather than according to their assigned intervention) and

For peer review only - http://bmjopen.bmj.com/site/about/guidelines.xhtml

			naïve 'per-protocol' analyses (excluding trial participants who did not receive their assigned intervention) should be considered inappropriate. Analyses excluding eligible trial participants post-randomization should also be considered inappropriate, but post-randomization exclusions of ineligible participants (when eligibility was not confirmed until after randomization, and could not have been influenced by intervention group assignment) can be considered appropriate.<br><br>Score "Yes" if ITT or modified ITT analysis has been used, AND common analysis methods are used AND any covariates imbalanced at baseline are included as covariates in the analysis.<br>Score "No" otherwise. You may also score no if they use an uncommon analysis methods, such as 3SLS, or if they analyse dichotomous outcome using continuous methods (e.g., using linear regression instead of using the more appropriate logit or probit models) or if the is baseline imbalance but those covariates are not included in the model.<br><br>If the analytical specification is not provided score "no information"
4: Bias due to deviations from intended interventions - Justification	Question 4.3 answer justification	Open answer	Justification for coding decision<br>(Include a brief summary of justification for rating, mentioning your response to all sub questions, cite relevant pages).
4: Bias due to deviations from intended interventions - Assessment	Deviations from intended interventions: Spill-overs, cross-overs, contamination and performance bias: was the study adequately protected against spill-overs, cross-overs, contamination, and performance bias?	(0) Low risk of bias<br>(1) Medium risk of bias/Some concerns<br>(2) High risk of bias	Utliize your answers for questions 4.1 to 4.3 to determine the overall score.
4: Bias due to deviations from intended interventions - Justification	Deviations justification	Open answer	Justification for coding decision<br>(Include a brief summary of justification for rating, mentioning your response to all sub questions, cite relevant pages).<br><br>For example, intervention groups are geographically separated, authors use intention to treat estimation or instrumental variables to

For peer review only - http://bmjopen.bmj.com/site/about/guidelines.xhtml

			account for non-adherence, and survey questions are not likely to expose individuals in the control group to information about desirable behaviours ('survey effects').
5. Outcome measurement bias	5.1 Could the measurement of the outcome be different between the study arms?	(1) Yes;<br>(2) Probably yes;<br>(3) Probably no;<br>(4) No;<br>(5) No information;	5.1 Could the measurement of the outcome be different between the study arms?<br><br>Comparable methods of outcome measurement (data collection) involve the same measurement methods and thresholds, used at comparable time points. Differences between intervention groups may arise because of 'diagnostic detection bias' in the context of passive collection of outcome data, or if an intervention involves additional visits to a healthcare provider, leading to additional opportunities for outcome events to be identified.<br><br>Score 'yes' or 'probably yes' if the outcomes have been measured at different times periods for treatment and control arms or have used different methods of measurement.<br><br>Score 'no' or 'probably no' otherwise.<br><br>If no information is provided on the the time period and methods of data collection in treatment and control arms, then score 'no information'
5. Outcome measurement bias - Justification	Question 5.1 answer justification	Open answer	Justification for coding decision<br>(Include a brief summary of justification for rating, mentioning your response to all sub questions, cite relevant pages).
5. Outcome measurement bias	5.2. Were the outcome assessors (enumerators) blinded to the intervention assignment?	(1) Yes;<br>(2) Probably yes;<br>(3) Probably no;<br>(4) No;<br>(5) No information;	5.2. Were the outcome assessors (enumerators) blinded to the intervention assignment?<br><br>Score 'yes' or 'probably yes' if outcome assessors were blinded to intervention status and 'no' or 'probably no' otherwise.<br><br>If no information is provided on blinding of the outcome assessors, then score 'no information'.<br><br>Note that for participant-reported outcomes, the outcome assessor is the study participant.
5. Outcome measurement	Question 5.2 answer justification	Open answer	Justification for coding decision

For peer review only - http://bmjopen.bmj.com/site/about/guidelines.xhtml

bias - Justification			(Include a brief summary of justification for rating, mentioning your response to all sub questions, cite relevant pages).
5. Outcome measurement bias	5.3. If not blinded, could assessment of the outcome have been influenced by knowledge or administration of intervention received?	(1) Yes;<br>(2) Probably yes;<br>(3) Probably no;<br>(4) No;<br>(5) No information;	5.3. If not blinded, could assessment of the outcome have been influenced by knowledge or administration of intervention received?<br><br>Knowledge or administration of the assigned intervention could influence participant-reported outcomes (such as level of pain, immunization outcomes using recall), observer-reported outcomes involving some judgement, and intervention provider decision outcomes. They are unlikely to influence observer-reported outcomes that do not involve judgement, for example all-cause mortality.<br><br>For participant-reported outcomes, the assessment of outcome is potentially influenced by knowledge or administration of intervention received, leading to a judgement of at least 'Some concerns'. Review authors will need to judge whether it is likely that participants' reporting of the outcome was influenced by knowledge or administration of intervention received, in which case risk of bias is considered to be high.<br><br>Score 'yes' or 'probably yes' if assessment of outcome could have been influenced by knowledge of intervention received (e.g., if ANY of the immunization coverage outcomes are assessed through caregiver recall) and 'no' or 'probably no' otherwise (e.g., if immunization coverage outcome is assessed by immunization card).<br>If no information is available to make the assessment then score 'no information'.
5. Outcome measurement bias - Justification	Question 5.3 answer justification	Open answer	Justification for coding decision<br>(Include a brief summary of justification for rating, mentioning your response to all sub questions, cite relevant pages).
5. Outcome measurement bias - Assessment	Outcome measurement bias: Was the study free from biases in outcome measurement?	(0) Low risk of bias<br>(1) Medium risk of bias/Some concerns<br>(2) High risk of bias	Utliize your answers for questions 5.1 to 5.3 to determine the overall score.
5. Outcome measurement bias - Justification	Outcome measurement justification	Open answer	Justification for coding decision<br>(Include a brief summary of justification for rating, mentioning your response to all sub questions, cite relevant pages).

For peer review only - http://bmjopen.bmj.com/site/about/guidelines.xhtml

6. Reporting bias	6.1. Is a pre-analysis plan or protocol available which provides sufficient detail?	(1) Yes;<br>(2) Probably yes;<br>(3) Probably no;<br>(4) No;<br>(5) No information;	6.1. Is a pre-analysis plan or protocol available which provides sufficient detail?<br><br>Score 'yes' if they reference a preanalysis plan, and 'no' otherwise.
6. Reporting bias - Justification	Question 6.1 answer justification	Open answer	Justification for coding decision<br>(Include a brief summary of justification for rating, mentioning your response to all sub questions, cite relevant pages).
6. Reporting bias	6.2. Were all primary and secondary outcomes reported as per the pre-analysis plan/protocol?	(1) Yes;<br>(2) Probably yes;<br>(3) Probably no;<br>(4) No;<br>(5) No information;	6.2. Were all primary and secondary outcomes reported as per the pre-analysis plan/protocol?<br><br>Score "Yes" or "probably yes" if there is no evidence that outcomes were selectively reported (e.g. results for all relevant outcomes in the methods section are reported in the results section)<br><br>Score "No" or "probably no" if some important outcomes are subsequently omitted from the results or the significance and magnitude of important outcomes was not assessed or if multiple measurements of an outcome were made but only one or a subset is reported on the basis of the results (e.g. statistical significance)<br><br>Score "No information" if pre-analysis not available or the outcome intentions are not reported in sufficient detail to enable an assessment.
6. Reporting bias - Justification	Question 6.2 answer justification	Open answer	Justification for coding decision<br>(Include a brief summary of justification for rating, mentioning your response to all sub questions, cite relevant pages).
6. Reporting bias	6.3. Do reported results for the outcomes correspond to all intended analyses?	(1) Yes;<br>(2) Probably yes;<br>(3) Probably no;<br>(4) No;<br>(5) No information;	6.3. Do reported results for the outcomes correspond to all intended analyses?<br><br>A particular outcome domain may be analysed in multiple ways. Examples include: unadjusted and adjusted models; final value vs change from baseline vs analysis of covariance; transformations of variables; different definitions of composite outcomes (e.g. 'major adverse event'); conversion of continuously scaled outcome to categorical data with different cut-points; different sets of covariates for adjustment; and different strategies for dealing with missing data. Application of multiple methods generates multiple effect estimates for a specific outcome domain. If multiple estimates are generated but only one or a subset is reported on the basis of the results (e.g.

For peer review only - http://bmjopen.bmj.com/site/about/guidelines.xhtml

			statistical significance), there is a high risk of bias in the fully reported result.<br><br>Answer ‘No’ or ‘Probably No’ if:<br>There is clear evidence (usually through examination of a trial protocol or statistical analysis plan) that a domain was analysed in multiple ways, but data for only one or a subset of analyses is fully reported (without justification), and the fully reported result is likely to have been selected on the basis of the results. Selection on the basis of the results arises from a desire for findings to be newsworthy, sufficiently noteworthy to merit publication, or to confirm a prior hypothesis. For example, trialists who have a preconception or vested interest in showing that an experimental intervention is beneficial may be inclined to selectively report analyses that are favourable to the experimental intervention. Score probably no if authors do not report both adjusted and unadjusted models.<br><br>Answer ‘Yes’ or ‘Probably Yes’ if:<br>There is clear evidence (usually through examination of a trial protocol or statistical analysis plan) that all reported results for the outcome domain correspond to all intended analyses.<br>or<br>Not all intended analyses have been reported but authors have convincingly justified the reasons for not doing so.<br>or<br>There is only one possible way in which the outcome domain can be analysed (hence there is no opportunity to select from multiple analyses).<br>or<br>Analyses are inconsistent across different reports on the same trial, but the trialists have provided the reason for the inconsistency and it is not related to the nature of the results.<br><br>Answer ‘No information’ if:<br>Analysis intentions are not available, or the analysis intentions are not reported in sufficient detail to enable an assessment, and there is more than one way in which the outcome domain could have been analysed.
6. Reporting bias - Justification	Question 6.3 answer justification	Open answer	Justification for coding decision<br>(Include a brief summary of justification for rating, mentioning your response to all sub questions, cite relevant pages).

For peer review only - http://bmjopen.bmj.com/site/about/guidelines.xhtml

6. Reporting bias - Assessment	Analysis reporting: Was the study free from selective analysis reporting?	(0) Low risk of bias<br>(1) Medium risk of bias/Some concerns<br>(2) High risk of bias	Utliize your answers for questions 6.1 to 6.3 to determine the overall score.
6. Reporting bias - Justification	Analysis reporting justification	Open answer	Justification for coding decision<br>(Include a brief summary of justification for rating, mentioning your response to all sub questions, cite relevant pages).
7. Other bias - Assessment	Other risks of bias Is the study free from other sources of bias?	1= Yes,<br>4 = No	-
7. Other bias - Justification	Other bias justification	Open answer	Justification for coding decision<br>(Include a brief summary of justification for rating, mentioning your response to all sub questions, cite relevant pages). For example, information is collected using a different survey instrument in different intervention groups; measurement of the intervention received in unclear.
8. Blinding - observers - Assessment	Blinding of participants?	1 = Yes<br>2 = No<br>8 = unclear<br>9 = N/A	If there is no information, code NO. If there is information but it is ambiguous, code UNCLEAR.
8. Blinding - analysts - Assessment	Blinding of data analysts?	1 = Yes<br>2 = No<br>8 = unclear<br>9 = N/A	If there is no information, code NO. If there is information but it is ambiguous, code UNCLEAR.
8. Blinding - method(s)	Method(s) used to blind	Open answer (including describe method of placebo control) 9 = N/A	Describe method(s) used to blind
9. External validity - Random Sampling	Was random sampling used?	1 = Yes<br>2 = No	Was a random sampling method used (meaning everyone in the population had an equal chance of being selected for the study - note that this is different than random allocation to treatment versus control group)?<br>Score yes if: (a) there is a sampling frame. The sampling frame is the actual list of individuals that the sample will be drawn from. Ideally, it should include the entire target population (and nobody who is not part of that population). AND<br>(b) probibility sampling is used (e.g., simple random, stratified random, cluster random, etc.)<br>Score no if: individuals are selected based on non-random criteria, and not every individual has a chance of being included (e.g., a convenience sample, a purposive sample, a snowball sample, a voluntary response sample, etc.).

For peer review only - http://bmjopen.bmj.com/site/about/guidelines.xhtml

9. External validity - Random Sampling Justification	Justification for answer to random sampling question	Open answer	Justification for coding decision<br>(Include a brief summary of justification for rating, mentioning your response to all sub questions, cite relevant pages).Describe the sampling technique used.
9. External validity - Assessment	External validity	Open answer	a) What do authors say about external validity? Note any additional information related to generalizability.

For peer review only

Qualitative risk of bias tool

Critical appraisal of qualitative studies tool

This tool provides specific questions you should answer with regards to the study being appraised. Every question has three possible responses, which correspond to 'strong', 'weak', or 'none' – these are phrased slightly differently depending on the question. This process is subjective and relies on a basic level of familiarity with qualitative research methods. However, to further structure the assessment, each question is accompanied by a description of criteria you should consider before making your choice.

While attempting to answer a question, you may find that a study contains many of the relevant elements, but not all. Give this paper the benefit of the doubt – if the element being assessed is strong except for one minor element, choose the 'strong' option.

There is room for comments after each question. You are encouraged to utilize this space, though only the multiple answer response is mandatory. Use the space to document your thoughts if you feel uneasy about the choice made, or if you have any ideas which would help as we refine and revise the assessment tool. There is also room at the end for any additional notes on the paper as a whole.

The questions are arranged according to the order in which a standard academic article is arranged: Introduction, Methodology, Results, Discussion. This will allow you to answer the questions as you go over each section. That said, some documents will not follow this structure, and some academic articles are more fluid about where certain descriptions are located within the text. After you read through the article, go over all the questions once more to see whether any elements you thought were missing actually showed up in an unexpected place.

* Required

Assessor: *

Paper Title: *

Authors: *

Year: *

Introduction

*1. Is the research aim clearly stated? **

Look for this in either the abstract or the introduction. In the best studies, this will be an explicit formulation of the research aims/questions. There may be several. Other studies do not formally state the research aims as such, but they are clearly evident from the text. Note: you might find it useful to write down these research aims, as many of the other questions relate to them.

- Yes, a strong statement
- Yes, but an unclear or weak statement
- No

Question 1 – Notes:

*2. Is there a description of the context in which the study takes place? **

For peer review only - http://bmjopen.bmj.com/site/about/guidelines.xhtml

The study should make some reference to the geographical, temporal, or societal greater context within which the study topic is located. A study within a well-established area of research could likely be situated quite specifically. An exploratory study might cover a topic where there is little descriptive context to draw upon.

- Yes, a strong description
- Yes, but a weak description
- No

Question 2 – Notes:

*3. Is there a clear link to relevant literature? **

Literature should be cited not just in general, but in specific relation to the topic of the paper. In assessing whether the literature is relevant and sufficient, consider: Are most of the cited papers about the same area or topic as the study? Are both qualitative and quantitative studies cited? Are there at least some papers published within the last 10 years?

- Yes, a clear strong link
- Yes, but it could be improved
- No

Question 3 – Notes:

*4. Is there a clear link to theory? **

This can be achieved in many ways, and while it will often be in the introduction, it may show up elsewhere, so keep your eyes peeled. This requires a reference to a specific theory. There are two main ways this could happen: A reference could be made to an established theoretical framework (from the social sciences, from public health, etc.) which has been used to investigate similar questions, or which the authors think might apply in this case. The second possibility is that the authors describe a theoretical framework they have created or adapted themselves which they used in structuring their study, or that they refer to in considering their results.

- Yes, a strong one
- Yes, but a weak one
- No

Question 4 – Notes:

Methodology - Sampling

*5. Is there a description of the sampling procedure? **

Are there details about how sampling was actually conducted? A helpful way to consider this is whether you would be able to conduct this sampling yourself if you were to replicate the study. Every sampling approach has its own logic, but even for the more straightforward qualitative sampling, there should be some mention of this. Note: This

For peer review only - http://bmjopen.bmj.com/site/about/guidelines.xhtml

question does not evaluate whether this sampling is actually appropriate (see question 6). It only asks whether we have a clear idea of the process of sampling, at all.

- Yes, a strong description
- Yes, but a partial description
- No

Question 5 – Notes:

*6. Is the sampling strategy appropriate for the aims of the research? **

This question asks whether the sampling strategy was the right for one for choosing the participants/locations/etc. that could yield relevant information. If there is a strong claim of generalizability, was the sampling appropriate for achieving this? If the study attempts to present the views of a group, did sampling actually capture the people the study needed to hear from? Are there any groups which appear to be missing but clearly should have been spoken to in order to meet the research aims? Has saturation been achieved, and can we tell how this was established? Note that this is not a question of whether the method of collection (interview, focus group, etc.) would have yielded strong data – just whether the people recruited to the study were the right people.

- Yes, very appropriate
- Yes, but not completely appropriate
- No

Question 6 – Notes:

*7. Are sample characteristics sufficiently reported? **

The basic characteristics are often gender, location, ethnicity, and other demographic variables, but consider whether there are other sample characteristics specific to the question which should be reported. This might be clear by this point, but you might also want to look ahead and see whether the paper discusses any relevant characteristics that you would want to know about the sample in general (or you could return to this at the end).

- Yes, there's a sufficient description
- There's some description, but not enough
- No

Question 7 – Notes:

Methodology - Data Collection

*8. Is it clear how data were collected? **

Each method requires its own information. If interviews were used, is there detail on locations, presence of others, length of interview, questions asked, and other such details? For participant observation, is there a description of the

For peer review only - http://bmjopen.bmj.com/site/about/guidelines.xhtml

time spent in the field and the activities partaken in? For focus groups, is there detail on the number of participants, questions asked and setting?

- Yes, data collection is clearly described
- Yes, but more details are needed
- No

Question 8 – Notes:

*9. Are the methods of data recording reported? **

This could be as simple as 'the audio of interviews was taped', 'focus group summaries were written during the discussion', or 'interviews were transcribed, translated, and the translation was then verified'. Any of these would merit at least a 'weak' response, and more detail would be considered 'strong'.

- Yes, and reported well
- Yes, but only briefly mentioned
- No

Question 9 – Notes:

*10. Did the collection of the data address the research aims? **

Given the data needed to answer the question, is the method chosen actually well suited to obtaining such data? If we have knowledge of the questions in an interview guide or a focus group guide, are all the relevant subject areas covered? This isn't about whether there might be some problems in the collection (a bad interviewer could screw up even the best interview guide) but whether the method of data collection, if employed properly, would actually capture relevant data.

- Yes, completely
- Yes, but not entirely
- No

Question 10 – Notes:

Methodology - Analysis

*11. Are the methods of analysis explicitly stated? **

This should include a basic statement as to the approach used (thematic analysis, grounded theory, etc.) but also a breakdown of the steps taken (different rounds of coding, intermittent checks for quality, revisions, etc.)

- Yes, and stated well
- Yes, but more detail is needed
- No

For peer review only - http://bmjopen.bmj.com/site/about/guidelines.xhtml

Question 11 – Notes:

*12. Were there any inbuilt checks to assure the quality of the analysis? **

Some but not all of the following could be present. Was there more than one researcher involved in the analysis? Was there independent peer review during the analysis? Were inter-coder or intra-coder reliability checks conducted? Did the team meet regularly to discuss the analytic process? Did the researcher keep a journal of their thoughts and decisions?

- Yes, there were clear, strong checks throughout
- Yes, but they were weak checks
- No

Question 12 – Notes:

*13. Was there reflection on bias and positionality? **

Have the authors considered their potential biases in relation to the study? This should be a specific statement, and there should be mention of steps that were taken to address this, even if as basic as taking the time to write reflective notes or engaging in a group discussion of the issues.

- Yes, a clear reflection with detail provided
- Yes, but there's only a brief mention of this
- No

Question 13 – Notes:

Methodology - Researchers

14. Are there any details about the people who conducted the sampling, data collection, and analysis?

Not much is required, but look out for mentions of the training these people had, their positions/titles, their experience, etc.

- Yes, plenty
- Yes, a little
- No

Question 14 – Notes:

Results

*15. Is there enough data to support the claims? **

For peer review only - http://bmjopen.bmj.com/site/about/guidelines.xhtml

Variable, but consider whether major claims/themes are supported by more than a single quote or other type of original data. Look at whether the data presented is contextualised, and whether it demonstrates the claim that the authors suggest it does.

- Yes, there's plenty of clear, contextualised, relevant data
- There's some data, but it's weak
- No - there's little or no data presented that is supports the claims.

Question 15 – Notes:

*16. Are diverse viewpoints considered? **

At a basic level, we need to know who speakers are if they are quoted. This could be as minimal as ('22-year-old', or 'Mother, Group A'). Same goes for summaries with no direct quotes – it should be clear whose viewpoint the data comes from. The majority of the data presented will demonstrate the main themes/arguments, but no study is without some dissenting voices or unusual opinions. Are these ever mentioned? If there were many participants, but it appears that most quotes come from one or two individuals, there should be a reasonable explanation for why this is the case – and what it might mean for the study.

- Yes, thoroughly
- Yes, only occasionally
- No

Question 16 – Notes:

*17. Is there evidence which addresses every research aim? **

Look back to the research aims. Is there data relating to each one of them? If not, is there a clear reason for this? If a study ended up not finding data relevant to every aim, but acknowledges that this is the case and tries to account for the reason, this should still be marked as a yes. A 'null' result is still a result - sometimes collection and analysis can be excellent, yet no clear answer is found. If this appears to be the case, the study can still be considered 'strong'.

- Yes, every aim has some related evidence
- Yes, but some aims have distinctly less attending to them
- No

Question 17 – Notes:

Discussion

*18. Has the question been answered? **

A simple test is to go back to the research aims, formulate them as questions (if they're not already established as such) and see whether the authors have provided you with answers that are reasonable given the data presented. It should be clear how the data presented is appropriate and convincing evidence for the conclusion. In a strong paper,

For peer review only - http://bmjopen.bmj.com/site/about/guidelines.xhtml

the authors will make a clear and logical statement of how the results provide an answer to the question. A weaker paper might not cover all the questions, or might suggest questionable links between the data in the results and the conclusions reached.

- Yes, with a clear, logical, thorough answer
- Yes, but not well
- No

Question 18 – Notes:

*19. Are relevant literature, theory, or practice discussed in relation to the results? **

A good paper doesn't need to discuss all of these, but it should relate to at least one. Did it match up or challenge a theory? Does it have implications for practice? Are the results now situated within the broader literature?

- Yes, and discussed well
- Yes, but only briefly or generally discussed
- No

Question 19 – Notes:

*20. Has there been any triangulation? **

This takes many forms, but the basic question is – do the authors present any other sources of data which support or contradict their claims? These could be quantitative/qualitative/mixed studies, some form of evaluation of the conclusions by the participants themselves, comparison to other data recorded, etc. This could be in the results section or the discussion, so have another look.

- Yes, strong triangulation
- Yes, weak triangulation
- No

Question 20 – Notes:

*21. Are weaknesses considered? **

Mentioning them is a good step, but a strong paper will also argue how these weaknesses were attended to.

- Yes, major weaknesses considered and attended to
- Yes, but weaknesses are mentioned without much discussion
- No

Question 21 – Notes:

For peer review only - http://bmjopen.bmj.com/site/about/guidelines.xhtml

Ethics

22. Have ethical issues been taken into consideration? *

At the very basic level, there should be a description of informed consent. Also look for approval from an IRB (or other ethics committee as applicable), and whether issues of benefit/harm and confidentiality/secrecy were discussed with participants.

- Yes
- No

Question 22 – Notes:

General comments and thoughts:

Remember to look back at the questions before submitting.

Having read the paper as a whole, and having considered each question separately, you might now also have a clearer idea as to the answers to some questions. Have another look, particularly for questions where you noted some thoughts or concerns. You may want return to question 7 in particular, as you may now have a clearer idea of what sample characteristics should have been reported.

For peer review only

Cost evidence risk of bias tool

S.No.	Questions	Response
1a.	Is the form of economic evaluation clearly stated? Indicate (CBA/CEA/CUA/cost-minimization, or innovative approaches: Social Return on Investment (SROI), Multi-Criteria Appraisal (MCA). Response options [CBA, CEA, CUA, SROI, MCA, or N/A if only "total cost", also ok to insert descriptive results that do not conform, e.g. "cost transfer ratio"]	Yes, No
1b.	Is the perspective of the costing stated?	Yes, No
1d.	Cost data sources?	Yes, No
1e.1	Are unit costs reported (in Table)	Yes, No
1e.2	Are cost ingredients listed?	Yes, No
1e.3	Total cost description	Yes, No
1f.	Total cost reported?	not reported, Value if reported
1h.	Was cost per child immunised reported?	Yes, No, If yes, Cost
1j.	What exchange rate was used for currency adjustments? [e.g. 1.2 USD to 1 Euro]	value if given, Not reported
1j.	Exch rate year (estimated)	Year
1k.	Is time horizon for costs clearly stated? [Operationalise - was a base year of the costing reported?	Yes, No, If yes: year
7a.	Sensitivity analysis? [yes/ no]	n/a, Description if yes

For peer review only - http://bmjopen.bmj.com/site/about/guidelines.xhtml

Appendix 8: Risk of bias in the included studies

Quantitative risk of bias

Risk of bias in Randomised Controlled Trials

Of the included studies with experimental designs (k = 31) the majority (k = 23) have been identified as being at high risk of bias, and only two were rated as having a low risk of bias. Six studies were assessed as having some concerns. Of the six potential causes of bias that were analysed, outcome measurement bias and deviations from intended interventions were the most commonly documented issues. The most common pitfall related to outcome measurement bias was the use of caregiver reported (self-reported) measures of vaccinations received by a child in the absence and/or incompleteness of immunisation cards, which is common in L&MICs and is often influenced by the intervention itself. One such study stated that "The calculation of FIC coverage was based on data from the child's immunization card and/or the mother's recall. Mother's recall was taken in situations in which the card or specific data points on the card were missing.", (Gurley 2020). Relatedly, Demilew, (2021) reported that their outcome measures "...could be subject to recall bias or social desirability bias differentially by treatment group…". Deviations from intended interventions had to do with issues such as potential spillover related to geography (e.g. in Siddiqi 2020, centers were contiguously located) or transferable vouchers (e.g. Morris 2004). Other sources of bias in included RCT studies are reporting bias, and bias in the randomisation process. Supplementary Figure 2 illustrates the frequencies with which each cause of bias was identified across the 31 included RCTs.

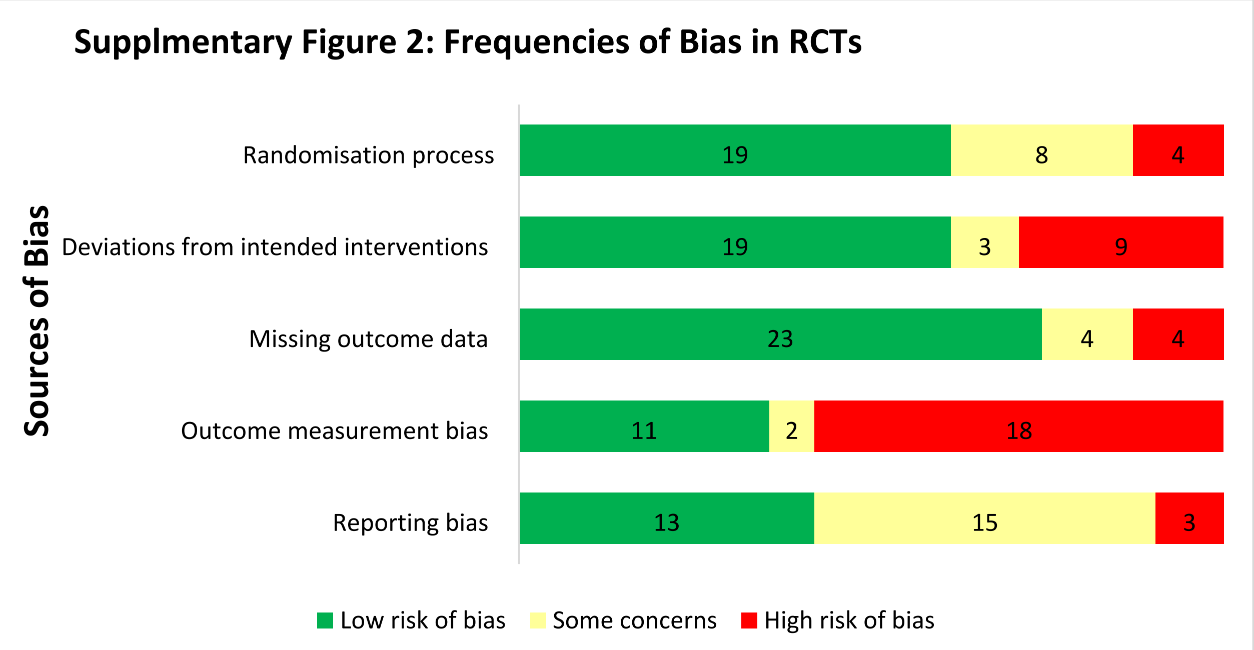

Bias in Quasi-Experimental Designs

Of the 30 included quasi-experimental studies, the majority (k = 27) present a high risk for bias, two were assessed as low risk of bias and one study was assessed as having some concerns. Like the experimental studies the most common identified causes of bias amongst quasi-experimental designs were issues related to outcome measurement bias and deviations from intended interventions. This is no surprise as self-reporting was again a common concern (e.g. Admassie, 2009 and Findley, 2013). Supplementary Figure 3 illustrates the frequencies of other biases in our included QEDs.

For peer review only - http://bmjopen.bmj.com/site/about/guidelines.xhtml

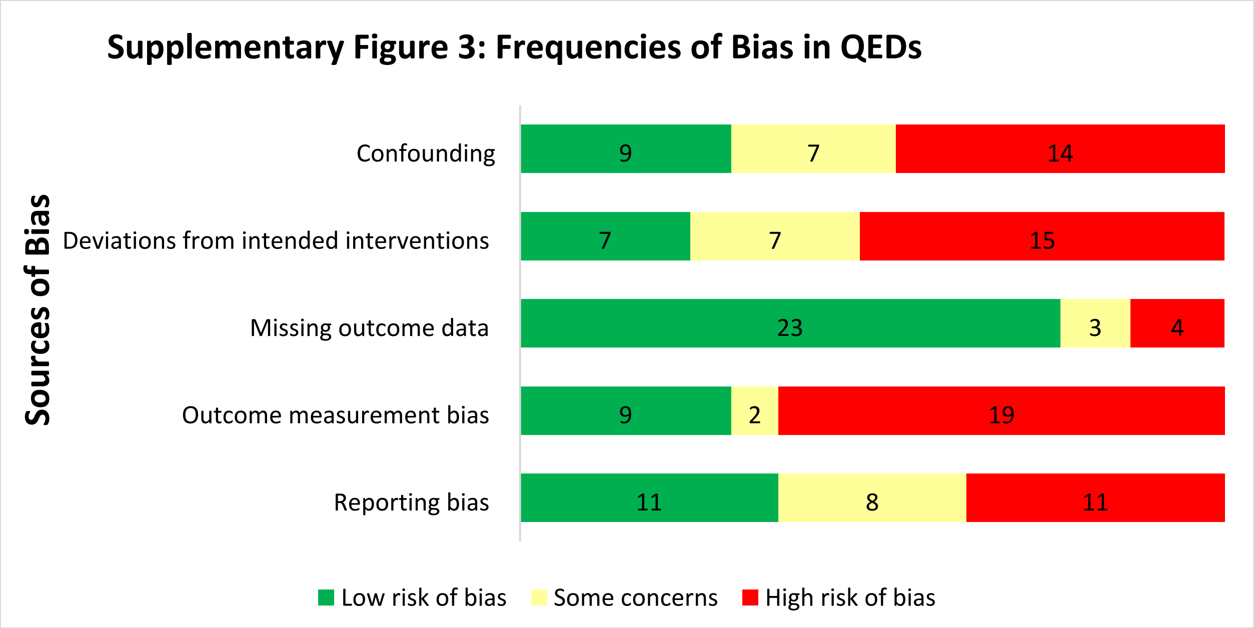

Qualitative risk of bias

Risk of bias assessments were conducted on 47 qualitative papers. Papers were scored as absent, weak, or present on 12 key elements and received corresponding scores of zero, one, or two for these ratings. Most (27) papers were missing at least some key elements, resulting in them receiving a zero value for these elements in their quality assessment scores (Supplementary Table 1; Supplementary Figure 4). However, 17 received quality assessment scores over 20, indicating that they received a value of two, or a "strong" rating, for most key elements (Supplementary Figure 5). The most common key elements to be missing were descriptions of sample characteristics and the analytic methods, with 17 studies failing to report on each of these (Supplementary Figure 5). The research aim was the most common key element to be stated strongly, with 39 papers clearly stating their aims.

Supplementary Table 1: Distribution of papers by strength of key elements

	Number
One or more key elements absent	27
One or more key elements weak	15
All key elements strong	5

For peer review only - http://bmjopen.bmj.com/site/about/guidelines.xhtml

Supplementary Figure 4: Distribution of papers by 12 key elements

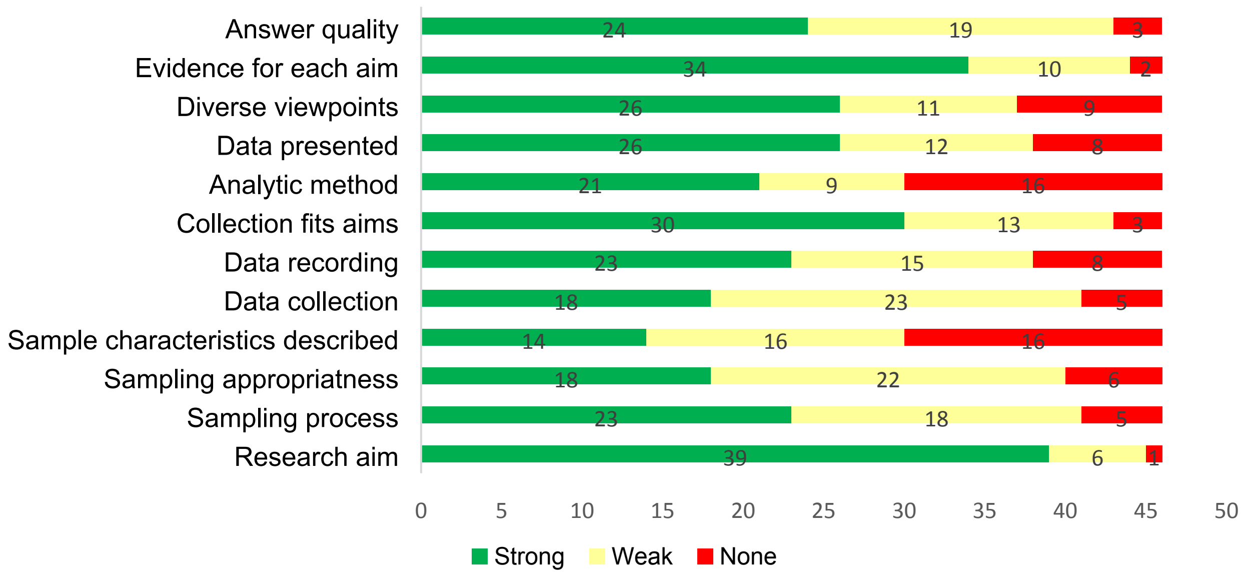

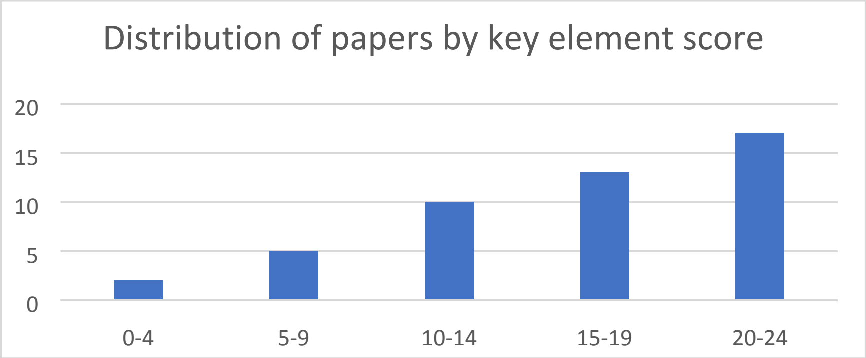

*Papers received scores of 0, 1 or 2 reflecting the strength of reporting on 12 key elements in the risk of bias assessment tool (Appendix E).

Supplementary Figure 5: Distribution of qualitative assessment scores*

Risk of bias in cost effectiveness estimates

The risk of bias analysis of the cost and cost-effectiveness analysis assessed the quality of underlying cost data, reporting, and analysis using information that was reported in the immunization studies. We assessed risk of bias along six primary dimensions which were adapted from a combination of tools, including: Doocy and Tappis (2017); Campbell Collaboration Economic Methods Policy Brief (Shemilt et al.,2008); and Methods for the Economic Evaluation of Health Care Programmes, (Drummond et al. 2015), that were adapted for cost analyses carried out in conjunction with impact evaluation studies of global development interventions.

This risk of bias tool specifically assesses the bias that arises from the collection and reporting of cost analysis in conjunction with impact evaluation studies of global development interventions. These studies often do not incorporate cost analyses. Indeed recent estimates suggest just 15-18% of impact analyses include any kind of cost analysis (Brown and Tanner 2019). This under-reporting of cost in conjunction with impact evaluations leads to very

For peer review only - http://bmjopen.bmj.com/site/about/guidelines.xhtml

small samples from which to draw inferences about the cost, and cost-effectiveness of development interventions. Moreover, often when estimates are included with impact evaluations, the quality of the underlying data is low. Cost may have been added as an "after-thought", rather than planned for in advance or treated as a research endeavor. Data sources for cost information may not be well-documented or may have been estimated using 'back-of-the-envelope' techniques (this phrase literally is used in write-ups of cost methods). There is often insufficient detail in reporting of cost to assess the quality of estimates and data and basic robustness checks of the analysis are very infrequently performed.

Finally, this tool specifically considers the elements of cost and effectiveness that were extracted for the analysis of incremental cost effectiveness. Our particular measure estimates the non-vaccine cost per dose of interventions to increase absolute immunization coverage by one percent, using available data extracted from the evaluations wherever possible, or by following the steps and calculations as outlined in Ozawa et al. (2018). The specific inputs to this analysis include estimates of: total intervention cost (net of vaccine cost); the number of vaccine doses provided to each child; the endline proportion of children that received immunisations in the treatment and control groups; and the cost per child immunised.

Dimensions of potential bias in costs reported and cost-effectiveness estimates

Planned, organized, cost analysis. A common challenge of cost analysis is a lack of planning which often can lead to poor underlying data quality. For example, cost estimates are subject to recall bias when assessed long after the program and evaluation are completed. Therefore, we examine three indicators of an organized or planned cost analysis. Specifically, we look for a clear description of the form of economic evaluation, and a description of the method used. We also look for a clear statement of analytical perspective--which is the choice of which actor has standing in the costing -- this may be the donor, the implementing partner, or perspective of the costing may be from a societal point of view. The analytical perspective is important because it determines whose costs and benefits will be counted in the costing. It may, for example, directly impact how the constituent components of total cost are counted. Although we considered a standard ROB question which asks: 'Is a well-defined research question posed in answerable form?', we find that impact evaluation studies do not pose research questions in the expected form. The three questions, given equal weight in the risk of bias assessment, were:

a. Is the form of economic evaluation clearly stated?
b. Is the perspective of the costing stated?
c. Was a method of costing described?

Quality data sources. The highest quality data for assessing the cost of interventions in low-and middle-income settings often are drawn from the expenditure reports or accounting statements of the program implementers. Expenditures are better quality because they represent actual, rather than planned expenses and they often are subject to audit, making them more reliable as compared with cost data taken from program budgets (Levin et al. 2018).

d. What is the quality of the primary data sources used for the cost estimates?

Descriptive, detailed cost information. The quality of descriptive detail on costs allows judgement into cost components and whether they align with intervention activities. Since the three criteria tend to be positively correlated, we give equal weight to the three indicators of detailed cost information. Specifically, we assessed the descriptive detail of reported costs:

e. Whether costs are reported by ingredients (or input or resource),
f. Whether unit costs are reported, and
g. Whether the information is presented in an organized cost table

We consider the **quality of cost estimates** that were key inputs to the cost synthesis, specifically

For peer review only - http://bmjopen.bmj.com/site/about/guidelines.xhtml

h. Total cost: What is the quality of the specific data components of total cost? Since total cost is a key input to the analysis we examine how it was reported. For example, some studies say simply, the total cost of the program was USD $3 Million. In the absence of descriptive information, i.e. the elements that comprise total cost, it is difficult to assess the quality or reliability of the total cost estimate.

i. Vaccine cost: Can we tell if vaccines were excluded? Since we need to exclude vaccine cost, it must be excluded or reported separately from total cost to be valid in this study.

j. Cost per child immunized: If cost per child immunized was not directly estimated and reported, we take total cost divided by the number of children in the treatment group multiplied by the proportion of children that were immunized. If the quality of underlying data are poor, this estimate may be biased – with unclear magnitude and direction.

k. Number of vaccines. Was the number of vaccines reported from observation of study participants, or was it reported per protocol? When we estimate cost per vaccine dose, the cost estimate will be biased downwards (less costly per dose) if the study does not report the actual number of doses administered. This is because some children may already have received a part of the vaccine protocol before enrolling in the study.

Are **key cost details** reported which allow us to adjust for time and currency differences? In this assessment of bias risk, the responses are given equal weight since we need both elements to accurately adjust for time and currency differences.

l. Are details of inflation and currency conversion clearly stated?

m. Is the time horizon for costs clearly stated?

We consider the **quality of cost analysis**, specifically:

n. Is sensitivity analysis conducted?

Results

We developed evaluation criteria and applied the protocol described above to the 22 immunisation evaluations that reported cost information. The results of this analysis are summarized below in Supplementary Figure 6, below.

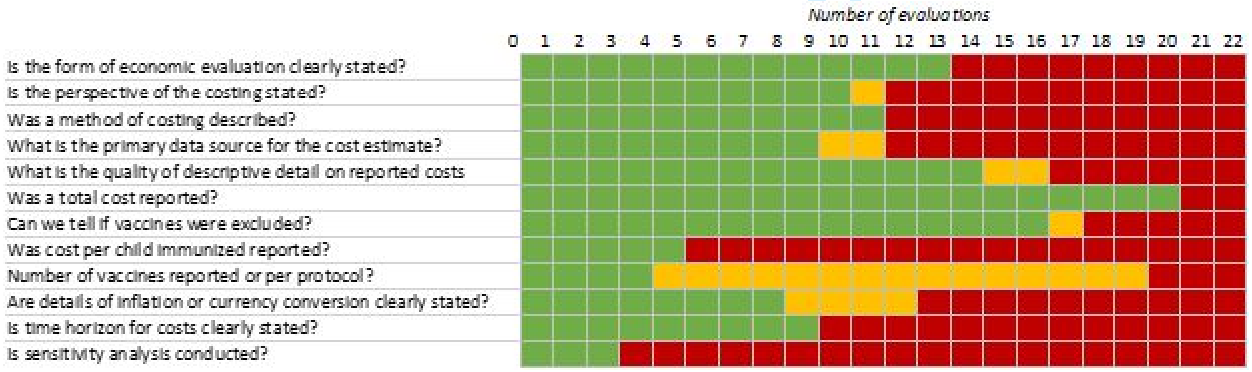

Note: green cells indicate that clear information was presented in the report to assess the risk of bias; yellow cells indicate incomplete information; and red cell indicate we found very little or no information to assess bias risk.

Supplementary Figure 6: Risk of bias assessment of cost and cost-effectiveness estimates used in the analysis

For peer review only - http://bmjopen.bmj.com/site/about/guidelines.xhtml

- *Just over half of the included evaluations appear to have carried out a planned, organized, cost analysis.* Thirteen of 22 evaluations clearly state the form of economic evaluation (i.e. cost-effectiveness analysis); ten studies report the perspective of the costing, which is key for judging the correct inclusion and exclusion criteria for the components of a total cost estimate; and eleven of 22 evaluations describe the method of costing that was used to collect cost data (i.e. the ingredients method).
- *Indications of the quality of underlying cost data are mixed.* Nine of the 22 evaluations used expenditure reports to generate cost estimates, two used budgets and the remaining 11 evaluations provided no information on the provenience of the underlying cost data that was used in the analysis.
- *The quality of the descriptive detail on reported costs was mixed. Just over half of all evaluations (14 of 22)* provided thorough, descriptive information on costs; two evaluations provided some descriptive information, e.g. a breakdown of key unit costs; and six evaluations gave very minimal or no descriptive information on costs.
- *In 17 of 22 evaluations, we have high confidence that total cost excludes vaccines costs.* A majority of evaluations reported an estimate of total cost (20 of 22 evaluations). In 16 of the 20 total cost estimates, there were clear indications that vaccine costs had been excluded from the estimate, and in one case, vaccine costs were included but reported separately so that we could subtract vaccine cost from total cost to derive the comparable total cost. It was not possible to tell if vaccines were excluded from the total cost estimates of five evaluations.
- *The cost per immunized child* – was only reported by the authors in five of 22 evaluations and for the remaining 16 was estimated by the authors to compile Supplementary Table 22.
- *The number of vaccine doses received per treated child was reported in only four evaluations;* in 15 studies, vaccine doses per child were estimated based on information "per protocol" which does not account for children's partial vaccination status at the point of enrollment in the study.
- *Fewer than half of the evaluations included the key information needed to adjust for time, currency, inflation or base year differences in the timing of expenditures.* This lack of reporting makes it very difficult to have complete confidence in the comparability of the cost estimates we generated.
- *Only three evaluations reported any kind of sensitivity analysis*, an indication of analytical robustness.

For peer review only

Appendix 9: Quantitative results – all community engagement interventions

Appendix A presents supplementary data for analyses presented in the main body of the manuscript for the primary outcomes of community engagement interventions, including funnel plots of publication bias and outlier analyses. It also includes a full presentation of the supplementary outcomes that were precluded from the main body of the manuscript due to word limitations.

Primary outcomes

Full immunisation

As a robustness check, we used robust variance analysis and included all dependent effects in the analysis, totaling 53 effects from the same 28 studies (*df* = 25.5). The overall average effect was slightly smaller but still significant ($\hat{\mu} = 0.11$ [95% CI: 0.04 to 0.18], p = .002). Sensitivity analyses show the effect to be sensitive to all values of Rho. The rank correlation test indicated funnel plot asymmetry ($p = 0.03$) but the regression test did not ($p = 0.57$; see Supplementary Figure 7). A trim and fill analysis indicated an identical effect size (see Supplementary Figure 7b)

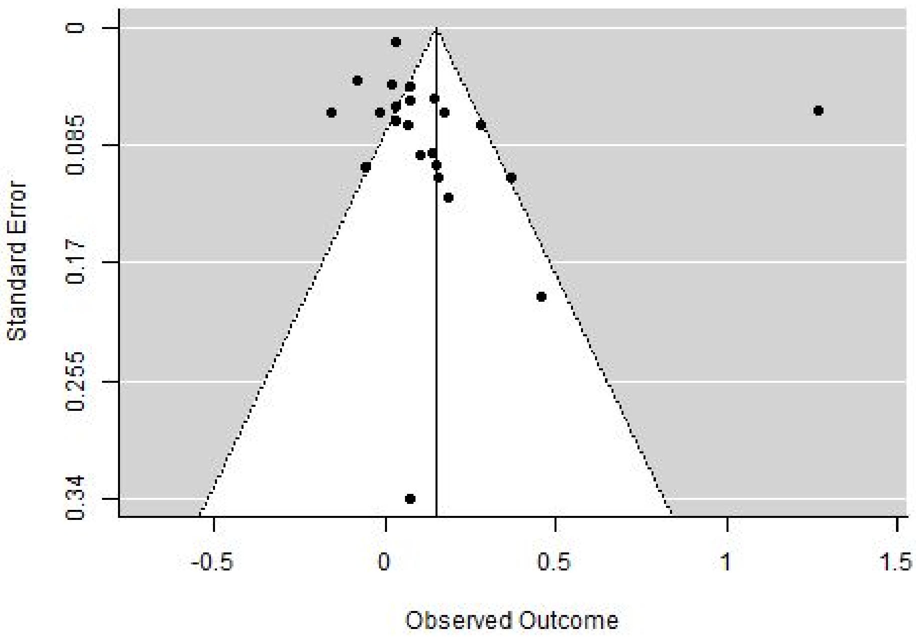

Supplementary Figure 7. Funnel plot for studies examining the effect of community engagement interventions on full immunisation

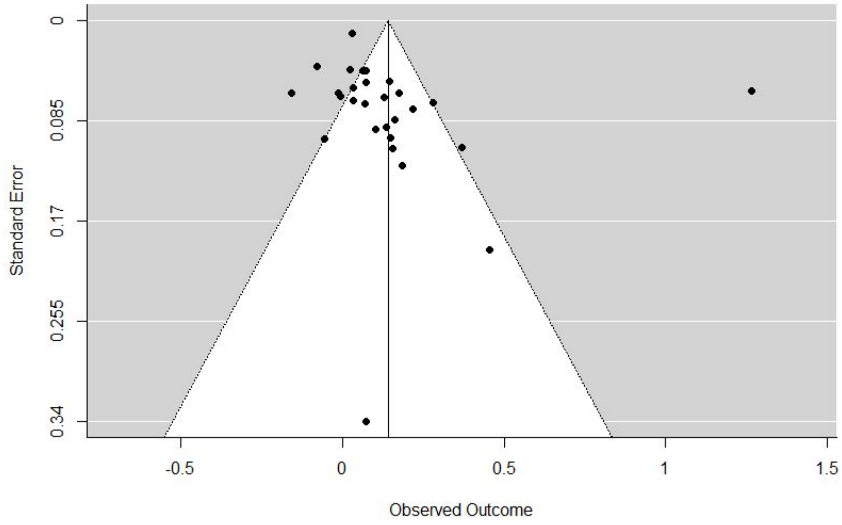

Supplementary Figure 7b. Trim and fill plot for studies examining the effect of community engagement interventions on full immunisation

DPT3

According to the Q-test, the true outcomes appear to be heterogeneous ($Q(21) = 90.43$, $p < 0.01$, $\hat{\tau}^2 = 0.01$, $I^2 = 76.78\%$). The rank correlation test indicated funnel plot asymmetry ($p = 0.04$) but not the regression test ($p = 0.06$), but trim and fill analyses indicated an identical effect size (Supplementary Figures 8 & 9). An examination of the studentized residuals revealed that none of the studies had a value larger than ± 3.05 and hence there was no indication of outliers in the context of this model. According to the Cook's distances, none of the studies could be considered to be overly influential.

Publication year was a significant moderator such that each additional year reduced the size of the effect by .014 standard deviation units ($\hat{B} = -0.014, p = 0.019$ [95% CI: -0.03 to -0.002]). In other words, new studies have found smaller effects. There were no other significant moderators in the context of this model.

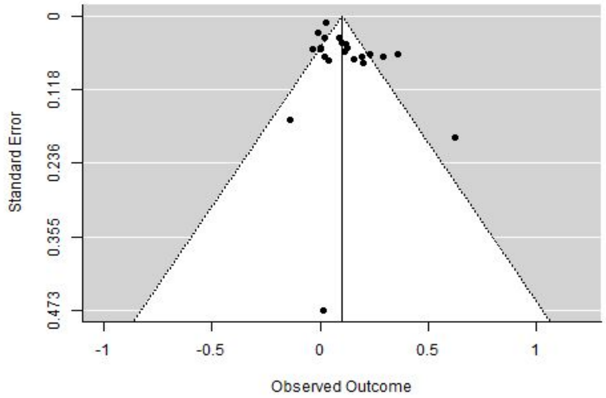

Supplementary Figure 8. Funnel plot for studies examining the effect of community engagement interventions on DPT3 vaccination

For peer review only - http://bmjopen.bmj.com/site/about/guidelines.xhtml

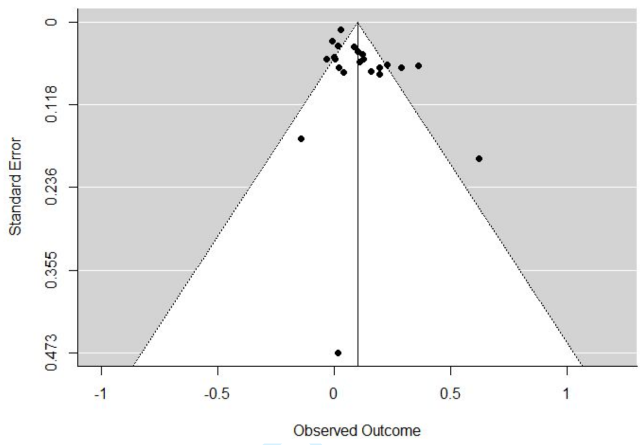

Supplementary Figure 9. Trim and fill plot for studies examining the effect of community engagement interventions on DPT3 vaccination

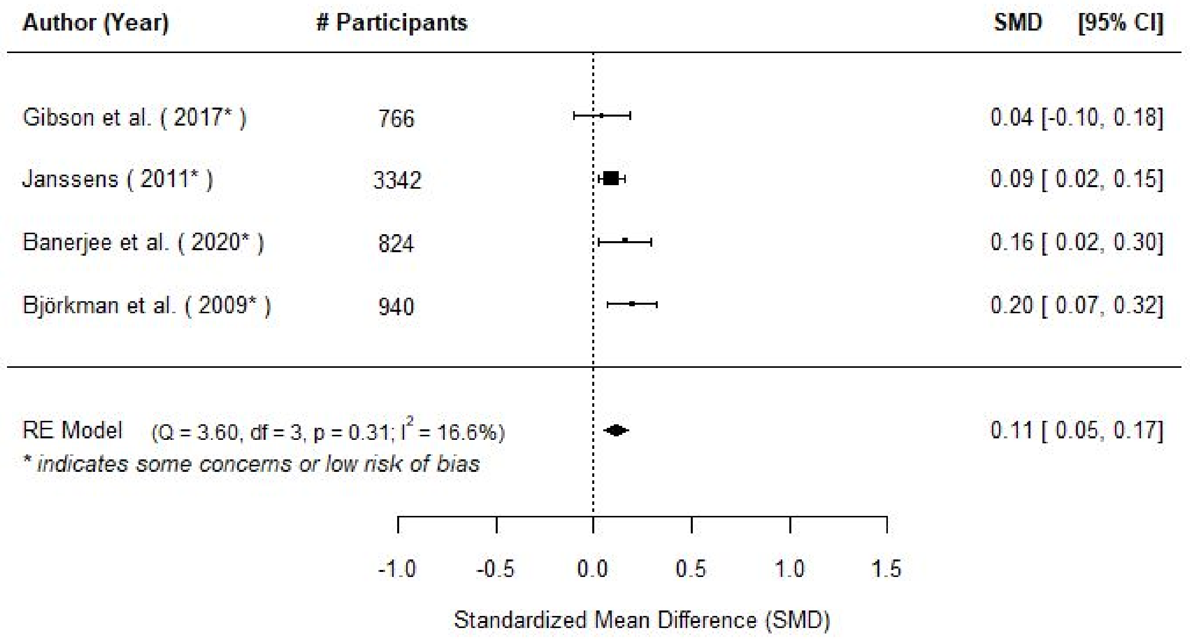

Supplementary Figure 10. *Forest plot showing the observed outcomes and the estimate of the random-effects model for the impact of community engagement interventions on DPT3 vaccination when low quality studies are removed*

As a robustness check, we used robust variance analysis and included all dependent effects in the analysis, totaling 36 effects from the same 22 studies (*df* = 18.2). The overall average effect was slightly smaller but still significant ($\hat{\mu} = 0.10$ [95% CI: 0.05 to 0.15], $p < .001$). Sensitivity analyses show the effect to be sensitive to all values of Rho.

Measles

An examination of the studentized residuals revealed that one study (Sankar 2013) had a value larger than ± 3.02 and may be a potential outlier in the context of this model. Indeed, sensitivity analysis leaving out Sankar (2013) would result in an increase in the average effect ($\hat{\mu} = 0.08$ [95% CI: 0.05 to 0.12], and it was still statistically

For peer review only - http://bmjopen.bmj.com/site/about/guidelines.xhtml

significant ($z = 4.44$ $p < 0.001$). According to the Cook's distances, none of the studies could be considered to be overly influential. A funnel plot of the estimates is shown in Figure A6. Both the rank correlation and the regression test indicated potential funnel plot asymmetry ($p = 0.05$ and $p = 0.02$, respectively). The trim and fill analysis (Supplementary Figure 13) estimates a smaller and non-significant effect ($\hat{\mu} = 0.02$ [95% CI: -0.02 to 0.06], $p = .36$ Supplementary Figure 11 presents the results when low quality studies are removed.

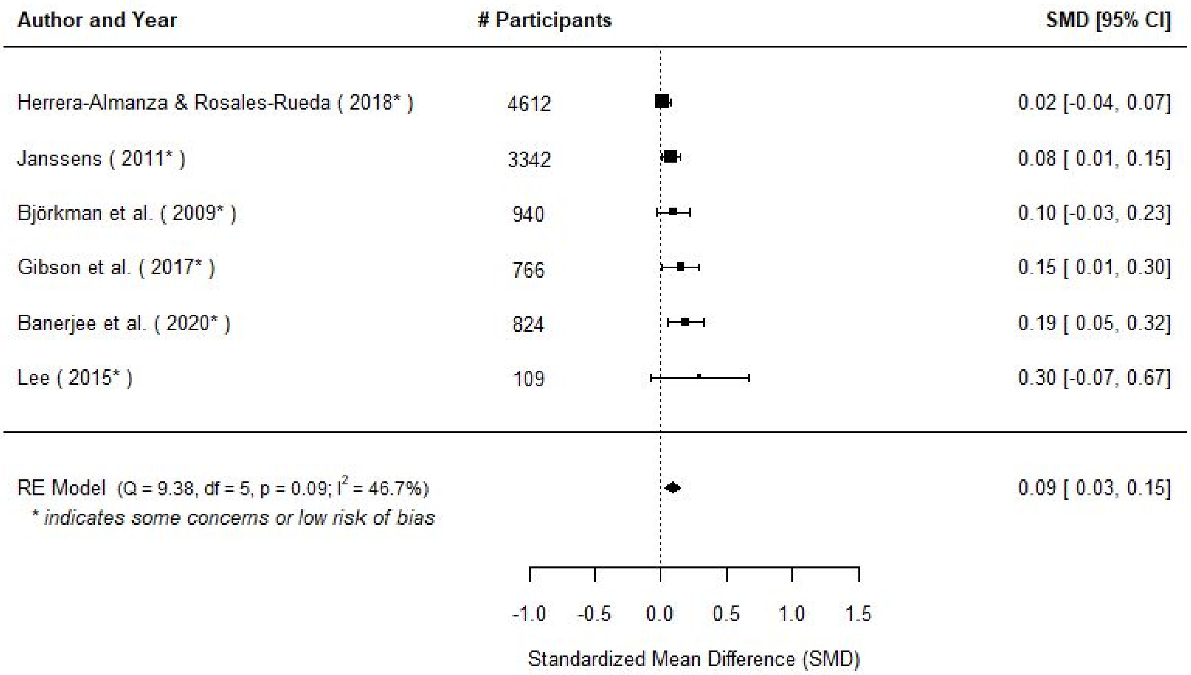

Supplementary Figure 11. Forest plot showing the observed outcomes and the estimate of the random-effects model for the impact of community engagement interventions on measles vaccination when low quality studies are removed

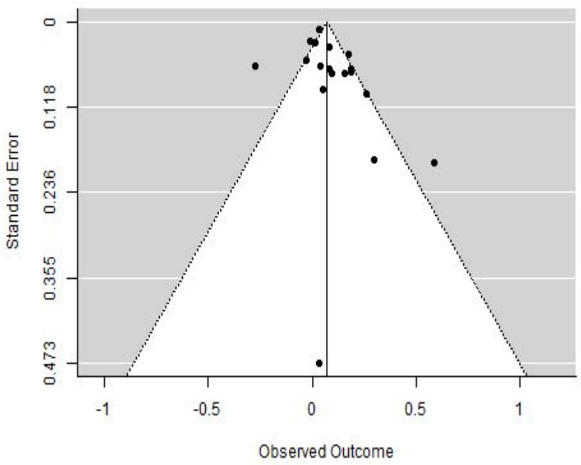

Supplementary Figure 12. Funnel plot for studies examining the effect of community engagement interventions on measles vaccination

For peer review only - http://bmjopen.bmj.com/site/about/guidelines.xhtml

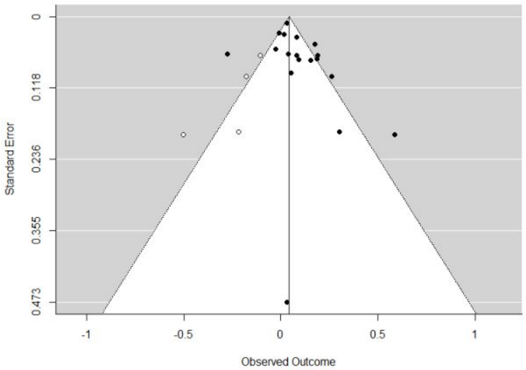

Supplementary Figure 13. Trim and fill funnel plot for studies examining the effect of community engagement interventions on measles vaccination

As a robustness check, we used robust variance analysis and included all dependent effects in the analysis, totaling 32 effects from the same 20 studies (*df* = 15.5). The overall average effect was slightly smaller but still significant ($\hat{\mu}$ $= 0.06$ [95% CI: 0.01 to 0.11], p = .03). Sensitivity analyses show the effect to be sensitive to all values of Rho.

Vaccination timeliness: Full immunisation schedule

We included a total of $k = 5$ studies in the analysis. The estimated average outcome was $\hat{\mu} = 0.15$ ([95% CI: 0.07 to 0.24], $z = 3.41$, $p < 0.001$; $Supplemenyary\ Figure$ 14), indicating a small but significant benefit to the treated group compared to the control group. According to the Q-test, there was no significant amount of heterogeneity in the true outcomes ($Q(4) = 4.43$, $p = 0.35$, $\hat{\tau}^2 = 0.00$, $I^2 = 9.66\%$). An examination of the studentized residuals revealed that none of the studies had a value larger than ± 2.58 and hence there was no indication of outliers in the context of this model. According to the Cook's distances, none of the studies could be considered to be overly influential. With only one study not assessed as high risk of bias, we were unable to conduct sensitivity analyses by study quality. With no significant heterogeneity, we did not test for moderations. There were no dependent effects, so we could not use RVE as a robustness check in this case.

For peer review only - http://bmjopen.bmj.com/site/about/guidelines.xhtml

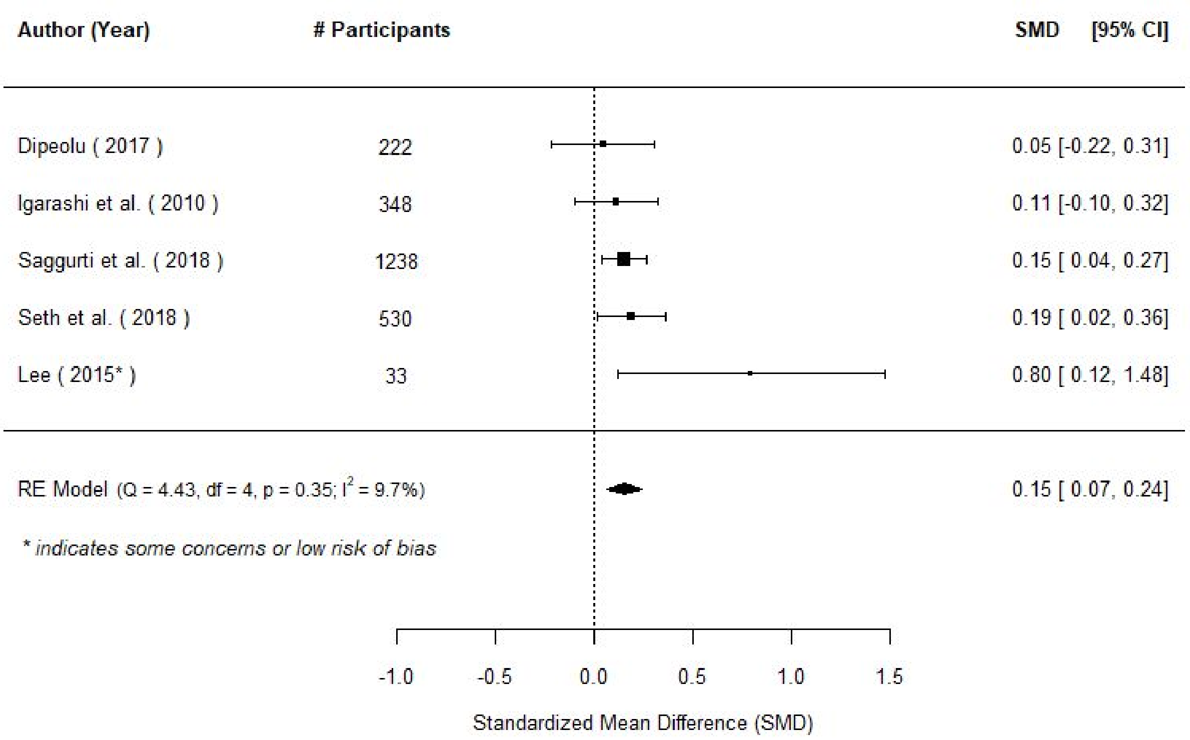

Supplementary Figure 14. Forest plot showing the observed outcomes and the estimate of the random-effects model for the impact of community engagement interventions on timeliness of full childhood immunisation

Vaccination timeliness: DPT3

A total of $k = 7$ studies examined the relationship between community engagement interventions and timeliness of DPT3 vaccination. The estimated average outcome was $\hat{\mu} = 0.09$ ([95% CI: 0.03 to 0.14], $z = 3.00$, $p < 0.01$), indicating a small but significant benefit to the intervention group compared to the control group (Supplementray Figure 15). According to the Q-test, there was no significant amount of heterogeneity in the true outcomes ($Q(6) = 5.09$, $p = 0.53$, $\hat{\tau}^2 = 0.00$, $I^2 = 0.00\%$). An examination of the studentized residuals revealed that none of the studies had a value larger than ± 2.69 and hence there was no indication of outliers in the context of this model. According to the Cook's distances, none of the studies could be considered to be overly influential. With no heterogeneity, moderator analyses were not appropriate. There were no dependent effects, so we could not use RVE as a robustness check in this case. When low quality studies are removed, only two studies remain, and the estimated average outcome increases to $\hat{\mu} = 0.11$ [95% CI: -0.05 to 0.27], but becomes non-significant $z = 1.32$, $p = 0.19$). However, with only two studies this should be interpreted with caution.

For peer review only - http://bmjopen.bmj.com/site/about/guidelines.xhtml

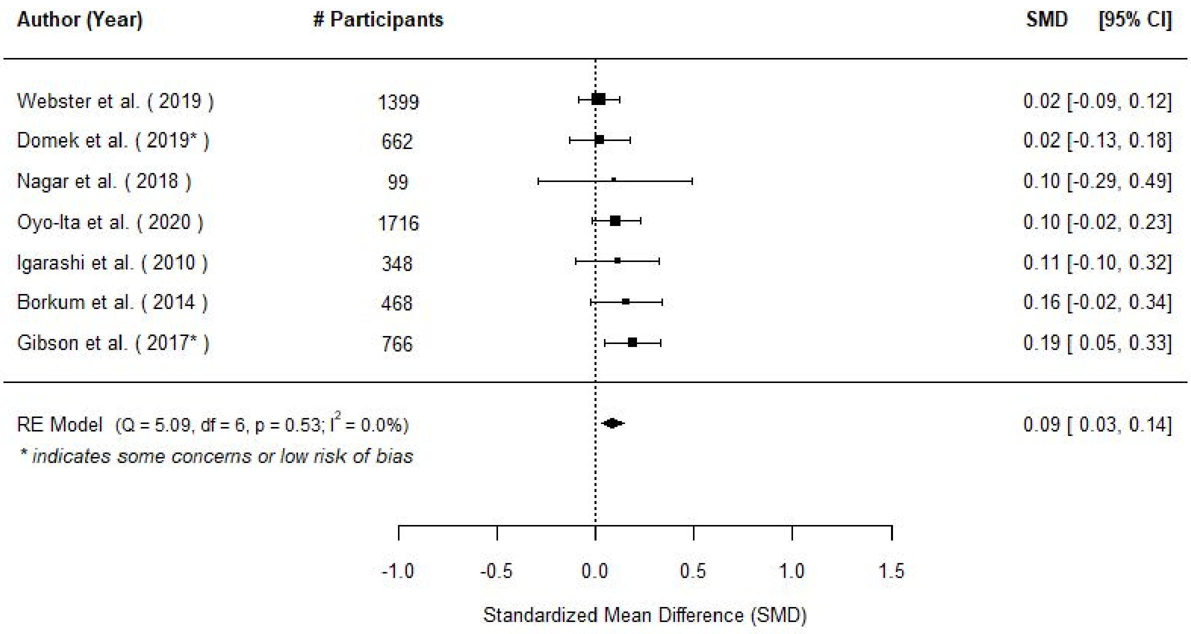

Supplementary Figure 15. *Forest plot showing the observed outcomes and the estimate of the random-effects model for the impact of community engagement interventions on the timeliness of DPT3 vaccination.*

Vaccination timeliness: Measles

Only $k = 2$ studies using community engagement interventions reported on the timeliness of measles vaccinations. The estimated average outcome was $\hat{\mu} = 0.23$ ([95% CI: 0.14 to 0.32], $z = 5.06$, $p < 0.001$;*see* Supplementary $Figure$ 16), indicating a small but significant benefit to the treated group compared to the control group. According to the Q-test, there was no significant amount of heterogeneity in the true outcomes ($Q(1) = 0.23.$, $p = 0.63$, $\hat{\tau}^2 = 0.00$, $I^2 = 0.00\%$). An examination of the studentized residuals revealed that none of the studies had a value larger than ± 2.69 and hence there was no indication of outliers in the context of this model. According to the Cook's distances, none of the studies could be considered to be overly influential. With no heterogeneity and only two studies contributing effects, moderator analyses were not appropriate and we were unable to conduct sensitivity analyses by study quality or test for publication bias. There were no dependent effects, so we could not use RVE as a robustness check in this case.

For peer review only - http://bmjopen.bmj.com/site/about/guidelines.xhtml

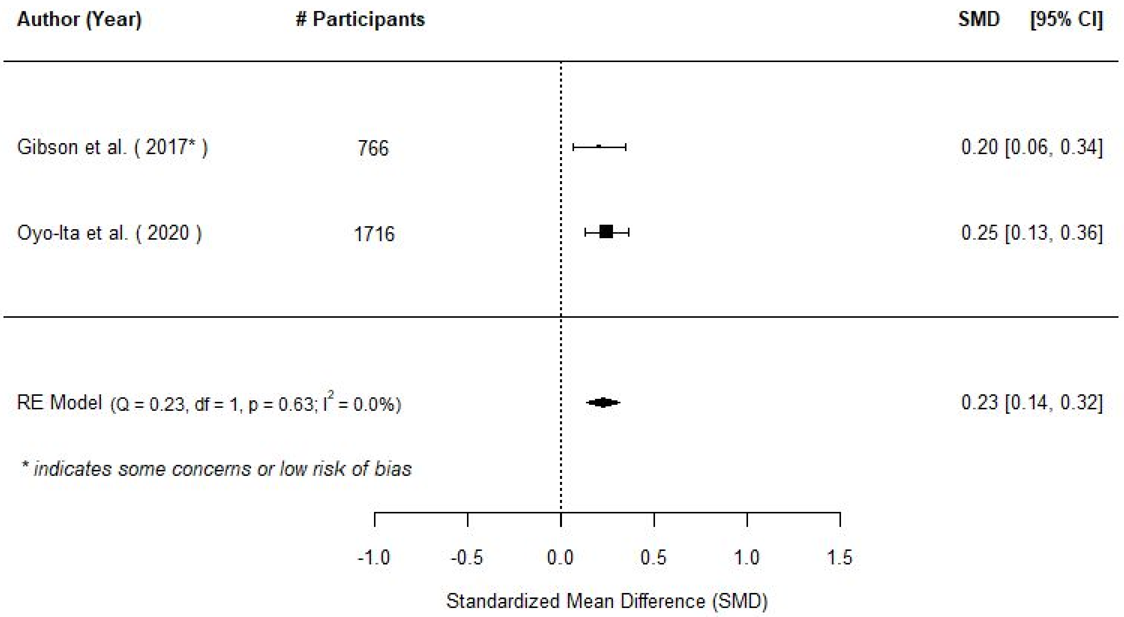

Supplementary Figure 16. *Forest plot showing the observed outcomes and the estimate of the random-effects model for the impact of community engagement interventions on the timeliness of measles vaccination.*

Secondary outcomes

Partial immunisation

We included a total of $k = 9$ studies the analysis. The estimated average outcome based on the random-effects model was $\hat{\mu} = 0.23$ ([95% CI: 0.09 to 0.37], $z = 3.15$, $p = 0.002$), indicating a benefit for the intervention group compared to the control group (see Supplementary Figure 17).

According to the Q-test, the true outcomes appear to be heterogeneous ($Q(8) = 219.11$, $p < 0.001$, $\hat{\tau}^2 = 0.04$, $I^2 = 96.351\%$). An examination of the studentized residuals revealed that none of the studies had a value larger than ± 2.77 and hence there was no indication of outliers in the context of this model. Likewise, according to the Cook's distances, none of the studies could be considered to be overly influential. With eight out of the nine studies being assessed as high risk of bias, we were unable to conduct sensitivity analysis by study quality.

For peer review only - http://bmjopen.bmj.com/site/about/guidelines.xhtml

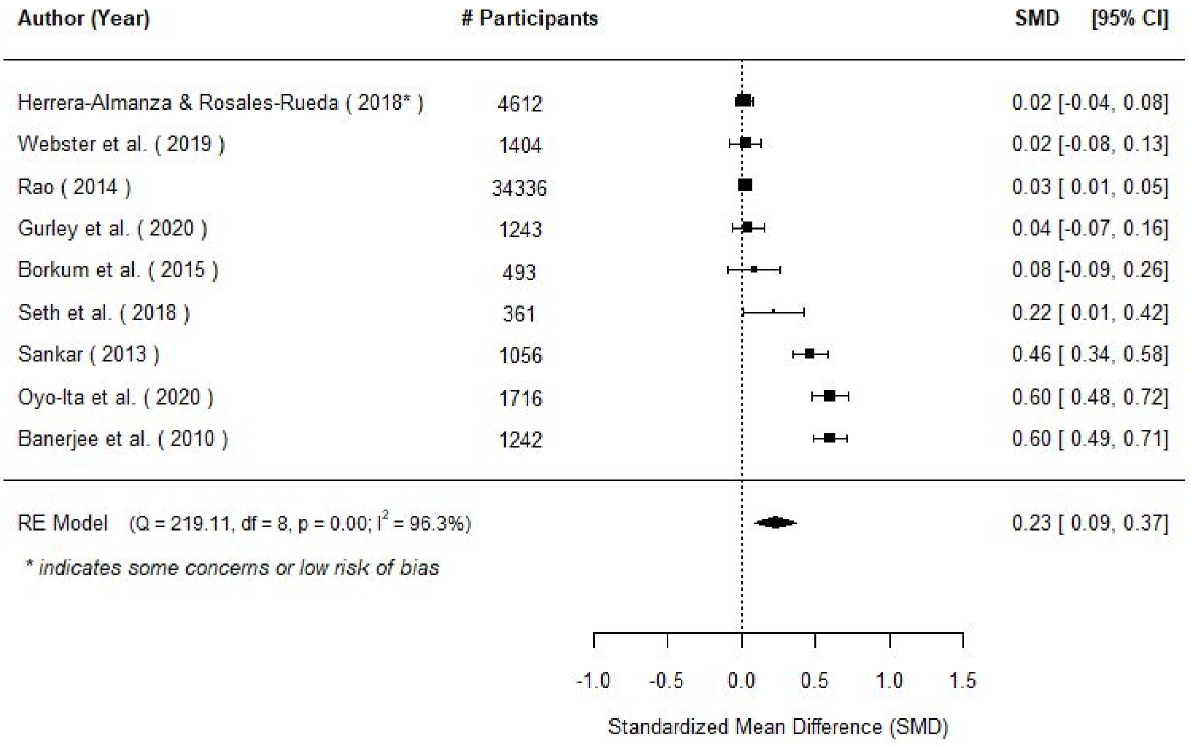

Supplementary Figure 17. Forest plot showing the observed outcomes and the estimate of the random-effects model for the impact of community engagement interventions on partial childhood immunisation

As a robustness check, we used robust variance analysis and included all dependent effects in the analysis, totaling 13 effects from the same 9 studies (df = 7.96). The overall average effect was slightly smaller but still significant ($\hat{\mu}$ = 0.21 [95% CI: 0.03 to 0.38], p = .03). Sensitivity analyses show the effect to be sensitive to all values of Rho.

BCG

We included a total of $\mathrm{k} = 12$ studies in the analysis. The estimated average outcome based on the random-effects model was $\hat{\mu} = 0.06$ ([95% CI: 0.01 to 0.11], $z = 2.28$, $p = 0.02$), indicating a very small but significant benefit to the intervention participants compared to the control participants (see Supplementary Figure 18). According to the Q -test, the true outcomes appear to be heterogeneous ($Q(11) = 84.22$, $p < 0.01$, $\hat{\tau}^2 = 0.00$, $I^2 = 86.94\%$). An examination of the studentized residuals revealed that one study (Banerjee 2010) had a value larger than ± 2.87 and may be a potential outlier in the context of this model. According to the Cook's distances, Banerjee (2010) could also be considered to be overly influential. Sensitivity analyses leaving each study out indicated that removing Banerjee (2010) would reduce the overall average effect ($\hat{\mu} = 0.02$ (95% CI: -0.005 to 0.04), and the resulting effect would be non-significant ($z = 1.51$, $p = 0.13$). Moderator analysis revealed that publication year was a significant predictor such that more recent studies find smaller effects that older studies, with each additional year reducing the size of the effect by .03 standard deviation units ($\beta = -0.03$ [95% CI: -0.05 to -0.001], p = .04). No other moderators were significant (see Supplementary Table 3). Only three studies were high or moderate quality. When those three studies were synthesised, the resulting effect was reduced ($\hat{\mu} = 0.01$ [95% CI: -0.08 to 0.10]) and no longer significant ($z = 0.22$, $p = 0.82$).

For peer review only - http://bmjopen.bmj.com/site/about/guidelines.xhtml

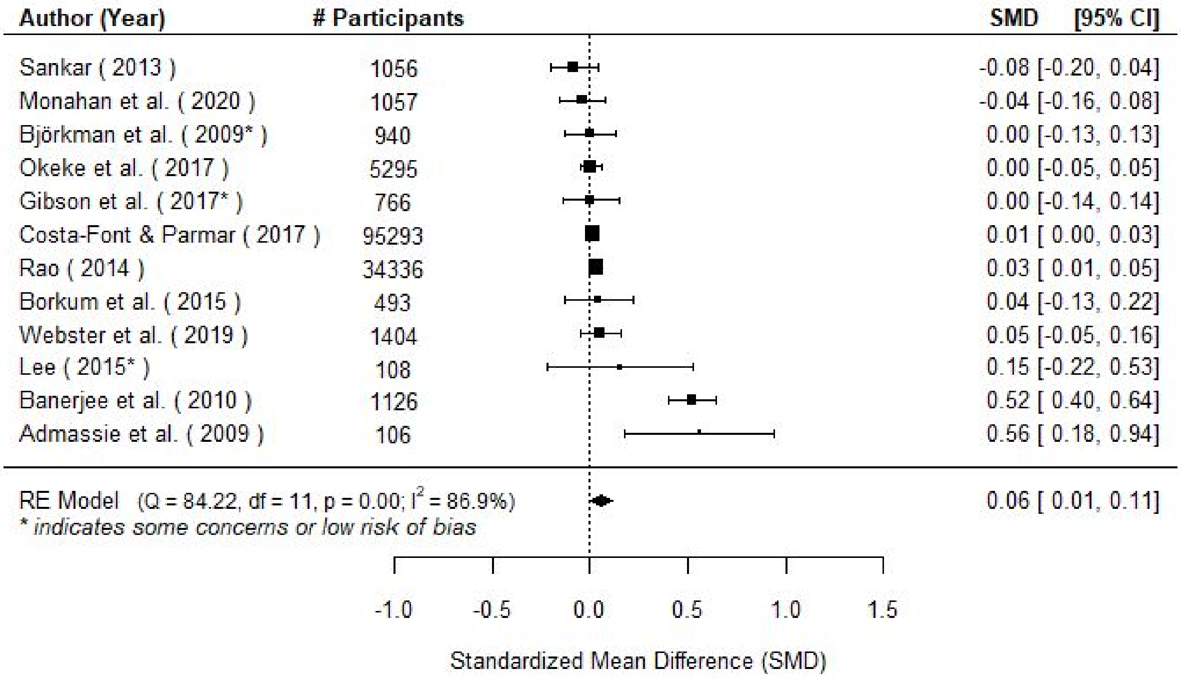

Supplementary Figure 18. Forest plot showing the observed outcomes and the estimate of the random-effects model for the impact of community engagement interventions on BCG vaccination.

A funnel plot of the estimates is shown in Supplementary Figure *18b*. The regression test indicated funnel plot asymmetry ($p = 0.04$) but not the rank correlation test ($p = 0.20$).

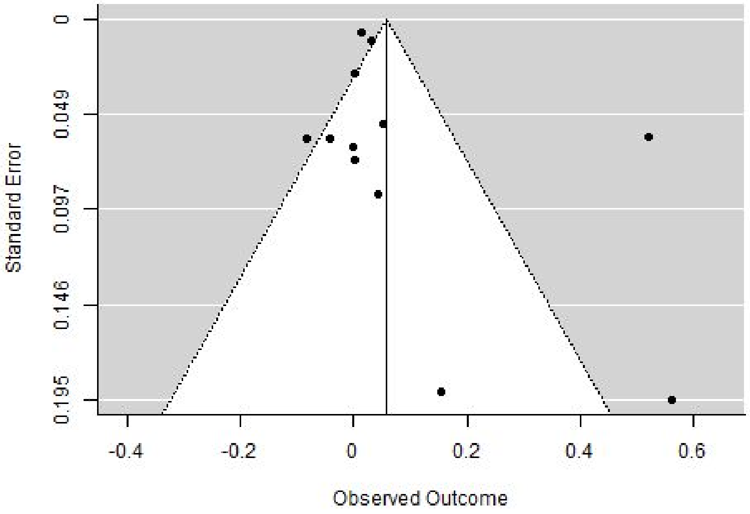

Supplementary Figure 18b*: Funnel plot*

As a robustness check, we used robust variance analysis and included all dependent effects in the analysis, totaling 16 effects from the same 12 studies (*df* = 7.4). The overall average effect was slightly smaller and non-significant ($\hat{\mu} = 0.04$ [95% CI: -0.02 to 0.10], *p* = .20). Sensitivity analyses show the effect to be sensitive to all values of Rho.

DPT1

For peer review only - http://bmjopen.bmj.com/site/about/guidelines.xhtml

We included a total of $k = 8$ studies in the analysis. The estimated average outcome based on the random-effects model was $\hat{\mu} = 0.04$ ([95% CI: -0.04 to 0.11], $z = 0.99$, $p = 0.32$), indicating no difference between the intervention group and the control group (see Supplementary Figure 19). According to the Q-test, the true outcomes appear to be heterogeneous ($Q(7) = 30.18$, $p < 0.01$, $\hat{\tau}^2 = 0.01$, $I^2 = 76.81\%$). An examination of the studentized residuals revealed that none of the studies had a value larger than ± 2.73 and hence there was no indication of outliers in the context of this model. According to the Cook's distances, none of the studies could be considered to be overly influential. When we removed studies assessed as high risk of bias, two studies remained, and the average effect increased slightly ($\hat{\mu} = 0.04$ ([95% CI: -0.04 to 0.11]), but was still non-significant ($z = 0.99$, $p = 0.32$). Again, with only two studies contributing effects, this must be interpreted with caution.

We tested for potential sources of heterogeneity and found that study design was a significant predictor of the effect such that studies using quasi-experimental designs had smaller effects than RCTs by .15 standard deviation units ($\beta = -0.15$ [95% CI: -0.29 to -0.02], $p = .03$). We also found that there was a significant difference in the size of effects between programmes implemented by government agencies and those that were not, such that programmes implemented by government agencies (either alone or in tandem with another agency) had larger effects than programmes not implemented by a government agency by .17 standard deviation units ($\beta = 0.17$ [95% CI: 0.01 to 0.34], $p = .04$). No other moderators were significant (see Supplementary Table 3).

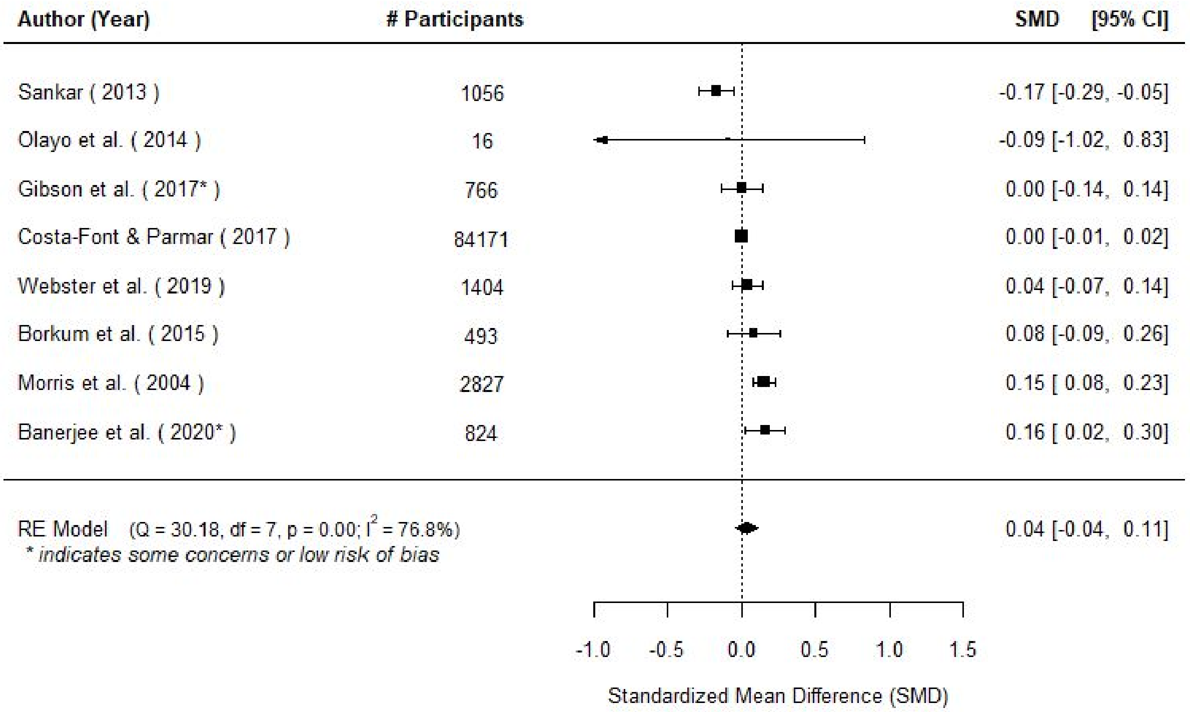

Supplementary Figure 19. Forest plot showing the observed outcomes and the estimate of the random-effects model for the impact of community engagement interventions on DPT1 vaccination.

As a robustness check, we used robust variance analysis and included all dependent effects in the analysis, totaling 21 effects from the same 8 studies (*df* = 5.24). The overall average effect was smaller and still non-significant ($\hat{\mu} = 0.01$ [95% CI: -0.06 to 0.09], $p = .66$). Sensitivity analyses show the effect to be sensitive to all values of Rho.

DPT2

For peer review only - http://bmjopen.bmj.com/site/about/guidelines.xhtml

We included a total of $k = 5$ studies in the analysis. The estimated average outcome based on the random-effects model was $\hat{\mu} = 0.07$ (95% CI: 0.01 to 0.12). Therefore, the average outcome differed significantly from zero ($z = 2.23$, $p = 0.03$), indicating small but significant benefit to the treated group compared to the control group. According to the Q-test, there was no significant amount of heterogeneity in the true outcomes ($Q(4) = 3.34$, $p = 0.50$, $\hat{\tau}^2 = 0.00$, $I^2 = 0.00\%$). An examination of the studentized residuals revealed that none of the studies had a value larger than ± 2.58 and hence there was no indication of outliers in the context of this model. According to the Cook's distances, none of the studies could be considered to be overly influential. With no heterogeneity present, we did not examine potential sources of variation. When high risk of bias studies were removed, the resulting average effect increased slightly ($\hat{\mu} = 0.11$ [95% CI: 0.01 to 0.20]) and was still significantly different from zero ($z = 2.12$, $p = 0.03$).

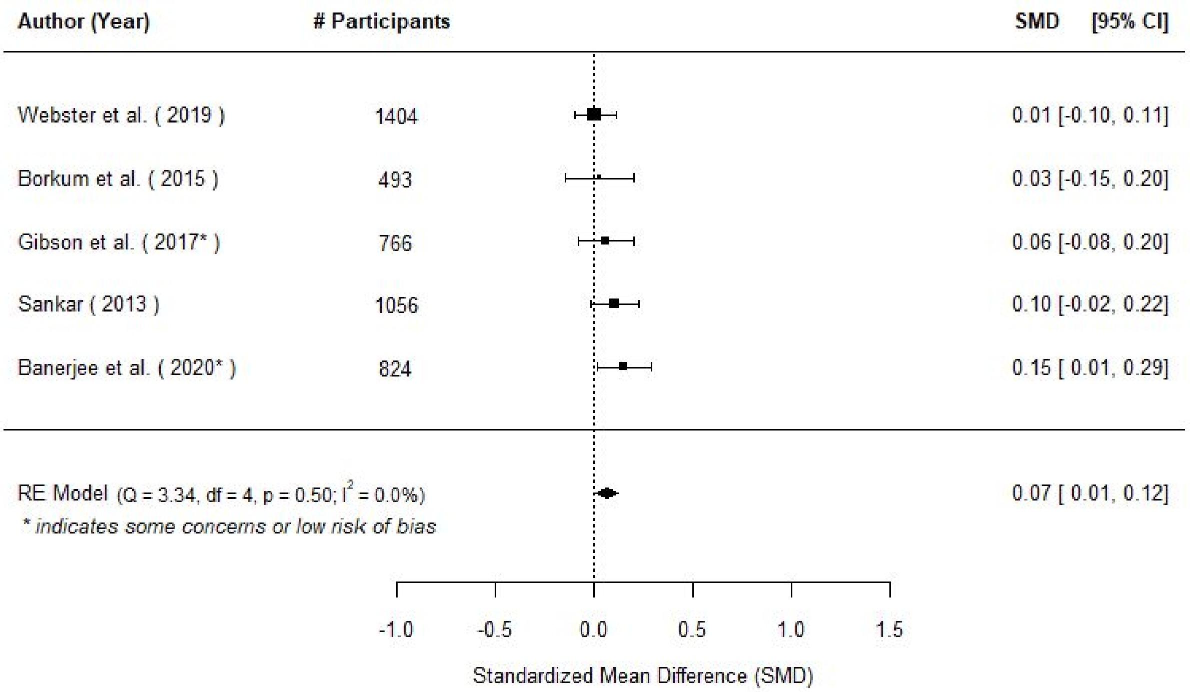

Supplementary Figure 20. *Forest plot showing the observed outcomes and the estimate of the random-effects model for the impact of community engagement interventions on DPT2 vaccination.*

This group of studies was not sufficiently powered for a robustness check using robust variance analysis (*df* = 3.51).

OPV0

We included a total of $k = 5$ studies in the analysis. The estimated average outcome based on the random-effects model was $\hat{\mu} = 0.10$ (95% CI: -0.06 to 0.26). Therefore, the average outcome did not differ significantly from zero ($z = 1.24$, $p = 0.22$), indicating no difference between the intervention and control groups (see Supplementary Figure 21). According to the Q-test, the true outcomes appear to be heterogeneous ($Q(4) = 45.74$, $p < 0.01$, $\hat{\tau}^2 = 0.03$, $I^2 = 91.26\%$). An examination of the studentized residuals revealed that one study (Sankar 2013) had a value larger than ± 2.58 and may be a potential outlier in the context of this model. According to the Cook's distances, Sankar (2013) could also be considered to be overly influential. Indeed, sensitivity analyses leaving each study out indicated that removing Sankar (2013) would reduce the overall average effect ($\hat{\mu}$ = -0.01 [95% CI: -0.01 to 0.03]) which would become non-significant (z = 1.02, p = .31).

Exposure to the intervention (in months) was a significant predictor of variation on OPV0 vaccinations such that each additional month of exposure increased the size of the effect by 0.02 standard deviation units (β = .02, [95%

For peer review only - http://bmjopen.bmj.com/site/about/guidelines.xhtml

CI: 0.01 to 0.03]; $p < .001$). Publication year was also a significant predictor of variation on OPV0 vaccinations such that each additional year decreased the size of the effect by 0.07 standard deviation units (β = -0.07, [95% CI: -0.11 to -0.02]; $p = 0.002$), meaning more recent studies found smaller effects than older studies. With only one study that was not assessed as high risk of bias, we could not conduct a sensitivity analysis by study quality.

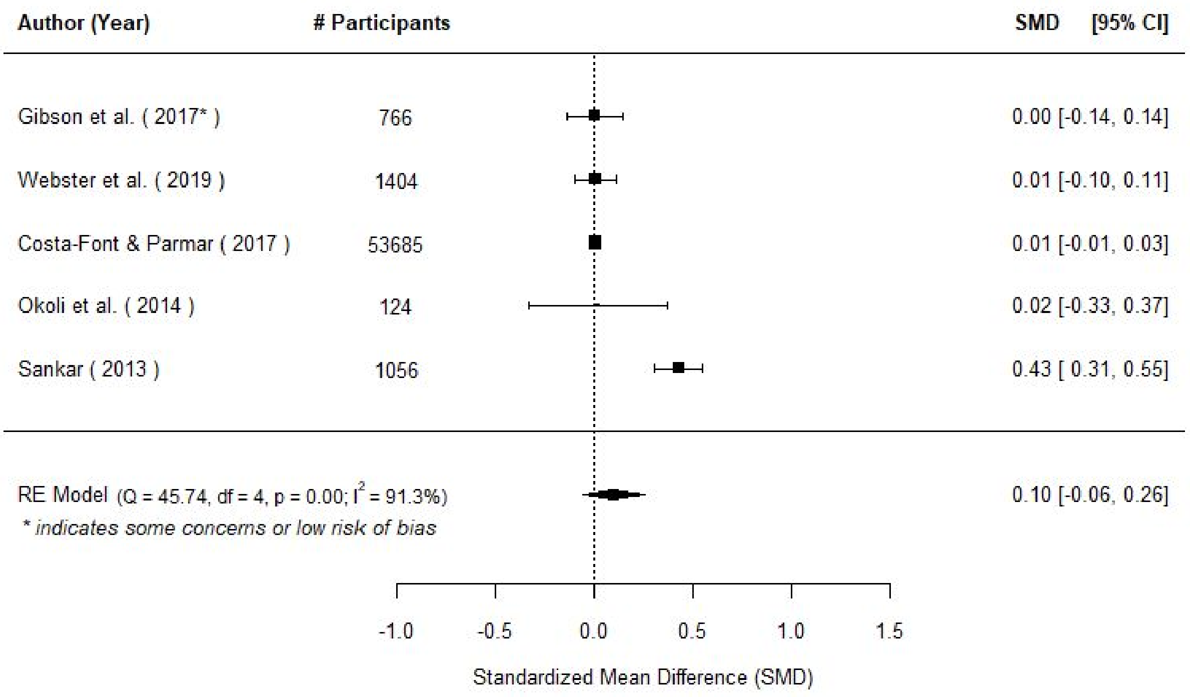

Supplementary Figure 21. Forest plot showing the observed outcomes and the estimate of the random-effects model for the impact of community engagement interventions on OPV0 vaccination.

This group of studies was not sufficiently powered for a robustness check using robust variance analysis ($df = 3.76$).

OPV1

We included a total of $k = 5$ studies in the analysis. The estimated average outcome based on the random-effects model was $\hat{\mu} = 0.08$ ([95% CI: 0.004 to 0.15], $z = 2.06$, $p = 0.04$), indicating a very small but significant benefit to the treated group compared to the control group (see Supplementary Figure 22). According to the Q-test, there was no significant amount of heterogeneity in the true outcomes ($Q(4) = 4.77$, $p = 0.31$, $\hat{\tau}^2 = 0.00$, $I^2 = 16.17\%$), thus we did not test for sources of heterogeneity. An examination of the studentized residuals revealed that none of the studies had a value larger than ± 2.58 and hence there was no indication of outliers in the context of this model. According to the Cook's distances, none of the studies could be considered to be overly influential.

For peer review only - http://bmjopen.bmj.com/site/about/guidelines.xhtml

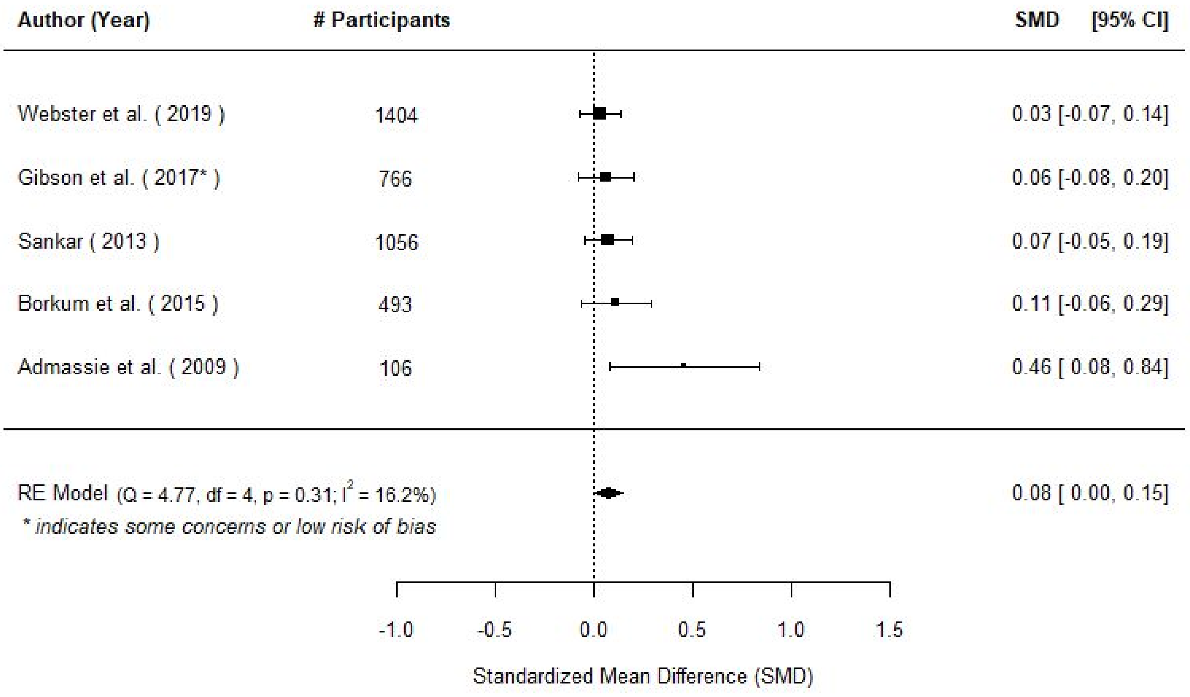

Supplementary Figure 22. Forest plot showing the observed outcomes and the estimate of the random-effects model for the impact of community engagement interventions on OPV1 vaccination.

This group of studies was not sufficiently powered for a robustness check using robust variance analysis (*df* = 3.02).

OPV2

We included a total of $k = 5$ studies in the analysis. The estimated average outcome based on the random-effects model was $\hat{\mu} = 0.24$ ([95% CI: 0.07 to 0.40], $z = 2.84$, $p < 0.01$), indicating a small but significant benefit to the treated group compared to the control group (see Supplementary Figure CEOPV2). According to the Q-test, the true outcomes appear to be heterogeneous ($Q(4) = 22.32$, $p < 0.01$, $\hat{\tau}^2 = 0.03$, $I^2 = 82.08\%$). An examination of the studentized residuals revealed that none of the studies had a value larger than ± 2.58 and hence there was no indication of outliers in the context of this model. According to the Cook's distances, none of the studies could be considered to be overly influential. With only one study that was not assessed as high risk of bias, we could not conduct a sensitivity analysis by study quality.

We tested for potential sources of heterogeneity and found several significant moderators in the context of this model (see SupplementaryTable 3). Exposure to intervention was significant such that each additional month of exposure increased the average effect by .02 standard deviation units ($\beta = 0.02$ [95% CI: 0.01 to 0.02], $p < .0001$). In other words, longer interventions produced larger effects. Publication year was also significant such that older studies reported larger effects than more recent studies. Specifically, each additional year decreased the average effect by .05 standard deviation units ($\beta = -0.05$ [95% CI: -0.08 to -0.02], $p < 0.001$). Finally, region was a significant predictor such that Sub-Saharan Africa had smaller effects that South Asia by .26 standard deviation units ($\beta = -0.26$ [95% CI: -0.39 to -0.13], $p < 0.001$).

For peer review only - http://bmjopen.bmj.com/site/about/guidelines.xhtml

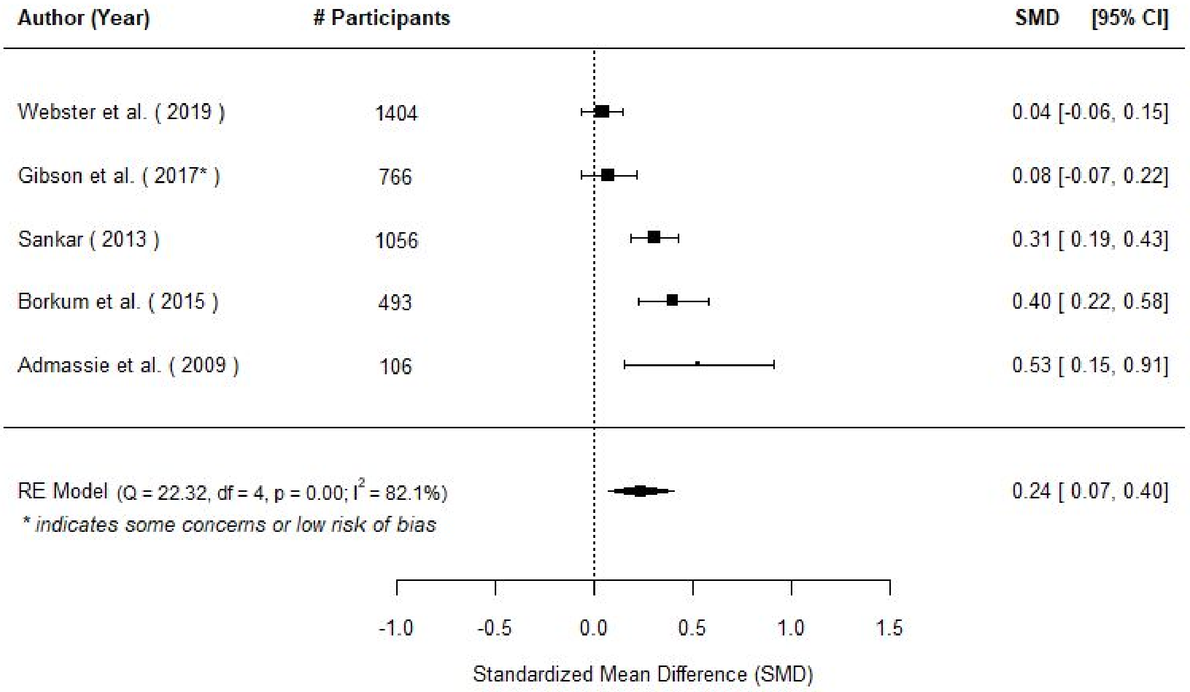

Supplementary Figure 23. Forest plot showing the observed outcomes and the estimate of the random-effects model for the impact of community engagement interventions on OPV2 vaccination.

This group of studies was not sufficiently powered for a robustness check using robust variance analysis (*df* = 3.75).

OPV3

We included total of $k = 9$ studies in the analysis. The estimated average outcome based on the random-effects model was $\hat{\mu} = 0.24$ ([95% CI: 0.09 to 0.40], $z = 3.06$, $p = 0.002$), indicating a moderate and significant benefit to the intervention group compared to the control group (see Supplementary Figure 24). According to the Q-test, the true outcomes appear to be heterogeneous ($Q(8) = 222.99$, $p < 0.001$, $\hat{\tau}^2 = 0.05$, $I^2 = 96.41\%$). An examination of the studentized residuals revealed that one study (Sankar 2013) had a value larger than ± 2.77 and may be a potential outlier in the context of this model. According to the Cook's distances, one study (Sankar 2013) could be considered to be overly influential. Sensitivity analyses leaving each study out indicated that removing Sankar (2013) would substantially reduce the overall average effect ($\hat{\mu} = 0.08$ [95% CI: 0.01 to 0.15]), but the effect would still be positive and significant ($z = 2.39$, $p = .02$). When low quality studies were removed, the resulting effect increased, but was no longer significant ($\hat{\hat{\mu}} = 0.32$ [95% CI: -0.15 to 0.79], $z = 1.32$, $p = 0.19$). With only two studies of high or moderate quality, this result should be interpreted with caution. There were no significant sources of heterogeneity (see Supplementary Table 3).

For peer review only - http://bmjopen.bmj.com/site/about/guidelines.xhtml

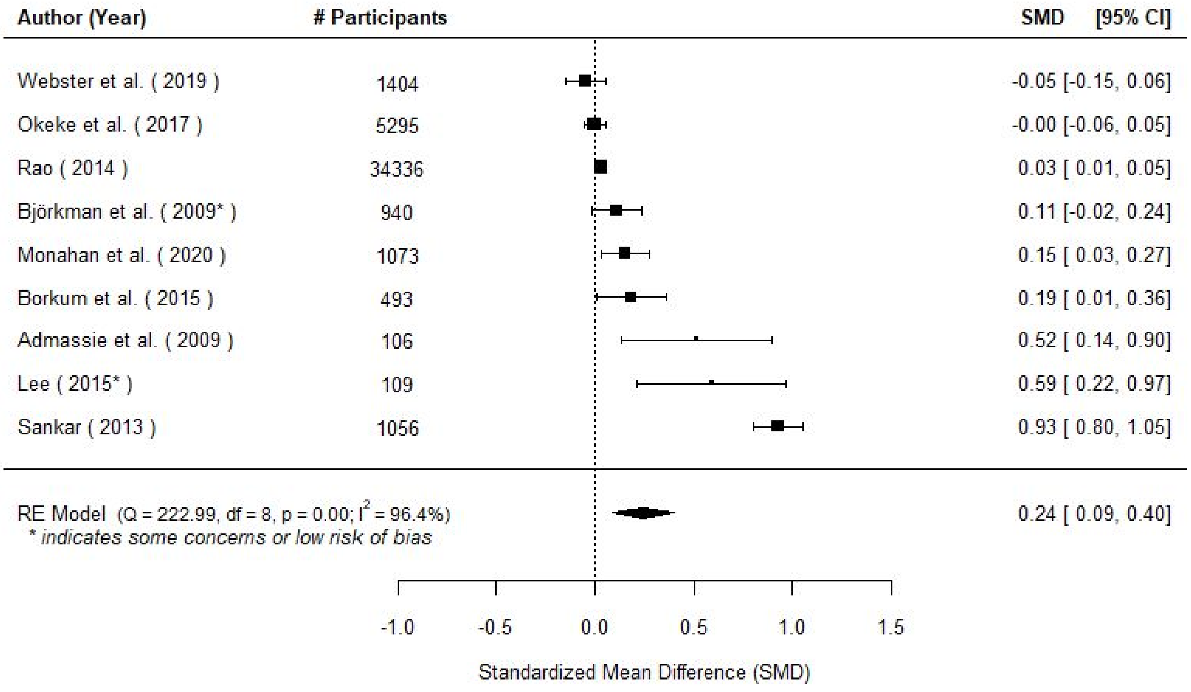

Supplementary Figure 24. Forest plot showing the observed outcomes and the estimate of the random-effects model for the impact of community engagement interventions on OPV3 vaccination.

As a robustness check, we used robust variance analysis and included all dependent effects in the analysis, totaling 10 effects from the same 9 studies (*df* = 6.74). The overall average effect was identical, but no longer significant ($\hat{\mu}$ = 0.24 [95% CI: -0.02 to 0.51], *p* = .06). Sensitivity analyses show the effect to be sensitive to all values of Rho.

Dropouts

We included a total of $k = 5$ studies in the analysis. The estimated average outcome based on the random-effects model was $\hat{\mu} = 0.03$ ([95% CI: -0.11 to 0.16], $z = 0.36$, $p = 0.72$), indicating no significant difference between the intervention group and the control group (see Supplementary Figure 25). According to the Q-test, the true outcomes appear to be heterogeneous ($Q(4) = 44.86$, $p < 0.001$, $\hat{\tau}^2 = 0.02$, $I^2 = 91.08\%$). An examination of the studentized residuals revealed that one study (Webster et al. 2019) had a value larger than ± 2.58 and may be a potential outlier in the context of this model. Indeed, sensitivity analyses indicated that removing Webster and colleagues (2019) would increase the average effect to $\hat{\mu} = 0.09$ (95% CI: -0.01 to 0.19), but the average outcome still did not differ significantly from zero ($z = 1.70 p = 0.09$). According to the Cook's distances, none of the studies could be considered to be overly influential. With only one study assessed as having a low risk of bias (Banerjee et al. 2020) and the remaining studies at high risk of bias, we were unable to conduct sensitivity analysis by study quality. Only publication year was a significant predictor such that more recently published studies had higher average effects than older studies. Specifically, each additional year increased the average effect by .33 standard deviation units ($\beta = 0.33$ [95% CI: 0.10 to 0.57], $p = 0.01$).

For peer review only - http://bmjopen.bmj.com/site/about/guidelines.xhtml

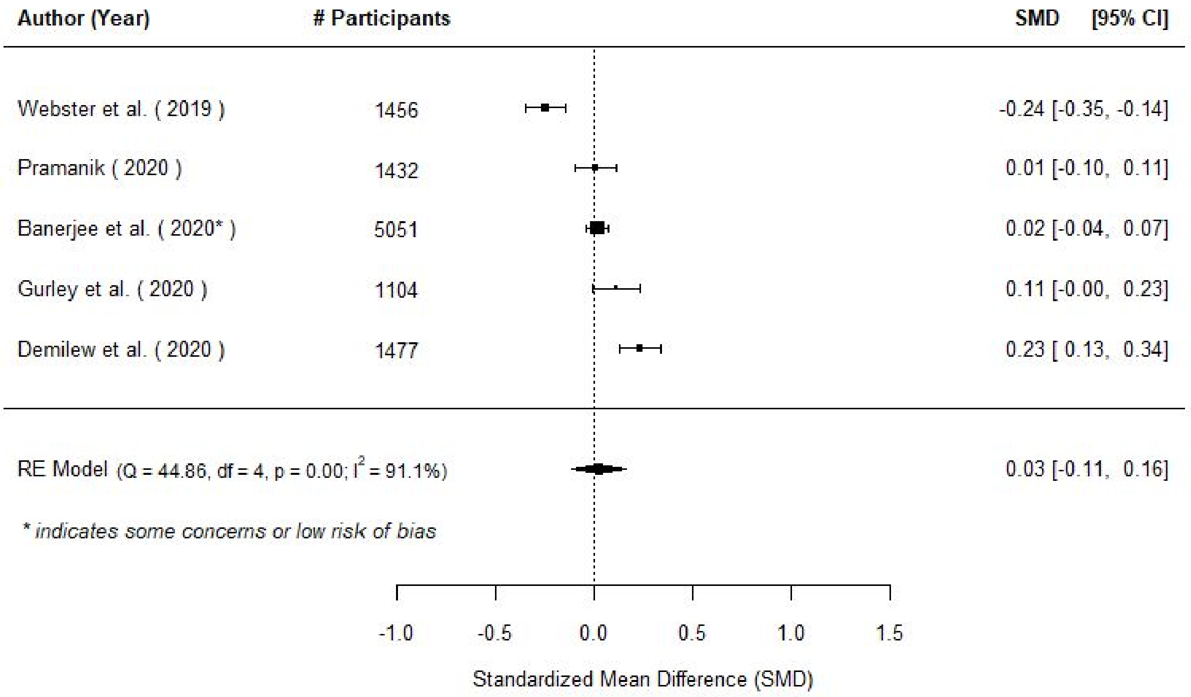

Supplementary Figure 25. Forest plot showing the observed outcomes and the estimate of the random-effects model for the impact of community engagement interventions on childhood morbidity.

This group of studies was not sufficiently powered for a robustness check using robust variance analysis (*df* = 3.98).

Morbidity

We used reports of diarrhea (most typically in the past two weeks) as a proxy for childhood morbidity. In all cases, effects were reverse coded such that positive effects always indicate a benefit to the treated group. Thus, a positive effect here would be interpreted as a reduction in diarrhea among treated participants compared to control participants. A total of $k = 10$ studies were included in the analysis. The estimated average outcome was $\hat{\mu} = 0.01$ ([95% CI: -0.06 to 0.08], $z = 0.34$, $p = 0.73$), indicating no significant difference between intervention group and control group (Supplementary Figure 26). According to the Q-test, the true outcomes appear to be heterogeneous ($Q(9) = 52.37$, $p < 0.01$, $\hat{\tau}^2 = 0.01$, $I^2 = 82.82\%$). When high risk of bias studies were removed, only two studies remained. The resulting estimated average outcome was identical ($\hat{\mu} = 0.01$ [95% CI: -0.21 to 0.23]), and was still non-significant ($z = 0.09$, $p = 0.93$).

An examination of the studentized residuals revealed that one study (Assegaai et al. 2018) had a value larger than ± 2.81 and may be a potential outlier in the context of this model. According to the Cook's distances, Assegaai and colleagues (2018) could also be considered to be overly influential. Indeed, sensitivity analyses leaving each study out indicated that removing Assegaai and colleagues (2018) would reduce the overall average effect ($\hat{\mu}$ = -0.02 (95% CI: -0.08 to 0.05), making the effect negative but still non-significant (z = -0.47, p = .64). A funnel plot of the estimates is shown in Supplementary Figure 26b. Neither the rank correlation nor the regression test indicated any funnel plot asymmetry ($p = 0.60$ and $p = 0.49$, respectively). We tested for sources of heterogeneity, but there were no significant moderators (see Supplementary Table 3)

For peer review only - http://bmjopen.bmj.com/site/about/guidelines.xhtml

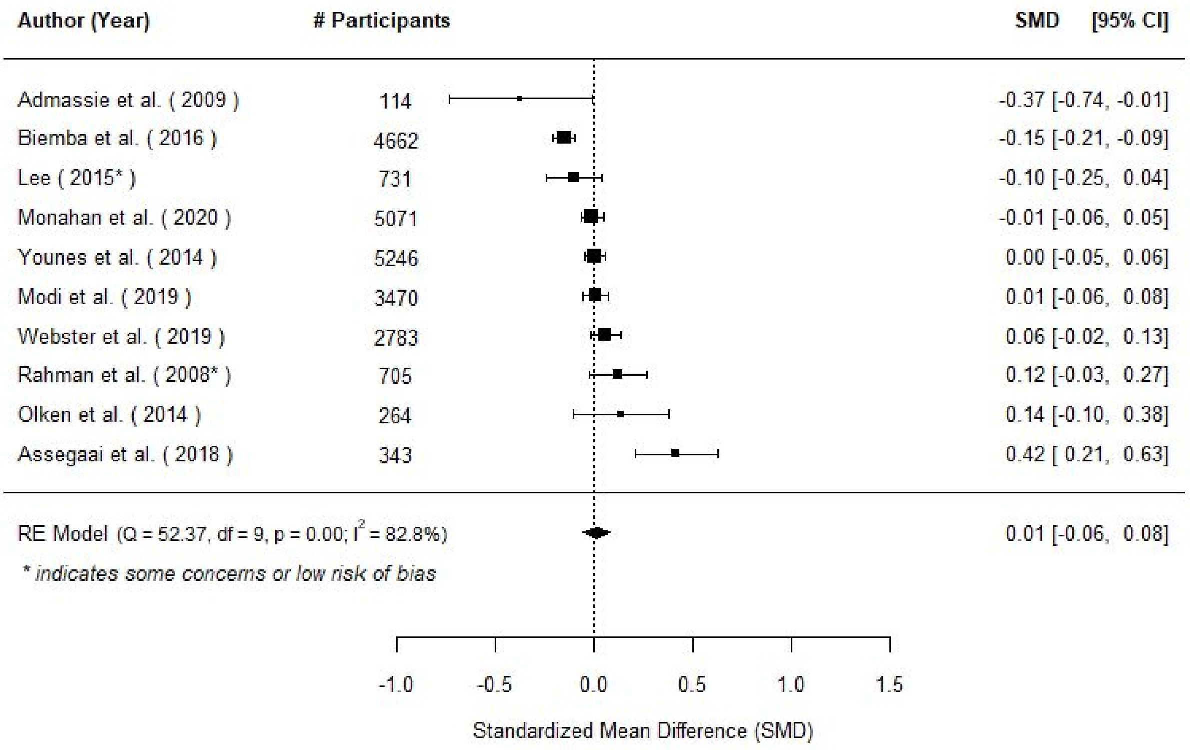

Supplementary Figure 26. Forest plot showing the observed outcomes and the estimate of the random-effects model for the impact of community engagement interventions on childhood morbidity.

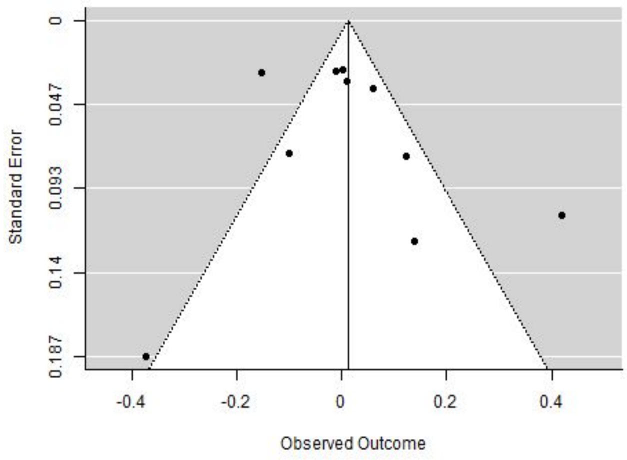

Supplementary Figure 26b. Funnel plot for studies examining the effect of community engagement interventions on childhood morbidity

As a robustness check, we used robust variance analysis and included all dependent effects in the analysis, totaling 26 effects from the same 10 studies (*df* = 7.69). The overall average effect was identical and still non-significant ($\hat{\mu}$ = 0.01 [95% CI: −0.09 to 0.10], *p* = .86). Sensitivity analyses show the effect to be sensitive to all values of Rho.

For peer review only - http://bmjopen.bmj.com/site/about/guidelines.xhtml

Mortality

We included total of $k = 6$ studies in the analysis. The estimated average outcome based on the random-effects model was $\hat{\mu} = -0.04$ (95% CI: -0.09 to 0.01; *see Supplementary Figure C27*). Therefore, the average outcome did not differ significantly from zero ($z = -1.66$, $p = 0.10$), indicating no difference in mortality between the treatment and control groups. According to the Q-test, the true outcomes appear to be heterogeneous ($Q(5) = 19.28$, $p < 0.01$, $\hat{\tau}^2 = 0.00$, $I^2 = 74.07\%$). An examination of the studentized residuals revealed that none of the studies had a value larger than ± 2.64 and hence there was no indication of outliers in the context of this model. According to the Cook's distances, none of the studies could be considered to be overly influential. When high risk of bias studies were removed, only two studies remained. The resulting estimated average outcome was identical ($\hat{\mu} = -0.04$ [95% CI: -0.12 to 0.04]), and was still non-significant ($z = -1.07$, $p = 0.29$). We tested for sources of heterogeneity, and only publication year was significant such that each additional year increased the effect by .01 standard deviation units ($\hat{B} = 0.01$, $p < 0.001$ [95% CI: 0.01 to 0.02]). There were no other significant moderators (see Supplementary Table 3).

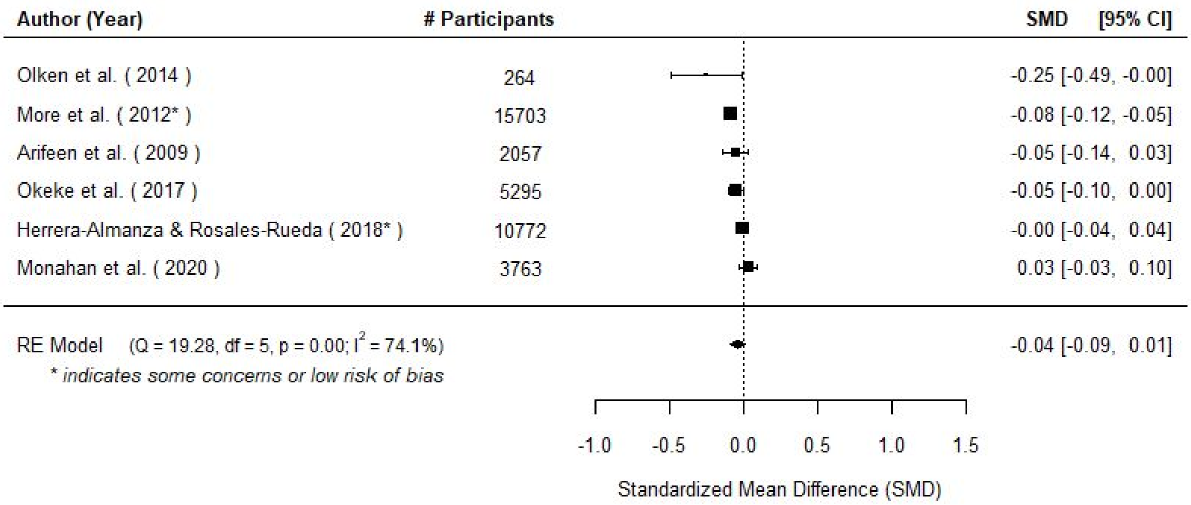

Supplementary Figure 27. Forest plot showing the observed outcomes and the estimate of the random-effects model for the impact of community engagement interventions on childhood morbidity.

As a robustness check, we used robust variance analysis and included all dependent effects in the analysis, totaling 25 effects from the same 6 studies (*df* = 4.07). The overall average effect was identical and still non-significant ($\hat{\mu} = -0.04$ [95% CI: -0.09 to 0.01], *p* = .12). Sensitivity analyses show the effect to be sensitive to all values of Rho.

Immunisation knowledge

We included a total of $k = 9$ studies in the analysis. The estimated average outcome based on the random-effects model was $\hat{\mu} = 0.19$ (95% CI: 0.07 to 0.31). Therefore, the average outcome differed significantly from zero ($z = 3.02$, $p < 0.01$), indicating a significant benefit to the treatment group compared to the control group (see Supplementary Figure 28). According to the Q-test, the true outcomes appear to be heterogeneous ($Q(8) = 62.87$, $p < 0.01$, $\hat{\tau}^2 = 0.03$, $I^2 = 87.28\%$). An examination of the studentized residuals revealed that one study (Banwat et al. 2015) had a value larger than ± 2.77 and may be a potential outlier in the context of this model. Indeed, sensitivity analyses leaving each study out indicated that removing Banwat and colleagues (2015) would reduce the

For peer review only - http://bmjopen.bmj.com/site/about/guidelines.xhtml

overall average effect ($\hat{\mu}$ = 0.11 (95% CI: -0.02 to 0.20), but the effect would still be positive and significant (z = 2.33, *p* = .02). According to the Cook's distances, none of the studies could be considered to be overly influential.

We examined potential sources of heterogeneity, and study design was a significant predictor of immunisation knowledge such that studies using quasi-experimental designs had larger average effects than RCT designs by .44 standard deviation units ($\hat{\beta} = 0.44$ [95% CI: 0.23 to 0.64], $p < 0.001$). Region was also a significant predictor, but region was perfectly confounded with study design (e.g. all studies from South Asia were RCTs, while all studies from Sub-Saharan Africa were all quasi-experimental designs), so the effects were larger in Sub-Saharan Africa than from South Asia by the same .44 standard deviation units ($\beta = 0.44$ [95% CI: 0.23 to 0.64], $p < 0.001$). No other moderators were significant (see Supplementary Table 3)

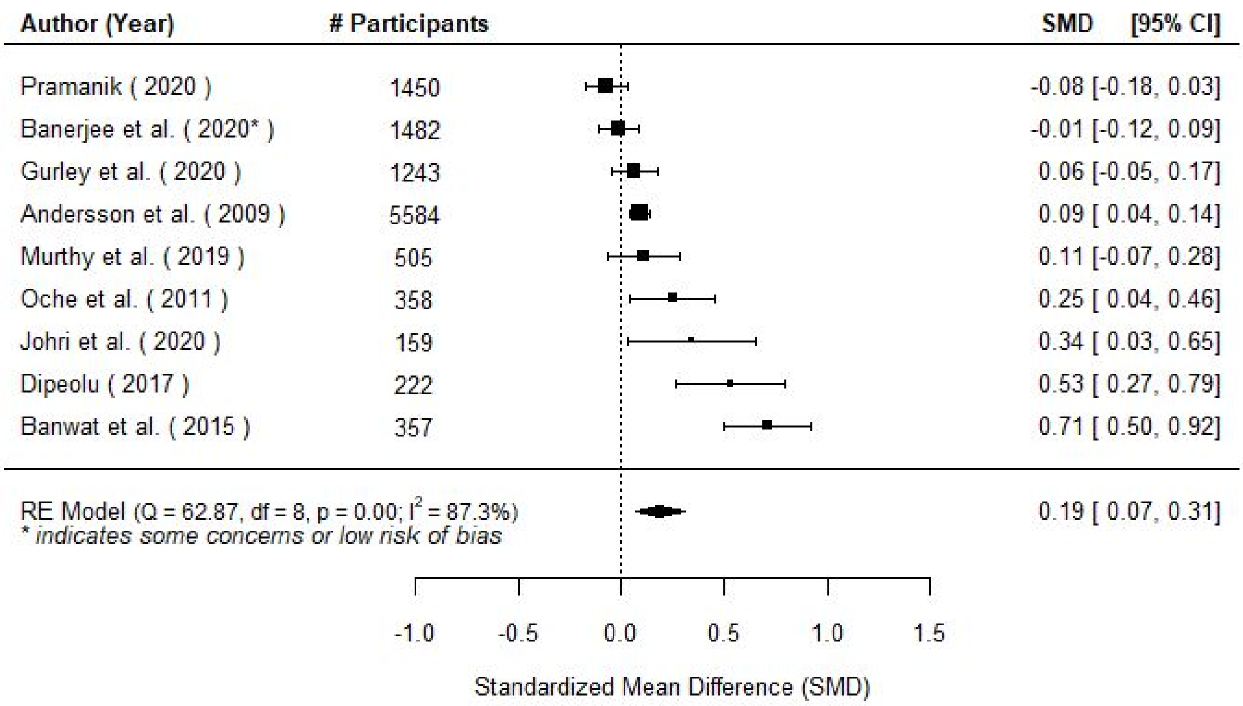

Supplementary Figure 28. Forest plot showing the observed outcomes and the estimate of the random-effects model for the impact of community engagement interventions on childhood morbidity.

As a robustness check, we used robust variance analysis and included all dependent effects in the analysis, totaling 13 effects from the same 9 studies (*df* = 7.69). The overall average effect slightly smaller and become non-significant ($\hat{\mu} = 0.17$ [95% CI: -0.02 to 0.37], *p* = .07). Sensitivity analyses show the effect to be sensitive to all values of Rho.

Immunisation attitudes

We included a total of $k = 6$ studies in the analysis. The estimated average outcome was $\hat{\mu} = 0.14$ ([95% CI: -0.03 to 0.31], $z = 1.60$, $p = 0.11$), indicating no significant different between the intervention and control groups (see Supplementary Figure 29). According to the Q-test, the true outcomes appear to be heterogeneous ($Q(5) = 50.43$, $p < 0.01$, $\hat{\tau}^2 = 0.04$, $I^2 = 90.09\%$). An examination of the studentized residuals revealed that one study (Banwat et al. 2015) had a value larger than ± 2.64 and may be a potential outlier in the context of this model. Indeed, sensitivity analyses leaving each study out indicated that removing Banwat and colleagues (2015) would reduce the overall average effect ($\hat{\mu}$ = 0.04 (95% CI: -0.05 to 0.14), but the effect would still be positive and non-significant (z = 0.92, *p* = .36). According to the Cook's distances, none of the studies could be considered to be overly influential.

For peer review only - http://bmjopen.bmj.com/site/about/guidelines.xhtml

With only one study not assessed as high risk of bias, we were unable to conduct a sensitivity analysis by study quality. We tested for sources of heterogeneity, but of the moderators we were able to test, none were significant (see Supplementary Table 3)

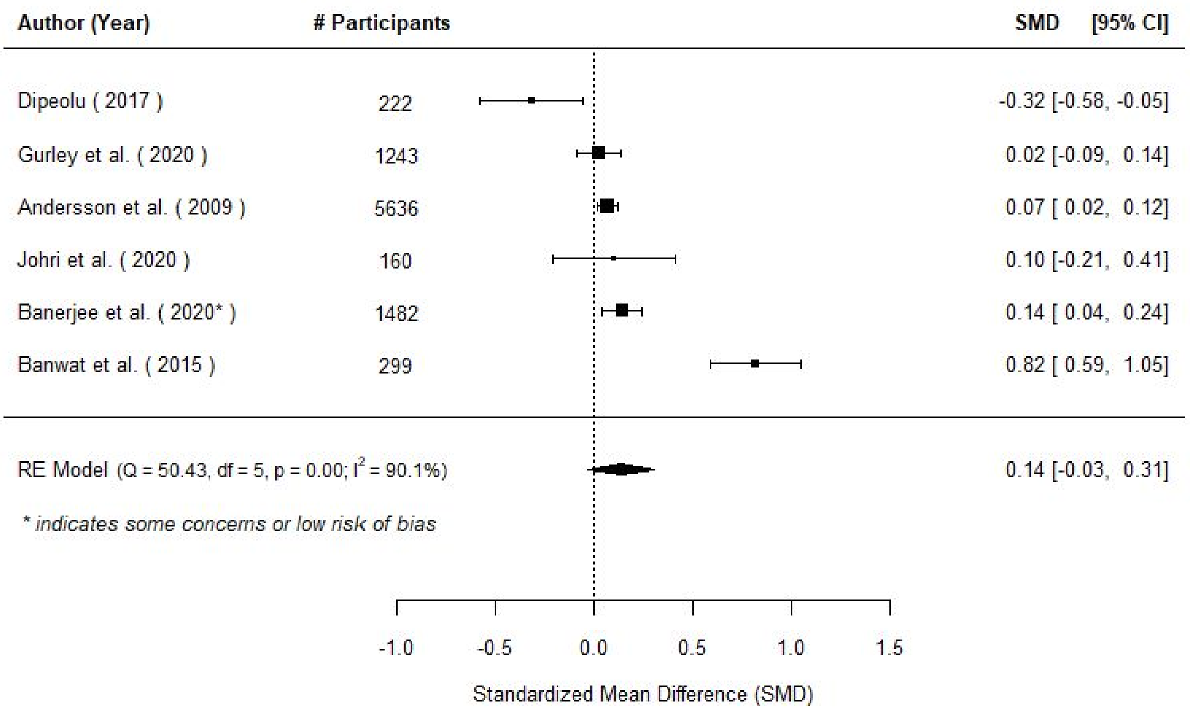

Supplementary Figure 29. Funnel plot for studies examining the effect of community engagement interventions on immunisation attitudes

There were no dependent effects, so we did not complete a robustness check using RVE for this analysis.

Vaccination card availability

We included total of $k = 4$ studies in the analysis. The estimated average outcome was $\hat{\mu} = -0.01$ (95% CI: -0.05 to 0.02). The average outcome did not differ significantly from zero ($z = -0.84$, $p = 0.40$), indicating no difference between the intervention group and the control group on vaccination card availability (see Supplementary Figure 30). According to the Q-test, there was no significant amount of heterogeneity in the true outcomes ($Q(3) = 2.27$, $p = 0.52$, $\hat{\tau}^2 = 0.00$, $I^2 = 0.00\%$). One study (Herrera-Almanza & Rosales-Rueda 2018) had a relatively large weight compared to the rest of the studies (i.e., weight $\geq 3/k$, so a weight at least 3 times as large as having equal weights across studies). However, this was also the only study assessed as having a low risk of bias. The three other studies in the analysis were assessed as high risk of bias. An examination of the studentized residuals revealed that none of the studies had a value larger than ± 2.50 and hence there was no indication of outliers in the context of this model. According to the Cook's distances, none of the studies could be considered to be overly influential. With no heterogeneity present, we did not test for moderation.

For peer review only - http://bmjopen.bmj.com/site/about/guidelines.xhtml

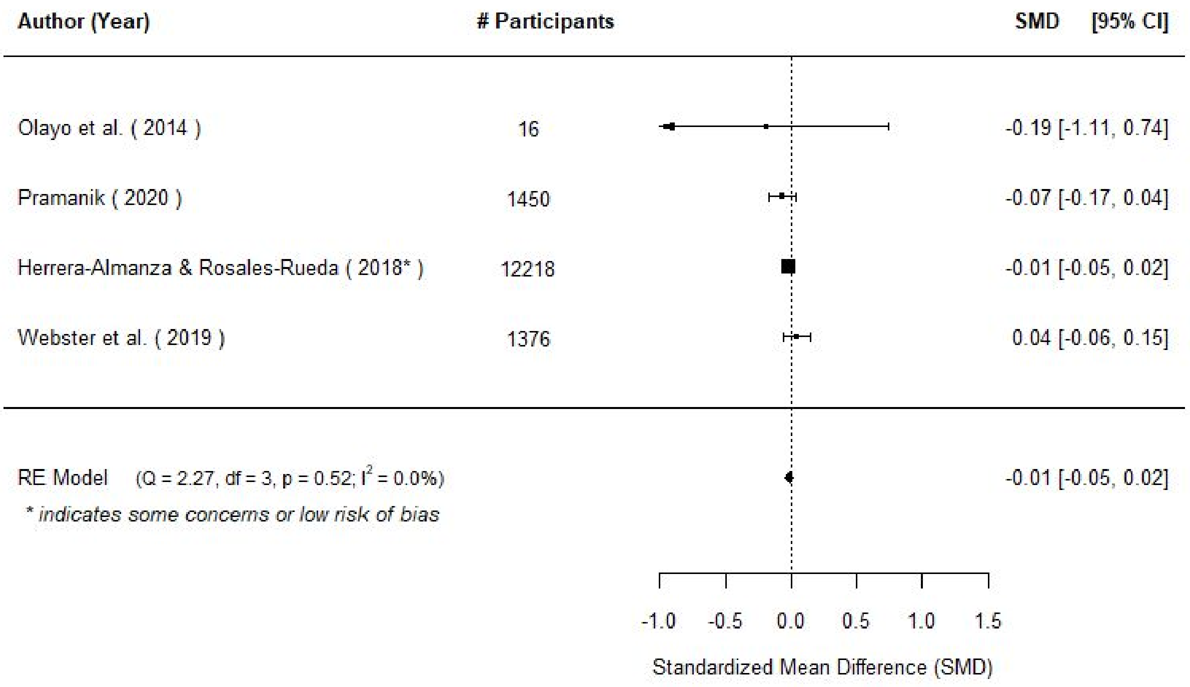

Supplementary Figure 30. Forest plot showing the observed outcomes and the estimate of the random-effects model for the impact of community engagement interventions on vaccination card availability.

There were no dependent effects, so we did not complete a robustness check using RVE for this analysis.

Experience and satisfaction with health services

Only two studies using community engagement interventions reported on experience and satisfaction with health services. The estimated average outcome based on the random-effects model was $\hat{\mu} = 0.04$ (95% CI: -0.15 to 0.23). Therefore, the average outcome did not differ significantly from zero ($z = 0.38$, $p = 0.70$, see Supplementary Figure 31). Given the small number of studies, this result should be interpreted with caution. According to the Q-test, the true outcomes appear to be homogeneous ($Q(1) = 1.93$, $p = 0.16$, $\hat{\tau}^2 = 0.01$, $I^2 = 48.22\%$; With only two studies and no heterogeneity, moderator analyses were not appropriate and tests of publication bias are not valid, and we were not powered for a robustness check using RVE.

For peer review only - http://bmjopen.bmj.com/site/about/guidelines.xhtml

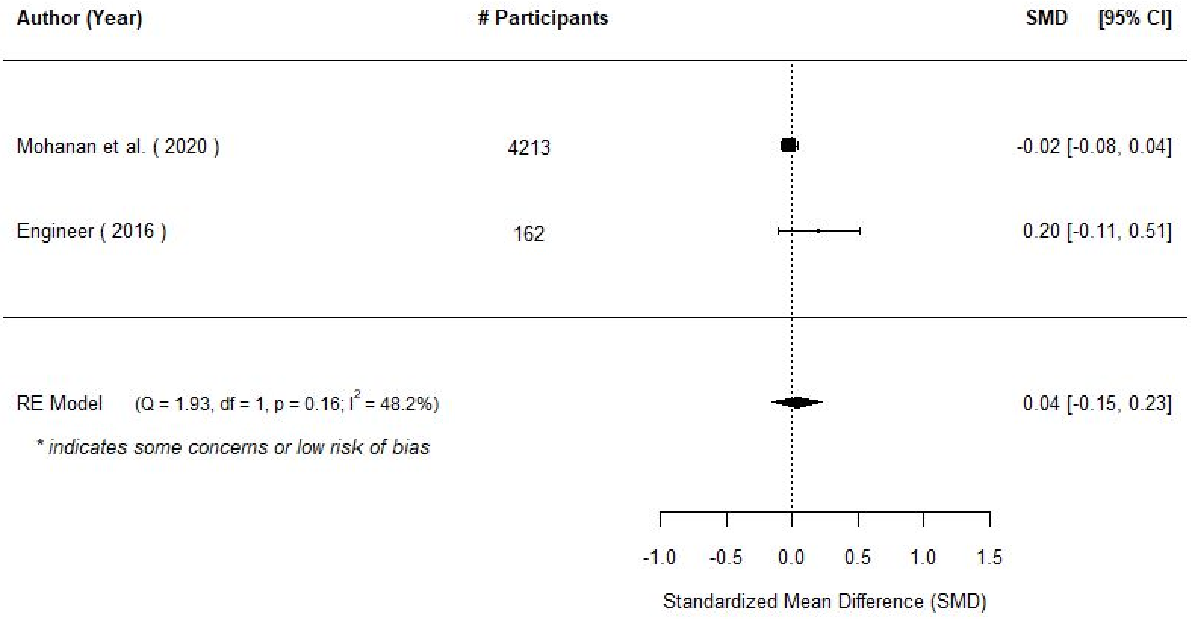

Supplementary Figure 31. Forest plot showing the observed outcomes and the estimate of the random-effects model for community engagement interventions on experience and satisfaction with health services.

Formal health worker's motivation, capacity and performance

Only two studies using community engagement interventions reported on formal health worker's motivation, capacity and performance. The estimated average outcome based on the random-effects model was $\hat{\mu} = 0.11$ (95% CI: -0.07 to 0.29). Therefore, the average outcome did not differ significantly from zero ($z = 0.38$, $p = 0.70$, see Supplementary Figure 32). Given the small number of studies, this result should be interpreted with caution. According to the Q-test, the true outcomes appear to be homogeneous ($Q(1) = 0.04$, $p = 0.84$, $\hat{\tau}^2 = 0.00$, $I^2 = 0.00$ %; With only two studies and no heterogeneity, moderator analyses were not appropriate and tests of publication bias are not valid, and we were not powered for a robustness check using RVE.

For peer review only - http://bmjopen.bmj.com/site/about/guidelines.xhtml

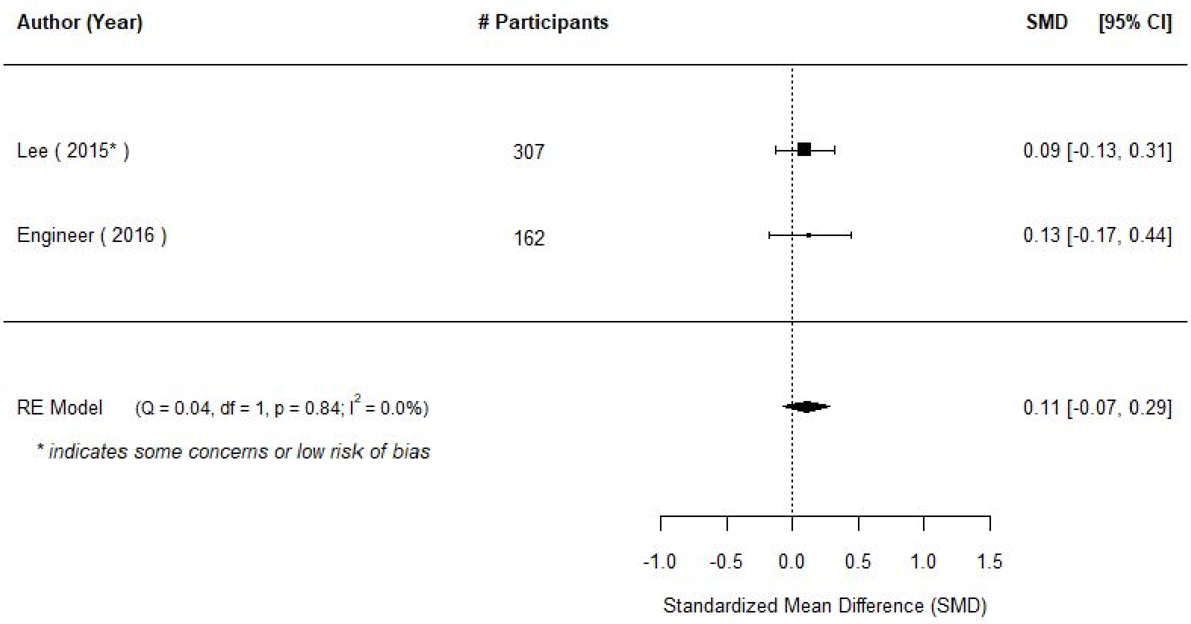

Supplementary Figure 32. Forest plot showing the observed outcomes and the estimate of the random-effects model for community engagement interventions on formal health worker's motivation, capacity and performance.

For peer review only - http://bmjopen.bmj.com/site/about/guidelines.xhtml

Supplementary Table 2: Summary of quantitative result

	TOTAL SAMPLE		ENGAGEMENT AS THE INTERVENTION	ENGAGEMENT IN THE DESIGN	ENGAGEMENT IN IMPLEMENTATION AUTONOMY	MULTIPLE ENGAGEMENT TYPES
	RVE (dependent effects)	RE (independent effects)	g, [95% CI], I^2, (k)	g, [95% CI], I^2, (k)	g, [95% CI], I^2, (k)	g, [95% CI], I^2, (k)
	g, [95% CI], I^2, (k; # of effects)	g, [95% CI], I^2, (k)				
**FULL IMMUNISATION**	**0.11**, [0.04, 0.18],** 87.27, (28; 53)	**0.14**, [0.06, 0.23],** 94.46, (28)	**0.08**, [0.03, 0.13],** 70.00, (12)	**010*, [0.02, 0.19],** 23.83, (5)	0.23, [-0.001, 0.47], 73.07, (2)	0.22, [-0.12, 0.56], 97.94, (9)
**PARTIAL IMMUNISATION**	**0.21*, [0.03, 0.38],** 95.90, (9; 13)	**0.23**, [0.09, 0.37],** 96.35, (9)	0.31, [-0.25, 0.87], 98.96, (2)	**0.14*, [0.01, 0.27],** 0.00, (2)	N/A	0.28, [-0.01, 0.56], 97.05, (4)
**MEASLES**	**0.06*, [0.01, 0.11],** 72.46, (20; 34)	**0.07**, [0.03, 0.11],** 73.64, (20)	**0.10***, [0.05, 0.15],** 60.29, (10)	**0.11*, [0.02, 0.21],** 0.00, (2)	0.03, [-0.09, 0.15], 54.84, (2)	0.03, [-0.10, 0.16], 86.76, (6)
**BCG**	0.04, [-0.02, 0.10], 79.17, (12; 16)	**0.06*, [0.01, 0.11],** 86.94, (12)	**0.02***, [0.01, 0.03],** 00.00, (4)	0.02, [-0.09, 0.13], 0.00, (2)	0.03, [-0.05, 0.11], 0.00, (2)	0.22, [-0.07, 0.52], 96.02, (4)
**DPT1**	0.01, [-0.06, 0.09], 62.55, (8; 21)	0.04, [-0.04, 0.11], 76.81, (8)	0.10, [-0.03, 0.22] 90.21, (3)	0.03, [-0.08.0.14], 0.00, (2)	N/A	**-0.17**, [-0.29, -0.05],** 0.00, (2)
**DPT2**	Not powered	**0.07*, [0.01, 0.12],** 0.00, (5)	N/A	0.05, [-0.06, 0.16], 0.00, (2)	N/A	N/A
**DPT3**	**0.10**, [0.05, 0.15],** 76.42, (22; 36)	**0.10***, [0.06, 0.14],** 76.78, (22)	**0.09**, [0.03, 0.15],** 73.17, (6)	0.04, [-0.01, 0.08], 0.00, (6)	0.11, [-0.05, 0.28], 80.12, (3)	**0.20**, [0.06, 0.34],** 87.93, (7)
**OPV0**	Not powered	0.10, [-0.06, 0.26], 91.26, (5)	N/A	0.01, [-0.13, 0.14], 0.00, (2)	N/A	N/A
**OPV1**	Not powered	**0.08*, [0.004, 0.15],** 16.17, (5)	N/A	0.08, [-0.03, 0.19], 0.00, (2)	N/A	0.22, [-0.15, 0.59], 72.32, (2)
**OPV2**	Not powered	**0.24**, [0.07, 0.40],** 82.08, (5)	N/A	0.23, [-0.09, 0.55], 87.44, (2)	N/A	**0.34***, [0.19, 0.50],** 15.51, (2)

For peer review only - http://bmjopen.bmj.com/site/about/guidelines.xhtml

**OPV3**	0.24, [-0.02, 0.51], 96.42, (9; 10)	**0.24**, [0.09, 0.40],** 96.41, (9)	0.16, [-0.02, 0.34], 83.83, (3)	N/A	0.03, [-0.12, 0.18], 70.38, (2)	0.48, [-0.24, 1.20], 98.92, (3)
**TIMELINESS (ALL)**	**0.11**, [0.06, 0.16],** 39.92, (11; 54)	----	----	----	----	----
**TIMELINESS (DTP3)**	No dependent effects	**0.09**, [0.03, 0.14],** 0.00, (7)	N/A	**0.12**, [0.03, 0.21],** 0.00, (4)	0.04, [-0.06, 0.13], 0.00, (2)	N/A
**TIMELINESS (MEASLES)**	No dependent effects	**0.23***, [0.14, 0.32],** 0.00, (2)	N/A	N/A	N/A	N/A
**TIMELINESS (COMPLETE IMMUNISATIONS)**	No dependent effects	**0.15***, [0.07, 0.24],** 9.66 (5)	N/A	**0.15*, [0.004, 0.29],** 0.00, (2)	0.38, [-0.28, 1.03], 72.02, (2)	N/A
**DROPOUTS**	Not powered	0.03, [-0.11, 0.16], 91.08, (5)	0.02, [-0.03, 0.06], 0.00, (2)	N/A	N/A	**0.18**, [0.06, 0.30],** 57.38, (2)
**MORBIDITY**	0.01, [-0.09, 0.10], 83.32, (10; 26)	0.01, [-0.06, 0.08], 82.82, (10)	-0.004, [-0.11, 0.10], 88.86, (5)	0.05, [-0.06, 0.15], 45.71, (2)	N/A	-0.10, [-0.60, 0.40], 80.88, (2)
**MORTALITY**	-0.04, [-0.09, 0.01], 69.91, (6; 25)	-0.04, [-0.09, 0.01], 74.07, (6)	-0.04, [-0.11, 0.04], 80.68, (3)	N/A	N/A	-0.04, [-0.10, 0.03], 62.75, (3)
**IMMUNISATION KNOWLEDGE**	0.17, [-0.02, 0.37], 87.30, (9; 13)	**0.19*, [0.07, 0.31],** 87.28, (9)	0.20, [-0.08, 0.49], 93.80, (4)	**0.31*, [0.04, 0.58],** 72.34, (3)	N/A	**0.09***, [0.04, 0.13],** 0.00, (2)
**IMMUNISATION ATTITUDES**	No dependent effects	0.14, [-0.03, 0.31], 90.09, (6)	0.47, [-0.19, 1.13], 96.36, (2)	-0.11, [-0.53, 0.30], 75.61, (2)	N/A	**0.06*, [0.01, 0.11],** 0.00, (2)
**VACCINATION CARD AVAILABILITY/RETENTION**	No dependent effects	-0.01, [-0.05, 0.02], 00.00, (4)	N/A	N/A	N/A	-0.01, [-0.05, 0.02], 00.00, (2)
**EXPERIENCE AND SATISFACTION WITH HEALTH SERVICES**	Not powered	0.04, [-0.015, .023], 48.22. (2)	N/A	N/A	N/A	N/A
**FORMAL HEALTH WORKER CAPACITY AND PERFORMANCE**	Not powered	0.11, [-0.07, 0.29], 0.00, (2)	N/A	N/A	N/A	N/A

Supplementary Table 3: Summary of all moderator analyses

For peer review only - http://bmjopen.bmj.com/site/about/guidelines.xhtml

	EXP INTMO	EVAL INMO	DESIGN	YEAR	REGION	CARD	POST INT V CFB	GOV IMPLEMENT	CADRES	VACCINE HESITENCY	BASELINE COVERAGE
	**B (SE)**	**B (SE)**	**B (SE)**	**B (SE)**	**B (SE)**	**B (SE)**	**B (SE)**	**B (SE)**	**B (SE)**	**B (SE)**	**B (SE)**

For peer review only

ALL EFFECTS RVE (K = 56)	-0.001 (0.003)	0.002 (0.006)	-0.10 (0.10)	-0.019* (0.007)	NP	0.06 (0.05)	-0.08 (0.05)	-0.08 (0.04)	-0.02 (0.05)	-0.04 (0.05)	-0.31 (0.15)
FULL IMMUNISATION RVE (K = 28)	0.0001 (0.002)	NP	-0.03 (0.09)	NP	NS (ALL COMPARISONS)	0.04 (0.11)	-0.16 (0.12)	-0.06 (0.10)	0.02 (0.09)	0.10 (0.19)	-0.13 (0.15)
FULL IMMUNISATION IND RE (K = 28)	-0.0002 (0.004)	-0.01 (0.02)	-0.05 (0.11)	-0.01 (0.01)	NS (ALL COMPARISONS)	0.04 (0.12)	-0.13 (0.10)	0.06 (0.12)	-0.06 (0.12)	0.17 (0.11)	-0.33 (0.25)
FULL IMMUNISATION IND EAI (K = 12)	-0.003* (0.002)	0.01 (0.01)	-0.06 (0.06)	0.01 (0.01)	0.02 (0.07)	-0.01 (0.06)	-0.04 (0.08)	0.08 (0.06)	-0.09 (0.06)	0.08 (0.07)	-0.06 (0.11)
FULL IMMUNISATION IND EID (K = 5)	N/A[C]	N/A[C]	N/A[C]	N/A[C]	N/A[C]	N/A[C]	N/A[C]	N/A[C]	N/A[C]	N/A[C]	N/A[C]
FULL IMMUNISATION IND EII (K = 2)	NP	NP	NP	NP	NP	NP	NP	NP	NP	NP	NP
FULL IMMUNISATION IND MET (K = 9)	0.005 (0.01)	-0.06 (0.06)	-0.12 (0.38)	-0.06 (0.03)	NS (ALL COMPARISONS)	N/A[B]	-0.49 (0.27)	0.04 (0.35)	N/A[B]	0.33 (0.31)	-0.66 (0.68)
PARTIAL IMMUNISATION RVE (K = 9)	NP	NP	NP	NP	NP	NP	0.10 (0.18)	NP	NP	0.27 (0.12)	N/A[E]
PARTIAL IMMUNISATION IND RE (K = 9)	-0.003 (0.006)	-0.01 (0.01)	-0.10 (0.19)	-0.03 (0.03)	-0.03 (0.19)	0.06 (0.21)	0.09 (0.18)	-0.30 (0.20)	-0.30 (0.19)	0.18 (0.19)	N/A[E]
PARTIAL IMMUNISATION IND EAI (K = 2)	NP	NP	NP	NP	NP	NP	NP	NP	NP	NP	N/A[E]
PARTIAL IMMUNISATION IND EID (K = 2)	NP	NP	NP	NP	NP	NP	NP	NP	NP	NP	N/A[E]
PARTIAL IMMUNISATION IND EII (K = 1)	NP	NP	NP	NP	NP	NP	NP	NP	NP	NP	N/A[E]
PARTIAL IMMUNISATION IND MET (K = 4)	0.01 (0.02)	-0.01 (0.01)	-0.10 (0.35)	-0.02 0.06)	0.05 (0.36)	N/A[B]	0.35 (0.33)	N/A[A]	N/A[B]	0.08 (0.35)	N/A[E]
MEASLES RVE (K = 20)	0.0001 (0.001)	NP	-0.03 (0.05)	-0.001 (0.004)	NP	0.01 (0.04)	-0.04 (0.03)	0.03 (0.03)	0.04 (0.04)	NP	N/A[E]
MEASLES IND RE (K = 20)	0.000 (0.001)	-0.003 (0.003)	-0.05 (0.06)	-0.003 (0.007)	NS (ALL COMPARISONS)	0.02 (0.07)	-0.04 (0.06)	0.06 (0.06)	0.05 (0.06)	0.13 (0.09)	N/A[D]
MEASLES: IND EAI (K = 10)	-0.001 (0.001)	-0.004 (0.006)	0.07 (0.05)	0.004 (0.01)	NS (ALL COMPARISONS)	0.02 (0.08)	0.05 (0.05)	-0.003 (0.05)	0.03 (0.05)	0.10 (0.09)	N/A[D]
MEASLES: IND EID (K = 2)	NP	NP	NP	NP	NP	NP	NP	NP	NP	NP	N/A[D]
MEASLES: IND EII (K = 2)	NP	NP	NP	NP	NP	NP	NP	NP	NP	NP	N/A[D]
MEASLES: IND MET (K = 6)	0.004 (0.01)	-0.004 (0.01)	-0.15 (0.31)	-0.04 (0.03)	0.18 (0.25)	0.07 (0.33)	-0.33 (0.19)	0.31 (0.20)	0.18 (0.25)	N/A[B]	N/A[D]

For peer review only - http://bmjopen.bmj.com/site/about/guidelines.xhtml

**BCG RVE (K = 12)**	NP	NP	-0.10 (0.10)	-0.03 (0.02)	-0.08 (0.12)	NP	-0.12 (0.10)	-0.10 (0.14)	NP	0.37*** (0.03)	N/A[D]
**BCG IND RE (K = 12)**	0.001 (0.004)	-0.01 (0.01)	-0.05 (0.11)	-0.03 ** (0.01)	0.004 (0.11)	-0.02 (0.04)	-0.12 (0.12)	-0.04 (0.13)	0.06 (0.13)	N/A[B]	N/A[D]
BCG: IND EAI (K = 4)	N/A[C]	N/A[C]	N/A[C]	N/A[C]	N/A[C]	N/A[C]	N/A[C]	N/A[C]	N/A[C]	N/A[C]	N/A[D]
BCG: IND EID (K = 2)	NP	NP	NP	NP	NP	NP	NP	NP	NP	NP	N/A[D]
BCG: IND EII (K = 2)	NP	NP	NP	NP	NP	NP	NP	NP	NP	NP	N/A[D]
**BCG: IND MET (K = 4)**	-0.002 (0.02)	N/A[B]	N/A[B]	-0.08 (0.04)	0.03 (0.41)	N/A[A]	-0.55*** (0.07)	0.03 (0.41)	0.03 (0.41)	N/A[B]	N/A[D]
**DPT1: RVE (K = 8)**	NP	NP	-0.10 (0.10)	0.001 (0.01)	NP	NP	NP	NP	NP	0.07 (0.03)	-0.13 (0.20)
**DPT1: IND RE (K = 8)**	-0.005 (0.01)	N/A[A]	-0.15* (0.07)	-0.004 (0.01)	NS (ALL COMPARI SONS)	-0.08 (0.10)	0.02 (0.11)	0.17* (0.08)	N/A[B]	N/A[B]	N/A[E]
DPT1: IND EAI (K = 3)	NP	NP	NP	NP	NP	NP	NP	NP	NP	NP	NP
DPT1: IND EID (K = 2)	NP	NP	NP	NP	NP	NP	NP	NP	NP	NP	NP
DPT1: IND EII (K = 1)	NP	NP	NP	NP	NP	NP	NP	NP	NP	NP	NP
DPT1: IND MET (K = 2)	NP	NP	NP	NP	NP	NP	NP	NP	NP	NP	NP
**DPT2 RVE (K = 5)**	NP	NP	NP	NP	NP	NP	NP	NP	NP	NP	NP
**DPT2 IND RE (K = 5)**	N/A[C]	N/A[C]	N/A[C]	N/A[C]	N/A[C]	N/A[C]	N/A[C]	N/A[C]	N/A[C]	N/A[C]	N/A[C]
DPT2 IND EAI (K = 0)	NP	NP	NP	NP	NP	NP	NP	NP	NP	NP	NP
DPT2 IND EID (K = 2)	NP	NP	NP	NP	NP	NP	NP	NP	NP	NP	NP
DPT2 IND EII (K = 0)	NP	NP	NP	NP	NP	NP	NP	NP	NP	NP	NP
DPT2 IND MET (K = 3)	NP	NP	NP	NP	NP	NP	NP	NP	NP	NP	NP
**DPT3 RVE (K = 22)**	0.0003 (0.001)	NP	-0.03 (0.05)	-0.02 (0.01)	-0.01 (0.05)	NP	-0.01 (0.05)	-0.04 (0.05)	NP	NP	-0.50 (0.21)
**DPT3 IND RE (K = 22)**	-0.001 (0.001)	-0.002 (0.004)	0.05 (0.05)	-0.014* (0.006)	-0.02 (0.05)	0.14 (0.09)	-0.01 (0.05)	-0.01 (0.05)	-0.03 (0.06)	0.04 (0.06)	-0.40 (0.22)
**DPT3 IND EAI (K = 6)**	-0.001 (0.002)	0.01 (0.01)	-0.05 (0.07)	0.01 (0.01)	N/A[B]	N/A[A]	-0.08 (0.07)	0.05 (0.07)	-0.04 (0.07)	-0.03 (0.08)	-0.40 (0.22)
DPT3 IND EID (K = 6)	N/A[C]	N/A[C]	N/A[C]	N/A[C]	N/A[C]	N/A[C]	N/A[C]	N/A[C]	N/A[C]	N/A[C]	N/A[C]
DPT3 IND EII (K = 3)	NP	NP	NP	NP	NP	NP	NP	NP	NP	NP	NP
**DPT3 IND MET (K =7)**	0.003 (0.006)	-0.01** (0.004)	0.07 (0.16)	-0.03* (0.01)	-0.10 (0.15)	N/A[A]	-0.25 (0.15)	0.05 (0.17)	-0.04 (0.17)	-0.02 (0.17)	-0.56** (0.18)
OPV0 RVE (K = 5)	NP	NP	NP	NP	NP	NP	NP	NP	NP	NP	NP
**OPV0 IND RE (K = 5)**	0.02*** (0.004)	N/A[A]	0.16 (0.19)	-0.07** (0.02)	-0.20 (0.18)	0.16 (0.19)	N/A[B]	-0.21 (0.17)	N/A[A]	N/A[A]	N/A[D]
OPV0 IND EAI (K = 1)	NP	NP	NP	NP	NP	NP	NP	NP	NP	NP	N/A[D]
OPV0 IND EID (K = 2)	NP	NP	NP	NP	NP	NP	NP	NP	NP	NP	N/A[D]
OPV0 IND EII (K = 1)	NP	NP	NP	NP	NP	NP	NP	NP	NP	NP	N/A[D]
OPV0 IND MET (K = 1)	NP	NP	NP	NP	NP	NP	NP	NP	NP	NP	N/A[D]
OPV1 RVE (K = 5)	NP	NP	NP	NP	NP	NP	NP	NP	NP	NP	N/A[D]

For peer review only - http://bmjopen.bmj.com/site/about/guidelines.xhtml

OPV1 IND RE (K = 5)	N/A[C]	N/A[C]	N/A[C]	N/A[C]	N/A[C]	N/A[C]	N/A[C]	N/A[C]	N/A[C]	N/A[C]	N/A[D]
OPV01 IND EAI (K = 0)	NP	NP	NP	NP	NP	NP	NP	NP	NP	NP	N/A[D]
OPV1 IND EID (K = 2)	NP	NP	NP	NP	NP	NP	NP	NP	NP	NP	N/A[D]
OPV1 IND EII (K = 1)	NP	NP	NP	NP	NP	NP	NP	NP	NP	NP	N/A[D]
OPV1 IND MET (K = 2)	NP	NP	NP	NP	NP	NP	NP	NP	NP	NP	N/A[D]
OPV2 RVE (K = 5)	NP	NP	NP	NP	NP	NP	NP	NP	NP	NP	N/A[D]
OPV2 IND RE (K = 5)	0.02*** (0.003)	N/A[A]	0.22 (0.18)	-0.05** (0.02)	-0.26*** (0.07)	0.27 (0.07)	N/A[B]	0.03 (0.25)	N/A[B]	N/A[A]	N/A[D]
OPV02 IND EAI (K = 0)	NP	NP	NP	NP	NP	NP	NP	NP	NP	NP	N/A[D]
OPV2 IND EID (K = 2)	NP	NP	NP	NP	NP	NP	NP	NP	NP	NP	N/A[D]
OPV2 IND EII (K = 1)	NP	NP	NP	NP	NP	NP	NP	NP	NP	NP	N/A[D]
OPV2 IND MET (K = 2)	NP	NP	NP	NP	NP	NP	NP	NP	NP	NP	N/A[D]
OPV3 RVE (K = 9)	NP	NP	-0.03 (0.05)	NP	-0.24 (0.31)	NP	0.26 (0.30)	NP	-0.18 (0.33)	NP	N/A[D]
OPV3 IND RE (K = 9)	-0.001 (0.01)	-0.01 (0.01)	0.17 (0.23)	-0.03 (0.03)	-0.12 (0.23)	0.04 (0.32)	0.14 (0.25)	-0.34 (0.27)	-0.03 (0.27)	N/A[A]	N/A[D]
OPV3 IND EAI (K = 3)	NP	NP	NP	NP	NP	NP	NP	NP	NP	NP	N/A[D]
OPV3 IND EID (K = 1)	NP	NP	NP	NP	NP	NP	NP	NP	NP	NP	N/A[D]
OPV3 IND EII (K = 2)	NP	NP	NP	NP	NP	NP	NP	NP	NP	NP	N/A[D]
OPV3 IND MET (K = 3)	NP	NP	NP	NP	NP	NP	NP	NP	NP	NP	N/A[D]
TIMELINESS RVE (K = 11)	NP	NP	NP	NP	NP	NP	NP	NP	NP	NP	N/A[D]
DPT3 TIMELINESS RE (K = 7)	N/A[C]	N/A[C]	N/A[C]	N/A[C]	N/A[C]	N/A[C]	N/A[C]	N/A[C]	N/A[C]	N/A[C]	N/A[C]
DPT3 TIMELINESS EAI (K = 0)	---	---	---	---	---	---	---	---	---	---	---
DPT3 TIMELINESS EID (K = 4)	N/A[C]	N/A[C]	N/A[C]	N/A[C]	N/A[C]	N/A[C]	N/A[C]	N/A[C]	N/A[C]	N/A[C]	N/A[C]
DPT3 TIMELINESS EII (K =2)	NP	NP	NP	NP	NP	NP	NP	NP	NP	NP	NP
DPT3 TIMELINESS MET (K = 1)	NP	NP	NP	NP	NP	NP	NP	NP	NP	NP	NP
MEASLES TIMELINESS RE (K = 2)	NP	NP	NP	NP	NP	NP	NP	NP	NP	NP	NP
MEASLES TIMELINESS EAI (K = 0)	---	---	---	---	---	---	---	---	---	---	---
MEASLES TIMELINESS EID (K = 1)	NP	NP	NP	NP	NP	NP	NP	NP	NP	NP	NP
MEASLES TIMELINESS EII (K =0)	---	---	---	---	---	---	---	---	---	---	---
MEASLES TIMELINESS MET (K = 1)	NP	NP	NP	NP	NP	NP	NP	NP	NP	NP	NP
FI TIMELINESS RE (K =5)	N/A[C]	N/A[C]	N/A[C]	N/A[C]	N/A[C]	N/A[C]	N/A[C]	N/A[C]	N/A[C]	N/A[C]	N/A[C]
FI TIMELINESS EAI (K = 1)	NP	NP	NP	NP	NP	NP	NP	NP	NP	NP	NP
FI TIMELINESS EID (K = 2)	NP	NP	NP	NP	NP	NP	NP	NP	NP	NP	NP
FI TIMELINESS EII (K = 2)	NP	NP	NP	NP	NP	NP	NP	NP	NP	NP	NP
FI TIMELINESS MET (K = 0)	---	---	---	---	---	---	---	---	---	---	---
DROPOUT RVE (K = 5)	NP	NP	NP	NP	NP	NP	NP	NP	NP	NP	NP
DROPOUT RE (K = 5)	0.04 (0.04)	0.03 (0.03)	N/A[A]	0.33** (0.12)	-0.050 (0.183)	N/A[B]	N/A[A]	-0.23 (0.13)	N/A[A]	0.05 (0.18)	N/A[D]
DROPOUT EAI (K =2)	NP	NP	NP	NP	NP	NP	NP	NP	NP	NP	NP
DROPOUT EID (K = 0)	---	---	---	---	---	---	---	---	---	---	---

For peer review only - http://bmjopen.bmj.com/site/about/guidelines.xhtml

DROPOUT EII (K =1)	NP	NP	NP	NP	NP	NP	NP	NP	NP	NP	NP
DROPOUT MET (K = 2)	NP	NP	NP	NP	NP	NP	NP	NP	NP	NP	NP
**MORBIDITY RVE (K = 10)**	NP	NP	NP	**0.01 (0.01)**	NP	N/A[E]	**0.02 (0.08)**	NP	NP	N/A[A]	N/A[E]
**MORBIDITY RE (K = 10)**	**0.003 (0.005)**	**-0.01 (0.01)**	**-0.04 0.11**	**0.01 (0.01)**	**NS (ALL COMPARISONS)**	**-0.08 (0.10)**	**0.13 (0.15)**	**0.08 (0.11)**	**-0.05 (0.13)**	N/A[A]	N/A[E]
**MORBIDITY EAI (K = 5)**	**0.02** (0.01)**	**-0.01 (0.02)**	N/A[B]	**0.03 (0.05)**	**0.04 (0.22)**	N/A[D]	**0.12 (0.21)**	**0.26 (0.22)**	**0.12 (0.27)**	N/A[A]	N/A[E]
MORBIDITY EID (K = 2)	NP	NP	NP	NP	NP	NP	NP	NP	NP	NP	NP
MORBIDITY EII (K = 1)	NP	NP	NP	NP	NP	NP	NP	NP	NP	NP	NP
MORBIDITY MET (K = 2)	NP	NP	NP	NP	NP	NP	NP	NP	NP	NP	NP
**MORTALITY: RVE (K = 6)**	NP	NP	NP	NP	NP	N/A[E]	NP	NP	NP	N/A[A]	N/A[D]
**MORTALITY RE (6)**	**-0.002 (0.001)**	**0.002 (0.002)**	**0.03 (0.05)**	**0.01*** (0.003)**	**NS (ALL COMPARISONS)**	N/A[D]	**-0.05 (0.05)**	**0.02 (0.05)**	**0.02 (0.05)**	N/A[A]	N/A[D]
**MORTALITY EAI (3)**	NP	NP	NP	NP	NP	NP	NP	NP	NP	NP	NP
**MORTALITY EID (0)**	NP	NP	NP	NP	NP	NP	NP	NP	NP	NP	NP
**MORTALITY EII (0)**	NP	NP	NP	NP	NP	NP	NP	NP	NP	NP	NP
**MORTALITY MET (3)**	NP	NP	NP	NP	NP	NP	NP	NP	NP	NP	NP
**KNOWLEDGE RVE (K = 7)**	NP	NP	**-0.10 (0.10)**	NP	NP	NP	**-0.06 (0.13)**	NP	NP	NP	N/A[D]
**KNOWLEDGE RE (K = 9)**	**-0.02 (0.01)**	**-0.01 (0.02)**	**0.44*** (0.11)**	**-0.01 (0.02)**	**0.44*** (0.11)**	N/A[D]	**-0.05 (0.18)**	**0.05 (0.23)**	N/A[A]	**-0.01 (0.23)**	N/A[D]
**KNOWLEDGE EAI (K = 4)**	**N/A[E]**	**N/A[E]**	**0.52* (0.21)**	**-0.05 (0.05)**	**0.52* (0.21)**	**N/A[E]**	**N/A[B]**	**N/A[B]**	**N/A[A]**	**N/A[A]**	N/A[D]
KNOWLEDGE EID (K = 3)	NP	NP	NP	NP	NP	NP	NP	NP	NP	NP	NP
KNOWLEDGE EII (K = 0)	---	---	---	---	---	---	---	---	---	---	---
KNOWLEDGE MET (K = 2)	NP	NP	NP	NP	NP	NP	NP	NP	NP	NP	NP
ATTITUDES RVE (NO DEPENDENT EFFECTS)	N/A	N/A	N/A	N/A	N/A	N/A	N/A	N/A	N/A		
**ATTITUDES RE (K = 6)**	**0.01 (0.02)**	**-0.004 (0.02)**	**0.17 (0.34)**	**-0.01 (0.04)**	**0.17 (0.34)**	N/A[E]	**0.38 (0.29)**	**-0.09 (0.43)**	N/A[A]	N/A[B]	N/A[D]
ATTITUDES EAI (K = 2)	NP	NP	NP	NP	NP	NP	NP	NP	NP	NP	NP
ATTITUDES EID (K = 2)	NP	NP	NP	NP	NP	NP	NP	NP	NP	NP	NP
ATTITUDES EII (K = 0)	---	---	---	---	---	---	---	---	---	---	---
ATTITUDES MET (K = 2)	NP	NP	NP	NP	NP	NP	NP	NP	NP	NP	NP
CARD AVAIL/ RETENTION RVE (NO DEPENDENT EFFECTS)	N/A	N/A	N/A	N/A	N/A	N/A	N/A	N/A	N/A	N/A	N/A
CARD AVAIL/ RETENTION RE (K = 4)	N/A[C]	N/A[C]	N/A[C]	N/A[C]	N/A[C]	N/A[C]	N/A[C]	N/A[C]	N/A[C]	N/A[C]	N/A[D]
CARD AVAIL/ RETENTION EAI (K = 1)	NP	NP	NP	NP	NP	NP	NP	NP	NP	NP	NP
CARD AVAIL/ RETENTION EID (K = 0)	---	---	---	---	---	---	---	---	---	---	---

For peer review only

CARD AVAIL/ RETENTION EII (K = 1)	NP	NP	NP	NP	NP	NP	NP	NP	NP	NP	NP
CARD AVAIL/ RETENTION MET (K = 2)	NP	NP	NP	NP	NP	NP	NP	NP	NP	NP	NP
SATISFACTION W/ HEALTH WORKERS (K = 2)	NP	NP	NP	NP	NP	NP	NP	NP	NP	NP	NP
SATISFACTION W/ HEALTH WORKERS (K = 1)	NP	NP	NP	NP	NP	NP	NP	NP	NP	NP	NP
SATISFACTION W/ HEALTH WORKERS EAI (K = 1)	NP	NP	NP	NP	NP	NP	NP	NP	NP	NP	NP
SATISFACTION W/ HEALTH WORKERS EID (K = 1)	NP	NP	NP	NP	NP	NP	NP	NP	NP	NP	NP
SATISFACTION W/ HEALTH WORKERS EII (K = 0)	--	--	--	--	--	--	--	--	--	--	--
SATISFACTION W/ HEALTH WORKERS MET (K = 0)	--	--	--	--	--	--	--	--	--	--	--
FORMAL HEALTH WORKERS MOTIVATION, CAPACITY, AND PERFORMANCE (K = 2)	NP	NP	NP	NP	NP	NP	NP	NP	NP	NP	NP
FORMAL HEALTH WORKERS MOTIVATION, CAPACITY, AND PERFORMANCE (K = 1)	NP	NP	NP	NP	NP	NP	NP	NP	NP	NP	NP
FORMAL HEALTH WORKERS MOTIVATION, CAPACITY, AND PERFORMANCE EAI (K = 1)	NP	NP	NP	NP	NP	NP	NP	NP	NP	NP	NP
FORMAL HEALTH WORKERS MOTIVATION, CAPACITY, AND PERFORMANCE EID (K = 1)	NP	NP	NP	NP	NP	NP	NP	NP	NP	NP	NP
FORMAL HEALTH WORKERS MOTIVATION, CAPACITY, AND PERFORMANCE EII (K = 0)	--	--	--	--	--	--	--	--	--	--	--
FORMAL HEALTH WORKERS MOTIVATION, CAPACITY, AND PERFORMANCE MET (K = 0)	--	--	--	--	--	--	--	--	--	--	--

Note. NS = non-significant, NP = not powered, EAI = engagement as the intervention, EID = engagement in the intervention design, EII = engagement in implementation autonomy, MET = multiple engagement types, RVE = robust variance estimation, RE = random effects model using independent effects.

[a] Not applicable because all studies were in the same moderator group

For peer review only

[b] Not applicable because only one study was in a different moderator group

[c] Not applicable because there was no heterogeneity

[d] Not applicable because the moderator does not apply to the outcome category

[e] Not applicable because there was missing data

* $p < .05$, ** $p < .01$, *** $p < .001$

For peer review only

For peer review only - http://bmjopen.bmj.com/site/about/guidelines.xhtml

Appendix 10: Quantitative results – Engagement as intervention

Appendix 10 presents supplementary data for analyses presented in the main body of the manuscript for the primary outcomes of community engagement interventions using engagement as the intervention, including funnel plots of publication bias and outlier analyses. It also includes a full presentation of the supplementary outcomes that were precluded from the main body of the manuscript due to word limitations.

Full immunisation

Studies that used engagement as the intervention had a significant effect on full childhood immunisation ($\hat{\mu} = 0.08$ [95% CI: 0.03 to 0.13], $z = 3.02$, $p < 0.01$). According to the Q-test, the true outcomes appear to be heterogeneous ($Q(11) = 36.67$, $p < 0.01$, $\hat{\tau}^2 = 0.005$, $I^2 = 70.00\%$). An examination of the studentized residuals revealed that none of the studies had a value larger than ± 2.87 and hence there was no indication of outliers in the context of this model. According to the Cook's distances, none of the studies could be considered to be overly influential. The forest plot is presented below (Supplemenary Figure 33). With only one high or moderate quality study, sensitivity analysis by study quality could not be completed for this body of evidence. A funnel plot of the estimates is shown in Figure B1B. Neither the rank correlation nor the regression test indicated any funnel plot asymmetry ($p = 0.20$ and $p = 0.07$, respectively). Exposure to the intervention (in months) was the only significant source of heterogeneity such that for each additional month of intervention exposure, effects decreased by .003 standard deviation units ($\hat{B} = -0.003, p = 0.049$ [95% CI: -0.01 to -0.00001]).

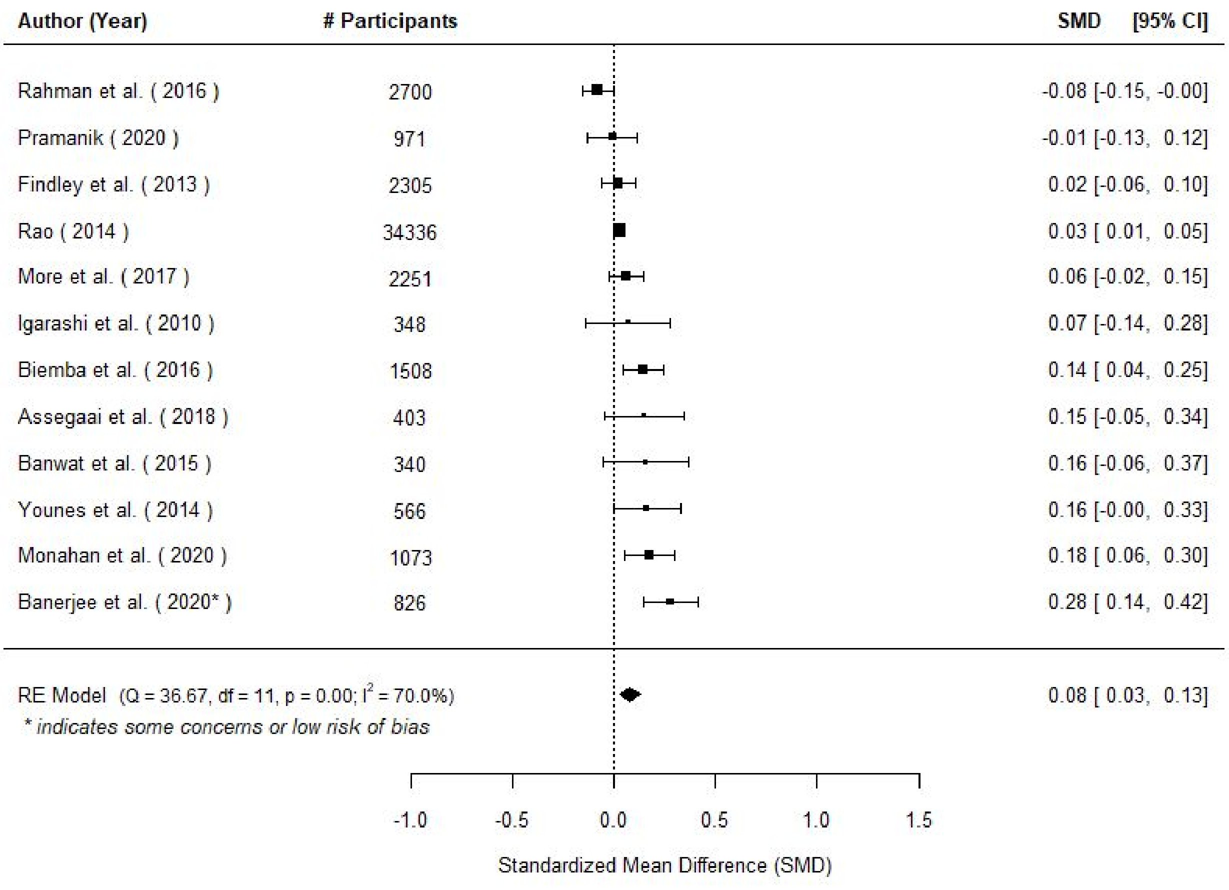

Supplementary Figure 33. Forest plot showing the observed outcomes and the estimate of the random-effects model for interventions with community engagement as the intervention on full immunisation

For peer review only - http://bmjopen.bmj.com/site/about/guidelines.xhtml

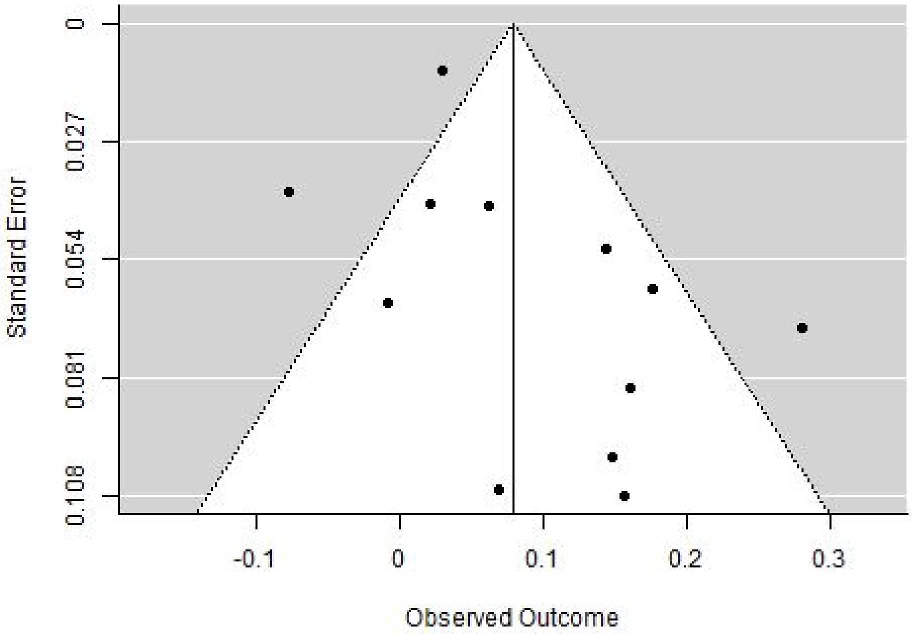

Supplementary Figure 33b. Forest plot showing the observed outcomes and the estimate of the random-effects model for interventions with community engagement as the intervention on full immunisation

DPT3

Studies that used engagement as the intervention also had a significant effect on DPT3 vaccination ($\hat{\mu} = 0.09$ [95% CI: 0.03 to 0.15], $z = 3.01$, $p < 0.01$). According to the Q-test, the true outcomes appear to be heterogeneous ($\mathrm{Q}(5) = 18.64$, $\mathrm{p} < 0.01$, $\hat{\tau}^2 = 0.00$, $\mathrm{I}^2 = 73.17\%$). An examination of the studentized residuals revealed that none of the studies had a value larger than ± 2.64 and hence there was no indication of outliers in the context of this model. According to the Cook's distances, none of the studies could be considered to be overly influential. None of the moderators we tested were significant sources of heterogeneity (see Supplementary Table 3 in Appendix 9).

For peer review only - http://bmjopen.bmj.com/site/about/guidelines.xhtml

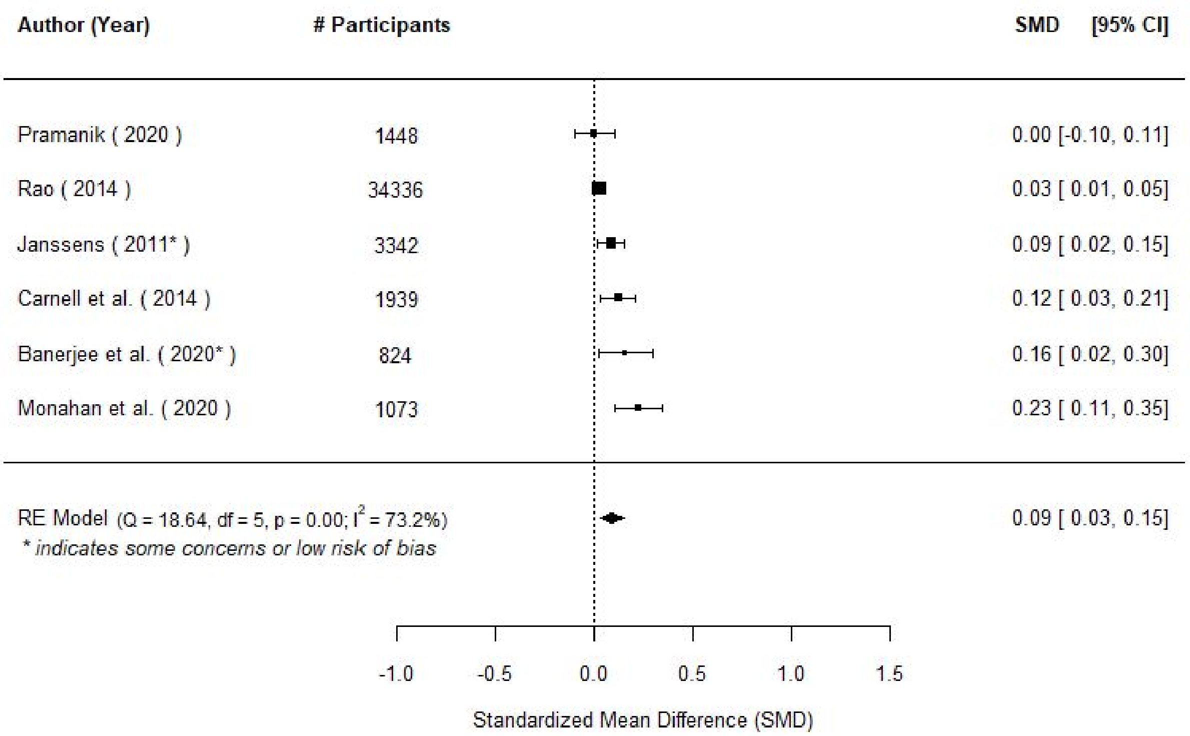

Supplementary Figure 34. Forest plot showing the observed outcomes and the estimate of the random-effects model for interventions with community engagement as the intervention on DPT3

Measles

Studies that used engagement as the intervention also had a significant effect on measles vaccination ($\hat{\mu} = 0.10$ [95% CI: 0.05 to 0.15], $z = 3.89$, $p < 0.001$). According to the Q-test, the true outcomes appear to be heterogeneous ($Q(9) = 22.67$, $p = 0.007$, $\hat{\tau}^2 = 0.003$, $I^2 = 60.29\%$). An examination of the studentized residuals revealed that none of the studies had a value larger than ± 2.81 and hence there was no indication of outliers in the context of this model. According to the Cook's distances, none of the studies could be considered to be overly influential.

A funnel plot of the estimates is shown in Figure 35b. The regression test indicated funnel plot asymmetry ($p < 0.01$) but not the rank correlation test ($p = 0.38$). None of the moderators we tested were significant sources of heterogeneity (see Supplementary Table 3 in Appendix 9).

For peer review only - http://bmjopen.bmj.com/site/about/guidelines.xhtml

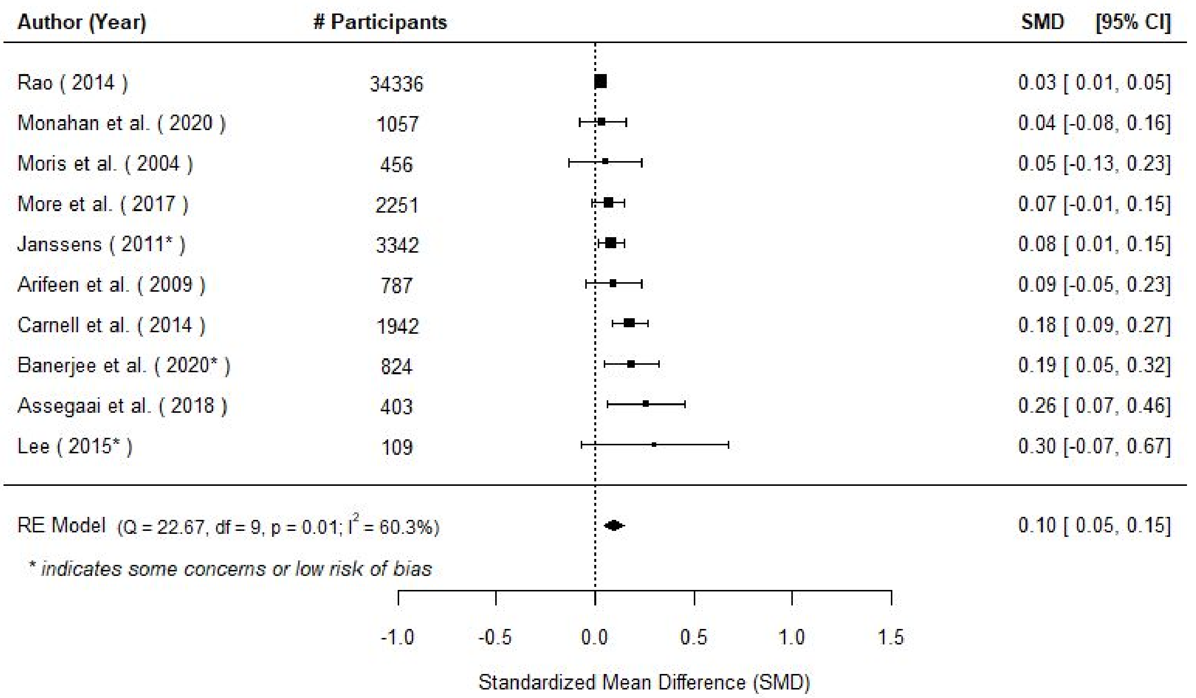

Supplementary Figure 35. Forest plot showing the observed outcomes and the estimate of the random-effects model for interventions with community engagement as the intervention on measles

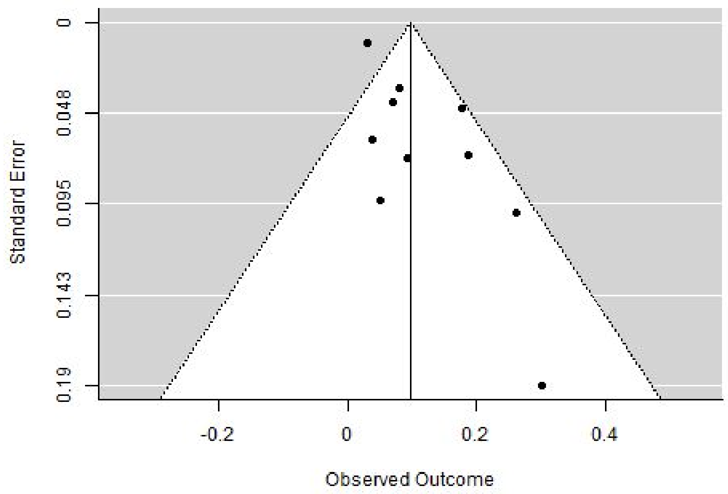

Supplementary Figure 35b. Funnel plot showing studies of community engagement as the intervention on measles

Timeliness of full childhood immunisation

Only one study (Saggurti 2018) fell into this intervention/outcome category, thus we were unable to perform a statistical synthesis. This quasi-experimental study from India found a small but significant impact of their programme on full childhood immunisation (g = 0.15 [95% CI: 0.04 to 0.27]), but like most studies, it was assessed as having a high risk of bias.

For peer review only - http://bmjopen.bmj.com/site/about/guidelines.xhtml

Timeliness of DPT3 vaccination

No studies examining timeliness of DPT3 vaccinations used engagement as the intervention.

Timeliness of measles vaccination

No studies examining timeliness of measles vaccinations used engagement as the intervention.

Secondary outcomes

Partial immunisation

Only two studies reporting on partial immunisation used interventions with community engagement as the intervention. The estimated average outcome based on the random-effects model was $\hat{\mu} = 0.31$ ([95% CI: -0.25 to $0.87]$, $z = 1.10$, $p = 0.27$), indicating no significant difference between the intervention group and the control group on partial immunisation (see Supplementary Figure 36). Given the small number of studies, this result should be interpreted with caution. According to the Q-test, the true outcomes appear to be heterogeneous ($Q(1) = 95.93$, $p < 0.001$, $\hat{\tau}^2 = 0.16$, $I^2 = 98.96\%$). With only two studies, moderator analyses were not appropriate and tests of publication bias are not valid.

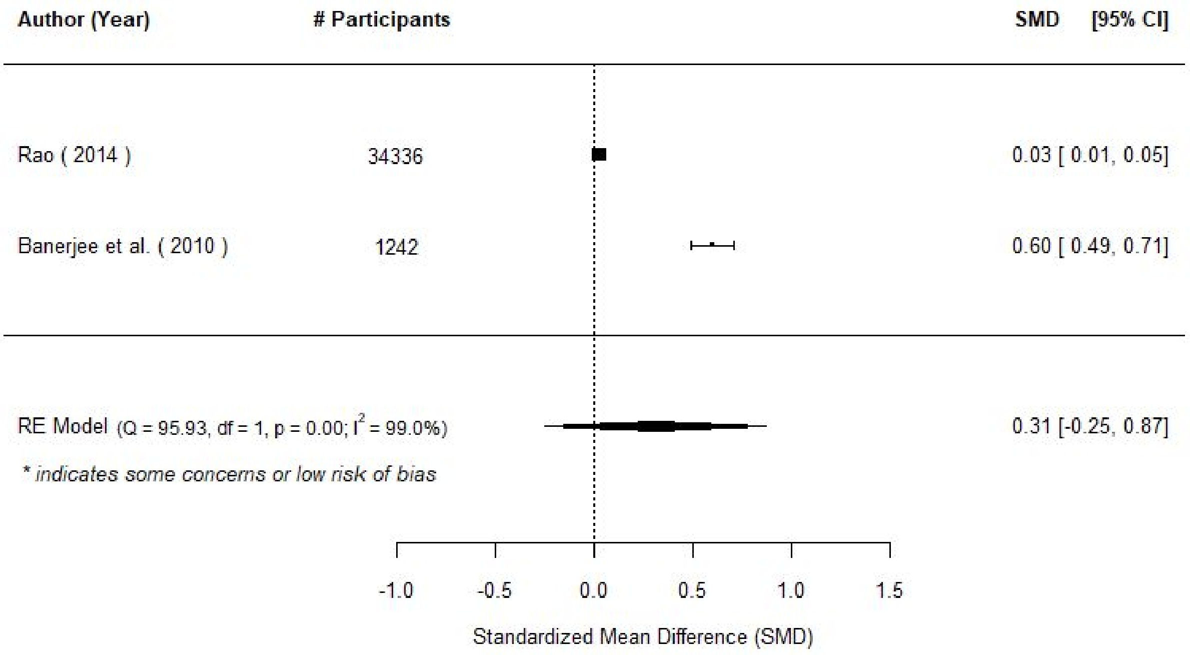

Supplementary Figure 36. Forest plot showing the observed outcomes and the estimate of the random-effects model for interventions with community engagement as the intervention on partial immunisation.

BCG

For peer review only - http://bmjopen.bmj.com/site/about/guidelines.xhtml

We included a total of $k = 4$ studies the analysis. The estimated average outcome based on the random-effects model was $\hat{\mu} = 0.02$ (95% CI: 0.01 to 0.03). Therefore, the average outcome differed significantly from zero ($z = 3.32$, $p < 0.01$), indicating a very small but significant benefit to the treated group compared to the control group (see Supplementary Figure 37). According to the Q-test, there was no significant amount of heterogeneity in the true outcomes ($Q(3) = 2.98$, $p = 0.39$, $\hat{\tau}^2 = 0.00$, $I^2 = 0.00\%$), thus we did not examine potential sources of heterogeneity for this model. An examination of the studentized residuals revealed that none of the studies had a value larger than ± 2.50 and hence there was no indication of outliers in the context of this model. According to the Cook's distances, none of the studies could be considered to be overly influential.

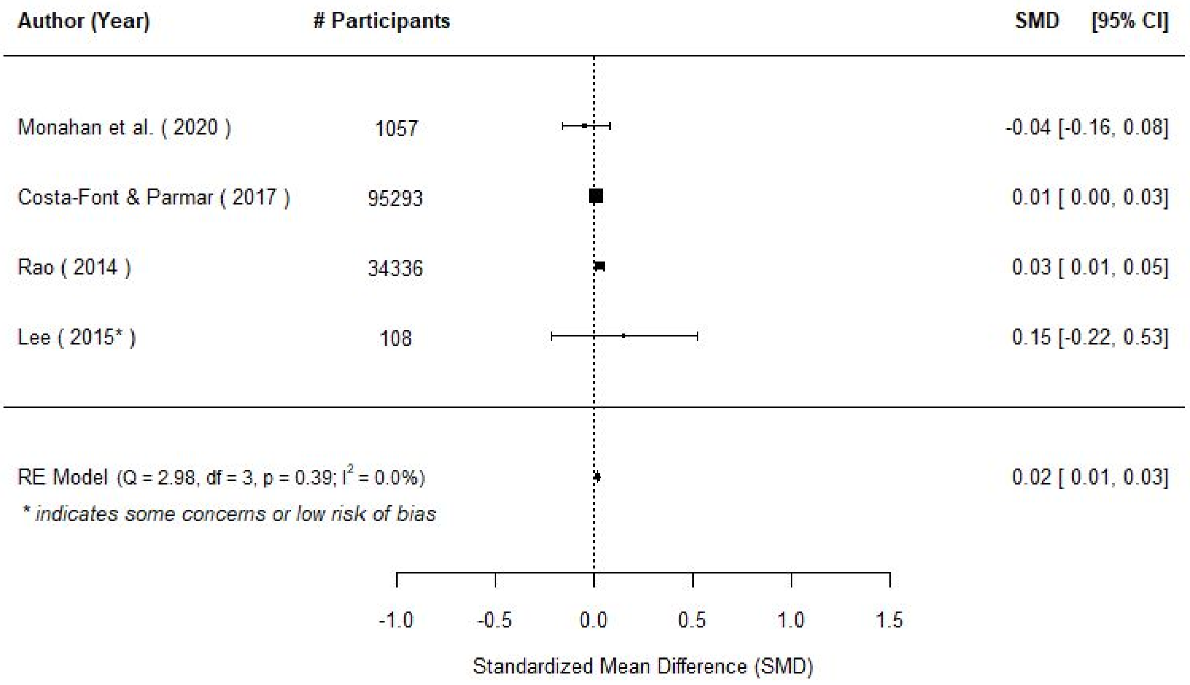

Supplementary Figure 37. Forest plot showing the observed outcomes and the estimate of the random-effects model for interventions with community engagement as the intervention on BCG vaccination.

DPT1

We included a total of $k = 3$ studies in the analysis. The estimated average outcome based on the random-effects model was $\hat{\mu} = 0.10$ ([95% CI: -0.03 to 0.22], $z = 1.53$, $p = 0.13$), indicating no difference between the treatment and control groups (see Supplementary Figure 38). According to the Q-test, the true outcomes appear to be heterogeneous ($Q(2) = 20.42$, $p < 0.01$, $\hat{\tau}^2 = 0.01$, $I^2 = 90.21\%$). An examination of the studentized residuals revealed that one study (Costa-Font & Parmar 2017) had a value larger than ± 2.39 and may be a potential outlier in the context of this model. Indeed, sensitivity analyses leaving each study out indicated that removing Costa-Font & Parmar (2017) would increase the overall average effect ($\hat{\mu} = 0.16$ (95% CI: 0.09 to 0.22), and the resulting effect would be positive and significant ($z = 4.72$, $p < .001$). According to the Cook's distances, none of the studies could be considered to be overly influential. With only three studies, we were unable to conduct moderator analyses or test for publication bias. Only one study (Banerjee et al. 2020) was not assessed as high risk of bias.

For peer review only - http://bmjopen.bmj.com/site/about/guidelines.xhtml

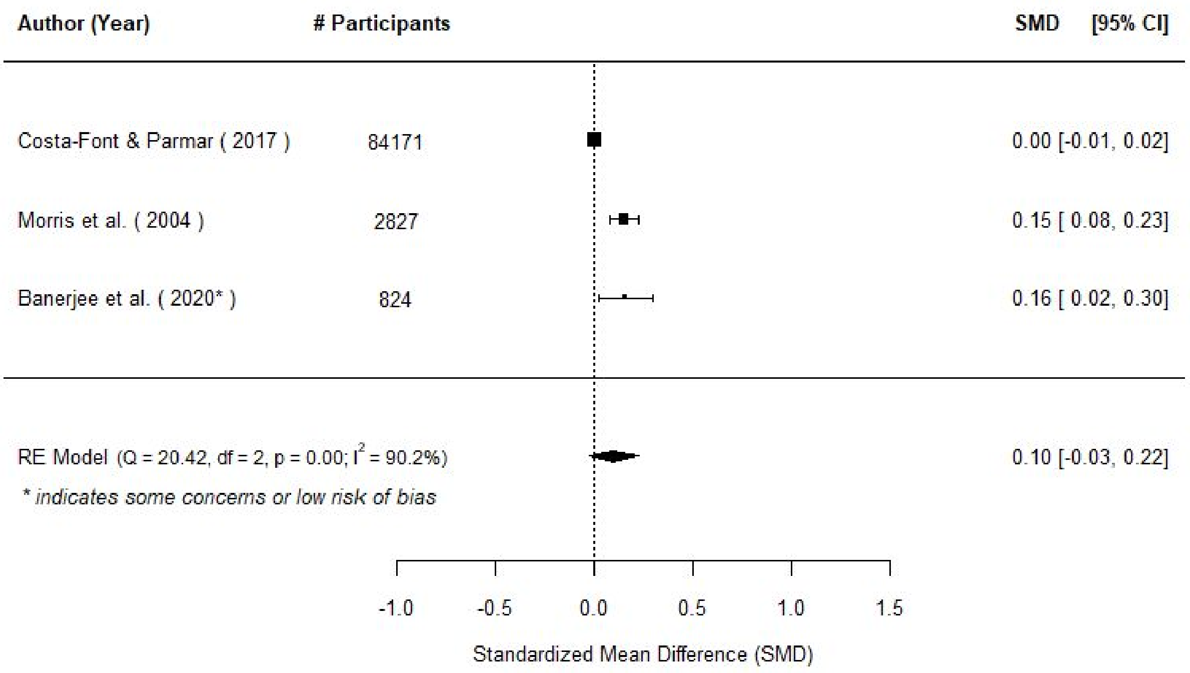

Supplementray Figure 38. Forest plot showing the observed outcomes and the estimate of the random-effects model for interventions with community engagement as the intervention on DPT1 vaccination.

DPT2

Only one study (Banerjee et al. 2020) fell into this intervention/outcome category, thus we were unable to perform a statistical synthesis. This cluster RCT from India found a small but significant positive effect of their programme on DPT2 vaccination (g = 0.15 [95% CI: 0.01 to 0.29]), but like most studies, it was assessed as having a high risk of bias.

OPV0

Only one study (Costa-Font & Parmar 2017) fell into this intervention/outcome category, thus we were unable to perform a statistical synthesis. This quasi-experimental study from India found a null effect of their programme on OPV0 vaccination (g = 0.01 [95% CI: -0.01 to 0.03]), but like most studies, it was assessed as having a high risk of bias.

OPV1

There were no studies reporting on OPV1 vaccination that used interventions with community engagement as the intervention.

OPV2

There were no studies reporting on OPV2 vaccination that used interventions with community engagement as the intervention.

For peer review only - http://bmjopen.bmj.com/site/about/guidelines.xhtml

OPV3

We included a total of $k = 3$ studies in the analysis. The estimated average outcome based on the random-effects model was $\hat{\mu} = 0.16$ ([95% CI: -0.02 to 0.34], $z = 1.78$, $p = 0.07$), indicating no significant difference between the intervention group and the control group on OPV3 vaccination (see Supplementary Figure 39). According to the Q-test, the true outcomes appear to be heterogeneous ($Q(2) = 12.37$, $p = 0.002$, $\hat{\tau}^2 = 0.02$, $^2 = 83.83\%$). An examination of the studentized residuals revealed that one study (Lee 2015) had a value larger than ± 2.39 and may be a potential outlier in the context of this model. Indeed, sensitivity analyses leaving each study out indicated that removing Lee (2015) would reduce the overall average effect ($\hat{\mu} = 0.08$ [95% CI: -0.04 to 0.19]), though in either case the effect is not significantly different from zero ($z = 1.29$, $p = 0.20$). According to the Cook's distances, none of the studies could be considered to be overly influential. With only three studies contributing effects, moderator analyses were not appropriate.

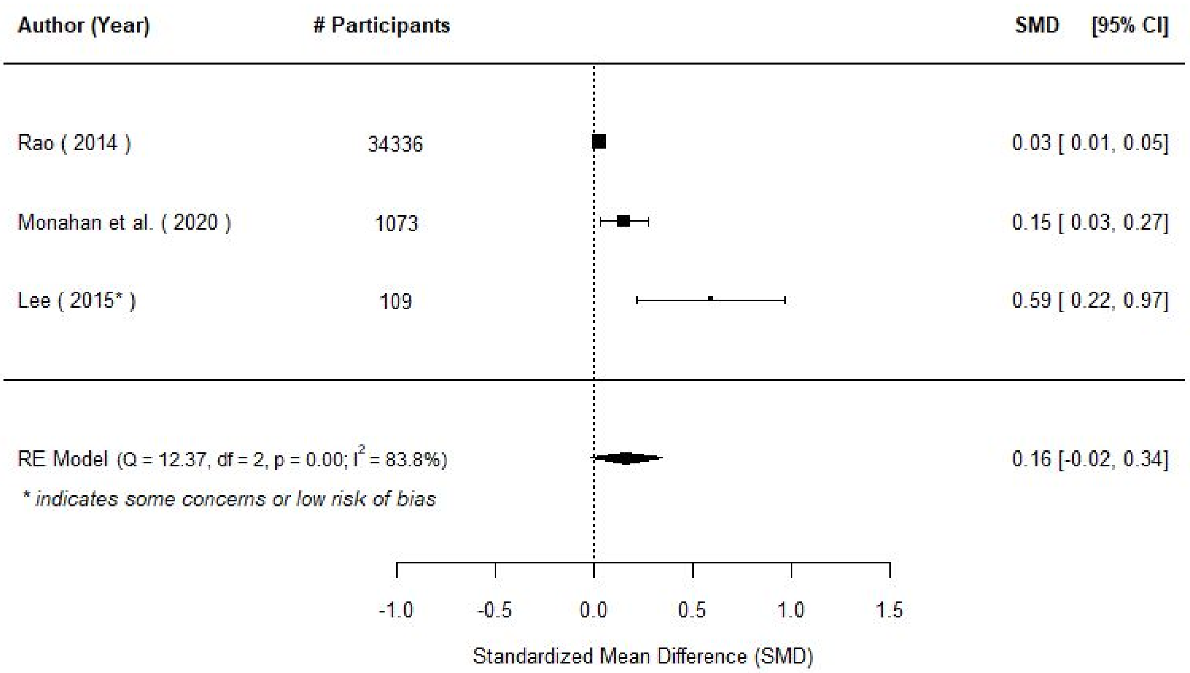

Supplementary Figure 39. Forest plot showing the observed outcomes and the estimate of the random-effects model for interventions with community engagement as the intervention on OPV3 vaccination.

Dropouts

Only two studies reporting on vaccination dropouts used interventions with community engagement as the intervention. The estimated average outcome based on the random-effects model was $\hat{\mu} = 0.02$ ([95% CI: -0.03 to 0.06], $z = 0.63$, $p = 0.53$), indicating no significant difference between the intervention group and the control group on vaccination dropouts (see Supplementary Figure 40). Given the small number of studies, this result should be interpreted with caution. According to the Q-test, the true outcomes appear to be homogeneous ($\mathrm{Q}(1) = 0.03$, $\mathrm{p} = 0.86$, $\hat{\tau}^2 = 0.00$, $\mathrm{I}^2 = 0.00\%$). With only two studies and no heterogeneity, moderator analyses were not appropriate and tests of publication bias are not valid.

For peer review only - http://bmjopen.bmj.com/site/about/guidelines.xhtml

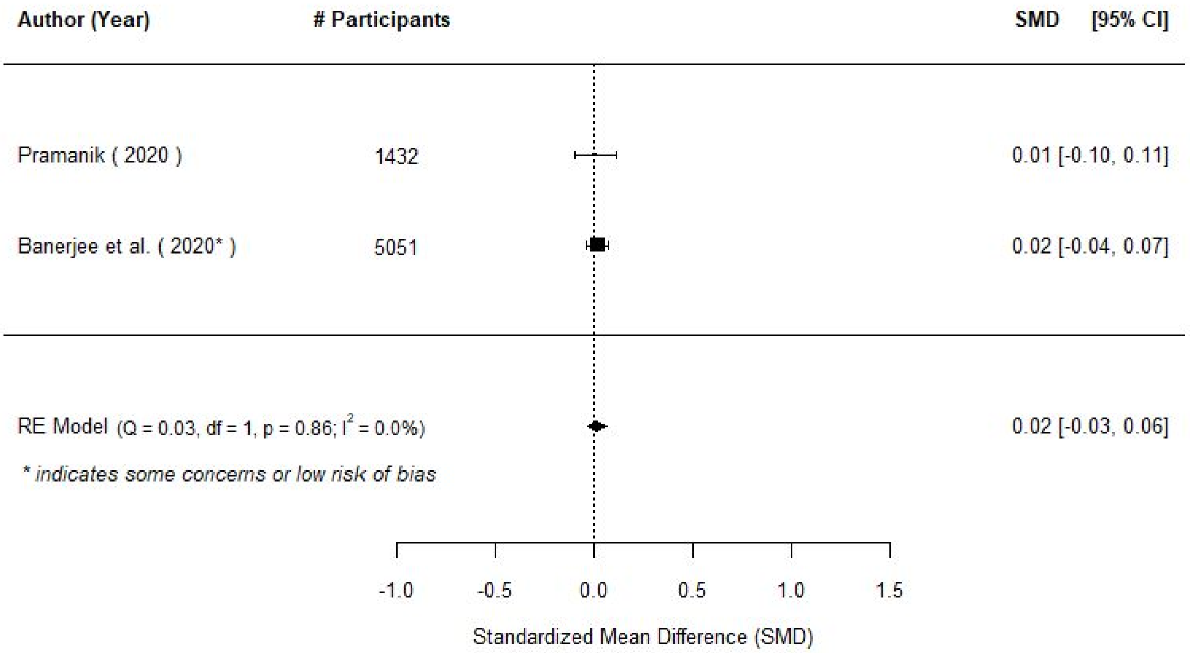

Supplementary Figure 40. Forest plot showing the observed outcomes and the estimate of the random-effects model for interventions with community engagement as the intervention on vaccination dropouts.

Morbidity

We used reports of diarrhea (most typically in the past two weeks) as a proxy for childhood morbidity. In all cases, effects were reverse coded such that positive effects always indicate a benefit to the treated group. Thus, a positive effect here would be interpreted as a reduction in diarrhea among treated participants compared to control participants. We included a total of $k = 5$ studies in the analysis. The estimated average outcome based on the random-effects model was $\hat{\mu} = -0.004$ (95% CI: -0.11 to 0.10). Therefore, the average outcome did not differ significantly from zero ($z = -0.08$, $p = 0.94$), indicating no difference between the treatment and control groups on childhood morbidity (see Supplementary Figure 41). According to the Q-test, the true outcomes appear to be heterogeneous ($Q(4) = 35.91$, $p < 0.01$, $\hat{\tau}^2 = 0.01$, $I^2 = 88.86\%$). An examination of the studentized residuals revealed that one study (Assegaai et al. 2018) had a value larger than ± 2.58 and may be a potential outlier in the context of this model. Indeed, sensitivity analyses leaving each study out indicated that removing Assegaai and colleagues (2018) would reduce the overall average effect ($\hat{\mu}$ = -0.06 (95% CI: -0.14 to 0.02). While the resulting effect would be negative, the effect would still be non-significant (z = -1.50, p = .13). According to the Cook's distances, none of the studies could be considered to be overly influential. There was only one study not assessed as high risk of bias, so we were unable to do a sensitivity analysis by study quality. Exposure to the intervention was a significant source of heterogeneity such that each additional month of exposure increased the positive impact on morbidity by 0.02 standard deviation units $\hat{\mu} = 0.02$ (95% CI: 0.01 to 0.03, $p = 0.002$). No other moderators were significant (see Supplementary Table 3).

For peer review only - http://bmjopen.bmj.com/site/about/guidelines.xhtml

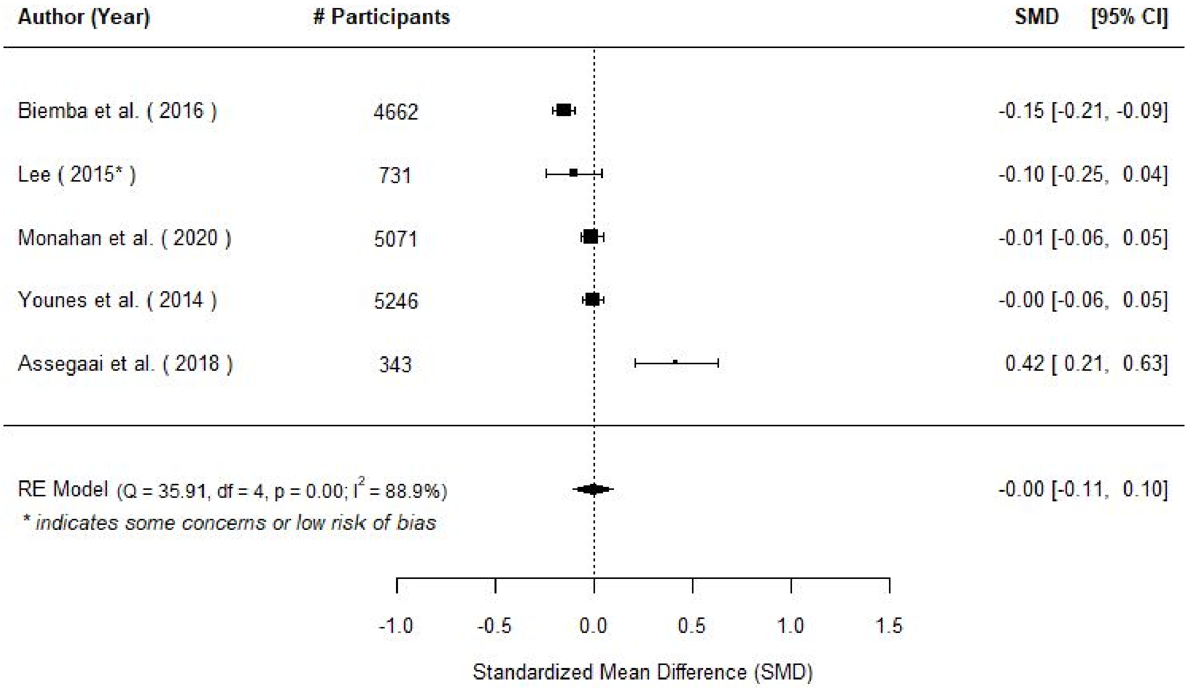

Supplementary Figure 41. Forest plot showing the observed outcomes and the estimate of the random-effects model for interventions with community engagement as the intervention on morbidity.

Mortality

We included a total of $k = 3$ studies in the analysis. The estimated average outcome based on the random-effects model was $\hat{\mu} = -0.04$ (95% CI: -0.11 to 0.04). Therefore, the average outcome did not differ significantly from zero ($z = -0.94$, $p = 0.34$), indicating no difference between the treatment and control group (see Supplementary Figure 42). According to the Q-test, the true outcomes appear to be heterogeneous ($Q(2) = 10.35$, $p < 0.01$, $\hat{\tau}^2 = 0.00$, $I^2 = 80.68\%$). An examination of the studentized residuals revealed that one study (Monahan et al. 2020) had a value larger than ± 2.39 and may be a potential outlier in the context of this model. Indeed, sensitivity analyses leaving each study out indicated that removing Monahan and colleagues (2020) would reduce the overall average effect ($\hat{\mu}$ = 0.01 [95% CI: -0.14 to 0.16]), but the effect would still be positive and non-significant (z = 0.10, p = .92). According to the Cook's distances, none of the studies could be considered to be overly influential. With only three studies contributing effects, we were unable to test for potential sources of heterogeneity.

For peer review only - http://bmjopen.bmj.com/site/about/guidelines.xhtml

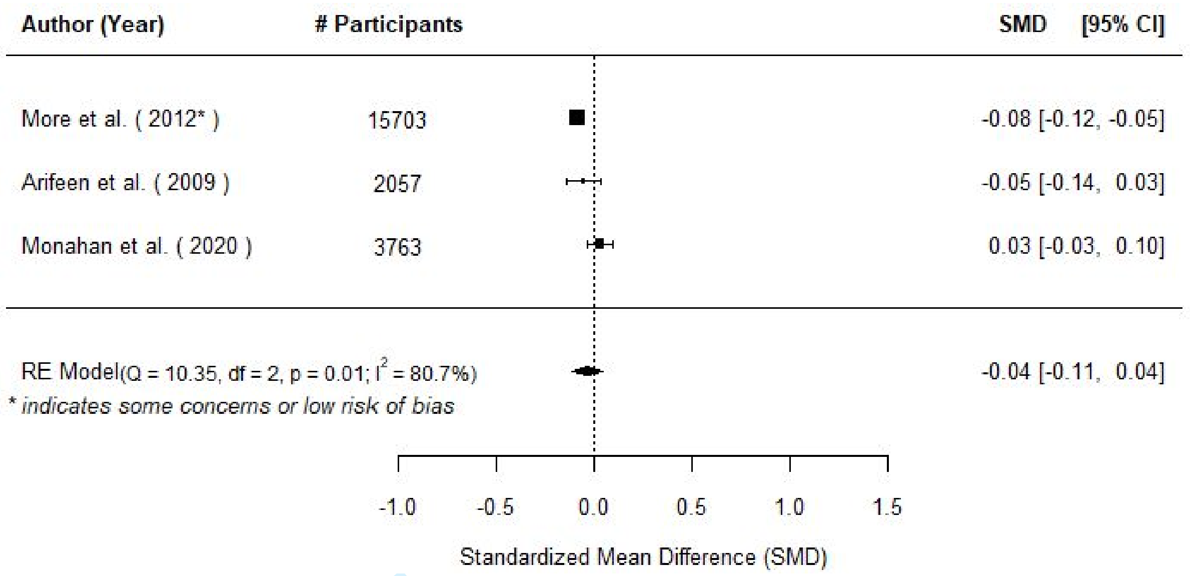

Supplementary Figure 42. Forest plot showing the observed outcomes and the estimate of the random-effects model for interventions with community engagement as the intervention on mortality.

Immunisation knowledge

We included a total of $k = 4$ studies in the analysis. The estimated average outcome based on the random-effects model was $\hat{\mu} = 0.20$ (95% CI: -0.08 to 0.49). Therefore, the average outcome did not differ significantly from zero ($z = 1.41$, $p = 0.16$), indicating no difference between the treatment and control groups. According to the Q-test, the true outcomes appear to be heterogeneous ($Q(3) = 48.39$, $p < 0.01$, $\hat{\tau}^2 = 0.08$, $I^2 = 93.80\%$). An examination of the studentized residuals revealed that one study (Banwat et al. 2015) had a value larger than ± 2.50 and may be a potential outlier in the context of this model. Indeed, sensitivity analyses leaving each study out indicated that removing Banwat and colleagues (2015) would reduce the overall average effect ($\hat{\mu}$ = 0.02 (95% CI: -0.12 to 0.17), but the effect would still be positive and non-significant (z = 0.35, p = .73). According to the Cook's distances, none of the studies could be considered to be overly influential.

We examined potential sources of heterogeneity, and study design was a significant predictor of immunisation knowledge such that studies using quasi-experimental designs had larger average effects than RCT designs by .52 standard deviation units ($\hat{\beta} = 0.52$ [95% CI: 0.11 to 0.94], p = 0.01). Region was also a significant predictor, but region was perfectly confounded with study design (e.g. all studies from South Asia were RCTs, while all studies from Sub-Saharan Africa were all quasi-experimental designs), so the effects were larger in Sub-Saharan Africa than from South Asia by the same .52 standard deviation units ($\beta = 0.52$ [95% CI: 0.11 to 0.94], p = 0.01). No other moderators were significant (see Supplementary Table 3 in Appendix 9)

For peer review only - http://bmjopen.bmj.com/site/about/guidelines.xhtml

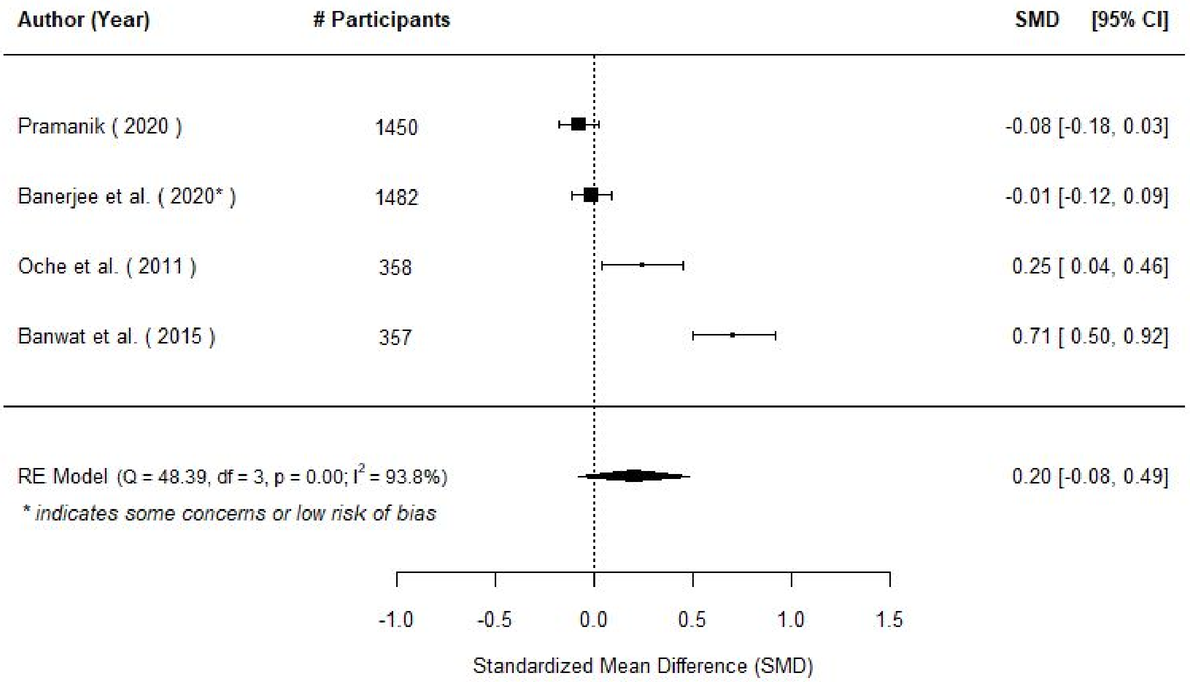

Supplementary Figure 43. Forest plot showing the observed outcomes and the estimate of the random-effects model for interventions with community engagement as the intervention on immunisation attitudes.

Immunisation attitudes

Only two studies reporting on immunisation attitudes used interventions with community engagement as the intervention. The estimated average outcome based on the random-effects model was $\hat{\mu} = 0.47$ ([95% CI: -0.19 to 1.13], $z = 1.40$, $p = 0.16$), indicating no significant difference between the intervention group and the control group (see Supplementary Figure 44). Given the small number of studies, this result should be interpreted with caution. According to the Q-test, the true outcomes appear to be heterogeneous ($\mathrm{Q}(1) = 27.51$, $\mathrm{p} < 0.001$, $\hat{\tau}^2 = 0.22$, $\mathrm{I}^2 = 96.36\%$; With only two studies and no heterogeneity, moderator analyses were not appropriate and tests of publication bias are not valid.

For peer review only - http://bmjopen.bmj.com/site/about/guidelines.xhtml

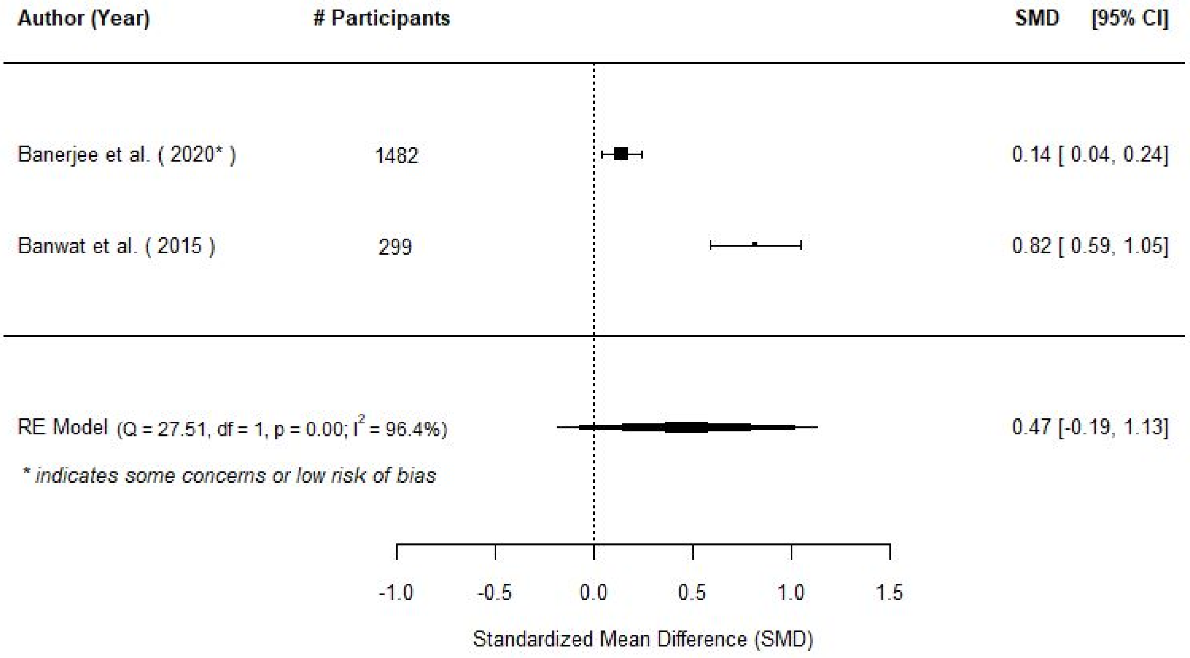

Supplementary Figure 44. Forest plot showing the observed outcomes and the estimate of the random-effects model for interventions with community engagement as the intervention on immunisation attitudes.

Vaccination card retention

Only one study (Pramanik 2020) fell into this intervention/outcome category, thus we were unable to perform a statistical synthesis. This cluster RCT from India found a null effect of their programme on vaccination card retention/availability (g = -0.07 [95% CI: -0.17 to 0.04]), but like most studies, it was assessed as having a high risk of bias.

Experience and satisfaction with health services

Only one study (Mohanan et al. 2020) fell into this intervention/outcome category, thus we were unable to perform a statistical synthesis. This cluster RCT from India did not find a significant effect of their programme on satisfaction with health services (g = -0.02 [95% CI: -0.08 to 0.04]), but like most studies, it was assessed as having a high risk of bias.

Formal health worker's motivation, capacity and performance

Only one study (Lee 2015) fell into this intervention/outcome category, thus we were unable to perform a statistical synthesis. This cluster RCT from Zambia did not find a significant effect of their programme on formal health worker's motivation, capacity, and performance (g = 0.09 [95% CI: -0.13 to 0.31]). This study was assessed as was assessed as having some concerns related to risk of bias.

For peer review only - http://bmjopen.bmj.com/site/about/guidelines.xhtml

Appendix 11: Quantitative results - community engagement in intervention design

Appendix 11 presents supplementary data for analyses presented in the main body of the manuscript for the primary outcomes of community engagement interventions using engagement in the intervention design, including funnel plots of publication bias and outlier analyses. It also includes a full presentation of the supplementary outcomes that were precluded from the main body of the manuscript due to word limitations.

Full immunisation

When studies used community engagement in the design, there was a significant effect on full childhood immunisation ($\hat{\mu} = 0.10$ [95% CI: 0.02 to 0.19],$z = 2.40$, $p = 0.02$). According to the Q-test, there was no significant amount of heterogeneity in the true outcomes ($Q(4) = 5.25$, $p = 0.26$, $\hat{\tau}^2 = 0.00$, $I^2 = 23.83\%$). An examination of the studentized residuals revealed that none of the studies had a value larger than ± 2.58 and hence there was no indication of outliers in the context of this model. According to the Cook's distances, none of the studies could be considered to be overly influential.

With no heterogeneity among effects, moderator analyses were not appropriate. When low quality studies were removed, two studies remained, and the summary effect increased ($\hat{\mu} = 0.14$ [95% CI: -0.01 to 0.29, but the effect was no longer significant, ($z = 1.89$, $p = 0.06$).

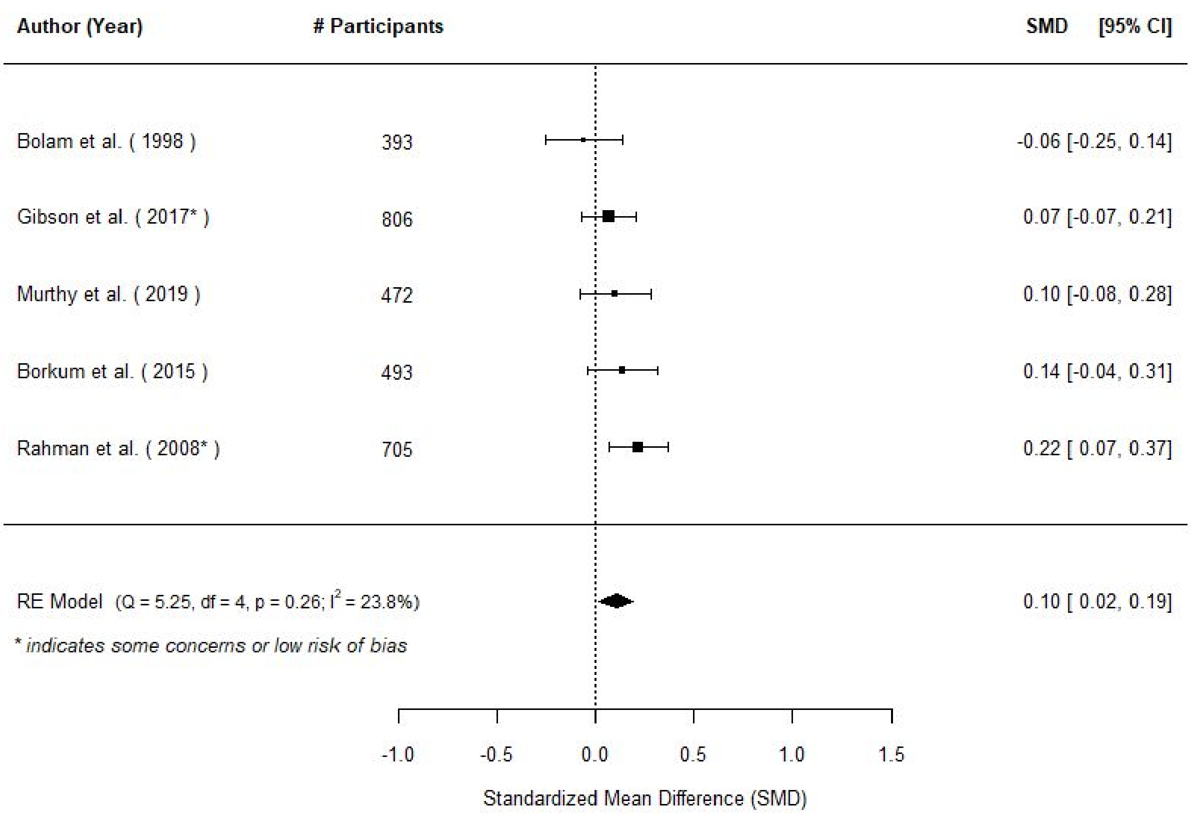

Supplementary Figure 45. Forest plot showing the observed outcomes and the estimate of the random-effects model for interventions with community engagement in the design on full immunisation

DPT3

When studies used community engagement in the design, there was not a significant effect on DPT3 vaccination ($\hat{\mu} = 0.04$ [95% CI: -0.01 to 0.08], $z = 1.69$, $p = 0.09$). According to the Q-test, there was no significant amount of heterogeneity in the true outcomes ($Q(5) = 4.36$, $p = 0.50$, $\hat{\tau}^2 = 0.00$, $I^2 = 0.00\%$). An examination of the studentized residuals revealed that none of the studies had a value larger than ± 2.64 and hence there was no indication of outliers in the context of this model. According to the Cook's distances, none of the studies could be considered to be overly influential. With no heterogeneity, we did not perform moderator analyses. With only one study of low or medium quality, we were also unable to complete sensitivity analyses for this body of evidence.

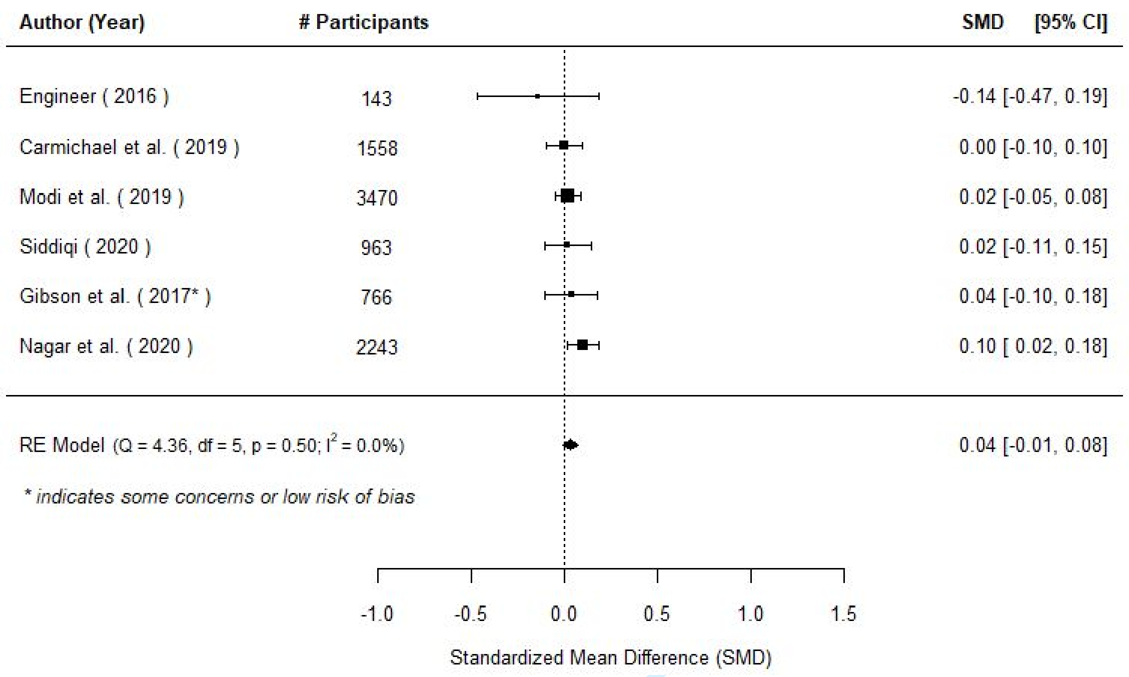

Supplementary Figure 46. Forest plot showing the observed outcomes and the estimate of the random-effects model for interventions with community engagement in the design on DPT3 vaccination

Measles

When studies used community engagement in the design, there was a significant effect on meales vaccination ($\hat{\mu} = 0.11$ [95% CI: 0.02 to 0.21], $z = 2.36$, $p = 0.02$). According to the Q-test, there was no significant amount of heterogeneity in the true outcomes ($Q(1) = 0.56$, $p = 0.46$, $\hat{\tau}^2 = 0.00$, $I^2 = 0.00\%$). With only two studies and no heterogeneity among effects, moderator analyses were not appropriate.

For peer review only - http://bmjopen.bmj.com/site/about/guidelines.xhtml

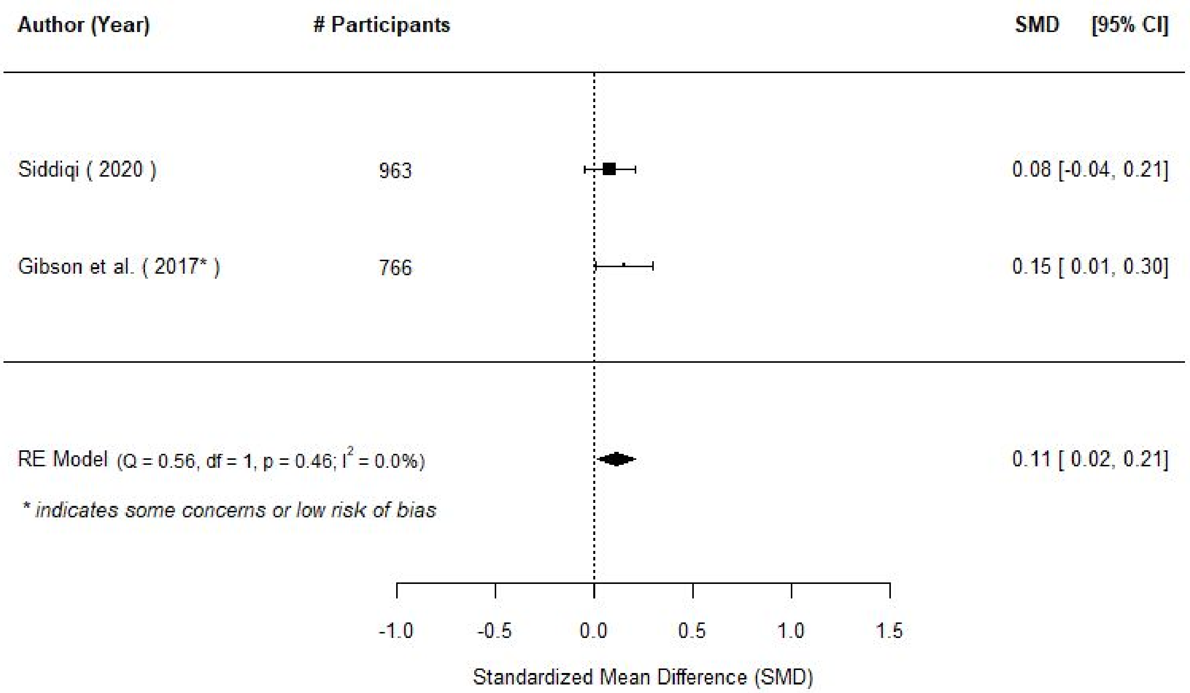

Supplementary Figure 47. Forest plot showing the observed outcomes and the estimate of the random-effects model for interventions with community engagement in the design on measles vaccination

Timeliness of full childhood immunisation

When studies used community engagement in the design, there was also a significant effect on timeliness full childhood immunisation $\hat{\mu} = 0.15$ ([95% CI: 0.004 to 0.29], $z = 2.01$, $p = 0.04$). According to the Q-test, there was no significant amount of heterogeneity in the true outcomes ($Q(1) = 0.81$, $p = 0.37$, $\hat{\tau}^2 = 0.00$, $I^2 = 0.00\%$). Both studies were assessed as high risk of bias. With only two studies and no heterogeneity, moderator analyses were not appropriate.

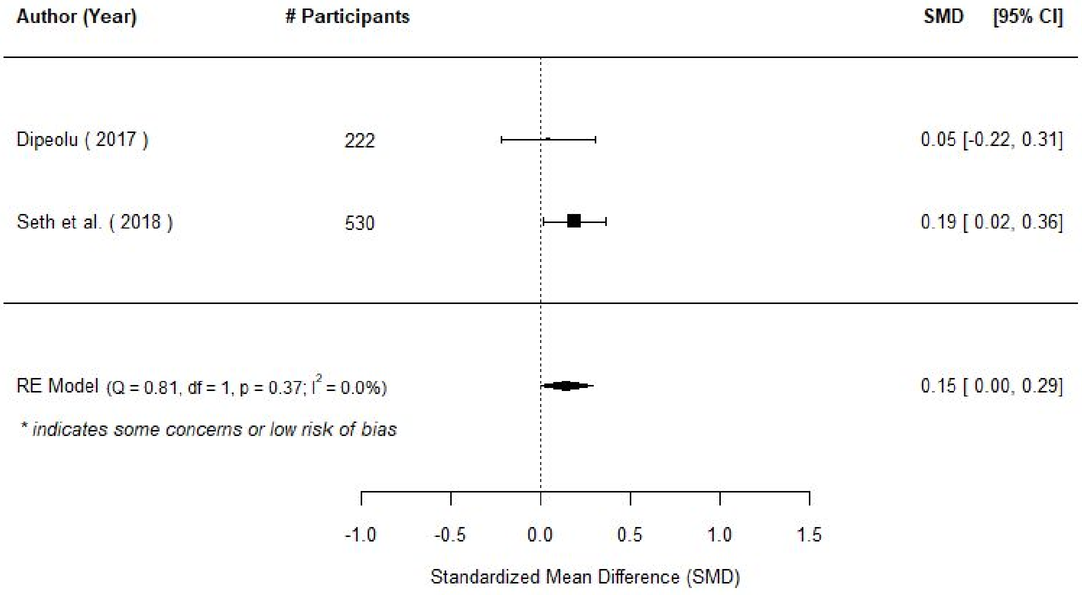

For peer review only - http://bmjopen.bmj.com/site/about/guidelines.xhtml

Supplementary Figure 48. Forest plot showing the observed outcomes and the estimate of the random-effects model for interventions with community engagement in the design on the timeliness of full childhood immunisation

Timeliness of DPT3 vaccination

When studies used community engagement in the design, there was also a significant effect on the timeliness of DPT3 vaccinations ($\hat{\mu} = 0.12$ [95% CI: 0.03 to 0.21], $z = 2.73$, $p < 0.01$). According to the Q-test, there was no significant amount of heterogeneity in the true outcomes ($Q(3) = 2.62$, $p = 0.45$, $\hat{\tau}^2 = 0.00$, $I^2 = 0.00\%$). An examination of the studentized residuals revealed that none of the studies had a value larger than ± 2.50 and hence there was no indication of outliers in the context of this model. According to the Cook's distances, none of the studies could be considered to be overly influential. With no heterogeneity indicated, we did not test for moderation.

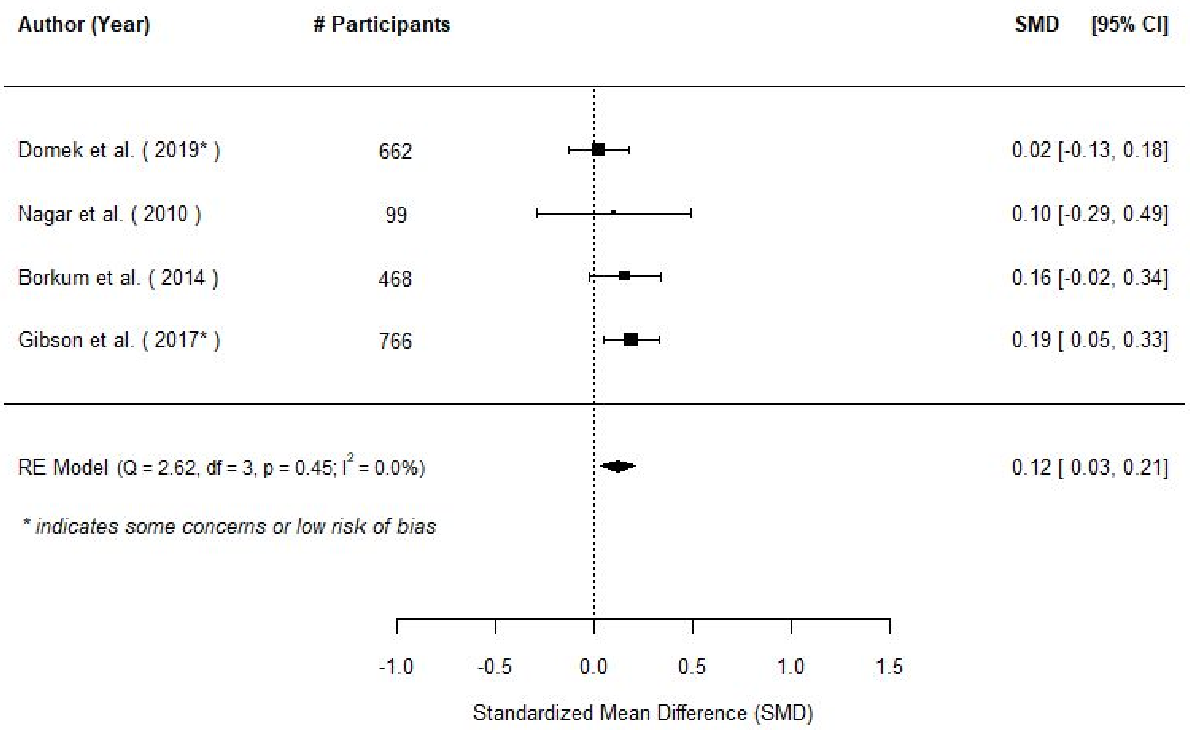

Supplementary Figure 49. Forest plot showing the observed outcomes and the estimate of the random-effects model for interventions with community engagement in the design of the intervention on the timeliness of DPT3 vaccinations.

Timeliness of measles vaccination

Only one study reporting on timeliness of measles vaccination used an intervention with engagement in the design (Gibson et al. 2017). This RCT conducted in Kenya found a small but significant positive effect (g = 0.20 [95% CI; 0.06 to 0.34]). This study was assessed as having some concerns of bias.

Secondary outcomes

Partial immunisation

For peer review only - http://bmjopen.bmj.com/site/about/guidelines.xhtml

Only two studies reporting on partial immunisation used interventions with community engagement in the intervention design. The estimated average outcome based on the random-effects model was $\hat{\mu} = 0.14$ ([95% CI: 0.01 to $0.27]$, $z = 2.05$, $p = 0.04$), indicating a small but significant benefit to the intervention group compared to the control group (see Supplementary Figure 50). Given the small number of studies, this result should be interpreted with caution. According to the Q-test, the true outcomes appear to be homogeneous ($Q(1) = 0.91$, $p = 0.34$, $\hat{\tau}^2 = 0.00$, $I^2 = 0.00$ %). With only two studies and no heterogeneity, moderator analyses were not appropriate and tests of publication bias are not valid.

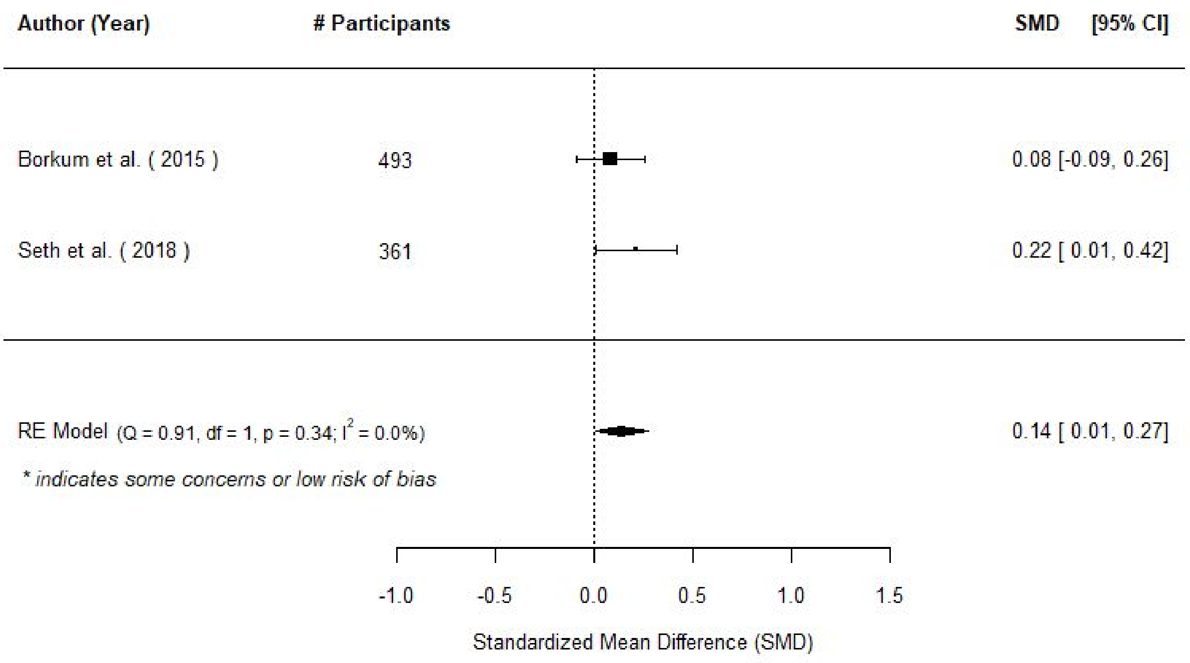

Supplementary Figure 50. Forest plot showing the observed outcomes and the estimate of the random-effects model for interventions with community engagement in the design of the intervention on partial childhood immunisation.

BCG

Only two studies reporting on BCG vaccination used interventions with community engagement in the intervention design. The estimated average outcome based on the random-effects model was $\hat{\mu} = 0.02$ ([95% CI: -0.09 to $0.13]$, $z = 0.32$, $p = 0.75$), indicating no difference between the intervention group and the control group (see Supplementary Figure 51). Given the small number of studies, this result should be interpreted with caution. According to the Q-test, the true outcomes appear to be homogeneous ($Q(1) = 0.11$, $p = 0.75$, $\hat{\tau}^2 = 0.00$, $I^2 = 0.00\%$). With only two studies and no heterogeneity, moderator analyses were not appropriate and tests of publication bias are not valid.

For peer review only - http://bmjopen.bmj.com/site/about/guidelines.xhtml

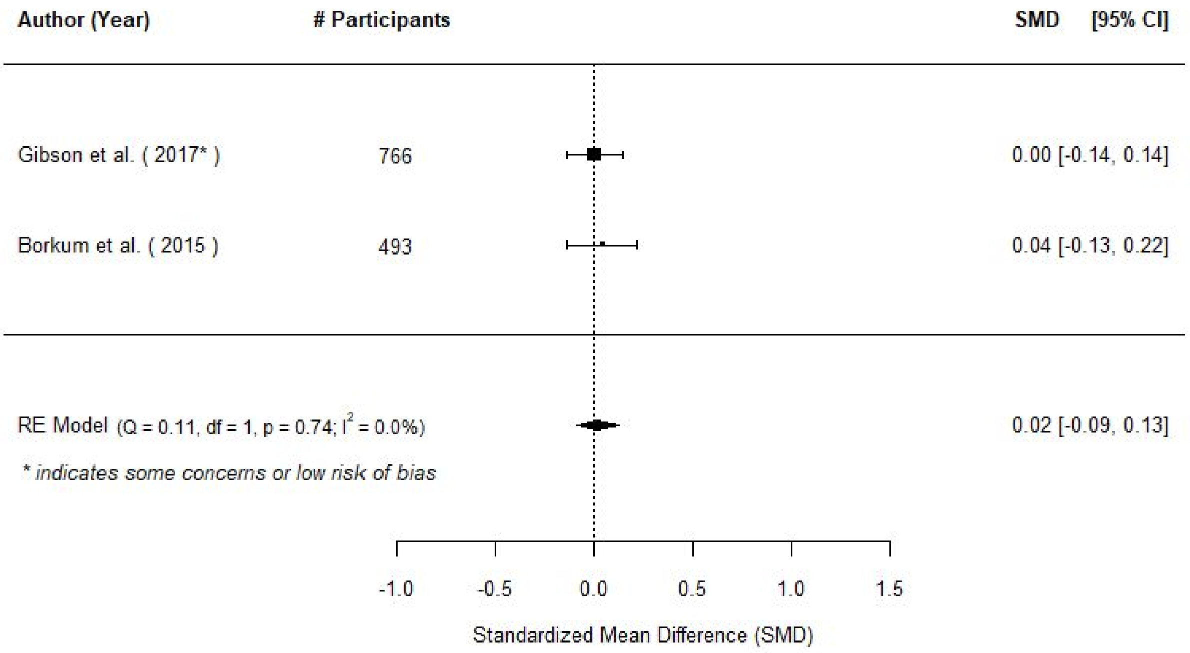

Supplementary Figure 51. Forest plot showing the observed outcomes and the estimate of the random-effects model for interventions with community engagement in the design of the intervention on BCG vaccination.

DPT1

Only two studies reporting on DPT1 vaccination used interventions with community engagement in the intervention design. The estimated average outcome based on the random-effects model was $\hat{\mu} = 0.03$ (95% CI: -0.08 to 0.14). Therefore, the average outcome did not differ significantly from zero ($z = 0.60$, $p = 0.55$, see Supplementary Figure 52), indicating no difference between the treatment group and the control group on DPT1 vaccination. Given the small number of studies, this result should be interpreted with caution. According to the Q-test, the true outcomes appear to be homogeneous ($Q(1) = 0.48$, $p = 0.49$, $\hat{\tau}^2 = 0.00$, $I^2 = 0.00\%$). With only two studies and no heterogeneity, moderator analyses were not appropriate and tests of publication bias are not valid.

For peer review only - http://bmjopen.bmj.com/site/about/guidelines.xhtml

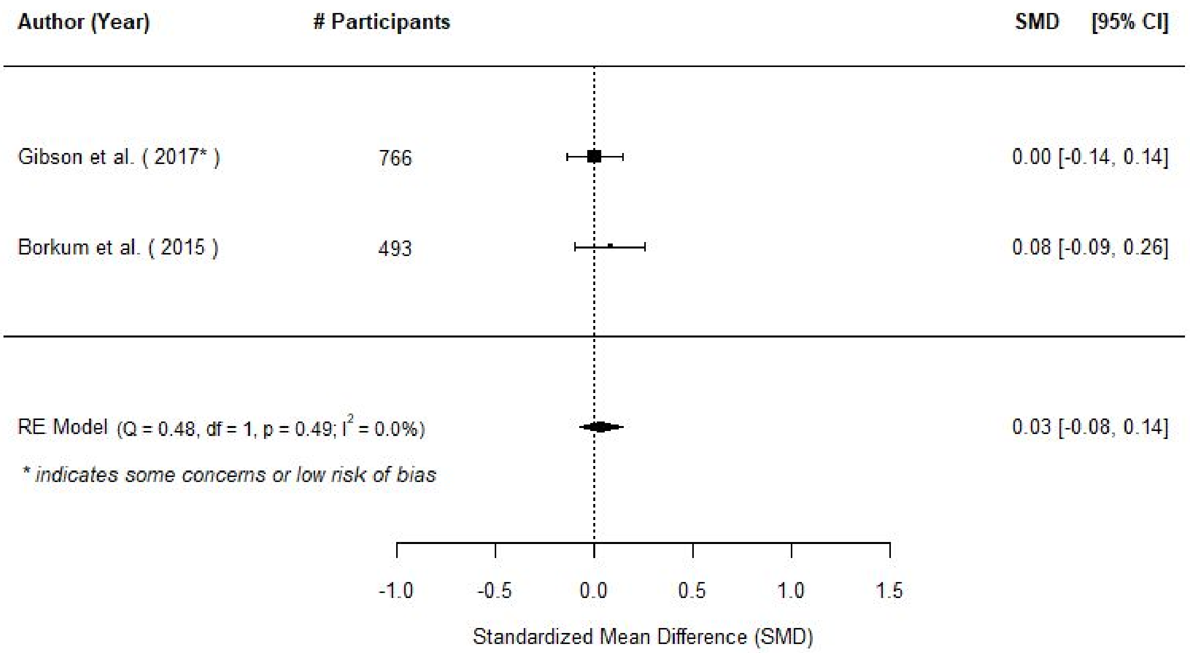

Supplementary Figure 52. Forest plot showing the observed outcomes and the estimate of the random-effects model for interventions with community engagement in the design on the intervention on DPT1 vaccination.

DPT2

Only two studies reporting on DPT2 vaccination used interventions with community engagement in the intervention design. The estimated average outcome based on the random-effects model was $\hat{\mu} = 0.05$ (95% CI: -0.06 to 0.16). Therefore, the average outcome did not differ significantly from zero ($z = 0.83$, $p = 0.41$, see Supplementary Figure 53), indicating no difference between the treatment group and the control group on DPT2 vaccination. Given the small number of studies, this result should be interpreted with caution. According to the Q-test, the true outcomes appear to be homogeneous ($Q(1) = 0.07,\ p = 0.41,\ \hat{\tau}^2 = 0.00,\ I^2 = 0.00\%$). With only two studies and no heterogeneity, moderator analyses were not appropriate and tests of publication bias are not valid.

For peer review only - http://bmjopen.bmj.com/site/about/guidelines.xhtml

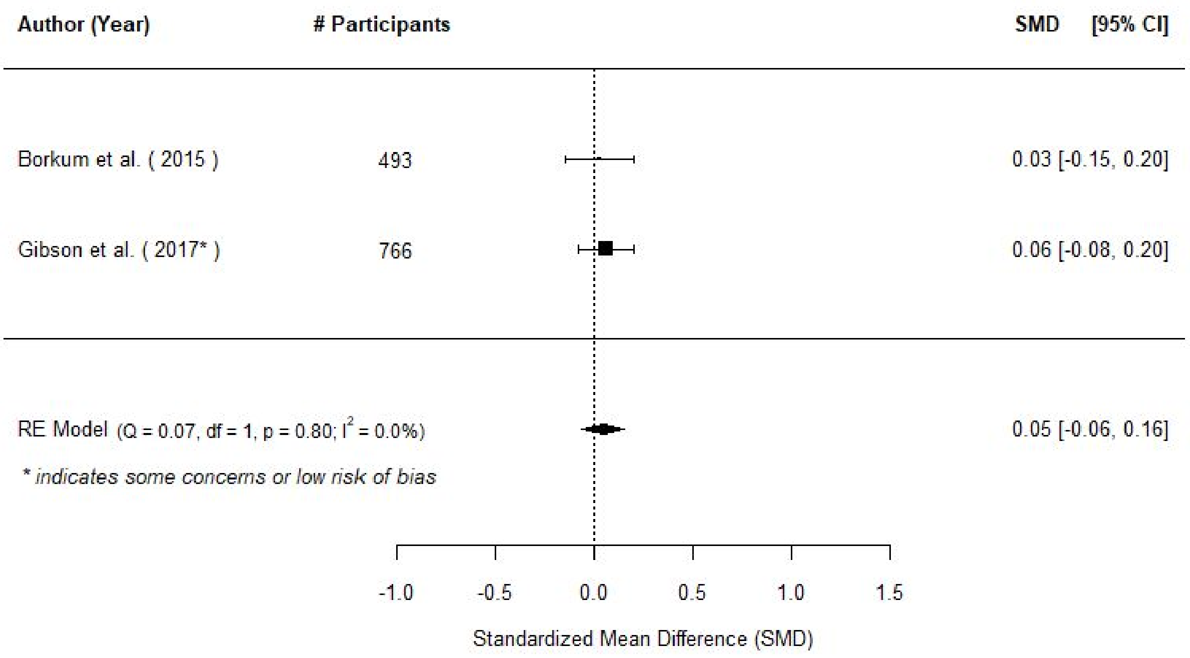

Supplementary Figure 53. Forest plot showing the observed outcomes and the estimate of the random-effects model for interventions with community engagement in the design on the intervention on DPT2 vaccination.

OPV0

Only two studies reporting on immunisation attitudes used interventions with community engagement in the intervention design. The estimated average outcome based on the random-effects model was $\hat{\mu} = 0.01$ (95% CI: -0.13 to 0.14). Therefore, the average outcome did not differ significantly from zero ($z = 0.08$, $p = 0.94$, see Supplementray Figure 54). Given the small number of studies, this result should be interpreted with caution. According to the Q-test, the true outcomes appear to be heterogeneous ($\mathrm{Q}(1) = 0.01$, $\mathrm{p} = 0.93$, $\hat{\tau}^2 = 0.00$, $\mathrm{I}^2 = 0.00\%$). With only two studies and no heterogeneity, moderator analyses were not appropriate and tests of publication bias are not valid.

For peer review only - http://bmjopen.bmj.com/site/about/guidelines.xhtml

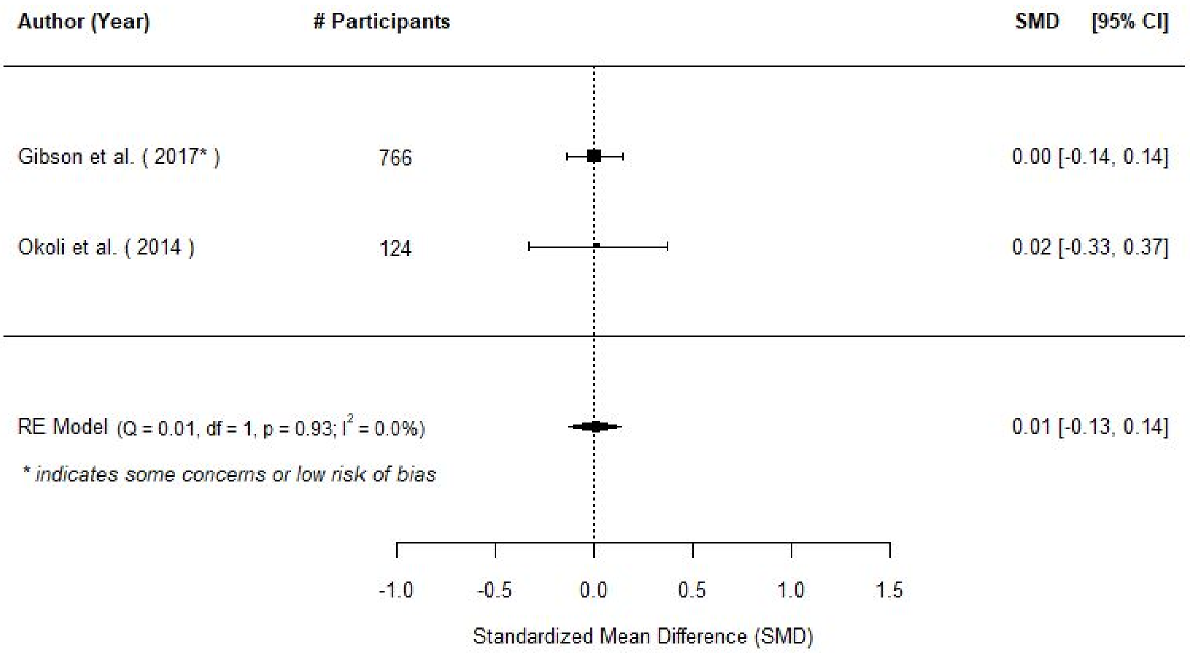

Supplementary Figure 54. Forest plot showing the observed outcomes and the estimate of the random-effects model for interventions with community engagement in the design on the intervention on OPV0 vaccination.

OPV1

Only two studies reporting on OPV1 vaccination used interventions with community engagement in the intervention design. The estimated average outcome based on the random-effects model was $\hat{\mu} = 0.08$ (95% CI: -0.03 to 0.19). Therefore, the average outcome did not differ significantly from zero ($z = 1.44$, $p = 0.15$, see Supplementary Figure 55). Given the small number of studies, this result should be interpreted with caution. According to the Q-test, the true outcomes appear to be homogeneous ($\mathrm{Q}(1) = 0.20, p = 0.65,\ \hat{\tau}^2 = 0.00,\ \mathrm{I}^2 = 0.00\%$). With only two studies, moderator analyses were not appropriate and tests of publication bias are not valid.

For peer review only - http://bmjopen.bmj.com/site/about/guidelines.xhtml

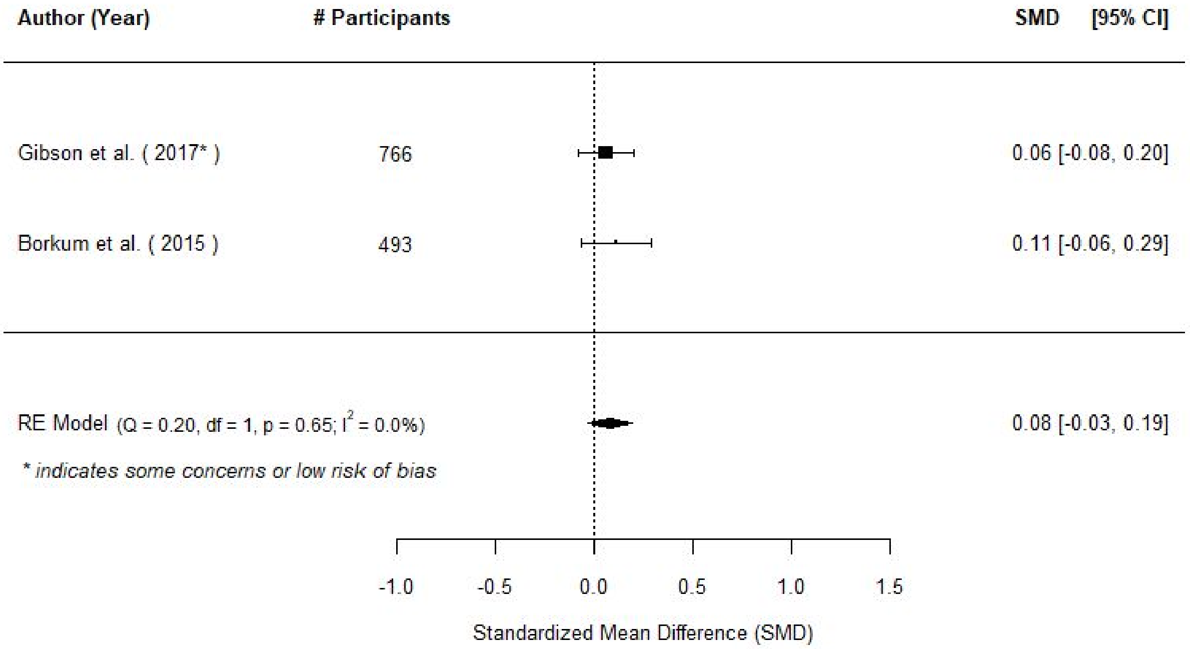

Supplementary Figure 55. Forest plot showing the observed outcomes and the estimate of the random-effects model for interventions with community engagement in the design on the intervention on OPV1 vaccination.

OPV2

Only two studies reporting on OPV2 vaccination used interventions with community engagement in the intervention design. The estimated average outcome based on the random-effects model was $\hat{\mu} = 0.23$ (95% CI: -0.09 to 0.55). Therefore, the average outcome did not differ significantly from zero ($z = 1.43$, $p = 0.15$, see Supplementray Figure 56), indicating no significant difference between the intervention group and the control group on OPV2 vaccinations. Given the small number of studies, this result should be interpreted with caution. According to the Q-test, the true outcomes appear to be heterogeneous ($Q(1) = 7.96, p = 0.004$, $\hat{\tau}^2 = 0.05$, $I^2 = 87.44\%$). With only two studies, moderator analyses were not appropriate and tests of publication bias are not valid.

For peer review only - http://bmjopen.bmj.com/site/about/guidelines.xhtml

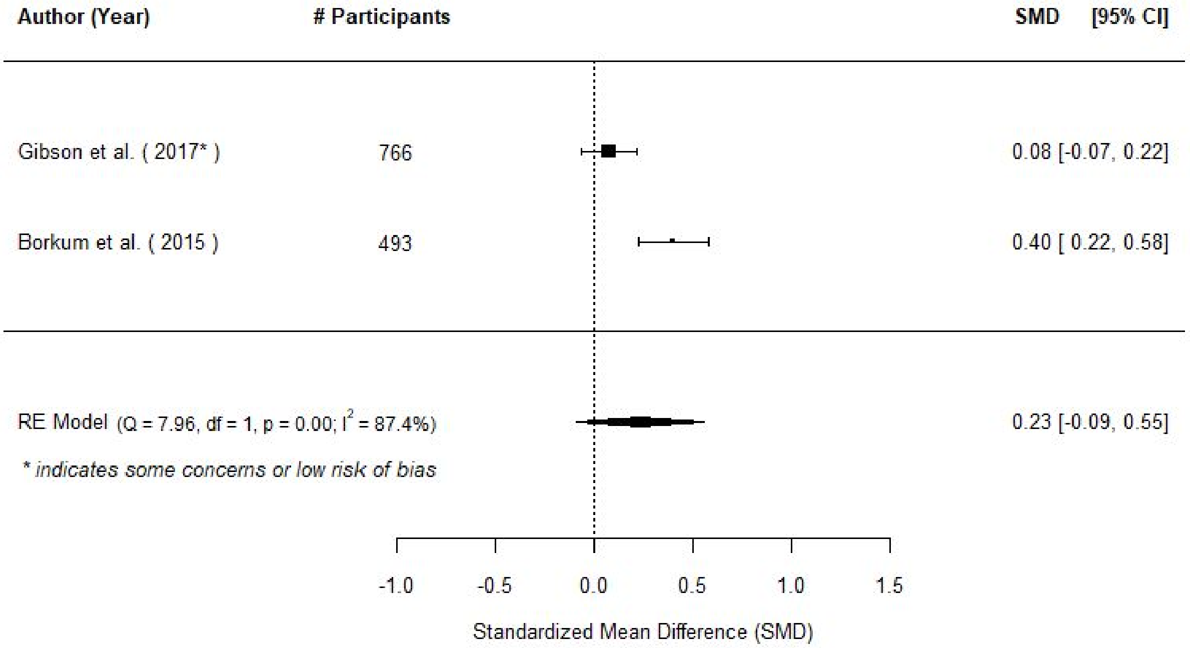

Supplementary Figure 56. Forest plot showing the observed outcomes and the estimate of the random-effects model for interventions with community engagement in the design on the intervention on OPV2 vaccination.

OPV3

Only one study (Borkum 2015) fell into this intervention/outcome category, thus we were unable to perform a statistical synthesis. This cluster RCT from India found a small but significant positive effect of their programme on OPV3 vaccination (g = 0.19 [95% CI: 0.01 to 0.36]), but again, it was assessed as having a high risk of bias.

Dropouts

There were no studies using engagement in the intervention design that reported on vaccination dropouts.

Morbidity

We used reports of diarrhea (most typically in the past two weeks) as a proxy for childhood morbidity. In all cases, effects were reverse coded such that positive effects always indicate a benefit to the treated group. Thus, a positive effect here would be interpreted as a reduction in diarrhea among treated participants compared to control participants. Only two studies reporting on childhood morbidity used interventions with community engagement in the intervention design. The estimated average outcome based on the random-effects model was $\hat{\mu} = 0.05$ (95% CI: -0.06 to 0.15). Therefore, the average outcome did not differ significantly from zero ($z = 0.88$, $p = 0.38$, see Supplementray Figure 57). Given the small number of studies, this result should be interpreted with caution. According to the Q-test, the true outcomes appear to be homogeneous ($\mathrm{Q}(1) = 1.84$, $\mathrm{p} = 0.17$, $\hat{\tau}^2 = 0.003$, $\mathrm{I}^2 = 45.$ 71%). With only two studies, moderator analyses were not appropriate and tests of publication bias are not valid.

For peer review only - http://bmjopen.bmj.com/site/about/guidelines.xhtml

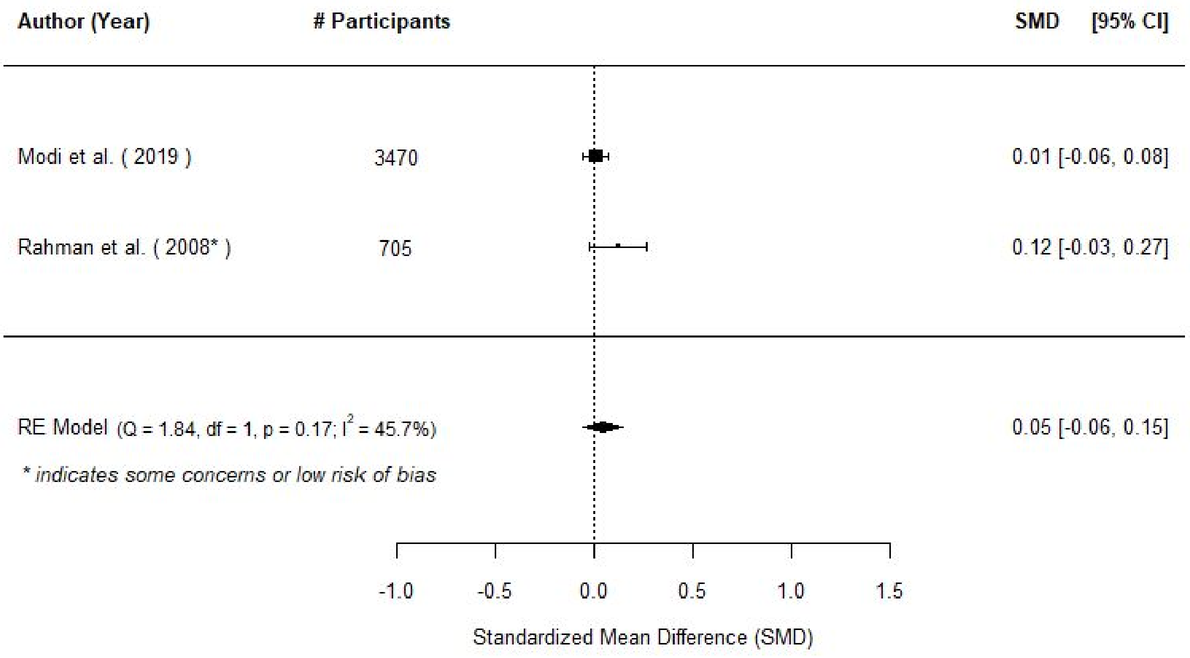

Supplementary Figure 57. Forest plot showing the observed outcomes and the estimate of the random-effects model for interventions with community engagement in the design on the intervention on childhood morbidity.

Mortality

There were no studies using engagement in the intervention design that reported on child mortality.

Immunisation knowledge

We included a total of $k = 3$ studies in the analysis. The estimated average outcome based on the random-effects model was $\hat{\mu} = 0.31$ (95% CI: 0.04 to 0.58). Therefore, the average outcome differed significantly from zero ($z = 2.26$, $p = 0.02$), indicating a moderate significant benefit to the intervention group compared to the control group (see Supplementary Figure 58). According to the Q-test, the true outcomes appear to be heterogeneous ($\mathrm{Q}(2) = 7.23$, $\mathrm{p} = 0.03$, $\hat{\tau}^2 = 0.04$, $\mathrm{I}^2 = 72.34\%$). An examination of the studentized residuals revealed that one study (Murthy et al. 2019) had a value larger than ± 2.39 and may be a potential outlier in the context of this model. Indeed, sensitivity analyses leaving each study out indicated that removing Murthy and colleagues (2019) would reduce the overall average effect ($\hat{\mu} = 0.19$ (95% CI: -0.03 to 0.41), and the effect would still be positive but would be non-significant ($z = 1.67$, $p = .09$). According to the Cook's distances, none of the studies could be considered to be overly influential. With only three studies, we were unable to test for sources of heterogeneity.

For peer review only - http://bmjopen.bmj.com/site/about/guidelines.xhtml

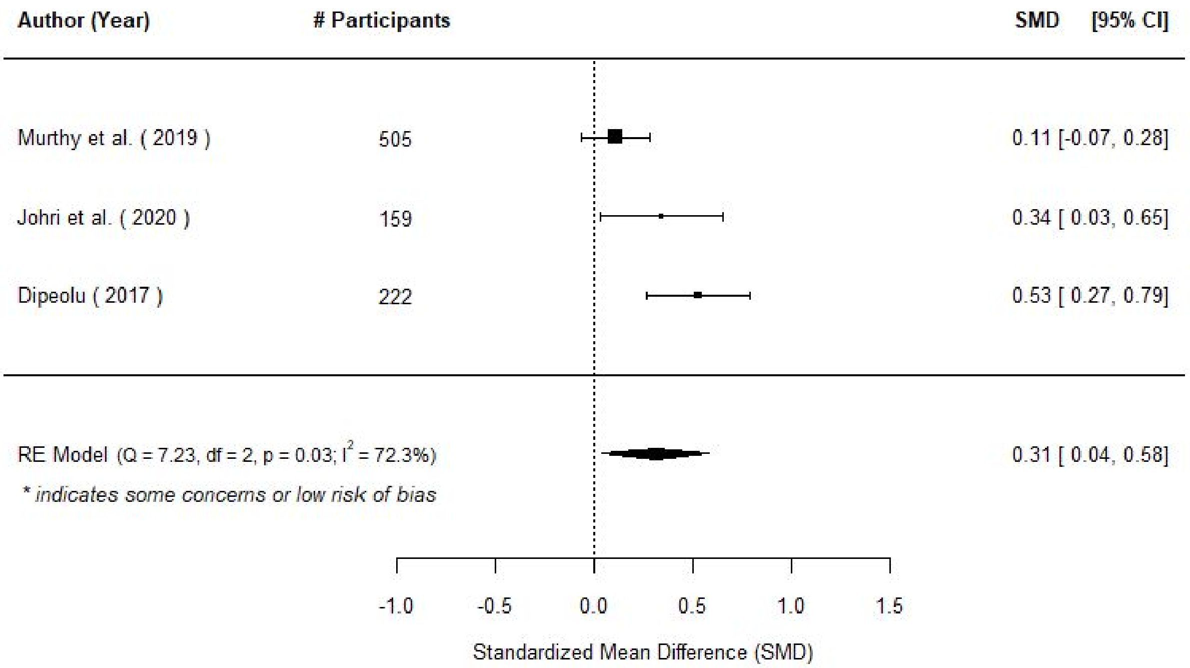

Supplementray Figure 58. Forest plot showing the observed outcomes and the estimate of the random-effects model for interventions with community engagement in the design on the intervention on childhood morbidity.

Immunisation attitudes

Only two studies reporting on immunisation attitudes used interventions with community engagement in the intervention design. The estimated average outcome based on the random-effects model was $\hat{\mu} = -0.11$ (95% CI: -0.53 to 0.30). Therefore, the average outcome did not differ significantly from zero ($z = -0.55$, $p = 0.58$, see Supplementary Figure 59). Given the small number of studies, this result should be interpreted with caution. According to the Q-test, the true outcomes appear to be heterogeneous ($Q(1) = 4.10$, $p = 0.04$, $\hat{\tau}^2 = 0.07$, $I^2 = 75.61\%$). With only two studies, moderator analyses were not appropriate and tests of publication bias are not valid.

For peer review only - http://bmjopen.bmj.com/site/about/guidelines.xhtml

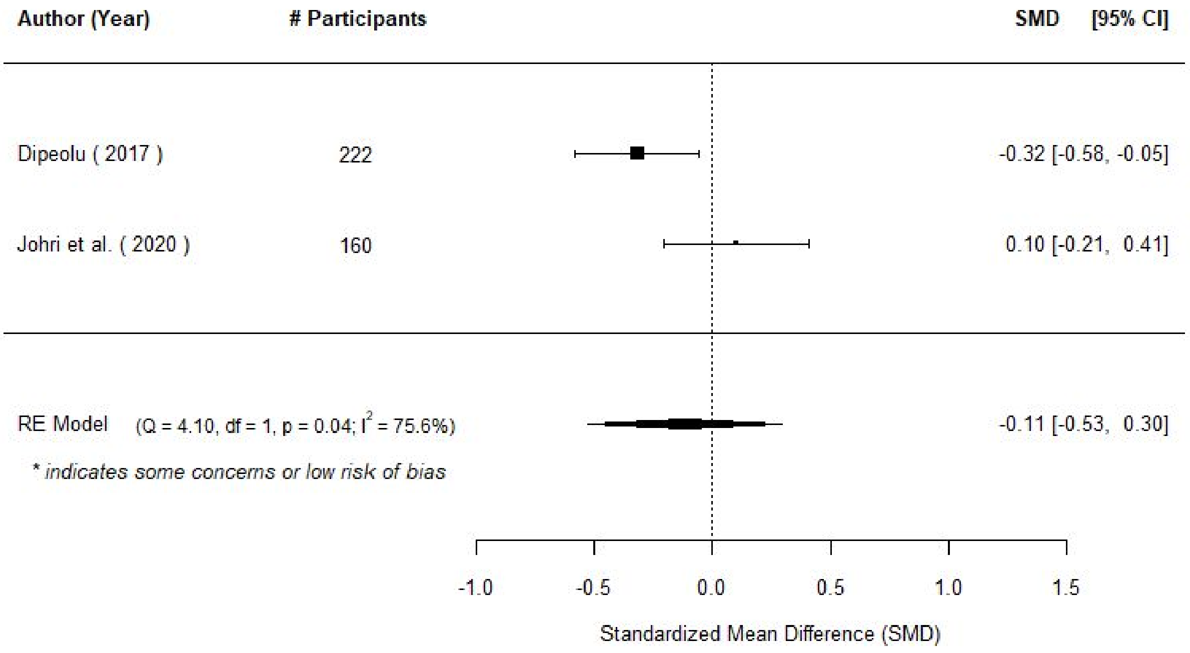

Supplementary Figure 59. Forest plot showing the observed outcomes and the estimate of the random-effects model for interventions with community engagement in the design on the intervention on immunisation attitudes.

Vaccination card retention

No studies reporting on vaccination card retention/availability used engagement in the intervention design.

Experience and satisfaction with health services

Only one study (Engineer 2016) fell into this intervention/outcome category, thus we were unable to perform a statistical synthesis. This cluster RCT from Afghanistan did not find a significant effect of their programme on satisfaction with health services (g = 0.20 [95% CI: -0.11 to 0.51]), but as with the other study reporting on this outcome, it was assessed as having a high risk of bias.

For peer review only

Appendix 12: Community engagement in implementation autonomy of interventions

Appendix 12 presents supplementary data for analyses presented in the main body of the manuscript for the primary outcomes of community engagement in implementation autonomy, including funnel plots of publication bias and outlier analyses. It also includes a full presentation of the supplementary outcomes that were precluded from the main body of the manuscript due to word limitations.

Full immunisation

Only k = 2 studies reported on the impact of interventions with community engagement in implementation autonomy on full childhood immunisation. There was no effect on full immunisation ($\hat{\mu} = 0.23$ [95% CI: -0.001 to 0.47], $z = 1.95$, $p = 0.051$). According to the Q-test, the true outcomes appear to be homogeneous ($Q(1) = 3.71$, $p = 0.054$, $\hat{\tau}^2 = 0.02$, $I^2 = 73.07\%$, see Supplementray Figure 60). With only two studies, we could not test for moderation or publication bias, nor conduct sensitivity analysis by study quality.

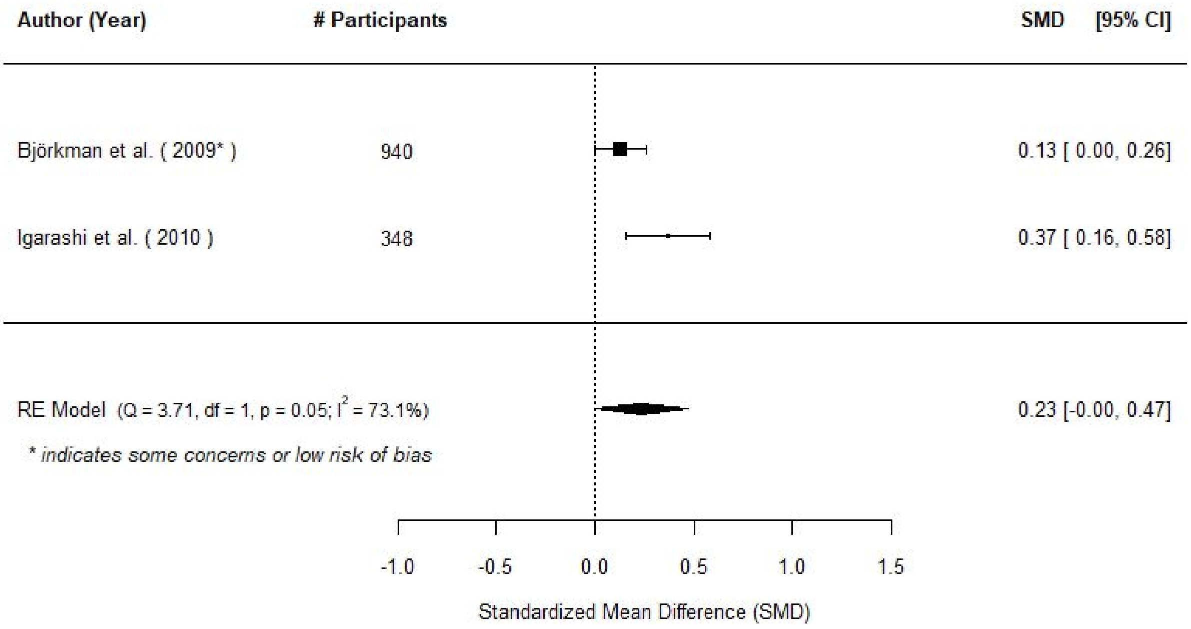

Supplementary Figure 60. Forest plot showing the observed outcomes and the estimate of the random-effects model for interventions with community engagement in implementation autonomy on full childhood immunisation.

DPT3

Only k = 3 studies examined the impact of interventions with community engagement in implementation autonomy on DPT3 vaccination. The average outcome based on the random-effects model indicated there was no effect on DPT3 vaccination ($\hat{\mu} = 0.11$ [95% CI: -0.05 to 0.28], $z = 1.38$, $p = 0.17$). According to the Q-test, the true outcomes appear to be heterogeneous ($Q(2) = 10.06$, $p < 0.01$, $\hat{\tau}^2 = 0.02$, $I^2 = 80.12\%$). An examination of the studentized residuals revealed that one study (Webster 2019) had a value larger than ± 2.39 and may be a potential outlier in the context of this model. Indeed, sensitivity analyses leaving each study out indicated that removing Webster (2019) would increase the overall average effect ($\hat{\mu}$= 0.20 [95% CI: 0.10 to 0.29]), with the effect still positive and significant (z = 3.99, p < .001). According to the Cook's distances, none of the studies could be considered to be overly influential.

With only three studies, moderator analyses and tests of publication bias were not appropriate. With two of the three studies assessed as high risk of bias, we were also unable to conduct a sensitivity analysis by study quality.

For peer review only - http://bmjopen.bmj.com/site/about/guidelines.xhtml

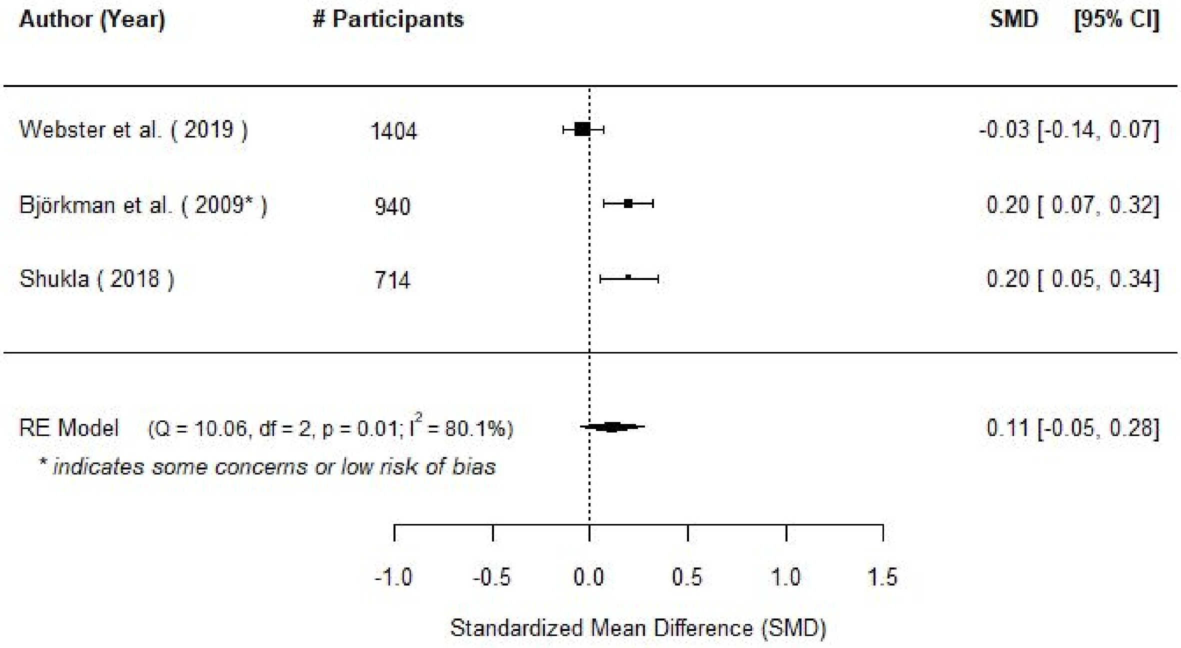

Supplementray Figure 61. Forest plot showing the observed outcomes and the estimate of the random-effects model for interventions with community engagement in implementation autonomy on DPT3 vaccination

Measles

Only $k = 2$ studies examined the impact of interventions with community engagement in implementation autonomy on measles vaccination. The average outcome based on the random-effects model indicated there was no effect on measles vaccination ($\hat{\mu} = 0.03$ [95% CI: -0.09 to 0.15], $z = 0.47$, $p = 0.64$). According to the Q-test, the true outcomes appear to be homogeneous ($Q(1) = 2.21$, $p = 0.14$, $\hat{\tau}^2 = 0.004$, $I^2 = 54.84\%$; see Supplementary Figure 62). With only two studies we were unable to test for publication bias.

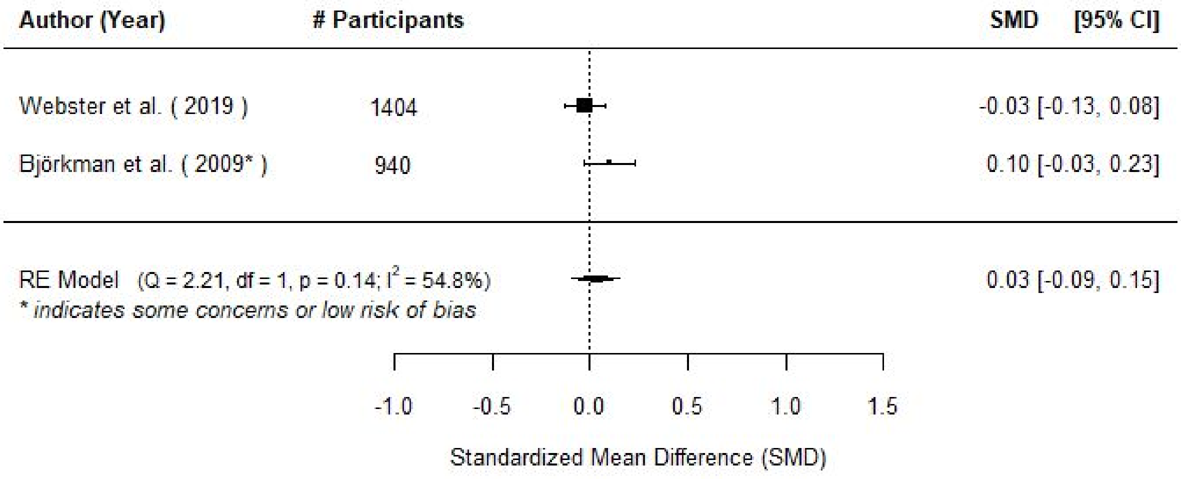

Supplementary Figure 62. Forest plot showing the observed outcomes and the estimate of the random-effects model for interventions with community engagement in implementation autonomy on measles vaccination

For peer review only - http://bmjopen.bmj.com/site/about/guidelines.xhtml

Timeliness of full childhood immunisation

Only k = 2 studies examined the impact of interventions with community engagement in implementation autonomy on the timeliness of full childhood immunisation. The average outcome based on the random-effects model indicated there was no effect on the timeliness of full childhood immunisation ($\hat{\mu} = 0.38$ [95% CI: -0.28 to 1.03], $z = 1.13$, $p = 0.26$). According to the Q-test, the true outcomes appear to be homogeneous ($Q(1) = 3.57$, $p = 0.06$, $\hat{\tau}^2 = 0.17$, $I^2 = 72.02\%$). There was no indication of outliers in the context of this model, and with only two studies, we could not test for moderation. One study was assessed as having a high risk of bias (Igarashi et al. 2010) while the other was assessed as some concerns related to risk of bias (Lee 2015).

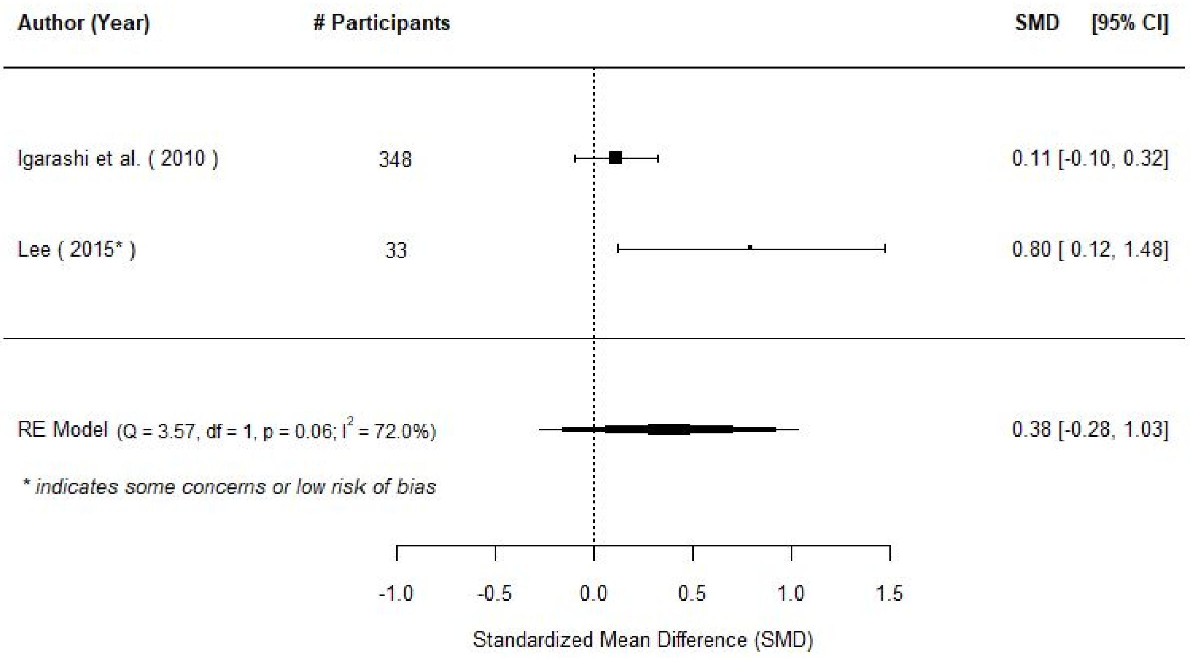

Supplementary Figure 63. Forest plot showing the observed outcomes and the estimate of the random-effects model for interventions with community engagement in implementation autonomy on DPT3 vaccination

DPT3 vaccination timeliness

Only k = 2 studies examined the impact of interventions with community engagement in implementation autonomy on DPT3 vaccination timeliness. The average outcome based on the random-effects model indicated there was no effect on the timeliness of DPT3 vaccination ($\hat{\mu} = 0.04$ [95% CI: -0.06 to 0.13], $z = 0.75$, $p = 0.45$). According to the Q-test, the true outcomes appear to be homogeneous ($Q(1) = 0.63$, $p = 0.45$, $\hat{\tau}^2 = 0.00$, $I^2 = 0.00\%$). There was no indication of outliers in the context of this model, and with only two studies, we could not test for moderation. Both studies were assessed as having a high risk of bias

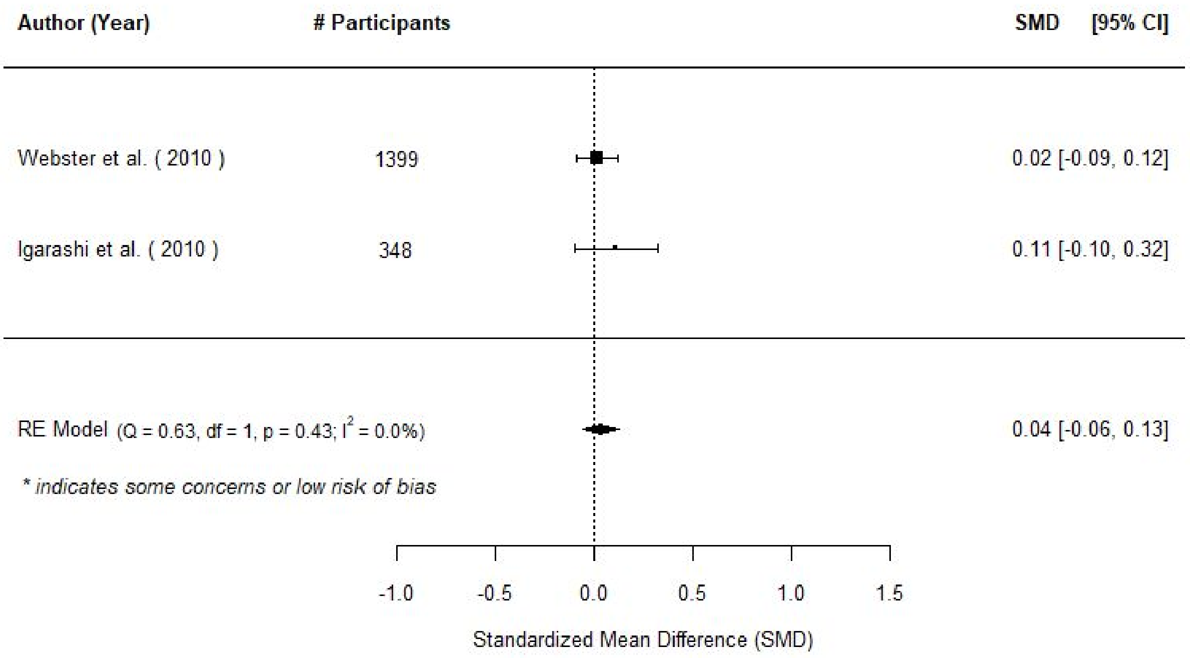

Supplementray Figure 64. Forest plot showing the observed outcomes and the estimate of the random-effects model for interventions with community engagement in implementation autonomy on DPT3 vaccination timeliness

Measles vaccination timeliness

No studies reporting on the timeliness of measles vaccinations used community engagement in implementation autonomy.

Secondary outcomes

Partial immunisation

Only one study (Webster et al. 2019) fell into this intervention/outcome category, thus we were unable to perform a statistical synthesis. This cluster RCT from Uganda found a null effect of their programme on partial childnood immunisation (*g* = 0.02 [95% CI: -0.08 to 0.13]), but like most studies, it was assessed as having a high risk of bias.

BCG

We included k = 2 studies in this analysis. The estimated average outcome was $\hat{\mu} = 0.03$ ([95% CI: -0.05 to 0.11], $z = 0.75$, $p = 0.46$) indicating no difference between the treated group and the untreated group (Supplementary Figure 62). According to the Q-test, the true outcomes appear to be homogeneous ($Q(1) = 0.37$, $p = 0.54$, $\hat{\tau}^2 = 0.00$, $I^2 = 00.00\%$). With only two studies and no heterogeneity, moderator analyses were not appropriate and tests of publication bias are not valid.

For peer review only - http://bmjopen.bmj.com/site/about/guidelines.xhtml

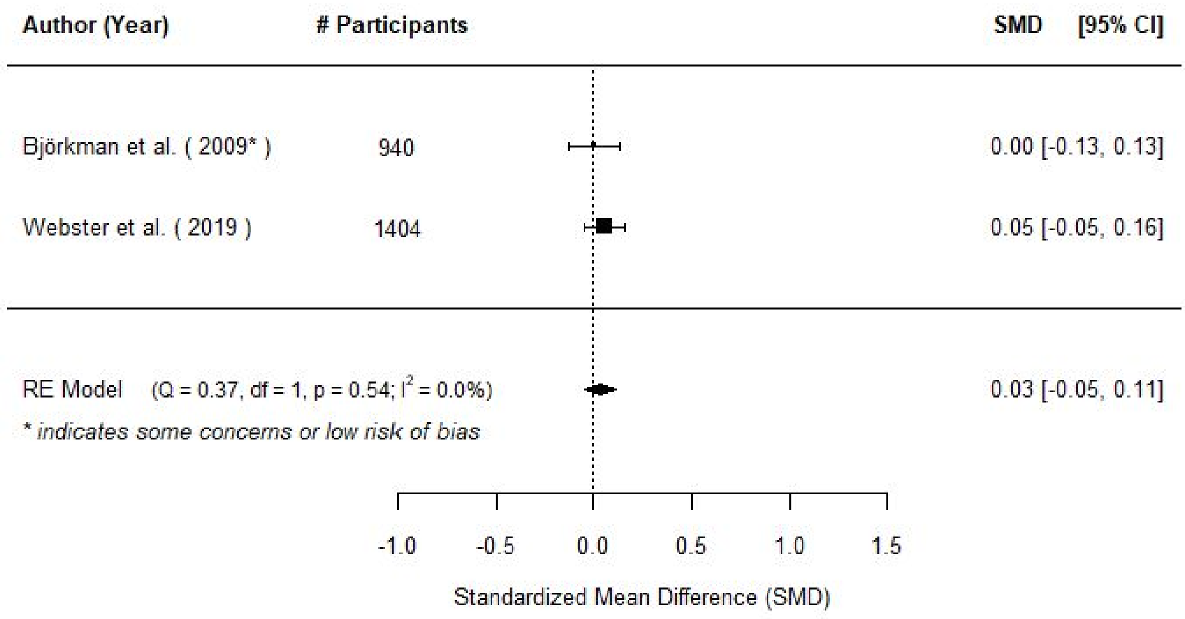

Supplementary Figure 65. Forest plot showing the observed outcomes and the estimate of the random-effects model for interventions with community engagement in implementation autonomy on BCG vaccination

DPT1

Only one study (Webster et al. 2019) fell into this intervention/outcome category, thus we were unable to perform a statistical synthesis. This cluster RCT from Uganda found a null effect of their programme on DPT1 vaccination (*g* = 0.04 [95% CI: -0.07 to 0.14]), but like most studies, it was assessed as having a high risk of bias.

DPT2

Only one study (Webster et al. 2019) fell into this intervention/outcome category, thus we were unable to perform a statistical synthesis. This cluster RCT from Uganda found a null effect of their programme on DPT1 vaccination (*g* = 0.01 [95% CI: -0.10 to 0.11]), but like most studies, it was assessed as having a high risk of bias.

OPV0

Only one study (Webster et al. 2019) fell into this intervention/outcome category, thus we were unable to perform a statistical synthesis. This cluster RCT from Uganda found a null effect of their programme on OPV0 vaccination (*g* = 0.01 [95% CI: -0.10 to 0.11]), but like most studies, it was assessed as having a high risk of bias.

OPV1

Only one study (Webster et al. 2019) fell into this intervention/outcome category, thus we were unable to perform a statistical synthesis. This cluster RCT from Uganda found a null effect of their programme on OPV1 vaccination (*g* = 0.03 [95% CI: -0.07 to 0.14]), but like most studies, it was assessed as having a high risk of bias.

For peer review only - http://bmjopen.bmj.com/site/about/guidelines.xhtml

OPV2

Only one study (Webster et al. 2019) fell into this intervention/outcome category, thus we were unable to perform a statistical synthesis. This cluster RCT from Uganda found a null effect of their programme on OPV2 vaccination (*g* = 0.04 [95% CI: -0.06 to 0.15]), but again, it was assessed as having a high risk of bias.

OPV3

Only two studies examined the impact of interventions with community engagement in implementation autonomy on OPV3 vaccination. The estimated average outcome was $\hat{\mu} = 0.03$ [95% CI: -0.12 to 0.18], which was not significant ($z = 0.35$, $p = 0.73$), indicating there was no difference between the intervention group and the control group. According to the Q-test, the true outcomes appear to be homogeneous ($Q(1) = 3.38$, $p = 0.07$, $\hat{\tau}^2 = 0.01$, $I^2 = 70.38\%$; see Supplementray Figure 66). With only two studies we were unable to test for publication bias or sources of heterogeneity.

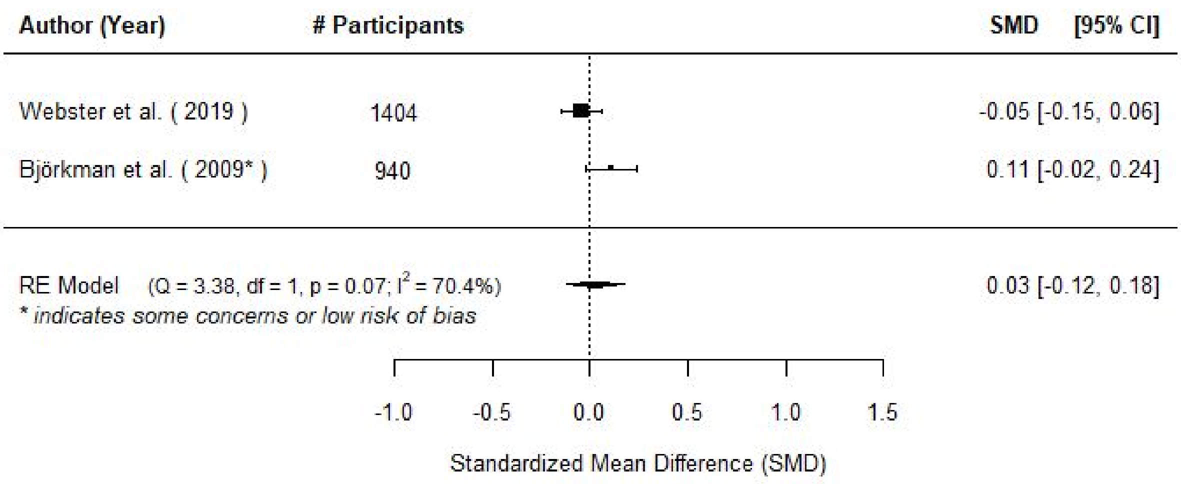

Supllementary Figure 66. Forest plot showing the observed outcomes and the estimate of the random-effects model for interventions with community engagement in implementation autonomy on OPV3 vaccinations.

Dropouts

Only one study (Webster et al. 2019) fell into this intervention/outcome category, thus we were unable to perform a statistical synthesis. This cluster RCT from Uganda found a small but significant negative impact of their programme on full childhood immunisation (*g* = -0.24 [95% CI: -0.35 to -0.14]), but like most studies, it was assessed as having a high risk of bias.

Morbidity

We used reports of diarrhea (most typically in the past two weeks) as a proxy for childhood morbidity. In all cases, effects were reverse coded such that positive effects always indicate a benefit to the treated group. Thus a positive

For peer review only - http://bmjopen.bmj.com/site/about/guidelines.xhtml

effect here would be interpreted as a reduction in diarrhea among treated participants compared to control participants. Only one study (Webster et al. 2019) fell into this intervention/outcome category, thus we were unable to perform a statistical synthesis. This cluster RCT from Uganda found a null effect of their programme on morbidity (g = 0.06 [95% CI: -0.02 to 0.13]), but like most studies, it was assessed as having a high risk of bias.

Mortality

There were no studies using community engagement in implementation autonomy that reported on child mortality.

Immunisation knowledge

There were no studies reporting on immunisation knowledge that used community engagement in implementation autonomy.

Immunisation attitudes

There were no studies reporting on immunisation attitudes that used interventions with community engagement in implementation autonomy.

Vaccination card retention

Only one study (Webster et al. 2019) fell into this intervention/outcome category, thus we were unable to perform a statistical synthesis. This cluster RCT from Uganda found a very small positive but non-significant effect of their programme on full childhood immunisation (g = 0.04 [95% CI: -0.06 to -0.15]), but like most studies, it was assessed as having a high risk of bias.

Experience and satisfaction with health services

No studies reporting on experience and satisfaction with health services used engagement in implementation autonomy.

Formal health worker's motivation, capacity and performance

No studies reporting on formal health worker's motivation, capacity and performance used engagement in implementation autonomy.

For peer review only - http://bmjopen.bmj.com/site/about/guidelines.xhtml

Appendix 13: Multiple engagement types

Full immunisation

We included k = 9 studies examining the impact of interventions with multiple engagement types on full childhood immunisation. The average outcome based on the random-effects model indicated that there was no effect on full childhood immunisation ($\hat{\mu} = 0.22$ [95% CI: -0.12 to 0.56], $z = 1.27$, $p = 0.20$). According to the Q-test, the true outcomes appear to be heterogeneous ($Q(8) = 388.56$, $p < 0.01$, $\hat{\tau}^2 = 0.25$, $I^2 = 97.94\%$, see Supplementary Figure 67). There was no indication of outliers in the context of this model, and no high or moderate quality studies, so we were unable to perform a sensitivity analysis. None of the moderators were significant sources of heterogeneity in the context of this model, including whether the specific combination of engagement types led to different effects (see Supplementary Table 3), but for full childhood immunisation we found no differences by engagement packages ($\hat{\beta} = 0.36$, p = 0.30 [95% CI: -0.31 to 1.02]).

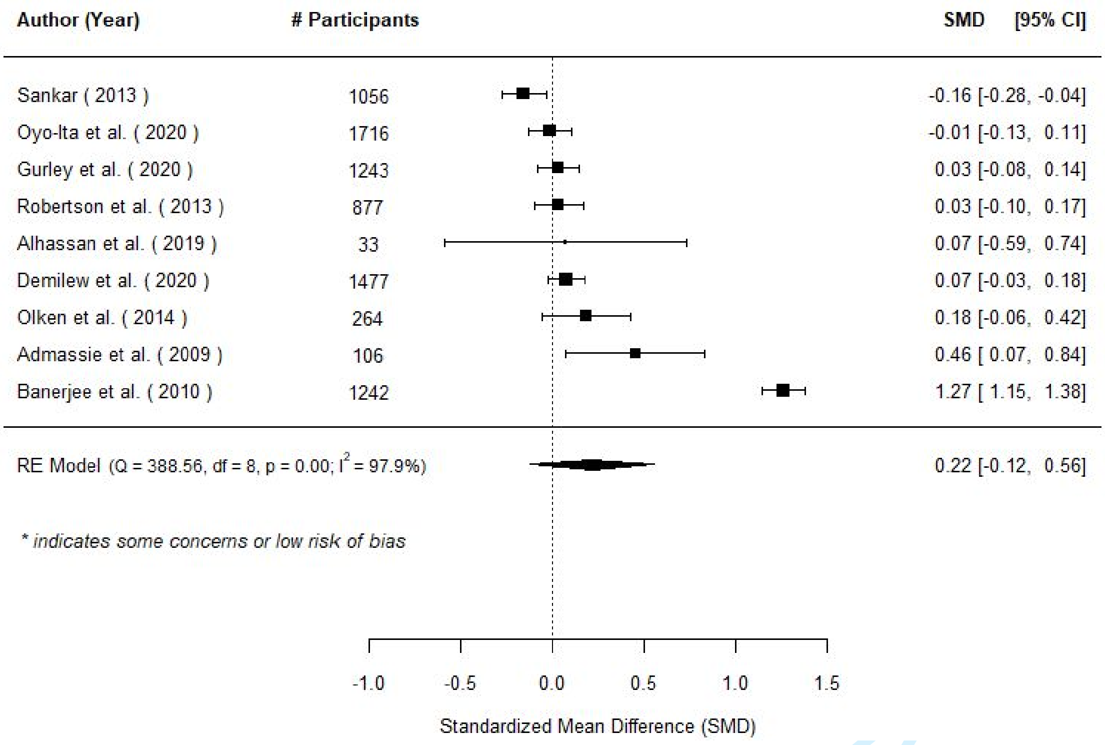

Supplementary Figure 67. Forest plot showing the observed outcomes and the estimate of the random-effects model for interventions with multiple engagement types on full immunisation

DPT3

We included k = 7 studies examining the impact of interventions with multiple engagement types on DPT3 vaccination. The average outcome based on the random-effects model indicated that there was a significant effect on DPT3 vaccination ($\hat{\mu} = 0.20$ *[95% CI:* 0.06 *to* 0.34], $z = 2.85$, $p < 0.01$). According to the Q-test, the true outcomes appear to be heterogeneous ($Q(6) = 49.71$, $p < 0.01$, $\hat{\tau}^2 = 0.03$, $I^2 = 87.93\%$). An examination of the studentized residuals revealed that none of the studies had a value larger than ± 2.69 and hence there was no indication of outliers in the context of this model. According to the Cook's distances, none of the studies could be considered to be overly influential. With no high or moderate quality studies, we were unable to perform a sensitivity analysis. There were several moderators that were significant sources of heterogeneity. Evaluation period was significant such that each additional month between the end of the intervention and the collection of outcome

For peer review only - http://bmjopen.bmj.com/site/about/guidelines.xhtml

data reduced the size of the effect by .01 standard deviation units ($\hat{\beta} = -0.01, p = 0.003$ [95% CI: -0.02 to -0.004]), suggesting smaller long term effect of the interventions. Publication year was also significant, such that each additional year reduced the size of the effect by .03 standard deviation units ($\hat{\beta} = -0.03, p = 0.03$ [95% CI: -0.05 to -0.003]). In other words, more recent studies have found smaller effects. Finally, baseline DPT3 coverage rates were significant such that a one unit increase in baseline DPT3 coverage was associated with a decrease of .56 in the effect of the programme ($\hat{\beta} = -0.56, p = 0.002$ [95% CI: -0.90 to -0.21]). In other words, the programmes were significantly more effective in areas with lower baseline coverage rates. We also tested whether the specific combination of engagement types led to different effects, but for DPT3 vaccination we found no differences by engagement packages ($\hat{\beta} = -0.07, p = 0.66$ [95% CI: -0.40 to -0.25]).

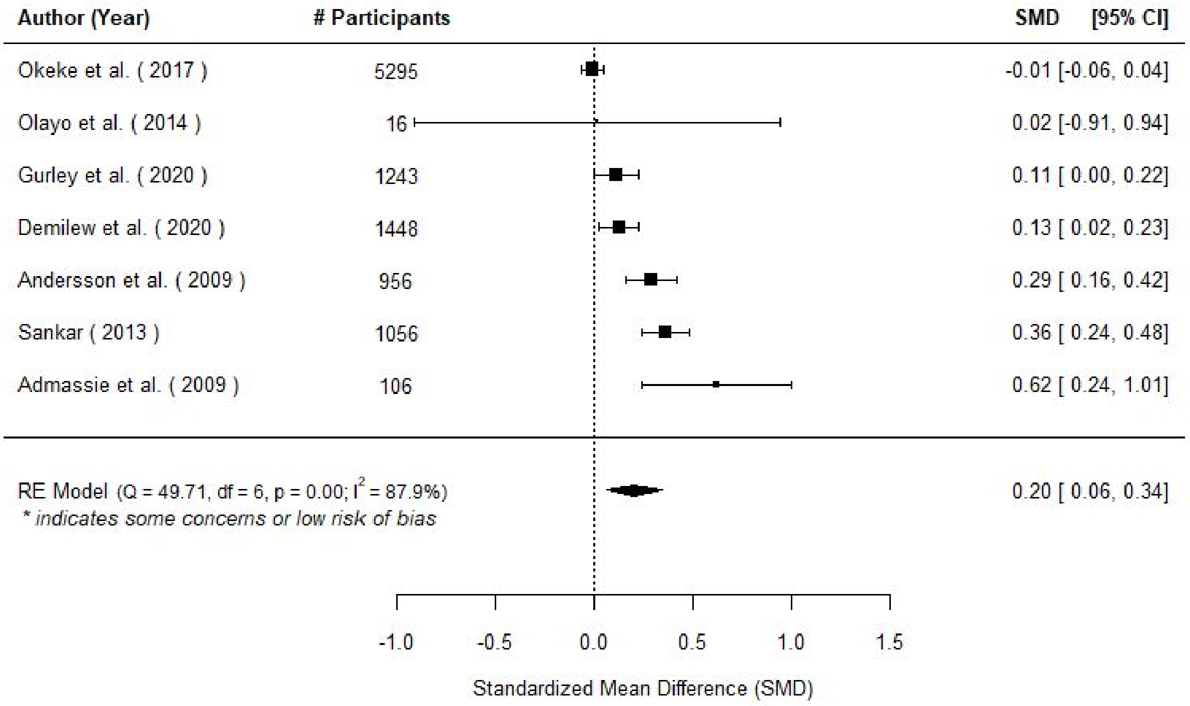

Supplementary Figure 68. Forest plot showing the observed outcomes and the estimate of the random-effects model for interventions with multiple engagement types on DPT3 vaccination

Measles

We included k = 6 studies examining the impact of interventions with multiple engagement types on measles vaccination. The average outcome based on the random-effects model indicated that there was no effect on measles coverage ($\hat{\mu} = 0.03$ [95% CI: -0.10 to 0.16], $z = 0.44$, $p = 0.66$). According to the Q-test, the true outcomes appear to be heterogeneous ($Q(5) = 37.76$, $p < 0.01$, $\hat{\tau}^2 = 0.02$, $I^2 = 86.76\%$; see Supplementary Figure 69). An examination of the studentized residuals revealed that one study (Sankar 2013) had a value larger than ± 2.64 and may be a potential outlier in the context of this model. According to the Cook's distances, none of the studies could be considered to be overly influential. Indeed, sensitivity analyses leaving each study out indicated that removing Sankar (2013) would increase the overall average effect ($\hat{\mu} = 0.09$ (95% CI: -0.02 to 0.19), but the effect would still be non-significant ($z = 1.58$ $p = 0.11$). With only one high or medium quality study, we were unable to conduct sensitivity analysis by study quality. None of the moderators that could be tested were significant in the context of this model (see Supplementary Table 3). For measles vaccinations, we were unable to test whether different combinations of community engagement produced different results because all but one study used a combination of engagement in the implementation and engagement as the intervention.

For peer review only - http://bmjopen.bmj.com/site/about/guidelines.xhtml

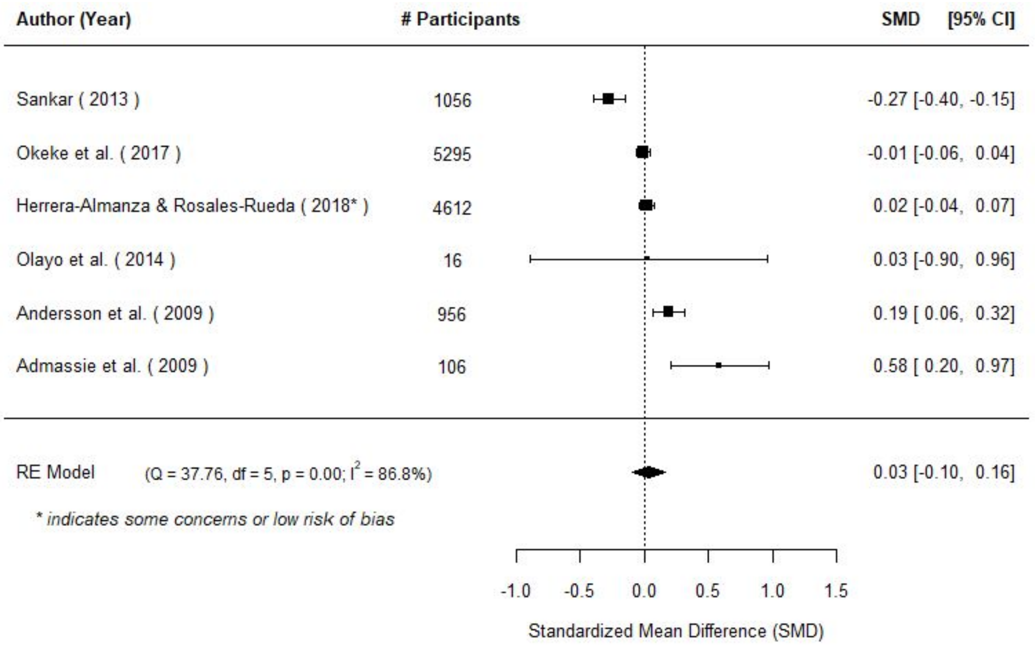

Supplementary Figure 69. Forest plot showing the observed outcomes and the estimate of the random-effects model for interventions with multiple engagement types on measles vaccination

Timeliness of full childhood immunisation

There were no studies using multiple engagement types that reported on the timeliness of full childhood immunisation.

Timeliness of DPT3 vaccination

Only one study reporting on timeliness of DPT3 vaccination used an intervention with multiple engagement types (Oyo-Ita et al. 2020). This RCT conducted in Nigeria found a small but not-significant positive effect (g = 0.10 [95%CI; -.0.2 to 0.23).This study was assessed as having a high risk of bias.

Timeliness of measles vaccination

Finally, only one study reporting on timeliness of measles vaccination used an intervention with multiple engagement types (Oyo-Ita et al. 2020). This RCT conducted in Nigeria found a moderate and significant positive effect (g = 0.25 [95% CI; 0.13 to 0.36]). They used a combination of engagement in implementation autonomy and engagement as the intervention. This study was also assessed as having a high risk of bias.

Secondary outcomes

Partial immunisation

We included a total of $k = 4$ studies in the analysis. The estimated average outcome based on the random-effects model was $\hat{\mu} = 0.28$ (95% CI: -0.01 to 0.56). Therefore, the average outcome did not differ significantly from zero ($z = 1.91$, $p = 0.06$), indicating no difference between the intervention and control groups (see Supplementary Figure 70). According to the Q-test, the true outcomes appear to be heterogeneous ($Q(3) = 101.75$, $p < 0.01$, $\hat{\tau}^2 = 0.08$, $I^2 = 97.05\%$). An examination of the studentized residuals revealed that none of the studies had a value

For peer review only - http://bmjopen.bmj.com/site/about/guidelines.xhtml

larger than ± 2.50 and hence there was no indication of outliers in the context of this model. According to the Cook's distances, none of the studies could be considered to be overly influential. Of the moderators we were able to test, none were significant sources of heterogeneity (see Supplementary Table 3). We were unable to test for differences among engagement packages because only one study used a combination of engagement in the design and engagement as the intervention (Gurley et al. 2020) while the remaining studies used a combination of engagement in implementation autonomy and engagement as the intervention.

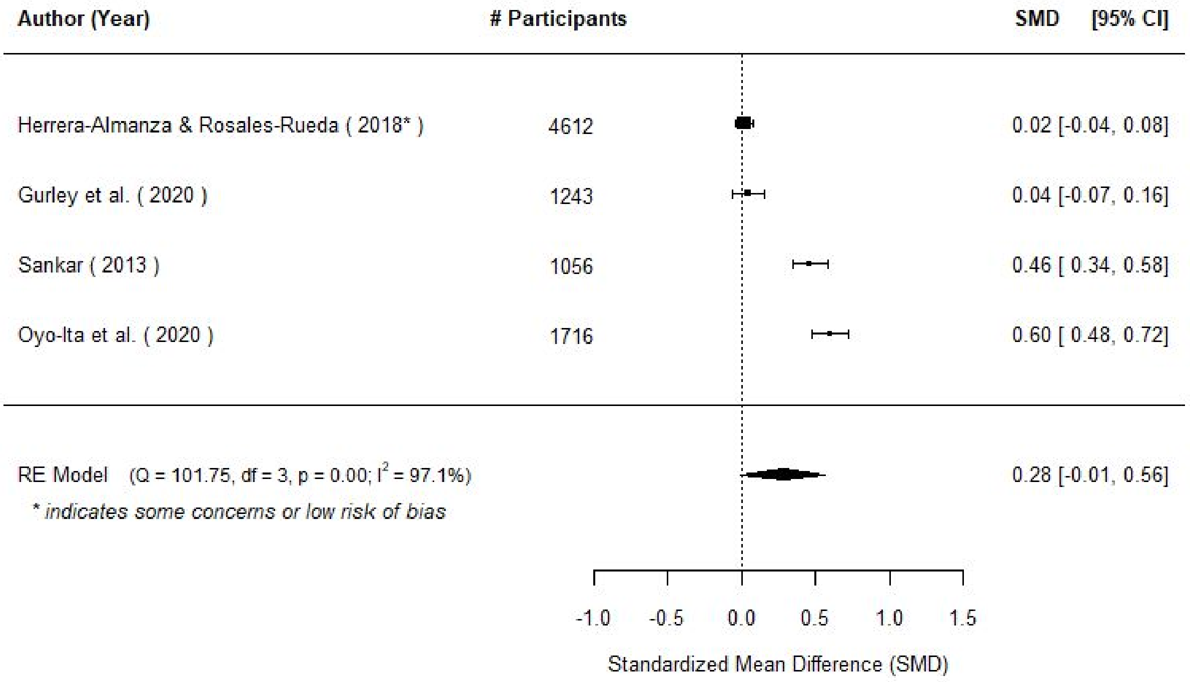

Supplementary Figure 70. Forest plot showing the observed outcomes and the estimate of the random-effects model for interventions with multiple engagement types on partial immunisation.

BCG

We included a total of $k = 4$ studies in the analysis. The estimated average outcome based on the random-effects model was $\hat{\mu} = 0.22$ (95% CI: -0.07 to 0.52). Therefore, the average outcome did not differ significantly from zero ($z = 1.48$, $p = 0.14$), indicating no differences between the treatment and control groups (see Supplementary Figure 71). According to the Q-test, the true outcomes appear to be heterogeneous ($Q(3) = 75.36$, $p < 0.01$, $\hat{\tau}^2 = 0.08$, $I^2 = 96.02\%$). An examination of the studentized residuals revealed that one study (Banerjee 2010) had a value larger than ± 2.50 and may be a potential outlier in the context of this model. Indeed, sensitivity analyses leaving each study out indicated that removing Banerjee (2010) would reduce the overall average effect ($\hat{\mu} = 0.05$ (95% CI: -0.13 to 0.23), but the effect is still positive and non-significant ($z = 0.56$, $p = 0.58$). According to the Cook's distances, none of the studies could be considered to be overly influential. With all studies assessed as high risk of bias, we were unable to conduct sensitivity analysis by study quality. We tested all moderators and only post-intervention versus change from baseline was a significant predictor of BCG vaccination, such that studies examining change from baseline had higher effects than studies examining post-intervention changes by .55 standard deviation units ($\hat{\beta} = -0.55$ [95% CI: -0.69 to -0.404], $p < .001$). We could not test for moderation by engagement components because all but one study used a combination of engagement in implementation autonomy and engagement as the intervention.

For peer review only - http://bmjopen.bmj.com/site/about/guidelines.xhtml

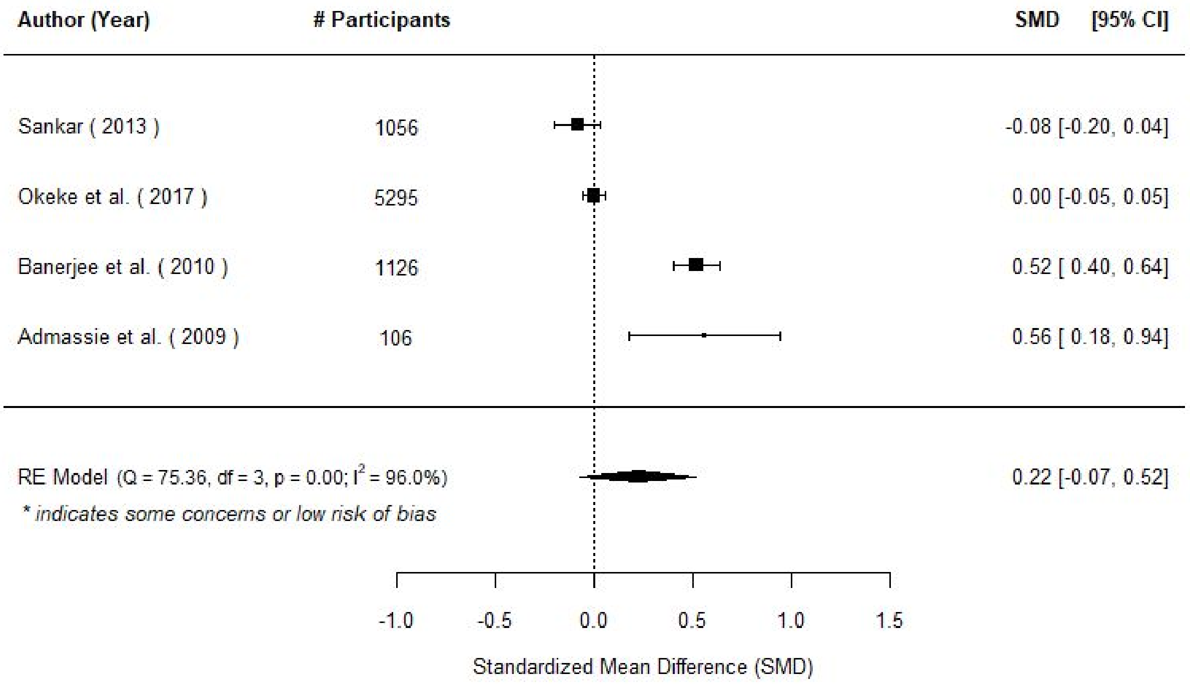

Supplemenatary Figure 71. Forest plot showing the observed outcomes and the estimate of the random-effects model for interventions with multiple engagement types on BCG vaccination.

DPT1

Only two studies reporting on DPT1 vaccination used interventions with multiple engagement types. The estimated average outcome based on the random-effects model was $\hat{\mu} = -0.17$ ([95% CI: -0.29 to -0.05], $z = -2.81$, $p = 0.005$), thus there was a significant negative impact of the programmes on DPT1 vaccination, (see Supplementary Figure 72). Given the small number of studies, this result should be interpreted with caution. According to the Q-test, the true outcomes appear to be homogeneous ($Q(1) = 0.03$, $p = 0.87$, $\hat{\tau}^2 = 0.00$, $I^2 = 0.00\%$). With only two studies, moderator analyses were not appropriate and tests of publication bias are not valid.

For peer review only - http://bmjopen.bmj.com/site/about/guidelines.xhtml

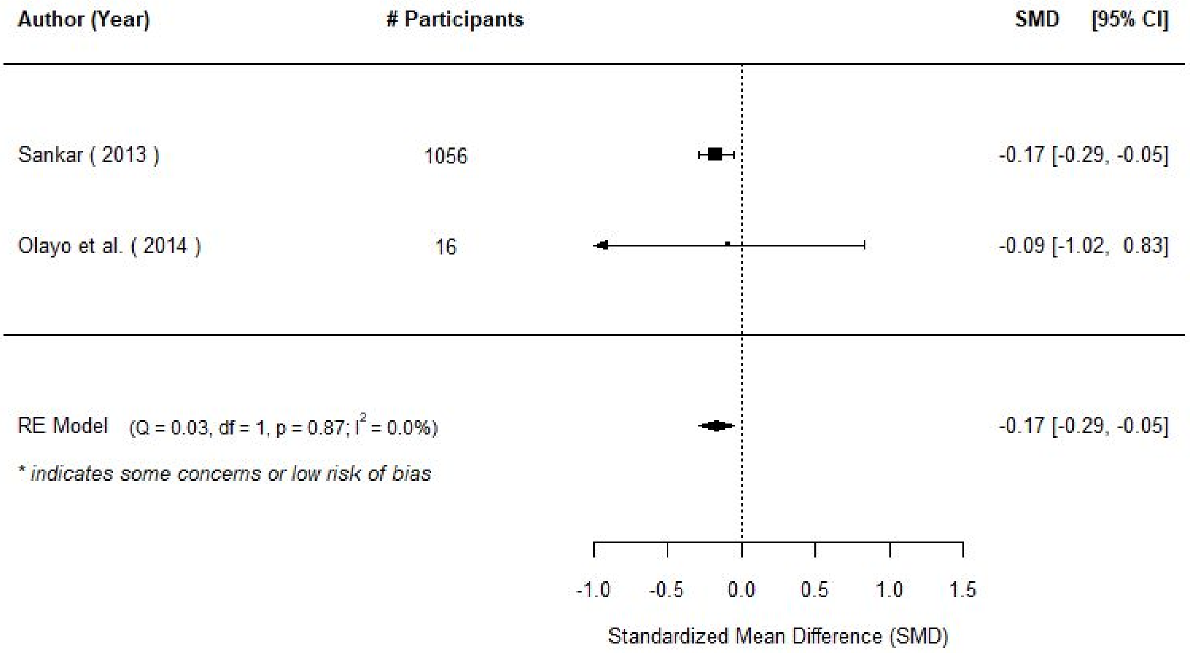

Supplementary Figure 72. Forest plot showing the observed outcomes and the estimate of the random-effects model for interventions with multiple engagement types on DPT1 vaccination.

DPT2

Only one study (Sankar 2013) fell into this intervention/outcome category, thus we were unable to perform a statistical synthesis. This quasi-experimental study from India found a null effect of their programme on OPV0 vaccination ($g = 0.10$ [95% CI: -0.02 to 0.22]), but like most studies, it was assessed as having a high risk of bias. Their programme included a combination of engagement in the implementation and engagement as the intervention.

OPV0

Only one study (Sankar 2013) fell into this intervention/outcome category, thus we were unable to perform a statistical synthesis. This quasi-experimental study from India found a significant positive effect of their programme on OPV0 vaccination ($g = 0.43$ [95% CI: 0.31 to 0.55]), but like most studies, it was assessed as having a high risk of bias. Their programme included a combination of engagement in implementation autonomy and engagement as the intervention.

OPV1

Only two studies reporting on OPV1 vaccination used interventions with multiple engagement types. The estimated average outcome based on the random-effects model was $\hat{\mu} = 0.22$ (95% CI: -0.15 to 0.59). Therefore, the average outcome did not differ significantly from zero ($z = 1.17$, $p = 0.24$, see Supplementary Figure 73). Given the small number of studies, this result should be interpreted with caution. According to the Q-test, the true outcomes appear to be homogeneous ($Q(1) = 3.61$, $p = 0.06$, $\hat{\tau}^2 = 0.05$, $I^2 = 72.32\%$). With only two studies, moderator analyses were not appropriate and tests of publication bias are not valid.

For peer review only - http://bmjopen.bmj.com/site/about/guidelines.xhtml

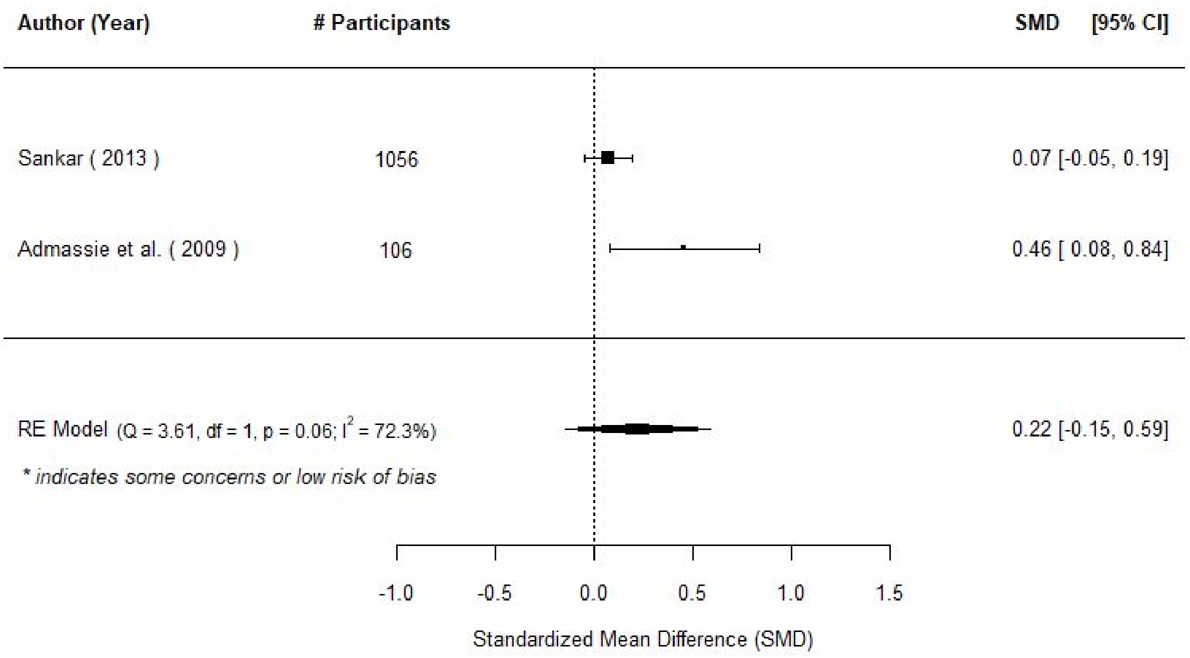

Supplementray Figure 73. Forest plot showing the observed outcomes and the estimate of the random-effects model for interventions with multiple engagement types on OPV1 vaccination.

OPV2

Only two studies reporting on OPV2 vaccination used interventions with multiple engagement types. The estimated average outcome based on the random-effects model was $\hat{\mu} = 0.34$ (95% CI: 0.19 to 0.50, $z = 4.28$, $p < 0.001$), indicating a small but significant benefit to the treated group compared to the control participants (see Supplementary Figure 74). Given the small number of studies, this result should be interpreted with caution. According to the Q-test, the true outcomes appear to be homogeneous ($Q(1) = 1.18, p = 0.28$, $\hat{\tau}^2 = 0.004$, $I^2 = 15.51\%$). With only two studies, moderator analyses were not appropriate and tests of publication bias are not valid. Both studies used a combination of engagement in implementation autonomy and engagement as the intervention.

For peer review only - http://bmjopen.bmj.com/site/about/guidelines.xhtml

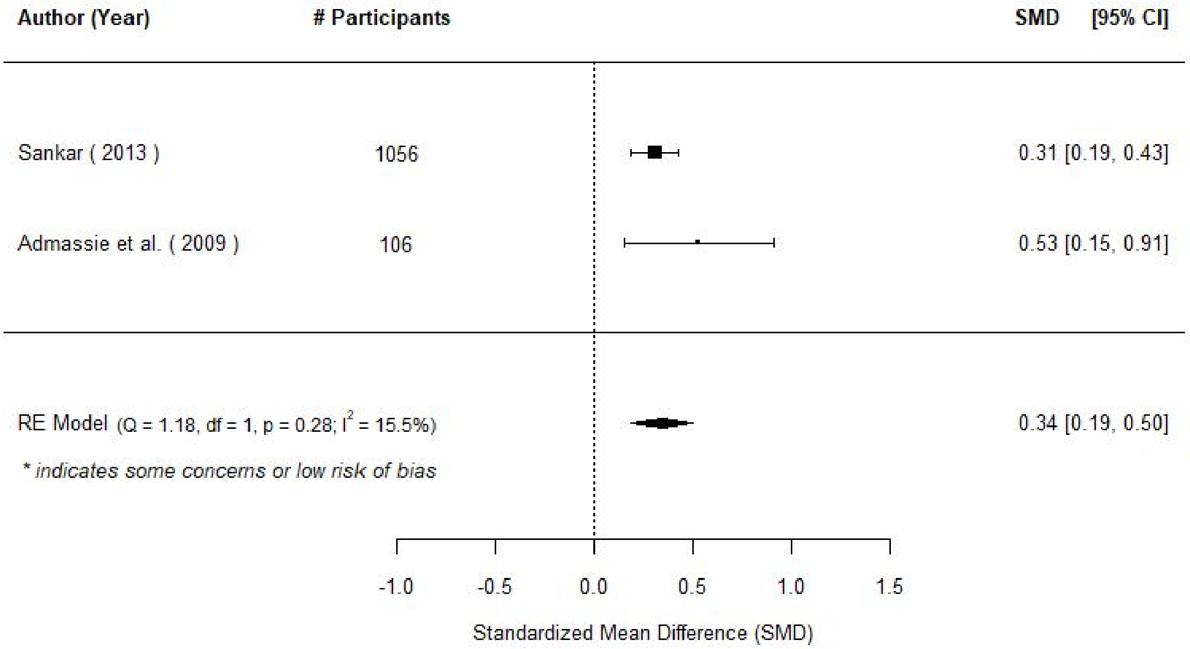

Supplementary Figure 74. Forest plot showing the observed outcomes and the estimate of the random-effects model for interventions with multiple engagement types on OPV2 vaccination.

OPV3

We included a total of $k = 3$ studies in the analysis. The estimated average outcome based on the random-effects model was $\hat{\mu} = 0.48$ ([95% CI: -0.24 to 1.20], $z = 1.30$, $p = 0.19$), indicating no difference between the intervention group and the control group (see Supplementary Figure 75). According to the Q-test, the true outcomes appear to be heterogeneous ($Q(2) = 185.96$, $p < 0.001$, $\hat{\tau}^2 = 0.39$, $I^2 = 98.92\%$). An examination of the studentized residuals revealed that none of the studies had a value larger than ± 2.39 and hence there was no indication of outliers in the context of this model. According to the Cook's distances, none of the studies could be considered to be overly influential. With only three studies contributing effects, we were unable to examine sources of heterogeneity.

For peer review only - http://bmjopen.bmj.com/site/about/guidelines.xhtml

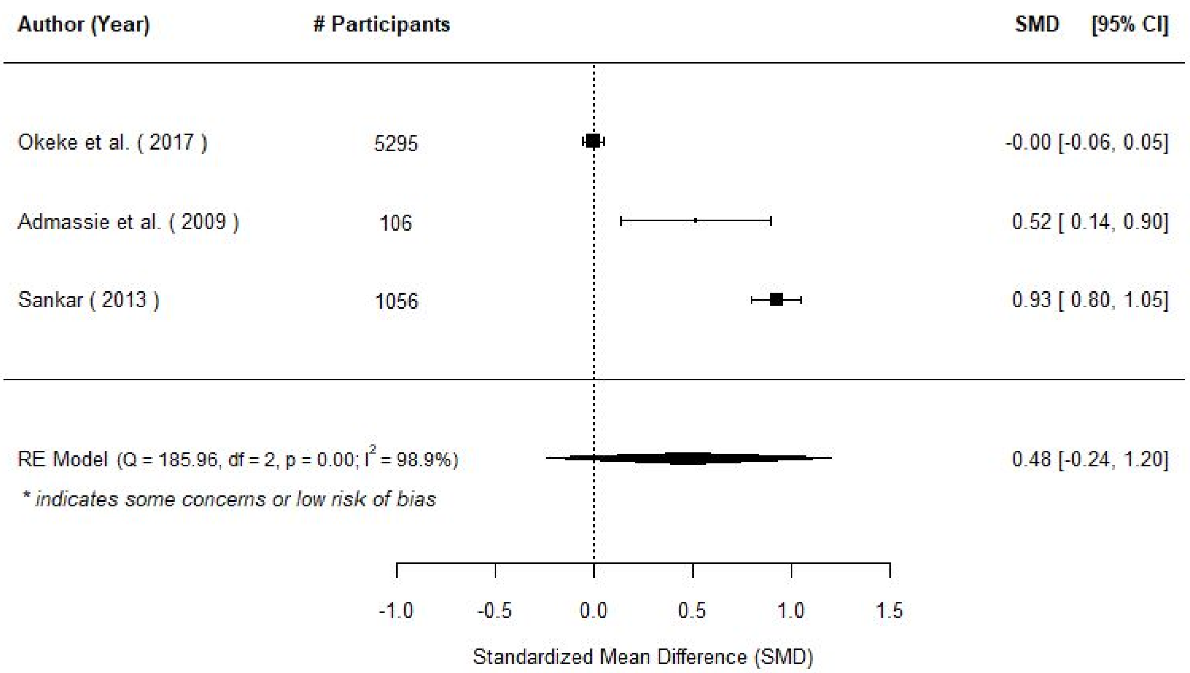

Supplementary Figure 75. Forest plot showing the observed outcomes and the estimate of the random-effects model for interventions with multiple engagement types on OPV3 vaccination.

Dropouts

Only two studies reporting on immunisation dropouts used interventions with multiple engagement types. All effects were computed such that a positive effect reflects a benefit to the treated group (e.g. a reduction in dropouts). The estimated average outcome based on the random-effects model was $\hat{\mu} = 0.18$ (95% CI: 0.06 to 0.30). Therefore, the average outcome differed significantly from zero, indicating a small but significant benefit to the treated group compared to the control group ($z = 2.92$, $p = 0.004$, see Supplementary Figure 76). Given the small number of studies, this result should be interpreted with caution. According to the Q-test, the true outcomes appear to be homogeneous ($Q(1) = 2.35$, $p = 0.13$, $\hat{\tau}^2 = 0.004$, $I^2 = 57.38\%$). With only two studies, moderator analyses were not appropriate and tests of publication bias are not valid.

For peer review only - http://bmjopen.bmj.com/site/about/guidelines.xhtml

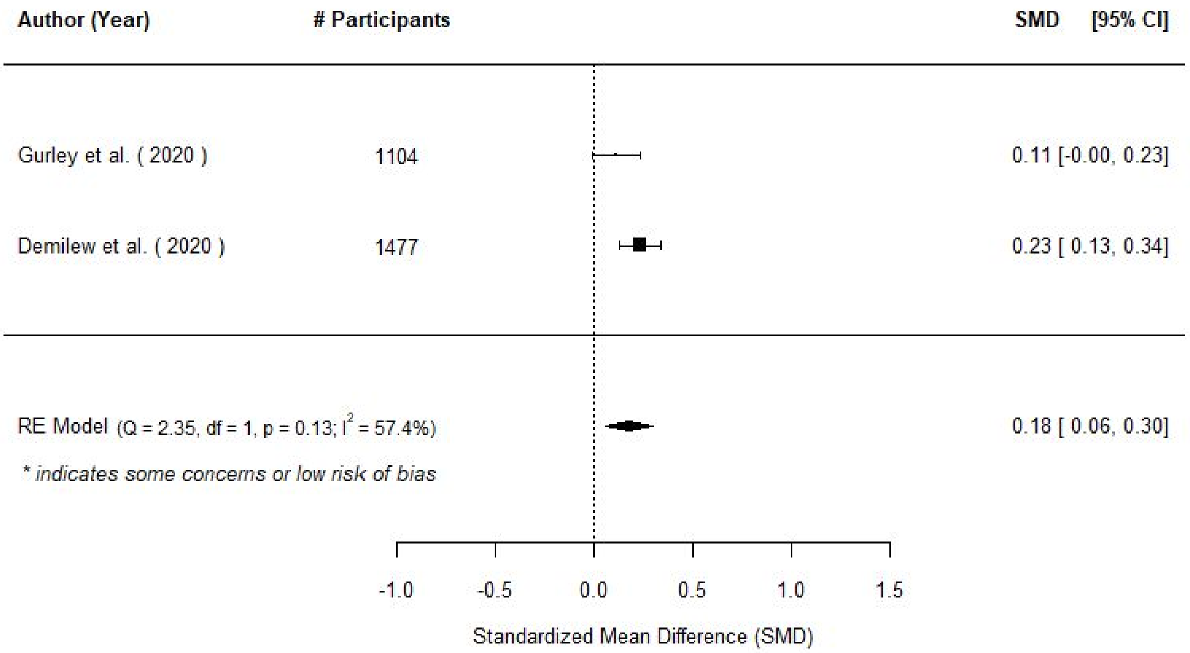

Supplementary Figure 76. Forest plot showing the observed outcomes and the estimate of the random-effects model for interventions with multiple engagement types on vaccination dropouts.

Morbidity

We used reports of diarrhea (most typically in the past two weeks) as a proxy for childhood morbidity. In all cases, effects were reverse coded such that positive effects always indicate a benefit to the treated group. Thus a positive effect here would be interpreted as a reduction in diarrhea among treated participants compared to control participants. Only two studies reporting on childhood morbidity used interventions with multiple engagement types. The estimated average outcome based on the random-effects model was $\hat{\mu} = -0.10$ (95% CI: -0.60 to 0.40). Therefore, the average outcome did not differ significantly from zero, indicating no difference between the treated group compared to the control group ($z = -0.38$, $p = 0.70$, see Supplementary Figure 77). Given the small number of studies, this result should be interpreted with caution. According to the Q-test, the true outcomes appear to be heterogeneous ($Q(1) = 5.23$, $p = 0.02$, $\hat{\tau}^2 = 0.11$, $I^2 = 80.88\%$). With only two studies, moderator analyses were not appropriate and tests of publication bias are not valid.

For peer review only - http://bmjopen.bmj.com/site/about/guidelines.xhtml

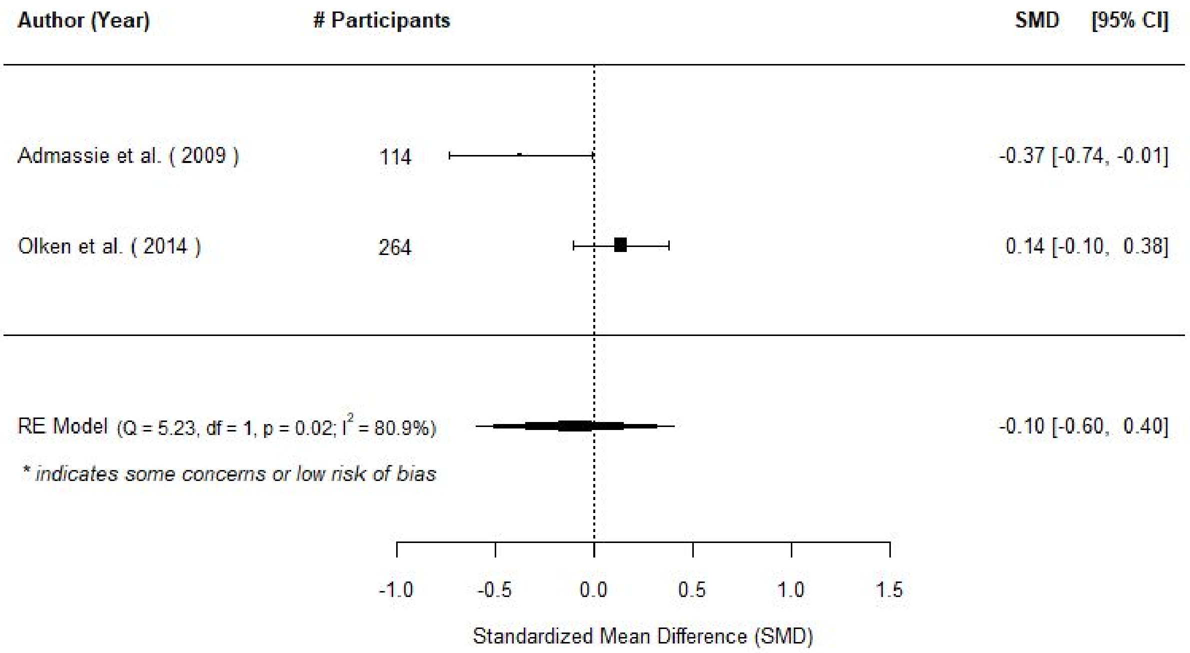

Supplementary Figure 77. Forest plot showing the observed outcomes and the estimate of the random-effects model for interventions with multiple engagement types on childhood morbidity.

Mortality

We included a total of $k = 3$ studies in the analysis. The observed outcomes ranged from -0.25 to -0.002. The estimated average outcome based on the random-effects model was $\hat{\mu} = -0.04$ (95% CI: -0.10 to 0.03, *see Supplementary Figure* 78). Therefore, the average outcome did not differ significantly from zero ($z = -1.15$, $p = 0.25$), indicating no difference between the intervention participants and control participants on child mortality. The Q-test for heterogeneity was not significant, but some heterogeneity may still be present in the true outcomes ($Q(2) = 5.37$, $p = 0.07$, $\hat{\tau}^2 = 0.0018$, $I^2 = 62.75\%$). An examination of the studentized residuals revealed that none of the studies had a value larger than ± 2.39 and hence there was no indication of outliers in the context of this model. According to the Cook's distances, none of the studies could be considered to be overly influential. With only three studies, moderator analyses were not appropriate and tests of publication bias are not valid.

For peer review only - http://bmjopen.bmj.com/site/about/guidelines.xhtml

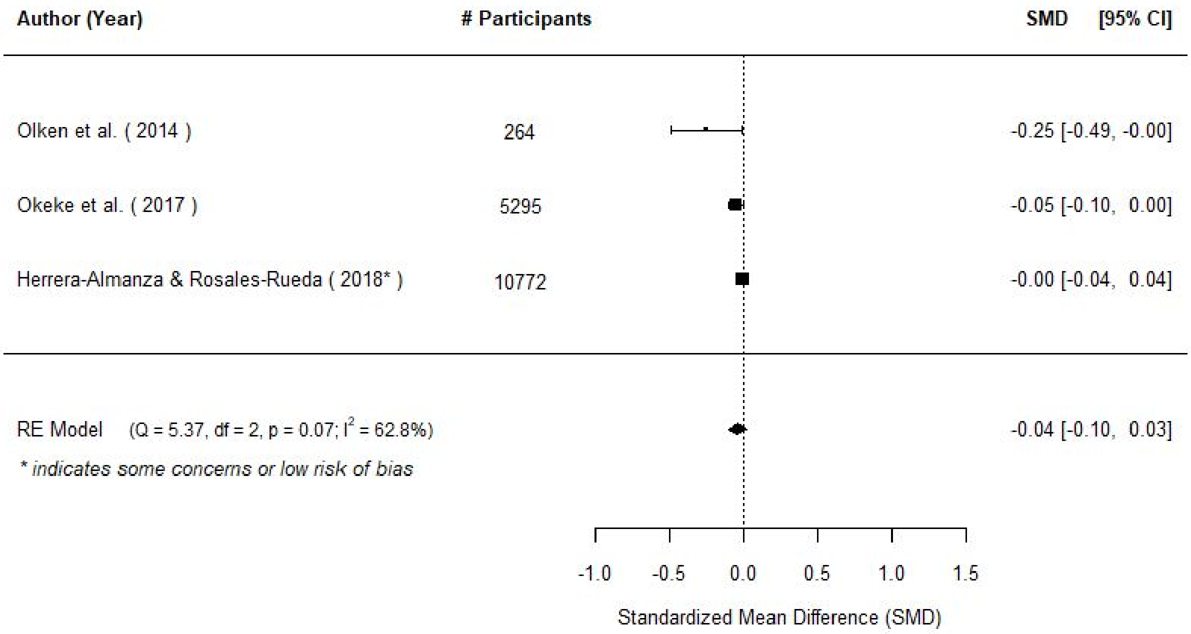

Supplementary Figure 78. Forest plot showing the observed outcomes and the estimate of the random-effects model for interventions with multiple engagement types on childhood morbidity.

Immunisation knowledge

Only two studies reporting on immunisation knowledge used interventions with multiple engagement types. The estimated average outcome based on the random-effects model was $\hat{\mu} =\ 0.09$ (95% CI: 0.04 to 0.13). Therefore, the average outcome differed significantly from zero, indicating a small but significant benefit to the treated group compared to the control group ($z = 3.55$, $p < 0.001$), see Supplementary Figure 79). Given the small number of studies, this result should be interpreted with caution. According to the Q-test, the true outcomes appear to be homogeneous ($\mathrm{Q}(1) = 0.19$, $\mathrm{p} = 0.66$, $\hat{\tau}^2 = 0.00$, $\mathrm{I}^2 = 0.00\%$). With only two studies, moderator analyses were not appropriate and tests of publication bias are not valid.

For peer review only - http://bmjopen.bmj.com/site/about/guidelines.xhtml

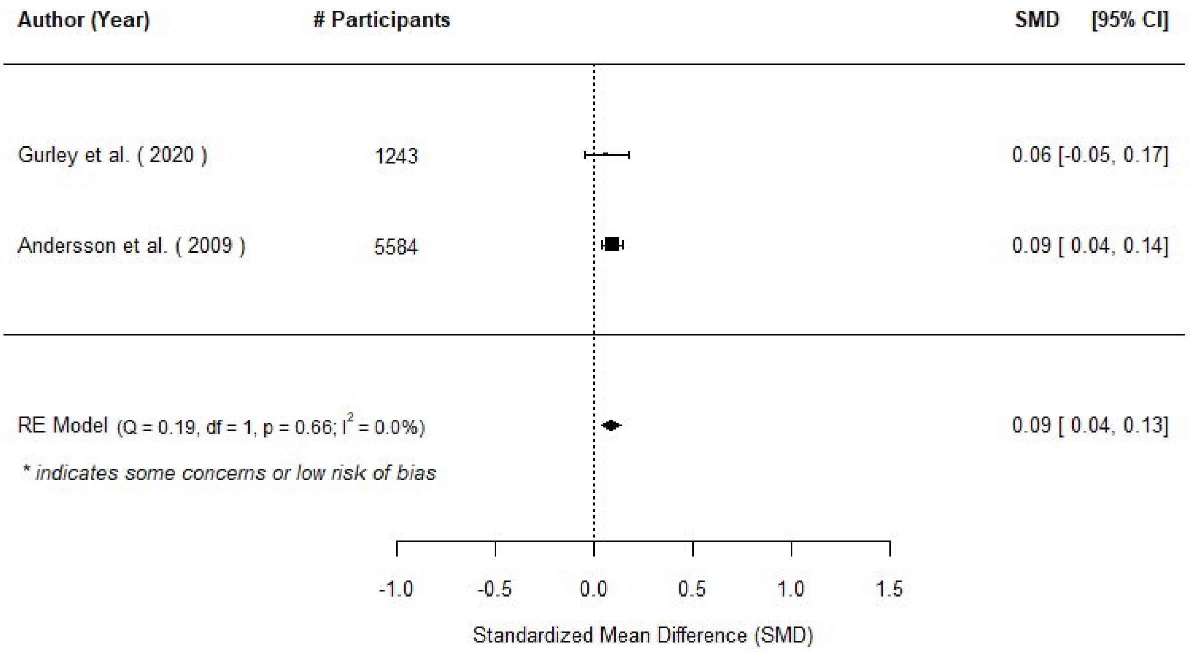

Supplementary Figure 79. Forest plot showing the observed outcomes and the estimate of the random-effects model for interventions with multiple engagement types on immunisation knowledge.

Immunisation attitudes

Only two studies reporting on immunisation attitudes used interventions with multiple engagement types. The estimated average outcome based on the random-effects model was $\hat{\mu} = 0.06$ (95% CI: 0.01 to 0.11). Therefore, the average outcome differed significantly from zero, indicating a small but significant benefit to the treated group compared to the control group ($z = 2.50, p = 0.01$), see Supplemenatry Figure 80). Given the small number of studies, this result should be interpreted with caution. According to the Q-test, the true outcomes appear to be homogeneous ($\mathrm{Q}(1) = 0.50$, $\mathrm{p} = 0.48$, $\hat{\tau}^2 = 0.00$, $\mathrm{I}^2 = 0.00\%$). With only two studies, moderator analyses were not appropriate and tests of publication bias are not valid.

For peer review only - http://bmjopen.bmj.com/site/about/guidelines.xhtml

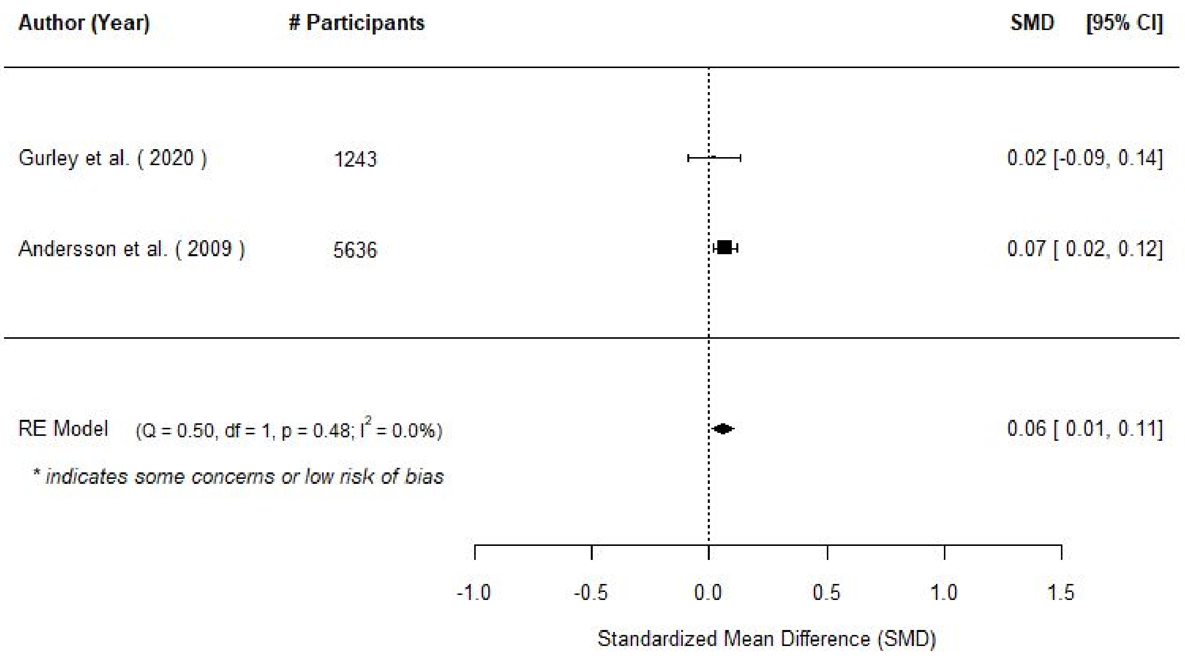

Supplementary Figure 80. Forest plot showing the observed outcomes and the estimate of the random-effects model for interventions with multiple engagement types on immunisation attitudes

Vaccination card availability

Only two studies reporting on vaccination card availability/retention used interventions with multiple engagement types. The estimated average outcome based on the random-effects model was $\hat{\mu} = -0.01$ (95% CI: -0.05 to 0.02). Therefore, the average outcome did not differ significantly from zero ($z = -0.78$, $p = 0.43$, see Supplementary Figure 81). Given the small number of studies, this result should be interpreted with caution. According to the Q-test, the true outcomes appear to be homogeneous ($\mathrm{Q}(5) = 0.13$, $\mathrm{p} = 0.43$, $\hat{\tau}^2 = 0.00$, $\mathrm{I}^2 = 0.00\%$; With only two studies and no heterogeneity, moderator analyses were not appropriate and tests of publication bias are not valid.

For peer review only - http://bmjopen.bmj.com/site/about/guidelines.xhtml

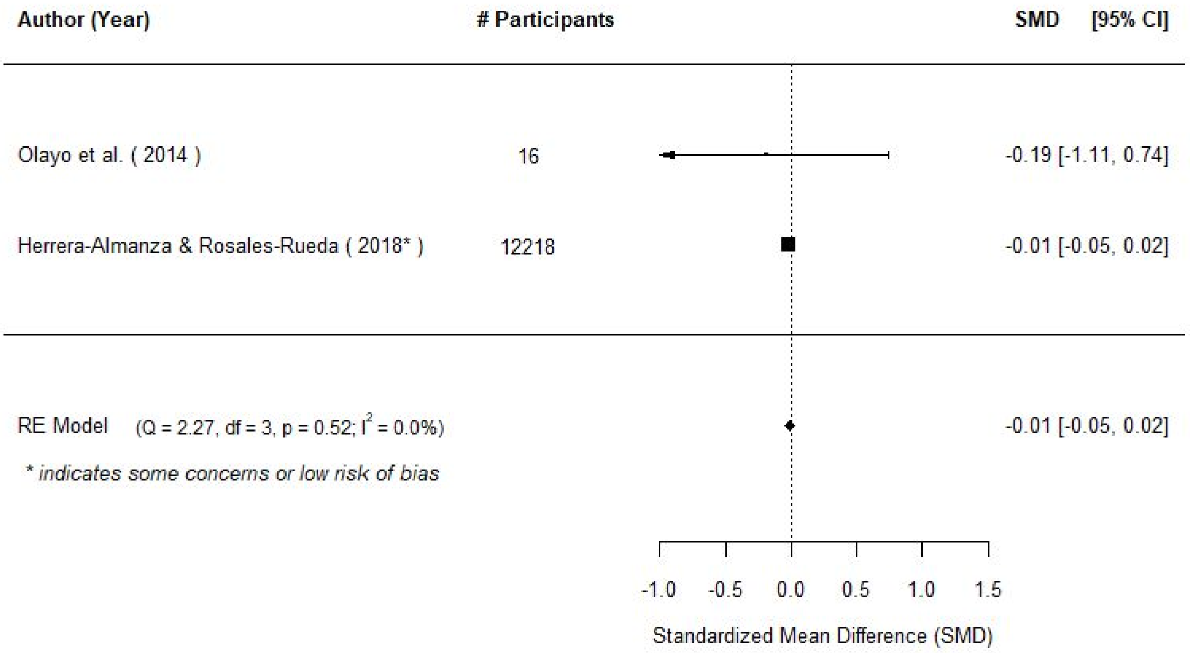

Supplementary Figure 81. Forest plot showing the observed outcomes and the estimate of the random-effects model for interventions with multiple engagement types on vaccination card availability

For peer review only - http://bmjopen.bmj.com/site/about/guidelines.xhtml

Appendix 14: Qualitative synthesis of results

Barriers to immunisation

Most information on barriers came from studies which used engagement as the intervention. Often, multiple barriers were cited in the same sentence. The vast majority of the barriers were identified through individual interviews and focus group discussions in the primary papers from which they were drawn. In addition, these were often supported by the authors' reflections, implementers' experiences, and observations. Sensitivity analysis, in which only qualitative papers that received an assessment score greater than 20 were included, indicated similar results. Therefore, we view the strength of the evidence to be high. Behavioural, social and practical barriers faced by caregivers were more consistently cited than direct constraints within the health system (Supplementary Figure 82, Supplemetary Table 4).

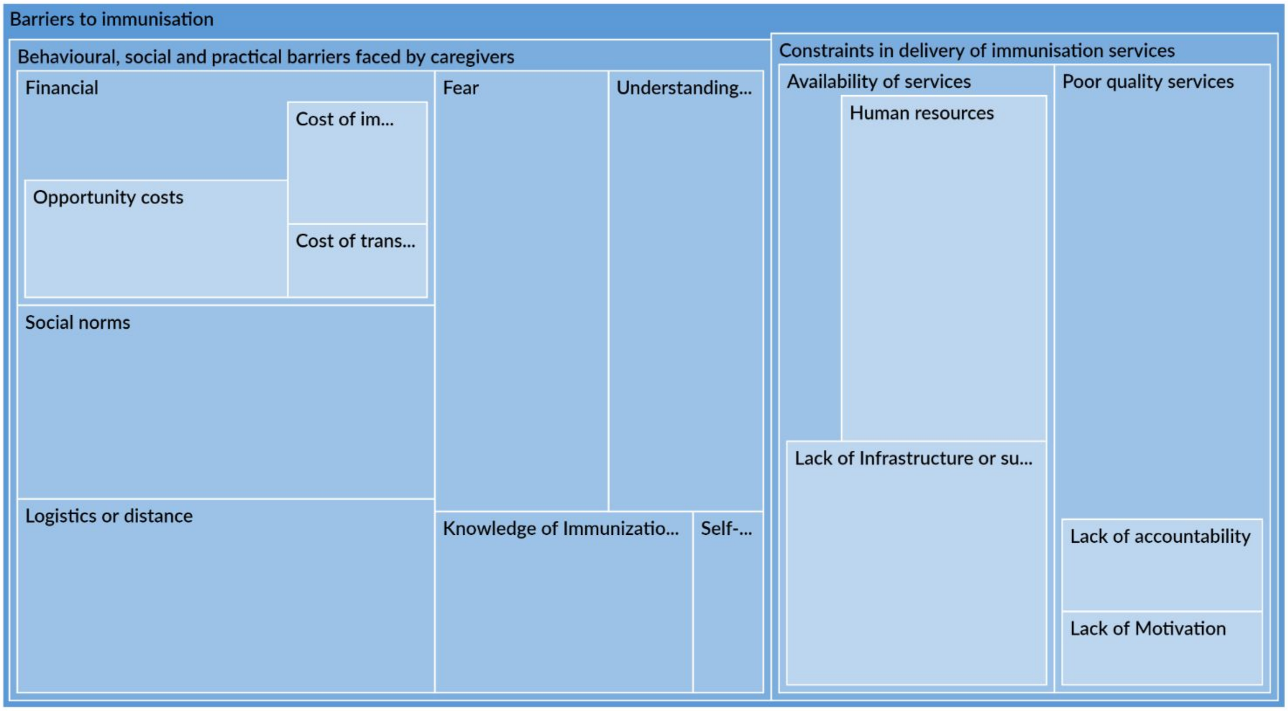

Supplementary Figure 82: Summary figure: Behavioural, social and practical barriers faced by caregivers were more consistently experienced than constraints in delivery of immunisation services[1]

The size of the rectangles reflects the number of times a unique theme was mentioned by an IE and its associated papers. Larger rectangles indicate that a theme was mentioned more times. Nested boxes reflect the proportion of times the main theme text also fell into the sub-them.

Supplementary Table 4: Comparison of the barriers to immunisation in communities in which community engagement activities were undertaken.

	Community engagement as the intervention	Community engagement in the design of the intervention	Community engagement in the implementation autonomy of the intervention

[1] The hierarchy charts showing barriers by type of community engagement are in Appendix H: barriers to immunisation

For peer review only - http://bmjopen.bmj.com/site/about/guidelines.xhtml

**Availability of resources**	Staff<br>Absenteeism<br>Stockouts<br>Building infrastructure	Staff<br>Stockouts	Staff<br>Post abandonment<br>Stockouts
**Quality of services**	Attitudes of health workers	Attitudes of health workers<br>Quality affected attendance	Attitudes of health workers<br>Services not implemented as intended
**Costs**	Other costs<br>Cost of the vaccine*	Other costs	Other costs<br>Cost of the vaccine
**Logistics or distance**	Distance	Convenience<br>Distance and access	Convenience<br>Transport
**Social norms**	Religious beliefs<br>Beliefs of others		Spousal consent<br>Religious beliefs
**Fear**	Side effects (and overcoming this fear)<br>Trust in the government or medicine	Side effects (from past experience)	
**Understanding of importance**	Cost and importance<br>Fear and importance		

Behavioural, social and practical barriers faced by caregivers

The most consistent behavioural, social or practical barrier faced by caregivers was cost. However, the kind of cost which posed a barrier was inconsistent, and mostly not that of the vaccination itself. Associated costs include opportunity costs, transport costs, and the costs of treating side effects. The cost of the immunisation itself was mentioned as a barrier by community members that received engagement as the intervention or engagement in the implementation of the intervention.

Logistics and distance were also consistent barriers to immunisation in settings where all three types of interventions occurred. The nature of the logistical or distance challenge was variable. Distance alone was reported as an issue in the communities with engagement as the intervention. However, Olken 2014 (World bank 2018) mention other logistical barriers such as wait times and language barriers. In settings where the community was involved in the design or implementation of the intervention, convenience was an issue in addition to distance. Community members in these areas reported issues related to wait times and the timing of clinic visits. Adamu 2019 (Adamu 2019 pre-implementation and Adamu 2019 Dissertation) mention demand being lower than it could be because women were unable to arrive at clinics at specified times due to competing demands.

Fear of side effects was common in communities in which engagement was the intervention or engagement was used in the design of the intervention. The concern was consistently related to known side effects and affected by past experience of side effect and trust in the government or medicine. The issue of vaccines causing sterilization was also mentioned. In one case, the intervention reduced these fears (Gurley 2020 (Gurley 2020)).

Social norms around immunisation posed consistent barriers; however, the source of the social restriction was variable. Spousal consent limited immunisations in areas where engagement was used in the implementation of interventions;

For peer review only - http://bmjopen.bmj.com/site/about/guidelines.xhtml

women could not vaccinate their children without the explicit approval of their husband, who may refuse. Religious beliefs sometimes resulted in the preference for non-Western medicine. In areas that received engagement as the intervention, religious beliefs and the influence of beliefs held by others sometimes inhibited immunisation. In particular, mother-in-laws and other family members who do not value immunisation may pose a barrier to immunisations.

Failure to *understand the importance of vaccination* was not consistently observed but reported in settings that received engagement as the intervention. When this barrier was mentioned, it was always paired with another, such as cost or fear. Authors implied that these other barriers were experienced and could not be overcome because there was a lack of understanding of the importance of vaccinations.

Constraints in delivery of immunisation services

Constraints in the delivery of immunisation services were widely reported but did not seem to be as common of a barrier as the behavioural, social and practical barriers faced by caregivers. The *availability of services* was the most consistently reported delivery barrier across all three types of engagement. Limited staff and stockouts were dominant delivery challenges. Communities that had engagement as the intervention or were engaged in implementation consistently have issues with staff being assigned but unavailable. Poor infrastructure, such as dilapidated buildings, was a challenge in communities that were involved in the design of the intervention. In areas where engagement was the intervention or the community was involved in the design of the intervention, the *quality of services* was also a more consistently reported barrier than any behavioural, social, or practical barrier faced by caregivers. Often, these were related to health worker attitudes, either a lack of motivation or conflict with caregivers.

Barriers to immunisation by type of community engagement

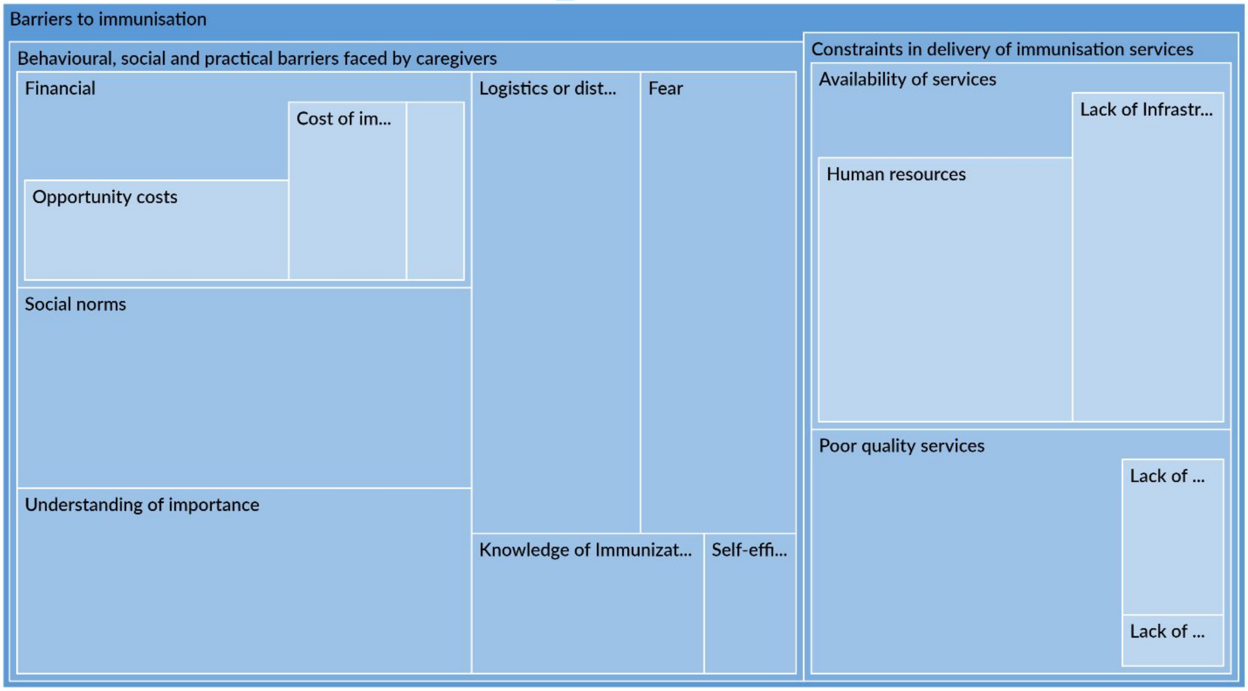

Figure 83: Engagement as intervention

For peer review only - http://bmjopen.bmj.com/site/about/guidelines.xhtml

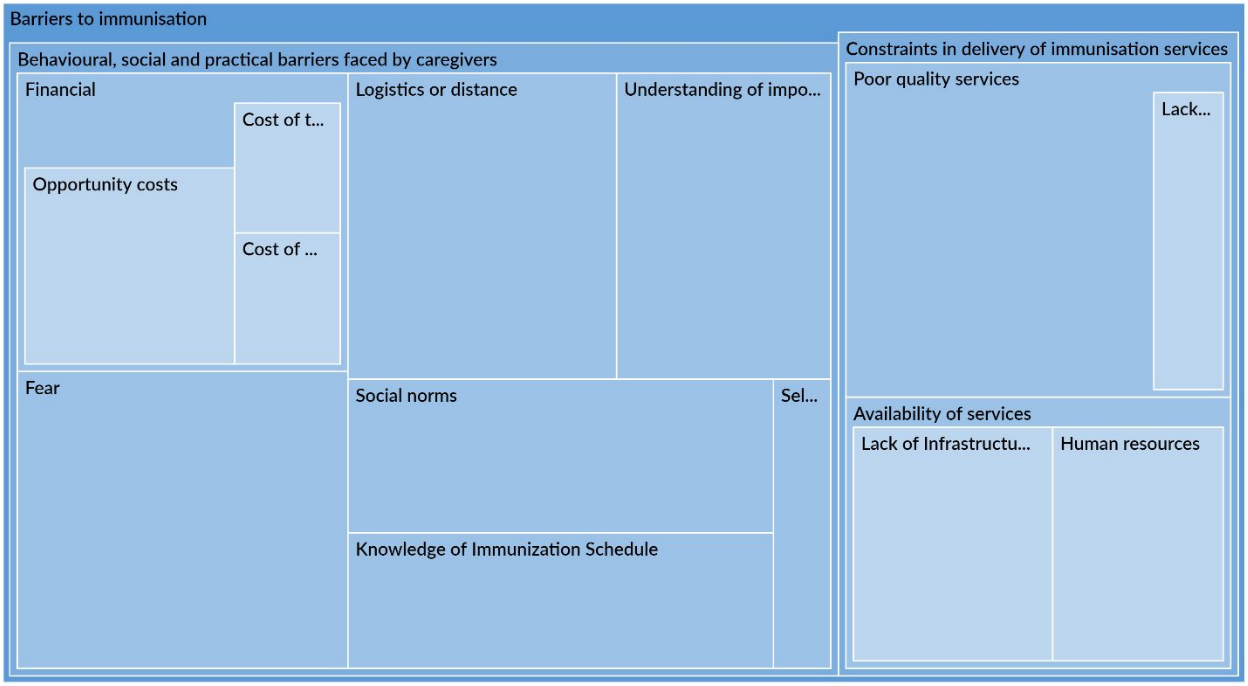

Figure 84: Engagement in design

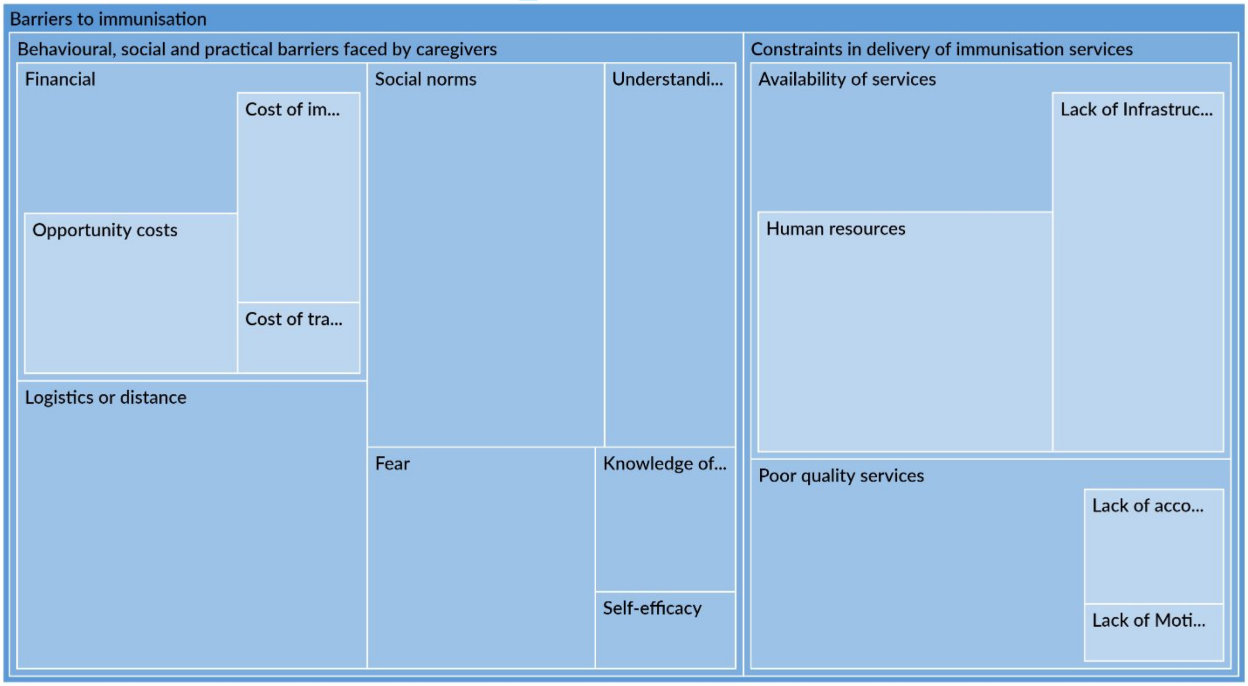

Figure 85: Engagement in implementation autonomy

Supplementary Table 5: Characteristic quotes of themes identified related to barriers to immunisation for interventions were community engagement.

For peer review only - http://bmjopen.bmj.com/site/about/guidelines.xhtml

Primary paper	Paper from which citation comes	Theme demonstrated	Quote	Page number
Financial				
Okeke et al 2017	Okeke et al 2017	Cost of vaccination	costs associated with use of services and lack of adequate transportation, hindered uptake.	54
Andersson 2009	Mitchell 2009	Other costs	Yet the groups noted that immediate financial and time costs associated with having a child vaccinated weigh very heavily in comparison with the potential costs associated with measles in the future.	7
Social norms				
Banerjee et al 2020	Appendix K	Religion	Religion emerged as an implicit theme. Only two respondents mentioned explicitly that religious belief might inhibit immunisation: that God would decide the fate of their child.	7
Andersson 2009	Cockroft 2009	Beliefs of others	"My mother-in-law says 'What kind of children have you produced that they ought to be vaccinated? We were never vaccinated, so why are you behaving so delicately?'" (female group, Khanewal)	8
Understanding of importance				
Olken 2014	Grayman 2013	Costs and importance	Typically the answer is that the parents are too busy working on their farms, or they feel that posyandu is no longer necessary after they are older than two years of age.	66
Paramanik 2018	Paramanik 2020	Fear and importance	As reported by ASHAs the mothers-in-law were not able to accept the concept of vaccination. They apparently argued on the ground that they had children too and that they never took them for any vaccination, that post vaccination the child gets fever which causes sleep disturbance for the child and in turn negatively affects the whole family.	84

For peer review only - http://bmjopen.bmj.com/site/about/guidelines.xhtml

Logistics or distance

Alhassan 2019	Alhassan 2019	Distance	In Ghana, access to maternal and child health services is impeded by longer travel times to health facilities.	2
Oyo-Ita 2020	Oyo-Ita 2020	Distance	children whose mothers found it difficult to reach the health facility	

Fear

Banerjee et al 2020	Appendix K	Trust	A couple of respondents suggested this could be interacting with a lack of trust of front line health workers.	20
Oyo-Ita 2020	Oyo-Ita 2020	Side effects	She said she doesn't want anybody to give her child injection so that the child will not become sick	31
Gurley 2020	Gurley 2020	Overcoming fear of side effects	We observed changes in how FGD respondents discussed side effects over the course of the study, from a perception that they were severe and not worth the risk of vaccination, to a sense they could be handled and are not a reason to forgo vaccination. I also took my child for vaccination. She cried for two days and I said let her cry; at least she will be safe in future from diseases. Mothers FGD, September 2017, intervention village	35

Human resources

Gurley 2020	Gurley 2020	Staff	In addition to suboptimal ANM clinical quality, nine ANM posts were vacant during the study period, resulting in suboptimal availability of services in some communities, which was beyond our influence	38
Olken 2014	Rahayu 2008	Absenteeism	the community is often disappointed with the services of puskesmas officers who do not attend posyandu services as this means that immunisations must be postponed until the posyandu session scheduled for the next month.	33

For peer review only - http://bmjopen.bmj.com/site/about/guidelines.xhtml

Infrastructure and supplies				
Okeke et al 2017	Okeke et al 2017	Building infrastructure	That is supposed to be our office. If you get there now, half of it is just sand; even the doors to this clinic are not closing, they are eating up by termite. the condition of two of the facilities was perceived to be detrimental to their ability to deliver care.	43
Findley et al 2013	Doctor et al 2011	Vaccine stockout and long wait times	67% of parents were unable to receive all immunisations reported lack of vaccine as a problem, and 13% had difficulties with the long wait.	17
Multiple barriers				
Okeke et al 2017	Okeke et al 2017	Cost of vaccination and logistics	costs associated with use of services and lack of adequate transportation, hindered uptake.	
Arifeen 2019	Billah 2018	Many simultaneous barriers	Furthermore, the lack of trust in formal health care providers, high out of pocket costs of health care in the public sector, and challenges with accessibility has led to the rapid proliferation of informal health providers to fill the gap between supply and demand across both rural and urban areas	2
Tandon 1988	Islam 2013	Many simultaneous barriers	low capacity to supervise monitor and implement micro plans at district level, lack of effective vaccine distribution to immunisation sites, ageing and poorly maintained Cold Chain, lack of adequately trained human resources, low managerial and support capacity at the state and district immunisation units and weak management of fund flows.	10

Supplementary Table 6: Characteristic quotes of themes identified related to barriers to immunisation for interventions that used community engagement in their design.

For peer review only - http://bmjopen.bmj.com/site/about/guidelines.xhtml

Primary paper	Paper from which citation comes	Theme demonstrated	Quote	Page number
Financial				
Anderson 2009	Andersson 2009	Other costs	Discussions in our focus groups confirmed the importance of poverty as a barrier to vaccination in many cases, as parents described being unable to afford the costs of the supposedly “free” immunisations: travel costs, opportunity costs, and demands for unofficial payments.	10
Fear				
Dipeolu 2017	Oladepo 2019	Fear of side effects	Prominent factors perceived as affecting timely and full completion of routine immunisations for children aged below 12 months include lack of awareness of immunisation (61.6%) and of subsequent doses (58.4%), and fear of side effects (59.7%). Others include rumours (for example, an association with future infertility)	464
Adamu 2019	Adamu 2019 Dissertation	Past experience	Experiences with the side effects of vaccines can influence the behaviour of caregivers and reduce their motivation to immunize eligible children.	134
Logistics				
Andersson 2009	Mitchell 2009	Physical access to facilities	In both urban and rural areas, access to a government facility providing vaccinations, a key equity factor, was a determining factor for uptake.	8
Nagar 2018	Nagar 2016	Convenience	not knowing where to go, not having time or mutually convenient time, facing long wait times at the camp, having fear of side effects, and acting under misguided advice	5
Human resources				

For peer review only - http://bmjopen.bmj.com/site/about/guidelines.xhtml

Borkum 2014	Rangarajan 2013	Staff	Staff vacancies are common, especially for lady health visitors and pharmacists. Few of the PHCs surveyed are fully staffed.	
Infrastructure or supplies				
Domek 2019	Domek et al 2018 Vaccine hesitancy	Stock outs	Of note, Guatemala experienced significant political instability during our study period, which led to considerable vaccine shortages experienced by all of our clinics.	3
Poor quality				
Dipeolu 2017	Dipeolu 2017	Attitudes of health workers	Attitudes and behaviours of healthcare workers such as treating mothers in an unfriendly, disrespectful, or even abusive manner are frequently cited as discouraging children's vaccination. Healthcare workers reportedly screamed at mothers who forgot the child's card, missed a scheduled vaccination	49
Gurely 2020	Gurley 2020	Quality affected attendance	For example, the existence of ANM vaccinators did not ensure their quality, and we observed that suboptimal clinical ANM quality discouraged retention across the vaccine schedule and perhaps negatively impacted beneficiaries' trust in the health system.	37

Supplementary Table 7: Characteristic quotes of themes identified related to barriers to immunisation for interventions that used community engagement in their implementation

**Primary paper**	**Paper from which citation comes**	**Theme demonstrated**	**Quote**	**Page number**
Financial				
Okeke et al 2017	Okeke et al 2017	Cost of the vaccine itself	costs associated with use of services and lack of adequate transportation, hindered uptake.	54

For peer review only - http://bmjopen.bmj.com/site/about/guidelines.xhtml

Adamu 2019	Adamu 2019 Dissertation	Other costs associated with vaccination	However, the cost of treating vaccine reactions like fever that might occur following immunisation are borne for the caregivers, which can result in out-of-pocket expenditure.	133
Social norms				
Adamu 2019	Adamu 2019 Dissertation	Spousal consent	In addition, we found that the high level of social control that men have over women in this area could also cause MOV among children. Caregivers reported that without their husband's consent, they still cannot vaccinate their children, even if they're in a health facility for other preventive or curative services.	133
Olken 2014	Rahayu 2008	Religious beliefs	The community trusts and believes in the choice that their parents made to use the dukun beranak, to the point where it becomes the norm.	34
Logistics or distance				
Webster 2019	Webster et al 2019	Convenience	Key reasons cited for non-immunisation were related to lack of convenience; the aforementioned distance between homes and health facilities, moving to the fields during farming seasons,	42
Alhassan 2019	Alhassan 2019	Transport	In Ghana, access to maternal and child health services is impeded by longer travel times to health facilities.	2
Human resources				
Olken 2014	Febriany 2011	Post abandonment	Poor facilities adversely affected the availability and utilization of MCH and basic education services. The midwife did not want to live in the village because here there is no electricity and no water (FGD Female-NTT).	9

For peer review only

For peer review only - http://bmjopen.bmj.com/site/about/guidelines.xhtml

Herrara-Almanza 2018	Herrara-Almanza & Rossales-Rueda 2018	Lack of personnel	Poor transportation infrastructure and a shortage of medical personnel limit the basic health access to the population living in remote areas	7
Infrastructure or supplies				
Webster 2019	Webster et al 2019	Vaccine stockouts	Furthermore, vaccine stock-outs at health facilities discouraged caregivers from attending vaccination services.	42
Multiple barriers				
Webster 2019	Webster et al 2019	Demand and supply side	Health system factors affecting immunisation service delivery and uptake in northern Uganda include interruptions in the vaccine cold chain due to poor management of equipment and supplies, limited supervision of health teams by the district health teams, low staffing levels, long distances to the health facilities especially in the hard-to-reach areas, and limited resources to support outreach services. There is also a lack of good quality data to support decision-making.	1
Olken 2014	Rahayu 2008	Poor quality and post abandonment	Dissatisfaction is often related to the midwife's character, ineffective medicine, minimal experience of the midwife, difficulties in reaching the midwife, and a midwife's absence from the post.	68
Herrara-Almanza 2018	Herrara-Almanza & Rossales-Rueda 2018	Transport and personnel	Poor transportation infrastructure and a shortage of medical personnel limit the basic health access to the population living in remote areas	7

Facilitators of immunisation

Fewer studies discussed facilitators of immunisation as compared to barriers. Much of the information coded came from studies in which the intervention was engagement. To a substantial extent, the sources of this information were key individual interviews and focus group discussions. These were often supported by surveyor or enumerator's observations. In some instances, discussion on facilitators in the primary papers was informed by existing literature.

For peer review only - http://bmjopen.bmj.com/site/about/guidelines.xhtml

A sensitivity analysis, comprising 19 qualitative papers with a risk of bias assessment score of 20 or higher, indicated similar results. Therefore, we view the evidence to be of high strength. Facilitators related to behavioural, social and practical factors faced by caregivers were more common than the facilitators related to the delivery of immunisation services (Supplementary Figure 86). A segregated analysis of the facilitators by the engagement type was not possible due to the limited information coded on this theme.

Behavioural, social and practical barriers faced by caregivers

The most consistent facilitator across all intervention types was the caregiver's *understanding of importance* of immunisation. In general, studies associated caregivers' awareness and perception of the benefits of vaccination to an enabling environment for immunisation uptake (Adamu 2019; Banerjee 2020). In most instances caregivers were able to link benefits of vaccines to the diseases it prevented (Anderson (Cockroft 2009); Pramanik 2020).

Lack of logistics or distance related issues and enabling *social norms* were also consistently sighted as facilitators of immunisation. When it came to logistics, easy access either in the form of proximity to a health care facility (Anderson (Cockroft 2009)) or provision of transportation to an immunisation camp (Olken 2014; Rahayu 2008) were cited as enabling factors. *Social norms* were mentioned as a facilitator in instances where mothers had a significant influence of over decisions regarding her child's health. In addition, peer influence was also mentioned as a factor (Banerjee 2020). Though less consistently cited than the other factors, f*avourable socio-economic characteristics* such as maternal education (Admassie 2009) were also cited as facilitators of immunisation.

Constraints in delivery of immunisation services

Delivery of immunisation services was less consistently reported as a facilitator as compared to behavioural, social and practical factors. The most consistentdelivery related facilitator was the *availability of infrastructure or supplies* which included provision of good quality of services, availability of adequate healthcare providers at the point of care and availability of vaccine supplies.

For peer review only

For peer review only - http://bmjopen.bmj.com/site/about/guidelines.xhtml

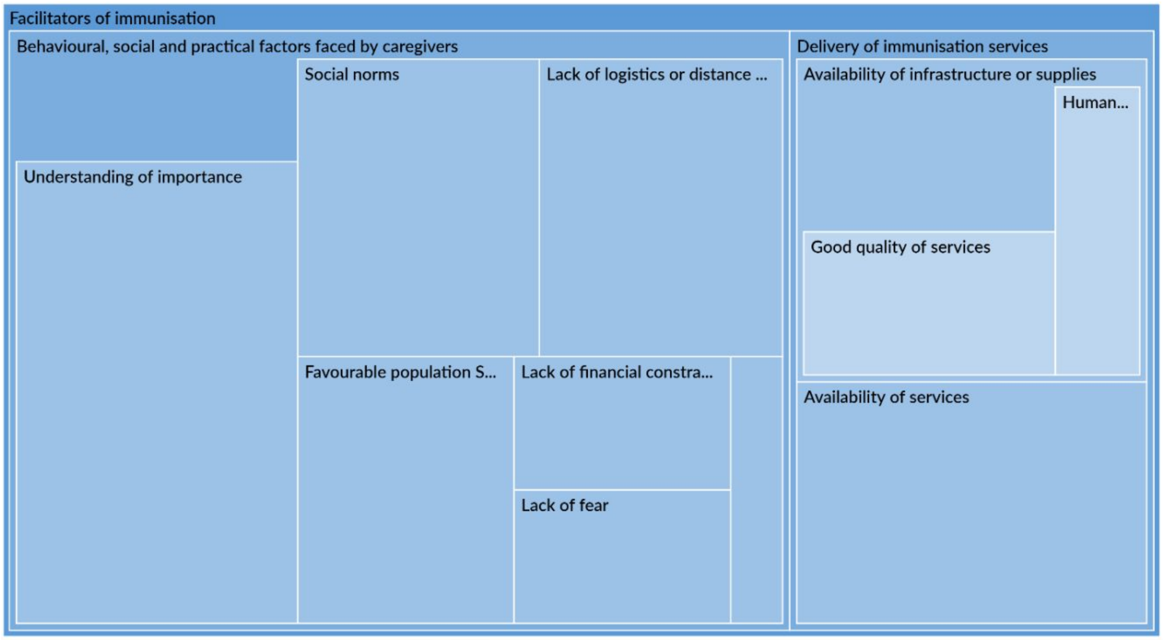

Supplementary Figure 86: Summary figure: Facilitators related to behavioural, social and practical factors faced by caregivers were more common than those related to delivery of immunisation services

The size of the rectangles reflects the number of times a unique theme was mentioned by an IE and its associated papers. Larger rectangles indicate that a theme was mentioned more times. Nested boxes reflect the proportion of times the main theme text also fell into the sub-them.

For peer review only - http://bmjopen.bmj.com/site/about/guidelines.xhtml

Facilitators of immunisation by type of community engagement

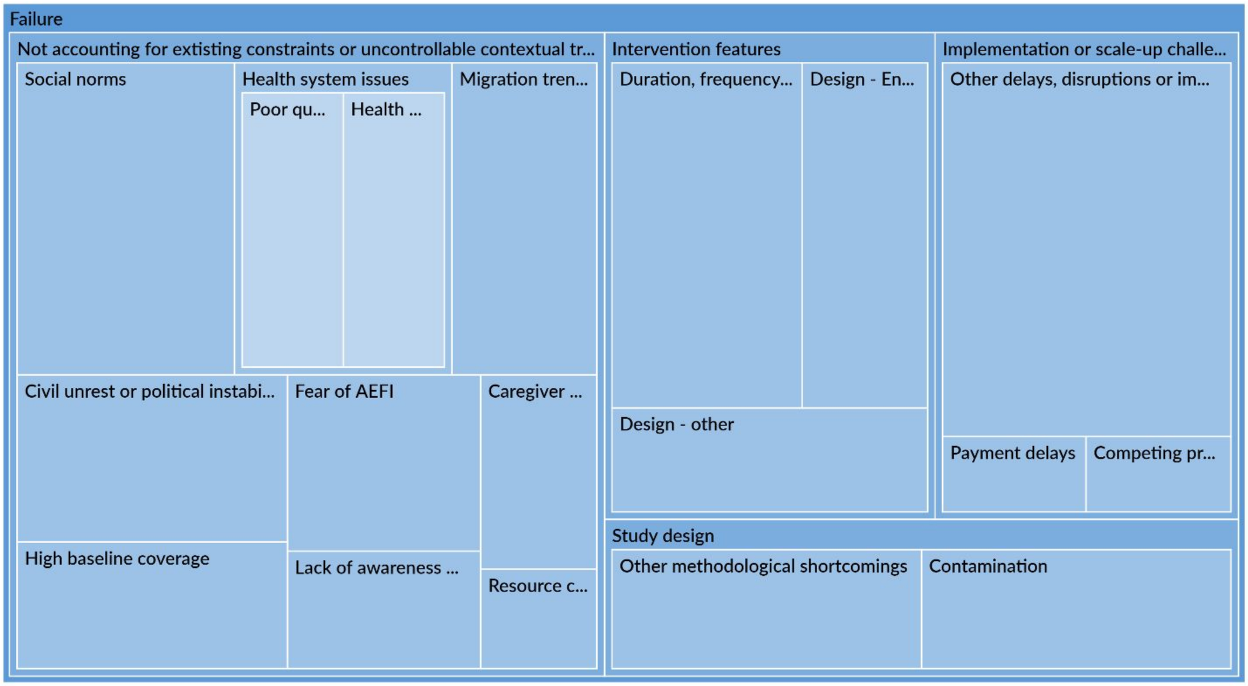

Supplementary Figure 87: Engagement as intervention

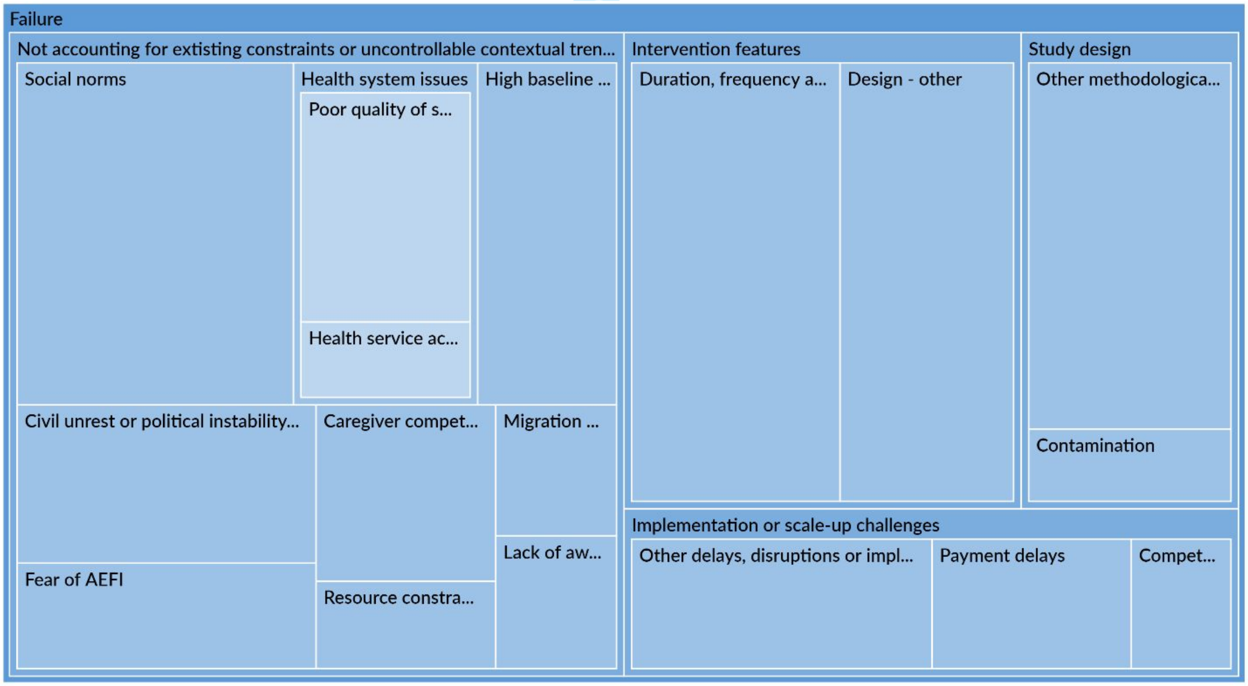

Supplementary Figure 88: Engagement in design

For peer review only - http://bmjopen.bmj.com/site/about/guidelines.xhtml

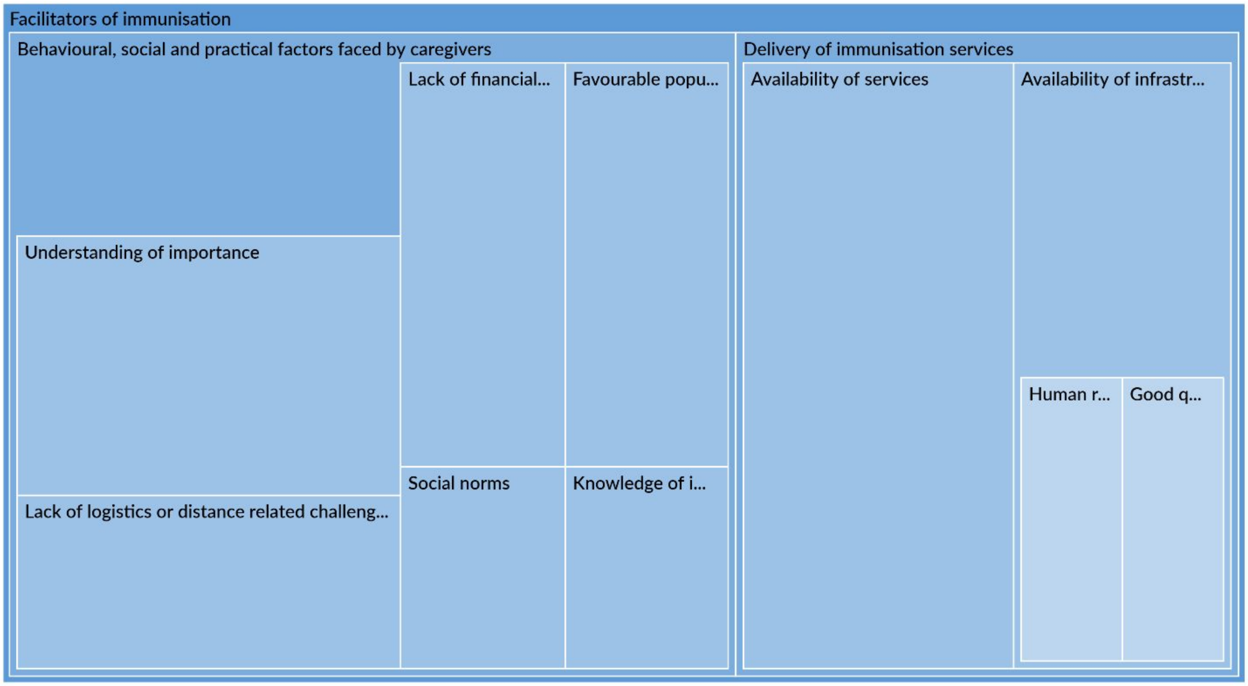

Supplementary Figure 89: Engagement in implementation autonomy

Supplementary Table 8: Characteristic quotes of themes identified related to facilitators of immunisation for community engagement interventions

**Primary paper**	**Paper from which citation comes**	**Theme demonstrated**	**Quote**	**Page number**
Understanding of importance				
Adamu et al. 2019	Adamu 2019 Dissertation	Awareness of benefits	In addition, caregiver beliefs about the capabilities of immunization was overwhelmingly strong: "… this vaccination is very important because it prevents infection from measles, cough, hepatitis, fever, pneumonia and yellow fever.	129
Oyo-Ita et al. 2020	Oyo-Ita et al. 2020	Positive perception of vaccination	It was found from the baseline qualitative study that respondents were generally knowledgeable about and had a positive attitude towards vaccination. They believed vaccines prevented their children from acquiring deadly infections and attributed low numbers of deaths of children to vaccination	31
Socio-economic characteristics				

For peer review only - http://bmjopen.bmj.com/site/about/guidelines.xhtml

Andersson et al. 2009	Cockroft 2009	Importance of maternal education	Mother's education was related to measles vaccination in all four districts, in urban and rural sites.	10
Social norms				
Pramanik 2020	Pramanik 2020	Household decision-making	Key informants further mentioned that in rare cases, it was seen that presence of a knowledgeable daughter in law in the family compelled families to vaccinate their children	85
Availability of infrastructure, supplies or services				
Olken et al. 2014	Rahayu 2008	Availability of medical supplies	The midwife has comprehensive equipment. She has infusion equipment, blood pressure monitor, injections, medicines, scales for infants. The midwife also provides a room in the polindes for mothers who have just given birth. (Women's FGD, Kuanek, East Miomaffo, TTU, NTT)	25
Andersson et al. 2009	Cockroft 2009	Outreach by healthcare workers	in rural sites a vaccination team visiting the community also increased the likelihood that the child had received measles vaccine; this effect was much stronger in rural Khanewal.	5
Adamu et al. 2019	Adamu 2019 Dissertation	Availability of vaccination services	In this study, some caregivers indicated that recommended birth doses of vaccines are provided in the labor room as soon as the child is born. This practice saves time and reduces the chance of missed opportunities in this service delivery point.	134

For peer review only

Reasons for intervention success

Most studies with significant positive quantitative impacts provide some plausible explanation for why an intervention succeeded. These were often based on authors' notes, experiences and impressions of *why* their hypothesis was proven true. We expect the authors to be a good source of this information because of their knowledge of causal linkages and assumptions made in the intervention theory of change. Individual interviews and focus group discussions supported the authors' conclusions. The sensitivity analysis, which included qualitative papers with an assessment of score of 20 or higher, showed similar results. Given this, we consider the evidence to be of high strength.

Overall, reasons for intervention success were consistently attributed to specific *intervention features* across all engagement types. These included community engagement, leadership and supportive supervision, customisation to local context, incentives, health system integration and other design features. Though not as common, success was also attributed to *existing or changing favourable characteristics* within a given context such as availability of and access to good quality health services as well as positive participant views with respect to the intervention (Supplementary Figure 90; Supplementary Table 9).

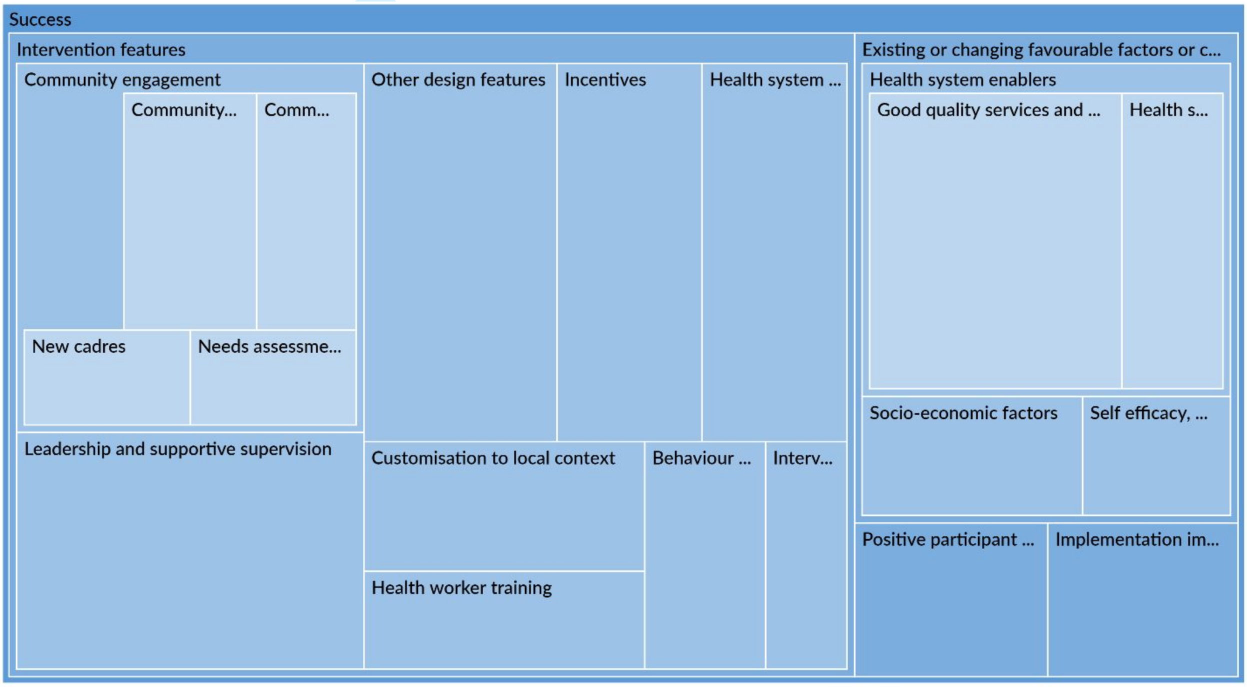

Supplementary Figure 90: Reasons for success: a summary figure

The size of the rectangles reflects the number of times a unique theme was mentioned by an IE and its associated papers. Larger rectangles indicate that a theme was mentioned more times. Nested boxes reflect the proportion of times the main theme text also fell into the sub-theme.

Supplementary Table 9: Comparison of the reasons for intervention success by type of community engagement.

For peer review only - http://bmjopen.bmj.com/site/about/guidelines.xhtml

	**Community engagement as the intervention**	**Community engagement in design**	**Community engagement in implementation autonomy of interventions**
**Intervention features –** *community engagement*	Community dialogues<br>Creation of new cadres	Community dialogues<br>Needs assessments	Community involvement in planning & implementation
**Intervention features –** *Leadership & supportive supervision*	Encouragement from leaders	Supportive supervision	Involvement of leadership<br>Health worker autonomy
**Intervention features –** *Health system integration*	Involvement of government in implementation	Separate workforce dedicated to the intervention	Intersectoral integration of health programmes
**Intervention features –** *Health worker training*	Training on monitoring health cards of children	Training of all health personnel involved in the intervention	Training of all health personnel involved in the intervention
**Favourable contextual factors –** *Health system enablers*	Good quality of services<br>Health service access	Good quality of services	Good quality of services
**Favourable contextual factors –** *Socio-economic factors*	Female literacy	General literacy levels<br>High maternal education	Primary schooling of girls and women
**Improvements in intervention Implementation over time**	-	Health worker performance improvement over time	Resolution of fidelity/adherence issues
**Positive participant/ beneficiary views**	Caregiver perception	Health worker perception	Health worker perception

.

For peer review only - http://bmjopen.bmj.com/site/about/guidelines.xhtml

Community engagement as the intervention

Studies in which engagement was the intervention consistently attributed success to characteristics associated with intervention features and relatively less consistently to existing or changing favourable characteristics within a given context.

Notably, success was attributed to *intervention features* associated with community engagement such as community dialogues which were participatory in nature and improved relationship between the health system and communities (Assegaai 2018 (Padayachee 2013); Findley 2013). Involvement of community members such as traditional and religious leaders in planning and implementation of an intervention also came up as a reason for success (Oyo-Ita 2020). In a few instances, success was also attributed to acceptance of health worker cadres by the communities as long as these health workers belonged to the communities they served (Biemba 2016).

Studies also attributed intervention success to non-engagement *intervention features* including incentives given to caregivers, leadership and supportive supervision which improved overall health service delivery and health worker performance. As noted above, some studies also cited *existing or changing favourable characteristics within a given context* as reasons for success. Among these, those related to the health system, particularly, good service quality (Rao 2016) and, to a lesser extent, access to services (Banwat 2015 (Banwat 2014)) were consistently reported. Interestingly, none of the studies in this engagement category mentioned *implementation improvements* as a reason for intervention success.

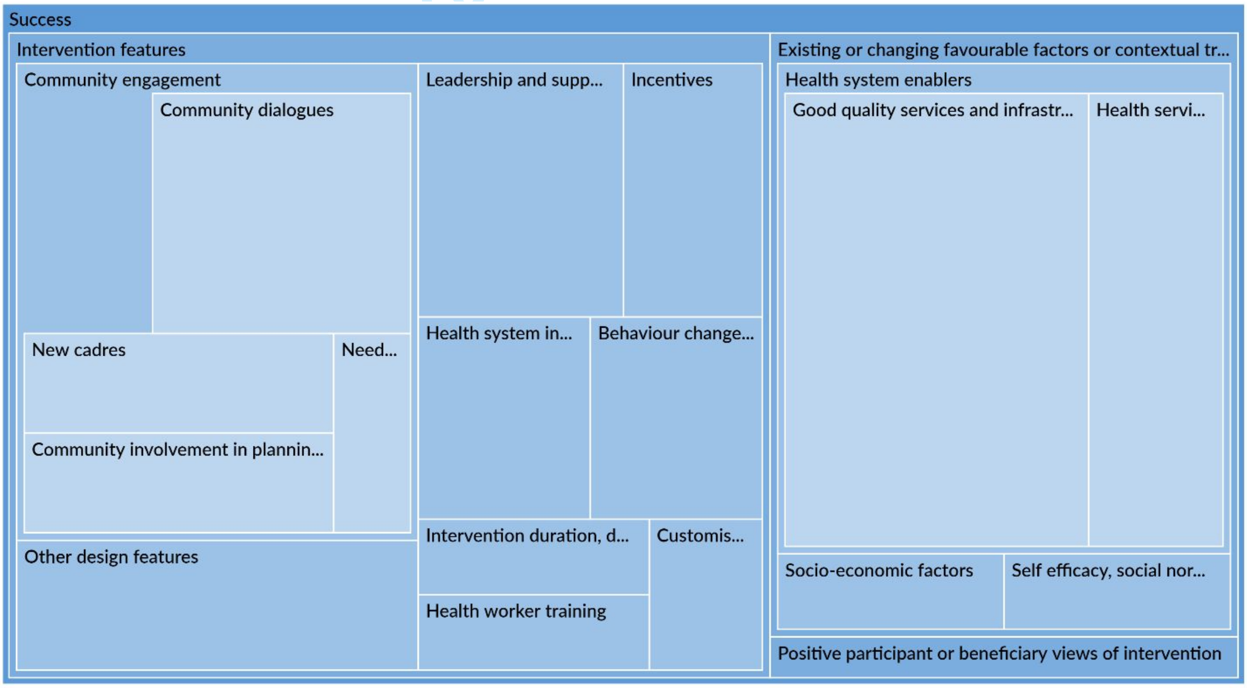

Supplementary Figure 91: Reasons for success of interventions with engagement as intervention

Supplementary Table 10: Characteristic quotes of themes identified related to reasons for success for interventions were community engagement.

Primary paper	Paper from which citation comes	Sub-theme demonstrated	Quote	Page number

Intervention features

Assegaai 2018	Padayachee 2013	*Community dialogue*	Interviewees spoke of the tangible benefits of the community dialogues and proactive household approaches as expanding access, improving relationships with communities, and increasing knowledge and uptake of services.	76
Oyo-Ita 2020	-	Community involvement in planning and implementation	Inclusion of TRLs in the planning, implementation and evaluation of an intervention is useful in ensuring support from the community.	42
Biemba 2016	Phiri 2017	Leadership and supportive supervision	CHAs reported that supervision by experienced health workers was valuable to them because it reinforced skills they learned in training and provided general encouragement. This study finds that the quality and frequency of supervision is heavily influenced by the proximity of supervisors' work station to that of the CHAs.	5
Banerjee 2020	-	Incentives	Most primary caregivers expressed positive opinions about recharges. Some admitted to finding them motivating or influencing their decision to immunise their child (whether or not they actually received them). This was linked to generally liking free things ('What's not to like?'), their self-assessed poverty level, or their trust in the government 'investing in them'.	53

For peer review only - http://bmjopen.bmj.com/site/about/guidelines.xhtml

Existing or changing favourable factors or contextual trends				
Rao et al. 2014	Rao et al. 2016	Good quality of services and infrastructure	Moreover, the ASHA worker is envisioned not to work in isolation but as an integral part of the public health system, working together with other health personnel to both ease their work-pressures and assist them in performing their duties more efficiently. For instance, the ASHA worker works with the ANM and the anganwadi worker to organize monthly village health days. Therefore, the extent to which the increase in information provision via other health workers is attributable to the ASHA worker is not quantifiable in this context.	25
Findley et al. 2013	-	Good quality of services and infrastructure	simultaneous improvement in the quality of care provided by the CHW and nurse-midwives at the health post gave women the confidence that they could go to the health post to seek advice and care.	10
Banwat 2015	Banwat 2014-	Health service access and availability	A child was also found to be most likely to be fully and timely immunized if he/she lived within 30 minutes walking distance from the health facility.	66

Community engagement in design

The most consistentreasons for success were related to *intervention features* with the most reported characteristics being community engagement and provision of incentives to caregivers. Within community engagement, participatory community dialogues (Andersson 2009), stakeholder consultations on intervention design (Modi 2019) and community involvement in planning and implementation (Adamu 2019) were associated with intervention success. Receiving incentives was perceived to be motivating and empowering by the intended beneficiaries (Banerjee 2020; Nagar 2020).

For peer review only - http://bmjopen.bmj.com/site/about/guidelines.xhtml

Besides community engagement and incentives, o*ther intervention features* that were consistently reported included leadership and supportive supervision, health worker training and customisation to local context.

Very few studies discussed or attributed success to reasons other than intervention features. Nevertheless, the few studies that did, referred to *existing or changing favourable contextual factors* such as a high prevalence of maternal education (Johri 2020) or *improved implementation* by resolution of issues related to adherence (Modi 2019). A few studies also discussed *positive participant or beneficiary views* that could have potentially contributed to intervention success.

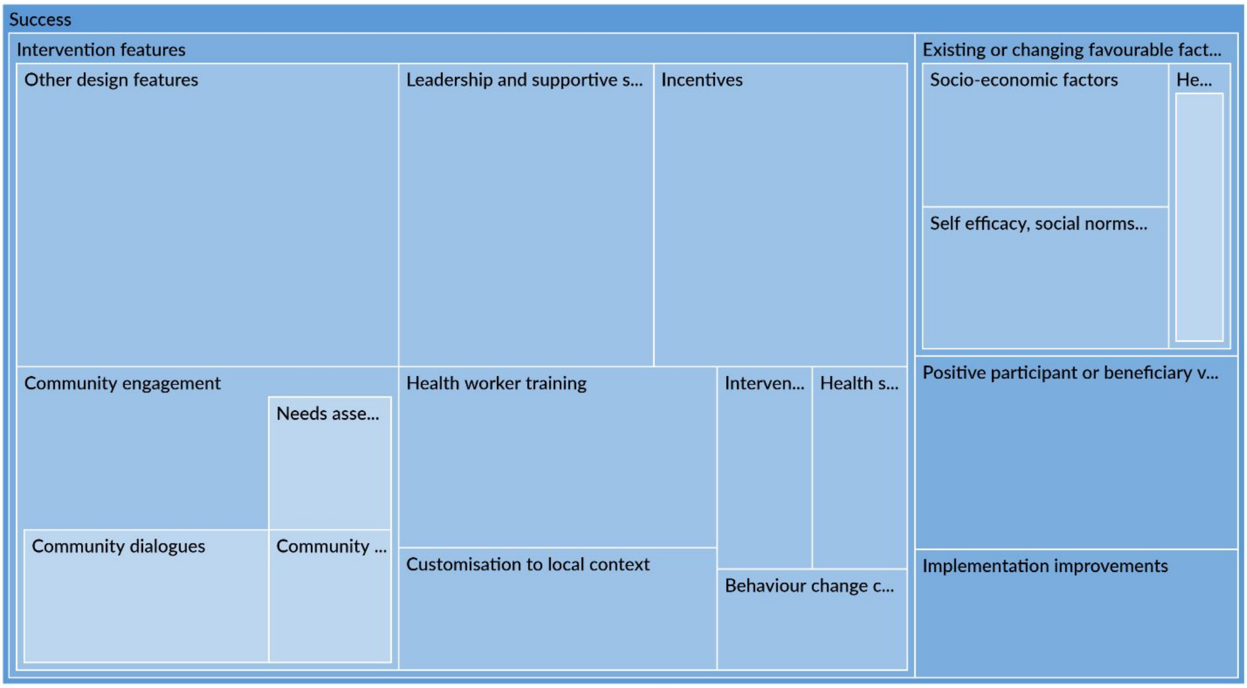

Supplementray Figure 92: Reasons for success of interventions with engagement in design

Supplementary Table 11: Characteristic quotes of themes identified related to reasons for success for interventions that used community engagement in their design.

**Primary paper**	**Paper from which citation comes**	**Sub-theme demonstrated**	**Quote**	**Page number**
Intervention features				
Andersson 2009	-	Community dialogue	The structured discussion rounds sometimes led to action plans in the intervention communities beyond stimulating discussion about vaccinations within households. Particularly in those villages with poor access to vaccination services, plans included sharing transport to vaccination points and providing care for some children while parents took others to be vaccinated. These community initiatives may have helped to maintain vaccination levels in the face of generally falling levels.	8

For peer review only - http://bmjopen.bmj.com/site/about/guidelines.xhtml

Adamu 2019	Adamu 2019 CFIR	Leadership and supportive supervision	Participants expressed satisfaction with the supervisory plan that was put in place for the quality improvement program as it enabled quick feedback. Supervisory visits were conducted by different stakeholders that are higher-ranking officials within the health systems. These include local government and zonal primary health-care management board officials. "We receive supervision from local government, they use to come and supervised us to check how we conduct our duties."– PHC 3	468
Assegaai 2018	Khuzwayo 2017	Referral to caregivers	Referrals to clinics by the teams were regarded as an important aspect of bringing services closer to the communities. Respondents said that they would not have gone to the clinic had they not been referred by the WBOT, for example for deworming of children, or continuation of care for complicated cases.	3
Existing or changing favourable factors or contextual trends				
Banerjee 2020	-	Self efficacy, social norms and awareness	By and large the main, explicitly stated driver for primary caregivers to get their child vaccinated is their positive perception or attitude toward immunization: that immunization will benefit their child. This acts as a push even for primary caregivers who have limited knowledge, who don't understand exactly how or why immunization is beneficial. Some of these primary caregivers hold strong personal conviction of the importance of immunization, despite their lack of knowledge, while others are undecided or unsure about the importance of immunization, but are still overall positive. In both cases, it seems that the positive perception or attitude is influenced by others: what others say and do. This is often mediated by trust: either trust in government and authority (e.g. ANM), household or family members, other primary caregivers in the community, or people in positions of power in the community (e.g. religious leaders in Mewat). Interestingly, improved knowledge and awareness was stated by a number of respondents as a key factor to convince primary caregivers (themselves, or others) to attend immunization camps. This demonstrates, as was mentioned in response to ANM performance, that	Appendix K – qualitative findings

For peer review only - http://bmjopen.bmj.com/site/about/guidelines.xhtml

			primary caregivers value information about vaccines	
Gibson et al. 2017	-	Socio-economic factors	The success of SMS reminders to elicit a behaviour is multifactorial; the content of the message, the type of behaviour being reminded, indirect and direct costs incurred, literacy level, and other contextual factors all being potential explanatory factors.	e436
Implementation improvements				
Modi 2019	-	Operational improvements	Finally, we identified critical operational requirements that improved adherence to the intervention; this included supportive supervision, timely resolution of technology problems, and change management, including monetary and/ or nonmonetary incentives depending on the context.	20

Community engagement in implementation autonomy of intervention

Similar to the previous two engagement categories, reasons for success in interventions with engagement in implementation were also consistently attributed to certain *intervention features*. These characteristics were associated with certain aspects of *community engagement* itself such as conducting stakeholder consultations (Modi 2019) or involvement of community members such as health workers in intervention planning and implementation (Adamu 2019). Other aspects of intervention features such as *integration with the health system, customisation to the local context* and *leadership and supportive supervision* were also consistently cited.

Like the engagement in design category, crediting intervention success to *existing or changing favourable contextual factors* or *improved implementation* was less consistently reported among studies in which community engagement was involved in the implementation of the intervention. Nevertheless, *health system enablers* such as good quality of services and access to health services were mentioned as factors which could have contributed to intervention success.

For peer review only

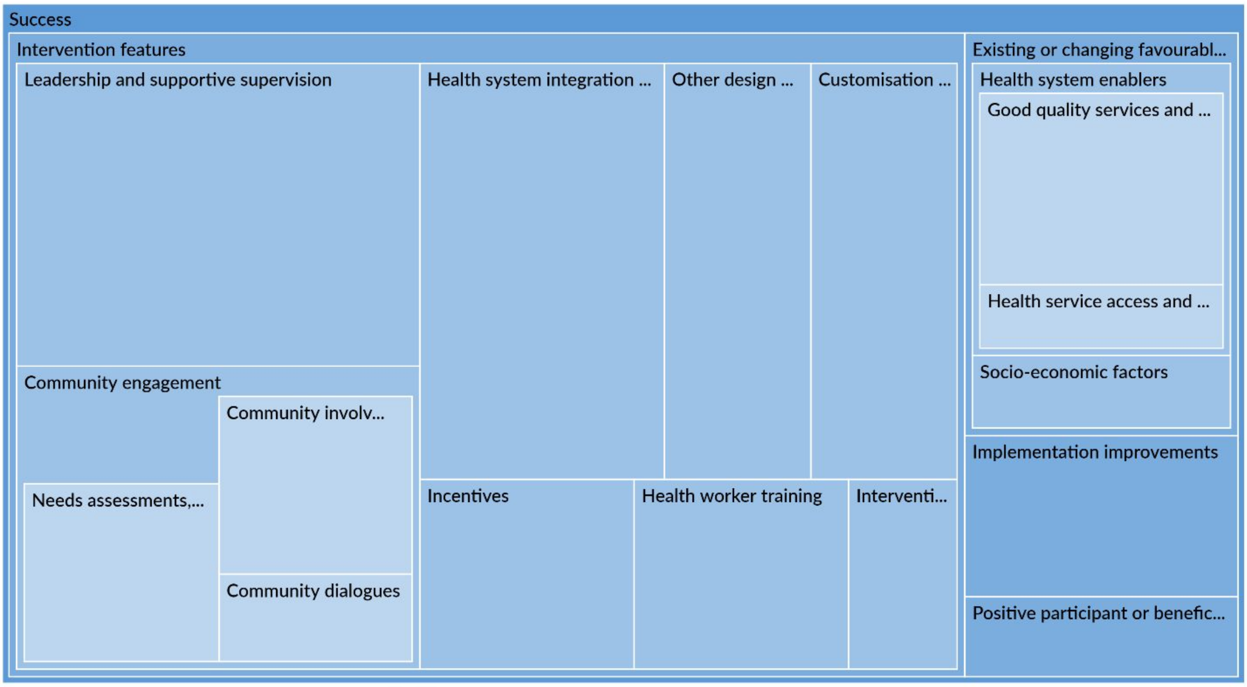

Supplementary Figure 93: Reasons for success of interventions with engagement in implementation autonomy

Supplementary Table 12: Characteristic quotes of themes identified related to reasons for success for interventions that used community engagement in implementation.

Primary paper	Paper from which citation comes	Sub-theme demonstrated	Quote	Page number
Intervention features				
Adamu 2019	-	Community involvement in planning and implementation	Stakeholders were systematically involved in the planning and execution of the QI program. The change ideas were selected by frontline health workers and they tailored them to their local context. Also, change ideas are multi-faceted and multimodal, and this is suitable for addressing complex problems in complex health systems.	9
Sankar 2013	-	Needs assessments, pilots or stakeholder consultations	The project interventions were developed in keeping with the especial needs of the population, and rooted in the conditions and circumstances of the area. It drew heavily from the understanding and opinions of local people. The results reflect the fruits of a highly contextualised program.	40

For peer review only - http://bmjopen.bmj.com/site/about/guidelines.xhtml

Shukla 2018	Anwari 2015	Leadership and supportive supervision	District Health Offices and DHCCs were less well established compared to the Provincial Public Health Directorates and PPHCCs; the ministry did not have adequate resources to equip them well. Despite these challenges, the leadership and involvement of the ministry in the intervention mattered. The provincial and district health governance leaders were inspired to improve their governance because the ministry leaders were interested in the pilot intervention.	14
Existing or changing favourable factors or contextual trends				
Admassie 2009	-	Good quality services and infrastructure	The impact of the programme on the proportion of children who got vaccines against<br><br>major childhood illnesses also varies according to supply side variables (see Table 6). The programme has larger effects in villages with one HEW and with better quality health posts.	441
Herrera-Almanza and Rosales-Rueda 2018	-	Health service access and availability	However, the fact that we find results on vaccination uptake is an indication that the health workers were somehow present in remote areas.	29
Admassie 2009	-	Socio-economic factors	This implies that encouraging and supporting primary schooling for girls and women will enhance the programme's impact on proportion of children vaccinated against major childhood illnesses.	441
Implementation improvements				
Mayumana 2017	Binyaruka 2017	Managing vaccine stockouts	The verification system under P4P also meant that district supervision was intensified, providing more opportunities for district managers to identify and address stock-outs of a wider range of drugs.	97

For peer review only - http://bmjopen.bmj.com/site/about/guidelines.xhtml

Reasons for intervention failure

Most studies that did not find a significant positive impact attributed the failure of the intervention to a specific cause. The most consistently reported reason for failure across all engagement types was *not accounting for contextual constraints,* though the exact nature of these reasons varied. Social norms such as misconception regarding immunisation and health worker attrition were reported as common challenges in interventions that were community engagement. Interventions that used engagement in design consistently reported poor access to health services and caregivers' skepticism regarding benefits of immunisation as the reasons for failure. Poor quality of health services was noted as an issue in interventions where engagement occurred in implementation.

The other consistently reported reasons for failure were attributed to inadequate *intervention features* and *implementation challenges.* Among the intervention features, inadequate duration, frequency or exposure to the intervention were the most notable reasons for failure across all engagement types. Though relatively less common, the nature of the community engagement itself was also attributed to failure in interventions that used engagement in implementation or as intervention. Among implementation challenges, disruption due to inadequate implementation instructions, difficulty in accessing the implementation sites due to geographic proximity and competing priorities of health workers were noted as some of the prevalent challenges (Supplementary Figure 94; Supplementary Table 13).

Another less consistently reported reason for failure was study design issues such as contamination between treatment and control other methodological challenges such as biased sampling frame and issues with enrolling study populations. The reasons for failure were largely based on authors' notes or experiences and individual interviews. We expect the authors to be a good source of this information because of their knowledge of causal linkages and assumptions made in the intervention theory of change. Focus group discussions and implementers' experiences supported these findings. The sensitivity analysis, which included qualitative papers with an assessment of score of 20 or higher, showed similar results. Except for one sub-theme which was dominated by one or two studies, other sub-themes were consistently distributed across the evidence base (indicated in Supplementary Table 13). Therefore, we consider the evidence to be of high strength.

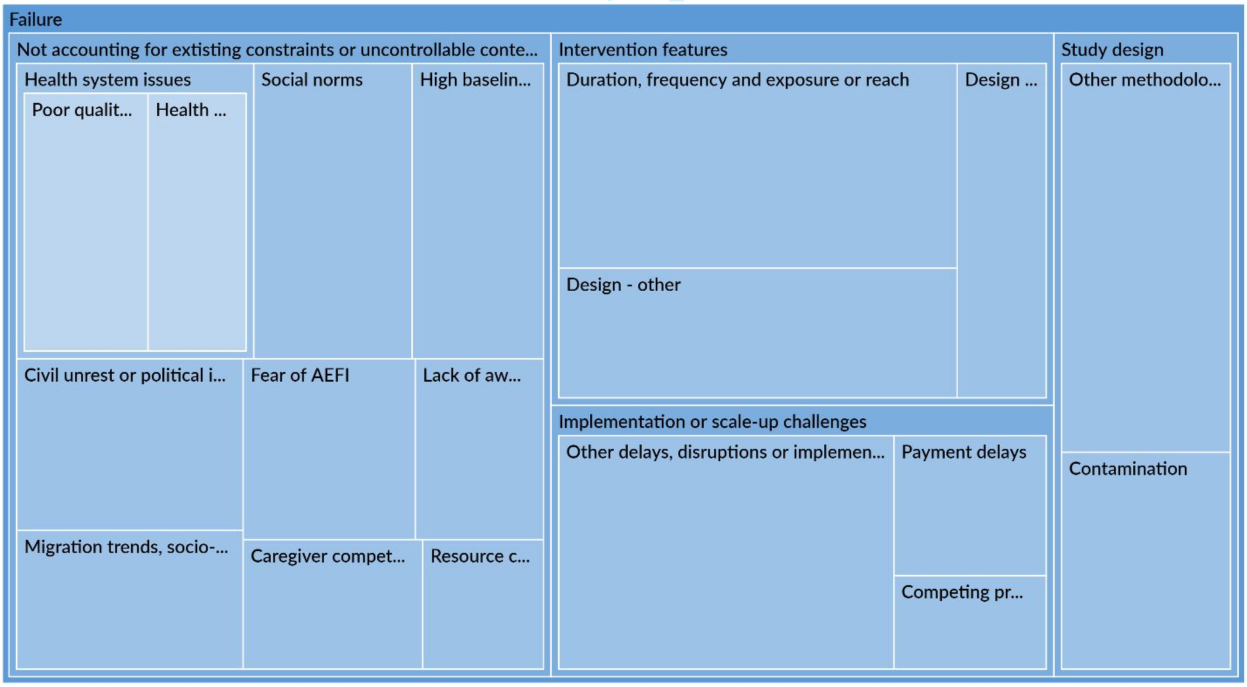

Supplementary Figure 94: Summary figure: reasons for failure

Supplementary Table 13: Comparison of the reasons for intervention failure by type of community engagement**.**

For peer review only - http://bmjopen.bmj.com/site/about/guidelines.xhtml

	**Community engagement as the intervention**	**Community engagement in design**	**Community engagement in implementation autonomy of the intervention**
**Not accounting for contextual constraints –** *health system issues*	Health worker attrition	Issues with accessing health services	Poor quality of services
**Not accounting for contextual constraints –** *prevalent social norms*	Fear of sterilization	Skepticism towards perceived value of immunisation	-
**Not accounting for contextual constraints –** *baseline immunisation coverage*	High baseline coverage	High baseline coverage	High BCG coverage
**Not accounting for contextual constraints –** *unrest, insecurity or other*	Civil unrest<br>Natural calamity	Political instability	-
**Intervention features –** *Duration, dose or frequency of intervention*	Inadequate intervention duration or exposure	Inadequate intervention frequency	Inadequate intervention duration or exposure
**Intervention feature –** *issue in design of engagement*	Lack of collective action	-	Elite capture
**Implementation challenges** *– disruptions or delays*	Resource constraint due to multiple programmes running at the same time	Fidelity issues	Staff turnover*
**Study design –**	Contamination due to similar programmes	Contamination due to similar programmes	Contamination between treatment and control

For peer review only

For peer review only - http://bmjopen.bmj.com/site/about/guidelines.xhtml

Contamination			

*Most of the references coded under this theme were from Olken 2014 and associated papers.

Community engagement as the intervention

Not accounting for contextual trends were the most consistently reported reason for failure. Social norms, health system issues, migration, civil unrest and fear of adverse events following immunisation were reported consistently within existing constraints. Most of the challenges related to social norms mentioned misconception about immunisation (Banerjee 2020) and limited decision-making powers of mothers (Gurley 2020) as the reason for low immunisation uptake. Civil unrest, political instability and natural calamities also hindered a few interventions from achieving intended impacts (Pramanik 2020; Morris 2004).

The other reasons for failure comprised issues related to *intervention features, implementation issues* and, to a lesser extent, *study design.* Issues with intervention feature ranged from inadequate duration or frequency of the intervention to certain aspects of community engagement failing to elicit a positive response among study participants (Pramanik 2020; More 2012). Implementation issues were consistently reported as reasons for failure. Some of the challenges mentioned were competing demands by different programmes running simultaneously (Carnell 2014), high staff turnover (Gurley 2020; Olken 2014) and low implementation fidelity (Oyo-Ita 2020). These issues are also discussed in detail under the ‘uptake and fidelity challenges theme’.

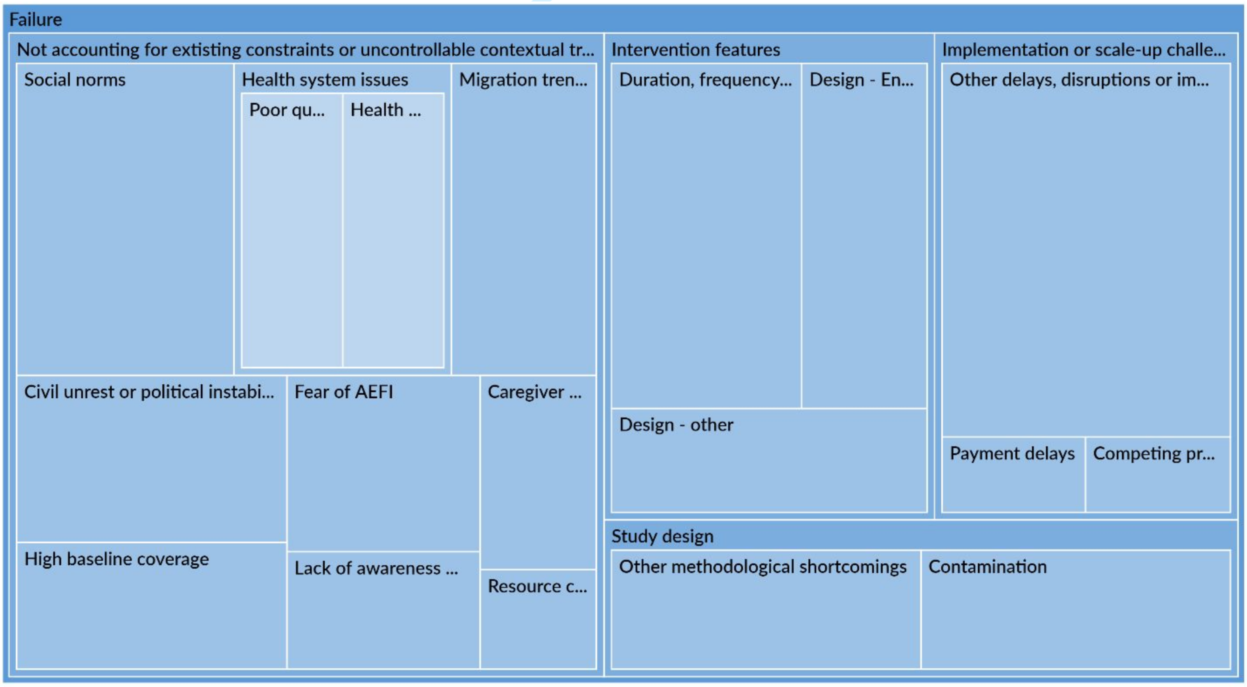

Supplementary Figure 95: Reasons for failure of interventions with engagement as intervention

Supplementray Table 14: Characteristic quotes of themes identified related to reasons for failure for interventions were community engagement.

For peer review only - http://bmjopen.bmj.com/site/about/guidelines.xhtml

Primary paper	Paper from which citation comes	Sub-theme demonstrated	Quote	Page number
Implementation or scale-up challenges				
Carnell 2014	-	Attrition of implementation personnel	The ESHE and RHB teams both faced personnel challenges in Amhara, which may have affected management and performance.	559
Gurley 2020	-	Infrastructure constraints	Community constraints around screening spaces and economic activities also introduced challenges.	73
Not accounting for existing constraints or uncontrollable trends				
More 2012	-	Lack of access to health care	The third issue was the complexity of urban health care. Antenatal care was the norm and the nadir for institutional delivery in trial clusters was 75%. Around 57% of antenatal care and 30% of deliveries were in the private sector (this in a slumdwelling population). Open access to private providers, and to institutions at all levels of the public sector hierarchy, is a challenge to systematic health care delivery. Our findings confirmed the tendency to bypass public maternity homes, which should handle uncomplicated deliveries, in favour of tertiary institutions.	E1001257
Pramanik 2020	-	Natural calamity	Assam has a wonderful collection of festivals that are of the utmost importance. And more tragically when the Brahmaputra River floods the priority is survival. The project simply has to adapt to these ebbs and flows, but there is a pressure to deliver to a schedule that inevitably leads to a box ticking approach that is inconsistent with the development of ownership by the community.	68

For peer review only - http://bmjopen.bmj.com/site/about/guidelines.xhtml

Oyo-Ita 2020	-	Fear of AEFI	These responses are indications that fear of side effects can hinder vaccine uptake. This may have contributed to non-impact on the proportion of fully vaccinated children in this study, as the TRLs in the post-intervention qualitative study still mentioned this as a common reason for poor uptake of vaccines.	32
Intervention features				
Olken 2014	Grayman 2013	Engagement failure due to elite capture	In general, researchers discovered that Generasi provides opportunities for elites to retain and fortify their social standing. As brokers delivering CDD resources into their communities, local elites leverage Generasi to accumulate additional status for themselves. Without exception, every Generasi actor that the researchers met held other leadership roles in their community and maintained close ties with the local structures of village and sub-district governance.	48
Robertson 2013	-	Inadequate exposure to the intervention	Our study was limited by the short intervention period. Whether the effects of the programmes would change with time is unclear. The follow-up survey was done 2 months after interventions had finished, so fear of penalties should not have biased responses from CCT households, although the effects of the programmes could have attenuated by the time of the survey.	8
Study design				
More 2017	-	Contamination due to other programmes	although we found no evidence of contamination of control clusters by the intervention, government schemes and the activities of municipal and non-governmental providers might have improved health in control clusters.	E347

Community engagement in design

Most consistently reported reasons for failure of interventions in which communities were engaged in the design broadly fell under *not accounting for contextual constraints.* Among these, social norms were the most consistently reported reason with vaccine hesitancy being one of the driving forces behind immunisation refusal

For peer review only - http://bmjopen.bmj.com/site/about/guidelines.xhtml

(Banerjee 2020; Gurley 2020). Other reasons for failure within this theme were high baseline immunisation coverage, health system issues related to quality of services, and civil unrest or political instability. Prevailing socio-economic trends, lack of awareness, fear of AEFI and caregiver competing priorities were also attributed to intervention failure, though less consistently reported as compared to other factors.

Besides existing constraints, a few studies also attributed failure *intervention features* with the insufficient duration, frequency and exposure of intervention as a possible reason for failure. A much smaller subset of studies also referred to issues related to study design and implementation or scale-up challenges.

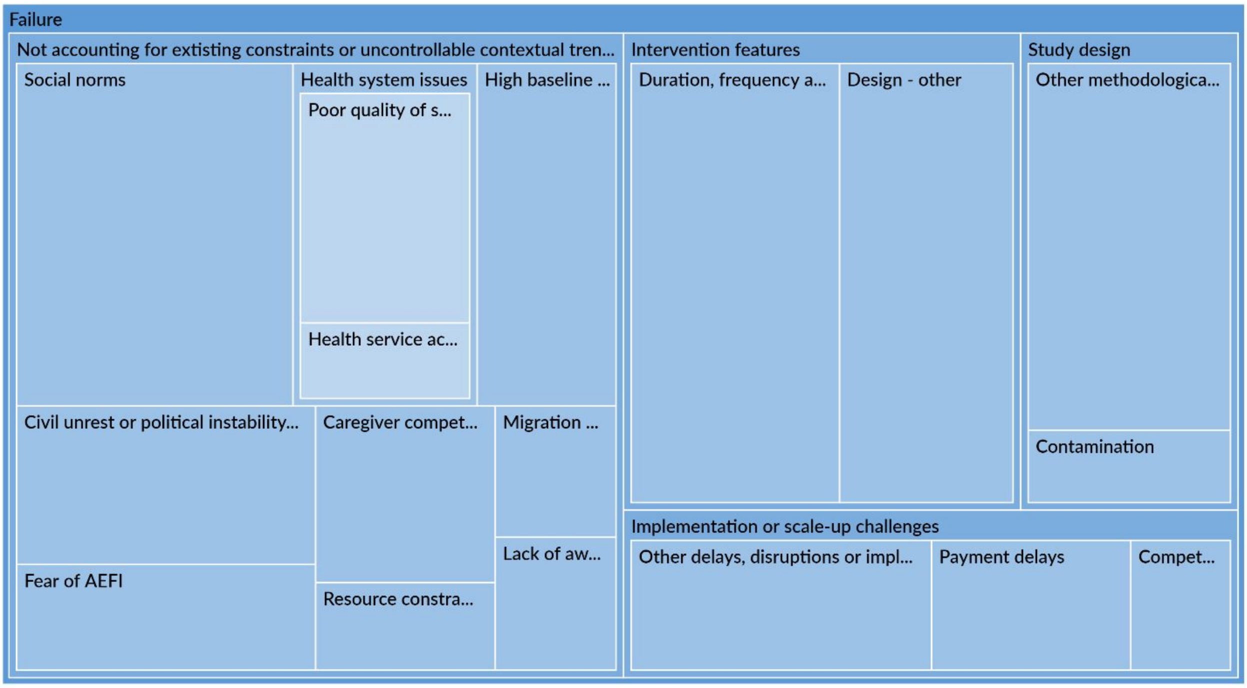

Supplementary Figure 96: Reasons for failure of interventions with engagement in design

Supplementary Table 15: Characteristic quotes of themes identified related to reasons for failure for interventions that used community engagement in their design.

**Primary paper**	**Paper from which citation comes**	**Sub-theme demonstrated**	**Quote**	**Page number**
Implementation or scale-up challenges				
Gurley 2020	-	Staff turnover	On the implementer side, staff turnover at NYST and research activities introduced delays in video production.	73
Engineer 2016	-	Payment delays	Problems with implementation likely dampened any potential effect. The scheme was rolled out in phases, but there were some delays, particularly with the initial payments.	456

Not accounting for existing constraints or uncontrollable trends

Gibson 2017	-	High baseline immunisation coverage	SMS reminders were probably not effective at improving full immunisation and vaccine-specific coverages in this study because of high baseline coverage levels and because SMS reminders might not have addressed the demand side deficiencies in this study area.	e436
Banerjee 2020	-	Caregiver competing priorities	Across districts, there are 'hard-to-convince' populations who are not affected by incentives. Some ANMs listed specific populations groups they felt continued to be unaffected by the programme, and the incentives specifically, such as migrant workers, daily wage workers and the Muslim community. In Panipat, the top reason given by ANMs for resistance to immunisation by the few still unconvinced (migrants, Muslims) was the fever brought on as an after-effect by a certain vaccine (penta)30 and direct costs involved for daily wage workers.	92
Gurley 2020	-	Social norms	It is worth noting that in contrast to the high intent recorded through the household survey, the process evaluation indicated there were individuals who actively chose to not vaccinate their children. This may be a function of social desirability bias in the household survey, though we posit that it may also reflect family constraints or logistical barriers that prevent a mother from taking a vaccine decision regardless of own personal intention to vaccinate. As noted elsewhere, mothers are not the sole or primary decision-makers and other family members reported lower intent to vaccinate.	75

Intervention features

Siddiqi 2020	-	Inadequate exposure to the intervention	Our findings also provide evidence of the strong reliance on the immunization card being the established immunization recall method and we may also postulate that the short duration of the study did not provide enough time to 'institutionalize' the use of the bracelets.	12

For peer review only - http://bmjopen.bmj.com/site/about/guidelines.xhtml

Okoli 2014	Baba-Ari 2018	Communication failure	Experiences of the beneficiaries with the programme point to problems with programme communication as they had a poor understanding of the aims of the CCT programme aside from receiving its immediate cash benefits and as such might revert to their original behaviours after the programme ends.	940
Study design				
Domek 2019	-	Selection bias in study enrollment	This may have been partly because our study population had higher baseline immunization coverage than we were adequately powered to assess. These higher than expected completion rates were likely due in part to a selection bias in enrolling children as they presented for their first immunization visit, which would have unintentionally excluded children who either presented significantly delayed or not at all for vaccines.	6197

Community engagement in implementation autonomy of interventions

Fewer studies under this engagement classification reported reasons for failure as compared to success. Among the reasons cited for failure, those related to *not accounting for contextual constraints* were the most consistently reported followed by *implementation challenges*. Most of the references to implementation challenges or failures were made by Olken 2014 and its associated studies which discussed multiple implementation barriers to a block-level grant intervention. Though less consistently reported, some studies also mentioned issues with intervention features or study design as the reasons for intervention failure. Highlighting the failure of community engagement, Olken 2014 (Grayman 2013) discussed how elite capture wherein local community leaderships and elites leveraged the intervention to retain social standing became an issue for the programme.

For peer review only - http://bmjopen.bmj.com/site/about/guidelines.xhtml

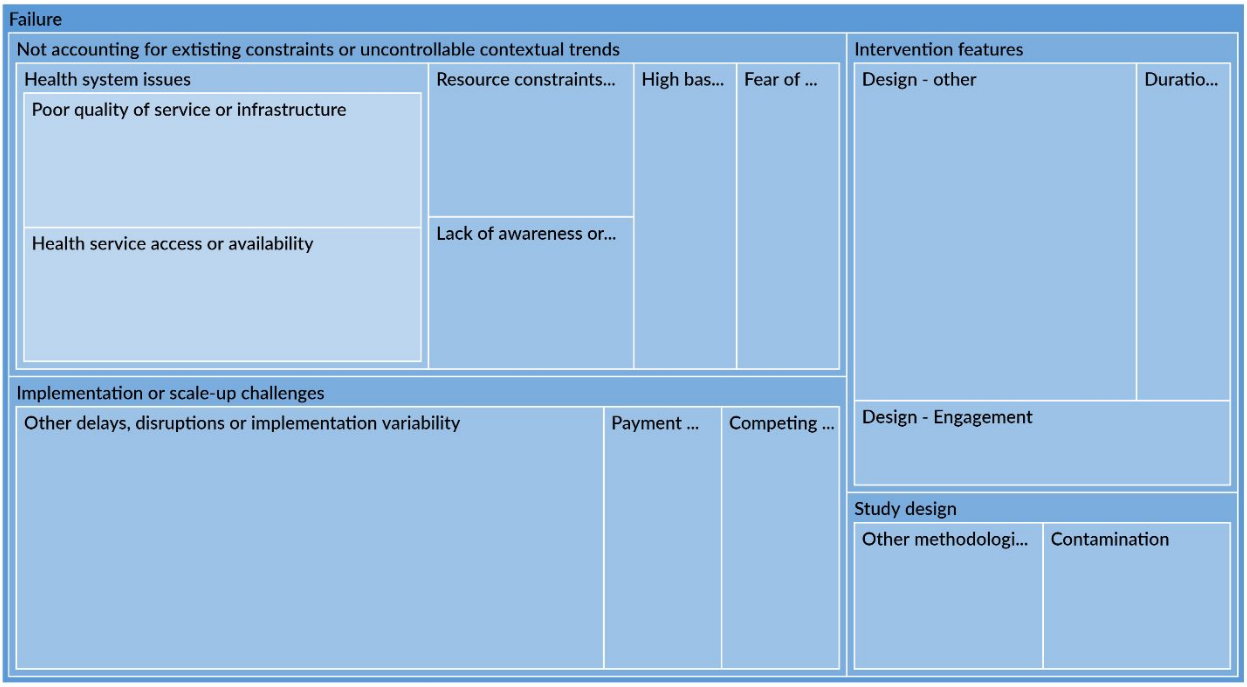

Supplementary Figure 97: Reasons for failure of interventions with engagement in implementation autonomy

Supplementary Table 16: Characteristic quotes of themes identified related to reasons for failure for interventions that used community engagement in implementation.

**Primary paper**	**Paper from which citation comes**	**Sub-theme demonstrated**	**Quote**	**Page number**
Implementation or scale-up challenges				
Oyo-Ita 2020	-	Low implementation fidelity	The non-impact on up-to-date vaccination could have been accounted for by the weak link in the intervention caused by not sharing data directly with community members as planned.	38
Olken 2014	World Bank 2018	Staff turnover	High village-level staff turnover may partially explain why Generasi staff in the villages are so unfamiliar with the bonus system. The entire Generasi team in Desa Lelaok (Petis subdistrict, Pamekasan district) and the PK in Desa Rampe (Nelle subdistrict, Lembata) had never heard of the bonus system, but all of them had started within the past year.	38

For peer review only - http://bmjopen.bmj.com/site/about/guidelines.xhtml

Webster 2019	-	Competing priorities of health workers	Although the proportion of respondents reporting a home visit by a VHT decreased from baseline to endline in both control and intervention clusters the proportion of visits where immunization was the reason for the visit increased. The decrease in VHT home visits may be due in part to observations that in the latter months of the intervention, VHT services were sought by other organisations involved in the refugee response in some of the project areas, who paid VHTs higher allowances.	47
Not accounting for existing constraints or uncontrollable trends				
Okeke 2017	-	Resource constraints or scarcity	Lack of electricity and water were also frequently cited as problems.	43
Okeke 2017	-	Poor quality of services or infrastructure	The data suggest that part of the reason why the program did not have larger impacts is that other dimensions of quality did not improve. For example, clinic infrastructure in many cases remained poor, as did availability of drugs and supplies.	49
Intervention features				
Webster 2019	-	Intervention design failure	The main mechanisms of change identified were improved accessibility to immunization services through increased numbers of vaccination outreaches, increased VHT motivation through monthly allowances together with social motivation in outdoing each other in defaulter tracing, use of community resources to achieve outcomes that is the support of local community leadership; and increased interaction between health workers and HCWs and VHTs at monthly VHT/HCW meetings. These identified mechanisms of change were predominantly due to supplementary activities that were implemented in both intervention and control clusters.	49
Study design				

For peer review only - http://bmjopen.bmj.com/site/about/guidelines.xhtml

Webster 2019	-	Contamination between treatment and control	There was some contamination of control clusters, largely due to transfer of health workers from intervention to control sites. At two control sites it was evident that the health workers sought to set up a similar defaulter tracing approach.	49

Reasons for heterogenous impacts

A small subset of studies is also coded under the theme *heterogeneous impacts*. Even though they reported overall significant positive quantitative findings, these studies discussed their heterogenous or non-uniform results with respect to not achieving the intended impact among certain subgroups, study sites or reported null results for some of the immunisation-related outcomes. Each of these studies offer a slightly different perspective on their heterogeneous results. For instance, Adamu et al. 2019 and Carnell et al. 2014 postulate that the characteristics of individuals involved in the intervention implementation, contextual factors or implementation processes could have led to partial intervention success or measurable positive gain in only select study sites. Interestingly, both Banerjee et al. 2020 and Gibson et al. 2017 highlight very different challenges with respect to their interventions comprising incentives to caregivers. Banerjee et al. describe how incentives designed to motivate caregivers did not work on Muslim minorities due to high vaccine hesitancy which stemmed from the belief that vaccination causes sterlisation. On the other hand, the Gibson study reported that their SMS reminders did not work in the absence of incentives, implying that reminders alone were insufficient to encourage behaviour change among caregivers and result in immunisation uptake. This shows that the benefit of providing incentives may not be consistent across different contexts and uptake may be influenced by other socio-economic factors. Both studies further elaborate the limits to which incentives can address deep rooted mistrust of vaccination among certain subgroups. Overall, the discussion on these studies provide important insights about how external factors, implementation variability or invalid theory of change assumptions may contribute to partial intervention success or failure. We also looked through the literature for *unintended impacts* but there was insufficient information to draw any broader conclusions.

Uptake and fidelity challenges

Uptake and fidelity challenges were generally identified by authors through primary data or personal experience and knowledge of the intervention. Because this theme relates to how the project was implemented, we expect that authors are a good source of this information. Individual interviews and focus group discussions supported the findings. Themes were broadly consistent in the full analysis and in sensitivity analysis in which on the qualitative papers with a quality assessment score greater than 20 were included. However, there are several instances in which a single sub-theme is dominated by one or two studies. These are indicated in Supplementary Table 17 and discussed in the text. Given this, we believe that the evidence on uptake and fidelity challenges has only moderate strength.

The most consistently reported challenges were related to administration, mobilization, fidelity, and contamination (Supplementary Figure 98; Supplementray Table 17). Administrative challenges were cited consistently across all three types of interventions, but their nature varied. Although technical issues and politics plagued interventions that used community engagement in the design of interventions or as the intervention, these issues were not common in interventions that used engagement in their implementation. Staffing was a challenge in interventions that used engagement in implementation or as the intervention. Implementation fidelity was mentioned as a challenge by all three types of interventions. In all cases, the most consistent issue was that realities on the ground forced changes to the intervention. The issue of mobilizing people into the intervention appears to be common; however, this category is dominated by two papers (Pereze 2020 and Olken 2014) which cite a variety of mobilization challenges. As such, the evidence on this theme is somewhat weak. Contamination was also consistently reported. For the most part, it was mentioned due to its absence. Interventions whose goal was

For peer review only - http://bmjopen.bmj.com/site/about/guidelines.xhtml

engagement discussed this topic much more than interventions with engagement in their design or implementation.

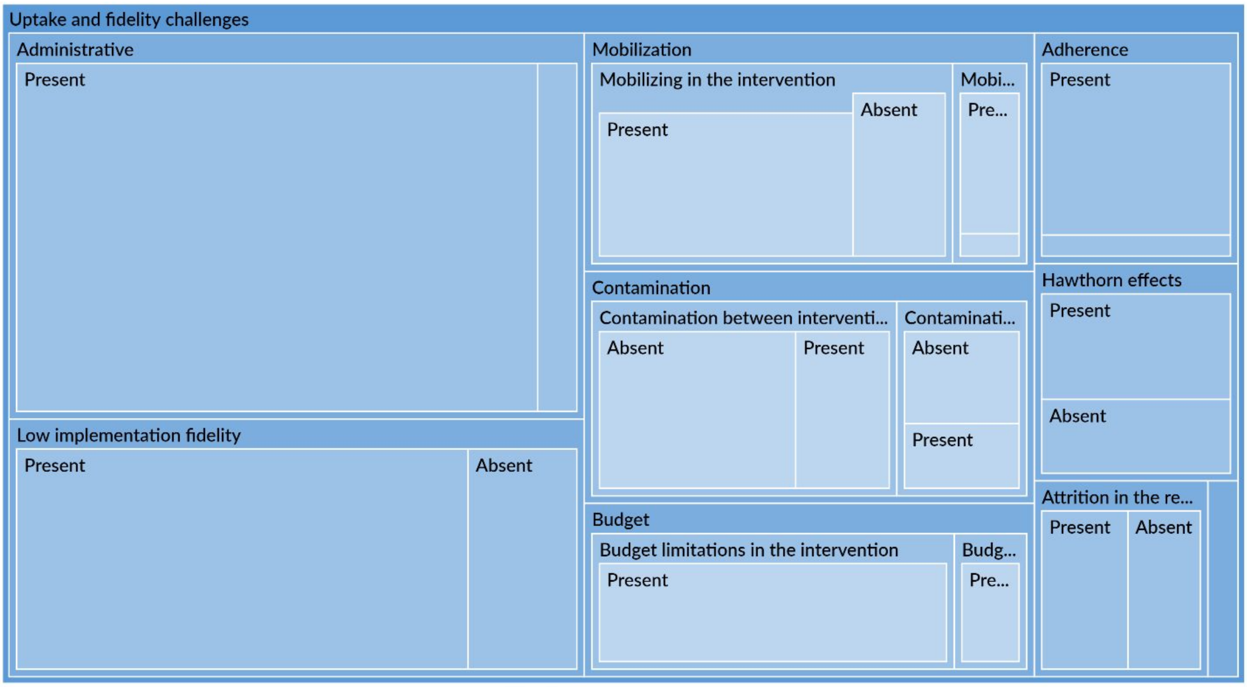

Supplementary Figure 98: Summary figure: uptake and fidelity challenges[2]

Supplementary Table 17: Comparison of the challenges faced by interventions using different types of community engagement. Numbers reflect relative frequency.

	**Community engagement as the intervention**	**Community engagement in the design of the intervention**	**Community engagement in the implementation of the intervention**
**Administrative challenges**	Technical issues<br>Politics<br>Coordination<br>Staffing	Technical issues<br>Politics<br>Bureaucracy<br>Security	Funding disbursement<br>Communication across stakeholders<br>Staffing
**Mobilization challenges**	Gender based religious and cultural norms prevented participation***	Recruiting people into the intervention due to work, distance, and competing priorities*	Recruiting facilitators and participants into the intervention due to other opportunities, miscommunication of intervention expectations, declines in incentives, caregiving obligations, and more**
**Fidelity challenges**	Failure to account for on the ground realities resulted in changes or failures in intervention implementation. Improper or insufficient supervision was also	Failure to account for on the ground realities resulted in changes or failures in intervention implementation	Failure to account for on the ground realities resulted in changes or failures in intervention implementation

[2] Coding is presented in a present/absent format because, in some cases, authors explicitly stated that a challenge was not encountered.

For peer review only - http://bmjopen.bmj.com/site/about/guidelines.xhtml

		common.			
**Contamination**		Authors were more likely to discuss the absence of this issue than to report upon its presence.	Authors were more likely to discuss the absence of this issue than to report upon its presence.		
**Budget restrictions**					Inadequate or delayed pay resulted in activities not being implemented.

*Most of this evidence came from a Pereze 2020 which cited multiple reasons for mobilisation challenges
**All evidence on challenges came from Olken 2014
***All evidence from Olken 2014 also applies to this group as Olken 2014 falls into both categories

Community engagement as the intervention

Discussions of *administrative challenges* consistently related to coordination, technical issues, staffing, and politics. The single study that reported on the absence of certain administrative issues also reported on the presence of many others. This is likely because it was a 3ie report, which tend to be quite detailed and include significant qualitative components.

Issues with the faithful *implementation* of these interventions were consistently the result of improper supervision and management or a failure to account for practicalities on the ground. We found one case where the administrative challenges directly led to low implementation fidelity. However, a non-trivial proportion of studies reported statistics indicating high fidelity.

The studies that discussed contamination largely did so in order to demonstrate that contamination was not a concern. Authors consistently focused on the issue of contamination between intervention and control sites. This was often addressed with vague statements. Distance was largely used to avoid contamination.

Discussion of challenges in mobilization interventions was dominated by a single source (Olken et al 2014 also discussed the engagement in implementation section). However, Rahman 2016 also discussed several mobilization issues that resulted from religious and cultural norms, largely around gender, preventing participation.

For peer review only

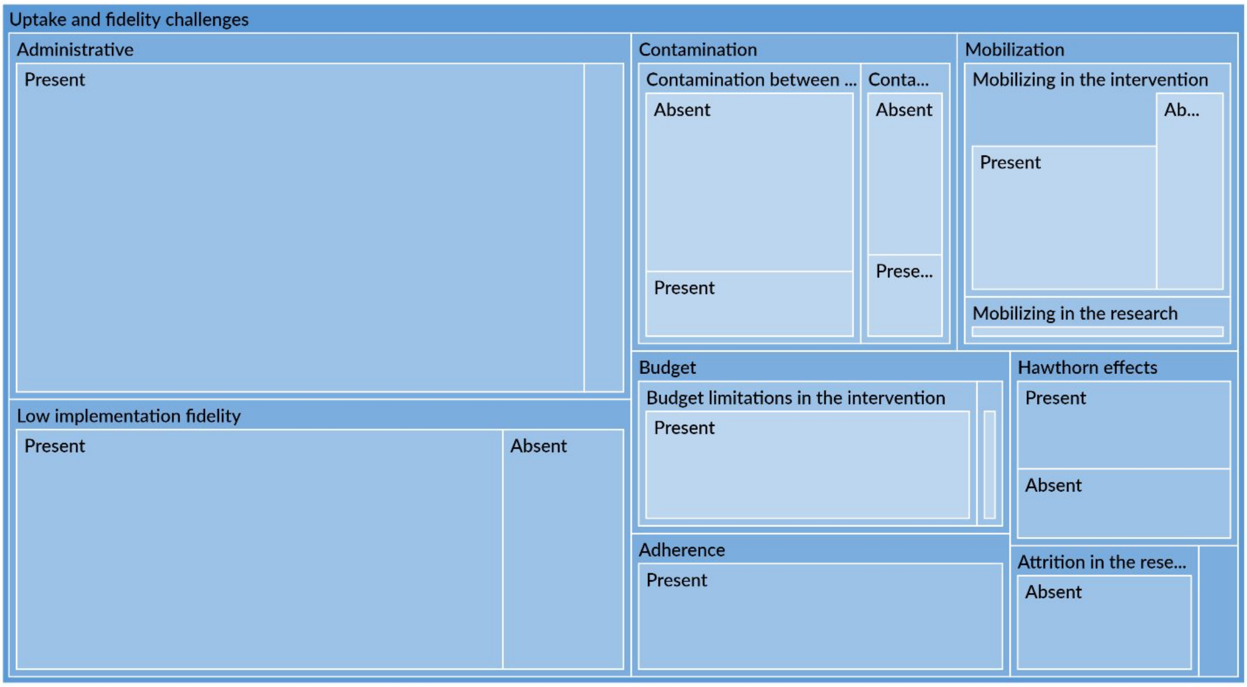

Supplementary Figure 99: Engagement as intervention

Supplementary Table 18: Characteristic quotes of themes identified related to uptake and fidelity challenges for interventions were community engagement.

**Primary paper**	**Paper from which citation comes**	**Sub-theme demonstrated**	**Quote**	**Page number**
*Administrative challenges**				
Assengai et al 2018	Khuzwayo et al 2017	Coordination challenges	Respondents report a lack of communication about visit scheduling.	3
			This is compounded by dual reporting lines in many parts of the country, where CHWs remain linked to and receive stipends through NGO intermediaries, while being accountable to PHC facility managers.	
Demilew et al 2019	Demilew et al 2020	Technical and political challenges	The Mobile Intervention was left from being implemented because of Internet service inaccessibility that resulted from the state of emergency declared by the Ethiopian government.	23

For peer review only - http://bmjopen.bmj.com/site/about/guidelines.xhtml

Banerjee et al 2020	Banerjee et al 2020	Political challenges	This study has faced various degrees of support from our primary government stakeholder since its inception, and this is the main reason why the timeline has been extended multiple times over the past seven years (when discussions with the Haryana government first began). We faced issues in continuity and buy-in especially when higher-ranking officers were transferred.	69
		Absence of administrative barriers	Given that J-PAL South Asia was also responsible for implementation, we recruited a dedicated team with relevant experience to do this. As a result, we were acutely aware of what was happening in the field, and able to identify and resolve issues quickly.	71
Implementation fidelity				
Biemba et al 2016	Phiri et al 2017	Inadequate supervision	Supervision and mentorship is crucial to optimizing the skills of CHAs, but this study outlines that supervision is not always implemented as intended and that in the absence of regular supervision	7
Morris et al 2004	Morris et al 2004	Expectations did not match realities on the ground	The service-level package was not implemented in accordance with protocol because no legal means could be identified of transferring resources	2034
Demilew et al 2019	Demilew et al 2020	Administrative challenges caused implementation challenges	This intervention was designed on the expectation that the text message portion could be rolled out in tandem with the ECIIN, an immunisation tracking program that planned to have HEWs report immunisation dates, mother's data, child's data, using text messages. Due to data issues and conflict in the region ECIIN was discontinued.	34
Gurley et al 2020	Gurley et al 2020	High fidelity	The types of screenings were conducted as planned, with 446 mothers group screenings, 445 VHND screenings, 223 men's screenings, and 444 HTR screenings (Table 6). Within a single village, this translated to an average of 12 sessions held in mothers groups, 12 held in VHNDs, 12 held in HTR areas, and 6 held in men's groups.	23
Contamination				
Moore et al 2017	Moore et al 2017	Absence of contamination	Our intervention and control groups were generally similar, with high coverage and fidelity to planned activities and negligible contamination.	e347

For peer review only - http://bmjopen.bmj.com/site/about/guidelines.xhtml

Paraminik et al 2018	Paraminik et al 2020	Distance reduced contamination	To mitigate potential contamination between intervention and control villages, we attempted to ensure that the intervention and control villages are sufficiently far apart from one another.	25

*Challenges related to mobilisation are not presented because this information largely came from a single paper making the identification of characteristic issues *across* papers impossible.

Community engagement in the design of the intervention

Among interventions that used community engagement in their design, the most consistently reported *administrative challenge* related to technical issues, largely surrounding phones and internet connections (Supplementary Table 17). However, other administrative challenges were related to combinations of politics, bureaucracy, and security concerns. The single paper that indicated a lack of administrative challenges reported successful syncing to the server, but also reported on other technological issues.

Mobilization challenges largely related to recruiting people into the intervention. Most of this information came from a 2020 (Pereze et al 2020) paper which presented information on the implementation fidelity and acceptability of an intervention that combined in-person and digital education to promote vaccination (Johri 2018). Authors describe challenges with participants being unable to attend due to work, distance, and competing priorities. Limited access to phones made mobilization difficult as well.

Acknowledgement of *low implementation fidelity* was consistently accompanied by discussions of why changes were made or the implications of these changes for results. Generally, changes in the intervention plan were forced due to practical constraints that had not been accounted for and were expected to result in an attenuation of impacts. Reports of high fidelity were succinct and responded to concerns a reader may be expected to have.

Contamination can occur between the intervention and control or due to ongoing activities of other programs. The former of these was reported more consistently than the latter. Authors were more likely to discuss the absence of this issue than to report upon its presence. Some offered explanations as to why contamination was not a concern or steps taken to avoid contamination. Others reported on results related to measures of contamination.

For peer review only

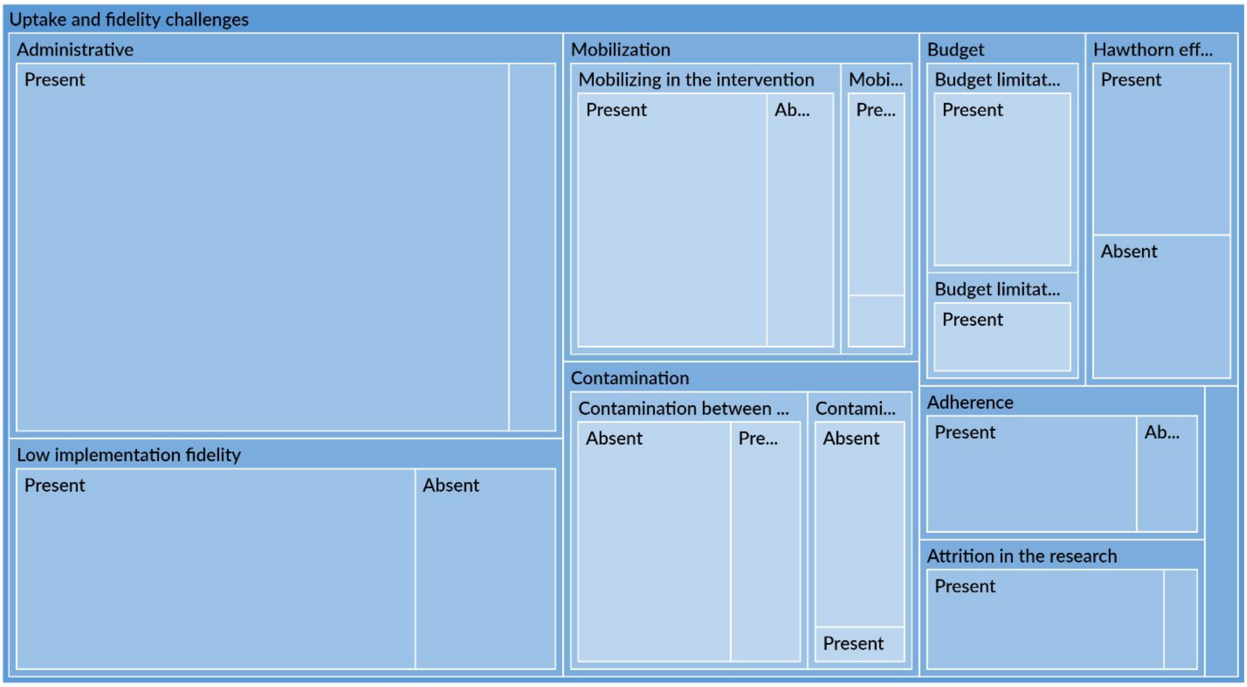

Supplementary Figure 100: Engagement in design

Supplementary Table 19: Characteristic quotes of themes identified related to uptake and fidelity challenges for interventions that used community engagement in their design

**Primary paper**	**Paper from which citation comes**	**Sub-theme demonstrated**	**Quote**	**Page number**
*Administrative challenges**				
Nagar et al 2020	Nagar et al 2020	Technological problems	The most prevalent issues reported by ANMs using the KB App included having to reenter data after an app crash (60.9%), having issues with scanning the pendant (65.2%), and issues with the application crashing (56.5%), and time required to enter data (33.3%).	138
Modi et al 2019	Modi et al 2015	Technological problems	Limited phone memory occasionally created technology-related issues, with the increasing requirement to store data on the mobile phone.	10
		Absence of technological problems	The failure rate, defined as the proportion of forms that failed to get synced with the server, was <1%.	266
Implementation fidelity				

For peer review only - http://bmjopen.bmj.com/site/about/guidelines.xhtml

Gruley et al 2020	Gruley et al 2020	Reason changes were implemented	Last, due to resource constraints, the project was not able to implement the intervention component for sharing videos from phone to phone using Bluetooth technology.	22
Engineer et al 2019	Engineer et al 2016	Implications of low fidelity for the research	Problems with implementation likely dampened any potential effect. The scheme was rolled out in phases, but there were some delays, particularly with the initial payments.	456
Banerjee et al 2010	Banerjee et al 2010	High implementation fidelity	Review of records showed that of 1336 planned camps, 95% (1269) took place.	2
Contamination				
Borkum et al 2014	Borkum et al 2014	Absence of contamination with other interventions	Because FLWs and households in the treatment and control groups received non-TBGI Ananya program interventions that were being implemented simultaneously across Begusarai, the RCT was designed to measure the value-added of TBGI beyond these other Ananya interventions. It was not designed to measure the impact of TBGI introduced in isolation.	6
Nagar et al 2018	Nagar et al 2016	Steps to avoid contamination	a cluster randomised approach further allowed non-contamination of social signaling interventions within the contacts of a given village	34
Johri et al 2018	Pereze et al 2020	Little contamination observed	According to the analysis of the records, only people who belonged to the intervention group attended the community meetings. The people who communicated with or received calls from the IVR platform were from the intervention group, except for one (1/166) control group member.	15

*Challenges related to mobilisation are not presented because this information largely came from a single paper making the identification of characteristic issues *across* papers impossible.

Community engagement in the implementation autonomy of the intervention

Administrative challenges in interventions that used engagement in their implementation largely related to funding disbursement, communication across stakeholders, staffing issues (Supplementary Table 17). One study (Bjorkman et al 2009) reported that they did not believe administrative differences affected results. This was

For peer review only - http://bmjopen.bmj.com/site/about/guidelines.xhtml

coded as the absence of an administrative challenge,but does not mean that administrative issues were not encountered.

Implementation fidelity appears to have been more of a challenge for these interventions than for those that leveraged community engagement in their design. These issues were consistently related to the intervention not functioning as expected. In some cases, this was because the reality on the ground did not reflect expectations. However, two studies that reference challenges with implementation fidelity also report upon successes in implementing according to plan.

All reports of challenges *mobilising* participants into interventions that were implemented by the community came from a paper related to an intervention that used block grants to support maternal and child health and education (Olken 2014). Challenges were encountered in the recruitment of both facilitators and participants. These related to other opportunities, miscommunication of intervention expectations, declines in incentives, caregiving obligations, and more. However, Herrara-Almanza 2018 report that community health workers felt strong responsibility for the wellbeing of their communities and attrition was low.

Interventions that used engagement in their implementation also experienced significant *budgetary constraints*. Almost universally, these constraints were the result of inadequate or delayed pay. Often, this resulted in activities not being implemented.

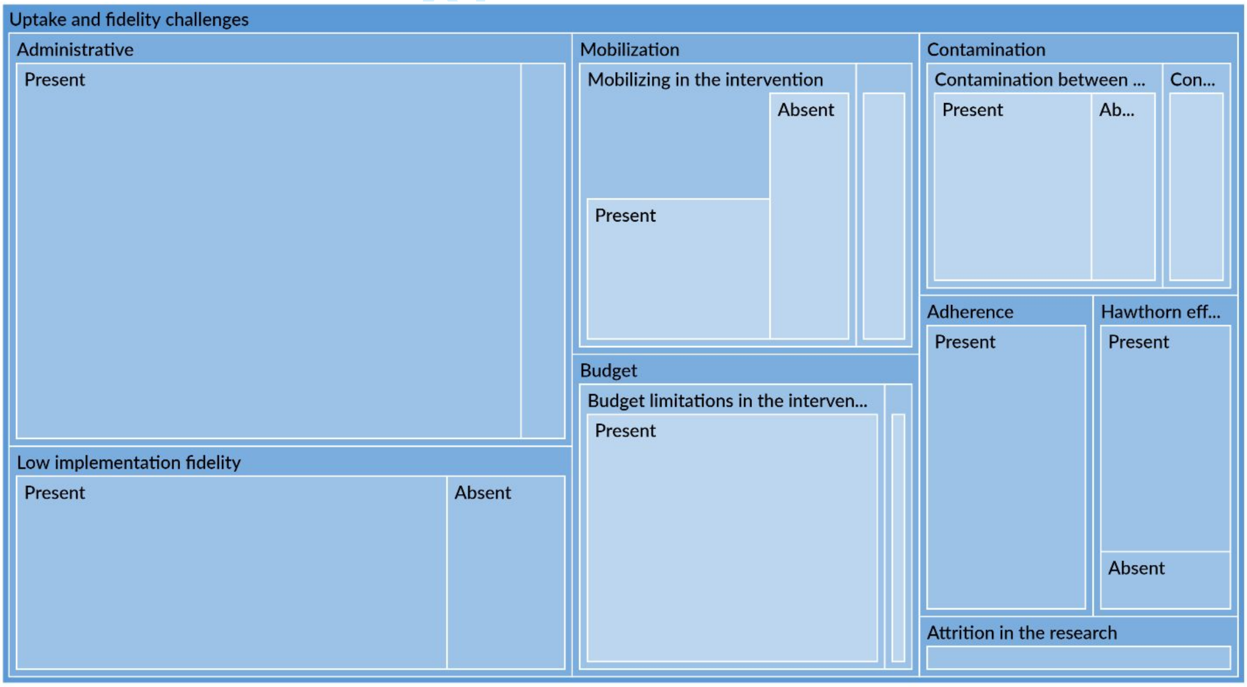

Supplementary Figure 101: Engagement in implementation autonomy of the intervention

Supplementary Table 20: Characteristic quotes of themes identified related to uptake and fidelity challenges for interventions that used community engagement in implementing the intervention.

**Primary paper**	**Paper from which citation comes**	**Sub-theme demonstrated**	**Quote**	**Page number**
Administrative challenges*				
Olken 2014	Febriany et al 2011	Delays in funding	The most frequent complaint by FDs regarding barriers to program	14

			implementation was the delays in aid disbursement.	
Mayumana et al 2017	Chimbutu et al 2011	Stakeholder communication problem	The NTPI"s programme, however, did not start in 2007 as planned… The major reason for this is on the implementation challenges between the two partners. The MOHSW preferred an accelerated approach while NORAD preferred an implementation framework to be designed by the Ifakara Health Institute (IHI).	16
Oyo-Ita 2020	Oyo-Ita 2020	Staffing and bureaucracy challenges	Low staffing of facilities constrained health staff from deploying the defaulters' register. Some complained that they had many registers other than the defaulters' register to fill.	18
Implementation fidelity				
Oyo-Ita 2020	Oyo-Ita 2020	Inconsistent implementation	The community engagement did not use town hall meetings as planned. Only one town hall meeting was held.	18
			The TRL training intervention was carried out as planned.	17
Okeke et al 2017	Okeke et al 2017	Expectations did not match realities on the ground	Contrary to the role of the states outlined in the MOU, policymakers in Enugu state reported playing no role in the implementation of the scheme. One suggested that the division of responsibility drawn up in the MOU did not align with the setup of health care in Nigeria given that the state government has responsibility for secondary care only.	50
Mayumana et al 2017	Olfasdottir et al 2014	Intervention did not function as expected	Firstly, the work load on staff is considerably higher than they are contracted for, which is not reflected in the remuneration; payments are delayed and overtime and eligible allowances are not always paid causing demotivation among staff.	7
		Intervention was implemented according to clear standards	The direct and transparent payment of funds, as well as more frequent contact with their managers was reported to enhance trust and improve the relationship between health workers and their managers.	64

For peer review only - http://bmjopen.bmj.com/site/about/guidelines.xhtml

Budgetary constraints				
Okeke et al 2017	Okeke et al 2017	Pay was severely delayed	Some local government don't have the money to pay and they have not paid for a period of time. Most of them [midwives] have even finished their service and gone out without receiving any penny from the local government.	41
Mayumana et al 2017	Mayumana et al 2017	Activities were suspended	According to the qualitative data one of the obstacles for providing more frequent supervision visits was a lack of financial resources:<br>We do supervision every quarter. Our plan is to do supervision monthly but due to limited budget we haven't yet done this.	6

*Challenges related to mobilization are not presented because this information came from a single paper making the identification of characteristic issues *across* papers impossible.

Sensitivity analysis for qualitative evidence

We carried out a sensitivity analysis in which we considered only the 17 qualitative studies associated with 12 impact evaluations that had a risk of bias assessment score of 20 or higher. Broadly, the themes that emerged from this analysis were consistent with those of the full analysis. We did not have enough qualitative evidence to conduct a sensitivity analysis by engagement type. An important caveat of this analysis is that nearly half of the papers rated as high "quality" were associated with three impact evaluations. The panel figures below provide comparison of the emerging themes in the full analysis versus the sensitivity analysis by comparing hierarchy charts related to barriers, facilitators, reasons for project success or failure, and uptake and fidelity challenges.

Barriers to immunisation

The most common barriers were broadly similar across the full analysis and the sensitivity analysis. *Behavioural, social and practical barriers faced by caregivers* were the most consistently reported barriers followed by *constraints in delivery of immunisation services* (Supplementary Figure 102). The most common barriers faced by caregivers were unavailability of immunisation services, financial constraints and social norms.

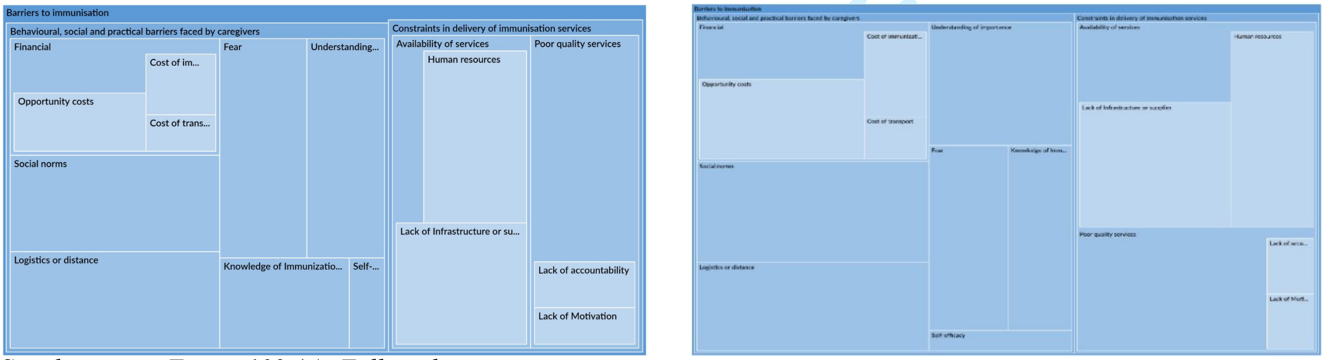

Supplementary Figure 102 (a): Full analysis
(b): Sensitivity analysis

Facilitators of immunisation

The most common facilitators of immunisation were broadly similar across the full analysis and the sensitivity analysis. *Behavioural, social and practical factors faced by caregivers* were the most consistently reported facilitators of immunisation in a given context followed by good *delivery of immunisation services*

(Supplementray Figure 103). The most consistently reported facilitators were availability of immunisation services, enabling social norms and caregivers' understanding of importance of immunisation.

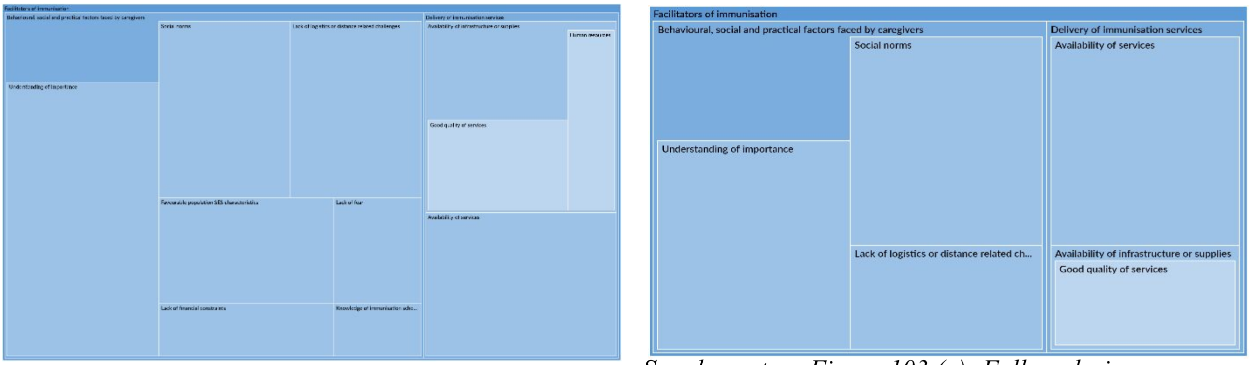

Supplementary Figure 103 (a): Full analysis

(b): Sensitivity analysis

Reasons for intervention success

The most consistently reported reasons for success were broadly similar across the full analysis and the sensitivity analysis. Success was consistently attributed to *intervention features* including leadership and supportive supervision, community engagement, health worker training, incentives and customisation to local context. Though not as common, success was also attributed to *existing or changing favourable characteristics* within a given context such as positive participant views, enabling social norms and availability of and access to good quality health services. Given that a very small subset of papers were included in the sensitivity analysis, a few themes that emerged in the full analysis were missing in the sensitivity analysis. Supplementary Figure 104 provides the hierarchy charts comparing the two analyses.

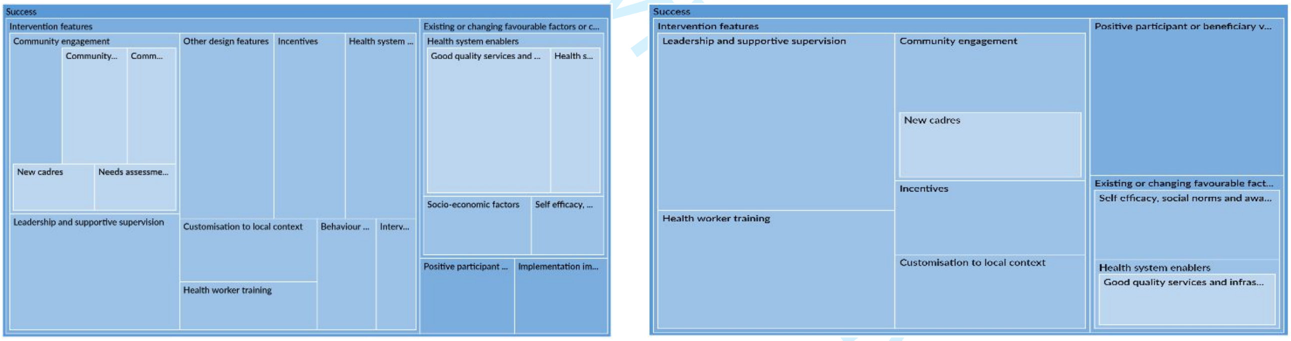

Supplementary Figure 104 (a): Full analysis

(b): Sensitivity analysis

Reasons for intervention failure

The most consistently reported reasons for failure were broadly similar across the full analysis and the sensitivity analysis. The most consistent reasons for failure were attributed to *accounting for contextual constraints* and inadequate *intervention features*. Social norms, health system related issues, political or civil unrest and high baseline coverage were some of the most common contextual reasons for failure. Among the intervention features, inadequate duration, frequency or exposure to the intervention were the most notable reasons for failure. Though not as prominently reported as compared to the full analysis, *implementation challenges* were also noted to have caused intervention failure in the sensitivity analysis. Among these, disruption due to inadequate implementation instructions and competing priorities of health workers were noted as some of the prevalent challenges.

For peer review only - http://bmjopen.bmj.com/site/about/guidelines.xhtml

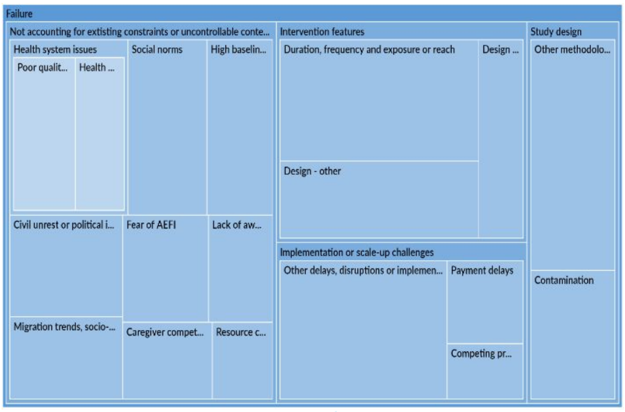

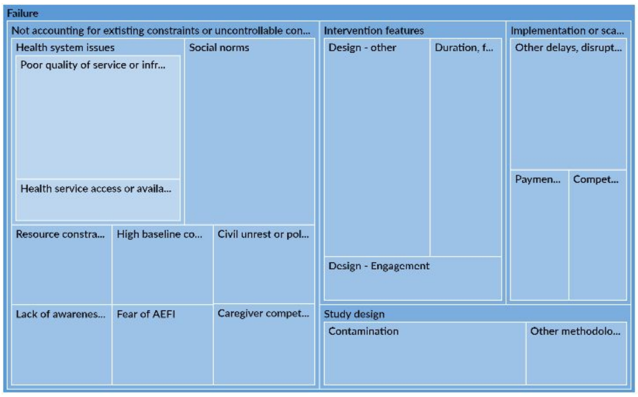

Supplementary Figure 105 (a): Full analysis

(b): Sensitivity analysis

Uptake and fidelity challenges

The most consistently reported uptake and fidelity issues were broadly similar across the full analysis and the sensitivity analysis. The most common challenges consistently reported challenges were related to administration, mobilization, fidelity, contamination and budget constraints. Administrative challenges were cited consistently. Similar to the full analysis, the primary most consistent issue was that realities on the ground forced changes to the intervention.

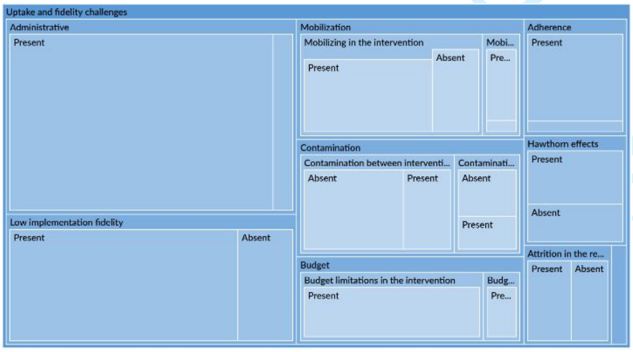

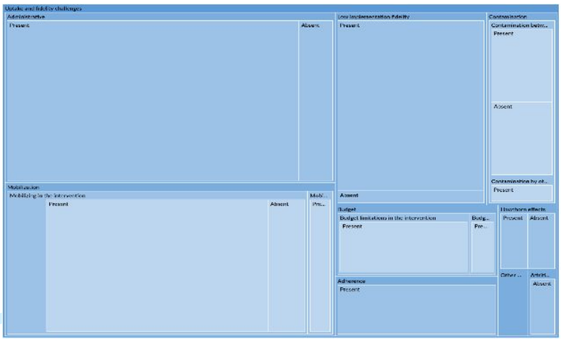

Supplementary Figure 106 (a): Full analysis

(b): Sensitivity analysis

Appendix 15: Synthesis of cost evidence

This section reports on the results of the analysis of the inventory of included studies (see Supplementray Table 21) and of the estimates of the non-vaccine cost per dose of interventions to increase absolute immunisation coverage by one percent (see Supplementary Table 22).

For peer review only

Characteristics of included studies

Twenty-two of the evaluations selected for inclusion in the systematic review reported some type of cost analysis. Of these, 18 used an experimental design and the remaining 4 used quasi-experimental methods to identify the impact of treatment.

Of the 22 studies: 17 studies reported a total cost of the intervention; 12 included a cost-effectiveness analysis; three included cost-efficiency analysis; two studies included a cost-benefit analysis and two analysed cost per quality-adjusted life year (QALY).

Supplementary Table 21: Characteristics of studies including any type of cost analysis.

					Type of Cost Analysis			
Author, Date	Country	Evaluation design	Intervention exposure (months)	Total Cost	Cost-efficiency Analysis	Cost-effectiveness Analysis	Cost-Benefit Analysis	Cost per QALY or DALY
Andersson, 2013	Pakistan	Experimental	8	✓				
Banerjee, 2010	India	Experimental	18			✓		
Banerjee, 2020	India	Experimental	14	✓		✓		
Björkman, 2009	Uganda	Experimental	0.17	✓				
Borkum, 2020	India	Experimental	12		✓	✓		
Demilew, 2020	Ethiopia	Experimental	17	✓				
Gurley, 2020	India	Experimental	11	✓		✓		
Johri, 2009	India	Experimental	3	✓				
Manoj, 2020	India	Experimental	4	✓				
More, 2017	India	Experimental	24	✓				
Morris, 2020	Honduras	Experimental	24	✓		✓		
Nagar, 2020	India	Experimental	20	✓		✓		
Olken, 2014	Indonesia	Experimental	24	✓		✓		
Oyo-Ita, 2020	Nigeria	Experimental	18	✓		✓		
Pramanik, 2018	India	Experimental	13	✓				
Seth, 2010	India	Experimental	9.7			✓		
Webster, 2019	Uganda	Experimental	12	✓			✓	
Admassie, 2017	Ethiopia	Quasi-experimental	48		✓			
Carnell, 2014	Ethiopia	Quasi-experimental	48	✓				
Findley, 2004	Nigeria	Quasi-experimental	24	✓		✓		
Saggurti, 2009	India	Quasi-experimental	2			✓		✓

By country. Fourteen of the community participation interventions to improve child immunisation studies were conducted in Asian countries: of these, 12 studies took place in India; and one each in Pakistan and Indonesia. One study was conducted in a Central American country, Honduras and seven studies were conducted in African countries: three in Ethiopia; and two each in Nigeria and Uganda

For peer review only - http://bmjopen.bmj.com/site/about/guidelines.xhtml

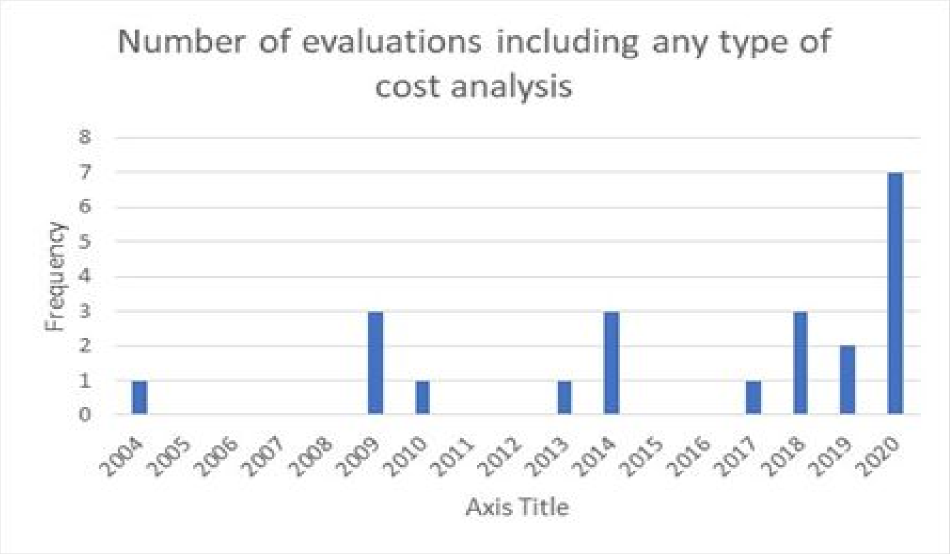

Supplemenary Figure 107: Number of evaluations with any type of cost analysis

The production of cost evidence in published evaluations of community participation interventions to improve child immunisation in low- and middle-income countries was uneven over the years. The first recorded evaluation with any cost analysis was published in 2004. Between 2009 and 2019, no more than three of the identified evaluations included any type of cost analysis in any given year. In 2020, the largest number of evaluations, seven, were published in a single year. The seven evaluations published in 2020 were required to generate cost analysis in partial fulfillment of the grant requirements in 3ie's Innovations in Increasing Immunisation Evidence Programme. Funding for these studies was provided by the Bill and Melinda Gates Foundation.

Non-vaccine cost per dose of intervention to increase immunisation coverage by one percent

Of the 22 included studies reporting cost and impact information, we estimate the non-vaccine cost per dose of intervention to increase immunisation coverage by one percent for 14. Because several studies report results by treatment arm, we report results for a total of 17 treatment arms. These results are summarised in detail in Supplementary Table 22.

Three studies and one treatment arm were dropped from the analysis. In the case of Johri, 2020 this was because the proportion of children vaccinated was not reported as an outcome of this evaluation. Morris 2020 was dropped because it is unclear how to combine the PRAF II costs reported in a separate paper with intervention as described in Moris, 2020, in addition, the evaluation found a null result on the immunisation outcomes. Findley, 2013 was dropped because the reported cost information isinsufficient for generating a total cost estimate or cost per vaccine dose, and "immunisation status" is not clearly defined. In the case of Banerjee, 2020, no coverage change information was reported for the Gossip seeds treatment arm which was dropped from the analysis as a result.

Six studies, Findley, 2004; Pramanik, 2018; Webster, 2019; Oyo-Ita, 2020, Modi, 2019; and Demilew, 2020 reported negative or null results and were excluded from the analysis of non-vaccine cost per dose of intervention to increase immunisation coverage by one percent.

The median non-vaccine cost per dose of intervention to increase immunisation coverage by one percent for the 14 estimates was US $2.30 (all costs are reported in 2019 US dollars). In comparison, the average was US $ 43.45, which is driven by three observations, Olken 2014 (US $23.10); Carnell, 2014 (US $641.08); and Gurley, 2020 (US $29.98). The

For peer review only - http://bmjopen.bmj.com/site/about/guidelines.xhtml

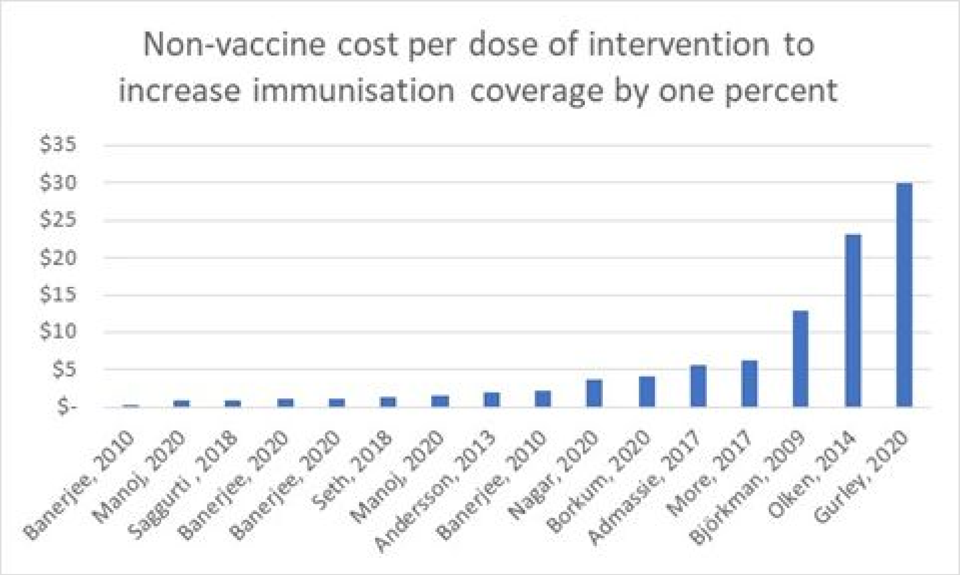

Supplementary Figure 108: Non-vaccine cost per dose of intervention to increase immunisation coverage by one percent

Supplementray Figure 106 illustrates that the range of estimates varied from a minimum of US $0.31 to a maximum of US $29.98. The lowest cost per vaccine dose was observed in Banerjee, 2010 for the "camps with incentives" intervention at US $0.31 per vaccine dose to increase immunisation coverage by one percent. Thehighest cost was observed in Carnell, 2014 at $641.08 - where the five-year intervention included system-building activities such as building and staffing a health post. In addition, the transparency of cost reporting in Carnell is very low, which further contributes to uncertainty in this cost estimate Indeed, Munk et. Al (2019) dropped this study from their systematic review of costs because they judged the available cost information to beinsufficient to calculate the cost per vaccine dose.

The cost-efficiency of the interventions was measured by calculating the non-vaccine intervention cost per vaccine dose. Cost-efficiency of the interventions ranged from a minimum of US $3.87 per vaccine dose to a maximum of US $ 5,449 per vaccine dose. The average cost-efficiency was US $204.62 per vaccine dose (when the maximum value is excluded). The second-highest estimate of the non-vaccine intervention cost per vaccine dose was US $204.62. Thus the observed variation in cost-efficiency is at least partly explained by including the most costly intervention, i.e. from Carnell (2014), which included health-system building activities (i.e. building and staffing a health post).

For peer review only - http://bmjopen.bmj.com/site/about/guidelines.xhtml

Supplementary Table 22: Detailed estimates of non-vaccine intervention cost per dose and absolute coverage change

Author, Date	Country	Total Cost USD $2019	Treatment	Outcome	Vaccines Reported (list)	Number of doses	Non-vaccine intervention cost per vaccine dose ($ 2019 USD)	Absolute coverage change (Intervention - Control)	Non-vaccine cost per dose of intervention to increase immunization coverage by one percent	Impact notes	Cost notes	Immunization notes
Oyo-Ita, 2020	Nigeria	$15,158	Training of traditional and religious leaders; health workers; and community mobilization to improve vaccination	Proportion of children with up-to-date vaccination	Pentavalent3, Measles	5 per protocol	$4.59	Null effect	negative / null result	Unadjusted proportion, endline (intervention - control)	Incremental to no intervention control group, excludes healthcare system-building costs	46% is baseline up to date vaccination in intervention, 48% control
Demilew, 2020	Ethiopia	$224,269	A system of tracking children's immunisation status using a poster and stamp system	Probability of receiving all 3 doses of DPT/PCV	DPT - Diphtheria, pertussis, and tetanus / PCV - Pneumococcal Conjugate Vaccine	6 per protocol	$23	Null effect	negative / null result	Unadjusted proportion, endline (intervention - control)	Incremental to no intervention control group, excludes healthcare system-building costs	Baseline immunization not reported
Seth, 2018	India	$2,178	Reminders + Incentives	Proportion of total number of immunizations received by a child divided by the total number of immunizations required	BCG, DPT, H influenzae type B, Polio, Hepatitus B, Rotavirus, Measles	3.21 per child, 574 doses	$4	3.1	$1.252	Unadjusted proportion, (end of study - baseline)	Incremental to no intervention control group, excludes healthcare system-building costs	40% is baseline immunization coverage in treatment and 39% in control
Banerjee, 2010	India	$4,427.87	Camps without incentives	Proportion of children fully immunised	Full immunisation is one dose of BCG vaccine, three doses of DPT (diphtheria-pertussis, tetanus)	2.35 per child	$27.62	12.0	$2.30	Unadjusted proportion, endline (intervention - control)	intervention control group, excludes healthcare system-building costs	2% is baseline full immunsation rate in the study area
Banerjee, 2010		$4,296.98	Camps with incentives	Proportion of children fully immunised	vaccine, three doses of oral polio vaccine, and one dose of measles vaccine.	2.85 per child	$10.12	33.0	$0.31	Unadjusted proportion, endline (intervention - control)	intervention control group, excludes healthcare system-building costs	2% is baseline full immunsation rate in the study area
Johri, 2009	India	-	-		-	-	-	-	-	-	-	-
Andersson, 2013	Pakistan	$70,981.46	Three structured community discussions of of local vaccination rates; costs and benefits of childhood vaccination; and local action plans.	Uptake of full DPT-3 and Measles vaccination in children 12-23 months	DPT-3, Measles	5 per protocol	$50.16	24	$2.07	Unadjusted proportion, endline (intervention - control)	Incremental to no intervention control group, excludes healthcare system-building costs	Baseline immunization not reported
Findley , 2004	Nigeria	-	-		-	-	-	-	negative / null result	-	-	-
Moris, 2020	Honduras	-	-		-	-	-	-	-	-	-	
Pramanik, 2018	India	$101,636.61	Community-based learning cycles where a community identifies a problem, makes an action plan, takes action and learns from the process	Full immunization take-up	1 BCG dose; 3 doses OPV; 3 doses DPT or Pentavalent vaccine, and one measles vaccine	8 per child 12 - 23 months	$17.20	FIC = -1%	negative / null result	Unadjusted proportion, endline (intervention - control)	Incremental to no intervention control group, excludes healthcare system-building costs	75% is baseline full immunization coverage in treatment and 76% in control

Author, Date	Country	Total Cost USD $2019	Treatment	Outcome	Vaccines Reported (list)	Number of doses	Non-vaccine intervention cost per vaccine dose ($ 2019 USD)	Absolute coverage change (Intervention - Control)	Non-vaccine cost per dose of intervention to increase immunization coverage by one percent	Impact notes	Cost notes	Immunization notes
Saggurti , 2018	India	$39,597.45	Participatory behavior communcation on maternal, neonatal, child health and promoting collectivization processes through women's self-help groups (SHGs)	Age-appropriate immunization among children under one year	9 doses - 1 BCG, 1 OPV, 3 OPV, 1,2,3,; 3 DPT, 1 Measles	9 per child under 1 year	$9.37	9	$1.03	Quasi-experimental. DID estimate. Intervention and control proportions were adjusted for baseline covariates	Incremental to no intervention control group, excludes healthcare system-building costs	Baseline immunization not reported
Admassie, 2017	Ethiopia	$1,008,991.46	Received Measles vaccine for children 9 to 11 months	Proportion of children 12- 60 months old vaccinated against major diseases. tuberculosis, diphtheria, whooping cough (pertussis), tetanus, polio and measles	8 doses: tuberculosis, polio, diphtheria- pertussis- tetanus, and measles	8 per child 12 - 60 months	$56.19	9.9	$5.68	Quasi-experimental. Intervention and control proportions were adjusted for baseline covariates	Incremental cost of running Health Services Extension Programme (HSEP) in the treatment areas, includes system-building cost to construct and staff a health post	Baseline immunization not reported
More, 2017	India	$213,420.20	Community resource centres	Proportion of children aged 12- 23 months fully immunised	BCG, diphtheria, pertussis, and tetanus [three doses], polio, hepatitis B virus [three doses], and measles	8 per protocol	$37.02	5.8	$6.34	Unadjusted proportion endline, (intervention - control). Change was positive but not statistically significantly different from zero	Incremental to no intervention control group,excludes healthcare system-building costs	65% is baseline full immunization coverage in treatment and 63% in control
Olken, 2014	Indonesia	$12,264,782.40	Village-level block grants for maternal and child health and education that incorporated relative performance incentives	Proportion of children under two years of age with BCG vaccine	1 dose, BCG	1.00	$36.97	1.60	$23.11	Adjusted average standardized effect (absolute coverage change) was positive but not statistically significantly different from zero.	Incremental cost of preference-weighted incentive block grant program for vaccines relative to the cost of the program of community block grants without incentives	65.3% is baseline full immunization coverage
Borkum, 2020	India	$39,571.93	A program that offers non-monetary incentives for teamwork and goal-setting activities to improve maternal and child health at health susbcenters in India	Proportion of children ages 6-11 months who recieved DPT-3 by the age of 6 months	DPT-3	585	$22.54	5.4	$4.17	Adjusted proportion (intervention - control). Difference is positive but not signficantly different from zero	Incremental to no intervention control group, excludes healthcare system-building costs	45.5% is baseline mean for a child fully immunized except measles in the treatment group
Manoj, 2020	India	$61,344.88	Information treatment	Full immunization of children 12-23 months	8 doses: BCG, measles, and 3 doses each for Polio and DPT	8 per protocol	$14.10	8.8	$1.60	Unadjusted increase in proportion immunised in intervention relative to control. We report separately by treatment arm because there is corresponding cost info.	Incremental to no intervention control group, excludes healthcare system-building costs	Baseline immunization not reported
Manoj, 2020	India	$61,344.88	Information + Facilitation treatment	Full immunization of children 12-23 months	8 doses: BCG, measles, and 3 doses each for Polio and DPT	8 per protocol	$12.63	13.4	$0.94	Unadjusted increase in proportion immunised in intervention relative to control. We report separately by treatment arm because there is corresponding cost info.	Incremental to no intervention control group, excludes healthcare system-building costs	Baseline immunization not reported

Björkman, 2009	Uganda	$182,003.09	Community-based monitoring of public primary health care providers	Proportion of children ages 13-24 months who received required doses of DPT	3 doses: DPT	3	$76.83	6.0	$12.81	Adjusted percent increase in 1-year old immunisation rate	Back of the envelope total cost, incremental intervention cost relaitve to control	Baseline immunization not reported
Banerjee, 2020	India	$342,595.94	High slope incentives			12 per pro	$13.81	11	$1.21	Adjusted percent change in fully immunised	Incremental to no intervention control group, excludes healthcare system-building costs	20.8% - 50.05% is the range of baseline means, depending on the district for a child fully vaccinated
		$367,078.25	Low slope incentives			12 per pro	$13.85	12	$1.14	Adjusted percent change in fully immunised	Incremental to no intervention control group, excludes healthcare system-building costs	20.8% - 50.05% is the range of baseline means, depending on the district for a child fully vaccinated
		$78,072.73	Gossip seeds	Proportion of children under 12 months who received 1 dose of BCG, 4 doses of the oral polio vaccine, 3 doses of penta, 3 doses of rotavirus and 1 dose of measles	BCG, penta doses 1- 3 and measles-1	12 per pro	NA	-	-	No coverage change reported for the Gossip seeds treatment.	Incremental to no intervention control group, excludes healthcare system-building costs	20.8% - 50.05% is the range of baseline means, depending on the district for a child fully vaccinated
Nagar, 2020	India	$275,702.59	Tracking system: The Khushi Baby platform, a wearable digital health record (pendant) and with patient-specific, dialect-specific voice reminders	Full and timely Immunization as verified by MAMTA card for children 12-23 months	8 doses: Polio 1-3, BCG, Pentavalent 1-3, Measles	6 per protocol	$41.17	11.2	$3.68	Unadjusted increase in proportion immunised in intervention relative to control	Incremental to no intervention control group, excludes healthcare system-building costs	25.4% is intervention baseline, full immunisation without birth doses and 22.5% is control
Gurley, 2020	India	$175,603.81	PATH - community-led videos	Proportion of children 6 to 17 months of age who had received all age-appropriate vaccines in India's vaccine schedule	13 doses: Brith doses (OPV, BCG, HepB); DPT1-3; HepB1-3; MR; OPV1-3	13 per protcol	$26.39	0.88	$29.98	Unadjusted proportion, endline (intervention - control). Difference is positive but not signficantly different from zero	Incremental to no intervention control group, excludes healthcare system-building costs	52.88% is baseline percentage of children in the State of Uttar Pradesh who received all necessary vaccines in 2012- 2013
Carnell , 2014	Ethiopia	$27,316,577.57	Three pillars approach Eshe project: strengthening health systems, improving health workers' performance, and engaging the community	Percentage of children aged 12-23 months, who received DPT3 vaccination, or Measles	2 doses: DPT3, Measles	2 per protocol	$5,449.19	8.5	$641.08	Coverage change is the average for DPT3 and Measles. Cost data very low transparency, making cost estimate unreliable	System-building' activities are evident in this intervention. However, cost data hvae very low transparency. To calculate total cost for treating vaccinated	Baseline immunization not reported
Modi, 2019	India	$160,881.37	A mobile-phone app to assist ASHAs and health providers to schedule and track home health visits and follow-on care	Proportion of infants (6-8 months) who received all three doses of DPT or Pentavalent vaccine	3 doses: DPT or Pentavalent vaccine	3 per protocol		null result	negative / null result	Unadjusted proportion, endline (intervention - control)	Incremental to no intervention control group, excludes healthcare system-building costs	Baseline immunization not reported
Webster, 2019	Uganda	$224,224.18	mReach data platform and the 'Fifth Child', a community engagement strategy to increase immunisation	Proportion of children ages 12-23 months with DPT3 and MCV combined immunization coverage	2 doses: DPT3, Measles	2 per protocol	$204.63	null result	negative / null result	Unadjusted proportion, endline (intervention - control)	Incremental to no intervention control group, excludes healthcare system-building costs	Baseline immunization not reported

For peer review only - http://bmjopen.bmj.com/site/about/guidelines.xhtml

PRISMA 2020 Checklist

Section and Topic	Item #	Checklist item	Location where item is reported #page no
**TITLE**			
Title	1	Identify the report as a systematic review.	p.1
**ABSTRACT**			
Abstract	2	See the PRISMA 2020 for Abstracts checklist.	pp. 2-3
**INTRODUCTION**			
Rationale	3	Describe the rationale for the review in the context of existing knowledge.	pp. 4-5
Objectives	4	Provide an explicit statement of the objective(s) or question(s) the review addresses.	p. 4
**METHODS**			
Eligibility criteria	5	Specify the inclusion and exclusion criteria for the review and how studies were grouped for the syntheses.	pp. 5-6, appendix 1
Information sources	6	Specify all databases, registers, websites, organisations, reference lists and other sources searched or consulted to identify studies. Specify the date when each source was last searched or consulted.	pp. 5-6, appendix 3
Search strategy	7	Present the full search strategies for all databases, registers and websites, including any filters and limits used.	p. 5, appendix 3, 4
Selection process	8	Specify the methods used to decide whether a study met the inclusion criteria of the review, including how many reviewers screened each record and each report retrieved, whether they worked independently, and if applicable, details of automation tools used in the process.	p. 5
Data collection process	9	Specify the methods used to collect data from reports, including how many reviewers collected data from each report, whether they worked independently, any processes for obtaining or confirming data from study investigators, and if applicable, details of automation tools used in the process.	p. 6
Data items	10a	List and define all outcomes for which data were sought. Specify whether all results that were compatible with each outcome domain in each study were sought (e.g. for all measures, time points, analyses), and if not, the methods used to decide which results to collect.	p. 6, appendix 6
	10b	List and define all other variables for which data were sought (e.g. participant and intervention characteristics, funding sources). Describe any assumptions made about any missing or unclear information.	p. 6, appendix 6
Study risk of bias assessment	11	Specify the methods used to assess risk of bias in the included studies, including details of the tool(s) used, how many reviewers assessed each study and whether they worked independently, and if applicable, details of automation tools used in the process.	p. 7, appendix 7
Effect measures	12	Specify for each outcome the effect measure(s) (e.g. risk ratio, mean difference) used in the synthesis or presentation of results.	p. 7
Synthesis methods	13a	Describe the processes used to decide which studies were eligible for each synthesis (e.g. tabulating the study intervention characteristics and comparing against the planned groups for each synthesis (item #5)).	p. 7
	13b	Describe any methods required to prepare the data for presentation or synthesis, such as handling of missing summary statistics, or data conversions.	p. 7
	13c	Describe any methods used to tabulate or visually display results of individual studies and syntheses.	p. 7
	13d	Describe any methods used to synthesize results and provide a rationale for the choice(s). If meta-analysis was performed, describe the model(s), method(s) to identify the presence and extent of statistical heterogeneity, and software package(s) used.	p. 7
	13e	Describe any methods used to explore possible causes of heterogeneity among study results (e.g. subgroup analysis, meta-regression).	p. 7
	13f	Describe any sensitivity analyses conducted to assess robustness of the synthesized results.	p. 7

For peer review only

For peer review only - http://bmjopen.bmj.com/site/about/guidelines.xhtml

PRISMA 2020 Checklist

Section and Topic	Item #	Checklist item	Location where item is reported #page no
Reporting bias assessment	14	Describe any methods used to assess risk of bias due to missing results in a synthesis (arising from reporting biases).	p. 7
Certainty assessment	15	Describe any methods used to assess certainty (or confidence) in the body of evidence for an outcome.	NA
**RESULTS**			
Study selection	16a	Describe the results of the search and selection process, from the number of records identified in the search to the number of studies included in the review, ideally using a flow diagram.	p. 8, figure 1
	16b	Cite studies that might appear to meet the inclusion criteria, but which were excluded, and explain why they were excluded.	pp. 6, 8, appendix 4
Study characteristics	17	Cite each included study and present its characteristics.	p. 8, table 1
Risk of bias in studies	18	Present assessments of risk of bias for each included study.	p. 21, appendix 8
Results of individual studies	19	For all outcomes, present, for each study: (a) summary statistics for each group (where appropriate) and (b) an effect estimate and its precision (e.g. confidence/credible interval), ideally using structured tables or plots.	p. 8
Results of syntheses	20a	For each synthesis, briefly summarise the characteristics and risk of bias among contributing studies.	pp. 9, 21
	20b	Present results of all statistical syntheses conducted. If meta-analysis was done, present for each the summary estimate and its precision (e.g. confidence/credible interval) and measures of statistical heterogeneity. If comparing groups, describe the direction of the effect.	pp. 21-26, appendix 9-13
	20c	Present results of all investigations of possible causes of heterogeneity among study results.	pp. 21-26, appendix 9-13
	20d	Present results of all sensitivity analyses conducted to assess the robustness of the synthesized results.	pp. 21-26, appendix 9-13
Reporting biases	21	Present assessments of risk of bias due to missing results (arising from reporting biases) for each synthesis assessed.	pp. 21-26, appendix 9-13
Certainty of evidence	22	Present assessments of certainty (or confidence) in the body of evidence for each outcome assessed.	NA
**DISCUSSION**			
Discussion	23a	Provide a general interpretation of the results in the context of other evidence.	p. 26
	23b	Discuss any limitations of the evidence included in the review.	pp. 3, 26
	23c	Discuss any limitations of the review processes used.	p. 3
	23d	Discuss implications of the results for practice, policy, and future research.	p. 28
**OTHER INFORMATION**			

For peer review only

PRISMA 2020 Checklist

Section and Topic	Item #	Checklist item	Location where item is reported #page no
Registration and protocol	24a	Provide registration information for the review, including register name and registration number, or state that the review was not registered.	p. 3
	24b	Indicate where the review protocol can be accessed, or state that a protocol was not prepared.	p. 3
	24c	Describe and explain any amendments to information provided at registration or in the protocol.	Appendix 2
Support	25	Describe sources of financial or non-financial support for the review, and the role of the funders or sponsors in the review.	p. 29
Competing interests	26	Declare any competing interests of review authors.	p. 29
7Availability of data, code and other materials	27	Report which of the following are publicly available and where they can be found: template data collection forms; data extracted from included studies; data used for all analyses; analytic code; any other materials used in the review.	p. 29

From: Page MJ, McKenzie JE, Bossuyt PM, Boutron I, Hoffmann TC, Mulrow CD, et al. The PRISMA 2020 statement: an updated guideline for reporting systematic reviews. BMJ 2021;372:n71. doi: 10.1136/bmj.n71

For more information, visit: http://www.prisma-statement.org/

For peer review only

Use of community engagement interventions to improve child immunisation in low- and middle-income countries: a systematic review and meta-analysis

Journal:	*BMJ Open*
Manuscript ID	bmjopen-2022-061568.R1
Article Type:	Original research
Date Submitted by the Author:	07-Jul-2022
Complete List of Authors:	Jain, Monica; International Initiative for Impact Evaluation Dehli, Evaluation Office<br>Shisler, Shannon; International Initiative for Impact Evaluation<br>Lane, Charlotte ; International Initiative for Impact Evaluation<br>Bagai, Avantika; International Initiative for Impact Evaluation, Evaluation Office<br>Brown, Elizabeth; University of California Berkeley, Center for Effective Global Action<br>Engelbert, Mark; International Initiative for Impact Evaluation, Synthesis and Reviews
**Primary Subject Heading**:	Public health
Secondary Subject Heading:	Global health
Keywords:	Public health < INFECTIOUS DISEASES, Community child health < PAEDIATRICS, PUBLIC HEALTH

I, the Submitting Author has the right to grant and does grant on behalf of all authors of the Work (as defined in the below author licence), an exclusive licence and/or a non-exclusive licence for contributions from authors who are: i) UK Crown employees; ii) where BMJ has agreed a CC-BY licence shall apply, and/or iii) in accordance with the terms applicable for US Federal Government officers or employees acting as part of their official duties; on a worldwide, perpetual, irrevocable, royalty-free basis to BMJ Publishing Group Ltd ("BMJ") its licensees and where the relevant Journal is co-owned by BMJ to the co-owners of the Journal, to publish the Work in this journal and any other BMJ products and to exploit all rights, as set out in our licence.

The Submitting Author accepts and understands that any supply made under these terms is made by BMJ to the Submitting Author unless you are acting as an employee on behalf of your employer or a postgraduate student of an affiliated institution which is paying any applicable article publishing charge ("APC") for Open Access articles. Where the Submitting Author wishes to make the Work available on an Open Access basis (and intends to pay the relevant APC), the terms of reuse of such Open Access shall be governed by a Creative Commons licence – details of these licences and which Creative Commons licence will apply to this Work are set out in our licence referred to above.

Other than as permitted in any relevant BMJ Author's Self Archiving Policies, I confirm this Work has not been accepted for publication elsewhere, is not being considered for publication elsewhere and does not duplicate material already published. I confirm all authors consent to publication of this Work and authorise the granting of this licence.

For peer review only

Use of community engagement interventions to improve child immunisation in low- and middle-income countries: a systematic review and meta-analysis

Monica Jain, Shannon Shisler, Charlotte Lane, Avantika Bagai, Elizabeth Brown, Mark Engelbert

International Initiative for Impact Evaluation (Monica Jain PhD, Shannon Shisler PhD, Charlotte Lane PhD, Avantika Bagai M.Sc and Mark Engelbert PhD) and **Center for Effective Global Action, University of Berkeley, California, USA** (Elizabeth Brown PhD)

Correspondence to:
Dr. Monica Jain
Evaluation Office, International Initiative for Impact Evaluation (3ie), New Delhi, 110017, India
Email: mjain@3ieimpact.org

For peer review only

Keywords:

Immunization

Immunization Programs

Mass Vaccination

Poverty Areas

Public Health Administration

Word count:

Body: 4,212

Abstract: 299

For peer review only - http://bmjopen.bmj.com/site/about/guidelines.xhtml

Abstract

Objective

To support evidence informed decision making, we systematically examine the effectiveness and cost-effectiveness of community engagement interventions on routine childhood immunisation outcomes in LMICs and identify contextual, design, and implementation features associated with effectiveness.

Design

Mixed-methods systematic review and meta-analysis.

Data sources

21 databases of academic and grey literature and 12 additional websites were searched in May 2019 and May 2020.

Eligibility Criteria for selecting studies

We included experimental and quasi-experimental impact evaluations of community engagement interventions considering outcomes related to routine child immunisation in LMICs. No language, publication type, or date restrictions were imposed.

Data extraction and synthesis

Two independent researchers extracted summary data from published reports and appraised quantitative risk of bias using adapted Cochrane tools. Random effects meta-analysis was used to examine effects on the primary outcome, full immunisation coverage.

Results

Our search identified over 43000 studies and 61 were eligible for analysis. The average pooled effect of community engagement interventions on full immunisation coverage was SMD 0.14 (95% CI: 0.06 to 0.23, $I^2 = 94.46$). The most common source of risk to the quality of evidence (risk of bias) was outcome reporting bias: most studies used caregiver-reported measures of vaccinations received by a child in the absence or incompleteness of immunisation cards. Reasons consistently cited for intervention success include appropriate intervention design, including building in community engagement features; addressing common contextual barriers of immunisation and leveraging facilitators; and accounting for existing implementation constraints.

For peer review only - http://bmjopen.bmj.com/site/about/guidelines.xhtml

The median intervention cost per treated child per vaccine dose (excluding the cost of vaccines) to increase absolute immunisation coverage by one percent was USD 3.68.

Conclusion

Community engagement interventions are successful in improving outcomes related to routine child immunisation. The findings are robust to exclusion of studies assessed as high risk of bias.

Protocol registration

DOI: 10.1002/cl2.1119.

Strengths and limitations of this study

- Thorough literature search of twenty-one major electronic databases and reporting as per Preferred Reporting Items for Systematic Reviews and Meta-Analysis guidelines.
- Presents a nuanced framework of community engagement with a typology that differentiates three types of interventions: interventions in which community engagement is embedded, and those interventions that engage community in their design or implementation.
- The effects of community engagement interventions are robust to exclusion of studies assessed as high risk of bias for almost all the primary outcomes. The effects are also uniform across geographies and baseline immunisation rates.
- For some immunisation outcomes the evidence base for drawing conclusions is adequate, for others it is limited.
- Evidence base is skewed across the three engagement types with a relatively large evidence base for those interventions in which community engagement is embedded and limited for those interventions using engagement in implementation autonomy

For peer review only - http://bmjopen.bmj.com/site/about/guidelines.xhtml

Introduction

Immunisation is one of the most cost-effective ways to prevent and control life-threatening infectious diseases. From 2001 to 2020, projects that introduced or increased coverage of vaccines averted an estimated 14 million deaths, 350 million cases of illness, 8 million cases of long-term disability and 700 million disability-adjusted life-years.[1] Nonetheless, rates of routine vaccination of children in low- and middle-income countries (LMICs) are low or stagnant. In 2019, an estimated 19.7 million infants did not receive routine immunisations. Around 60% of these children live in ten LMICs, including Ethiopia, India, Nigeria, and Pakistan as of 2019.[2]

Community engagement approaches feature prominently in global immunisation strategies.[3] However, there is a dearth of rigorous and systematic evidence on effectiveness of community engagement approaches to improve routine childhood immunisations specifically in LMICs. In our search we could find only two systematic reviews for LMICs which analysed effectiveness of community monitoring interventions and preventive interventions delivered by community health workers, respectively.[4,5] As such, previous systematic reviews do not provide adequate guidance to stakeholders interested in understanding whether and how alternative community engagement interventions work in LMICs to improve routine childhood immunisations and at what cost. There is, therefore, a need to make such evidence available to guide policymakers and public health practitioners in making informed decisions about these interventions. To address this knowledge gap, we conducted a systematic review examining the effects of community engagement interventions on outcomes related to childhood immunisation in LMICs, determining their cost-effectiveness, and identifying contextual, design and implementation features that may be associated with intervention effectiveness.

Methods

Overview

The protocol of this systematic review with meta-analysis is registered with The Campbell Collaboration.[6] We followed the Campbell and Cochrane Collaborations' guidelines for systematic reviewing[7-10] and drew on theory-based mixed methods impact evaluation[11] and

For peer review only - http://bmjopen.bmj.com/site/about/guidelines.xhtml

systematic review[12,13] concepts. We followed the PRISMA reporting guidelines. The amendments to the information provided in the protocol is reported in Appendix 1 pp.3-4.

Conceptual framework

For our review we defined "communities" in reference to the lowest level of the health service delivery system (or whatever level provides routine immunisation services in the local context). A community is a group of people who serve or are served by a particular primary health facility. Thus, communities encompass a wide range of stakeholders, including caregivers, health service providers, and influential community members such as religious or other traditional leaders.

WHO 2020 defines community engagement as "*a process of developing relationships that enable stakeholders to work together to address health-related issues and promote well-being to achieve positive health impact and outcomes*."[14] For this review we developed a framework that classified community engagement interventions based on *process* of engagement as in the WHO definition. It also corresponds to the "utilitarian perspective" of community engagement captured and articulated in Brunton et al. (2017)[15]: "*In utilitarian perspectives, health (and other) services reach out to engage particular communities that they have identified require assistance and the intervention is devised within existing policy, practice, and resource frameworks*." In addition, our framework goes beyond one-way communication to include some consultation or dialogue with the community or some decision making by them. We considered three points within an intervention during which engagement could occur as elaborated below and in Appendix 2 pp. 5-10.

Engagement in the design of interventions: community input or feedback was sought before implementing an intervention (e.g., pilot, needs assessment, formative evaluation, and outreach).

Engagement in implementation autonomy of interventions: community was utilized in intervention implementation as health care workers, facilitators, or problem solvers and only if they had some opportunity to affect or influence its implementation.

Engagement as the intervention (engagement is embedded): a serious attempt was made to gain community buy-in for activities or new cadres of community-based structures were established (e.g., village health committees or community health volunteers).

For peer review only - http://bmjopen.bmj.com/site/about/guidelines.xhtml

Research Questions

The research questions for this review were:

1. What evidence exists regarding the effectiveness of community engagement interventions in improving routine immunisation coverage of children in LMICs?

2. Is there evidence for heterogeneous effects of community engagement strategies (i.e., does effectiveness vary by geographical region, gender or programme implementation)?

3. What factors relating to programme design, implementation, and context are associated with better or worse outcomes along the causal chain? Do these vary by the kind of community engagement?

4. What is the cost-effectiveness of different community engagement interventions in improving children routine immunisation outcomes?

Search strategy

We implemented a systematic and comprehensive search strategy, in consultation with an information specialist. In May 2019 and May 2020, we searched 17 academic databases for experimental and quasi-experimental impact evaluations of community engagement interventions considering outcomes related to routine child immunisation in LMICs (using the World Bank country income classifications to determine LMIC status at the time the intervention began). We also searched 17 additional websites for grey literature. The list of sources searched and an example set of search strings are provided in Appendix 3 pp. 11-15. We complemented this with citation tracking and contacting experts. The grey literature search was conducted by AB with support from external consultant reviewers. Given the limitations of the search functions on websites we searched for grey literature, it was not possible to use the same complex search strings used in academic databases, and search strategies were developed on a site-by-site basis.

For peer review only - http://bmjopen.bmj.com/site/about/guidelines.xhtml

Inclusion / exclusion criteria (PICOS)

Table 1: PICOS inclusion and exclusion criteria

**Characteristics**	**Inclusion criteria**
Population	Rural, peri-urban and urban populations living in low and middle-income countries.
Interventions	Interventions involving community participation.
Comparisons	A comparison group or counterfactual that does not receive the intervention or business as usual.
Outcomes	Full, partial, timely immunisation of children and other outcomes such as morbidity, mortality, etc.
Study design	To answer question 1, 2 and 4, experimental and quasi-experimental studies.<br><br>To answer question 3, qualitative studies, descriptive quantitative studies, process evaluations, project documents, formative research studies, protocols, baseline and midline and endline/final impact evaluation reports, policy briefs and website content.
Other	No inclusion restrictions by publication status or language.

The population, intervention, comparators, outcomes, and study designs (PICOS) eligible for inclusion in the study are provided in Table 1. No language, publication type or date restrictions were imposed. Because our definition of community focused on the lowest levels of health facilities, we excluded interventions targeting higher levels of the health system (e.g., state-level officials) (Appendix 4 pp. 16-27). The primary outcomes considered in this review were coverage rates for (a) full immunisation, which is typically defined as the percentage of one-year-olds who

For peer review only - http://bmjopen.bmj.com/site/about/guidelines.xhtml

have received one dose of Bacille Calmette-Guérin[i], (b) third dose of DPT or pentavalent, (c) first does of measles, or (d) the timeliness of any of these doses. Additional antigen-specific immunisation coverage outcomes and secondary outcomes reflecting upstream conditions (e.g., attitudes about vaccination and access to immunisation services) and downstream effects (e.g., morbidity and mortality) of the primary outcomes were also included. Official health records and parent recall were considered acceptable sources of measures of immunisation coverage. The former was used when both measures were reported separately.

This review includes experimental and quasi-experimental studies that estimate the causal impact of an intervention, as compared to usual practice, by establishing a counterfactual. Specifically, studies with the following evaluation designs are included: randomised controlled trials, regression discontinuity designs, instrumental variables estimation, statistical matching (e.g., propensity score matching), difference-in-differences (or any mathematical equivalent), fixed effects estimation, and interrupted time series. We excluded studies for which the reported quantitative data could not be meaningfully converted to an effect size. In cases of relevant missing or incomplete data, we contacted study authors to obtain the required information. If we were unable to obtain the necessary data, we reported the characteristics of the study but did not include these studies in the meta-analysis. We conducted additional searches for economic and qualitative evidence on the included impact evaluations (Appendix 5 pp. 28-36).

Screening

At both the title and abstract and full-text screening stages, all papers were double screened by research consultants and supervised by MJ, ME and AB. Reconciliation meetings were held to resolve disagreements and MJ and ME made final decisions on unresolved cases. The same reviewers manually searched for qualitative papers and project documents on Google Scholar and websites of implementing organizations and screened the papers for inclusion as they were identified.

Data analysis

---

[i] In some countries, other vaccinations such as those for JE encephalitis and yellow fever are administered to children as a part of the routine immunisation schedule. In those contexts, we went by the definition of full immunisation mentioned in the impact evaluation study.

For peer review only - http://bmjopen.bmj.com/site/about/guidelines.xhtml

Studies were coded for their engagement type by two reviewers (MJ and AB) who independently reviewed the intervention description and coded these against the definitions provided above. If studies allowed for engagement at several stages of the intervention, they could be coded as having more than one engagement type. We used Microsoft Excel to extract descriptive information and effect sizes from included studies using double coding. Coders reconciled their answers and a study author made final decisions in case of disagreements. For qualitative analysis, all impact evaluations and additional documentation identified in the search were coded in Nvivo. Cost data was single coded and checked by a study author (Appendix 6 pp. 37-64).

To avoid double-counting of evidence from different papers focusing on the same study, we linked these papers prior to analysis. We extracted data from the most recent publication. When data was reported over multiple time periods, we extracted data for each period. Where authors reported the same outcome using more than one analytical model, we extracted data from the authors' preferred model specification. When the preference was not specified, we used the model with the most controls. Where studies reported outcomes related to multiple treatment arms and only one comparison group, we estimated an effect size for each of the treatment arms.

To assess quantitative risk of bias, we created an adapted version of the Cochrane guidelines for assessing randomized controlled trials and non-randomised studies.[16,17] These assessments were conducted by two independent reviewers. Coders reconciled their answers and a study author made final decisions in case of disagreements. For testing the sensitivity of the results to low-quality studies, we ran each analysis with and without studies scoring high risk of bias.

We critically appraised qualitative and mixed-methods studies using an adapted version of the nine-item framework developed by the Critical Appraisal Skills Programme.[18] In addition, we carried out a sensitivity analysis in which we considered only the high-quality qualitative studies that had a risk of bias assessment score of 20 or higher, indicating low risk of bias. For cost evidence, we assessed risk of bias along six primary dimensions adapted from a combination of tools, including: Doocy and Tappis (2017);[19] Campbell Collaboration Economic Methods Policy Brief;[20] and Methods for the Economic Evaluation of Health Care Programmes[21] (Appendix 7 pp. 65-89).

For peer review only - http://bmjopen.bmj.com/site/about/guidelines.xhtml

We calculated the standardised mean difference (SMD), or Cohen's *d*, its variance and standard error for each effect, converting effects reported in other metrics as necessary, using formulae provided in Borenstein and colleagues (2009).[22] In all cases we then adjusted Cohen's *d* to Hedges' *g* as defined in Ellis (2010).[23] For studies reporting regression results, we followed the approach of Keef and Roberts (2004)[24] using the regression coefficient and the pooled standard deviation of the outcome.

The amount of heterogeneity (i.e.,τ^2) was estimated using the DerSimonian-Laird estimator.[25] The Q-test for heterogeneity[26] and the I^2 statistic[27] are reported. We complement this with an assessment of heterogeneity of effect sizes graphically using forest plots. We identified outliers using studentized residuals and Cook's distance, and where outliers were indicated, we conducted a leave-one-out analysis and report the resulting effect sizes. Whenever feasible, we conducted moderator analyses using meta-regression to investigate sources of heterogeneity.[ii] The analysis was carried out using R (version 4.0.4)[28] and the **metafor** package (version 2.4.0).[29] All analyses used a random effects model because we did not reasonably expect the included studies to be functionally identical and the goal was to generalize to the larger population.[30]

Qualitative analysis followed a mix of inductive and deductive coding approaches to identify themes related to barriers and facilitators, reasons for intervention success or failure, and uptake and fidelity challenges. An initial set of themes was developed based on familiarity with the literature. However, as new topics were identified, new themes were added. Themes were also disaggregated if it became clear they were too broad. Research consultants conducted coding with oversight from CL and AB.

Patient and public involvement in research

There was no patient or public involvement in this research.

Ethics statement

[ii] All but two moderators were chosen *a priori.* Baseline coverage and vaccine hesitancy were added after feedback from an initial peer review from the Campbell Collaboration (co-publisher of this work).

For peer review only - http://bmjopen.bmj.com/site/about/guidelines.xhtml

Ethical consent from an Ethics Committee or Institutional Board was not required as this study does not involve human subjects.

Results

Our search identified over 43,000 records, which were reduced to 29,481 unique abstracts after de-duplication (Figure 1). After title and abstract screening, we considered 1,285 studies for full-text screening and could not locate an additional 44, published mostly before 2000. We excluded articles at full-text for not satisfying the inclusion criteria by country (129), study type (evaluation study) (304), evaluation method (213), outcome (235) and community engagement type (172). We ultimately identified 61 impact evaluations (Table 2)[31-92] that assessed the effects of community engagement interventions on outcomes related to routine child immunisation in LMICs. We identified one publication in Spanish[45], with all others in English. Five studies did not include sufficient data to calculate an effect size, thus 56 studies were included in the meta-analysis. Inter-rater reliabilities were calculated on a sample of studies, and ranged from 28% (mean effect of the intervention to 100% (e.g., country, publication year, study design). All studies were reconciled prior to analyses.

[Place figure 1 here]

For peer review only - http://bmjopen.bmj.com/site/about/guidelines.xhtml

Table 2 describing characteristics of the included studies

Author	Country	Summary of intervention	Engagement type	Study design	Duration (in months)	Outcomes
Admassie 2009[31]	Ethiopia	Formation of a cadre of community-based health extension workers and using community resources for construction of health posts.<br><br>The study evaluates intervention effectiveness and cost-effectiveness.	Multiple (EII[iii] + EAI[iv])	PSM	48	FIC, BCG, DPT3, OPV1, OPV2, OPV3, measles, morbidity
Adamu 2019[32]	Nigeria	Quality improvement programme where health workers use iterative processes to develop localised and contextually relevant plans to resolve health service delivery and demand bottlenecks.<br><br>The study evaluates intervention effectiveness.	Multiple (EID[v] + EII)	ITS	1	Dropouts

[iii] EII: Engagement in implementation autonomy

[iv] EAI: Engagement as intervention

[v] EID: Engagement in Design

For peer review only - http://bmjopen.bmj.com/site/about/guidelines.xhtml

Alhassan 2019[33]	Ghana	Using a bottom-up approach, the intervention first recruited and trained community groups to identify service delivery gaps in healthcare facilities.<br><br>The study evaluates intervention effectiveness.	Multiple (EII + EAI)	RCT	10	FIC
Andersson 2009[34]	Pakistan	Community dialogues to address barriers to vaccination. The guidelines for the dialogue were created after consultation with the intended beneficiaries.<br><br>The study evaluates intervention effectiveness and cost-effectiveness.	Multiple (EID + EAI)	RCT	8	Knowledge about immunisation, attitude about immunisation, community norms, readiness to vaccinate, household norms & decision-making measles, DPT3
Arifeen 2009[35]	Bangladesh	Formation of a cadre of village health volunteers and enlisting support of local religious leaders to convey messages about child health.<br><br>The study evaluates intervention effectiveness.	EAI	RCT	71	Measles, mortality
Assegaai 2018[36]	South Africa	Lay community-based workers were formalised as community health workers and served as a part of the outreach teams.	EAI	DID	36	FIC, measles, morbidity

For peer review only - http://bmjopen.bmj.com/site/about/guidelines.xhtml

		The study evaluates intervention effectiveness.				
Banerjee 2010[37]	India	Provision of immunisation services and incentives to caregivers. A trusted community-based organisation was a key stakeholder in design and delivery of the intervention.<br><br>The study evaluates intervention effectiveness and cost-effectiveness.	Treatment 1: EAI<br>Treatment 2: Multiple (EID + EAI)	RCT	18	FIC, BCG, partial immunisation
Banerjee 2020[38]	India	This evaluation tested two different interventions: 1. Incentives to caregivers. The community's feedback was solicited on the kind of incentive. 2. Community influencers were identified to spread information about immunisation.<br><br>The study evaluates intervention effectiveness and cost-effectiveness.	EID<br><br>EAI	RCT	14	Knowledge about immunisation, attitude about immunisation, FIC, DPT1, DPT2, DPT3, measles
Banwat 2015[39]	Nigeria	Female members of the community whose children are fully immunised were nominated in each community to serve as peer educators.<br><br>The study evaluates intervention effectiveness.	EAI	CBA	-	FIC, knowledge about immunisation, attitude about immunisation, readiness to vaccinate

For peer review only - http://bmjopen.bmj.com/site/about/guidelines.xhtml

Biemba 2016[40]	Zambia	A national policy to create a cadre of well-trained and motivated community-based health workers.<br><br>The study evaluates intervention effectiveness.	EAI	DID	23	FIC, morbidity
Björkman 2009[41]	Uganda	Communities were involved in monitoring the quality of health services and the performance of health service providers.<br><br>The study evaluates intervention effectiveness and cost-effectiveness.	EII	RCT	0.16	FIC, BCG, OPV0, OPV3, DPT1, DPT3, measles, Partial routine immunisation, mortality
Bolam 1998[42]	Nepal	Training for community health workers and midwives which was developed in collaboration with health workers and experts.<br>The study evaluates intervention effectiveness.	EID	RCT	3	FIC
Borkum 2014[43] Carmichael 2019[44] (linked study)	India	Performance-based incentives to frontline workers. The nature of incentives was decided upon in consultation with the frontline workers.<br><br>The study evaluates intervention effectiveness and cost-effectiveness.	EID	RCT	12	Health card availability, CHW capacity, FIC, BCG, DPT1, DPT2, DPT3, OPV1, OPV2, OPV3, measles, partial immunisation, timeliness

For peer review only

Calderón-Ortiz & Mejía-Mejía 1996[45]	Mexico	Creation of a community-based cadre of volunteers to register and track children in the community for immunisation.<br>The study evaluates intervention effectiveness.	EAI	CBA	4	FIC, BCG, DPT3, OPV3, measles
Carnell 2014[46]	Ethiopia	Formation of a cadre of community health workers to mobilize the community and encourage uptake of health services.<br><br>The study evaluates intervention effectiveness and cost-effectiveness.	EAI	DID	60	DPT3, measles
Costa-Font 2017[47]	India	Establishment of the village health and sanitation committees to monitor health service provision at the community level.<br><br>The study evaluates intervention effectiveness.	EAI	IV	-	BCG, DPT1, OPV0
Demilew 2020[48]	Ethiopia	A poster/stamp system that reminded health workers of the child's immunisation status and simultaneously encouraged caregivers to immunise their children. The intervention was designed in consultation with health workers.	Multiple (EID + EAI)	RCT	17	FIC, BCG, DPT1, DPT2, DPT3, partial immunisation

For peer review only - http://bmjopen.bmj.com/site/about/guidelines.xhtml

		The study evaluates intervention effectiveness and cost-effectiveness.				
Dipeolu 2017[49]	Nigeria	Text message reminders to mothers regarding immunisation schedule. The messages were field tested with mothers to get the content right.<br>The study evaluates intervention effectiveness.	EID	DID	9	Knowledge about immunisation, attitude about immunisation, timeliness
Domek 2019[50]	Guatemala	SMS text messages to caregivers. A prior feasibility and acceptability study was conducted for the intervention.<br>The study evaluates intervention effectiveness.	EID	RCT	2	Timeliness
Engineer 2016[51]	Afghanistan	Pay-for-performance bonuses paid quarterly to health workers. The bonus amount was revised after receiving health worker feedback.<br>The study evaluates intervention effectiveness.	EID	RCT	24	Experience & satisfaction with health services, Formal HW motivation, capacity & performance, DPT3
Findley 2013[52]	Nigeria	Formation of a cadre of community volunteers to facilitate group discussions on health and track/register women/children for health services.<br><br>The study evaluates intervention effectiveness and cost-effectiveness.	EAI	DID	24	FIC

For peer review only - http://bmjopen.bmj.com/site/about/guidelines.xhtml

For peer review only

Gibson 2017[53]	Kenya	SMS reminders and monetary incentives to caregivers. A feasibility study was conducted in 2013 for this intervention.<br>The study evaluates intervention effectiveness.	EID	RCT	12	Community norms, FIC, BCG, DPT1, DPT2, DPT3, OPV0, OPV1, OPV2, measles, timeliness
Goel 2012[54]	India	A multi-component campaign which involved women groups in awareness generation to improve health service uptake.<br>The study evaluates intervention effectiveness.	EAI	DID	48	FIC
Gurley 2020[55]	India	Community members were trained to design and produce culturally appropriate, 'hyper-local' videos to promote health seeking behaviours.<br>The study evaluates intervention effectiveness and cost-effectiveness.	Multiple (EID + EAI)	RCT	11	Knowledge about immunisation, attitude about immunisation, FIC, DPT3, partial immunisation, timeliness, dropouts
Herrera-Almanza 2018[56]	Madagascar	Community-based primary health care services intervention that included the deployment of volunteer community health workers in remote areas.<br>The study evaluates intervention effectiveness.	Multiple (EII + EAI)	DID	26	Health card availability, OPV3, DPT3, measles, mortality, partial immunisation
Igarashi 2010[57]	Zambia	The GMP+ sessions were conducted by medical personnel from Public Health Centers. During these sessions, community volunteers provided some	EII	CBA	43	FIC, timeliness, attitude about immunisation, community norms

For peer review only - http://bmjopen.bmj.com/site/about/guidelines.xhtml

		operational and managerial support to ensure the effective implementation of the sessions. The study evaluates intervention effectiveness.				
Janssens 2011[58]	India	Dissemination of health promoting messages to women in the community who are encouraged to further spread the awareness. The study evaluates intervention effectiveness.	EAI	IV	56.4	DPT3, measles
Johri 2020[59]	India	Interventions, designed through formative research, to increase caregiver knowledge and adherence to childhood immunisation. The study evaluates intervention effectiveness.	EID	RCT	3	Knowledge about immunisation, Awareness of place, time, schedule for vacc., attitude about immunisation
Lee 2015[60]	Zambia	Creation of a new cadre of frontline workers from the community, called community health assistants, to provide primary health care services. The study evaluates intervention effectiveness.	EAU	RCT	3	Formal HW motivation, capacity & performance, BCG, OPV3, measles, timeliness, CHW capacity, morbidity
Mayumana 2017[61]	Tanzania	Payment-for-performance scheme for health facilities. Health workers and health facility governing committees decided the allocation of funds. The study evaluates intervention effectiveness.	EII	DID	30	Stockouts

For peer review only - http://bmjopen.bmj.com/site/about/guidelines.xhtml

Memon 2015[62]	Pakistan	Formation of community health committees to promote perinatal and new-born care. Formative research informed the intervention design.<br>The study evaluates intervention effectiveness.	Multiple (EID + EAI)	DID	16	FIC
Modi 2019[63]	India	The mHealth intervention package consisting of mobile phone-based job aids for community health workers. The intervention was piloted in 2015.<br>The study evaluates intervention effectiveness and cost-effectiveness.	EID	RCT	12	DPT3, morbidity
Mohanan 2020[64]	India	Social accountability interventions to promote community-based collective action to improve delivery of health and nutrition services to children.<br><br>The study evaluates intervention effectiveness and cost-effectiveness.	EAI	RCT	12	Experience & satisfaction with health services, attitudes about health providers, formal health worker supply, FIC, BCG, DPT3, OPV1, OPV3, IPV, measles, morbidity, mortality
More 2012[65]	India	Urban slum-dweller women's groups used community dialogues to address barriers to improving perinatal health.<br>The study evaluates intervention effectiveness.	EAI	RCT	36	Mortality

For peer review only - http://bmjopen.bmj.com/site/about/guidelines.xhtml

More 2017[66]	India	The intervention comprised multiple activities like home visits to caregivers, groups meetings, community events and other supportive services.<br><br>The study evaluates intervention effectiveness and cost-effectiveness.	EAI	RCT	24	Health card availability, attitudes about health providers, FIC, BCG, measles, partial immunisation
Morris 2004[67]	Honduras	Monetary vouchers to women in the communities and setting up of community-based committees to oversee health service quality and access.<br><br>The study evaluates intervention effectiveness and cost-effectiveness.	EAI	RCT	24	DPT1, measles
Murthy 2019[68]	India	Voice call reminders to pregnant women and caregivers. The message content was tested for appropriateness through community focused groups.<br>The study evaluates intervention effectiveness.	EID	RCT	21	Knowledge about immunisation, FIC
Nagar 2018[69]	India	A digital pendant-based health record of the child and a voice call reminder system. A formative study was conducted in 2016 and communities were consulted on the design of the pendant.<br>The study evaluates intervention effectiveness.	EID	RCT	3	Timeliness

For peer review only - http://bmjopen.bmj.com/site/about/guidelines.xhtml

Nagar 2020[70]	India	A digital pendant-based health record of the child. Health providers used a mobile application to scan the pendant to update the child's medical history. Prior formative research informed the intervention design.<br><br>The study evaluates intervention effectiveness and cost-effectiveness.	EID	RCT	20	FIC, DPT1, DPT2, DPT3
Nzioki 2017[71]	Kenya	Formation of a cadre of community health workers.<br>The study evaluates intervention effectiveness.	EAI	CBA	0	FIC
Oche 2011[72]	Nigeria	Group meetings with caregivers and dialogues with community leaders to improve uptake of routine immunization services.<br>The study evaluates intervention effectiveness.	EAI	DID	9	Knowledge about immunisation, DPT1, DPT3, dropouts
Okeke 2017[73]	Nigeria	A national scheme to create, train and deploy a cadre of midwives to serve underserved rural and remote populations in Nigeria.<br>The study evaluates intervention effectiveness.	Multiple (EII + EAI)	DID	40	BCG, DPT3, OPV3, measles, mortality
Okoli 2014[74]	Nigeria	A conditional cash transfer program to encourage uptake of health services. Community groups were consulted while deciding the cash amount.<br>The study evaluates intervention effectiveness.	EID	ITS	7 to 18	OPV0

For peer review only - http://bmjopen.bmj.com/site/about/guidelines.xhtml

Olayo 2014[75]	Kenya	Formation of a cadre of community health workers who then facilitated dialogue at the community level and supported other community-based workers.<br>The study evaluates intervention effectiveness.	Multiple (EII + EAI)	DID	24	Health card availability, DPT1, DPT3, measles
Olken 2014[76]	Indonesia	Block grants for maternal and child health that incorporated relative performance incentives were implemented in villages through creation of village-level health committees.<br><br>The study evaluates intervention effectiveness and cost-effectiveness.	Multiple (EII + EAI)	RCT	18 to 30	FIC, morbidity, mortality
Oyo-Ita 2020[77]	Nigeria	A multi-component intervention involving traditional and religious leaders for engaging communities in planning and delivery of immunisation services.<br><br>The study evaluates intervention effectiveness and cost-effectiveness.	Multiple (EII + EAI)	RCT	18	FIC, partial immunisation, Timeliness
Pramanik 2020[78]	India	Trained facilitators from local NGOs interacted with the communities to enable communities to leverage their own strengths for addressing their concerns related to child health.	EAI	RCT	13	Knowledge about immunisation, attitude about immunisation, attitudes about health providers, health card

For peer review only - http://bmjopen.bmj.com/site/about/guidelines.xhtml

		The study evaluates intervention effectiveness and cost-effectiveness.				availability, FIC, DPT1, DPT2, DPT3, timeliness, dropouts
Rahman 2008[79]	Pakistan	Mental health support program with counselling sessions for pregnant and post-partum women. Prior intervention pilots informed the intervention design. The study evaluates intervention effectiveness.	EID	RCT	11	FIC, morbidity
Rahman 2016[80]	Bangladesh	New cadre of community health workers delivered essential maternal, neonatal, and child healthcare and nutrition services. The study evaluates intervention effectiveness.	EAI	DID	48	FIC, morbidity
Rao 2014[81]	India	Creation of a cadre of community health workers to improve basic health outcomes through community engagement. The study evaluates intervention effectiveness.	EAI	DID	60	BCG, DPT3, OPV3, measles, FIC, partial immunisation, supply of CHWs
Robertson 2013[82]	Zimbabwe	Use of cash transfers for behaviour change. Local NGO and community leaders were involved in beneficiary targeting and compliance monitoring. The intervention was also tested for feasibility during a prior study. The study evaluates intervention effectiveness.	Multiple (EID + EII)	RCT	12	FIC, community norms, OPV0
Roy 2008[83]	Bangladesh	Rural maintenance programme recruited and trained women for road maintenance, health awareness,	EAI	DID	11	FIC, BCG, DPT1, DPT2, DPT3, OPV0, OPV1,

For peer review only - http://bmjopen.bmj.com/site/about/guidelines.xhtml

		numeracy, human rights, gender equity, health and nutrition, and business management.				OPV2, OPV3, measles, partial immunisation
Saggurti 2018[84]	India	Formation of health-focused self-help groups with women of reproductive age coming from the most marginalized communities.<br><br>The study evaluates intervention effectiveness and cost-effectiveness.	EAI	DID	2	Timeliness
Sankar 2013[85]	India	Formation of committees with representatives of the community, local government and service providers to ensure better convergence and coordination of service delivery.<br>The study evaluates intervention effectiveness.	Multiple (EII +EAI)	DID	30	FIC, BCG, DPT1, DPT2, DPT3, OPV0, OPV1, OPV2, OPV3, measles, partial immunisation, timeliness
Seth 2018[86]	India	The study evaluated two different interventions: role of compliance-linked incentives versus text messaging to improve childhood immunisations. Incentive amount was determined after input was received from the local investigators as well as the community workers.<br><br>The study evaluates intervention effectiveness and cost-effectiveness.	EID	RCT	9.7	Partial immunisation, timeliness, attitude about immunisation, attitudes about health providers

For peer review only - http://bmjopen.bmj.com/site/about/guidelines.xhtml

Shukla 2018[87]	Afghanistan	Community representatives along with health officials identify the health needs of the communities and communicate those to the service providers.<br>The study evaluates intervention effectiveness.	EII	DID	6	Supply of CHWs, DPT3
Siddiqi 2020[88]	Pakistan	Visual reminders to caregivers in the form of wearable bracelets for the child. The bracelets were designed in consultation with the caregivers.<br>The study evaluates intervention effectiveness.	EID	RCT	12	DPT3, measles
Tandon 1988[89]	India	Enlisting community-based volunteers to motivate and encourage family members to utilise maternal and child health services.<br>The study evaluates intervention effectiveness.	EAI	CBA	120	FIC, BCG, DPT2, DPT3, OPV2
USAID 2008[90]	Ethiopia	Creation of a cadre of community health promoters to carry out behaviour change communication activities in the communities.<br>The study evaluates intervention effectiveness.	EAI	CBA	48	FIC, BCG, DPT1, DPT3, OPV3, morbidity, health card availability, measles, dropouts
Webster 2019[91]	Uganda	Community-based outreach and follow-up with caregivers to improve immunisation uptake and reduce defaulters.<br><br>The study evaluates intervention effectiveness and cost-effectiveness.	EII	RCT	12	Health card availability, BCG, DPT1, DPT2, DPT3, OPV0, OPV1, OPV2, OPV3, IPV, measles, partial immunisation, timeliness, attitude about

For peer review only

						immunisation, dropouts, morbidity, mortality
Younes 2014[92]	Bangladesh	The intervention involved 162 women's groups who used participatory approaches to discuss maternal and neonatal health issues.<br>The study evaluates intervention effectiveness.	EAI	DID	20	FIC, morbidity

For peer review only - http://bmjopen.bmj.com/site/about/guidelines.xhtml

Risk of Bias

Of the 31 included studies with experimental designs, only two had a low risk of bias, six had some concerns and 23 had a high risk of bias. Of the 30 included quasi-experimental studies, only two were assessed as low risk of bias, one as some concerns and 27 as high risk of bias. Biases arising from outcome measurement and deviations from intended interventions were the most common across both the study designs.

Although only five qualitative studies scored strong on all key elements, most studies received strong scores on most key elements and had quality appraisal scores greater than 20, indicating low risk of bias. The most common elements found to be missing were sample characteristics and analytic methods. The quality of the cost evidence in the 22 studies that included such evidence was mixed (further information and visualisations for the risk of bias appraisals can be found in Appendix 8 pp. 90-97).

Community Engagement Interventions

Full Immunisation

A total of $k = 28$ studies examined the effect of community engagement interventions on full childhood immunisation and found $\hat{\mu} = 0.14$ ([95% CI: 0.06 to 0.23], $z = 3.28$, $p = .001$), indicating a small but significant benefit for the treated group of .14 standard deviation units (Figure 2). The rank correlation test indicated funnel plot asymmetry ($p = 0.03$) but the regression test did not ($p = 0.57$; see Supplementary Figure 7 in Appendix 9 pp 96). The true effects appear to be heterogeneous ($I^2 = 94.5\%$, $\tau = 0.21$). Outlier analyses revealed that Banerjee (2010) may be a potential outlier, and sensitivity analyses removing Banerjee (2010) reduced the overall average effect ($\mu = 0.08$ [95% CI: 0.04 to 0.12]), but it was still positive and significant ($z = 4.12$, $p < .001$).[vi]

Sensitivity analyses were also conducted to examine the robustness of the results to the exclusion of low-quality studies. When studies assessed as high risk of bias were removed, the resulting effect was slightly larger and still statistically significant ($\hat{\mu} = 0.18$ [95% CI: 0.08 to 0.27]), $k =$

[vi] For all other outcomes, outlier analyses will be presented in the Appendices.

For peer review only - http://bmjopen.bmj.com/site/about/guidelines.xhtml

$4, z = 3.67,\ p < 0.001$). We examined several potential sources of heterogeneity, including exposure to the intervention, evaluation period, study design, year, geographical region, data source, whether the intervention was implemented by a government agency (either alone or in combination with another agency), whether new cadres of health workers were established, presence of vaccine hesitancy, and baseline vaccine coverage rates. There were no significant moderators in the context of this model (see Appendix 9, Supplementary Table 3 pp 122-128).

[Place figure 2 here]

DPT 3

A total of $k = 22$ studies examined the effect on DPT3 vaccination coverage and found a small but significant benefit to the treated group compared to the untreated group ($\hat{\mu} = 0.10$ [95% CI: 0.06 to 0.14], $z = 4.75$, $p < 0.001$; Figure 3). The true outcomes appear to be heterogeneous (I^2 = 76.8%, $\tau = 0.08$). When low quality studies were removed, the average effect increased slightly ($\hat{\mu} = 0.11$ [95% CI: 0.05 to 0.17], $k = 4$), and was still statistically significant ($z = 3.70$, $p < 0.001$;see Appendix 9, Supplementary Figure 10, p. 98). Publication year was a significant source of heterogeneity; each additional year reduced the size of the effect by .014 standard deviation units (see Appendix 9, Supplementary Table 3 pp 122-128).

[Place figure 3 here]

Measles

A total of $k = 20$ studies examined the effect on measles vaccination coverage and found a very small but significant benefit for the treated group compared to the untreated group ($\hat{\mu} = 0.07$ ([95% CI: 0.03 to 0.11], $z = 3.22$, $p < 0.01$; *see Figure 4*). When low quality studies were removed, the average effect increased ($\hat{\mu} = 0.09$, k = 6, [95% CI: 0.03 to 0.15]) and was still statistically significant ($z = 2.98$ $p = 0.003$; see Appendix 9, Supplementary Figure 11, pp. 99). The true outcomes appear to be heterogeneous (I^2 = 73.6%, $\tau = 0.07$). None of the moderators were significant sources of heterogeneity (see Appendix 9, Supplementary Table 3 pp 122-128).

[Place figure 4 here]

Vaccination Timeliness

For peer review only - http://bmjopen.bmj.com/site/about/guidelines.xhtml

We found a small but significant effect on all three timeliness outcomes: full immunisation timeliness ($\hat{\mu} = 0.15$ [95% CI: 0.07 to 0.24], $z = 3.41$, $p < 0.001$); DPT3 timeliness ($\hat{\mu} = 0.09$ [95% CI: 0.03 to 0.14], $z = 3.00$, $p < 0.01$); and measles timeliness ($\hat{\mu} = 0.23$ [95% CI: 0.14 to 0.32], $z = 5.06$, $p < 0.001$). For all timeliness outcomes, tests of heterogeneity were not significant (p > .05). For full immunisation and measles timeliness outcomes, the sensitivity analysis could not be conducted due to an inadequate number of studies. For DPT3, the average effect increased but became non-significant when low quality studies were removed. (See Appendix 9, pp.100-103 for detailed analysis).

Subgroups of community engagement interventions

Studies that used engagement as the intervention had a significant positive effect on full childhood immunisation, DPT3 vaccination and measles vaccination, but evidence was insufficient to synthesize measures of vaccination timeliness (Table 3).

When studies used community engagement in the design, there was a significant positive effect on full childhood immunisation and measles vaccination but not on DPT3 vaccination. Additionally, there was a positive significant effect on timeliness of full childhood immunisation and DPT3 vaccination. No studies using engagement in the design reported on timeliness of measles vaccinations.

For engagement in implementation autonomy, the analysis is based on a limited number of studies and we found no significant effect on either coverage or timeliness outcomes. There were no studies reporting on the timeliness of measles vaccination.

Finally, some studies combined multiple engagement types in their interventions. These interventions had a significant effect on DPT3 vaccination but not on measles vaccination or full childhood vaccination. Evidence was insufficient to synthesise measures of vaccination timeliness.

Secondary outcomes analyses and supplementary material can be found in Appendices 10-13, pp 131-179.

Table 3. Overview of the primary outcomes by engagement type

For peer review only - http://bmjopen.bmj.com/site/about/guidelines.xhtml

	**Random effects summary estimate for all interventions**	**Engagement as the intervention**	**Engagement in the design**	**Engagement in the implementation autonomy**	**Multiple engagement types**
	g, [95% CI], I^2, (k)	g, [95% CI], I^2, (k)	g, [95% CI], I^2, (k)	g, [95% CI], I^2, (k)	g, [95% CI], I^2, (k)
**Full immunisation**	**0.14**, [0.06, 0.23],** 94.46, (28)	**0.08**, [0.03, 0.13],** 70.00, (12)	**010*, [0.02, 0.19],** 23.83, (5)	0.23, [-0.001, 0.47], 73.07, (2)	0.22, [-0.12, 0.56], 97.94, (9)
**Measles**	**0.07**, [0.03, 0.11],** 73.64, (20)	**0.10***, [0.05, 0.15],** 60.29, (10)	**0.11*, [0.02, 0.21],** 0.00, (2)	0.03, [-0.09, 0.15], 54.84, (2)	0.03, [-0.10, 0.16], 86.76, (6)
**DPT3**	**0.10***, [0.06, 0.14],** 76.78, (22)	**0.09**, [0.03, 0.15],** 73.17, (6)	0.04, [-0.01, 0.08], 0.00, (6)	0.11, [-0.05, 0.28], 80.12, (3)	**0.20**, [0.06, 0.34],** 87.93, (7)
**Timeliness (DTP3)**	**0.09**, [0.03, 0.14],** 0.00, (7)	N/A	**0.12**, [0.03, 0.21],** 0.00, (4)	0.04, [-0.06, 0.13], 0.00, (2)	N/A
**Timeliness (Measles)**	**0.23***, [0.14, 0.32],** 0.00, (3)	N/A	N/A	N/A	N/A
**Timeliness (full immunisations)**	**0.15***, [0.07, 0.24],** 9.66 (5)	N/A	**0.15*, [0.004, 0.29], 7.202, (2)**	0.38, [-0.28, 1.03], 0.00, (2)	N/A

***** $p < .05$, ** $p < .01$, *** $p < .001$,**

Qualitative findings

For peer review only - http://bmjopen.bmj.com/site/about/guidelines.xhtml

Programme design characteristics were associated with intervention success or failure across all engagement types. Certain aspects of community engagement itself, such as conducting stakeholder consultations, holding community dialogues or involving community leaders were associated with better immunisation outcomes. Non-engagement intervention design features also affected intervention success. These design features include incentives given to caregivers and leadership and supportive supervision, which improved overall health service delivery and health worker performance. Among the studies that attributed intervention failure to programme design, inadequate duration, frequency or exposure to the intervention were the most notable themes.

The importance of accounting for *contextual* barriers to or facilitators of immunisation emerged consistently. Limited availability of services, especially insufficient staff and vaccine supply, were dominant barriers to immunisation, affecting outcomes in the early portion of the causal chain. Other common barriers to immunisation included practical barriers faced by caregivers such as costs, largely indirect, and logistics (wait time and language barriers) or distance. There was more variation in barriers related to social norms, fear, and an understanding of the importance of immunisation by type of engagement. Poor quality of services, including uninviting attitudes of health workers, posed a barrier to immunisation in communities that received engagement as the intervention or were engaged in the design of the intervention.

However, we also found that certain contextual factors could become facilitators of immunisation outcomes, provided an intervention has adequately situated itself to leverage them. Across all engagement types, studies associated caregivers' awareness and perception of the benefits of vaccination with improved immunisation outcomes. Similarly, availability of health infrastructure and good quality of services were also associated with improved intervention uptake and its impact.

Implementation failures, such as low fidelity, were a common reason for intervention failure. Across all engagement types, interventions did not properly account for existing implementation constraints and practicalities on the ground and were forced to change their implementation plans. Many of these issues were encountered due to uncontrollable factors or invalid theory of change assumptions. For instance, programme design may not have accounted for the unavailability of intended participants due to competing priorities, thereby potentially invalidating the assumption of beneficiary exposure in the casual pathway. Administrative challenges were cited consistently

For peer review only - http://bmjopen.bmj.com/site/about/guidelines.xhtml

though their nature varied across engagement types ranging from technical limitations (such as limited cellphone service) to political constraints to insufficient staffing levels.

Results were broadly consistent when only qualitative studies with a quality appraisal score greater than 20 were considered. The full qualitative synthesis and sensitivity analysis is available in appendix 14 pp. 180-228.

Cost effectiveness findings

Among the 14 studies for which we could calculate cost-effectiveness, we found that the median intervention cost per treated child per vaccine dose (excluding the cost of vaccines) to increase absolute immunisation coverage by one percent was USD 3.68 (all costs are reported in 2019 US dollars) and the average cost was USD 46.60. There were three outlier observations that drove up this average cost and without them cost per vaccine dose to increase absolute immunisation coverage by one percent averaged USD 3.97 (Appendix 15 pp. 229-237).

Discussion

Principal findings

We found that community engagement interventions had a small, but significant, positive effect on all primary immunisation outcomes related to coverage and their timeliness. We also found that certain features of interventions may contribute to their success. These include (a) appropriate intervention design, including building in community engagement features; (b) addressing common contextual barriers of immunisation and leveraging facilitators; and (c) accounting for existing implementation constraints and practicalities on the ground. The median intervention cost per treated child per vaccine dose (excluding the cost of vaccines) to increase absolute immunisation coverage by one percent was USD 3.68.

Among the four types of community engagement interventions, we found that engagement as the intervention (embedded community engagement), which involves creation of community buy-in or development of new community-based structures or cadres, had consistent positive effects on more primary vaccination coverage outcomes than the others. We also found engaging the community in the design of the intervention had a positive effect on most primary outcomes related to coverage. We found no ubiquitous patterns of heterogeneity among the primary outcomes.

For peer review only - http://bmjopen.bmj.com/site/about/guidelines.xhtml

While zero dose children were not the specific focus of this review, we can offer some insights based on our analyses of both DPT1 and BCG outcomes, which reflect access to initial dosing. Community engagement interventions did not show an effect on DPT1, but the evidence base was of low quality, with six of eight studies assessed as having a high risk of bias. There was a small but significant effect of community engagement interventions on BCG, but here again, the evidence base was of lower quality, with nine of 12 studies assessed as having a high risk of bias. In both cases, the evidence base was smaller in size than for the primary outcomes. As we find positive effects of community engagement on children returning for DPT and measles doses, it may be that barriers to vaccination, like availability of health services, for zero dose children are different and unless those are addressed, community engagement itself may not be enough.

Strengths and weaknesses

Our systematic review uses a detailed framework of community engagement interventions to assess their effectiveness for improving outcomes related to routine child immunisation in LMICs. As far as we are aware, ours is the first systematic review to do this. Sensitivity analyses excluding high risk of bias studies showed that the effect was slightly larger and still statistically significant for almost all the primary outcomes for which we had sufficient data. The effects were also uniform across geographies and baseline immunisation rates.

We drew on 61 studies for meta-analysis, comprising 31 RCTs and 30 quasi experimental studies. However, only 56 studies provided sufficient information for calculating effect sizes and thus were included in meta-analytic models. For full immunisation, DPT3 and measles coverage, we could draw upon 28, 22 and 20 studies respectively for pooled effects. However, for the timeliness of these coverage outcomes we had only 0 to 7 studies to assess the pooled effects. Thus, while for some outcomes the evidence base for drawing conclusions is adequate, for others it is limited. Among the four kinds of community engagement interventions, there was a relatively large evidence base for those with engagement as the intervention and those with multiple engagement types, while for interventions using engagement in implementation autonomy, the evidence was quite limited.

We identified additional documentation comprising qualitative studies, project reports, formative/process evaluations and observation studies for 39 of the 61 included impact evaluations. However, the crucial qualitative papers which help us gain a deeper understanding of overall intervention mechanisms of change were found for only 17 of the 61 IEs. Likewise, only

For peer review only - http://bmjopen.bmj.com/site/about/guidelines.xhtml

14 of the primary studies included in this review both estimated the intervention cost and reported it with sufficient detail for the review team to calculate the cost-effectiveness of the treatment. Low quality cost data and the unavailability of underlying cost data contributed to the small number of primary studies included in the cost-effectiveness analysis.

The quantitative evidence was mostly low quality, though the randomised studies were generally of higher quality and less likely to have confounding bias than the quasi-experimental studies. The quality of qualitative studies was generally high. The quality of the cost evidence was mixed. Despite the quality concerns about quantitative evidence, the sensitivity analysis conducted by excluding low-quality studies corroborated the overall findings. Despite a comprehensive search strategy and the inclusion of grey literature, publication bias was detected for the three primary coverage outcomes (full immunisation, DPT3, and measles). While bias correction analyses indicated an identical effect size for full immunisation and DPT3, the effect for measles was reduced when publication bias was corrected for. Timeliness outcomes had an insufficient number of studies to test for publication bias, which limits our ability to interpret heterogeneity.

Agreement and disagreement with other reviews

The findings from this review are broadly consistent with Molina et al. 2016[5] which found positive effects of community monitoring interventions on immunisation coverage. Another review by Gilmore and McAuliffe (2013)[4] examined the effectiveness of preventive interventions delivered by community health workers for maternal and child health in LMICs on essential new born care and found some evidence in its support through narrative synthesis, but found the evidence base to be insufficient to draw firm conclusions.

Limitations

There are several potential limitations to the current review: a) there were few analyses that were sufficiently powered to test for publication bias, thus we cannot rule this out in many cases; b) many of the moderator analyses were underpowered, meaning that in many cases we were unable to explore heterogeneity. This was particularly true in the context of the subgroup analyses of the four intervention types. In addition, it is likely that there is inter-dependency among moderator variables, but the current study did not allow for us to disentangle these confounds. Future studies may aim to better assess how moderators may work in tandem to affect the magnitude of change; c) even in cases where the average effect was significant, forest plots demonstrate that some of the included studies reported a small negative affect, which may have important implications when

For peer review only - http://bmjopen.bmj.com/site/about/guidelines.xhtml

making decisions related to programme design and implementation; d) we also observed very few studies which focused on sub-population groups. This is particularly problematic given the focus on LMICs, where equity is important to consider when trying to increase coverage; e) most of the community engagement interventions were in combination with other intervention components, thus we were not able to establish their unique contribution to changes in outcomes; f) inclusion of primary studies into this review was based on the description of the community engagement aspects of the intervention. We may have excluded studies that should ideally have been included because of inadequate reporting of intervention components.

Implications for policy and practice

COVID-19 has impacted routine child immunisation negatively in some countries, and community engagement interventions could be an effective way to counteract this decline. The positive effects of community engagement interventions can be expected across a variety of settings, although some engagement approaches appear to be more effective than others. Positive design features should be integrated into these interventions, including features such as holding community dialogues or involving community leaders, and non-community engagement features such as local supportive supervision and incentives to healthcare workers or caregivers. Wherever possible, binding contextual barriers to immunisation, such as weak health systems and social norms, should be accounted for in the design of interventions. Existing contextual facilitators for immunisation, such as good existing health systems or high maternal education, could be leveraged for increasing intervention impacts. Important implementation pre-conditions, such as regular internet service or sufficient staffing, should be assessed and established before the implementation or addressed through the design itself. Close monitoring of intervention implementation along with good understanding of context is important to help make necessary modifications in case of unexpected challenges, such as political instability.

Further research

For better-quality evidence and deeper mechanistic understanding, policy makers and practitioners should consider prioritising funding or commissioning research in the following areas: (a) ways of ameliorating outcome measurement bias due to self-reported immunisation coverage outcomes, as this was a principal source of bias; (b) better reporting of interventions, more rounded analysis of why the interventions worked through mixed-methods evaluations and greater focus on intermediate outcomes for improved understanding of causal mechanisms; (c) collection and reporting of high quality cost data to enable cost-effectiveness analysis, which is important for

For peer review only - http://bmjopen.bmj.com/site/about/guidelines.xhtml

decision making within budget constraints; and (d) focus on sub-group analysis, including for zero dose children, for ensuring immunisation services for the most marginalized children.

Contributors

MJ, ME and AB conceived the review and wrote the initial protocol. ME and AB did the systematic search. ME, MJ and AB screened and identified studies and MJ and AB made final decisions regarding study inclusion. SS did the statistical analysis. CL and AB did the qualitative analysis. EB synthesised the cost evidence. MJ provided critical inputs on the whole analysis, checked data, coordinated the review and had full access to all materials and results. External consultants supported the authors in search, screening, data extraction and critical appraisal of quantitative and qualitative evidence base. All authors critically reviewed and revised the manuscript and approved the final document for submission. MJ is responsible for the overall content and is the guarantor.

Funding and competing interests

This research was funded through a grant from the Bill & Melinda Gates Foundation (OPP1115129) to the International Initiative for Impact Evaluation (3ie). Through this grant, 3ie provided funding and technical assistance for seven impact evaluations of community engagement interventions for immunisation as a part of its immunisation evidence programme. This technical assistance included, but was not limited to: reviewing study designs, analysis plans, and data collection instruments; advising research teams on how to improve study components and address challenges that arise during the course of the evaluation; and supporting grantees in engaging with stakeholders to promote uptake and use of evidence generated by the evaluations.

As members of 3ie staff, authors MJ, AB and ME have all had varying levels of involvement in reviewing deliverables for these evaluations and providing research teams with technical assistance. Several procedural safeguards and transparency measures were put in place to mitigate the risk this conflict of interest imposed. First, all candidate studies, including those funded by 3ie, underwent a rigorous multi-step screening process, including review at the title, abstract, and full-text levels. To qualify for inclusion in the SR, a study was judged to meet the inclusion criteria related to study design, outcomes and population by two independent screeners who have reviewed the full text of the study. The 3ie study authors were responsible for assessing whether the studies met the inclusion criteria for community engagement because of the complexity of the framework.

For peer review only - http://bmjopen.bmj.com/site/about/guidelines.xhtml

However, these authors have no financial interest in this area and have not published any prior reviews on the topic. The remaining study authors have no conflicts of interest to declare.

Role of the funding source

The funder of the study had no role in study design, data collection, data analysis, data interpretation, or writing of the report.

Data availability statement

The impact evaluations and other documents included in this systematic review are public and available. The quantitative, qualitative and cost-related data extracted from these studies are available from the corresponding author upon request.

Acknowledgments

This research has been undertaken as a part of 3ie's immunisation evidence programme, supported by the Bill and Melinda Gates Foundation, Seattle, USA. We would like to thank Sohail Agha, Senior Program Officer, Gates Foundation, Seattle, for his continued support and engagement on the evidence programme and the review. Special thanks to Molly Abbruzzese whose guidance helped shape the scope of our evidence programme and aided the conceptualisation of this review. We thank Lisa Menning, Danielle Pedi, Jennifer (Fluder) Siler and Pedja Stocjicic for their valuable feedback on the review.

We wish to thank John Eyers, Yoav Vardy, Daniela Anda, Shradha Parsekar, Sejal Luthra, Yue Zhan, Aditi Hombali, Lalitha Vadrevu, Reva Datar, Pankhuri Jha, Beáta Berkovics, Ashton Baafi, Meital Kupfer David Atika, Himani Aggarwal, Agrima Sahore, Mansi Wadhwa, and Harini Narayanan for excellent support and research assistance. We would further like to thank Marie Gaarder, Executive Director, Birte Snilstevit, Director of Synthesis and Reviews Office and Sebastian Martinez, Director of Evaluation Office, 3ie, for their guidance on the review process.

References

1. Ozawa S, Clark S, Portnoy A, et al. Estimated economic impact of vaccinations in 73 low-and middle-income countries, 2001–2020. Bulletin of the World Health Organization. 2017;95(9):629. doi: 10.2471/BLT.16.178475.

For peer review only - http://bmjopen.bmj.com/site/about/guidelines.xhtml

2. World Health Organization. Immunization Coverage. World Health Organisation. 2019 Available from: https://www.who.int/news-room/fact-sheets/detail/immunization-coverage.
3. World health Organization. Immunization Agenda 2030: A Global Strategy to Leave No One Behind. World Health Organization, Geneva. 2020 Available from: https://www.who.int/teams/immunization-vaccines-and-biologicals/strategies/ia2030
4. Gilmore B, McAuliffe E. Effectiveness of community health workers delivering preventive interventions for maternal and child health in low- and middle-income countries: a systematic review. BMC public health 2013;13(1):1-14.
5. Molina E, Carella L, Pacheco A, Cruces G, Gasparini L. Community monitoring interventions to curb corruption and increase access and quality of service delivery in low- and middle-income countries: a systematic review. Campbell Systematic Reviews 2016;12(1):1-204.
6. Jain M, Engelbert M, Gaarder M, Bagai A, Eyers J. PROTOCOL: Use of community participation interventions to improve child immunisation in low-and middle-income countries: A systematic review and meta-analysis. Campbell Systematic Reviews. 2020;16(4). doi: 10.1002/cl2.1119.
7. The Steering Group of the Campbell Collaboration. Campbell Systematic Reviews: Policies and Guidelines. The Campbell Collaboration. 2017.
8. Hammerstrøm K, Wade A, Jørgensen A.-MK. Searching for studies: A guide to information retrieval for Campbell Systematic Reviews. Campbell Systematic Reviews 2010;Supplement 1. Available at: www.campbellcollaboration.org/lib/download/969/
9. Higgins J, Green S. Cochrane handbook for systematic reviews of interventions. (version 5.0.2, updated September 2009). The Cochrane Collaboration. 2011.
10. Shadish W, Myers D. Research design policy brief. Oslo: Campbell Collaboration 2014. Available from: http://www.campbellcollaboration.org/artman2/uploads/1/C2_Research_Design_Policy_Brief-2.pdf
11. White H. Theory-based Impact Evaluation: Principles and Practice. Working paper series No. 3. New Delhi: 3ie. 2009.
12. Snilstveit B. Systematic reviews: from 'bare bones' reviews to policy relevance. Journal of development effectiveness. 2012;4(3):388-408. doi: 10.1080/19439342.2012.709875.

For peer review only - http://bmjopen.bmj.com/site/about/guidelines.xhtml

13. Waddington H, Aloe AM, Becker BJ, et al. Quasi-experimental study designs series—paper 6: risk of bias assessment. Journal of Clinical Epidemiology. 2017;89:43-52. doi: 10.1016/j.jclinepi.2017.02.015.

14. World Health Organization. Community engagement: a health promotion guide for universal health coverage in the hands of the people. Geneva: World Health Organization; 2020. Licence: CC BY-NC-SA 3.0 IGO. Available from https://www.who.int/publications/i/item/9789240010529

15. Brunton G, Thomas J, O'Mara-Eves A, Jamal F, Oliver S, Kavanagh J. Narratives of community engagement: a systematic review-derived conceptual framework for public health interventions. BMC Public Health 2017;17(1):944. doi: 10.1186/s12889-017-4958-4.

16. Sterne JAC, Savović J, Page MJ, et al. RoB 2: a revised tool for assessing risk of bias in randomised trials. BMJ 2019;366:l4898. doi: 10.1136/bmj.l4898.

17. Sterne JAC, Hernán MA, Reeves BC, et al. ROBINS-I: a tool for assessing risk of bias in non-randomized studies of interventions. BMJ 2016; 355; i4919; doi: 10.1136/bmj.i4919.

18. Critical Appraisal Skills Programme. CASP Qualitative Checklist. 2018. Available at: https://casp-uk.b-cdn.net/wp-content/uploads/2018/03/CASP-Qualitative-Checklist-2018_fillable_form.pdf.

19. Doocy S, Tappis H. Cash-based approaches in humanitarian emergencies: a systematic review. Campbell Systematic Reviews. 2017;13(1):1-200. doi: 10.4073/csr.2017.17.

20. Shemilt I, Mugford M, Byford S, et al. The Campbell Collaboration Economics Methods Policy Brief. Oslo: Campbell Collaboration. 2008. http://www.campbellcollaboration.org/artman2/uploads/1/Economic_Methods_Policy_Brief.pdf

21. Drummond MF, Sculpher MJ, Claxton K, Stoddart GL, Torrance GW. Methods for the economic evaluation of health care programmes. Oxford: Oxford university press, 2015.

22. Borenstein M, Hedges LV, Higgins JPT, Rothstein H. Introduction to Meta Analysis (Statistics in Practice). Chichester: John Wiley & Sons, 2009.

23. Ellis PD. The essential guide to effect sizes: Statistical power, meta-analysis, and the interpretation of research results. Cambridge: Cambridge University Press, 2010.

24. Keef SP, Roberts LA. The meta-analysis of partial effect sizes. British Journal of Mathematical and Statistical Psychology. 2004;57(1):97-129. doi: 10.1348/000711004849303.

For peer review only - http://bmjopen.bmj.com/site/about/guidelines.xhtml

25. DerSimonian R, Laird N. Meta-analysis in clinical trials. Controlled clinical trials 1986;7(3):177-88. doi:10.1016/0197-2456(86)90046-2

26. Cochran WG. The combination of estimates from different experiments. Biometrics 1954;10:101-129. doi: 10.2307/3001666.

27. Higgins JPT, Thompson SG. 2002. Quantifying heterogeneity in a meta-analysis. Statistics in medicine 2002;21(11):1539-58. doi:10.1002/sim.1186.

28. R Core Team. R: A language and environment for statistical computing. Vienna, Austria: R Foundation for Statistical Computing, 2018. Available from https://www.R-project.org/

29. Viechtbauer W. Conducting meta-analyses in r with the metafor package. Journal of Statistical Software 2010;36(3):1–48. doi: 10.18637/jss.v036.i03

30. Borenstein M, Hedges LV, Higgins JP, Rothstein HR. Introduction to meta-analysis. 2nd ed. John Wiley & Sons; 2021.

31. Admassie A, Abebaw D, Woldemichael AD. Impact evaluation of the Ethiopian health services extension programme. Journal of Development Effectiveness. 2009;1(4):430-49. doi: 10.1080/19439340903375724.

32. Adamu AA, Uthman OA, Gadanya MA, Wiysonge CS. Implementation and evaluation of a collaborative quality improvement program to improve immunization rate and reduce missed opportunities for vaccination in primary health-care facilities: a time series study in Kano, Nigeria. Expert review of vaccines. 2019;18(9):969-91. doi: 10.1080/14760584.2019.1647782

33. Alhassan RK, Nketiah-Amponsah E, Ayanore MA, Afaya A, Salia SM, Milipaak J, Ansah EK, Owusu-Agyei S. Impact of a bottom-up community engagement intervention on maternal and child health services utilization in Ghana: a cluster randomised trial. BMC public health. 2019;19(1):1-1. doi: 10.1186/s12889-019-7180-8.

34. Andersson N, Cockcroft A, Ansari NM, et al. Evidence-based discussion increases childhood vaccination uptake: a randomised cluster controlled trial of knowledge translation in Pakistan. BMC International Health and Human Rights. 2009;9(1):1-9. doi: 10.1186/1472-698X-9-S1-S8

35. Arifeen SE, Hoque DE, Akter T, et al. Effect of the Integrated Management of Childhood Illness strategy on childhood mortality and nutrition in a rural area in Bangladesh: a cluster randomised trial. The Lancet. 2009;374(9687):393-403. doi: 10.1016/S0140-6736(09)60828-X.

For peer review only - http://bmjopen.bmj.com/site/about/guidelines.xhtml

36. Assegaai T, Reagon G, Schneider H. Evaluating the effect of ward-based outreach teams on primary healthcare performance in North West Province, South Africa: A plausibility design using routine data. South African Medical Journal. 2018;108(4):329-35. doi: 10.7196/SAMJ.2017.v108i4.12755.

37. Banerjee AV, Duflo E, Glennerster R, Kothari D. Improving immunisation coverage in rural India: clustered randomised controlled evaluation of immunisation campaigns with and without incentives. The BMJ. 2010;340:c2220. doi: 10.1136/bmj.c2220.

38. Banerjee A, Chandrasekhar A, Duflo E, et al. Evaluating the Impact of Interventions to Improve Full Immunisation Rates in Haryana, India. Impact Evaluation Report 126. New Delhi: International Initiative for Impact Evaluation (3ie) 2020. https://doi.org/10.23846/TW10IE126.

39. Banwat ME, Lar LA, Abok IA, Yiltok ES. Effect of peer education on knowledge, attitude and completeness of childhood routine immunization in a rural community of Plateau State. Research Journal of Health Sciences. 2015;3(4):264-74.

40. Biemba G, Yeboah-Antwi K, Vosburg KB, et al. Effect of deploying community health assistants on appropriate treatment for diarrhoea, malaria and pneumonia: quasi-experimental study in two districts of Zambia. Tropical Medicine & International Health. 2016;21(8):985-94. doi: 10.1111/tmi.12730.

41. Björkman M, Svensson J. Power to the people: evidence from a randomized field experiment on community-based monitoring in Uganda. The Quarterly Journal of Economics. 2009;124(2):735-69. doi: 10.1162/qjec.2009.124.2.735

42. Bolam A, Manandhar DS, Shrestha P, Ellis M, Anthony MD. The effects of postnatal health education for mothers on infant care and family planning practices in Nepal: a randomised controlled trial. Bmj. 1998;316(7134):805-11. doi: 10.1136/bmj.316.7134.805.

43. Borkum E, Rangarajan A, Rotz D, Sridharan S, Sethi S, Manorajini M. Evaluation of the Team-Based Goals and Performance-Based Incentives (TBGI) Innovation in Bihar. Mathematica Policy Research; 2014. https://ideas.repec.org/p/mpr/mprres/d8e1097122ff47a6bf42580c82677834.html.

44. Carmichael S, Mehta K, Raheel H, et al. Effects of team-based goals and non-monetary incentives on front-line health worker performance and maternal health behaviours: a cluster

For peer review only - http://bmjopen.bmj.com/site/about/guidelines.xhtml

randomised controlled trial in Bihar, India. BMJ Global Health. 2019;4(4):e001146. doi: :10.1136/bmjgh-2018-001146

45. Calderón-Ortiz, R, Jesus M. Estrategia de contratacion permanente dentro del Programa de Vacunacion Universa. Salud Pública de México 1996;38(4):243-48. https://www.redalyc.org/articulo.oa?id=10638404
46. Carnell MA, Dougherty L, Pomeroy AM, Karim AM, Mekonnen YM, Mulligan BE. Effectiveness of scaling up the 'Three Pillars' approach to accelerating MDG 4 progress in Ethiopia. Journal of health, population, and nutrition. 2014;32(4):549.
47. Costa-Font J, Parmar D. Political agency and public healthcare: Evidence from India. WIDER Working Paper; 2016;number 6640. Munich: UNU-WIDER. https://doi.org/10.35188/UNU-WIDER/2016/179-6.
48. Demilew A, Girma M, McElwee E, Datta S, Barofsky J, Disasa T. Improving Immunisation through Supportive Feedback and Nonmonetary Incentives: Impact Evaluation in Ethiopia. Impact Evaluation Report. New Delhi: International Initiative for Impact Evaluation (3ie) 2020.
49. Dipeolu IO. Effect of Mobile-Phone Reminder Test Messages on Mothers' Knowledge and Completion of Routine Immunisation in Rural Areas of Oyo State, Nigeria. PhD Thesis, Ibadan, Nigeria: University of Ibadan 2017. https://library.adhl.africa/handle/123456789/11865.
50. Domek GJ, Contreras-Roldan IL, Bull S, et al. Text message reminders to improve infant immunization in Guatemala: A randomized clinical trial. Vaccine. 2019;37(42):6192-200. doi: 10.1016/j.vaccine.2019.08.046.
51. Engineer CY, Dale E, Agarwal A, et al. Effectiveness of a pay-for-performance intervention to improve maternal and child health services in Afghanistan: a cluster-randomized trial. International journal of epidemiology. 2016;45(2):451-9. doi: 10.1093/ije/dyv362.
52. Findley SE, Uwemedimo OT, Doctor HV, Green C, Adamu F, Afenyadu GY. Early results of an integrated maternal, newborn, and child health program, Northern Nigeria, 2009 to 2011. BMC Public Health. 2013;13(1):1-2. doi: 10.1186/1471-2458-13-1034
53. Gibson DG, Ochieng B, Kagucia EW, et al. Mobile phone-delivered reminders and incentives to improve childhood immunisation coverage and timeliness in Kenya (M-SIMU): a cluster

For peer review only - http://bmjopen.bmj.com/site/about/guidelines.xhtml

randomised controlled trial. The Lancet Global Health. 2017;5(4):e428-38. doi: 10.1016/S2214-109X(17)30072-4.

54. Goel S, Dogra V, Gupta SK, et al. Effectiveness of Muskaan Ek Abhiyan (the smile campaign) for strengthening routine immunization in Bihar, India. Indian pediatrics. 2012;49(2):103-8. doi: 10.1007/s13312-012-0023-7.
55. Gurley N, Shearer J, Srivastava Y, Mahapatra S, Desmond M. Impacts of community-led video education to increase vaccination coverage in Uttar Pradesh, India: A mixed methods randomised controlled trial. Impact Evaluation Report 125. 2020th ed. New Delhi: International Initiative for Impact Evaluation (3ie). https://doi.org/10.23846/TW10IE125.
56. Herrera-Almanza C, Rosales-Rueda MF. Reducing the Cost of Remoteness: Community-Based Health Interventions and Fertility Choices. Journal of Health Economics. 2020;73:102365. doi: 10.1016/j.jhealeco.2020.102365.
57. Igarashi K, Sasaki S, Fujino Y, et al. The impact of an immunization programme administered through the Growth Monitoring Programme Plus as an alternative way of implementing Integrated Management of Childhood Illnesses in urban-slum areas of Lusaka, Zambia. Transactions of the Royal Society of Tropical Medicine and Hygiene. 2010;104(9):577-82. doi:10.1016/j.trstmh.2010.05.008
58. Janssens W. Externalities in program evaluation: the impact of a women's empowerment program on immunization. Journal of the European Economic Association. 2011;9(6):1082-113. doi: 10.1111/j.1542-4774.2011.01041.x.
59. Johri M, Chandra D, Kone KG, et al. Social and Behavior Change Communication Interventions Delivered Face-to-Face and by a Mobile Phone to Strengthen Vaccination Uptake and Improve Child Health in Rural India: Randomized Pilot Study. JMIR mHealth and uHealth. 2020;8(9):e20356. doi: 10.2196/20356.
60. Lee SS. Three field experiments on incentives for health workers. PhD Thesis, Cambridge: Harvard University; 2015. https://dash.harvard.edu/handle/1/17467500.
61. Mayumana I, Borghi J, Anselmi L, Mamdani M, Lange S. Effects of Payment for Performance on accountability mechanisms: Evidence from Pwani, Tanzania. Social Science & Medicine. 2017;179:61-73. doi: 10.1016/j.socscimed.2017.02.022.
62. Memon ZA, Khan GN, Soofi SB, Baig IY, Bhutta ZA. Impact of a community-based perinatal and newborn preventive care package on perinatal and neonatal mortality in a remote

For peer review only - http://bmjopen.bmj.com/site/about/guidelines.xhtml

mountainous district in Northern Pakistan. BMC pregnancy and childbirth. 2015;15(1):1-9. doi: 10.1186/s12884-015-0538-8.

63. Modi D, Dholakia N, Gopalan R, et al. mHealth intervention "ImTeCHO" to improve delivery of maternal, neonatal, and child care services—A cluster-randomized trial in tribal areas of Gujarat, India. PLoS medicine. 2019;16(10):e1002939. doi: 10.1371/journal.pmed.1002939.

64. Mohanan M, Rajan VS, Swanson K, Thirumurthy H. Information and Facilitation Interventions for Accountability in Health and Nutrition: Evidence from a Randomized Trial in India. Economic Research Initiatives at Duke (ERID) Working Paper. Durham: Duke University; 2020(295). https://www.ssrn.com/abstract=3544786.

65. More NS, Bapat U, Das S, et al. Community mobilization in Mumbai slums to improve perinatal care and outcomes: a cluster randomized controlled trial. PLoS medicine. 2012;9(7):e1001257. doi: 10.1371/journal.pmed.1001257.

66. More NS, Das S, Bapat U, et al. Community resource centres to improve the health of women and children in informal settlements in Mumbai: a cluster-randomised, controlled trial. The Lancet Global Health. 2017;5(3):e335-49. doi: 10.1016/S2214-109X(16)30363-1.

67. Morris SS, Flores R, Olinto P, Medina JM. Monetary incentives in primary health care and effects on use and coverage of preventive health care interventions in rural Honduras: cluster randomised trial. The Lancet. 2004;364(9450):2030-7 doi: 10.1016/S0140-6736(04)17515-6.

68. Murthy N, Chandrasekharan S, Prakash MP, et al. The Impact of an mHealth voice message service (mMitra) on infant care knowledge, and practices among low-income women in India: findings from a pseudo-randomized controlled trial. Maternal and child health journal. 2019;23(12):1658-69. doi: 10.1007/s10995-019-02805-5.

69. Nagar R, Venkat P, Stone LD, Engel KA, Sadda P, Shahnawaz M. A cluster randomized trial to determine the effectiveness of a novel, digital pendant and voice reminder platform on increasing infant immunization adherence in rural Udaipur, India. Vaccine. 2018;36(44):6567-77. doi: 10.1016/j.vaccine.2017.11.023.

70. Nagar R, Ambiya MS, Singh P, et al. A Mixed-Methods Evaluation to Determine the Effects of a Novel MHealth Platform for Maternal Child Health Tracking in Rural Udaipur, India." 3ie Series Report 129. New Delhi: International Initiative for Impact Evaluation (3ie); 2020. https://developmentevidence.3ieimpact.org/search-result-details/impact-evaluation-

For peer review only - http://bmjopen.bmj.com/site/about/guidelines.xhtml

repository/a-mixed-methods-evaluation-to-determine-the-effects-of-a-novel-mhealth-platform-for-maternal-child-health-tracking-in-rural-udaipur-india/8984.

71. Nzioki JM, Ouma J, Ombaka JH, Onyango RO. Community health worker interventions are key to optimal infant immunization coverage, evidence from a pretest-posttest experiment in Mwingi, Kenya. Pan African Medical Journal. 2017;28(1). doi: 10.11604/pamj.2017.28.21.11255.
72. Oche MO, Umar AS, Ibrahim MT, Sabitu K. An assessment of the impact of health education on maternal knowledge and practice of childhood immunization in Kware, Sokoto State. Journal of Public Health and Epidemiology. 2011;3(10):440-7. https://academicjournals.org/article/article1379497483_Oche%20et%20al.pdf.
73. Okeke E, Glick P, Abubakar IS, et al. Better Obstetrics in Rural Nigeria: Evaluating the Midwives Service Scheme. 3ie Impact Evaluation Report 56. International Initiative for Impact Evaluation (3ie); 2017. https://www.3ieimpact.org/evidence-hub/publications/impact-evaluations/better-obstetrics-rural-nigeria-evaluating-midwives.
74. Okoli U, Morris L, Oshin A, Pate MA, Aigbe C, Muhammad A. Conditional Cash Transfer Schemes in Nigeria: Potential Gains for Maternal and Child Health Service Uptake in a National Pilot Programme. BMC Pregnancy and Childbirth 2014;14(1):408. doi: 10.1186/s12884-014-0408-9.
75. Olayo R, Wafula C, Aseyo E, Loum C, Kaseje D. A quasi-experimental assessment of the effectiveness of the Community Health Strategy on health outcomes in Kenya. BMC health services research. 2014;14(1):1-3. doi: 10.1186/1472-6963-14-S1-S3.
76. Olken BA, Onishi J, Wong S. Should aid reward performance? Evidence from a field experiment on health and education in Indonesia. American Economic Journal: Applied Economics. 2014;6(4):1-34. doi: 10.1257/app.6.4.1.
77. Oyo-Ita, A, Bosch-Capblanch X, Ross A, et al. Impacts of Engaging Communities through Traditional and Religious Leaders on Vaccination Coverage in Cross River State, Nigeria. Impact Evaluation Report 127. 2020th ed. New Delhi: International Initiative for Impact Evaluation (3ie); 2020. https://doi.org/10.23846/TW10IE127.
78. Pramanik S, Ghosh A, Goswami A, et al. Impacts of the Stimulate, Appreciate, Learn, and Transfer community engagement approach to increase immunisation coverage in Assam,

For peer review only - http://bmjopen.bmj.com/site/about/guidelines.xhtml

India, 3ie Grantee Final Report. New Delhi: International Initiative for Impact Evaluation (3ie); 2020.

79. Rahman A, Malik A, Sikander S, Roberts C, Creed F. Cognitive behaviour therapy-based intervention by community health workers for mothers with depression and their infants in rural Pakistan: a cluster-randomised controlled trial. The Lancet. 2008;372(9642):902-9. doi: 10.1016/S0140-6736(08)61400-2.

80. Rahman M, Yunus FM, Shah R, et al. A controlled before-and-after perspective on the improving maternal, neonatal, and child survival program in rural Bangladesh: an impact analysis. PLoS One. 2016;11(9):e0161647. doi: 10.1371/journal.pone.0161647.

81. Rao T. The Impact of a Community Health Worker Program on Childhood Immunization: Evidence from India's 'ASHA' Workers. Available at SSRN 2444391. 2014. http://www.ssrn.com/abstract=2444391.

82. Robertson L, Mushati P, Eaton JW, et al. Effects of unconditional and conditional cash transfers on child health and development in Zimbabwe: a cluster-randomised trial. The Lancet. 2013;381(9874):1283-92. doi: 10.1016/S0140-6736(12)62168-0.

83. Roy SK, Bilkes F, Islam K, et al. Impact of pilot project of Rural Maintenance Programme (RMP) on destitute women: CARE, Bangladesh. Food and nutrition bulletin. 2008;29(1):67-75. doi: 10.1177/156482650802900108.

84. Saggurti N, Atmavilas Y, Porwal A, et al. Effect of health intervention integration within women's self-help groups on collectivization and healthy practices around reproductive, maternal, neonatal and child health in rural India. PLoS One. 2018;13(8):e0202562. doi: 10.1371/journal.pone.0202562.

85. Sankar D. Improving Early Childhood Development through Community Mobilization and Integrated Planning for Children. Results from the Evaluation of Bachpan Program, Ratlam District, Madhya Pradesh, India. Discussion Paper Series 59. Washington, D.C.: The World Bank; 2013. http://crossasia-repository.ub.uni-heidelberg.de/3467/.

86. Seth R, Akinboyo I, Chhabra A, et al. Mobile phone incentives for childhood immunizations in rural India. Pediatrics. 2018;141(4):e20173455. doi: 10.1542/peds.2017-3455.

For peer review only - http://bmjopen.bmj.com/site/about/guidelines.xhtml

87. Shukla M. Impact of a health governance intervention on provincial health system performance in Afghanistan: a quasi-experimental study. Health systems & reform. 2018;4(3):249-66. doi: 10.1080/23288604.2018.1477536.
88. Siddiqi DA, Ali RF, Munir M, Shah MT, Khan AJ, Chandir S. Effect of vaccine reminder and tracker bracelets on routine childhood immunization coverage and timeliness in urban Pakistan (2017-18): a randomized controlled trial. BMC public health. 2020;20(1):1086. doi: 10.1186/s12889-020-09088-4.
89. Tandon BN, Sahai A. Immunization in India: contribution of Integrated Child Development Services scheme to expanded programme of immunization. Journal of tropical pediatrics. 1988;34(6):309-12. doi: 10.1093/tropej/34.6.309.
90. USAID. Essential Services for Health in Ethiopia: Final Report. Washington, DC: USAID; 2008. https://publications.jsi.com/JSIInternet/Inc/Common/_download_pub.cfm?id=10111&lid=3.
91. Webster J, Landegger J, Bruce J, et al. Impacts of IRC's Fifth Child Community Engagement Strategy to Increase Immunisation in Northern Uganda. 3ie Grantee Final Report. New Delhi: International Initiative for Impact Evaluation (3ie); 2019. https://www.3ieimpact.org/sites/default/files/2019-02/gfr-TW10.1018-IRC-Imunization-Program-Uganda.pdf.
92. Younes L, Houweling TA, Azad K, et al. The effect of participatory women's groups on infant feeding and child health knowledge, behaviour and outcomes in rural Bangladesh: a controlled before-and-after study. J Epidemiol Community Health. 2015 Apr 1;69(4):374-81. doi: 10.1136/jech-2014-204271.

Supplementary material

Supplementary material includes

1. Appendix 1: Amendments to information provided in the protocol
2. Appendix 2: Overview of community engagement framework
3. Appendix 3: Full search terms for one database.
4. Appendix 4: List of excluded impact evaluation studies
5. Appendix 5: Qualitative and cost-evidence search strategy
6. Appendix 6: Data extraction tools
7. Appendix 7: Risk of bias assessment tools

For peer review only - http://bmjopen.bmj.com/site/about/guidelines.xhtml

8. Appendix 8: Risk of bias in the included studies
9. Appendix 9: Quantitative results – all community engagement interventions
10. Appendix 10: Quantitative results – Engagement as intervention
11. Appendix 11: Quantitative results - community engagement in intervention design
12. Appendix 12: Community engagement in implementation autonomy of interventions
13. Appendix 13: Multiple engagement types
14. Appendix 14: Qualitative synthesis of results
15. Appendix 15: Synthesis of cost evidence

Figure legends

Figure 1. PRISMA flow diagram

Figure 2. Forest plot showing the observed outcomes and the estimate of the random-effects model for the impact of community engagement interventions on full childhood immunisation. Note that # (number of) Participants is specific to each effect and thus may not reflect the sample size for the full study.

Figure 3. Forest plot showing the observed outcomes and the estimate of the random-effects model for the impact of community engagement interventions on DPT3 vaccination. Note that # Participants is specific to each effect and thus may not reflect the sample size for the full study.

Figure 4. Forest plot showing the observed outcomes and the estimate of the random-effects model for the impact of community engagement interventions on measles vaccination. Note that # Participants is specific to each effect and thus may not reflect the sample size for the full study.

For peer review only - http://bmjopen.bmj.com/site/about/guidelines.xhtml

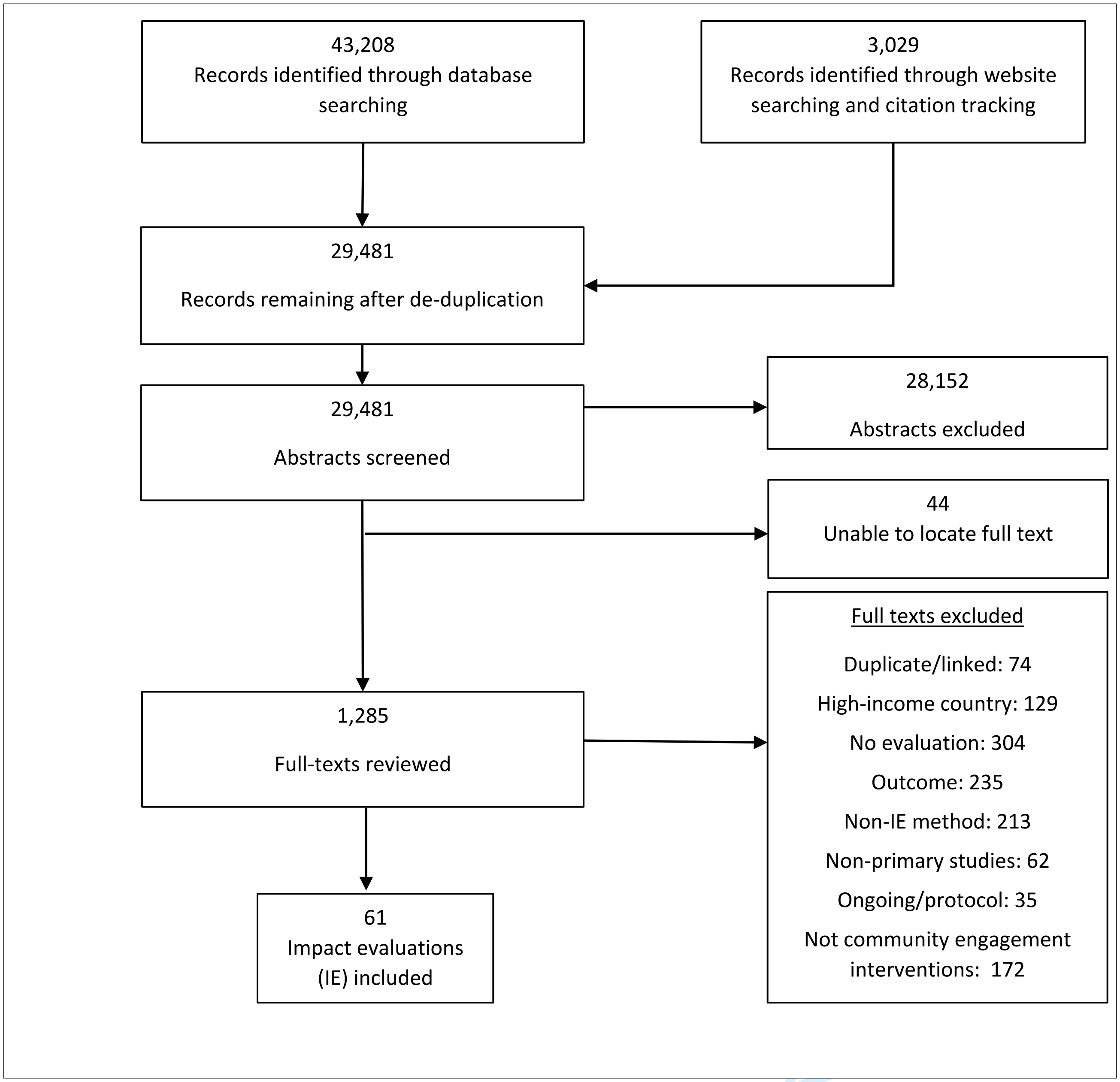

For peer review only - http://bmjopen.bmj.com/site/about/guidelines.xhtml

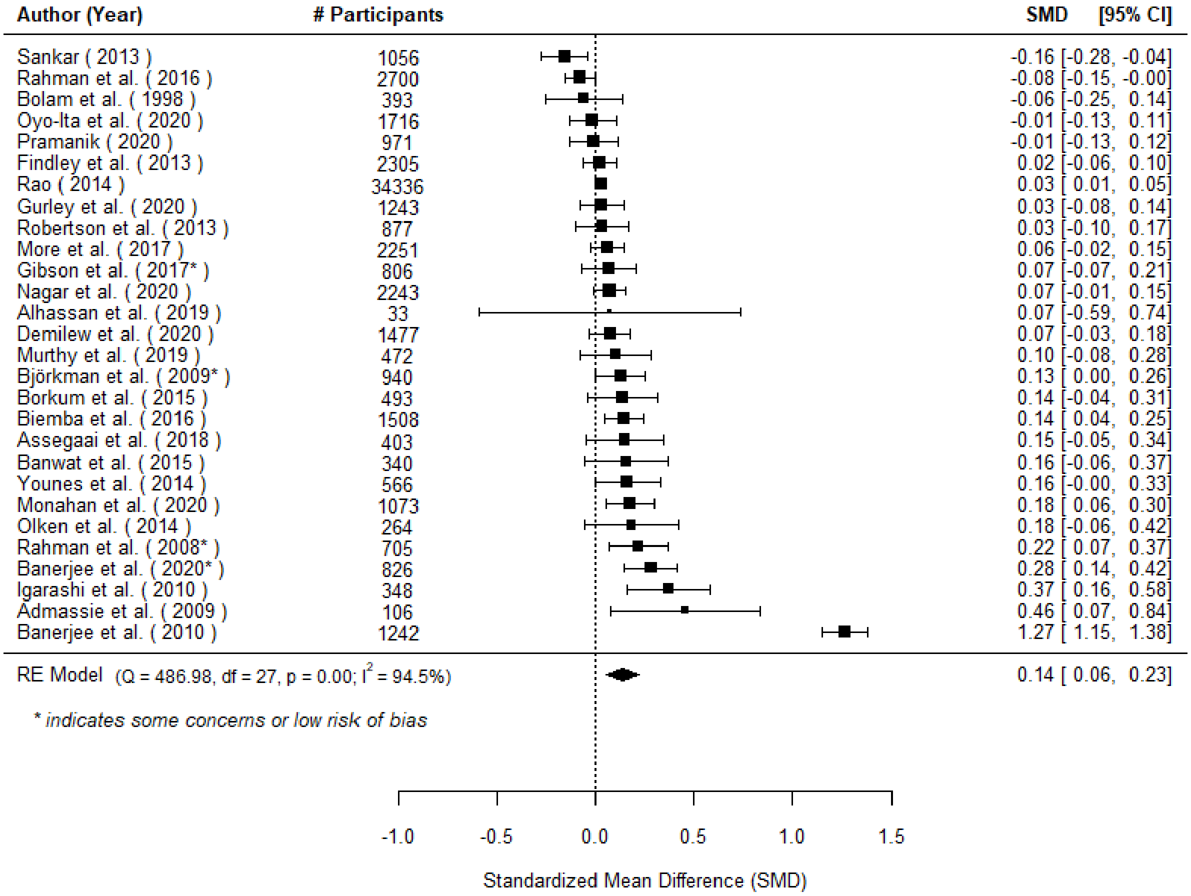
Author (Year)
Participants
SMD [95% CI]
Sankar (2013) 1056 -0.16 [-0.28, -0.04]
Rahman et al. (2016) 2700 -0.08 [-0.15, -0.00]
Bolam et al. (1998) 393 -0.06 [-0.25, 0.14]
Oyo-Ita et al. (2020) 1716 -0.01 [-0.13, 0.11]
Pramanik (2020) 971 -0.01 [-0.13, 0.12]
Findley et al. (2013) 2305 0.02 [-0.06, 0.10]
Rao (2014) 34336 0.03 [0.01, 0.05]
Gurley et al. (2020) 1243 0.03 [-0.08, 0.14]
Robertson et al. (2013) 877 0.03 [-0.10, 0.17]
More et al. (2017) 2251 0.06 [-0.02, 0.15]
Gibson et al. (2017*) 806 0.07 [-0.07, 0.21]
Nagar et al. (2020) 2243 0.07 [-0.01, 0.15]
Alhassan et al. (2019) 33 0.07 [-0.59, 0.74]
Demilew et al. (2020) 1477 0.07 [-0.03, 0.18]
Murthy et al. (2019) 472 0.10 [-0.08, 0.28]
Björkman et al. (2009*) 940 0.13 [0.00, 0.26]
Borkum et al. (2015) 493 0.14 [-0.04, 0.31]
Biemba et al. (2016) 1508 0.14 [0.04, 0.25]
Assegaai et al. (2018) 403 0.15 [-0.05, 0.34]
Banwat et al. (2015) 340 0.16 [-0.06, 0.37]
Younes et al. (2014) 566 0.16 [-0.00, 0.33]
Monahan et al. (2020) 1073 0.18 [0.06, 0.30]
Olken et al. (2014) 264 0.18 [-0.06, 0.42]
Rahman et al. (2008*) 705 0.22 [0.07, 0.37]
Banerjee et al. (2020*) 826 0.28 [0.14, 0.42]
Igarashi et al. (2010) 348 0.37 [0.16, 0.58]
Admassie et al. (2009) 106 0.46 [0.07, 0.84]
Banerjee et al. (2010) 1242 1.27 [1.15, 1.38]
RE Model (Q = 486.98, df = 27, p = 0.00; I² = 94.5%) 0.14 [0.06, 0.23]
* indicates some concerns or low risk of bias
-1.0
-0.5
0.0
0.5
1.0
1.5
Standardized Mean Difference (SMD)

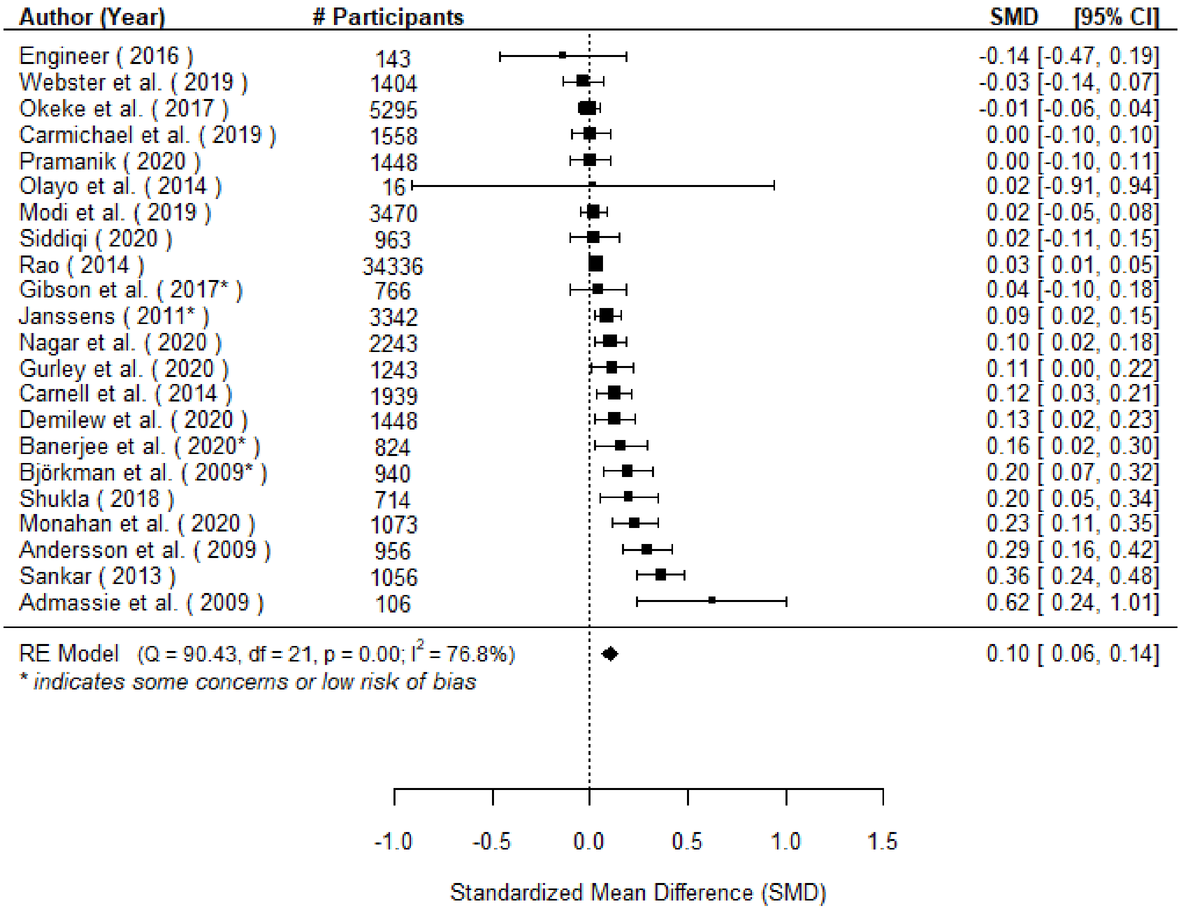
Author (Year)
Participants
SMD [95% CI]
Engineer (2016) 143 -0.14 [-0.47, 0.19]
Webster et al. (2019) 1404 -0.03 [-0.14, 0.07]
Okeke et al. (2017) 5295 -0.01 [-0.06, 0.04]
Carmichael et al. (2019) 1558 0.00 [-0.10, 0.10]
Pramanik (2020) 1448 0.00 [-0.10, 0.11]
Olayo et al. (2014) 16 0.02 [-0.91, 0.94]
Modi et al. (2019) 3470 0.02 [-0.05, 0.08]
Siddiqi (2020) 963 0.02 [-0.11, 0.15]
Rao (2014) 34336 0.03 [0.01, 0.05]
Gibson et al. (2017*) 766 0.04 [-0.10, 0.18]
Janssens (2011*) 3342 0.09 [0.02, 0.15]
Nagar et al. (2020) 2243 0.10 [0.02, 0.18]
Gurley et al. (2020) 1243 0.11 [0.00, 0.22]
Carnell et al. (2014) 1939 0.12 [0.03, 0.21]
Demilew et al. (2020) 1448 0.13 [0.02, 0.23]
Banerjee et al. (2020*) 824 0.16 [0.02, 0.30]
Björkman et al. (2009*) 940 0.20 [0.07, 0.32]
Shukla (2018) 714 0.20 [0.05, 0.34]
Monahan et al. (2020) 1073 0.23 [0.11, 0.35]
Andersson et al. (2009) 956 0.29 [0.16, 0.42]
Sankar (2013) 1056 0.36 [0.24, 0.48]
Admassie et al. (2009) 106 0.62 [0.24, 1.01]
RE Model (Q = 90.43, df = 21, p = 0.00; I2 = 76.8%) 0.10 [0.06, 0.14]
* indicates some concerns or low risk of bias
-1.0 -0.5 0.0 0.5 1.0 1.5
Standardized Mean Difference (SMD)

review only

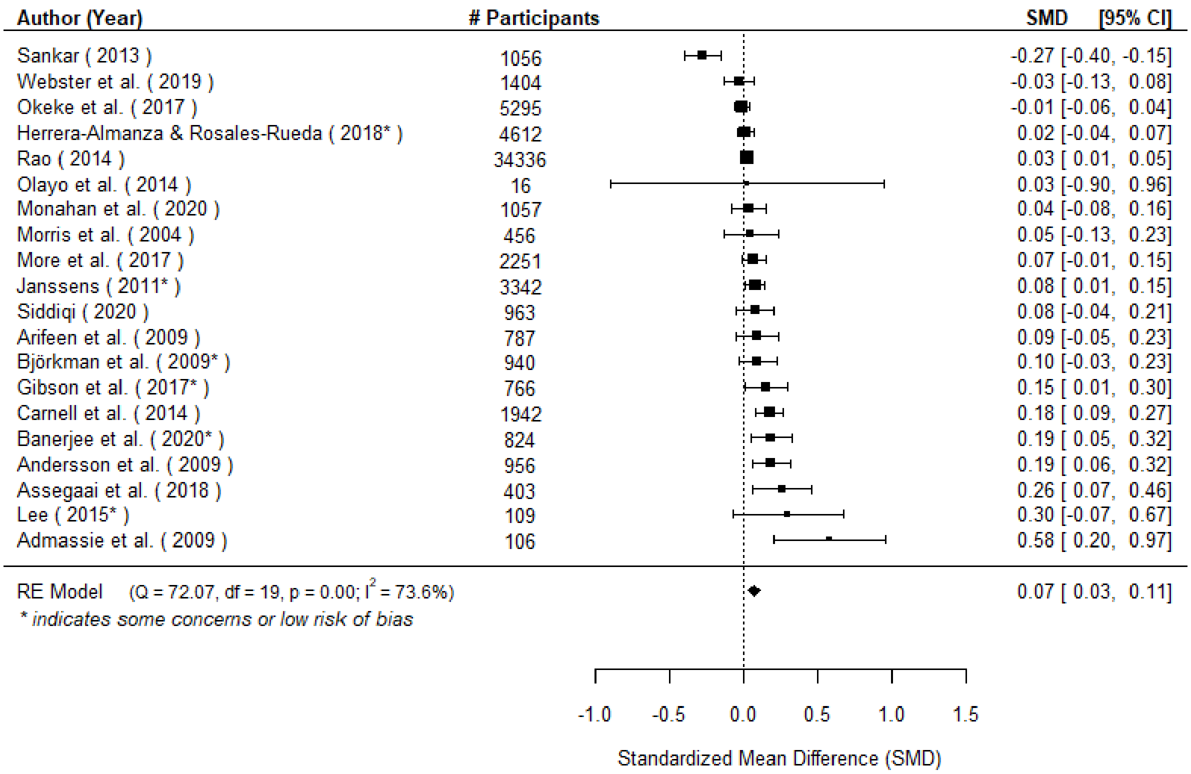
Author (Year)
Participants
SMD [95% CI]
Sankar (2013) 1056 -0.27 [-0.40, -0.15]
Webster et al. (2019) 1404 -0.03 [-0.13, 0.08]
Okeke et al. (2017) 5295 -0.01 [-0.06, 0.04]
Herrera-Almanza & Rosales-Rueda (2018*) 4612 0.02 [-0.04, 0.07]
Rao (2014) 34336 0.03 [0.01, 0.05]
Olayo et al. (2014) 16 0.03 [-0.90, 0.96]
Monahan et al. (2020) 1057 0.04 [-0.08, 0.16]
Morris et al. (2004) 456 0.05 [-0.13, 0.23]
More et al. (2017) 2251 0.07 [-0.01, 0.15]
Janssens (2011*) 3342 0.08 [0.01, 0.15]
Siddiqi (2020) 963 0.08 [-0.04, 0.21]
Arifeen et al. (2009) 787 0.09 [-0.05, 0.23]
Björkman et al. (2009*) 940 0.10 [-0.03, 0.23]
Gibson et al. (2017*) 766 0.15 [0.01, 0.30]
Carnell et al. (2014) 1942 0.18 [0.09, 0.27]
Banerjee et al. (2020*) 824 0.19 [0.05, 0.32]
Andersson et al. (2009) 956 0.19 [0.06, 0.32]
Assegaai et al. (2018) 403 0.26 [0.07, 0.46]
Lee (2015*) 109 0.30 [-0.07, 0.67]
Admassie et al. (2009) 106 0.58 [0.20, 0.97]
RE Model (Q = 72.07, df = 19, p = 0.00; I² = 73.6%) 0.07 [0.03, 0.11]
* indicates some concerns or low risk of bias
-1.0 -0.5 0.0 0.5 1.0 1.5
Standardized Mean Difference (SMD)

review only

Use of community engagement interventions to improve child immunisation in low- and middle-income countries: a systematic review and meta-analysis

Supplementary material

Table of Contents

APPENDIX 1: AMENDMENTS TO INFORMATION PROVIDED IN THE PROTOCOL 3

APPENDIX 2: OVERVIEW OF COMMUNITY ENGAGEMENT FRAMEWORK 5

APPENDIX 3: SEARCH STRATEGY AND SEARCH TERMS FOR IMPACT EVALUATIONS 11

APPENDIX 4: LIST OF EXCLUDED IMPACT EVALUATION STUDIES 16

APPENDIX 5: QUALITATIVE AND COST-EVIDENCE SEARCH STRATEGY 28

Qualitative search protocol 28

Cost evidence search protocol 30

List of included qualitative studies 30

List of included other studies 33

APPENDIX 6: DATA EXTRACTION TOOLS 37

Quantitative data extraction tool 37

Qualitative data extraction tool 42

Cost data extraction tool 57

APPENDIX 7: RISK OF BIAS ASSESSMENT TOOLS 65

Quantitative risk of bias tool 65

Qualitative risk of bias tool 81

Cost evidence risk of bias tool 89

APPENDIX 8: RISK OF BIAS IN THE INCLUDED STUDIES 90

Quantitative risk of bias 90

For peer review only

For peer review only - http://bmjopen.bmj.com/site/about/guidelines.xhtml

Qualitative risk of bias 93

Risk of bias in cost effectiveness estimates 94

APPENDIX 9: QUANTITATIVE RESULTS – ALL COMMUNITY ENGAGEMENT INTERVENTIONS 98

APPENDIX 10: QUANTITATIVE RESULTS – ENGAGEMENT AS INTERVENTION 131

APPENDIX 11: QUANTITATIVE RESULTS - COMMUNITY ENGAGEMENT IN INTERVENTION DESIGN 144

APPENDIX 12: COMMUNITY ENGAGEMENT IN IMPLEMENTATION AUTONOMY OF INTERVENTIONS 158

APPENDIX 13: MULTIPLE ENGAGEMENT TYPES 165

APPENDIX 14: QUALITATIVE SYNTHESIS OF RESULTS 180

Barriers to immunisation 180

Facilitators of immunisation 190

Reasons for intervention success 196

Reasons for intervention failure 206

Reasons for heterogenous impacts 216

Uptake and fidelity challenges 216

APPENDIX 15: SYNTHESIS OF COST EVIDENCE 229

For peer review only - http://bmjopen.bmj.com/site/about/guidelines.xhtml

Appendix 1: Amendments to information provided in the protocol

1. In the report, we have replaced the word 'participation' for 'engagement', a change that is also reflected in our review title and objectives. Though the IAP2 framework uses the terms "public participation" and "community engagement" interchangeably, we realise that this may lead to confusion as there may be inter researcher differences in how these terms are perceived and used. Therefore, for maintaining consistency, we have used community engagement throughout the report and avoided any references to community participation. From a linguistic perspective, these words are used interchangeability in our review, as is evident from our protocol (please refer to pp. 1-3). Therefore, this change does not alter the scope of our review in any way. In the protocol we had proposed to use the IAP2 spectrum of community engagement for determination of interventions to be included in the review. However, after pilot testing of the IAP2 framework on a range of community engagement interventions, we found that most interventions are based on a more "utilitarian perspective" put forth in Brunton et al. (2017). Thus, for this review we ultimately settled on the community engagement framework which focuses on process of engagement rather than its intensity. Our approach corresponds to some degree to the "extent of engagement" part of the conceptual framework developed in Brunton et al. (2017) and we also kept the spirit of IAP2 framework by including interventions in which engagement goes beyond one-way communication, i.e. beyond the inform level of IAP2, to include some consultation or dialogue with the community or some decision making by the community members. We found this approach to be relatively easier to apply, less subjective, less prone to classification error and potentially useful to practitioners
2. In the section on objectives, we have clubbed what were originally review questions 3 and 4 in the protocol (p. 5). These questions related to understanding how implementation features and contextual factors such as barriers and facilitators to immunisation, are associated with relative success or failure of community engagement interventions. When we set out to answer these questions through our qualitative synthesis, we realised that all these factors are interlinked and, in various combinations, may influence the outcomes along the causal chain. Therefore, we combined the two objectives and have presented our analysis and results accordingly. This modification to the review objectives does not alter our study's scope.
3. We had intended to include the relevant Chinese literature in the review. However, because of political disruptions in Hong Kong, it did not materialize.
4. We have added many more secondary outcomes as per our evidence gap map framework and also their categorization has changed. But data was not available in primary studies for most of them, so they could not be analysed in this review.
5. In the protocol we stated that 'When multiple papers report different results on an identical outcome, we will contact the authors to enquire about the differences and choose the results that more accurately reflect the impact of the intervention as relevant to our research questions. If contacting the author does not yield a clear decision, we will use results from the paper with the latest publication date.' However, due to the large scope and limited resources of our review, we instead used results from the most recent paper.
6. In the protocol, we stated that 'Where multiple outcomes are reported from different specifications, we will select the specification with the lowest risk of bias in attributing impact, for example, the most appropriately specified outcomes equation.' However, given that risk of bias could not be assessed until after inclusion decisions were made, we instead used the authors preferred specification. If this was not explicitly stated, but the authors reported one specification as the "main results" and other specifications as "robustness checks" we used the "main results" specification. If neither of these applied, we used the specification with the most controls.
7. In the protocol we stated that 'When multiple treatment arms, we will divide the control group by the number of treatment arms.' Instead, we chose independent effects, choosing either a) the treatment arm

For peer review only - http://bmjopen.bmj.com/site/about/guidelines.xhtml

with the most components, b) the treatment arm that was identified by the authors as the one that would be scaled up, or c) when number of components was equal, the arm that was the most cost effective (e.g. choosing unconditional cash transfers (UCTs) over conditional cash transfers as the administrative costs related to UCTs are significantly lower)

For peer review only

For peer review only - http://bmjopen.bmj.com/site/about/guidelines.xhtml

Appendix 2: Overview of community engagement framework

The focus of our review is community engagement interventions, which are increasingly being emphasised in international and national policy frameworks as a means to improve immunisation coverage and reach marginalised communities (UNICEF 2018, WHO 2015, WHO 2017b; WHO 2017a).

The most common approach for categorizing community engagement interventions is probably the International Association for Public Participation (IAP2) framework (iap2.org), which identifies five levels of engagement ranging from inform to empower corresponding to increasing community influence over the decisions. This framework corresponds to the "social justice perspective" in Brunton et al. (2017) in which the community engagement is rooted in concerns about social justice, which requires that the health needs are identified by communities themselves and they mobilise themselves into action to make changes within the community. However, after pilot testing of the IAP2 framework on a range of community engagement interventions, we determined that most interventions are based on a more "utilitarian perspective." These two perspectives of community engagement are very well captured and articulated in Brunton et al. (2017), who in their systematic review of community engagement narratives in public health point out that:

"Historically, interventions to promote health were driven by professionals, with little or no input from the targeted populations; more recently, community engagement has become central to national strategy and guidance for promoting public health, because, from a 'utilitarian' point of view, it is thought that more acceptable and appropriate interventions will result, which may result in improved service use and outcomes. Interventions that are based on a utilitarian perspective seek to involve communities in order to improve the effectiveness of the intervention. The intervention itself may be decided upon before the community is invited for its views; or, while the intervention itself is not designed by community members they may be involved in other ways, such as priority setting, or in its delivery. In utilitarian perspectives, health (and other) services reach out to engage particular communities that they have identified require assistance and the intervention is devised within existing policy, practice, and resource frameworks."

Due to the difference in how the IAP2 framework approaches community engagement and how interventions are actually implemented, it was difficult to systematically categorize interventions using the IAP2 framework with a high degree of consistency among coders. In addition, even for the interventions rooted in "social justice" perspective, their description in the studies was too limited to identify them and map their intensity of community engagement without introducing a lot of subjectivity and non-systematicness.

To avoid this misclassification as far as possible, we experimented with different frameworks and ultimately settled on the one which focuses on process of engagement rather than its intensity. We found that focusing on when and how the community is engaged is a more practical framework for the kind of community engagement interventions that have been evaluated in real world settings. Our approach corresponds to some degree to the "extent of engagement" part of the conceptual framework developed in Brunton et al. (2017) and we also kept the spirit of IAP2 framework by including interventions in which engagement goes beyond one-way communication, i.e. beyond the inform level of IAP2, to include some consultation or dialogue with the community or some decision making by the community members. We found this approach to be relatively easier to apply, less subjective, less prone to classification error and potentially useful to practitioners. The development of this process-oriented framework took a period of around one month and involved three of our core team members

We consider three points within an intervention during which engagement can occur: engagement can occur in the design of the intervention, engagement can occur in the implementation of the intervention, or the intervention may be engagement. We break these categories into further sub-groups representing specific ways through which

For peer review only - http://bmjopen.bmj.com/site/about/guidelines.xhtml

engagement can occur; for example, the development of new cadres of health workers, pilot studies, and the involvement of the community in governance and decision making.

This framework adds a new dimension to the discussion of types of engagement. As practitioners consider the design of their programs, they can use the evidence provided through this framework to determine when and how best to engage with the community.

Engagement as the intervention (engagement is embedded): In these interventions a serious attempt was made to gain community buy-in for activities or new cadres of community-based structures were established, such as village health committees or community health volunteers. While, in a few cases, the development of new cadres can be solely for the purpose of didactic teaching and one-way communication, it generally results in dynamic discussion and two-way communication. As such, we chose to include these interventions. Interventions which are themselves community engagement can motivate communities to take ownership of service delivery and address local problems with local solutions.

Engagement in the design of interventions: In these interventions community input or feedback was sought before the implementation of an intervention. Such feedback can take the form of a pilot, needs assessment, formative evaluation, or other outreach effort. This form of engagement must occur before the implementer undertakes an action. These interventions allow the community to influence the form of the ultimate action taken. Depending on the weight that is given to the feedback, these engagement activities can align to any of the IAP2 categories other than inform, which was not considered.

Engagement in implementation autonomy of interventions: In many interventions the community is not asked for input in their design, but is utilized in their implementation as health care workers, facilitators, or problem solvers. In the spirit of IAP2 framework to go beyond inform level interventions, we only included those interventions in this category where the community members involved in the implementation of the intervention had some opportunity to affect or influence its implementation. This broadly aligns with the involve, collaborate and empower levels of engagement. Due to the inclusion criteria of some autonomy in implementation, the interventions under this category generally involved an existing community led governance structure which weighed in on implementation decisions or the community providing resources without which the intervention could not be implemented. We excluded interventions in which community members, like community health workers or frontline health workers, were involved in implementation, but no new cadres of health workers were created and they could not influence its implementation. For example, community health workers supplying hygiene kits or doing home visits. In addition, interventions which built capacity of existing cadres of community members or provided supportive supervision were excluded. For example, m-health apps for community workers and training of peer facilitators or community health workers or frontline health workers that only allowed for a one-way transfer of knowledge.

For peer review only - http://bmjopen.bmj.com/site/about/guidelines.xhtml

Examples of engagement types

ENGAGEMENT AS THE INTERVENTION (ENGAGEMENT IS EMBEDDED)

Effect of health intervention integration within women's self-help groups on collectivization and healthy practices around reproductive, maternal, neonatal and child health in rural India
Health-focused self-help groups were created for women of reproductive age in marginalized communities.

Effect of peer education on knowledge, attitude and completeness of childhood routine immunization in a rural community of Plateau State.
A new cadre of peer educators was created. Women were trained to provide women with information about routine childhood immunisation. This does not qualify as engagement in implementation because the intervention was the training of peer educators and not the subsequent actions of the peer educators.

Impacts of engaging communities through traditional and religious leaders on vaccination coverage in Cross River State, Nigeria
Traditional and religious leaders were trained to utilize their leadership role to support immunisation. After the training, these leaders then presented data at ward development committee meetings and engaged with the community to encourage immunsation.

ENGAGEMENT IN THE DESIGN OF AN INTERVENTION

Cognitive behaviour therapy-based intervention by community health workers for mothers with depression and their infants in rural Pakistan: a cluster-randomised controlled trial
Many pilots directly seek the feedback of community members. In this study, health workers and depressed mothers were asked about the relevance and usefulness of the intervention before scaling.

Mobile Phone Incentives for Childhood Immunizations in Rural India
Caregivers were given mobile phone credit incentives for completing imminizations. The amount of the incentive was decided upon through conversations that involved community members.

ENGAGEMENT IN THE IMPLEMENTATION AUTONOMY OF AN INTERVENTION

The impact of an immunization programme administered through the Growth Monitoring Programme Plus as an alternative way of implementing Integrated Management of Childhood Illnesses in urban-slum areas of Lusaka, Zambia
The GMP+ sessions were conducted by medical personnel from Public Health Centers. During these session, community volunteers provided some operational and managerial support to ensure the effective implementation of the sessions.

Effects of payment for performance on accountability mechanisms: Evidence from Pwani, Tanzania
The intervention is a performance-based financing mechanism. The community health committee was involved in decisions about how to spend the funds gained through the pay for performance mechanism.

Interventions are defined based on the actions of the external actors, ie implementers, not the community itself. If an intervention spurred the community to take action, the categorization of the intervention is based on the intervention which spurred the action and not the actions that the community took as a result of the intervention. This is because another community might take different actions as a result of the same intervention. The distinction is especially important for interventions to empower communities to improve their own systems. In these cases, the intervention is the activity that empowered the community; this intervention is engagement. For example, interventions which develop village committees to identify local challenges and solutions are themselves engagement interventions. This is because the intervention is the development of the committee and not the actions of the committee. A similar committee in a different area may choose to take different actions, even if the implementing agency does the same development process. Although the community may have engaged in the design of the committee, they likely were not engaged in the design of development process which brought about the committee. For interventions to be qualified as engagement in the design or implementation, the intervention must be an action by an implementer which the community influenced. For example, community members could be consulted in the design of materials used in

For peer review only - http://bmjopen.bmj.com/site/about/guidelines.xhtml

outreach activities (Murthy 2019, Nagar 2020). In the example of the development of a new committee, the implementer influenced the action of the community, the community did not influence the action of the implementer.

We defined "communities" in reference to the lowest level of the health service delivery system (or whatever level provides routine immunisation services in the local context). A community is a group of people who are served by a particular primary health facility. Thus, communities encompass a wide range of stakeholders, including caregivers, health service providers, and influential community members such as religious or other traditional leaders. Therefore, our review included any intervention that was directed towards any of these types of community members. Interventions that targeted higher levels of the health system, such as state-level officials, were excluded.

How the interventions might work

A 2015 3ie scoping paper (Sabarwal et al. 2015) systematically mapped the literature on immunisation interventions involving community engagement. Several programme managers and policy experts provided insight regarding why community engagement could be the key to improving immunisation outcomes for children in areas where the coverage has stagnated or declined or that are hard to reach. The findings from the scoping study indicate that working with or engaging communities could help develop a better understanding of the context, target population, problems and barriers, and lead to identification of contextually relevant solutions and desired outcomes, and mobilising community support for them. Because individuals usually function under the influence of social norms, efforts to change these norms can be effective in changing behaviour (Bicchieri and Xiao 2009; Reynolds Subašić and Tindall 2015). People respond to their peers and community and, while activities such as information and education campaigns might have some influence, individuals might feel bound by collective decisions, preventing sustained change (Riedy et al. 2012). The role of peers and of social norms in shaping attitudes towards vaccination is particularly important given that vaccine hesitancy has been documented in countries of all income levels (although it takes different forms in different countries; Dubé, Gagnon, Nickels, Jeram, and Schuster 2014). Community engagement may be effective at overcoming these barriers to immunisation. In both high-income countries (HICs; O'Mara-Eves et al. 2013) and L&MICs (De Buck et al. 2017), community engagement has been an effective model of modifying health behaviours in particular. Hence, community engagement could be an important determinant of success or failure of an intervention aimed at improving immunisation coverage.

Due to the variability in contexts, activities to address the barriers to immunisation through community engagement also vary. Therefore, no single theory of change can capture all the different ways that community engagement will affect immunisation outcomes. Furthermore, there is no strict correspondence between types of engagement and intervention activities. For the most part, each activity can be structured such that community engagement is the intervention (engagement is embedded) or the community is engaged in the design or implementation of the intervention. For example, consider an intervention involving village health committees in which community members are adequately represented. These committees can be created by a sponsoring agency with little or no input from the actual community. In this case, the intervention is engagement. On the other hand, community members may develop a village health committee during the design phase of major clinic infrastructure projects to ensure the efficient management of the intervention. Hence, these committees could arise from community involvement in the design of the intervention. In addition, an existing village health committee could be leveraged for the implementation of community meetings at which committee members lead a dialogue on immunisation.

Broadly, we expect that community engagement activities will increase awareness of vaccine-preventable diseases, knowledge of where and when to get vaccinations, and motivation to get vaccinations among caregivers (Figure 1). Community engagement may also increase skill, motivation, and accountability of health workers. These ought to lead to improved demand for and delivery of services, which will increase the number of vaccinated children and could potentially reduce child morbidity and mortality. It is important to note that child mortality and morbidity are affected by critical factors beyond immunisation, such as access to safe food and adequate nutrition, safe water and

For peer review only - http://bmjopen.bmj.com/site/about/guidelines.xhtml

quality care by a trained health provider when needed. Without these critical enabling factors immunisation alone may not be effective in reduction of child mortality and morbidity.

For peer review only

For peer review only - http://bmjopen.bmj.com/site/about/guidelines.xhtml

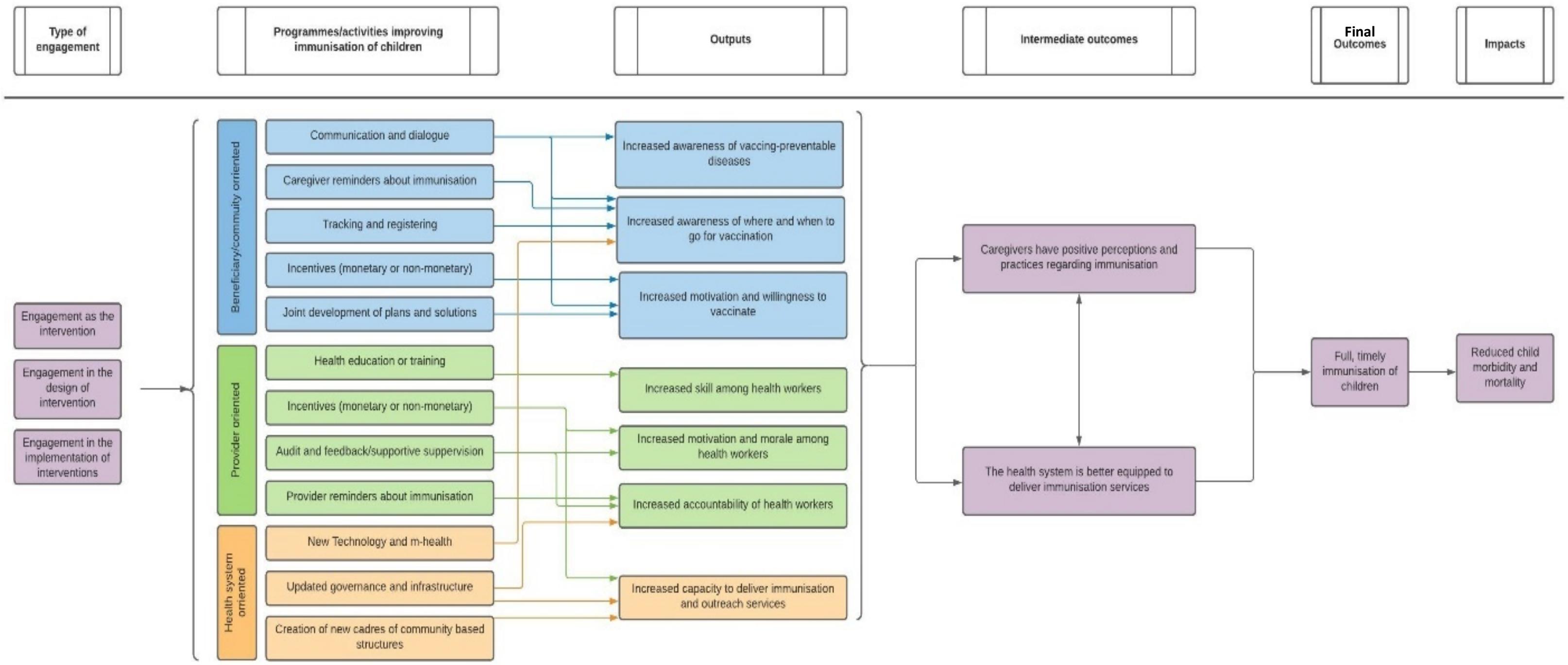

Supplementary Figure 1: Intervention theory of chan

Only

For peer review only - http://bmjopen.bmj.com/site/about/guidelines.xhtml

Appendix 3: Search strategy and search terms for impact evaluations

We searched the academic databases and websites listed below on 17 May 2019 and updated the search on 5 May, 2020. A full record of the applied search terms is provided in Appendix C.

1. MEDLINE
2. CAB Global Health
3. EMBASE
4. Cochrane Controlled Trials Register (CENTRAL)
5. CINAHL
6. PsycINFO
7. Popline
8. Africa-wide information
9. Academic search complete
10. Scopus
11. Campbell Library
12. Google Scholar
13. EconLit
14. IDEAS/RePEc
15. WHO Global Index Medicus
16. Pascal-Francis
17. Open-Grey
18. Grey Literature Report
19. Social Science Research Network (SSRN)
20. Eldis
21. GAVI
22. Epistemonikos
23. Innovations for Poverty Action (IPA)
24. Abdul Latif Jameel Poverty Action Lab (J-PAL)
25. 3ie Impact Evaluation Repository
26. 3ie Systematic Review Repository
27. Registry of International Development Impact Evaluations (RIDIE)
28. Global Development Network
29. World Bank Development Impact Evaluation (DIME) and Impact Evaluation Policy Papers
30. Inter-American Development Bank
31. Center for Global Development
32. Center for Effective Global Action (CEGA)
33. DFID Research for Development (R4D)
34. USAID

Website searches for capturing grey literature were carried out from January to May 2020

Search terms

L&MICs

2. (Afghanistan or Albania or Algeria or Angola or Argentina or Armenia or Armenian or Azerbaijan or Bangladesh or Benin or Byelarus or Byelorussian or Belarus or Belorussian or Belorussia or Belize or Bhutan or Bolivia or Bosnia or Herzegovina or Hercegovina or Botswana or Brazil or Bulgaria or Burkina Faso or Burkina Fasso or Upper Volta or Burundi or Urundi or Cambodia or Khmer Republic or Kampuchea or

For peer review only - http://bmjopen.bmj.com/site/about/guidelines.xhtml

Cameroon or Cameroons or Cameron or Camerons or Cape Verde or Cabo Verde or Central African Republic or Chad or Tchad or China or Colombia or Comoros or Comoro Islands or Comores or Mayotte or Congo or Zaire or Costa Rica or Cote d'Ivoire or Ivory Coast or Cuba or Djibouti or French Somaliland or Dominica or Dominican Republic or East Timor or East Timur or Timor Leste or Ecuador or Egypt or United Arab Republic or El Salvador or Eritrea or Ethiopia or Fiji or Gabon or Gabonese Republic or Gambia or Gaza or Georgia Republic or Georgian Republic or Ghana or Grenada or Guatemala or Guinea or Guiana or Guyana or Haiti or Honduras or India or Maldives or Indonesia or Iran or Iraq or Jamaica or Jordan or Kazakhstan or Kazakh or Kenya or Kiribati or Korea or Kosovo or Kyrgyzstan or Kirghizia or Kyrgyz Republic or Kirghiz or Kirgizstan or Lao PDR or Laos or Lebanon or Lesotho or Basutoland or Liberia or Libya or Macedonia or Madagascar or Malagasy Republic or Malaysia or Malaya or Malay or Sabah or Sarawak or Malawi or Mali or Marshall Islands or Mauritania or Mauritius or Agalega Islands or Mexico or Micronesia or Middle East or Moldova or Moldovia or Moldovian or Mongolia or Montenegro or Morocco or Ifni or Mozambique or Myanmar or Myanma or Burma or Namibia or Nepal or Netherlands Antilles or Nicaragua or Niger or Nigeria or Muscat or Pakistan or Palau or Palestine or Panama or Paraguay or Peru or Philippines or Philipines or Phillipines or Phillippines or Papua New Guinea or Romania or Rumania or Roumania or Rwanda or Ruanda or Saint Lucia or St Lucia or Saint Vincent or St Vincent or Grenadines or Samoa or Samoan Islands or Navigator Island or Navigator Islands or Sao Tome or Senegal or Serbia or Montenegro or Seychelles or Sierra Leone or Sri Lanka or Solomon Islands or Somalia or Sudan or Suriname or Surinam or Swaziland or Eswatini or South Africa or Syria or Tajikistan or Tadzhikistan or Tadjikistan or Tadzhik or Tanzania or Thailand or Togo or Togolese Republic or Tonga or Tunisia or Turkey or Turkmenistan or Turkmen or Uganda or Ukraine or Uzbekistan or Uzbek or Vanuatu or New Hebrides or Venezuela or Vietnam or Viet Nam or West Bank or Yemen or Zambia or Zimbabwe or Rhodesia)

3. (Africa or Asia or Caribbean or West Indies or South America or Latin America or Central America)
4. ((developing or less* developed or least developed or under developed or underdeveloped or middle income or low* income or underserved or under served or deprived or poor* or resource limited or resource constrained) adj (countr* or nation? or population? or world or state* or emerging econom* or global south))
5. ((developing or less* developed or least developed or under developed or underdeveloped or middle income or low* income or resource limited or resource constrained) adj (economy or economies))
6. (low* adj (gdp or gnp or gross domestic or gross national))
7. (low adj3 middle adj3 countr*)
8. (lmic or lmics or third world or lami countr*)
9. transitional countr*
10. 1 OR 2 OR 3 OR 4 OR 5 OR 6 OR 7

Immunisation

1. (immuniz* or immunis* or vaccin* or inoculat* or innoculat* or immunotherap* or prophyla*)

Young children and caregivers

1. (child* or infant* or newborn* or neonat* or prenatal or pre natal or antenatal or ante natal or baby or babies or toddler* or preschool* or parent* or mother* or father* or maternal or paternal or caregiver* or grandparent* or grandmother* or grandfather* or family member*)

Impact evaluation methods

For peer review only - http://bmjopen.bmj.com/site/about/guidelines.xhtml

1. (random* or experiment* or (match* adj2 (propensity or coarsened or covariate)) or "propensity score" or "difference in difference*" or "difference-in-difference*" or "differences in difference*" or "differences-in-difference*" or "double difference*" or "quasi-experimental" or "quasi experimental" or "quasi-experiment" or "quasi experiment" or ((estimator or counterfactual) and evaluation*) or "instrumental variable*" or (IV adj2 (estimation or approach)) or regression discontinuity or time series or segment* regression)

- **Example full search strategy**

Below we present a draft of the full search strategy used to search MEDLINE. Note that in MEDLINE's syntax, terms with strokes (e.g., Immunization/) denote Medical Subject Heading (MeSH) terms, while strings appended with "ti,ab,kw" are searched in the title, abstract, and keyword fields of records in the database.

Ovid MEDLINE(R) and Epub Ahead of Print, In-Process & Other Non-Indexed Citations, Daily and Versions(R) <1946 to May 15, 2019> Searched 16th May 2019

1 (immuniz* or immunis* or vaccin* or inoculat* or innoculat* or immunotherap* or prophyla*).ti,ab,kw. (672732)

2 immunization/ or immunization, passive/ or immunization schedule/ or immunization, secondary/ or immunotherapy, active/ or vaccination/ or Immunization Programs/ or mass vaccination/ (158163)

3 Tuberculosis Vaccines/ or BCG Vaccine/ or Diphtheria-Tetanus Vaccine/ or Meningococcal Vaccines/ or Pertussis Vaccine/ or Diphtheria-Tetanus-acellular Pertussis Vaccines/ or Diphtheria-Tetanus-Pertussis Vaccine/ or Diphtheria-Tetanus Vaccine/ or Measles Vaccine/ or Mumps Vaccine/ or Rubella Vaccine/ or Measles-Mumps-Rubella Vaccine/ or Poliovirus Vaccines/ or Poliovirus Vaccine, Inactivated/ or Poliovirus Vaccine, Oral/ or Japanese Encephalitis Vaccines/ or Rotavirus Vaccine/ (49639)

4 or/1-3 (720583)

5 developing countries.sh,kf. (83271)

6 (Africa or Asia or Caribbean or West Indies or South America or Latin America or Central America).ti,ab,kw. (200981)

7 Africa/ or Asia/ or Caribbean/ or West Indies/ or South America/ or Latin America/ or Central America/ (73389)

8 (Africa or Central America or South America or Caribbean or Central Asia or Afghanistan or Albania or Algeria or Angola or Argentina or Armenia or Armenian or Azerbaijan or Bangladesh or Benin or Byelarus or Byelorussian or Belarus or Belorussian or Belorussia or Belize or Bhutan or Bolivia or Bosnia or Herzegovina or Hercegovina or Botswana or Brazil or Bulgaria or Burkina Faso or Burkina Fasso or Upper Volta or Burundi or Urundi or Cambodia or Khmer Republic or Kampuchea or Cameroon or Cameroons or Cameron or Camerons or Cape Verde or Cabo Verde or Central African Republic or Chad or Tchad or China or Colombia or Comoros or Comoro Islands or Comores or Mayotte or Congo or Zaire or Costa Rica or Cote d'Ivoire or Ivory Coast or Cuba or Djibouti or French Somaliland or Dominica or Dominican Republic or East Timor or East Timur or Timor Leste or Ecuador or Egypt or United Arab Republic or El Salvador or Eritrea or Ethiopia or Fiji or Gabon or Gabonese Republic or Gambia or Gaza or Georgia Republic or Georgian Republic or Ghana or Grenada or Guatemala or Guinea or Guiana or Guyana or Haiti or Honduras or India or Maldives or Indonesia or Iran or Iraq or Jamaica or Jordan or Kazakhstan or Kazakh or Kenya or Kiribati or Korea or Kosovo or Kyrgyzstan or Kirghizia or Kyrgyz Republic or Kirghiz or Kirgizstan or Lao PDR or Laos or Lebanon or Lesotho or Basutoland or Liberia or Libya or Macedonia or Madagascar or Malagasy Republic or Malaysia or Malaya or Malay or Sabah or Sarawak or Malawi or Mali or Marshall Islands or Mauritania

For peer review only - http://bmjopen.bmj.com/site/about/guidelines.xhtml

or Mauritius or Agalega Islands or Mexico or Micronesia or Middle East or Moldova or Moldovia or Moldovian or Mongolia or Montenegro or Morocco or Ifni or Mozambique or Myanmar or Myanma or Burma or Namibia or Nepal or Netherlands Antilles or Nicaragua or Niger or Nigeria or Muscat or Pakistan or Palau or Palestine or Panama or Paraguay or Peru or Philippines or Philipines or Phillipines or Phillippines or Papua New Guinea or Romania or Rumania or Roumania or Rwanda or Ruanda or Saint Lucia or St Lucia or Saint Vincent or St Vincent or Grenadines or Samoa or Samoan Islands or Navigator Island or Navigator Islands or Sao Tome or Senegal or Serbia or Montenegro or Seychelles or Sierra Leone or Sri Lanka or Solomon Islands or Somalia or Sudan or Suriname or Surinam or Swaziland or Eswatini or South Africa or Syria or Tajikistan or Tadzhikistan or Tadjikistan or Tadzhik or Tanzania or Thailand or Togo or Togolese Republic or Tonga or Tunisia or Turkey or Turkmenistan or Turkmen or Uganda or Ukraine or Uzbekistan or Uzbek or Vanuatu or New Hebrides or Venezuela or Vietnam or Viet Nam or West Bank or Yemen or Zambia or Zimbabwe or Rhodesia).ti,ab,kw,sh. (1387951)

9 ((developing or less* developed or least developed or under developed or underdeveloped or middle income or low* income or underserved or under served or deprived or poor* or resource limited or resource constrained) adj (countr* or nation? or population? or world or state*)).ti,ab,kw. (91955)

10 ((developing or less* developed or least developed or under developed or underdeveloped or middle income or low* income or resource limited or resource constrained) adj (economy or economies)).ti,ab,kw. (504)

11 (low* adj (gdp or gnp or gross domestic or gross national)).ti,ab,kw. (234)

12 (low adj3 middle adj3 countr*).ti,ab,kw. (12819)

13 (lmic or lmics or third world or lami countr*).ti,ab,kw. (6486)

14 (transitional countr* or emerging econom* or global south).ti,ab,kw. (873)

15 or/5-14 (1487811)

16 4 and 15 (80257)

17 Parents/ or Fathers/ or Mothers/ or Grandparents/ or Caregivers/ or Single Parent/ or Pregnant Women/ or Child, Preschool/ or Infant/ or Infant, Newborn/ or Infant, Low Birth Weight/ or Infant, Small for Gestational Age/ or Infant, Very Low Birth Weight/ or Infant, Extremely Low Birth Weight/ or Infant, Postmature/ or Infant, Premature/ or Infant, Extremely Premature/ (1597959)

18 (child* or infant* or newborn* or neonat* or neo nat* or prenatal or pre natal or ante natal or antenatal or baby or babies or toddler* or preschool* or parent* or mother* or father* or maternal or paternal).ti,ab,kw. (2284298)

19 or/17-18 (2893768)

20 16 and 19 (28515)

21 (random* or experiment* or (match* adj2 (propensity or coarsened or covariate)) or "propensity score" or ("difference in difference*" or "difference-in-difference*" or "differences in difference*" or "differences-in-difference*" or "double difference*") or ("quasi-experimental" or "quasi experimental" or "quasi-experiment" or "quasi experiment") or ((estimator or counterfactual) and evaluation*) or "instrumental variable*" or (IV adj2 (estimation or approach)) or regression discontinuity or time series or segment* regression).ti,ab,kw. (2983909)

For peer review only - http://bmjopen.bmj.com/site/about/guidelines.xhtml

22 Randomized Controlled Trial/ or Random Allocation/ or Evaluation Studies/ or Propensity Score/ or Interrupted Time Series Analysis/ or Controlled Before-After Studies/ or Controlled Clinical Trial/ or Non-Randomized Controlled Trials as Topic/ (896249)

23 or/21-22 (3415351)

24 Cost Analysis/ or Cost-Benefit Analysis/ or Quality-Adjusted Life Years/ or Economics, Medical/ or Cost of Illness/ or Health Care Costs/ or Direct Service Costs/ or Budgets/ or Health Care Sector/ or Public Expenditures/ (193420)

25 (cost-effective* or cost-benefit).ti,ab,kw. (130711)

26 ("life year" or "life years" or qaly* or daly*).ti,ab,kw. (18749)

27 ((economic* or cost*) adj6 (mortality or death* or markov)).ti,ab,kw. (17246)

28 ("cost minimi*" or "cost-utilit*" or "economic evaluation*" or "economic review*" or "cost outcome" or "cost analys*" or "economic analys*" or "budget* impact analys*").ti,ab,kw. (27594)

29 or/24-28 (301485)

30 (review or meta-analysis).pt. (2557796)

31 meta-analysis/ or "systematic review"/ (163880)

32 cochrane database of systematic reviews.jn. (14162)

33 (systematic review or literature review).ti. (130870)

34 or/30-33 (2590211)

35 23 or 29 or 34 (5946083)

36 20 and 35 (8309)

37 exp Animals/ (22312530)

38 Humans/ (17732234)

39 37 not (37 and 38) (4580296)

40 36 not 39 (7984)

For peer review only - http://bmjopen.bmj.com/site/about/guidelines.xhtml

Appendix 4: List of excluded impact evaluation studies

We excluded the following studies at full-text screening for not meeting our community engagement criteria.

Reference list

Aaby, Peter, Adam Roth, Henrik Ravn, Bitiguida Mutna Napirna, Amabelia Rodrigues, Ida Maria Lisse, Lone Stensballe, et al. 2011. "Randomized Trial of BCG Vaccination at Birth to Low-Birth-Weight Children: Beneficial Nonspecific Effects in the Neonatal Period?" The Journal of Infectious Diseases 204 (2): 245–52. https://doi.org/10/ds6pbx.

Aggarwal, Shilpa. 2018. "The Long Road to Health: Healthcare Utilization Impacts of a Road Pavement Policy in Rural India." Working paper. Hyderabad: Indian School of Business. http://eprints.exchange.isb.edu/884/.

Ahmed, Tashrik, Aneesa Arur, Damien de Walque, and Gil Shapira. 2019. "Incentivizing Quantity and Quality of Care: Evidence from an Impact Evaluation of Performance-Based Financing in the Health Sector in Tajikistan." Policy Research Working Papers 8951. Washington, DC: The World Bank. https://doi.org/10.1596/1813-9450-8951.

Alatas, Vivi, Arun Chandrasekhar, Markus Mobius, Benjamin Olken, and Cindy Paladines. 2019. "When Celebrities Speak: A Nationwide Twitter Experiment Promoting Vaccination in Indonesia." NBER Working Paper Series 25589. Cambridge, MA: National Bureau of Economic Research. https://doi.org/10.3386/w25589.

Amaral, João, Eleanor Gouws, Jennifer Bryce, Álvaro Jorge Madeiro Leite, Antonio Ledo Alves da Cunha, and Cesar G. Victora. 2004. "Effect of Integrated Management of Childhood Illness (IMCI) on Health Worker Performance in Northeast-Brazil." Cadernos de Saúde Pública 20 (suppl 2): S209–19. https://doi.org/10/dbvvrz.

Andrade, Mônica Viegas, Flávia Chein, Laetícia Rodrigues de Souza, and Jaume Puig-Junoy. 2012. "Income Transfer Policies and the Impacts on the Immunization of Children: The Bolsa Família Program." Cadernos de Saúde Pública 28 (7): 1347–58. https://doi.org/10/gghf2x.

Andreoni, James, Michael Callen, Muhammad Yasir Khan, Karrar Jaffar, and Charles Sprenger. 2016. "Using Preference Estimates to Customize Incentives: An Application to Polio Vaccination Drives in Pakistan." NBER Working Paper Series 22019. Cambridge, MA: National Bureau of Economic Research. https://doi.org/10.3386/w22019.

Anjum, Qudsia, Aamir Omair, S. N. Bazmi Inam, Yousuf Ahmed, Yaseen Usman, and Shazia Shaikh. 2004. "Improving Vaccination Status of Children under Five through Health Education." JPMA. The Journal of the Pakistan Medical Association 54 (12): 610–13.

Aquino, Rosana, Nelson F. de Oliveira, and Mauricio L. Barreto. 2009. "Impact of the Family Health Program on Infant Mortality in Brazilian Municipalities." American Journal of Public Health 99 (1): 87–93. https://doi.org/10/btntq8.

Arraiz, Irani, and Sandra Rozo. 2011. "Same Bureaucracy, Different Outcomes in Human Capital? How Indigenous and Rural Non-Indigenous Areas in Panama Responded to the CCT." OVE Working Papers 03/11. Washington, D.C.: Inter-American Development Bank. http://www.ssrn.com/abstract=1847124.

Atnafu, Asfaw, Kate Otto, and Christopher H. Herbst. 2017. "The Role of MHealth Intervention on Maternal and Child Health Service Delivery: Findings from a Randomized Controlled Field Trial in Rural Ethiopia." MHealth 3 (September). https://doi.org/10/gcmhjc.

Attanasio, Orazio, Luis Carlos Gómez, Patricia Heredia, and Marcos Vera-Hernández. n.d. "The Short-Term Impact of a Conditional Cash Subsidy on Child Health and Nutrition in Colombia," 15.

Ayieko, Philip, Stephen Ntoburi, John Wagai, Charles Opondo, Newton Opiyo, Santau Migiro, Annah Wamae, et al. 2011. "A Multifaceted Intervention to Implement Guidelines and Improve Admission Paediatric Care in Kenyan District Hospitals: A Cluster Randomised Trial." Edited by Igor Rudan. PLoS Medicine 8 (4). https://doi.org/10/dtj73r.

Balasubramaniam, Sudharsanam, Somesh Kumar, Reena Sethi, Elaine Charurat, Kamlesh Lalchandani, Anne Schuster, and Bulbul Sood. 2018. "Quasi-Experimental Study of Systematic Screening for Family Planning

For peer review only - http://bmjopen.bmj.com/site/about/guidelines.xhtml

Services among Postpartum Women Attending Village Health and Nutrition Days in Jharkhand, India." International Journal of Integrated Care 18 (1). https://doi.org/10/gg9pgr.

Banerjee, Rakesh, and Ashish Sachdeva. 2015. "Pathways to Preventive Health, Evidence from India's Rural Road Program." USC-INET Research Paper 15–19. Los Angeles, CA: USC Dornsife Institute for New Economic Thinking. http://www.ssrn.com/abstract=2636999.

Bangure, Donewell, Daniel Chirundu, Notion Gombe, Tawanda Marufu, Gibson Mandozana, Mufuta Tshimanga, and Lucia Takundwa. 2015. "Effectiveness of Short Message Services Reminder on Childhood Immunization Programme in Kadoma, Zimbabwe - a Randomized Controlled Trial, 2013." BMC Public Health 15 (1). https://doi.org/10/f63zrg.

Barham, Tania. 2005. "The Impact of the Mexican Conditional Cash Transfer Program on Immunization Rates." Boulder: University of Colorado. https://ibs.colorado.edu/barham/wp/CCTimmunWBfin.pdf.

Barham, Tania, Logan E. Brenzel, and John A. Maluccio. 2007. "Beyond 80%: Are There New Ways of Increasing Vaccination Coverage? Evaluation of CCT Programs in Mexico and Nicaragua." HNP Discussion Paper 41537. Washington: World Bank. http://www.ssrn.com/abstract=993760.

Barham, Tania, and John A. Maluccio. 2009. "Eradicating Diseases: The Effect of Conditional Cash Transfers on Vaccination Coverage in Rural Nicaragua." Journal of Health Economics 28 (3): 611–21. https://doi.org/10/czhvx3.

Basinga, Paulin, P. J. Gertler, Agnes Binagwaho, A. L. B. Soucat, J. R. Sturdy, and C. M. J. Vermeersch. 2009. "Impact of Performance Based Financing in Rwanda: Health Facility Level Analysis." Working Paper 32. New Delhi: Global Development Network. http://www.gdn.int/impact-performance-based-financing-rwanda-health-facility-level-analysis.

Basinga, Paulin, Paul J Gertler, Agnes Binagwaho, Agnes LB Soucat, Jennifer Sturdy, and Christel MJ Vermeersch. 2011. "Effect on Maternal and Child Health Services in Rwanda of Payment to Primary Health-Care Providers for Performance: An Impact Evaluation." The Lancet 377 (9775): 1421–28. https://doi.org/10/b2f469.

Beck, Simon, Anni-Maria Pulkki-Brännström, and Miguel San Sebastián. 2015. "Basic Income – Healthy Outcome? Effects on Health of an Indian Basic Income Pilot Project: A Cluster Randomised Trial." Journal of Development Effectiveness 7 (1): 111–26. https://doi.org/10/f3n3kb.

Berhane, Yemane, and Joyce Pickering. 1993. "Are Reminder Stickers Effective in Reducing Immunization Dropout Rates in Addis Ababa, Ethiopia?" Journal of Tropical Medicine and Hygiene 96 (3): 139–45. https://europepmc.org/article/med/8505766.

Bernal, Pedro, and Sebastian Martinez. 2020. "In-Kind Incentives and Health Worker Performance: Experimental Evidence from El Salvador." Journal of Health Economics 70 (March). https://doi.org/10/ghpbkj.

Binyaruka, Peter, Edith Patouillard, Timothy Powell-Jackson, Giulia Greco, Ottar Maestad, and Josephine Borghi. 2015. "Effect of Paying for Performance on Utilisation, Quality, and User Costs of Health Services in Tanzania: A Controlled before and after Study." Edited by Jan Ostermann. PLOS ONE 10 (8). https://doi.org/10/gghf22.

Bonfrer, Igna, Lyn Breebaart, and Ellen Van de Poel. 2016. "The Effects of Ghana's National Health Insurance Scheme on Maternal and Infant Health Care Utilization." Edited by Osman Alimamy Sankoh. PLOS ONE 11 (11). https://doi.org/10/f9q46z.

Bonfrer, Igna, Ellen Van de Poel, and Eddy Van Doorslaer. 2014. "The Effects of Performance Incentives on the Utilization and Quality of Maternal and Child Care in Burundi." Social Science & Medicine 123 (December): 96–104. https://doi.org/10/f6vdkg.

Bossio, Juan Carlos, Ivan Sanchis, Gustavo Ariel Armando, Sergio Javier Arias, and Humberto Jure. 2019. "Resultado de una estrategia de recordatorios previos y posteriores a la fecha de vacunación para mejorar la oportunidad de la vacunación a los seis meses." Cadernos de Saúde Pública 35 (12). https://doi.org/10/ghpbkt.

Bradley, Janet, and Susan Igras. 2005. "Improving the Quality of Child Health Services: Participatory Action by Providers." International Journal for Quality in Health Care 17 (5): 391–99. https://doi.org/10/bdjp6m.

Brenner, Stephan, Rachel P. Chase, Shannon A. McMahon, Julia Lohmann, Christopher J. Makwero, Adamson S. Muula, and Manuela De Allegri. 2020. "Effect Heterogeneity in Responding to Performance-Based Incentives:

For peer review only - http://bmjopen.bmj.com/site/about/guidelines.xhtml

A Quasi-Experimental Comparison of Impacts on Health Service Indicators between Hospitals and Health Centers in Malawi." Health Systems & Reform 6 (1). https://doi.org/10/gg2njg.
Briere, E. C., T. K. Ryman, E. Cartwright, E. T. Russo, K. A. Wannemuehler, B. L. Nygren, S. Kola, et al. 2012. "Impact of Integration of Hygiene Kit Distribution with Routine Immunizations on Infant Vaccine Coverage and Water Treatment and Handwashing Practices of Kenyan Mothers." Journal of Infectious Diseases 205 (suppl 1): S56–64. https://doi.org/10/f3wknp.
Brown, V. B., O. A. Oluwatosin, J. O. Akinyemi, and A. A. Adeyemo. 2016. "Effects of Community Health Nurse-Led Intervention on Childhood Routine Immunization Completion in Primary Health Care Centers in Ibadan, Nigeria." Journal of Community Health 41 (2): 265–73. https://doi.org/10/f8czr8.
Brown, Victoria Bolanle, Oyeninhun Abimbola Oluwatosin, and Martins Olusola Ogundeji. 2017. "Impact of Training Intervention on Immunization Providers' Knowledge and Practice of Routine Immunization in Ibadan, South-Western Nigeria: A Primary Health Care Experience." Pan African Medical Journal 26. https://doi.org/10/ghpbkv.
Brughal, R F, and J P Kevany. 1996. "Maximizing Immunization Coverage through Home Visits: A Controlled Trial in an Urban Area of Ghana." Bulletin of the World Health Organization 74 (5): 517–24. https://www.ncbi.nlm.nih.gov/pmc/articles/PMC2486871/.
Busso, Matias, Julian Cristia, and Sarah Humpage. 2015. "Did You Get Your Shots? Experimental Evidence on the Role of Reminders." Journal of Health Economics 44 (December): 226–37. https://doi.org/10/f75n4m.
Cahyadi, Nur, Rema Hanna, Benjamin A. Olken, Rizal Adi Prima, Elan Satriawan, Ekki Syamsulhakim, and Rema Hanna. 2018. "Cumulative Impacts of Conditional Cash Transfer Programs: Experimental Evidence from Indonesia." NBER Working Paper Series 24670. Cambridge: National Bureau of Economic Research. https://www.nber.org/papers/w24670.
Carrillo, Bladimir, Wilman J Iglesias, and Juan C Trujillo. 2015. "Attainments and Limitations of an Early Childhood Programme in Colombia." Health Policy and Planning 30 (7): 906–16. https://doi.org/10/f7vmx5.
Carvalho, Natalie, Naveen Thacker, Subodh S. Gupta, and Joshua A. Salomon. 2014. "More Evidence on the Impact of India's Conditional Cash Transfer Program, Janani Suraksha Yojana: Quasi-Experimental Evaluation of the Effects on Childhood Immunization and Other Reproductive and Child Health Outcomes." Edited by Jeremy D. Goldhaber-Fiebert. PLoS ONE 9 (10). https://doi.org/10/gghf24.
Chakrabarti, Averi, Karen A. Grépin, and Stéphane Helleringer. 2019. "The Impact of Supplementary Immunization Activities on Routine Vaccination Coverage: An Instrumental Variable Analysis in Five Low-Income Countries." Edited by Vijayaprasad Gopichandran. PLOS ONE 14 (2). https://doi.org/10/gghf25.
Chandir, S., A.J. Khan, H. Hussain, H.R. Usman, S. Khowaja, N.A. Halsey, and S.B. Omer. 2010. "Effect of Food Coupon Incentives on Timely Completion of DTP Immunization Series in Children from a Low-Income Area in Karachi, Pakistan: A Longitudinal Intervention Study." Vaccine 28 (19): 3473–78. https://doi.org/10/cz4mk3.
Chansa, Collins, Ashis Das, Jumana Qamruddin, Jed Friedman, Akafwilangachi Mkandawire, and Monique Vledder. 2015. "Linking Results to Performance: Evidence from a Results Based Financing Pre-Pilot Project in Katete District, Zambia." Discussion Paper 98265. Washington, DC: The World Bank. https://openknowledge.worldbank.org/handle/10986/22390?show=full.
Chelagat, T., G. Kokwaro, J. Onyango, and J. Rice. 2020. "Effect of Project-Based Experiential Learning on the Health Service Delivery Indicators: A Quasi-Experiment Study." BMC Health Services Research 20 (1). https://doi.org/10/gg9pgv.
Chen, Li, Xiaozhen Du, Lin Zhang, Michelle Helena van Velthoven, Qiong Wu, Ruikan Yang, Ying Cao, et al. 2016. "Effectiveness of a Smartphone App on Improving Immunization of Children in Rural Sichuan Province, China: A Cluster Randomized Controlled Trial." BMC Public Health 16 (1). https://doi.org/10/f82vfx.
Chen, Yvonne Jie, Namrata Chindarkar, and Yun Xiao. 2019. "Effect of Reliable Electricity on Health Facilities, Health Information, and Child and Maternal Health Services Utilization: Evidence from Rural Gujarat, India." Journal of Health, Population and Nutrition 38 (1). https://doi.org/10/ghpbk6.

For peer review only - http://bmjopen.bmj.com/site/about/guidelines.xhtml

Cristia, Julian, William N. Evans, and Beomsoo Kim. 2015. "Improving the Health Coverage of the Rural Poor: Does Contracting-out Mobile Medical Teams Work?" The Journal of Development Studies 51 (3). https://doi.org/10/ghpwp8.

Cristia, Julián P, William N Evans, and Beomsoo Kim. 2011. "Does Contracting-out Primary Care Services Work? The Case of Rural Guatemala." IDB Working Paper Series IDB-WP-273. Washington. https://www.econstor.eu/handle/10419/88975.

Cristia, Julian, Ariadna García Prado, and Cecilia Peluffo. 2015. "The Impact of Contracting in and Contracting out Basic Health Services: The Guatemalan Experience." World Development 70 (June): 215–27. https://doi.org/10/gghf27.

Cruzado de la Vega, Viviana. 2017. "Pagos por desempeño para mejorar estado nutricional infantil: impacto de los convenios de apoyo presupuestario en tres regiones peruanas con alta prevalencia de desnutrición cronica infantil, 2010-2014." Revista Peruana de Medicina Experimental y Salud Pública 34 (3): 365–76. https://doi.org/10/gghf28.

Daoud, Adel, and Bernhard Reinsberg. 2019. "Structural Adjustment, State Capacity and Child Health: Evidence from IMF Programmes." International Journal of Epidemiology 48 (2): 445–54. https://doi.org/10/gfqr5z.

Dicko, Alassane, Sidy O Toure, Mariam Traore, Issaka Sagara, Ousmane B Toure, Mahamadou S Sissoko, Alpha T Diallo, et al. 2011. "Increase in EPI Vaccines Coverage after Implementation of Intermittent Preventive Treatment of Malaria in Infant with Sulfadoxine -Pyrimethamine in the District of Kolokani, Mali: Results from a Cluster Randomized Control Trial." BMC Public Health 11 (1). https://doi.org/10/cvr2cj.

Dissieka, Romance, Marissa Soohoo, Amynah Janmohamed, and David Doledec. 2019. "Providing Mothers with Mobile Phone Message Reminders Increases Childhood Immunisation and Vitamin A Supplementation Coverage in Côte d'Ivoire: A Randomised Controlled Trial." Journal of Public Health in Africa 10 (1): 56–60. https://doi.org/10/ghpbk9.

Djibuti, Mamuka, George Gotsadze, Akaki Zoidze, George Mataradze, Laura C Esmail, and Jillian Clare Kohler. 2009. "The Role of Supportive Supervision on Immunization Program Outcome - a Randomized Field Trial from Georgia." BMC International Health and Human Rights 9 (S1). https://doi.org/10/dxd2rk.

Domek, Gretchen J., Ingrid L. Contreras-Roldan, Sheana Bull, Sean T. O'Leary, Guillermo Antonio Bolaños Ventura, Michael Bronsert, Allison Kempe, and Edwin J. Asturias. 2019. "Text Message Reminders to Improve Infant Immunization in Guatemala: A Randomized Clinical Trial." Vaccine 37 (42): 6192–6200. https://doi.org/10/ghpbmb.

Drain, Paul K, Josoa S Ralaivao, Alexander Rakotonandrasana, and Mary A Carnell. 2003. "Introducing Auto-Disable Syringes to the National Immunization Programme in Madagascar." Bulletin of the World Health Organization, 8.

D'Souza, Prathibha V, and J Umarani. 2014. "Teaching Package Improves Mothers Knowledge on Vaccine Preventable Diseases and Vaccination: A Quasi Experimental Study." International Journal of Research in Medical Sciences 2 (3): 976–82. https://doi.org/10/ghpv3h.

Dykstra, Sarah, Amanda Glassman, Charles Kenny, and Justin Sandefur. 2019. "Regression Discontinuity Analysis of Gavi's Impact on Vaccination Rates." Journal of Development Economics 140 (September): 12–25. https://doi.org/10/gg9pgw.

Edoka, Ijeoma, Tim Ensor, Barbara McPake, Rogers Amara, Fu-Min Tseng, and Joseph Edem-Hotah. 2016. "Free Health Care for Under-Fives, Expectant and Recent Mothers? Evaluating the Impact of Sierra Leone's Free Health Care Initiative." Health Economics Review 6 (1). https://doi.org/10/gghf29.

Eichler, Rena, Paul Auxila, Uder Antoine, and Bernateau Desmangles. 2007. "Performance-Based Incentives for Health: Six Years of Results from Supply-Side Programs in Haiti." CGD Working Paper 121. Washington, DC: Center for Global Development. http://www.ssrn.com/abstract=1003249.

Ekhaguere, Osayame A, Rosena O Oluwafemi, Bolaji Badejoko, Lawal O Oyeneyin, Azeez Butali, Elizabeth D Lowenthal, and Andrew P Steenhoff. 2019. "Automated Phone Call and Text Reminders for Childhood Immunisations (PRIMM): A Randomised Controlled Trial in Nigeria." BMJ Global Health 4 (2). https://doi.org/10/gghf3b.

For peer review only

Eze, G U, and A O Adeleye. 2015. “Enhancing Routine Immunization Performance Using Innovative Technology in an Urban Area of Nigeria.” West African Journal of Medicine 34 (1): 3–10. https://pubmed.ncbi.nlm.nih.gov/26902809/.

Gajate-Garrido, Gissele, and Clement Ahiadeke. 2012. “The Effect of Parents’ Insurance Enrollment on Health Care Utilization: Evidence from Ghana.” SSRN Scholarly Paper ID 2158824. Rochester, NY: Social Science Research Network. https://doi.org/10.2139/ssrn.2158824.

Garly, M. L., C. L. Martins, C. Bale, F. da Costa, F. Dias, H. Whittle, and P. Aaby. 1999. “Early Two-Dose Measles Vaccination Schedule in Guinea-Bissau: Good Protection and Coverage in Infancy.” International Journal of Epidemiology 28 (2): 347–52. https://doi.org/10/cgqfgz.

Gauri, Varun. 2009. “Do International Treaties Promote Development? The Convention on the Rights of the Child and Basic Immunization.” Policy Research Working Paper 4964. Washington: The World Bank. https://doi.org/10.1596/1813-9450-4964.

Gauri, Varun, and Peyvand Khaleghian. 2002. “Immunization in Developing Countries: Its Political and Organizational Determinants.” Policy Research Working Paper 2769. Washington: The World Bank. https://www.infona.pl/resource/bwmeta1.element.elsevier-4a3b393c-c1da-3dff-9b7c-91c05767fb9d.

Gilbert, Sarah Skye, Ngwegwe Bulula, Emmanuel Yohana, Jenny Thompson, Emily Beylerian, Laurie Werner, and Jessica C. Shearer. 2020. “The Impact of an Integrated Electronic Immunization Registry and Logistics Management Information System (EIR-ELMIS) on Vaccine Availability in Three Regions in Tanzania: A Pre-Post and Time-Series Analysis.” Vaccine 38 (3): 562–69. https://doi.org/10/ghpbsr.

Goodson, James L., Manisha A. Kulkarni, Jodi L. Vanden Eng, Kathleen A. Wannemuehler, Annett H. Cotte, Rachelle E. Desrochers, Bakolalao Randriamanalina, and Elizabeth T. Luman. 2012. “Improved Equity in Measles Vaccination from Integrating Insecticide-Treated Bednets in a Vaccination Campaign, Madagascar: Equity in Measles Vaccination in Madagascar.” Tropical Medicine & International Health 17 (4): 430–37. https://doi.org/10/fxsrbw.

Guindon, G. Emmanuel. 2014. “The Impact of Health Insurance on Health Services Utilization and Health Outcomes in Vietnam.” Health Economics, Policy and Law 9 (4): 359–82. https://doi.org/10/f6g47g.

Gultiano, Soccoro A., and Elizabeth M. King. 2006. “A Better Start in Life: Evaluation Results from an Early Childhood Development Program.” Philippine Journal of Development 33 (1/2): 101–28. https://ideas.repec.org/p/phd/pjdevt/pjd_2006_vol__xxxiii_nos__1and2-d.html.

Habib, Muhammad Atif, Sajid Soofi, Simon Cousens, Saeed Anwar, Najib ul Haque, Imran Ahmed, Noshad Ali, Rehman Tahir, and Zulfiqar A Bhutta. 2017. “Community Engagement and Integrated Health and Polio Immunisation Campaigns in Conflict-Affected Areas of Pakistan: A Cluster Randomised Controlled Trial.” The Lancet Global Health 5 (6): e593–603. https://doi.org/10/f962m2.

Hagiwara, Akiko, Mika Ueyama, Asad Ramlawi, and Yasuyuki Sawada. 2013. “Is the Maternal and Child Health (MCH) Handbook Effective in Improving Health-Related Behavior? Evidence from Palestine.” Journal of Public Health Policy 34 (1): 31–45. https://doi.org/10/ghpbss.

Haji, Adam Hassan. 2017. “Evaluation of SMS and Sticker Reminders in Reducing Dropout Rates in Routine Child Immunization in Selected Districts in Kenya.” Master Thesis, Juja: Jomo Kenyatta University of Agriculture and Technology. http://ir.jkuat.ac.ke/handle/123456789/3069.

Hajizadeh, Mohammad, Jody Heymann, Erin Strumpf, Sam Harper, and Arijit Nandi. 2015. “Paid Maternity Leave and Childhood Vaccination Uptake: Longitudinal Evidence from 20 Low-and-Middle-Income Countries.” Social Science & Medicine 140 (September): 104–17. https://doi.org/10/f7ppdx.

Hategeka, Celestin, Hinda Ruton, and Michael R. Law. 2019. “Effect of a Community Health Worker MHealth Monitoring System on Uptake of Maternal and Newborn Health Services in Rwanda.” Global Health Research and Policy 4 (1). https://doi.org/10/ghpbst.

Heinrich, Carolyn J., and Matthew T. Knowles. 2020. “A Fine Predicament: Conditioning, Compliance and Consequences in a Labeled Cash Transfer Program.” World Development 129 (May). https://doi.org/10/ghpbsv.

For peer review only - http://bmjopen.bmj.com/site/about/guidelines.xhtml

Helleringer, Stephane, Patrick O. Asuming, and Jalaa Abdelwahab. 2016. “The Effect of Mass Vaccination Campaigns against Polio on the Utilization of Routine Immunization Services: A Regression Discontinuity Design.” Vaccine 34 (33): 3817–22. https://doi.org/10/f8w56m.

Hu, Yu, Yaping Chen, Ying Wang, Quanwei Song, and Qian Li. 2017. “Prenatal Vaccination Education Intervention Improves Both the Mothers’ Knowledge and Children’s Vaccination Coverage: Evidence from Randomized Controlled Trial from Eastern China.” Human Vaccines & Immunotherapeutics 13 (6): 1477–84. https://doi.org/10/gghf3f.

Huillery, Elise, and Juliette Seban. 2019. “Financial Incentives, Efforts, and Performances in the Health Sector: Experimental Evidence from the Democratic Republic of Congo.” Economic Development and Cultural Change, March. https://doi.org/10/ghpbsq.

Hutchinson, Paul, Peter Lance, David K. Guilkey, Mohammad Shahjahan, and Shahida Haque. 2006. “Measuring the Cost-Effectiveness of a National Health Communication Program in Rural Bangladesh.” Journal of Health Communication 11 (sup2): 91–121. https://doi.org/10/b7wdkt.

Ikilezi, Gloria, Orvalho J. Augusto, Joseph L. Dieleman, Kenneth Sherr, and Stephen S. Lim. 2020. “Effect of Donor Funding for Immunization from GAVI and Other Development Assistance Channels on Vaccine Coverage: Evidence from 120 Low and Middle Income Recipient Countries.” Vaccine 38 (3): 588–96. https://doi.org/10/ghpwdv.

Ikilezi, Gloria, Orvalho J Augusto, Alyssa Sbarra, Kenneth Sherr, Joseph L Dieleman, and Stephen S Lim. 2020. “Determinants of Geographical Inequalities for DTP3 Vaccine Coverage in Sub-Saharan Africa.” Vaccine 38 (18): 3447–54. https://doi.org/10/ghpwdw.

Jaupart, Pascal, Lizzie Dipple, and Stefan Dercon. 2019. “Has Gavi Lived up to Its Promise? Quasi-Experimental Evidence on Country Immunisation Rates and Child Mortality.” BMJ Global Health 4 (6). https://doi.org/10/ghpwdx.

Kagucia, E Wangeci. 2018. “MHealth Interventions to Improve Measles Vaccination Coverage and Timeliness: An Assessment of the Immediate and Long-Term Impact on Vaccine-Seeking in Rural Kenya.” PhD Thesis, Baltimore, MD: Johns Hopkins University. https://jscholarship.library.jhu.edu/handle/1774.2/61130.

Kamatsuchi, Mahoko, Adrian Gheorghe, and Dina Balabanova. 2019. “The Global Scale and Implications of Delivering Multiple Interventions through Integrated Child Health Events.” BMJ Global Health 4 (4). https://doi.org/10/ghpwd2.

Kandpal, Eeshani, Harold Alderman, Jed Friedman, Deon Filmer, Junko Onishi, and Jorge Avalos. 2016. “A Conditional Cash Transfer Program in the Philippines Reduces Severe Stunting.” The Journal of Nutrition 146 (9): 1793–1800. https://doi.org/10/f8zrr6.

Karing, Anne. 2018. “Social Signaling and Childhood Immunization: A Field Experiment in Sierra Leone.” Berkeley Working Paper. Berkeley: University of California. https://economics.yale.edu/sites/default/files/jmp_socialsignaling.pdf.

Kawakatsu, Yoshito, Tomohiko Sugishita, Kennedy Oruenjo, Stephen Wakhule, Kennedy Kibosia, Eric Were, and Sumihisa Honda. 2015. “Effectiveness of and Factors Related to Possession of a Mother and Child Health Handbook: An Analysis Using Propensity Score Matching.” Health Education Research 30 (October): 935–46. https://doi.org/10/f749wb.

Kazi, Abdul Momin, Murtaza Ali, Khurram Zubair, Hussain Kalimuddin, Abdul Nafey Kazi, Saleem Perwaiz Iqbal, Jean-Paul Collet, and Syed Asad Ali. 2018. “Effect of Mobile Phone Text Message Reminders on Routine Immunization Uptake in Pakistan: Randomized Controlled Trial.” JMIR Public Health and Surveillance 4 (1). https://doi.org/10/ghpwd3.

Kern, Ana Paula, Marcel de Toledo Vieira, and Ricardo da Silva Freguglia. 2018. “Impactos Do Programa Bolsa Família Na Imunização Das Crianças.” Working paper. https://www.anpec.org.br/encontro/2018/submissao/files_I/i12-cb6dfc0f070c8ca9e77abc8563c3801f.pdf.

Khaleghian, Peyvand. 2003. “Decentralization and Public Services: The Case of Immunization.” Policy Research Working Paper 2989. Washington: The World Bank. https://openknowledge.worldbank.org/handle/10986/19159?show=full.

For peer review only - http://bmjopen.bmj.com/site/about/guidelines.xhtml

Kusuma, Dian, Hasbullah Thabrany, Budi Hidayat, Margaret McConnell, Peter Berman, and Jessica Cohen. 2017. "New Evidence on the Impact of Large-Scale Conditional Cash Transfers on Child Vaccination Rates: The Case of a Clustered-Randomized Trial in Indonesia." World Development 98 (October): 497–505. https://doi.org/10/ghpwd4.

Lamanna, Camillo, and Lauren Byrne. 2019. "A Pilot Study of a Novel, Incentivised MHealth Technology to Monitor the Vaccine Supply Chain in Rural Zambia." Pan African Medical Journal 33. https://doi.org/10/ghpwp5.

Levy, Dan, and Jim Ohls. 2010. "Evaluation of Jamaica's PATH Conditional Cash Transfer Programme." Journal of Development Effectiveness 2 (4): 421–41. https://doi.org/10/dtkp26.

Lin, Meng Ann. 2016. "Stimulating Demand: An Assessment of the Conditional Cash Transfer Project in Afghanistan." PhD Thesis, Baltimore: Johns Hopkins University. https://jscholarship.library.jhu.edu/handle/1774.2/39740.

Liu, Yong, Zhaokang Yuan, Yuxi Liu, Upali W Jayasinghe, and Mark F Harris. 2014. "Changing Community Health Service Delivery in Economically Less-Developed Rural Areas in China: Impact on Service Use and Satisfaction." BMJ Open 4 (2). https://doi.org/10/f6mwb9.

Loevinsohn, B P, and E Gareaballah. 1992. "Missed Opportunities for Immunization during Visits for Curative Care: A Randomized Cross-over Trial in Sudan." Bulletin of the World Health Organization 70 (3): 335–39. https://www.ncbi.nlm.nih.gov/pmc/articles/PMC2393287/.

Loevinsohn, Benjamin P., and Michael E. Loevinsohn. 1986. "Improvement in Coverage of Primary Health Care in a Developing Country through Use of Food Incentives." The Lancet 327 (8493): 1314–16. https://doi.org/10/bjgw7d.

Looij, Frank van de, Dudzai Mureyi, Chenjerai Sisimayi, Jaap Koot, Portia Manangazira, and Nyasha Musuka. 2015. "Early Evidence from Results-Based Financing in Rural Zimbabwe." African Health Monitor 6: 32–36. https://collaboration.worldbank.org/content/sites/collaboration-for-development/en/groups/results-based-financing/groups/oba-rbf-newsroom/documents.entry.html/2016/01/14/early_evidence_from-Kgih.html.

Maluccio, John A., and Rafael Flores. 2005. "Impact Evaluation of a Conditional Cash Transfer Program the Nicaraguan Red de Protección Social." Research report 141. 0 ed. Washington, DC: International Food Policy Research Institute. https://doi.org/10.2499/0896291464RR141.

Mampe, Tumelo, Helen Schneider, and Gavin Reagon. 2016. "Effectiveness of Ward Based Outreach Teams in the North West Province: An Evaluation." Cape Town: University of the Western Cape. http://rgdoi.net/10.13140/RG.2.2.36191.66725.

Masuda, Kazuya, and Chikako Yamauchi. 2020. "How Does Female Education Reduce Adolescent Pregnancy and Improve Child Health? Evidence from Uganda's Universal Primary Education for Fully Treated Cohorts." The Journal of Development Studies 56 (1): 63–86. https://doi.org/10/ggv6vq.

Mathanga, Don P., Elizabeth T. Luman, Carl H. Campbell, Chimwemwe Silwimba, and Grace Malenga. 2009. "Integration of Insecticide-Treated Net Distribution into Routine Immunization Services in Malawi: A Pilot Study." Tropical Medicine & International Health 14 (7): 792–801. https://doi.org/10/b44rkh.

Mazumder, S., S. Taneja, R. Bahl, P. Mohan, T. A. Strand, H. Sommerfelt, B. R. Kirkwood, et al. 2014. "Effect of Implementation of Integrated Management of Neonatal and Childhood Illness Programme on Treatment Seeking Practices for Morbidities in Infants: Cluster Randomised Trial." BMJ 349 (August). https://doi.org/10/gb3snq.

McMahon, Shannon A., Stephan Brenner, Julia Lohmann, Christopher Makwero, Aleksandra Torbica, Don P. Mathanga, Adamson S. Muula, and Manuela De Allegri. 2016. "Evaluating Complex Health Financing Interventions: Using Mixed Methods to Inform Further Implementation of a Novel PBI Intervention in Rural Malawi." BMC Health Services Research 16 (1). https://doi.org/10/f8zjkr.

Mehta, Kala M., Francois Rerolle, Sonali V. Rammohan, Davis C. Albohm, George Muwowo, Heidi Moseson, Lesley Sept, Hau L. Lee, and Eran Bendavid. 2016. "Systematic Motorcycle Management and Health Care Delivery: A Field Trial." American Journal of Public Health 106 (1): 87–94. https://doi.org/10/f8hd4m.

For peer review only - http://bmjopen.bmj.com/site/about/guidelines.xhtml

Mensah, Joseph, Joseph R Oppong, and Christoph M Schmidt. 2009. "Ghana's National Health Insurance Scheme in the Context of the Health MDGs: An Empirical Evaluation Using Propensity Score Matching." Ruhr Economic Papers 157. https://papers.ssrn.com/sol3/papers.cfm?abstract_id=1532169.

Mohan, Pavitrra, Baya Kishore, Sharad Singh, Rajiv Bahl, Anju Puri, and Rajesh Kumar. 2012. "Assessment of Implementation of Integrated Management of Neonatal and Childhood Illness in India." Journal of Health, Population and Nutrition 29 (6): 629–38. https://doi.org/10/ghpwft.

Musa, O I, D B Parakoyi, and A A Akanbi. 2006. "Evaluation of Health Education Intervention on Safe Immunization Injection among Health Workers in Ilorin, Nigeria." Annals of African Medicine 5 (3): 122–28. https://www.semanticscholar.org/paper/Evaluation-of-Health-Education-Intervention-on-Safe-Musa-Parakoyi/5f3eaa0090f82149c7de44c442ec094d31a04591?p2df.

Nanyunja, Miriam, Rosamund F. Lewis, Issa Makumbi, Rachel Seruyange, Eva Kabwongera, Possy Mugyenyi, and Ambrose Talisuna. 2003. "Impact of Mass Measles Campaigns among Children Less than 5 Years Old in Uganda." The Journal of Infectious Diseases 187 (s1): S63–68. https://doi.org/10/bzksr6.

Nasir, Narila Mutia, Yuli Amran, and Yasuhide Nakamura. 2017. "Changing Knowledge and Practices of Mothers on Newborn Care through Mother Class: An Intervention Study in Indonesia." Journal of Tropical Pediatrics 63 (6): 440–46. https://doi.org/10/gghf3q.

Onishi, Junko. 2014. "Philippines Conditional Cash Transfer Program Impact Evaluation 2012." World Bank Report 75533-PH. Washington, DC: The World Bank. https://assessments.hpc.tools/sites/default/files/assessments/Philippines%20Conditional%20Cash%20Transfer%20Program%2C%20Impact%20Evaluation%202012.pdf.

Owais, Aatekah, Beenish Hanif, Amna R Siddiqui, Ajmal Agha, and Anita KM Zaidi. 2011. "Does Improving Maternal Knowledge of Vaccines Impact Infant Immunization Rates? A Community-Based Randomized-Controlled Trial in Karachi, Pakistan." BMC Public Health 11 (1). https://doi.org/10/bpswbk.

Özer, Mustafa, Jan Fidrmuc, and Mehmet Ali Eryurt. 2018. "Maternal Education and Childhood Immunization in Turkey." Health Economics 27 (8): 1218–29. https://doi.org/10/gdkgd3.

Padilla, Alcides de J., and Juan C. Trujillo. 2015. "An Impact Assessment of the Child Growth, Development and Care Program in the Caribbean Region of Colombia." Cadernos de Saúde Pública 31 (10): 2099–2109. https://doi.org/10/gghf3w.

Pandey, Priyanka, Ashwini R. Sehgal, Michelle Riboud, David Levine, and Madhav Goyal. 2007. "Informing Resource-Poor Populations and the Delivery of Entitled Health and Social Services in Rural India: A Cluster Randomized Controlled Trial." JAMA 298 (16): 1867–75. https://doi.org/10/ddxdhz.

Pathak, Yuvraj, and Karen Macours. 2017. "Women's Political Reservation, Early Childhood Development, and Learning in India." Economic Development and Cultural Change 65 (4): 741–66. https://doi.org/10/ghpwf6.

Powell-Jackson, Timothy, Camilla Fabbri, Varun Dutt, Sarah Tougher, and Kultar Singh. 2018. "Effect and Cost-Effectiveness of Educating Mothers about Childhood DPT Vaccination on Immunisation Uptake, Knowledge, and Perceptions in Uttar Pradesh, India: A Randomised Controlled Trial." Edited by James K. Tumwine. PLOS Medicine 15 (3). https://doi.org/10/gc4pqg.

Pramanik, Santanu, Arpita Ghosh, Rituu B. Nanda, Marlou de Rouw, Philip Forth, and Sandra Albert. 2018. "Impact Evaluation of a Community Engagement Intervention in Improving Childhood Immunization Coverage: A Cluster Randomized Controlled Trial in Assam, India." BMC Public Health 18 (1). https://doi.org/10/gdhbjf.

Prinja, Shankar, Ruby Nimesh, Aditi Gupta, Pankaj Bahuguna, Madhu Gupta, and Jarnail Singh Thakur. 2017. "Impact of M-Health Application Used by Community Health Volunteers on Improving Utilisation of Maternal, New-Born and Child Health Care Services in a Rural Area of Uttar Pradesh, India." Tropical Medicine & International Health 22 (7): 895–907. https://doi.org/10/gghf35.

Prosser, Wendy, Philippe Jaillard, Emmanuelle Assy, Shawn T. Brown, Graça Matsinhe, Mawutondji Dekoun, and Bruce Y. Lee. 2017. "System Redesign of the Immunization Supply Chain: Experiences from Benin and Mozambique." Vaccine 35 (17): 2162–66. https://doi.org/10/f96jsd.

For peer review only - http://bmjopen.bmj.com/site/about/guidelines.xhtml

Rahman, Mohammad Mahbubur, and Saseendran Pallikadavath. 2019. “Maternal and Child Health Care Services’ Utilization Data from the Fourth Round of District Level Household Survey in India.” Data in Brief 23 (April). https://doi.org/10/ghpwgm.

Rajkotia, Yogesh, Omer Zang, Pierre Nguimkeu, Jessica Gergen, Iva Djurovic, Paula Vaz, Franscisco Mbofana, and Kebba Jobarteh. 2017. “The Effect of a Performance-Based Financing Program on HIV and Maternal/Child Health Services in Mozambique—an Impact Evaluation.” Health Policy and Planning 32 (10): 1386–96. https://doi.org/10/gcfv5b.

Rasella, Davide, Rosana Aquino, Carlos AT Santos, Rômulo Paes-Sousa, and Mauricio L Barreto. 2013. “Effect of a Conditional Cash Transfer Programme on Childhood Mortality: A Nationwide Analysis of Brazilian Municipalities.” The Lancet 382 (9886): 57–64. https://doi.org/10/f2kfx4.

Reinbold, Gary W. 2019. “Effects of the Convention on the Rights of the Child on Child Mortality and Vaccination Rates: A Synthetic Control Analysis.” BMC International Health and Human Rights 19 (1). https://doi.org/10/ghgdqs.

Robinson, J Stephen, Barton R Burkhalter, Barbie Rasmussen, and Ristianto Sugiono. 2001. “Low-Cost on-the-Job Peer Training of Nurses Improved Immunization Coverage in Indonesia.” Bulletin of the World Health Organization 79 (2): 150–58. https://www.scielosp.org/article/bwho/2001.v79n2/150-158/en/.

Roth, A. E., C. Stabell Benn, H. Ravn, A. Rodrigues, I. M. Lisse, M. Yazdanbakhsh, H. Whittle, and P. Aaby. 2010. “Effect of Revaccination with BCG in Early Childhood on Mortality: Randomised Trial in Guinea-Bissau.” BMJ 340 (mar15 1). https://doi.org/10/c8q74b.

Rusa, Louis, Jean de Dieu Ngirabega, Willy Janssen, Stefaan Van Bastelaere, Denis Porignon, and Werner Vandenbulcke. 2009. “Performance-Based Financing for Better Quality of Services in Rwandan Health Centres: 3-Year Experience.” Tropical Medicine & International Health 14 (7): 830–37. https://doi.org/10/cc5zvh.

Ryman, Tove K., Elizabeth C. Briere, Emily Cartwright, Karen Schlanger, Kathleen A. Wannemuehler, Elizabeth T. Russo, Steve Kola, et al. 2012. “Integration of Routine Vaccination and Hygiene Interventions: A Comparison of 2 Strategies in Kenya.” The Journal of Infectious Diseases 205 (suppl_1): S65–76. https://doi.org/10/f3wkpb.

Ryman, Tove K., Ajay Trakroo, Aaron Wallace, Satish Kumar Gupta, Karen Wilkins, Pankaj Mehta, and Vance Dietz. 2011. “Implementation and Evaluation of the Reaching Every District (RED) Strategy in Assam, India, 2005–2008.” Vaccine 29 (14): 2555–60. https://doi.org/10/bjqf85.

Salami, Lamidhi, Edgard-Marius Dona Ouendo, and Benjamin Fayomi. 2016. “Effects of Results Based Financing Models on Data Quality Improvement in Benin on 2014.” Universal Journal of Public Health 4 (6): 324–31. https://doi.org/10/ghpwgc.

Salami, Lamidhi, Edgard-Marius Ouendo, and Benjamin Fayomi. 2018. “Effects of Results Based Financing Models on the Performance of Exposed Health Zones in Benin.” International Journal Of Community Medicine And Public Health 5 (10): 4188–99. https://doi.org/10/ghpwgd.

Sangwan, Santosh, and Anju Manocha. 2009. “Maternal Knowledge and Child Health.” Journal of Human Ecology 25 (1): 51–54. https://doi.org/10/gghf33.

Sato, Ryoko, and Abdullahi Belel. 2020. “The Effect of Performance-Based Financing on Child Vaccinations in Northern Nigeria.” Vaccine 38 (9): 2209–15. https://doi.org/10/ghpwgf.

Schlumberger, M., A. Bamoko, T.M. Yaméogo, F. Rouvet, R. Ouedraogo, B. Traoré, M. Tinto, et al. 2015. “Impact positif sur le Programme élargi de vaccinations de l’envoi de SMS de rappel à partir d’un registre informatisé, Bobo-Dioulasso (Burkina Faso).” Bulletin de la Société de pathologie exotique 108 (5): 349–54. https://doi.org/10/gghf34.

Sengupta, Nandana, and Aakanksha Sinha. 2018. “Is India’s Safe Motherhood Scheme Leading to Better Child Health Care Practices?” Global Social Welfare 5 (1): 49–58. https://doi.org/10/ghpwgg.

Shei, Amie, Federico Costa, Mitermayer G Reis, and Albert I Ko. 2014. “The Impact of Brazil’s Bolsa Família Conditional Cash Transfer Program on Children’s Health Care Utilization and Health Outcomes.” BMC International Health and Human Rights 14 (1). https://doi.org/10/gbf36j.

For peer review only - http://bmjopen.bmj.com/site/about/guidelines.xhtml

Sherry, Tisamarie B., Sebastian Bauhoff, and Manoj Mohanan. 2017. "Multitasking and Heterogeneous Treatment Effects in Pay-for-Performance in Health Care: Evidence from Rwanda." American Journal of Health Economics 3 (2): 192–226. https://doi.org/10/ghpwgh.

Shuaib, Waqas, Julia Marielly Suarez, Juan David Romero, Carlos Dillon Pamello, Richard Alweis, Aizaaz Ali Khan, Syed Raza Shah, Hassan Shahid, Serge B. PierreCharles, and Laura Rosemary Sanchez. 2016. "Transforming Patient Care by Introducing an Electronic Medical Records Initiative in a Developing Country." Health Informatics Journal 22 (4): 975–83. https://doi.org/10/gghf36.

Sinha, Nistha, and Joanne K Yoong. 2009. "Long-Term Financial Incentives and Investment in Daughters: Evidence from Conditional Cash Transfers in North India." RAND Labor and Population working paper series WR-667. Santa Monica: RAND Corporation. https://doi.org/10.1596/1813-9450-4860.

Soeters, Robert, Peter Bob Peerenboom, Pacifique Mushagalusa, and Célestin Kimanuka. 2011. "Performance-Based Financing Experiment Improved Health Care in the Democratic Republic of Congo." Health Affairs 30 (8): 1518–27. https://doi.org/10/c6w2rq.

Steinhardt, L. C., I. Aman, I. Pakzad, B. Kumar, L. P. Singh, and D. H. Peters. 2011. "Removing User Fees for Basic Health Services: A Pilot Study and National Roll-out in Afghanistan." Health Policy and Planning 26 (Suppl. 2): ii92–103. https://doi.org/10/drchbp.

Subedi, Sudarshan, Bishnu Prasad Sharma, and Chiranjivi Adhikari. 2018. "Cost and Consequences of Integrated Public Health Campaign in Baglung District, Nepal." Economic Journal of Development Issues 23 & 24 (January): 113–22. https://doi.org/10/ghpwgk.

Talukder, Md., Ubaidur Rob, Syed Abu Musa, Ashish Bajracharya, Kaji Keya, Forhana Noor, Eshita Jahan, Md. Hossain, Jyotirmoy Saha, and Benjamin Bellows. 2014. "Evaluation of the Impact of the Voucher Program for Improving Maternal Health Behavior and Status in Bangladesh." Dhaka: Population Council. https://doi.org/10.31899/rh10.1000.

Tanner, Jeffery, Ryotaro Hayashi, and Yunsun Li. 2015. "Improving Coverage and Utilization of Maternal and Child Health Services in Lao PDR: Impact Evaluation of the Community Nutrition Project." IEG Working Paper 99929. Washington: The World Bank Group. https://documents.worldbank.org/en/publication/documents-reports/documentdetail/441841468187767237/improving-coverage-and-utilization-of-maternal-and-child-health-services-in-lao-pdr-impact-evaluation-of-the-community-nutrition-project.

Thomas, Ranjeeta Alice. 2011. "Essays on Ex Ante Evaluations of Cash Transfer Programs." PhD Thesis, York: University of York. http://etheses.whiterose.ac.uk/2004/.

Thomson, Dana R, Cheryl Amoroso, Sidney Atwood, Matthew H Bonds, Felix Cyamatare Rwabukwisi, Peter Drobac, Karen E Finnegan, et al. 2018. "Impact of a Health System Strengthening Intervention on Maternal and Child Health Outputs and Outcomes in Rural Rwanda 2005–2010." BMJ Global Health 3 (2). https://doi.org/10/gdcrw5.

Thysen, Sanne Marie, Stine Byberg, Marie Pedersen, Amabelia Rodrigues, Henrik Ravn, Cesario Martins, Christine Stabell Benn, Peter Aaby, and Ane Bærent Fisker. 2014. "BCG Coverage and Barriers to BCG Vaccination in Guinea-Bissau: An Observational Study." BMC Public Health 14 (1). https://doi.org/10/f6k4xv.

Uddin, Md. Jasim, Md. Shamsuzzaman, Lily Horng, Alain Labrique, Lavanya Vasudevan, Kelsey Zeller, Mridul Chowdhury, Charles P. Larson, David Bishai, and Nurul Alam. 2016. "Use of Mobile Phones for Improving Vaccination Coverage among Children Living in Rural Hard-to-Reach Areas and Urban Streets of Bangladesh." Vaccine 34 (2): 276–83. https://doi.org/10/f766t2.

Unger, Jean-Pierre. 1991. "Can Intensive Campaigns Dynamize Front Line Health Services? The Evaluation of an Immunization Campaign in Thiès Health District, Senegal." Social Science & Medicine 32 (3): 249–59. https://doi.org/10/bc5fn2.

Usman, Hussain R., Saeed Akhtar, Faiza Habib, and Imtiaz Jehan. 2009. "Redesigned Immunization Card and Center-Based Education to Reduce Childhood Immunization Dropouts in Urban Pakistan: A Randomized Controlled Trial." Vaccine 27 (3): 467–72. https://doi.org/10/bcv8fd.

For peer review only - http://bmjopen.bmj.com/site/about/guidelines.xhtml

Usman, Hussain R., Mohammad H. Rahbar, Sibylle Kristensen, Sten H. Vermund, Russell S. Kirby, Faiza Habib, and Eric Chamot. 2011. “Randomized Controlled Trial to Improve Childhood Immunization Adherence in Rural Pakistan: Redesigned Immunization Card and Maternal Education.” Tropical Medicine & International Health 16 (3): 334–42. https://doi.org/10/dm9tm7.

Vaidyanathan, Radha. 2019. “Immunization Coverage among Under-Five Children Living along a School Student through Child-to-Child and Child-to-Parent Information, Education and Communication Strategy.” Indian Journal of Public Health 63 (4): 334–40. https://doi.org/10/ghpwgq.

Van de Poel, Ellen, Gabriela Flores, Por Ir, and Owen O’Donnell. 2016. “Impact of Performance-Based Financing in a Low-Resource Setting: A Decade of Experience in Cambodia.” Health Economics 25 (6): 688–705. https://doi.org/10/f8kzhk.

Varghese, Beena, Reetabrata Roy, Somen Saha, and Sidsel Roalkvam. 2014. “Fostering Maternal and Newborn Care in India the Yashoda Way: Does This Improve Maternal and Newborn Care Practices during Institutional Delivery?” Edited by Hamid Reza Baradaran. PLoS ONE 9 (1). https://doi.org/10/f5s7zr.

Verma, M, J Chhatwal, and P V Varughese. 1995. “Antenatal Period: An Educational Opportunity.” Indian Pediatrics 32: 171–77. https://www.semanticscholar.org/paper/Antenatal-period%3A-an-educational-opportunity.-Verma-Chhatwal/b44c9c679879ada229d8a2719b5637155190f4d1?p2df.

Wadhwa, Sagar. 2019. “Conditional Cash Transfers and Parental Investment in Daughters: Evidence from India.” Paper presented at the Population Association of America 2019 Annual Meeting, Austin, TX. http://paa2019.populationassociation.org/abstracts/193315.

Wagstaff, Adam, and Shengchao Yu. 2007. “Do Health Sector Reforms Have Their Intended Impacts ? The World Bank’S Health VIII Project in Gansu Province, China.” Journal of Health Economics 26: 505–35. https://doi.org/10/cx4kc6.

Wallace, Aaron S., Kenny Peetosutan, Andi Untung, Marisa Ricardo, Prima Yosephine, Kathleen Wannemuehler, David W. Brown, et al. 2019. “Home-Based Records and Vaccination Appointment Stickers as Parental Reminders to Reduce Vaccination Dropout in Indonesia: A Cluster-Randomized Controlled Trial.” Vaccine 37 (45): 6814–23. https://doi.org/10/ghpwgw.

Walque, Damien de, Paul Jacob Robyn, Hamadou Saidou, Gaston Sorgho, and Maria Steenland. 2017. “Looking into the Performance-Based Financing Black Box: Evidence from an Impact Evaluation in the Health Sector in Cameroon.” Policy Research Working Paper 8162. Washington, DC: The World Bank. https://doi.org/10.1596/1813-9450-8162.

Wang, Paul C., Albert Mwango, Sarah Moberley, Benjamin J. Brockman, Alison L. Connor, Penelope Kalesha-Masumbu, Simon Mutembo, et al. 2015. “A Cluster Randomised Trial on the Impact of Integrating Early Infant HIV Diagnosis with the Expanded Programme on Immunization on Immunization and HIV Testing Rates in Rural Health Facilities in Southern Zambia.” Edited by David Joseph Diemert. PLOS ONE 10 (10). https://doi.org/10/f779tz.

Weldemariam, Mesele. 2010. “The Impact of Fiscal Decentralization on Education and Health Outcomes in Ethiopia: A Regional Panel Data Analysis.” Master Thesis, Addis Ababa: Addis Ababa University. http://etd.aau.edu.et/handle/123456789/14217?show=full.

Wong, Benjamin KC, Shaza A Fadel, Shally Awasthi, Ajay Khera, Rajesh Kumar, Geetha Menon, and Prabhat Jha. 2019. “The Impact of Measles Immunization Campaigns in India Using a Nationally Representative Sample of 27,000 Child Deaths.” ELife 8 (March). https://doi.org/10/ghpwgx.

Yoong, Joanne. 2007. “Does Decentralization Hurt Childhood Immunization?” Stanford: Stanford University. http://citeseerx.ist.psu.edu/viewdoc/summary?doi=10.1.1.370.4021.

Yoosuf, Abdul-Sattar. 1993. “Group Learning by Mothers about Primary Health Care.” World Health Forum 14 (1): 20–22. https://pubmed.ncbi.nlm.nih.gov/8439363/.

Zang, Omer, Sebastien Djienouassi, Gaston Sorgho, and Jean Claude Taptue. 2015. “Impact of Performance-Based Financing on Health-Care Quality and Utilization in Urban Areas of Cameroon.” African Health Monitor 7 (21): 10–14. https://indexmedicus.afro.who.int/aim/opac_css/doc_num.php?explnum_id=72209.

For peer review only - http://bmjopen.bmj.com/site/about/guidelines.xhtml

Zeng, Wu, Donald S Shepard, Jean de Dieu Rusatira, Aaron P Blaakman, and Bernice M Nsitou. 2018. "Evaluation of Results-Based Financing in the Republic of the Congo: A Comparison Group Pre–Post Study." Health Policy and Planning 33 (3): 392–400. https://doi.org/10/gcvmpw.

Zeng, Wu, Daxin Sun, Henry Mphwanthe, Tianwen Huan, Jae Eun Nam, Pascal Saint-Firmin, Gerald Manthalu, Suneeta Sharma, and Arin Dutta. 2019. "The Impact and Cost-Effectiveness of User Fee Exemption by Contracting out Essential Health Package Services in Malawi." BMJ Global Health 4 (2). https://doi.org/10/gghf4b.

Zizien, Zawora Rita, Catherine Korachais, Philippe Compaoré, Valéry Ridde, and Vincent De Brouwere. 2019. "Contribution of the Results-based Financing Strategy to Improving Maternal and Child Health Indicators in Burkina Faso." The International Journal of Health Planning and Management 34 (1): 111–29. https://doi.org/10/gd2zrp.

Zombré, David, Manuela De Allegri, and Valéry Ridde. 2020. "No Effects of Pilot Performance-Based Intervention Implementation and Withdrawal on the Coverage of Maternal and Child Health Services in the Koulikoro Region, Mali: An Interrupted Time Series Analysis." Health Policy and Planning 35 (4): 379–87. https://doi.org/10/ggnfpf.

For peer review only

Appendix 5: Qualitative and cost-evidence search strategy

Qualitative search protocol

Purpose

The purpose of this review is to inform policy recommendations related to community engagement interventions to improve immunisation outcomes in low- and middle-income countries. Specifically, we would like to provide insights related to (1) what to do / not do and (2) where to do it / not do it. We want to know not only what works, but why and how.

Our primary, qualitative research question is: What factors relating to programme design, implementation, context, and mechanism are associated with better or worse outcomes along the causal chain? Do these vary by the level of community engagement?

Sub questions are:

1. What pre-intervention activities and characteristics of local context facilitate or inhibit the effectiveness of an intervention?
    a. What characteristics of local context facilitate or inhibit the effectiveness of an intervention? These include but are not limited to feasibility, clinic readiness, acceptability, need, barriers, and demographic composition.
    b. What is the evidence regarding the likelihood that pre-intervention activities, including diagnostics, formative research, and community engagement, affect the effectiveness of an intervention?
2. What is the evidence supporting or refuting the existence of certain steps along the ToC?
    a. Which steps in the causal chain tend to be barriers to or facilitators of impact? Where does the causal chain tend to be interrupted?
    b. What time varying contextual factors affect the relationships seen in the ToC?

In order to respond to these questions, 3ie seeks to identify qualitative papers related to the quantitative papers included in the broader systematic review on effectiveness of community engagement interventions for improving immunisation outcomes. This information will contextualise the results of the quantitative papers. The qualitative papers will be instrumental in expanding our understanding of why and how certain interventions were successful (or not).

This document outlines the suggested approach for identifying these qualitative papers.

Required steps

1. Create a unique dropbox folder for each study using the first author's last name and year as the folder title
2. For each step outlined below, save all potentially relevant documents to this file as they are identified.

    a. Consider this stage to be equivalent to title and abstract screening. If you identify an article that can be quickly discarded as irrelevant (such as budgetary information), there is no need to include. However, if you think that there may be any information related to our research questions, please include at this stage.

    b. If relevant websites are identified that cannot be easily converted to PDF format, create a word document. Write the title of the webpage and provide the link below it.

If a trial registration number for clinicaltrials.gov is provided

For peer review only - http://bmjopen.bmj.com/site/about/guidelines.xhtml

Use this approach if a trial registration number for clinicaltrials.gov is provided. This will be referenced in the abstract and / methods section and appear in a form similar to that found in Robertson et al 2013:

This trial is registered with ClinicalTrials.gov, number NCT00966849.

If no trial registration number is indicated, proceed to the following section (Section 2: If the project has a unique name).

1.1 Go to the website clinialTrials.gov

1.2 Under "find a study" in the field "other terms," enter the trial registration number.

1.3 There should only be one hit. Click on the title under "study title"

1.4 Three tabs should be available under the basic study information. They read "study details," "tabular view," and "study results." Select "study results"

1.5 Scroll to the bottom of the page. Under "more information" there are sections titled "Publication of results" and "other publications"

   a. In most (but not all) cases, there should be a list of publications here. Save the relevant files to the folder

After this, proceed to section 2.

If the project has an unique name

Use this approach if the intervention is named. If a project name is not given in the paper, but the project is registered on clinicaltrials.gov, use the project title provided there.

2.1 Search the project name on google scholar.

   a. Review each search result until you find 5 in a row that are irrelevant.

   b. At this point, stop and proceed to step 2.2.

2.2 If the funder's name is not provided, proceed directly to step 2.3. If the funder's name is provided, search the project name on the funder's website.

   a. Review each search result until you find 5 in a row that are irrelevant.

   b. At this point, stop and proceed to step 2.3.

2.3 If the implementer's name is not provided, proceed directly to section 3. If the implementer's name is provided, search the project name on the implementer's website.

   a. Review each search result until you find 5 in a row that are irrelevant.

Other options

If the trial is not registered and does not have a formal name, the following approaches can be used as a last resort:

For peer review only - http://bmjopen.bmj.com/site/about/guidelines.xhtml

3.1 If funder and / or implementer names are provided, go to their websites. Search for the article on their website. It may link to a program page.

a. If no program page is identified, try to manually search through their website.

3.2 Search the full article name on google and google scholar. Review the top 10 hits on each to see if they provide more information about the program.

If during either of these steps, a program name and / or trial registration number are identified, return to the relevant steps above.

Searching funder and implementer websites

Some of these websites will have good, built-in search functions. If these are useful, use them. However, if the provided search functionality is not adequate, type site:HomepageOfFunderWebsite SearchTerm. For example, if I wanted to search the World Bank website for information about COVID, I would search “site:www.worldbank.org COVID” in google (Note: no space between “site:” and the url). This will provide me hits from the World Bank website related to COVID.

If no linked articles are identified

At the end of each day, email Avantika (abagai@3ieimpact.org) and Charlotte (clane@3ieimpact.org) the title of all papers for which you were unable to identify linked articles.

Cost evidence search protocol

The evaluations included in the cost and cost-effectiveness evidence were drawn from the 61 evaluations that were identified for the systematic review. Since cost reporting and analysis is often not required by donors or included in impact evaluations of global development interventions, we undertook structured outreach to the authors of the 61 included studies to request any additional cost evidence. In case the lead or corresponding author email addresses were nonfunctional, we contacted one of the co-authors.

We successfully contacted 58 out of 61 study authors that reported intervention effectiveness only or intervention effectiveness and partial cost information and requested estimates of the intervention costs as well as any additional raw data files, summary tables, sensitivity analyses, cost evidence, published reports or notes, and non-published documentation, methods or analyses relating to cost-effectiveness. In the first two weeks of this request, our team obtained responses from a total of 25 study authors, of which 12 produced or pointed us in the direction of economic evidence for their respective interventions and 7 authors confirmed that any such costing analysis was not undertaken as part of their study. We excluded any studies that did not report estimates of both costs and effectiveness and identified 22 evaluations with both cost and effectiveness estimates.

List of included qualitative studies

Ministry of Public Health and Sanitation. 2012. National Communication strategy for community health services. Nairobi, Kenya: Ministry of Public Health and Sanitation, Republic of Kenya. https://www.slideshare.net/chskenya/chs-kenya-national-communicationstrategyforcommunityhealthservices20122017

Adamu, Abdu A., O. A. Uthman, M. A. Gadanya, and C. S. Wiysonge. 2020. "Using the consolidated framework for implementation research (CFIR) to assess the implementation context of a quality improvement program to reduce missed opportunities for vaccination in Kano, Nigeria: a mixed methods study." *Hum Vaccin Immunother* 16 (2):465-475. doi: 10.1080/21645515.2019.1654798.

For peer review only - http://bmjopen.bmj.com/site/about/guidelines.xhtml

Anwari, Z., M. Shukla, B. A. Maseed, G. F. Wardak, S. Sardar, J. Matin, G. S. Rashed, S. A. Hamedi, H. Sahak, A. H. Aziz, M. Boyd-Boffa, and R. Trasi. 2015. "Implementing people-centred health systems governance in 3 provinces and 11 districts of Afghanistan: a case study." *Confl Health* 9:2. doi: 10.1186/1752-1505-9-2.

Baba-Ari, F., E. A. Eboreime, and M. Hossain. 2018. "Conditional Cash Transfers for Maternal Health Interventions: Factors Influencing Uptake in North-Central Nigeria." *Int J Health Policy Manag* 7 (10):934-942. doi: 10.15171/ijhpm.2018.56.

Balarajan, Yarlini, and Michael R. Reich. 2016. "Political economy of child nutrition policy: A qualitative study of India's Integrated Child Development Services (ICDS) scheme." *Food Policy* 62:88-98. doi: https://doi.org/10.1016/j.foodpol.2016.05.001.

Billah, S. M., D. E. Hoque, M. Rahman, A. Christou, N. S. Mugo, K. Begum, T. Tahsina, Q. S. Rahman, E. K. Chowdhury, T. M. Haque, R. Khan, A. Siddik, J. Bryce, R. E. Black, and S. El Arifeen. 2018. "Feasibility of engaging "Village Doctors" in the Community-based Integrated Management of Childhood Illness (C-IMCI): experience from rural Bangladesh." *J Glob Health* 8 (2):020413. doi: 10.7189/jogh.08.020413.

Chimhutu, Victor. 2011. "Pay for Performance in Maternal Health in Tanzania: Perceptions, Expectations and Experiences in Mvomero district."

Chimhutu, Victor, Nils Gunnar Songstad, Marit Tjomsland, Mwifadhi Mrisho, and Karen Marie Moland. 2016. "The inescapable question of fairness in Pay-for-performance bonus distribution: a qualitative study of health workers' experiences in Tanzania." *Globalization and Health* 12 (1):77. doi: 10.1186/s12992-016-0213-5.

Chimhutu, Victor, Marit Tjomsland, and Mwifadhi Mrisho. 2019. "Experiences of care in the context of payment for performance (P4P) in Tanzania." *Globalization and Health* 15 (1):59. doi: 10.1186/s12992-019-0503-9.

Chimhutu, Victor, Marit Tjomsland, Nils Gunnar Songstad, Mwifadhi Mrisho, and Karen Marie Moland. 2015. "Introducing payment for performance in the health sector of Tanzania- the policy process." *Globalization and Health* 11 (1):38. doi: 10.1186/s12992-015-0125-9.

Doctor, H. V., S. E. Findley, A. Ager, G. Cometto, G. Y. Afenyadu, F. Adamu, and C. Green. 2012. "Using community-based research to shape the design and delivery of maternal health services in Northern Nigeria." *Reprod Health Matters* 20 (39):104-12. doi: 10.1016/s0968-8080(12)39615-8.

Grayman, Jesse Hession, Siti Ruhanawati, and Nelti Anggraini. 2014. "Opportunities and approaches for better nutrition outcomes through PNPM generasi : a qualitative study."

Henning, M. J., J. M. Zulu, C. Michelo, S. Simmons Zuilkowski, and C. Hubner. 2020. "Adolescent Mothers' Experiences With Community Health Assistants in Rural Zambia." *Int Q Community Health Educ* 40 (4):353-361. doi: 10.1177/0272684x19896737.

Khuzwayo, L. S., and M. Moshabela. 2017. "The perceived role of ward-based primary healthcare outreach teams in rural KwaZulu-Natal, South Africa." *Afr J Prim Health Care Fam Med* 9 (1):e1-e5. doi: 10.4102/phcfm.v9i1.1388.

Lignou, Sapfo, Sushmita Das, Jigna Mistry, Glyn Alcock, Neena Shah More, David Osrin, and Sarah J. L. Edwards. 2016. "Reconstructing communities in cluster trials?" *Trials* 17 (1):166. doi: 10.1186/s13063-016-1284-6.

Marcus, Tessa S., Jannie Hugo, and Champak C. Jinabhai. 2017. "Which primary care model? A qualitative analysis of ward-based outreach teams in South Africa." *African journal of primary health care & family medicine* 9 (1):e1-e8. doi: 10.4102/phcfm.v9i1.1252.

Moosa, S., A. Derese, and W. Peersman. 2017. "Insights of health district managers on the implementation of primary health care outreach teams in Johannesburg, South Africa: a descriptive study with focus group discussions." *Hum Resour Health* 15 (1):7. doi: 10.1186/s12960-017-0183-6.

Okeke, Edward N., Emma Pitchforth, Josephine Exley, Peter Glick, Isa Sadeeq Abubakar, Amalavoyal V. Chari, Usman Bashir, Kun Gu, and Obinna Onwujekwe. 2017. "Going to scale: design and implementation challenges of a program to increase access to skilled birth attendants in Nigeria." *BMC Health Services Research* 17 (1):356. doi: 10.1186/s12913-017-2284-2.

Oladepo, O., I. O. Dipeolu, and O. Oladunni. 2019. "Nigerian rural mothers' knowledge of routine childhood immunizations and attitudes about use of reminder text messages for promoting timely completion." *J Public Health Policy* 40 (4):459-477. doi: 10.1057/s41271-019-00180-7.

For peer review only - http://bmjopen.bmj.com/site/about/guidelines.xhtml

Oladepo, Oladimeji, Isaac Oluwafemi Dipeolu, and Opeyemi Oladunni. 2020. "Outcome of reminder text messages intervention on completion of routine immunization in rural areas, Nigeria." *Health Promotion International*. doi: 10.1093/heapro/daaa092.

Olafsdottir, Anna Elisabet, Iddy Mayumana, Irene Mashasi, Ikunda Njau, Masuma Mamdani, Edith Patouillard, Peter Binyaruka, Salim Abdulla, and Josephine Borghi. 2014. "Pay for performance: an analysis of the context of implementation in a pilot project in Tanzania." *BMC Health Services Research* 14 (1):392. doi: 10.1186/1472-6963-14-392.

Pérez, Myriam Cielo, Dinesh Chandra, Georges Koné, Rohit Singh, Valery Ridde, Marie-Pierre Sylvestre, Aaditeshwar Seth, and Mira Johri. 2020. "Implementation fidelity and acceptability of an intervention to improve vaccination uptake and child health in rural India: a mixed methods evaluation of a pilot cluster randomized controlled trial." *Implementation Science Communications* 1 (1):88. doi: 10.1186/s43058-020-00077-7.

Phiri, S. C., M. L. Prust, C. P. Chibawe, R. Misapa, J. W. van den Broek, and N. Wilmink. 2017. "An exploration of facilitators and challenges in the scale-up of a national, public sector community health worker cadre in Zambia: a qualitative study." *Hum Resour Health* 15 (1):40. doi: 10.1186/s12960-017-0214-3.

Rahayu;, Sri Kusumastuti, Nina Toyamah;, Stella Aleida Hutagalung;, Meuthia Rosfadhila; &, and Muhammad Syukri. 2008. Qualitative Baseline Study for PNPM Generasi and PKH : The Availability and Use of the Maternal and Child Health Services and Basic Education Services in the Provinces of West Java and East Nusa Tenggara. In *Development Economics Working Papers 22536*: East Asian Bureau of Economic Research.

Rahman, A. 2007. "Challenges and opportunities in developing a psychological intervention for perinatal depression in rural Pakistan--a multi-method study." *Archives of women's mental health* 10 (5):211-219. doi: 10.1007/s00737-007-0193-9.

Rahman, Atiya, Margaret Leppard, Sarawat Rashid, Nauruj Jahan, and Hashima E. Nasreen. 2016. "Community perceptions of behaviour change communication interventions of the maternal neonatal and child health programme in rural Bangladesh: an exploratory study." *BMC Health Services Research* 16 (1):389. doi: 10.1186/s12913-016-1632-y.

Robertson, Laura, Phyllis Mushati, Morten Skovdal, Jeffrey W. Eaton, Jeremiah C. Makoni, Tom Crea, Gideon Mavise, Lovemore Dumba, Christina Schumacher, Lorraine Sherr, Constance Nyamukapa, and Simon Gregson. 2014. "Involving Communities in the Targeting of Cash Transfer Programs for Vulnerable Children: Opportunities and Challenges." *World Development* 54:325-337. doi: https://doi.org/10.1016/j.worlddev.2013.09.002.

Sacks, Audrey and Grayman, Jesse Hession. 2018. Indonesia - Long-Term Generasi Qualitative Study (English)

Shah, P., N. Madhiwala, S. Shah, G. Desai, K. Dave, N. Dholakia, S. Patel, S. Desai, and D. Modi. 2019. "High uptake of an innovative mobile phone application among community health workers in rural India: An implementation study." *Natl Med J India* 32 (5):262-269. doi: 10.4103/0970-258x.295956.

Shelley, K. D., Y. W. Belete, S. C. Phiri, M. Musonda, E. C. Kawesha, E. M. Muleya, C. P. Chibawe, J. W. van den Broek, and K. B. Vosburg. 2016. "Implementation of the Community Health Assistant (CHA) Cadre in Zambia: A Process Evaluation to Guide Future Scale-Up Decisions." *J Community Health* 41 (2):398-408. doi: 10.1007/s10900-015-0110-5.

Skovdal, Morten, Phyllis Mushati, Laura Robertson, Shungu Munyati, Lorraine Sherr, Constance Nyamukapa, and Simon Gregson. 2013. "Social acceptability and perceived impact of a community-led cash transfer programme in Zimbabwe." *BMC Public Health* 13 (1):342. doi: 10.1186/1471-2458-13-342.

Skovdal, Morten, Laura Robertson, Phyllis Mushati, Lovemore Dumba, Lorraine Sherr, Constance Nyamukapa, and Simon Gregson. 2013. "Acceptability of conditions in a community-led cash transfer programme for orphaned and vulnerable children in Zimbabwe." *Health Policy and Planning* 29 (7):809-817. doi: 10.1093/heapol/czt060.

Vita, Febriany, Febriany Vita, Toyamah Nina, Toyamah Nina, Sodo Robert Justin, Sodo Robert Justin, Budiyati Sri, and Budiyati Sri. 2011. Qualitative Impact Study for PNPM Generasi and Pkh on the Provision and the

For peer review only - http://bmjopen.bmj.com/site/about/guidelines.xhtml

Utilization of Maternal and Child Health Services and Basic Education Services in the Provinces of West Java and East Nusa Tenggara. In *SMERU Research Institute*: SMERU Research Institute.

Zulu, J. M., J. Kinsman, C. Michelo, and A. K. Hurtig. 2013. "Developing the national community health assistant strategy in Zambia: a policy analysis." *Health Res Policy Syst* 11:24. doi: 10.1186/1478-4505-11-24.

Zulu, Joseph Mumba, John Kinsman, Charles Michelo, and Anna-Karin Hurtig. 2014. "Hope and despair: community health assistants' experiences of working in a rural district in Zambia." *Human Resources for Health* 12 (1):30. doi: 10.1186/1478-4491-12-30.

List of included other studies

Adamu, A. A., O. A. Uthman, M. A. Gadanya, O. O. Adetokunboh, and C. S. Wiysonge. 2019. "A multilevel analysis of the determinants of missed opportunities for vaccination among children attending primary healthcare facilities in Kano, Nigeria: Findings from the pre-implementation phase of a collaborative quality improvement programme." *PLoS One* 14 (7):e0218572. doi: 10.1371/journal.pone.0218572.

Adamu, Abdu Abdullahi. 2019. "Using quality improvement approach to address missed opportunities for vaccination in Kano Metropolis, Nigeria." PhD, Faculty of Medicine and Health Sciences, Stellenbosch University.

Alhassan, Robert Kaba. 2017. "Healthcare quality in Ghana: Improving healthcare quality and health worker motivation to promote sustainable health insurance." PhD, Faculty of Medicine (AMC-UvA), University of Amsterdam.

Banwat, ME., PW; Bupwatd, LA; Lar, AA; Apagu, and AI. Zoakah. 2014. "Effect of peer education on timeliness and completeness of routine immunization: An assessment in rural communities in North-Central Nigeria." *International Journal of Community Research* 3 (3):60-67.

Bekele, Abebe, Mengistu Kefale, and Mekonnen Tadesse. 2016. "Preliminary Assessment of the Implementation of the Health Services Extension Program: The case of Southern Ethiopia." *The Ethiopian Journal of Health Development* 22 (3).

Binyaruka, P., and J. Borghi. 2017. "Improving quality of care through payment for performance: examining effects on the availability and stock-out of essential medical commodities in Tanzania." *Trop Med Int Health* 22 (1):92-102. doi: 10.1111/tmi.12809.

Binyaruka, P., E. Patouillard, T. Powell-Jackson, G. Greco, O. Maestad, and J. Borghi. 2015. "Effect of Paying for Performance on Utilisation, Quality, and User Costs of Health Services in Tanzania: A Controlled Before and After Study." *PLoS One* 10 (8):e0135013. doi: 10.1371/journal.pone.0135013.

Binyaruka, P., B. Robberstad, G. Torsvik, and J. Borghi. 2018a. "Does payment for performance increase performance inequalities across health providers? A case study of Tanzania." *Health Policy Plan* 33 (9):1026-1036. doi: 10.1093/heapol/czy084.

Binyaruka, Peter, and Laura Anselmi. 2020. "Understanding efficiency and the effect of pay-for-performance across health facilities in Tanzania." *BMJ Global Health* 5 (5):e002326. doi: 10.1136/bmjgh-2020-002326.

Binyaruka, Peter, Bjarne Robberstad, Gaute Torsvik, and Josephine Borghi. 2018b. "Who benefits from increased service utilisation? Examining the distributional effects of payment for performance in Tanzania." *International Journal for Equity in Health* 17 (1):14. doi: 10.1186/s12939-018-0728-x.

Borghi, J., R. Little, P. Binyaruka, E. Patouillard, and A. Kuwawenaruwa. 2015. "In Tanzania, the many costs of pay-for-performance leave open to debate whether the strategy is cost-effective." *Health Aff (Millwood)* 34 (3):406-14. doi: 10.1377/hlthaff.2014.0608.

Borkum, Evan;, Anitha; Sivasankaran, Divya; Vohra, Swetha; Sridharan, Dana; Rotz, Lakshmi; Ramnakrishnan, Mercy; Manoranjini, Sukhmani; Sethi, and Anu. Rangarajan. 2015. "Evaluation of the Team-Based Goals and Performance-Based Incentives (TBGI) Intervention in Bihar: Findings from the 30-month Follow-up." *Mathematica Policy Research*.

Carmichael, Suzan L, Kala Mehta, Hina Raheel, Sridhar Srikantiah, Indrajit Chaudhuri, Shamik Trehan, Sunil Mohanty, Evan Borkum, Tanmay Mahapatra, Yingjie Weng, Rajani Kaimal, Anita Sivasankaran, Swetha Sridharan, Dana Rotz, Usha Kiran Tarigopula, Debarshi Bhattacharya, Yamini Atmavilas, Wolfgang Munar, Anu Rangarajan, and Gary L Darmstadt. 2019. "Effects of team-based goals and non-monetary incentives on front-

For peer review only - http://bmjopen.bmj.com/site/about/guidelines.xhtml

line health worker performance and maternal health behaviours: a cluster randomised controlled trial in Bihar, India." *BMJ Global Health* 4 (4):e001146. doi: 10.1136/bmjgh-2018-001146.

Cockcroft, A., N. Andersson, K. Omer, N. M. Ansari, A. Khan, U. U. Chaudhry, and U. Ansari. 2009. "One size does not fit all: local determinants of measles vaccination in four districts of Pakistan." *BMC Int Health Hum Rights* 9 Suppl 1 (Suppl 1):S4. doi: 10.1186/1472-698x-9-s1-s4.

Costa-i-Font, Joan and Parmar, Divya. 2017. Political Agency and Public Health Care: Evidence from India. In *CESifo Working Paper Series*. India: United Nationa University- UNU-WIDER.

Crea, Thomas M., Andrew D. Reynolds, Aakanksha Sinha, Jeffrey W. Eaton, Laura A. Robertson, Phyllis Mushati, Lovemore Dumba, Gideon Mavise, J. C. Makoni, Christina M. Schumacher, Constance A. Nyamukapa, and Simon Gregson. 2015. "Effects of cash transfers on Children's health and social protection in Sub-Saharan Africa: differences in outcomes based on orphan status and household assets." *BMC Public Health* 15 (1):511. doi: 10.1186/s12889-015-1857-4.

Doctor, H. V., and T. Dahiru. 2010. "Utilization of non-skilled birth attendants in northern Nigeria: a rough terrain to the health-related MDGs." *Afr J Reprod Health* 14 (2):37-45.

Doctor, H. V., S. E. Findley, G. Cometto, and G. Y. Afenyadu. 2013. "Awareness of critical danger signs of pregnancy and delivery, preparations for delivery, and utilization of skilled birth attendants in Nigeria." *J Health Care Poor Underserved* 24 (1):152-70. doi: 10.1353/hpu.2013.0032.

Doctor;, Henry V., Sally E. Findley;, Radheshyam Bairagi; and, and Tukur Dahiru. 2011. "Northern Nigeria Maternal, Newborn and Child Health Programme: Selected Analyses from Population-Based Baseline Survey." *The Open Demography Journal* 4:11-21.

Domek, G. J., I. L. Contreras-Roldan, E. J. Asturias, M. Bronsert, G. A. Bolaños Ventura, S. T. O'Leary, A. Kempe, and S. Bull. 2018. "Characteristics of mobile phone access and usage in rural and urban Guatemala: assessing feasibility of text message reminders to increase childhood immunizations." *Mhealth* 4:9. doi: 10.21037/mhealth.2018.03.05.

Domek, G. J., I. L. Contreras-Roldan, S. T. O'Leary, S. Bull, A. Furniss, A. Kempe, and E. J. Asturias. 2016. "SMS text message reminders to improve infant vaccination coverage in Guatemala: A pilot randomized controlled trial." *Vaccine* 34 (21):2437-2443. doi: 10.1016/j.vaccine.2016.03.065.

Domek, G. J., S. T. O'Leary, S. Bull, M. Bronsert, I. L. Contreras-Roldan, G. A. Bolaños Ventura, A. Kempe, and E. J. Asturias. 2018. "Measuring vaccine hesitancy: Field testing the WHO SAGE Working Group on Vaccine Hesitancy survey tool in Guatemala." *Vaccine* 36 (35):5273-5281. doi: 10.1016/j.vaccine.2018.07.046.

Findley, S. E., O. T. Uwemedimo, H. V. Doctor, C. Green, F. Adamu, and G. Y. Afenyadu. 2013. "Comparison of high- versus low-intensity community health worker intervention to promote newborn and child health in Northern Nigeria." *Int J Womens Health* 5:717-28. doi: 10.2147/ijwh.s49785.

Gibson, D. G., B. Ochieng, E. W. Kagucia, D. Obor, F. Odhiambo, K. L. O'Brien, and D. R. Feikin. 2015. "Individual level determinants for not receiving immunization, receiving immunization with delay, and being severely underimmunized among rural western Kenyan children." *Vaccine* 33 (48):6778-85. doi: 10.1016/j.vaccine.2015.10.021.

Grant, Carolyn, Dipty Nawal, Sai Mala Guntur, Manish Kumar, Indrajit Chaudhuri, Christine Galavotti, Tanmay Mahapatra, Kunal Ranjan, Gangesh Kumar, Sunil Mohanty, Mohammed Aftab Alam, Aritra Das, and Safia Jiwani. 2018. "'We pledge to improve the health of our entire community': Improving health worker motivation and performance in Bihar, India through teamwork, recognition, and non-financial incentives." *PLOS ONE* 13 (8):e0203265. doi: 10.1371/journal.pone.0203265.

Mirkuzie Woldie, Sudhakar Narayan Morankar, Garumma T. Feyissa, and Ronald Labonte, and David Sanders. 2015. "Coverage of child health services in rural districts of Ethiopia with the health services extension program." *Journal of Public Health and Epidemiology* 7 (7):223-231. doi: https://doi.org/10.5897/JPHE2015.0733.

Mitchell, Steven, Neil Andersson, Noor Mohammad Ansari, Khalid Omer, José Legorreta Soberanis, and Anne Cockcroft. 2009. "Equity and vaccine uptake: a cross-sectional study of measles vaccination in Lasbela District,

For peer review only - http://bmjopen.bmj.com/site/about/guidelines.xhtml

Pakistan." *BMC international health and human rights* 9 Suppl 1 (Suppl 1):S7-S7. doi: 10.1186/1472-698X-9-S1-S7.

Modi, D., R. Gopalan, S. Shah, S. Venkatraman, G. Desai, S. Desai, and P. Shah. 2015. "Development and formative evaluation of an innovative mHealth intervention for improving coverage of community-based maternal, newborn and child health services in rural areas of India." *Glob Health Action* 8:26769. doi: 10.3402/gha.v8.26769.

Modi, D., J. Patel, S. Desai, and P. Shah. 2016. "Accessing completeness of pregnancy, delivery, and death registration by Accredited Social Health Activists [ASHA] in an innovative mHealth project in the tribal areas of Gujarat: A cross-sectional study." *J Postgrad Med* 62 (3):170-2. doi: 10.4103/0022-3859.183168.

Modi, Dhiren, Somen Saha, Prakash Vaghela, Kapilkumar Dave, Ankit Anand, Shrey Desai, and Pankaj Shah. 2020. "Costing and Cost-Effectiveness of a Mobile Health Intervention (ImTeCHO) in Improving Infant Mortality in Tribal Areas of Gujarat, India: Cluster Randomized Controlled Trial." *JMIR Mhealth Uhealth* 8 (10):e17066. doi: 10.2196/17066.

Moore., Charity. 2008. Accessing Honduras' CCT Programme PRAF, Programa de Asignacion familiar: Expected and unexpected realities. Brazil: International Poverty Centre.

Nagar, Ruchit B. 2016. "Tying Community Engagement With Appropriate Technology At The Last Mile: A Cluster Randomized Trial To Determine The Effectiveness Of A Novel, Digital Pendant And Voice Reminder Platform On Increasing Infant Immunization Adherence Among Mothers In Rural Udaipur, India." School of Public Health, Yale University.

Negusse, H., E. McAuliffe, and M. MacLachlan. 2007. "Initial community perspectives on the Health Service Extension Programme in Welkait, Ethiopia." *Hum Resour Health* 5:21. doi: 10.1186/1478-4491-5-21.

Oduenyi, Chioma, Victor Ordu, and Ugo Okoli. 2019. "Assessing the operational effectiveness of a maternal and child health (MCH) conditional cash transfer pilot programme in Nigeria." *BMC Pregnancy and Childbirth* 19 (1):298. doi: 10.1186/s12884-019-2418-0.

Okeke, Edward, Peter Glick, Amalavoyal Chari, Isa Sadeeq Abubakar, Emma Pitchforth, Josephine Exley, Usman Bashir, Kun Gu, and Obinna Onwujekwe. 2016. "The effect of increasing the supply of skilled health providers on pregnancy and birth outcomes: evidence from the midwives service scheme in Nigeria." *BMC Health Services Research* 16 (1):425. doi: 10.1186/s12913-016-1688-8.

Onwujekwe, Obinna, Tim Ensor, Pamela Ogbozor, Chinyere Okeke, Uche Ezenwaka, Joseph P. Hicks, Enyi Etiaba, Benjamin Uzochukwu, Bassey Ebenso, and Tolib Mirzoev. 2020. "Was the Maternal Health Cash Transfer Programme in Nigeria Sustainable and Cost-Effective?" *Frontiers in public health* 8:582072-582072. doi: 10.3389/fpubh.2020.582072.

Padayachee, Thesandree;, Natasha; Chetty, Muzi; Matse, Tumelo; Mampe, and Helen Schneider. 2013. Progress in the establishment of ward based outreach teams: experiences in the North West Province. University of Western Cape.

Quayyum, Z., M. N. Khan, T. Quayyum, H. E. Nasreen, M. Chowdhury, and T. Ensor. 2013. ""Can community level interventions have an impact on equity and utilization of maternal health care" - evidence from rural Bangladesh." *Int J Equity Health* 12:22. doi: 10.1186/1475-9276-12-22.

Rahman, Mahfuzar, Fatema Tuz Jhohura, Sabuj Kanti Mistry, Tridib Roy Chowdhury, Tanveen Ishaque, Rasheduzzaman Shah, and Kaosar Afsana. 2015. "Assessing Community Based Improved Maternal Neonatal Child Survival (IMNCS) Program in Rural Bangladesh." *PLOS ONE* 10 (9):e0136898. doi: 10.1371/journal.pone.0136898.

Rangarajan;, Anu, Evan Borkum;, Swetha Sridharan;, Dana Rotz;, Mercy Manoranjini;, Seth Morgan;, Lalit Dandona;, Rakhi Dandona;, Priyanka Chaman;, and G. Anil Kumar. 2013. "Baseline Findings from the Ananya Evaluation." *Mathematica Policy Research Reports* 3e3c44fb11b84a07a3063c242.

Robertson, Laura, Phyllis Mushati, Jeffrey W. Eaton, Lorraine Sherr, Jeremiah C. Makoni, Morten Skovdal, Tom Crea, Gideon Mavise, Lovemore Dumba, Christina Schumacher, Shungu Munyati, Constance Nyamukapa, and Simon Gregson. 2012. "Household-based cash transfer targeting strategies in Zimbabwe: are we reaching the

For peer review only

For peer review only - http://bmjopen.bmj.com/site/about/guidelines.xhtml

most vulnerable children?" *Social science & medicine (1982)* 75 (12):2503-2508. doi: 10.1016/j.socscimed.2012.09.031.

Robinson, Victor C. 2006. Summary Report Women's TheRuralMaintenance Program: ACashforWorkProgram.

USAID. 2008. Essential Services for Health in Ethiopia: Final report November 2003- September 2008. Ethiopia: USAID.

UNICEF. 2010. Evaluation report of the community health strategy implementation in Kenya. Division of Community Health Services

Venkat, Preethi. 2016. "Intermediate Assessment Of The Khushi Baby Crct: Implementation Of A Novel Mhealth Solution For Vaccination Record Keeping In Rural Udaipur, Rajasthan, India."

Wakadha, H., S. Chandir, E. V. Were, A. Rubin, D. Obor, O. S. Levine, D. G. Gibson, F. Odhiambo, K. F. Laserson, and D. R. Feikin. 2013. "The feasibility of using mobile-phone based SMS reminders and conditional cash transfers to improve timely immunization in rural Kenya." *Vaccine* 31 (6):987-93. doi: 10.1016/j.vaccine.2012.11.093.

For peer review only

Appendix 6: Data extraction tools

Quantitative data extraction tool

1. VARIABLE LABEL	EXPLANATION
Study ID	This is the study ID - it should match the study ID from the Outcome Mapping Sheet (e.g., SC-SR_1)
Estimate ID	The estimate ID will provide a specific number for each effect size extracted and should include the original study number, underscore, then the unique ID number (e.g., SC-SR1_1, SC-SR1_2 and so on)
Author	For 1 author: leading author last name (e.g., Gomez) For 2 authors: both author last names with ampersand in between (e.g., Smith & Bahn) For 3 or more authors: leading author last name followed by et al. (e.g., Gupta et al.)
Year	Year published
Design	0=Experimental Design (e.g., RCT), 1=Quasi-Experimental Design
How Counterfactual is Chosen?	Free text (e.g., random control trial, propensity score matching, etc) - Multiple codes are ok
Analysis type for this effect size	Free text, what type of analysis was used (Regression, 2SLS, ANCOVA, etc.)- Multiple codes are ok
Country	Country of intervention
Region	Region/continent of intervention
Estimate Type	Type of data for this effect size: 1 = Continuous - means and SDs, 2 = Continuous - mean difference and SD, 3 = Dichotomous outcome - proportions, 4 = Regression data - dichotomous outcome (e.g., logistic regression) 5 = Regression data - continuous outcome (e.g., linear regression)
Comparison	1=No intervention (service delivery as usual), 2=Other intervention, 3=Pipeline (wait-list) control (still service delivery as usual)
Describe Comparison Group	If answer above is (1) no intervention, type N/A, if (2) Other Intervention, list what intervention the control group is receiving, if (3) Pipeline control, report when the control group will receive the intervention in relation to the treatment group (e.g., one year later)
Subgroup	Is this analysis of a subgroup? 0=no, 1=yes

For peer review only - http://bmjopen.bmj.com/site/about/guidelines.xhtml

If yes to subgroup, describe	Free text, describe the subgoup if applicable (e.g., boys, girls). If no subgroup, type N/A
Source	Note the page number, table number, column, and row you used to extract the data
Treatment Effect	1=Intention to Treat (ITT), 2=Average Treatment Effect on the Treated (ATET), 3=Average Treatment Effect (ATE) 4 = Local Average Treatment Effect (LATE)
Intervention	Free text, what is the intervention
Engagement as intervention	1=yes, 0=No
Engagement as intervention: developing community buy-in	1=yes, 0=No
Engagement as intervention: creation of new cadres or health committees	1=yes, 0=No
Engagement as intervention: both	1=yes, 0=No
Engagement in design	1=yes, 0=No
Engagement in design: community decision makinh	1=yes, 0=No
Engagement in design: community feedback	1=yes, 0=No
Engagement in implementation	1=yes, 0=No
Engagement in implementation: governance and decisions	1=yes, 0=No
Engagement in implementation: provision of resources	1=yes, 0=No
Multiple engagement types	1=yes, 0=No
Engagement in design and engagement as intervention	1=yes, 0=No
Engagement in design and implementation	1=yes, 0=No
Engagement in implementation and engagement as intervention	1=yes, 0=No
Exposure to intervention (in months)	How long is the intervention exposure itself?
Evaluation period (in months)	The total number of months elapsed between offering an intervention and the point at which an outcome measure is taken post intervention, or as a follow-up measurement. If less than one month, use decimals (e.g., one week would be .25)

For peer review only - http://bmjopen.bmj.com/site/about/guidelines.xhtml

Post-intervention or change from baseline?	0 = Post-intervention, 1 = Change from baseline
Source of the Outcome data (only use for outcomes in category JAA)	1=Immuniation Card, 2=Recall, 3=Combination of both immunisation card and recall, 4=Health Admin Data, 5=N/A
Author definition of outcome	Free text - How does the author define the outcome?
**OUTCOME CODES**	
**Thinking and Feeling**	Code 1 under any applicable columns. See OUTCOME GUIDANCE document for further explanations.
**Social Processes**	Code 1 under any applicable columns. See OUTCOME GUIDANCE document for further explanations.
**Readiness to Vaccinate**	Code 1 under any applicable columns. See OUTCOME GUIDANCE document for further explanations.
**Practical Factors**	Code 1 under any applicable columns. See OUTCOME GUIDANCE document for further explanations.
**Community Health Workers**	Code 1 under any applicable columns. See OUTCOME GUIDANCE document for further explanations.
**Vaccinators**	Code 1 under any applicable columns. See OUTCOME GUIDANCE document for further explanations.
**IAC Admins**	Code 1 under any applicable columns. See OUTCOME GUIDANCE document for further explanations.
**Health information systems**	Code 1 under any applicable columns. See OUTCOME GUIDANCE document for further explanations.
**Vaccine Availability**	Code 1 under any applicable columns. See OUTCOME GUIDANCE document for further explanations.
**Resources**	Code 1 under any applicable columns. See OUTCOME GUIDANCE document for further explanations.
**Vaccination coverage**	Code 1 under any applicable columns. See OUTCOME GUIDANCE document for further explanations.
**Health Outcomes**	Code 1 under any applicable columns. See OUTCOME GUIDANCE document for further explanations.
**EFFECT SIZE DATA EXTRACTION**	
Reverse Sign (i.e., decrease is good)	Record 0='no' if an increase is good, record 1='yes' if a decrease is good and the sign needs to be reversed.
Unit of analysis	What is the unit of analysis? UOA for this effect size: 1= Individual, 2= Household, 3= Group (e.g. community

For peer review only - http://bmjopen.bmj.com/site/about/guidelines.xhtml

	organisation), 4= Health Center, 5 = Village, 6 = Other, 7 = Not clear
mean_t	Outcome mean for the treatment group
sd_t	Outcome standard deviation for treatment group
mean_c	Outcome mean for the comparison group
sd_c	Outcome standard deviation for control group
mean_overall_diff	Overall mean difference (treatment - control)
diff se	Standard error of the overall mean difference
Diff _t	t-statistic of mean difference
Odds ratio	Odds ratio reported in the study
OR_se	Odds ratio standard error reported in the study
Risk ratio	Risk ratio reported in study
RR_se	Risk ratio standard error
reg_coeff	Report the regression coefficient of the treatment effect
reg_SE	Report the associated standard error of the regression coefficient.
reg_t	Report the associated t statistic of the effect size (coefficient/SE)
Exact p value	Exact p value if given, if not, record as written in the manuscript (e.g., p < .001, or p > .05)
clust_t	Number of clusters - treatment group
clust_c	Number of clusters - control group
clust_T	Number of clusters - total sample
n_t	Sample size - treatment group
n_c	Sample size - control group
n_T	Sample size - total sample
Does the sample size need to be adjusted (if so complete column CL)?	Code as ‘yes’ or no’
periods (1 if cross sectional)	Record how many periods of evaluation there are (e.g., cross section is 1, panel data with 3 measurements is 3)
Treatment Variable	Record the treatment variable as written in the model (e.g., the variable name the author uses, such as ("Intervention x Time")
dataset	Record if data comes from an identified dataset

For peer review only - http://bmjopen.bmj.com/site/about/guidelines.xhtml

coder	Record your name
Notes	Record any notes important for the team
n_T_revised	THIS IS FOR SENIOR QUANT LEAD TO FILL OUT
sp	THIS IS FOR SENIOR QUANT LEAD TO FILL OUT
d	THIS IS FOR SENIOR QUANT LEAD TO FILL OUT
g	THIS IS FOR SENIOR QUANT LEAD TO FILL OUT
var(d)	THIS IS FOR SENIOR QUANT LEAD TO FILL OUT
se(d)	THIS IS FOR SENIOR QUANT LEAD TO FILL OUT
CI_l	THIS IS FOR SENIOR QUANT LEAD TO FILL OUT
CI_u	THIS IS FOR SENIOR QUANT LEAD TO FILL OUT
remove	THIS IS FOR PROJECT MANAGER TO FILL OUT
Formula Used	THIS IS FOR SENIOR QUANT LEAD TO FILL OUT
yi_1	THIS IS FOR SENIOR QUANT LEAD TO FILL OUT
yi_rev	THIS IS FOR SENIOR QUANT LEAD TO FILL OUT
yi	THIS IS FOR SENIOR QUANT LEAD TO FILL OUT
vi	THIS IS FOR SENIOR QUANT LEAD TO FILL OUT
wi	THIS IS FOR SENIOR QUANT LEAD TO FILL OUT
ywi	THIS IS FOR SENIOR QUANT LEAD TO FILL OUT
95ci_lower	THIS IS FOR SENIOR QUANT LEAD TO FILL OUT
95ci_upper	THIS IS FOR SENIOR QUANT LEAD TO FILL OUT
cilow_3sf	THIS IS FOR SENIOR QUANT LEAD TO FILL OUT
cihigh_3sf	THIS IS FOR SENIOR QUANT LEAD TO FILL OUT
ci	THIS IS FOR SENIOR QUANT LEAD TO FILL OUT
wb_yi	THIS IS FOR SENIOR QUANT LEAD TO FILL OUT
Checked	THIS IS FOR EFFECT SIZE RELIABILITY CHECKER TO FILL OUT
ROB Category	THIS IS FOR SENIOR QUANT LEAD OR PM TO FILL OUT

For peer review only

Qualitative data extraction tool

**Name**	**Description**
Causal mechanism	This set of codes describes the causal mechanisms observed or hypothesized
Barriers to immunisation	Select any state reasons that people were not immunised. Try to use previously added codes to reduce possible redundancies, but add codes if needed.
Demand side barriers	Barriers related to low demand for immunisations
Fear	Low demand for immunisation due to fear of side effects or other factors
Financial	Select the financial reason that people were not immunised Add sub-codes as needed.
Cost of immunisation	People were not immunised due to the cost of the immunisation Do not add sub-codes
Cost of transport	People were not immunised due to the cost of transport.
Opportunity costs	People were not immunised due to opportunity costs
Knowledge of Immunisation Schedule	Low demand for immunisation due to limited knowledge of its importance. This includes instances when the person simply does not feel that immunisations are important (regardless of "book knowledge")
Logistics or distance	Barriers related to logistics or distance. For example, it is too far away, I need to arrange for XXX to happen while I am gone...
Self-efficacy	Issues related to not feeling empowered to get vaccinated. This includes not knowing how to go about getting vaccinations. For example, not knowing when or where to get vaccinated
Social norms	Low demand for immunisation due to social pressures and social norms
Understanding of importance	Low demand for immunisation due to limited knowledge of its importance. This includes instances when the person simply does not feel that immunisations are important (regardless of "book knowledge")
Supply side barriers	Limitations to supply that cause problems for immunisation
Availability of services	Services may not be physically present. This includes clinics being closed.
Human resources	Limited human resources may reduce the availability of immunisations. Ex: Appropriate staff were not present to provide the injection
Lack of Infrastructure or supplies	Use code if the lack of immunisation was because there were no drugs, vaccines, cold chain or other items related to physical infrastructure of the health facility

For peer review only - http://bmjopen.bmj.com/site/about/guidelines.xhtml

Poor quality services	Immunisations are not common because there is low quality of service. Providers may not be cooperative, slow, unprofessional/rude, unempathetic, abusive, or inconsistent.
Lack of accountability	Use code for text referring to issues related to accountability, corruption, governance and transparency in providing services.
Lack of Motivation	Use if for text indicating that the health workers are not motivated to perform their duties due to any reason like lack of monetary incentives, recognition, low job satisfaction and other issues.
Baseline conditions and descriptive characteristics	Discussions of the baseline conditions and descriptive characteristics of the region or population in which the intervention took place. This does not necessarily come from a baseline survey, but can be through other sources. Do not add sub-codes
Health systems context	Discussion of the health system functioning and context Example: The clinic served a population of 1,000 people Do not add sub-codes
Immunisation rates	Discussion of baseline immunisation rates. Example: Immunisation rates were low at baseline. Do not add sub-codes
Political context	Discussion of the political context Example: Political unrest made the intervention difficult to implement. Do not add sub-codes
Socioeconomic status and demographics	Discussions of SES and demographics at baseline. Example: The community was mostly farmers. Population size Do not add sub-codes
Facilitators of immunisation	Select any state reasons that people were immunised. Try to use previously added codes to reduce possible redundancies, but add codes if needed.
Demand Side Facilitators	
Favourable population SES characteristics	For instance those from a higher wealth quantile, urban areas or mothers with higher educational qualifications are more likely to get their children immunised. Do not add sub-codes.
Knowledge of immunisation schedule	Use this code if caregivers are aware of all immunisations in the schedule and know which vaccination is due when. Do not add sub-codes.
Lack of fear	Text indicating that there was no fear or vaccine hesitancy. Do not add sub-codes
Lack of financial constraints	Caregivers can afford the cost of immunisation, cost of transportation to visit healthcare facilities, taking children for immunisation does not come at a high opportunity cost for the caregiver. Do not add sub-codes.

For peer review only - http://bmjopen.bmj.com/site/about/guidelines.xhtml

Lack of logistics or distance related challenges	For instance, caregivers were able to access health services because they lived within a 5km radius of a healthcare facility or they received outreach services regularly. Do not add sub-codes.
Self-efficacy	Caregivers feel empowered to get their children vaccinated. For example, they know when and where to get vaccinated. Do not add sub-codes.
Social norms	The social environment owing to prevailing social pressures and norms is encouraging towards child immunisation. For instance, the mother in law in the household or the traditional/religious leaders in the community support immunisation of children. Do not add sub-codes.
Understanding of importance	Caregivers are aware about the importance of immunisation and are willing to get their children vaccinated. For example, caregivers are able to correctly identify vaccine preventable illness and therefore, more likely to get their children immunised. Do not add sub-codes.
Supply-side facilitators	
Availability of infrastructure or supplies	
Good quality of services	
High accountability	
High motivation	
Human resources	
Availability of services	
Impacts	What impacts were ultimately achieved? Use this code for general impacts evaluated through the IE that do not fall into one of the other groups. Do not add sub-codes
Descriptive impacts	Include authors' descriptions of impacts that were not directly measured through the IE. Ex: There was generally an increase over time in most sites
Equity considerations	If impacts on marginalized groups are considered, select the type of marginalized group considered. Do not add sub-codes
Ethnic minority	Impacts on ethnic minorities Do not add sub-codes
Other	Impacts on other marginalized groups, like scheduled castes in India. Do not add sub-codes
Religious minority	Impacts on religious minorities Do not add sub-codes
Women	Impacts on women Do not add sub-codes

For peer review only - http://bmjopen.bmj.com/site/about/guidelines.xhtml

Long term impacts	Impact measured over a timeframe of more than 5 years from the end of the intervention Do not add additional sub-codes
Unintended impacts	Only use this sub-code if the impact is stated as unintended. Do not add sub-codes
Reasons for project success or failure	Select text in which authors discuss the reasons for the success or failure of the project. This could be related to intervention design, implementation, or the research approach. Example: The intervention was not successful due to a lack of political will. Do not add sub-codes
Failure	
Implementation or scale-up challenges	
Competing priorities of health workers	
Other delays, disruptions or implementation variability	
Payment delays	
Intervention	
Design - Engagement	
Design - other	
Duration, frequency and exposure or reach	
Not accounting for existing constraints or uncontrollable contextual trends	
Caregiver competing priorities	
Civil unrest or political instability or natural calamity	
Fear of AEFI	
Health system issues	
Health service access or availability	
Poor quality of service or infrastructure	Also includes issues related to health worker demotivation, recruitment or retention.

For peer review only - http://bmjopen.bmj.com/site/about/guidelines.xhtml

High baseline coverage	The interventions are not geared to/designed to achieve last mile coverage or breakthrough stagnating rates.
Lack of awareness or understanding	
Migration	
Resource constraints or scarcity	Infrastructure, electricity, cellular network, etc.
Social norms	
Wider socio-economic or health-related progress	
Study design	
Contamination	
Other methodological shortcomings	
Mixed results	
Success	
Existing or changing favourable factors or contextual trends	
Health system enablers	
Good quality services and infrastructure	Includes enablers like good outreach, service monitoring, etc.
Health service access and availability	
Self efficacy, social norms and awareness	
Socio-economic factors	
Implementation improvements	
Intervention features	
Community engagement	
Behaviour change communication	
Community dialogues	

For peer review only

Community involvement in planning and implementation	
Incentives	
Needs assessments, pilots or stakeholder consultations	
Other types of engagement	
Customisation to local context	
Health system integration and organisational structure	
Health worker training	
Intervention duration, dose and exposure	
Leadership and supportive supervision	
Other design features	
Positive participant or beneficiary views of intervention	
Theory of change	Information related to the ToC. Do not code here, but use sub-codes. Do not add sub-codes.
Assumptions	when discussing a causal chain mechanism, we often make assumptions about what will happen. These assumptions are often implicit. We may print materials with the assumption that people will be able to read them. We may train people with the assumption they speak our language. When these assumptions are stated, use the sub-codes to indicate if these assumptions were validated or not. Do not add sub-codes
Incorrect assumption	This indicates that there was an assumption in the causal chain mechanism and the assumption was not correct. For example, a study assumed that increasing supply would increase immunisations. Here, we are assuming that people want immunisations and that low supply is the problem. If instead, we find that people did not want the immunisations to begin with, the assumption was incorrect and would be indicated here. Do not add sub-codes

For peer review only

For peer review only - http://bmjopen.bmj.com/site/about/guidelines.xhtml

Valid assumption	this indicates that there was an assumption in the causal chain mechanism and the assumption was valid. For example, a study assumed that increasing supply would increase immunisations. Here, we are assuming that people want immunisations and that low supply is the problem. If this proves to be true, the text should be selected here. Do not add sub-codes
Causal chain mechanisms	Descriptions of how change is expected to occur. These causal chains are often represented as flow charts, or at least they can be. Text that reflects a causal chain mechanism will discuss expected actions and reactions. "We did this and we expected that." Do not add sub-codes
Conclusions	Authors conclusions. Any summary of the take-home message of the article. Tends to be "this worked," "this did not work," "in conclusion." Do not add sub-codes
Cost	Discussions of costs, including the costs of the intervention and any form of cost-analysis. Only select key "take-home message." Do not add sub-codes
Data source	For anything related to causal mechanisms, uptake, and uptake and fidelity challenges, indicate what data source provided this information. Do not add additional sub-codes
Author notes or experiences	
FGDs	
Implementer interpretations or experiences	
Individual interviews	
Literature Review	Use this code if the text alludes to existing evidence on the subject
Observations	If the data comes from direct observations. This must be enumerators looking at and noting the state of something. If it is simply "general impressions," use the notes/interpretations or experience codes. Do not add sub-codes
Intervention description	This set of codes describes the intervention
Beneficiary selection	Select relevant information regarding how beneficiaries were selected for the intervention. Note: This is how beneficiaries of the intervention were selected, NOT a research sampling design or how research participants were selected. Do not add sub-codes
Community involvement	Description of how the community was involved in beneficiary selection. Example: A community council decided who was eligible. Do not add sub-codes
Equity considerations	Descriptions of efforts to ensure beneficiary selection was fair and equitable. Example: There was a quota to ensure adequate representation for ethnic minorities Do not add sub-groups

For peer review only - http://bmjopen.bmj.com/site/about/guidelines.xhtml

Other selection		Other descriptions of the selection process and eligibility criteria Do not add sub-codes
Researcher involvement		Description of the researcher involvement in selection, including selection criteria designed for academic analysis Example: If only those who were involved in and RCT received the intervention, then the sampling frame for the RCT would also be how beneficiaries for the intervention were selected. Do not add sub-codes
Community engagement		This set of codes describes community engagement in the interventions
Community mobilization		Select the relevant reference to community mobilization, regardless of how this phrase is used Do not add sub-codes
Absent		Statements that there was no effort at community mobilisation Example: A challenge was that community members were not aware of the intervention activities. Do not add sub-codes
Definitions of community mobilization		The author's definition for community mobilisation Do not add sub-codes
Present - with trust building		Descriptions of community engagement that was done in such a way as to establish trust and buy-in. Example: A local organisation was recruited to inform community members of the intervention and explain its importance. Note: expect significant double coding with "Developing community buy-in, Present" Do not add sub-codes
Present - without trust building		Descriptions of community mobilization that was done without establishing trust and buy-in. Example: Simply informing the community of an intervention Do not add sub-codes
Inclusion criteria		Select the relevant reason this intervention / activity was included in the SR. Inclusion criteria into the research project should fall under "sampling frame." Do not use main code, only use sub-codes Do not add sub-codes
Community engagement as the intervention		Select the way in which community engagement was used as the intervention itself Do not add sub-codes
Developing community buy-in		Descriptions of activities whose primary purpose was the establishment of community buy-in / trust Do not add sub codes
Absent		Explicit statements that efforts to establish community buy-in / trust were not made should be assigned this code Example: Authors state that the intervention proceeded without engaging the community Do not add sub-codes
Present		If there were activities whose primary purpose was to establish community buy in or trust, select this code Example: Meetings with local leaders to get their support Note: expect significant double coding with "community mobilization, present - with trust building" Do not add sub-codes
New cadres		Description of the development of new cadres of community based structures or systems for health outreach Examples: Developing community health workers, health volunteers, or the establishment of committees Do not add sub-codes

For peer review only - http://bmjopen.bmj.com/site/about/guidelines.xhtml

Community engagement in designing the intervention	Select the type of community engagement that was sought before on the design of the intervention Do not add sub-codes
Absent	The community was not given the opportunity to provide feedback or make decisions on the design of the intervention Example: Explicit statements that the community was not consulted on the design of the intervention Do not add sub-codes
Community decision making	The community was given the opportunity to make decisions regarding the design of an intervention. This could vary from taking iterative feedback from the community to consensus building on the design of the intervention to the design being community led
Absent	The community did not have decision making power. Example: The intervention design was established a priori and not subject to change Do not add sub-codes
Community led	The community had the ultimate decision-making power and / or the intervention design was community led. Example: The community identified which barriers would be targeted and developed an action plan Do not add sub-codes
Partial	The community had some decision-making power Example: Decisions were made through collaboration between the community, implementers, and / or researchers Do not add sub-codes
Community feedback	The community was given the opportunity to provide feedback on the design of the intervention
Formative evaluation or stakeholder consultation	
Needs assessment	
Pilot	
There was a previous pilot	There was a pilot in which it is explicitly stated that community feedback was taken Do not add sub-codes
This was a pilot	The present study is a pilot in which community feedback is taken
Community engagement in implementation	Communities had some opportunity to affect the implementation of the intervention. For example, community is required to spend resources on building health infrastructure and decides whether the intervention will be implemented in their community or those where community members come together to form governance structures such as health committees . OR The community has some responsibility for making decisions regarding the delivery of an intervention. Do not add sub-codes

For peer review only - http://bmjopen.bmj.com/site/about/guidelines.xhtml

	Governance and decisions	
	Absent	Explicit statements that the community did not have decision making power. Example: Statements that the community was excluded from implementation. This may be stated as a problem during the discussion. Do not add sub-codes
	Present	The community has some form of decision making in the implementation of the intervention. This could include a community led governance structure. Example: There was a community group monitoring the implementation of the intervention. Do not add-sub codes
	Provision of resources	The community provides resources during the implementation Examples: Community provides building materials Do not add sub-codes
Component 1		Each unique component should be coded separately. A unique component is defined as the set of activates that are dependent on one another. Activities within separate components are not dependent on one another. Select the depth of engagement for the initial description of the component but code all subsequent activities to the main code. Example: A sticker based reminder and a digital reminder could each be implemented independently and would be separate components Do not add sub-codes
	Does not include engagement	Descriptions of interventions that involved no community engagement Example: The production of cold chain transport of vaccines Do not add sub-codes
	Includes some engagement	Description of activities that involved some community engagement Example: Health clinics holding vaccination days and posting fliers Do not add sub-codes
	Primary focus is engagement	Description of activities whose primary focus was community engagement Example: The establishment of community health councils Do not add sub-codes
Component 10		
	Does not include engagement	
	Includes some engagement	
	Primary focus is engagement	
Component 2		
	Does not include engagement	
	Includes some engagement	
	Primary focus is engagement	

For peer review only - http://bmjopen.bmj.com/site/about/guidelines.xhtml

Component 3

Does not include engagement

Includes some engagement

Primary focus is engagement

Component 4

Does not include engagement

Includes some engagement

Primary focus is engagement

Component 5

Does not include engagement

Includes some engagement

Primary focus is engagement

Component 6

Does not include engagement

Includes some engagement

Primary focus is engagement

Component 7

Does not include engagement

Includes some engagement

Primary focus is engagement

Component 8

Does not include engagement

Includes some engagement

Primary focus is engagement

Component 9

Does not include engagement

For peer review only - http://bmjopen.bmj.com/site/about/guidelines.xhtml

Includes some engagement	
Primary focus is engagement	
Future directions for implementation	Discussion of next steps, how the program could be re-designed, or suggestions for the future Do not add sub-codes
Intervention exposure or reach	Discussions of the proportion of the eligible population that the intervention tried to reach. For example, the number of people who received the video (regardless of whether it was actually watched). Do not add sub-codes
Objective	Statement of the goal, objective, or target of the intervention Do not add sub-codes
Participant views of the intervention	Text related to how participants perceived the intervention
Negative views	Participants did not view the intervention positively
Positive views	Participants viewed the intervention positively
Personnel implementing the program	Description of who implemented the program Do not add sub-codes
Target group	Description of the target group for this program Note: This could be different from beneficiary selection if (for example) fathers were provided with information to get them to have their wives immunise their children. Do not add additional sub-codes
Uptake	Program uptake is defined as initial engagement with the intervention. This could be attending the first meeting, or expressing interest. Use sub-codes when appropriate and only primary code when the text does not fall into the other two. Do not add additional sub-codes
Assessment of uptake	Description of how uptake was assessed Example: Attendance sheets were used to collect information on attendance at the first meeting. Do not add sub-codes Do not add sub-codes
Description of uptake	Description of uptake Example: Uptake was high, with 80% of eligible women participating in the intervention Do not add sub-codes
Research design	This set of codes describes the research design and provides information on internal and external validity. Do not add sub-codes
Author discussion of external validity	Any explicit discussion by authors Do not add sub-codes
Conflict of interest	Discussion of conflicts of interests. This could include discussions related to the independence of evaluators and data collectors from the implementers and donors. Select the relevant code indicating presence / absence of a conflict Do not add sub-codes Do not add sub-codes

For peer review only - http://bmjopen.bmj.com/site/about/guidelines.xhtml

No	Select any statement directly indicating that there was no conflict of interest Example: The data collection team was hired as external contractors Do not add sub-codes
Unclear	Select any text that makes a potential conflict of interest unclear Do not add sub-codes
Yes	Select any text that indicates a clear conflict of interest Example: The implementers were the data collectors Do not add sub-codes
Data collection	Discussion of data collection. Use main code for general discussions and only use sub-codes as appropriate Do not add sub-codes
Response accuracy	Any measure or discussion of response accuracy Example: We expect that some of our findings may be related to social desirability bias Do not add sub-codes
Retrospective	Text indicating that data collection was retrospective. Include discussion of challenges this may have caused Do not add additional sub-codes
Sampling frame	Descriptions of the sampling frame. Only select text related to how people were ultimately enrolled / selected. Text related to geographic distribution of villages, the method of randomisation (e.g. computer generated vs. paper based), and other details about the preparation for selection is not needed. Example: Women of child bearing age were selected when they presented at the clinic. Do not add sub-codes
Data limitations	Discussions of the limitations to the data that pose challenges (eg. we could not analyse X because of Y) Do not add additional sub-codes
Ethical approval	Statement of ethical approval Do not add sub-codes
Future directions for research	Descriptions of future directions for research Do not add sub-codes
Other limitations	Text related to limitations in the research design that are not data related. Example: Failure in randomisation Example: Failure in randomisation Do not add sub-codes
Uptake and fidelity challenges	This set of codes describes challenges with uptake and fidelity. Most include sub-codes titled "present" and "absent." In each case, present reflects a statement that this challenge occurred and absent reflects a statement that this challenge was not encountered. Add sub-codes if needed
Adherence	Discussion of people (not) completing the intervention activities (eg. stopped attending training sessions). Could involve discussions of variability in engagement with the intervention or discussions of continued engagement with the intervention. Do not add sub-codes
Absent	There were not challenges related to adherence. ie adherence was high

For peer review only

For peer review only - http://bmjopen.bmj.com/site/about/guidelines.xhtml

Present	There were problems with adherence. ie adherence was low
Administrative	Challenges related to record keeping, monitoring, and other administrative activities Do not add sub-codes
Absent	There were not administrative challenges. Example: All paperwork was conducted quickly and on time
Present	There were administrative challenges Ex: Paperwork was finished late and this delayed implementation
Attrition in the research	People did not complete the research study (eg. did not respond to all rounds of data collection Do not add sub-codes
Absent	There was not attrition in the research study. ie attrition was low
Present	There was significant attrition in the research study. ie attrition was high
Budget	Challenges due to budget limitations Do not add sub-codes
Budget limitations in the intervention	The intervention could not be implemented in a desired way due to budget limitations Do not add sub-codes
Absent	There were not budgetary limitations to the intervention. Ex: Due to the generous funding of our donor, we were able to.....
Present	There were budgetary limitations to the intervention Ex: We had planned X, but due to budget limitations has to Y
Budget limitations in the research study	The research study could not be implemented as desired due to budget limitations Do not add sub-codes
Absent	There were not budgetary limitations to the research study Ex: Due to the generous funding of our donor, we were able to ......
Present	There were budgetary constraints in the research study Ex: We were not able to collect X data due to limited budget
Contamination	Select the appropriate type of contamination described Do not add sub-codes
Contamination between intervention and control	The control group was incidentally exposed to the intervention. Example: Nearby villages were aware of education campaigns conducted.
Absent	Explicit statements that this contamination did not occur Do not add sub-codes
Present	Contamination between intervention and control occurred
Contamination by other programs	Other ongoing programs may have affected results Do not add additional sub-codes
Absent	There was not contamination by other programs Ex: No other similar programs were functioning in the area
Present	There was contamination by other programs Ex: The Red Cross was conducting a similar program in control villages at the time of our intervention

For peer review only - http://bmjopen.bmj.com/site/about/guidelines.xhtml

Hawthorn effects	Any description of the change in behaviour (real or reported) that is the result of people knowing they are being observed, without any real intent to adopt or maintain these behaviours. Example: Respondents in the UCT group may have been affected by the evaluation process: community awareness about the aims of the project could have affected actual or reported behaviours. Do not add sub-codes
Absent	Hawthorn effects were not observed or expected Ex: By using an enumeration team that was unaffiliated with the intervention, we expect to have reduced the chances that people adjusted their behaviour simply due to the enumerator's presence
Present	Hawthorn effects were observed or expected Ex: Due to significant marketing around the desirability of certain behaviours, people may have changed how they acted in front of enumerators without actually adopting certain behaviours when they were not observed.
Low implementation fidelity	Deviations in implementation of the intervention from what was planned. Note: this must be from what was planned, not simply what would be considered desirable Do not add sub-codes
Absent	Fidelity challenges were not present. The intervention was implemented as intended. Ex: The intervention was implemented as intended
Present	The intervention was not implemented as intended. Ex: Due to confusion among facilitators, some changed the order in which material was presented.
Mobilization	Challenges related to mobilizing people to participate Do not add sub-codes
Mobilizing in the intervention	People were not willing to engage with the intervention Do not add sub-codes
Absent	Low participation was not a challenge observed. ie participation was high.
Present	The challenge of low participation was encountered. ie participation was low
Mobilizing in the research	Challenges getting those who participated in the intervention to join the research project. (eg. refuse to be interviewed) Do not add sub-codes
Absent	Low participation was not a challenge observed. ie participation was high.
Present	The challenge of low participation was encountered. ie participation was low
Other Challenges	

For peer review only

For peer review only - http://bmjopen.bmj.com/site/about/guidelines.xhtml

Cost data extraction tool

Study Characteristics	Variable Label	Explanation	Source
**Coder**			
Study ID	Study ID	This is the study ID - it should match the study ID from the Outcome Mapping Sheet (e.g., SC-SR_1)	Data extraction tool_immunisation
	Study ID Component		
Year	Year	Year published	Data extraction tool_immunisation
Design	Design	0=Experimental Design (e.g., RCT), 1=Quasi-Experimental Design	Data extraction tool_immunisation
Country	Country	Country of intervention	Data extraction tool_immunisation
Study Link			
Which Vaccines were studied?			
How many vaccine doses were delivered?			
Source of info about vaccines			
Outcomes	[Source from CEA Inventory]		
Target population			
Implementing partners		What organization(s) carried out the intervention? [Please list names of organization(s) that implemented the program, e.g. IRC, Government of Uganda. Etc)]	Studies
Analytical perspective		Was the perspective of the costing stated explicitly? [Yes, No]. Analytical perspective is the choice of who has standing in the costing and determines whose costs and benefits will be counted. Perspective should be stated explicitly in the costing, so here it is ok to do a kw search for "perspective" to check	Studies
Analytical perspective		What is the analytical perspective of the costing (i.e. donor, financial cost, economic or social perspective).	Studies
Treatment arms		How many treatment arms were included in the study [# of treatment arms]	
Comparison	Comparison	1=No intervention (service delivery as usual), 2=Other intervention, 3=Pipeline (wait-list) control (still service delivery as usual)	Data extraction tool_immunisation
Describe Comparison Group	Describe Comparison Group	If answer above is (1) no intervention, type N/A, if (2) Other Intervention, list what intervention the control group is receiving, if (3) Pipeline control, report when the control group will receive the intervention in relation to the treatment group (e.g., one year later)	Data extraction tool_immunisation
Subgroup	Subgroup	Is this analysis of a subgroup? 0=no, 1=yes	Data extraction tool_immunisation

For peer review only - http://bmjopen.bmj.com/site/about/guidelines.xhtml

If yes to subgroup, describe	If yes to subgroup, describe	Free text, describe the subgoup if applicable (e.g., boys, girls). If no subgroup, type N/A	Data extraction tool_immunisation
Source	Source	Note the page number, table number, column, and row you used to extract the data	Data extraction tool_immunisation
Treatment Effect	Treatment Effect	1=Intention to Treat (ITT), 2=Average Treatment Effect on the Treated (ATET), 3=Average Treatment Effect (ATE) 4 = Local Average Treatment Effect (LATE)	Data extraction tool_immunisation
Summary of intervention	Summary of intervention	Free text, what is the intervention	Intervention_community engagement_dataset [col E]
Component #	Component #	List in numbers 1-6	Intervention_community engagement_dataset [col H]
Description	Description	Description [Col I in Intervention_community engagement_dataset]	Intervention_community engagement_dataset [col I]
Notes	Notes	Description [Col S in Intervention_community engagement_dataset]	Intervention_community engagement_dataset [col S]
Exposure to intervention (in months)	Exposure to intervention (in months)	How long is the intervention exposure itself?	Data extraction tool_immunisation
Evaluation period (in months)	Evaluation period (in months)	The total number of months elapsed between offering an intervention and the point at which an outcome measure is taken post intervention, or as a follow-up measurement. If less than one month, use decimals (e.g., one week would be .25)	Data extraction tool_immunisation
Post-intervention or change from baseline?	Post-intervention or change from baseline?	0 = Post-intervention, 1 = Change from baseline	Data extraction tool_immunisation
Source of the Outcome data (only use for outcomes in category JAA)	Source of the Outcome data (only use for outcomes in category JAA)	1=Immunisation Card, 2=Recall, 3=Combination of both immunisation card and recall, 4=Health Admin Data, 5=N/A	Data extraction tool_immunisation
Author definition of outcome	Author definition of outcome	Free text - How does the author define the outcome?	Data extraction tool_immunisation
Activities		What are the main (non-evaluation) activities undertaken by the project? (e.g. program design, targeting, community outreach, training of trainers, training of beneficiaries, M&E, etc.). Describe	Studies
Activities Data Source		Was the source of descriptive information about program activities taken from a table or taken from text? [Text, Table, Other(describe)]	Studies
Timeline		Is an intervention timeline included with the report? [Yes, No, If yes, page #]	Studies
Ingredients (unit cost of ingredients)		Is a list of cost ingredients or inputs for CEA, CBA, or CUA provided? [Yes, No]	Studies

For peer review only - http://bmjopen.bmj.com/site/about/guidelines.xhtml

		Ingredients will be items like "personnel", "staff", "travel", "office support", "program materials", etc. Note that searches for the unit costs of activities should include text searches as well as searches of tables.	
Are unit costs reported?		Look for a table with units or unit costs reported. Unit costs give the value of individual items that were used during the intervention, for example the unit cost of a staff person, e.g. a "health specialist" would be given in terms of monthly wages, or compensation or salary. Note that searches for the unit costs of activities should include text searches as well as searches of tables. [Yes, No, If Yes Page #]	Studies
Cost data sources		Was a source of cost data reported? (i.e. financial reports or accounting systems of implementing NGOs or donors (expenditures), NGO or donor (budgets), (market prices) Describe (or copy from text). Clarify - expenditure is what's actually spent, budget is what's planned for spending / not audited, often incorrect and not updated.	Studies
Costing methodology		Was a method of costing described? (i.e. key works: gross costing, micro-costing, ingredients method, activity-based costing method) [if yes, what was the method].	
Type of efficiency analysis (see glossary)		Indicate (CBA/CEA/CUA/cost-minimization, or innovative approaches: Social Return on Investment (SROI), Multi-Criteria Appraisal (MCA). Response options [CBA, CEA, CUA, SROI, MCA, or N/A if only "total cost", also ok to insert descriptive results that do not conform, e.g. “cost transfer ratio”]	Studies
Any cost-effectiveness analysis?	Source [from CEA Inventory]		
Any quant discussion of costs? (Full program)	Source [from CEA Inventory]		
If yes, what type? [Page number]	Source [from CEA Inventory]		
Currency of cost reporting		What is the currency in which costs were reported, i.e. USD, Rwandan Francs, etc.	Studies
Exchange rate		What exchange rate was used for currency adjustments? [e.g. 1.2 USD to 1 Euro]	
Exch rate year (estimated)			
Exchange rate date		What was the date, month, year of the exchange rate used? [If the exchange rate was calculated as an average over the implementation period, copy verbatim from text the definition of average exchange rate used]	

For peer review only

Was there discussion of a discount rate?		[Yes, No]	Studies
Discount rate		Which is the discount rate used in the analysis [this will be a % , please give the value]	Studies
If no discount rate is applied, was any justification provided?		Paste explanation provided in text. We consider any acknowledgement of the discount rate to be justification. For example, ok to report" "we did not discount costs or benefits" as justification	Studies
Inflation adjustment		Does the report mention if costs are given in "real" or "nominal" terms? Look also for "inflation"	Studies
Inflation adjustment of cost reported		If costs are reported in real terms, what inflation index was used to adjust for inflation?	Studies
Base year of the costing	base_year	Was a "base year" (or "start year") of the costing reported? [Yes, No] [If yes, What year?]	Studies
Average cost per number of participants		Was the average cost of the intervention reported per participant? [Yes, No] [If Yes, $ Amount per participant, and currency]	Studies
What is the denominator used in average intervention cost?	ac_ denominator	[Number, Description (please copy description verbatim from report)]	Studies
Average cost per DALY or QALY?		Is the average cost per DALY or QALY reported? [Yes, No, If Yes Page #], Also, add note if cost ‘per capita’ was given rather than average cost per number of participants.	Studies
Total intervention cost (excluding vaccine cost)	total_cost	Report total cost of the intervention, and page.	Studies
Did the total intervention cost reported above include the cost of vaccines?	vac_incl	A "No" indicates that cost ingredients were reported, but there is no reporting of vaccine costs. "Unclear" means we cannot tell from the information given if vaccines were included in total cost [Yes, No, Unclear]	Studies
Total cost description	total_cost_ desc	If there is a description of what is included in total cost, copy description from text here	Studies
Government contribution included in total cost?	tc_gov_ contb	[Yes, No]	
Is more than one total cost given in the report?		[Yes, No, If Yes, please describe additional "total cost" that is reported	
Number of immunisations			
Cost per vaccine delivered, cost per additional immunisation administered or cost per dose (of vaccine). Type of vaccine is given in col I			

For peer review only - http://bmjopen.bmj.com/site/about/guidelines.xhtml

Description and page number of cost per additional vaccine delivered.		Note if average costs per additional vaccine or marginal costs per additional vaccine is reported, where marginal costs should exclude fixed costs.	
Cost per additional child immunised_ All	cpc_all	[Yes, No, If Yes, please report cost per additional child immunised_all]	
Description and page number of cost per additional CHILD Immunised			
Cost per additional child immunised_DPT3	cpc_dpt3	[Yes, No, If Yes, please report cost per additional child immunised with DPT3]. Please make a note if only DPT1 was reported.	
Cost per additional child immunised_Measles	cpc_measles	[Yes, No, If Yes, please report cost per additional child immunised with measles]	
Baseline immunisation coverage of the target population_All		What was the reported baseline rate of immunisation (all immunisations) in the target population? [Percentage, or range]	Studies
Was baseline immunisation coverage of All immunisations reported for the target population, general population, or sample?		Was baseline immunisation coverage of All immunisations reported for the target population, general population, or sample? [target population, general population, sample]	Studies
Baseline immunisation coverage of the target population_DPT3		What was the reported baseline rate of immunisation (DPT3) in the target population? [Percentage, or range]	Studies
Was baseline immunisation coverage of DPT3 reported for the target population, general population, or sample?		Was baseline immunisation coverage of DPT3 reported for the target population, general population, or sample? [target population, general population, sample]	Studies
Baseline immunisation coverage of the target population_Measles		What was the reported baseline rate of immunisation (Measles) in the target population? [Percentage, or range]	Studies
Final immunisation coverage of the target population_All		What was the reported final rate of immunisation (all immunisations) in the target population? [Percentage, or range]	Studies
Final immunisation coverage of the target population_DPT3		What was the reported final rate of immunisation (DPT3) in the target population? [Percentage, or range]	Studies
Final immunisation coverage of the target population_Measles		What was the reported final rate of immunisation (Measles) in the target population? [Percentage, or range]	Studies
Non-compliance		Was there any evidence of non-compliance with treatment assignment. Non-compliance is where individuals assigned to treatment do not take the treatment, or the case where individuals assigned to control DO take the treatment. Ok to keyword search on "non-compliance", "non compliance", "noncompliance" [Yes, No] [If Yes, copy related text on non-compliance]	Studies

For peer review only - http://bmjopen.bmj.com/site/about/guidelines.xhtml

Spillover		Was there any evidence of spillover where individuals assigned to control receive a benefit or incur a 'cost' that results from the treatment intervention? [Yes, No] [If Yes, copy related text on spillovers detected]	Studies
Attrition or drop out		Was there evidence that participants dropped out of the study before follow-up could be completed? [Yes, No] [If Yes, copy related text on drop outs]	Studies
Vaccination coverage_All	JAA01. Full routine immunisation for children	Binary measure of whether or not children have received all routine vaccinations for the relevant country or region.	Data extraction tool_immunisation
Vaccination coverage_DPT3	JAA05. DPT3	Binary measure of whether or not children have received the third dose of the DPT or pentavalent vaccine. If the study does not specifically say "DPT3" (or "pentavalent 3"), but refers to "complete DPT/penta vaccination" or something similar, then use this code.	Data extraction tool_immunisation
Vaccination coverage_Measles	JAA11. Measles	Binary measure of whether or not children have received the measles vaccine	Data extraction tool_immunisation
Costs for significant outcomes only?		Does the report mention that costs will be reported only where significant outcomes are observed? [Yes, No]	

List of coded variables

VARIABLE LABEL	EXPLANATION
Study ID	This is the study ID - it should match the study ID from the Outcome Mapping Sheet (e.g., SC-SR_1)
Estimate ID	The estimate ID will provide a specific number for each effect size extracted and should include the original study number, underscore, then the unique ID number (e.g., SC-SR1_1, SC-SR1_2 and so on)
Author	For 1 author: leading author last name (e.g., Gomez)<br>For 2 authors: both author last names with ampersand in between (e.g., Smith & Bahn)<br>For 3 or more authors: leading author last name followed by et al. (e.g., Gupta et al.)
Year	Year published
Design	0=Experimental Design (e.g., RCT), 1=Quasi-Experimental Design
How Counterfactual is Chosen	Free text (e.g., random control trial, propensity score matching, etc) - Multiple codes are ok
Analysis type for this effect size	Free text, what type of analysis was used (Regression, 2SLS, ANCOVA, etc.)- Multiple codes are ok
Country	Country of intervention
Estimate Type	Type of data for this effect size: 1 = Continuous - means and SDs, 2 = Continuous - mean difference and SD, 3 = Dichotomous outcome - proportions, 4 = Regression data - dichotomous outcome (e.g., logistic regression)

For peer review only - http://bmjopen.bmj.com/site/about/guidelines.xhtml

	5 = Regression data - continuous outcome (e.g., linear regression)
Comparison	1=No intervention (service delivery as usual), 2=Other intervention, 3=Pipeline (wait-list) control (still service delivery as usual)
Describe Comparison Group	If answer above is (1) no intervention, type N/A, if (2) Other Intervention, list what intervention the control group is receiving, if (3) Pipeline control, report when the control group will receive the intervention in relation to the treatment group (e.g., one year later)
Subgroup	Is this analysis of a subgroup? 0=no, 1=yes
If yes to subgroup, describe	Free text, describe the subgoup if applicable (e.g., boys, girls). If no subgroup, type N/A
Source	Note the page number, table number, column, and row you used to extract the data
Treatment Effect	1=Intention to Treat (ITT), 2=Average Treatment Effect on the Treated (ATET), 3=Average Treatment Effect (ATE) 4 = Local Average Treatment Effect (LATE)
Intervention	Free text, what is the intervention
Exposure to intervention (in months)	How long is the intervention exposure itself?
Evaluation period (in months)	The total number of months elapsed between offering an intervention and the point at which an outcome measure is taken post intervention, or as a follow-up measurement. If less than one month, use decimals (e.g., one week would be .25)
Post-intervention or change from baseline?	0 = Post-intervention, 1 = Change from baseline
Source of the Outcome data (only use for outcomes in category JAA)	1=Immunization Card, 2=Recall, 3=Combination of both immunization card and recall, 4=Health Admin Data, 5=N/A, 6 = unclear
Author definition of outcome	Free text - How does the author define the outcome?
Intervention	Coded as engagement as intervention, engagement in the intervention design, engagement in implementation autonomy, or multiple engagement types (place a 1 in the appropriate column
If yes to multiple engagement types	Identify which engagement types are used.
Implemented by the government	0 = no, 1 = yes
Were new cadres of health workers formed?	0 = no, 1 = yes
Was vaccination hesitency identified as a barrier?	0 = no, 1 = yes
Baseline rates of full immunisation	record percentage vaccinated
Baseline rates of DPT3	record percentage vaccinated
**OUTCOME CODES**	
	Code 1 under any applicable columns.
**EFFECT SIZE DATA EXTRACTION**	
Reverse Sign (i.e., decrease is good)	Record 0='no' if an increase is good, record 1='yes' if a decrease is good and the sign needs to be reversed.
Unit of analysis	What is the unit of analysis? UOA for this effect size: 1= Individual, 2= Household, 3= Group (e.g.

For peer review only - http://bmjopen.bmj.com/site/about/guidelines.xhtml

	community organisation), 4= Health Center, 5 = Village, 6 = Other, 7 = Not clear
mean_t	Outcome mean for the treatment group
sd_t	Outcome standard deviation for treatment group
mean_c	Outcome mean for the comparison group
sd_c	Outcome standard deviation for control group
mean_overall_diff	Overall mean difference (treatment - control)
diff se	Standard error of the overall mean difference
Diff _t	t-statistic of mean difference
Odds ratio	Odds ratio reported in the study
OR_se	Odds ratio standard error reported in the study
Risk ratio	Risk ratio reported in study
RR_se	Risk ratio standard error
reg_coeff	Report the regression coefficient of the treatment effect
reg_SE	Report the associated standard error of the regression coefficient.
reg_t	Report the associated t statistic of the effect size (coefficient/SE)
Exact p value	Exact p value if given, if not, record as written in the manuscript (e.g., p < .001, or p > .05)
clust_t	Number of clusters - treatment group
clust_c	Number of clusters - control group
clust_T	Number of clusters - total sample
n_t	Sample size - treatment group
n_c	Sample size - control group
n_T	Sample size - total sample
periods (1 if cross sectional)	Record how many periods of evaluation there are (e.g., cross section is 1, panel data with 3 measurements is 3)
Treatment Variable	Record the treatment variable as written in the model (e.g., the variable name the author uses, such as ("Intervention x Time")
dataset	Record if data comes from an identified dataset
coder	Record your name
Notes	Record any notes important for the team
ROB Category	0 = low risk of bias, 1 = some concerns, 2 = high risk of bias

For peer review only - http://bmjopen.bmj.com/site/about/guidelines.xhtml

Appendix 7: Risk of bias assessment tools

Quantitative risk of bias tool

Code	Question	Coding format	Criteria
General	ID	EPPI ID	
Genera	Study first author	Open answer	
General	Time taken to complete assessment	Minutes	
General	Design type: What type of study design is used?	1= Randomised controlled trial (RCT) (random assignment to households/individuals) or quasi-RCT<br>2 = Cluster-RCT (quasi-RCT)<br>3 = Pseudo-RCT	-
General	Methods used for analysis: Which methods are used to control for selection bias and confounding?	1 = Statistical matching (PSM, CEM, covariate matching)<br>2 = Difference in differences (DID) estimation methods<br>3 = IV-regression (2-stage least squares or bivariate probit)<br>4 = Heckman selection model<br>5 = Fixed effects or random effects regression<br>6 = Covariate adjusted estimation<br>7 = Propensity weighted regression<br>8 = Comparison of means<br>9 = Other (please state)	-
General	Design and analysis method description	Open answer	Briefly describe the study design and analysis method undertaken by the authors.
General	Study population	Open answer	Provide any details in the paper that describe how the study population was selected, answering the question: what is the sampling strategy to recruit participants from that population into the evaluation?
General	Type of comparison group	1=No intervention (service delivery as usual)<br>2=Other intervention<br>3=Pipeline (wait-list) control (still service delivery as usual)	Indicate type of comparison group
General	Type of comparison group (if other)	Open answer	

For peer review only - http://bmjopen.bmj.com/site/about/guidelines.xhtml

General	Ethical clearance	Open answer	Provide any details of ethical research clearances granted. Report unclear if this information is not available.
General	Study registration	Open answer	Provide any details of study registration, including registry IDs, etc.
1: Bias arising from randomisation process	1.1 Was the allocation sequence random?	(1) Yes;<br>(2) Probably yes;<br>(3) Probably no;<br>(4) No;<br>(5) No information;	1.1 Was the allocation sequence random?<br>Answer 'Yes' if a random component was used in the sequence generation process. Examples include computer-generated random numbers; reference to a random number table; coin tossing; shuffling cards or envelopes; throwing dice; or drawing lots. Minimization is generally implemented with a random element (at least when the scores are equal), so an allocation sequence that is generated using minimization should generally be considered to be random.<br><br>Answer 'No' if no random element was used in generating the allocation sequence or the sequence is predictable. Examples include alternation; methods based on dates (of birth or admission); patient record numbers; allocation decisions made by clinicians or participants; allocation based on the availability of the intervention; or any other systematic or haphazard method.<br><br>Answer 'No information' if the only information about randomization methods is a statement that the study is randomized.<br><br>Note: In some situations a judgement may be made to answer 'Probably no' or 'Probably yes'. For example, if the study was large, conducted by an independent trials unit or carried out for regulatory purposes, it may be reasonable to assume that the sequence was random. Alternatively, if other (contemporary) trials by the same investigator team have clearly used non-random sequences, it might be reasonable to assume that the current study was done using similar methods.
1: Bias arising from randomisation process	Question 1.1 answer justification	Open answer	Justification for coding decision<br>(Include a brief summary of justification for rating, mentioning your response to all sub questions, cite relevant pages).
1: Bias arising from randomisation process	1.2 Did baseline differences between intervention groups suggest a problem with the randomisation process?	(1) Yes;<br>(2) Probably yes;<br>(3) Probably no;<br>(4) No;<br>(5) No information;	1.2 Did baseline differences between intervention groups suggest a problem with the randomization process?<br><br>Note that differences that are compatible with chance do not lead to a risk of bias.

For peer review only - http://bmjopen.bmj.com/site/about/guidelines.xhtml

			Answer 'No' if no imbalances are apparent or if any observed imbalances are compatible with chance.<br><br>Answer 'Yes' if there are imbalances that indicate problems with the randomization process, including:<br>(1) substantial differences between intervention group sizes, compared with the intended allocation ratio;<br>or<br>(2) a substantial excess in statistically significant differences in baseline characteristics between intervention groups, beyond that expected by chance (i.e., on more than 10% of the characteristics reported); or<br>(3) imbalance in one or more key prognostic factors, or baseline measures of outcome variables, that is very unlikely to be due to chance and for which the between-group difference is big enough to result in bias in the intervention effect estimate.<br>Also answer 'Yes' if there are other reasons to suspect that the randomization process was problematic, such as:<br>(4) excessive similarity in baseline characteristics that is not compatible with chance; or<br>(5) surprising absence of one or more key baseline characteristics that would be expected to be reported.<br><br>Answer 'No information' when there is no useful baseline information available (e.g. abstracts, or studies that reported only baseline characteristics of participants in the final analysis).<br><br>Note: Trialists may undertake analyses that attempt to deal with flawed randomization by controlling for imbalances in prognostic factors at baseline. To remove the risk of bias caused by problems in the randomization process, it would be necessary to know, and measure, all the prognostic factors that were imbalanced at baseline. It is unlikely that all important prognostic factors are known and measured, so such analyses will at best reduce the risk of bias.
1: Bias arising from randomisation process	Question 1.2 answer justification	Open answer	Justification for coding decision<br>(Include a brief summary of justification for rating, mentioning your response to all sub questions, cite relevant pages).
1: Bias arising from	Assignment mechanism: Was the allocation or identification	(0) Low risk of bias<br>(1) Medium risk of bias/Some concerns	Utliize your answers for questions 1.1 and 1.2 to determine the overall score.

For peer review only - http://bmjopen.bmj.com/site/about/guidelines.xhtml

randomisation process	mechanism random or as good as random?	(2) High risk of bias	
1: Assignment mechanism - Justification	Assignment justification	Open answer	Justification for coding decision<br>(Include a brief summary of justification for rating, mentioning your response to all sub questions, cite relevant pages).
2: Unit of analysis - Assessment	Unit of analysis: Is unit of analysis in cluster allocation addressed in standard error calculation?	1=Yes<br>2=No<br>3=Not reported/unclear<br>4=Not applicable	Score "Yes" if Unit of Analysis = Unit of Randomization OR if UoA ≠ UoR and standard errors are clustered at the UoR level OR data is collapsed to the UoR level<br><br>Score "Not reported/unclear" if not enough information is provided on the way the standard errors were calculated or what the unit of analysis is.<br><br>Score "Not applicable" if it is not a cluster RCT.<br><br>Score "No" otherwise.Score "Yes" if Unit of Analysis = Unit of Randomization OR if UoA ≠ UoR and standard errors are clustered at the UoR level OR data is collapsed to the UoR level<br><br>Score "Not reported/unclear" if not enough information is provided on the way the standard errors were calculated or what the unit of analysis is.<br><br>Score "Not applicable" if it is not a cluster RCT.<br><br>Score "No" otherwise.
2: Unit of analysis - Justification	Question 2 answer justification (provide page numbers)	Open answer	-
3. Bias due to missing outcome data (panel data)	3.1. Was there attrition, if yes, then what was the rate in treatment and control/comparison groups?	(1) Yes;<br>(2) Probably yes;<br>(3) Probably no;<br>(4) No;<br>(5) No information;	3.1. Was there attrition, if yes, then what was the rate in treatment and control/comparison groups?<br><br>In case of attrition, answer 'yes' or 'probably yes', otherwise answer 'no' or 'probably no'.<br><br>If there is no discussion on attrition then score 'no information'.<br><br>Specify the rate of attrition by the study arms.<br>Specify the attrition rate assumed for power calculations.

For peer review only - http://bmjopen.bmj.com/site/about/guidelines.xhtml

			Has the study discussed the ability of the follow-up study to detect the hypothesised outcome effect with the sample size attained?
3. Bias due to missing outcome data – Justification (panel data)	Question 3.1 answer justification	Open answer	Justification for coding decision<br>(Include a brief summary of justification for rating, mentioning your response to all sub questions, cite relevant pages).
3. Bias due to missing outcome data (panel data)	3.2. Does the study establish that attrition is randomly distributed (e.g. by examining correlation with determinants of outcomes, in both treatment and comparison groups or by presenting data showing balance on key characteristics across treatment and control)?	(1) Yes;<br>(2) Probably yes;<br>(3) Probably no;<br>(4) No;<br>(5) No information;	3.2. Does the study establish that attrition is randomly distributed (e.g. by examining correlation with determinants of outcomes, in both treatment and comparison groups or by presenting data showing balance on key characteristics across treatment and control)?<br><br>Score 'yes' or 'probably yes' if balance on key characteristics between attritors and non-attritors for each intervention group (including control) using baseline data has been presented and finds that there is no systematic difference on at least observable dimensions.<br><br>Score 'no' or 'probably no' if the study finds that there is a systematic difference on observables between attritors and non-attritors in any of the intervention groups.<br><br>If the baseline balance table between atritors and non-attritors is not presented or discussed then score 'no information'.
3. Bias due to missing outcome data – Justification (panel data)	Question 3.2 answer justification	Open answer	Justification for coding decision<br>(Include a brief summary of justification for rating, mentioning your response to all sub questions, cite relevant pages).
3. Bias due to missing outcome data – (panel data)	3.3. If there is non-random attrition, have authors used convincing statistical techniques to identify and adjust for the attrition bias?	(1) Yes;<br>(2) Probably yes;<br>(3) Probably no;<br>(4) No;<br>(5) No information;<br>(6) Not applicable	3.3. If there is non-random attrition, have authors used convicing statistical techniques to identify and adjust for the attrition bias?<br><br>If the study has used convincing parametric or non-parametric techniques (like ignorable maximum likelihood, Manski-Lee bounds) to identify and adjust for attrition bias, then score 'yes' or 'probably yes'. If the techniques used are not convincing then score 'no' or 'probably no'. If the study does not discuss using any statiscal techniques for identifying and adjusting for attrition bias then score 'no information'.

For peer review only - http://bmjopen.bmj.com/site/about/guidelines.xhtml

			If there is no non-random attrition, then this question does not apply and the score should be 'not applicable'
3. Bias due to missing outcome data – (panel data)	Question 3.3 answer justification	Open answer	Justification for coding decision<br>(Include a brief summary of justification for rating, mentioning your response to all sub questions, cite relevant pages).
3. Bias due to missing outcome data – Justification (panel data)	Bias due to missing outcome data justification	Open answer	Justification for coding decision<br>(Include a brief summary of justification for rating, mentioning your response to all sub questions, cite relevant pages).
3. Bias due to missing outcome data - Assessment		(0) Low risk of bias<br>(1) Medium risk of bias/Some concerns<br>(2) High risk of bias	Utliize your answers for questions 3.1 to 3.3 to determine the overall score.
3. Bias due to missing outcome data (repeated cross section)	3.1. Was there difference in sample size across baseline and follow-up surveys among treatment and control groups? If yes, what was the rate of difference among treatment and control/comparison groups? Was it lower or higher than baseline?	(1) Yes;<br>(2) Probably yes;<br>(3) Probably no;<br>(4) No;<br>(5) No information;	3.1. Was there difference in sample size across baseline and follow-up surveys among treatment and control groups? If yes, what was the rate of difference among treatment and control/comparison groups? Was it lower or higher than baseline?<br><br>In case of difference, answer 'yes' or 'probably yes', otherwise answer 'no' or 'probably no'.<br><br>If there is no information to assess the differences, then score 'no information'.<br><br>Specify the rate of difference by the study arms.<br>Specify the non-compliance and/or attrition rate assumed for power calculations.<br>Has the study discussed the ability of the follow-up study to detect the hypothesised outcome effect with the sample size attained?
3. Bias due to missing outcome data – Justification (repeated cross section)	Question 3.1 answer justification	Open answer	Justification for coding decision<br>(Include a brief summary of justification for rating, mentioning your response to all sub questions, cite relevant pages).
3. Bias due to missing outcome	3.2. Does the study establish that the sampling frame for baseline and follow-up	(1) Yes;<br>(2) Probably yes;<br>(3) Probably no;	3.2. Does the study establish that the sampling frame for baseline and follow-up surveys is same? Are the clusters (eg health center) and sub-clusters (eg. villages) the same across baseline and follow-up surveys?

For peer review only - http://bmjopen.bmj.com/site/about/guidelines.xhtml

data (repeated cross section)	surveys is same? Are the clusters (eg health center) and sub-clusters (eg. villages) the same across baseline and follow-up surveys?	(4) No;<br>(5) No information;	Score 'yes' or 'probably yes' if sampling frame (clusters and sub-clusters) are the same for baseline and follow-up surveys<br><br>Score 'no' or 'probably no' if the clusters or sub-clusters were different across baseline and follow-up surveys.<br><br>If there is no information on sampling frame across baseline or follow-up surveys, then score 'no information'.
3. Bias due to missing outcome data – Justification (repeated cross section)	Question 3.2 answer justification	Open answer	Justification for coding decision<br>(Include a brief summary of justification for rating, mentioning your response to all sub questions, cite relevant pages).
3. Bias due to missing outcome data –(repeated cross section)	3.3. Was the sample selection of respondents carried out in the same way as in the baseline? Was the intended number of respondents in the clusters/sub-clusters same across baseline and follow-up surveys?	(1) Yes;<br>(2) Probably yes;<br>(3) Probably no;<br>(4) No;<br>(5) No information;	3.3. Was the sample selection of respondents carried out in the same way as in the baseline? Was the intended number of respondents in the sub-clusters same across baseline and follow-up surveys?<br><br>Score 'yes' or 'probably yes' if the study used same listing process for selection of respondents and their intended number was same across baseline and follow-up surveys,<br><br>Score 'no' or 'probably no' if the study used different listing process for selection of respondents or their intended number was different across baseline and follow-up surveys.<br><br>If there is no information on the selection process of respondents then score 'no information'.
3. Bias due to missing outcome data - Justification (repeated cross section)	Question 3.3 answer justification	Open answer	Justification for coding decision<br>(Include a brief summary of justification for rating, mentioning your response to all sub questions, cite relevant pages).
3. Bias due to missing outcome data – Assessment	Bias due to missing outcome data	(0) Low risk of bias<br>(1) Medium risk of bias/Some concerns<br>(2) High risk of bias	Utliize your answers for questions 3.1 to 3.3 to determine the overall score using the criteria below

For peer review only - http://bmjopen.bmj.com/site/about/guidelines.xhtml

(repeated cross section)			
Bias due to missing outcome data – Justification (repeated cross section)	Bias due to missing outcome data justification	Open answer	Justification for coding decision (Include a brief summary of justification for rating, mentioning your response to all sub questions, cite relevant pages).
3. Bias due to missing outcome data - Assessment	(0) Low risk of bias<br>(1) Medium risk of bias/Some concerns<br>(2) High risk of bias		Utliize your answers for questions 3.1 to 3.3 to determine the overall score.
3.Bias due to missing outcome data - Justification	Bias due to missing outcome data justification	Open answer	Justification for coding decision (Include a brief summary of justification for rating, mentioning your response to all sub questions, cite relevant pages).
4: Bias due to deviations from intended interventions - Assessment	4.1. Was the study adequately protected against spill-overs/ contamination?	(1) Yes;<br>(2) Probably yes;<br>(3) Probably no;<br>(4) No;<br>(5) No information;	4.1. Was the study adequately protected against spill-overs/ contamination?<br><br>Score "Yes" if the intervention is unlikely to spill-over to comparisons (e.g. participants and non-participants are geographically and/or socially separated from one another and general equilibrium effects are not likely) and that the treatment and comparisons are isolated from other interventions which might explain changes in outcomes.<br><br>Score "No" if allocation was at the individual or classroom level and there are likely spill-overs within households and communities which are not controlled for (such as using trasferable vouchers), or other interventions likely to affect outcomes operating at the same time in either group.<br><br>Score "no information" if spill-overs and/or contamination are not discussed
4: Bias due to deviations from intended interventions - Justification	Question 4.1 answer justification	Open answer	Justification for coding decision (Include a brief summary of justification for rating, mentioning your response to all sub questions, cite relevant pages).

For peer review only - http://bmjopen.bmj.com/site/about/guidelines.xhtml

4: Bias due to deviations from intended interventions - Assessment	4.2. Was the process of being monitored free from bias?	(1) Yes;<br>(2) Probably yes;<br>(3) Probably no;<br>(4) No;<br>(5) No information;	4.2. Was the process of being monitored free from bias?<br><br>Score "Yes" if the authors state explicitly that the process of monitoring the intervention is blinded, or argue convincingly why it is not likely that intervention delivery could affect the performance of participants in treatment and comparison groups in different ways (such as resulting in Hawthorne or John Henry effects). or there is nothing in the surveys that might have given the control participants an idea of what the other group might receive (e.g. provide cash transfer to the intervention area participants or reveal informmation that they did not have before or they did but there is no risk that this has changed their behaviours;<br><br>Score "No" if the the authors do not use an appropriate method to prevent Hawthorne and John Henry Effects (e.g. blinding of monitoring or other methods to ensure consistent monitoring across groups) and there is a risk that the intervention delivery or survey process could have changed the behaviours of treatment and comparison groups in different ways.<br><br>Score "no information" if the authors do not discuss potential bias due to monitoring of intervention.<br><br>Hawthorne effects may result where participants know that they are being observed and John Henry Effects may result from participant knowledge of being compared.
4: Bias due to deviations from intended interventions - Justification	Question 4.2 answer justification	Open answer	Justification for coding decision<br>(Include a brief summary of justification for rating, mentioning your response to all sub questions, cite relevant pages).
4: Bias due to deviations from intended interventions - Assessment	4.3. Was an appropriate analysis used to estimate the effect of assignment to intervention?	(1) Yes;<br>(2) Probably yes;<br>(3) Probably no;<br>(4) No;<br>(5) No information;	44.3. Was an appropriate analysis used to estimate the effect of assignment to intervention?<br><br>Both intention-to-treat (ITT) analyses and modified intention to treat (mITT) analyses excluding participants with missing outcome data should be considered appropriate. (Missing outcome data are addressed in a separate domain). Both ‘as treated’ analyses (in which trial participants are grouped according to the intervention that they received, rather than according to their assigned intervention) and

For peer review only - http://bmjopen.bmj.com/site/about/guidelines.xhtml

			naïve ‘per-protocol’ analyses (excluding trial participants who did not receive their assigned intervention) should be considered inappropriate. Analyses excluding eligible trial participants post-randomization should also be considered inappropriate, but post-randomization exclusions of ineligible participants (when eligibility was not confirmed until after randomization, and could not have been influenced by intervention group assignment) can be considered appropriate.<br><br>Score "Yes" if ITT or modified ITT analysis has been used, AND common analysis methods are used AND any covariates imbalanced at baseline are included as covariates in the analysis.<br>Score "No" otherwise. You may also score no if they use an uncommon analysis methods, such as 3SLS, or if they analyse dichotomous outcome using continuous methods (e.g., using linear regression instead of using the more appropriate logit or probit models) or if the is baseline imbalance but those covariates are not included in the model.<br><br>If the analytical specification is not provided score "no information"
4: Bias due to deviations from intended interventions - Justification	Question 4.3 answer justification	Open answer	Justification for coding decision<br>(Include a brief summary of justification for rating, mentioning your response to all sub questions, cite relevant pages).
4: Bias due to deviations from intended interventions - Assessment	Deviations from intended interventions: Spill-overs, cross-overs, contamination and performance bias: was the study adequately protected against spill-overs, cross-overs, contamination, and performance bias?	(0) Low risk of bias<br>(1) Medium risk of bias/Some concerns<br>(2) High risk of bias	Utliize your answers for questions 4.1 to 4.3 to determine the overall score.
4: Bias due to deviations from intended interventions - Justification	Deviations justification	Open answer	Justification for coding decision<br>(Include a brief summary of justification for rating, mentioning your response to all sub questions, cite relevant pages).<br><br>For example, intervention groups are geographically separated, authors use intention to treat estimation or instrumental variables to

For peer review only - http://bmjopen.bmj.com/site/about/guidelines.xhtml

			account for non-adherence, and survey questions are not likely to expose individuals in the control group to information about desirable behaviours ('survey effects').
5. Outcome measurement bias	5.1 Could the measurement of the outcome be different between the study arms?	(1) Yes;<br>(2) Probably yes;<br>(3) Probably no;<br>(4) No;<br>(5) No information;	5.1 Could the measurement of the outcome be different between the study arms?<br><br>Comparable methods of outcome measurement (data collection) involve the same measurement methods and thresholds, used at comparable time points. Differences between intervention groups may arise because of 'diagnostic detection bias' in the context of passive collection of outcome data, or if an intervention involves additional visits to a healthcare provider, leading to additional opportunities for outcome events to be identified.<br><br>Score 'yes' or 'probably yes' if the outcomes have been measured at different times periods for treatment and control arms or have used different methods of measurement.<br><br>Score 'no' or 'probably no' otherwise.<br><br>If no information is provided on the the time period and methods of data collection in treatment and control arms, then score 'no information'
5. Outcome measurement bias - Justification	Question 5.1 answer justification	Open answer	Justification for coding decision<br>(Include a brief summary of justification for rating, mentioning your response to all sub questions, cite relevant pages).
5. Outcome measurement bias	5.2. Were the outcome assessors (enumerators) blinded to the intervention assignment?	(1) Yes;<br>(2) Probably yes;<br>(3) Probably no;<br>(4) No;<br>(5) No information;	5.2. Were the outcome assessors (enumerators) blinded to the intervention assignment?<br><br>Score 'yes' or 'probably yes' if outcome assessors were blinded to intervention status and 'no' or 'probably no' otherwise.<br><br>If no information is provided on blinding of the outcome assessors, then score 'no information'.<br><br>Note that for participant-reported outcomes, the outcome assessor is the study participant.
5. Outcome measurement	Question 5.2 answer justification	Open answer	Justification for coding decision

For peer review only - http://bmjopen.bmj.com/site/about/guidelines.xhtml

bias - Justification			(Include a brief summary of justification for rating, mentioning your response to all sub questions, cite relevant pages).
5. Outcome measurement bias	5.3. If not blinded, could assessment of the outcome have been influenced by knowledge or administration of intervention received?	(1) Yes;<br>(2) Probably yes;<br>(3) Probably no;<br>(4) No;<br>(5) No information;	5.3. If not blinded, could assessment of the outcome have been influenced by knowledge or administration of intervention received?<br><br>Knowledge or administration of the assigned intervention could influence participant-reported outcomes (such as level of pain, immunization outcomes using recall), observer-reported outcomes involving some judgement, and intervention provider decision outcomes. They are unlikely to influence observer-reported outcomes that do not involve judgement, for example all-cause mortality.<br><br>For participant-reported outcomes, the assessment of outcome is potentially influenced by knowledge or administration of intervention received, leading to a judgement of at least 'Some concerns'. Review authors will need to judge whether it is likely that participants' reporting of the outcome was influenced by knowledge or administration of intervention received, in which case risk of bias is considered to be high.<br><br>Score 'yes' or 'probably yes' if assessment of outcome could have been influenced by knowledge of intervention received (e.g., if ANY of the immunization coverage outcomes are assessed through caregiver recall) and 'no' or 'probably no' otherwise (e.g., if immunization coverage outcome is assessed by immunization card).<br>If no information is available to make the assessment then score 'no information'.
5. Outcome measurement bias - Justification	Question 5.3 answer justification	Open answer	Justification for coding decision<br>(Include a brief summary of justification for rating, mentioning your response to all sub questions, cite relevant pages).
5. Outcome measurement bias - Assessment	Outcome measurement bias: Was the study free from biases in outcome measurement?	(0) Low risk of bias<br>(1) Medium risk of bias/Some concerns<br>(2) High risk of bias	Utliize your answers for questions 5.1 to 5.3 to determine the overall score.
5. Outcome measurement bias - Justification	Outcome measurement justification	Open answer	Justification for coding decision<br>(Include a brief summary of justification for rating, mentioning your response to all sub questions, cite relevant pages).

For peer review only - http://bmjopen.bmj.com/site/about/guidelines.xhtml

6. Reporting bias	6.1. Is a pre-analysis plan or protocol available which provides sufficient detail?	(1) Yes;<br>(2) Probably yes;<br>(3) Probably no;<br>(4) No;<br>(5) No information;	6.1. Is a pre-analysis plan or protocol available which provides sufficient detail?<br><br>Score 'yes' if they reference a preanalysis plan, and 'no' otherwise.
6. Reporting bias - Justification	Question 6.1 answer justification	Open answer	Justification for coding decision<br>(Include a brief summary of justification for rating, mentioning your response to all sub questions, cite relevant pages).
6. Reporting bias	6.2. Were all primary and secondary outcomes reported as per the pre-analysis plan/protocol?	(1) Yes;<br>(2) Probably yes;<br>(3) Probably no;<br>(4) No;<br>(5) No information;	6.2. Were all primary and secondary outcomes reported as per the pre-analysis plan/protocol?<br><br>Score "Yes" or "probably yes" if there is no evidence that outcomes were selectively reported (e.g. results for all relevant outcomes in the methods section are reported in the results section)<br><br>Score "No" or "probably no" if some important outcomes are subsequently omitted from the results or the significance and magnitude of important outcomes was not assessed or if multiple measurements of an outcome were made but only one or a subset is reported on the basis of the results (e.g. statistical significance)<br><br>Score "No information" if pre-analysis not available or the outcome intentions are not reported in sufficient detail to enable an assessment.
6. Reporting bias - Justification	Question 6.2 answer justification	Open answer	Justification for coding decision<br>(Include a brief summary of justification for rating, mentioning your response to all sub questions, cite relevant pages).
6. Reporting bias	6.3. Do reported results for the outcomes correspond to all intended analyses?	(1) Yes;<br>(2) Probably yes;<br>(3) Probably no;<br>(4) No;<br>(5) No information;	6.3. Do reported results for the outcomes correspond to all intended analyses?<br><br>A particular outcome domain may be analysed in multiple ways. Examples include: unadjusted and adjusted models; final value vs change from baseline vs analysis of covariance; transformations of variables; different definitions of composite outcomes (e.g. 'major adverse event'); conversion of continuously scaled outcome to categorical data with different cut-points; different sets of covariates for adjustment; and different strategies for dealing with missing data. Application of multiple methods generates multiple effect estimates for a specific outcome domain. If multiple estimates are generated but only one or a subset is reported on the basis of the results (e.g.

For peer review only

			statistical significance), there is a high risk of bias in the fully reported result.<br><br>Answer 'No' or 'Probably No' if:<br>There is clear evidence (usually through examination of a trial protocol or statistical analysis plan) that a domain was analysed in multiple ways, but data for only one or a subset of analyses is fully reported (without justification), and the fully reported result is likely to have been selected on the basis of the results. Selection on the basis of the results arises from a desire for findings to be newsworthy, sufficiently noteworthy to merit publication, or to confirm a prior hypothesis. For example, trialists who have a preconception or vested interest in showing that an experimental intervention is beneficial may be inclined to selectively report analyses that are favourable to the experimental intervention. Score probably no if authors do not report both adjusted and unadjusted models.<br><br>Answer 'Yes' or 'Probably Yes' if:<br>There is clear evidence (usually through examination of a trial protocol or statistical analysis plan) that all reported results for the outcome domain correspond to all intended analyses.<br>or<br>Not all intended analyses have been reported but authors have convincingly justified the reasons for not doing so.<br>or<br>There is only one possible way in which the outcome domain can be analysed (hence there is no opportunity to select from multiple analyses).<br>or<br>Analyses are inconsistent across different reports on the same trial, but the trialists have provided the reason for the inconsistency and it is not related to the nature of the results.<br><br>Answer 'No information' if:<br>Analysis intentions are not available, or the analysis intentions are not reported in sufficient detail to enable an assessment, and there is more than one way in which the outcome domain could have been analysed.
6. Reporting bias - Justification	Question 6.3 answer justification	Open answer	Justification for coding decision<br>(Include a brief summary of justification for rating, mentioning your response to all sub questions, cite relevant pages).

For peer review only - http://bmjopen.bmj.com/site/about/guidelines.xhtml

6. Reporting bias - Assessment	Analysis reporting: Was the study free from selective analysis reporting?	(0) Low risk of bias<br>(1) Medium risk of bias/Some concerns<br>(2) High risk of bias	Utliize your answers for questions 6.1 to 6.3 to determine the overall score.
6. Reporting bias - Justification	Analysis reporting justification	Open answer	Justification for coding decision<br>(Include a brief summary of justification for rating, mentioning your response to all sub questions, cite relevant pages).
7. Other bias - Assessment	Other risks of bias Is the study free from other sources of bias?	1= Yes,<br>4 = No	-
7. Other bias - Justification	Other bias justification	Open answer	Justification for coding decision<br>(Include a brief summary of justification for rating, mentioning your response to all sub questions, cite relevant pages). For example, information is collected using a different survey instrument in different intervention groups; measurement of the intervention received in unclear.
8. Blinding - observers - Assessment	Blinding of participants?	1 = Yes<br>2 = No<br>8 = unclear<br>9 = N/A	If there is no information, code NO. If there is information but it is ambiguous, code UNCLEAR.
8. Blinding - analysts - Assessment	Blinding of data analysts?	1 = Yes<br>2 = No<br>8 = unclear<br>9 = N/A	If there is no information, code NO. If there is information but it is ambiguous, code UNCLEAR.
8. Blinding - method(s)	Method(s) used to blind	Open answer (including describe method of placebo control) 9 = N/A	Describe method(s) used to blind
9. External validity - Random Sampling	Was random sampling used?	1 = Yes<br>2 = No	Was a random sampling method used (meaning everyone in the population had an equal chance of being selected for the study - note that this is different than random allocation to treatment versus control group)?<br>Score yes if: (a) there is a sampling frame. The sampling frame is the actual list of individuals that the sample will be drawn from. Ideally, it should include the entire target population (and nobody who is not part of that population). AND<br>(b) probibility sampling is used (e.g., simple random, stratified random, cluster random, etc.)<br>Score no if: individuals are selected based on non-random criteria, and not every individual has a chance of being included (e.g., a convenience sample, a purposive sample, a snowball sample, a voluntary response sample, etc.).

For peer review only - http://bmjopen.bmj.com/site/about/guidelines.xhtml

9. External validity - Random Sampling Justification	Justification for answer to random sampling question	Open answer	Justification for coding decision<br>(Include a brief summary of justification for rating, mentioning your response to all sub questions, cite relevant pages).Describe the sampling technique used.
9. External validity - Assessment	External validity	Open answer	a) What do authors say about external validity? Note any additional information related to generalizability.

For peer review only

Qualitative risk of bias tool

Critical appraisal of qualitative studies tool

This tool provides specific questions you should answer with regards to the study being appraised. Every question has three possible responses, which correspond to ‘strong’, ‘weak’, or ‘none’ – these are phrased slightly differently depending on the question. This process is subjective and relies on a basic level of familiarity with qualitative research methods. However, to further structure the assessment, each question is accompanied by a description of criteria you should consider before making your choice.

While attempting to answer a question, you may find that a study contains many of the relevant elements, but not all. Give this paper the benefit of the doubt – if the element being assessed is strong except for one minor element, choose the ‘strong’ option.

There is room for comments after each question. You are encouraged to utilize this space, though only the multiple answer response is mandatory. Use the space to document your thoughts if you feel uneasy about the choice made, or if you have any ideas which would help as we refine and revise the assessment tool. There is also room at the end for any additional notes on the paper as a whole.

The questions are arranged according to the order in which a standard academic article is arranged: Introduction, Methodology, Results, Discussion. This will allow you to answer the questions as you go over each section. That said, some documents will not follow this structure, and some academic articles are more fluid about where certain descriptions are located within the text. After you read through the article, go over all the questions once more to see whether any elements you thought were missing actually showed up in an unexpected place.

* Required

Assessor: *

Paper Title: *

Authors: *

Year: *

Introduction

*1. Is the research aim clearly stated? **

Look for this in either the abstract or the introduction. In the best studies, this will be an explicit formulation of the research aims/questions. There may be several. Other studies do not formally state the research aims as such, but they are clearly evident from the text. Note: you might find it useful to write down these research aims, as many of the other questions relate to them.

- Yes, a strong statement
- Yes, but an unclear or weak statement
- No

Question 1 – Notes:

*2. Is there a description of the context in which the study takes place? **

For peer review only - http://bmjopen.bmj.com/site/about/guidelines.xhtml

The study should make some reference to the geographical, temporal, or societal greater context within which the study topic is located. A study within a well-established area of research could likely be situated quite specifically. An exploratory study might cover a topic where there is little descriptive context to draw upon.

- Yes, a strong description
- Yes, but a weak description
- No

Question 2 – Notes:

*3. Is there a clear link to relevant literature? **

Literature should be cited not just in general, but in specific relation to the topic of the paper. In assessing whether the literature is relevant and sufficient, consider: Are most of the cited papers about the same area or topic as the study? Are both qualitative and quantitative studies cited? Are there at least some papers published within the last 10 years?

- Yes, a clear strong link
- Yes, but it could be improved
- No

Question 3 – Notes:

*4. Is there a clear link to theory? **

This can be achieved in many ways, and while it will often be in the introduction, it may show up elsewhere, so keep your eyes peeled. This requires a reference to a specific theory. There are two main ways this could happen: A reference could be made to an established theoretical framework (from the social sciences, from public health, etc.) which has been used to investigate similar questions, or which the authors think might apply in this case. The second possibility is that the authors describe a theoretical framework they have created or adapted themselves which they used in structuring their study, or that they refer to in considering their results.

- Yes, a strong one
- Yes, but a weak one
- No

Question 4 – Notes:

Methodology - Sampling

*5. Is there a description of the sampling procedure? **

Are there details about how sampling was actually conducted? A helpful way to consider this is whether you would be able to conduct this sampling yourself if you were to replicate the study. Every sampling approach has its own logic, but even for the more straightforward qualitative sampling, there should be some mention of this. Note: This

For peer review only - http://bmjopen.bmj.com/site/about/guidelines.xhtml

For peer review only

question does not evaluate whether this sampling is actually appropriate (see question 6). It only asks whether we have a clear idea of the process of sampling, at all.

- Yes, a strong description
- Yes, but a partial description
- No

Question 5 – Notes:

*6. Is the sampling strategy appropriate for the aims of the research? **

This question asks whether the sampling strategy was the right for one for choosing the participants/locations/etc. that could yield relevant information. If there is a strong claim of generalizability, was the sampling appropriate for achieving this? If the study attempts to present the views of a group, did sampling actually capture the people the study needed to hear from? Are there any groups which appear to be missing but clearly should have been spoken to in order to meet the research aims? Has saturation been achieved, and can we tell how this was established? Note that this is not a question of whether the method of collection (interview, focus group, etc.) would have yielded strong data – just whether the people recruited to the study were the right people.

- Yes, very appropriate
- Yes, but not completely appropriate
- No

Question 6 – Notes:

*7. Are sample characteristics sufficiently reported? **

The basic characteristics are often gender, location, ethnicity, and other demographic variables, but consider whether there are other sample characteristics specific to the question which should be reported. This might be clear by this point, but you might also want to look ahead and see whether the paper discusses any relevant characteristics that you would want to know about the sample in general (or you could return to this at the end).

- Yes, there's a sufficient description
- There's some description, but not enough
- No

Question 7 – Notes:

Methodology - Data Collection

*8. Is it clear how data were collected? **

Each method requires its own information. If interviews were used, is there detail on locations, presence of others, length of interview, questions asked, and other such details? For participant observation, is there a description of the

For peer review only - http://bmjopen.bmj.com/site/about/guidelines.xhtml

time spent in the field and the activities partaken in? For focus groups, is there detail on the number of participants, questions asked and setting?

- Yes, data collection is clearly described
- Yes, but more details are needed
- No

Question 8 – Notes:

*9. Are the methods of data recording reported? **

This could be as simple as 'the audio of interviews was taped', 'focus group summaries were written during the discussion', or 'interviews were transcribed, translated, and the translation was then verified'. Any of these would merit at least a 'weak' response, and more detail would be considered 'strong'.

- Yes, and reported well
- Yes, but only briefly mentioned
- No

Question 9 – Notes:

*10. Did the collection of the data address the research aims? **

Given the data needed to answer the question, is the method chosen actually well suited to obtaining such data? If we have knowledge of the questions in an interview guide or a focus group guide, are all the relevant subject areas covered? This isn't about whether there might be some problems in the collection (a bad interviewer could screw up even the best interview guide) but whether the method of data collection, if employed properly, would actually capture relevant data.

- Yes, completely
- Yes, but not entirely
- No

Question 10 – Notes:

Methodology - Analysis

*11. Are the methods of analysis explicitly stated? **

This should include a basic statement as to the approach used (thematic analysis, grounded theory, etc.) but also a breakdown of the steps taken (different rounds of coding, intermittent checks for quality, revisions, etc.)

- Yes, and stated well
- Yes, but more detail is needed
- No

For peer review only - http://bmjopen.bmj.com/site/about/guidelines.xhtml

Question 11 – Notes:

*12. Were there any inbuilt checks to assure the quality of the analysis? **

Some but not all of the following could be present. Was there more than one researcher involved in the analysis? Was there independent peer review during the analysis? Were inter-coder or intra-coder reliability checks conducted? Did the team meet regularly to discuss the analytic process? Did the researcher keep a journal of their thoughts and decisions?

- Yes, there were clear, strong checks throughout
- Yes, but they were weak checks
- No

Question 12 – Notes:

*13. Was there reflection on bias and positionality? **

Have the authors considered their potential biases in relation to the study? This should be a specific statement, and there should be mention of steps that were taken to address this, even if as basic as taking the time to write reflective notes or engaging in a group discussion of the issues.

- Yes, a clear reflection with detail provided
- Yes, but there's only a brief mention of this
- No

Question 13 – Notes:

Methodology - Researchers

14. Are there any details about the people who conducted the sampling, data collection, and analysis?

Not much is required, but look out for mentions of the training these people had, their positions/titles, their experience, etc.

- Yes, plenty
- Yes, a little
- No

Question 14 – Notes:

Results

*15. Is there enough data to support the claims? **

For peer review only - http://bmjopen.bmj.com/site/about/guidelines.xhtml

Variable, but consider whether major claims/themes are supported by more than a single quote or other type of original data. Look at whether the data presented is contextualised, and whether it demonstrates the claim that the authors suggest it does.

- Yes, there's plenty of clear, contextualised, relevant data
- There's some data, but it's weak
- No - there's little or no data presented that is supports the claims.

Question 15 – Notes:

*16. Are diverse viewpoints considered? **

At a basic level, we need to know who speakers are if they are quoted. This could be as minimal as ('22-year-old', or 'Mother, Group A'). Same goes for summaries with no direct quotes – it should be clear whose viewpoint the data comes from. The majority of the data presented will demonstrate the main themes/arguments, but no study is without some dissenting voices or unusual opinions. Are these ever mentioned? If there were many participants, but it appears that most quotes come from one or two individuals, there should be a reasonable explanation for why this is the case – and what it might mean for the study.

- Yes, thoroughly
- Yes, only occasionally
- No

Question 16 – Notes:

*17. Is there evidence which addresses every research aim? **

Look back to the research aims. Is there data relating to each one of them? If not, is there a clear reason for this? If a study ended up not finding data relevant to every aim, but acknowledges that this is the case and tries to account for the reason, this should still be marked as a yes. A 'null' result is still a result - sometimes collection and analysis can be excellent, yet no clear answer is found. If this appears to be the case, the study can still be considered 'strong'.

- Yes, every aim has some related evidence
- Yes, but some aims have distinctly less attending to them
- No

Question 17 – Notes:

Discussion

*18. Has the question been answered? **

A simple test is to go back to the research aims, formulate them as questions (if they're not already established as such) and see whether the authors have provided you with answers that are reasonable given the data presented. It should be clear how the data presented is appropriate and convincing evidence for the conclusion. In a strong paper,

For peer review only - http://bmjopen.bmj.com/site/about/guidelines.xhtml

the authors will make a clear and logical statement of how the results provide an answer to the question. A weaker paper might not cover all the questions, or might suggest questionable links between the data in the results and the conclusions reached.

- Yes, with a clear, logical, thorough answer
- Yes, but not well
- No

Question 18 – Notes:

*19. Are relevant literature, theory, or practice discussed in relation to the results? **

A good paper doesn't need to discuss all of these, but it should relate to at least one. Did it match up or challenge a theory? Does it have implications for practice? Are the results now situated within the broader literature?

- Yes, and discussed well
- Yes, but only briefly or generally discussed
- No

Question 19 – Notes:

*20. Has there been any triangulation? **

This takes many forms, but the basic question is – do the authors present any other sources of data which support or contradict their claims? These could be quantitative/qualitative/mixed studies, some form of evaluation of the conclusions by the participants themselves, comparison to other data recorded, etc. This could be in the results section or the discussion, so have another look.

- Yes, strong triangulation
- Yes, weak triangulation
- No

Question 20 – Notes:

*21. Are weaknesses considered? **

Mentioning them is a good step, but a strong paper will also argue how these weaknesses were attended to.

- Yes, major weaknesses considered and attended to
- Yes, but weaknesses are mentioned without much discussion
- No

Question 21 – Notes:

For peer review only - http://bmjopen.bmj.com/site/about/guidelines.xhtml

Ethics

22. Have ethical issues been taken into consideration? *

At the very basic level, there should be a description of informed consent. Also look for approval from an IRB (or other ethics committee as applicable), and whether issues of benefit/harm and confidentiality/secrecy were discussed with participants.

- Yes
- No

Question 22 – Notes:

General comments and thoughts:

Remember to look back at the questions before submitting.

Having read the paper as a whole, and having considered each question separately, you might now also have a clearer idea as to the answers to some questions. Have another look, particularly for questions where you noted some thoughts or concerns. You may want return to question 7 in particular, as you may now have a clearer idea of what sample characteristics should have been reported.

For peer review only

Cost evidence risk of bias tool

S.No.	Questions	Response
1a.	Is the form of economic evaluation clearly stated? Indicate (CBA/CEA/CUA/cost-minimization, or innovative approaches: Social Return on Investment (SROI), Multi-Criteria Appraisal (MCA). Response options [CBA, CEA, CUA, SROI, MCA, or N/A if only "total cost", also ok to insert descriptive results that do not conform, e.g. “cost transfer ratio”]	Yes, No
1b.	Is the perspective of the costing stated?	Yes, No
1d.	Cost data sources?	Yes, No
1e.1	Are unit costs reported (in Table)	Yes, No
1e.2	Are cost ingredients listed?	Yes, No
1e.3	Total cost description	Yes, No
1f.	Total cost reported?	not reported, Value if reported
1h.	Was cost per child immunised reported?	Yes, No, If yes, Cost
1j.	What exchange rate was used for currency adjustments? [e.g. 1.2 USD to 1 Euro]	value if given, Not reported
1j.	Exch rate year (estimated)	Year
1k.	Is time horizon for costs clearly stated? [Operationalise - was a base year of the costing reported?	Yes, No, If yes: year
7a.	Sensitivity analysis? [yes/ no]	n/a, Description if yes

For peer review only - http://bmjopen.bmj.com/site/about/guidelines.xhtml

Appendix 8: Risk of bias in the included studies

Quantitative risk of bias

Risk of bias in Randomised Controlled Trials

Of the included studies with experimental designs (k = 31) the majority (k = 23) have been identified as being at high risk of bias, and only two were rated as having a low risk of bias. Six studies were assessed as having some concerns. Of the six potential causes of bias that were analysed, outcome measurement bias and deviations from intended interventions were the most commonly documented issues. The most common pitfall related to outcome measurement bias was the use of caregiver reported (self-reported) measures of vaccinations received by a child in the absence and/or incompleteness of immunisation cards, which is common in L&MICs and is often influenced by the intervention itself. One such study stated that "The calculation of FIC coverage was based on data from the child's immunization card and/or the mother's recall. Mother's recall was taken in situations in which the card or specific data points on the card were missing.", (Gurley 2020). Relatedly, Demilew, (2021) reported that their outcome measures "...could be subject to recall bias or social desirability bias differentially by treatment group…". Deviations from intended interventions had to do with issues such as potential spillover related to geography (e.g. in Siddiqi 2020, centers were contiguously located) or transferable vouchers (e.g. Morris 2004). Other sources of bias in included RCT studies are reporting bias, and bias in the randomisation process. Supplementary Figure 2a illustrates the frequencies with which each cause of bias was identified across the 31 included RCTs, while Supplementary Figure 2b shows the risk of bias assessments for each individual study and each risk of bias category for RCT studies.

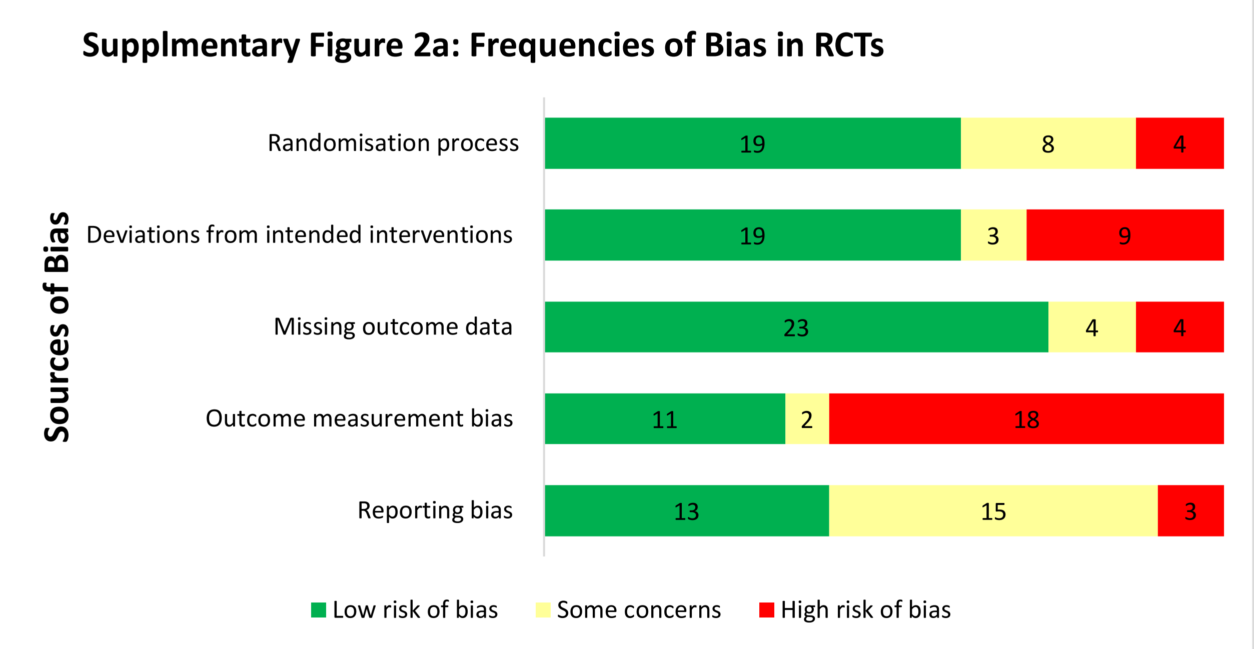

For peer review only - http://bmjopen.bmj.com/site/about/guidelines.xhtml

Supplementary Figure 2b: Risk of bias assessments for individual RCT studies

Study first author	Assignment mechanism: Was the allocation or identification mechanism random or as good as random?	Unit of analysis: Is unit of analysis in cluster allocation addressed in standard error calculation ?	Bias due to missing outcome data	Was the study adequately protected against spill-overs, cross-overs, contamination, and performance bias?	Outcome measurement bias: Was the study free from biases in outcome measurement?	Analysis reporting: Was the study free from selective analysis reporting?	Overall Risk of Bias Assessment
Alhassan, 2019	Yellow	Green	Green	Red	Green	Green	Red
Andersson, 2009	Green	Green	Green	Green	Red	Yellow	Red
Arifeen, 2009	Green	Yellow	Red	Green	Green	Green	Red
Banerjee, 2010	Green	Green	Red	Green	Green	Yellow	Red
Banerjee, 2020	Green	Green	Green	Green	Green	Green	Green
Björkman, 2009	Yellow	Green	Yellow	Green	Green	Yellow	Yellow
Bolam, 1998	Yellow	Green	Yellow	Red	Red	Yellow	Red
Borkum, 2014	Green	Green	Green	Yellow	Red	Yellow	Red
Demilew, 2020	Red	Green	Green	Green	Red	Green	Red
Domek, 2019	Green	Green	Green	Green	Green	Yellow	Yellow
Engineer, 2016	Yellow	Green	Green	Green	Red	Red	Red
Gibson, 2017	Green	Green	Green	Green	Green	Yellow	Yellow
Gurley, 2020	Red	Green	Red	Green	Red	Green	Red
Johri, 2018	Yellow	Green	Green	Green	Yellow	Yellow	Yellow
Lee, 2015	Green	Green	Green	Green	Green	Yellow	Yellow
Modi, 2019	Yellow	Green	Green	Green	Red	Yellow	Red
Mohanan, 2020	Yellow	Green	Green	Yellow	Red	Green	Red
More, 2012	Green	Green	Green	Green	Green	Green	Green
More, 2017	Green	Green	Green	Green	Red	Green	Red
Morris, 2004	Green	Green	Green	Red	Red	Green	Red
Murthy, 2019	Red	Green	Yellow	Red	Red	Yellow	Red
Nagar, 2018	Red	Yellow	Yellow	Yellow	Green	Yellow	Red
Nagar, 2020	Green	Green	Green	Green	Red	Red	Red
Olken, 2014	Green	Green	Green	Red	Red	Green	Red
Oyo-Ita, 2020	Green	Green	Green	Green	Red	Green	Red
Pramanik, 2020	Green	Green	Red	Green	Red	Green	Red
Rahman, 2008	Yellow	Yellow	Green	Green	Yellow	Yellow	Yellow
Robertson, 2013	Green	Yellow	Green	Red	Red	Yellow	Red
Seth, 2018	Green	Green	Green	Red	Green	Yellow	Red
Siddiqi, 2020	Green	Green	Green	Red	Red	Green	Red
Webster, 2019	Green	Green	Green	Red	Red	Red	Red

Note: Green cells signify low risk of bias, yellow cells signify some concerns related to risk of bias, and red cells signify high risk of bias.

For peer review only - http://bmjopen.bmj.com/site/about/guidelines.xhtml

Bias in Quasi-Experimental Designs

Of the 30 included quasi-experimental studies, the majority (k = 27) present a high risk for bias, two were assessed as low risk of bias and one study was assessed as having some concerns. Like the experimental studies the most common identified causes of bias amongst quasi-experimental designs were issues related to outcome measurement bias and deviations from intended interventions. This is no surprise as self-reporting was again a common concern (e.g. Admassie, 2009 and Findley, 2013). Supplementary Figure 3a illustrates the frequencies of other biases in our included QEDs, while Supplementary Figure 3b shows the risk of bias assessments for each individual study and each risk of bias category for QED studies.

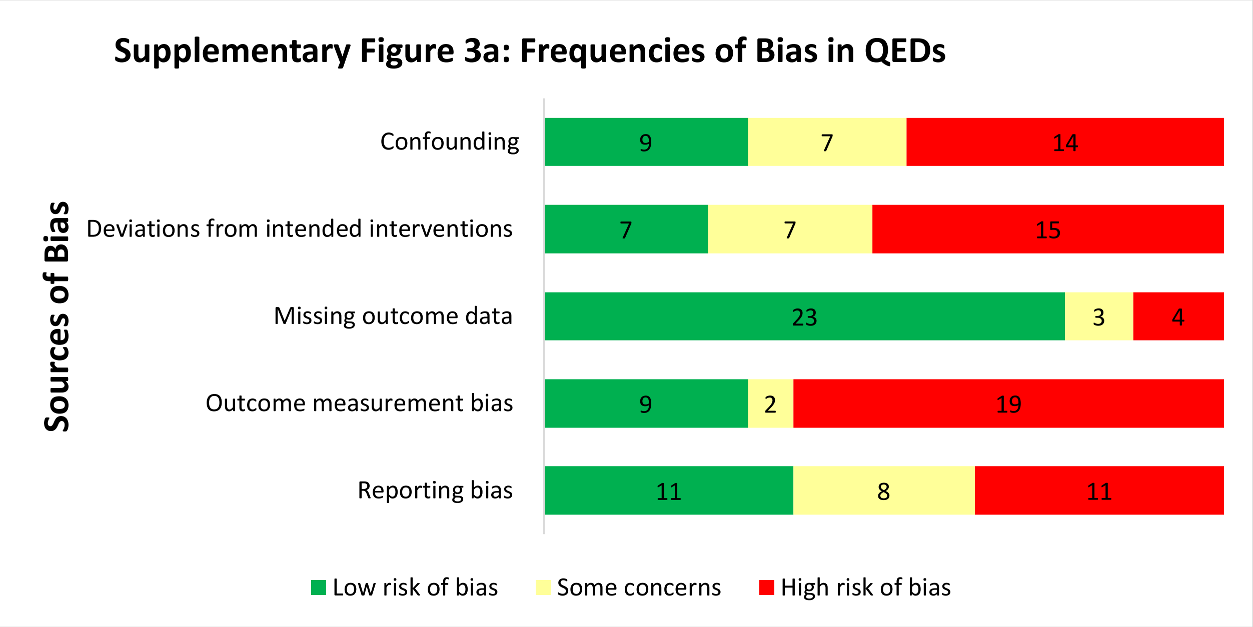

Supplelmentary Figure 3b: Risk of bias assessments for individual QED studies						
Study first author	Was the identification method free from any sources of bias	Bias due to missing outcome data	Was the study adequately protected against spill-overs, cross-overs, contamination, and performance bias?	Outcome measurement bias: Was the study free from biases in outcome measurement?	Analysis reporting: Was the study free from selective analysis reporting?	Overall Risk of Bias Assessment
Adamu, 2019						
Admassie, 2009						
Assegaai, 2018						
Banwat, 2015						
Biemba, 2016						
Calderón-Ortiz, 1996						

For peer review only - http://bmjopen.bmj.com/site/about/guidelines.xhtml

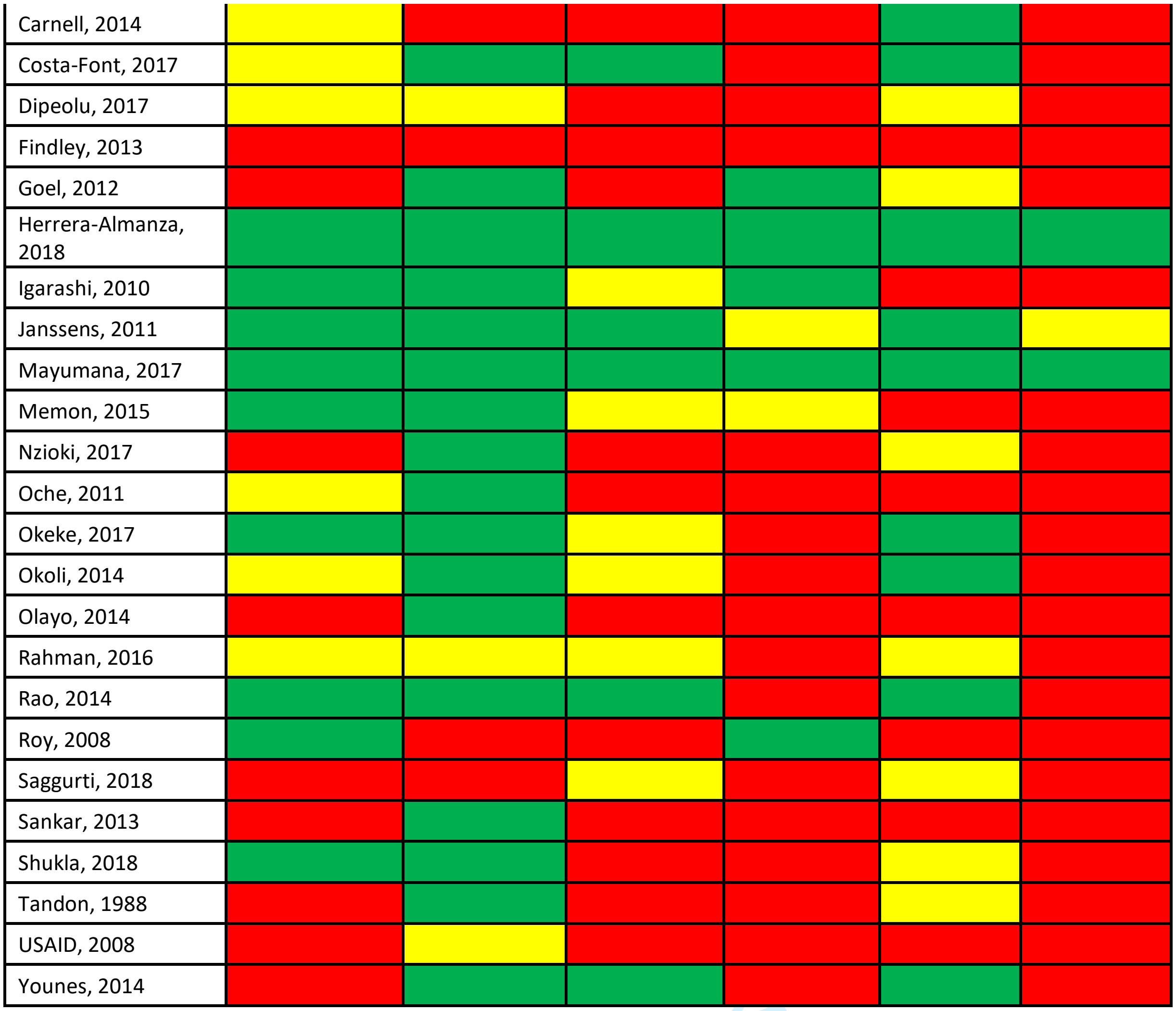

Qualitative risk of bias

Risk of bias assessments were conducted on 47 qualitative papers. Papers were scored as absent, weak, or present on 12 key elements and received corresponding scores of zero, one, or two for these ratings. Most (27) papers were missing at least some key elements, resulting in them receiving a zero value for these elements in their quality assessment scores (Supplementary Table 1; Supplementary Figure 4). However, 17 received quality assessment scores over 20, indicating that they received a value of two, or a "strong" rating, for most key elements (Supplementary Figure 5). The most common key elements to be missing were descriptions of sample characteristics and the analytic methods, with 17 studies failing to report on each of these (Supplementary Figure 5). The research aim was the most common key element to be stated strongly, with 39 papers clearly stating their aims.

Supplementary Table 1: Distribution of papers by strength of key elements

	Number
One or more key elements absent	27
One or more key elements weak	15

For peer review only - http://bmjopen.bmj.com/site/about/guidelines.xhtml

All key elements strong	5

Supplementary Figure 4: Distribution of papers by 12 key elements

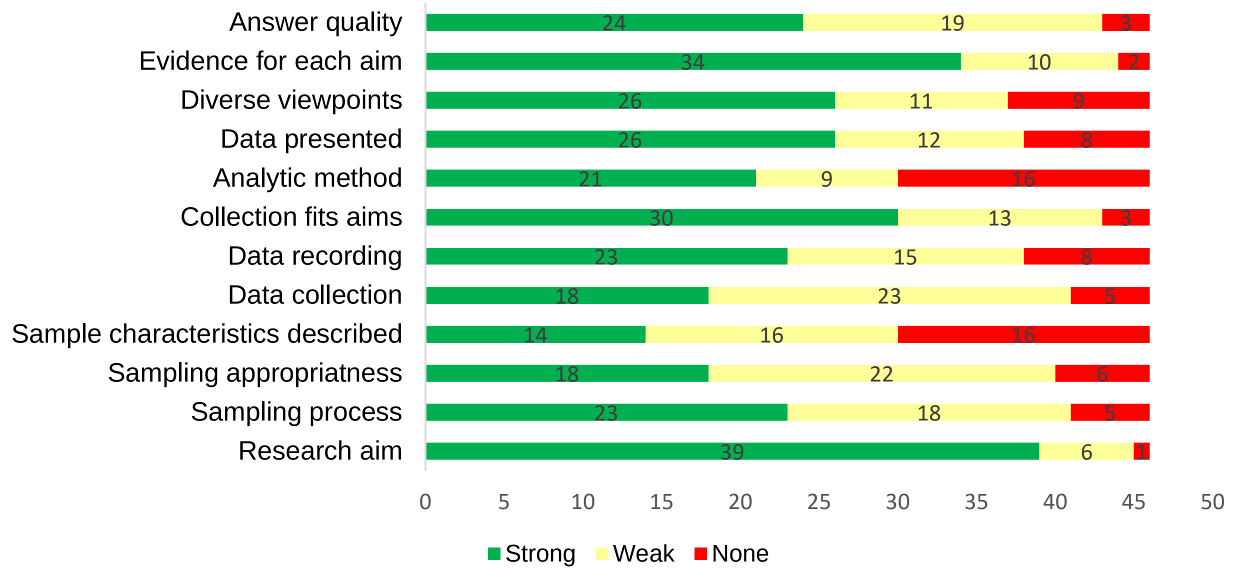

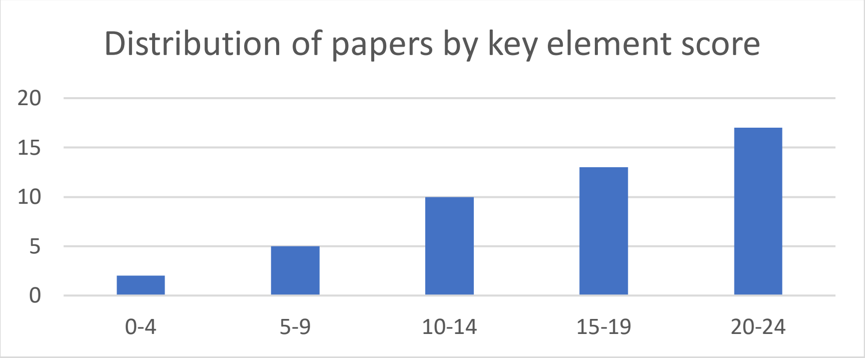

*Papers received scores of 0, 1 or 2 reflecting the strength of reporting on 12 key elements in the risk of bias assessment tool (Appendix E).

Supplementary Figure 5: Distribution of qualitative assessment scores*

Risk of bias in cost effectiveness estimates

The risk of bias analysis of the cost and cost-effectiveness analysis assessed the quality of underlying cost data, reporting, and analysis using information that was reported in the immunization studies. We assessed risk of bias along six primary dimensions which were adapted from a combination of tools, including: Doocy and Tappis (2017); Campbell Collaboration Economic Methods Policy Brief (Shemilt et al.,2008); and Methods for the Economic Evaluation of Health Care Programmes, (Drummond et al. 2015), that were adapted for cost analyses carried out in conjunction with impact evaluation studies of global development interventions.

For peer review only - http://bmjopen.bmj.com/site/about/guidelines.xhtml

This risk of bias tool specifically assesses the bias that arises from the collection and reporting of cost analysis in conjunction with impact evaluation studies of global development interventions. These studies often do not incorporate cost analyses. Indeed recent estimates suggest just 15-18% of impact analyses include any kind of cost analysis (Brown and Tanner 2019). This under-reporting of cost in conjunction with impact evaluations leads to very small samples from which to draw inferences about the cost, and cost-effectiveness of development interventions. Moreover, often when estimates are included with impact evaluations, the quality of the underlying data is low. Cost may have been added as an "after-thought", rather than planned for in advance or treated as a research endeavor. Data sources for cost information may not be well-documented or may have been estimated using 'back-of-the-envelope' techniques (this phrase literally is used in write-ups of cost methods). There is often insufficient detail in reporting of cost to assess the quality of estimates and data and basic robustness checks of the analysis are very infrequently performed.

Finally, this tool specifically considers the elements of cost and effectiveness that were extracted for the analysis of incremental cost effectiveness. Our particular measure estimates the non-vaccine cost per dose of interventions to increase absolute immunization coverage by one percent, using available data extracted from the evaluations wherever possible, or by following the steps and calculations as outlined in Ozawa et al. (2018). The specific inputs to this analysis include estimates of: total intervention cost (net of vaccine cost); the number of vaccine doses provided to each child; the endline proportion of children that received immunisations in the treatment and control groups; and the cost per child immunised.

Dimensions of potential bias in costs reported and cost-effectiveness estimates

Planned, organized, cost analysis. A common challenge of cost analysis is a lack of planning which often can lead to poor underlying data quality. For example, cost estimates are subject to recall bias when assessed long after the program and evaluation are completed. Therefore, we examine three indicators of an organized or planned cost analysis. Specifically, we look for a clear description of the form of economic evaluation, and a description of the method used. We also look for a clear statement of analytical perspective--which is the choice of which actor has standing in the costing -- this may be the donor, the implementing partner, or perspective of the costing may be from a societal point of view. The analytical perspective is important because it determines whose costs and benefits will be counted in the costing. It may, for example, directly impact how the constituent components of total cost are counted. Although we considered a standard ROB question which asks: 'Is a well-defined research question posed in answerable form?', we find that impact evaluation studies do not pose research questions in the expected form. The three questions, given equal weight in the risk of bias assessment, were:

a. Is the form of economic evaluation clearly stated?
b. Is the perspective of the costing stated?
c. Was a method of costing described?

Quality data sources. The highest quality data for assessing the cost of interventions in low-and middle-income settings often are drawn from the expenditure reports or accounting statements of the program implementers. Expenditures are better quality because they represent actual, rather than planned expenses and they often are subject to audit, making them more reliable as compared with cost data taken from program budgets (Levin et al. 2018).

d. What is the quality of the primary data sources used for the cost estimates?

Descriptive, detailed cost information. The quality of descriptive detail on costs allows judgement into cost components and whether they align with intervention activities. Since the three criteria tend to be positively correlated, we give equal weight to the three indicators of detailed cost information. Specifically, we assessed the descriptive detail of reported costs:

e. Whether costs are reported by ingredients (or input or resource),
f. Whether unit costs are reported, and

For peer review only - http://bmjopen.bmj.com/site/about/guidelines.xhtml

g. Whether the information is presented in an organized cost table

We consider the **quality of cost estimates** that were key inputs to the cost synthesis, specifically

h. Total cost: What is the quality of the specific data components of total cost? Since total cost is a key input to the analysis we examine how it was reported. For example, some studies say simply, the total cost of the program was USD $3 Million. In the absence of descriptive information, i.e. the elements that comprise total cost, it is difficult to assess the quality or reliability of the total cost estimate.
i. Vaccine cost: Can we tell if vaccines were excluded? Since we need to exclude vaccine cost, it must be excluded or reported separately from total cost to be valid in this study.
j. Cost per child immunized: If cost per child immunized was not directly estimated and reported, we take total cost divided by the number of children in the treatment group multiplied by the proportion of children that were immunized. If the quality of underlying data are poor, this estimate may be biased – with unclear magnitude and direction.
k. Number of vaccines. Was the number of vaccines reported from observation of study participants, or was it reported per protocol? When we estimate cost per vaccine dose, the cost estimate will be biased downwards (less costly per dose) if the study does not report the actual number of doses administered. This is because some children may already have received a part of the vaccine protocol before enrolling in the study.

Are **key cost details** reported which allow us to adjust for time and currency differences? In this assessment of bias risk, the responses are given equal weight since we need both elements to accurately adjust for time and currency differences.

l. Are details of inflation and currency conversion clearly stated?
m. Is the time horizon for costs clearly stated?

We consider the **quality of cost analysis**, specifically:

n. Is sensitivity analysis conducted?

Results

We developed evaluation criteria and applied the protocol described above to the 22 immunisation evaluations that reported cost information. The results of this analysis are summarized below in Supplementary Figure 6, below.

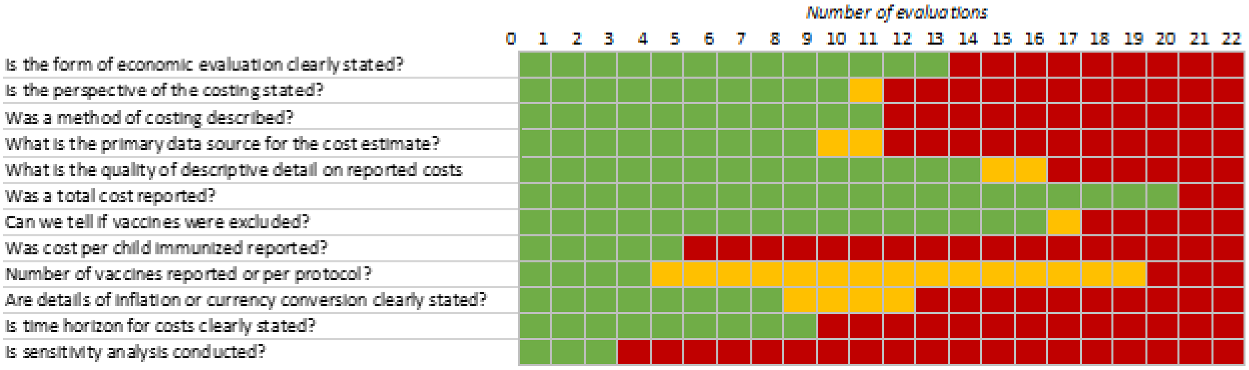

Note: green cells indicate that clear information was presented in the report to assess the risk of bias; yellow cells indicate incomplete information; and red cell indicate we found very little or no information to assess bias risk.

For peer review only - http://bmjopen.bmj.com/site/about/guidelines.xhtml

Supplementary Figure 6: Risk of bias assessment of cost and cost-effectiveness estimates used in the analysis

- *Just over half of the included evaluations appear to have carried out a planned, organized, cost analysis.* Thirteen of 22 evaluations clearly state the form of economic evaluation (i.e. cost-effectiveness analysis); ten studies report the perspective of the costing, which is key for judging the correct inclusion and exclusion criteria for the components of a total cost estimate; and eleven of 22 evaluations describe the method of costing that was used to collect cost data (i.e. the ingredients method).
- *Indications of the quality of underlying cost data are mixed.* Nine of the 22 evaluations used expenditure reports to generate cost estimates, two used budgets and the remaining 11 evaluations provided no information on the provenience of the underlying cost data that was used in the analysis.
- *The quality of the descriptive detail on reported costs was mixed. Just over half of all evaluations (14 of 22)* provided thorough, descriptive information on costs; two evaluations provided some descriptive information, e.g. a breakdown of key unit costs; and six evaluations gave very minimal or no descriptive information on costs.
- *In 17 of 22 evaluations, we have high confidence that total cost excludes vaccines costs.* A majority of evaluations reported an estimate of total cost (20 of 22 evaluations). In 16 of the 20 total cost estimates, there were clear indications that vaccine costs had been excluded from the estimate, and in one case, vaccine costs were included but reported separately so that we could subtract vaccine cost from total cost to derive the comparable total cost. It was not possible to tell if vaccines were excluded from the total cost estimates of five evaluations.
- *The cost per immunized child* – was only reported by the authors in five of 22 evaluations and for the remaining 16 was estimated by the authors to compile Supplementary Table 22.
- *The number of vaccine doses received per treated child was reported in only four evaluations;* in 15 studies, vaccine doses per child were estimated based on information "per protocol" which does not account for children's partial vaccination status at the point of enrollment in the study.
- *Fewer than half of the evaluations included the key information needed to adjust for time, currency, inflation or base year differences in the timing of expenditures.* This lack of reporting makes it very difficult to have complete confidence in the comparability of the cost estimates we generated.
- *Only three evaluations reported any kind of sensitivity analysis*, an indication of analytical robustness.

For peer review only

Appendix 9: Quantitative results – all community engagement interventions

Appendix A presents supplementary data for analyses presented in the main body of the manuscript for the primary outcomes of community engagement interventions, including funnel plots of publication bias and outlier analyses. It also includes a full presentation of the supplementary outcomes that were precluded from the main body of the manuscript due to word limitations.

Primary outcomes

Full immunisation

As a robustness check, we used robust variance analysis and included all dependent effects in the analysis, totaling 53 effects from the same 28 studies (*df* = 25.5). The overall average effect was slightly smaller but still significant ($\hat{\mu} = 0.11$ [95% CI: 0.04 to 0.18], *p* = .002). Sensitivity analyses show the effect to be sensitive to all values of Rho. The rank correlation test indicated funnel plot asymmetry ($p = 0.03$) but the regression test did not ($p = 0.57$; see Supplementary Figure 7). A trim and fill analysis indicated an identical effect size (see Supplementary Figure 7b)

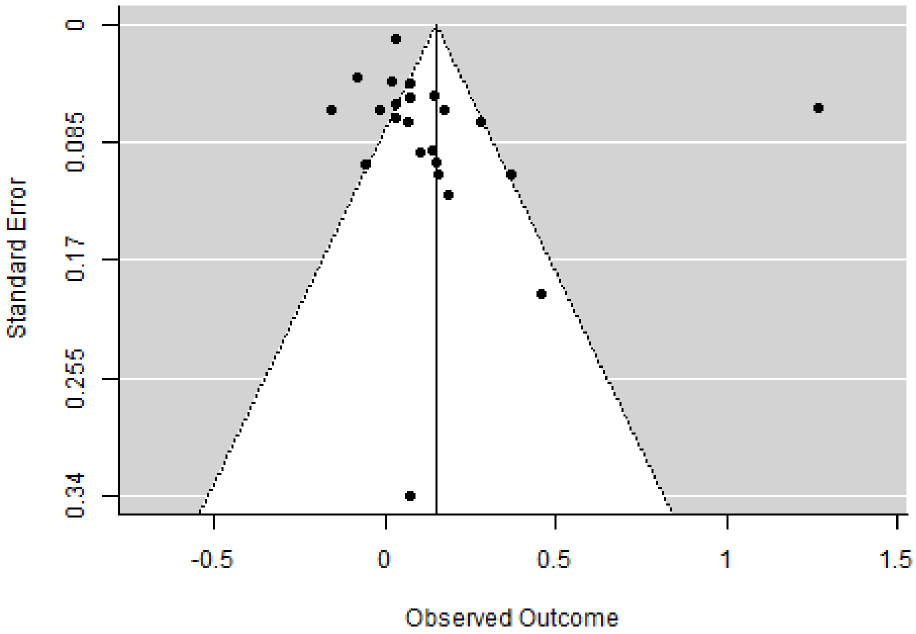

Supplementary Figure 7. Funnel plot for studies examining the effect of community engagement interventions on full immunisation

For peer review only - http://bmjopen.bmj.com/site/about/guidelines.xhtml

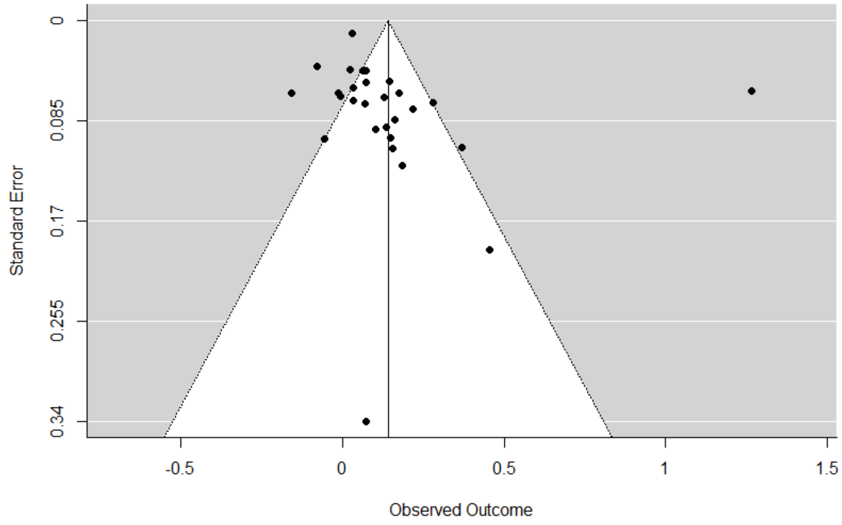

Supplementary Figure 7b. Trim and fill plot for studies examining the effect of community engagement interventions on full immunisation

DPT3

According to the Q-test, the true outcomes appear to be heterogeneous ($Q(21) = 90.43$, $p < 0.01$, $\hat{\tau}^2 = 0.01$, $I^2 = 76.78\%$). The rank correlation test indicated funnel plot asymmetry ($p = 0.04$) but not the regression test ($p = 0.06$), but trim and fill analyses indicated an identical effect size (Supplementary Figures 8 & 9). An examination of the studentized residuals revealed that none of the studies had a value larger than ± 3.05 and hence there was no indication of outliers in the context of this model. According to the Cook's distances, none of the studies could be considered to be overly influential.

Publication year was a significant moderator such that each additional year reduced the size of the effect by .014 standard deviation units ($\hat{B} = -0.014, p = 0.019$ [95% CI: -0.03 to -0.002]). In other words, new studies have found smaller effects. There were no other significant moderators in the context of this model.

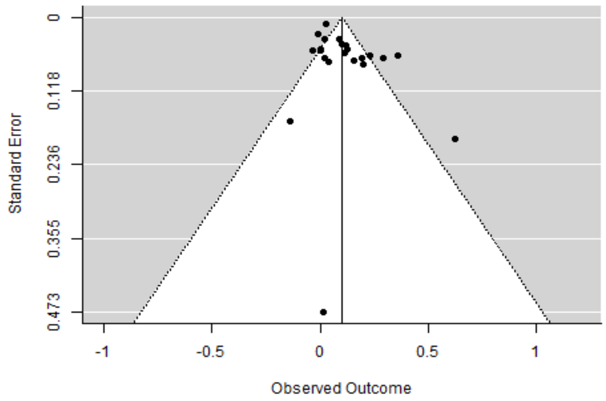

Supplementary Figure 8. Funnel plot for studies examining the effect of community engagement interventions on DPT3 vaccination

For peer review only - http://bmjopen.bmj.com/site/about/guidelines.xhtml

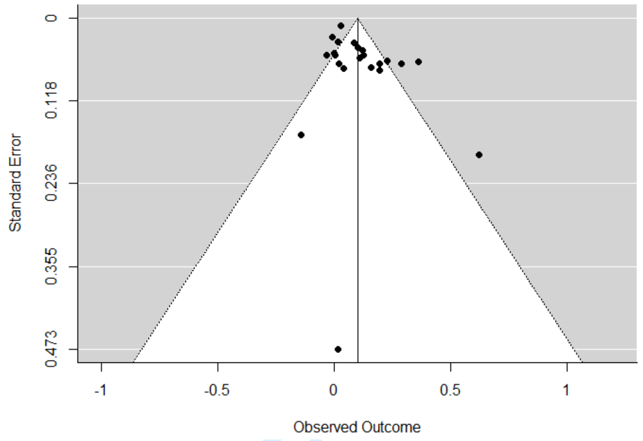

Supplementary Figure 9. Trim and fill plot for studies examining the effect of community engagement interventions on DPT3 vaccination

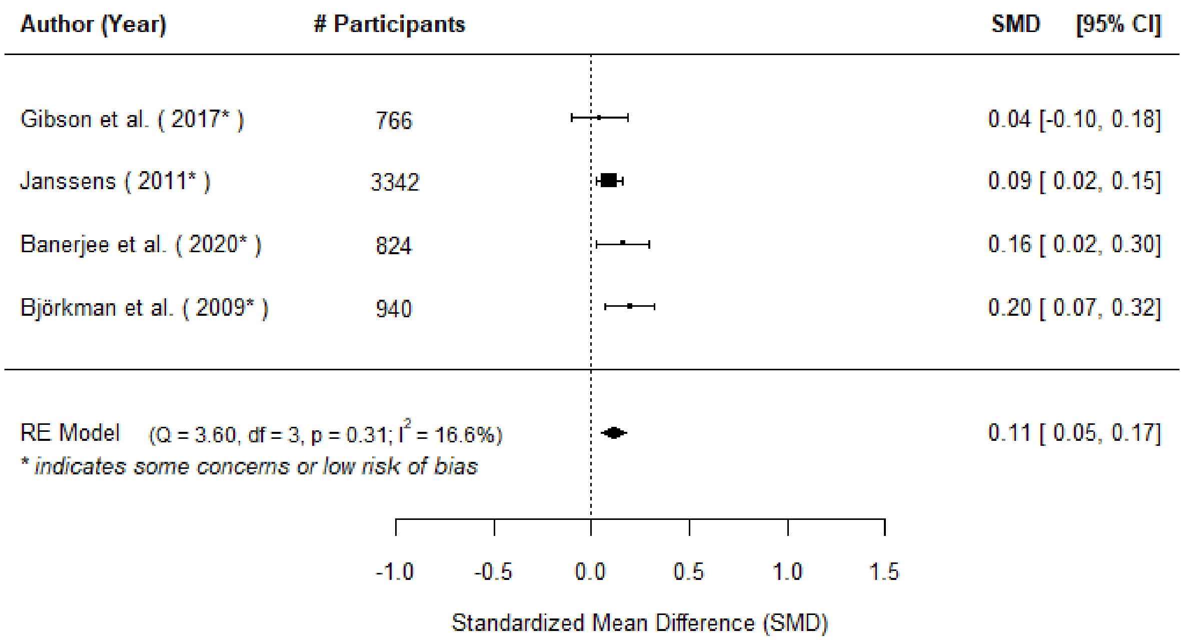

Supplementary Figure 10. *Forest plot showing the observed outcomes and the estimate of the random-effects model for the impact of community engagement interventions on DPT3 vaccination when low quality studies are removed*

As a robustness check, we used robust variance analysis and included all dependent effects in the analysis, totaling 36 effects from the same 22 studies (*df* = 18.2). The overall average effect was slightly smaller but still significant ($\hat{\mu} = 0.10$ [95% CI: 0.05 to 0.15], $p < .001$). Sensitivity analyses show the effect to be sensitive to all values of Rho.

Measles

An examination of the studentized residuals revealed that one study (Sankar 2013) had a value larger than ± 3.02 and may be a potential outlier in the context of this model. Indeed, sensitivity analysis leaving out Sankar (2013) would result in an increase in the average effect ($\hat{\mu} = 0.08$ [95% CI: 0.05 to 0.12], and it was still statistically

For peer review only - http://bmjopen.bmj.com/site/about/guidelines.xhtml

significant ($z = 4.44\ p < 0.001$). According to the Cook's distances, none of the studies could be considered to be overly influential. A funnel plot of the estimates is shown in Figure A6. Both the rank correlation and the regression test indicated potential funnel plot asymmetry ($p = 0.05$ and $p = 0.02$, respectively). The trim and fill analysis (Supplementary Figure 13) estimates a smaller and non-significant effect ($\hat{\mu} = 0.02$ [95% CI: -0.02 to 0.06], $p =$ .36 Supplementary Figure 11 presents the results when low quality studies are removed.

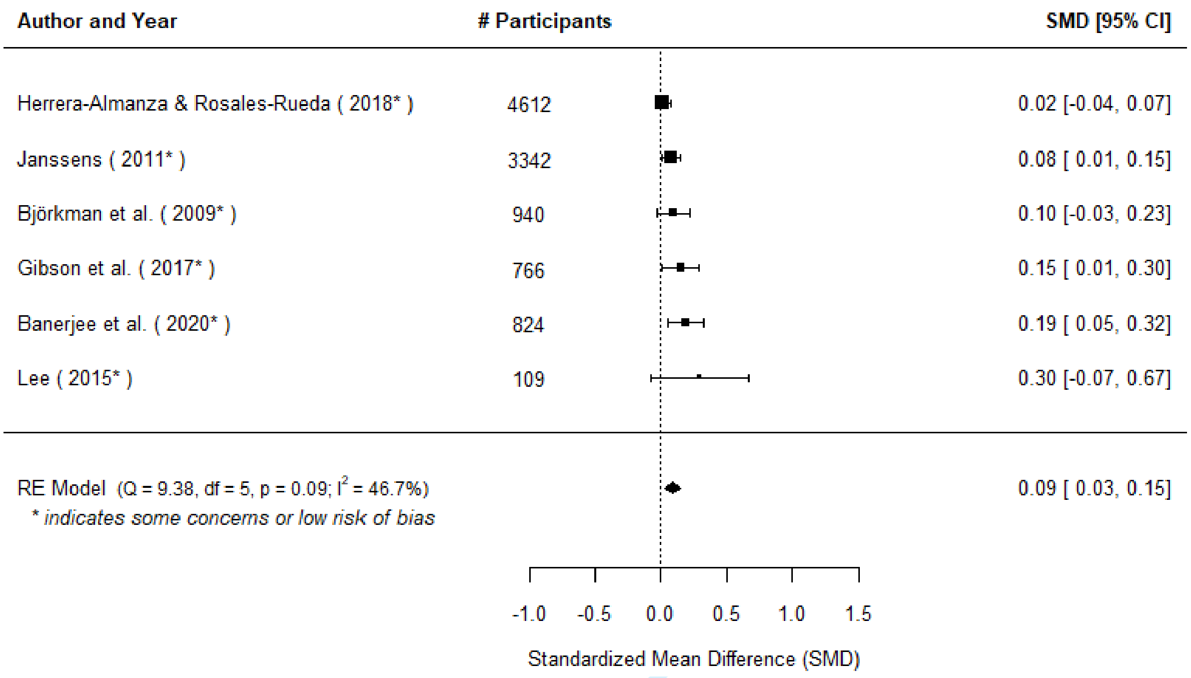

Supplementary Figure 11. Forest plot showing the observed outcomes and the estimate of the random-effects model for the impact of community engagement interventions on measles vaccination when low quality studies are removed

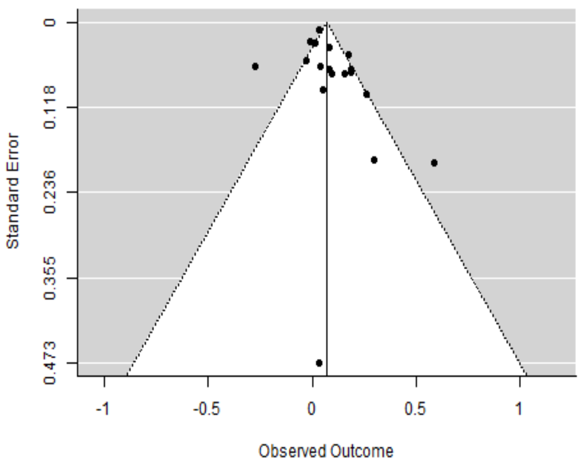

Supplementary Figure 12. Funnel plot for studies examining the effect of community engagement interventions on measles vaccination

For peer review only - http://bmjopen.bmj.com/site/about/guidelines.xhtml

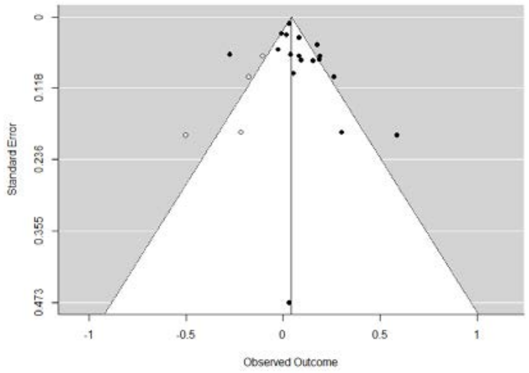

Supplementary Figure 13. Trim and fill funnel plot for studies examining the effect of community engagement interventions on measles vaccination

As a robustness check, we used robust variance analysis and included all dependent effects in the analysis, totaling 32 effects from the same 20 studies (*df* = 15.5). The overall average effect was slightly smaller but still significant ($\hat{\mu} = 0.06$ [95% CI: 0.01 to 0.11], p = .03). Sensitivity analyses show the effect to be sensitive to all values of Rho.

Vaccination timeliness: Full immunisation schedule

We included a total of $k = 5$ studies in the analysis. The estimated average outcome was $\hat{\mu} = 0.15$ ([95% CI: 0.07 to 0.24], $z = 3.41$, $p < 0.001$; $Supplemenyary\ Figure$ 14), indicating a small but significant benefit to the treated group compared to the control group. According to the Q-test, there was no significant amount of heterogeneity in the true outcomes ($Q(4) = 4.43$, $p = 0.35$, $\hat{\tau}^2 = 0.00$, $I^2 = 9.66\%$). An examination of the studentized residuals revealed that none of the studies had a value larger than ± 2.58 and hence there was no indication of outliers in the context of this model. According to the Cook's distances, none of the studies could be considered to be overly influential. With only one study not assessed as high risk of bias, we were unable to conduct sensitivity analyses by study quality. With no significant heterogeneity, we did not test for moderations. There were no dependent effects, so we could not use RVE as a robustness check in this case.

For peer review only - http://bmjopen.bmj.com/site/about/guidelines.xhtml

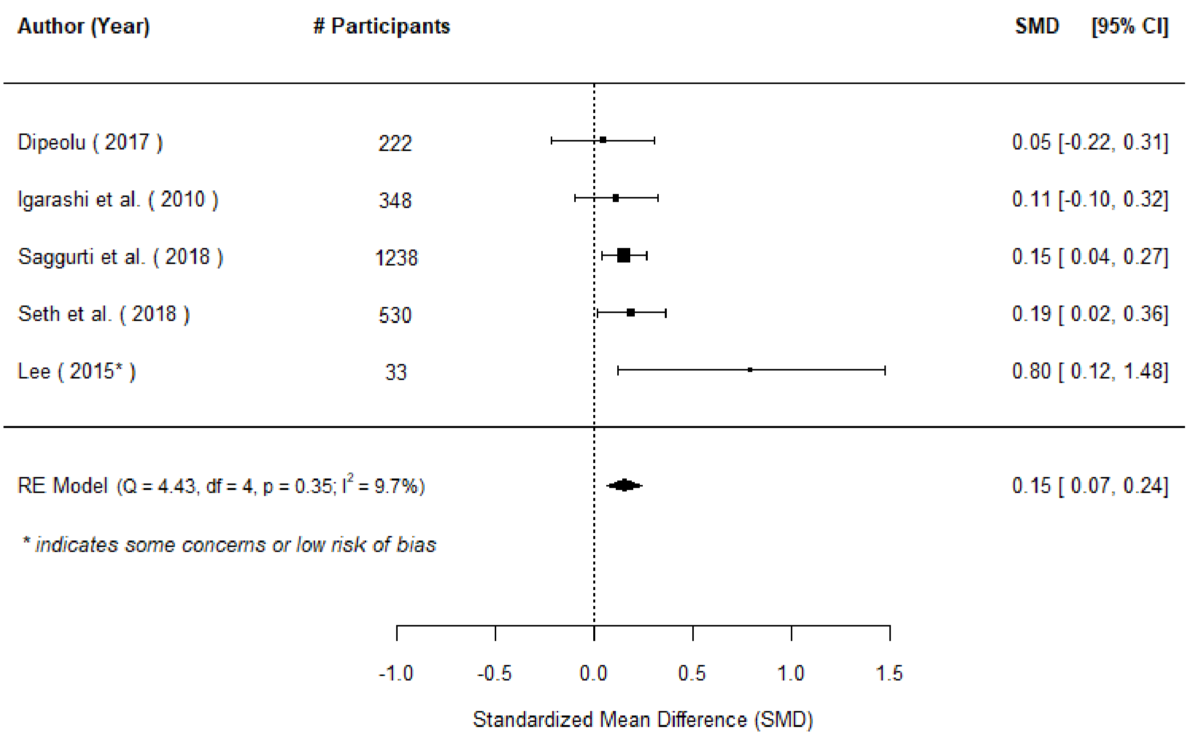

Supplementary Figure 14. Forest plot showing the observed outcomes and the estimate of the random-effects model for the impact of community engagement interventions on timeliness of full childhood immunisation

Vaccination timeliness: DPT3

A total of $k = 7$ studies examined the relationship between community engagement interventions and timeliness of DPT3 vaccination. The estimated average outcome was $\hat{\mu} = 0.09$ ([95% CI: 0.03 to 0.14], $z = 3.00$, $p < 0.01$), indicating a small but significant benefit to the intervention group compared to the control group (Supplementray Figure 15). According to the Q-test, there was no significant amount of heterogeneity in the true outcomes ($Q(6) = 5.09$, $p = 0.53$, $\hat{\tau}^2 = 0.00$, $I^2 = 0.00\%$). An examination of the studentized residuals revealed that none of the studies had a value larger than ± 2.69 and hence there was no indication of outliers in the context of this model. According to the Cook's distances, none of the studies could be considered to be overly influential. With no heterogeneity, moderator analyses were not appropriate. There were no dependent effects, so we could not use RVE as a robustness check in this case. When low quality studies are removed, only two studies remain, and the estimated average outcome increases to $\hat{\mu} = 0.11$ [95% CI: -0.05 to 0.27], but becomes non-significant $z = 1.32$, $p = 0.19$). However, with only two studies this should be interpreted with caution.

For peer review only - http://bmjopen.bmj.com/site/about/guidelines.xhtml

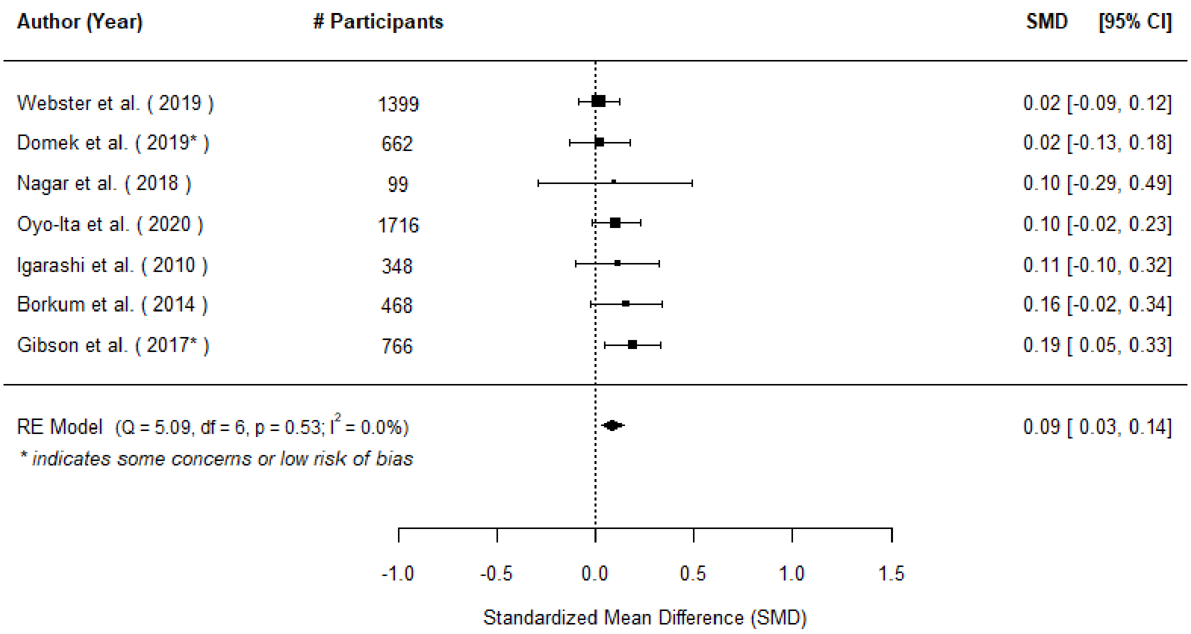

Supplementary Figure 15. Forest plot showing the observed outcomes and the estimate of the random-effects model for the impact of community engagement interventions on the timeliness of DPT3 vaccination.

Vaccination timeliness: Measles

Only $k = 2$ studies using community engagement interventions reported on the timeliness of measles vaccinations. The estimated average outcome was $\hat{\mu} = 0.23$ ([95% CI: 0.14 to 0.32]$, z = 5.06, p < 0.001; see$ Supplementary $Figure$ 16), indicating a small but significant benefit to the treated group compared to the control group. According to the Q-test, there was no significant amount of heterogeneity in the true outcomes ($(Q(1) = 0.23., p = 0.63, \hat{\tau}^2 = 0.00, I^2 = 0.00\%)$). An examination of the studentized residuals revealed that none of the studies had a value larger than ± 2.69 and hence there was no indication of outliers in the context of this model. According to the Cook's distances, none of the studies could be considered to be overly influential. With no heterogeneity and only two studies contributing effects, moderator analyses were not appropriate and we were unable to conduct sensitivity analyses by study quality or test for publication bias. There were no dependent effects, so we could not use RVE as a robustness check in this case.

For peer review only - http://bmjopen.bmj.com/site/about/guidelines.xhtml

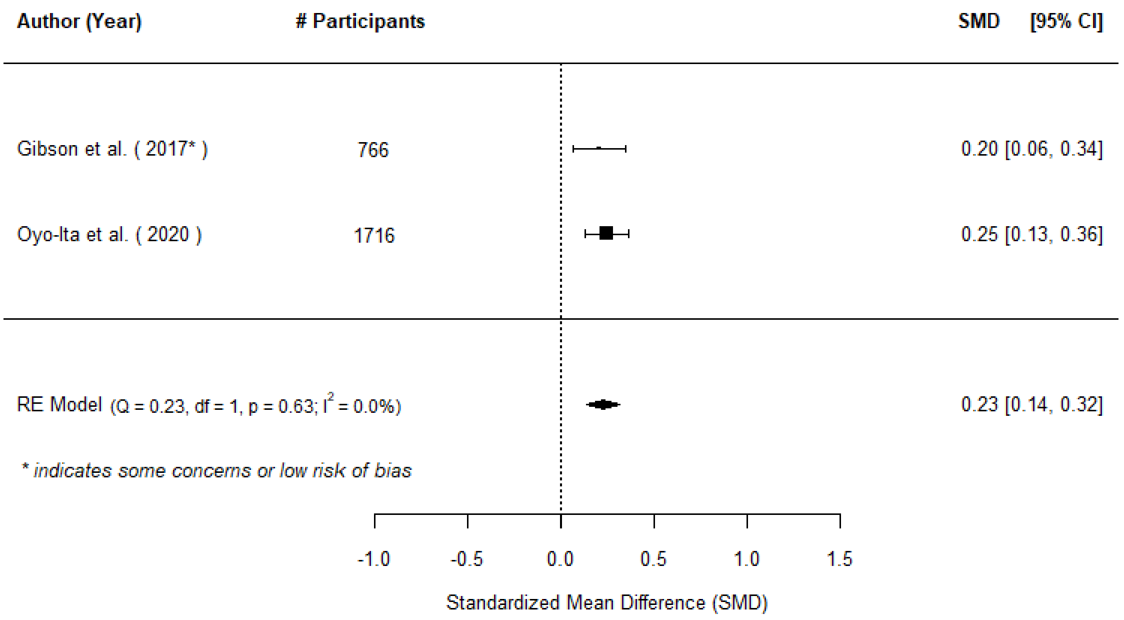

Supplementary Figure 16. *Forest plot showing the observed outcomes and the estimate of the random-effects model for the impact of community engagement interventions on the timeliness of measles vaccination.*

Secondary outcomes

Partial immunisation

We included a total of $k = 9$ studies the analysis. The estimated average outcome based on the random-effects model was $\hat{\mu} = 0.23$ ([95% CI: 0.09 to 0.37], $z = 3.15$, $p = 0.002$), indicating a benefit for the intervention group compared to the control group (see Supplementary Figure 17).

According to the Q-test, the true outcomes appear to be heterogeneous ($Q(8) = 219.11$, $p < 0.001$, $\hat{\tau}^2 = 0.04$, $I^2 = 96.351\%$). An examination of the studentized residuals revealed that none of the studies had a value larger than ± 2.77 and hence there was no indication of outliers in the context of this model. Likewise, according to the Cook's distances, none of the studies could be considered to be overly influential. With eight out of the nine studies being assessed as high risk of bias, we were unable to conduct sensitivity analysis by study quality.

For peer review only - http://bmjopen.bmj.com/site/about/guidelines.xhtml

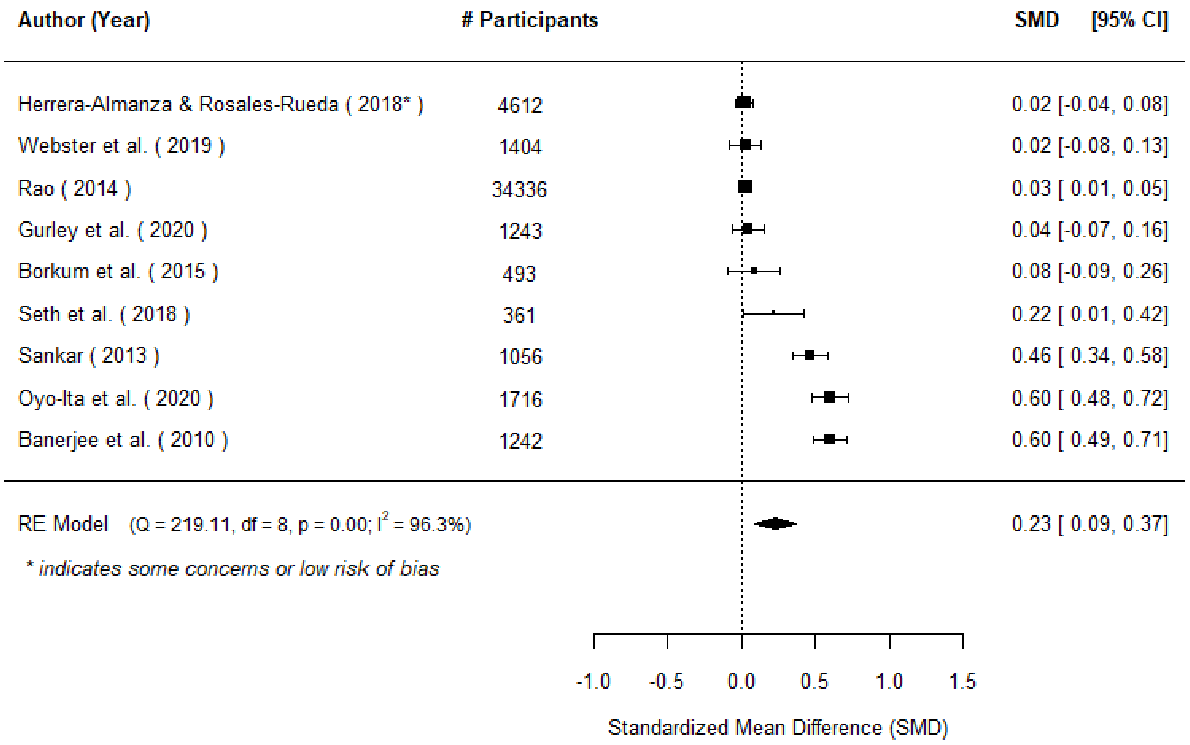

Supplementary Figure 17. Forest plot showing the observed outcomes and the estimate of the random-effects model for the impact of community engagement interventions on partial childhood immunisation

As a robustness check, we used robust variance analysis and included all dependent effects in the analysis, totaling 13 effects from the same 9 studies (*df* = 7.96). The overall average effect was slightly smaller but still significant ($\hat{\mu}$ = 0.21 [95% CI: 0.03 to 0.38], *p* = .03). Sensitivity analyses show the effect to be sensitive to all values of Rho.

BCG

We included a total of k = 12 studies in the analysis. The estimated average outcome based on the random-effects model was $\hat{\mu} = 0.06$ ([95% CI: 0.01 to 0.11], $z = 2.28$, $p = 0.02$), indicating a very small but significant benefit to the intervention participants compared to the control participants (see Supplementary Figure 18). According to the Q-test, the true outcomes appear to be heterogeneous ($Q(11) = 84.22$, $p < 0.01$, $\hat{\tau}^2 = 0.00$, $I^2 = 86.94\%$). An examination of the studentized residuals revealed that one study (Banerjee 2010) had a value larger than ± 2.87 and may be a potential outlier in the context of this model. According to the Cook's distances, Banerjee (2010) could also be considered to be overly influential. Sensitivity analyses leaving each study out indicated that removing Banerjee (2010) would reduce the overall average effect ($\hat{\mu} = 0.02$ (95% CI: -0.005 to 0.04), and the resulting effect would be non-significant ($z = 1.51$, $p = 0.13$). Moderator analysis revealed that publication year was a significant predictor such that more recent studies find smaller effects that older studies, with each additional year reducing the size of the effect by .03 standard deviation units ($\beta = -0.03$ [95% CI: -0.05 to -0.001], $p = .04$). No other moderators were significant (see Supplementary Table 3). Only three studies were high or moderate quality. When those three studies were synthesised, the resulting effect was reduced ($\hat{\mu} = 0.01$ [95% CI: -0.08 to 0.10]) and no longer significant ($z = 0.22$, $p = 0.82$).

For peer review only - http://bmjopen.bmj.com/site/about/guidelines.xhtml

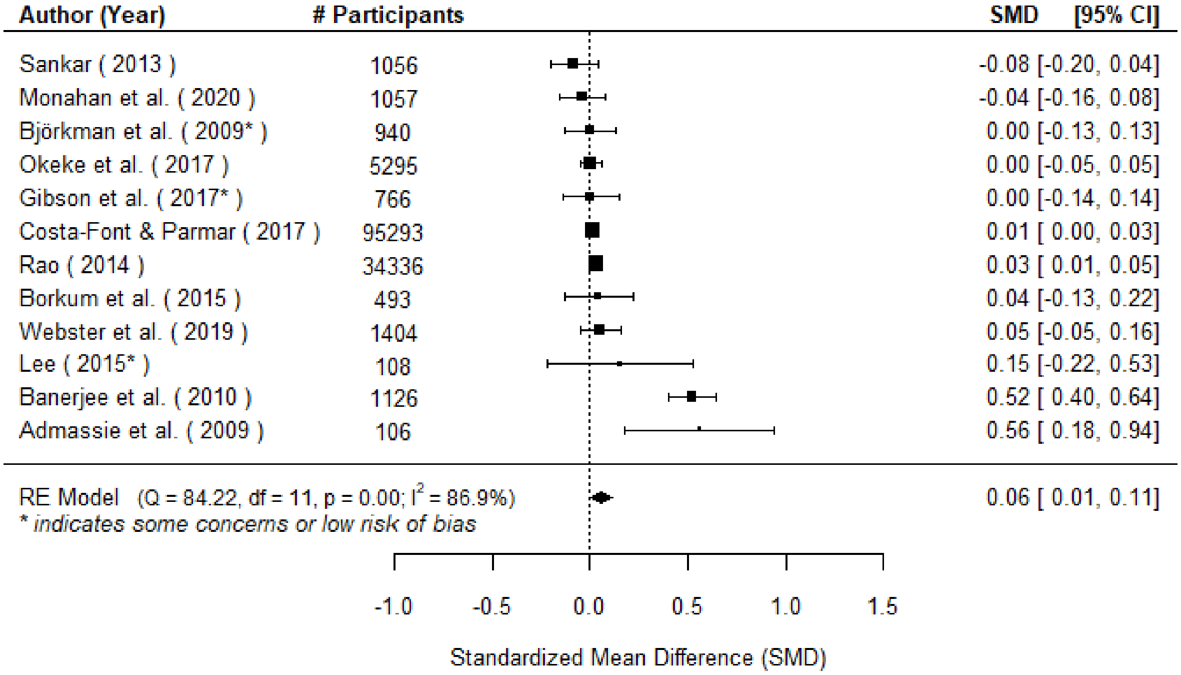

Supplementary Figure 18. Forest plot showing the observed outcomes and the estimate of the random-effects model for the impact of community engagement interventions on BCG vaccination.

A funnel plot of the estimates is shown in Supplementary Figure *18b*. The regression test indicated funnel plot asymmetry ($p = 0.04$) but not the rank correlation test ($p = 0.20$).

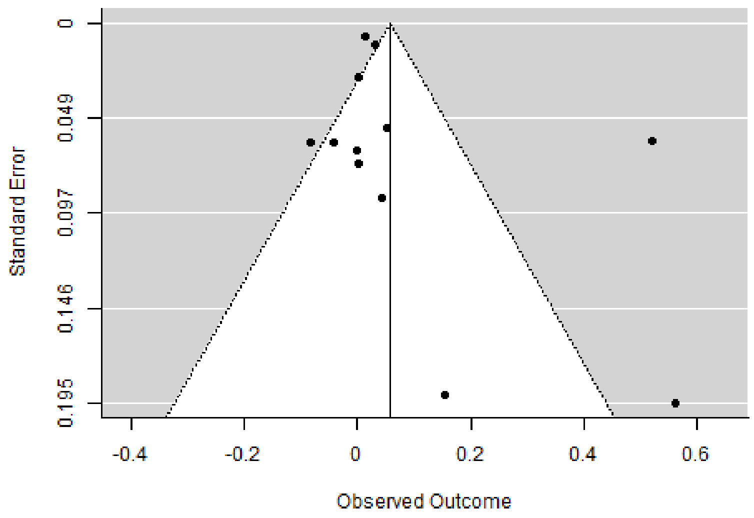

Supplementary Figure 18b: *Funnel plot*

As a robustness check, we used robust variance analysis and included all dependent effects in the analysis, totaling 16 effects from the same 12 studies (*df* = 7.4). The overall average effect was slightly smaller and non-significant ($\hat{\mu} = 0.04$ [95% CI: −0.02 to 0.10], *p* = .20). Sensitivity analyses show the effect to be sensitive to all values of Rho.

For peer review only - http://bmjopen.bmj.com/site/about/guidelines.xhtml

DPT1

We included a total of $k = 8$ studies in the analysis. The estimated average outcome based on the random-effects model was $\hat{\mu} = 0.04$ ([95% CI: -0.04 to 0.11], $z = 0.99$, $p = 0.32$), indicating no difference between the intervention group and the control group (see Supplementary Figure 19). According to the Q-test, the true outcomes appear to be heterogeneous ($Q(7) = 30.18$, $p < 0.01$, $\hat{\tau}^2 = 0.01$, $I^2 = 76.81\%$). An examination of the studentized residuals revealed that none of the studies had a value larger than ± 2.73 and hence there was no indication of outliers in the context of this model. According to the Cook's distances, none of the studies could be considered to be overly influential. When we removed studies assessed as high risk of bias, two studies remained, and the average effect increased slightly ($\hat{\mu} = 0.04$ ([95% CI: -0.04 to 0.11]), but was still non-significant ($z = 0.99$, $p = 0.32$). Again, with only two studies contributing effects, this must be interpreted with caution.

We tested for potential sources of heterogeneity and found that study design was a significant predictor of the effect such that studies using quasi-experimental designs had smaller effects than RCTs by .15 standard deviation units ($\beta = -0.15$ [95% CI: -0.29 to -0.02], $p = .03$). We also found that there was a significant difference in the size of effects between programmes implemented by government agencies and those that were not, such that programmes implemented by government agencies (either alone or in tandem with another agency) had larger effects than programmes not implemented by a government agency by .17 standard deviation units ($\beta = 0.17$ [95% CI: 0.01 to 0.34], $p = .04$). No other moderators were significant (see Supplementary Table 3).

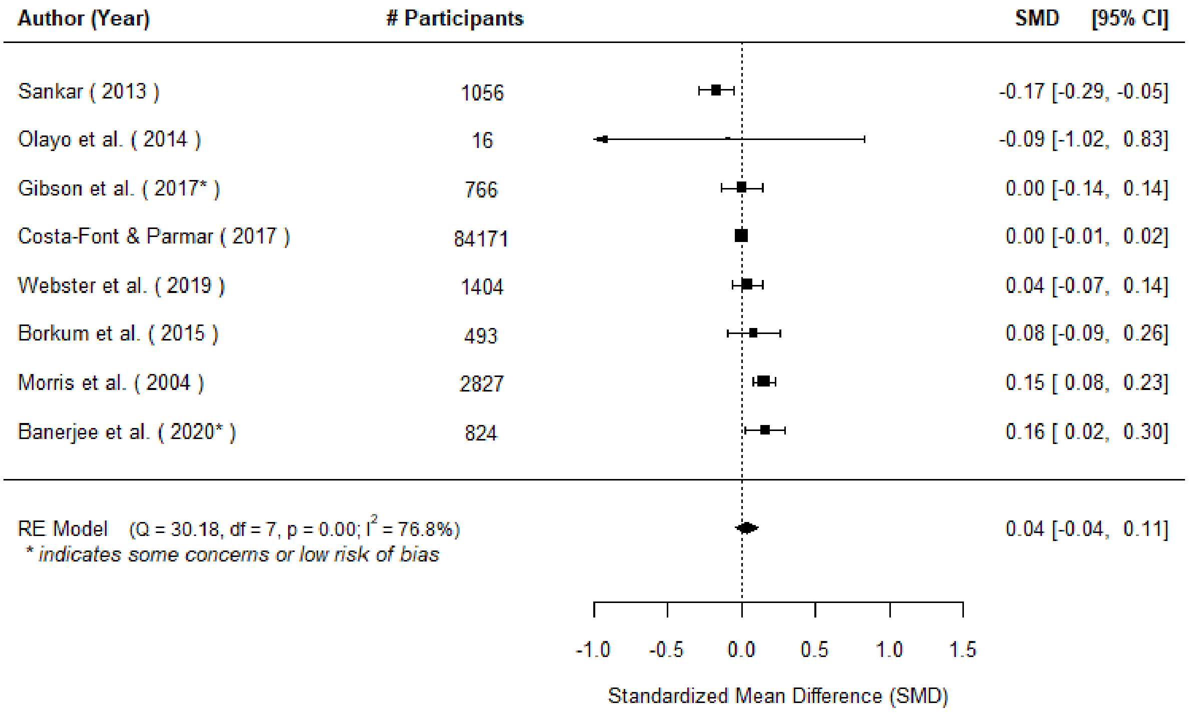

Supplementary Figure 19. Forest plot showing the observed outcomes and the estimate of the random-effects model for the impact of community engagement interventions on DPT1 vaccination.

As a robustness check, we used robust variance analysis and included all dependent effects in the analysis, totaling 21 effects from the same 8 studies ($df = 5.24$). The overall average effect was smaller and still non-significant ($\hat{\mu} = 0.01$ [95% CI: -0.06 to 0.09], $p = .66$). Sensitivity analyses show the effect to be sensitive to all values of Rho.

DPT2

For peer review only - http://bmjopen.bmj.com/site/about/guidelines.xhtml

We included a total of $k = 5$ studies in the analysis. The estimated average outcome based on the random-effects model was $\hat{\mu} = 0.07$ (95% CI: 0.01 to 0.12). Therefore, the average outcome differed significantly from zero ($z = 2.23$, $p = 0.03$), indicating small but significant benefit to the treated group compared to the control group. According to the Q-test, there was no significant amount of heterogeneity in the true outcomes ($Q(4) = 3.34$, $p = 0.50$, $\hat{\tau}^2 = 0.00$, $I^2 = 0.00\%$). An examination of the studentized residuals revealed that none of the studies had a value larger than ± 2.58 and hence there was no indication of outliers in the context of this model. According to the Cook's distances, none of the studies could be considered to be overly influential. With no heterogeneity present, we did not examine potential sources of variation. When high risk of bias studies were removed, the resulting average effect increased slightly ($\hat{\mu} = 0.11$ [95% CI: 0.01 to 0.20]) and was still significantly different from zero ($z = 2.12$, $p = 0.03$).

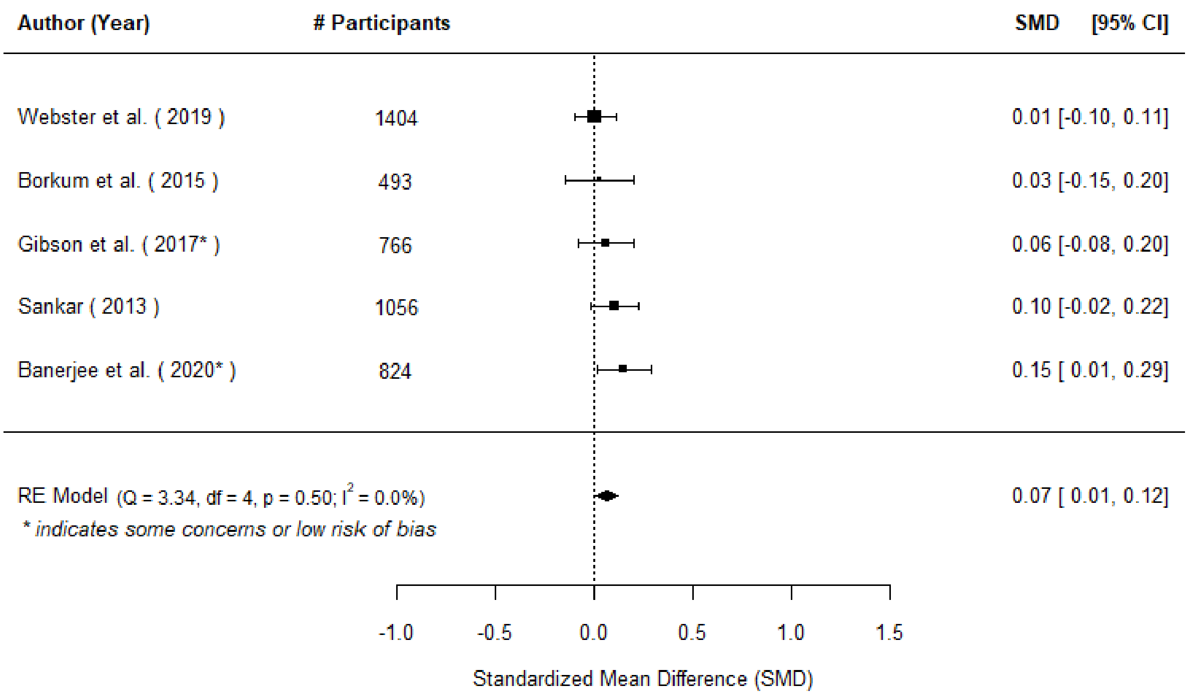

Supplementary Figure 20*. Forest plot showing the observed outcomes and the estimate of the random-effects model for the impact of community engagement interventions on DPT2 vaccination.*

This group of studies was not sufficiently powered for a robustness check using robust variance analysis (*df* = 3.51).

OPV0

We included a total of $k = 5$ studies in the analysis. The estimated average outcome based on the random-effects model was $\hat{\mu} = 0.10$ (95% CI: -0.06 to 0.26). Therefore, the average outcome did not differ significantly from zero ($z = 1.24$, $p = 0.22$), indicating no difference between the intervention and control groups (see Supplementary Figure 21). According to the Q-test, the true outcomes appear to be heterogeneous ($Q(4) = 45.74$, $p < 0.01$, $\hat{\tau}^2 = 0.03$, $I^2 = 91.26\%$). An examination of the studentized residuals revealed that one study (Sankar 2013) had a value larger than ± 2.58 and may be a potential outlier in the context of this model. According to the Cook's distances, Sankar (2013) could also be considered to be overly influential. Indeed, sensitivity analyses leaving each study out indicated that removing Sankar (2013) would reduce the overall average effect ($\hat{\mu}$ = -0.01 [95% CI: -0.01 to 0.03]) which would become non-significant (z = 1.02, p = .31).

Exposure to the intervention (in months) was a significant predictor of variation on OPV0 vaccinations such that each additional month of exposure increased the size of the effect by 0.02 standard deviation units (β = .02, [95%

For peer review only - http://bmjopen.bmj.com/site/about/guidelines.xhtml

CI: 0.01 to 0.03]; $p < .001$). Publication year was also a significant predictor of variation on OPV0 vaccinations such that each additional year decreased the size of the effect by 0.07 standard deviation units (β = -0.07, [95% CI: −0.11 to −0.02]; $p = 0.002$), meaning more recent studies found smaller effects than older studies. With only one study that was not assessed as high risk of bias, we could not conduct a sensitivity analysis by study quality.

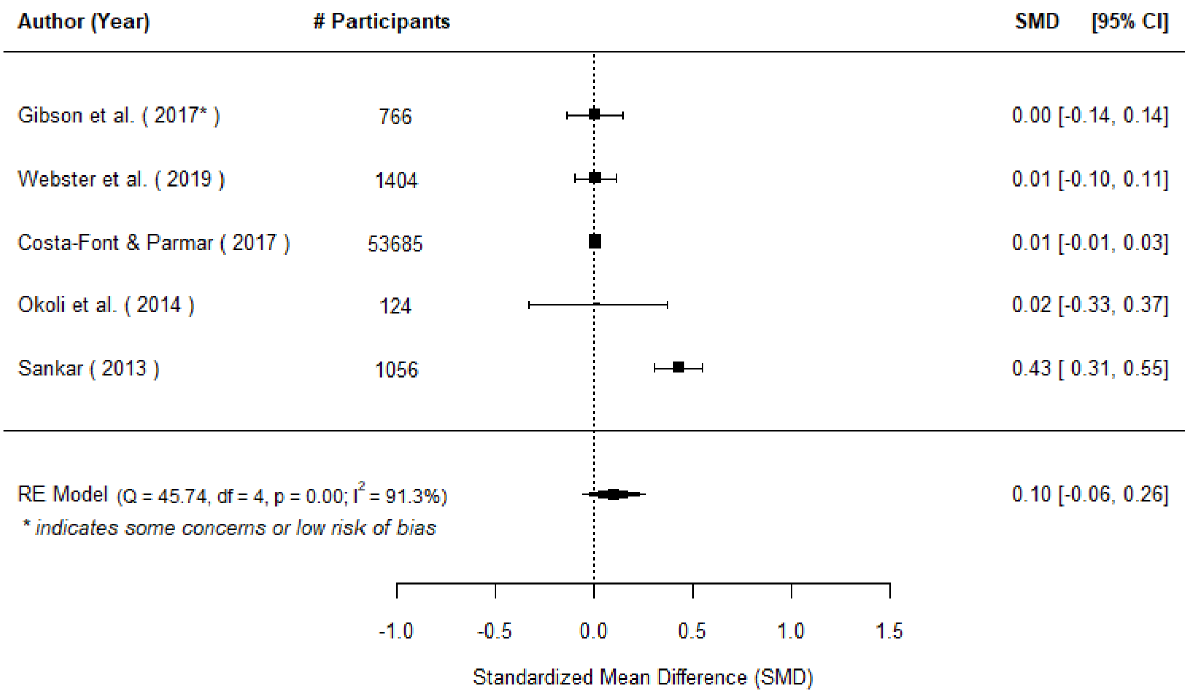

Supplementary Figure 21. Forest plot showing the observed outcomes and the estimate of the random-effects model for the impact of community engagement interventions on OPV0 vaccination.

This group of studies was not sufficiently powered for a robustness check using robust variance analysis (*df* = 3.76).

OPV1

We included a total of $k = 5$ studies in the analysis. The estimated average outcome based on the random-effects model was $\hat{\mu} = 0.08$ ([95% CI: 0.004 to 0.15], $z = 2.06$, $p = 0.04$), indicating a very small but significant benefit to the treated group compared to the control group (see Supplementary Figure 22). According to the Q-test, there was no significant amount of heterogeneity in the true outcomes ($Q(4) = 4.77$, $p = 0.31$, $\hat{\tau}^2 = 0.00$, $I^2 = 16.17\%$), thus we did not test for sources of heterogeneity. An examination of the studentized residuals revealed that none of the studies had a value larger than ± 2.58 and hence there was no indication of outliers in the context of this model. According to the Cook's distances, none of the studies could be considered to be overly influential.

For peer review only - http://bmjopen.bmj.com/site/about/guidelines.xhtml

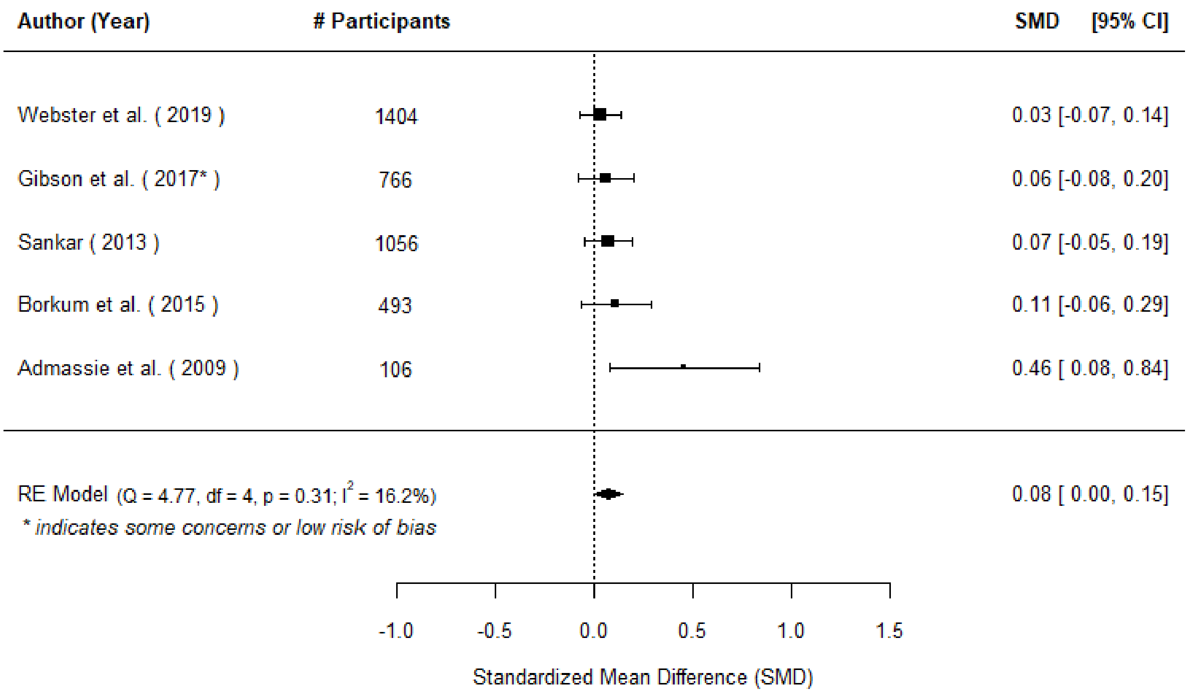

Supplementary Figure 22. Forest plot showing the observed outcomes and the estimate of the random-effects model for the impact of community engagement interventions on OPV1 vaccination.

This group of studies was not sufficiently powered for a robustness check using robust variance analysis (*df* = 3.02).

OPV2

We included a total of $k = 5$ studies in the analysis. The estimated average outcome based on the random-effects model was $\hat{\mu} = 0.24$ ([95% CI: 0.07 to 0.40], $z = 2.84$, $p < 0.01$), indicating a small but significant benefit to the treated group compared to the control group (see Supplementary Figure CEOPV2). According to the Q-test, the true outcomes appear to be heterogeneous ($Q(4) = 22.32$, $p < 0.01$, $\hat{\tau}^2 = 0.03$, $I^2 = 82.08\%$). An examination of the studentized residuals revealed that none of the studies had a value larger than ± 2.58 and hence there was no indication of outliers in the context of this model. According to the Cook's distances, none of the studies could be considered to be overly influential. With only one study that was not assessed as high risk of bias, we could not conduct a sensitivity analysis by study quality.

We tested for potential sources of heterogeneity and found several significant moderators in the context of this model (see SupplementaryTable 3). Exposure to intervention was significant such that each additional month of exposure increased the average effect by .02 standard deviation units ($\beta = 0.02$ [95% CI: 0.01 to 0.02], $p < .0001$). In other words, longer interventions produced larger effects. Publication year was also significant such that older studies reported larger effects than more recent studies. Specifically, each additional year decreased the average effect by .05 standard deviation units ($\beta = -0.05$ [95% CI: -0.08 to -0.02], $p < 0.001$). Finally, region was a significant predictor such that Sub-Saharan Africa had smaller effects that South Asia by .26 standard deviation units ($\beta = -0.26$ [95% CI: -0.39 to -0.13], $p < 0.001$).

For peer review only - http://bmjopen.bmj.com/site/about/guidelines.xhtml

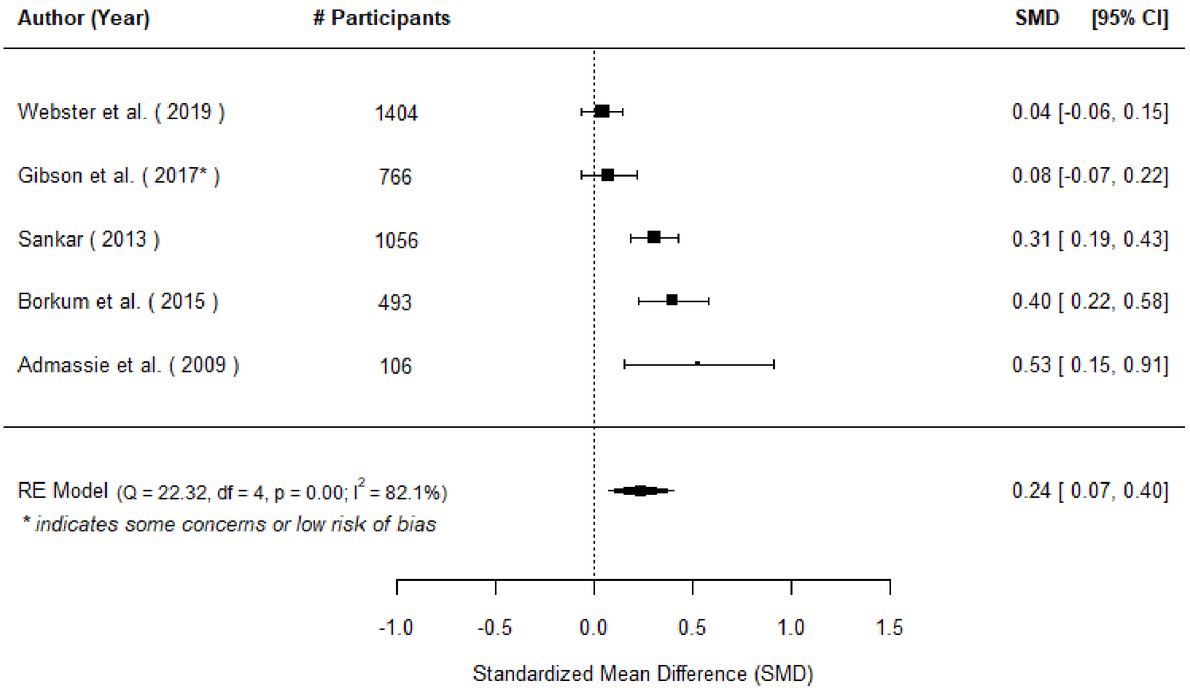

Supplementary Figure 23. Forest plot showing the observed outcomes and the estimate of the random-effects model for the impact of community engagement interventions on OPV2 vaccination.

This group of studies was not sufficiently powered for a robustness check using robust variance analysis (*df* = 3.75).

OPV3

We included total of $k = 9$ studies in the analysis. The estimated average outcome based on the random-effects model was $\hat{\mu} = 0.24$ ([95% CI: 0.09 to 0.40], $z = 3.06$, $p = 0.002$), indicating a moderate and significant benefit to the intervention group compared to the control group (see Supplementary Figure 24). According to the Q-test, the true outcomes appear to be heterogeneous ($Q(8) = 222.99$, $p < 0.001$, $\hat{\tau}^2 = 0.05$, $I^2 = 96.41\%$). An examination of the studentized residuals revealed that one study (Sankar 2013) had a value larger than ± 2.77 and may be a potential outlier in the context of this model. According to the Cook's distances, one study (Sankar 2013) could be considered to be overly influential. Sensitivity analyses leaving each study out indicated that removing Sankar (2013) would substantially reduce the overall average effect ($\hat{\mu}$ = 0.08 [95% CI: 0.01 to 0.15]), but the effect would still be positive and significant (z = 2.39, p = .02). When low quality studies were removed, the resulting effect increased, but was no longer significant ($\hat{\mu} = 0.32$ [95% CI: -0.15 to 0.79], $z = 1.32$, $p = 0.19$). With only two studies of high or moderate quality, this result should be interpreted with caution. There were no significant sources of heterogeneity (see Supplementary Table 3).

For peer review only - http://bmjopen.bmj.com/site/about/guidelines.xhtml

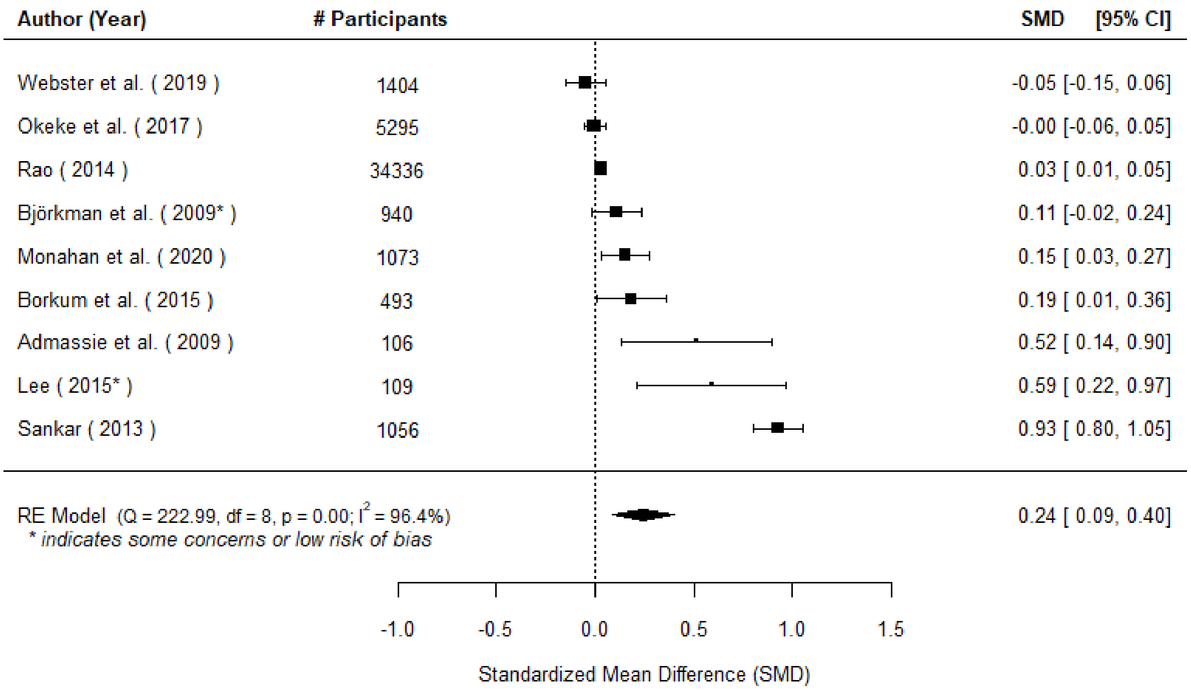

Supplementary Figure 24. Forest plot showing the observed outcomes and the estimate of the random-effects model for the impact of community engagement interventions on OPV3 vaccination.

As a robustness check, we used robust variance analysis and included all dependent effects in the analysis, totaling 10 effects from the same 9 studies (df = 6.74). The overall average effect was identical, but no longer significant ($\hat{\mu} = 0.24$ [95% CI: -0.02 to 0.51], p = .06). Sensitivity analyses show the effect to be sensitive to all values of Rho.

Dropouts

We included a total of $k = 5$ studies in the analysis. The estimated average outcome based on the random-effects model was $\hat{\mu} = 0.03$ ([95% CI: -0.11 to 0.16], $z = 0.36$, $p = 0.72$), indicating no significant difference between the intervention group and the control group (see Supplementary Figure 25). According to the Q-test, the true outcomes appear to be heterogeneous ($Q(4) = 44.86$, $p < 0.001$, $\hat{\tau}^2 = 0.02$, $I^2 = 91.08\%$). An examination of the studentized residuals revealed that one study (Webster et al. 2019) had a value larger than ± 2.58 and may be a potential outlier in the context of this model. Indeed, sensitivity analyses indicated that removing Webster and colleagues (2019) would increase the average effect to $\hat{\mu} = 0.09$ (95% CI: -0.01 to 0.19), but the average outcome still did not differ significantly from zero ($z = 1.70 p = 0.09$). According to the Cook's distances, none of the studies could be considered to be overly influential. With only one study assessed as having a low risk of bias (Banerjee et al. 2020) and the remaining studies at high risk of bias, we were unable to conduct sensitivity analysis by study quality. Only publication year was a significant predictor such that more recently published studies had higher average effects than older studies. Specifically, each additional year increased the average effect by .33 standard deviation units ($\beta = 0.33$ [95% CI: 0.10 to 0.57], $p = 0.01$).

For peer review only - http://bmjopen.bmj.com/site/about/guidelines.xhtml

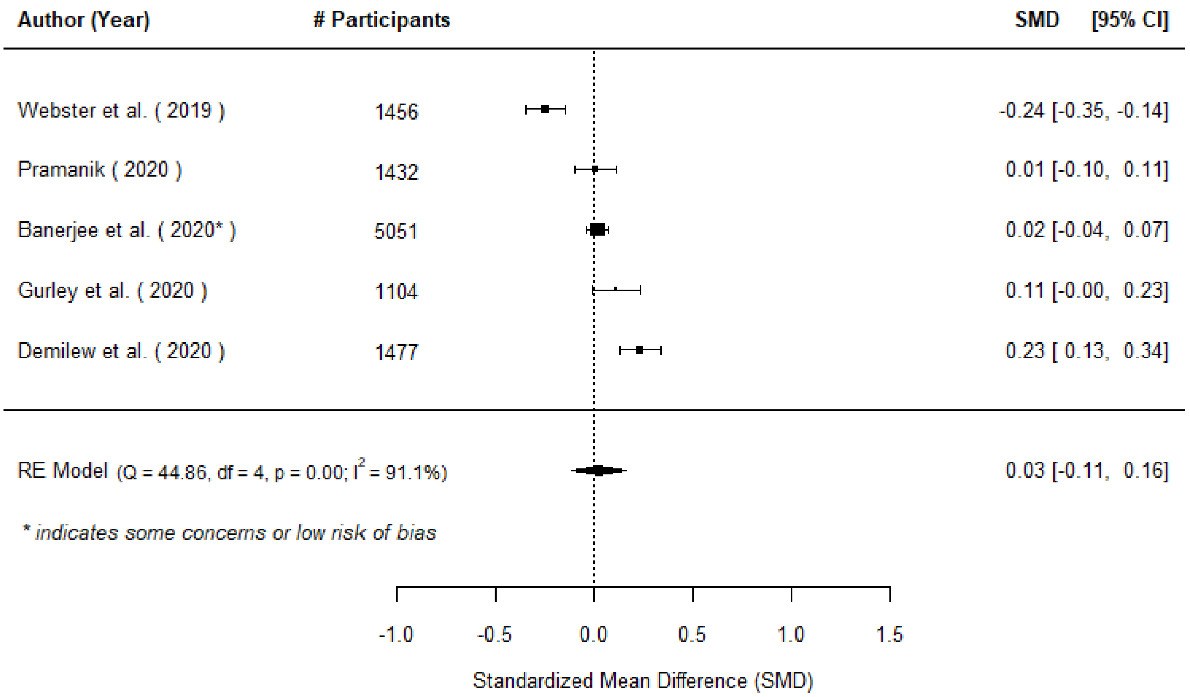

Supplementary Figure 25. Forest plot showing the observed outcomes and the estimate of the random-effects model for the impact of community engagement interventions on childhood morbidity.

This group of studies was not sufficiently powered for a robustness check using robust variance analysis (*df* = 3.98).

Morbidity

We used reports of diarrhea (most typically in the past two weeks) as a proxy for childhood morbidity. In all cases, effects were reverse coded such that positive effects always indicate a benefit to the treated group. Thus, a positive effect here would be interpreted as a reduction in diarrhea among treated participants compared to control participants. A total of $k = 10$ studies were included in the analysis. The estimated average outcome was $\hat{\mu} = 0.01$ ([95% CI: -0.06 to 0.08], $z = 0.34$, $p = 0.73$), indicating no significant difference between intervention group and control group (Supplementary Figure 26). According to the Q-test, the true outcomes appear to be heterogeneous ($Q(9) = 52.37$, $p < 0.01$, $\hat{\tau}^2 = 0.01$, $I^2 = 82.82\%$). When high risk of bias studies were removed, only two studies remained. The resulting estimated average outcome was identical ($\hat{\mu} = 0.01$ [95% CI: -0.21 to 0.23]), and was still non-significant ($z = 0.09$, $p = 0.93$).

An examination of the studentized residuals revealed that one study (Assegaai et al. 2018) had a value larger than ± 2.81 and may be a potential outlier in the context of this model. According to the Cook's distances, Assegaai and colleagues (2018) could also be considered to be overly influential. Indeed, sensitivity analyses leaving each study out indicated that removing Assegaai and colleagues (2018) would reduce the overall average effect ($\hat{\mu}$ = -0.02 (95% CI: -0.08 to 0.05), making the effect negative but still non-significant (z = -0.47, *p* = .64). A funnel plot of the estimates is shown in Supplementary Figure 26b. Neither the rank correlation nor the regression test indicated any funnel plot asymmetry ($p = 0.60$ and $p = 0.49$, respectively). We tested for sources of heterogeneity, but there were no significant moderators (see Supplementary Table 3)

For peer review only - http://bmjopen.bmj.com/site/about/guidelines.xhtml

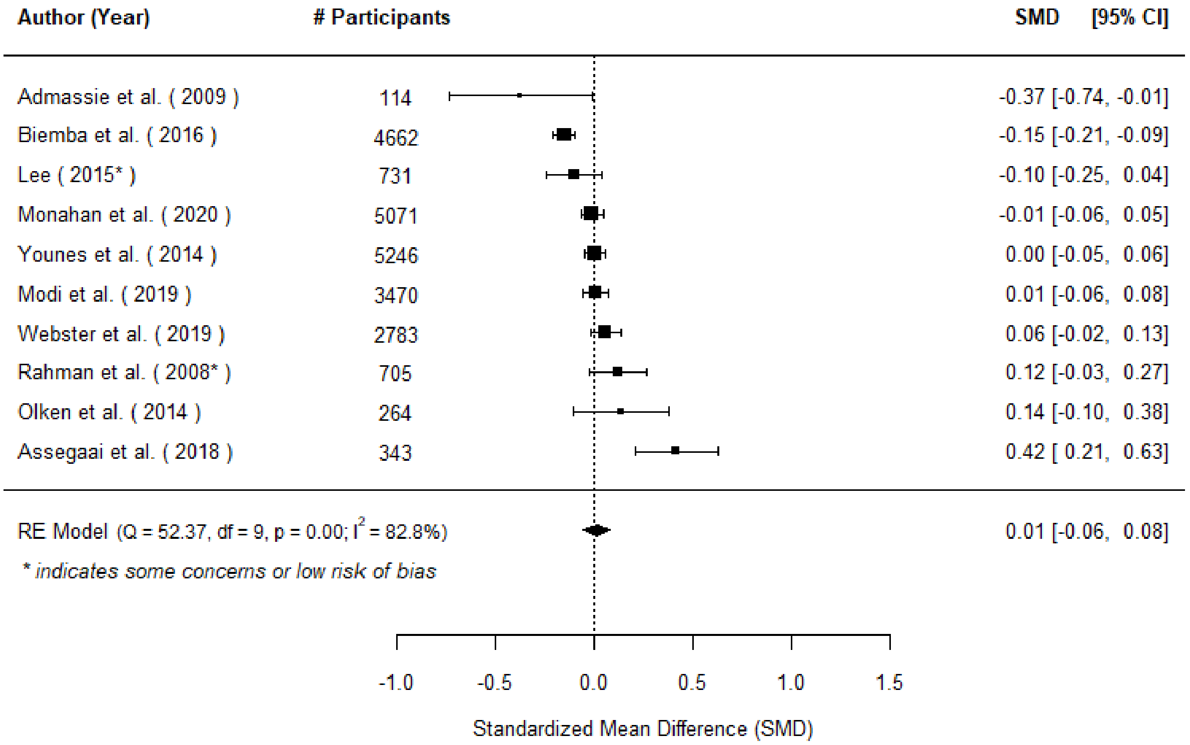

Supplementary Figure 26. Forest plot showing the observed outcomes and the estimate of the random-effects model for the impact of community engagement interventions on childhood morbidity.

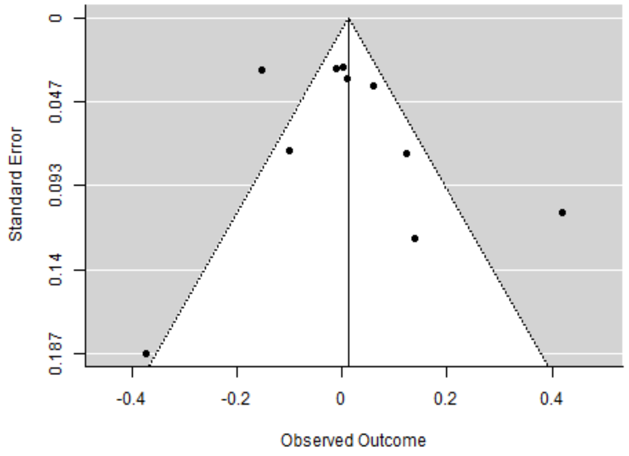

Supplementary Figure 26b. Funnel plot for studies examining the effect of community engagement interventions on childhood morbidity

As a robustness check, we used robust variance analysis and included all dependent effects in the analysis, totaling 26 effects from the same 10 studies (*df* = 7.69). The overall average effect was identical and still non-significant ($\hat{\mu}$ = 0.01 [95% CI: −0.09 to 0.10], *p* = .86). Sensitivity analyses show the effect to be sensitive to all values of Rho.

For peer review only - http://bmjopen.bmj.com/site/about/guidelines.xhtml

Mortality

We included total of $k = 6$ studies in the analysis. The estimated average outcome based on the random-effects model was $\hat{\mu} = -0.04$ (95% CI: -0.09 to 0.01; *see Supplementary Figure C27*). Therefore, the average outcome did not differ significantly from zero ($z = -1.66$, $p = 0.10$), indicating no difference in mortality between the treatment and control groups. According to the Q-test, the true outcomes appear to be heterogeneous ($Q(5) = 19.28$, $p < 0.01$, $\hat{\tau}^2 = 0.00$, $I^2 = 74.07\%$). An examination of the studentized residuals revealed that none of the studies had a value larger than ± 2.64 and hence there was no indication of outliers in the context of this model. According to the Cook's distances, none of the studies could be considered to be overly influential. When high risk of bias studies were removed, only two studies remained. The resulting estimated average outcome was identical ($\hat{\mu} = -0.04$ [95% CI: -0.12 to 0.04]), and was still non-significant ($z = -1.07$, $p = 0.29$). We tested for sources of heterogeneity, and only publication year was significant such that each additional year increased the effect by .01 standard deviation units ($\hat{B} = 0.01, p < 0.001$ [95% CI: 0.01 to 0.02]). There were no other significant moderators (see Supplementary Table 3).

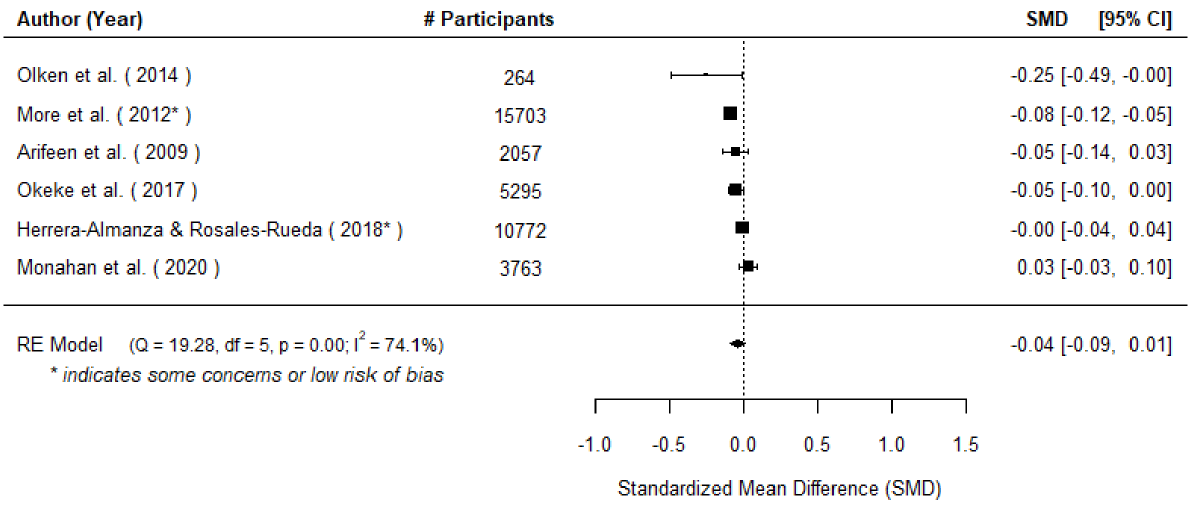

Supplementary Figure 27. Forest plot showing the observed outcomes and the estimate of the random-effects model for the impact of community engagement interventions on childhood morbidity.

As a robustness check, we used robust variance analysis and included all dependent effects in the analysis, totaling 25 effects from the same 6 studies (*df* = 4.07). The overall average effect was identical and still non-significant ($\hat{\mu} = -0.04$ [95% CI: -0.09 to 0.01], *p* = .12). Sensitivity analyses show the effect to be sensitive to all values of Rho.

Immunisation knowledge

We included a total of $k = 9$ studies in the analysis. The estimated average outcome based on the random-effects model was $\hat{\mu} = 0.19$ (95% CI: 0.07 to 0.31). Therefore, the average outcome differed significantly from zero ($z = 3.02$, $p < 0.01$), indicating a significant benefit to the treatment group compared to the control group (see Supplementary Figure 28). According to the Q-test, the true outcomes appear to be heterogeneous ($Q(8) = 62.87$, $p < 0.01$, $\hat{\tau}^2 = 0.03$, $I^2 = 87.28\%$). An examination of the studentized residuals revealed that one study (Banwat et al. 2015) had a value larger than ± 2.77 and may be a potential outlier in the context of this model. Indeed, sensitivity analyses leaving each study out indicated that removing Banwat and colleagues (2015) would reduce the overall average effect ($\hat{\mu}$ = 0.11 (95% CI: -0.02 to 0.20), but the effect would still be positive and significant (z = 2.33, *p* = .02). According to the Cook's distances, none of the studies could be considered to be overly influential.

For peer review only - http://bmjopen.bmj.com/site/about/guidelines.xhtml

We examined potential sources of heterogeneity, and study design was a significant predictor of immunisation knowledge such that studies using quasi-experimental designs had larger average effects than RCT designs by .44 standard deviation units ($\hat{\beta} = 0.44$ [95% CI: 0.23 to 0.64], $p < 0.001$). Region was also a significant predictor, but region was perfectly confounded with study design (e.g. all studies from South Asia were RCTs, while all studies from Sub-Saharan Africa were all quasi-experimental designs), so the effects were larger in Sub-Saharan Africa than from South Asia by the same .44 standard deviation units ($\beta = 0.44$ [95% CI: 0.23 to 0.64], $p < 0.001$). No other moderators were significant (see Supplementary Table 3)

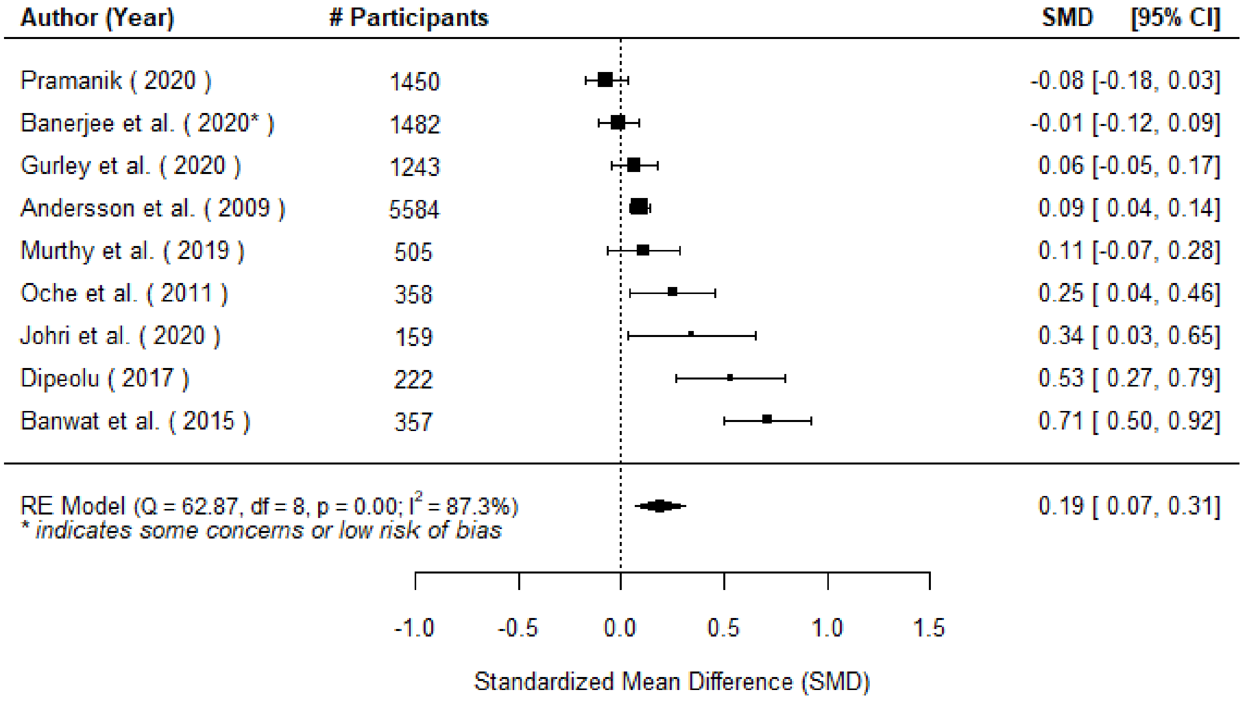

Supplementary Figure 28. Forest plot showing the observed outcomes and the estimate of the random-effects model for the impact of community engagement interventions on childhood morbidity.

As a robustness check, we used robust variance analysis and included all dependent effects in the analysis, totaling 13 effects from the same 9 studies ($df = 7.69$). The overall average effect slightly smaller and become non-significant ($\hat{\mu} = 0.17$ [95% CI: -0.02 to 0.37], $p = .07$). Sensitivity analyses show the effect to be sensitive to all values of Rho.

Immunisation attitudes

We included a total of $k = 6$ studies in the analysis. The estimated average outcome was $\hat{\mu} = 0.14$ ([95% CI: -0.03 to 0.31], $z = 1.60$, $p = 0.11$), indicating no significant different between the intervention and control groups (see Supplementary Figure 29). According to the Q-test, the true outcomes appear to be heterogeneous ($Q(5) = 50.43$, $p < 0.01$, $\hat{\tau}^2 = 0.04$, $I^2 = 90.09\%$). An examination of the studentized residuals revealed that one study (Banwat et al. 2015) had a value larger than ± 2.64 and may be a potential outlier in the context of this model. Indeed, sensitivity analyses leaving each study out indicated that removing Banwat and colleagues (2015) would reduce the overall average effect ($\hat{\mu} = 0.04$ (95% CI: -0.05 to 0.14), but the effect would still be positive and non-significant ($z = 0.92$, $p = .36$). According to the Cook's distances, none of the studies could be considered to be overly influential. With only one study not assessed as high risk of bias, we were unable to conduct a sensitivity analysis by study quality. We tested for sources of heterogeneity, but of the moderators we were able to test, none were significant (see Supplementary Table 3)

For peer review only - http://bmjopen.bmj.com/site/about/guidelines.xhtml

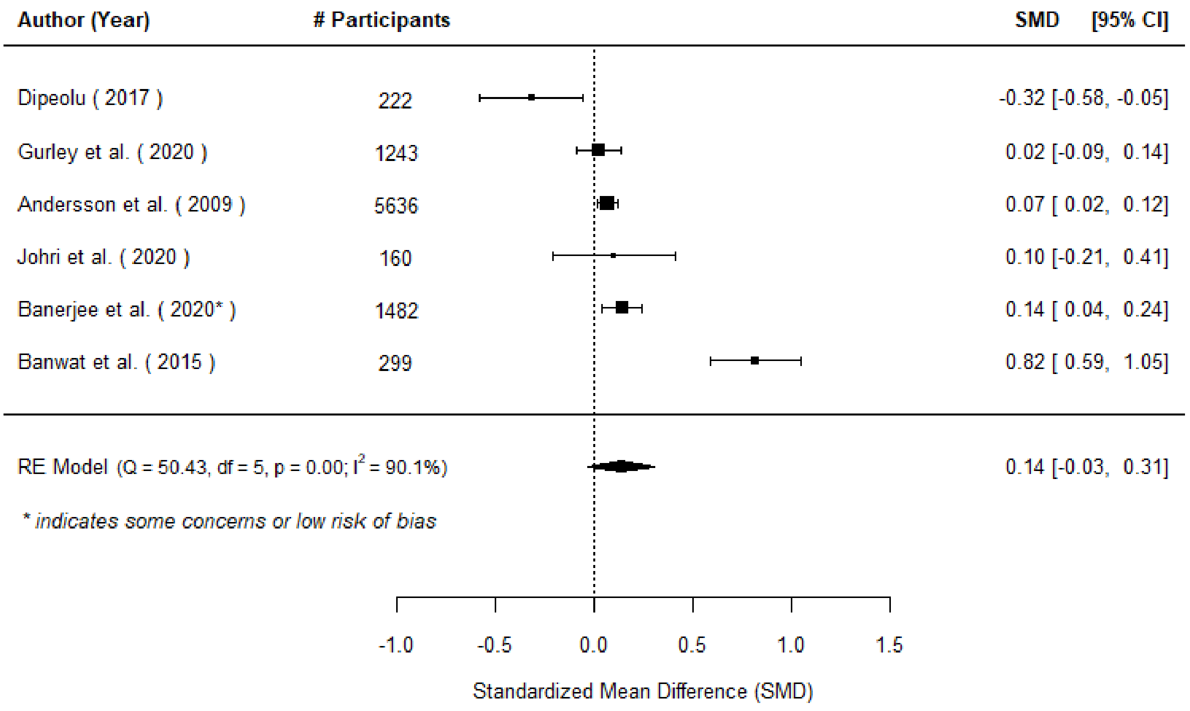

Supplementary Figure 29. Funnel plot for studies examining the effect of community engagement interventions on immunisation attitudes

There were no dependent effects, so we did not complete a robustness check using RVE for this analysis.

Vaccination card availability

We included total of $k = 4$ studies in the analysis. The estimated average outcome was $\hat{\mu} = -0.01$ (95% CI: -0.05 to 0.02). The average outcome did not differ significantly from zero ($z = -0.84$, $p = 0.40$), indicating no difference between the intervention group and the control group on vaccination card availability (see Supplementary Figure 30). According to the Q-test, there was no significant amount of heterogeneity in the true outcomes ($Q(3) = 2.27$, $p = 0.52$, $\hat{\tau}^2 = 0.00$, $I^2 = 0.00\%$). One study (Herrera-Almanza & Rosales-Rueda 2018) had a relatively large weight compared to the rest of the studies (i.e., weight $\geq 3/k$, so a weight at least 3 times as large as having equal weights across studies). However, this was also the only study assessed as having a low risk of bias. The three other studies in the analysis were assessed as high risk of bias. An examination of the studentized residuals revealed that none of the studies had a value larger than ± 2.50 and hence there was no indication of outliers in the context of this model. According to the Cook's distances, none of the studies could be considered to be overly influential. With no heterogeneity present, we did not test for moderation.

For peer review only - http://bmjopen.bmj.com/site/about/guidelines.xhtml

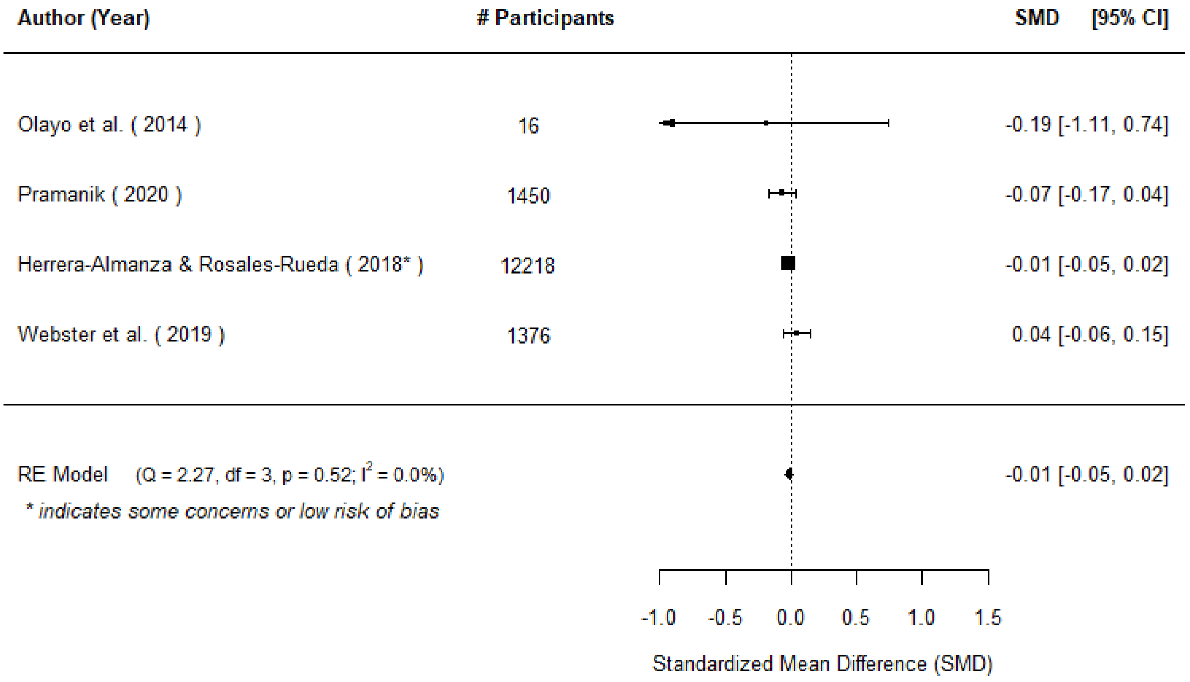

Supplementary Figure 30. Forest plot showing the observed outcomes and the estimate of the random-effects model for the impact of community engagement interventions on vaccination card availability.

There were no dependent effects, so we did not complete a robustness check using RVE for this analysis.

Experience and satisfaction with health services

Only two studies using community engagement interventions reported on experience and satisfaction with health services. The estimated average outcome based on the random-effects model was $\hat{\mu} = 0.04$ (95% CI: -0.15 to 0.23). Therefore, the average outcome did not differ significantly from zero ($z = 0.38$, $p = 0.70$, see Supplementary Figure 31). Given the small number of studies, this result should be interpreted with caution. According to the Q-test, the true outcomes appear to be homogeneous ($Q(1) = 1.93$, $p = 0.16$, $\hat{\tau}^2 = 0.01$, $I^2 = 48.22\%$; With only two studies and no heterogeneity, moderator analyses were not appropriate and tests of publication bias are not valid, and we were not powered for a robustness check using RVE.

For peer review only - http://bmjopen.bmj.com/site/about/guidelines.xhtml

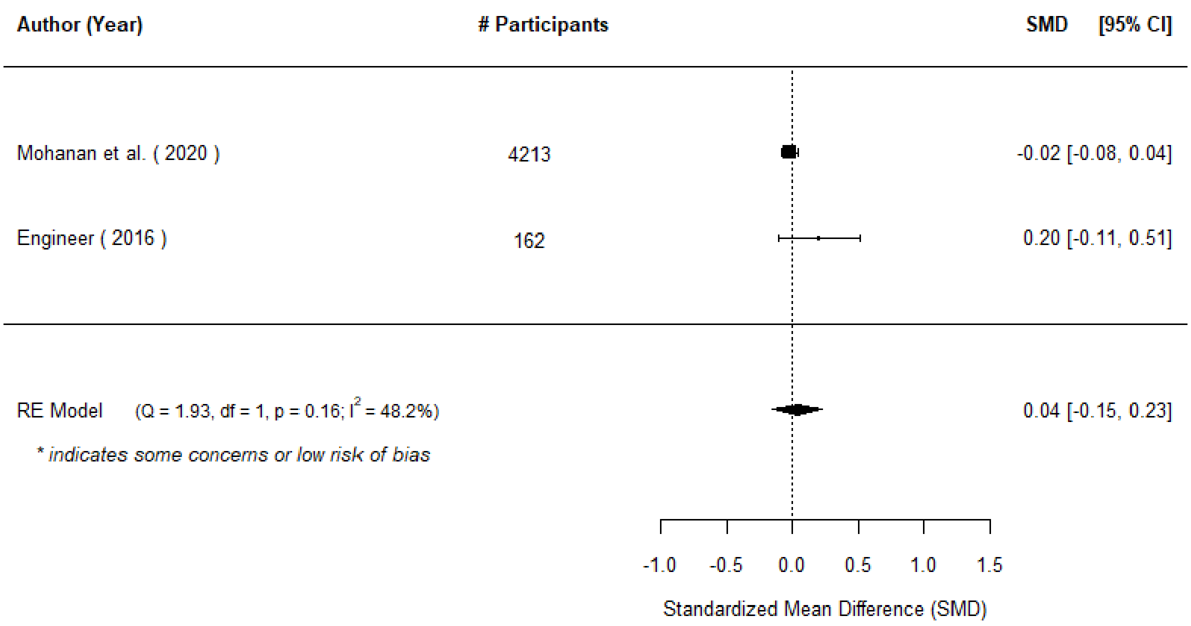

Supplementary Figure 31. Forest plot showing the observed outcomes and the estimate of the random-effects model for community engagement interventions on experience and satisfaction with health services.

Formal health worker's motivation, capacity and performance

Only two studies using community engagement interventions reported on formal health worker's motivation, capacity and performance. The estimated average outcome based on the random-effects model was $\hat{\mu} = 0.11$ (95% CI: -0.07 to 0.29). Therefore, the average outcome did not differ significantly from zero ($z = 0.38$, $p = 0.70$, see Supplementary Figure 32). Given the small number of studies, this result should be interpreted with caution. According to the Q-test, the true outcomes appear to be homogeneous ($Q(1) = 0.04$, $p = 0.84$, $\hat{\tau}^2 = 0.00$, $I^2 = 0.00\%$; With only two studies and no heterogeneity, moderator analyses were not appropriate and tests of publication bias are not valid, and we were not powered for a robustness check using RVE.

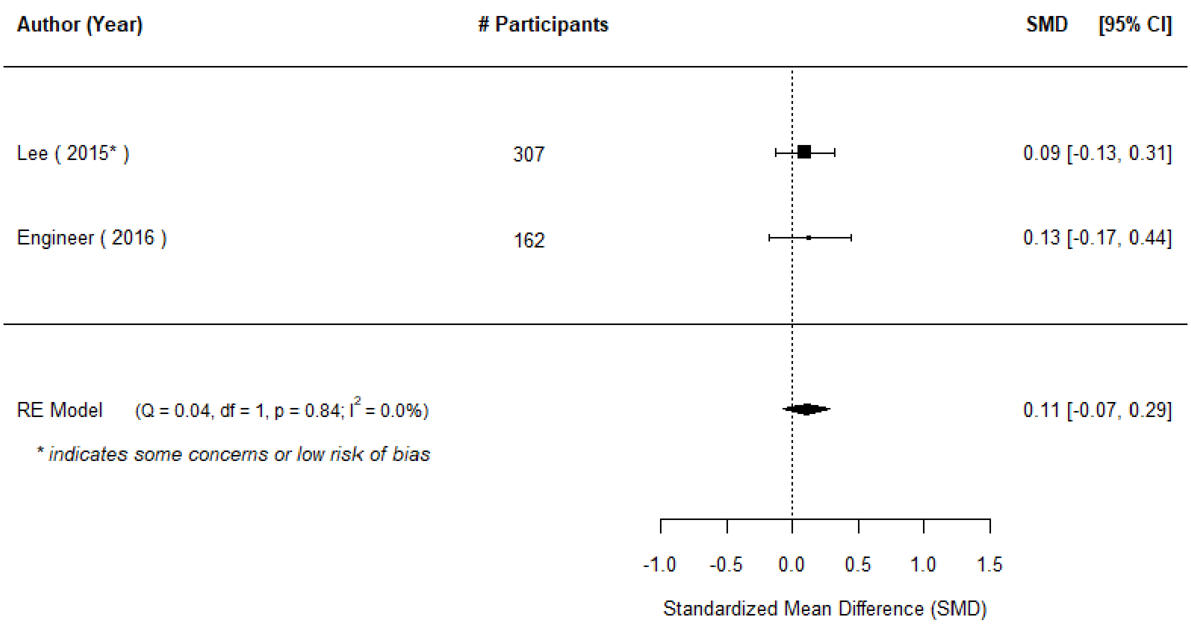

For peer review only - http://bmjopen.bmj.com/site/about/guidelines.xhtml

Supplementary Figure 32. Forest plot showing the observed outcomes and the estimate of the random-effects model for community engagement interventions on formal health worker's motivation, capacity and performance.

For peer review only

For peer review only - http://bmjopen.bmj.com/site/about/guidelines.xhtml

Supplementary Table 2: Summary of quantitative result

	TOTAL SAMPLE		ENGAGEMENT AS THE INTERVENTION	ENGAGEMENT IN THE DESIGN	ENGAGEMENT IN IMPLEMENTATION AUTONOMY	MULTIPLE ENGAGEMENT TYPES
	RVE (dependent effects)	RE (independent effects)	g, [95% CI], I^2, (k)	g, [95% CI], I^2, (k)	g, [95% CI], I^2, (k)	g, [95% CI], I^2, (k)
	g, [95% CI], I^2, (k; # of effects)	g, [95% CI], I^2, (k)				
**FULL IMMUNISATION**	**0.11**, [0.04, 0.18],** 87.27, (28; 53)	**0.14**, [0.06, 0.23],** 94.46, (28)	**0.08**, [0.03, 0.13],** 70.00, (12)	**010*, [0.02, 0.19],** 23.83, (5)	0.23, [-0.001, 0.47], 73.07, (2)	0.22, [-0.12, 0.56], 97.94, (9)
**PARTIAL IMMUNISATION**	**0.21*, [0.03, 0.38],** 95.90, (9; 13)	**0.23**, [0.09, 0.37],** 96.35, (9)	0.31, [-0.25, 0.87], 98.96, (2)	**0.14*, [0.01, 0.27],** 0.00, (2)	N/A	0.28, [-0.01, 0.56], 97.05, (4)
**MEASLES**	**0.06*, [0.01, 0.11],** 72.46, (20; 34)	**0.07**, [0.03, 0.11],** 73.64, (20)	**0.10***, [0.05, 0.15],** 60.29, (10)	**0.11*, [0.02, 0.21],** 0.00, (2)	0.03, [-0.09, 0.15], 54.84, (2)	0.03, [-0.10, 0.16], 86.76, (6)
**BCG**	0.04, [-0.02, 0.10], 79.17, (12; 16)	**0.06*, [0.01, 0.11],** 86.94, (12)	**0.02***, [0.01, 0.03],** 00.00, (4)	0.02, [-0.09, 0.13], 0.00, (2)	0.03, [-0.05, 0.11], 0.00, (2)	0.22, [-0.07, 0.52], 96.02, (4)
**DPT1**	0.01, [-0.06, 0.09], 62.55, (8; 21)	0.04, [-0.04, 0.11], 76.81, (8)	0.10, [-0.03, 0.22] 90.21, (3)	0.03, [-0.08.0.14], 0.00, (2)	N/A	**-0.17**, [-0.29, -0.05],** 0.00, (2)
**DPT2**	Not powered	**0.07*, [0.01, 0.12],** 0.00, (5)	N/A	0.05, [-0.06, 0.16], 0.00, (2)	N/A	N/A
**DPT3**	**0.10**, [0.05, 0.15],** 76.42, (22; 36)	**0.10***, [0.06, 0.14],** 76.78, (22)	**0.09**, [0.03, 0.15],** 73.17, (6)	0.04, [-0.01, 0.08], 0.00, (6)	0.11, [-0.05, 0.28], 80.12, (3)	**0.20**, [0.06, 0.34],** 87.93, (7)
**OPV0**	Not powered	0.10, [-0.06, 0.26], 91.26, (5)	N/A	0.01, [-0.13, 0.14], 0.00, (2)	N/A	N/A
**OPV1**	Not powered	**0.08*, [0.004, 0.15],** 16.17, (5)	N/A	0.08, [-0.03, 0.19], 0.00, (2)	N/A	0.22, [-0.15, 0.59], 72.32, (2)
**OPV2**	Not powered	**0.24**, [0.07, 0.40],** 82.08, (5)	N/A	0.23, [-0.09, 0.55], 87.44, (2)	N/A	**0.34***, [0.19, 0.50],** 15.51, (2)

For peer review only - http://bmjopen.bmj.com/site/about/guidelines.xhtml

OPV3	0.24, [-0.02, 0.51], 96.42, (9; 10)	0.24**, [0.09, 0.40], 96.41, (9)	0.16, [-0.02, 0.34], 83.83, (3)	N/A	0.03, [-0.12, 0.18], 70.38, (2)	0.48, [-0.24, 1.20], 98.92, (3)
TIMELINESS (ALL)	0.11**, [0.06, 0.16], 39.92, (11; 54)	----	----	----	----	----
TIMELINESS (DTP3)	No dependent effects	0.09**, [0.03, 0.14], 0.00, (7)	N/A	0.12**, [0.03, 0.21], 0.00, (4)	0.04, [-0.06, 0.13], 0.00, (2)	N/A
TIMELINESS (MEASLES)	No dependent effects	0.23***, [0.14, 0.32], 0.00, (2)	N/A	N/A	N/A	N/A
TIMELINESS (COMPLETE IMMUNISATIONS)	No dependent effects	0.15***, [0.07, 0.24], 9.66 (5)	N/A	0.15*, [0.004, 0.29], 0.00, (2)	0.38, [-0.28, 1.03], 72.02, (2)	N/A
DROPOUTS	Not powered	0.03, [-0.11, 0.16], 91.08, (5)	0.02, [-0.03, 0.06], 0.00, (2)	N/A	N/A	0.18**, [0.06, 0.30], 57.38, (2)
MORBIDITY	0.01, [-0.09, 0.10], 83.32, (10; 26)	0.01, [-0.06, 0.08], 82.82, (10)	-0.004, [-0.11, 0.10], 88.86, (5)	0.05, [-0.06, 0.15], 45.71, (2)	N/A	-0.10, [-0.60, 0.40], 80.88, (2)
MORTALITY	-0.04, [-0.09, 0.01], 69.91, (6; 25)	-0.04, [-0.09, 0.01], 74.07, (6)	-0.04, [-0.11, 0.04], 80.68, (3)	N/A	N/A	-0.04, [-0.10, 0.03], 62.75, (3)
IMMUNISATION KNOWLEDGE	0.17, [-0.02, 0.37], 87.30, (9; 13)	0.19*, [0.07, 0.31], 87.28, (9)	0.20, [-0.08, 0.49], 93.80, (4)	0.31*, [0.04, 0.58], 72.34, (3)	N/A	0.09***, [0.04, 0.13], 0.00, (2)
IMMUNISATION ATTITUDES	No dependent effects	0.14, [-0.03, 0.31], 90.09, (6)	0.47, [-0.19, 1.13], 96.36, (2)	-0.11, [-0.53, 0.30], 75.61, (2)	N/A	0.06*, [0.01, 0.11], 0.00, (2)
VACCINATION CARD AVAILABILITY/RETENTION	No dependent effects	-0.01, [-0.05, 0.02], 00.00, (4)	N/A	N/A	N/A	-0.01, [-0.05, 0.02], 00.00, (2)
EXPERIENCE AND SATISFACTION WITH HEALTH SERVICES	Not powered	0.04, [-0.015, .023], 48.22. (2)	N/A	N/A	N/A	N/A
FORMAL HEALTH WORKER CAPACITY AND PERFORMANCE	Not powered	0.11, [-0.07, 0.29], 0.00, (2)	N/A	N/A	N/A	N/A

Supplementary Table 3: Summary of all moderator analyses

For peer review only

For peer review only - http://bmjopen.bmj.com/site/about/guidelines.xhtml

	EXP INTMO	EVAL INMO	DESIGN	YEAR	REGION	CARD	POST INT V CFB	GOV IMPLEMENT	CADRES	VACCINE HESITENCY	BASELINE COVERAGE
	**B (SE)**	**B (SE)**	**B (SE)**	**B (SE)**	**B (SE)**	**B (SE)**	**B (SE)**	**B (SE)**	**B (SE)**	**B (SE)**	**B (SE)**
**ALL EFFECTS RVE (K = 56)**	-0.001 (0.003)	0.002 (0.006)	-0.10 (0.10)	-0.019* (0.007)	NP	0.06 (0.05)	-0.08 (0.05)	-0.08 (0.04)	-0.02 (0.05)	-0.04 (0.05)	-0.31 (0.15)
**FULL IMMUNISATION RVE (K = 28)**	0.0001 (0.002)	NP	-0.03 (0.09)	NP	NS (ALL COMPARISONS)	0.04 (0.11)	-0.16 (0.12)	-0.06 (0.10)	0.02 (0.09)	0.10 (0.19)	-0.13 (0.15)
**FULL IMMUNISATION IND RE (K = 28)**	-0.0002 (0.004)	-0.01 (0.02)	-0.05 (0.11)	-0.01 (0.01)	NS (ALL COMPARISONS)	0.04 (0.12)	-0.13 (0.10)	0.06 (0.12)	-0.06 (0.12)	0.17 (0.11)	-0.33 (0.25)
**FULL IMMUNISATION IND EAI (K = 12)**	-0.003* (0.002)	0.01 (0.01)	-0.06 (0.06)	0.01 (0.01)	0.02 (0.07)	-0.01 (0.06)	-0.04 (0.08)	0.08 (0.06)	-0.09 (0.06)	0.08 (0.07)	-0.06 (0.11)
**FULL IMMUNISATION IND EID (K = 5)**	N/A[C]	N/A[C]	N/A[C]	N/A[C]	N/A[C]	N/A[C]	N/A[C]	N/A[C]	N/A[C]	N/A[C]	N/A[C]
**FULL IMMUNISATION IND EII (K = 2)**	NP	NP	NP	NP	NP	NP	NP	NP	NP	NP	NP
**FULL IMMUNISATION IND MET (K = 9)**	0.005 (0.01)	-0.06 (0.06)	-0.12 (0.38)	-0.06 (0.03)	NS (ALL COMPARISONS)	N/A[B]	-0.49 (0.27)	0.04 (0.35)	N/A[B]	0.33 (0.31)	-0.66 (0.68)
**PARTIAL IMMUNISATION RVE (K = 9)**	NP	NP	NP	NP	NP	NP	0.10 (0.18)	NP	NP	0.27 (0.12)	N/A[E]
**PARTIAL IMMUNISATION IND RE (K = 9)**	-0.003 (0.006)	-0.01 (0.01)	-0.10 (0.19)	-0.03 (0.03)	-0.03 (0.19)	0.06 (0.21)	0.09 (0.18)	-0.30 (0.20)	-0.30 (0.19)	0.18 (0.19)	N/A[E]
PARTIAL IMM**UNISATION** IND EAI (K = 2)	NP	NP	NP	NP	NP	NP	NP	NP	NP	NP	N/A[E]
PARTIAL IMM**UNISATION** IND EID (K = 2)	NP	NP	NP	NP	NP	NP	NP	NP	NP	NP	N/A[E]
PARTIAL IMM**UNISATION** IND EII (K = 1)	NP	NP	NP	NP	NP	NP	NP	NP	NP	NP	N/A[E]
PARTIAL IMM**UNISATION** IND MET (K = 4)	0.01 (0.02)	-0.01 (0.01)	-0.10 (0.35)	-0.02 0.06)	0.05 (0.36)	N/A[B]	0.35 (0.33)	N/A[A]	N/A[B]	0.08 (0.35)	N/A[E]
**MEASLES RVE (K = 20)**	0.0001 (0.001)	NP	-0.03 (0.05)	-0.001 (0.004)	NP	0.01 (0.04)	-0.04 (0.03)	0.03 (0.03)	0.04 (0.04)	NP	N/A[E]

For peer review only - http://bmjopen.bmj.com/site/about/guidelines.xhtml

**MEASLES IND RE (K = 20)**	**0.000 (0.001)**	**-0.003 (0.003)**	**-0.05 (0.06)**	**-0.003 (0.007)**	**NS (ALL COMPARISONS)**	**0.02 (0.07)**	**-0.04 (0.06)**	**0.06 (0.06)**	**0.05 (0.06)**	**0.13 (0.09)**	N/A[D]
**MEASLES: IND EAI (K = 10)**	**-0.001 (0.001)**	**-0.004 (0.006)**	**0.07 (0.05)**	**0.004 (0.01)**	**NS (ALL COMPARISONS)**	**0.02 (0.08)**	**0.05 (0.05)**	**-0.003 (0.05)**	**0.03 (0.05)**	**0.10 (0.09)**	N/A[D]
MEASLES: IND EID (K = 2)	NP	NP	NP	NP	NP	NP	NP	NP	NP	NP	N/A[D]
**MEASLES: IND EII (K = 2)**	NP	NP	NP	NP	NP	NP	NP	NP	NP	NP	N/A[D]
**MEASLES: IND MET (K = 6)**	**0.004 (0.01)**	**-0.004 (0.01)**	**-0.15 (0.31)**	**-0.04 (0.03)**	**0.18 (0.25)**	**0.07 (0.33)**	**-0.33 (0.19)**	**0.31 (0.20)**	**0.18 (0.25)**	N/A[B]	N/A[D]
**BCG RVE (K = 12)**	**NP**	**NP**	**-0.10 (0.10)**	**-0.03 (0.02)**	**-0.08 (0.12)**	**NP**	**-0.12 (0.10)**	**-0.10 (0.14)**	**NP**	**0.37*** (0.03)**	N/A[D]
**BCG IND RE (K = 12)**	**0.001 (0.004)**	**-0.01 (0.01)**	**-0.05 (0.11)**	**-0.03 ** (0.01)**	**0.004 (0.11)**	**-0.02 (0.04)**	**-0.12 (0.12)**	**-0.04 (0.13)**	**0.06 (0.13)**	N/A[B]	N/A[D]
BCG: IND EAI (K = 4)	N/A[C]	N/A[C]	N/A[C]	N/A[C]	N/A[C]	N/A[C]	N/A[C]	N/A[C]	N/A[C]	N/A[C]	N/A[D]
BCG: IND EID (K = 2)	NP	NP	NP	NP	NP	NP	NP	NP	NP	NP	N/A[D]
BCG: IND EII (K = 2)	NP	NP	NP	NP	NP	NP	NP	NP	NP	NP	N/A[D]
**BCG: IND MET (K = 4)**	**-0.002 (0.02)**	**N/A[B]**	**N/A[B]**	**-0.08 (0.04)**	**0.03 (0.41)**	**N/A[A]**	**-0.55*** (0.07)**	**0.03 (0.41)**	**0.03 (0.41)**	N/A[B]	N/A[D]
**DPT1: RVE (K = 8)**	NP	NP	**-0.10 (0.10)**	**0.001 (0.01)**	NP	NP	NP	NP	NP	**0.07 (0.03)**	**-0.13 (0.20)**
**DPT1: IND RE (K = 8)**	**-0.005 (0.01)**	N/A[A]	**-0.15* (0.07)**	**-0.004 (0.01)**	**NS (ALL COMPARISONS)**	**-0.08 (0.10)**	**0.02 (0.11)**	**0.17* (0.08)**	N/A[B]	N/A[B]	N/A[E]
DPT1: IND EAI (K = 3)	NP	NP	NP	NP	NP	NP	NP	NP	NP	NP	NP
DPT1: IND EID (K = 2)	NP	NP	NP	NP	NP	NP	NP	NP	NP	NP	NP
DPT1: IND EII (K = 1)	NP	NP	NP	NP	NP	NP	NP	NP	NP	NP	NP
DPT1: IND MET (K = 2)	NP	NP	NP	NP	NP	NP	NP	NP	NP	NP	NP
**DPT2 RVE (K = 5)**	NP	NP	NP	NP	NP	NP	NP	NP	NP	NP	NP
**DPT2 IND RE (K = 5)**	**N/A[C]**	**N/A[C]**	**N/A[C]**	**N/A[C]**	**N/A[C]**	**N/A[C]**	**N/A[C]**	**N/A[C]**	**N/A[C]**	**N/A[C]**	**N/A[C]**
DPT2 IND EAI (K = 0)	NP	NP	NP	NP	NP	NP	NP	NP	NP	NP	NP
DPT2 IND EID (K = 2)	NP	NP	NP	NP	NP	NP	NP	NP	NP	NP	NP
DPT2 IND EII (K = 0)	NP	NP	NP	NP	NP	NP	NP	NP	NP	NP	NP
DPT2 IND MET (K = 3)	NP	NP	NP	NP	NP	NP	NP	NP	NP	NP	NP
**DPT3 RVE (K = 22)**	**0.0003 (0.001)**	NP	**-0.03 (0.05)**	**-0.02 (0.01)**	**-0.01 (0.05)**	NP	**-0.01 (0.05)**	**-0.04 (0.05)**	**NP**	**NP**	**-0.50 (0.21)**
**DPT3 IND RE (K = 22)**	**-0.001 (0.001)**	**-0.002 (0.004)**	**0.05 (0.05)**	**-0.014* (0.006)**	**-0.02 (0.05)**	**0.14 (0.09)**	**-0.01 (0.05)**	**-0.01 (0.05)**	**-0.03 (0.06)**	**0.04 (0.06)**	**-0.40 (0.22)**
**DPT3 IND EAI (K = 6)**	**-0.001 (0.002)**	**0.01 (0.01)**	**-0.05 (0.07)**	**0.01 (0.01)**	N/A[B]	N/A[A]	**-0.08 (0.07)**	**0.05 (0.07)**	**-0.04 (0.07)**	**-0.03 (0.08)**	**-0.40 (0.22)**
DPT3 IND EID (K = 6)	N/A[C]	N/A[C]	N/A[C]	N/A[C]	N/A[C]	N/A[C]	N/A[C]	N/A[C]	N/A[C]	N/A[C]	N/A[C]
DPT3 IND EII (K = 3)	NP	NP	NP	NP	NP	NP	NP	NP	NP	NP	NP

For peer review only - http://bmjopen.bmj.com/site/about/guidelines.xhtml

**DPT3 IND MET (K =7)**	**0.003 (0.006)**	**-0.01** (0.004)**	**0.07 (0.16)**	**-0.03* (0.01)**	**-0.10 (0.15)**	N/A[A]	**-0.25 (0.15)**	**0.05 (0.17)**	**-0.04 (0.17)**	**-0.02 (0.17)**	**-0.56** (0.18)**
OPV0 RVE (K = 5)	NP	NP	NP	NP	NP	NP	NP	NP	NP	NP	NP
**OPV0 IND RE (K = 5)**	**0.02*** (0.004)**	N/A[A]	**0.16 (0.19)**	**-0.07** (0.02)**	**-0.20 (0.18)**	**0.16 (0.19)**	N/A[B]	**-0.21 (0.17)**	N/A[A]	N/A[A]	N/A[D]
OPV0 IND EAI (K = 1)	NP	NP	NP	NP	NP	NP	NP	NP	NP	NP	N/A[D]
OPV0 IND EID (K = 2)	NP	NP	NP	NP	NP	NP	NP	NP	NP	NP	N/A[D]
OPV0 IND EII (K = 1)	NP	NP	NP	NP	NP	NP	NP	NP	NP	NP	N/A[D]
OPV0 IND MET (K = 1)	NP	NP	NP	NP	NP	NP	NP	NP	NP	NP	N/A[D]
OPV1 RVE (K = 5)	NP	NP	NP	NP	NP	NP	NP	NP	NP	NP	N/A[D]
**OPV1 IND RE (K = 5)**	N/A[C]	N/A[C]	N/A[C]	N/A[C]	N/A[C]	N/A[C]	N/A[C]	N/A[C]	N/A[C]	N/A[C]	N/A[D]
OPV01 IND EAI (K = 0)	NP	NP	NP	NP	NP	NP	NP	NP	NP	NP	N/A[D]
OPV1 IND EID (K = 2)	NP	NP	NP	NP	NP	NP	NP	NP	NP	NP	N/A[D]
OPV1 IND EII (K = 1)	NP	NP	NP	NP	NP	NP	NP	NP	NP	NP	N/A[D]
OPV1 IND MET (K = 2)	NP	NP	NP	NP	NP	NP	NP	NP	NP	NP	N/A[D]
OPV2 RVE (K = 5)	NP	NP	NP	NP	NP	NP	NP	NP	NP	NP	N/A[D]
**OPV2 IND RE (K = 5)**	**0.02*** (0.003)**	N/A[A]	**0.22 (0.18)**	**-0.05** (0.02)**	**-0.26*** (0.07)**	**0.27 (0.07)**	N/A[B]	**0.03 (0.25)**	N/A[B]	N/A[A]	N/A[D]
OPV02 IND EAI (K = 0)	NP	NP	NP	NP	NP	NP	NP	NP	NP	NP	N/A[D]
OPV2 IND EID (K = 2)	NP	NP	NP	NP	NP	NP	NP	NP	NP	NP	N/A[D]
OPV2 IND EII (K = 1)	NP	NP	NP	NP	NP	NP	NP	NP	NP	NP	N/A[D]
OPV2 IND MET (K = 2)	NP	NP	NP	NP	NP	NP	NP	NP	NP	NP	N/A[D]
**OPV3 RVE (K = 9)**	**NP**	**NP**	**-0.03 (0.05)**	**NP**	**-0.24 (0.31)**	**NP**	**0.26 (0.30)**	**NP**	**-0.18 (0.33)**	**NP**	N/A[D]
**OPV3 IND RE (K = 9)**	**-0.001 (0.01)**	**-0.01 (0.01)**	**0.17 (0.23)**	**-0.03 (0.03)**	**-0.12 (0.23)**	**0.04 (0.32)**	**0.14 (0.25)**	**-0.34 (0.27)**	**-0.03 (0.27)**	N/A[A]	N/A[D]
OPV3 IND EAI (K = 3)	NP	NP	NP	NP	NP	NP	NP	NP	NP	NP	N/A[D]
OPV3 IND EID (K = 1)	NP	NP	NP	NP	NP	NP	NP	NP	NP	NP	N/A[D]
OPV3 IND EII (K = 2)	NP	NP	NP	NP	NP	NP	NP	NP	NP	NP	N/A[D]
OPV3 IND MET (K = 3)	NP	NP	NP	NP	NP	NP	NP	NP	NP	NP	N/A[D]
**TIMELINESS RVE (K = 11)**	NP	NP	NP	NP	NP	NP	NP	NP	NP	NP	N/A[D]
**DPT3 TIMELINESS RE (K = 7)**	N/A[C]	N/A[C]	N/A[C]	N/A[C]	N/A[C]	N/A[C]	N/A[C]	N/A[C]	N/A[C]	N/A[C]	N/A[C]
DPT3 TIMELINESS EAI (K = 0)	--	--	--	--	--	--	--	--	--	--	--
**DPT3 TIMELINESS EID (K = 4)**	N/A[C]	N/A[C]	N/A[C]	N/A[C]	N/A[C]	N/A[C]	N/A[C]	N/A[C]	N/A[C]	N/A[C]	N/A[C]
DPT3 TIMELINESS EII (K =2)	NP	NP	NP	NP	NP	NP	NP	NP	NP	NP	NP
DPT3 TIMELINESS MET (K = 1)	NP	NP	NP	NP	NP	NP	NP	NP	NP	NP	NP
MEASLES TIMELINESS RE (K = 2)	NP	NP	NP	NP	NP	NP	NP	NP	NP	NP	NP
MEASLES TIMELINESS EAI (K = 0)	--	--	--	--	--	--	--	--	--	--	--
MEASLES TIMELINESS EID (K = 1)	NP	NP	NP	NP	NP	NP	NP	NP	NP	NP	NP
MEASLES TIMELINESS EII (K =0)	--	--	--	--	--	--	--	--	--	--	--
MEASLES TIMELINESS MET (K = 1)	NP	NP	NP	NP	NP	NP	NP	NP	NP	NP	NP
FI TIMELINESS RE (K =5)	N/A[C]	N/A[C]	N/A[C]	N/A[C]	N/A[C]	N/A[C]	N/A[C]	N/A[C]	N/A[C]	N/A[C]	N/A[C]
FI TIMELINESS EAI (K = 1)	NP	NP	NP	NP	NP	NP	NP	NP	NP	NP	NP

For peer review only

FI TIMELINESS EID (K = 2)	NP	NP	NP	NP	NP	NP	NP	NP	NP	NP	NP
FI TIMELINESS EII (K = 2)	NP	NP	NP	NP	NP	NP	NP	NP	NP	NP	NP
FI TIMELINESS MET (K = 0)	--	--	--	--	--	--	--	--	--	--	--
DROPOUT RVE (K = 5)	NP	NP	NP	NP	NP	NP	NP	NP	NP	NP	NP
**DROPOUT RE (K = 5)**	**0.04 (0.04)**	**0.03 (0.03)**	N/A[A]	0.33** (0.12)	**-0.050 (0.183)**	N/A[B]	N/A[A]	**-0.23 (0.13)**	N/A[A]	**0.05 (0.18)**	N/A[D]
DROPOUT EAI (K =2)	NP	NP	NP	NP	NP	NP	NP	NP	NP	NP	NP
DROPOUT EID (K = 0)	--	--	--	--	--	--	--	--	--	--	--
DROPOUT EII (K =1)	NP	NP	NP	NP	NP	NP	NP	NP	NP	NP	NP
DROPOUT MET (K = 2)	NP	NP	NP	NP	NP	NP	NP	NP	NP	NP	NP
**MORBIDITY RVE (K = 10)**	NP	NP	NP	**0.01 (0.01)**	NP	N/A[E]	**0.02 (0.08)**	NP	NP	N/A[A]	N/A[E]
**MORBIDITY RE (K = 10)**	**0.003 (0.005)**	**-0.01 (0.01)**	**-0.04 0.11**	**0.01 (0.01)**	**NS (ALL COMPARI SONS)**	**-0.08 (0.10)**	**0.13 (0.15)**	**0.08 (0.11)**	**-0.05 (0.13)**	N/A[A]	N/A[E]
**MORBIDITY EAI (K = 5)**	0.02** (0.01)	**-0.01 (0.02)**	N/A[B]	**0.03 (0.05)**	**0.04 (0.22)**	N/A[D]	**0.12 (0.21)**	**0.26 (0.22)**	**0.12 (0.27)**	N/A[A]	N/A[E]
MORBIDITY EID (K = 2)	NP	NP	NP	NP	NP	NP	NP	NP	NP	NP	NP
MORBIDITY EII (K = 1)	NP	NP	NP	NP	NP	NP	NP	NP	NP	NP	NP
MORBIDITY MET (K = 2)	NP	NP	NP	NP	NP	NP	NP	NP	NP	NP	NP
**MORTALITY: RVE (K = 6)**	NP	NP	NP	NP	NP	N/A[E]	NP	NP	NP	N/A[A]	N/A[D]
**MORTALITY RE (6)**	**-0.002 (0.001)**	**0.002 (0.002)**	**0.03 (0.05)**	0.01*** (0.003)	**NS (ALL COMPARI SONS)**	N/A[D]	**-0.05 (0.05)**	**0.02 (0.05)**	**0.02 (0.05)**	N/A[A]	N/A[D]
**MORTALITY EAI (3)**	NP	NP	NP	NP	NP	NP	NP	NP	NP	NP	NP
**MORTALITY EID (0)**	NP	NP	NP	NP	NP	NP	NP	NP	NP	NP	NP
**MORTALITY EII (0)**	NP	NP	NP	NP	NP	NP	NP	NP	NP	NP	NP
**MORTALITY MET (3)**	NP	NP	NP	NP	NP	NP	NP	NP	NP	NP	NP
**KNOWLEDGE RVE (K = 7)**	NP	NP	**-0.10 (0.10)**	NP	NP	NP	**-0.06 (0.13)**	NP	NP	NP	N/A[D]
**KNOWLEDGE RE (K = 9)**	**-0.02 (0.01)**	**-0.01 (0.02)**	0.44*** (0.11)	**-0.01 (0.02)**	0.44*** (0.11)	N/A[D]	**-0.05 (0.18)**	**0.05 (0.23)**	N/A[A]	**-0.01 (0.23)**	N/A[D]
**KNOWLEDGE EAI (K = 4)**	**N/A[E]**	**N/A[E]**	0.52* (0.21)	**-0.05 (0.05)**	0.52* (0.21)	**N/A[E]**	**N/A[B]**	**N/A[B]**	**N/A[A]**	**N/A[A]**	N/A[D]
KNOWLEDGE EID (K = 3)	NP	NP	NP	NP	NP	NP	NP	NP	NP	NP	NP
KNOWLEDGE EII (K = 0)	--	--	--	--	--	--	--	--	--	--	--
KNOWLEDGE MET (K = 2)	NP	NP	NP	NP	NP	NP	NP	NP	NP	NP	NP
ATTITUDES RVE (NO DEPENDENT EFFECTS)	N/A	N/A	N/A	N/A	N/A	N/A	N/A	N/A	N/A		
**ATTITUDES RE (K = 6)**	**0.01 (0.02)**	**-0.004 (0.02)**	**0.17 (0.34)**	**-0.01 (0.04)**	**0.17 (0.34)**	N/A[E]	**0.38 (0.29)**	**-0.09 (0.43)**	N/A[A]	N/A[B]	N/A[D]

For peer review only - http://bmjopen.bmj.com/site/about/guidelines.xhtml

ATTITUDES EAI (K = 2)	NP	NP	NP	NP	NP	NP	NP	NP	NP	NP	NP
ATTITUDES EID (K = 2)	NP	NP	NP	NP	NP	NP	NP	NP	NP	NP	NP
ATTITUDES EII (K = 0)	--	--	--	--	--	--	--	--	--	--	--
ATTITUDES MET (K = 2)	NP	NP	NP	NP	NP	NP	NP	NP	NP	NP	NP
CARD AVAIL/ RETENTION RVE (NO DEPENDENT EFFECTS)	N/A	N/A	N/A	N/A	N/A	N/A	N/A	N/A	N/A	N/A	N/A
CARD AVAIL/ RETENTION RE (K = 4)	N/A[C]	N/A[C]	N/A[C]	N/A[C]	N/A[C]	N/A[C]	N/A[C]	N/A[C]	N/A[C]	N/A[C]	N/A[D]
CARD AVAIL/ RETENTION EAI (K = 1)	NP	NP	NP	NP	NP	NP	NP	NP	NP	NP	NP
CARD AVAIL/ RETENTION EID (K = 0)	--	--	--	--	--	--	--	--	--	--	--
CARD AVAIL/ RETENTION EII (K = 1)	NP	NP	NP	NP	NP	NP	NP	NP	NP	NP	NP
CARD AVAIL/ RETENTION MET (K = 2)	NP	NP	NP	NP	NP	NP	NP	NP	NP	NP	NP
SATISFACTION W/ HEALTH WORKERS (K = 2)	NP	NP	NP	NP	NP	NP	NP	NP	NP	NP	NP
SATISFACTION W/ HEALTH WORKERS (K = 1)	NP	NP	NP	NP	NP	NP	NP	NP	NP	NP	NP
SATISFACTION W/ HEALTH WORKERS EAI (K = 1)	NP	NP	NP	NP	NP	NP	NP	NP	NP	NP	NP
SATISFACTION W/ HEALTH WORKERS EID (K = 1)	NP	NP	NP	NP	NP	NP	NP	NP	NP	NP	NP
SATISFACTION W/ HEALTH WORKERS EII (K = 0)	--	--	--	--	--	--	--	--	--	--	--
SATISFACTION W/ HEALTH WORKERS MET (K = 0)	--	--	--	--	--	--	--	--	--	--	--
FORMAL HEALTH WORKERS MOTIVATION, CAPACITY, AND PERFORMANCE (K = 2)	NP	NP	NP	NP	NP	NP	NP	NP	NP	NP	NP
FORMAL HEALTH WORKERS MOTIVATION, CAPACITY, AND PERFORMANCE (K = 1)	NP	NP	NP	NP	NP	NP	NP	NP	NP	NP	NP
FORMAL HEALTH WORKERS MOTIVATION, CAPACITY, AND PERFORMANCE EAI (K = 1)	NP	NP	NP	NP	NP	NP	NP	NP	NP	NP	NP
FORMAL HEALTH WORKERS MOTIVATION, CAPACITY, AND PERFORMANCE EID (K = 1)	NP	NP	NP	NP	NP	NP	NP	NP	NP	NP	NP

For peer review only

For peer review only - http://bmjopen.bmj.com/site/about/guidelines.xhtml

FORMAL HEALTH WORKERS MOTIVATION, CAPACITY, AND PERFORMANCE EII (K = 0)	--	--	--	--	--	--	--	--	--	--	--
FORMAL HEALTH WORKERS MOTIVATION, CAPACITY, AND PERFORMANCE MET (K = 0)	--	--	--	--	--	--	--	--	--	--	--

Note. NS = non-significant, NP = not powered, EAI = engagement as the intervention, EID = engagement in the intervention design, EII = engagement in implementation autonomy, MET = multiple engagement types, RVE = robust variance estimation, RE = random effects model using independent effects.

[a] Not applicable because all studies were in the same moderator group
[b] Not applicable because only one study was in a different moderator group
[c] Not applicable because there was no heterogeneity
[d] Not applicable because the moderator does not apply to the outcome category
[e] Not applicable because there was missing data
* $p < .05$, ** $p < .01$, *** $p < .001$

For peer review only

Appendix 10: Quantitative results – Engagement as intervention

Appendix 10 presents supplementary data for analyses presented in the main body of the manuscript for the primary outcomes of community engagement interventions using engagement as the intervention, including funnel plots of publication bias and outlier analyses. It also includes a full presentation of the supplementary outcomes that were precluded from the main body of the manuscript due to word limitations.

Full immunisation

Studies that used engagement as the intervention had a significant effect on full childhood immunisation ($\hat{\mu} = 0.08$ [95% CI: 0.03 to 0.13], $z = 3.02$, $p < 0.01$). According to the Q-test, the true outcomes appear to be heterogeneous ($Q(11) = 36.67, p < 0.01, \hat{\tau}^2 = 0.005, I^2 = 70.00\%$). An examination of the studentized residuals revealed that none of the studies had a value larger than ± 2.87 and hence there was no indication of outliers in the context of this model. According to the Cook's distances, none of the studies could be considered to be overly influential. The forest plot is presented below (Supplemenary Figure 33). With only one high or moderate quality study, sensitivity analysis by study quality could not be completed for this body of evidence. A funnel plot of the estimates is shown in Figure B1B. Neither the rank correlation nor the regression test indicated any funnel plot asymmetry ($p = 0.20$ and $p = 0.07$, respectively). Exposure to the intervention (in months) was the only significant source of heterogeneity such that for each additional month of intervention exposure, effects decreased by .003 standard deviation units ($\hat{B} = -0.003, p = 0.049$ [95% CI: -0.01 to -0.00001]).

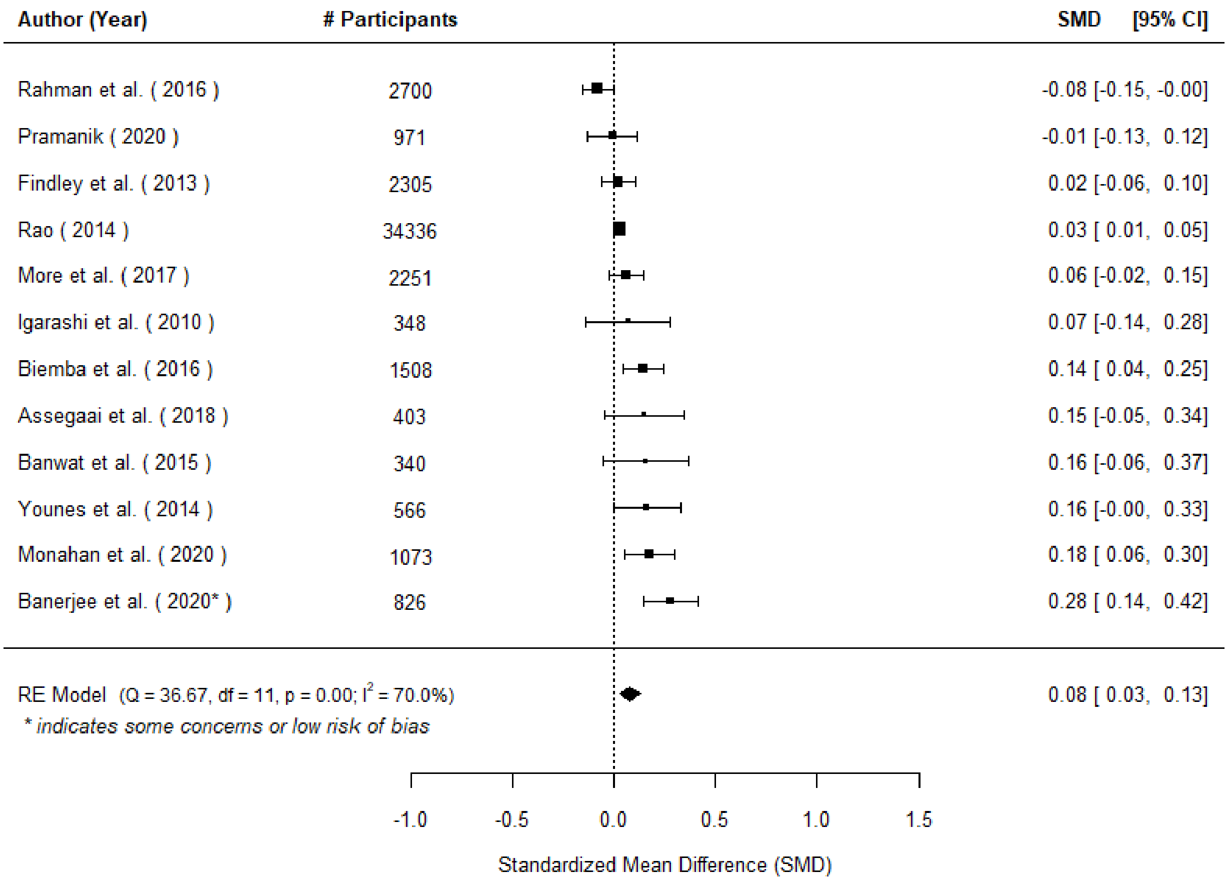

Supplementary Figure 33. Forest plot showing the observed outcomes and the estimate of the random-effects model for interventions with community engagement as the intervention on full immunisation

For peer review only - http://bmjopen.bmj.com/site/about/guidelines.xhtml

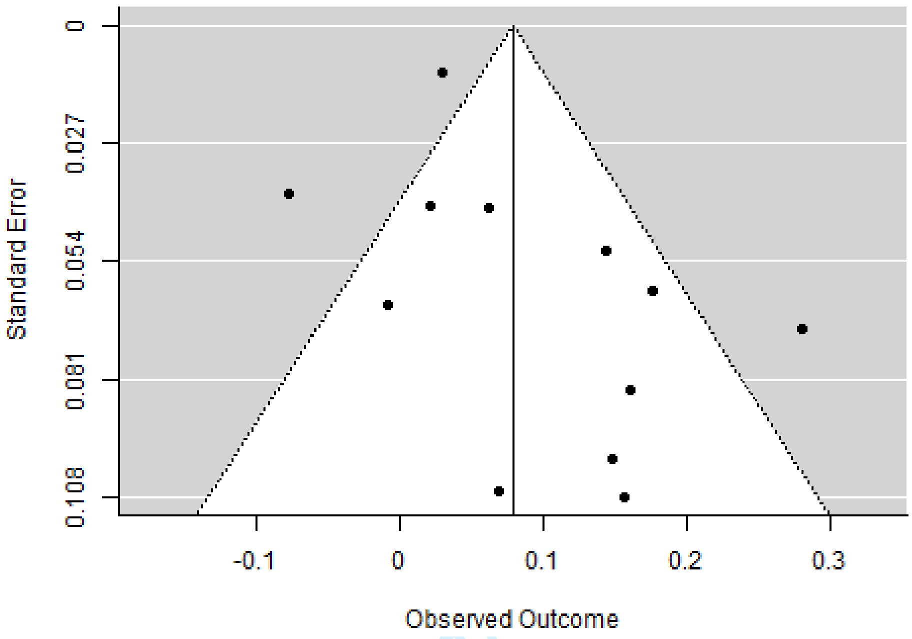

Supplementary Figure 33b. Forest plot showing the observed outcomes and the estimate of the random-effects model for interventions with community engagement as the intervention on full immunisation

DPT3

Studies that used engagement as the intervention also had a significant effect on DPT3 vaccination ($\hat{\mu} = 0.09$ [95% CI: 0.03 to 0.15], $z = 3.01$, $p < 0.01$). According to the Q-test, the true outcomes appear to be heterogeneous ($Q(5) = 18.64$, $p < 0.01$, $\hat{\tau}^2 = 0.00$, $I^2 = 73.17\%$). An examination of the studentized residuals revealed that none of the studies had a value larger than ± 2.64 and hence there was no indication of outliers in the context of this model. According to the Cook's distances, none of the studies could be considered to be overly influential. None of the moderators we tested were significant sources of heterogeneity (see Supplementary Table 3 in Appendix 9).

For peer review only - http://bmjopen.bmj.com/site/about/guidelines.xhtml

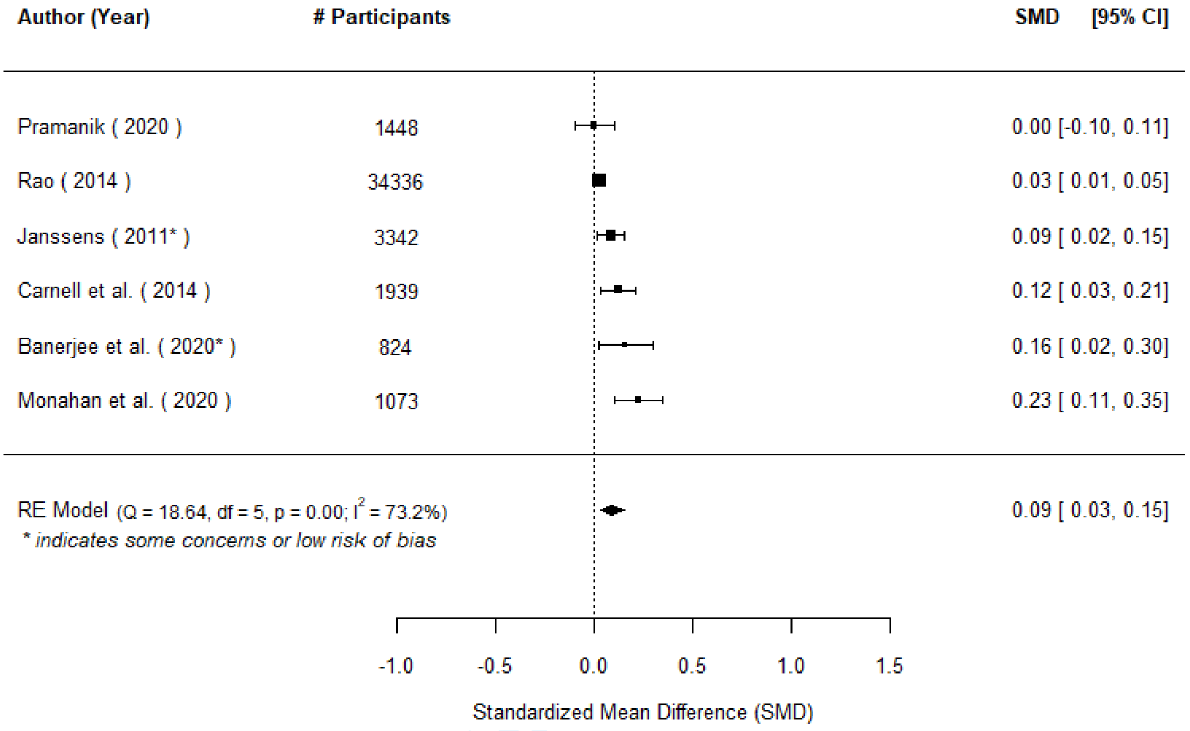

Supplementary Figure 34. Forest plot showing the observed outcomes and the estimate of the random-effects model for interventions with community engagement as the intervention on DPT3

Measles

Studies that used engagement as the intervention also had a significant effect on measles vaccination ($\hat{\mu} = 0.10$ [95% CI: 0.05 to 0.15], $z = 3.89$, $p < 0.001$). According to the Q-test, the true outcomes appear to be heterogeneous ($Q(9) = 22.67$, $p = 0.007$, $\hat{\tau}^2 = 0.003$, $I^2 = 60.29\%$). An examination of the studentized residuals revealed that none of the studies had a value larger than ± 2.81 and hence there was no indication of outliers in the context of this model. According to the Cook's distances, none of the studies could be considered to be overly influential.

A funnel plot of the estimates is shown in Figure 35b. The regression test indicated funnel plot asymmetry ($p < 0.01$) but not the rank correlation test ($p = 0.38$). None of the moderators we tested were significant sources of heterogeneity (see Supplementary Table 3 in Appendix 9).

For peer review only - http://bmjopen.bmj.com/site/about/guidelines.xhtml

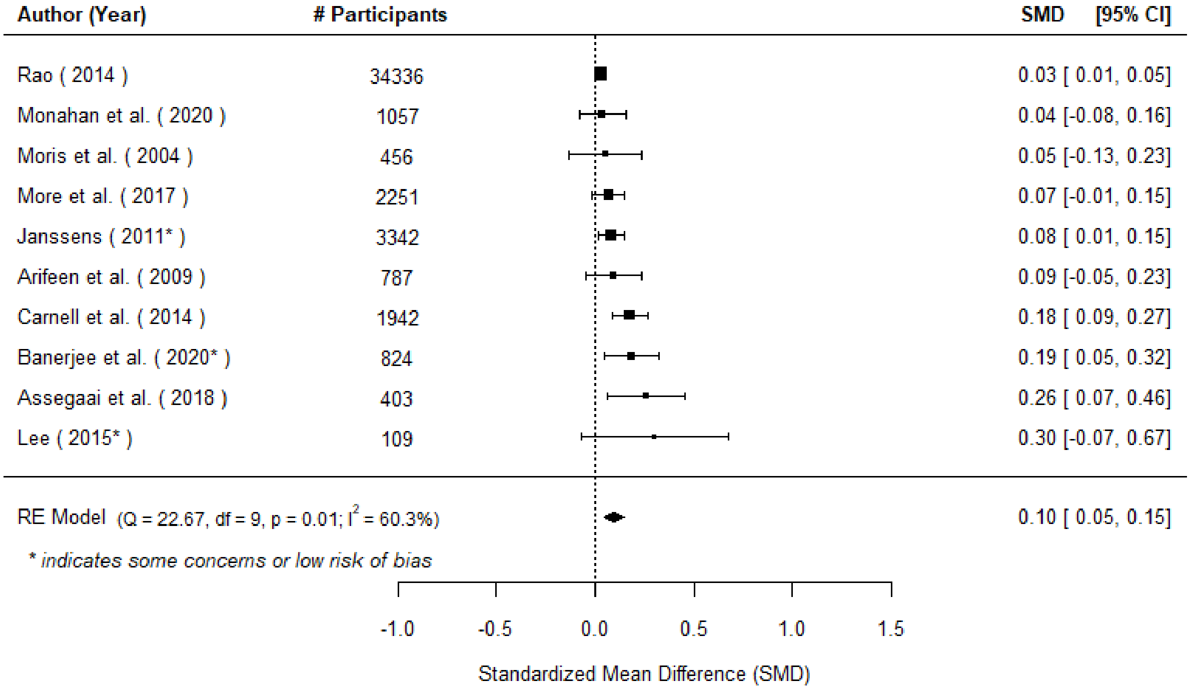

Supplementary Figure 35. Forest plot showing the observed outcomes and the estimate of the random-effects model for interventions with community engagement as the intervention on measles

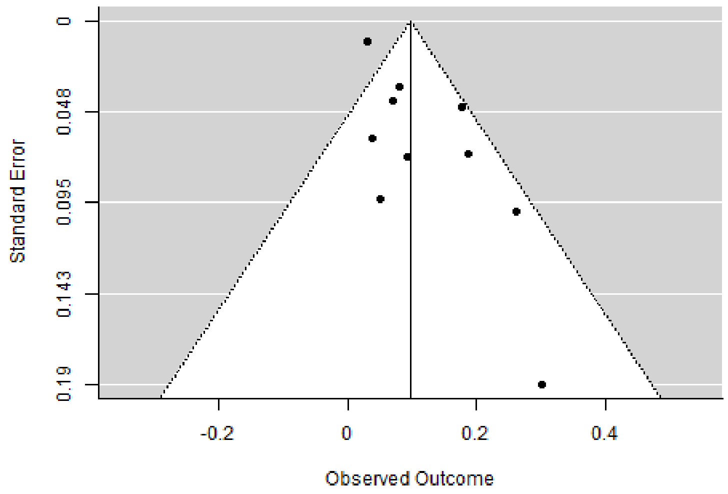

Supplementary Figure 35b. Funnel plot showing studies of community engagement as the intervention on measles

Timeliness of full childhood immunisation

Only one study (Saggurti 2018) fell into this intervention/outcome category, thus we were unable to perform a statistical synthesis. This quasi-experimental study from India found a small but significant impact of their programme on full childhood immunisation (g = 0.15 [95% CI: 0.04 to 0.27]), but like most studies, it was assessed as having a high risk of bias.

For peer review only - http://bmjopen.bmj.com/site/about/guidelines.xhtml

Timeliness of DPT3 vaccination

No studies examining timeliness of DPT3 vaccinations used engagement as the intervention.

Timeliness of measles vaccination

No studies examining timeliness of measles vaccinations used engagement as the intervention.

For peer r

Secondary outcomes

Partial immunisation

Only two studies reporting on partial immunisation used interventions with community engagement as the intervention. The estimated average outcome based on the random-effects model was $\hat{\mu} = 0.31$ ([95% CI: -0.25 to 0.87], $z = 1.10, p = 0.27$), indicating no significant difference between the intervention group and the control group on partial immunisation (see Supplementary Figure 36). Given the small number of studies, this result should be interpreted with caution. According to the Q-test, the true outcomes appear to be heterogeneous ($Q(1) = 95.93$, $p < 0.001$, $\hat{\tau}^2 = 0.16$, $I^2 = 98.96\%$). With only two studies, moderator analyses were not appropriate and tests of publication bias are not valid.

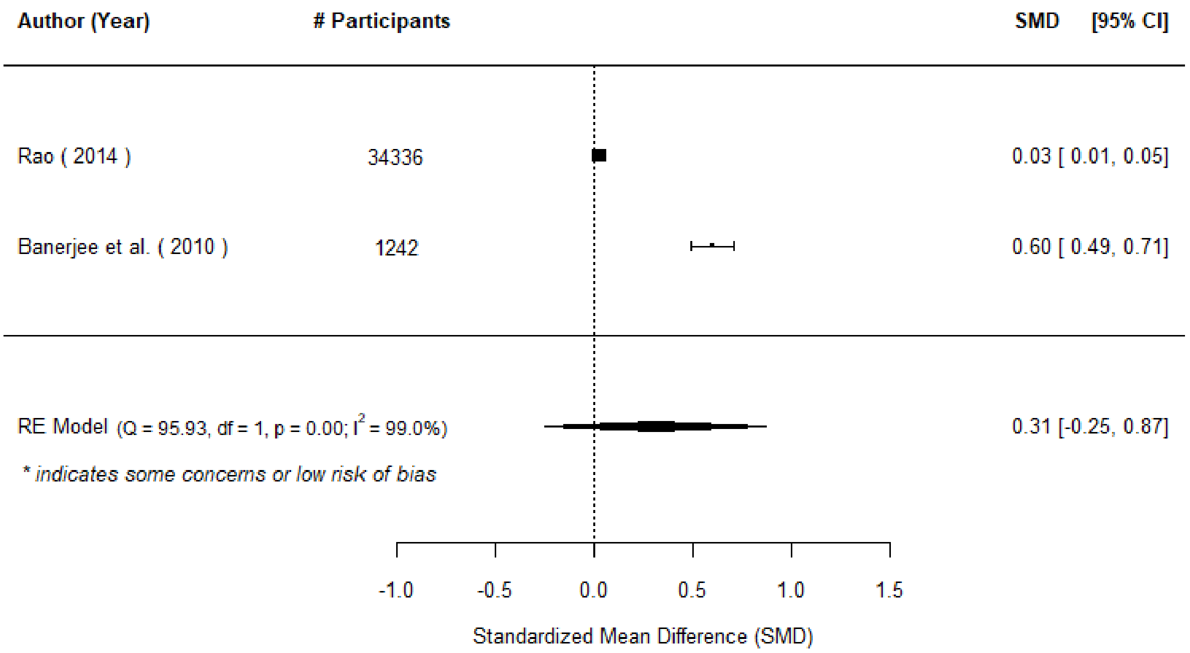

Supplementary Figure 36. *Forest plot showing the observed outcomes and the estimate of the random-effects model for interventions with community engagement as the intervention on partial immunisation.*

BCG

For peer review only - http://bmjopen.bmj.com/site/about/guidelines.xhtml

We included a total of $k = 4$ studies the analysis. The estimated average outcome based on the random-effects model was $\hat{\mu} = 0.02$ (95% CI: 0.01 to 0.03). Therefore, the average outcome differed significantly from zero ($z = 3.32$, $p < 0.01$), indicating a very small but significant benefit to the treated group compared to the control group (see Supplementary Figure 37). According to the Q-test, there was no significant amount of heterogeneity in the true outcomes ($Q(3) = 2.98$, $p = 0.39$, $\hat{\tau}^2 = 0.00$, $I^2 = 0.00\%$), thus we did not examine potential sources of heterogeneity for this model. An examination of the studentized residuals revealed that none of the studies had a value larger than ± 2.50 and hence there was no indication of outliers in the context of this model. According to the Cook's distances, none of the studies could be considered to be overly influential.

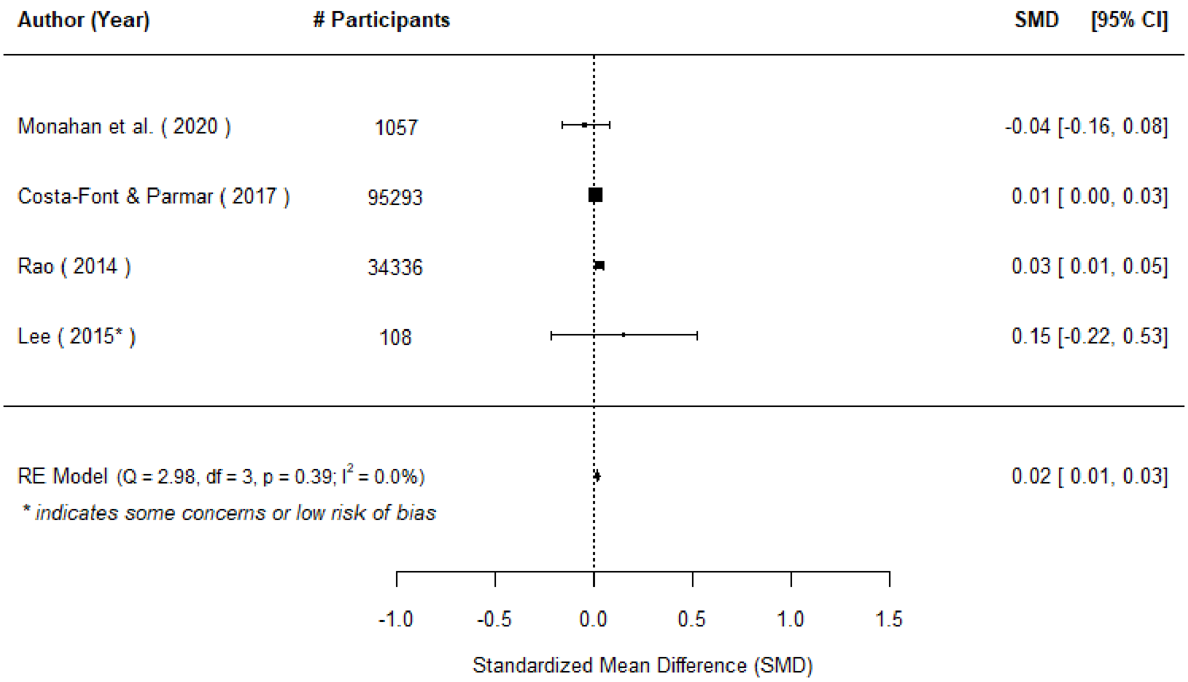

Supplementary Figure 37. *Forest plot showing the observed outcomes and the estimate of the random-effects model for interventions with community engagement as the intervention on BCG vaccination.*

DPT1

We included a total of $k = 3$ studies in the analysis. The estimated average outcome based on the random-effects model was $\hat{\mu} = 0.10$ ([95% CI: -0.03 to 0.22], $z = 1.53$, $p = 0.13$), indicating no difference between the treatment and control groups (see Supplementary Figure 38). According to the Q-test, the true outcomes appear to be heterogeneous ($Q(2) = 20.42$, $p < 0.01$, $\hat{\tau}^2 = 0.01$, $I^2 = 90.21\%$). An examination of the studentized residuals revealed that one study (Costa-Font & Parmar 2017) had a value larger than ± 2.39 and may be a potential outlier in the context of this model. Indeed, sensitivity analyses leaving each study out indicated that removing Costa-Font & Parmar (2017) would increase the overall average effect ($\hat{\mu}$ = 0.16 (95% CI: 0.09 to 0.22), and the resulting effect would be positive and significant (z = 4.72, p < .001). According to the Cook's distances, none of the studies could be considered to be overly influential. With only three studies, we were unable to conduct moderator analyses or test for publication bias. Only one study (Banerjee et al. 2020) was not assessed as high risk of bias.

For peer review only - http://bmjopen.bmj.com/site/about/guidelines.xhtml

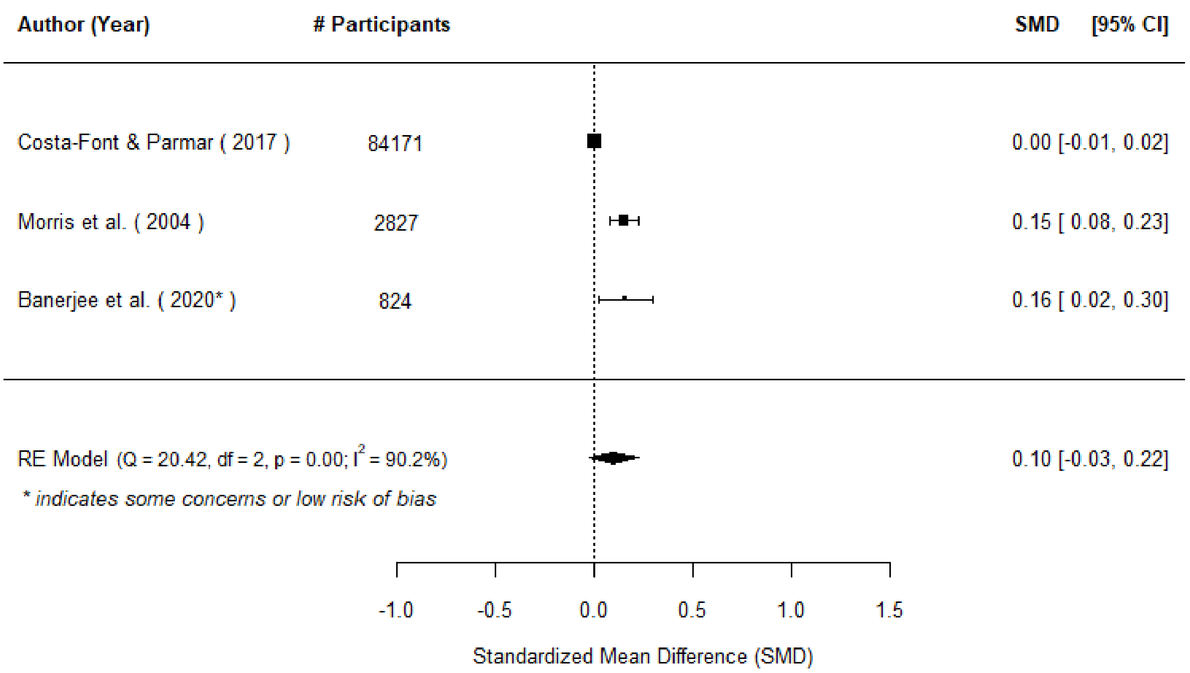

Supplementray Figure 38. *Forest plot showing the observed outcomes and the estimate of the random-effects model for interventions with community engagement as the intervention on DPT1 vaccination.*

DPT2

Only one study (Banerjee et al. 2020) fell into this intervention/outcome category, thus we were unable to perform a statistical synthesis. This cluster RCT from India found a small but significant positive effect of their programme on DPT2 vaccination (*g* = 0.15 [95% CI: 0.01 to 0.29]), but like most studies, it was assessed as having a high risk of bias.

OPV0

Only one study (Costa-Font & Parmar 2017) fell into this intervention/outcome category, thus we were unable to perform a statistical synthesis. This quasi-experimental study from India found a null effect of their programme on OPV0 vaccination (*g* = 0.01 [95% CI: -0.01 to 0.03]), but like most studies, it was assessed as having a high risk of bias.

OPV1

There were no studies reporting on OPV1 vaccination that used interventions with community engagement as the intervention.

OPV2

There were no studies reporting on OPV2 vaccination that used interventions with community engagement as the intervention.

For peer review only - http://bmjopen.bmj.com/site/about/guidelines.xhtml

OPV3

We included a total of $k = 3$ studies in the analysis. The estimated average outcome based on the random-effects model was $\hat{\mu} = 0.16$ ([95% CI: -0.02 to 0.34], $z = 1.78$, $p = 0.07$), indicating no significant difference between the intervention group and the control group on OPV3 vaccination (see Supplementary Figure 39). According to the Q-test, the true outcomes appear to be heterogeneous ($Q(2) = 12.37$, $p = 0.002$, $\hat{\tau}^2 = 0.02$, $^2 = 83.83\%$). An examination of the studentized residuals revealed that one study (Lee 2015) had a value larger than ± 2.39 and may be a potential outlier in the context of this model. Indeed, sensitivity analyses leaving each study out indicated that removing Lee (2015) would reduce the overall average effect ($\hat{\mu} = 0.08$ [95% CI: -0.04 to 0.19]), though in either case the effect is not significantly different from zero ($z = 1.29$, $p = 0.20$). According to the Cook's distances, none of the studies could be considered to be overly influential. With only three studies contributing effects, moderator analyses were not appropriate.

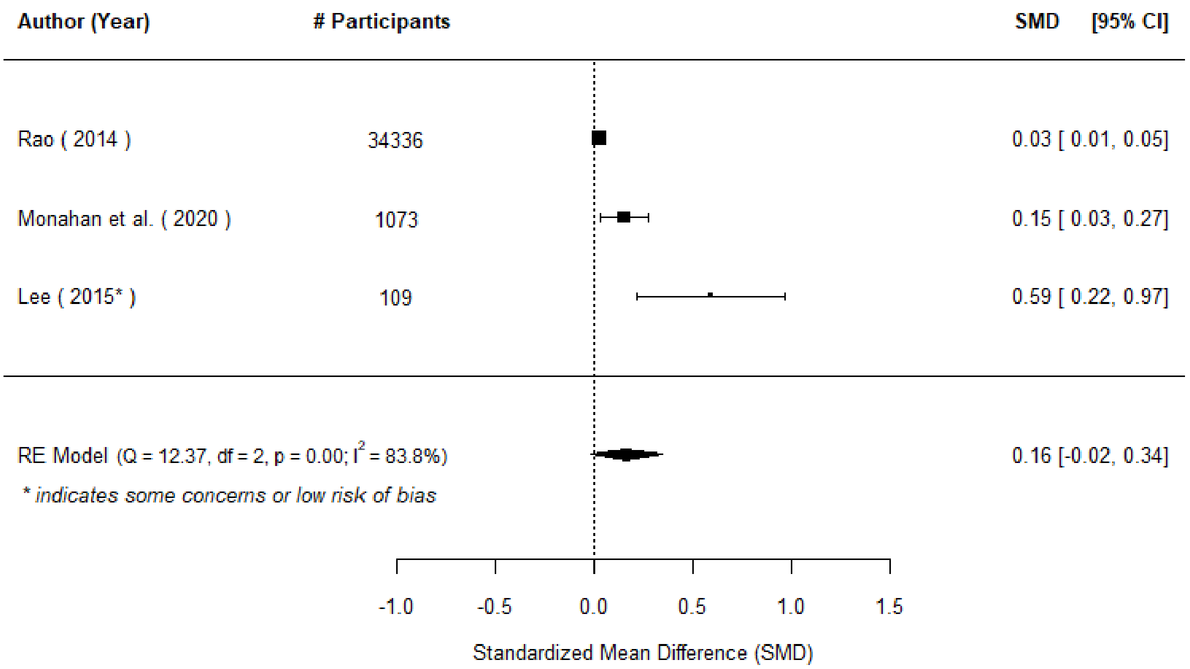

Supplementary Figure 39. Forest plot showing the observed outcomes and the estimate of the random-effects model for interventions with community engagement as the intervention on OPV3 vaccination.

Dropouts

Only two studies reporting on vaccination dropouts used interventions with community engagement as the intervention. The estimated average outcome based on the random-effects model was $\hat{\mu} = 0.02$ ([95% CI: -0.03 to 0.06], $z = 0.63$, $p = 0.53$), indicating no significant difference between the intervention group and the control group on vaccination dropouts (see Supplementary Figure 40). Given the small number of studies, this result should be interpreted with caution. According to the Q-test, the true outcomes appear to be homogeneous ($Q(1) = 0.03$, $p = 0.86$, $\hat{\tau}^2 = 0.00$, $I^2 = 0.00\%$). With only two studies and no heterogeneity, moderator analyses were not appropriate and tests of publication bias are not valid.

For peer review only - http://bmjopen.bmj.com/site/about/guidelines.xhtml

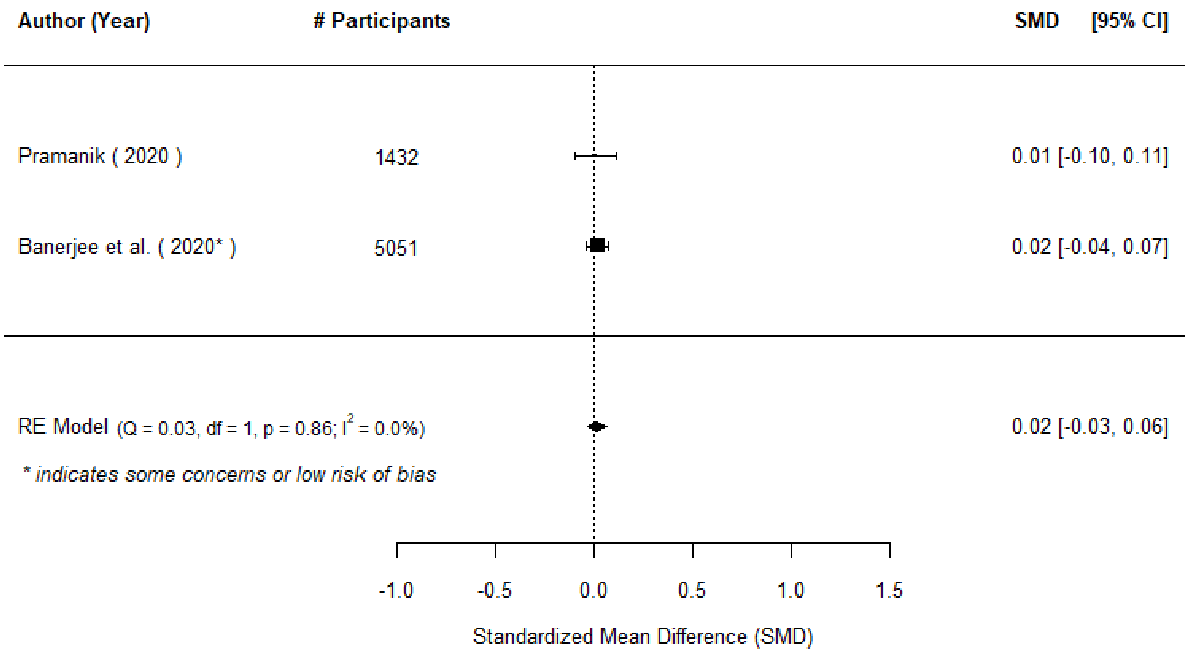

Supplementary Figure 40. Forest plot showing the observed outcomes and the estimate of the random-effects model for interventions with community engagement as the intervention on vaccination dropouts.

Morbidity

We used reports of diarrhea (most typically in the past two weeks) as a proxy for childhood morbidity. In all cases, effects were reverse coded such that positive effects always indicate a benefit to the treated group. Thus, a positive effect here would be interpreted as a reduction in diarrhea among treated participants compared to control participants. We included a total of $k = 5$ studies in the analysis. The estimated average outcome based on the random-effects model was $\hat{\mu} = -0.004$ (95% CI: -0.11 to 0.10). Therefore, the average outcome did not differ significantly from zero ($z = -0.08$, $p = 0.94$), indicating no difference between the treatment and control groups on childhood morbidity (see Supplementary Figure 41). According to the Q-test, the true outcomes appear to be heterogeneous ($Q(4) = 35.91$, $p < 0.01$, $\hat{\tau}^2 = 0.01$, $I^2 = 88.86\%$). An examination of the studentized residuals revealed that one study (Assegaai et al. 2018) had a value larger than ± 2.58 and may be a potential outlier in the context of this model. Indeed, sensitivity analyses leaving each study out indicated that removing Assegaai and colleagues (2018) would reduce the overall average effect ($\hat{\mu}$ = -0.06 (95% CI: -0.14 to 0.02). While the resulting effect would be negative, the effect would still be non-significant (z = -1.50, *p* = .13). According to the Cook's distances, none of the studies could be considered to be overly influential. There was only one study not assessed as high risk of bias, so we were unable to do a sensitivity analysis by study quality. Exposure to the intervention was a significant source of heterogeneity such that each additional month of exposure increased the positive impact on morbidity by 0.02 standard deviation units $\hat{\mu} = 0.02$ (95% CI: 0.01 to $0.03, p = 0.002$). No other moderators were significant (see Supplementary Table 3).

For peer review only - http://bmjopen.bmj.com/site/about/guidelines.xhtml

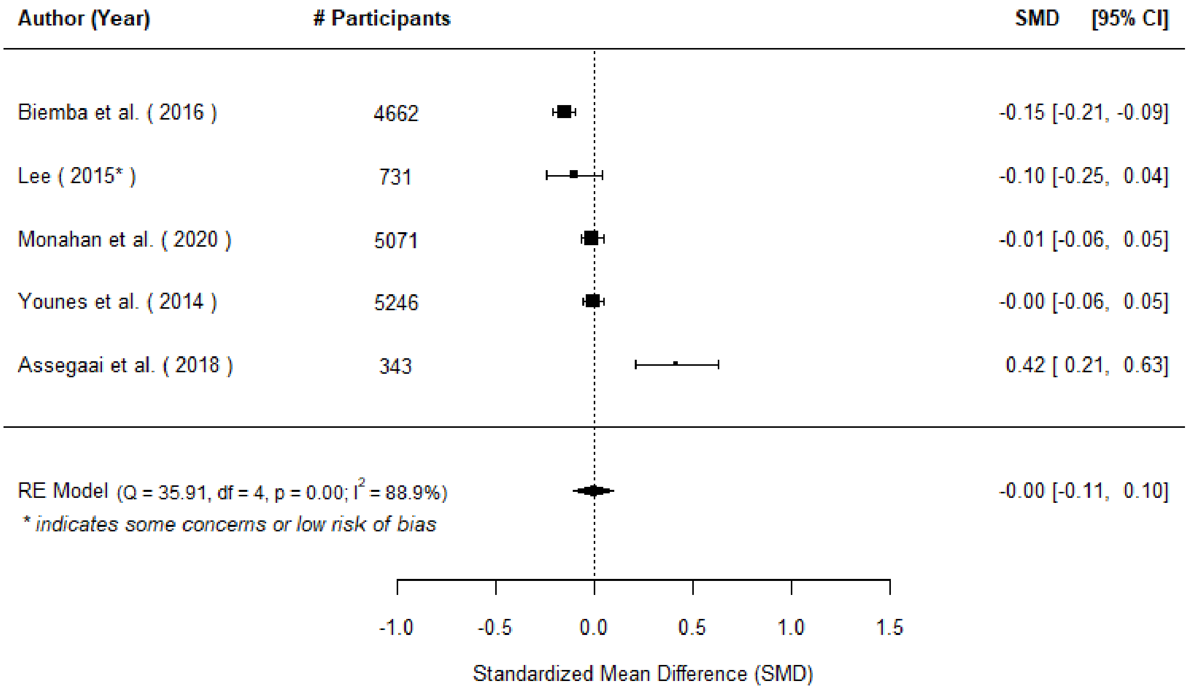

Supplementary Figure 41. Forest plot showing the observed outcomes and the estimate of the random-effects model for interventions with community engagement as the intervention on morbidity.

Mortality

We included a total of $k = 3$ studies in the analysis. The estimated average outcome based on the random-effects model was $\hat{\mu} = -0.04$ (95% CI: -0.11 to 0.04). Therefore, the average outcome did not differ significantly from zero ($z = -0.94$, $p = 0.34$), indicating no difference between the treatment and control group (see Supplementary Figure 42). According to the Q-test, the true outcomes appear to be heterogeneous ($Q(2) = 10.35$, $p < 0.01$, $\hat{\tau}^2 = 0.00$, $I^2 = 80.68\%$). An examination of the studentized residuals revealed that one study (Monahan et al. 2020) had a value larger than ± 2.39 and may be a potential outlier in the context of this model. Indeed, sensitivity analyses leaving each study out indicated that removing Monahan and colleagues (2020) would reduce the overall average effect ($\hat{\mu}$ = 0.01 [95% CI: -0.14 to 0.16]), but the effect would still be positive and non-significant (z = 0.10, p = .92). According to the Cook's distances, none of the studies could be considered to be overly influential. With only three studies contributing effects, we were unable to test for potential sources of heterogeneity.

For peer review only - http://bmjopen.bmj.com/site/about/guidelines.xhtml

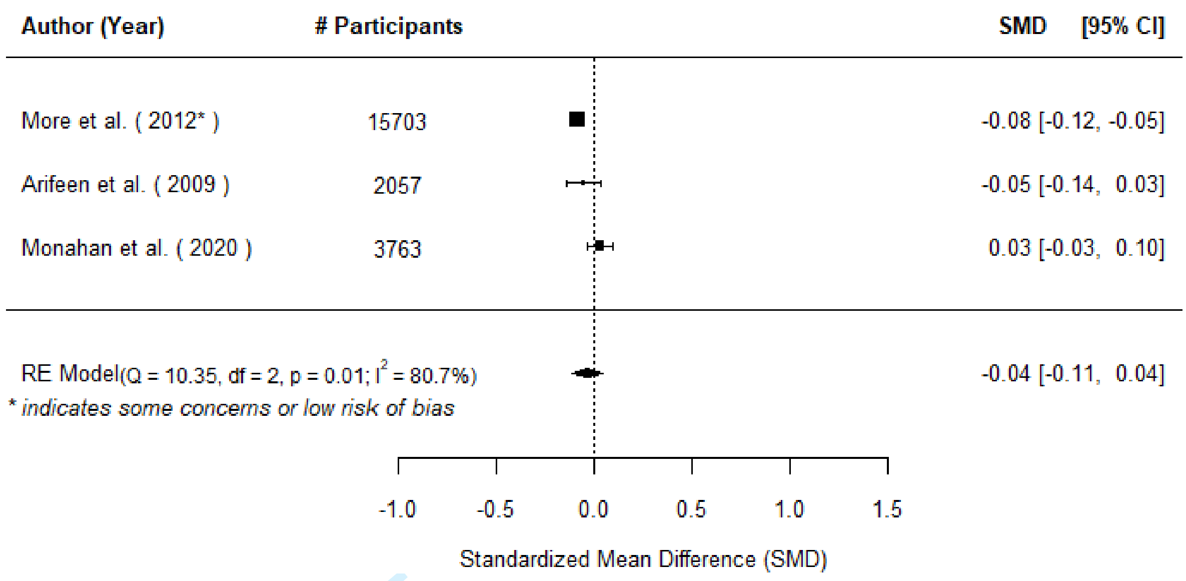

Supplementary Figure 42. Forest plot showing the observed outcomes and the estimate of the random-effects model for interventions with community engagement as the intervention on mortality.

Immunisation knowledge

We included a total of $k = 4$ studies in the analysis. The estimated average outcome based on the random-effects model was $\hat{\mu} = 0.20$ (95% CI: -0.08 to 0.49). Therefore, the average outcome did not differ significantly from zero ($z = 1.41$, $p = 0.16$), indicating no difference between the treatment and control groups. According to the Q-test, the true outcomes appear to be heterogeneous ($Q(3) = 48.39$, $p < 0.01$, $\hat{\tau}^2 = 0.08$, $I^2 = 93.80\%$). An examination of the studentized residuals revealed that one study (Banwat et al. 2015) had a value larger than ± 2.50 and may be a potential outlier in the context of this model. Indeed, sensitivity analyses leaving each study out indicated that removing Banwat and colleagues (2015) would reduce the overall average effect ($\hat{\mu}$ = 0.02 (95% CI: -0.12 to 0.17), but the effect would still be positive and non-significant (z = 0.35, p = .73). According to the Cook's distances, none of the studies could be considered to be overly influential.

We examined potential sources of heterogeneity, and study design was a significant predictor of immunisation knowledge such that studies using quasi-experimental designs had larger average effects than RCT designs by .52 standard deviation units ($\hat{\beta} = 0.52$ [95% CI: 0.11 to 0.94], p = 0.01). Region was also a significant predictor, but region was perfectly confounded with study design (e.g. all studies from South Asia were RCTs, while all studies from Sub-Saharan Africa were all quasi-experimental designs), so the effects were larger in Sub-Saharan Africa than from South Asia by the same .52 standard deviation units ($\beta = 0.52$ [95% CI: 0.11 to 0.94], p = 0.01). No other moderators were significant (see Supplementary Table 3 in Appendix 9)

For peer review only - http://bmjopen.bmj.com/site/about/guidelines.xhtml

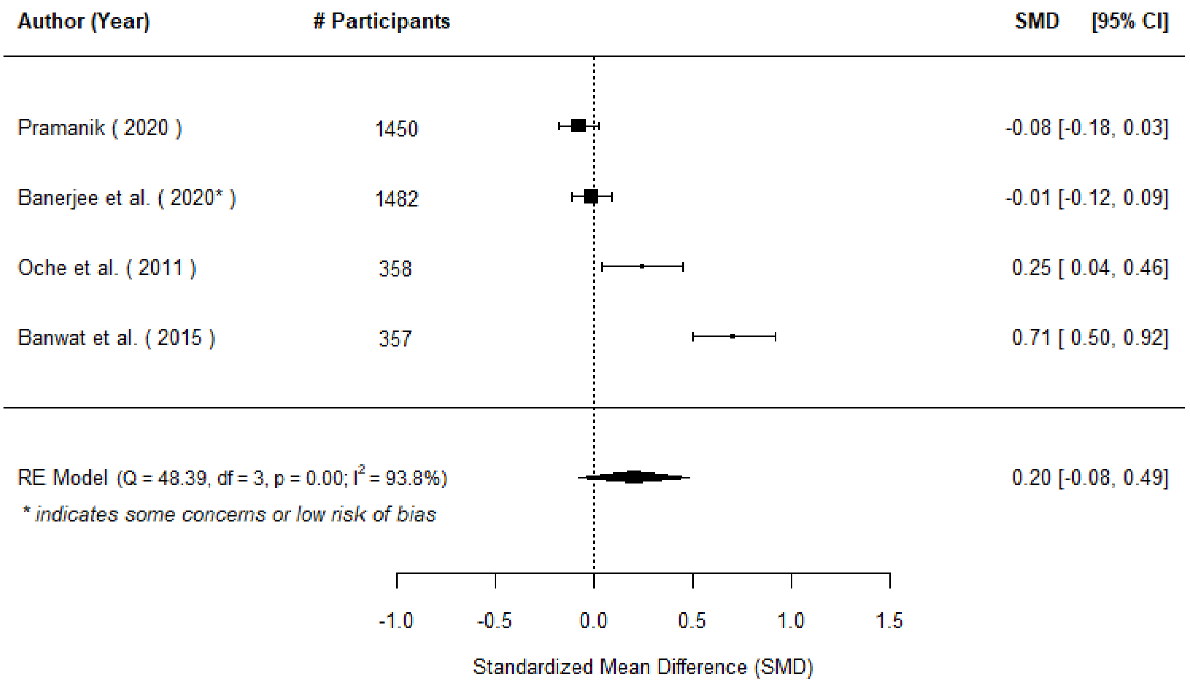

Supplementary Figure 43. Forest plot showing the observed outcomes and the estimate of the random-effects model for interventions with community engagement as the intervention on immunisation attitudes.

Immunisation attitudes

Only two studies reporting on immunisation attitudes used interventions with community engagement as the intervention. The estimated average outcome based on the random-effects model was $\hat{\mu} = 0.47$ ([95% CI: -0.19 to $1.13], z = 1.40, p = 0.16$), indicating no significant difference between the intervention group and the control group (see Supplementary Figure 44). Given the small number of studies, this result should be interpreted with caution. According to the Q-test, the true outcomes appear to be heterogeneous ($Q(1) = 27.51$, $p < 0.001$, $\hat{\tau}^2 = 0.22$, $I^2 = 96.36\%$; With only two studies and no heterogeneity, moderator analyses were not appropriate and tests of publication bias are not valid.

For peer review only - http://bmjopen.bmj.com/site/about/guidelines.xhtml

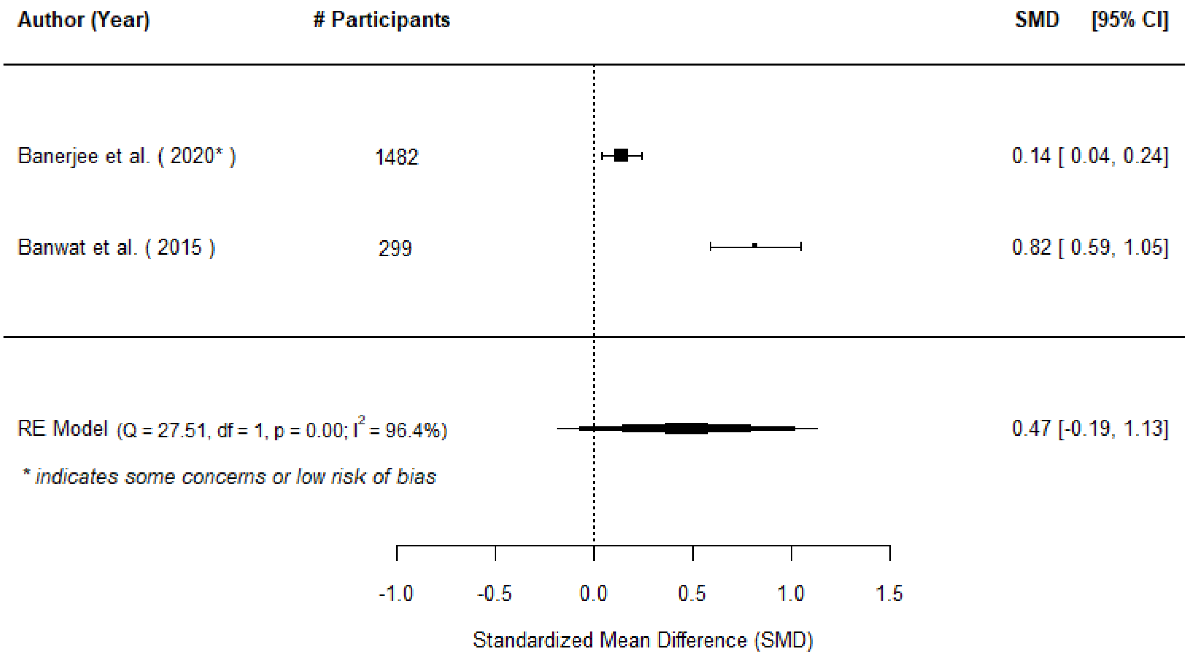

Supplementary Figure 44. Forest plot showing the observed outcomes and the estimate of the random-effects model for interventions with community engagement as the intervention on immunisation attitudes.

Vaccination card retention

Only one study (Pramanik 2020) fell into this intervention/outcome category, thus we were unable to perform a statistical synthesis. This cluster RCT from India found a null effect of their programme on vaccination card retention/availability (*g* = -0.07 [95% CI: -0.17 to 0.04]), but like most studies, it was assessed as having a high risk of bias.

Experience and satisfaction with health services

Only one study (Mohanan et al. 2020) fell into this intervention/outcome category, thus we were unable to perform a statistical synthesis. This cluster RCT from India did not find a significant effect of their programme on satisfaction with health services (*g* = -0.02 [95% CI: -0.08 to 0.04]), but like most studies, it was assessed as having a high risk of bias.

Formal health worker's motivation, capacity and performance

Only one study (Lee 2015) fell into this intervention/outcome category, thus we were unable to perform a statistical synthesis. This cluster RCT from Zambia did not find a significant effect of their programme on formal health worker's motivation, capacity, and performance (*g* = 0.09 [95% CI: -0.13 to 0.31]). This study was assessed as was assessed as having some concerns related to risk of bias.

For peer review only - http://bmjopen.bmj.com/site/about/guidelines.xhtml

Appendix 11: Quantitative results - community engagement in intervention design

Appendix 11 presents supplementary data for analyses presented in the main body of the manuscript for the primary outcomes of community engagement interventions using engagement in the intervention design, including funnel plots of publication bias and outlier analyses. It also includes a full presentation of the supplementary outcomes that were precluded from the main body of the manuscript due to word limitations.

Full immunisation

When studies used community engagement in the design, there was a significant effect on full childhood immunisation ($\hat{\mu} = 0.10$ [95% CI: 0.02 to 0.19], $z = 2.40$, $p = 0.02$). According to the Q-test, there was no significant amount of heterogeneity in the true outcomes ($Q(4) = 5.25$, $p = 0.26$, $\hat{\tau}^2 = 0.00$, $I^2 = 23.83\%$). An examination of the studentized residuals revealed that none of the studies had a value larger than ± 2.58 and hence there was no indication of outliers in the context of this model. According to the Cook's distances, none of the studies could be considered to be overly influential.

With no heterogeneity among effects, moderator analyses were not appropriate. When low quality studies were removed, two studies remained, and the summary effect increased ($\hat{\mu} = 0.14$ [95% CI: -0.01 to 0.29, but the effect was no longer significant, ($z = 1.89$, $p = 0.06$).

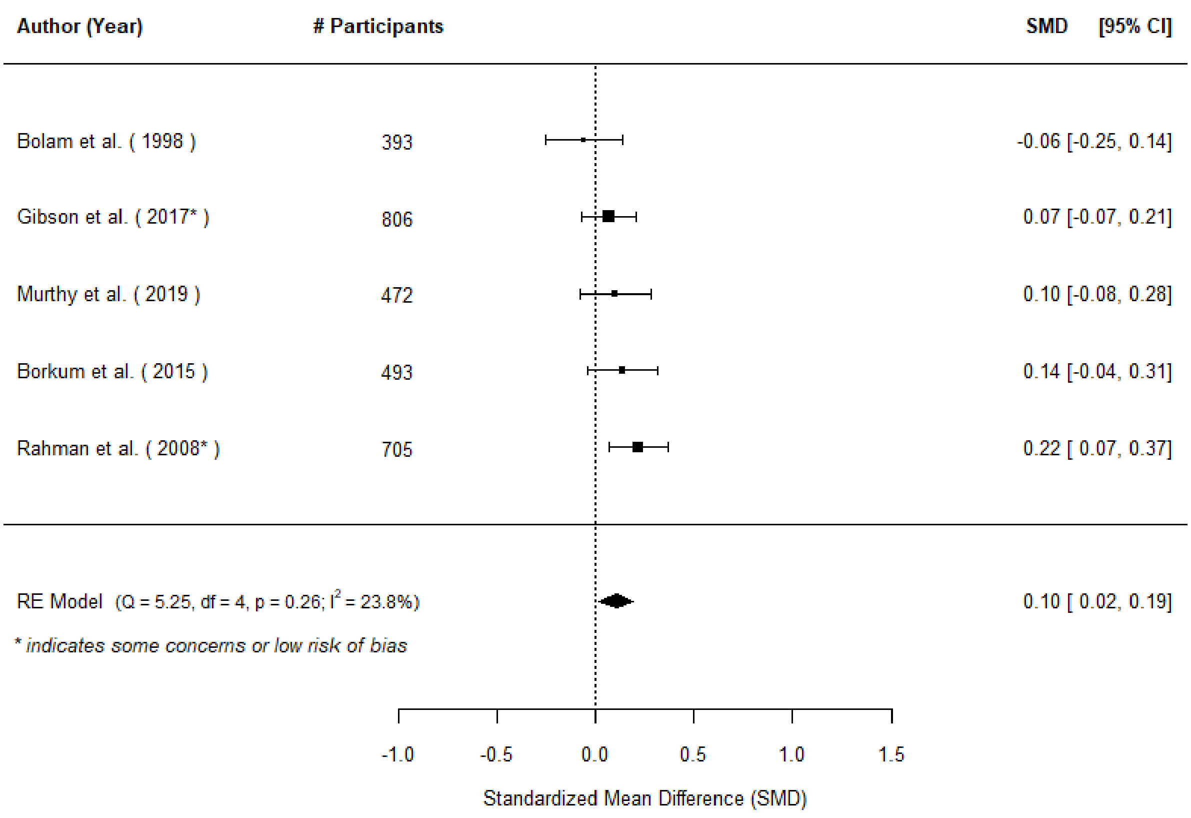

Supplementary Figure 45. Forest plot showing the observed outcomes and the estimate of the random-effects model for interventions with community engagement in the design on full immunisation

DPT3

For peer review only - http://bmjopen.bmj.com/site/about/guidelines.xhtml

When studies used community engagement in the design, there was not a significant effect on DPT3 vaccination ($\hat{\mu} = 0.04$ [95% CI: -0.01 to 0.08], $z = 1.69, p = 0.09$). According to the Q-test, there was no significant amount of heterogeneity in the true outcomes ($Q(5) = 4.36$, $p = 0.50$, $\hat{\tau}^2 = 0.00$, $I^2 = 0.00\%$). An examination of the studentized residuals revealed that none of the studies had a value larger than ± 2.64 and hence there was no indication of outliers in the context of this model. According to the Cook's distances, none of the studies could be considered to be overly influential. With no heterogeneity, we did not perform moderator analyses. With only one study of low or medium quality, we were also unable to complete sensitivity analyses for this body of evidence.

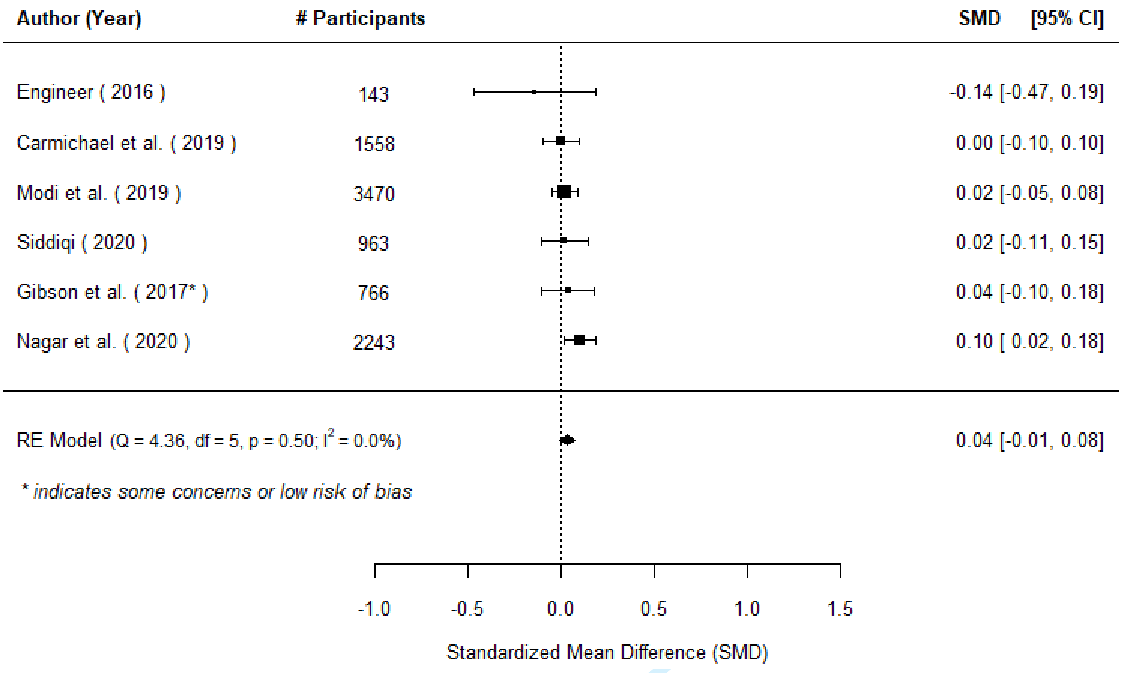

Supplementary Figure 46. Forest plot showing the observed outcomes and the estimate of the random-effects model for interventions with community engagement in the design on DPT3 vaccination

Measles

When studies used community engagement in the design, there was a significant effect on meales vaccination ($\hat{\mu} = 0.11$ [95% CI: 0.02 to 0.21], $z = 2.36$, $p = 0.02$). According to the Q-test, there was no significant amount of heterogeneity in the true outcomes ($Q(1) = 0.56$, $p = 0.46$, $\hat{\tau}^2 = 0.00$, $I^2 = 0.00\%$). With only two studies and no heterogeneity among effects, moderator analyses were not appropriate.

For peer review only - http://bmjopen.bmj.com/site/about/guidelines.xhtml

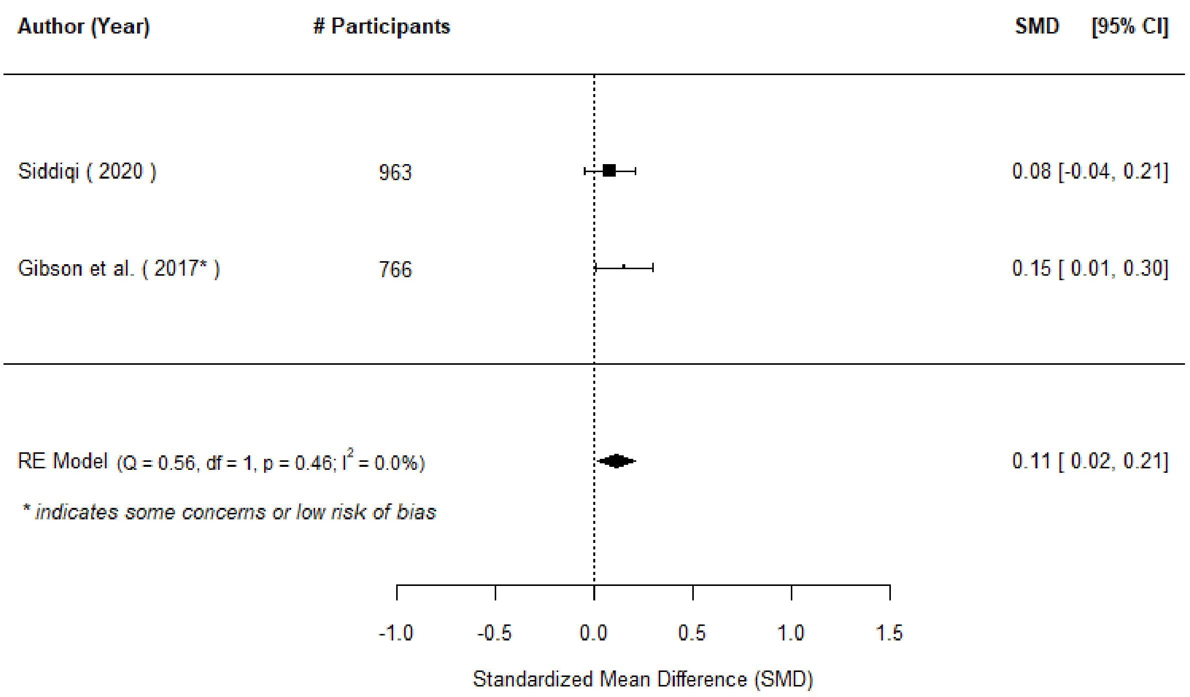

Supplementary Figure 47. Forest plot showing the observed outcomes and the estimate of the random-effects model for interventions with community engagement in the design on measles vaccination

Timeliness of full childhood immunisation

When studies used community engagement in the design, there was also a significant effect on timeliness full childhood immunisation $\hat{\mu} = 0.15$ ([95% CI: 0.004 to 0.29], $z = 2.01$, $p = 0.04$). According to the Q-test, there was no significant amount of heterogeneity in the true outcomes ($Q(1) = 0.81$, $p = 0.37$, $\hat{\tau}^2 = 0.00$, $I^2 = 0.00\%$). Both studies were assessed as high risk of bias. With only two studies and no heterogeneity, moderator analyses were not appropriate.

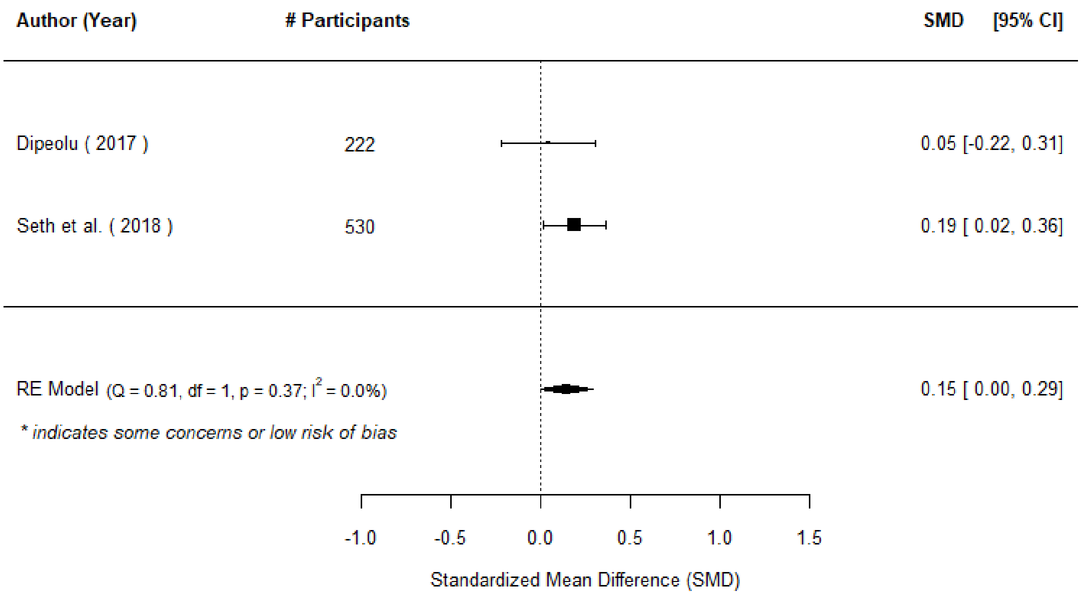

For peer review only - http://bmjopen.bmj.com/site/about/guidelines.xhtml

Supplementary Figure 48. Forest plot showing the observed outcomes and the estimate of the random-effects model for interventions with community engagement in the design on the timeliness of full childhood immunisation

Timeliness of DPT3 vaccination

When studies used community engagement in the design, there was also a significant effect on the timeliness of DPT3 vaccinations ($\hat{\mu} = 0.12$ [95% CI: 0.03 to 0.21], $z = 2.73$, $p < 0.01$). According to the Q-test, there was no significant amount of heterogeneity in the true outcomes ($Q(3) = 2.62$, $p = 0.45$, $\hat{\tau}^2 = 0.00$, $I^2 = 0.00\%$). An examination of the studentized residuals revealed that none of the studies had a value larger than ± 2.50 and hence there was no indication of outliers in the context of this model. According to the Cook's distances, none of the studies could be considered to be overly influential. With no heterogeneity indicated, we did not test for moderation.

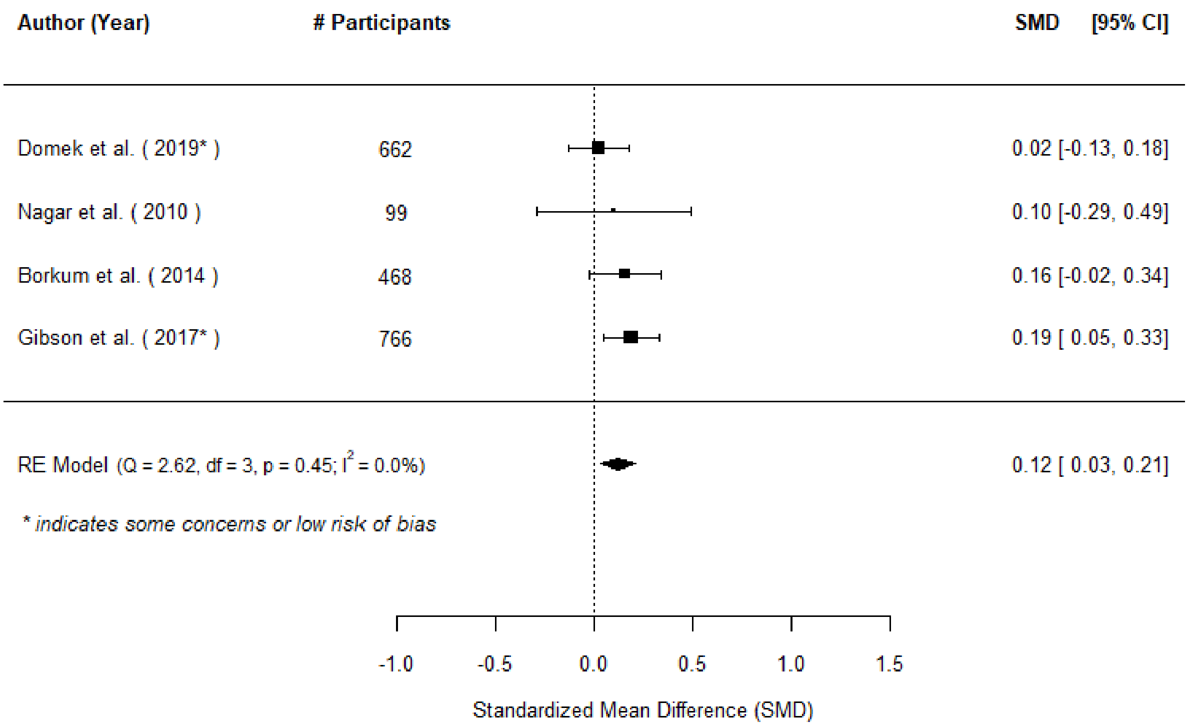

Supplementary Figure 49. Forest plot showing the observed outcomes and the estimate of the random-effects model for interventions with community engagement in the design of the intervention on the timeliness of DPT3 vaccinations.

Timeliness of measles vaccination

Only one study reporting on timeliness of measles vaccination used an intervention with engagement in the design (Gibson et al. 2017). This RCT conducted in Kenya found a small but significant positive effect (g = 0.20 [95% CI; 0.06 to 0.34]). This study was assessed as having some concerns of bias.

Secondary outcomes

Partial immunisation

For peer review only - http://bmjopen.bmj.com/site/about/guidelines.xhtml

Only two studies reporting on partial immunisation used interventions with community engagement in the intervention design. The estimated average outcome based on the random-effects model was $\hat{\mu} = 0.14$ ([95% CI: 0.01 to 0.27], $z = 2.05$, $p = 0.04$), indicating a small but significant benefit to the intervention group compared to the control group (see Supplementary Figure 50). Given the small number of studies, this result should be interpreted with caution. According to the Q-test, the true outcomes appear to be homogeneous ($Q(1) = 0.91$, $p = 0.34$, $\hat{\tau}^2 = 0.00$, $I^2 = 0.00\%$). With only two studies and no heterogeneity, moderator analyses were not appropriate and tests of publication bias are not valid.

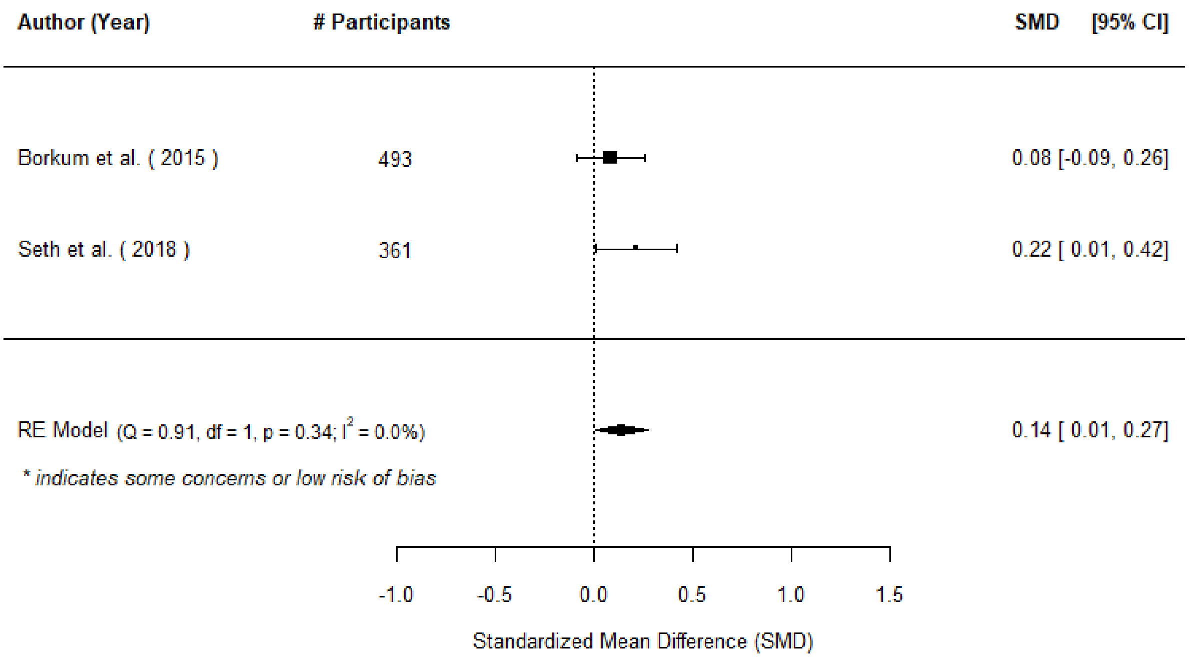

Supplementary Figure 50. Forest plot showing the observed outcomes and the estimate of the random-effects model for interventions with community engagement in the design of the intervention on partial childhood immunisation.

BCG

Only two studies reporting on BCG vaccination used interventions with community engagement in the intervention design. The estimated average outcome based on the random-effects model was $\hat{\mu} = 0.02$ ([95% CI: -0.09 to 0.13], $z = 0.32$, $p = 0.75$), indicating no difference between the intervention group and the control group (see Supplementary Figure 51). Given the small number of studies, this result should be interpreted with caution. According to the Q-test, the true outcomes appear to be homogeneous ($Q(1) = 0.11$, $p = 0.75$, $\hat{\tau}^2 = 0.00$, $I^2 = 0.00\%$). With only two studies and no heterogeneity, moderator analyses were not appropriate and tests of publication bias are not valid.

For peer review only - http://bmjopen.bmj.com/site/about/guidelines.xhtml

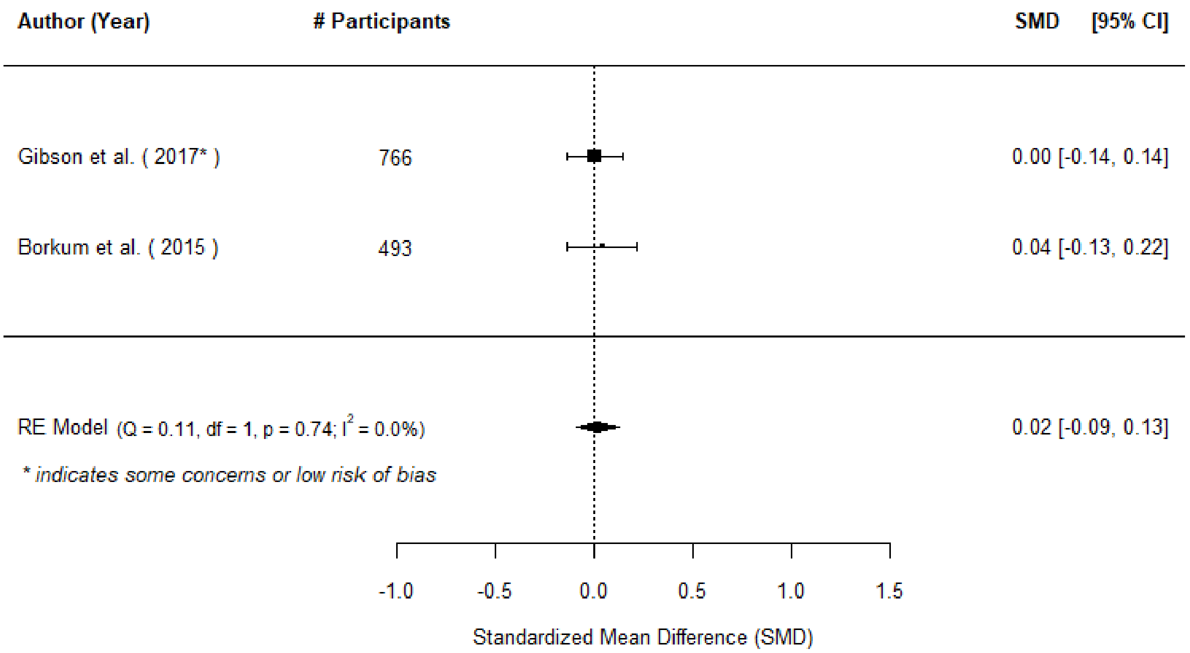

Supplementary Figure 51. Forest plot showing the observed outcomes and the estimate of the random-effects model for interventions with community engagement in the design of the intervention on BCG vaccination.

DPT1

Only two studies reporting on DPT1 vaccination used interventions with community engagement in the intervention design. The estimated average outcome based on the random-effects model was $\hat{\mu} = 0.03$ (95% CI: -0.08 to 0.14). Therefore, the average outcome did not differ significantly from zero ($z = 0.60$, $p = 0.55$, see Supplementary Figure 52), indicating no difference between the treatment group and the control group on DPT1 vaccination. Given the small number of studies, this result should be interpreted with caution. According to the Q-test, the true outcomes appear to be homogeneous ($Q(1) = 0.48$, $p = 0.49$, $\hat{\tau}^2 = 0.00$, $I^2 = 0.00\%$). With only two studies and no heterogeneity, moderator analyses were not appropriate and tests of publication bias are not valid.

For peer review only - http://bmjopen.bmj.com/site/about/guidelines.xhtml

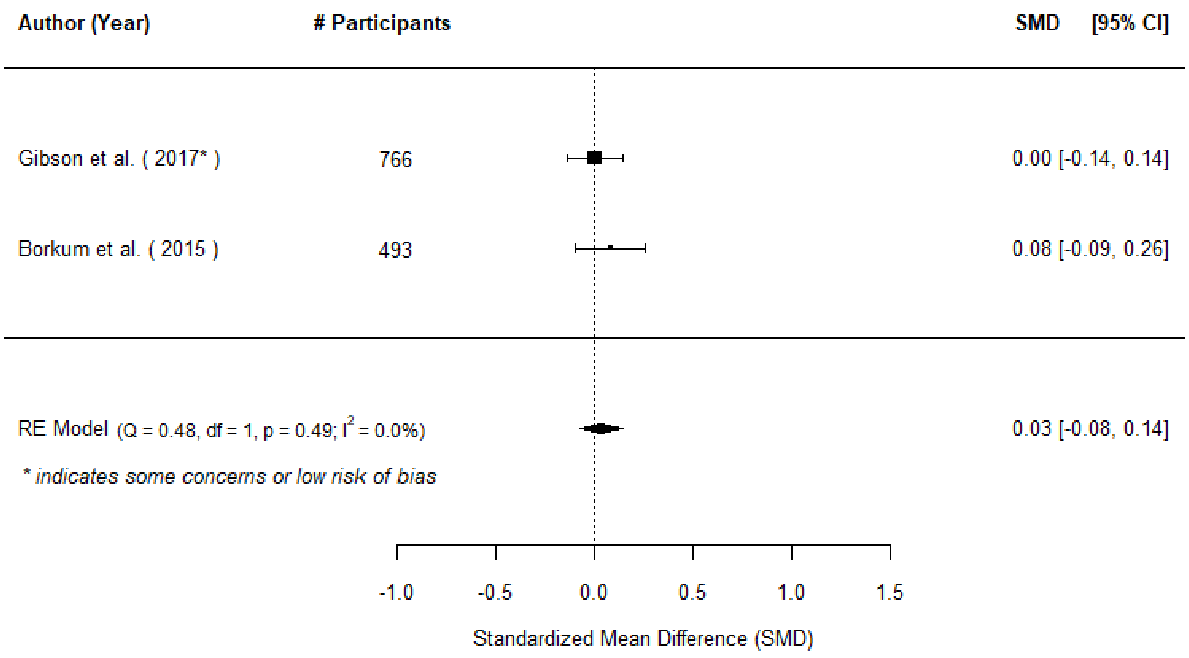

Supplementary Figure 52. Forest plot showing the observed outcomes and the estimate of the random-effects model for interventions with community engagement in the design on the intervention on DPT1 vaccination.

DPT2

Only two studies reporting on DPT2 vaccination used interventions with community engagement in the intervention design. The estimated average outcome based on the random-effects model was $\hat{\mu} =\ 0.05$ (95% CI: -0.06 to 0.16). Therefore, the average outcome did not differ significantly from zero ($z = 0.83$, $p = 0.41$, see Supplementary Figure 53), indicating no difference between the treatment group and the control group on DPT2 vaccination. Given the small number of studies, this result should be interpreted with caution. According to the Q-test, the true outcomes appear to be homogeneous ($\mathrm{Q(1)} = 0.07$, $\mathrm{p} = 0.41$, $\hat{\tau}^2 = 0.00$, $\mathrm{I}^2 = 0.00\%$). With only two studies and no heterogeneity, moderator analyses were not appropriate and tests of publication bias are not valid.

For peer review only - http://bmjopen.bmj.com/site/about/guidelines.xhtml

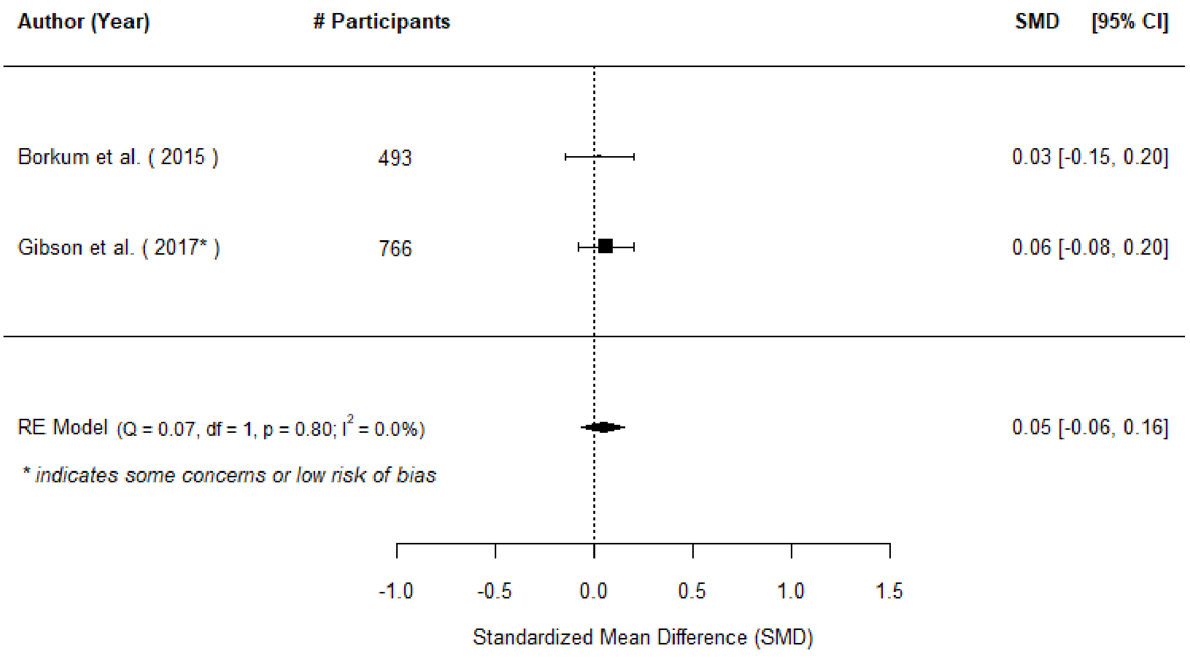

Supplementary Figure 53. Forest plot showing the observed outcomes and the estimate of the random-effects model for interventions with community engagement in the design on the intervention on DPT2 vaccination.

OPV0

Only two studies reporting on immunisation attitudes used interventions with community engagement in the intervention design. The estimated average outcome based on the random-effects model was $\hat{\mu} = 0.01$ (95% CI: -0.13 to 0.14). Therefore, the average outcome did not differ significantly from zero ($z = 0.08$, $p = 0.94$, see Supplementray Figure 54). Given the small number of studies, this result should be interpreted with caution. According to the Q-test, the true outcomes appear to be heterogeneous ($\mathrm{Q}(1) = 0.01$, $\mathrm{p} = 0.93$, $\hat{\tau}^2 = 0.00$, $\mathrm{I}^2 = 0.00\%$). With only two studies and no heterogeneity, moderator analyses were not appropriate and tests of publication bias are not valid.

For peer review only - http://bmjopen.bmj.com/site/about/guidelines.xhtml

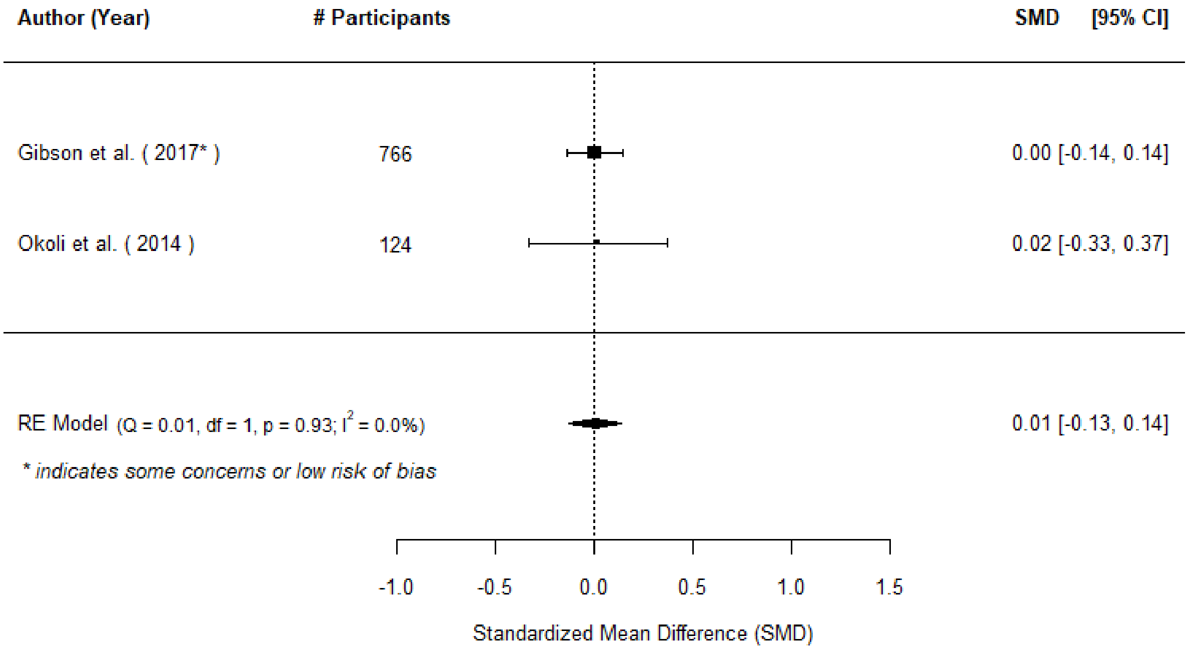

Supplementary Figure 54. Forest plot showing the observed outcomes and the estimate of the random-effects model for interventions with community engagement in the design on the intervention on OPV0 vaccination.

OPV1

Only two studies reporting on OPV1 vaccination used interventions with community engagement in the intervention design. The estimated average outcome based on the random-effects model was $\hat{\mu} = 0.08$ (95% CI: -0.03 to 0.19). Therefore, the average outcome did not differ significantly from zero ($z = 1.44$, $p = 0.15$, see Supplementary Figure 55). Given the small number of studies, this result should be interpreted with caution. According to the Q-test, the true outcomes appear to be homogeneous ($\mathrm{Q}(1) = 0.20, p = 0.65$, $\hat{\tau}^2 = 0.00$, $\mathrm{I}^2 = 0.00\%$). With only two studies, moderator analyses were not appropriate and tests of publication bias are not valid.

For peer review only - http://bmjopen.bmj.com/site/about/guidelines.xhtml

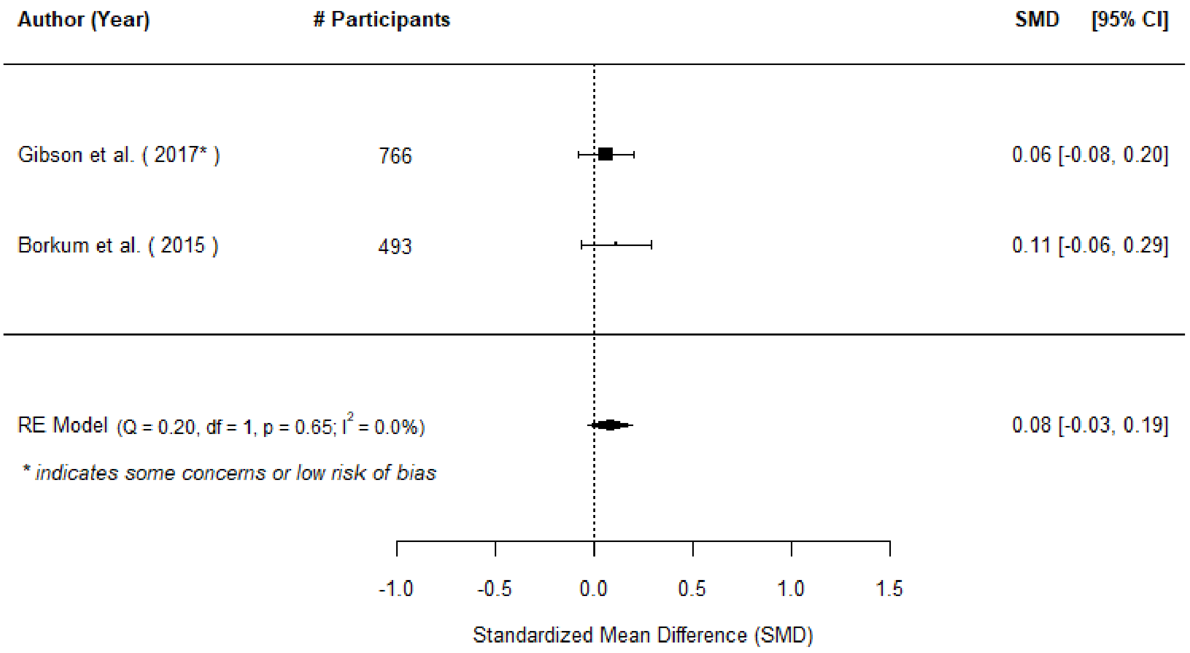

Supplementary Figure 55. Forest plot showing the observed outcomes and the estimate of the random-effects model for interventions with community engagement in the design on the intervention on OPV1 vaccination.

OPV2

Only two studies reporting on OPV2 vaccination used interventions with community engagement in the intervention design. The estimated average outcome based on the random-effects model was $\hat{\mu} = 0.23$ (95% CI: -0.09 to 0.55). Therefore, the average outcome did not differ significantly from zero ($z = 1.43$, $p = 0.15$, see Supplementray Figure 56), indicating no significant difference between the intervention group and the control group on OPV2 vaccinations. Given the small number of studies, this result should be interpreted with caution. According to the Q-test, the true outcomes appear to be heterogeneous ($Q(1) = 7.96, p = 0.004$, $\hat{\tau}^2 = 0.05$, $I^2 = 87.44\%$). With only two studies, moderator analyses were not appropriate and tests of publication bias are not valid.

For peer review only - http://bmjopen.bmj.com/site/about/guidelines.xhtml

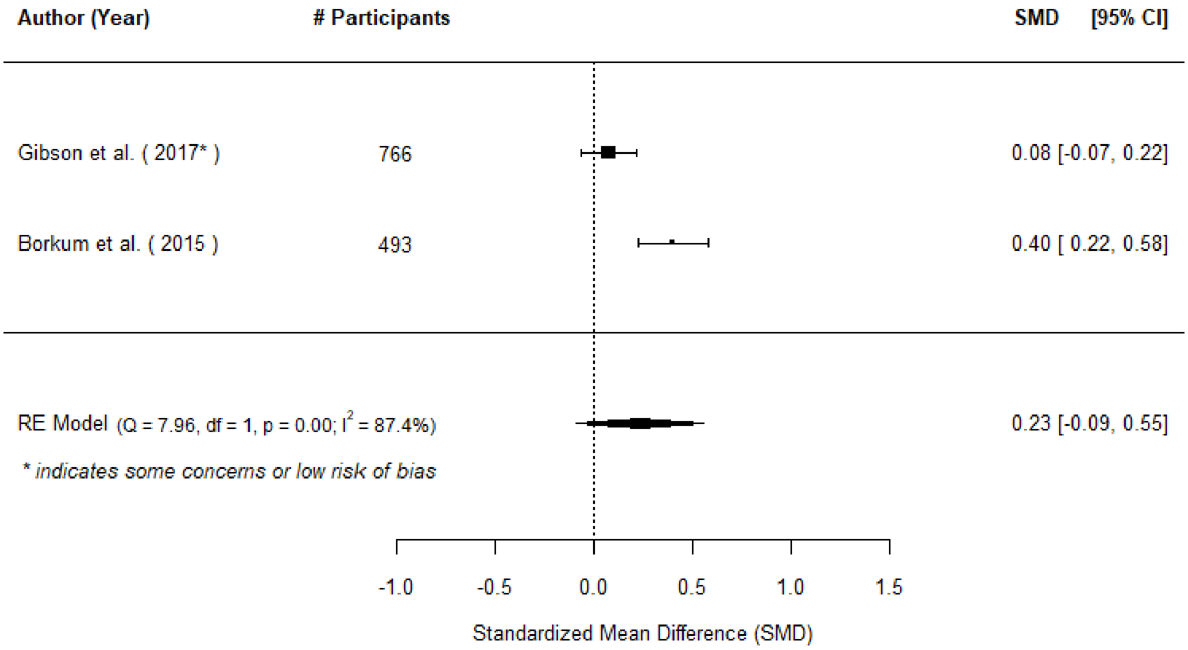

Supplementary Figure 56. Forest plot showing the observed outcomes and the estimate of the random-effects model for interventions with community engagement in the design on the intervention on OPV2 vaccination.

OPV3

Only one study (Borkum 2015) fell into this intervention/outcome category, thus we were unable to perform a statistical synthesis. This cluster RCT from India found a small but significant positive effect of their programme on OPV3 vaccination (g = 0.19 [95% CI: 0.01 to 0.36]), but again, it was assessed as having a high risk of bias.

Dropouts

There were no studies using engagement in the intervention design that reported on vaccination dropouts.

Morbidity

We used reports of diarrhea (most typically in the past two weeks) as a proxy for childhood morbidity. In all cases, effects were reverse coded such that positive effects always indicate a benefit to the treated group. Thus, a positive effect here would be interpreted as a reduction in diarrhea among treated participants compared to control participants. Only two studies reporting on childhood morbidity used interventions with community engagement in the intervention design. The estimated average outcome based on the random-effects model was $\hat{\mu} = 0.05$ (95% CI: -0.06 to 0.15). Therefore, the average outcome did not differ significantly from zero ($z = 0.88$, $p = 0.38$, see Supplementray Figure 57). Given the small number of studies, this result should be interpreted with caution. According to the Q-test, the true outcomes appear to be homogeneous ($\mathrm{Q}(1) = 1.84$, $\mathrm{p} = 0.17$, $\hat{\tau}^2 = 0.003$, $\mathrm{I}^2 = 45.71\%$). With only two studies, moderator analyses were not appropriate and tests of publication bias are not valid.

For peer review only - http://bmjopen.bmj.com/site/about/guidelines.xhtml

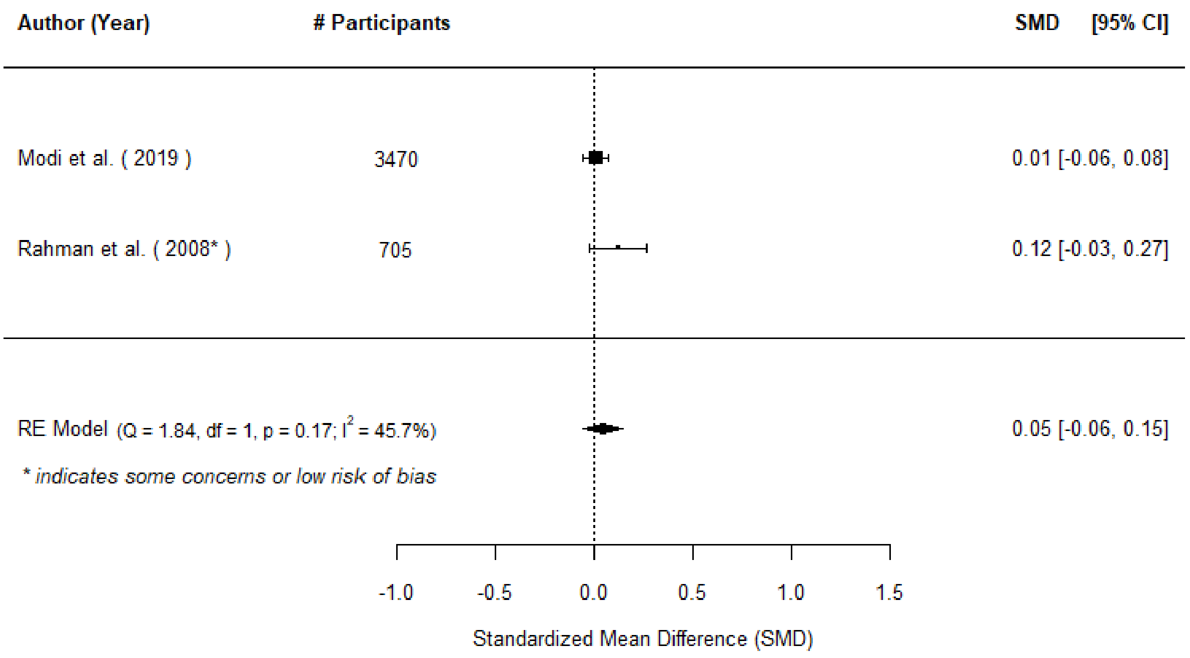

Supplementary Figure 57. Forest plot showing the observed outcomes and the estimate of the random-effects model for interventions with community engagement in the design on the intervention on childhood morbidity.

Mortality

There were no studies using engagement in the intervention design that reported on child mortality.

Immunisation knowledge

We included a total of $k = 3$ studies in the analysis. The estimated average outcome based on the random-effects model was $\hat{\mu} = 0.31$ (95% CI: 0.04 to 0.58). Therefore, the average outcome differed significantly from zero ($z = 2.26$, $p = 0.02$), indicating a moderate significant benefit to the intervention group compared to the control group (see Supplementary Figure 58). According to the Q-test, the true outcomes appear to be heterogeneous ($\mathrm{Q}(2) = 7.23$, $\mathrm{p} = 0.03$, $\hat{\tau}^2 = 0.04$, $\mathrm{I}^2 = 72.34\%$). An examination of the studentized residuals revealed that one study (Murthy et al. 2019) had a value larger than ± 2.39 and may be a potential outlier in the context of this model. Indeed, sensitivity analyses leaving each study out indicated that removing Murthy and colleagues (2019) would reduce the overall average effect ($\hat{\mu} = 0.19$ (95% CI: -0.03 to 0.41), and the effect would still be positive but would be non-significant ($z = 1.67$, $p = .09$). According to the Cook's distances, none of the studies could be considered to be overly influential. With only three studies, we were unable to test for sources of heterogeneity.

For peer review only - http://bmjopen.bmj.com/site/about/guidelines.xhtml

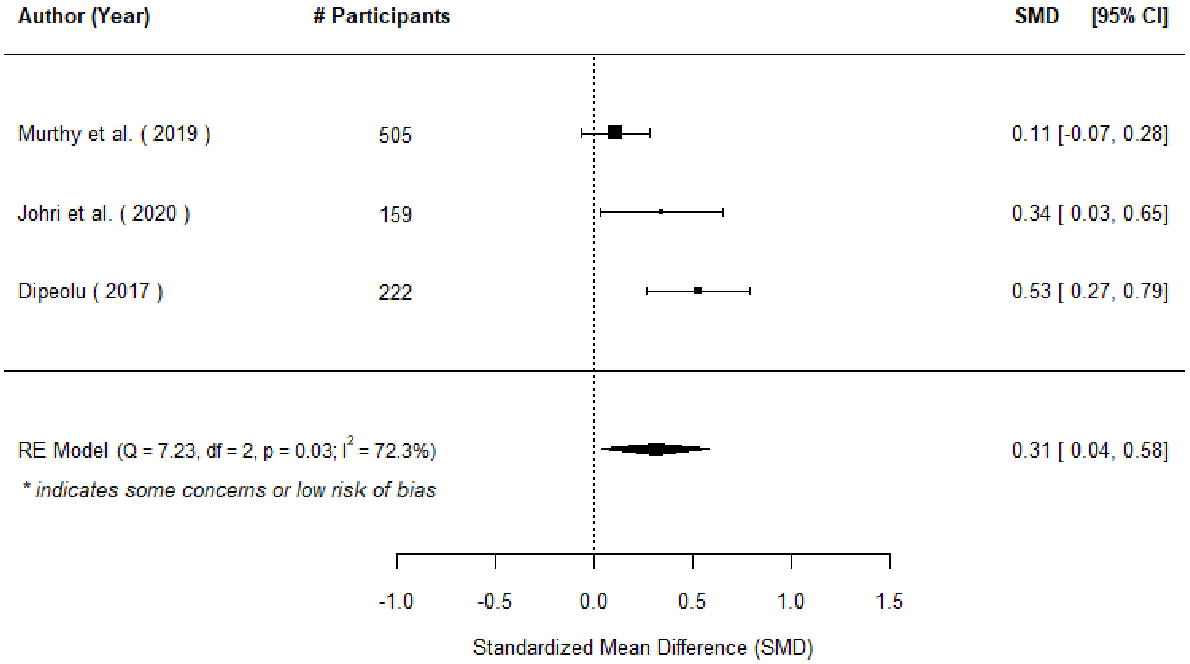

Supplementray Figure 58. Forest plot showing the observed outcomes and the estimate of the random-effects model for interventions with community engagement in the design on the intervention on childhood morbidity.

Immunisation attitudes

Only two studies reporting on immunisation attitudes used interventions with community engagement in the intervention design. The estimated average outcome based on the random-effects model was $\hat{\mu} = -0.11$ (95% CI: -0.53 to 0.30). Therefore, the average outcome did not differ significantly from zero ($z = -0.55$, $p = 0.58$, see Supplementary Figure 59). Given the small number of studies, this result should be interpreted with caution. According to the Q-test, the true outcomes appear to be heterogeneous ($\mathrm{Q}(1) = 4.10$, $\mathrm{p} = 0.04$, $\hat{\tau}^2 = 0.07$, $\mathrm{I}^2 = 75.61\%$). With only two studies, moderator analyses were not appropriate and tests of publication bias are not valid.

For peer review only - http://bmjopen.bmj.com/site/about/guidelines.xhtml

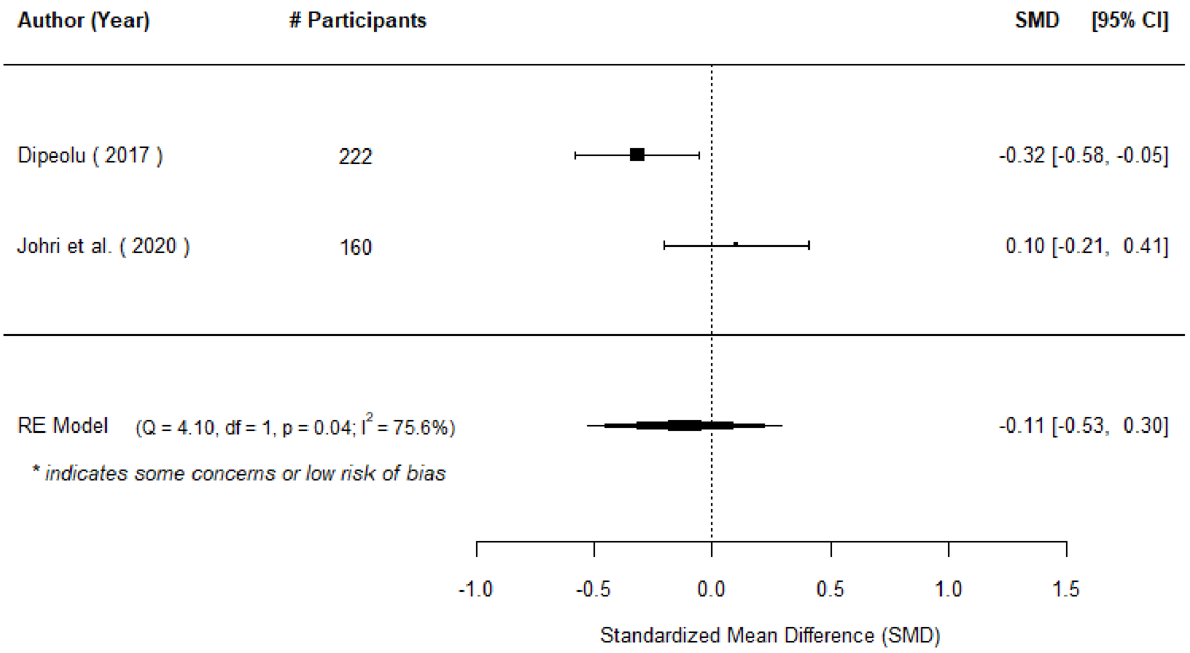

Supplementary Figure 59. Forest plot showing the observed outcomes and the estimate of the random-effects model for interventions with community engagement in the design on the intervention on immunisation attitudes.

Vaccination card retention

No studies reporting on vaccination card retention/availability used engagement in the intervention design.

Experience and satisfaction with health services

Only one study (Engineer 2016) fell into this intervention/outcome category, thus we were unable to perform a statistical synthesis. This cluster RCT from Afghanistan did not find a significant effect of their programme on satisfaction with health services (g = 0.20 [95% CI: -0.11 to 0.51]), but as with the other study reporting on this outcome, it was assessed as having a high risk of bias.

For peer review only - http://bmjopen.bmj.com/site/about/guidelines.xhtml

Appendix 12: Community engagement in implementation autonomy of interventions

Appendix 12 presents supplementary data for analyses presented in the main body of the manuscript for the primary outcomes of community engagement in implementation autonomy, including funnel plots of publication bias and outlier analyses. It also includes a full presentation of the supplementary outcomes that were precluded from the main body of the manuscript due to word limitations.

Full immunisation

Only k = 2 studies reported on the impact of interventions with community engagement in implementation autonomy on full childhood immunisation. There was no effect on full immunisation ($\hat{\mu} = 0.23$ [95% CI: -0.001 to $0.47], z = 1.95, p = 0.051$). According to the Q-test, the true outcomes appear to be homogeneous ($Q(1) = 3.71$, $p = 0.054$, $\hat{\tau}^2 = 0.02$, $I^2 = 73.07\%$, see Supplementray Figure 60). With only two studies, we could not test for moderation or publication bias, nor conduct sensitivity analysis by study quality.

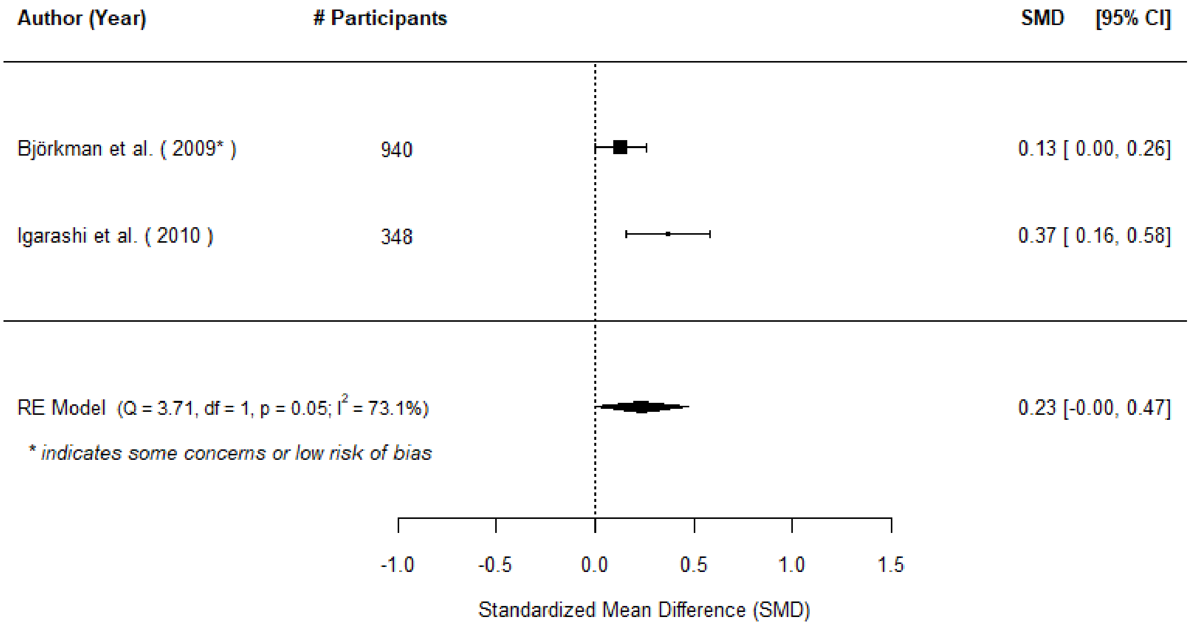

Supplementary Figure 60. Forest plot showing the observed outcomes and the estimate of the random-effects model for interventions with community engagement in implementation autonomy on full childhood immunisation.

DPT3

Only k = 3 studies examined the impact of interventions with community engagement in implementation autonomy on DPT3 vaccination. The average outcome based on the random-effects model indicated there was no effect on DPT3 vaccination ($\hat{\mu} = 0.11$ [95% CI: -0.05 to $0.28], z = 1.38, p = 0.17$). According to the Q-test, the true outcomes appear to be heterogeneous ($Q(2) = 10.06$, $p < 0.01$, $\hat{\tau}^2 = 0.02$, $I^2 = 80.12\%$). An examination of the studentized residuals revealed that one study (Webster 2019) had a value larger than ± 2.39 and may be a potential outlier in the context of this model. Indeed, sensitivity analyses leaving each study out indicated that removing Webster (2019) would increase the overall average effect ($\hat{\mu}$= 0.20 [95% CI: 0.10 to 0.29]), with the effect still positive and significant (z = 3.99, p < .001). According to the Cook's distances, none of the studies could be considered to be overly influential.

With only three studies, moderator analyses and tests of publication bias were not appropriate. With two of the three studies assessed as high risk of bias, we were also unable to conduct a sensitivity analysis by study quality.

For peer review only - http://bmjopen.bmj.com/site/about/guidelines.xhtml

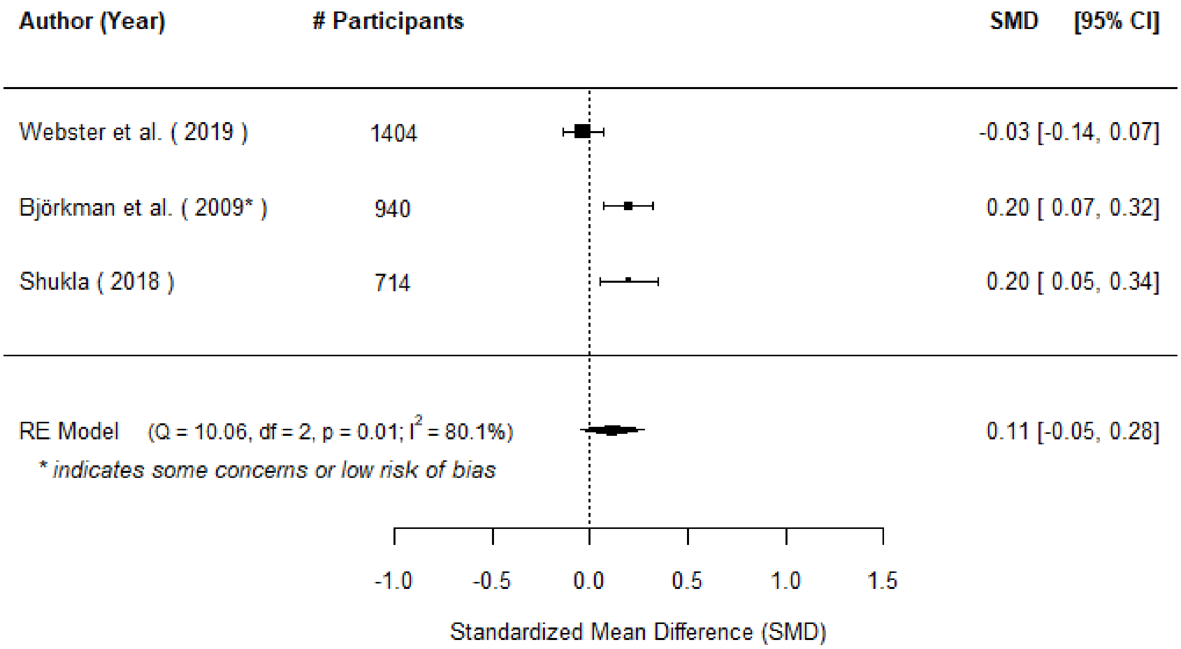

Supplementray Figure 61. Forest plot showing the observed outcomes and the estimate of the random-effects model for interventions with community engagement in implementation autonomy on DPT3 vaccination

Measles

Only $k = 2$ studies examined the impact of interventions with community engagement in implementation autonomy on measles vaccination. The average outcome based on the random-effects model indicated there was no effect on measles vaccination ($\hat{\mu} = 0.03$ [95% CI: -0.09 to 0.15], $z = 0.47$, $p = 0.64$). According to the Q-test, the true outcomes appear to be homogeneous ($Q(1) = 2.21$, $p = 0.14$, $\hat{\tau}^2 = 0.004$, $I^2 = 54.84\%$; see Supplementary Figure 62). With only two studies we were unable to test for publication bias.

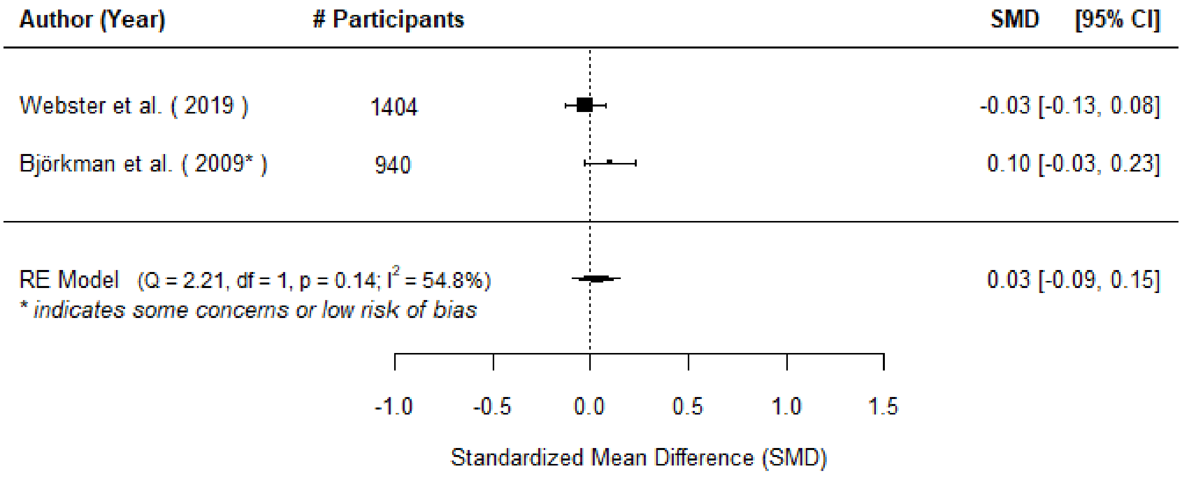

Supplementary Figure 62. Forest plot showing the observed outcomes and the estimate of the random-effects model for interventions with community engagement in implementation autonomy on measles vaccination

For peer review only - http://bmjopen.bmj.com/site/about/guidelines.xhtml

Timeliness of full childhood immunisation

Only k = 2 studies examined the impact of interventions with community engagement in implementation autonomy on the timeliness of full childhood immunisation. The average outcome based on the random-effects model indicated there was no effect on the timeliness of full childhood immunisation ($\hat{\mu} = 0.38$ [95% CI: -0.28 to 1.03], $z = 1.13$, $p = 0.26$). According to the Q-test, the true outcomes appear to be homogeneous ($Q(1) = 3.57$, $p = 0.06$, $\hat{\tau}^2 = 0.17$, $I^2 = 72.02\%$). There was no indication of outliers in the context of this model, and with only two studies, we could not test for moderation. One study was assessed as having a high risk of bias (Igarashi et al. 2010) while the other was assessed as some concerns related to risk of bias (Lee 2015).

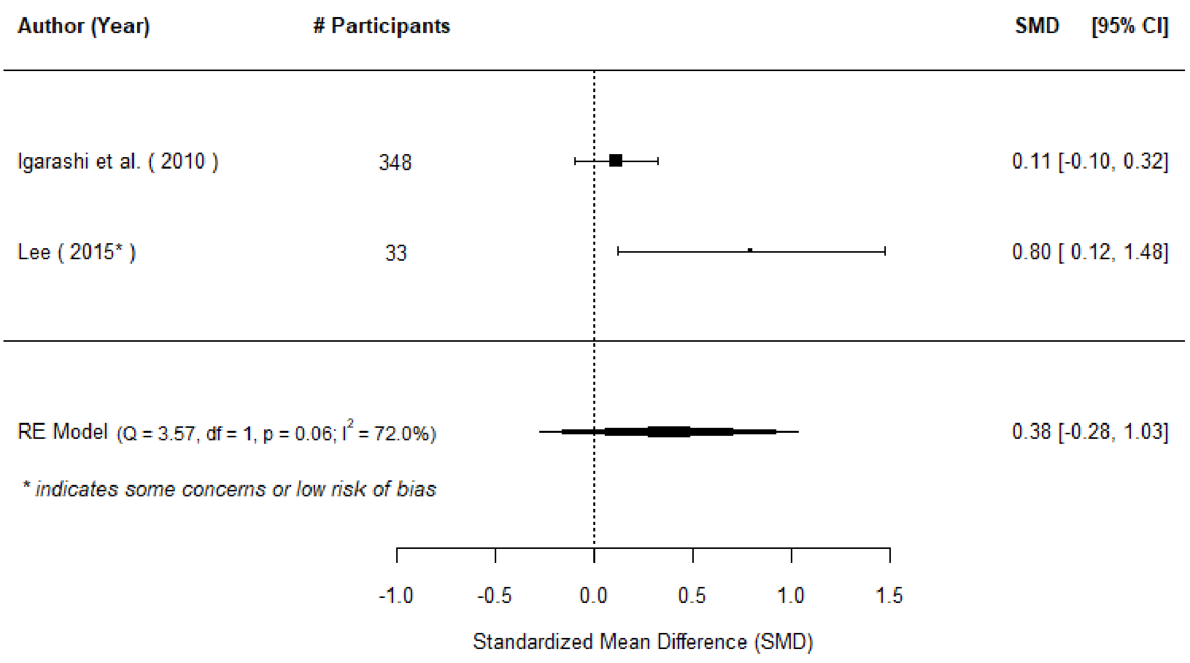

Supplementary Figure 63. Forest plot showing the observed outcomes and the estimate of the random-effects model for interventions with community engagement in implementation autonomy on DPT3 vaccination

DPT3 vaccination timeliness

Only k = 2 studies examined the impact of interventions with community engagement in implementation autonomy on DPT3 vaccination timeliness. The average outcome based on the random-effects model indicated there was no effect on the timeliness of DPT3 vaccination ($\hat{\mu} = 0.04$ [95% CI: -0.06 to 0.13], $z = 0.75$, $p = 0.45$). According to the Q-test, the true outcomes appear to be homogeneous ($Q(1) = 0.63$, $p = 0.45$, $\hat{\tau}^2 = 0.00$, $I^2 = 0.00\%$). There was no indication of outliers in the context of this model, and with only two studies, we could not test for moderation. Both studies were assessed as having a high risk of bias

For peer review only - http://bmjopen.bmj.com/site/about/guidelines.xhtml

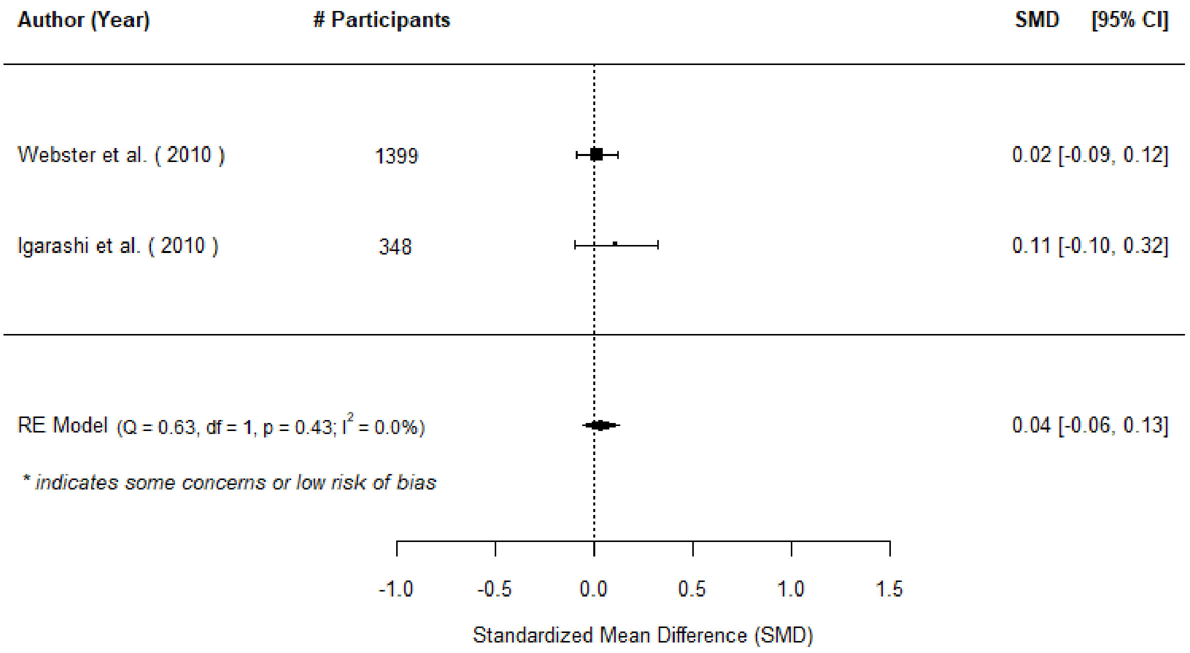

Supplementray Figure 64. Forest plot showing the observed outcomes and the estimate of the random-effects model for interventions with community engagement in implementation autonomy on DPT3 vaccination timeliness

Measles vaccination timeliness

No studies reporting on the timeliness of measles vaccinations used community engagement in implementation autonomy.

Secondary outcomes

Partial immunisation

Only one study (Webster et al. 2019) fell into this intervention/outcome category, thus we were unable to perform a statistical synthesis. This cluster RCT from Uganda found a null effect of their programme on partial childnood immunisation (*g* = 0.02 [95% CI: -0.08 to 0.13]), but like most studies, it was assessed as having a high risk of bias.

BCG

We included k = 2 studies in this analysis. The estimated average outcome was $\hat{\mu} = 0.03$ ([95% CI: -0.05 to 0.11], $z = 0.75$, $p = 0.46$) indicating no difference between the treated group and the untreated group (Supplementary Figure 62). According to the Q-test, the true outcomes appear to be homogeneous ($Q(1) = 0.37$, $p = 0.54$, $\hat{\tau}^2 = 0.00$, $I^2 = 00.00\%$). With only two studies and no heterogeneity, moderator analyses were not appropriate and tests of publication bias are not valid.

For peer review only - http://bmjopen.bmj.com/site/about/guidelines.xhtml

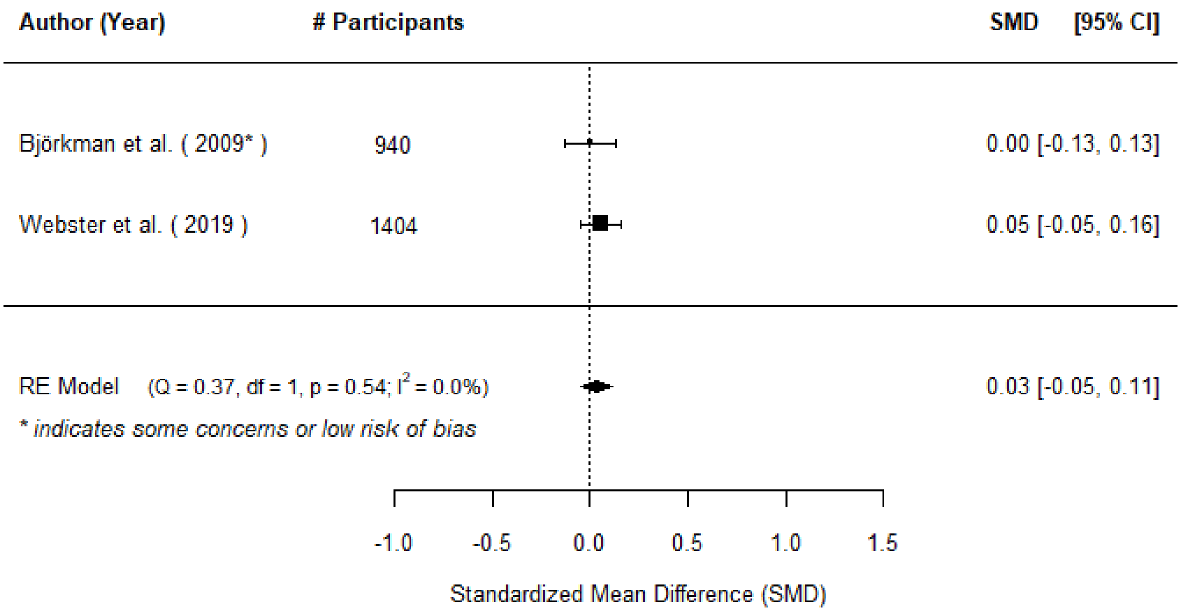

Supplementary Figure 65. Forest plot showing the observed outcomes and the estimate of the random-effects model for interventions with community engagement in implementation autonomy on BCG vaccination

DPT1

Only one study (Webster et al. 2019) fell into this intervention/outcome category, thus we were unable to perform a statistical synthesis. This cluster RCT from Uganda found a null effect of their programme on DPT1 vaccination (g = 0.04 [95% CI: -0.07 to 0.14]), but like most studies, it was assessed as having a high risk of bias.

DPT2

Only one study (Webster et al. 2019) fell into this intervention/outcome category, thus we were unable to perform a statistical synthesis. This cluster RCT from Uganda found a null effect of their programme on DPT1 vaccination (g = 0.01 [95% CI: -0.10 to 0.11]), but like most studies, it was assessed as having a high risk of bias.

OPV0

Only one study (Webster et al. 2019) fell into this intervention/outcome category, thus we were unable to perform a statistical synthesis. This cluster RCT from Uganda found a null effect of their programme on OPV0 vaccination (g = 0.01 [95% CI: -0.10 to 0.11]), but like most studies, it was assessed as having a high risk of bias.

OPV1

Only one study (Webster et al. 2019) fell into this intervention/outcome category, thus we were unable to perform a statistical synthesis. This cluster RCT from Uganda found a null effect of their programme on OPV1 vaccination (g = 0.03 [95% CI: -0.07 to 0.14]), but like most studies, it was assessed as having a high risk of bias.

For peer review only - http://bmjopen.bmj.com/site/about/guidelines.xhtml

OPV2

Only one study (Webster et al. 2019) fell into this intervention/outcome category, thus we were unable to perform a statistical synthesis. This cluster RCT from Uganda found a null effect of their programme on OPV2 vaccination (*g* = 0.04 [95% CI: -0.06 to 0.15]), but again, it was assessed as having a high risk of bias.

OPV3

Only two studies examined the impact of interventions with community engagement in implementation autonomy on OPV3 vaccination. The estimated average outcome was $\hat{\mu} = 0.03$ [95% CI: -0.12 to 0.18], which was not significant ($z = 0.35$, $p = 0.73$), indicating there was no difference between the intervention group and the control group. According to the Q-test, the true outcomes appear to be homogeneous ($Q(1) = 3.38$, $p = 0.07$, $\hat{\tau}^2 = 0.01$, $I^2 = 70.38\%$; see Supplementray Figure 66). With only two studies we were unable to test for publication bias or sources of heterogeneity.

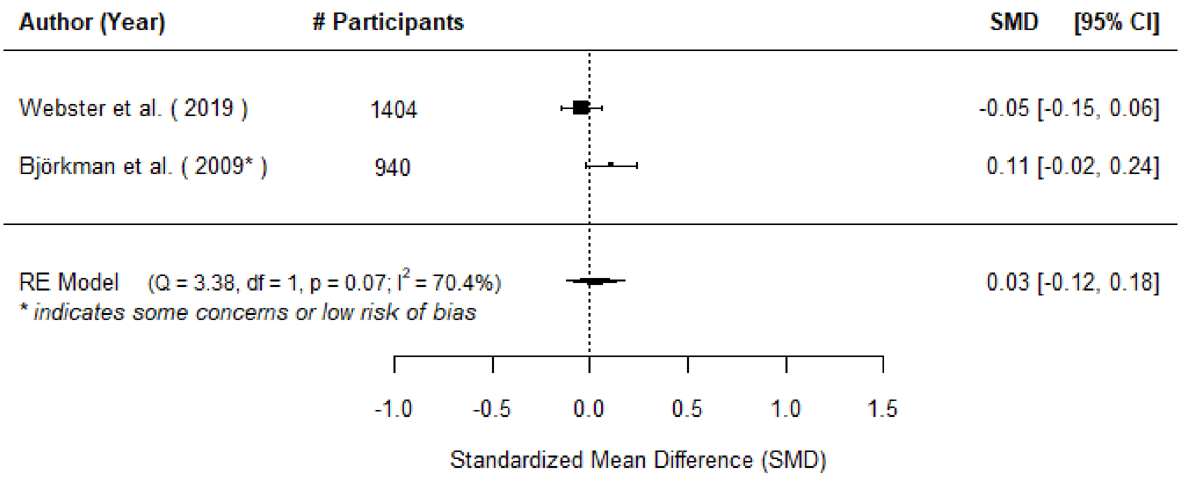

Supllementary Figure 66. Forest plot showing the observed outcomes and the estimate of the random-effects model for interventions with community engagement in implementation autonomy on OPV3 vaccinations.

Dropouts

Only one study (Webster et al. 2019) fell into this intervention/outcome category, thus we were unable to perform a statistical synthesis. This cluster RCT from Uganda found a small but significant negative impact of their programme on full childhood immunisation (*g* = -0.24 [95% CI: -0.35 to -0.14]), but like most studies, it was assessed as having a high risk of bias.

Morbidity

We used reports of diarrhea (most typically in the past two weeks) as a proxy for childhood morbidity. In all cases, effects were reverse coded such that positive effects always indicate a benefit to the treated group. Thus a positive

For peer review only - http://bmjopen.bmj.com/site/about/guidelines.xhtml

effect here would be interpreted as a reduction in diarrhea among treated participants compared to control participants. Only one study (Webster et al. 2019) fell into this intervention/outcome category, thus we were unable to perform a statistical synthesis. This cluster RCT from Uganda found a null effect of their programme on morbidity (g = 0.06 [95% CI: -0.02 to 0.13]), but like most studies, it was assessed as having a high risk of bias.

Mortality

There were no studies using community engagement in implementation autonomy that reported on child mortality.

Immunisation knowledge

There were no studies reporting on immunisation knowledge that used community engagement in implementation autonomy.

Immunisation attitudes

There were no studies reporting on immunisation attitudes that used interventions with community engagement in implementation autonomy.

Vaccination card retention

Only one study (Webster et al. 2019) fell into this intervention/outcome category, thus we were unable to perform a statistical synthesis. This cluster RCT from Uganda found a very small positive but non-significant effect of their programme on full childhood immunisation (g = 0.04 [95% CI: -0.06 to -0.15]), but like most studies, it was assessed as having a high risk of bias.

Experience and satisfaction with health services

No studies reporting on experience and satisfaction with health services used engagement in implementation autonomy.

Formal health worker's motivation, capacity and performance

No studies reporting on formal health worker's motivation, capacity and performance used engagement in implementation autonomy.

For peer review only - http://bmjopen.bmj.com/site/about/guidelines.xhtml

Appendix 13: Multiple engagement types

Full immunisation

We included k = 9 studies examining the impact of interventions with multiple engagement types on full childhood immunisation. The average outcome based on the random-effects model indicated that there was no effect on full childhood immunisation ($\hat{\mu} = 0.22$ [95% CI: -0.12 to 0.56], $z = 1.27$, $p = 0.20$). According to the Q-test, the true outcomes appear to be heterogeneous ($Q(8) = 388.56$, $p < 0.01$, $\hat{\tau}^2 = 0.25$, $I^2 = 97.94\%$, see Supplementary Figure 67). There was no indication of outliers in the context of this model, and no high or moderate quality studies, so we were unable to perform a sensitivity analysis. None of the moderators were significant sources of heterogeneity in the context of this model, including whether the specific combination of engagement types led to different effects (see Supplementary Table 3), but for full childhood immunisation we found no differences by engagement packages ($\hat{\beta} = 0.36$, p = 0.30 [95% CI: -0.31 to 1.02]).

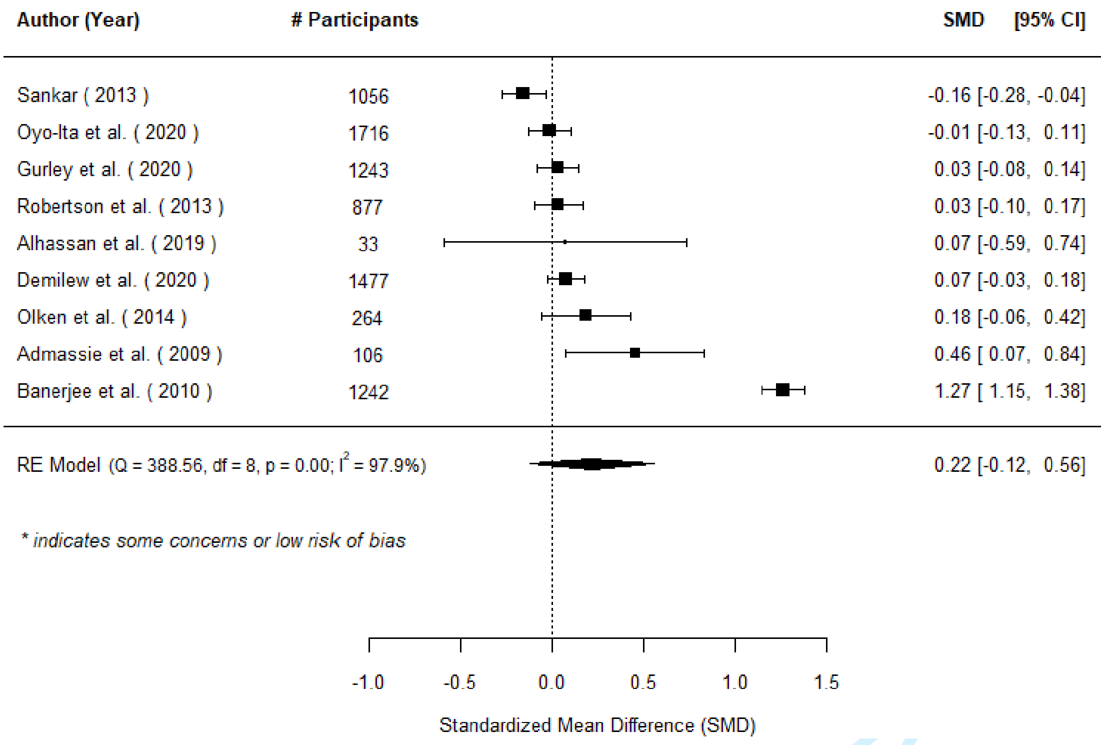

Supplementary Figure 67. Forest plot showing the observed outcomes and the estimate of the random-effects model for interventions with multiple engagement types on full immunisation

DPT3

We included k = 7 studies examining the impact of interventions with multiple engagement types on DPT3 vaccination. The average outcome based on the random-effects model indicated that there was a significant effect on DPT3 vaccination ($\hat{\mu} = 0.20$ *[95% CI:* 0.06 *to* 0.34], $z = 2.85$, $p < 0.01$). According to the Q-test, the true outcomes appear to be heterogeneous ($Q(6) = 49.71$, $p < 0.01$, $\hat{\tau}^2 = 0.03$, $I^2 = 87.93\%$). An examination of the studentized residuals revealed that none of the studies had a value larger than ± 2.69 and hence there was no indication of outliers in the context of this model. According to the Cook's distances, none of the studies could be considered to be overly influential. With no high or moderate quality studies, we were unable to perform a sensitivity analysis. There were several moderators that were significant sources of heterogeneity. Evaluation period was significant such that each additional month between the end of the intervention and the collection of outcome

For peer review only - http://bmjopen.bmj.com/site/about/guidelines.xhtml

data reduced the size of the effect by .01 standard deviation units ($\hat{\beta} = -0.01, p = 0.003$ [95% CI: -0.02 to -0.004]), suggesting smaller long term effect of the interventions. Publication year was also significant, such that each additional year reduced the size of the effect by .03 standard deviation units ($\hat{\beta} = -0.03, p = 0.03$ [95% CI: -0.05 to -0.003]). In other words, more recent studies have found smaller effects. Finally, baseline DPT3 coverage rates were significant such that a one unit increase in baseline DPT3 coverage was associated with a decrease of .56 in the effect of the programme ($\hat{\beta} = -0.56, p = 0.002$ [95% CI: -0.90 to -0.21]). In other words, the programmes were significantly more effective in areas with lower baseline coverage rates. We also tested whether the specific combination of engagement types led to different effects, but for DPT3 vaccination we found no differences by engagement packages ($\hat{\beta} = -0.07, p = 0.66$ [95% CI: -0.40 to -0.25]).

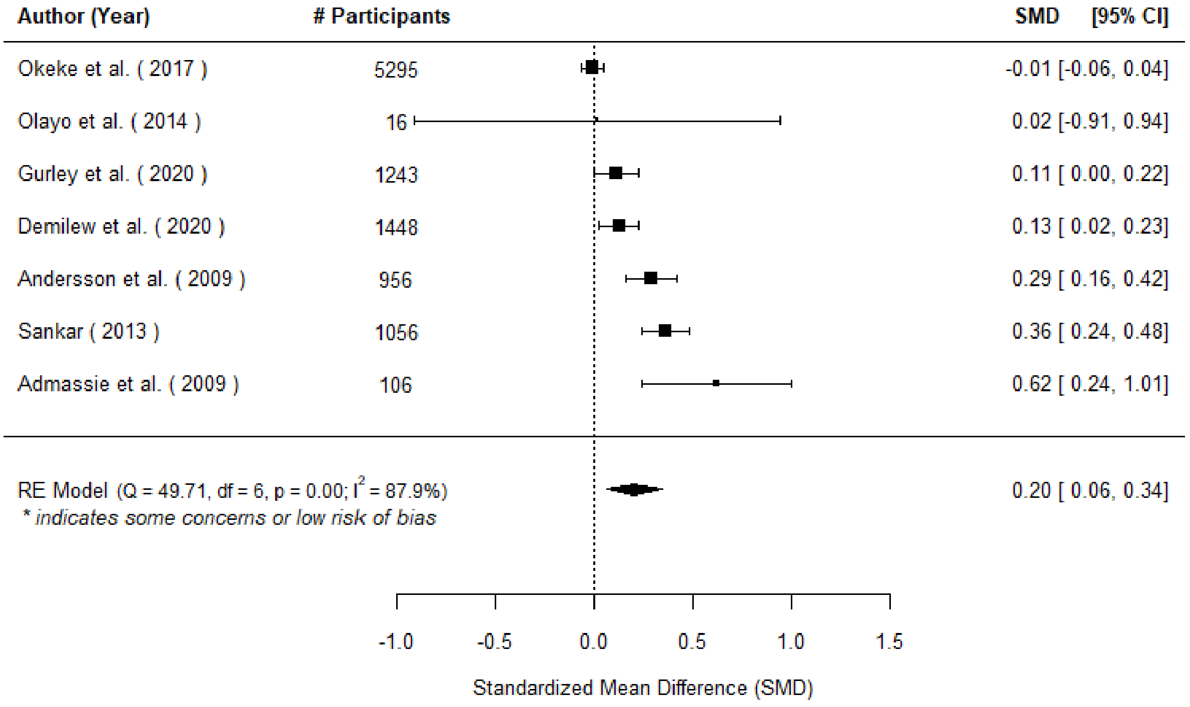

Supplementary Figure 68. Forest plot showing the observed outcomes and the estimate of the random-effects model for interventions with multiple engagement types on DPT3 vaccination

Measles

We included k = 6 studies examining the impact of interventions with multiple engagement types on measles vaccination. The average outcome based on the random-effects model indicated that there was no effect on measles coverage ($\hat{\mu} = 0.03$ [95% CI: -0.10 to 0.16], $z = 0.44$, $p = 0.66$). According to the Q-test, the true outcomes appear to be heterogeneous ($Q(5) = 37.76$, $p < 0.01$, $\hat{\tau}^2 = 0.02$, $I^2 = 86.76\%$; see Supplementary Figure 69). An examination of the studentized residuals revealed that one study (Sankar 2013) had a value larger than ± 2.64 and may be a potential outlier in the context of this model. According to the Cook's distances, none of the studies could be considered to be overly influential. Indeed, sensitivity analyses leaving each study out indicated that removing Sankar (2013) would increase the overall average effect ($\hat{\mu} = 0.09$ (95% CI: -0.02 to 0.19), but the effect would still be non-significant ($z = 1.58$ $p = 0.11$). With only one high or medium quality study, we were unable to conduct sensitivity analysis by study quality. None of the moderators that could be tested were significant in the context of this model (see Supplementary Table 3). For measles vaccinations, we were unable to test whether different combinations of community engagement produced different results because all but one study used a combination of engagement in the implementation and engagement as the intervention.

For peer review only - http://bmjopen.bmj.com/site/about/guidelines.xhtml

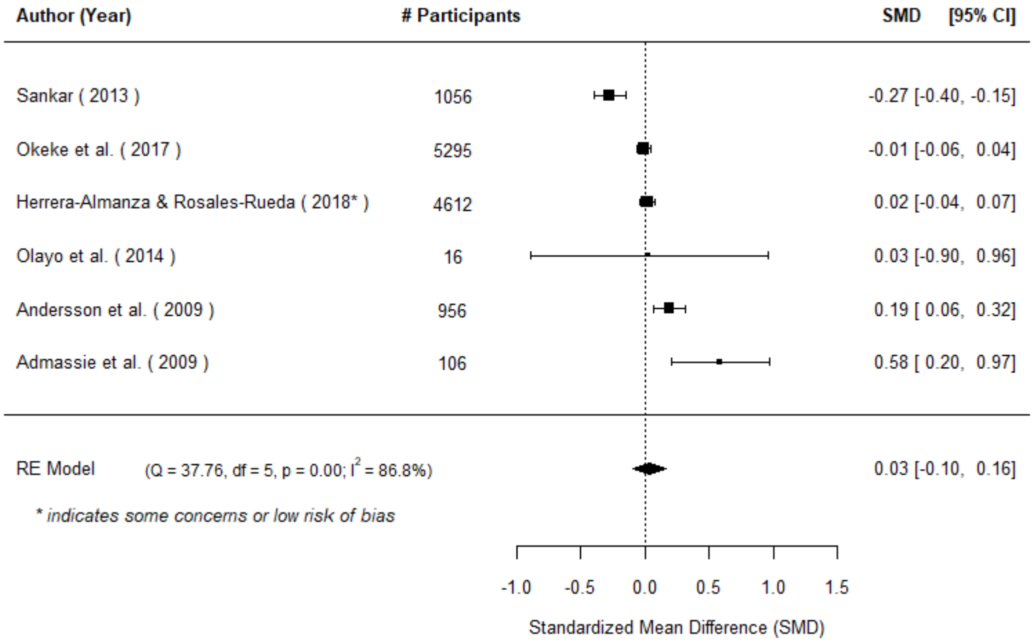

Supplementary Figure 69. Forest plot showing the observed outcomes and the estimate of the random-effects model for interventions with multiple engagement types on measles vaccination

Timeliness of full childhood immunisation

There were no studies using multiple engagement types that reported on the timeliness of full childhood immunisation.

Timeliness of DPT3 vaccination

Only one study reporting on timeliness of DPT3 vaccination used an intervention with multiple engagement types (Oyo-Ita et al. 2020). This RCT conducted in Nigeria found a small but not-significant positive effect (g = 0.10 [95%CI; -.0.2 to 0.23).This study was assessed as having a high risk of bias.

Timeliness of measles vaccination

Finally, only one study reporting on timeliness of measles vaccination used an intervention with multiple engagement types (Oyo-Ita et al. 2020). This RCT conducted in Nigeria found a moderate and significant positive effect (g = 0.25 [95% CI; 0.13 to 0.36]). They used a combination of engagement in implementation autonomy and engagement as the intervention. This study was also assessed as having a high risk of bias.

Secondary outcomes

Partial immunisation

We included a total of $k = 4$ studies in the analysis. The estimated average outcome based on the random-effects model was $\hat{\mu} = 0.28$ (95% CI: -0.01 to 0.56). Therefore, the average outcome did not differ significantly from zero ($z = 1.91$, $p = 0.06$), indicating no difference between the intervention and control groups (see Supplementary Figure 70). According to the Q-test, the true outcomes appear to be heterogeneous ($Q(3) = 101.75$, $p < 0.01$, $\hat{\tau}^2 = 0.08$, $I^2 = 97.05\%$). An examination of the studentized residuals revealed that none of the studies had a value

For peer review only - http://bmjopen.bmj.com/site/about/guidelines.xhtml

larger than ± 2.50 and hence there was no indication of outliers in the context of this model. According to the Cook's distances, none of the studies could be considered to be overly influential. Of the moderators we were able to test, none were significant sources of heterogeneity (see Supplementary Table 3). We were unable to test for differences among engagement packages because only one study used a combination of engagement in the design and engagement as the intervention (Gurley et al. 2020) while the remaining studies used a combination of engagement in implementation autonomy and engagement as the intervention.

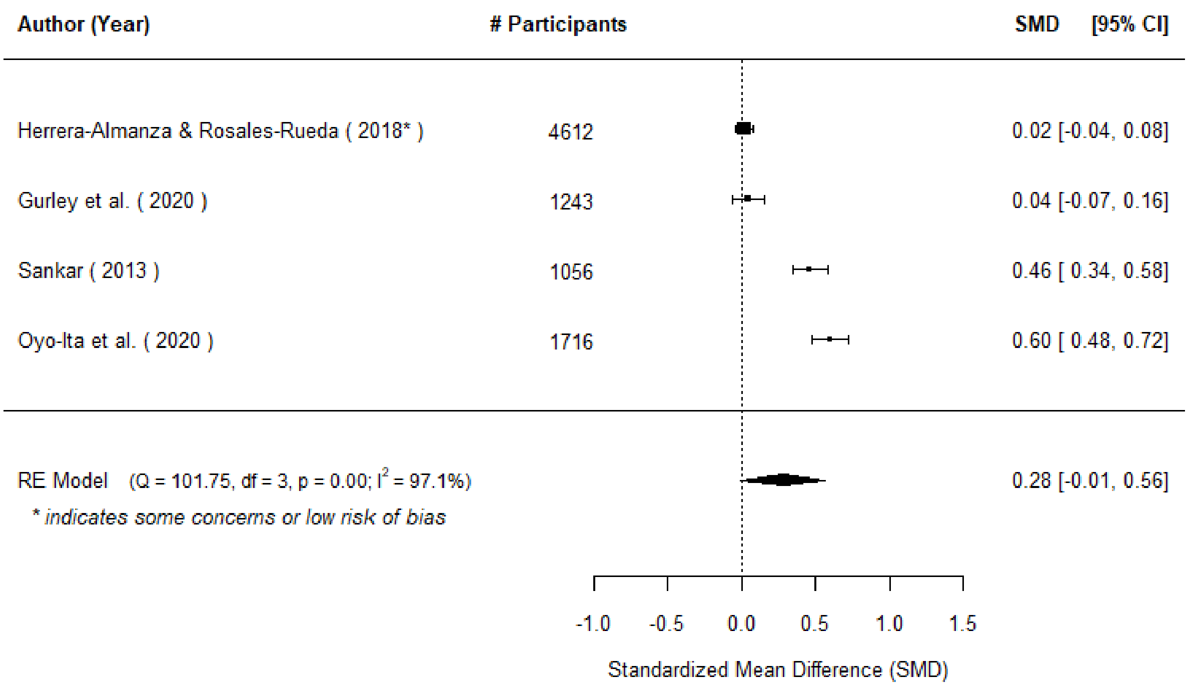

Supplementary Figure 70. Forest plot showing the observed outcomes and the estimate of the random-effects model for interventions with multiple engagement types on partial immunisation.

BCG

We included a total of $k = 4$ studies in the analysis. The estimated average outcome based on the random-effects model was $\hat{\mu} = 0.22$ (95% CI: -0.07 to 0.52). Therefore, the average outcome did not differ significantly from zero ($z = 1.48$, $p = 0.14$), indicating no differences between the treatment and control groups (see Supplementary Figure 71). According to the Q-test, the true outcomes appear to be heterogeneous ($Q(3) = 75.36$, $p < 0.01$, $\hat{\tau}^2 = 0.08$, $I^2 = 96.02\%$). An examination of the studentized residuals revealed that one study (Banerjee 2010) had a value larger than ± 2.50 and may be a potential outlier in the context of this model. Indeed, sensitivity analyses leaving each study out indicated that removing Banerjee (2010) would reduce the overall average effect ($\hat{\mu} = 0.05$ (95% CI: -0.13 to 0.23), but the effect is still positive and non-significant ($z = 0.56$, $p = 0.58$). According to the Cook's distances, none of the studies could be considered to be overly influential. With all studies assessed as high risk of bias, we were unable to conduct sensitivity analysis by study quality. We tested all moderators and only post-intervention versus change from baseline was a significant predictor of BCG vaccination, such that studies examining change from baseline had higher effects than studies examining post-intervention changes by .55 standard deviation units ($\hat{\beta} = -0.55$ [95% CI: -0.69 to -0.404], $p <$ .001). We could not test for moderation by engagement components because all but one study used a combination of engagement in implementation autonomy and engagement as the intervention.

For peer review only - http://bmjopen.bmj.com/site/about/guidelines.xhtml

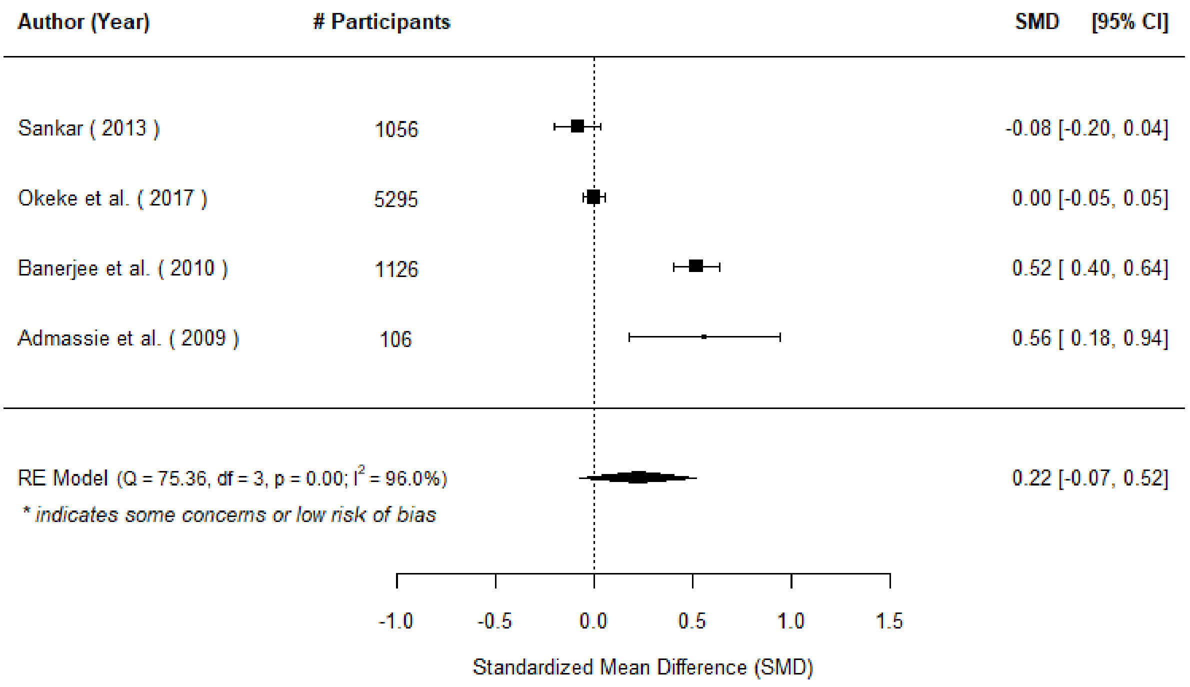

Supplemenatary Figure 71. Forest plot showing the observed outcomes and the estimate of the random-effects model for interventions with multiple engagement types on BCG vaccination.

DPT1

Only two studies reporting on DPT1 vaccination used interventions with multiple engagement types. The estimated average outcome based on the random-effects model was $\hat{\mu} = -0.17$ ([95% CI: -0.29 to -0.05], $z = -2.81$, $p = 0.005$), thus there was a significant negative impact of the programmes on DPT1 vaccination, (see Supplementary Figure 72). Given the small number of studies, this result should be interpreted with caution. According to the Q-test, the true outcomes appear to be homogeneous ($Q(1) = 0.03, p = 0.87$, $\hat{\tau}^2 = 0.00$, $I^2 = 0.00\%$). With only two studies, moderator analyses were not appropriate and tests of publication bias are not valid.

For peer review only - http://bmjopen.bmj.com/site/about/guidelines.xhtml

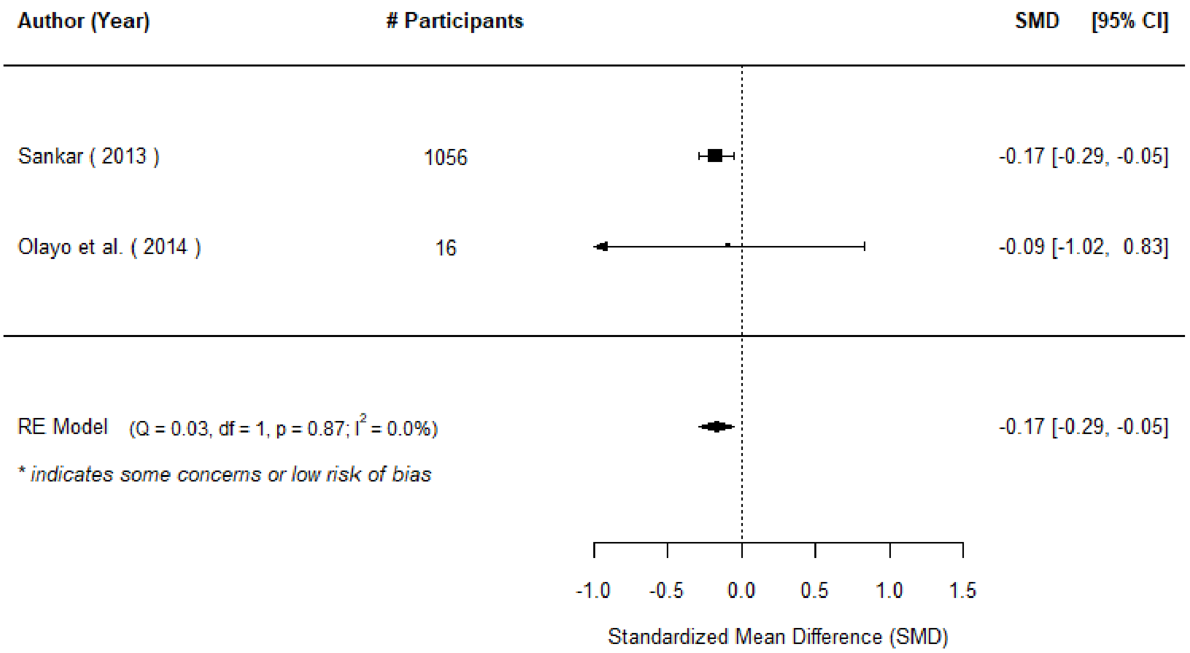

Supplementary Figure 72. Forest plot showing the observed outcomes and the estimate of the random-effects model for interventions with multiple engagement types on DPT1 vaccination.

DPT2

Only one study (Sankar 2013) fell into this intervention/outcome category, thus we were unable to perform a statistical synthesis. This quasi-experimental study from India found a null effect of their programme on OPV0 vaccination (g = 0.10 [95% CI: -0.02 to 0.22]), but like most studies, it was assessed as having a high risk of bias. Their programme included a combination of engagement in the implementation and engagement as the intervention.

OPV0

Only one study (Sankar 2013) fell into this intervention/outcome category, thus we were unable to perform a statistical synthesis. This quasi-experimental study from India found a significant positive effect of their programme on OPV0 vaccination (g = 0.43 [95% CI: 0.31 to 0.55]), but like most studies, it was assessed as having a high risk of bias. Their programme included a combination of engagement in implementation autonomy and engagement as the intervention.

OPV1

Only two studies reporting on OPV1 vaccination used interventions with multiple engagement types. The estimated average outcome based on the random-effects model was $\hat{\mu} = 0.22$ (95% CI: -0.15 to 0.59). Therefore, the average outcome did not differ significantly from zero ($z = 1.17$, $p = 0.24$, see Supplementary Figure 73). Given the small number of studies, this result should be interpreted with caution. According to the Q-test, the true outcomes appear to be homogeneous ($\mathrm{Q}(1) = 3.61$, $p = 0.06$, $\hat{\tau}^2 = 0.05$, $\mathrm{I}^2 = 72.32\%$). With only two studies, moderator analyses were not appropriate and tests of publication bias are not valid.

For peer review only - http://bmjopen.bmj.com/site/about/guidelines.xhtml

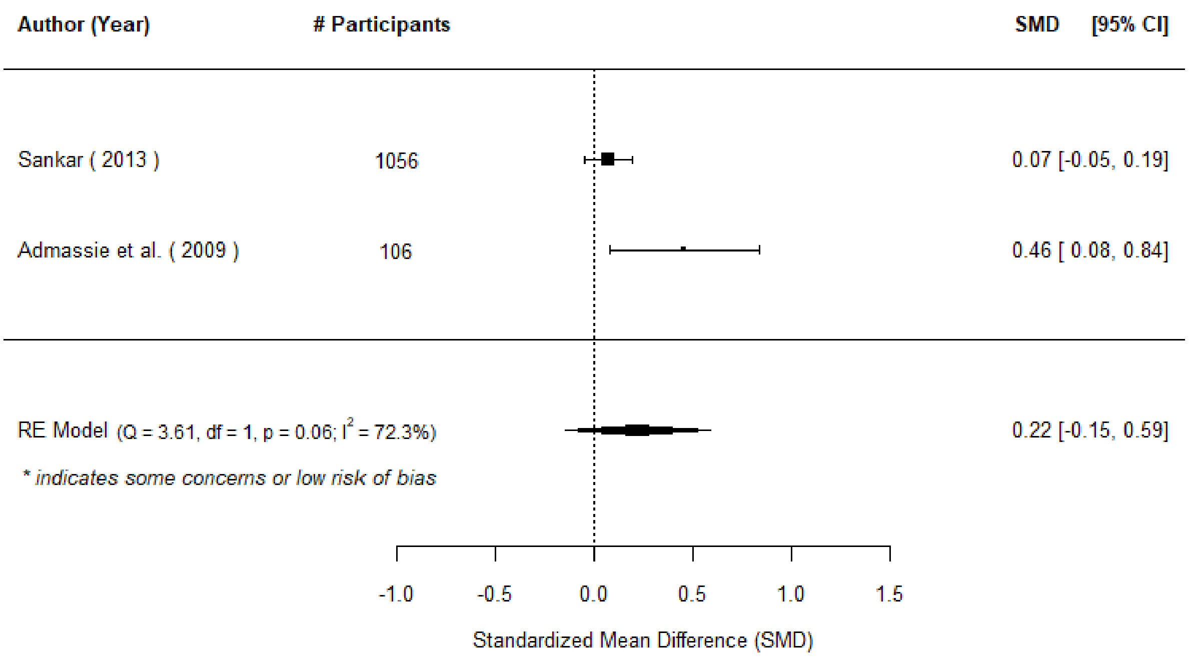

Supplementray Figure 73. Forest plot showing the observed outcomes and the estimate of the random-effects model for interventions with multiple engagement types on OPV1 vaccination.

OPV2

Only two studies reporting on OPV2 vaccination used interventions with multiple engagement types. The estimated average outcome based on the random-effects model was $\hat{\mu} = 0.34$ (95% CI: 0.19 to $0.50, z = 4.28$, $p < 0.001$), indicating a small but significant benefit to the treated group compared to the control participants (see Supplementary Figure 74). Given the small number of studies, this result should be interpreted with caution. According to the Q-test, the true outcomes appear to be homogeneous ($Q(1) = 1.18, p = 0.28$, $\hat{\tau}^2 = 0.004$, $I^2 = 15.51\%$). With only two studies, moderator analyses were not appropriate and tests of publication bias are not valid. Both studies used a combination of engagement in implementation autonomy and engagement as the intervention.

For peer review only - http://bmjopen.bmj.com/site/about/guidelines.xhtml

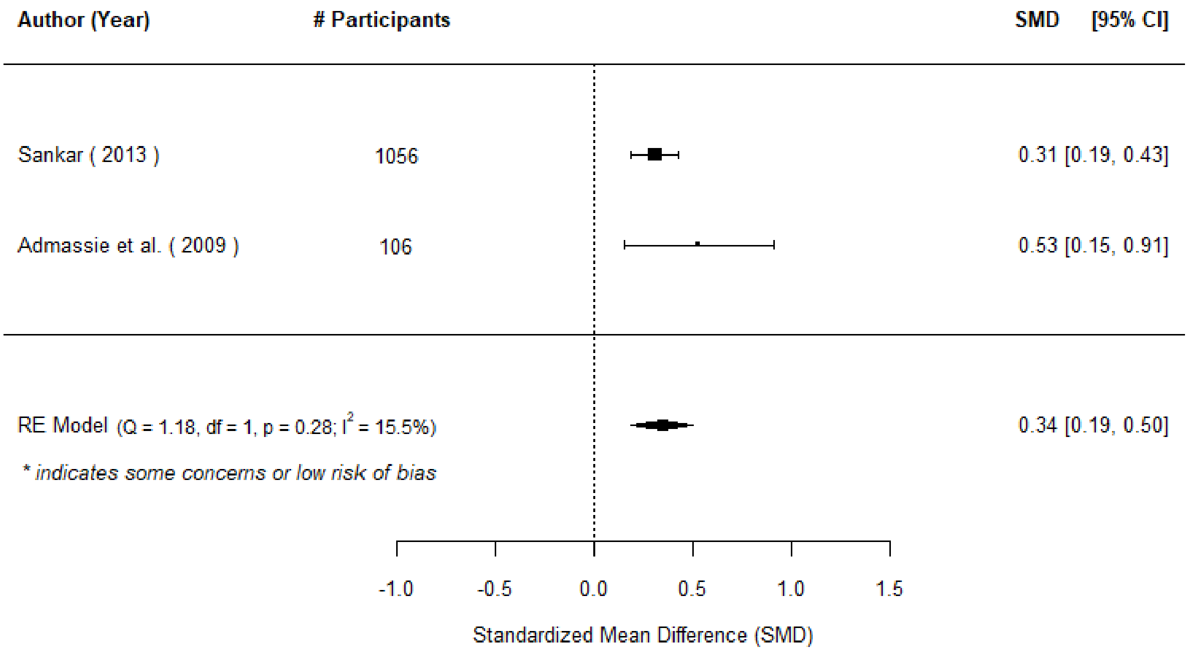

Supplementary Figure 74. Forest plot showing the observed outcomes and the estimate of the random-effects model for interventions with multiple engagement types on OPV2 vaccination.

OPV3

We included a total of $k = 3$ studies in the analysis. The estimated average outcome based on the random-effects model was $\hat{\mu} = 0.48$ ([95% CI: -0.24 to 1.20], $z = 1.30$, $p = 0.19$), indicating no difference between the intervention group and the control group (see Supplementary Figure 75). According to the Q-test, the true outcomes appear to be heterogeneous ($Q(2) = 185.96$, $p < 0.001$, $\hat{\tau}^2 = 0.39$, $I^2 = 98.92\%$). An examination of the studentized residuals revealed that none of the studies had a value larger than ± 2.39 and hence there was no indication of outliers in the context of this model. According to the Cook's distances, none of the studies could be considered to be overly influential. With only three studies contributing effects, we were unable to examine sources of heterogeneity.

For peer review only - http://bmjopen.bmj.com/site/about/guidelines.xhtml

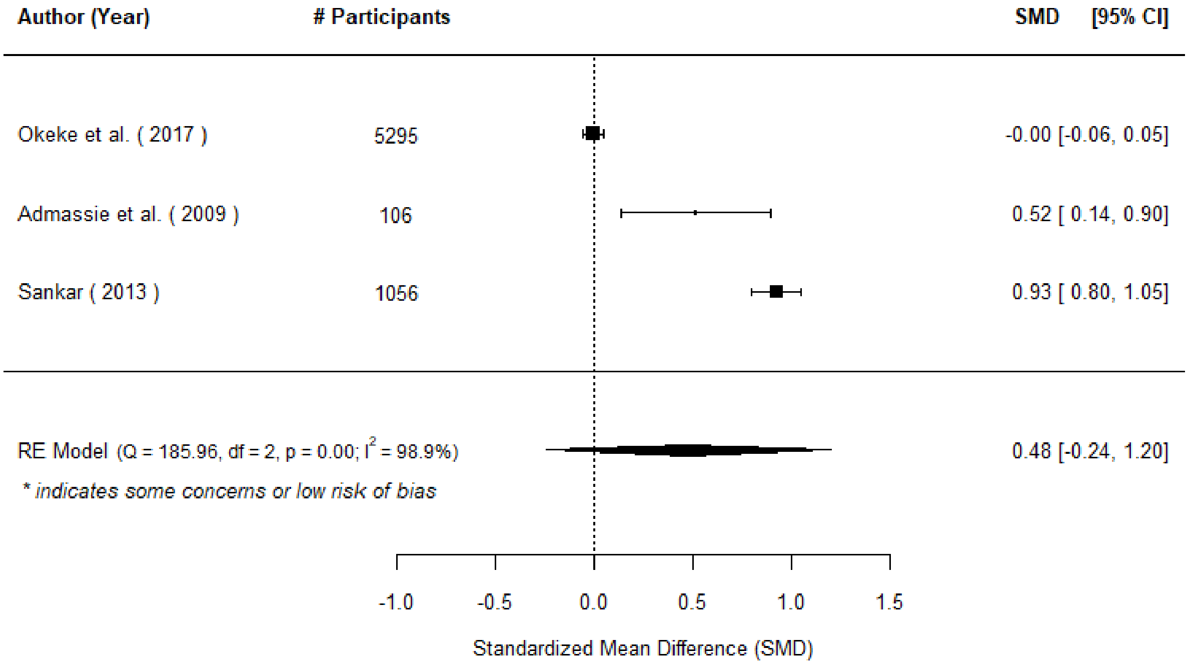

Supplementary Figure 75. Forest plot showing the observed outcomes and the estimate of the random-effects model for interventions with multiple engagement types on OPV3 vaccination.

Dropouts

Only two studies reporting on immunisation dropouts used interventions with multiple engagement types. All effects were computed such that a positive effect reflects a benefit to the treated group (e.g. a reduction in dropouts). The estimated average outcome based on the random-effects model was $\hat{\mu} =$ 0.18 (95% CI: 0.06 to 0.30). Therefore, the average outcome differed significantly from zero, indicating a small but significant benefit to the treated group compared to the control group ($z = 2.92$, $p = 0.004$, see Supplementary Figure 76). Given the small number of studies, this result should be interpreted with caution. According to the Q-test, the true outcomes appear to be homogeneous ($Q(1) = 2.35$, $p = 0.13$, $\hat{\tau}^2 = 0.004$, $I^2 = 57.38\%$). With only two studies, moderator analyses were not appropriate and tests of publication bias are not valid.

For peer review only - http://bmjopen.bmj.com/site/about/guidelines.xhtml

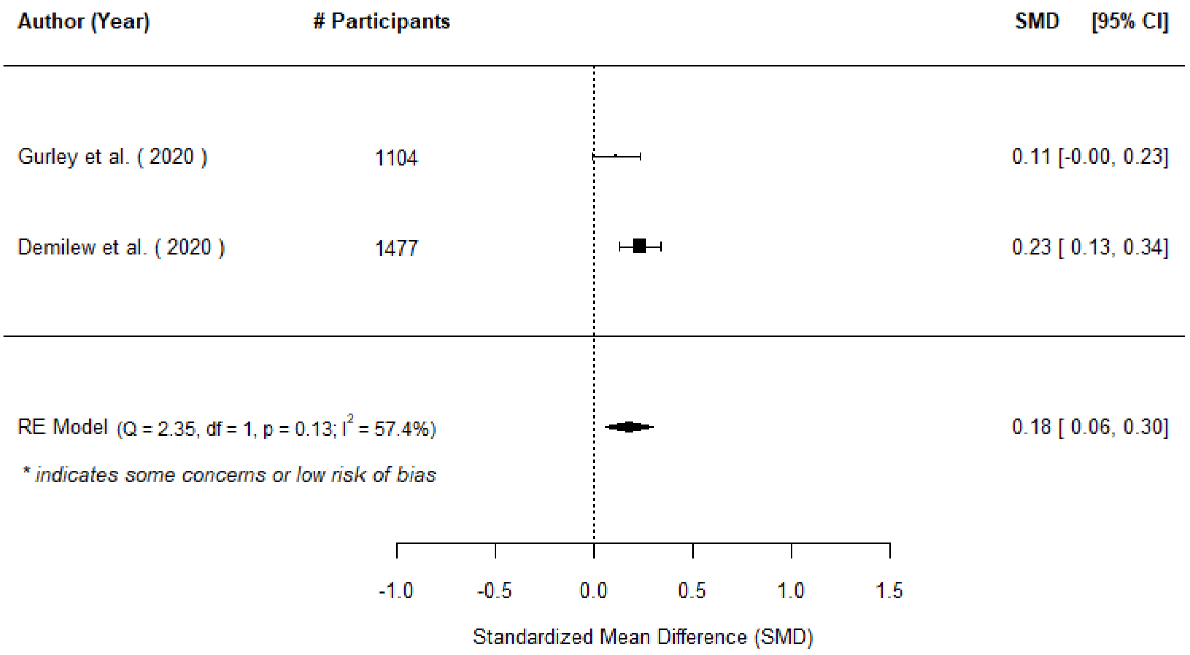

Supplementary Figure 76. Forest plot showing the observed outcomes and the estimate of the random-effects model for interventions with multiple engagement types on vaccination dropouts.

Morbidity

We used reports of diarrhea (most typically in the past two weeks) as a proxy for childhood morbidity. In all cases, effects were reverse coded such that positive effects always indicate a benefit to the treated group. Thus a positive effect here would be interpreted as a reduction in diarrhea among treated participants compared to control participants. Only two studies reporting on childhood morbidity used interventions with multiple engagement types. The estimated average outcome based on the random-effects model was $\hat{\mu} = -0.10$ (95% CI: -0.60 to 0.40). Therefore, the average outcome did not differ significantly from zero, indicating no difference between the treated group compared to the control group ($z = -0.38$, $p = 0.70$, see Supplementary Figure 77). Given the small number of studies, this result should be interpreted with caution. According to the Q-test, the true outcomes appear to be heterogeneous ($\mathrm{Q}(1) = 5.23$, $\mathrm{p} = 0.02$, $\hat{\tau}^2 = 0.11$, $\mathrm{I}^2 = 80.88\%$). With only two studies, moderator analyses were not appropriate and tests of publication bias are not valid.

For peer review only - http://bmjopen.bmj.com/site/about/guidelines.xhtml

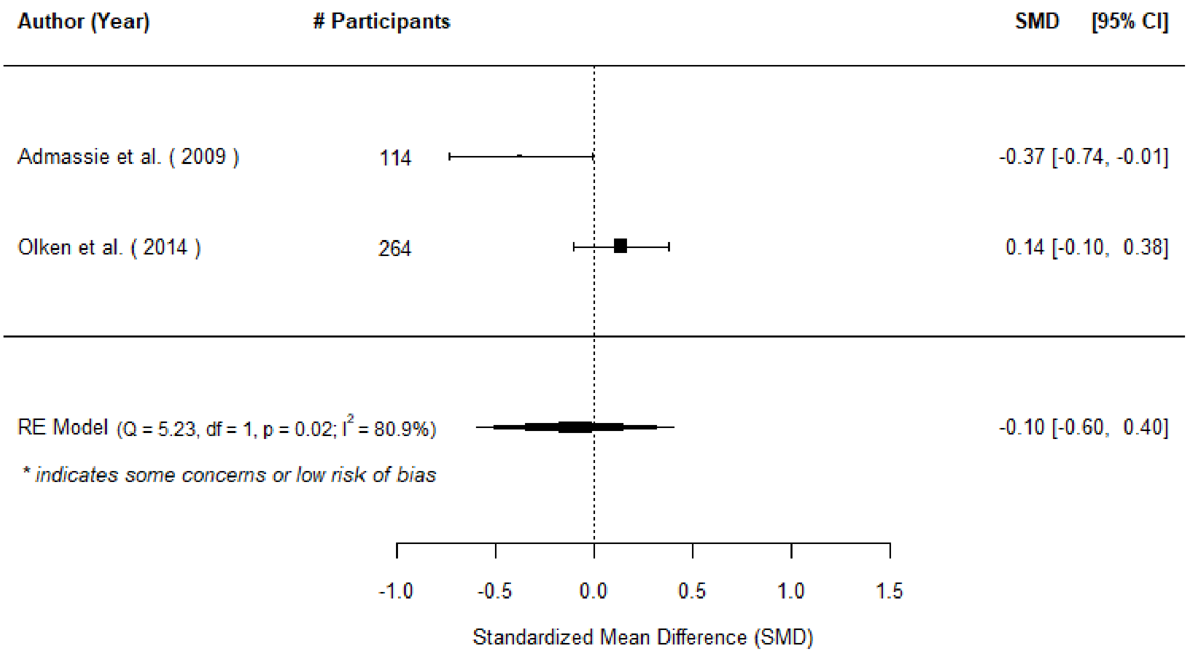

Supplementary Figure 77. Forest plot showing the observed outcomes and the estimate of the random-effects model for interventions with multiple engagement types on childhood morbidity.

Mortality

We included a total of $k = 3$ studies in the analysis. The observed outcomes ranged from -0.25 to -0.002. The estimated average outcome based on the random-effects model was $\hat{\mu} = -0.04$ (95% CI: -0.10 to 0.03, $see\ Supplementary\ Figure$ 78). Therefore, the average outcome did not differ significantly from zero ($z = -1.15$, $p = 0.25$), indicating no difference between the intervention participants and control participants on child mortality. The Q-test for heterogeneity was not significant, but some heterogeneity may still be present in the true outcomes ($Q(2) = 5.37$, $p = 0.07$, $\hat{\tau}^2 = 0.0018$, $I^2 = 62.75\%$). An examination of the studentized residuals revealed that none of the studies had a value larger than ± 2.39 and hence there was no indication of outliers in the context of this model. According to the Cook's distances, none of the studies could be considered to be overly influential. With only three studies, moderator analyses were not appropriate and tests of publication bias are not valid.

For peer review only - http://bmjopen.bmj.com/site/about/guidelines.xhtml

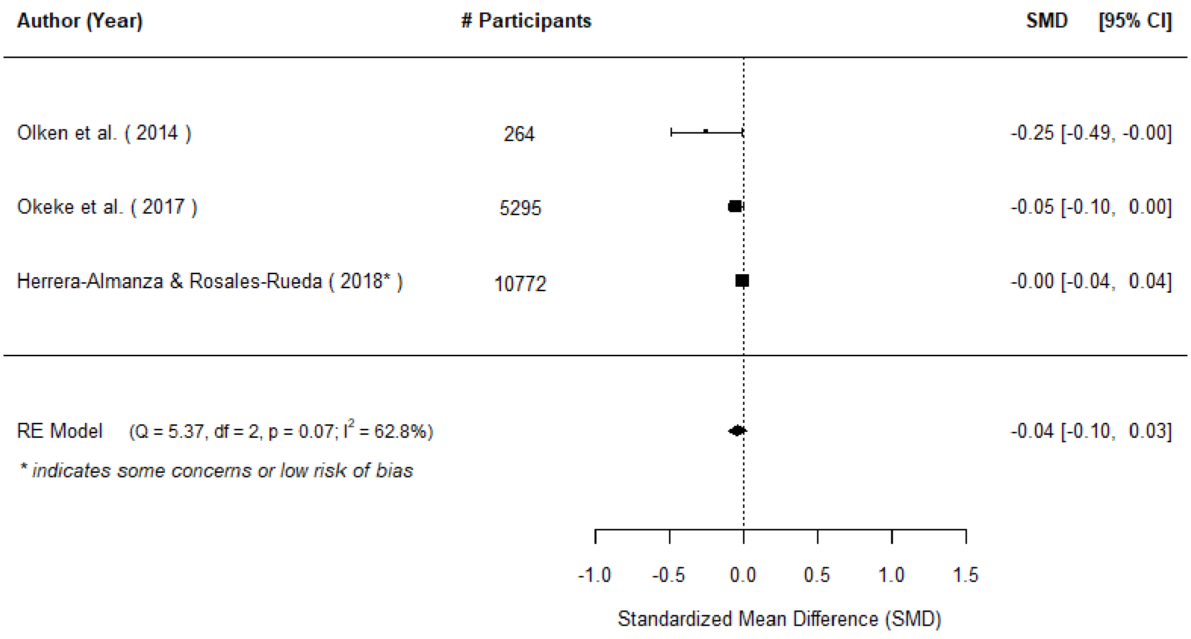

Supplementary Figure 78. Forest plot showing the observed outcomes and the estimate of the random-effects model for interventions with multiple engagement types on childhood morbidity.

Immunisation knowledge

Only two studies reporting on immunisation knowledge used interventions with multiple engagement types. The estimated average outcome based on the random-effects model was $\hat{\mu} =$ 0.09 (95% CI: 0.04 to 0.13). Therefore, the average outcome differed significantly from zero, indicating a small but significant benefit to the treated group compared to the control group ($z = 3.55$, $p < 0.001$), see Supplementary Figure 79). Given the small number of studies, this result should be interpreted with caution. According to the Q-test, the true outcomes appear to be homogeneous ($\mathrm{Q}(1) = 0.19$, $\mathrm{p} = 0.66$, $\hat{\tau}^2 = 0.00$, $\mathrm{I}^2 = 0.00\%$). With only two studies, moderator analyses were not appropriate and tests of publication bias are not valid.

For peer review only - http://bmjopen.bmj.com/site/about/guidelines.xhtml

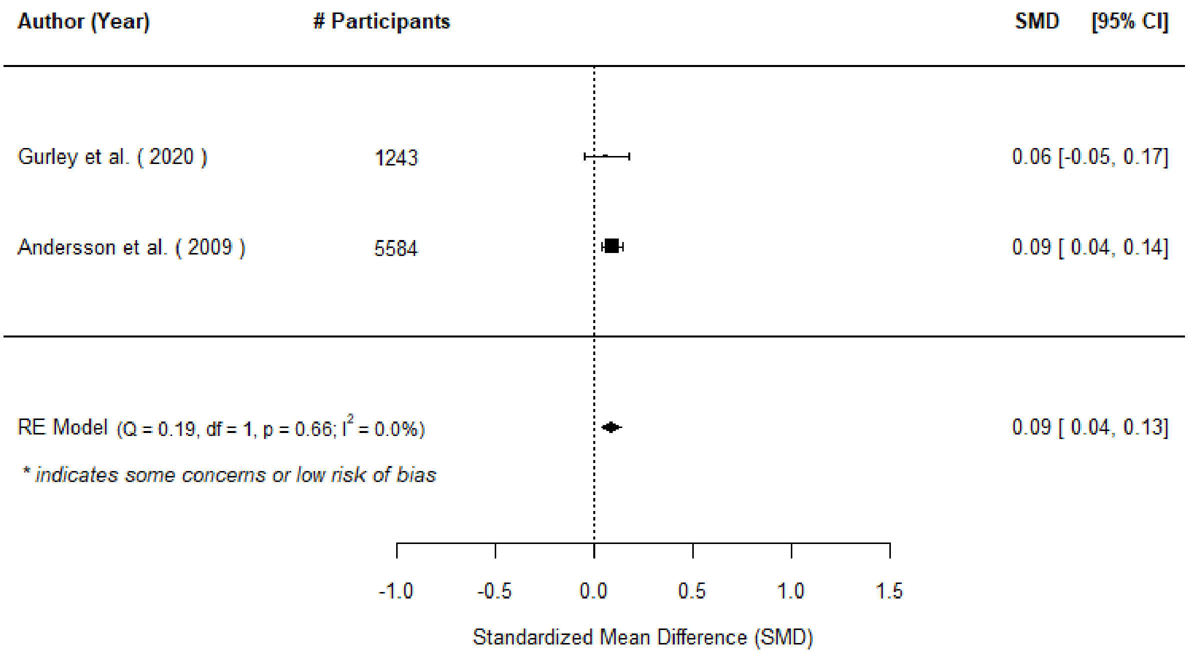

Supplementary Figure 79. Forest plot showing the observed outcomes and the estimate of the random-effects model for interventions with multiple engagement types on immunisation knowledge.

Immunisation attitudes

Only two studies reporting on immunisation attitudes used interventions with multiple engagement types. The estimated average outcome based on the random-effects model was $\hat{\mu} =$ 0.06 (95% CI: 0.01 to 0.11). Therefore, the average outcome differed significantly from zero, indicating a small but significant benefit to the treated group compared to the control group ($z = 2.50$, $p = 0.01$), see Supplemenatry Figure 80). Given the small number of studies, this result should be interpreted with caution. According to the Q-test, the true outcomes appear to be homogeneous ($\mathrm{Q}(1) = 0.50$, $\mathrm{p} = 0.48$, $\hat{\tau}^2 = 0.00$, $\mathrm{I}^2 = 0.00\%$). With only two studies, moderator analyses were not appropriate and tests of publication bias are not valid.

For peer review only - http://bmjopen.bmj.com/site/about/guidelines.xhtml

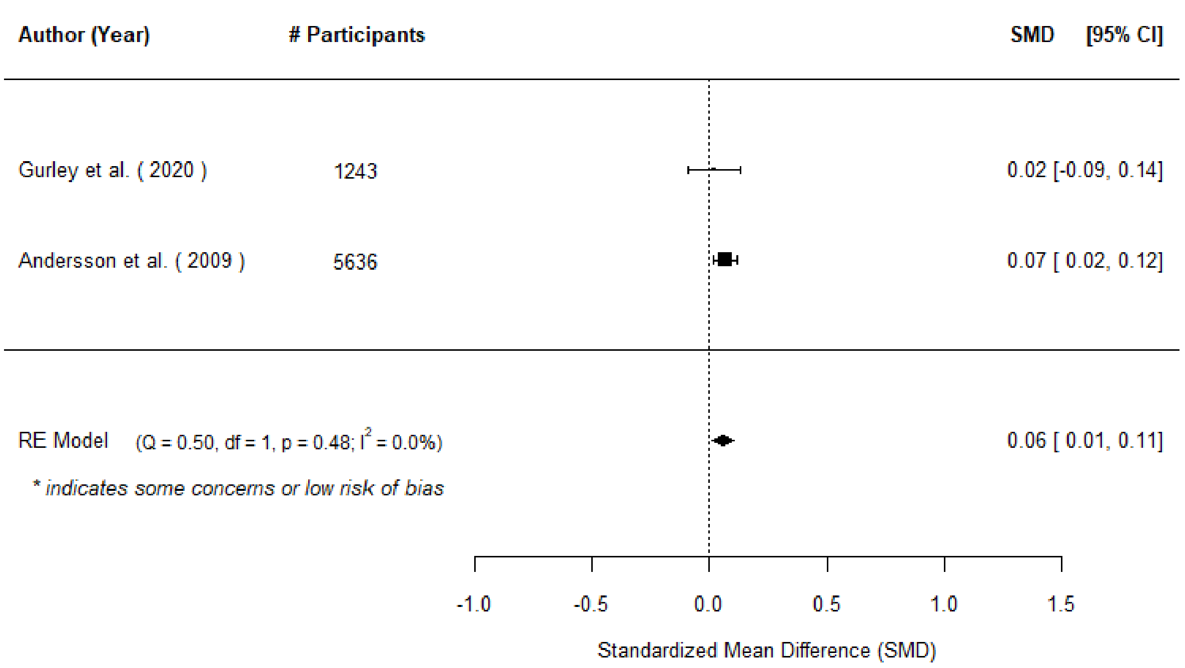

Supplementary Figure 80. Forest plot showing the observed outcomes and the estimate of the random-effects model for interventions with multiple engagement types on immunisation attitudes

Vaccination card availability

Only two studies reporting on vaccination card availability/retention used interventions with multiple engagement types. The estimated average outcome based on the random-effects model was $\hat{\mu} = -0.01$ (95% CI: -0.05 to 0.02). Therefore, the average outcome did not differ significantly from zero ($z = -0.78$, $p = 0.43$, see Supplementary Figure 81). Given the small number of studies, this result should be interpreted with caution. According to the Q-test, the true outcomes appear to be homogeneous ($\mathrm{Q}(5) = 0.13$, $\mathrm{p} = 0.43$, $\hat{\tau}^2 = 0.00$, $\mathrm{I}^2 = 0.00\%$; With only two studies and no heterogeneity, moderator analyses were not appropriate and tests of publication bias are not valid.

For peer review only - http://bmjopen.bmj.com/site/about/guidelines.xhtml

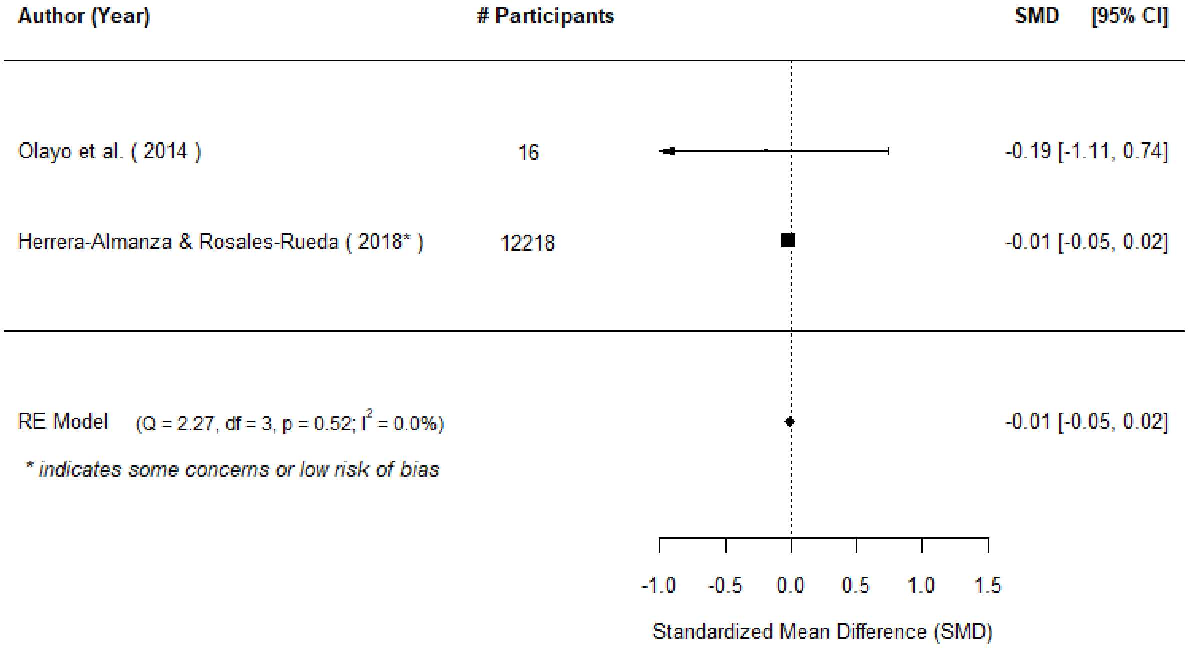

Supplementary Figure 81. Forest plot showing the observed outcomes and the estimate of the random-effects model for interventions with multiple engagement types on vaccination card availability

For peer review only - http://bmjopen.bmj.com/site/about/guidelines.xhtml

Appendix 14: Qualitative synthesis of results

Barriers to immunisation

Most information on barriers came from studies which used engagement as the intervention. Often, multiple barriers were cited in the same sentence. The vast majority of the barriers were identified through individual interviews and focus group discussions in the primary papers from which they were drawn. In addition, these were often supported by the authors' reflections, implementers' experiences, and observations. Sensitivity analysis, in which only qualitative papers that received an assessment score greater than 20 were included, indicated similar results. Therefore, we view the strength of the evidence to be high. Behavioural, social and practical barriers faced by caregivers were more consistently cited than direct constraints within the health system (Supplementary Figure 82, Supplemetary Table 4).

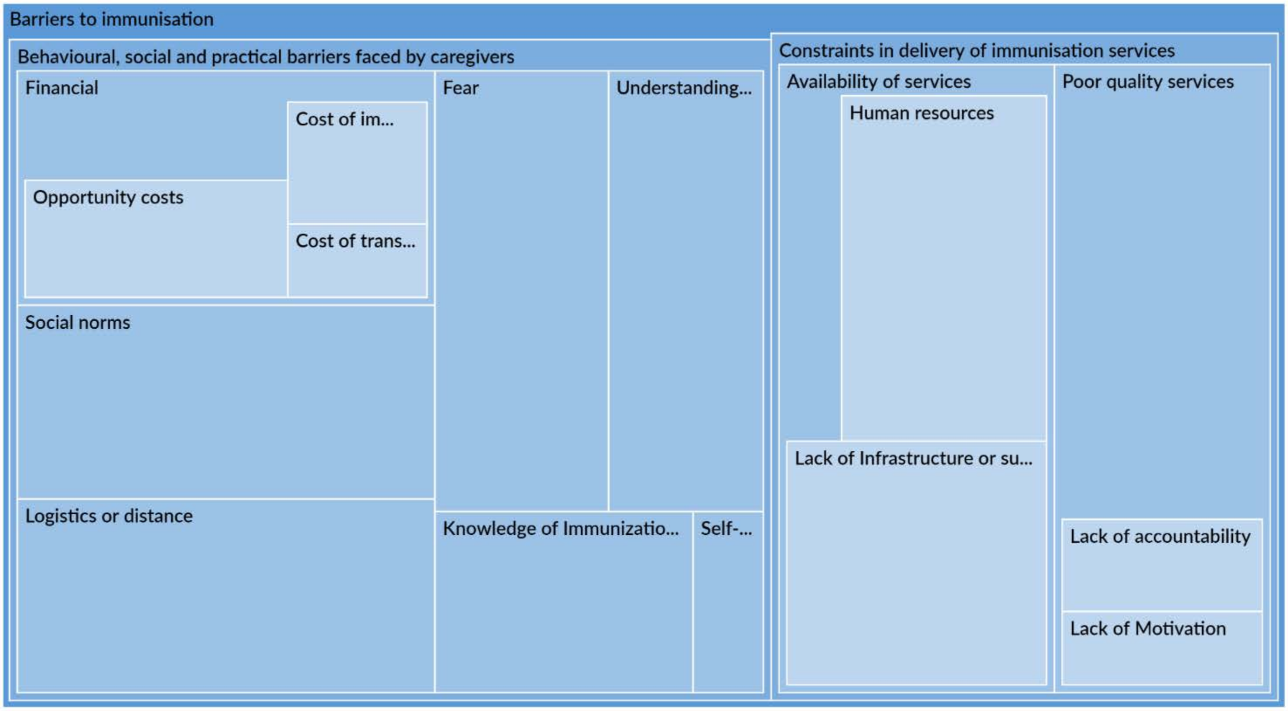

Supplementary Figure 82: Summary figure: Behavioural, social and practical barriers faced by caregivers were more consistently experienced than constraints in delivery of immunisation services[1]

The size of the rectangles reflects the number of times a unique theme was mentioned by an IE and its associated papers. Larger rectangles indicate that a theme was mentioned more times. Nested boxes reflect the proportion of times the main theme text also fell into the sub-them.

Supplementary Table 4: Comparison of the barriers to immunisation in communities in which community engagement activities were undertaken.

	**Community engagement as the intervention**	**Community engagement in the design of the intervention**	**Community engagement in the implementation autonomy of the intervention**

[1] The hierarchy charts showing barriers by type of community engagement are in Appendix H: barriers to immunisation

For peer review only - http://bmjopen.bmj.com/site/about/guidelines.xhtml

**Availability of resources**	Staff<br>Absenteeism<br>Stockouts<br>Building infrastructure	Staff<br>Stockouts	Staff<br>Post abandonment<br>Stockouts
**Quality of services**	Attitudes of health workers	Attitudes of health workers<br>Quality affected attendance	Attitudes of health workers<br>Services not implemented as intended
**Costs**	Other costs<br>Cost of the vaccine*	Other costs	Other costs<br>Cost of the vaccine
**Logistics or distance**	Distance	Convenience<br>Distance and access	Convenience<br>Transport
**Social norms**	Religious beliefs<br>Beliefs of others		Spousal consent<br>Religious beliefs
**Fear**	Side effects (and overcoming this fear)<br>Trust in the government or medicine	Side effects (from past experience)	
**Understanding of importance**	Cost and importance<br>Fear and importance		

Behavioural, social and practical barriers faced by caregivers

The most consistent behavioural, social or practical barrier faced by caregivers was cost. However, the kind of cost which posed a barrier was inconsistent, and mostly not that of the vaccination itself. Associated costs include opportunity costs, transport costs, and the costs of treating side effects. The cost of the immunisation itself was mentioned as a barrier by community members that received engagement as the intervention or engagement in the implementation of the intervention.

Logistics and distance were also consistent barriers to immunisation in settings where all three types of interventions occurred. The nature of the logistical or distance challenge was variable. Distance alone was reported as an issue in the communities with engagement as the intervention. However, Olken 2014 (World bank 2018) mention other logistical barriers such as wait times and language barriers. In settings where the community was involved in the design or implementation of the intervention, convenience was an issue in addition to distance. Community members in these areas reported issues related to wait times and the timing of clinic visits. Adamu 2019 (Adamu 2019 pre-implementation and Adamu 2019 Dissertation) mention demand being lower than it could be because women were unable to arrive at clinics at specified times due to competing demands.

Fear of side effects was common in communities in which engagement was the intervention or engagement was used in the design of the intervention. The concern was consistently related to known side effects and affected by past experience of side effect and trust in the government or medicine. The issue of vaccines causing sterilization was also mentioned. In one case, the intervention reduced these fears (Gurley 2020 (Gurley 2020)).

Social norms around immunisation posed consistent barriers; however, the source of the social restriction was variable. Spousal consent limited immunisations in areas where engagement was used in the implementation of interventions;

For peer review only - http://bmjopen.bmj.com/site/about/guidelines.xhtml

women could not vaccinate their children without the explicit approval of their husband, who may refuse. Religious beliefs sometimes resulted in the preference for non-Western medicine. In areas that received engagement as the intervention, religious beliefs and the influence of beliefs held by others sometimes inhibited immunisation. In particular, mother-in-laws and other family members who do not value immunisation may pose a barrier to immunisations.

Failure to *understand the importance of vaccination* was not consistently observed but reported in settings that received engagement as the intervention. When this barrier was mentioned, it was always paired with another, such as cost or fear. Authors implied that these other barriers were experienced and could not be overcome because there was a lack of understanding of the importance of vaccinations.

Constraints in delivery of immunisation services

Constraints in the delivery of immunisation services were widely reported but did not seem to be as common of a barrier as the behavioural, social and practical barriers faced by caregivers. The *availability of services* was the most consistently reported delivery barrier across all three types of engagement. Limited staff and stockouts were dominant delivery challenges. Communities that had engagement as the intervention or were engaged in implementation consistently have issues with staff being assigned but unavailable. Poor infrastructure, such as dilapidated buildings, was a challenge in communities that were involved in the design of the intervention. In areas where engagement was the intervention or the community was involved in the design of the intervention, the *quality of services* was also a more consistently reported barrier than any behavioural, social, or practical barrier faced by caregivers. Often, these were related to health worker attitudes, either a lack of motivation or conflict with caregivers.

Barriers to immunisation by type of community engagement

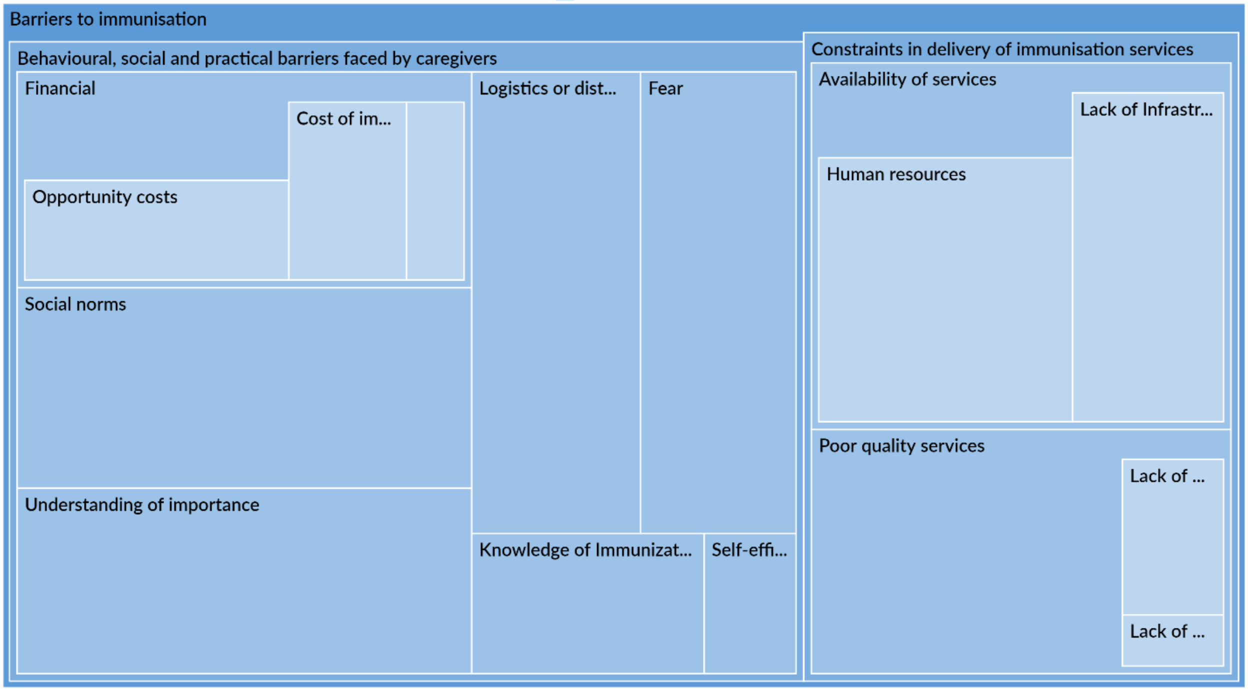

Figure 83: Engagement as intervention

For peer review only - http://bmjopen.bmj.com/site/about/guidelines.xhtml

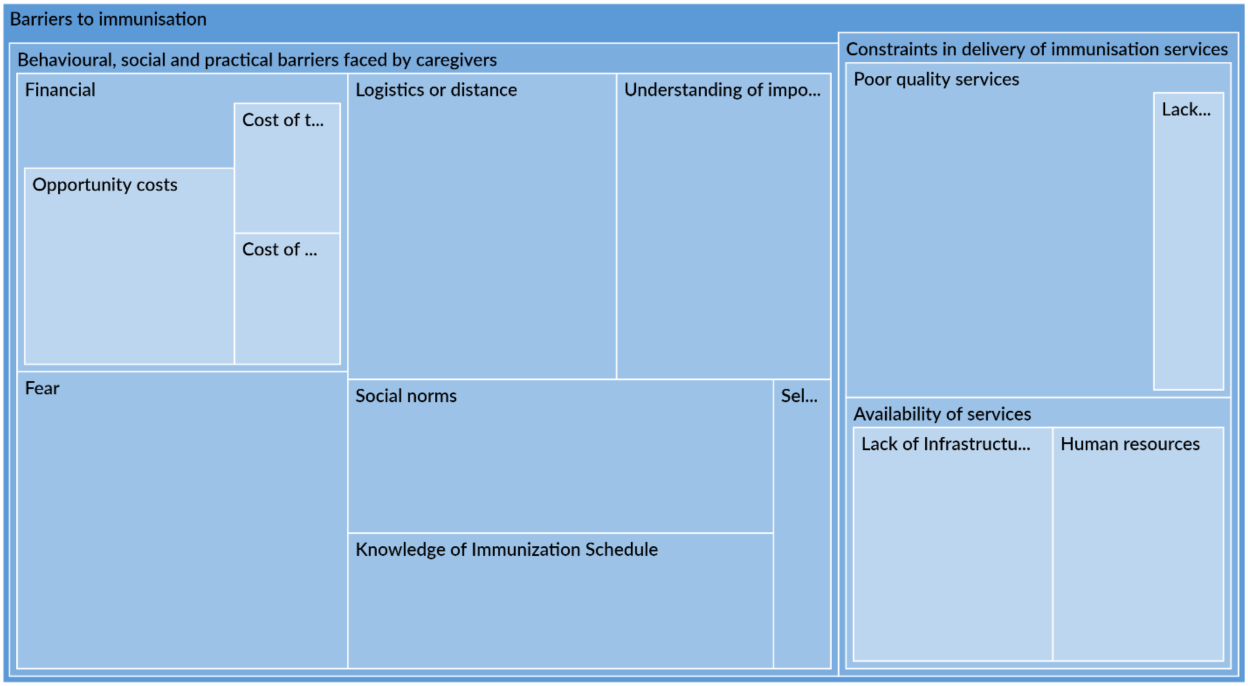

Figure 84: Engagement in design

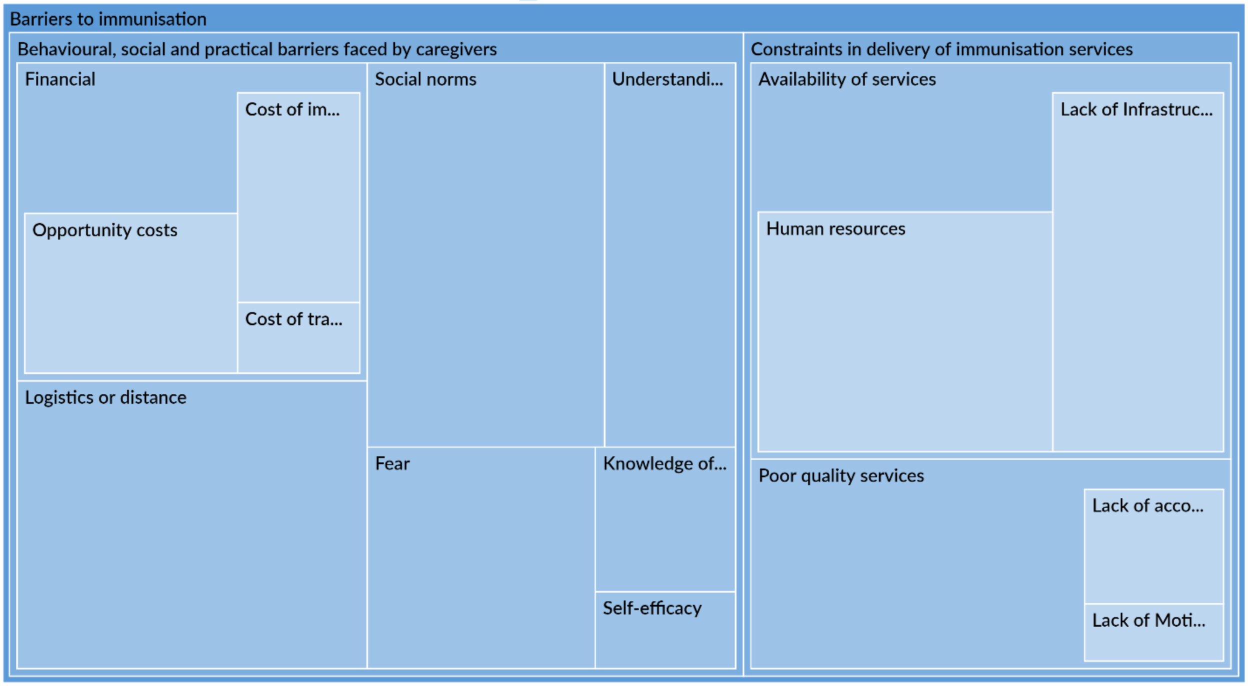

Figure 85: Engagement in implementation autonomy

Supplementary Table 5: Characteristic quotes of themes identified related to barriers to immunisation for interventions were community engagement.

For peer review only - http://bmjopen.bmj.com/site/about/guidelines.xhtml

Primary paper	Paper from which citation comes	Theme demonstrated	Quote	Page number
Financial				
Okeke et al 2017	Okeke et al 2017	Cost of vaccination	costs associated with use of services and lack of adequate transportation, hindered uptake.	54
Andersson 2009	Mitchell 2009	Other costs	Yet the groups noted that immediate financial and time costs associated with having a child vaccinated weigh very heavily in comparison with the potential costs associated with measles in the future.	7
Social norms				
Banerjee et al 2020	Appendix K	Religion	Religion emerged as an implicit theme. Only two respondents mentioned explicitly that religious belief might inhibit immunisation: that God would decide the fate of their child.	7
Andersson 2009	Cockroft 2009	Beliefs of others	"My mother-in-law says 'What kind of children have you produced that they ought to be vaccinated? We were never vaccinated, so why are you behaving so delicately?'" (female group, Khanewal)	8
Understanding of importance				
Olken 2014	Grayman 2013	Costs and importance	Typically the answer is that the parents are too busy working on their farms, or they feel that posyandu is no longer necessary after they are older than two years of age.	66
Paramanik 2018	Paramanik 2020	Fear and importance	As reported by ASHAs the mothers-in-law were not able to accept the concept of vaccination. They apparently argued on the ground that they had children too and that they never took them for any vaccination, that post vaccination the child gets fever which causes sleep disturbance for the child and in turn negatively affects the whole family.	84

For peer review only - http://bmjopen.bmj.com/site/about/guidelines.xhtml

Logistics or distance

Alhassan 2019	Alhassan 2019	Distance	In Ghana, access to maternal and child health services is impeded by longer travel times to health facilities.	2
Oyo-Ita 2020	Oyo-Ita 2020	Distance	children whose mothers found it difficult to reach the health facility	

Fear

Banerjee et al 2020	Appendix K	Trust	A couple of respondents suggested this could be interacting with a lack of trust of front line health workers.	20
Oyo-Ita 2020	Oyo-Ita 2020	Side effects	She said she doesn't want anybody to give her child injection so that the child will not become sick	31
Gurley 2020	Gurley 2020	Overcoming fear of side effects	We observed changes in how FGD respondents discussed side effects over the course of the study, from a perception that they were severe and not worth the risk of vaccination, to a sense they could be handled and are not a reason to forgo vaccination. I also took my child for vaccination. She cried for two days and I said let her cry; at least she will be safe in future from diseases. Mothers FGD, September 2017, intervention village	35

Human resources

Gurley 2020	Gurley 2020	Staff	In addition to suboptimal ANM clinical quality, nine ANM posts were vacant during the study period, resulting in suboptimal availability of services in some communities, which was beyond our influence	38
Olken 2014	Rahayu 2008	Absenteeism	the community is often disappointed with the services of puskesmas officers who do not attend posyandu services as this means that immunisations must be postponed until the posyandu session scheduled for the next month.	33

For peer review only - http://bmjopen.bmj.com/site/about/guidelines.xhtml

Infrastructure and supplies				
Okeke et al 2017	Okeke et al 2017	Building infrastructure	That is supposed to be our office. If you get there now, half of it is just sand; even the doors to this clinic are not closing, they are eating up by termite. the condition of two of the facilities was perceived to be detrimental to their ability to deliver care.	43
Findley et al 2013	Doctor et al 2011	Vaccine stockout and long wait times	67% of parents were unable to receive all immunisations reported lack of vaccine as a problem, and 13% had difficulties with the long wait.	17
Multiple barriers				
Okeke et al 2017	Okeke et al 2017	Cost of vaccination and logistics	costs associated with use of services and lack of adequate transportation, hindered uptake.	
Arifeen 2019	Billah 2018	Many simultaneous barriers	Furthermore, the lack of trust in formal health care providers, high out of pocket costs of health care in the public sector, and challenges with accessibility has led to the rapid proliferation of informal health providers to fill the gap between supply and demand across both rural and urban areas	2
Tandon 1988	Islam 2013	Many simultaneous barriers	low capacity to supervise monitor and implement micro plans at district level, lack of effective vaccine distribution to immunisation sites, ageing and poorly maintained Cold Chain, lack of adequately trained human resources, low managerial and support capacity at the state and district immunisation units and weak management of fund flows.	10

Supplementary Table 6: Characteristic quotes of themes identified related to barriers to immunisation for interventions that used community engagement in their design.

For peer review only - http://bmjopen.bmj.com/site/about/guidelines.xhtml

Primary paper	Paper from which citation comes	Theme demonstrated	Quote	Page number
Financial				
Anderson 2009	Andersson 2009	Other costs	Discussions in our focus groups confirmed the importance of poverty as a barrier to vaccination in many cases, as parents described being unable to afford the costs of the supposedly "free" immunisations: travel costs, opportunity costs, and demands for unofficial payments.	10
Fear				
Dipeolu 2017	Oladepo 2019	Fear of side effects	Prominent factors perceived as affecting timely and full completion of routine immunisations for children aged below 12 months include lack of awareness of immunisation (61.6%) and of subsequent doses (58.4%), and fear of side effects (59.7%). Others include rumours (for example, an association with future infertility)	464
Adamu 2019	Adamu 2019 Dissertation	Past experience	Experiences with the side effects of vaccines can influence the behaviour of caregivers and reduce their motivation to immunize eligible children.	134
Logistics				
Andersson 2009	Mitchell 2009	Physical access to facilities	In both urban and rural areas, access to a government facility providing vaccinations, a key equity factor, was a determining factor for uptake.	8
Nagar 2018	Nagar 2016	Convenience	not knowing where to go, not having time or mutually convenient time, facing long wait times at the camp, having fear of side effects, and acting under misguided advice	5
Human resources				

For peer review only - http://bmjopen.bmj.com/site/about/guidelines.xhtml

Borkum 2014	Rangarajan 2013	Staff	Staff vacancies are common, especially for lady health visitors and pharmacists. Few of the PHCs surveyed are fully staffed.	
Infrastructure or supplies				
Domek 2019	Domek et al 2018 Vaccine hesitancy	Stock outs	Of note, Guatemala experienced significant political instability during our study period, which led to considerable vaccine shortages experienced by all of our clinics.	3
Poor quality				
Dipeolu 2017	Dipeolu 2017	Attitudes of health workers	Attitudes and behaviours of healthcare workers such as treating mothers in an unfriendly, disrespectful, or even abusive manner are frequently cited as discouraging children's vaccination. Healthcare workers reportedly screamed at mothers who forgot the child's card, missed a scheduled vaccination	49
Gurely 2020	Gurley 2020	Quality affected attendance	For example, the existence of ANM vaccinators did not ensure their quality, and we observed that suboptimal clinical ANM quality discouraged retention across the vaccine schedule and perhaps negatively impacted beneficiaries' trust in the health system.	37

Supplementary Table 7: Characteristic quotes of themes identified related to barriers to immunisation for interventions that used community engagement in their implementation

**Primary paper**	**Paper from which citation comes**	**Theme demonstrated**	**Quote**	**Page number**
Financial				
Okeke et al 2017	Okeke et al 2017	Cost of the vaccine itself	costs associated with use of services and lack of adequate transportation, hindered uptake.	54

For peer review only - http://bmjopen.bmj.com/site/about/guidelines.xhtml

Adamu 2019	Adamu 2019 Dissertation	Other costs associated with vaccination	However, the cost of treating vaccine reactions like fever that might occur following immunisation are borne for the caregivers, which can result in out-of-pocket expenditure.	133
Social norms				
Adamu 2019	Adamu 2019 Dissertation	Spousal consent	In addition, we found that the high level of social control that men have over women in this area could also cause MOV among children. Caregivers reported that without their husband's consent, they still cannot vaccinate their children, even if they're in a health facility for other preventive or curative services.	133
Olken 2014	Rahayu 2008	Religious beliefs	The community trusts and believes in the choice that their parents made to use the dukun beranak, to the point where it becomes the norm.	34
Logistics or distance				
Webster 2019	Webster et al 2019	Convenience	Key reasons cited for non-immunisation were related to lack of convenience; the aforementioned distance between homes and health facilities, moving to the fields during farming seasons,	42
Alhassan 2019	Alhassan 2019	Transport	In Ghana, access to maternal and child health services is impeded by longer travel times to health facilities.	2
Human resources				
Olken 2014	Febriany 2011	Post abandonment	Poor facilities adversely affected the availability and utilization of MCH and basic education services. The midwife did not want to live in the village because here there is no electricity and no water (FGD Female-NTT).	9

For peer review only - http://bmjopen.bmj.com/site/about/guidelines.xhtml

Herrara-Almanza 2018	Herrara-Almanza & Rossales-Rueda 2018	Lack of personnel	Poor transportation infrastructure and a shortage of medical personnel limit the basic health access to the population living in remote areas	7
Infrastructure or supplies				
Webster 2019	Webster et al 2019	Vaccine stockouts	Furthermore, vaccine stock-outs at health facilities discouraged caregivers from attending vaccination services.	42
Multiple barriers				
Webster 2019	Webster et al 2019	Demand and supply side	Health system factors affecting immunisation service delivery and uptake in northern Uganda include interruptions in the vaccine cold chain due to poor management of equipment and supplies, limited supervision of health teams by the district health teams, low staffing levels, long distances to the health facilities especially in the hard-to-reach areas, and limited resources to support outreach services. There is also a lack of good quality data to support decision-making.	1
Olken 2014	Rahayu 2008	Poor quality and post abandonment	Dissatisfaction is often related to the midwife's character, ineffective medicine, minimal experience of the midwife, difficulties in reaching the midwife, and a midwife's absence from the post.	68
Herrara-Almanza 2018	Herrara-Almanza & Rossales-Rueda 2018	Transport and personnel	Poor transportation infrastructure and a shortage of medical personnel limit the basic health access to the population living in remote areas	7

Facilitators of immunisation

Fewer studies discussed facilitators of immunisation as compared to barriers. Much of the information coded came from studies in which the intervention was engagement. To a substantial extent, the sources of this information were key individual interviews and focus group discussions. These were often supported by surveyor or enumerator's observations. In some instances, discussion on facilitators in the primary papers was informed by existing literature.

For peer review only - http://bmjopen.bmj.com/site/about/guidelines.xhtml

A sensitivity analysis, comprising 19 qualitative papers with a risk of bias assessment score of 20 or higher, indicated similar results. Therefore, we view the evidence to be of high strength. Facilitators related to behavioural, social and practical factors faced by caregivers were more common than the facilitators related to the delivery of immunisation services (Supplementary Figure 86). A segregated analysis of the facilitators by the engagement type was not possible due to the limited information coded on this theme.

Behavioural, social and practical barriers faced by caregivers

The most consistent facilitator across all intervention types was the caregiver's *understanding of importance* of immunisation. In general, studies associated caregivers' awareness and perception of the benefits of vaccination to an enabling environment for immunisation uptake (Adamu 2019; Banerjee 2020). In most instances caregivers were able to link benefits of vaccines to the diseases it prevented (Anderson (Cockroft 2009); Pramanik 2020).

Lack of logistics or distance related issues and enabling *social norms* were also consistently sighted as facilitators of immunisation. When it came to logistics, easy access either in the form of proximity to a health care facility (Anderson (Cockroft 2009)) or provision of transportation to an immunisation camp (Olken 2014; Rahayu 2008) were cited as enabling factors. *Social norms* were mentioned as a facilitator in instances where mothers had a significant influence of over decisions regarding her child's health. In addition, peer influence was also mentioned as a factor (Banerjee 2020). Though less consistently cited than the other factors, f*avourable socio-economic characteristics* such as maternal education (Admassie 2009) were also cited as facilitators of immunisation.

Constraints in delivery of immunisation services

Delivery of immunisation services was less consistently reported as a facilitator as compared to behavioural, social and practical factors. The most consistentdelivery related facilitator was the *availability of infrastructure or supplies* which included provision of good quality of services, availability of adequate healthcare providers at the point of care and availability of vaccine supplies.

For peer review only - http://bmjopen.bmj.com/site/about/guidelines.xhtml

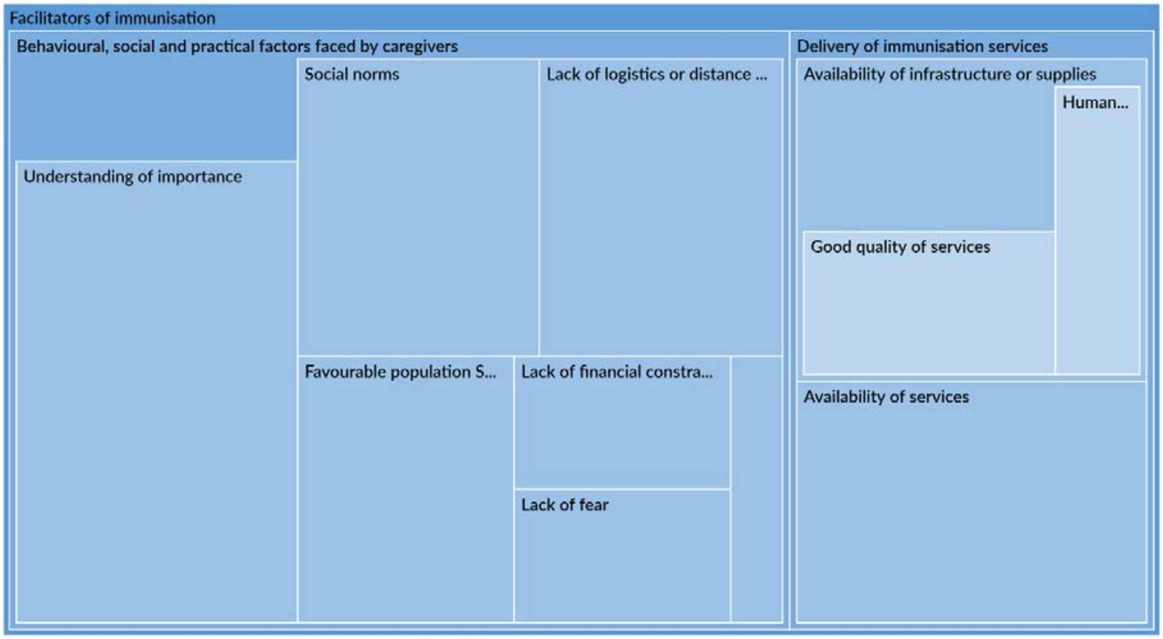

Supplementary Figure 86: Summary figure: Facilitators related to behavioural, social and practical factors faced by caregivers were more common than those related to delivery of immunisation services

The size of the rectangles reflects the number of times a unique theme was mentioned by an IE and its associated papers. Larger rectangles indicate that a theme was mentioned more times. Nested boxes reflect the proportion of times the main theme text also fell into the sub-them.

For peer review only - http://bmjopen.bmj.com/site/about/guidelines.xhtml

Facilitators of immunisation by type of community engagement

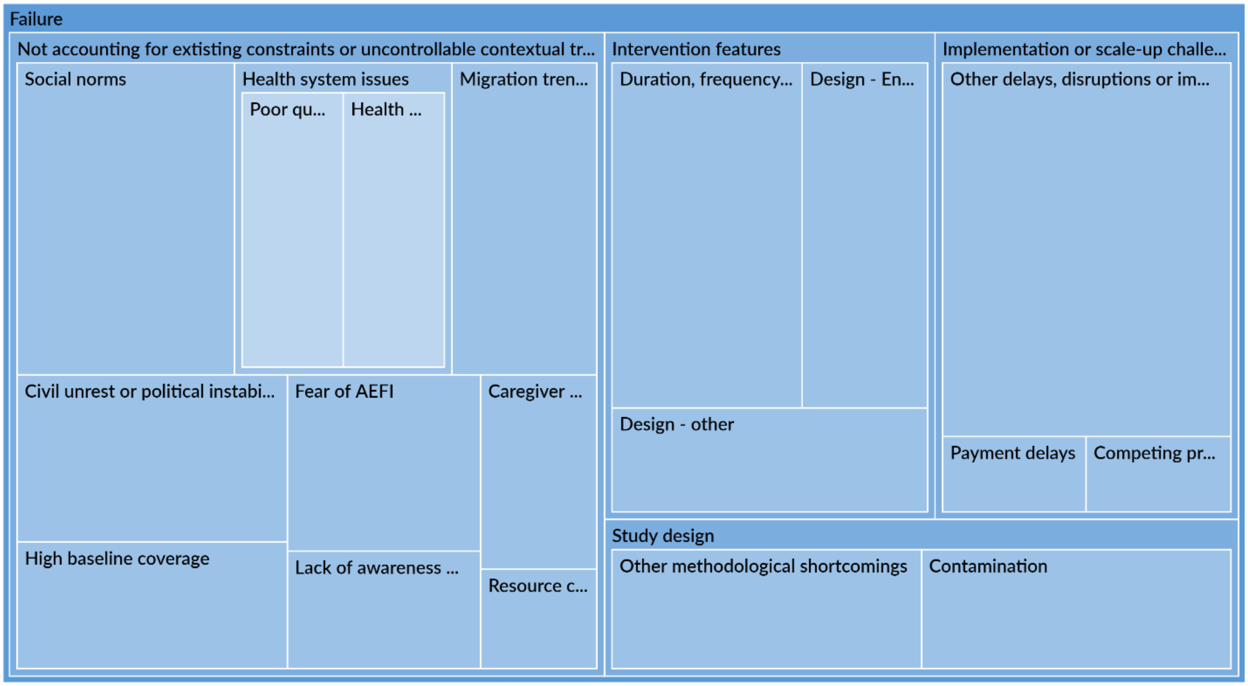

Supplementary Figure 87: Engagement as intervention

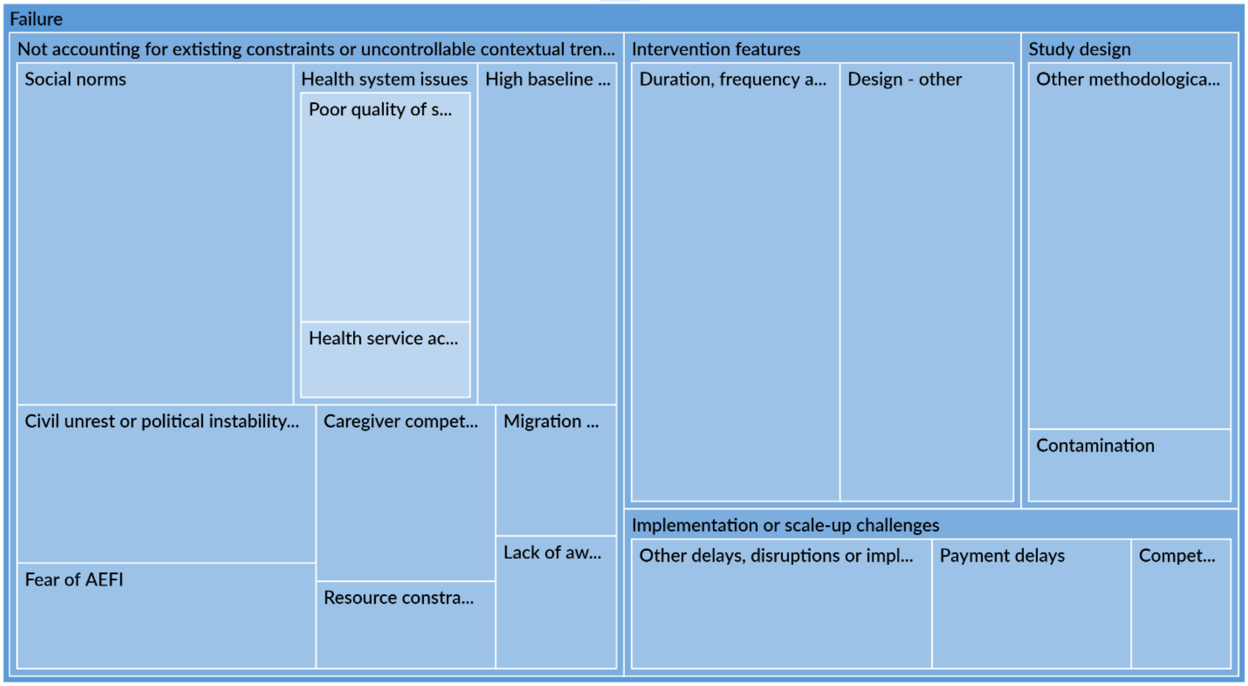

Supplementary Figure 88: Engagement in design

For peer review only - http://bmjopen.bmj.com/site/about/guidelines.xhtml

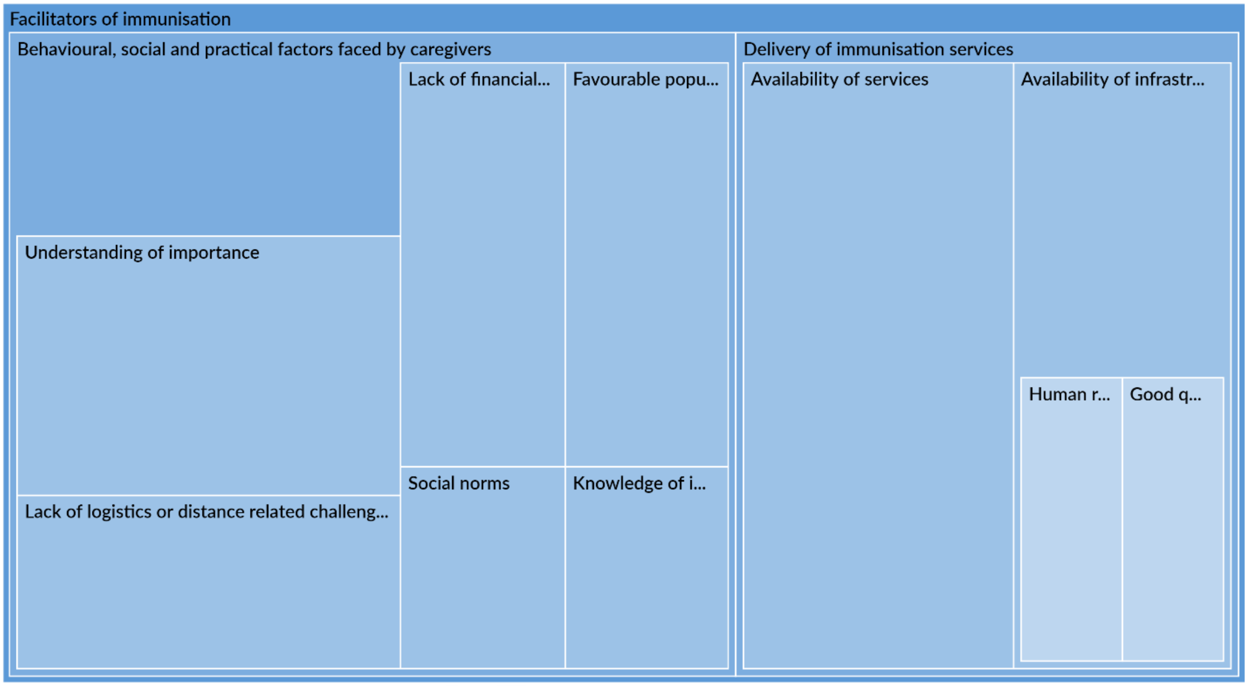

Supplementary Figure 89: Engagement in implementation autonomy

Supplementary Table 8: Characteristic quotes of themes identified related to facilitators of immunisation for community engagement interventions

Primary paper	Paper from which citation comes	Theme demonstrated	Quote	Page number
Understanding of importance				
Adamu et al. 2019	Adamu 2019 Dissertation	Awareness of benefits	In addition, caregiver beliefs about the capabilities of immunization was overwhelmingly strong: "… this vaccination is very important because it prevents infection from measles, cough, hepatitis, fever, pneumonia and yellow fever.	129
Oyo-Ita et al. 2020	Oyo-Ita et al. 2020	Positive perception of vaccination	It was found from the baseline qualitative study that respondents were generally knowledgeable about and had a positive attitude towards vaccination. They believed vaccines prevented their children from acquiring deadly infections and attributed low numbers of deaths of children to vaccination	31
Socio-economic characteristics				

For peer review only - http://bmjopen.bmj.com/site/about/guidelines.xhtml

Andersson et al. 2009	Cockroft 2009	Importance of maternal education	Mother's education was related to measles vaccination in all four districts, in urban and rural sites.	10
Social norms				
Pramanik 2020	Pramanik 2020	Household decision-making	Key informants further mentioned that in rare cases, it was seen that presence of a knowledgeable daughter in law in the family compelled families to vaccinate their children	85
Availability of infrastructure, supplies or services				
Olken et al. 2014	Rahayu 2008	Availability of medical supplies	The midwife has comprehensive equipment. She has infusion equipment, blood pressure monitor, injections, medicines, scales for infants. The midwife also provides a room in the polindes for mothers who have just given birth. (Women's FGD, Kuanek, East Miomaffo, TTU, NTT)	25
Andersson et al. 2009	Cockroft 2009	Outreach by healthcare workers	in rural sites a vaccination team visiting the community also increased the likelihood that the child had received measles vaccine; this effect was much stronger in rural Khanewal.	5
Adamu et al. 2019	Adamu 2019 Dissertation	Availability of vaccination services	In this study, some caregivers indicated that recommended birth doses of vaccines are provided in the labor room as soon as the child is born. This practice saves time and reduces the chance of missed opportunities in this service delivery point.	134

For peer review only - http://bmjopen.bmj.com/site/about/guidelines.xhtml

Reasons for intervention success

Most studies with significant positive quantitative impacts provide some plausible explanation for why an intervention succeeded. These were often based on authors' notes, experiences and impressions of *why* their hypothesis was proven true. We expect the authors to be a good source of this information because of their knowledge of causal linkages and assumptions made in the intervention theory of change. Individual interviews and focus group discussions supported the authors' conclusions. The sensitivity analysis, which included qualitative papers with an assessment of score of 20 or higher, showed similar results. Given this, we consider the evidence to be of high strength.

Overall, reasons for intervention success were consistently attributed to specific *intervention features* across all engagement types. These included community engagement, leadership and supportive supervision, customisation to local context, incentives, health system integration and other design features. Though not as common, success was also attributed to *existing or changing favourable characteristics* within a given context such as availability of and access to good quality health services as well as positive participant views with respect to the intervention (Supplementary Figure 90; Supplementary Table 9).

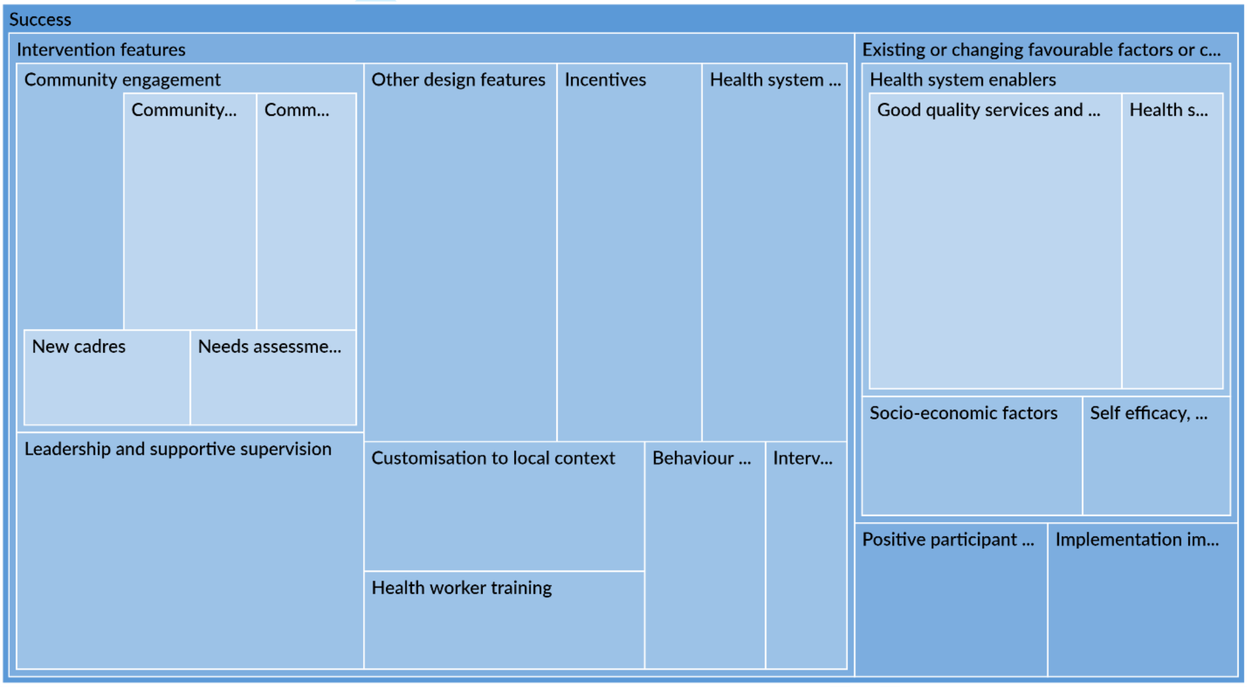

Supplementary Figure 90: Reasons for success: a summary figure

The size of the rectangles reflects the number of times a unique theme was mentioned by an IE and its associated papers. Larger rectangles indicate that a theme was mentioned more times. Nested boxes reflect the proportion of times the main theme text also fell into the sub-theme.

Supplementary Table 9: Comparison of the reasons for intervention success by type of community engagement.

For peer review only - http://bmjopen.bmj.com/site/about/guidelines.xhtml

	Community engagement as the intervention	Community engagement in design	Community engagement in implementation autonomy of interventions
**Intervention features –** *community engagement*	Community dialogues<br>Creation of new cadres	Community dialogues<br>Needs assessments	Community involvement in planning & implementation
**Intervention features –** *Leadership & supportive supervision*	Encouragement from leaders	Supportive supervision	Involvement of leadership<br>Health worker autonomy
**Intervention features –** *Health system integration*	Involvement of government in implementation	Separate workforce dedicated to the intervention	Intersectoral integration of health programmes
**Intervention features –** *Health worker training*	Training on monitoring health cards of children	Training of all health personnel involved in the intervention	Training of all health personnel involved in the intervention
**Favourable contextual factors –** *Health system enablers*	Good quality of services<br>Health service access	Good quality of services	Good quality of services
**Favourable contextual factors –** *Socio-economic factors*	Female literacy	General literacy levels<br>High maternal education	Primary schooling of girls and women
**Improvements in intervention Implementation over time**	-	Health worker performance improvement over time	Resolution of fidelity/adherence issues
**Positive participant/ beneficiary views**	Caregiver perception	Health worker perception	Health worker perception

.

For peer review only - http://bmjopen.bmj.com/site/about/guidelines.xhtml

Community engagement as the intervention

Studies in which engagement was the intervention consistently attributed success to characteristics associated with intervention features and relatively less consistently to existing or changing favourable characteristics within a given context.

Notably, success was attributed to *intervention features* associated with community engagement such as community dialogues which were participatory in nature and improved relationship between the health system and communities (Assegaai 2018 (Padayachee 2013); Findley 2013). Involvement of community members such as traditional and religious leaders in planning and implementation of an intervention also came up as a reason for success (Oyo-Ita 2020). In a few instances, success was also attributed to acceptance of health worker cadres by the communities as long as these health workers belonged to the communities they served (Biemba 2016).

Studies also attributed intervention success to non-engagement *intervention features* including incentives given to caregivers, leadership and supportive supervision which improved overall health service delivery and health worker performance. As noted above, some studies also cited *existing or changing favourable characteristics within a given context* as reasons for success. Among these, those related to the health system, particularly, good service quality (Rao 2016) and, to a lesser extent, access to services (Banwat 2015 (Banwat 2014)) were consistently reported. Interestingly, none of the studies in this engagement category mentioned *implementation improvements* as a reason for intervention success.

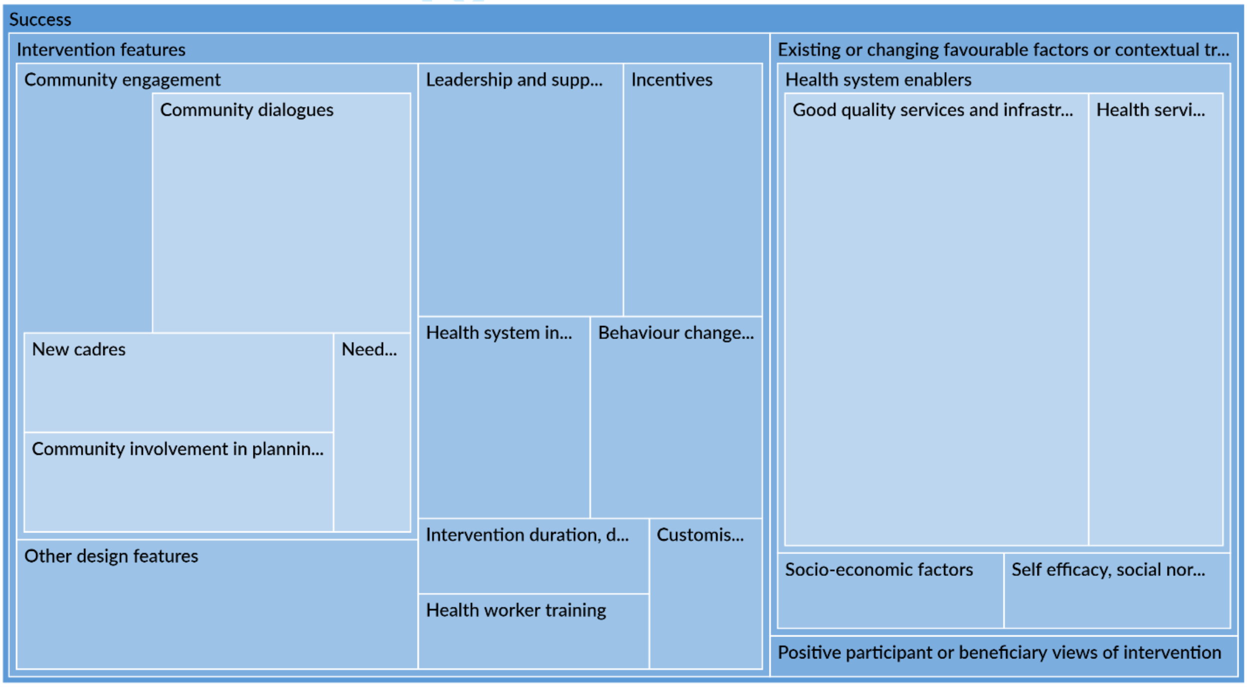

Supplementary Figure 91: Reasons for success of interventions with engagement as intervention

Supplementary Table 10: Characteristic quotes of themes identified related to reasons for success for interventions were community engagement.

Primary paper	Paper from which citation comes	Sub-theme demonstrated	Quote	Page number

For peer review only - http://bmjopen.bmj.com/site/about/guidelines.xhtml

Intervention features				
Assegaai 2018	Padayachee 2013	*Community dialogue*	Interviewees spoke of the tangible benefits of the community dialogues and proactive household approaches as expanding access, improving relationships with communities, and increasing knowledge and uptake of services.	76
Oyo-Ita 2020	-	Community involvement in planning and implementation	Inclusion of TRLs in the planning, implementation and evaluation of an intervention is useful in ensuring support from the community.	42
Biemba 2016	Phiri 2017	Leadership and supportive supervision	CHAs reported that supervision by experienced health workers was valuable to them because it reinforced skills they learned in training and provided general encouragement. This study finds that the quality and frequency of supervision is heavily influenced by the proximity of supervisors' work station to that of the CHAs.	5
Banerjee 2020	-	Incentives	Most primary caregivers expressed positive opinions about recharges. Some admitted to finding them motivating or influencing their decision to immunise their child (whether or not they actually received them). This was linked to generally liking free things ('What's not to like?'), their self-assessed poverty level, or their trust in the government 'investing in them'.	53

For peer review only - http://bmjopen.bmj.com/site/about/guidelines.xhtml

Existing or changing favourable factors or contextual trends

Rao et al. 2014	Rao et al. 2016	Good quality of services and infrastructure	Moreover, the ASHA worker is envisioned not to work in isolation but as an integral part of the public health system, working together with other health personnel to both ease their work-pressures and assist them in performing their duties more efficiently. For instance, the ASHA worker works with the ANM and the anganwadi worker to organize monthly village health days. Therefore, the extent to which the increase in information provision via other health workers is attributable to the ASHA worker is not quantifiable in this context.	25
Findley et al. 2013	-	Good quality of services and infrastructure	simultaneous improvement in the quality of care provided by the CHW and nurse-midwives at the health post gave women the confidence that they could go to the health post to seek advice and care.	10
Banwat 2015	Banwat 2014-	Health service access and availability	A child was also found to be most likely to be fully and timely immunized if he/she lived within 30 minutes walking distance from the health facility.	66

Community engagement in design

The most consistentreasons for success were related to *intervention features* with the most reported characteristics being community engagement and provision of incentives to caregivers. Within community engagement, participatory community dialogues (Andersson 2009), stakeholder consultations on intervention design (Modi 2019) and community involvement in planning and implementation (Adamu 2019) were associated with intervention success. Receiving incentives was perceived to be motivating and empowering by the intended beneficiaries (Banerjee 2020; Nagar 2020).

For peer review only - http://bmjopen.bmj.com/site/about/guidelines.xhtml

Besides community engagement and incentives, o*ther intervention features* that were consistently reported included leadership and supportive supervision, health worker training and customisation to local context.

Very few studies discussed or attributed success to reasons other than intervention features. Nevertheless, the few studies that did, referred to *existing or changing favourable contextual factors* such as a high prevalence of maternal education (Johri 2020) or *improved implementation* by resolution of issues related to adherence (Modi 2019). A few studies also discussed *positive participant or beneficiary views* that could have potentially contributed to intervention success.

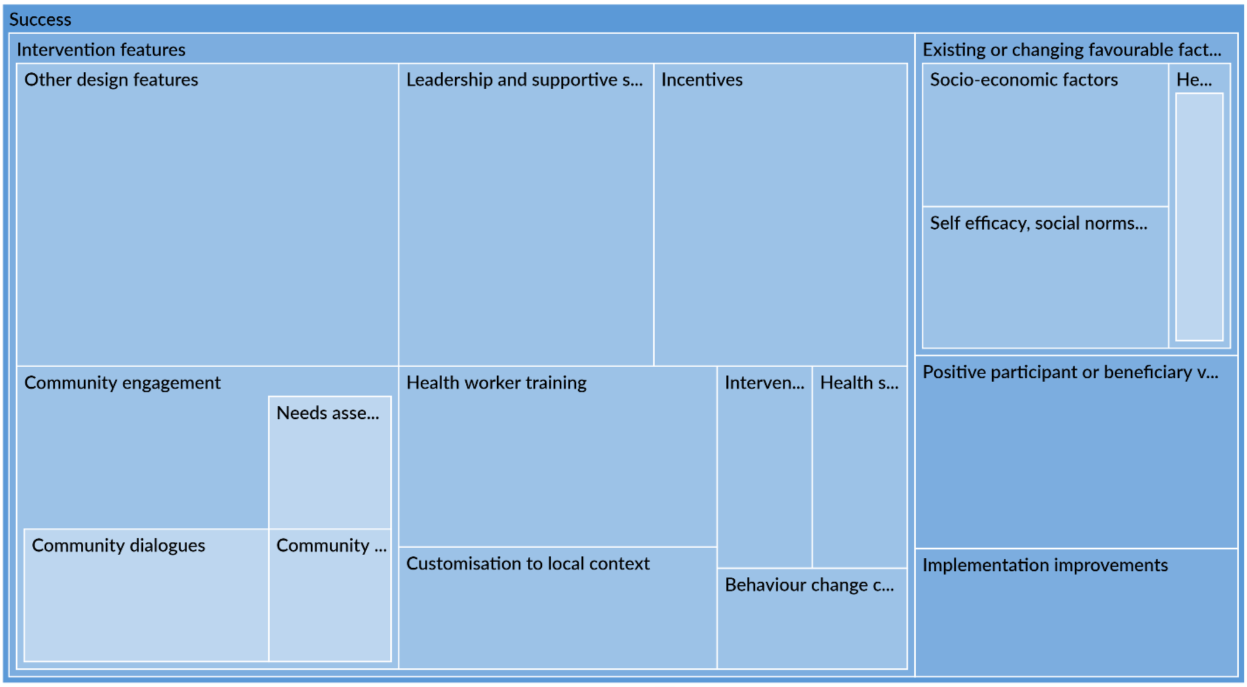

Supplementray Figure 92: Reasons for success of interventions with engagement in design

Supplementary Table 11: Characteristic quotes of themes identified related to reasons for success for interventions that used community engagement in their design.

**Primary paper**	**Paper from which citation comes**	**Sub-theme demonstrated**	**Quote**	**Page number**
Intervention features				
Andersson 2009	-	Community dialogue	The structured discussion rounds sometimes led to action plans in the intervention communities beyond stimulating discussion about vaccinations within households. Particularly in those villages with poor access to vaccination services, plans included sharing transport to vaccination points and providing care for some children while parents took others to be vaccinated. These community initiatives may have helped to maintain vaccination levels in the face of generally falling levels.	8

For peer review only - http://bmjopen.bmj.com/site/about/guidelines.xhtml

Adamu 2019	Adamu 2019 CFIR	Leadership and supportive supervision	Participants expressed satisfaction with the supervisory plan that was put in place for the quality improvement program as it enabled quick feedback. Supervisory visits were conducted by different stakeholders that are higher-ranking officials within the health systems. These include local government and zonal primary health-care management board officials. "We receive supervision from local government, they use to come and supervised us to check how we conduct our duties."– PHC 3	468
Assegaai 2018	Khuzwayo 2017	Referral to caregivers	Referrals to clinics by the teams were regarded as an important aspect of bringing services closer to the communities. Respondents said that they would not have gone to the clinic had they not been referred by the WBOT, for example for deworming of children, or continuation of care for complicated cases.	3
Existing or changing favourable factors or contextual trends				
Banerjee 2020	-	Self efficacy, social norms and awareness	By and large the main, explicitly stated driver for primary caregivers to get their child vaccinated is their positive perception or attitude toward immunization: that immunization will benefit their child. This acts as a push even for primary caregivers who have limited knowledge, who don't understand exactly how or why immunization is beneficial. Some of these primary caregivers hold strong personal conviction of the importance of immunization, despite their lack of knowledge, while others are undecided or unsure about the importance of immunization, but are still overall positive. In both cases, it seems that the positive perception or attitude is influenced by others: what others say and do. This is often mediated by trust: either trust in government and authority (e.g. ANM), household or family members, other primary caregivers in the community, or people in positions of power in the community (e.g. religious leaders in Mewat). Interestingly, improved knowledge and awareness was stated by a number of respondents as a key factor to convince primary caregivers (themselves, or others) to attend immunization camps. This demonstrates, as was mentioned in response to ANM performance, that	Appendix K – qualitative findings

For peer review only - http://bmjopen.bmj.com/site/about/guidelines.xhtml

			primary caregivers value information about vaccines	
Gibson et al. 2017	-	Socio-economic factors	The success of SMS reminders to elicit a behaviour is multifactorial; the content of the message, the type of behaviour being reminded, indirect and direct costs incurred, literacy level, and other contextual factors all being potential explanatory factors.	e436
Implementation improvements				
Modi 2019	-	Operational improvements	Finally, we identified critical operational requirements that improved adherence to the intervention; this included supportive supervision, timely resolution of technology problems, and change management, including monetary and/ or nonmonetary incentives depending on the context.	20

Community engagement in implementation autonomy of intervention

Similar to the previous two engagement categories, reasons for success in interventions with engagement in implementation were also consistently attributed to certain *intervention features*. These characteristics were associated with certain aspects of *community engagement* itself such as conducting stakeholder consultations (Modi 2019) or involvement of community members such as health workers in intervention planning and implementation (Adamu 2019). Other aspects of intervention features such as *integration with the health system, customisation to the local context* and *leadership and supportive supervision* were also consistently cited.

Like the engagement in design category, crediting intervention success to *existing or changing favourable contextual factors* or *improved implementation* was less consistently reported among studies in which community engagement was involved in the implementation of the intervention. Nevertheless, *health system enablers* such as good quality of services and access to health services were mentioned as factors which could have contributed to intervention success.

For peer review only - http://bmjopen.bmj.com/site/about/guidelines.xhtml

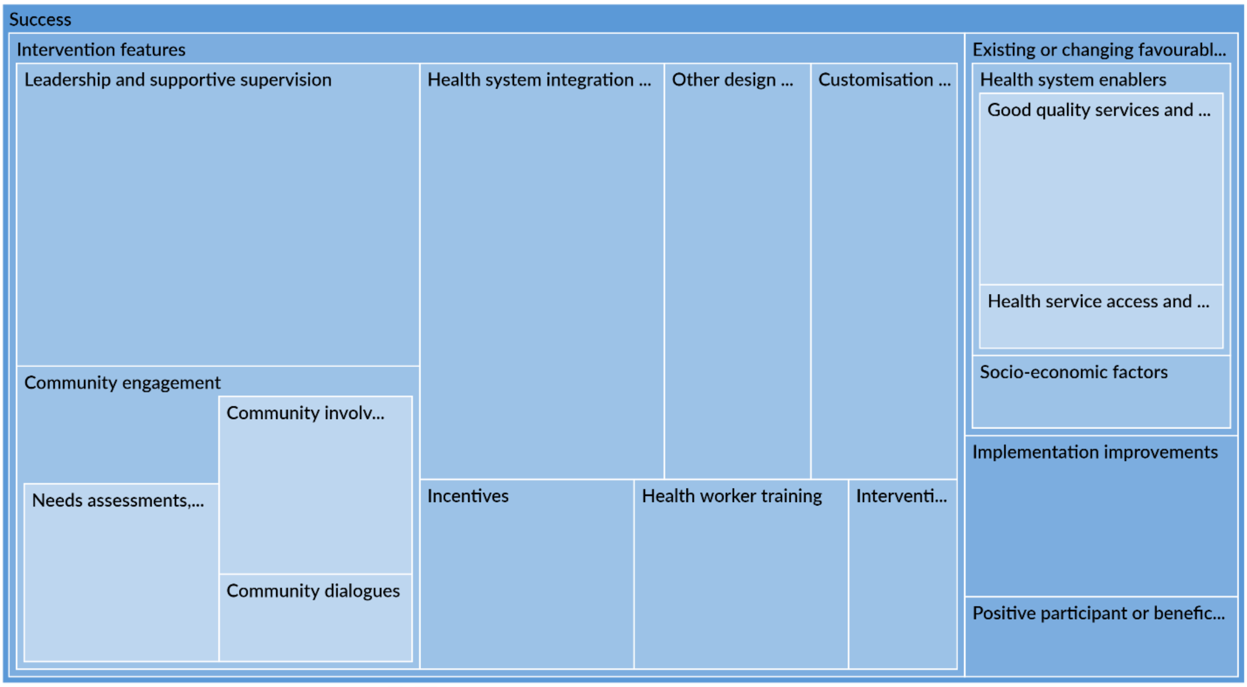

Supplementary Figure 93: Reasons for success of interventions with engagement in implementation autonomy

Supplementary Table 12: Characteristic quotes of themes identified related to reasons for success for interventions that used community engagement in implementation.

**Primary paper**	**Paper from which citation comes**	**Sub-theme demonstrated**	**Quote**	**Page number**
Intervention features				
Adamu 2019	-	Community involvement in planning and implementation	Stakeholders were systematically involved in the planning and execution of the QI program. The change ideas were selected by frontline health workers and they tailored them to their local context. Also, change ideas are multi-faceted and multimodal, and this is suitable for addressing complex problems in complex health systems.	9
Sankar 2013	-	Needs assessments, pilots or stakeholder consultations	The project interventions were developed in keeping with the especial needs of the population, and rooted in the conditions and circumstances of the area. It drew heavily from the understanding and opinions of local people. The results reflect the fruits of a highly contextualised program.	40

For peer review only - http://bmjopen.bmj.com/site/about/guidelines.xhtml

Shukla 2018	Anwari 2015	Leadership and supportive supervision	District Health Offices and DHCCs were less well established compared to the Provincial Public Health Directorates and PPHCCs; the ministry did not have adequate resources to equip them well. Despite these challenges, the leadership and involvement of the ministry in the intervention mattered. The provincial and district health governance leaders were inspired to improve their governance because the ministry leaders were interested in the pilot intervention.	14
Existing or changing favourable factors or contextual trends				
Admassie 2009	-	Good quality services and infrastructure	The impact of the programme on the proportion of children who got vaccines against<br><br>major childhood illnesses also varies according to supply side variables (see Table 6). The programme has larger effects in villages with one HEW and with better quality health posts.	441
Herrera-Almanza and Rosales-Rueda 2018	-	Health service access and availability	However, the fact that we find results on vaccination uptake is an indication that the health workers were somehow present in remote areas.	29
Admassie 2009	-	Socio-economic factors	This implies that encouraging and supporting primary schooling for girls and women will enhance the programme's impact on proportion of children vaccinated against major childhood illnesses.	441
Implementation improvements				
Mayumana 2017	Binyaruka 2017	Managing vaccine stockouts	The verification system under P4P also meant that district supervision was intensified, providing more opportunities for district managers to identify and address stock-outs of a wider range of drugs.	97

For peer review only - http://bmjopen.bmj.com/site/about/guidelines.xhtml

Reasons for intervention failure

Most studies that did not find a significant positive impact attributed the failure of the intervention to a specific cause. The most consistently reported reason for failure across all engagement types was *not accounting for contextual constraints*, though the exact nature of these reasons varied. Social norms such as misconception regarding immunisation and health worker attrition were reported as common challenges in interventions that were community engagement. Interventions that used engagement in design consistently reported poor access to health services and caregivers' skepticism regarding benefits of immunisation as the reasons for failure. Poor quality of health services was noted as an issue in interventions where engagement occurred in implementation.

The other consistently reported reasons for failure were attributed to inadequate *intervention features* and *implementation challenges.* Among the intervention features, inadequate duration, frequency or exposure to the intervention were the most notable reasons for failure across all engagement types. Though relatively less common, the nature of the community engagement itself was also attributed to failure in interventions that used engagement in implementation or as intervention. Among implementation challenges, disruption due to inadequate implementation instructions, difficulty in accessing the implementation sites due to geographic proximity and competing priorities of health workers were noted as some of the prevalent challenges (Supplementary Figure 94; Supplementary Table 13).

Another less consistently reported reason for failure was study design issues such as contamination between treatment and control other methodological challenges such as biased sampling frame and issues with enrolling study populations. The reasons for failure were largely based on authors' notes or experiences and individual interviews. We expect the authors to be a good source of this information because of their knowledge of causal linkages and assumptions made in the intervention theory of change. Focus group discussions and implementers' experiences supported these findings. The sensitivity analysis, which included qualitative papers with an assessment of score of 20 or higher, showed similar results. Except for one sub-theme which was dominated by one or two studies, other sub-themes were consistently distributed across the evidence base (indicated in Supplementary Table 13). Therefore, we consider the evidence to be of high strength.

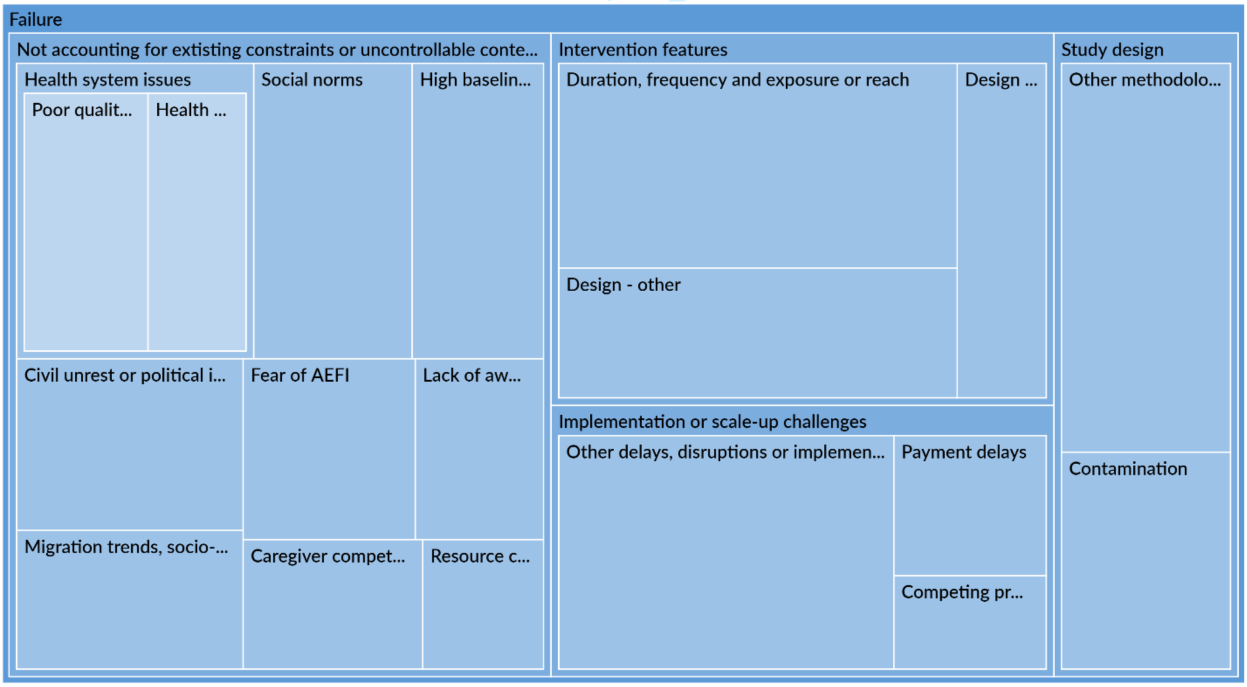

Supplementary Figure 94: Summary figure: reasons for failure

Supplementary Table 13: Comparison of the reasons for intervention failure by type of community engagement**.**

For peer review only - http://bmjopen.bmj.com/site/about/guidelines.xhtml

	**Community engagement as the intervention**	**Community engagement in design**	**Community engagement in implementation autonomy of the intervention**
**Not accounting for contextual constraints –** *health system issues*	Health worker attrition	Issues with accessing health services	Poor quality of services
**Not accounting for contextual constraints –** *prevalent social norms*	Fear of sterilization	Skepticism towards perceived value of immunisation	-
**Not accounting for contextual constraints –** *baseline immunisation coverage*	High baseline coverage	High baseline coverage	High BCG coverage
**Not accounting for contextual constraints –** *unrest, insecurity or other*	Civil unrest<br>Natural calamity	Political instability	-
**Intervention features –** *Duration, dose or frequency of intervention*	Inadequate intervention duration or exposure	Inadequate intervention frequency	Inadequate intervention duration or exposure
**Intervention feature –** *issue in design of engagement*	Lack of collective action	-	Elite capture
**Implementation challenges –** *disruptions or delays*	Resource constraint due to multiple programmes running at the same time	Fidelity issues	Staff turnover*
**Study design –**	Contamination due to similar programmes	Contamination due to similar programmes	Contamination between treatment and control

For peer review only - http://bmjopen.bmj.com/site/about/guidelines.xhtml

Contamination			

*Most of the references coded under this theme were from Olken 2014 and associated papers.

Community engagement as the intervention

Not accounting for contextual trends were the most consistently reported reason for failure. Social norms, health system issues, migration, civil unrest and fear of adverse events following immunisation were reported consistently within existing constraints. Most of the challenges related to social norms mentioned misconception about immunisation (Banerjee 2020) and limited decision-making powers of mothers (Gurley 2020) as the reason for low immunisation uptake. Civil unrest, political instability and natural calamities also hindered a few interventions from achieving intended impacts (Pramanik 2020; Morris 2004).

The other reasons for failure comprised issues related to *intervention features, implementation issues* and, to a lesser extent, *study design.* Issues with intervention feature ranged from inadequate duration or frequency of the intervention to certain aspects of community engagement failing to elicit a positive response among study participants (Pramanik 2020; More 2012). Implementation issues were consistently reported as reasons for failure. Some of the challenges mentioned were competing demands by different programmes running simultaneously (Carnell 2014), high staff turnover (Gurley 2020; Olken 2014) and low implementation fidelity (Oyo-Ita 2020). These issues are also discussed in detail under the 'uptake and fidelity challenges theme'.

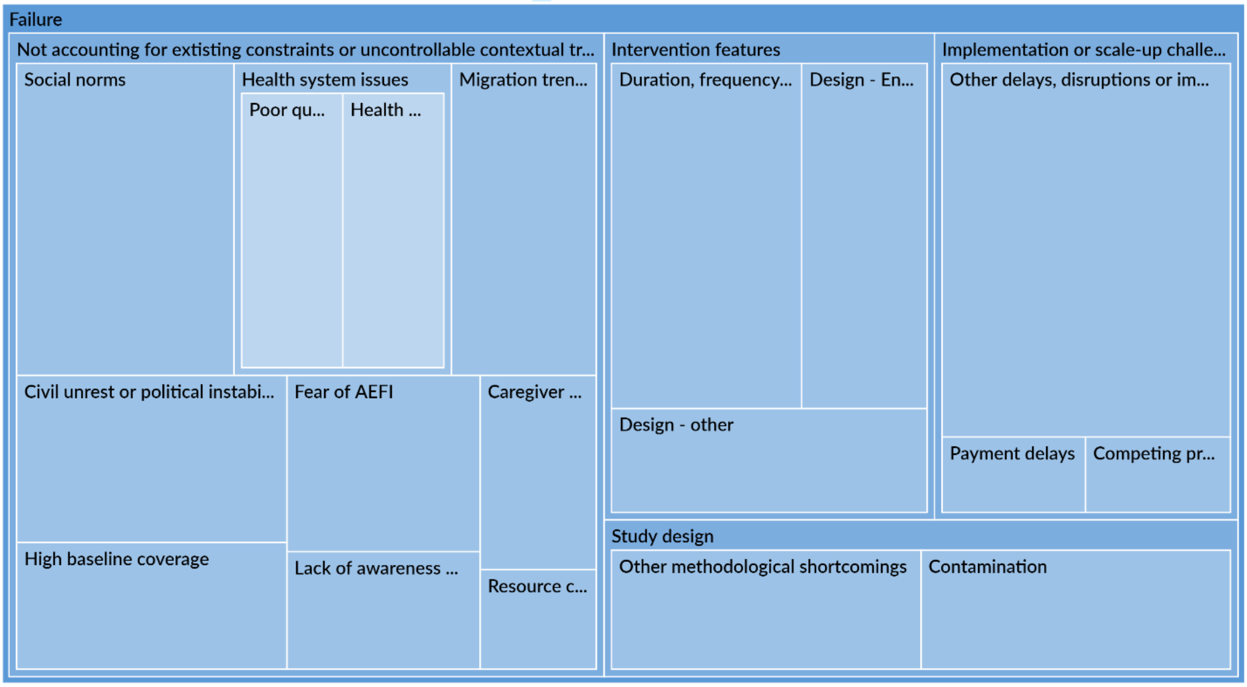

Supplementary Figure 95: Reasons for failure of interventions with engagement as intervention

Supplementray Table 14: Characteristic quotes of themes identified related to reasons for failure for interventions were community engagement.

For peer review only - http://bmjopen.bmj.com/site/about/guidelines.xhtml

Primary paper	Paper from which citation comes	Sub-theme demonstrated	Quote	Page number
Implementation or scale-up challenges				
Carnell 2014	-	Attrition of implementation personnel	The ESHE and RHB teams both faced personnel challenges in Amhara, which may have affected management and performance.	559
Gurley 2020	-	Infrastructure constraints	Community constraints around screening spaces and economic activities also introduced challenges.	73
Not accounting for existing constraints or uncontrollable trends				
More 2012	-	Lack of access to health care	The third issue was the complexity of urban health care. Antenatal care was the norm and the nadir for institutional delivery in trial clusters was 75%. Around 57% of antenatal care and 30% of deliveries were in the private sector (this in a slumdwelling population). Open access to private providers, and to institutions at all levels of the public sector hierarchy, is a challenge to systematic health care delivery. Our findings confirmed the tendency to bypass public maternity homes, which should handle uncomplicated deliveries, in favour of tertiary institutions.	E1001257
Pramanik 2020	-	Natural calamity	Assam has a wonderful collection of festivals that are of the utmost importance. And more tragically when the Brahmaputra River floods the priority is survival. The project simply has to adapt to these ebbs and flows, but there is a pressure to deliver to a schedule that inevitably leads to a box ticking approach that is inconsistent with the development of ownership by the community.	68

For peer review only

Oyo-Ita 2020	-	Fear of AEFI	These responses are indications that fear of side effects can hinder vaccine uptake. This may have contributed to non-impact on the proportion of fully vaccinated children in this study, as the TRLs in the post-intervention qualitative study still mentioned this as a common reason for poor uptake of vaccines.	32
Intervention features				
Olken 2014	Grayman 2013	Engagement failure due to elite capture	In general, researchers discovered that Generasi provides opportunities for elites to retain and fortify their social standing. As brokers delivering CDD resources into their communities, local elites leverage Generasi to accumulate additional status for themselves. Without exception, every Generasi actor that the researchers met held other leadership roles in their community and maintained close ties with the local structures of village and sub-district governance.	48
Robertson 2013	-	Inadequate exposure to the intervention	Our study was limited by the short intervention period. Whether the effects of the programmes would change with time is unclear. The follow-up survey was done 2 months after interventions had finished, so fear of penalties should not have biased responses from CCT households, although the effects of the programmes could have attenuated by the time of the survey.	8
Study design				
More 2017	-	Contamination due to other programmes	although we found no evidence of contamination of control clusters by the intervention, government schemes and the activities of municipal and non-governmental providers might have improved health in control clusters.	E347

Community engagement in design

Most consistently reported reasons for failure of interventions in which communities were engaged in the design broadly fell under *not accounting for contextual constraints.* Among these, social norms were the most consistently reported reason with vaccine hesitancy being one of the driving forces behind immunisation refusal

For peer review only - http://bmjopen.bmj.com/site/about/guidelines.xhtml

(Banerjee 2020; Gurley 2020). Other reasons for failure within this theme were high baseline immunisation coverage, health system issues related to quality of services, and civil unrest or political instability. Prevailing socio-economic trends, lack of awareness, fear of AEFI and caregiver competing priorities were also attributed to intervention failure, though less consistently reported as compared to other factors.

Besides existing constraints, a few studies also attributed failure *intervention features* with the insufficient duration, frequency and exposure of intervention as a possible reason for failure. A much smaller subset of studies also referred to issues related to study design and implementation or scale-up challenges.

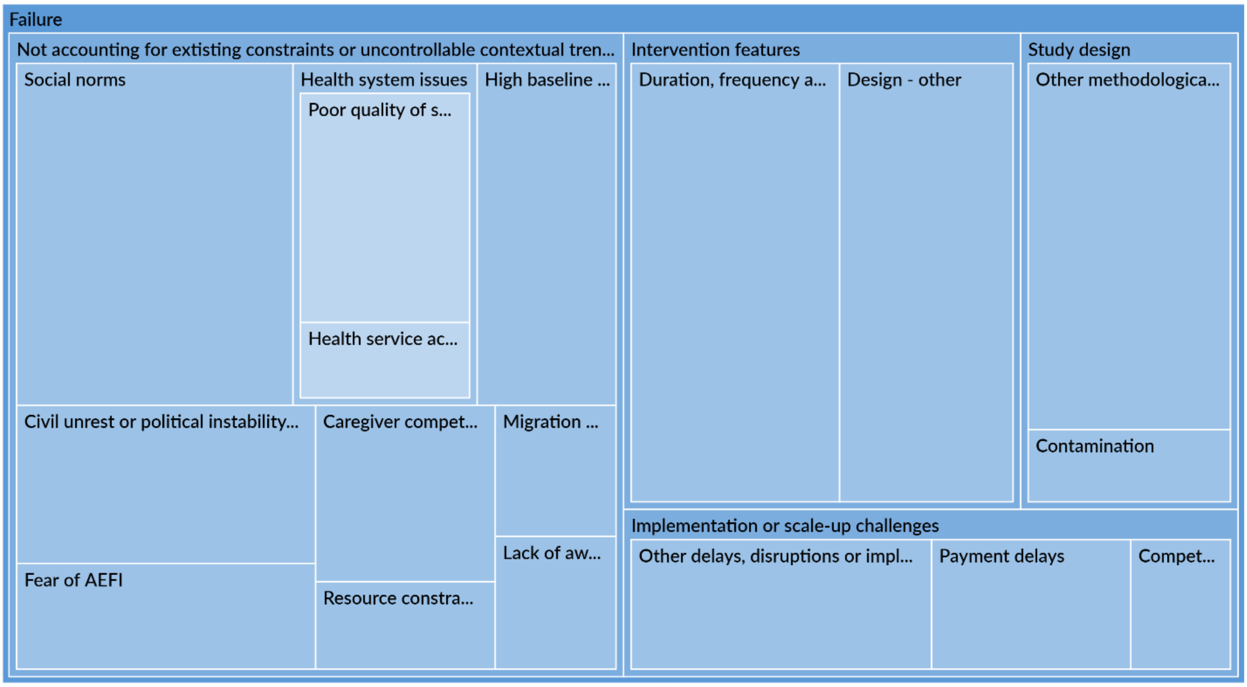

Supplementary Figure 96: Reasons for failure of interventions with engagement in design

Supplementary Table 15: Characteristic quotes of themes identified related to reasons for failure for interventions that used community engagement in their design.

**Primary paper**	**Paper from which citation comes**	**Sub-theme demonstrated**	**Quote**	**Page number**
Implementation or scale-up challenges				
Gurley 2020	-	Staff turnover	On the implementer side, staff turnover at NYST and research activities introduced delays in video production.	73
Engineer 2016	-	Payment delays	Problems with implementation likely dampened any potential effect. The scheme was rolled out in phases, but there were some delays, particularly with the initial payments.	456

For peer review only - http://bmjopen.bmj.com/site/about/guidelines.xhtml

Not accounting for existing constraints or uncontrollable trends

Gibson 2017	-	High baseline immunisation coverage	SMS reminders were probably not effective at improving full immunisation and vaccine-specific coverages in this study because of high baseline coverage levels and because SMS reminders might not have addressed the demand side deficiencies in this study area.	e436
Banerjee 2020	-	Caregiver competing priorities	Across districts, there are 'hard-to-convince' populations who are not affected by incentives. Some ANMs listed specific populations groups they felt continued to be unaffected by the programme, and the incentives specifically, such as migrant workers, daily wage workers and the Muslim community. In Panipat, the top reason given by ANMs for resistance to immunisation by the few still unconvinced (migrants, Muslims) was the fever brought on as an after-effect by a certain vaccine (penta)30 and direct costs involved for daily wage workers.	92
Gurley 2020	-	Social norms	It is worth noting that in contrast to the high intent recorded through the household survey, the process evaluation indicated there were individuals who actively chose to not vaccinate their children. This may be a function of social desirability bias in the household survey, though we posit that it may also reflect family constraints or logistical barriers that prevent a mother from taking a vaccine decision regardless of own personal intention to vaccinate. As noted elsewhere, mothers are not the sole or primary decision-makers and other family members reported lower intent to vaccinate.	75

Intervention features

Siddiqi 2020	-	Inadequate exposure to the intervention	Our findings also provide evidence of the strong reliance on the immunization card being the established immunization recall method and we may also postulate that the short duration of the study did not provide enough time to 'institutionalize' the use of the bracelets.	12

For peer review only - http://bmjopen.bmj.com/site/about/guidelines.xhtml

Okoli 2014	Baba-Ari 2018	Communication failure	Experiences of the beneficiaries with the programme point to problems with programme communication as they had a poor understanding of the aims of the CCT programme aside from receiving its immediate cash benefits and as such might revert to their original behaviours after the programme ends.	940
Study design				
Domek 2019	-	Selection bias in study enrollment	This may have been partly because our study population had higher baseline immunization coverage than we were adequately powered to assess. These higher than expected completion rates were likely due in part to a selection bias in enrolling children as they presented for their first immunization visit, which would have unintentionally excluded children who either presented significantly delayed or not at all for vaccines.	6197

Community engagement in implementation autonomy of interventions

Fewer studies under this engagement classification reported reasons for failure as compared to success. Among the reasons cited for failure, those related to *not accounting for contextual constraints* were the most consistently reported followed by *implementation challenges*. Most of the references to implementation challenges or failures were made by Olken 2014 and its associated studies which discussed multiple implementation barriers to a block-level grant intervention. Though less consistently reported, some studies also mentioned issues with intervention features or study design as the reasons for intervention failure. Highlighting the failure of community engagement, Olken 2014 (Grayman 2013) discussed how elite capture wherein local community leaderships and elites leveraged the intervention to retain social standing became an issue for the programme.

For peer review only - http://bmjopen.bmj.com/site/about/guidelines.xhtml

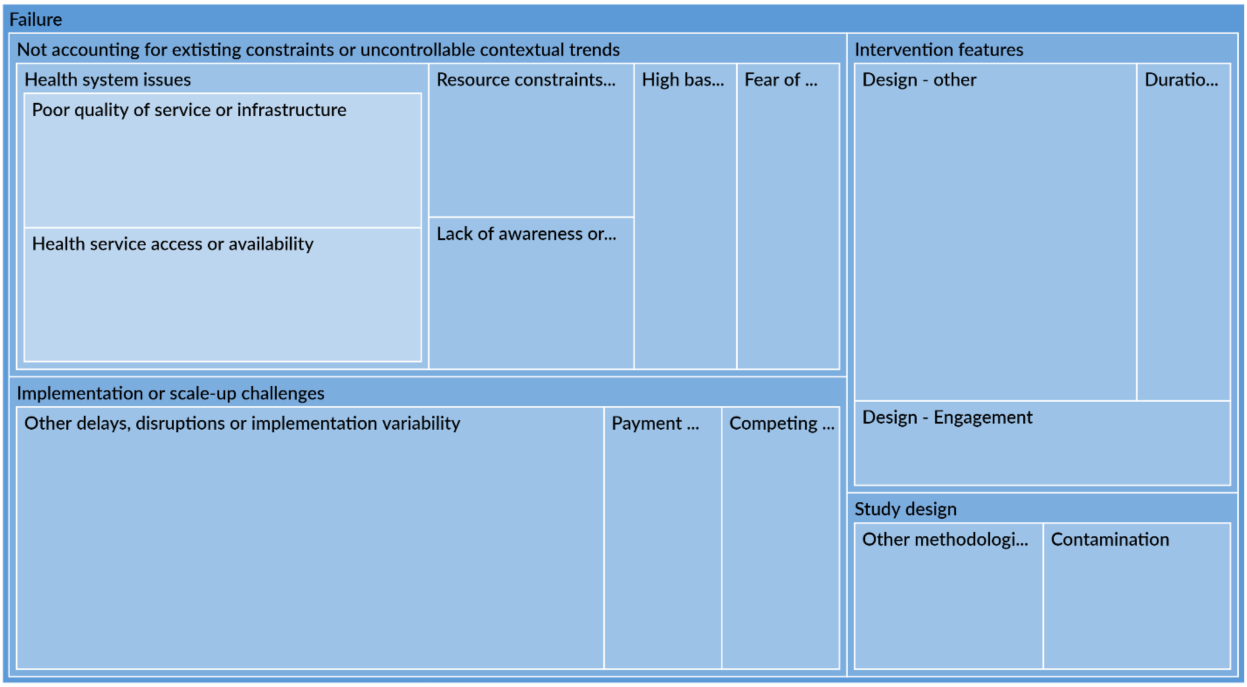

Supplementary Figure 97: Reasons for failure of interventions with engagement in implementation autonomy

Supplementary Table 16: Characteristic quotes of themes identified related to reasons for failure for interventions that used community engagement in implementation.

**Primary paper**	**Paper from which citation comes**	**Sub-theme demonstrated**	**Quote**	**Page number**
Implementation or scale-up challenges				
Oyo-Ita 2020	-	Low implementation fidelity	The non-impact on up-to-date vaccination could have been accounted for by the weak link in the intervention caused by not sharing data directly with community members as planned.	38
Olken 2014	World Bank 2018	Staff turnover	High village-level staff turnover may partially explain why Generasi staff in the villages are so unfamiliar with the bonus system. The entire Generasi team in Desa Lelaok (Petis subdistrict, Pamekasan district) and the PK in Desa Rampe (Nelle subdistrict, Lembata) had never heard of the bonus system, but all of them had started within the past year.	38

For peer review only - http://bmjopen.bmj.com/site/about/guidelines.xhtml

Webster 2019	-	Competing priorities of health workers	Although the proportion of respondents reporting a home visit by a VHT decreased from baseline to endline in both control and intervention clusters the proportion of visits where immunization was the reason for the visit increased. The decrease in VHT home visits may be due in part to observations that in the latter months of the intervention, VHT services were sought by other organisations involved in the refugee response in some of the project areas, who paid VHTs higher allowances.	47
Not accounting for existing constraints or uncontrollable trends				
Okeke 2017	-	Resource constraints or scarcity	Lack of electricity and water were also frequently cited as problems.	43
Okeke 2017	-	Poor quality of services or infrastructure	The data suggest that part of the reason why the program did not have larger impacts is that other dimensions of quality did not improve. For example, clinic infrastructure in many cases remained poor, as did availability of drugs and supplies.	49
Intervention features				
Webster 2019	-	Intervention design failure	The main mechanisms of change identified were improved accessibility to immunization services through increased numbers of vaccination outreaches, increased VHT motivation through monthly allowances together with social motivation in outdoing each other in defaulter tracing, use of community resources to achieve outcomes that is the support of local community leadership; and increased interaction between health workers and HCWs and VHTs at monthly VHT/HCW meetings. These identified mechanisms of change were predominantly due to supplementary activities that were implemented in both intervention and control clusters.	49
Study design				

For peer review only - http://bmjopen.bmj.com/site/about/guidelines.xhtml

Webster 2019	-	Contamination between treatment and control	There was some contamination of control clusters, largely due to transfer of health workers from intervention to control sites. At two control sites it was evident that the health workers sought to set up a similar defaulter tracing approach.	49

Reasons for heterogenous impacts

A small subset of studies is also coded under the theme *heterogeneous impacts*. Even though they reported overall significant positive quantitative findings, these studies discussed their heterogenous or non-uniform results with respect to not achieving the intended impact among certain subgroups, study sites or reported null results for some of the immunisation-related outcomes. Each of these studies offer a slightly different perspective on their heterogeneous results. For instance, Adamu et al. 2019 and Carnell et al. 2014 postulate that the characteristics of individuals involved in the intervention implementation, contextual factors or implementation processes could have led to partial intervention success or measurable positive gain in only select study sites. Interestingly, both Banerjee et al. 2020 and Gibson et al. 2017 highlight very different challenges with respect to their interventions comprising incentives to caregivers. Banerjee et al. describe how incentives designed to motivate caregivers did not work on Muslim minorities due to high vaccine hesitancy which stemmed from the belief that vaccination causes sterlisation. On the other hand, the Gibson study reported that their SMS reminders did not work in the absence of incentives, implying that reminders alone were insufficient to encourage behaviour change among caregivers and result in immunisation uptake. This shows that the benefit of providing incentives may not be consistent across different contexts and uptake may be influenced by other socio-economic factors. Both studies further elaborate the limits to which incentives can address deep rooted mistrust of vaccination among certain subgroups. Overall, the discussion on these studies provide important insights about how external factors, implementation variability or invalid theory of change assumptions may contribute to partial intervention success or failure. We also looked through the literature for *unintended impacts* but there was insufficient information to draw any broader conclusions.

Uptake and fidelity challenges

Uptake and fidelity challenges were generally identified by authors through primary data or personal experience and knowledge of the intervention. Because this theme relates to how the project was implemented, we expect that authors are a good source of this information. Individual interviews and focus group discussions supported the findings. Themes were broadly consistent in the full analysis and in sensitivity analysis in which on the qualitative papers with a quality assessment score greater than 20 were included. However, there are several instances in which a single sub-theme is dominated by one or two studies. These are indicated in Supplementary Table 17 and discussed in the text. Given this, we believe that the evidence on uptake and fidelity challenges has only moderate strength.

The most consistently reported challenges were related to administration, mobilization, fidelity, and contamination (Supplementary Figure 98; Supplementray Table 17). Administrative challenges were cited consistently across all three types of interventions, but their nature varied. Although technical issues and politics plagued interventions that used community engagement in the design of interventions or as the intervention, these issues were not common in interventions that used engagement in their implementation. Staffing was a challenge in interventions that used engagement in implementation or as the intervention. Implementation fidelity was mentioned as a challenge by all three types of interventions. In all cases, the most consistent issue was that realities on the ground forced changes to the intervention. The issue of mobilizing people into the intervention appears to be common; however, this category is dominated by two papers (Pereze 2020 and Olken 2014) which cite a variety of mobilization challenges. As such, the evidence on this theme is somewhat weak. Contamination was also consistently reported. For the most part, it was mentioned due to its absence. Interventions whose goal was

For peer review only - http://bmjopen.bmj.com/site/about/guidelines.xhtml

engagement discussed this topic much more than interventions with engagement in their design or implementation.

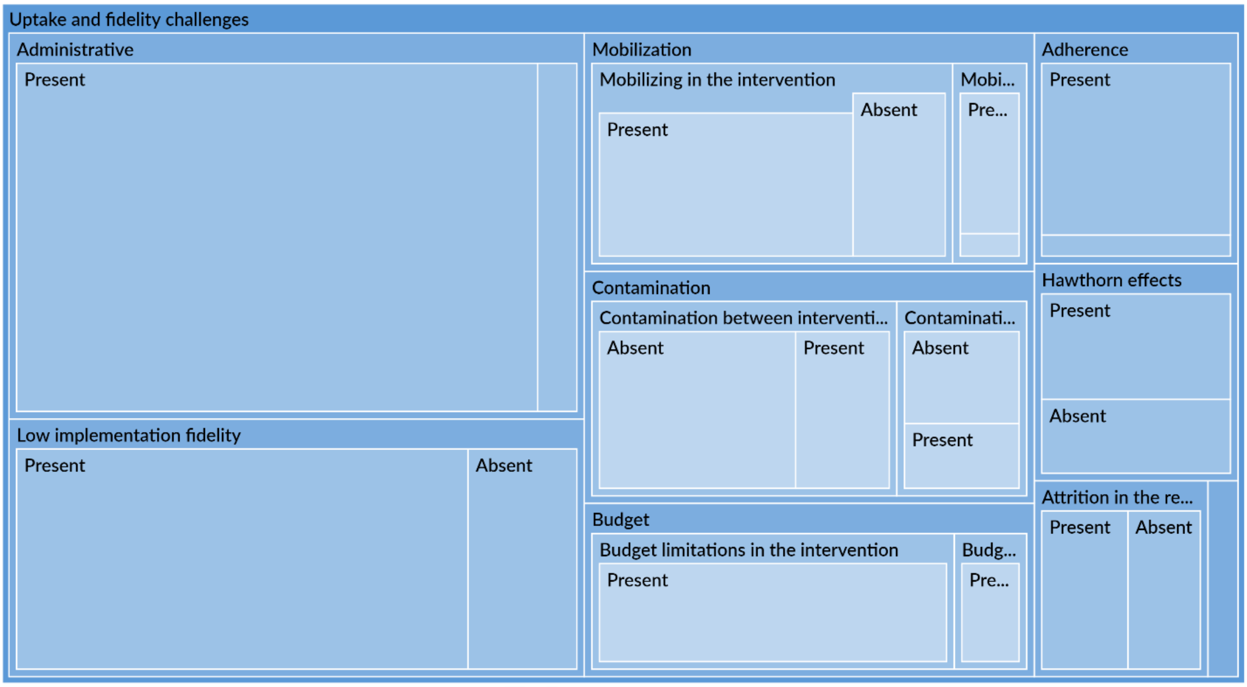

Supplementary Figure 98: Summary figure: uptake and fidelity challenges[2]

Supplementary Table 17: Comparison of the challenges faced by interventions using different types of community engagement. Numbers reflect relative frequency.

	**Community engagement as the intervention**	**Community engagement in the design of the intervention**	**Community engagement in the implementation of the intervention**
**Administrative challenges**	Technical issues<br>Politics<br>Coordination<br>Staffing	Technical issues<br>Politics<br>Bureaucracy<br>Security	Funding disbursement<br>Communication across stakeholders<br>Staffing
**Mobilization challenges**	Gender based religious and cultural norms prevented participation***	Recruiting people into the intervention due to work, distance, and competing priorities*	Recruiting facilitators and participants into the intervention due to other opportunities, miscommunication of intervention expectations, declines in incentives, caregiving obligations, and more**
**Fidelity challenges**	Failure to account for on the ground realities resulted in changes or failures in intervention	Failure to account for on the ground realities resulted in changes or failures in intervention implementation	Failure to account for on the ground realities resulted in changes or failures in intervention implementation

[2] Coding is presented in a present/absent format because, in some cases, authors explicitly stated that a challenge was not encountered.

For peer review only - http://bmjopen.bmj.com/site/about/guidelines.xhtml

		implementation. Improper or insufficient supervision was also common.			
**Contamination**		Authors were more likely to discuss the absence of this issue than to report upon its presence.	Authors were more likely to discuss the absence of this issue than to report upon its presence.		
**Budget restrictions**					Inadequate or delayed pay resulted in activities not being implemented.

*Most of this evidence came from a Pereze 2020 which cited multiple reasons for mobilisation challenges
**All evidence on challenges came from Olken 2014
***All evidence from Olken 2014 also applies to this group as Olken 2014 falls into both categories

Community engagement as the intervention

Discussions of *administrative challenges* consistently related to coordination, technical issues, staffing, and politics. The single study that reported on the absence of certain administrative issues also reported on the presence of many others. This is likely because it was a 3ie report, which tend to be quite detailed and include significant qualitative components.

Issues with the faithful *implementation* of these interventions were consistently the result of improper supervision and management or a failure to account for practicalities on the ground. We found one case where the administrative challenges directly led to low implementation fidelity. However, a non-trivial proportion of studies reported statistics indicating high fidelity.

The studies that discussed contamination largely did so in order to demonstrate that contamination was not a concern. Authors consistently focused on the issue of contamination between intervention and control sites. This was often addressed with vague statements. Distance was largely used to avoid contamination.

Discussion of challenges in mobilization interventions was dominated by a single source (Olken et al 2014 also discussed the engagement in implementation section). However, Rahman 2016 also discussed several mobilization issues that resulted from religious and cultural norms, largely around gender, preventing participation.

For peer review only

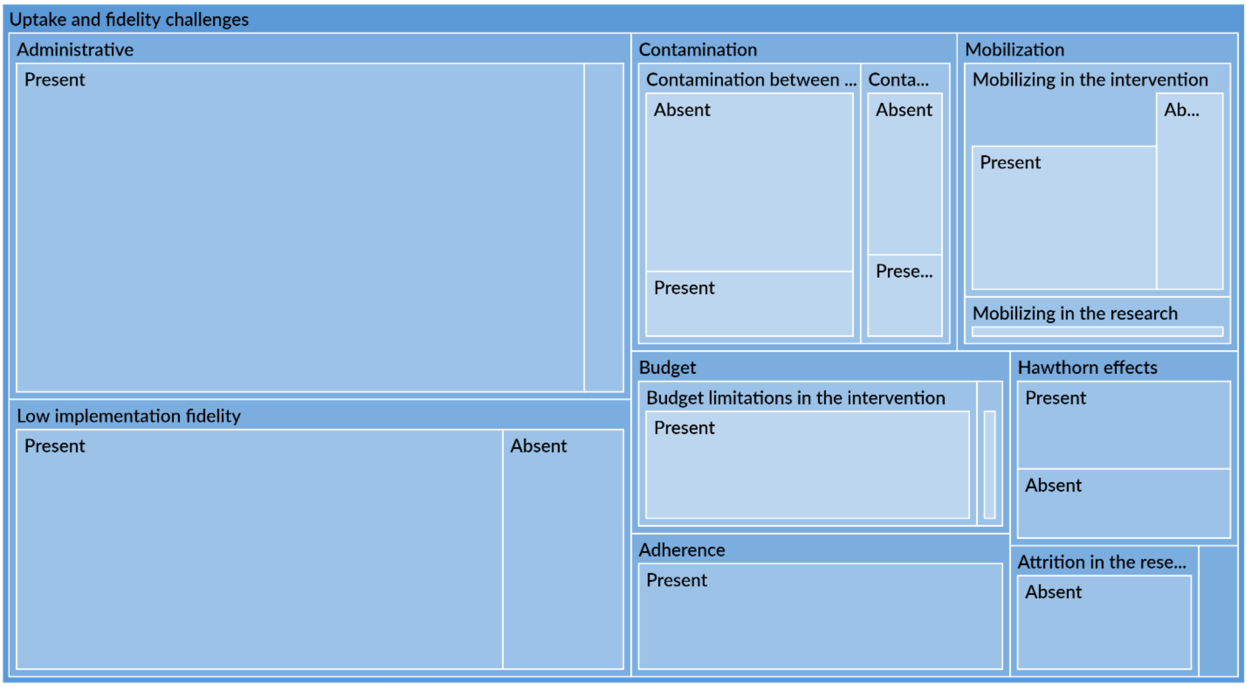

Supplementary Figure 99: Engagement as intervention

Supplementary Table 18: Characteristic quotes of themes identified related to uptake and fidelity challenges for interventions were community engagement.

**Primary paper**	**Paper from which citation comes**	**Sub-theme demonstrated**	**Quote**	**Page number**
*Administrative challenges**				
Assengai et al 2018	Khuzwayo et al 2017	Coordination challenges	Respondents report a lack of communication about visit scheduling.<br><br>This is compounded by dual reporting lines in many parts of the country, where CHWs remain linked to and receive stipends through NGO intermediaries, while being accountable to PHC facility managers.	3
Demilew et al 2019	Demilew et al 2020	Technical and political challenges	The Mobile Intervention was left from being implemented because of Internet service inaccessibility that resulted from the state of emergency declared by the Ethiopian government.	23

For peer review only - http://bmjopen.bmj.com/site/about/guidelines.xhtml

Banerjee et al 2020	Banerjee et al 2020	Political challenges	This study has faced various degrees of support from our primary government stakeholder since its inception, and this is the main reason why the timeline has been extended multiple times over the past seven years (when discussions with the Haryana government first began). We faced issues in continuity and buy-in especially when higher-ranking officers were transferred.	69
		Absence of administrative barriers	Given that J-PAL South Asia was also responsible for implementation, we recruited a dedicated team with relevant experience to do this. As a result, we were acutely aware of what was happening in the field, and able to identify and resolve issues quickly.	71
Implementation fidelity				
Biemba et al 2016	Phiri et al 2017	Inadequate supervision	Supervision and mentorship is crucial to optimizing the skills of CHAs, but this study outlines that supervision is not always implemented as intended and that in the absence of regular supervision	7
Morris et al 2004	Morris et al 2004	Expectations did not match realities on the ground	The service-level package was not implemented in accordance with protocol because no legal means could be identified of transferring resources	2034
Demilew et al 2019	Demilew et al 2020	Administrative challenges caused implementation challenges	This intervention was designed on the expectation that the text message portion could be rolled out in tandem with the ECIIN, an immunisation tracking program that planned to have HEWs report immunisation dates, mother's data, child's data, using text messages. Due to data issues and conflict in the region ECIIN was discontinued.	34
Gurley et al 2020	Gurley et al 2020	High fidelity	The types of screenings were conducted as planned, with 446 mothers group screenings, 445 VHND screenings, 223 men's screenings, and 444 HTR screenings (Table 6). Within a single village, this translated to an average of 12 sessions held in mothers groups, 12 held in VHNDs, 12 held in HTR areas, and 6 held in men's groups.	23
Contamination				
Moore et al 2017	Moore et al 2017	Absence of contamination	Our intervention and control groups were generally similar, with high coverage and fidelity to planned activities and negligible contamination.	e347

For peer review only - http://bmjopen.bmj.com/site/about/guidelines.xhtml

Paraminik et al 2018	Paraminik et al 2020	Distance reduced contamination	To mitigate potential contamination between intervention and control villages, we attempted to ensure that the intervention and control villages are sufficiently far apart from one another.	25

*Challenges related to mobilisation are not presented because this information largely came from a single paper making the identification of characteristic issues *across* papers impossible.

Community engagement in the design of the intervention

Among interventions that used community engagement in their design, the most consistently reported *administrative challenge* related to technical issues, largely surrounding phones and internet connections (Supplementary Table 17). However, other administrative challenges were related to combinations of politics, bureaucracy, and security concerns. The single paper that indicated a lack of administrative challenges reported successful syncing to the server, but also reported on other technological issues.

Mobilization challenges largely related to recruiting people into the intervention. Most of this information came from a 2020 (Pereze et al 2020) paper which presented information on the implementation fidelity and acceptability of an intervention that combined in-person and digital education to promote vaccination (Johri 2018). Authors describe challenges with participants being unable to attend due to work, distance, and competing priorities. Limited access to phones made mobilization difficult as well.

Acknowledgement of *low implementation fidelity* was consistently accompanied by discussions of why changes were made or the implications of these changes for results. Generally, changes in the intervention plan were forced due to practical constraints that had not been accounted for and were expected to result in an attenuation of impacts. Reports of high fidelity were succinct and responded to concerns a reader may be expected to have.

Contamination can occur between the intervention and control or due to ongoing activities of other programs. The former of these was reported more consistently than the latter. Authors were more likely to discuss the absence of this issue than to report upon its presence. Some offered explanations as to why contamination was not a concern or steps taken to avoid contamination. Others reported on results related to measures of contamination.

For peer review only - http://bmjopen.bmj.com/site/about/guidelines.xhtml

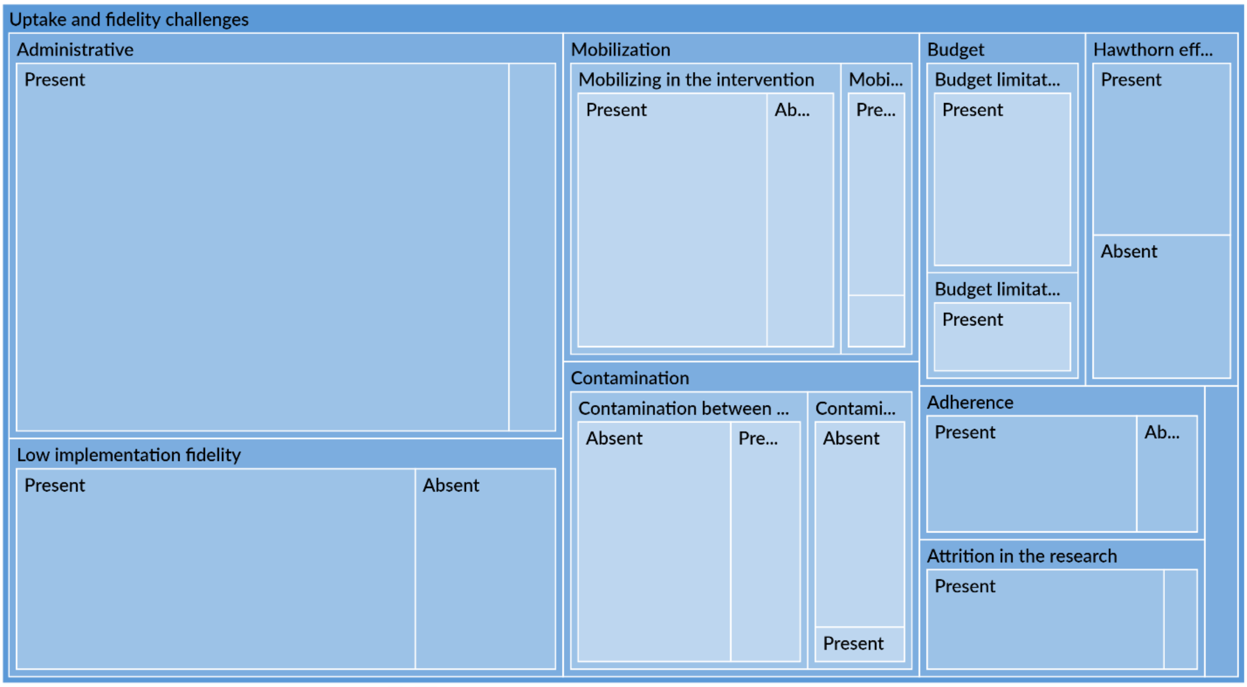

Supplementary Figure 100: Engagement in design

Supplementary Table 19: Characteristic quotes of themes identified related to uptake and fidelity challenges for interventions that used community engagement in their design

**Primary paper**	**Paper from which citation comes**	**Sub-theme demonstrated**	**Quote**	**Page number**
*Administrative challenges**				
Nagar et al 2020	Nagar et al 2020	Technological problems	The most prevalent issues reported by ANMs using the KB App included having to reenter data after an app crash (60.9%), having issues with scanning the pendant (65.2%), and issues with the application crashing (56.5%), and time required to enter data (33.3%).	138
Modi et al 2019	Modi et al 2015	Technological problems	Limited phone memory occasionally created technology-related issues, with the increasing requirement to store data on the mobile phone.	10
		Absence of technological problems	The failure rate, defined as the proportion of forms that failed to get synced with the server, was <1%.	266
Implementation fidelity				

For peer review only - http://bmjopen.bmj.com/site/about/guidelines.xhtml

Gruley et al 2020	Gruley et al 2020	Reason changes were implemented	Last, due to resource constraints, the project was not able to implement the intervention component for sharing videos from phone to phone using Bluetooth technology.	22
Engineer et al 2019	Engineer et al 2016	Implications of low fidelity for the research	Problems with implementation likely dampened any potential effect. The scheme was rolled out in phases, but there were some delays, particularly with the initial payments.	456
Banerjee et al 2010	Banerjee et al 2010	High implementation fidelity	Review of records showed that of 1336 planned camps, 95% (1269) took place.	2
Contamination				
Borkum et al 2014	Borkum et al 2014	Absence of contamination with other interventions	Because FLWs and households in the treatment and control groups received non-TBGI Ananya program interventions that were being implemented simultaneously across Begusarai, the RCT was designed to measure the value-added of TBGI beyond these other Ananya interventions. It was not designed to measure the impact of TBGI introduced in isolation.	6
Nagar et al 2018	Nagar et al 2016	Steps to avoid contamination	a cluster randomised approach further allowed non-contamination of social signaling interventions within the contacts of a given village	34
Johri et al 2018	Pereze et al 2020	Little contamination observed	According to the analysis of the records, only people who belonged to the intervention group attended the community meetings. The people who communicated with or received calls from the IVR platform were from the intervention group, except for one (1/166) control group member.	15

*Challenges related to mobilisation are not presented because this information largely came from a single paper making the identification of characteristic issues *across* papers impossible.

Community engagement in the implementation autonomy of the intervention

Administrative challenges in interventions that used engagement in their implementation largely related to funding disbursement, communication across stakeholders, staffing issues (Supplementary Table 17). One study (Bjorkman et al 2009) reported that they did not believe administrative differences affected results. This was

For peer review only - http://bmjopen.bmj.com/site/about/guidelines.xhtml

coded as the absence of an administrative challenge,but does not mean that administrative issues were not encountered.

Implementation fidelity appears to have been more of a challenge for these interventions than for those that leveraged community engagement in their design. These issues were consistently related to the intervention not functioning as expected. In some cases, this was because the reality on the ground did not reflect expectations. However, two studies that reference challenges with implementation fidelity also report upon successes in implementing according to plan.

All reports of challenges *mobilising* participants into interventions that were implemented by the community came from a paper related to an intervention that used block grants to support maternal and child health and education (Olken 2014). Challenges were encountered in the recruitment of both facilitators and participants. These related to other opportunities, miscommunication of intervention expectations, declines in incentives, caregiving obligations, and more. However, Herrara-Almanza 2018 report that community health workers felt strong responsibility for the wellbeing of their communities and attrition was low.

Interventions that used engagement in their implementation also experienced significant *budgetary constraints*. Almost universally, these constraints were the result of inadequate or delayed pay. Often, this resulted in activities not being implemented.

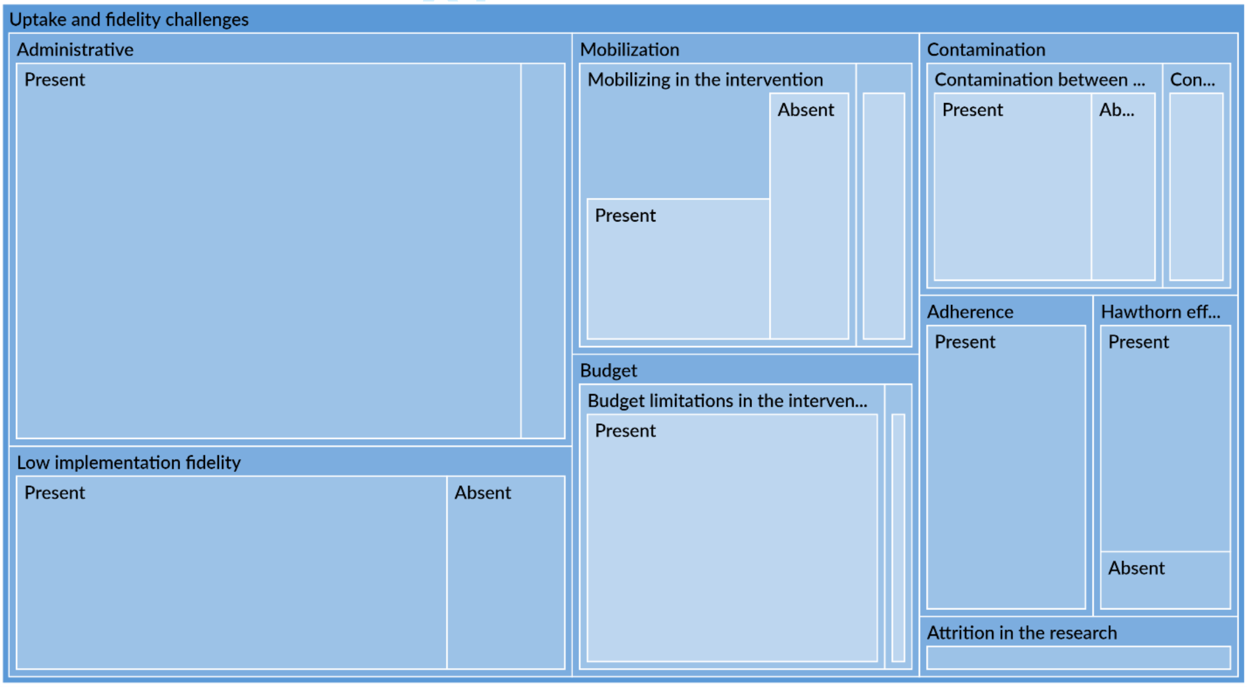

Supplementary Figure 101: Engagement in implementation autonomy of the intervention

Supplementary Table 20: Characteristic quotes of themes identified related to uptake and fidelity challenges for interventions that used community engagement in implementing the intervention.

**Primary paper**	**Paper from which citation comes**	**Sub-theme demonstrated**	**Quote**	**Page number**
Administrative challenges*				
Olken 2014	Febriany et al	Delays in funding	The most frequent complaint by FDs	14

For peer review only - http://bmjopen.bmj.com/site/about/guidelines.xhtml

	2011		regarding barriers to program implementation was the delays in aid disbursement.	
Mayumana et al 2017	Chimbutu et al 2011	Stakeholder communication problem	The NTPI"s programme, however, did not start in 2007 as planned… The major reason for this is on the implementation challenges between the two partners. The MOHSW preferred an accelerated approach while NORAD preferred an implementation framework to be designed by the Ifakara Health Institute (IHI).	16
Oyo-Ita 2020	Oyo-Ita 2020	Staffing and bureaucracy challenges	Low staffing of facilities constrained health staff from deploying the defaulters' register. Some complained that they had many registers other than the defaulters' register to fill.	18
Implementation fidelity				
Oyo-Ita 2020	Oyo-Ita 2020	Inconsistent implementation	The community engagement did not use town hall meetings as planned. Only one town hall meeting was held.	18
			The TRL training intervention was carried out as planned.	17
Okeke et al 2017	Okeke et al 2017	Expectations did not match realities on the ground	Contrary to the role of the states outlined in the MOU, policymakers in Enugu state reported playing no role in the implementation of the scheme. One suggested that the division of responsibility drawn up in the MOU did not align with the setup of health care in Nigeria given that the state government has responsibility for secondary care only.	50
Mayumana et al 2017	Olfasdottir et al 2014	Intervention did not function as expected	Firstly, the work load on staff is considerably higher than they are contracted for, which is not reflected in the remuneration; payments are delayed and overtime and eligible allowances are not always paid causing demotivation among staff.	7
		Intervention was implemented according to clear standards	The direct and transparent payment of funds, as well as more frequent contact with their managers was reported to enhance trust and improve the relationship between health workers and	64

For peer review only

			their managers.	
Budgetary constraints				
Okeke et al 2017	Okeke et al 2017	Pay was severely delayed	Some local government don't have the money to pay and they have not paid for a period of time. Most of them [midwives] have even finished their service and gone out without receiving any penny from the local government.	41
Mayumana et al 2017	Mayumana et al 2017	Activities were suspended	According to the qualitative data one of the obstacles for providing more frequent supervision visits was a lack of financial resources: We do supervision every quarter. Our plan is to do supervision monthly but due to limited budget we haven't yet done this.	6

*Challenges related to mobilization are not presented because this information came from a single paper making the identification of characteristic issues *across* papers impossible.

Sensitivity analysis for qualitative evidence

We carried out a sensitivity analysis in which we considered only the 17 qualitative studies associated with 12 impact evaluations that had a risk of bias assessment score of 20 or higher. Broadly, the themes that emerged from this analysis were consistent with those of the full analysis. We did not have enough qualitative evidence to conduct a sensitivity analysis by engagement type. An important caveat of this analysis is that nearly half of the papers rated as high "quality" were associated with three impact evaluations. The panel figures below provide comparison of the emerging themes in the full analysis versus the sensitivity analysis by comparing hierarchy charts related to barriers, facilitators, reasons for project success or failure, and uptake and fidelity challenges.

Barriers to immunisation

The most common barriers were broadly similar across the full analysis and the sensitivity analysis. *Behavioural, social and practical barriers faced by caregivers* were the most consistently reported barriers followed by *constraints in delivery of immunisation services* (Supplementary Figure 102). The most common barriers faced by caregivers were unavailability of immunisation services, financial constraints and social norms.

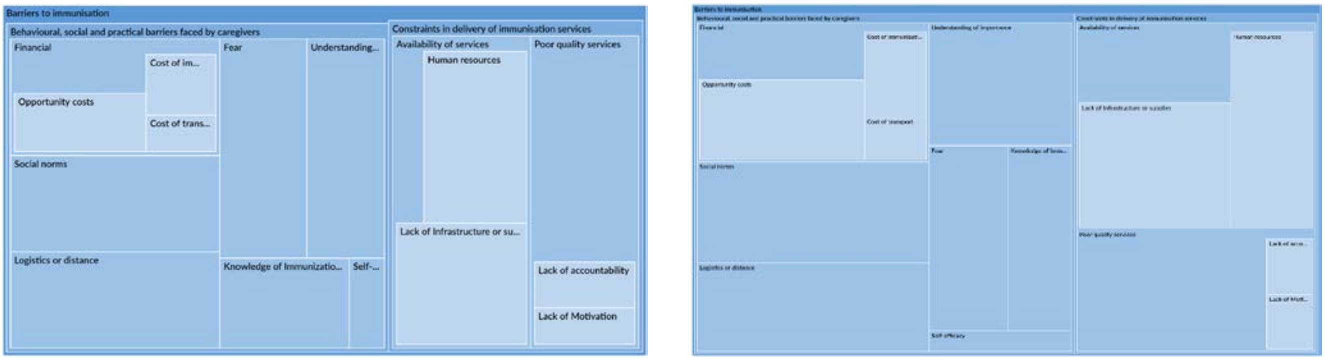

Supplementary Figure 102 (a): Full analysis *(b): Sensitivity analysis*

For peer review only - http://bmjopen.bmj.com/site/about/guidelines.xhtml

Facilitators of immunisation

The most common facilitators of immunisation were broadly similar across the full analysis and the sensitivity analysis. *Behavioural, social and practical factors faced by caregivers* were the most consistently reported facilitators of immunisation in a given context followed by good *delivery of immunisation services* (Supplementray Figure 103). The most consistently reported facilitators were availability of immunisation services, enabling social norms and caregivers' understanding of importance of immunisation.

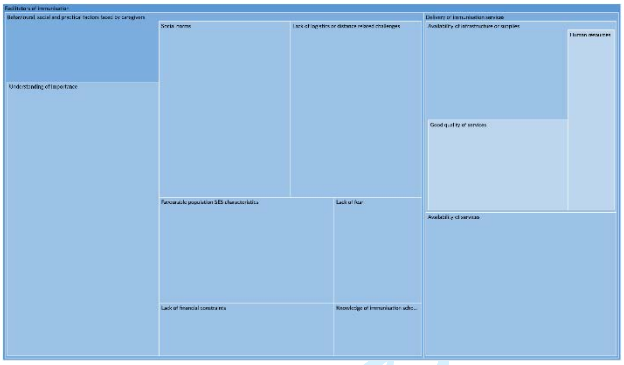

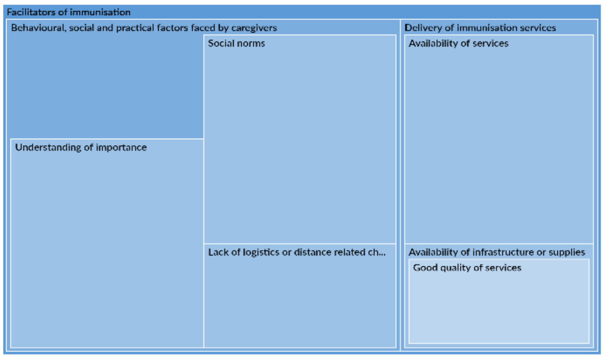

Supplementary Figure 103 (a): Full analysis *(b): Sensitivity analysis*

Reasons for intervention success

The most consistently reported reasons for success were broadly similar across the full analysis and the sensitivity analysis. Success was consistently attributed to *intervention features* including leadership and supportive supervision, community engagement, health worker training, incentives and customisation to local context. Though not as common, success was also attributed to *existing or changing favourable characteristics* within a given context such as positive participant views, enabling social norms and availability of and access to good quality health services. Given that a very small subset of papers were included in the sensitivity analysis, a few themes that emerged in the full analysis were missing in the sensitivity analysis. Supplementary Figure 104 provides the hierarchy charts comparing the two analyses.

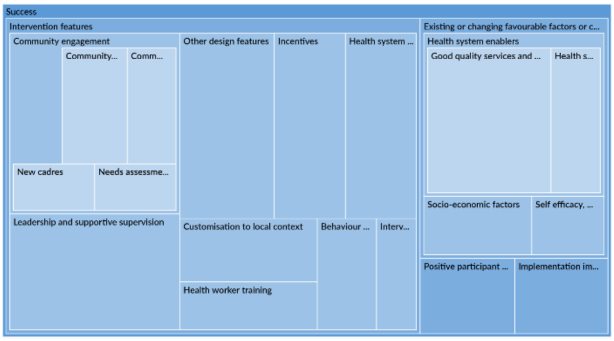

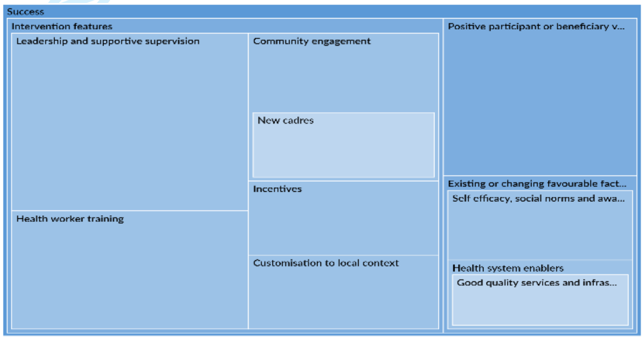

Supplementary Figure 104 (a): Full analysis *(b): Sensitivity analysis*

Reasons for intervention failure

The most consistently reported reasons for failure were broadly similar across the full analysis and the sensitivity analysis. The most consistent reasons for failure were attributed to *accounting for contextual constraints* and inadequate *intervention features*. Social norms, health system related issues, political or civil unrest and high baseline coverage were some of the most common contextual reasons for failure. Among the intervention features, inadequate duration, frequency or exposure to the intervention were the most notable reasons for failure. Though not as prominently reported as compared to the full analysis, *implementation challenges* were also noted to have caused intervention failure in the sensitivity analysis. Among these,

disruption due to inadequate implementation instructions and competing priorities of health workers were noted as some of the prevalent challenges.

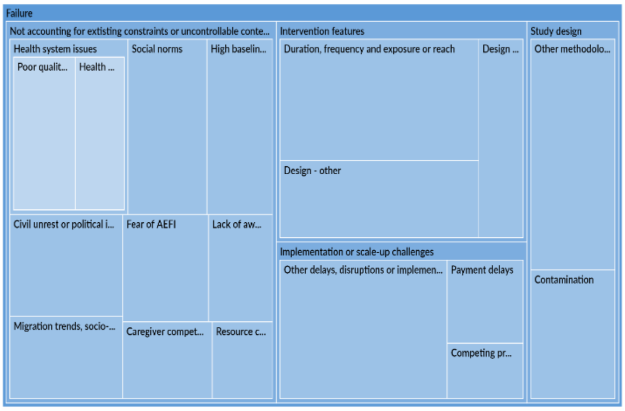

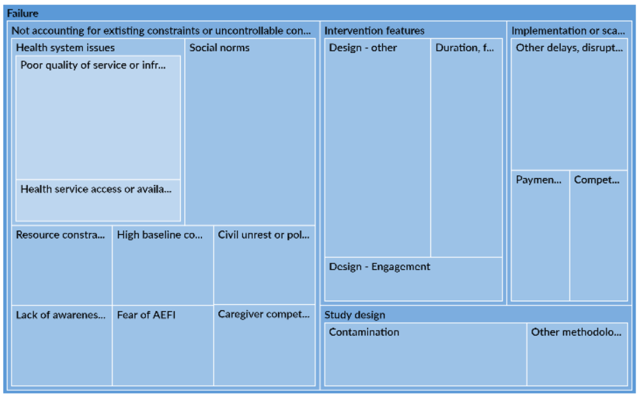

Supplementary Figure 105 (a): Full analysis *(b): Sensitivity analysis*

Uptake and fidelity challenges

The most consistently reported uptake and fidelity issues were broadly similar across the full analysis and the sensitivity analysis. The most common challenges consistently reported challenges were related to administration, mobilization, fidelity, contamination and budget constraints. Administrative challenges were cited consistently. Similar to the full analysis, the primary most consistent issue was that realities on the ground forced changes to the intervention.

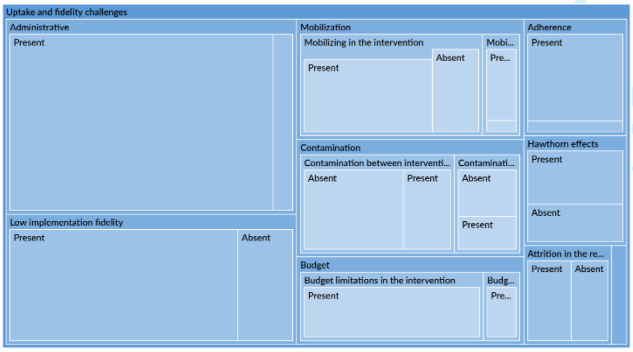

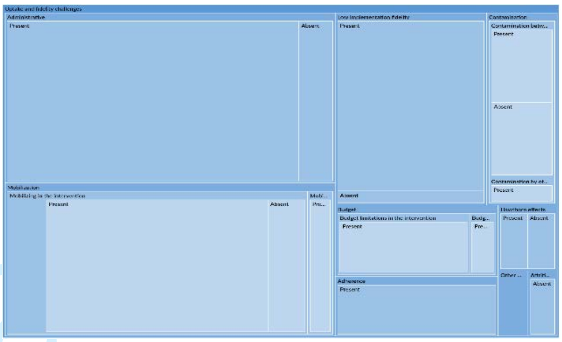

Supplementary Figure 106 (a): Full analysis *(b): Sensitivity analysis*

For peer review only - http://bmjopen.bmj.com/site/about/guidelines.xhtml

Appendix 15: Synthesis of cost evidence

This section reports on the results of the analysis of the inventory of included studies (see Table 21) and of the estimates of the non-vaccine cost per dose of interventions to increase absolute immunisation coverage by one percent (see Table 22).

Characteristics of included studies

Twenty-two of the evaluations selected for inclusion in the systematic review reported some type of cost analysis. Of these, 18 used an experimental design and the remaining 4 used quasi-experimental methods to identify the impact of treatment.

Of the 22 studies: 20 studies reported a total cost of the intervention; 12 included a cost-effectiveness analysis; three included cost-efficiency analysis; two studies included a cost-benefit analysis and two analysed cost per quality-adjusted life year (QALY).

Table 21: Characteristics of studies including any type of cost analysis.

					Type of Cost Analysis			
Author, Date	Country	Evaluation design	Intervention exposure (months)	Total Cost	Cost-efficiency Analysis	Cost-effectiveness Analysis	Cost-Benefit Analysis	Cost per QALY or DALY
Andersson, 2013	Pakistan	Experimental	8	✓				
Banerjee, 2010	India	Experimental	18	✓		✓		
Banerjee, 2020	India	Experimental	14	✓		✓		
Björkman, 2009	Uganda	Experimental	0.17	✓				
Borkum, 2020	India	Experimental	12	✓	✓	✓		
Demilew, 2020	Ethiopia	Experimental	17	✓				
Gurley, 2020	India	Experimental	11	✓		✓		
Johri, 2009	India	Experimental	3	✓				
Manoj, 2020	India	Experimental	4	✓				
Modi, 2019	India	Experimental	12	✓	✓	✓	✓	✓
More, 2017	India	Experimental	24	✓				
Morris, 2020	Honduras	Experimental	24	✓		✓		
Nagar, 2020	India	Experimental	20	✓		✓		
Olken, 2014	Indonesia	Experimental	24	✓		✓		
Oyo-Ita, 2020	Nigeria	Experimental	18	✓		✓		
Pramanik, 2018	India	Experimental	13	✓				
Seth, 2010	India	Experimental	9.7			✓		
Webster, 2019	Uganda	Experimental	12	✓			✓	
Admassie, 2017	Ethiopia	Quasi-experimental	48		✓			
Carnell, 2014	Ethiopia	Quasi-experimental	48	✓				
Findley, 2004	Nigeria	Quasi-experimental	24	✓		✓		
Saggurti, 2009	India	Quasi-experimental	2	✓		✓		✓

By country. Fourteen of the community participation interventions to improve child immunisation studies were conducted in Asian countries: of these, 12 studies took place in India; and one each in Pakistan and Indonesia. One study was conducted in a Central American country, Honduras and seven studies were conducted in African countries: three in Ethiopia; and two each in Nigeria and Uganda

For peer review only - http://bmjopen.bmj.com/site/about/guidelines.xhtml

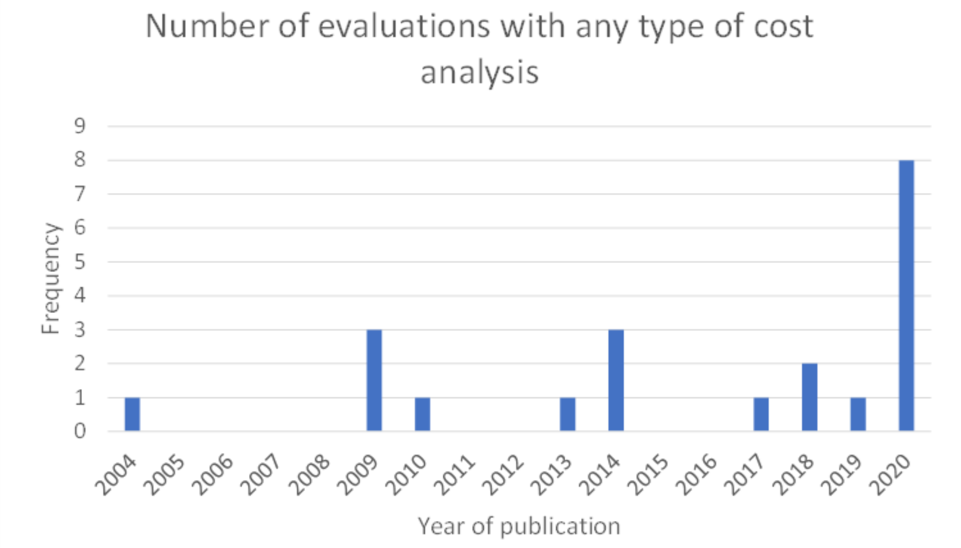

Supplemenary Figure 107: Number of evaluations with any type of cost analysis

The production of cost evidence in published evaluations of community participation interventions to improve child immunisation in low- and middle-income countries was uneven over the years. The first recorded evaluation with any cost analysis was published in 2004. Between 2009 and 2019, no more than three of the identified evaluations included any type of cost analysis in any given year. In 2020, the largest number of evaluations, eight, were published in a single year. The eight evaluations published in 2020 were required to generate cost analysis in partial fulfillment of the grant requirements in 3ie's Innovations in Increasing Immunisation Evidence Programme. Funding for these studies was provided by the Bill and Melinda Gates Foundation.

Non-vaccine cost per dose of intervention to increase immunisation coverage by one percent

Of the 22 included studies reporting cost and impact information, we estimate the non-vaccine cost per dose of intervention to increase immunisation coverage by one percent for 14. Because several studies report results by treatment arm, we report results for a total of 17 treatment arms. These results are summarised in detail in Table 22.

Three studies and one treatment arm were dropped from the analysis. In the case of Johri, 2020 this was because the proportion of children vaccinated was not reported as an outcome of this evaluation. Morris 2020 was dropped because it is unclear how to combine the PRAF II costs reported in a separate paper with intervention as described in Moris, 2020, in addition, the evaluation found a null result on the immunisation outcomes. Findley, 2013 was dropped because the reported cost information is insufficient for generating a total cost estimate or cost per vaccine dose, and "immunisation status" is not clearly defined. In the case of Banerjee, 2020, no coverage change information was reported for the Gossip seeds treatment arm which was dropped from the analysis as a result.

Six studies, Findley, 2004; Pramanik, 2018; Webster, 2019; Oyo-Ita, 2020, Modi, 2019; and Demilew, 2020 reported negative or null results and were excluded from the analysis of non-vaccine cost per dose of intervention to increase immunisation coverage by one percent.

The median non-vaccine cost per dose of intervention to increase immunisation coverage by one percent for the 14 estimates was US $3.68 (all costs are reported in 2019 US dollars). In comparison, the average was US $ 46.60 which is driven by three observations, Olken 2014 (US $23.10); Carnell, 2014 (US $641.08); and Gurley, 2020 (US $29.98).

For peer review only - http://bmjopen.bmj.com/site/about/guidelines.xhtml

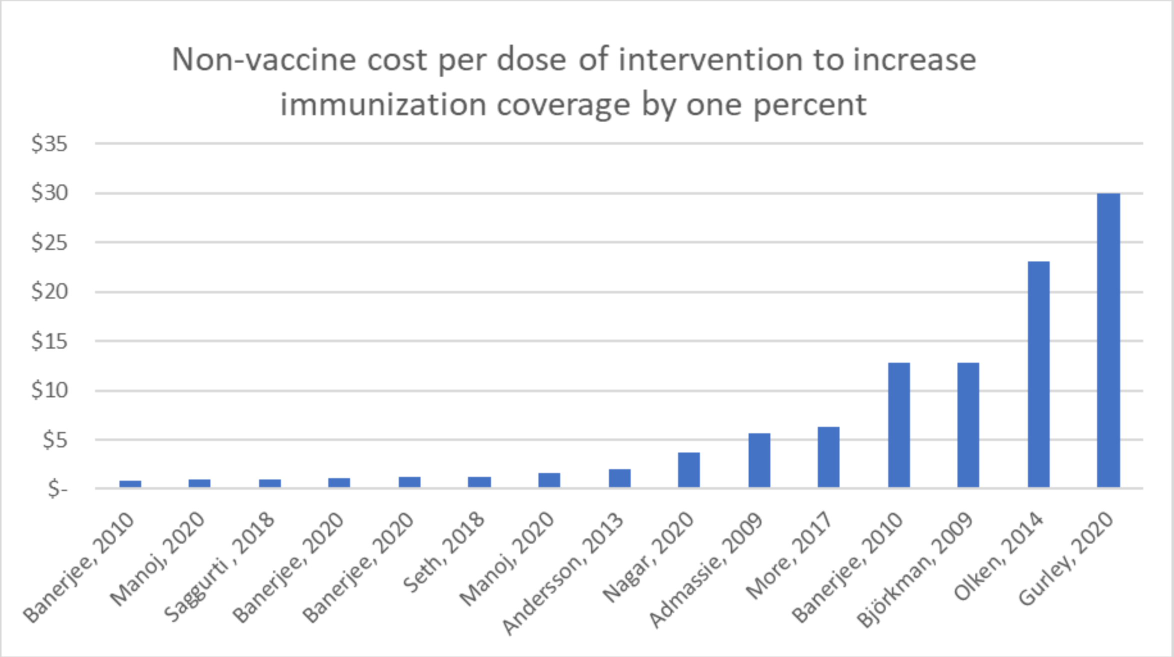

Supplementary Figure 108: Non-vaccine cost per dose of intervention to increase immunisation coverage by one percent

Supplementray Figure 108 illustrates that the range of estimates varied from a minimum of US $0.88 to a maximum of US $29.98. The lowest cost per vaccine dose was observed in Banerjee, 2010 for the "camps with incentives" intervention at US $0.88 per vaccine dose to increase immunisation coverage by one percent. The highest cost was observed in Carnell, 2014 at $641.08 - where the five-year intervention included system-building activities such as building and staffing a health post. In addition, the transparency of cost reporting in Carnell is very low, which further contributes to uncertainty in this cost estimate Indeed, Munk et. Al (2019) dropped this study from their systematic review of costs because they judged the available cost information to be insufficient to calculate the cost per vaccine dose. Since Carnell is an outlier observation, we take the intervention with the second-highest cost per incremental outcome which was reported in Gurley, 2020 at US $29.98.

For peer review only - http://bmjopen.bmj.com/site/about/guidelines.xhtml

Supplementary Table 22: Detailed estimates of non-vaccine intervention cost per dose and absolute coverage change

Author, Date	Country	Total Cost USD $2019	Treatment	Outcome	Vaccines Reported (list)	Number of doses	Non-vaccine intervention cost per vaccine dose ($ 2019 USD)	Absolute coverage change (Intervention - Control)	Non-vaccine cost per dose of intervention to increase immunization coverage by one percent	Impact notes	Cost notes	Immunization notes. Basel
Oyo-Ita, 2020	Nigeria	$15,158	Training of traditional and religious leaders; health workers; and community mobilization to improve vaccination	Proportion of children with up-to-date vaccination	Pentavalent3, Measles	5 per protocol	$4.59	Null effect	negative / null result	Unadjusted proportion, endline (intervention - control)	Incremental to no intervention control group, excludes healthcare system-building costs	46% is baseline up to date vaccination in intervention, 48% control
Demilew, 2020	Ethiopia	$224,269	A system of tracking children's immunisation status using a poster and stamp system	Probability of receiving all 3 doses of DPT/PCV	DPT - Diphtheria, pertussis, and tetanus / PCV - Pneumococcal Conjugate Vaccine	6 per protocol	$23	Null effect	negative / null result	Unadjusted proportion, endline (intervention - control)	Incremental to no intervention control group, excludes healthcare system-building costs	Baseline immunization not reported
Seth, 2018	India	$2,178	Reminders + Incentives	Proportion of total number of immunizations received by a child divided by the total number of immunizations required	BCG, DPT, H influenzae type B, Polio, Hepatitis B, Rotavirus, Measles	3.21 per child, 574 doses	$4	3.1	$1.252	Unadjusted proportion, (end of study - baseline)	Incremental to no intervention control group, excludes healthcare system-building costs	40% is baseline immunization coverage in treatment and 39% in control
Banerjee, 2010	India	$24,599.27	Camps without incentives	Proportion of children fully immunised	Full immunisation is one dose of BCG vaccine, three doses of DPT (diphtheria-pertussis, tetanus) vaccine, three doses of oral polio vaccine, and one dose of measles vaccine.	2.35 per child	$153.44	12.0	$12.79	Unadjusted proportion, endline (intervention - control)	Incremental to no intervention control group, excludes healthcare system-building costs	2% is baseline full immunsation rate in the study area
Banerjee, 2010		$12,408.09	Camps with incentives	Proportion of children fully immunised		2.85 per child	$29.22	33.0	$0.89	Unadjusted proportion, endline (intervention - control)	Incremental to no intervention control group, excludes healthcare system-building costs	2% is baseline full immunsation rate in the study area
Johri, 2009	India	-	-		-	-	-	-	-	-	-	-
Andersson, 2013	Pakistan	$70,981.46	Three structured community discussions of of local vaccination rates; costs and benefits of childhood vaccination; and local action plans.	Uptake of full DPT-3 and Measles vaccination in children 12-23 months	DPT-3, Measles	5 per protocol	$50.16	24	$2.07	Unadjusted proportion, endline (intervention - control)	Incremental to no intervention control group, excludes healthcare system-building costs	Baseline for DPT3 is 0.51 in treatment and 0.45 in control
Findley, 2004	Nigeria	-	-		-	-	-	-	negative / null result	-	-	-
Morris, 2020	Honduras	-	-		-	-	-	-	-	-	-	
Pramanik, 2018	India	$101,636.61	Community-based learning cycles where a community identifies a problem, makes an action plan, takes action and learns from the process	Full immunization take-up	1 BCG dose; 3 doses OPV; 3 doses DPT or Pentavalent vaccine, and one measles vaccine	8 per child 12-23 months	$17.20	RC = -1%	negative / null result	Unadjusted proportion, endline (intervention - control)	Incremental to no intervention control group, excludes healthcare system-building costs	75% is baseline full immunization coverage in treatment and 76% in control

For peer review only - http://bmjopen.bmj.com/site/about/guidelines.xhtml

For peer review only

233

Author, Date	Country	Total Cost USD $2019	Treatment	Outcome	Vaccines Reported (list)	Number of doses	Non-vaccine intervention cost per vaccine dose ($ 2019 USD)	Absolute coverage change (Intervention - Control)	Non-vaccine cost per dose of intervention to increase immunization coverage by one percent	Impact notes	Cost notes	Immunization notes
Saggurti , 2018	India	$39,597.45	Participatory behavior communication on maternal, neonatal, child health and promoting collectivization processes through women's self-help groups (SHGs)	Age-appropriate immunization among children under one year	9 doses - 1 BCG, 1 OPV, 3 OPV, 1,2,3,; 3 DPT, 1 Measles	9 per child under 1 year	$9.37	9	$1.03	Quasi-experimental. DID estimate. Intervention and control proportions were adjusted for baseline covariates	Incremental to no intervention control group, excludes healthcare system-building costs	Baseline full immunization was 42.9% in treatment and 52.5% in control
Admassie, 2009	Ethiopia	$1,008,991.46	Received Measles vaccine for children 9 to 11 months	Proportion of children 12–60 months old vaccinated against major diseases. tuberculosis, diphtheria, whooping cough (pertussis), tetanus, polio and measles	8 doses: tuberculosis, polio, diphtheria–pertussis–tetanus, and measles	8 per child 12 - 60 months	$56.19	9.9	$5.68	Quasi-experimental. Intervention and control proportions were adjusted for baseline covariates	Incremental cost of running Health Services Extension Programme (HSEP) in the treatment areas, includes system-building cost to construct and staff a health post	Baseline immunization not reported
More, 2017	India	$213,420.20	Community resource centres	Proportion of children aged 12–23 months fully immunised	BCG, diphtheria, pertussis, and tetanus [three doses], polio, hepatitis B virus [three doses], and measles	8 per protocol	$37.02	5.8	$6.34	Unadjusted proportion endline, (intervention - control). Change was positive but not statistically significantly different from zero	Incremental to no intervention control group,excludes healthcare system-building costs	65% is baseline full immunization coverage in treatment and 63% in control
Olken, 2014	Indonesia	$12,264,782.40	Village-level block grants for maternal and child health and education that incorporated relative performance incentives	Proportion of children under two years of age with BCG vaccine	1 dose, BCG	1.00	$36.97	1.60	$23.11	Adjusted average standardized effect (absolute coverage change) was positive but not statistically significantly different from zero.	Incremental cost of preference-weighted incentive block grant program for vaccines relative to the cost of the program of community block grants without incentives	65.3% is baseline full immunization coverage
Borkum, 2020	India	$39,571.93	A program that offers non-monetary incentives for teamwork and goal-setting activities to improve maternal and child health at health subcenters in India	Proportion of children ages 6-11 months who recieved DPT-3 by the age of 6 months	DPT-3	585	$22.54	5.4	$4.17	Adjusted proportion (intervention - control). Difference is positive but not signficantly different from zero	Incremental to no intervention control group, excludes healthcare system-building costs	45.5% is baseline mean for a child fully immunized except measles in the treatment group, and 39.9% in the control group
Manoj, 2020	India	$61,344.88	Information treatment	Full immunization of children 12-23 months	8 doses: BCG, measles, and 3 doses each for Polio and DPT	8 per protocol	$14.10	8.8	$1.60	Unadjusted increase in proportion immunised in intervention relative to control. We report separately by treatment arm because there is corresponding cost info.	Incremental to no intervention control group, excludes healthcare system-building costs	Baseline immunization not reported
Manoj, 2020	India	$61,344.88	Information + Facilitation treatment	Full immunization of children 12-23 months	8 doses: BCG, measles, and 3 doses each for Polio and DPT	8 per protocol	$12.63	13.4	$0.94	Unadjusted increase in proportion immunised in intervention relative to control. We report separately by treatment arm because there is corresponding cost info.	Incremental to no intervention control group, excludes healthcare system-building costs	Baseline immunization not reported
Björkman, 2009	Uganda	$182,003.09	Community-based monitoring of public primary health care providers	Proportion of children ages 13-24 months who received required doses of DPT	3 doses: DPT	3	$76.83	6.0	$12.81	Adjusted percent increase in 1-year old immunisation rate	Back of the envelope total cost, incremental intervention cost relaitve to control	Baseline immunization not reported
Banerjee, 2020	India	$342,595.94	High slope incentives			12 per pro	$13.81	11	$1.21	Adjusted percent change in fully immunised	Incremental to no intervention control group, excludes healthcare system-building costs	20.8% - 50.05% is the range of baseline means, depending on the district for a child fully vaccinated
		$367,078.25	Low slope incentives			12 per pro	$13.85	12	$1.14	Adjusted percent change in fully immunised	Incremental to no intervention control group, excludes healthcare system-building costs	20.8% - 50.05% is the range of baseline means, depending on the district for a child fully vaccinated
		$78,072.73	Gossip seeds	Proportion of children under 12 months who received 1 dose of BCG, 4 doses of the oral polio vaccine, 3 doses of penta, 3 doses of rotavirus and 1 dose of measles	BCG, penta doses 1–3 and measles-1	12 per pro	NA	-	-	No coverage change reported for the Gossip seeds treatment.	Incremental to no intervention control group, excludes healthcare system-building costs	20.8% - 50.05% is the range of baseline means, depending on the district for a child fully vaccinated
Nagar, 2020	India	$275,702.59	Tracking system: The Khushi Baby platform, a wearable digital health record (pendant) and with patient-specific, dialect-specific voice reminders	Full and timely Immunization as verified by MAMTA card for children 12-23 months	8 doses: Polio 1-3, BCG, Pentavalent 1-3, Measles	6 per protocol	$41.17	11.2	$3.68	Unadjusted increase in proportion immunised in intervention relative to control	Incremental to no intervention control group, excludes healthcare system-building costs	25.4% is intervention baseline, full immunisation without birth doses and 22.5% is control
Gurley, 2020	India	$175,603.81	PATH - community-led videos	Proportion of children 6 to 17 months of age who had received all age-appropriate vaccines in India's vaccine schedule	13 doses: Birth doses (OPV, BCG, HepB); DPT1-3; HepBI-3; MR; OPV1-3	13 per protcol	$26.39	0.88	$29.98	Unadjusted proportion, endline (intervention - control). Difference is positive but not signficantly different from zero	Incremental to no intervention control group, excludes healthcare system-building costs	51.94% in treatment and 53.85 in control for full immunization
Carnell , 2014	Ethiopia	$27,316,577.57	Three pillars approach Eshe project strengthening health systems, improving health workers' performance, and engaging the community	Percentage of children aged 12-23 months, who received DPT3 vaccination, or Measles	2 doses: DPT3, Measles	2 per protocol	$5,449.19	8.5	$641.08	Coverage change is the average for DPT3 and Measles. Cost data very low transparency, making cost estimate unreliable	System-building' activities are evident in this intervention. However, cost data have very low transparency. To calculate total cost for treating vaccinated	49.3% in treatment and 33.87% in control for DPT3
Modi, 2019	India	$160,881.37	A mobile-phone app to assist ASHAs and health providers to schedule and track home health visits and follow-on care	Proportion of infants (6-8 months) who received all three doses of DPT or Pentavalent vaccine	3 doses: DPT or Pentavalent vaccine	3 per protocol		null result	negative / null result	Unadjusted proportion, endline (intervention - control)	Incremental to no intervention control group, excludes healthcare system-building costs	71.1% in treatment and 75.5% in control for DPT3
Webster, 2019	Uganda	$224,224.18	mReach data platform and the 'Fifth Child', a community engagement strategy to increase immunization	Proportion of children ages 12-23 months with DPT3 and MCV combined immunization coverage	2 doses: DPT3, Measles	2 per protocol	$204.63	null result	negative / null result	Unadjusted proportion, endline (intervention - control)	Incremental to no intervention control group, excludes healthcare system-building costs	76.2% in treatment and 80.3 % in control for DPT3

For peer review only - http://bmjopen.bmj.com/site/about/guidelines.xhtml

view only

For peer review only

235

For peer review only

For peer review only - http://bmjopen.bmj.com/site/about/guidelines.xhtml

Björkman, 2009	Uganda	$182,003.09	Community-based monitoring of public primary health care providers	Proportion of children ages 13-24 months who received required doses of DPT	3 doses: DPT	3	$76.83	6.0	$12.81	Adjusted percent increase in 1-year old immunisation rate	Back of the envelope total cost, incremental intervention cost relaitve to control	Baseline immunization not reported
Banerjee, 2020	India	$342,595.94	High slope incentives			12 per pro	$13.81	11	$1.21	Adjusted percent change in fully immunised	Incremental to no intervention control group, excludes healthcare system-building costs	20.8% - 50.05% is the range of baseline means, depending on the district for a child fully vaccinated
		$367,078.25	Low slope incentives			12 per pro	$13.85	12	$1.14	Adjusted percent change in fully immunised	Incremental to no intervention control group, excludes healthcare system-building costs	20.8% - 50.05% is the range of baseline means, depending on the district for a child fully vaccinated
		$78,072.73	Gossip seeds	Proportion of children under 12 months who received 1 dose of BCG, 4 doses of the oral polio vaccine, 3 doses of penta, 3 doses of rotavirus and 1 dose of measles	BCG, penta doses 1–3 and measles-1	12 per pro	NA	-	-	No coverage change reported for the Gossip seeds treatment.	Incremental to no intervention control group, excludes healthcare system-building costs	20.8% - 50.05% is the range of baseline means, depending on the district for a child fully vaccinated
Nagar, 2020	India	$275,702.59	Tracking system: The Khushi Baby platform, a wearable digital health record (pendant) and with patient-specific, dialect-specific voice reminders	Full and timely Immunization as verified by MAMTA card for children 12-23 months	8 doses: Polio 1-3, BCG, Pentavalent 1-3, Measles	6 per protocol	$41.17	11.2	$3.68	Unadjusted increase in proportion immunised in intervention relative to control	Incremental to no intervention control group, excludes healthcare system-building costs	25.4% is intervention baseline, full immunisation without birth doses and 22.5% is control
Gurley, 2020	India	$175,603.81	PATH - community-led videos	Proportion of children 6 to 17 months of age who had received all age-appropriate vaccines in India's vaccine schedule	13 doses: Brith doses (OPV, BCG, HepB); DPT1-3; HepB1-3; MR; OPV1-3	13 per protcol	$26.39	0.88	$29.98	Unadjusted proportion, endline (intervention - control). Difference is positive but not signficantly different from zero	Incremental to no intervention control group, excludes healthcare system-building costs	52.88% is baseline percentage of children in the State of Uttar Pradesh who received all necessary vaccines in 2012–2013
Carnell , 2014	Ethiopia	$27,316,577.57	Three pillars approach Eshe project: strengthening health systems, improving health workers' performance, and engaging the community	Percentage of children aged 12-23 months, who received DPT3 vaccination, or Measles	2 doses: DPT3, Measles	2 per protocol	$5,449.19	8.5	$641.08	Coverage change is the average for DPT3 and Measles. Cost data very low transparency, making cost estimate unreliable	System-building' activities are evident in this intervention. However, cost data hvae very low transparency. To calculate total cost for treating vaccinated	Baseline immunization not reported
Modi, 2019	India	$160,881.37	A mobile-phone app to assist ASHAs and health providers to schedule and track home health visits and follow-on care	Proportion of infants (6-8 months) who received all three doses of DPT or Pentavalent vaccine	3 doses: DPT or Pentavalent vaccine	3 per protocol		null result	negative / null result	Unadjusted proportion, endline (intervention - control)	Incremental to no intervention control group, excludes healthcare system-building costs	Baseline immunization not reported
Webster, 2019	Uganda	$224,224.18	mReach data platform and the 'Fifth Child', a community engagement strategy to increase immunisation	Proportion of children ages 12-23 months with DPT3 and MCV combined immunization coverage	2 doses: DPT3, Measles	2 per protocol	$204.63	null result	negative / null result	Unadjusted proportion, endline (intervention - control)	Incremental to no intervention control group, excludes healthcare system-building costs	Baseline immunization not reported

For peer review only - http://bmjopen.bmj.com/site/about/guidelines.xhtml

PRISMA 2020 Checklist

Section and Topic	Item #	Checklist item	Location where item is reported #page no
**TITLE**			
Title	1	Identify the report as a systematic review.	p.1
**ABSTRACT**			
Abstract	2	See the PRISMA 2020 for Abstracts checklist.	pp. 2-3
**INTRODUCTION**			
Rationale	3	Describe the rationale for the review in the context of existing knowledge.	pp. 4-5
Objectives	4	Provide an explicit statement of the objective(s) or question(s) the review addresses.	p. 4
**METHODS**			
Eligibility criteria	5	Specify the inclusion and exclusion criteria for the review and how studies were grouped for the syntheses.	pp. 5-6, appendix 1
Information sources	6	Specify all databases, registers, websites, organisations, reference lists and other sources searched or consulted to identify studies. Specify the date when each source was last searched or consulted.	pp. 5-6, appendix 3
Search strategy	7	Present the full search strategies for all databases, registers and websites, including any filters and limits used.	p. 5, appendix 3, 4
Selection process	8	Specify the methods used to decide whether a study met the inclusion criteria of the review, including how many reviewers screened each record and each report retrieved, whether they worked independently, and if applicable, details of automation tools used in the process.	p. 5
Data collection process	9	Specify the methods used to collect data from reports, including how many reviewers collected data from each report, whether they worked independently, any processes for obtaining or confirming data from study investigators, and if applicable, details of automation tools used in the process.	p. 6
Data items	10a	List and define all outcomes for which data were sought. Specify whether all results that were compatible with each outcome domain in each study were sought (e.g. for all measures, time points, analyses), and if not, the methods used to decide which results to collect.	p. 6, appendix 6
	10b	List and define all other variables for which data were sought (e.g. participant and intervention characteristics, funding sources). Describe any assumptions made about any missing or unclear information.	p. 6, appendix 6
Study risk of bias assessment	11	Specify the methods used to assess risk of bias in the included studies, including details of the tool(s) used, how many reviewers assessed each study and whether they worked independently, and if applicable, details of automation tools used in the process.	p. 7, appendix 7
Effect measures	12	Specify for each outcome the effect measure(s) (e.g. risk ratio, mean difference) used in the synthesis or presentation of results.	p. 7
Synthesis methods	13a	Describe the processes used to decide which studies were eligible for each synthesis (e.g. tabulating the study intervention characteristics and comparing against the planned groups for each synthesis (item #5)).	p. 7
	13b	Describe any methods required to prepare the data for presentation or synthesis, such as handling of missing summary statistics, or data conversions.	p. 7
	13c	Describe any methods used to tabulate or visually display results of individual studies and syntheses.	p. 7
	13d	Describe any methods used to synthesize results and provide a rationale for the choice(s). If meta-analysis was performed, describe the model(s), method(s) to identify the presence and extent of statistical heterogeneity, and software package(s) used.	p. 7
	13e	Describe any methods used to explore possible causes of heterogeneity among study results (e.g. subgroup analysis, meta-regression).	p. 7
	13f	Describe any sensitivity analyses conducted to assess robustness of the synthesized results.	p. 7

For peer review only

For peer review only - http://bmjopen.bmj.com/site/about/guidelines.xhtml

PRISMA 2020 Checklist

Section and Topic	Item #	Checklist item	Location where item is reported #page no
Reporting bias assessment	14	Describe any methods used to assess risk of bias due to missing results in a synthesis (arising from reporting biases).	p. 7
Certainty assessment	15	Describe any methods used to assess certainty (or confidence) in the body of evidence for an outcome.	NA
**RESULTS**			
Study selection	16a	Describe the results of the search and selection process, from the number of records identified in the search to the number of studies included in the review, ideally using a flow diagram.	p. 8, figure 1
	16b	Cite studies that might appear to meet the inclusion criteria, but which were excluded, and explain why they were excluded.	pp. 6, 8, appendix 4
Study characteristics	17	Cite each included study and present its characteristics.	p. 8, table 1
Risk of bias in studies	18	Present assessments of risk of bias for each included study.	p. 21, appendix 8
Results of individual studies	19	For all outcomes, present, for each study: (a) summary statistics for each group (where appropriate) and (b) an effect estimate and its precision (e.g. confidence/credible interval), ideally using structured tables or plots.	p. 8
Results of syntheses	20a	For each synthesis, briefly summarise the characteristics and risk of bias among contributing studies.	pp. 9, 21
	20b	Present results of all statistical syntheses conducted. If meta-analysis was done, present for each the summary estimate and its precision (e.g. confidence/credible interval) and measures of statistical heterogeneity. If comparing groups, describe the direction of the effect.	pp. 21-26, appendix 9-13
	20c	Present results of all investigations of possible causes of heterogeneity among study results.	pp. 21-26, appendix 9-13
	20d	Present results of all sensitivity analyses conducted to assess the robustness of the synthesized results.	pp. 21-26, appendix 9-13
Reporting biases	21	Present assessments of risk of bias due to missing results (arising from reporting biases) for each synthesis assessed.	pp. 21-26, appendix 9-13
Certainty of evidence	22	Present assessments of certainty (or confidence) in the body of evidence for each outcome assessed.	NA
**DISCUSSION**			
Discussion	23a	Provide a general interpretation of the results in the context of other evidence.	p. 26
	23b	Discuss any limitations of the evidence included in the review.	pp. 3, 26
	23c	Discuss any limitations of the review processes used.	p. 3
	23d	Discuss implications of the results for practice, policy, and future research.	p. 28
**OTHER INFORMATION**			

For peer review only

For peer review only - http://bmjopen.bmj.com/site/about/guidelines.xhtml

PRISMA 2020 Checklist

Section and Topic	Item #	Checklist item	Location where item is reported #page no
Registration and protocol	24a	Provide registration information for the review, including register name and registration number, or state that the review was not registered.	p. 3
	24b	Indicate where the review protocol can be accessed, or state that a protocol was not prepared.	p. 3
	24c	Describe and explain any amendments to information provided at registration or in the protocol.	Appendix 2
Support	25	Describe sources of financial or non-financial support for the review, and the role of the funders or sponsors in the review.	p. 29
Competing interests	26	Declare any competing interests of review authors.	p. 29
7Availability of data, code and other materials	27	Report which of the following are publicly available and where they can be found: template data collection forms; data extracted from included studies; data used for all analyses; analytic code; any other materials used in the review.	p. 29

From: Page MJ, McKenzie JE, Bossuyt PM, Boutron I, Hoffmann TC, Mulrow CD, et al. The PRISMA 2020 statement: an updated guideline for reporting systematic reviews. BMJ 2021;372:n71. doi: 10.1136/bmj.n71

For more information, visit: http://www.prisma-statement.org/

For peer review only

BMJ Open

Use of community engagement interventions to improve child immunisation in low- and middle-income countries: a systematic review and meta-analysis

Journal:	*BMJ Open*
Manuscript ID	bmjopen-2022-061568.R2
Article Type:	Original research
Date Submitted by the Author:	08-Sep-2022
Complete List of Authors:	Jain, Monica; International Initiative for Impact Evaluation Dehli, Evaluation Office<br>Shisler, Shannon; International Initiative for Impact Evaluation<br>Lane, Charlotte ; International Initiative for Impact Evaluation<br>Bagai, Avantika; International Initiative for Impact Evaluation, Evaluation Office<br>Brown, Elizabeth; University of California Berkeley, Center for Effective Global Action<br>Engelbert, Mark; International Initiative for Impact Evaluation, Synthesis and Reviews
**Primary Subject Heading**:	Public health
Secondary Subject Heading:	Global health
Keywords:	Public health < INFECTIOUS DISEASES, Community child health < PAEDIATRICS, PUBLIC HEALTH

SCHOLARONE™
Manuscripts

I, the Submitting Author has the right to grant and does grant on behalf of all authors of the Work (as defined in the below author licence), an exclusive licence and/or a non-exclusive licence for contributions from authors who are: i) UK Crown employees; ii) where BMJ has agreed a CC-BY licence shall apply, and/or iii) in accordance with the terms applicable for US Federal Government officers or employees acting as part of their official duties; on a worldwide, perpetual, irrevocable, royalty-free basis to BMJ Publishing Group Ltd ("BMJ") its licensees and where the relevant Journal is co-owned by BMJ to the co-owners of the Journal, to publish the Work in this journal and any other BMJ products and to exploit all rights, as set out in our licence.

The Submitting Author accepts and understands that any supply made under these terms is made by BMJ to the Submitting Author unless you are acting as an employee on behalf of your employer or a postgraduate student of an affiliated institution which is paying any applicable article publishing charge ("APC") for Open Access articles. Where the Submitting Author wishes to make the Work available on an Open Access basis (and intends to pay the relevant APC), the terms of reuse of such Open Access shall be governed by a Creative Commons licence – details of these licences and which Creative Commons licence will apply to this Work are set out in our licence referred to above.

Other than as permitted in any relevant BMJ Author's Self Archiving Policies, I confirm this Work has not been accepted for publication elsewhere, is not being considered for publication elsewhere and does not duplicate material already published. I confirm all authors consent to publication of this Work and authorise the granting of this licence.

For peer review only

Use of community engagement interventions to improve child immunisation in low- and middle-income countries: a systematic review and meta-analysis

Monica Jain, Shannon Shisler, Charlotte Lane, Avantika Bagai, Elizabeth Brown, Mark Engelbert

International Initiative for Impact Evaluation (Monica Jain PhD, Shannon Shisler PhD, Charlotte Lane PhD, Avantika Bagai M.Sc and Mark Engelbert PhD) and **Center for Effective Global Action, University of Berkeley, California, USA** (Elizabeth Brown PhD)

Correspondence to:

Dr. Monica Jain

Evaluation Office, International Initiative for Impact Evaluation (3ie), New Delhi, 110017, India

Email: mjain@3ieimpact.org

For peer review only

Keywords:

Immunization

Immunization Programs

Mass Vaccination

Poverty Areas

Public Health Administration

Word count:

Body: 4,212

Abstract: 299

For peer review only - http://bmjopen.bmj.com/site/about/guidelines.xhtml

Abstract

Objective

To support evidence informed decision making, we systematically examine the effectiveness and cost-effectiveness of community engagement interventions on routine childhood immunisation outcomes in LMICs and identify contextual, design, and implementation features associated with effectiveness.

Design

Mixed-methods systematic review and meta-analysis.

Data sources

21 databases of academic and grey literature and 12 additional websites were searched in May 2019 and May 2020.

Eligibility Criteria for selecting studies

We included experimental and quasi-experimental impact evaluations of community engagement interventions considering outcomes related to routine child immunisation in LMICs. No language, publication type, or date restrictions were imposed.

Data extraction and synthesis

Two independent researchers extracted summary data from published reports and appraised quantitative risk of bias using adapted Cochrane tools. Random effects meta-analysis was used to examine effects on the primary outcome, full immunisation coverage.

Results

Our search identified over 43000 studies and 61 were eligible for analysis. The average pooled effect of community engagement interventions on full immunisation coverage was SMD 0.14 (95% CI: 0.06 to 0.23, $I^2 = 94.46$). The most common source of risk to the quality of evidence (risk of bias) was outcome reporting bias: most studies used caregiver-reported measures of vaccinations received by a child in the absence or incompleteness of immunisation cards. Reasons consistently cited for intervention success include appropriate intervention design, including building in community engagement features; addressing common contextual barriers of immunisation and leveraging facilitators; and accounting for existing implementation constraints.

For peer review only - http://bmjopen.bmj.com/site/about/guidelines.xhtml

The median intervention cost per treated child per vaccine dose (excluding the cost of vaccines) to increase absolute immunisation coverage by one percent was USD 3.68.

Conclusion

Community engagement interventions are successful in improving outcomes related to routine child immunisation. The findings are robust to exclusion of studies assessed as high risk of bias.

Protocol registration

DOI: 10.1002/cl2.1119.

Strengths and limitations of this study

- Thorough literature search of twenty-one major electronic databases and reporting as per Preferred Reporting Items for Systematic Reviews and Meta-Analysis guidelines.
- Presents a nuanced framework of community engagement with a typology that differentiates three types of interventions: interventions in which community engagement is embedded, and those interventions that engage community in their design or implementation.
- The effects of community engagement interventions are robust to exclusion of studies assessed as high risk of bias for almost all the primary outcomes. The effects are also uniform across geographies and baseline immunisation rates.
- For some immunisation outcomes the evidence base for drawing conclusions is adequate, for others it is limited.
- Evidence base is skewed across the three engagement types with a relatively large evidence base for those interventions in which community engagement is embedded and limited for those interventions using engagement in implementation autonomy

For peer review only - http://bmjopen.bmj.com/site/about/guidelines.xhtml

Introduction

Immunisation is one of the most cost-effective ways to prevent and control life-threatening infectious diseases. From 2001 to 2020, projects that introduced or increased coverage of vaccines averted an estimated 14 million deaths, 350 million cases of illness, 8 million cases of long-term disability and 700 million disability-adjusted life-years.[1] Nonetheless, rates of routine vaccination of children in low- and middle-income countries (LMICs) are low or stagnant. In 2019, an estimated 19.7 million infants did not receive routine immunisations. Around 60% of these children live in ten LMICs, including Ethiopia, India, Nigeria, and Pakistan as of 2019.[2]

Community engagement approaches feature prominently in global immunisation strategies.[3] However, there is a dearth of rigorous and systematic evidence on effectiveness of community engagement approaches to improve routine childhood immunisations specifically in LMICs. In our search we could find only two systematic reviews for LMICs which analysed effectiveness of community monitoring interventions and preventive interventions delivered by community health workers, respectively.[4,5] As such, previous systematic reviews do not provide adequate guidance to stakeholders interested in understanding whether and how alternative community engagement interventions work in LMICs to improve routine childhood immunisations and at what cost. There is, therefore, a need to make such evidence available to guide policymakers and public health practitioners in making informed decisions about these interventions. To address this knowledge gap, we conducted a systematic review examining the effects of community engagement interventions on outcomes related to childhood immunisation in LMICs, determining their cost-effectiveness, and identifying contextual, design and implementation features that may be associated with intervention effectiveness.

Methods

Overview

The protocol of this systematic review with meta-analysis is registered with The Campbell Collaboration.[6] We followed the Campbell and Cochrane Collaborations' guidelines for systematic reviewing[7-10] and drew on theory-based mixed methods impact evaluation[11] and

For peer review only - http://bmjopen.bmj.com/site/about/guidelines.xhtml

systematic review[12,13] concepts. We followed the PRISMA reporting guidelines. The amendments to the information provided in the protocol is reported in Appendix 1.

Conceptual framework

For our review we defined "communities" in reference to the lowest level of the health service delivery system (or whatever level provides routine immunisation services in the local context). A community is a group of people who serve or are served by a particular primary health facility. Thus, communities encompass a wide range of stakeholders, including caregivers, health service providers, and influential community members such as religious or other traditional leaders.

WHO 2020 defines community engagement as "*a process of developing relationships that enable stakeholders to work together to address health-related issues and promote well-being to achieve positive health impact and outcomes*."[14] For this review we developed a framework that classified community engagement interventions based on *process* of engagement as in the WHO definition. It also corresponds to the "utilitarian perspective" of community engagement captured and articulated in Brunton et al. (2017)[15]: "*In utilitarian perspectives, health (and other) services reach out to engage particular communities that they have identified require assistance and the intervention is devised within existing policy, practice, and resource frameworks*." In addition, our framework goes beyond one-way communication to include some consultation or dialogue with the community or some decision making by them. We considered three points within an intervention during which engagement could occur as elaborated below and in Appendix 2.

Engagement in the design of interventions: community input or feedback was sought before implementing an intervention (e.g., pilot, needs assessment, formative evaluation, and outreach).

Engagement in implementation autonomy of interventions: community was utilized in intervention implementation as health care workers, facilitators, or problem solvers and only if they had some opportunity to affect or influence its implementation.

Engagement as the intervention (engagement is embedded): a serious attempt was made to gain community buy-in for activities or new cadres of community-based structures were established (e.g., village health committees or community health volunteers).

For peer review only - http://bmjopen.bmj.com/site/about/guidelines.xhtml

Research Questions

The research questions for this review were:

1. What evidence exists regarding the effectiveness of community engagement interventions in improving routine immunisation coverage of children in LMICs?

2. Is there evidence for heterogeneous effects of community engagement strategies (i.e., does effectiveness vary by geographical region, gender or programme implementation)?

3. What factors relating to programme design, implementation, and context are associated with better or worse outcomes along the causal chain? Do these vary by the kind of community engagement?

4. What is the cost-effectiveness of different community engagement interventions in improving children routine immunisation outcomes?

Search strategy

We implemented a systematic and comprehensive search strategy, in consultation with an information specialist. In May 2019 and May 2020, we searched 17 academic databases for experimental and quasi-experimental impact evaluations of community engagement interventions considering outcomes related to routine child immunisation in LMICs (using the World Bank country income classifications to determine LMIC status at the time the intervention began). We also searched 17 additional websites for grey literature. The list of sources searched and an example set of search strings are provided in Appendix 3. We complemented this with citation tracking and contacting experts. The grey literature search was conducted by AB with support from external consultant reviewers. Given the limitations of the search functions on websites we searched for grey literature, it was not possible to use the same complex search strings used in academic databases, and search strategies were developed on a site-by-site basis.

For peer review only - http://bmjopen.bmj.com/site/about/guidelines.xhtml

Inclusion / exclusion criteria (PICOS)

Table 1: PICOS inclusion and exclusion criteria

**Characteristics**	**Inclusion criteria**
Population	Rural, peri-urban and urban populations living in low and middle-income countries.
Interventions	Interventions involving community participation.
Comparisons	A comparison group or counterfactual that does not receive the intervention or business as usual.
Outcomes	Full, partial, timely immunisation of children and other outcomes such as morbidity, mortality, etc.
Study design	To answer question 1, 2 and 4, experimental and quasi-experimental studies.<br>To answer question 3, qualitative studies, descriptive quantitative studies, process evaluations, project documents, formative research studies, protocols, baseline and midline and endline/final impact evaluation reports, policy briefs and website content.
Other	No inclusion restrictions by publication status or language.

The population, intervention, comparators, outcomes, and study designs (PICOS) eligible for inclusion in the study are provided in Table 1. No language, publication type or date restrictions were imposed. Because our definition of community focused on the lowest levels of health facilities, we excluded interventions targeting higher levels of the health system (e.g., state-level officials) (Appendix 4). The primary outcomes considered in this review were coverage rates for (a) full immunisation, which is typically defined as the percentage of one-year-olds who have

For peer review only - http://bmjopen.bmj.com/site/about/guidelines.xhtml

received one dose of Bacille Calmette-Guérin[i], (b) third dose of DPT or pentavalent, (c) first does of measles, or (d) the timeliness of any of these doses. Additional antigen-specific immunisation coverage outcomes and secondary outcomes reflecting upstream conditions (e.g., attitudes about vaccination and access to immunisation services) and downstream effects (e.g., morbidity and mortality) of the primary outcomes were also included. Official health records and parent recall were considered acceptable sources of measures of immunisation coverage. The former was used when both measures were reported separately.

This review includes experimental and quasi-experimental studies that estimate the causal impact of an intervention, as compared to usual practice, by establishing a counterfactual. Specifically, studies with the following evaluation designs are included: randomised controlled trials, regression discontinuity designs, instrumental variables estimation, statistical matching (e.g., propensity score matching), difference-in-differences (or any mathematical equivalent), fixed effects estimation, and interrupted time series. We excluded studies for which the reported quantitative data could not be meaningfully converted to an effect size. In cases of relevant missing or incomplete data, we contacted study authors to obtain the required information. If we were unable to obtain the necessary data, we reported the characteristics of the study but did not include these studies in the meta-analysis. We conducted additional searches for economic and qualitative evidence on the included impact evaluations (Appendix 5).

Screening

At both the title and abstract and full-text screening stages, all papers were double screened by research consultants and supervised by MJ, ME and AB. Reconciliation meetings were held to resolve disagreements and MJ and ME made final decisions on unresolved cases. The same reviewers manually searched for qualitative papers and project documents on Google Scholar and websites of implementing organizations and screened the papers for inclusion as they were identified.

Data analysis

[i] In some countries, other vaccinations such as those for JE encephalitis and yellow fever are administered to children as a part of the routine immunisation schedule. In those contexts, we went by the definition of full immunisation mentioned in the impact evaluation study.

For peer review only - http://bmjopen.bmj.com/site/about/guidelines.xhtml

Studies were coded for their engagement type by two reviewers (MJ and AB) who independently reviewed the intervention description and coded these against the definitions provided above. If studies allowed for engagement at several stages of the intervention, they could be coded as having more than one engagement type. We used Microsoft Excel to extract descriptive information and effect sizes from included studies using double coding. Coders reconciled their answers and a study author made final decisions in case of disagreements. For qualitative analysis, all impact evaluations and additional documentation identified in the search were coded in Nvivo. Cost data was single coded and checked by a study author (Appendix 6).

To avoid double-counting of evidence from different papers focusing on the same study, we linked these papers prior to analysis. We extracted data from the most recent publication. When data was reported over multiple time periods, we extracted data for each period. Where authors reported the same outcome using more than one analytical model, we extracted data from the authors' preferred model specification. When the preference was not specified, we used the model with the most controls. Where studies reported outcomes related to multiple treatment arms and only one comparison group, we estimated an effect size for each of the treatment arms.

To assess quantitative risk of bias, we created an adapted version of the Cochrane guidelines for assessing randomized controlled trials and non-randomised studies.[16,17] These assessments were conducted by two independent reviewers. Coders reconciled their answers and a study author made final decisions in case of disagreements. For testing the sensitivity of the results to low-quality studies, we ran each analysis with and without studies scoring high risk of bias.

We critically appraised qualitative and mixed-methods studies using an adapted version of the nine-item framework developed by the Critical Appraisal Skills Programme.[18] In addition, we carried out a sensitivity analysis in which we considered only the high-quality qualitative studies that had a risk of bias assessment score of 20 or higher, indicating low risk of bias. For cost evidence, we assessed risk of bias along six primary dimensions adapted from a combination of tools, including: Doocy and Tappis (2017);[19] Campbell Collaboration Economic Methods Policy Brief;[20] and Methods for the Economic Evaluation of Health Care Programmes[21] (Appendix 7).

We calculated the standardised mean difference (SMD), or Cohen's *d*, its variance and standard error for each effect, converting effects reported in other metrics as necessary, using formulae

For peer review only - http://bmjopen.bmj.com/site/about/guidelines.xhtml

provided in Borenstein and colleagues (2009).[22] In all cases we then adjusted Cohen's *d* to Hedges' *g* as defined in Ellis (2010).[23] For studies reporting regression results, we followed the approach of Keef and Roberts (2004)[24] using the regression coefficient and the pooled standard deviation of the outcome.

The amount of heterogeneity (i.e.,τ^2) was estimated using the DerSimonian-Laird estimator.[25] The Q-test for heterogeneity[26] and the I^2 statistic[27] are reported. We complement this with an assessment of heterogeneity of effect sizes graphically using forest plots. We identified outliers using studentized residuals and identified overly influential studies using Cook's distance. Where outliers were indicated, we report the resulting effect sizes when they are left out of the analysis. As an additional sensitivity test, we ran a full leave one out analysis for all models, and we report these results when and where they are useful. Whenever feasible, we conducted moderator analyses using meta-regression to investigate sources of heterogeneity.[ii] The analysis was carried out using R (version 4.0.4)[28] and the **metafor** package (version 2.4.0).[29] All analyses used a random effects model because we did not reasonably expect the included studies to be functionally identical and the goal was to generalize to the larger population.[30]

Qualitative analysis followed a mix of inductive and deductive coding approaches to identify themes related to barriers and facilitators, reasons for intervention success or failure, and uptake and fidelity challenges. An initial set of themes was developed based on familiarity with the literature. However, as new topics were identified, new themes were added. Themes were also disaggregated if it became clear they were too broad. Research consultants conducted coding with oversight from CL and AB.

Patient and public involvement in research

There was no patient or public involvement in this research.

Ethics statement

[ii] All but two moderators were chosen *a priori.* Baseline coverage and vaccine hesitancy were added after feedback from an initial peer review from the Campbell Collaboration (co-publisher of this work).

For peer review only - http://bmjopen.bmj.com/site/about/guidelines.xhtml

Ethical consent from an Ethics Committee or Institutional Board was not required as this study does not involve human subjects.

Results

Our search identified over 43,000 records, which were reduced to 29,481 unique abstracts after de-duplication (Figure 1). After title and abstract screening, we considered 1,285 studies for full-text screening and could not locate an additional 44, published mostly before 2000. We excluded articles at full-text for not satisfying the inclusion criteria by country (129), study type (evaluation study) (304), evaluation method (213), outcome (235) and community engagement type (172). We ultimately identified 61 impact evaluations (Table 2)[31-92] that assessed the effects of community engagement interventions on outcomes related to routine child immunisation in LMICs. We identified one publication in Spanish[45], with all others in English. Five studies did not include sufficient data to calculate an effect size, thus 56 studies were included in the meta-analysis. Inter-rater reliabilities were calculated on a sample of studies, and ranged from 28% (mean effect of the intervention to 100% (e.g., country, publication year, study design). All studies were reconciled prior to analyses.

[Place figure 1 here]

For peer review only - http://bmjopen.bmj.com/site/about/guidelines.xhtml

Table 2 describing characteristics of the included studies

Author	Country	Summary of intervention	Engagement type	Study design	Duration (in months)	Outcomes
Admassie 2009[31]	Ethiopia	Formation of a cadre of community-based health extension workers and using community resources for construction of health posts.<br><br>The study evaluates intervention effectiveness and cost-effectiveness.	Multiple (EII[iii] + EAI[iv])	PSM	48	FIC, BCG, DPT3, OPV1, OPV2, OPV3, measles, morbidity
Adamu 2019[32]	Nigeria	Quality improvement programme where health workers use iterative processes to develop localised and contextually relevant plans to resolve health service delivery and demand bottlenecks.<br><br>The study evaluates intervention effectiveness.	Multiple (EID[v] + EII)	ITS	1	Dropouts

[iii] EII: Engagement in implementation autonomy

[iv] EAI: Engagement as intervention

[v] EID: Engagement in Design

For peer review only - http://bmjopen.bmj.com/site/about/guidelines.xhtml

Alhassan 2019[33]	Ghana	Using a bottom-up approach, the intervention first recruited and trained community groups to identify service delivery gaps in healthcare facilities.<br><br>The study evaluates intervention effectiveness.	Multiple (EII + EAI)	RCT	10	FIC
Andersson 2009[34]	Pakistan	Community dialogues to address barriers to vaccination. The guidelines for the dialogue were created after consultation with the intended beneficiaries.<br><br>The study evaluates intervention effectiveness and cost-effectiveness.	Multiple (EID + EAI)	RCT	8	Knowledge about immunisation, attitude about immunisation, community norms, readiness to vaccinate, household norms & decision-making measles, DPT3
Arifeen 2009[35]	Bangladesh	Formation of a cadre of village health volunteers and enlisting support of local religious leaders to convey messages about child health.<br><br>The study evaluates intervention effectiveness.	EAI	RCT	71	Measles, mortality
Assegaai 2018[36]	South Africa	Lay community-based workers were formalised as community health workers and served as a part of the outreach teams.	EAI	DID	36	FIC, measles, morbidity

For peer review only - http://bmjopen.bmj.com/site/about/guidelines.xhtml

		The study evaluates intervention effectiveness.				
Banerjee 2010[37]	India	Provision of immunisation services and incentives to caregivers. A trusted community-based organisation was a key stakeholder in design and delivery of the intervention.<br><br>The study evaluates intervention effectiveness and cost-effectiveness.	Treatment 1: EAI<br>Treatment 2: Multiple (EID + EAI)	RCT	18	FIC, BCG, partial immunisation
Banerjee 2020[38]	India	This evaluation tested two different interventions:<br>1. Incentives to caregivers. The community's feedback was solicited on the kind of incentive.<br>2. Community influencers were identified to spread information about immunisation.<br><br>The study evaluates intervention effectiveness and cost-effectiveness.	EID<br><br>EAI	RCT	14	Knowledge about immunisation, attitude about immunisation, FIC, DPT1, DPT2, DPT3, measles
Banwat 2015[39]	Nigeria	Female members of the community whose children are fully immunised were nominated in each community to serve as peer educators.<br><br>The study evaluates intervention effectiveness.	EAI	CBA	-	FIC, knowledge about immunisation, attitude about immunisation, readiness to vaccinate

For peer review only - http://bmjopen.bmj.com/site/about/guidelines.xhtml

Biemba 2016[40]	Zambia	A national policy to create a cadre of well-trained and motivated community-based health workers.<br><br>The study evaluates intervention effectiveness.	EAI	DID	23	FIC, morbidity
Björkman 2009[41]	Uganda	Communities were involved in monitoring the quality of health services and the performance of health service providers.<br><br>The study evaluates intervention effectiveness and cost-effectiveness.	EII	RCT	0.16	FIC, BCG, OPV0, OPV3, DPT1, DPT3, measles, Partial routine immunisation, mortality
Bolam 1998[42]	Nepal	Training for community health workers and midwives which was developed in collaboration with health workers and experts.<br>The study evaluates intervention effectiveness.	EID	RCT	3	FIC
Borkum 2014[43] Carmichael 2019[44] (linked study)	India	Performance-based incentives to frontline workers. The nature of incentives was decided upon in consultation with the frontline workers.<br><br>The study evaluates intervention effectiveness and cost-effectiveness.	EID	RCT	12	Health card availability, CHW capacity, FIC, BCG, DPT1, DPT2, DPT3, OPV1, OPV2, OPV3, measles, partial immunisation, timeliness

For peer review only - http://bmjopen.bmj.com/site/about/guidelines.xhtml

Calderón-Ortiz & Mejía-Mejía 1996[45]	Mexico	Creation of a community-based cadre of volunteers to register and track children in the community for immunisation.<br>The study evaluates intervention effectiveness.	EAI	CBA	4	FIC, BCG, DPT3, OPV3, measles
Carnell 2014[46]	Ethiopia	Formation of a cadre of community health workers to mobilize the community and encourage uptake of health services.<br><br>The study evaluates intervention effectiveness and cost-effectiveness.	EAI	DID	60	DPT3, measles
Costa-Font 2017[47]	India	Establishment of the village health and sanitation committees to monitor health service provision at the community level.<br><br>The study evaluates intervention effectiveness.	EAI	IV	-	BCG, DPT1, OPV0
Demilew 2020[48]	Ethiopia	A poster/stamp system that reminded health workers of the child's immunisation status and simultaneously encouraged caregivers to immunise their children. The intervention was designed in consultation with health workers.	Multiple (EID + EAI)	RCT	17	FIC, BCG, DPT1, DPT2, DPT3, partial immunisation

For peer review only - http://bmjopen.bmj.com/site/about/guidelines.xhtml

		The study evaluates intervention effectiveness and cost-effectiveness.				
Dipeolu 2017[49]	Nigeria	Text message reminders to mothers regarding immunisation schedule. The messages were field tested with mothers to get the content right.<br>The study evaluates intervention effectiveness.	EID	DID	9	Knowledge about immunisation, attitude about immunisation, timeliness
Domek 2019[50]	Guatemala	SMS text messages to caregivers. A prior feasibility and acceptability study was conducted for the intervention.<br>The study evaluates intervention effectiveness.	EID	RCT	2	Timeliness
Engineer 2016[51]	Afghanistan	Pay-for-performance bonuses paid quarterly to health workers. The bonus amount was revised after receiving health worker feedback.<br>The study evaluates intervention effectiveness.	EID	RCT	24	Experience & satisfaction with health services, Formal HW motivation, capacity & performance, DPT3
Findley 2013[52]	Nigeria	Formation of a cadre of community volunteers to facilitate group discussions on health and track/register women/children for health services.<br><br>The study evaluates intervention effectiveness and cost-effectiveness.	EAI	DID	24	FIC

For peer review only - http://bmjopen.bmj.com/site/about/guidelines.xhtml

Gibson 2017[53]	Kenya	SMS reminders and monetary incentives to caregivers. A feasibility study was conducted in 2013 for this intervention.<br>The study evaluates intervention effectiveness.	EID	RCT	12	Community norms, FIC, BCG, DPT1, DPT2, DPT3, OPV0, OPV1, OPV2, measles, timeliness
Goel 2012[54]	India	A multi-component campaign which involved women groups in awareness generation to improve health service uptake.<br>The study evaluates intervention effectiveness.	EAI	DID	48	FIC
Gurley 2020[55]	India	Community members were trained to design and produce culturally appropriate, 'hyper-local' videos to promote health seeking behaviours.<br><br>The study evaluates intervention effectiveness and cost-effectiveness.	Multiple (EID + EAI)	RCT	11	Knowledge about immunisation, attitude about immunisation, FIC, DPT3, partial immunisation, timeliness, dropouts
Herrera-Almanza 2018[56]	Madagascar	Community-based primary health care services intervention that included the deployment of volunteer community health workers in remote areas.<br>The study evaluates intervention effectiveness.	Multiple (EII + EAI)	DID	26	Health card availability, OPV3, DPT3, measles, mortality, partial immunisation
Igarashi 2010[57]	Zambia	The GMP+ sessions were conducted by medical personnel from Public Health Centers. During these sessions, community volunteers provided some	EII	CBA	43	FIC, timeliness, attitude about immunisation, community norms

For peer review only - http://bmjopen.bmj.com/site/about/guidelines.xhtml

		operational and managerial support to ensure the effective implementation of the sessions.<br>The study evaluates intervention effectiveness.				
Janssens 2011[58]	India	Dissemination of health promoting messages to women in the community who are encouraged to further spread the awareness.<br>The study evaluates intervention effectiveness.	EAI	IV	56.4	DPT3, measles
Johri 2020[59]	India	Interventions, designed through formative research, to increase caregiver knowledge and adherence to childhood immunisation.<br>The study evaluates intervention effectiveness.	EID	RCT	3	Knowledge about immunisation, Awareness of place, time, schedule for vacc., attitude about immunisation
Lee 2015[60]	Zambia	Creation of a new cadre of frontline workers from the community, called community health assistants, to provide primary health care services.<br>The study evaluates intervention effectiveness.	EAU	RCT	3	Formal HW motivation, capacity & performance, BCG, OPV3, measles, timeliness, CHW capacity, morbidity
Mayumana 2017[61]	Tanzania	Payment-for-performance scheme for health facilities. Health workers and health facility governing committees decided the allocation of funds.<br>The study evaluates intervention effectiveness.	EII	DID	30	Stockouts

For peer review only - http://bmjopen.bmj.com/site/about/guidelines.xhtml

Memon 2015[62]	Pakistan	Formation of community health committees to promote perinatal and new-born care. Formative research informed the intervention design.<br>The study evaluates intervention effectiveness.	Multiple (EID + EAI)	DID	16	FIC
Modi 2019[63]	India	The mHealth intervention package consisting of mobile phone-based job aids for community health workers. The intervention was piloted in 2015.<br>The study evaluates intervention effectiveness and cost-effectiveness.	EID	RCT	12	DPT3, morbidity
Mohanan 2020[64]	India	Social accountability interventions to promote community-based collective action to improve delivery of health and nutrition services to children.<br><br>The study evaluates intervention effectiveness and cost-effectiveness.	EAI	RCT	12	Experience & satisfaction with health services, attitudes about health providers, formal health worker supply, FIC, BCG, DPT3, OPV1, OPV3, IPV, measles, morbidity, mortality
More 2012[65]	India	Urban slum-dweller women's groups used community dialogues to address barriers to improving perinatal health.<br>The study evaluates intervention effectiveness.	EAI	RCT	36	Mortality

For peer review only - http://bmjopen.bmj.com/site/about/guidelines.xhtml

More 2017[66]	India	The intervention comprised multiple activities like home visits to caregivers, groups meetings, community events and other supportive services.<br><br>The study evaluates intervention effectiveness and cost-effectiveness.	EAI	RCT	24	Health card availability, attitudes about health providers, FIC, BCG, measles, partial immunisation
Morris 2004[67]	Honduras	Monetary vouchers to women in the communities and setting up of community-based committees to oversee health service quality and access.<br><br>The study evaluates intervention effectiveness and cost-effectiveness.	EAI	RCT	24	DPT1, measles
Murthy 2019[68]	India	Voice call reminders to pregnant women and caregivers. The message content was tested for appropriateness through community focused groups.<br>The study evaluates intervention effectiveness.	EID	RCT	21	Knowledge about immunisation, FIC
Nagar 2018[69]	India	A digital pendant-based health record of the child and a voice call reminder system. A formative study was conducted in 2016 and communities were consulted on the design of the pendant.<br>The study evaluates intervention effectiveness.	EID	RCT	3	Timeliness

For peer review only - http://bmjopen.bmj.com/site/about/guidelines.xhtml

Nagar 2020[70]	India	A digital pendant-based health record of the child. Health providers used a mobile application to scan the pendant to update the child's medical history. Prior formative research informed the intervention design.<br><br>The study evaluates intervention effectiveness and cost-effectiveness.	EID	RCT	20	FIC, DPT1, DPT2, DPT3
Nzioki 2017[71]	Kenya	Formation of a cadre of community health workers.<br>The study evaluates intervention effectiveness.	EAI	CBA	0	FIC
Oche 2011[72]	Nigeria	Group meetings with caregivers and dialogues with community leaders to improve uptake of routine immunization services.<br>The study evaluates intervention effectiveness.	EAI	DID	9	Knowledge about immunisation, DPT1, DPT3, dropouts
Okeke 2017[73]	Nigeria	A national scheme to create, train and deploy a cadre of midwives to serve underserved rural and remote populations in Nigeria.<br>The study evaluates intervention effectiveness.	Multiple (EII + EAI)	DID	40	BCG, DPT3, OPV3, measles, mortality
Okoli 2014[74]	Nigeria	A conditional cash transfer program to encourage uptake of health services. Community groups were consulted while deciding the cash amount.<br>The study evaluates intervention effectiveness.	EID	ITS	7 to 18	OPV0

For peer review only - http://bmjopen.bmj.com/site/about/guidelines.xhtml

Olayo 2014[75]	Kenya	Formation of a cadre of community health workers who then facilitated dialogue at the community level and supported other community-based workers.<br>The study evaluates intervention effectiveness.	Multiple (EII + EAI)	DID	24	Health card availability, DPT1, DPT3, measles
Olken 2014[76]	Indonesia	Block grants for maternal and child health that incorporated relative performance incentives were implemented in villages through creation of village-level health committees.<br><br>The study evaluates intervention effectiveness and cost-effectiveness.	Multiple (EII + EAI)	RCT	18 to 30	FIC, morbidity, mortality
Oyo-Ita 2020[77]	Nigeria	A multi-component intervention involving traditional and religious leaders for engaging communities in planning and delivery of immunisation services.<br><br>The study evaluates intervention effectiveness and cost-effectiveness.	Multiple (EII + EAI)	RCT	18	FIC, partial immunisation, Timeliness
Pramanik 2020[78]	India	Trained facilitators from local NGOs interacted with the communities to enable communities to leverage their own strengths for addressing their concerns related to child health.	EAI	RCT	13	Knowledge about immunisation, attitude about immunisation, attitudes about health providers, health card

For peer review only - http://bmjopen.bmj.com/site/about/guidelines.xhtml

		The study evaluates intervention effectiveness and cost-effectiveness.				availability, FIC, DPT1, DPT2, DPT3, timeliness, dropouts
Rahman 2008[79]	Pakistan	Mental health support program with counselling sessions for pregnant and post-partum women. Prior intervention pilots informed the intervention design. The study evaluates intervention effectiveness.	EID	RCT	11	FIC, morbidity
Rahman 2016[80]	Bangladesh	New cadre of community health workers delivered essential maternal, neonatal, and child healthcare and nutrition services. The study evaluates intervention effectiveness.	EAI	DID	48	FIC, morbidity
Rao 2014[81]	India	Creation of a cadre of community health workers to improve basic health outcomes through community engagement. The study evaluates intervention effectiveness.	EAI	DID	60	BCG, DPT3, OPV3, measles, FIC, partial immunisation, supply of CHWs
Robertson 2013[82]	Zimbabwe	Use of cash transfers for behaviour change. Local NGO and community leaders were involved in beneficiary targeting and compliance monitoring. The intervention was also tested for feasibility during a prior study. The study evaluates intervention effectiveness.	Multiple (EID + EII)	RCT	12	FIC, community norms, OPV0
Roy 2008[83]	Bangladesh	Rural maintenance programme recruited and trained women for road maintenance, health awareness,	EAI	DID	11	FIC, BCG, DPT1, DPT2, DPT3, OPV0, OPV1,

For peer review only - http://bmjopen.bmj.com/site/about/guidelines.xhtml

		numeracy, human rights, gender equity, health and nutrition, and business management.				OPV2, OPV3, measles, partial immunisation
Saggurti 2018[84]	India	Formation of health-focused self-help groups with women of reproductive age coming from the most marginalized communities.<br><br>The study evaluates intervention effectiveness and cost-effectiveness.	EAI	DID	2	Timeliness
Sankar 2013[85]	India	Formation of committees with representatives of the community, local government and service providers to ensure better convergence and coordination of service delivery.<br>The study evaluates intervention effectiveness.	Multiple (EII +EAI)	DID	30	FIC, BCG, DPT1, DPT2, DPT3, OPV0, OPV1, OPV2, OPV3, measles, partial immunisation, timeliness
Seth 2018[86]	India	The study evaluated two different interventions: role of compliance-linked incentives versus text messaging to improve childhood immunisations. Incentive amount was determined after input was received from the local investigators as well as the community workers.<br><br>The study evaluates intervention effectiveness and cost-effectiveness.	EID	RCT	9.7	Partial immunisation, timeliness, attitude about immunisation, attitudes about health providers

For peer review only - http://bmjopen.bmj.com/site/about/guidelines.xhtml

Shukla 2018[87]	Afghanistan	Community representatives along with health officials identify the health needs of the communities and communicate those to the service providers. The study evaluates intervention effectiveness.	EII	DID	6	Supply of CHWs, DPT3
Siddiqi 2020[88]	Pakistan	Visual reminders to caregivers in the form of wearable bracelets for the child. The bracelets were designed in consultation with the caregivers. The study evaluates intervention effectiveness.	EID	RCT	12	DPT3, measles
Tandon 1988[89]	India	Enlisting community-based volunteers to motivate and encourage family members to utilise maternal and child health services. The study evaluates intervention effectiveness.	EAI	CBA	120	FIC, BCG, DPT2, DPT3, OPV2
USAID 2008[90]	Ethiopia	Creation of a cadre of community health promoters to carry out behaviour change communication activities in the communities. The study evaluates intervention effectiveness.	EAI	CBA	48	FIC, BCG, DPT1, DPT3, OPV3, morbidity, health card availability, measles, dropouts
Webster 2019[91]	Uganda	Community-based outreach and follow-up with caregivers to improve immunisation uptake and reduce defaulters. The study evaluates intervention effectiveness and cost-effectiveness.	EII	RCT	12	Health card availability, BCG, DPT1, DPT2, DPT3, OPV0, OPV1, OPV2, OPV3, IPV, measles, partial immunisation, timeliness, attitude about

For peer review only - http://bmjopen.bmj.com/site/about/guidelines.xhtml

						immunisation, dropouts, morbidity, mortality
Younes 2014[92]	Bangladesh	The intervention involved 162 women's groups who used participatory approaches to discuss maternal and neonatal health issues.<br>The study evaluates intervention effectiveness.	EAI	DID	20	FIC, morbidity

For peer review only

Risk of Bias

Of the 31 included studies with experimental designs, only two had a low risk of bias, six had some concerns and 23 had a high risk of bias. Of the 30 included quasi-experimental studies, only two were assessed as low risk of bias, one as some concerns and 27 as high risk of bias. Biases arising from outcome measurement and deviations from intended interventions were the most common across both the study designs.

Although only five qualitative studies scored strong on all key elements, most studies received strong scores on most key elements and had quality appraisal scores greater than 20, indicating low risk of bias. The most common elements found to be missing were sample characteristics and analytic methods. The quality of the cost evidence in the 22 studies that included such evidence was mixed (further information and visualisations for the risk of bias appraisals can be found in Appendix 8).

Community Engagement Interventions

Full Immunisation

A total of $k = 28$ studies examined the effect of community engagement interventions on full childhood immunisation and found $\hat{\mu} = 0.14$ ([95% CI: 0.06 to 0.23], $z = 3.28$, $p = .001$), indicating a small but significant benefit for the treated group of .14 standard deviation units (Figure 2). A 95% credibility/prediction interval for the true outcomes is given by -0.28 to 0.57. Hence, although the average outcome is estimated to be positive, in some studies the true outcome may in fact be negative.

The rank correlation test indicated funnel plot asymmetry ($p = 0.03$) but the regression test did not ($p = 0.57$; see Appendix 9 for additional information). The true effects appear to be heterogeneous (I^2 = 94.5%, $\tau = 0.21$). Outlier analyses revealed that Banerjee (2010) may be a potential outlier, and sensitivity analyses removing Banerjee (2010) reduced the overall average effect (μ = 0.08 [95% CI: 0.04 to 0.12]), but it was still positive and significant (z = 4.12, p <

For peer review only - http://bmjopen.bmj.com/site/about/guidelines.xhtml

.001).[vi] Sensitivity analysis using the leave one out approach indicates there are no other studies whose removal results in substantial changes to the average effect or overall heterogeneity.

Sensitivity analyses were also conducted to examine the robustness of the results to the exclusion of low-quality studies. When studies assessed as high risk of bias were removed, the resulting effect was slightly larger and still statistically significant ($\hat{\mu} = 0.18$ [95% CI: 0.08 to 0.27]), $k = 4, z = 3.67$, $p < 0.001$). We examined several potential sources of heterogeneity, including exposure to the intervention, evaluation period, study design, year, geographical region, data source, whether the intervention was implemented by a government agency (either alone or in combination with another agency), whether new cadres of health workers were established, presence of vaccine hesitancy, and baseline vaccine coverage rates. There were no significant moderators in the context of this model (see Appendix 9).

[Place figure 2 here]

DPT 3

A total of $k = 22$ studies examined the effect on DPT3 vaccination coverage and found a small but significant benefit to the treated group compared to the untreated group ($\hat{\mu} = 0.10$ [95% CI: 0.06 to 0.14], $z = 4.75$, $p < 0.001$; Figure 3). A 95% credibility/prediction interval for the true outcomes is given by -0.06 to 0.26. Hence, although the average outcome is estimated to be positive, in some studies the true outcome may in fact be negative.

The true outcomes appear to be heterogeneous ($I^2 = 76.8\%$, $\tau = 0.08$). When low quality studies were removed, the average effect increased slightly ($\hat{\mu} = 0.11$ [95% CI: 0.05 to 0.17], $k = 4$), and was still statistically significant ($z = 3.70$, $p < 0.001$;see Appendix 9). Publication year was a significant source of heterogeneity; each additional year reduced the size of the effect by .014 standard deviation units (see Appendix 9).

[Place figure 3 here]

Measles

[vi] For all other outcomes, outlier analyses will be presented in the Appendices.

For peer review only - http://bmjopen.bmj.com/site/about/guidelines.xhtml

A total of $k = 20$ studies examined the effect on measles vaccination coverage and found a very small but significant benefit for the treated group compared to the untreated group ($\hat{\mu} = 0.07$ ([95% CI: 0.03 to 0.11], $z = 3.22$, $p < 0.01$; *see Figure 4*). A 95% credibility/prediction interval for the true outcomes is given by -0.08 to 0.22. Hence, although the average outcome is estimated to be positive, in some studies the true outcome may in fact be negative.

When low quality studies were removed, the average effect increased ($\hat{\mu} = 0.09$, $k = 6$, [95% CI: 0.03 to 0.15]) and was still statistically significant ($z = 2.98$ $p = 0.003$; see Appendix 9). The true outcomes appear to be heterogeneous ($I^2 = 73.6\%$, $\tau = 0.07$). None of the moderators were significant sources of heterogeneity (see Appendix 9).

[Place figure 4 here]

Vaccination Timeliness

We found a small but significant effect on all three timeliness outcomes: full immunisation timeliness ($\hat{\mu} = 0.15$ [95% CI: 0.07 to 0.24], $z = 3.41$, $p < 0.001$, 95% prediction interval 0.04 to 0.27); DPT3 timeliness ($\hat{\mu} = 0.09$ [95% CI: 0.03 to 0.14], $z = 3.00$, $p < 0.01$, 95% prediction interval 0.03 to 0.14); and measles timeliness ($\hat{\mu} = 0.23$ [95% CI: 0.14 to 0.32], $z = 5.06$, $p < 0.001$, 95% prediction interval 0.14 to 0.32). For all timeliness outcomes, tests of heterogeneity were not significant ($p > .05$). For full immunisation and measles timeliness outcomes, the sensitivity analysis could not be conducted due to an inadequate number of studies. For DPT3, the average effect increased but became non-significant when low quality studies were removed. (See Appendix 9).

Subgroups of community engagement interventions

Studies that used engagement as the intervention had a significant positive effect on full childhood immunisation, DPT3 vaccination and measles vaccination, but evidence was insufficient to synthesize measures of vaccination timeliness (Table 3).

When studies used community engagement in the design, there was a significant positive effect on full childhood immunisation and measles vaccination but not on DPT3 vaccination. Additionally, there was a positive significant effect on timeliness of full childhood immunisation and DPT3

For peer review only - http://bmjopen.bmj.com/site/about/guidelines.xhtml

vaccination. No studies using engagement in the design reported on timeliness of measles vaccinations.

For engagement in implementation autonomy, the analysis is based on a limited number of studies and we found no significant effect on either coverage or timeliness outcomes. There were no studies reporting on the timeliness of measles vaccination.

Finally, some studies combined multiple engagement types in their interventions. These interventions had a significant effect on DPT3 vaccination but not on measles vaccination or full childhood vaccination. Evidence was insufficient to synthesise measures of vaccination timeliness.

Secondary outcomes analyses and supplementary material can be found in Appendices 10-13.

Table 3. Overview of the primary outcomes by engagement type

	**Random effects summary estimate for all interventions**	**Engagement as the intervention**	**Engagement in the design**	**Engagement in the implementation autonomy**	**Multiple engagement types**
	g, [95% CI], I^2, (k)	g, [95% CI], I^2, (k)	g, [95% CI], I^2, (k)	g, [95% CI], I^2, (k)	g, [95% CI], I^2, (k)
**Full immunisation**	**0.14**, [0.06, 0.23],** 94.46, (28)	**0.08**, [0.03, 0.13],** 70.00, (12)	**010*, [0.02, 0.19],** 23.83, (5)	0.23, [-0.001, 0.47], 73.07, (2)	0.22, [-0.12, 0.56], 97.94, (9)
**Measles**	**0.07**, [0.03, 0.11],** 73.64, (20)	**0.10***, [0.05, 0.15],** 60.29, (10)	**0.11*, [0.02, 0.21],** 0.00, (2)	0.03, [-0.09, 0.15], 54.84, (2)	0.03, [-0.10, 0.16], 86.76, (6)
**DPT3**	**0.10***, [0.06, 0.14],** 76.78, (22)	**0.09**, [0.03, 0.15],** 73.17, (6)	0.04, [-0.01, 0.08], 0.00, (6)	0.11, [-0.05, 0.28], 80.12, (3)	**0.20**, [0.06, 0.34],** 87.93, (7)

For peer review only - http://bmjopen.bmj.com/site/about/guidelines.xhtml

**Timeliness (DTP3)**	**0.09**, [0.03, 0.14],** 0.00, (7)	N/A	**0.12**, [0.03, 0.21],** 0.00, (4)	0.04, [-0.06, 0.13], 0.00, (2)	N/A
**Timeliness (Measles)**	**0.23***, [0.14, 0.32],** 0.00, (3)	N/A	N/A	N/A	N/A
**Timeliness (full immunisations)**	**0.15***, [0.07, 0.24],** 9.66 (5)	N/A	**0.15*, [0.004, 0.29], 7.202, (2)**	0.38, [-0.28, 1.03], 0.00, (2)	N/A

**** p < .05, ** p < .01, *** p < .001,***

Qualitative findings

Programme design characteristics were associated with intervention success or failure across all engagement types. Certain aspects of community engagement itself, such as conducting stakeholder consultations, holding community dialogues or involving community leaders were associated with better immunisation outcomes. Non-engagement intervention design features also affected intervention success. These design features include incentives given to caregivers and leadership and supportive supervision, which improved overall health service delivery and health worker performance. Among the studies that attributed intervention failure to programme design, inadequate duration, frequency or exposure to the intervention were the most notable themes.

The importance of accounting for *contextual* barriers to or facilitators of immunisation emerged consistently. Limited availability of services, especially insufficient staff and vaccine supply, were dominant barriers to immunisation, affecting outcomes in the early portion of the causal chain. Other common barriers to immunisation included practical barriers faced by caregivers such as costs, largely indirect, and logistics (wait time and language barriers) or distance. There was more variation in barriers related to social norms, fear, and an understanding of the importance of immunisation by type of engagement. Poor quality of services, including uninviting attitudes of

For peer review only - http://bmjopen.bmj.com/site/about/guidelines.xhtml

health workers, posed a barrier to immunisation in communities that received engagement as the intervention or were engaged in the design of the intervention.

However, we also found that certain contextual factors could become facilitators of immunisation outcomes, provided an intervention has adequately situated itself to leverage them. Across all engagement types, studies associated caregivers' awareness and perception of the benefits of vaccination with improved immunisation outcomes. Similarly, availability of health infrastructure and good quality of services were also associated with improved intervention uptake and its impact.

Implementation failures, such as low fidelity, were a common reason for intervention failure. Across all engagement types, interventions did not properly account for existing implementation constraints and practicalities on the ground and were forced to change their implementation plans. Many of these issues were encountered due to uncontrollable factors or invalid theory of change assumptions. For instance, programme design may not have accounted for the unavailability of intended participants due to competing priorities, thereby potentially invalidating the assumption of beneficiary exposure in the casual pathway. Administrative challenges were cited consistently though their nature varied across engagement types ranging from technical limitations (such as limited cellphone service) to political constraints to insufficient staffing levels.

Results were broadly consistent when only qualitative studies with a quality appraisal score greater than 20 were considered. The full qualitative synthesis and sensitivity analysis is available in Appendix 14.

Cost effectiveness findings

Among the 14 studies for which we could calculate cost-effectiveness, we found that the median intervention cost per treated child per vaccine dose ￼ (excluding the cost of vaccines) to increase absolute immunisation coverage by one percent was USD 3.68 (all costs are reported in 2019 US dollars) and the average cost was USD 46.60. There were three outlier observations that drove up this average cost and without them cost per vaccine dose to increase absolute immunisation coverage by one percent averaged USD 3.97 (Appendix 15).

For peer review only

For peer review only - http://bmjopen.bmj.com/site/about/guidelines.xhtml

Discussion

Principal findings

We found that community engagement interventions had a small, but significant, positive effect on all primary immunisation outcomes related to coverage and their timeliness. We also found that certain features of interventions may contribute to their success. These include (a) appropriate intervention design, including building in community engagement features; (b) addressing common contextual barriers of immunisation and leveraging facilitators; and (c) accounting for existing implementation constraints and practicalities on the ground. The median intervention cost per treated child per vaccine dose (excluding the cost of vaccines) to increase absolute immunisation coverage by one percent was USD 3.68.

Among the four types of community engagement interventions, we found that engagement as the intervention (embedded community engagement), which involves creation of community buy-in or development of new community-based structures or cadres, had consistent positive effects on more primary vaccination coverage outcomes than the others. We also found engaging the community in the design of the intervention had a positive effect on most primary outcomes related to coverage. We found no ubiquitous patterns of heterogeneity among the primary outcomes.

While zero dose children were not the specific focus of this review, we can offer some insights based on our analyses of both DPT1 and BCG outcomes, which reflect access to initial dosing. Community engagement interventions did not show an effect on DPT1, but the evidence base was of low quality, with six of eight studies assessed as having a high risk of bias. There was a small but significant effect of community engagement interventions on BCG, but here again, the evidence base was of lower quality, with nine of 12 studies assessed as having a high risk of bias. In both cases, the evidence base was smaller in size than for the primary outcomes. As we find positive effects of community engagement on children returning for DPT and measles doses, it may be that barriers to vaccination, like availability of health services, for zero dose children are different and unless those are addressed, community engagement itself may not be enough.

Strengths and weaknesses

Our systematic review uses a detailed framework of community engagement interventions to assess their effectiveness for improving outcomes related to routine child immunisation in LMICs. As far as we are aware, ours is the first systematic review to do this. Sensitivity analyses excluding high risk of bias studies showed that the effect was slightly larger and still statistically significant

For peer review only - http://bmjopen.bmj.com/site/about/guidelines.xhtml

for almost all the primary outcomes for which we had sufficient data. The effects were also uniform across geographies and baseline immunisation rates.

We drew on 61 studies for meta-analysis, comprising 31 RCTs and 30 quasi experimental studies. However, only 56 studies provided sufficient information for calculating effect sizes and thus were included in meta-analytic models. For full immunisation, DPT3 and measles coverage, we could draw upon 28, 22 and 20 studies respectively for pooled effects. However, for the timeliness of these coverage outcomes we had only 0 to 7 studies to assess the pooled effects. Thus, while for some outcomes the evidence base for drawing conclusions is adequate, for others it is limited. Among the four kinds of community engagement interventions, there was a relatively large evidence base for those with engagement as the intervention and those with multiple engagement types, while for interventions using engagement in implementation autonomy, the evidence was quite limited.

We identified additional documentation comprising qualitative studies, project reports, formative/process evaluations and observation studies for 39 of the 61 included impact evaluations. However, the crucial qualitative papers which help us gain a deeper understanding of overall intervention mechanisms of change were found for only 17 of the 61 IEs. Likewise, only 14 of the primary studies included in this review both estimated the intervention cost and reported it with sufficient detail for the review team to calculate the cost-effectiveness of the treatment. Low quality cost data and the unavailability of underlying cost data contributed to the small number of primary studies included in the cost-effectiveness analysis.

The quantitative evidence was mostly low quality, though the randomised studies were generally of higher quality and less likely to have confounding bias than the quasi-experimental studies. The quality of qualitative studies was generally high. The quality of the cost evidence was mixed. Despite the quality concerns about quantitative evidence, the sensitivity analysis conducted by excluding low-quality studies corroborated the overall findings. Despite a comprehensive search strategy and the inclusion of grey literature, publication bias was detected for the three primary coverage outcomes (full immunisation, DPT3, and measles). While bias correction analyses indicated an identical effect size for full immunisation and DPT3, the effect for measles was reduced when publication bias was corrected for. Timeliness outcomes had an insufficient number of studies to test for publication bias, which limits our ability to interpret heterogeneity.

For peer review only - http://bmjopen.bmj.com/site/about/guidelines.xhtml

Agreement and disagreement with other reviews

The findings from this review are broadly consistent with Molina et al. 2016[5] which found positive effects of community monitoring interventions on immunisation coverage. Another review by Gilmore and McAuliffe (2013)[4] examined the effectiveness of preventive interventions delivered by community health workers for maternal and child health in LMICs on essential newborn care and found some evidence in its support through narrative synthesis, but found the evidence base to be insufficient to draw firm conclusions.

Limitations

There are several potential limitations to the current review: a) there were few analyses that were sufficiently powered to test for publication bias, thus we cannot rule this out in many cases; b) many of the moderator analyses were underpowered, meaning that in many cases we were unable to explore heterogeneity. This was particularly true in the context of the subgroup analyses of the four intervention types. In addition, it is likely that there is inter-dependency among moderator variables, but the current study did not allow for us to disentangle these confounds. Future studies may aim to better assess how moderators may work in tandem to affect the magnitude of change; c) even in cases where the average effect was significant, forest plots demonstrate that some of the included studies reported a small negative affect, and prediction intervals often included both positive and negative values, which may have important implications when making decisions related to programme design and implementation; d) we also observed very few studies which focused on sub-population groups. This is particularly problematic given the focus on LMICs, where equity is important to consider when trying to increase coverage; e) most of the community engagement interventions were in combination with other intervention components, thus we were not able to establish their unique contribution to changes in outcomes; f) inclusion of primary studies into this review was based on the description of the community engagement aspects of the intervention. We may have excluded studies that should ideally have been included because of inadequate reporting of intervention components. Finally, funding for this project has concluded, thus we do not have the resources to update our literature search last conducted in May of 2020.

Implications for policy and practice

COVID-19 has impacted routine child immunisation negatively in some countries, and community engagement interventions could be an effective way to counteract this decline. The positive effects of community engagement interventions can be expected across a variety of settings, although some engagement approaches appear to be more effective than others. Positive design features

For peer review only - http://bmjopen.bmj.com/site/about/guidelines.xhtml

should be integrated into these interventions, including features such as holding community dialogues or involving community leaders, and non-community engagement features such as local supportive supervision and incentives to healthcare workers or caregivers. Wherever possible, binding contextual barriers to immunisation, such as weak health systems and social norms, should be accounted for in the design of interventions. Existing contextual facilitators for immunisation, such as good existing health systems or high maternal education, could be leveraged for increasing intervention impacts. Important implementation pre-conditions, such as regular internet service or sufficient staffing, should be assessed and established before the implementation or addressed through the design itself. Close monitoring of intervention implementation along with good understanding of context is important to help make necessary modifications in case of unexpected challenges, such as political instability.

Further research

For better-quality evidence and deeper mechanistic understanding, policy makers and practitioners should consider prioritising funding or commissioning research in the following areas: (a) ways of ameliorating outcome measurement bias due to self-reported immunisation coverage outcomes, as this was a principal source of bias; (b) better reporting of interventions, more rounded analysis of why the interventions worked through mixed-methods evaluations and greater focus on intermediate outcomes for improved understanding of causal mechanisms; (c) collection and reporting of high quality cost data to enable cost-effectiveness analysis, which is important for decision making within budget constraints; and (d) focus on sub-group analysis, including for zero dose children, for ensuring immunisation services for the most marginalized children. It would also be useful to conduct an update of this review to include evidence produced since our final literature search in May of 2020.

Contributors

MJ, ME and AB conceived the review and wrote the initial protocol. ME and AB did the systematic search. ME, MJ and AB screened and identified studies and MJ and AB made final decisions regarding study inclusion. SS did the statistical analysis. CL and AB did the qualitative analysis. EB synthesised the cost evidence. MJ provided critical inputs on the whole analysis, checked data, coordinated the review and had full access to all materials and results. External consultants supported the authors in search, screening, data extraction and critical appraisal of quantitative and

For peer review only - http://bmjopen.bmj.com/site/about/guidelines.xhtml

qualitative evidence base. All authors critically reviewed and revised the manuscript and approved the final document for submission. MJ is responsible for the overall content and is the guarantor.

Funding and competing interests

This research was funded through a grant from the Bill & Melinda Gates Foundation (OPP1115129) to the International Initiative for Impact Evaluation (3ie). Through this grant, 3ie provided funding and technical assistance for seven impact evaluations of community engagement interventions for immunisation as a part of its immunisation evidence programme. This technical assistance included, but was not limited to: reviewing study designs, analysis plans, and data collection instruments; advising research teams on how to improve study components and address challenges that arise during the course of the evaluation; and supporting grantees in engaging with stakeholders to promote uptake and use of evidence generated by the evaluations.

As members of 3ie staff, authors MJ, AB and ME have all had varying levels of involvement in reviewing deliverables for these evaluations and providing research teams with technical assistance. Several procedural safeguards and transparency measures were put in place to mitigate the risk this conflict of interest imposed. First, all candidate studies, including those funded by 3ie, underwent a rigorous multi-step screening process, including review at the title, abstract, and full-text levels. To qualify for inclusion in the SR, a study was judged to meet the inclusion criteria related to study design, outcomes and population by two independent screeners who have reviewed the full text of the study. The 3ie study authors were responsible for assessing whether the studies met the inclusion criteria for community engagement because of the complexity of the framework. However, these authors have no financial interest in this area and have not published any prior reviews on the topic. The remaining study authors have no conflicts of interest to declare.

Role of the funding source

The funder of the study had no role in study design, data collection, data analysis, data interpretation, or writing of the report.

Data availability statement

For peer review only - http://bmjopen.bmj.com/site/about/guidelines.xhtml

The impact evaluations and other documents included in this systematic review are public and available. The quantitative, qualitative and cost-related data extracted from these studies are available from the corresponding author upon request.

Acknowledgments

This research has been undertaken as a part of 3ie's immunisation evidence programme, supported by the Bill and Melinda Gates Foundation, Seattle, USA. We would like to thank Sohail Agha, Senior Program Officer, Gates Foundation, Seattle, for his continued support and engagement on the evidence programme and the review. Special thanks to Molly Abbruzzese whose guidance helped shape the scope of our evidence programme and aided the conceptualisation of this review. We thank Lisa Menning, Danielle Pedi, Jennifer (Fluder) Siler and Pedja Stocjicic for their valuable feedback on the review.

We wish to thank John Eyers, Yoav Vardy, Daniela Anda, Shradha Parsekar, Sejal Luthra, Yue Zhan, Aditi Hombali, Lalitha Vadrevu, Reva Datar, Pankhuri Jha, Beáta Berkovics, Ashton Baafi, Meital Kupfer David Atika, Himani Aggarwal, Agrima Sahore, Mansi Wadhwa, and Harini Narayanan for excellent support and research assistance. We would further like to thank Marie Gaarder, Executive Director, Birte Snilstevit, Director of Synthesis and Reviews Office and Sebastian Martinez, Director of Evaluation Office, 3ie, for their guidance on the review process.

References

1. Ozawa S, Clark S, Portnoy A, et al. Estimated economic impact of vaccinations in 73 low-and middle-income countries, 2001–2020. Bulletin of the World Health Organization. 2017;95(9):629. doi: 10.2471/BLT.16.178475.
2. World Health Organization. Immunization Coverage. World Health Organisation. 2019 Available from: https://www.who.int/news-room/fact-sheets/detail/immunization-coverage.
3. World health Organization. Immunization Agenda 2030: A Global Strategy to Leave No One Behind. World Health Organization, Geneva. 2020 Available from: https://www.who.int/teams/immunization-vaccines-and-biologicals/strategies/ia2030
4. Gilmore B, McAuliffe E. Effectiveness of community health workers delivering preventive interventions for maternal and child health in low- and middle-income countries: a systematic review. BMC public health 2013;13(1):1-14.

For peer review only - http://bmjopen.bmj.com/site/about/guidelines.xhtml

5. Molina E, Carella L, Pacheco A, Cruces G, Gasparini L. Community monitoring interventions to curb corruption and increase access and quality of service delivery in low- and middle-income countries: a systematic review. Campbell Systematic Reviews 2016;12(1):1-204.
6. Jain M, Engelbert M, Gaarder M, Bagai A, Eyers J. PROTOCOL: Use of community participation interventions to improve child immunisation in low-and middle-income countries: A systematic review and meta-analysis. Campbell Systematic Reviews. 2020;16(4). doi: 10.1002/cl2.1119.
7. The Steering Group of the Campbell Collaboration. Campbell Systematic Reviews: Policies and Guidelines. The Campbell Collaboration. 2017.
8. Hammerstrøm K, Wade A, Jørgensen A.-MK. Searching for studies: A guide to information retrieval for Campbell Systematic Reviews. Campbell Systematic Reviews 2010;Supplement 1. Available at: www.campbellcollaboration.org/lib/download/969/
9. Higgins J, Green S. Cochrane handbook for systematic reviews of interventions. (version 5.0.2, updated September 2009). The Cochrane Collaboration. 2011.
10. Shadish W, Myers D. Research design policy brief. Oslo: Campbell Collaboration 2014. Available from: http://www.campbellcollaboration.org/artman2/uploads/1/C2_Research_Design_Policy_Brief-2.pdf
11. White H. Theory-based Impact Evaluation: Principles and Practice. Working paper series No. 3. New Delhi: 3ie. 2009.
12. Snilstveit B. Systematic reviews: from ‘bare bones’ reviews to policy relevance. Journal of development effectiveness. 2012;4(3):388-408. doi: 10.1080/19439342.2012.709875.
13. Waddington H, Aloe AM, Becker BJ, et al. Quasi-experimental study designs series—paper 6: risk of bias assessment. Journal of Clinical Epidemiology. 2017;89:43-52. doi: 10.1016/j.jclinepi.2017.02.015.
14. World Health Organization. Community engagement: a health promotion guide for universal health coverage in the hands of the people. Geneva: World Health Organization; 2020. Licence: CC BY-NC-SA 3.0 IGO. Available from https://www.who.int/publications/i/item/9789240010529

For peer review only - http://bmjopen.bmj.com/site/about/guidelines.xhtml

15. Brunton G, Thomas J, O'Mara-Eves A, Jamal F, Oliver S, Kavanagh J. Narratives of community engagement: a systematic review-derived conceptual framework for public health interventions. BMC Public Health 2017;17(1):944. doi: 10.1186/s12889-017-4958-4.
16. Sterne JAC, Savović J, Page MJ, et al. RoB 2: a revised tool for assessing risk of bias in randomised trials. BMJ 2019;366:l4898. doi: 10.1136/bmj.l4898.
17. Sterne JAC, Hernán MA, Reeves BC, et al. ROBINS-I: a tool for assessing risk of bias in non-randomized studies of interventions. BMJ 2016; 355; i4919; doi: 10.1136/bmj.i4919.
18. Critical Appraisal Skills Programme. CASP Qualitative Checklist. 2018. Available at: https://casp-uk.b-cdn.net/wp-content/uploads/2018/03/CASP-Qualitative-Checklist-2018_fillable_form.pdf.
19. Doocy S, Tappis H. Cash-based approaches in humanitarian emergencies: a systematic review. Campbell Systematic Reviews. 2017;13(1):1-200. doi: 10.4073/csr.2017.17.
20. Shemilt I, Mugford M, Byford S, et al. The Campbell Collaboration Economics Methods Policy Brief. Oslo: Campbell Collaboration. 2008. http://www.campbellcollaboration.org/artman2/uploads/1/Economic_Methods_Policy_Brief.pdf
21. Drummond MF, Sculpher MJ, Claxton K, Stoddart GL, Torrance GW. Methods for the economic evaluation of health care programmes. Oxford: Oxford university press, 2015.
22. Borenstein M, Hedges LV, Higgins JPT, Rothstein H. Introduction to Meta Analysis (Statistics in Practice). Chichester: John Wiley & Sons, 2009.
23. Ellis PD. The essential guide to effect sizes: Statistical power, meta-analysis, and the interpretation of research results. Cambridge: Cambridge University Press, 2010.
24. Keef SP, Roberts LA. The meta-analysis of partial effect sizes. British Journal of Mathematical and Statistical Psychology. 2004;57(1):97-129. doi: 10.1348/000711004849303.
25. DerSimonian R, Laird N. Meta-analysis in clinical trials. Controlled clinical trials 1986;7(3):177-88. doi:10.1016/0197-2456(86)90046-2
26. Cochran WG. The combination of estimates from different experiments. Biometrics 1954;10:101-129. doi: 10.2307/3001666.
27. Higgins JPT, Thompson SG. 2002. Quantifying heterogeneity in a meta-analysis. Statistics in medicine 2002;21(11):1539-58. doi:10.1002/sim.1186.

For peer review only - http://bmjopen.bmj.com/site/about/guidelines.xhtml

28. R Core Team. R: A language and environment for statistical computing. Vienna, Austria: R Foundation for Statistical Computing, 2018. Available from https://www.R-project.org/
29. Viechtbauer W. Conducting meta-analyses in r with the metafor package. Journal of Statistical Software 2010;36(3):1–48. doi: 10.18637/jss.v036.i03
30. Borenstein M, Hedges LV, Higgins JP, Rothstein HR. Introduction to meta-analysis. 2nd ed. John Wiley & Sons; 2021.
31. Admassie A, Abebaw D, Woldemichael AD. Impact evaluation of the Ethiopian health services extension programme. Journal of Development Effectiveness. 2009;1(4):430-49. doi: 10.1080/19439340903375724.
32. Adamu AA, Uthman OA, Gadanya MA, Wiysonge CS. Implementation and evaluation of a collaborative quality improvement program to improve immunization rate and reduce missed opportunities for vaccination in primary health-care facilities: a time series study in Kano, Nigeria. Expert review of vaccines. 2019;18(9):969-91. doi: 10.1080/14760584.2019.1647782
33. Alhassan RK, Nketiah-Amponsah E, Ayanore MA, Afaya A, Salia SM, Milipaak J, Ansah EK, Owusu-Agyei S. Impact of a bottom-up community engagement intervention on maternal and child health services utilization in Ghana: a cluster randomised trial. BMC public health. 2019;19(1):1-1. doi: 10.1186/s12889-019-7180-8.
34. Andersson N, Cockcroft A, Ansari NM, et al. Evidence-based discussion increases childhood vaccination uptake: a randomised cluster controlled trial of knowledge translation in Pakistan. BMC International Health and Human Rights. 2009;9(1):1-9. doi: 10.1186/1472-698X-9-S1-S8
35. Arifeen SE, Hoque DE, Akter T, et al. Effect of the Integrated Management of Childhood Illness strategy on childhood mortality and nutrition in a rural area in Bangladesh: a cluster randomised trial. The Lancet. 2009;374(9687):393-403. doi: 10.1016/S0140-6736(09)60828-X.
36. Assegaai T, Reagon G, Schneider H. Evaluating the effect of ward-based outreach teams on primary healthcare performance in North West Province, South Africa: A plausibility design using routine data. South African Medical Journal. 2018;108(4):329-35. doi: 10.7196/SAMJ.2017.v108i4.12755.

For peer review only - http://bmjopen.bmj.com/site/about/guidelines.xhtml

37. Banerjee AV, Duflo E, Glennerster R, Kothari D. Improving immunisation coverage in rural India: clustered randomised controlled evaluation of immunisation campaigns with and without incentives. The BMJ. 2010;340:c2220. doi: 10.1136/bmj.c2220.

38. Banerjee A, Chandrasekhar A, Duflo E, et al. Evaluating the Impact of Interventions to Improve Full Immunisation Rates in Haryana, India. Impact Evaluation Report 126. New Delhi: International Initiative for Impact Evaluation (3ie) 2020. https://doi.org/10.23846/TW10IE126.

39. Banwat ME, Lar LA, Abok IA, Yiltok ES. Effect of peer education on knowledge, attitude and completeness of childhood routine immunization in a rural community of Plateau State. Research Journal of Health Sciences. 2015;3(4):264-74.

40. Biemba G, Yeboah-Antwi K, Vosburg KB, et al. Effect of deploying community health assistants on appropriate treatment for diarrhoea, malaria and pneumonia: quasi-experimental study in two districts of Zambia. Tropical Medicine & International Health. 2016;21(8):985-94. doi: 10.1111/tmi.12730.

41. Björkman M, Svensson J. Power to the people: evidence from a randomized field experiment on community-based monitoring in Uganda. The Quarterly Journal of Economics. 2009;124(2):735-69. doi: 10.1162/qjec.2009.124.2.735

42. Bolam A, Manandhar DS, Shrestha P, Ellis M, Anthony MD. The effects of postnatal health education for mothers on infant care and family planning practices in Nepal: a randomised controlled trial. Bmj. 1998;316(7134):805-11. doi: 10.1136/bmj.316.7134.805.

43. Borkum E, Rangarajan A, Rotz D, Sridharan S, Sethi S, Manorajini M. Evaluation of the Team-Based Goals and Performance-Based Incentives (TBGI) Innovation in Bihar. Mathematica Policy Research; 2014. https://ideas.repec.org/p/mpr/mprres/d8e1097122ff47a6bf42580c82677834.html.

44. Carmichael S, Mehta K, Raheel H, et al. Effects of team-based goals and non-monetary incentives on front-line health worker performance and maternal health behaviours: a cluster randomised controlled trial in Bihar, India. BMJ Global Health. 2019;4(4):e001146. doi: :10.1136/bmjgh-2018-001146

45. Calderón-Ortiz, R, Jesus M. Estrategia de contratacion permanente dentro del Programa de Vacunacion Universa. Salud Pública de México 1996;38(4):243-48. https://www.redalyc.org/articulo.oa?id=10638404

For peer review only - http://bmjopen.bmj.com/site/about/guidelines.xhtml

46. Carnell MA, Dougherty L, Pomeroy AM, Karim AM, Mekonnen YM, Mulligan BE. Effectiveness of scaling up the 'Three Pillars' approach to accelerating MDG 4 progress in Ethiopia. Journal of health, population, and nutrition. 2014;32(4):549.
47. Costa-Font J, Parmar D. Political agency and public healthcare: Evidence from India. WIDER Working Paper; 2016;number 6640. Munich: UNU-WIDER. https://doi.org/10.35188/UNU-WIDER/2016/179-6.
48. Demilew A, Girma M, McElwee E, Datta S, Barofsky J, Disasa T. Improving Immunisation through Supportive Feedback and Nonmonetary Incentives: Impact Evaluation in Ethiopia. Impact Evaluation Report. New Delhi: International Initiative for Impact Evaluation (3ie) 2020.
49. Dipeolu IO. Effect of Mobile-Phone Reminder Test Messages on Mothers' Knowledge and Completion of Routine Immunisation in Rural Areas of Oyo State, Nigeria. PhD Thesis, Ibadan, Nigeria: University of Ibadan 2017. https://library.adhl.africa/handle/123456789/11865.
50. Domek GJ, Contreras-Roldan IL, Bull S, et al. Text message reminders to improve infant immunization in Guatemala: A randomized clinical trial. Vaccine. 2019;37(42):6192-200. doi: 10.1016/j.vaccine.2019.08.046.
51. Engineer CY, Dale E, Agarwal A, et al. Effectiveness of a pay-for-performance intervention to improve maternal and child health services in Afghanistan: a cluster-randomized trial. International journal of epidemiology. 2016;45(2):451-9. doi: 10.1093/ije/dyv362.
52. Findley SE, Uwemedimo OT, Doctor HV, Green C, Adamu F, Afenyadu GY. Early results of an integrated maternal, newborn, and child health program, Northern Nigeria, 2009 to 2011. BMC Public Health. 2013;13(1):1-2. doi: 10.1186/1471-2458-13-1034
53. Gibson DG, Ochieng B, Kagucia EW, et al. Mobile phone-delivered reminders and incentives to improve childhood immunisation coverage and timeliness in Kenya (M-SIMU): a cluster randomised controlled trial. The Lancet Global Health. 2017;5(4):e428-38. doi: 10.1016/S2214-109X(17)30072-4.
54. Goel S, Dogra V, Gupta SK, et al. Effectiveness of Muskaan Ek Abhiyan (the smile campaign) for strengthening routine immunization in Bihar, India. Indian pediatrics. 2012;49(2):103-8. doi: 10.1007/s13312-012-0023-7.

For peer review only - http://bmjopen.bmj.com/site/about/guidelines.xhtml

55. Gurley N, Shearer J, Srivastava Y, Mahapatra S, Desmond M. Impacts of community-led video education to increase vaccination coverage in Uttar Pradesh, India: A mixed methods randomised controlled trial. Impact Evaluation Report 125. 2020th ed. New Delhi: International Initiative for Impact Evaluation (3ie). https://doi.org/10.23846/TW10IE125.

56. Herrera-Almanza C, Rosales-Rueda MF. Reducing the Cost of Remoteness: Community-Based Health Interventions and Fertility Choices. Journal of Health Economics. 2020;73:102365. doi: 10.1016/j.jhealeco.2020.102365.

57. Igarashi K, Sasaki S, Fujino Y, et al. The impact of an immunization programme administered through the Growth Monitoring Programme Plus as an alternative way of implementing Integrated Management of Childhood Illnesses in urban-slum areas of Lusaka, Zambia. Transactions of the Royal Society of Tropical Medicine and Hygiene. 2010;104(9):577-82. doi:10.1016/j.trstmh.2010.05.008

58. Janssens W. Externalities in program evaluation: the impact of a women's empowerment program on immunization. Journal of the European Economic Association. 2011;9(6):1082-113. doi: 10.1111/j.1542-4774.2011.01041.x.

59. Johri M, Chandra D, Kone KG, et al. Social and Behavior Change Communication Interventions Delivered Face-to-Face and by a Mobile Phone to Strengthen Vaccination Uptake and Improve Child Health in Rural India: Randomized Pilot Study. JMIR mHealth and uHealth. 2020;8(9):e20356. doi: 10.2196/20356.

60. Lee SS. Three field experiments on incentives for health workers. PhD Thesis, Cambridge: Harvard University; 2015. https://dash.harvard.edu/handle/1/17467500.

61. Mayumana I, Borghi J, Anselmi L, Mamdani M, Lange S. Effects of Payment for Performance on accountability mechanisms: Evidence from Pwani, Tanzania. Social Science & Medicine. 2017;179:61-73. doi: 10.1016/j.socscimed.2017.02.022.

62. Memon ZA, Khan GN, Soofi SB, Baig IY, Bhutta ZA. Impact of a community-based perinatal and newborn preventive care package on perinatal and neonatal mortality in a remote mountainous district in Northern Pakistan. BMC pregnancy and childbirth. 2015;15(1):1-9. doi: 10.1186/s12884-015-0538-8.

63. Modi D, Dholakia N, Gopalan R, et al. mHealth intervention “ImTeCHO” to improve delivery of maternal, neonatal, and child care services—A cluster-randomized trial in tribal areas of Gujarat, India. PLoS medicine. 2019;16(10):e1002939. doi: 10.1371/journal.pmed.1002939.

For peer review only - http://bmjopen.bmj.com/site/about/guidelines.xhtml

64. Mohanan M, Rajan VS, Swanson K, Thirumurthy H. Information and Facilitation Interventions for Accountability in Health and Nutrition: Evidence from a Randomized Trial in India. Economic Research Initiatives at Duke (ERID) Working Paper. Durham: Duke University; 2020(295). https://www.ssrn.com/abstract=3544786.

65. More NS, Bapat U, Das S, et al. Community mobilization in Mumbai slums to improve perinatal care and outcomes: a cluster randomized controlled trial. PLoS medicine. 2012;9(7):e1001257. doi: 10.1371/journal.pmed.1001257.

66. More NS, Das S, Bapat U, et al. Community resource centres to improve the health of women and children in informal settlements in Mumbai: a cluster-randomised, controlled trial. The Lancet Global Health. 2017;5(3):e335-49. doi: 10.1016/S2214-109X(16)30363-1.

67. Morris SS, Flores R, Olinto P, Medina JM. Monetary incentives in primary health care and effects on use and coverage of preventive health care interventions in rural Honduras: cluster randomised trial. The Lancet. 2004;364(9450):2030-7 doi: 10.1016/S0140-6736(04)17515-6.

68. Murthy N, Chandrasekharan S, Prakash MP, et al. The Impact of an mHealth voice message service (mMitra) on infant care knowledge, and practices among low-income women in India: findings from a pseudo-randomized controlled trial. Maternal and child health journal. 2019;23(12):1658-69. doi: 10.1007/s10995-019-02805-5.

69. Nagar R, Venkat P, Stone LD, Engel KA, Sadda P, Shahnawaz M. A cluster randomized trial to determine the effectiveness of a novel, digital pendant and voice reminder platform on increasing infant immunization adherence in rural Udaipur, India. Vaccine. 2018;36(44):6567-77. doi: 10.1016/j.vaccine.2017.11.023.

70. Nagar R, Ambiya MS, Singh P, et al. A Mixed-Methods Evaluation to Determine the Effects of a Novel MHealth Platform for Maternal Child Health Tracking in Rural Udaipur, India." 3ie Series Report 129. New Delhi: International Initiative for Impact Evaluation (3ie); 2020. https://developmentevidence.3ieimpact.org/search-result-details/impact-evaluation-repository/a-mixed-methods-evaluation-to-determine-the-effects-of-a-novel-mhealth-platform-for-maternal-child-health-tracking-in-rural-udaipur-india/8984.

71. Nzioki JM, Ouma J, Ombaka JH, Onyango RO. Community health worker interventions are key to optimal infant immunization coverage, evidence from a pretest-posttest experiment in Mwingi, Kenya. Pan African Medical Journal. 2017;28(1). doi: 10.11604/pamj.2017.28.21.11255.

For peer review only - http://bmjopen.bmj.com/site/about/guidelines.xhtml

72. Oche MO, Umar AS, Ibrahim MT, Sabitu K. An assessment of the impact of health education on maternal knowledge and practice of childhood immunization in Kware, Sokoto State. Journal of Public Health and Epidemiology. 2011;3(10):440-7. https://academicjournals.org/article/article1379497483_Oche%20et%20al.pdf.

73. Okeke E, Glick P, Abubakar IS, et al. Better Obstetrics in Rural Nigeria: Evaluating the Midwives Service Scheme. 3ie Impact Evaluation Report 56. International Initiative for Impact Evaluation (3ie); 2017. https://www.3ieimpact.org/evidence-hub/publications/impact-evaluations/better-obstetrics-rural-nigeria-evaluating-midwives.

74. Okoli U, Morris L, Oshin A, Pate MA, Aigbe C, Muhammad A. Conditional Cash Transfer Schemes in Nigeria: Potential Gains for Maternal and Child Health Service Uptake in a National Pilot Programme. BMC Pregnancy and Childbirth 2014;14(1):408. doi: 10.1186/s12884-014-0408-9.

75. Olayo R, Wafula C, Aseyo E, Loum C, Kaseje D. A quasi-experimental assessment of the effectiveness of the Community Health Strategy on health outcomes in Kenya. BMC health services research. 2014;14(1):1-3. doi: 10.1186/1472-6963-14-S1-S3.

76. Olken BA, Onishi J, Wong S. Should aid reward performance? Evidence from a field experiment on health and education in Indonesia. American Economic Journal: Applied Economics. 2014;6(4):1-34. doi: 10.1257/app.6.4.1.

77. Oyo-Ita, A, Bosch-Capblanch X, Ross A, et al. Impacts of Engaging Communities through Traditional and Religious Leaders on Vaccination Coverage in Cross River State, Nigeria. Impact Evaluation Report 127. 2020th ed. New Delhi: International Initiative for Impact Evaluation (3ie); 2020. https://doi.org/10.23846/TW10IE127.

78. Pramanik S, Ghosh A, Goswami A, et al. Impacts of the Stimulate, Appreciate, Learn, and Transfer community engagement approach to increase immunisation coverage in Assam, India, 3ie Grantee Final Report. New Delhi: International Initiative for Impact Evaluation (3ie); 2020.

79. Rahman A, Malik A, Sikander S, Roberts C, Creed F. Cognitive behaviour therapy-based intervention by community health workers for mothers with depression and their infants in rural Pakistan: a cluster-randomised controlled trial. The Lancet. 2008;372(9642):902-9. doi: 10.1016/S0140-6736(08)61400-2.

For peer review only - http://bmjopen.bmj.com/site/about/guidelines.xhtml

80. Rahman M, Yunus FM, Shah R, et al. A controlled before-and-after perspective on the improving maternal, neonatal, and child survival program in rural Bangladesh: an impact analysis. PLoS One. 2016;11(9):e0161647. doi: 10.1371/journal.pone.0161647.
81. Rao T. The Impact of a Community Health Worker Program on Childhood Immunization: Evidence from India's 'ASHA' Workers. Available at SSRN 2444391. 2014. http://www.ssrn.com/abstract=2444391.
82. Robertson L, Mushati P, Eaton JW, et al. Effects of unconditional and conditional cash transfers on child health and development in Zimbabwe: a cluster-randomised trial. The Lancet. 2013;381(9874):1283-92. doi: 10.1016/S0140-6736(12)62168-0.
83. Roy SK, Bilkes F, Islam K, et al. Impact of pilot project of Rural Maintenance Programme (RMP) on destitute women: CARE, Bangladesh. Food and nutrition bulletin. 2008;29(1):67-75. doi: 10.1177/156482650802900108.
84. Saggurti N, Atmavilas Y, Porwal A, et al. Effect of health intervention integration within women's self-help groups on collectivization and healthy practices around reproductive, maternal, neonatal and child health in rural India. PLoS One. 2018;13(8):e0202562. doi: 10.1371/journal.pone.0202562.
85. Sankar D. Improving Early Childhood Development through Community Mobilization and Integrated Planning for Children. Results from the Evaluation of Bachpan Program, Ratlam District, Madhya Pradesh, India. Discussion Paper Series 59. Washington, D.C.: The World Bank; 2013. http://crossasia-repository.ub.uni-heidelberg.de/3467/.
86. Seth R, Akinboyo I, Chhabra A, et al. Mobile phone incentives for childhood immunizations in rural India. Pediatrics. 2018;141(4):e20173455. doi: 10.1542/peds.2017-3455.
87. Shukla M. Impact of a health governance intervention on provincial health system performance in Afghanistan: a quasi-experimental study. Health systems & reform. 2018;4(3):249-66. doi: 10.1080/23288604.2018.1477536.
88. Siddiqi DA, Ali RF, Munir M, Shah MT, Khan AJ, Chandir S. Effect of vaccine reminder and tracker bracelets on routine childhood immunization coverage and timeliness in urban Pakistan (2017-18): a randomized controlled trial. BMC public health. 2020;20(1):1086. doi: 10.1186/s12889-020-09088-4.

For peer review only - http://bmjopen.bmj.com/site/about/guidelines.xhtml

89. Tandon BN, Sahai A. Immunization in India: contribution of Integrated Child Development Services scheme to expanded programme of immunization. Journal of tropical pediatrics. 1988;34(6):309-12. doi: 10.1093/tropej/34.6.309.

90. USAID. Essential Services for Health in Ethiopia: Final Report. Washington, DC: USAID; 2008. https://publications.jsi.com/JSIInternet/Inc/Common/_download_pub.cfm?id=10111&lid=3.

91. Webster J, Landegger J, Bruce J, et al. Impacts of IRC's Fifth Child Community Engagement Strategy to Increase Immunisation in Northern Uganda. 3ie Grantee Final Report. New Delhi: International Initiative for Impact Evaluation (3ie); 2019. https://www.3ieimpact.org/sites/default/files/2019-02/gfr-TW10.1018-IRC-Imunization-Program-Uganda.pdf.

92. Younes L, Houweling TA, Azad K, et al. The effect of participatory women's groups on infant feeding and child health knowledge, behaviour and outcomes in rural Bangladesh: a controlled before-and-after study. J Epidemiol Community Health. 2015 Apr 1;69(4):374-81. doi: 10.1136/jech-2014-204271.

Supplementary material

Supplementary material includes

1. Appendix 1: Amendments to information provided in the protocol
2. Appendix 2: Overview of community engagement framework
3. Appendix 3: Full search terms for one database.
4. Appendix 4: List of excluded impact evaluation studies
5. Appendix 5: Qualitative and cost-evidence search strategy
6. Appendix 6: Data extraction tools
7. Appendix 7: Risk of bias assessment tools
8. Appendix 8: Risk of bias in the included studies
9. Appendix 9: Quantitative results – all community engagement interventions
10. Appendix 10: Quantitative results – Engagement as intervention
11. Appendix 11: Quantitative results - community engagement in intervention design
12. Appendix 12: Community engagement in implementation autonomy of interventions
13. Appendix 13: Multiple engagement types
14. Appendix 14: Qualitative synthesis of results

For peer review only - http://bmjopen.bmj.com/site/about/guidelines.xhtml

15. Appendix 15: Synthesis of cost evidence

Figure legends

Figure 1. PRISMA flow diagram

Figure 2. Forest plot showing the observed outcomes and the estimate of the random-effects model for the impact of community engagement interventions on full childhood immunisation. Note that # (number of) Participants is specific to each effect and thus may not reflect the sample size for the full study.

Figure 3. Forest plot showing the observed outcomes and the estimate of the random-effects model for the impact of community engagement interventions on DPT3 vaccination. Note that # Participants is specific to each effect and thus may not reflect the sample size for the full study.

Figure 4. Forest plot showing the observed outcomes and the estimate of the random-effects model for the impact of community engagement interventions on measles vaccination. Note that # Participants is specific to each effect and thus may not reflect the sample size for the full study.

For peer review only

For peer review only - http://bmjopen.bmj.com/site/about/guidelines.xhtml

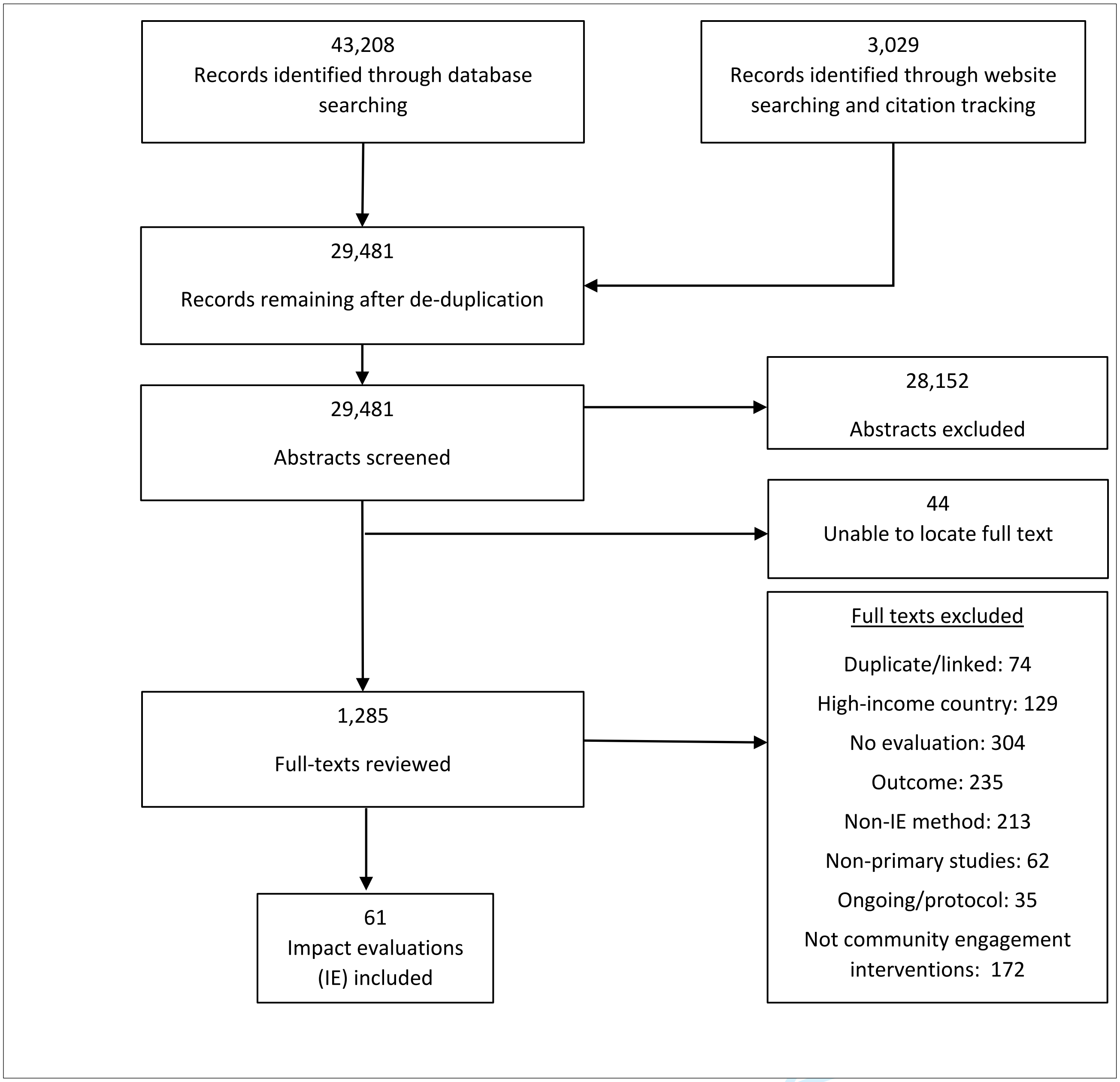
43,208
Records identified through database searching
3,029
Records identified through website searching and citation tracking
29,481
Records remaining after de-duplication
29,481
Abstracts screened
28,152
Abstracts excluded
44
Unable to locate full text
Full texts excluded
Duplicate/linked: 74
High-income country: 129
No evaluation: 304
Outcome: 235
Non-IE method: 213
Non-primary studies: 62
Ongoing/protocol: 35
Not community engagement interventions: 172
1,285
Full-texts reviewed
61
Impact evaluations (IE) included

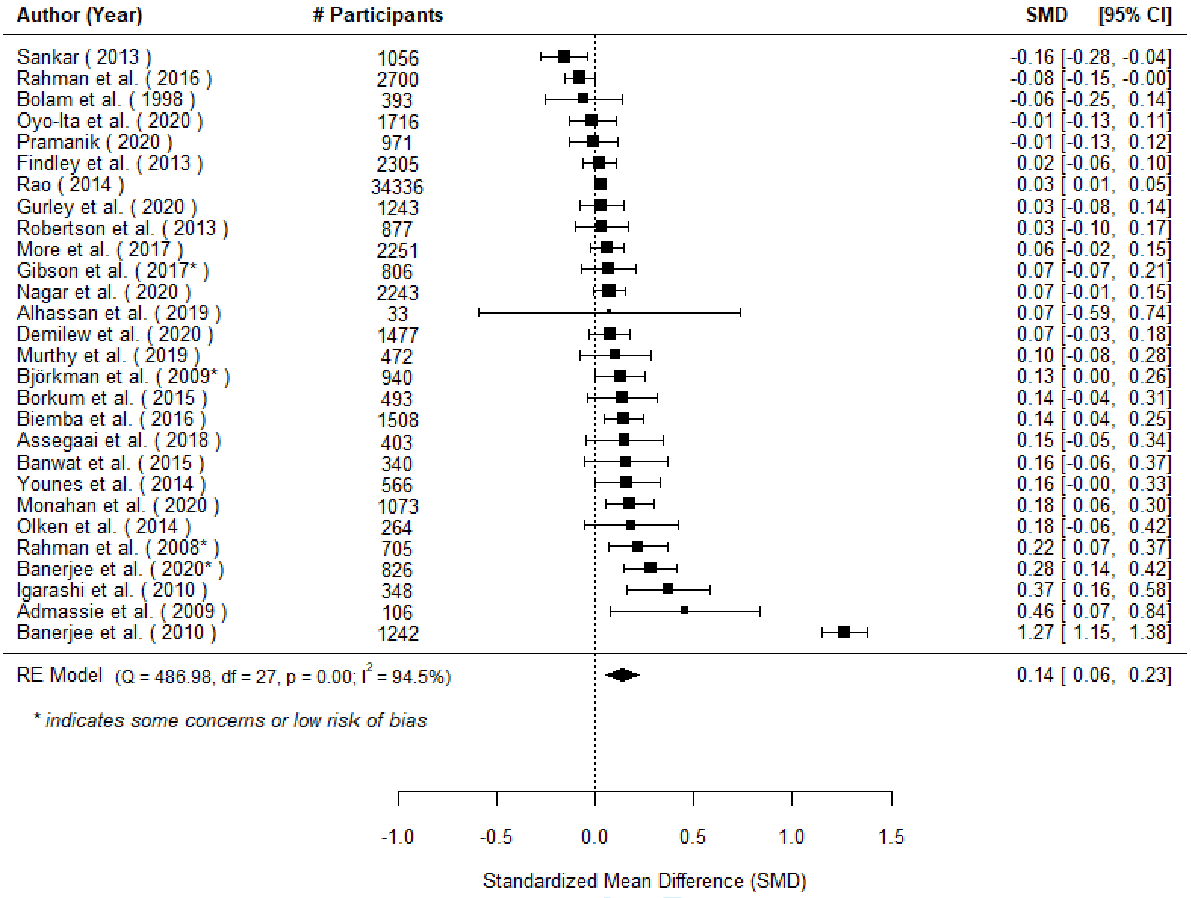
Author (Year) # Participants SMD [95% CI]
Sankar (2013) 1056 -0.16 [-0.28, -0.04]
Rahman et al. (2016) 2700 -0.08 [-0.15, -0.00]
Bolam et al. (1998) 393 -0.06 [-0.25, 0.14]
Oyo-Ita et al. (2020) 1716 -0.01 [-0.13, 0.11]
Pramanik (2020) 971 -0.01 [-0.13, 0.12]
Findley et al. (2013) 2305 0.02 [-0.06, 0.10]
Rao (2014) 34336 0.03 [0.01, 0.05]
Gurley et al. (2020) 1243 0.03 [-0.08, 0.14]
Robertson et al. (2013) 877 0.03 [-0.10, 0.17]
More et al. (2017) 2251 0.06 [-0.02, 0.15]
Gibson et al. (2017*) 806 0.07 [-0.07, 0.21]
Nagar et al. (2020) 2243 0.07 [-0.01, 0.15]
Alhassan et al. (2019) 33 0.07 [-0.59, 0.74]
Demilew et al. (2020) 1477 0.07 [-0.03, 0.18]
Murthy et al. (2019) 472 0.10 [-0.08, 0.28]
Björkman et al. (2009*) 940 0.13 [0.00, 0.26]
Borkum et al. (2015) 493 0.14 [-0.04, 0.31]
Biemba et al. (2016) 1508 0.14 [0.04, 0.25]
Assegaai et al. (2018) 403 0.15 [-0.05, 0.34]
Banwat et al. (2015) 340 0.16 [-0.06, 0.37]
Younes et al. (2014) 566 0.16 [-0.00, 0.33]
Monahan et al. (2020) 1073 0.18 [0.06, 0.30]
Olken et al. (2014) 264 0.18 [-0.06, 0.42]
Rahman et al. (2008*) 705 0.22 [0.07, 0.37]
Banerjee et al. (2020*) 826 0.28 [0.14, 0.42]
Igarashi et al. (2010) 348 0.37 [0.16, 0.58]
Admassie et al. (2009) 106 0.46 [0.07, 0.84]
Banerjee et al. (2010) 1242 1.27 [1.15, 1.38]
RE Model (Q = 486.98, df = 27, p = 0.00; I2 = 94.5%) 0.14 [0.06, 0.23]
* indicates some concerns or low risk of bias
-1.0 -0.5 0.0 0.5 1.0 1.5
Standardized Mean Difference (SMD)

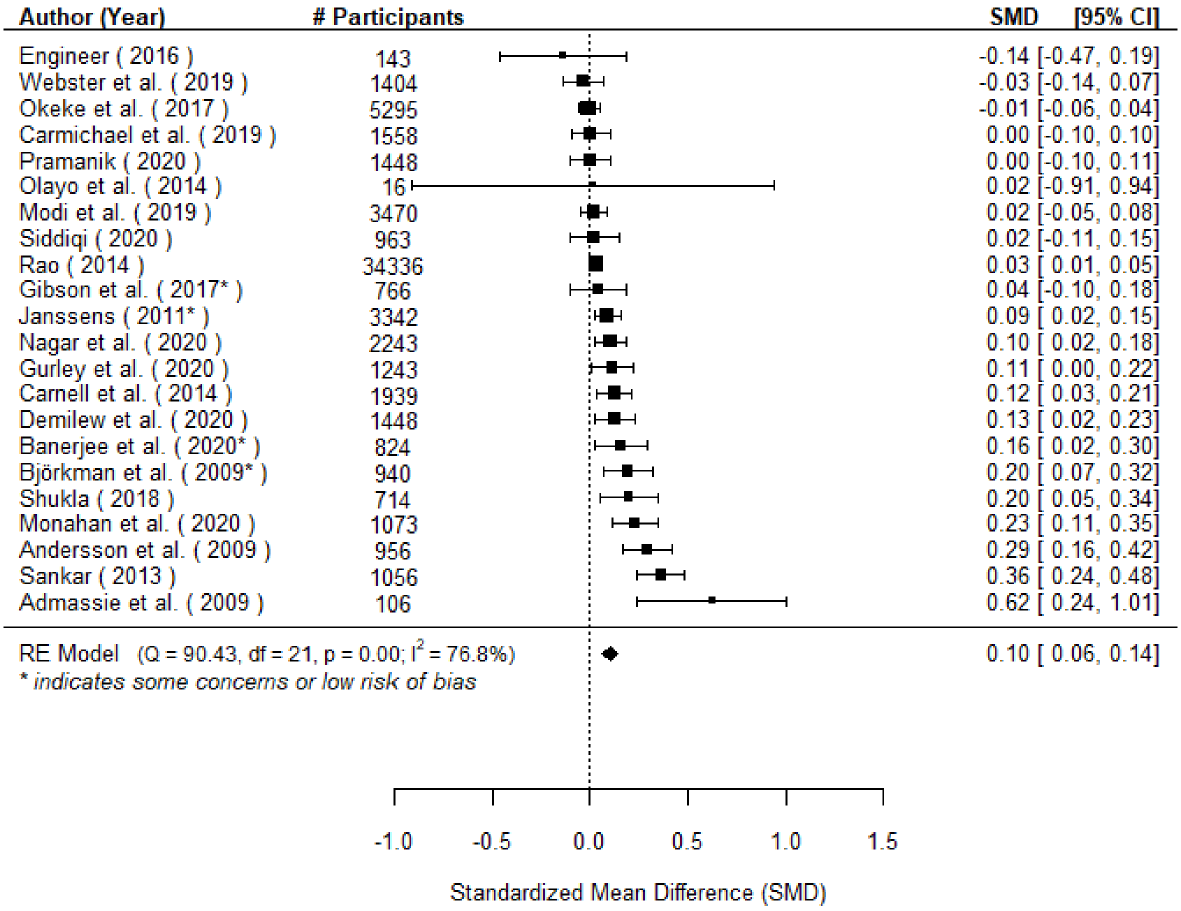
Author (Year) # Participants SMD [95% CI]
Engineer (2016) 143 -0.14 [-0.47, 0.19]
Webster et al. (2019) 1404 -0.03 [-0.14, 0.07]
Okeke et al. (2017) 5295 -0.01 [-0.06, 0.04]
Carmichael et al. (2019) 1558 0.00 [-0.10, 0.10]
Pramanik (2020) 1448 0.00 [-0.10, 0.11]
Olayo et al. (2014) 16 0.02 [-0.91, 0.94]
Modi et al. (2019) 3470 0.02 [-0.05, 0.08]
Siddiqi (2020) 963 0.02 [-0.11, 0.15]
Rao (2014) 34336 0.03 [0.01, 0.05]
Gibson et al. (2017*) 766 0.04 [-0.10, 0.18]
Janssens (2011*) 3342 0.09 [0.02, 0.15]
Nagar et al. (2020) 2243 0.10 [0.02, 0.18]
Gurley et al. (2020) 1243 0.11 [0.00, 0.22]
Carnell et al. (2014) 1939 0.12 [0.03, 0.21]
Demilew et al. (2020) 1448 0.13 [0.02, 0.23]
Banerjee et al. (2020*) 824 0.16 [0.02, 0.30]
Björkman et al. (2009*) 940 0.20 [0.07, 0.32]
Shukla (2018) 714 0.20 [0.05, 0.34]
Monahan et al. (2020) 1073 0.23 [0.11, 0.35]
Andersson et al. (2009) 956 0.29 [0.16, 0.42]
Sankar (2013) 1056 0.36 [0.24, 0.48]
Admassie et al. (2009) 106 0.62 [0.24, 1.01]
RE Model (Q = 90.43, df = 21, p = 0.00; I2 = 76.8%) 0.10 [0.06, 0.14]
* indicates some concerns or low risk of bias
-1.0 -0.5 0.0 0.5 1.0 1.5
Standardized Mean Difference (SMD)

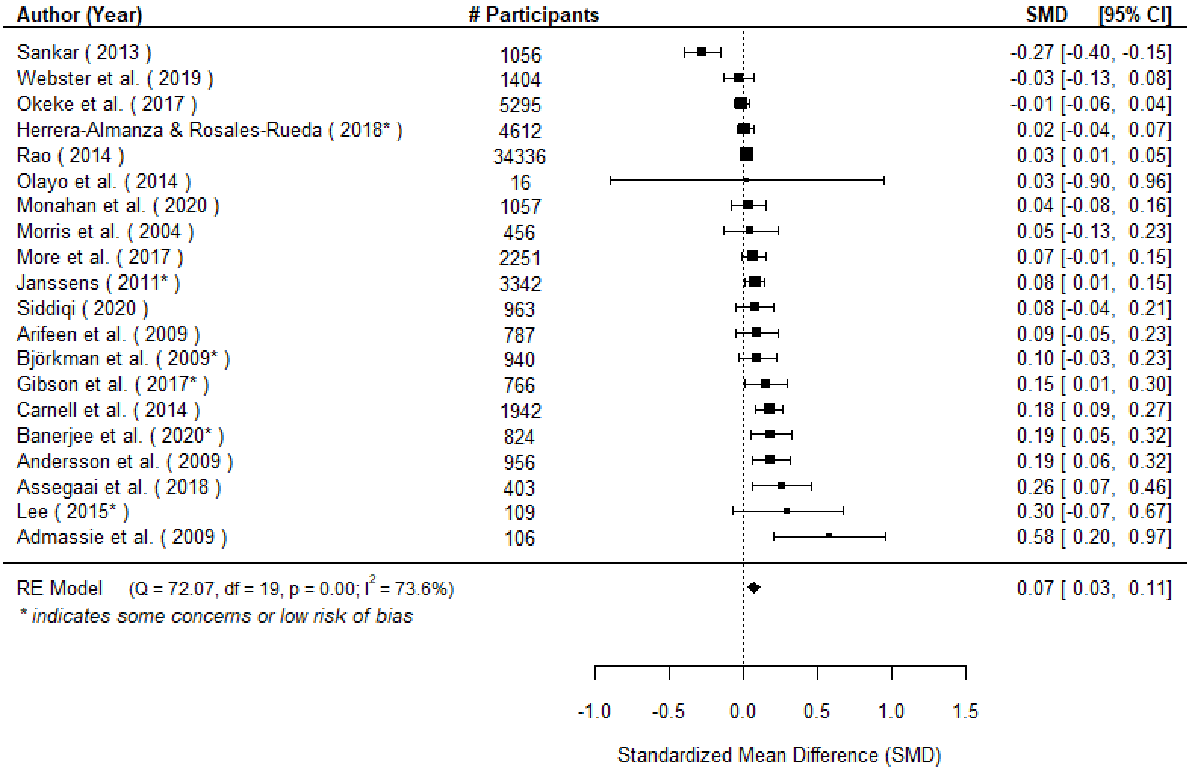
Author (Year) # Participants SMD [95% CI]
Sankar (2013) 1056 -0.27 [-0.40, -0.15]
Webster et al. (2019) 1404 -0.03 [-0.13, 0.08]
Okeke et al. (2017) 5295 -0.01 [-0.06, 0.04]
Herrera-Almanza & Rosales-Rueda (2018*) 4612 0.02 [-0.04, 0.07]
Rao (2014) 34336 0.03 [0.01, 0.05]
Olayo et al. (2014) 16 0.03 [-0.90, 0.96]
Monahan et al. (2020) 1057 0.04 [-0.08, 0.16]
Morris et al. (2004) 456 0.05 [-0.13, 0.23]
More et al. (2017) 2251 0.07 [-0.01, 0.15]
Janssens (2011*) 3342 0.08 [0.01, 0.15]
Siddiqi (2020) 963 0.08 [-0.04, 0.21]
Arifeen et al. (2009) 787 0.09 [-0.05, 0.23]
Björkman et al. (2009*) 940 0.10 [-0.03, 0.23]
Gibson et al. (2017*) 766 0.15 [0.01, 0.30]
Carnell et al. (2014) 1942 0.18 [0.09, 0.27]
Banerjee et al. (2020*) 824 0.19 [0.05, 0.32]
Andersson et al. (2009) 956 0.19 [0.06, 0.32]
Assegaai et al. (2018) 403 0.26 [0.07, 0.46]
Lee (2015*) 109 0.30 [-0.07, 0.67]
Admassie et al. (2009) 106 0.58 [0.20, 0.97]
RE Model (Q = 72.07, df = 19, p = 0.00; I2 = 73.6%) 0.07 [0.03, 0.11]
* indicates some concerns or low risk of bias
-1.0 -0.5 0.0 0.5 1.0 1.5
Standardized Mean Difference (SMD)

review only

Use of community engagement interventions to improve child immunisation in low- and middle-income countries: a systematic review and meta-analysis

Supplementary material

Table of Contents

APPENDIX 1: AMENDMENTS TO INFORMATION PROVIDED IN THE PROTOCOL 3

APPENDIX 2: OVERVIEW OF COMMUNITY ENGAGEMENT FRAMEWORK 5

APPENDIX 3: SEARCH STRATEGY AND SEARCH TERMS FOR IMPACT EVALUATIONS 11

APPENDIX 4: LIST OF EXCLUDED IMPACT EVALUATION STUDIES 16

APPENDIX 5: QUALITATIVE AND COST-EVIDENCE SEARCH STRATEGY 28

Qualitative search protocol 28

Cost evidence search protocol 30

List of included qualitative studies 30

List of included other studies 33

APPENDIX 6: DATA EXTRACTION TOOLS 37

Quantitative data extraction tool 37

Qualitative data extraction tool 42

Cost data extraction tool 57

APPENDIX 7: RISK OF BIAS ASSESSMENT TOOLS 65

Quantitative risk of bias tool 65

Qualitative risk of bias tool 81

Cost evidence risk of bias tool 89

APPENDIX 8: RISK OF BIAS IN THE INCLUDED STUDIES 90

Quantitative risk of bias 90

For peer review only - http://bmjopen.bmj.com/site/about/guidelines.xhtml

Qualitative risk of bias 93

Risk of bias in cost effectiveness estimates 94

APPENDIX 9: QUANTITATIVE RESULTS – ALL COMMUNITY ENGAGEMENT INTERVENTIONS 98

APPENDIX 10: QUANTITATIVE RESULTS – ENGAGEMENT AS INTERVENTION 131

APPENDIX 11: QUANTITATIVE RESULTS - COMMUNITY ENGAGEMENT IN INTERVENTION DESIGN 144

APPENDIX 12: COMMUNITY ENGAGEMENT IN IMPLEMENTATION AUTONOMY OF INTERVENTIONS 158

APPENDIX 13: MULTIPLE ENGAGEMENT TYPES 166

APPENDIX 14: QUALITATIVE SYNTHESIS OF RESULTS 181

Barriers to immunisation 181

Facilitators of immunisation 191

Reasons for intervention success 197

Reasons for intervention failure 207

Reasons for heterogenous impacts 217

Uptake and fidelity challenges 217

APPENDIX 15: SYNTHESIS OF COST EVIDENCE 230

For peer review only - http://bmjopen.bmj.com/site/about/guidelines.xhtml

Appendix 1: Amendments to information provided in the protocol

1. In the report, we have replaced the word 'participation' for 'engagement', a change that is also reflected in our review title and objectives. Though the IAP2 framework uses the terms "public participation" and "community engagement" interchangeably, we realise that this may lead to confusion as there may be inter researcher differences in how these terms are perceived and used. Therefore, for maintaining consistency, we have used community engagement throughout the report and avoided any references to community participation. From a linguistic perspective, these words are used interchangeability in our review, as is evident from our protocol (please refer to pp. 1-3). Therefore, this change does not alter the scope of our review in any way. In the protocol we had proposed to use the IAP2 spectrum of community engagement for determination of interventions to be included in the review. However, after pilot testing of the IAP2 framework on a range of community engagement interventions, we found that most interventions are based on a more "utilitarian perspective" put forth in Brunton et al. (2017). Thus, for this review we ultimately settled on the community engagement framework which focuses on process of engagement rather than its intensity. Our approach corresponds to some degree to the "extent of engagement" part of the conceptual framework developed in Brunton et al. (2017) and we also kept the spirit of IAP2 framework by including interventions in which engagement goes beyond one-way communication, i.e. beyond the inform level of IAP2, to include some consultation or dialogue with the community or some decision making by the community members. We found this approach to be relatively easier to apply, less subjective, less prone to classification error and potentially useful to practitioners
2. In the section on objectives, we have clubbed what were originally review questions 3 and 4 in the protocol (p. 5). These questions related to understanding how implementation features and contextual factors such as barriers and facilitators to immunisation, are associated with relative success or failure of community engagement interventions. When we set out to answer these questions through our qualitative synthesis, we realised that all these factors are interlinked and, in various combinations, may influence the outcomes along the causal chain. Therefore, we combined the two objectives and have presented our analysis and results accordingly. This modification to the review objectives does not alter our study's scope.
3. We had intended to include the relevant Chinese literature in the review. However, because of political disruptions in Hong Kong, it did not materialize.
4. We have added many more secondary outcomes as per our evidence gap map framework and also their categorization has changed. But data was not available in primary studies for most of them, so they could not be analysed in this review.
5. In the protocol we stated that 'When multiple papers report different results on an identical outcome, we will contact the authors to enquire about the differences and choose the results that more accurately reflect the impact of the intervention as relevant to our research questions. If contacting the author does not yield a clear decision, we will use results from the paper with the latest publication date.' However, due to the large scope and limited resources of our review, we instead used results from the most recent paper.
6. In the protocol, we stated that 'Where multiple outcomes are reported from different specifications, we will select the specification with the lowest risk of bias in attributing impact, for example, the most appropriately specified outcomes equation.' However, given that risk of bias could not be assessed until after inclusion decisions were made, we instead used the authors preferred specification. If this was not explicitly stated, but the authors reported one specification as the "main results" and other specifications as "robustness checks" we used the "main results" specification. If neither of these applied, we used the specification with the most controls.
7. In the protocol we stated that 'When multiple treatment arms, we will divide the control group by the number of treatment arms.' Instead, we chose independent effects, choosing either a) the treatment arm

For peer review only - http://bmjopen.bmj.com/site/about/guidelines.xhtml

with the most components, b) the treatment arm that was identified by the authors as the one that would be scaled up, or c) when number of components was equal, the arm that was the most cost effective (e.g. choosing unconditional cash transfers (UCTs) over conditional cash transfers as the administrative costs related to UCTs are significantly lower)

For peer review only

For peer review only - http://bmjopen.bmj.com/site/about/guidelines.xhtml

Appendix 2: Overview of community engagement framework

The focus of our review is community engagement interventions, which are increasingly being emphasised in international and national policy frameworks as a means to improve immunisation coverage and reach marginalised communities (UNICEF 2018, WHO 2015, WHO 2017b; WHO 2017a).

The most common approach for categorizing community engagement interventions is probably the International Association for Public Participation (IAP2) framework (iap2.org), which identifies five levels of engagement ranging from inform to empower corresponding to increasing community influence over the decisions. This framework corresponds to the "social justice perspective" in Brunton et al. (2017) in which the community engagement is rooted in concerns about social justice, which requires that the health needs are identified by communities themselves and they mobilise themselves into action to make changes within the community. However, after pilot testing of the IAP2 framework on a range of community engagement interventions, we determined that most interventions are based on a more "utilitarian perspective." These two perspectives of community engagement are very well captured and articulated in Brunton et al. (2017), who in their systematic review of community engagement narratives in public health point out that:

"Historically, interventions to promote health were driven by professionals, with little or no input from the targeted populations; more recently, community engagement has become central to national strategy and guidance for promoting public health, because, from a 'utilitarian' point of view, it is thought that more acceptable and appropriate interventions will result, which may result in improved service use and outcomes. Interventions that are based on a utilitarian perspective seek to involve communities in order to improve the effectiveness of the intervention. The intervention itself may be decided upon before the community is invited for its views; or, while the intervention itself is not designed by community members they may be involved in other ways, such as priority setting, or in its delivery. In utilitarian perspectives, health (and other) services reach out to engage particular communities that they have identified require assistance and the intervention is devised within existing policy, practice, and resource frameworks."

Due to the difference in how the IAP2 framework approaches community engagement and how interventions are actually implemented, it was difficult to systematically categorize interventions using the IAP2 framework with a high degree of consistency among coders. In addition, even for the interventions rooted in "social justice" perspective, their description in the studies was too limited to identify them and map their intensity of community engagement without introducing a lot of subjectivity and non-systematicness.

To avoid this misclassification as far as possible, we experimented with different frameworks and ultimately settled on the one which focuses on process of engagement rather than its intensity. We found that focusing on when and how the community is engaged is a more practical framework for the kind of community engagement interventions that have been evaluated in real world settings. Our approach corresponds to some degree to the "extent of engagement" part of the conceptual framework developed in Brunton et al. (2017) and we also kept the spirit of IAP2 framework by including interventions in which engagement goes beyond one-way communication, i.e. beyond the inform level of IAP2, to include some consultation or dialogue with the community or some decision making by the community members. We found this approach to be relatively easier to apply, less subjective, less prone to classification error and potentially useful to practitioners. The development of this process-oriented framework took a period of around one month and involved three of our core team members

We consider three points within an intervention during which engagement can occur: engagement can occur in the design of the intervention, engagement can occur in the implementation of the intervention, or the intervention may be engagement. We break these categories into further sub-groups representing specific ways through which

For peer review only - http://bmjopen.bmj.com/site/about/guidelines.xhtml

engagement can occur; for example, the development of new cadres of health workers, pilot studies, and the involvement of the community in governance and decision making.

This framework adds a new dimension to the discussion of types of engagement. As practitioners consider the design of their programs, they can use the evidence provided through this framework to determine when and how best to engage with the community.

Engagement as the intervention (engagement is embedded): In these interventions a serious attempt was made to gain community buy-in for activities or new cadres of community-based structures were established, such as village health committees or community health volunteers. While, in a few cases, the development of new cadres can be solely for the purpose of didactic teaching and one-way communication, it generally results in dynamic discussion and two-way communication. As such, we chose to include these interventions. Interventions which are themselves community engagement can motivate communities to take ownership of service delivery and address local problems with local solutions.

Engagement in the design of interventions: In these interventions community input or feedback was sought before the implementation of an intervention. Such feedback can take the form of a pilot, needs assessment, formative evaluation, or other outreach effort. This form of engagement must occur before the implementer undertakes an action. These interventions allow the community to influence the form of the ultimate action taken. Depending on the weight that is given to the feedback, these engagement activities can align to any of the IAP2 categories other than inform, which was not considered.

Engagement in implementation autonomy of interventions: In many interventions the community is not asked for input in their design, but is utilized in their implementation as health care workers, facilitators, or problem solvers. In the spirit of IAP2 framework to go beyond inform level interventions, we only included those interventions in this category where the community members involved in the implementation of the intervention had some opportunity to affect or influence its implementation. This broadly aligns with the involve, collaborate and empower levels of engagement. Due to the inclusion criteria of some autonomy in implementation, the interventions under this category generally involved an existing community led governance structure which weighed in on implementation decisions or the community providing resources without which the intervention could not be implemented. We excluded interventions in which community members, like community health workers or frontline health workers, were involved in implementation, but no new cadres of health workers were created and they could not influence its implementation. For example, community health workers supplying hygiene kits or doing home visits. In addition, interventions which built capacity of existing cadres of community members or provided supportive supervision were excluded. For example, m-health apps for community workers and training of peer facilitators or community health workers or frontline health workers that only allowed for a one-way transfer of knowledge.

For peer review only - http://bmjopen.bmj.com/site/about/guidelines.xhtml

Examples of engagement types

ENGAGEMENT AS THE INTERVENTION (ENGAGEMENT IS EMBEDDED)

Effect of health intervention integration within women's self-help groups on collectivization and healthy practices around reproductive, maternal, neonatal and child health in rural India

Health-focused self-help groups were created for women of reproductive age in marginalized communities.

Effect of peer education on knowledge, attitude and completeness of childhood routine immunization in a rural community of Plateau State.

A new cadre of peer educators was created. Women were trained to provide women with information about routine childhood immunisation. This does not qualify as engagement in implementation because the intervention was the training of peer educators and not the subsequent actions of the peer educators.

Impacts of engaging communities through traditional and religious leaders on vaccination coverage in Cross River State, Nigeria

Traditional and religious leaders were trained to utilize their leadership role to support immunisation. After the training, these leaders then presented data at ward development committee meetings and engaged with the community to encourage immunsation.

ENGAGEMENT IN THE DESIGN OF AN INTERVENTION

Cognitive behaviour therapy-based intervention by community health workers for mothers with depression and their infants in rural Pakistan: a cluster-randomised controlled trial

Many pilots directly seek the feedback of community members. In this study, health workers and depressed mothers were asked about the relevance and usefulness of the intervention before scaling.

Mobile Phone Incentives for Childhood Immunizations in Rural India

Caregivers were given mobile phone credit incentives for completing imminizations. The amount of the incentive was decided upon through conversations that involved community members.

ENGAGEMENT IN THE IMPLEMENTATION AUTONOMY OF AN INTERVENTION

The impact of an immunization programme administered through the Growth Monitoring Programme Plus as an alternative way of implementing Integrated Management of Childhood Illnesses in urban-slum areas of Lusaka, Zambia

The GMP+ sessions were conducted by medical personnel from Public Health Centers. During these session, community volunteers provided some operational and managerial support to ensure the effective implementation of the sessions.

Effects of payment for performance on accountability mechanisms: Evidence from Pwani, Tanzania

The intervention is a performance-based financing mechanism. The community health committee was involved in decisions about how to spend the funds gained through the pay for performance mechanism.

Interventions are defined based on the actions of the external actors, ie implementers, not the community itself. If an intervention spurred the community to take action, the categorization of the intervention is based on the intervention which spurred the action and not the actions that the community took as a result of the intervention. This is because another community might take different actions as a result of the same intervention. The distinction is especially important for interventions to empower communities to improve their own systems. In these cases, the intervention is the activity that empowered the community; this intervention is engagement. For example, interventions which develop village committees to identify local challenges and solutions are themselves engagement interventions. This is because the intervention is the development of the committee and not the actions of the committee. A similar committee in a different area may choose to take different actions, even if the implementing agency does the same development process. Although the community may have engaged in the design of the committee, they likely were not engaged in the design of development process which brought about the committee. For interventions to be qualified as engagement in the design or implementation, the intervention must be an action by an implementer which the community influenced. For example, community members could be consulted in the design of materials used in

For peer review only - http://bmjopen.bmj.com/site/about/guidelines.xhtml

outreach activities (Murthy 2019, Nagar 2020). In the example of the development of a new committee, the implementer influenced the action of the community, the community did not influence the action of the implementer.

We defined “communities” in reference to the lowest level of the health service delivery system (or whatever level provides routine immunisation services in the local context). A community is a group of people who are served by a particular primary health facility. Thus, communities encompass a wide range of stakeholders, including caregivers, health service providers, and influential community members such as religious or other traditional leaders. Therefore, our review included any intervention that was directed towards any of these types of community members. Interventions that targeted higher levels of the health system, such as state-level officials, were excluded.

How the interventions might work

A 2015 3ie scoping paper (Sabarwal et al. 2015) systematically mapped the literature on immunisation interventions involving community engagement. Several programme managers and policy experts provided insight regarding why community engagement could be the key to improving immunisation outcomes for children in areas where the coverage has stagnated or declined or that are hard to reach. The findings from the scoping study indicate that working with or engaging communities could help develop a better understanding of the context, target population, problems and barriers, and lead to identification of contextually relevant solutions and desired outcomes, and mobilising community support for them. Because individuals usually function under the influence of social norms, efforts to change these norms can be effective in changing behaviour (Bicchieri and Xiao 2009; Reynolds Subašić and Tindall 2015). People respond to their peers and community and, while activities such as information and education campaigns might have some influence, individuals might feel bound by collective decisions, preventing sustained change (Riedy et al. 2012). The role of peers and of social norms in shaping attitudes towards vaccination is particularly important given that vaccine hesitancy has been documented in countries of all income levels (although it takes different forms in different countries; Dubé, Gagnon, Nickels, Jeram, and Schuster 2014). Community engagement may be effective at overcoming these barriers to immunisation. In both high-income countries (HICs; O’Mara-Eves et al. 2013) and L&MICs (De Buck et al. 2017), community engagement has been an effective model of modifying health behaviours in particular. Hence, community engagement could be an important determinant of success or failure of an intervention aimed at improving immunisation coverage.

Due to the variability in contexts, activities to address the barriers to immunisation through community engagement also vary. Therefore, no single theory of change can capture all the different ways that community engagement will affect immunisation outcomes. Furthermore, there is no strict correspondence between types of engagement and intervention activities. For the most part, each activity can be structured such that community engagement is the intervention (engagement is embedded) or the community is engaged in the design or implementation of the intervention. For example, consider an intervention involving village health committees in which community members are adequately represented. These committees can be created by a sponsoring agency with little or no input from the actual community. In this case, the intervention is engagement. On the other hand, community members may develop a village health committee during the design phase of major clinic infrastructure projects to ensure the efficient management of the intervention. Hence, these committees could arise from community involvement in the design of the intervention. In addition, an existing village health committee could be leveraged for the implementation of community meetings at which committee members lead a dialogue on immunisation.

Broadly, we expect that community engagement activities will increase awareness of vaccine-preventable diseases, knowledge of where and when to get vaccinations, and motivation to get vaccinations among caregivers (Figure 1). Community engagement may also increase skill, motivation, and accountability of health workers. These ought to lead to improved demand for and delivery of services, which will increase the number of vaccinated children and could potentially reduce child morbidity and mortality. It is important to note that child mortality and morbidity are affected by critical factors beyond immunisation, such as access to safe food and adequate nutrition, safe water and

For peer review only - http://bmjopen.bmj.com/site/about/guidelines.xhtml

quality care by a trained health provider when needed. Without these critical enabling factors immunisation alone may not be effective in reduction of child mortality and morbidity.

For peer review only

For peer review only - http://bmjopen.bmj.com/site/about/guidelines.xhtml

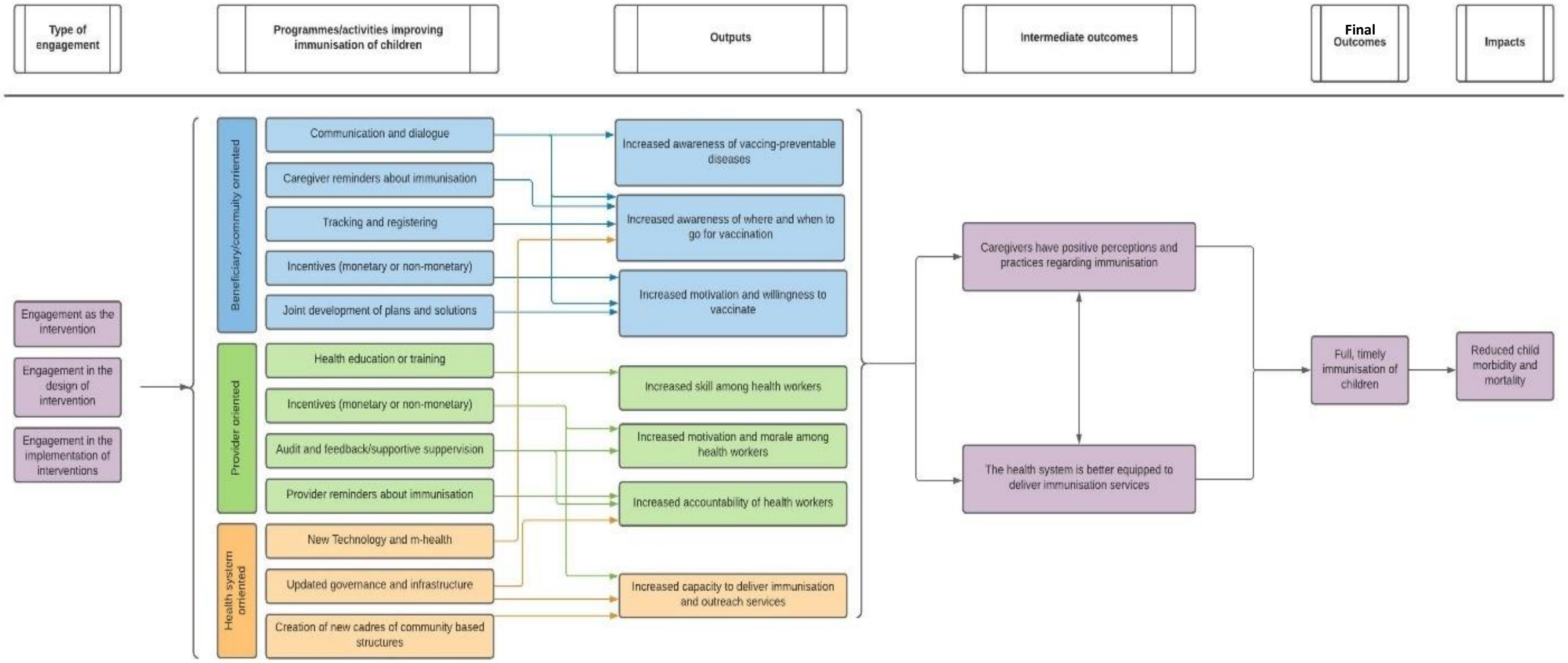

Supplementary Figure 1: Intervention theory of chan

Only

For peer review only - http://bmjopen.bmj.com/site/about/guidelines.xhtml

Appendix 3: Search strategy and search terms for impact evaluations

We searched the academic databases and websites listed below on 17 May 2019 and updated the search on 5 May, 2020. A full record of the applied search terms is provided in Appendix C.

1. MEDLINE
2. CAB Global Health
3. EMBASE
4. Cochrane Controlled Trials Register (CENTRAL)
5. CINAHL
6. PsycINFO
7. Popline
8. Africa-wide information
9. Academic search complete
10. Scopus
11. Campbell Library
12. Google Scholar
13. EconLit
14. IDEAS/RePEc
15. WHO Global Index Medicus
16. Pascal-Francis
17. Open-Grey
18. Grey Literature Report
19. Social Science Research Network (SSRN)
20. Eldis
21. GAVI
22. Epistemonikos
23. Innovations for Poverty Action (IPA)
24. Abdul Latif Jameel Poverty Action Lab (J-PAL)
25. 3ie Impact Evaluation Repository
26. 3ie Systematic Review Repository
27. Registry of International Development Impact Evaluations (RIDIE)
28. Global Development Network
29. World Bank Development Impact Evaluation (DIME) and Impact Evaluation Policy Papers
30. Inter-American Development Bank
31. Center for Global Development
32. Center for Effective Global Action (CEGA)
33. DFID Research for Development (R4D)
34. USAID

Website searches for capturing grey literature were carried out from January to May 2020

Search terms

L&MICs

2. (Afghanistan or Albania or Algeria or Angola or Argentina or Armenia or Armenian or Azerbaijan or Bangladesh or Benin or Byelarus or Byelorussian or Belarus or Belorussian or Belorussia or Belize or Bhutan or Bolivia or Bosnia or Herzegovina or Hercegovina or Botswana or Brazil or Bulgaria or Burkina Faso or Burkina Fasso or Upper Volta or Burundi or Urundi or Cambodia or Khmer Republic or Kampuchea or

Cameroon or Cameroons or Cameron or Camerons or Cape Verde or Cabo Verde or Central African Republic or Chad or Tchad or China or Colombia or Comoros or Comoro Islands or Comores or Mayotte or Congo or Zaire or Costa Rica or Cote d'Ivoire or Ivory Coast or Cuba or Djibouti or French Somaliland or Dominica or Dominican Republic or East Timor or East Timur or Timor Leste or Ecuador or Egypt or United Arab Republic or El Salvador or Eritrea or Ethiopia or Fiji or Gabon or Gabonese Republic or Gambia or Gaza or Georgia Republic or Georgian Republic or Ghana or Grenada or Guatemala or Guinea or Guiana or Guyana or Haiti or Honduras or India or Maldives or Indonesia or Iran or Iraq or Jamaica or Jordan or Kazakhstan or Kazakh or Kenya or Kiribati or Korea or Kosovo or Kyrgyzstan or Kirghizia or Kyrgyz Republic or Kirghiz or Kirgizstan or Lao PDR or Laos or Lebanon or Lesotho or Basutoland or Liberia or Libya or Macedonia or Madagascar or Malagasy Republic or Malaysia or Malaya or Malay or Sabah or Sarawak or Malawi or Mali or Marshall Islands or Mauritania or Mauritius or Agalega Islands or Mexico or Micronesia or Middle East or Moldova or Moldovia or Moldovian or Mongolia or Montenegro or Morocco or Ifni or Mozambique or Myanmar or Myanma or Burma or Namibia or Nepal or Netherlands Antilles or Nicaragua or Niger or Nigeria or Muscat or Pakistan or Palau or Palestine or Panama or Paraguay or Peru or Philippines or Philipines or Phillipines or Phillippines or Papua New Guinea or Romania or Rumania or Roumania or Rwanda or Ruanda or Saint Lucia or St Lucia or Saint Vincent or St Vincent or Grenadines or Samoa or Samoan Islands or Navigator Island or Navigator Islands or Sao Tome or Senegal or Serbia or Montenegro or Seychelles or Sierra Leone or Sri Lanka or Solomon Islands or Somalia or Sudan or Suriname or Surinam or Swaziland or Eswatini or South Africa or Syria or Tajikistan or Tadzhikistan or Tadjikistan or Tadzhik or Tanzania or Thailand or Togo or Togolese Republic or Tonga or Tunisia or Turkey or Turkmenistan or Turkmen or Uganda or Ukraine or Uzbekistan or Uzbek or Vanuatu or New Hebrides or Venezuela or Vietnam or Viet Nam or West Bank or Yemen or Zambia or Zimbabwe or Rhodesia)

3. (Africa or Asia or Caribbean or West Indies or South America or Latin America or Central America)
4. ((developing or less* developed or least developed or under developed or underdeveloped or middle income or low* income or underserved or under served or deprived or poor* or resource limited or resource constrained) adj (countr* or nation? or population? or world or state* or emerging econom* or global south))
5. ((developing or less* developed or least developed or under developed or underdeveloped or middle income or low* income or resource limited or resource constrained) adj (economy or economies))
6. (low* adj (gdp or gnp or gross domestic or gross national))
7. (low adj3 middle adj3 countr*)
8. (lmic or lmics or third world or lami countr*)
9. transitional countr*
10. 1 OR 2 OR 3 OR 4 OR 5 OR 6 OR 7

Immunisation

1. (immuniz* or immunis* or vaccin* or inoculat* or innoculat* or immunotherap* or prophyla*)

Young children and caregivers

1. (child* or infant* or newborn* or neonat* or prenatal or pre natal or antenatal or ante natal or baby or babies or toddler* or preschool* or parent* or mother* or father* or maternal or paternal or caregiver* or grandparent* or grandmother* or grandfather* or family member*)

Impact evaluation methods

For peer review only - http://bmjopen.bmj.com/site/about/guidelines.xhtml

1. (random* or experiment* or (match* adj2 (propensity or coarsened or covariate)) or "propensity score" or "difference in difference*" or "difference-in-difference*" or "differences in difference*" or "differences-in-difference*" or "double difference*" or "quasi-experimental" or "quasi experimental" or "quasi-experiment" or "quasi experiment" or ((estimator or counterfactual) and evaluation*) or "instrumental variable*" or (IV adj2 (estimation or approach)) or regression discontinuity or time series or segment* regression)

- **Example full search strategy**

Below we present a draft of the full search strategy used to search MEDLINE. Note that in MEDLINE's syntax, terms with strokes (e.g., Immunization/) denote Medical Subject Heading (MeSH) terms, while strings appended with "ti,ab,kw" are searched in the title, abstract, and keyword fields of records in the database.

Ovid MEDLINE(R) and Epub Ahead of Print, In-Process & Other Non-Indexed Citations, Daily and Versions(R) <1946 to May 15, 2019> Searched 16th May 2019

1 (immuniz* or immunis* or vaccin* or inoculat* or innoculat* or immunotherap* or prophyla*).ti,ab,kw. (672732)

2 immunization/ or immunization, passive/ or immunization schedule/ or immunization, secondary/ or immunotherapy, active/ or vaccination/ or Immunization Programs/ or mass vaccination/ (158163)

3 Tuberculosis Vaccines/ or BCG Vaccine/ or Diphtheria-Tetanus Vaccine/ or Meningococcal Vaccines/ or Pertussis Vaccine/ or Diphtheria-Tetanus-acellular Pertussis Vaccines/ or Diphtheria-Tetanus-Pertussis Vaccine/ or Diphtheria-Tetanus Vaccine/ or Measles Vaccine/ or Mumps Vaccine/ or Rubella Vaccine/ or Measles-Mumps-Rubella Vaccine/ or Poliovirus Vaccines/ or Poliovirus Vaccine, Inactivated/ or Poliovirus Vaccine, Oral/ or Japanese Encephalitis Vaccines/ or Rotavirus Vaccine/ (49639)

4 or/1-3 (720583)

5 developing countries.sh,kf. (83271)

6 (Africa or Asia or Caribbean or West Indies or South America or Latin America or Central America).ti,ab,kw. (200981)

7 Africa/ or Asia/ or Caribbean/ or West Indies/ or South America/ or Latin America/ or Central America/ (73389)

8 (Africa or Central America or South America or Caribbean or Central Asia or Afghanistan or Albania or Algeria or Angola or Argentina or Armenia or Armenian or Azerbaijan or Bangladesh or Benin or Byelarus or Byelorussian or Belarus or Belorussian or Belorussia or Belize or Bhutan or Bolivia or Bosnia or Herzegovina or Hercegovina or Botswana or Brazil or Bulgaria or Burkina Faso or Burkina Fasso or Upper Volta or Burundi or Urundi or Cambodia or Khmer Republic or Kampuchea or Cameroon or Cameroons or Cameron or Camerons or Cape Verde or Cabo Verde or Central African Republic or Chad or Tchad or China or Colombia or Comoros or Comoro Islands or Comores or Mayotte or Congo or Zaire or Costa Rica or Cote d'Ivoire or Ivory Coast or Cuba or Djibouti or French Somaliland or Dominica or Dominican Republic or East Timor or East Timur or Timor Leste or Ecuador or Egypt or United Arab Republic or El Salvador or Eritrea or Ethiopia or Fiji or Gabon or Gabonese Republic or Gambia or Gaza or Georgia Republic or Georgian Republic or Ghana or Grenada or Guatemala or Guinea or Guiana or Guyana or Haiti or Honduras or India or Maldives or Indonesia or Iran or Iraq or Jamaica or Jordan or Kazakhstan or Kazakh or Kenya or Kiribati or Korea or Kosovo or Kyrgyzstan or Kirghizia or Kyrgyz Republic or Kirghiz or Kirgizstan or Lao PDR or Laos or Lebanon or Lesotho or Basutoland or Liberia or Libya or Macedonia or Madagascar or Malagasy Republic or Malaysia or Malaya or Malay or Sabah or Sarawak or Malawi or Mali or Marshall Islands or Mauritania

For peer review only - http://bmjopen.bmj.com/site/about/guidelines.xhtml

or Mauritius or Agalega Islands or Mexico or Micronesia or Middle East or Moldova or Moldovia or Moldovian or Mongolia or Montenegro or Morocco or Ifni or Mozambique or Myanmar or Myanma or Burma or Namibia or Nepal or Netherlands Antilles or Nicaragua or Niger or Nigeria or Muscat or Pakistan or Palau or Palestine or Panama or Paraguay or Peru or Philippines or Philipines or Phillipines or Phillippines or Papua New Guinea or Romania or Rumania or Roumania or Rwanda or Ruanda or Saint Lucia or St Lucia or Saint Vincent or St Vincent or Grenadines or Samoa or Samoan Islands or Navigator Island or Navigator Islands or Sao Tome or Senegal or Serbia or Montenegro or Seychelles or Sierra Leone or Sri Lanka or Solomon Islands or Somalia or Sudan or Suriname or Surinam or Swaziland or Eswatini or South Africa or Syria or Tajikistan or Tadzhikistan or Tadjikistan or Tadzhik or Tanzania or Thailand or Togo or Togolese Republic or Tonga or Tunisia or Turkey or Turkmenistan or Turkmen or Uganda or Ukraine or Uzbekistan or Uzbek or Vanuatu or New Hebrides or Venezuela or Vietnam or Viet Nam or West Bank or Yemen or Zambia or Zimbabwe or Rhodesia).ti,ab,kw,sh. (1387951)

9 ((developing or less* developed or least developed or under developed or underdeveloped or middle income or low* income or underserved or under served or deprived or poor* or resource limited or resource constrained) adj (countr* or nation? or population? or world or state*)).ti,ab,kw. (91955)

10 ((developing or less* developed or least developed or under developed or underdeveloped or middle income or low* income or resource limited or resource constrained) adj (economy or economies)).ti,ab,kw. (504)

11 (low* adj (gdp or gnp or gross domestic or gross national)).ti,ab,kw. (234)

12 (low adj3 middle adj3 countr*).ti,ab,kw. (12819)

13 (lmic or lmics or third world or lami countr*).ti,ab,kw. (6486)

14 (transitional countr* or emerging econom* or global south).ti,ab,kw. (873)

15 or/5-14 (1487811)

16 4 and 15 (80257)

17 Parents/ or Fathers/ or Mothers/ or Grandparents/ or Caregivers/ or Single Parent/ or Pregnant Women/ or Child, Preschool/ or Infant/ or Infant, Newborn/ or Infant, Low Birth Weight/ or Infant, Small for Gestational Age/ or Infant, Very Low Birth Weight/ or Infant, Extremely Low Birth Weight/ or Infant, Postmature/ or Infant, Premature/ or Infant, Extremely Premature/ (1597959)

18 (child* or infant* or newborn* or neonat* or neo nat* or prenatal or pre natal or ante natal or antenatal or baby or babies or toddler* or preschool* or parent* or mother* or father* or maternal or paternal).ti,ab,kw. (2284298)

19 or/17-18 (2893768)

20 16 and 19 (28515)

21 (random* or experiment* or (match* adj2 (propensity or coarsened or covariate)) or "propensity score" or ("difference in difference*" or "difference-in-difference*" or "differences in difference*" or "differences-in-difference*" or "double difference*") or ("quasi-experimental" or "quasi experimental" or "quasi-experiment" or "quasi experiment") or ((estimator or counterfactual) and evaluation*) or "instrumental variable*" or (IV adj2 (estimation or approach)) or regression discontinuity or time series or segment* regression).ti,ab,kw. (2983909)

For peer review only - http://bmjopen.bmj.com/site/about/guidelines.xhtml

22 Randomized Controlled Trial/ or Random Allocation/ or Evaluation Studies/ or Propensity Score/ or Interrupted Time Series Analysis/ or Controlled Before-After Studies/ or Controlled Clinical Trial/ or Non-Randomized Controlled Trials as Topic/ (896249)

23 or/21-22 (3415351)

24 Cost Analysis/ or Cost-Benefit Analysis/ or Quality-Adjusted Life Years/ or Economics, Medical/ or Cost of Illness/ or Health Care Costs/ or Direct Service Costs/ or Budgets/ or Health Care Sector/ or Public Expenditures/ (193420)

25 (cost-effective* or cost-benefit).ti,ab,kw. (130711)

26 ("life year" or "life years" or qaly* or daly*).ti,ab,kw. (18749)

27 ((economic* or cost*) adj6 (mortality or death* or markov)).ti,ab,kw. (17246)

28 ("cost minimi*" or "cost-utilit*" or "economic evaluation*" or "economic review*" or "cost outcome" or "cost analys*" or "economic analys*" or "budget* impact analys*").ti,ab,kw. (27594)

29 or/24-28 (301485)

30 (review or meta-analysis).pt. (2557796)

31 meta-analysis/ or "systematic review"/ (163880)

32 cochrane database of systematic reviews.jn. (14162)

33 (systematic review or literature review).ti. (130870)

34 or/30-33 (2590211)

35 23 or 29 or 34 (5946083)

36 20 and 35 (8309)

37 exp Animals/ (22312530)

38 Humans/ (17732234)

39 37 not (37 and 38) (4580296)

40 36 not 39 (7984)

For peer review only - http://bmjopen.bmj.com/site/about/guidelines.xhtml

Appendix 4: List of excluded impact evaluation studies

We excluded the following studies at full-text screening for not meeting our community engagement criteria.

Reference list

Aaby, Peter, Adam Roth, Henrik Ravn, Bitiguida Mutna Napirna, Amabelia Rodrigues, Ida Maria Lisse, Lone Stensballe, et al. 2011. "Randomized Trial of BCG Vaccination at Birth to Low-Birth-Weight Children: Beneficial Nonspecific Effects in the Neonatal Period?" The Journal of Infectious Diseases 204 (2): 245–52. https://doi.org/10/ds6pbx.

Aggarwal, Shilpa. 2018. "The Long Road to Health: Healthcare Utilization Impacts of a Road Pavement Policy in Rural India." Working paper. Hyderabad: Indian School of Business. http://eprints.exchange.isb.edu/884/.

Ahmed, Tashrik, Aneesa Arur, Damien de Walque, and Gil Shapira. 2019. "Incentivizing Quantity and Quality of Care: Evidence from an Impact Evaluation of Performance-Based Financing in the Health Sector in Tajikistan." Policy Research Working Papers 8951. Washington, DC: The World Bank. https://doi.org/10.1596/1813-9450-8951.

Alatas, Vivi, Arun Chandrasekhar, Markus Mobius, Benjamin Olken, and Cindy Paladines. 2019. "When Celebrities Speak: A Nationwide Twitter Experiment Promoting Vaccination in Indonesia." NBER Working Paper Series 25589. Cambridge, MA: National Bureau of Economic Research. https://doi.org/10.3386/w25589.

Amaral, João, Eleanor Gouws, Jennifer Bryce, Álvaro Jorge Madeiro Leite, Antonio Ledo Alves da Cunha, and Cesar G. Victora. 2004. "Effect of Integrated Management of Childhood Illness (IMCI) on Health Worker Performance in Northeast-Brazil." Cadernos de Saúde Pública 20 (suppl 2): S209–19. https://doi.org/10/dbvvrz.

Andrade, Mônica Viegas, Flávia Chein, Laetícia Rodrigues de Souza, and Jaume Puig-Junoy. 2012. "Income Transfer Policies and the Impacts on the Immunization of Children: The Bolsa Família Program." Cadernos de Saúde Pública 28 (7): 1347–58. https://doi.org/10/gghf2x.

Andreoni, James, Michael Callen, Muhammad Yasir Khan, Karrar Jaffar, and Charles Sprenger. 2016. "Using Preference Estimates to Customize Incentives: An Application to Polio Vaccination Drives in Pakistan." NBER Working Paper Series 22019. Cambridge, MA: National Bureau of Economic Research. https://doi.org/10.3386/w22019.

Anjum, Qudsia, Aamir Omair, S. N. Bazmi Inam, Yousuf Ahmed, Yaseen Usman, and Shazia Shaikh. 2004. "Improving Vaccination Status of Children under Five through Health Education." JPMA. The Journal of the Pakistan Medical Association 54 (12): 610–13.

Aquino, Rosana, Nelson F. de Oliveira, and Mauricio L. Barreto. 2009. "Impact of the Family Health Program on Infant Mortality in Brazilian Municipalities." American Journal of Public Health 99 (1): 87–93. https://doi.org/10/btntq8.

Arraiz, Irani, and Sandra Rozo. 2011. "Same Bureaucracy, Different Outcomes in Human Capital? How Indigenous and Rural Non-Indigenous Areas in Panama Responded to the CCT." OVE Working Papers 03/11. Washington, D.C.: Inter-American Development Bank. http://www.ssrn.com/abstract=1847124.

Atnafu, Asfaw, Kate Otto, and Christopher H. Herbst. 2017. "The Role of MHealth Intervention on Maternal and Child Health Service Delivery: Findings from a Randomized Controlled Field Trial in Rural Ethiopia." MHealth 3 (September). https://doi.org/10/gcmhjc.

Attanasio, Orazio, Luis Carlos Gómez, Patricia Heredia, and Marcos Vera-Hernández. n.d. "The Short-Term Impact of a Conditional Cash Subsidy on Child Health and Nutrition in Colombia," 15.

Ayieko, Philip, Stephen Ntoburi, John Wagai, Charles Opondo, Newton Opiyo, Santau Migiro, Annah Wamae, et al. 2011. "A Multifaceted Intervention to Implement Guidelines and Improve Admission Paediatric Care in Kenyan District Hospitals: A Cluster Randomised Trial." Edited by Igor Rudan. PLoS Medicine 8 (4). https://doi.org/10/dtj73r.

Balasubramaniam, Sudharsanam, Somesh Kumar, Reena Sethi, Elaine Charurat, Kamlesh Lalchandani, Anne Schuster, and Bulbul Sood. 2018. "Quasi-Experimental Study of Systematic Screening for Family Planning

For peer review only - http://bmjopen.bmj.com/site/about/guidelines.xhtml

Services among Postpartum Women Attending Village Health and Nutrition Days in Jharkhand, India." International Journal of Integrated Care 18 (1). https://doi.org/10/gg9pgr.

Banerjee, Rakesh, and Ashish Sachdeva. 2015. "Pathways to Preventive Health, Evidence from India's Rural Road Program." USC-INET Research Paper 15–19. Los Angeles, CA: USC Dornsife Institute for New Economic Thinking. http://www.ssrn.com/abstract=2636999.

Bangure, Donewell, Daniel Chirundu, Notion Gombe, Tawanda Marufu, Gibson Mandozana, Mufuta Tshimanga, and Lucia Takundwa. 2015. "Effectiveness of Short Message Services Reminder on Childhood Immunization Programme in Kadoma, Zimbabwe - a Randomized Controlled Trial, 2013." BMC Public Health 15 (1). https://doi.org/10/f63zrg.

Barham, Tania. 2005. "The Impact of the Mexican Conditional Cash Transfer Program on Immunization Rates." Boulder: University of Colorado. https://ibs.colorado.edu/barham/wp/CCTimmunWBfin.pdf.

Barham, Tania, Logan E. Brenzel, and John A. Maluccio. 2007. "Beyond 80%: Are There New Ways of Increasing Vaccination Coverage? Evaluation of CCT Programs in Mexico and Nicaragua." HNP Discussion Paper 41537. Washington: World Bank. http://www.ssrn.com/abstract=993760.

Barham, Tania, and John A. Maluccio. 2009. "Eradicating Diseases: The Effect of Conditional Cash Transfers on Vaccination Coverage in Rural Nicaragua." Journal of Health Economics 28 (3): 611–21. https://doi.org/10/czhvx3.

Basinga, Paulin, P. J. Gertler, Agnes Binagwaho, A. L. B. Soucat, J. R. Sturdy, and C. M. J. Vermeersch. 2009. "Impact of Performance Based Financing in Rwanda: Health Facility Level Analysis." Working Paper 32. New Delhi: Global Development Network. http://www.gdn.int/impact-performance-based-financing-rwanda-health-facility-level-analysis.

Basinga, Paulin, Paul J Gertler, Agnes Binagwaho, Agnes LB Soucat, Jennifer Sturdy, and Christel MJ Vermeersch. 2011. "Effect on Maternal and Child Health Services in Rwanda of Payment to Primary Health-Care Providers for Performance: An Impact Evaluation." The Lancet 377 (9775): 1421–28. https://doi.org/10/b2f469.

Beck, Simon, Anni-Maria Pulkki-Brännström, and Miguel San Sebastián. 2015. "Basic Income – Healthy Outcome? Effects on Health of an Indian Basic Income Pilot Project: A Cluster Randomised Trial." Journal of Development Effectiveness 7 (1): 111–26. https://doi.org/10/f3n3kb.

Berhane, Yemane, and Joyce Pickering. 1993. "Are Reminder Stickers Effective in Reducing Immunization Dropout Rates in Addis Ababa, Ethiopia?" Journal of Tropical Medicine and Hygiene 96 (3): 139–45. https://europepmc.org/article/med/8505766.

Bernal, Pedro, and Sebastian Martinez. 2020. "In-Kind Incentives and Health Worker Performance: Experimental Evidence from El Salvador." Journal of Health Economics 70 (March). https://doi.org/10/ghpbkj.

Binyaruka, Peter, Edith Patouillard, Timothy Powell-Jackson, Giulia Greco, Ottar Maestad, and Josephine Borghi. 2015. "Effect of Paying for Performance on Utilisation, Quality, and User Costs of Health Services in Tanzania: A Controlled before and after Study." Edited by Jan Ostermann. PLOS ONE 10 (8). https://doi.org/10/gghf22.

Bonfrer, Igna, Lyn Breebaart, and Ellen Van de Poel. 2016. "The Effects of Ghana's National Health Insurance Scheme on Maternal and Infant Health Care Utilization." Edited by Osman Alimamy Sankoh. PLOS ONE 11 (11). https://doi.org/10/f9q46z.

Bonfrer, Igna, Ellen Van de Poel, and Eddy Van Doorslaer. 2014. "The Effects of Performance Incentives on the Utilization and Quality of Maternal and Child Care in Burundi." Social Science & Medicine 123 (December): 96–104. https://doi.org/10/f6vdkg.

Bossio, Juan Carlos, Ivan Sanchis, Gustavo Ariel Armando, Sergio Javier Arias, and Humberto Jure. 2019. "Resultado de una estrategia de recordatorios previos y posteriores a la fecha de vacunación para mejorar la oportunidad de la vacunación a los seis meses." Cadernos de Saúde Pública 35 (12). https://doi.org/10/ghpbkt.

Bradley, Janet, and Susan Igras. 2005. "Improving the Quality of Child Health Services: Participatory Action by Providers." International Journal for Quality in Health Care 17 (5): 391–99. https://doi.org/10/bdjp6m.

Brenner, Stephan, Rachel P. Chase, Shannon A. McMahon, Julia Lohmann, Christopher J. Makwero, Adamson S. Muula, and Manuela De Allegri. 2020. "Effect Heterogeneity in Responding to Performance-Based Incentives:

For peer review only - http://bmjopen.bmj.com/site/about/guidelines.xhtml

A Quasi-Experimental Comparison of Impacts on Health Service Indicators between Hospitals and Health Centers in Malawi.” Health Systems & Reform 6 (1). https://doi.org/10/gg2njg.
Briere, E. C., T. K. Ryman, E. Cartwright, E. T. Russo, K. A. Wannemuehler, B. L. Nygren, S. Kola, et al. 2012. “Impact of Integration of Hygiene Kit Distribution with Routine Immunizations on Infant Vaccine Coverage and Water Treatment and Handwashing Practices of Kenyan Mothers.” Journal of Infectious Diseases 205 (suppl 1): S56–64. https://doi.org/10/f3wknp.
Brown, V. B., O. A. Oluwatosin, J. O. Akinyemi, and A. A. Adeyemo. 2016. “Effects of Community Health Nurse-Led Intervention on Childhood Routine Immunization Completion in Primary Health Care Centers in Ibadan, Nigeria.” Journal of Community Health 41 (2): 265–73. https://doi.org/10/f8czr8.
Brown, Victoria Bolanle, Oyeninhun Abimbola Oluwatosin, and Martins Olusola Ogundeji. 2017. “Impact of Training Intervention on Immunization Providers’ Knowledge and Practice of Routine Immunization in Ibadan, South-Western Nigeria: A Primary Health Care Experience.” Pan African Medical Journal 26. https://doi.org/10/ghpbkv.
Brughal, R F, and J P Kevany. 1996. “Maximizing Immunization Coverage through Home Visits: A Controlled Trial in an Urban Area of Ghana.” Bulletin of the World Health Organization 74 (5): 517–24. https://www.ncbi.nlm.nih.gov/pmc/articles/PMC2486871/.
Busso, Matias, Julian Cristia, and Sarah Humpage. 2015. “Did You Get Your Shots? Experimental Evidence on the Role of Reminders.” Journal of Health Economics 44 (December): 226–37. https://doi.org/10/f75n4m.
Cahyadi, Nur, Rema Hanna, Benjamin A. Olken, Rizal Adi Prima, Elan Satriawan, Ekki Syamsulhakim, and Rema Hanna. 2018. “Cumulative Impacts of Conditional Cash Transfer Programs: Experimental Evidence from Indonesia.” NBER Working Paper Series 24670. Cambridge: National Bureau of Economic Research. https://www.nber.org/papers/w24670.
Carrillo, Bladimir, Wilman J Iglesias, and Juan C Trujillo. 2015. “Attainments and Limitations of an Early Childhood Programme in Colombia.” Health Policy and Planning 30 (7): 906–16. https://doi.org/10/f7vmx5.
Carvalho, Natalie, Naveen Thacker, Subodh S. Gupta, and Joshua A. Salomon. 2014. “More Evidence on the Impact of India’s Conditional Cash Transfer Program, Janani Suraksha Yojana: Quasi-Experimental Evaluation of the Effects on Childhood Immunization and Other Reproductive and Child Health Outcomes.” Edited by Jeremy D. Goldhaber-Fiebert. PLoS ONE 9 (10). https://doi.org/10/gghf24.
Chakrabarti, Averi, Karen A. Grépin, and Stéphane Helleringer. 2019. “The Impact of Supplementary Immunization Activities on Routine Vaccination Coverage: An Instrumental Variable Analysis in Five Low-Income Countries.” Edited by Vijayaprasad Gopichandran. PLOS ONE 14 (2). https://doi.org/10/gghf25.
Chandir, S., A.J. Khan, H. Hussain, H.R. Usman, S. Khowaja, N.A. Halsey, and S.B. Omer. 2010. “Effect of Food Coupon Incentives on Timely Completion of DTP Immunization Series in Children from a Low-Income Area in Karachi, Pakistan: A Longitudinal Intervention Study.” Vaccine 28 (19): 3473–78. https://doi.org/10/cz4mk3.
Chansa, Collins, Ashis Das, Jumana Qamruddin, Jed Friedman, Akafwilangachi Mkandawire, and Monique Vledder. 2015. “Linking Results to Performance: Evidence from a Results Based Financing Pre-Pilot Project in Katete District, Zambia.” Discussion Paper 98265. Washington, DC: The World Bank. https://openknowledge.worldbank.org/handle/10986/22390?show=full.
Chelagat, T., G. Kokwaro, J. Onyango, and J. Rice. 2020. “Effect of Project-Based Experiential Learning on the Health Service Delivery Indicators: A Quasi-Experiment Study.” BMC Health Services Research 20 (1). https://doi.org/10/gg9pgv.
Chen, Li, Xiaozhen Du, Lin Zhang, Michelle Helena van Velthoven, Qiong Wu, Ruikan Yang, Ying Cao, et al. 2016. “Effectiveness of a Smartphone App on Improving Immunization of Children in Rural Sichuan Province, China: A Cluster Randomized Controlled Trial.” BMC Public Health 16 (1). https://doi.org/10/f82vfx.
Chen, Yvonne Jie, Namrata Chindarkar, and Yun Xiao. 2019. “Effect of Reliable Electricity on Health Facilities, Health Information, and Child and Maternal Health Services Utilization: Evidence from Rural Gujarat, India.” Journal of Health, Population and Nutrition 38 (1). https://doi.org/10/ghpbk6.

For peer review only - http://bmjopen.bmj.com/site/about/guidelines.xhtml

Cristia, Julian, William N. Evans, and Beomsoo Kim. 2015. “Improving the Health Coverage of the Rural Poor: Does Contracting-out Mobile Medical Teams Work?” The Journal of Development Studies 51 (3). https://doi.org/10/ghpwp8.

Cristia, Julián P, William N Evans, and Beomsoo Kim. 2011. “Does Contracting-out Primary Care Services Work? The Case of Rural Guatemala.” IDB Working Paper Series IDB-WP-273. Washington. https://www.econstor.eu/handle/10419/88975.

Cristia, Julian, Ariadna García Prado, and Cecilia Peluffo. 2015. “The Impact of Contracting in and Contracting out Basic Health Services: The Guatemalan Experience.” World Development 70 (June): 215–27. https://doi.org/10/gghf27.

Cruzado de la Vega, Viviana. 2017. “Pagos por desempeño para mejorar estado nutricional infantil: impacto de los convenios de apoyo presupuestario en tres regiones peruanas con alta prevalencia de desnutrición cronica infantil, 2010-2014.” Revista Peruana de Medicina Experimental y Salud Pública 34 (3): 365–76. https://doi.org/10/gghf28.

Daoud, Adel, and Bernhard Reinsberg. 2019. “Structural Adjustment, State Capacity and Child Health: Evidence from IMF Programmes.” International Journal of Epidemiology 48 (2): 445–54. https://doi.org/10/gfqr5z.

Dicko, Alassane, Sidy O Toure, Mariam Traore, Issaka Sagara, Ousmane B Toure, Mahamadou S Sissoko, Alpha T Diallo, et al. 2011. “Increase in EPI Vaccines Coverage after Implementation of Intermittent Preventive Treatment of Malaria in Infant with Sulfadoxine -Pyrimethamine in the District of Kolokani, Mali: Results from a Cluster Randomized Control Trial.” BMC Public Health 11 (1). https://doi.org/10/cvr2cj.

Dissieka, Romance, Marissa Soohoo, Amynah Janmohamed, and David Doledec. 2019. “Providing Mothers with Mobile Phone Message Reminders Increases Childhood Immunisation and Vitamin A Supplementation Coverage in Côte d’Ivoire: A Randomised Controlled Trial.” Journal of Public Health in Africa 10 (1): 56–60. https://doi.org/10/ghpbk9.

Djibuti, Mamuka, George Gotsadze, Akaki Zoidze, George Mataradze, Laura C Esmail, and Jillian Clare Kohler. 2009. “The Role of Supportive Supervision on Immunization Program Outcome - a Randomized Field Trial from Georgia.” BMC International Health and Human Rights 9 (S1). https://doi.org/10/dxd2rk.

Domek, Gretchen J., Ingrid L. Contreras-Roldan, Sheana Bull, Sean T. O’Leary, Guillermo Antonio Bolaños Ventura, Michael Bronsert, Allison Kempe, and Edwin J. Asturias. 2019. “Text Message Reminders to Improve Infant Immunization in Guatemala: A Randomized Clinical Trial.” Vaccine 37 (42): 6192–6200. https://doi.org/10/ghpbmb.

Drain, Paul K, Josoa S Ralaivao, Alexander Rakotonandrasana, and Mary A Carnell. 2003. “Introducing Auto-Disable Syringes to the National Immunization Programme in Madagascar.” Bulletin of the World Health Organization, 8.

D’Souza, Prathibha V, and J Umarani. 2014. “Teaching Package Improves Mothers Knowledge on Vaccine Preventable Diseases and Vaccination: A Quasi Experimental Study.” International Journal of Research in Medical Sciences 2 (3): 976–82. https://doi.org/10/ghpv3h.

Dykstra, Sarah, Amanda Glassman, Charles Kenny, and Justin Sandefur. 2019. “Regression Discontinuity Analysis of Gavi’s Impact on Vaccination Rates.” Journal of Development Economics 140 (September): 12–25. https://doi.org/10/gg9pgw.

Edoka, Ijeoma, Tim Ensor, Barbara McPake, Rogers Amara, Fu-Min Tseng, and Joseph Edem-Hotah. 2016. “Free Health Care for Under-Fives, Expectant and Recent Mothers? Evaluating the Impact of Sierra Leone’s Free Health Care Initiative.” Health Economics Review 6 (1). https://doi.org/10/gghf29.

Eichler, Rena, Paul Auxila, Uder Antoine, and Bernateau Desmangles. 2007. “Performance-Based Incentives for Health: Six Years of Results from Supply-Side Programs in Haiti.” CGD Working Paper 121. Washington, DC: Center for Global Development. http://www.ssrn.com/abstract=1003249.

Ekhaguere, Osayame A, Rosena O Oluwafemi, Bolaji Badejoko, Lawal O Oyeneyin, Azeez Butali, Elizabeth D Lowenthal, and Andrew P Steenhoff. 2019. “Automated Phone Call and Text Reminders for Childhood Immunisations (PRIMM): A Randomised Controlled Trial in Nigeria.” BMJ Global Health 4 (2). https://doi.org/10/gghf3b.

For peer review only - http://bmjopen.bmj.com/site/about/guidelines.xhtml

Eze, G U, and A O Adeleye. 2015. "Enhancing Routine Immunization Performance Using Innovative Technology in an Urban Area of Nigeria." West African Journal of Medicine 34 (1): 3–10. https://pubmed.ncbi.nlm.nih.gov/26902809/.

Gajate-Garrido, Gissele, and Clement Ahiadeke. 2012. "The Effect of Parents' Insurance Enrollment on Health Care Utilization: Evidence from Ghana." SSRN Scholarly Paper ID 2158824. Rochester, NY: Social Science Research Network. https://doi.org/10.2139/ssrn.2158824.

Garly, M. L., C. L. Martins, C. Bale, F. da Costa, F. Dias, H. Whittle, and P. Aaby. 1999. "Early Two-Dose Measles Vaccination Schedule in Guinea-Bissau: Good Protection and Coverage in Infancy." International Journal of Epidemiology 28 (2): 347–52. https://doi.org/10/cgqfgz.

Gauri, Varun. 2009. "Do International Treaties Promote Development? The Convention on the Rights of the Child and Basic Immunization." Policy Research Working Paper 4964. Washington: The World Bank. https://doi.org/10.1596/1813-9450-4964.

Gauri, Varun, and Peyvand Khaleghian. 2002. "Immunization in Developing Countries: Its Political and Organizational Determinants." Policy Research Working Paper 2769. Washington: The World Bank. https://www.infona.pl/resource/bwmeta1.element.elsevier-4a3b393c-c1da-3dff-9b7c-91c05767fb9d.

Gilbert, Sarah Skye, Ngwegwe Bulula, Emmanuel Yohana, Jenny Thompson, Emily Beylerian, Laurie Werner, and Jessica C. Shearer. 2020. "The Impact of an Integrated Electronic Immunization Registry and Logistics Management Information System (EIR-ELMIS) on Vaccine Availability in Three Regions in Tanzania: A Pre-Post and Time-Series Analysis." Vaccine 38 (3): 562–69. https://doi.org/10/ghpbsr.

Goodson, James L., Manisha A. Kulkarni, Jodi L. Vanden Eng, Kathleen A. Wannemuehler, Annett H. Cotte, Rachelle E. Desrochers, Bakolalao Randriamanalina, and Elizabeth T. Luman. 2012. "Improved Equity in Measles Vaccination from Integrating Insecticide-Treated Bednets in a Vaccination Campaign, Madagascar: Equity in Measles Vaccination in Madagascar." Tropical Medicine & International Health 17 (4): 430–37. https://doi.org/10/fxsrbw.

Guindon, G. Emmanuel. 2014. "The Impact of Health Insurance on Health Services Utilization and Health Outcomes in Vietnam." Health Economics, Policy and Law 9 (4): 359–82. https://doi.org/10/f6g47g.

Gultiano, Soccoro A., and Elizabeth M. King. 2006. "A Better Start in Life: Evaluation Results from an Early Childhood Development Program." Philippine Journal of Development 33 (1/2): 101–28. https://ideas.repec.org/p/phd/pjdevt/pjd_2006_vol__xxxiii_nos__1and2-d.html.

Habib, Muhammad Atif, Sajid Soofi, Simon Cousens, Saeed Anwar, Najib ul Haque, Imran Ahmed, Noshad Ali, Rehman Tahir, and Zulfiqar A Bhutta. 2017. "Community Engagement and Integrated Health and Polio Immunisation Campaigns in Conflict-Affected Areas of Pakistan: A Cluster Randomised Controlled Trial." The Lancet Global Health 5 (6): e593–603. https://doi.org/10/f962m2.

Hagiwara, Akiko, Mika Ueyama, Asad Ramlawi, and Yasuyuki Sawada. 2013. "Is the Maternal and Child Health (MCH) Handbook Effective in Improving Health-Related Behavior? Evidence from Palestine." Journal of Public Health Policy 34 (1): 31–45. https://doi.org/10/ghpbss.

Haji, Adam Hassan. 2017. "Evaluation of SMS and Sticker Reminders in Reducing Dropout Rates in Routine Child Immunization in Selected Districts in Kenya." Master Thesis, Juja: Jomo Kenyatta University of Agriculture and Technology. http://ir.jkuat.ac.ke/handle/123456789/3069.

Hajizadeh, Mohammad, Jody Heymann, Erin Strumpf, Sam Harper, and Arijit Nandi. 2015. "Paid Maternity Leave and Childhood Vaccination Uptake: Longitudinal Evidence from 20 Low-and-Middle-Income Countries." Social Science & Medicine 140 (September): 104–17. https://doi.org/10/f7ppdx.

Hategeka, Celestin, Hinda Ruton, and Michael R. Law. 2019. "Effect of a Community Health Worker MHealth Monitoring System on Uptake of Maternal and Newborn Health Services in Rwanda." Global Health Research and Policy 4 (1). https://doi.org/10/ghpbst.

Heinrich, Carolyn J., and Matthew T. Knowles. 2020. "A Fine Predicament: Conditioning, Compliance and Consequences in a Labeled Cash Transfer Program." World Development 129 (May). https://doi.org/10/ghpbsv.

For peer review only - http://bmjopen.bmj.com/site/about/guidelines.xhtml

Helleringer, Stephane, Patrick O. Asuming, and Jalaa Abdelwahab. 2016. “The Effect of Mass Vaccination Campaigns against Polio on the Utilization of Routine Immunization Services: A Regression Discontinuity Design.” Vaccine 34 (33): 3817–22. https://doi.org/10/f8w56m.

Hu, Yu, Yaping Chen, Ying Wang, Quanwei Song, and Qian Li. 2017. “Prenatal Vaccination Education Intervention Improves Both the Mothers’ Knowledge and Children’s Vaccination Coverage: Evidence from Randomized Controlled Trial from Eastern China.” Human Vaccines & Immunotherapeutics 13 (6): 1477–84. https://doi.org/10/gghf3f.

Huillery, Elise, and Juliette Seban. 2019. “Financial Incentives, Efforts, and Performances in the Health Sector: Experimental Evidence from the Democratic Republic of Congo.” Economic Development and Cultural Change, March. https://doi.org/10/ghpbsq.

Hutchinson, Paul, Peter Lance, David K. Guilkey, Mohammad Shahjahan, and Shahida Haque. 2006. “Measuring the Cost-Effectiveness of a National Health Communication Program in Rural Bangladesh.” Journal of Health Communication 11 (sup2): 91–121. https://doi.org/10/b7wdkt.

Ikilezi, Gloria, Orvalho J. Augusto, Joseph L. Dieleman, Kenneth Sherr, and Stephen S. Lim. 2020. “Effect of Donor Funding for Immunization from GAVI and Other Development Assistance Channels on Vaccine Coverage: Evidence from 120 Low and Middle Income Recipient Countries.” Vaccine 38 (3): 588–96. https://doi.org/10/ghpwdv.

Ikilezi, Gloria, Orvalho J Augusto, Alyssa Sbarra, Kenneth Sherr, Joseph L Dieleman, and Stephen S Lim. 2020. “Determinants of Geographical Inequalities for DTP3 Vaccine Coverage in Sub-Saharan Africa.” Vaccine 38 (18): 3447–54. https://doi.org/10/ghpwdw.

Jaupart, Pascal, Lizzie Dipple, and Stefan Dercon. 2019. “Has Gavi Lived up to Its Promise? Quasi-Experimental Evidence on Country Immunisation Rates and Child Mortality.” BMJ Global Health 4 (6). https://doi.org/10/ghpwdx.

Kagucia, E Wangeci. 2018. “MHealth Interventions to Improve Measles Vaccination Coverage and Timeliness: An Assessment of the Immediate and Long-Term Impact on Vaccine-Seeking in Rural Kenya.” PhD Thesis, Baltimore, MD: Johns Hopkins University. https://jscholarship.library.jhu.edu/handle/1774.2/61130.

Kamatsuchi, Mahoko, Adrian Gheorghe, and Dina Balabanova. 2019. “The Global Scale and Implications of Delivering Multiple Interventions through Integrated Child Health Events.” BMJ Global Health 4 (4). https://doi.org/10/ghpwd2.

Kandpal, Eeshani, Harold Alderman, Jed Friedman, Deon Filmer, Junko Onishi, and Jorge Avalos. 2016. “A Conditional Cash Transfer Program in the Philippines Reduces Severe Stunting.” The Journal of Nutrition 146 (9): 1793–1800. https://doi.org/10/f8zrr6.

Karing, Anne. 2018. “Social Signaling and Childhood Immunization: A Field Experiment in Sierra Leone.” Berkeley Working Paper. Berkeley: University of California. https://economics.yale.edu/sites/default/files/jmp_socialsignaling.pdf.

Kawakatsu, Yoshito, Tomohiko Sugishita, Kennedy Oruenjo, Stephen Wakhule, Kennedy Kibosia, Eric Were, and Sumihisa Honda. 2015. “Effectiveness of and Factors Related to Possession of a Mother and Child Health Handbook: An Analysis Using Propensity Score Matching.” Health Education Research 30 (October): 935–46. https://doi.org/10/f749wb.

Kazi, Abdul Momin, Murtaza Ali, Khurram Zubair, Hussain Kalimuddin, Abdul Nafey Kazi, Saleem Perwaiz Iqbal, Jean-Paul Collet, and Syed Asad Ali. 2018. “Effect of Mobile Phone Text Message Reminders on Routine Immunization Uptake in Pakistan: Randomized Controlled Trial.” JMIR Public Health and Surveillance 4 (1). https://doi.org/10/ghpwd3.

Kern, Ana Paula, Marcel de Toledo Vieira, and Ricardo da Silva Freguglia. 2018. “Impactos Do Programa Bolsa Família Na Imunização Das Crianças.” Working paper. https://www.anpec.org.br/encontro/2018/submissao/files_I/i12-cb6dfc0f070c8ca9e77abc8563c3801f.pdf.

Khaleghian, Peyvand. 2003. “Decentralization and Public Services: The Case of Immunization.” Policy Research Working Paper 2989. Washington: The World Bank. https://openknowledge.worldbank.org/handle/10986/19159?show=full.

For peer review only - http://bmjopen.bmj.com/site/about/guidelines.xhtml

Kusuma, Dian, Hasbullah Thabrany, Budi Hidayat, Margaret McConnell, Peter Berman, and Jessica Cohen. 2017. "New Evidence on the Impact of Large-Scale Conditional Cash Transfers on Child Vaccination Rates: The Case of a Clustered-Randomized Trial in Indonesia." World Development 98 (October): 497–505. https://doi.org/10/ghpwd4.

Lamanna, Camillo, and Lauren Byrne. 2019. "A Pilot Study of a Novel, Incentivised MHealth Technology to Monitor the Vaccine Supply Chain in Rural Zambia." Pan African Medical Journal 33. https://doi.org/10/ghpwp5.

Levy, Dan, and Jim Ohls. 2010. "Evaluation of Jamaica's PATH Conditional Cash Transfer Programme." Journal of Development Effectiveness 2 (4): 421–41. https://doi.org/10/dtkp26.

Lin, Meng Ann. 2016. "Stimulating Demand: An Assessment of the Conditional Cash Transfer Project in Afghanistan." PhD Thesis, Baltimore: Johns Hopkins University. https://jscholarship.library.jhu.edu/handle/1774.2/39740.

Liu, Yong, Zhaokang Yuan, Yuxi Liu, Upali W Jayasinghe, and Mark F Harris. 2014. "Changing Community Health Service Delivery in Economically Less-Developed Rural Areas in China: Impact on Service Use and Satisfaction." BMJ Open 4 (2). https://doi.org/10/f6mwb9.

Loevinsohn, B P, and E Gareaballah. 1992. "Missed Opportunities for Immunization during Visits for Curative Care: A Randomized Cross-over Trial in Sudan." Bulletin of the World Health Organization 70 (3): 335–39. https://www.ncbi.nlm.nih.gov/pmc/articles/PMC2393287/.

Loevinsohn, Benjamin P., and Michael E. Loevinsohn. 1986. "Improvement in Coverage of Primary Health Care in a Developing Country through Use of Food Incentives." The Lancet 327 (8493): 1314–16. https://doi.org/10/bjgw7d.

Looij, Frank van de, Dudzai Mureyi, Chenjerai Sisimayi, Jaap Koot, Portia Manangazira, and Nyasha Musuka. 2015. "Early Evidence from Results-Based Financing in Rural Zimbabwe." African Health Monitor 6: 32–36. https://collaboration.worldbank.org/content/sites/collaboration-for-development/en/groups/results-based-financing/groups/oba-rbf-newsroom/documents.entry.html/2016/01/14/early_evidence_from-Kgih.html.

Maluccio, John A., and Rafael Flores. 2005. "Impact Evaluation of a Conditional Cash Transfer Program the Nicaraguan Red de Protección Social." Research report 141. 0 ed. Washington, DC: International Food Policy Research Institute. https://doi.org/10.2499/0896291464RR141.

Mampe, Tumelo, Helen Schneider, and Gavin Reagon. 2016. "Effectiveness of Ward Based Outreach Teams in the North West Province: An Evaluation." Cape Town: University of the Western Cape. http://rgdoi.net/10.13140/RG.2.2.36191.66725.

Masuda, Kazuya, and Chikako Yamauchi. 2020. "How Does Female Education Reduce Adolescent Pregnancy and Improve Child Health? Evidence from Uganda's Universal Primary Education for Fully Treated Cohorts." The Journal of Development Studies 56 (1): 63–86. https://doi.org/10/ggv6vq.

Mathanga, Don P., Elizabeth T. Luman, Carl H. Campbell, Chimwemwe Silwimba, and Grace Malenga. 2009. "Integration of Insecticide-Treated Net Distribution into Routine Immunization Services in Malawi: A Pilot Study." Tropical Medicine & International Health 14 (7): 792–801. https://doi.org/10/b44rkh.

Mazumder, S., S. Taneja, R. Bahl, P. Mohan, T. A. Strand, H. Sommerfelt, B. R. Kirkwood, et al. 2014. "Effect of Implementation of Integrated Management of Neonatal and Childhood Illness Programme on Treatment Seeking Practices for Morbidities in Infants: Cluster Randomised Trial." BMJ 349 (August). https://doi.org/10/gb3snq.

McMahon, Shannon A., Stephan Brenner, Julia Lohmann, Christopher Makwero, Aleksandra Torbica, Don P. Mathanga, Adamson S. Muula, and Manuela De Allegri. 2016. "Evaluating Complex Health Financing Interventions: Using Mixed Methods to Inform Further Implementation of a Novel PBI Intervention in Rural Malawi." BMC Health Services Research 16 (1). https://doi.org/10/f8zjkr.

Mehta, Kala M., Francois Rerolle, Sonali V. Rammohan, Davis C. Albohm, George Muwowo, Heidi Moseson, Lesley Sept, Hau L. Lee, and Eran Bendavid. 2016. "Systematic Motorcycle Management and Health Care Delivery: A Field Trial." American Journal of Public Health 106 (1): 87–94. https://doi.org/10/f8hd4m.

For peer review only - http://bmjopen.bmj.com/site/about/guidelines.xhtml

Mensah, Joseph, Joseph R Oppong, and Christoph M Schmidt. 2009. "Ghana's National Health Insurance Scheme in the Context of the Health MDGs: An Empirical Evaluation Using Propensity Score Matching." Ruhr Economic Papers 157. https://papers.ssrn.com/sol3/papers.cfm?abstract_id=1532169.

Mohan, Pavitrra, Baya Kishore, Sharad Singh, Rajiv Bahl, Anju Puri, and Rajesh Kumar. 2012. "Assessment of Implementation of Integrated Management of Neonatal and Childhood Illness in India." Journal of Health, Population and Nutrition 29 (6): 629–38. https://doi.org/10/ghpwft.

Musa, O I, D B Parakoyi, and A A Akanbi. 2006. "Evaluation of Health Education Intervention on Safe Immunization Injection among Health Workers in Ilorin, Nigeria." Annals of African Medicine 5 (3): 122–28. https://www.semanticscholar.org/paper/Evaluation-of-Health-Education-Intervention-on-Safe-Musa-Parakoyi/5f3eaa0090f82149c7de44c442ec094d31a04591?p2df.

Nanyunja, Miriam, Rosamund F. Lewis, Issa Makumbi, Rachel Seruyange, Eva Kabwongera, Possy Mugyenyi, and Ambrose Talisuna. 2003. "Impact of Mass Measles Campaigns among Children Less than 5 Years Old in Uganda." The Journal of Infectious Diseases 187 (s1): S63–68. https://doi.org/10/bzksr6.

Nasir, Narila Mutia, Yuli Amran, and Yasuhide Nakamura. 2017. "Changing Knowledge and Practices of Mothers on Newborn Care through Mother Class: An Intervention Study in Indonesia." Journal of Tropical Pediatrics 63 (6): 440–46. https://doi.org/10/gghf3q.

Onishi, Junko. 2014. "Philippines Conditional Cash Transfer Program Impact Evaluation 2012." World Bank Report 75533-PH. Washington, DC: The World Bank. https://assessments.hpc.tools/sites/default/files/assessments/Philippines%20Conditional%20Cash%20Transfer%20Program%2C%20Impact%20Evaluation%202012.pdf.

Owais, Aatekah, Beenish Hanif, Amna R Siddiqui, Ajmal Agha, and Anita KM Zaidi. 2011. "Does Improving Maternal Knowledge of Vaccines Impact Infant Immunization Rates? A Community-Based Randomized-Controlled Trial in Karachi, Pakistan." BMC Public Health 11 (1). https://doi.org/10/bpswbk.

Özer, Mustafa, Jan Fidrmuc, and Mehmet Ali Eryurt. 2018. "Maternal Education and Childhood Immunization in Turkey." Health Economics 27 (8): 1218–29. https://doi.org/10/gdkgd3.

Padilla, Alcides de J., and Juan C. Trujillo. 2015. "An Impact Assessment of the Child Growth, Development and Care Program in the Caribbean Region of Colombia." Cadernos de Saúde Pública 31 (10): 2099–2109. https://doi.org/10/gghf3w.

Pandey, Priyanka, Ashwini R. Sehgal, Michelle Riboud, David Levine, and Madhav Goyal. 2007. "Informing Resource-Poor Populations and the Delivery of Entitled Health and Social Services in Rural India: A Cluster Randomized Controlled Trial." JAMA 298 (16): 1867–75. https://doi.org/10/ddxdhz.

Pathak, Yuvraj, and Karen Macours. 2017. "Women's Political Reservation, Early Childhood Development, and Learning in India." Economic Development and Cultural Change 65 (4): 741–66. https://doi.org/10/ghpwf6.

Powell-Jackson, Timothy, Camilla Fabbri, Varun Dutt, Sarah Tougher, and Kultar Singh. 2018. "Effect and Cost-Effectiveness of Educating Mothers about Childhood DPT Vaccination on Immunisation Uptake, Knowledge, and Perceptions in Uttar Pradesh, India: A Randomised Controlled Trial." Edited by James K. Tumwine. PLOS Medicine 15 (3). https://doi.org/10/gc4pqg.

Pramanik, Santanu, Arpita Ghosh, Rituu B. Nanda, Marlou de Rouw, Philip Forth, and Sandra Albert. 2018. "Impact Evaluation of a Community Engagement Intervention in Improving Childhood Immunization Coverage: A Cluster Randomized Controlled Trial in Assam, India." BMC Public Health 18 (1). https://doi.org/10/gdhbjf.

Prinja, Shankar, Ruby Nimesh, Aditi Gupta, Pankaj Bahuguna, Madhu Gupta, and Jarnail Singh Thakur. 2017. "Impact of M-Health Application Used by Community Health Volunteers on Improving Utilisation of Maternal, New-Born and Child Health Care Services in a Rural Area of Uttar Pradesh, India." Tropical Medicine & International Health 22 (7): 895–907. https://doi.org/10/gghf35.

Prosser, Wendy, Philippe Jaillard, Emmanuelle Assy, Shawn T. Brown, Graça Matsinhe, Mawutondji Dekoun, and Bruce Y. Lee. 2017. "System Redesign of the Immunization Supply Chain: Experiences from Benin and Mozambique." Vaccine 35 (17): 2162–66. https://doi.org/10/f96jsd.

For peer review only - http://bmjopen.bmj.com/site/about/guidelines.xhtml

Rahman, Mohammad Mahbubur, and Saseendran Pallikadavath. 2019. “Maternal and Child Health Care Services' Utilization Data from the Fourth Round of District Level Household Survey in India.” Data in Brief 23 (April). https://doi.org/10/ghpwgm.

Rajkotia, Yogesh, Omer Zang, Pierre Nguimkeu, Jessica Gergen, Iva Djurovic, Paula Vaz, Franscisco Mbofana, and Kebba Jobarteh. 2017. “The Effect of a Performance-Based Financing Program on HIV and Maternal/Child Health Services in Mozambique—an Impact Evaluation.” Health Policy and Planning 32 (10): 1386–96. https://doi.org/10/gcfv5b.

Rasella, Davide, Rosana Aquino, Carlos AT Santos, Rômulo Paes-Sousa, and Mauricio L Barreto. 2013. “Effect of a Conditional Cash Transfer Programme on Childhood Mortality: A Nationwide Analysis of Brazilian Municipalities.” The Lancet 382 (9886): 57–64. https://doi.org/10/f2kfx4.

Reinbold, Gary W. 2019. “Effects of the Convention on the Rights of the Child on Child Mortality and Vaccination Rates: A Synthetic Control Analysis.” BMC International Health and Human Rights 19 (1). https://doi.org/10/ghgdqs.

Robinson, J Stephen, Barton R Burkhalter, Barbie Rasmussen, and Ristianto Sugiono. 2001. “Low-Cost on-the-Job Peer Training of Nurses Improved Immunization Coverage in Indonesia.” Bulletin of the World Health Organization 79 (2): 150–58. https://www.scielosp.org/article/bwho/2001.v79n2/150-158/en/.

Roth, A. E., C. Stabell Benn, H. Ravn, A. Rodrigues, I. M. Lisse, M. Yazdanbakhsh, H. Whittle, and P. Aaby. 2010. “Effect of Revaccination with BCG in Early Childhood on Mortality: Randomised Trial in Guinea-Bissau.” BMJ 340 (mar15 1). https://doi.org/10/c8q74b.

Rusa, Louis, Jean de Dieu Ngirabega, Willy Janssen, Stefaan Van Bastelaere, Denis Porignon, and Werner Vandenbulcke. 2009. “Performance-Based Financing for Better Quality of Services in Rwandan Health Centres: 3-Year Experience.” Tropical Medicine & International Health 14 (7): 830–37. https://doi.org/10/cc5zvh.

Ryman, Tove K., Elizabeth C. Briere, Emily Cartwright, Karen Schlanger, Kathleen A. Wannemuehler, Elizabeth T. Russo, Steve Kola, et al. 2012. “Integration of Routine Vaccination and Hygiene Interventions: A Comparison of 2 Strategies in Kenya.” The Journal of Infectious Diseases 205 (suppl_1): S65–76. https://doi.org/10/f3wkpb.

Ryman, Tove K., Ajay Trakroo, Aaron Wallace, Satish Kumar Gupta, Karen Wilkins, Pankaj Mehta, and Vance Dietz. 2011. “Implementation and Evaluation of the Reaching Every District (RED) Strategy in Assam, India, 2005–2008.” Vaccine 29 (14): 2555–60. https://doi.org/10/bjqf85.

Salami, Lamidhi, Edgard-Marius Dona Ouendo, and Benjamin Fayomi. 2016. “Effects of Results Based Financing Models on Data Quality Improvement in Benin on 2014.” Universal Journal of Public Health 4 (6): 324–31. https://doi.org/10/ghpwgc.

Salami, Lamidhi, Edgard-Marius Ouendo, and Benjamin Fayomi. 2018. “Effects of Results Based Financing Models on the Performance of Exposed Health Zones in Benin.” International Journal Of Community Medicine And Public Health 5 (10): 4188–99. https://doi.org/10/ghpwgd.

Sangwan, Santosh, and Anju Manocha. 2009. “Maternal Knowledge and Child Health.” Journal of Human Ecology 25 (1): 51–54. https://doi.org/10/gghf33.

Sato, Ryoko, and Abdullahi Belel. 2020. “The Effect of Performance-Based Financing on Child Vaccinations in Northern Nigeria.” Vaccine 38 (9): 2209–15. https://doi.org/10/ghpwgf.

Schlumberger, M., A. Bamoko, T.M. Yaméogo, F. Rouvet, R. Ouedraogo, B. Traoré, M. Tinto, et al. 2015. “Impact positif sur le Programme élargi de vaccinations de l'envoi de SMS de rappel à partir d'un registre informatisé, Bobo-Dioulasso (Burkina Faso).” Bulletin de la Société de pathologie exotique 108 (5): 349–54. https://doi.org/10/gghf34.

Sengupta, Nandana, and Aakanksha Sinha. 2018. “Is India's Safe Motherhood Scheme Leading to Better Child Health Care Practices?” Global Social Welfare 5 (1): 49–58. https://doi.org/10/ghpwgg.

Shei, Amie, Federico Costa, Mitermayer G Reis, and Albert I Ko. 2014. “The Impact of Brazil's Bolsa Família Conditional Cash Transfer Program on Children's Health Care Utilization and Health Outcomes.” BMC International Health and Human Rights 14 (1). https://doi.org/10/gbf36j.

For peer review only - http://bmjopen.bmj.com/site/about/guidelines.xhtml

Sherry, Tisamarie B., Sebastian Bauhoff, and Manoj Mohanan. 2017. “Multitasking and Heterogeneous Treatment Effects in Pay-for-Performance in Health Care: Evidence from Rwanda.” American Journal of Health Economics 3 (2): 192–226. https://doi.org/10/ghpwgh.

Shuaib, Waqas, Julia Marielly Suarez, Juan David Romero, Carlos Dillon Pamello, Richard Alweis, Aizaaz Ali Khan, Syed Raza Shah, Hassan Shahid, Serge B. PierreCharles, and Laura Rosemary Sanchez. 2016. “Transforming Patient Care by Introducing an Electronic Medical Records Initiative in a Developing Country.” Health Informatics Journal 22 (4): 975–83. https://doi.org/10/gghf36.

Sinha, Nistha, and Joanne K Yoong. 2009. “Long-Term Financial Incentives and Investment in Daughters: Evidence from Conditional Cash Transfers in North India.” RAND Labor and Population working paper series WR-667. Santa Monica: RAND Corporation. https://doi.org/10.1596/1813-9450-4860.

Soeters, Robert, Peter Bob Peerenboom, Pacifique Mushagalusa, and Célestin Kimanuka. 2011. “Performance-Based Financing Experiment Improved Health Care in the Democratic Republic of Congo.” Health Affairs 30 (8): 1518–27. https://doi.org/10/c6w2rq.

Steinhardt, L. C., I. Aman, I. Pakzad, B. Kumar, L. P. Singh, and D. H. Peters. 2011. “Removing User Fees for Basic Health Services: A Pilot Study and National Roll-out in Afghanistan.” Health Policy and Planning 26 (Suppl. 2): ii92–103. https://doi.org/10/drchbp.

Subedi, Sudarshan, Bishnu Prasad Sharma, and Chiranjivi Adhikari. 2018. “Cost and Consequences of Integrated Public Health Campaign in Baglung District, Nepal.” Economic Journal of Development Issues 23 & 24 (January): 113–22. https://doi.org/10/ghpwgk.

Talukder, Md., Ubaidur Rob, Syed Abu Musa, Ashish Bajracharya, Kaji Keya, Forhana Noor, Eshita Jahan, Md. Hossain, Jyotirmoy Saha, and Benjamin Bellows. 2014. “Evaluation of the Impact of the Voucher Program for Improving Maternal Health Behavior and Status in Bangladesh.” Dhaka: Population Council. https://doi.org/10.31899/rh10.1000.

Tanner, Jeffery, Ryotaro Hayashi, and Yunsun Li. 2015. “Improving Coverage and Utilization of Maternal and Child Health Services in Lao PDR: Impact Evaluation of the Community Nutrition Project.” IEG Working Paper 99929. Washington: The World Bank Group. https://documents.worldbank.org/en/publication/documents-reports/documentdetail/441841468187767237/improving-coverage-and-utilization-of-maternal-and-child-health-services-in-lao-pdr-impact-evaluation-of-the-community-nutrition-project.

Thomas, Ranjeeta Alice. 2011. “Essays on Ex Ante Evaluations of Cash Transfer Programs.” PhD Thesis, York: University of York. http://etheses.whiterose.ac.uk/2004/.

Thomson, Dana R, Cheryl Amoroso, Sidney Atwood, Matthew H Bonds, Felix Cyamatare Rwabukwisi, Peter Drobac, Karen E Finnegan, et al. 2018. “Impact of a Health System Strengthening Intervention on Maternal and Child Health Outputs and Outcomes in Rural Rwanda 2005–2010.” BMJ Global Health 3 (2). https://doi.org/10/gdcrw5.

Thysen, Sanne Marie, Stine Byberg, Marie Pedersen, Amabelia Rodrigues, Henrik Ravn, Cesario Martins, Christine Stabell Benn, Peter Aaby, and Ane Bærent Fisker. 2014. “BCG Coverage and Barriers to BCG Vaccination in Guinea-Bissau: An Observational Study.” BMC Public Health 14 (1). https://doi.org/10/f6k4xv.

Uddin, Md. Jasim, Md. Shamsuzzaman, Lily Horng, Alain Labrique, Lavanya Vasudevan, Kelsey Zeller, Mridul Chowdhury, Charles P. Larson, David Bishai, and Nurul Alam. 2016. “Use of Mobile Phones for Improving Vaccination Coverage among Children Living in Rural Hard-to-Reach Areas and Urban Streets of Bangladesh.” Vaccine 34 (2): 276–83. https://doi.org/10/f766t2.

Unger, Jean-Pierre. 1991. “Can Intensive Campaigns Dynamize Front Line Health Services? The Evaluation of an Immunization Campaign in Thiès Health District, Senegal.” Social Science & Medicine 32 (3): 249–59. https://doi.org/10/bc5fn2.

Usman, Hussain R., Saeed Akhtar, Faiza Habib, and Imtiaz Jehan. 2009. “Redesigned Immunization Card and Center-Based Education to Reduce Childhood Immunization Dropouts in Urban Pakistan: A Randomized Controlled Trial.” Vaccine 27 (3): 467–72. https://doi.org/10/bcv8fd.

For peer review only - http://bmjopen.bmj.com/site/about/guidelines.xhtml

Usman, Hussain R., Mohammad H. Rahbar, Sibylle Kristensen, Sten H. Vermund, Russell S. Kirby, Faiza Habib, and Eric Chamot. 2011. “Randomized Controlled Trial to Improve Childhood Immunization Adherence in Rural Pakistan: Redesigned Immunization Card and Maternal Education.” Tropical Medicine & International Health 16 (3): 334–42. https://doi.org/10/dm9tm7.

Vaidyanathan, Radha. 2019. “Immunization Coverage among Under-Five Children Living along a School Student through Child-to-Child and Child-to-Parent Information, Education and Communication Strategy.” Indian Journal of Public Health 63 (4): 334–40. https://doi.org/10/ghpwgq.

Van de Poel, Ellen, Gabriela Flores, Por Ir, and Owen O’Donnell. 2016. “Impact of Performance-Based Financing in a Low-Resource Setting: A Decade of Experience in Cambodia.” Health Economics 25 (6): 688–705. https://doi.org/10/f8kzhk.

Varghese, Beena, Reetabrata Roy, Somen Saha, and Sidsel Roalkvam. 2014. “Fostering Maternal and Newborn Care in India the Yashoda Way: Does This Improve Maternal and Newborn Care Practices during Institutional Delivery?” Edited by Hamid Reza Baradaran. PLoS ONE 9 (1). https://doi.org/10/f5s7zr.

Verma, M, J Chhatwal, and P V Varughese. 1995. “Antenatal Period: An Educational Opportunity.” Indian Pediatrics 32: 171–77. https://www.semanticscholar.org/paper/Antenatal-period%3A-an-educational-opportunity.-Verma-Chhatwal/b44c9c679879ada229d8a2719b5637155190f4d1?p2df.

Wadhwa, Sagar. 2019. “Conditional Cash Transfers and Parental Investment in Daughters: Evidence from India.” Paper presented at the Population Association of America 2019 Annual Meeting, Austin, TX. http://paa2019.populationassociation.org/abstracts/193315.

Wagstaff, Adam, and Shengchao Yu. 2007. “Do Health Sector Reforms Have Their Intended Impacts ? The World Bank’S Health VIII Project in Gansu Province, China.” Journal of Health Economics 26: 505–35. https://doi.org/10/cx4kc6.

Wallace, Aaron S., Kenny Peetosutan, Andi Untung, Marisa Ricardo, Prima Yosephine, Kathleen Wannemuehler, David W. Brown, et al. 2019. “Home-Based Records and Vaccination Appointment Stickers as Parental Reminders to Reduce Vaccination Dropout in Indonesia: A Cluster-Randomized Controlled Trial.” Vaccine 37 (45): 6814–23. https://doi.org/10/ghpwgw.

Walque, Damien de, Paul Jacob Robyn, Hamadou Saidou, Gaston Sorgho, and Maria Steenland. 2017. “Looking into the Performance-Based Financing Black Box: Evidence from an Impact Evaluation in the Health Sector in Cameroon.” Policy Research Working Paper 8162. Washington, DC: The World Bank. https://doi.org/10.1596/1813-9450-8162.

Wang, Paul C., Albert Mwango, Sarah Moberley, Benjamin J. Brockman, Alison L. Connor, Penelope Kalesha-Masumbu, Simon Mutembo, et al. 2015. “A Cluster Randomised Trial on the Impact of Integrating Early Infant HIV Diagnosis with the Expanded Programme on Immunization on Immunization and HIV Testing Rates in Rural Health Facilities in Southern Zambia.” Edited by David Joseph Diemert. PLOS ONE 10 (10). https://doi.org/10/f779tz.

Weldemariam, Mesele. 2010. “The Impact of Fiscal Decentralization on Education and Health Outcomes in Ethiopia: A Regional Panel Data Analysis.” Master Thesis, Addis Ababa: Addis Ababa University. http://etd.aau.edu.et/handle/123456789/14217?show=full.

Wong, Benjamin KC, Shaza A Fadel, Shally Awasthi, Ajay Khera, Rajesh Kumar, Geetha Menon, and Prabhat Jha. 2019. “The Impact of Measles Immunization Campaigns in India Using a Nationally Representative Sample of 27,000 Child Deaths.” ELife 8 (March). https://doi.org/10/ghpwgx.

Yoong, Joanne. 2007. “Does Decentralization Hurt Childhood Immunization?” Stanford: Stanford University. http://citeseerx.ist.psu.edu/viewdoc/summary?doi=10.1.1.370.4021.

Yoosuf, Abdul-Sattar. 1993. “Group Learning by Mothers about Primary Health Care.” World Health Forum 14 (1): 20–22. https://pubmed.ncbi.nlm.nih.gov/8439363/.

Zang, Omer, Sebastien Djienouassi, Gaston Sorgho, and Jean Claude Taptue. 2015. “Impact of Performance-Based Financing on Health-Care Quality and Utilization in Urban Areas of Cameroon.” African Health Monitor 7 (21): 10–14. https://indexmedicus.afro.who.int/aim/opac_css/doc_num.php?explnum_id=72209.

For peer review only - http://bmjopen.bmj.com/site/about/guidelines.xhtml

Zeng, Wu, Donald S Shepard, Jean de Dieu Rusatira, Aaron P Blaakman, and Bernice M Nsitou. 2018. “Evaluation of Results-Based Financing in the Republic of the Congo: A Comparison Group Pre–Post Study.” Health Policy and Planning 33 (3): 392–400. https://doi.org/10/gcvmpw.

Zeng, Wu, Daxin Sun, Henry Mphwanthe, Tianwen Huan, Jae Eun Nam, Pascal Saint-Firmin, Gerald Manthalu, Suneeta Sharma, and Arin Dutta. 2019. “The Impact and Cost-Effectiveness of User Fee Exemption by Contracting out Essential Health Package Services in Malawi.” BMJ Global Health 4 (2). https://doi.org/10/gghf4b.

Zizien, Zawora Rita, Catherine Korachais, Philippe Compaoré, Valéry Ridde, and Vincent De Brouwere. 2019. “Contribution of the Results-based Financing Strategy to Improving Maternal and Child Health Indicators in Burkina Faso.” The International Journal of Health Planning and Management 34 (1): 111–29. https://doi.org/10/gd2zrp.

Zombré, David, Manuela De Allegri, and Valéry Ridde. 2020. “No Effects of Pilot Performance-Based Intervention Implementation and Withdrawal on the Coverage of Maternal and Child Health Services in the Koulikoro Region, Mali: An Interrupted Time Series Analysis.” Health Policy and Planning 35 (4): 379–87. https://doi.org/10/ggnfpf.

For peer review only

Appendix 5: Qualitative and cost-evidence search strategy

Qualitative search protocol

Purpose

The purpose of this review is to inform policy recommendations related to community engagement interventions to improve immunisation outcomes in low- and middle-income countries. Specifically, we would like to provide insights related to (1) what to do / not do and (2) where to do it / not do it. We want to know not only what works, but why and how.

Our primary, qualitative research question is: What factors relating to programme design, implementation, context, and mechanism are associated with better or worse outcomes along the causal chain? Do these vary by the level of community engagement?

Sub questions are:

1. What pre-intervention activities and characteristics of local context facilitate or inhibit the effectiveness of an intervention?
    a. What characteristics of local context facilitate or inhibit the effectiveness of an intervention? These include but are not limited to feasibility, clinic readiness, acceptability, need, barriers, and demographic composition.
    b. What is the evidence regarding the likelihood that pre-intervention activities, including diagnostics, formative research, and community engagement, affect the effectiveness of an intervention?
2. What is the evidence supporting or refuting the existence of certain steps along the ToC?
    a. Which steps in the causal chain tend to be barriers to or facilitators of impact? Where does the causal chain tend to be interrupted?
    b. What time varying contextual factors affect the relationships seen in the ToC?

In order to respond to these questions, 3ie seeks to identify qualitative papers related to the quantitative papers included in the broader systematic review on effectiveness of community engagement interventions for improving immunisation outcomes. This information will contextualise the results of the quantitative papers. The qualitative papers will be instrumental in expanding our understanding of why and how certain interventions were successful (or not).

This document outlines the suggested approach for identifying these qualitative papers.

Required steps

1. Create a unique dropbox folder for each study using the first author's last name and year as the folder title
2. For each step outlined below, save all potentially relevant documents to this file as they are identified.

    a. Consider this stage to be equivalent to title and abstract screening. If you identify an article that can be quickly discarded as irrelevant (such as budgetary information), there is no need to include. However, if you think that there may be any information related to our research questions, please include at this stage.

    b. If relevant websites are identified that cannot be easily converted to PDF format, create a word document. Write the title of the webpage and provide the link below it.

If a trial registration number for clinicaltrials.gov is provided

For peer review only - http://bmjopen.bmj.com/site/about/guidelines.xhtml

Use this approach if a trial registration number for clinicaltrials.gov is provided. This will be referenced in the abstract and / methods section and appear in a form similar to that found in Robertson et al 2013:

This trial is registered with ClinicalTrials.gov, number NCT00966849.

If no trial registration number is indicated, proceed to the following section (Section 2: If the project has a unique name).

1.1 Go to the website clinialTrials.gov

1.2 Under “find a study” in the field “other terms,” enter the trial registration number.

1.3 There should only be one hit. Click on the title under “study title”

1.4 Three tabs should be available under the basic study information. They read “study details,” “tabular view,” and “study results.” Select “study results”

1.5 Scroll to the bottom of the page. Under “more information” there are sections titled “Publication of results” and “other publications”

   a. In most (but not all) cases, there should be a list of publications here. Save the relevant files to the folder

After this, proceed to section 2.

If the project has an unique name

Use this approach if the intervention is named. If a project name is not given in the paper, but the project is registered on clinicaltrials.gov, use the project title provided there.

2.1 Search the project name on google scholar.

   a. Review each search result until you find 5 in a row that are irrelevant.

   b. At this point, stop and proceed to step 2.2.

2.2 If the funder’s name is not provided, proceed directly to step 2.3. If the funder’s name is provided, search the project name on the funder’s website.

   a. Review each search result until you find 5 in a row that are irrelevant.

   b. At this point, stop and proceed to step 2.3.

2.3 If the implementer’s name is not provided, proceed directly to section 3. If the implementer’s name is provided, search the project name on the implementer's website.

   a. Review each search result until you find 5 in a row that are irrelevant.

Other options

If the trial is not registered and does not have a formal name, the following approaches can be used as a last resort:

For peer review only - http://bmjopen.bmj.com/site/about/guidelines.xhtml

3.1 If funder and / or implementer names are provided, go to their websites. Search for the article on their website. It may link to a program page.

a. If no program page is identified, try to manually search through their website.

3.2 Search the full article name on google and google scholar. Review the top 10 hits on each to see if they provide more information about the program.

If during either of these steps, a program name and / or trial registration number are identified, return to the relevant steps above.

Searching funder and implementer websites

Some of these websites will have good, built-in search functions. If these are useful, use them. However, if the provided search functionality is not adequate, type site:HomepageOfFunderWebsite SearchTerm. For example, if I wanted to search the World Bank website for information about COVID, I would search "site:www.worldbank.org COVID" in google (Note: no space between "site:" and the url). This will provide me hits from the World Bank website related to COVID.

If no linked articles are identified

At the end of each day, email Avantika (abagai@3ieimpact.org) and Charlotte (clane@3ieimpact.org) the title of all papers for which you were unable to identify linked articles.

Cost evidence search protocol

The evaluations included in the cost and cost-effectiveness evidence were drawn from the 61 evaluations that were identified for the systematic review. Since cost reporting and analysis is often not required by donors or included in impact evaluations of global development interventions, we undertook structured outreach to the authors of the 61 included studies to request any additional cost evidence. In case the lead or corresponding author email addresses were nonfunctional, we contacted one of the co-authors.

We successfully contacted 58 out of 61 study authors that reported intervention effectiveness only or intervention effectiveness and partial cost information and requested estimates of the intervention costs as well as any additional raw data files, summary tables, sensitivity analyses, cost evidence, published reports or notes, and non-published documentation, methods or analyses relating to cost-effectiveness. In the first two weeks of this request, our team obtained responses from a total of 25 study authors, of which 12 produced or pointed us in the direction of economic evidence for their respective interventions and 7 authors confirmed that any such costing analysis was not undertaken as part of their study. We excluded any studies that did not report estimates of both costs and effectiveness and identified 22 evaluations with both cost and effectiveness estimates.

List of included qualitative studies

Ministry of Public Health and Sanitation. 2012. National Communication strategy for community health services. Nairobi, Kenya: Ministry of Public Health and Sanitation, Republic of Kenya. https://www.slideshare.net/chskenya/chs-kenya-national-communicationstrategyforcommunityhealthservices20122017

Adamu, Abdu A., O. A. Uthman, M. A. Gadanya, and C. S. Wiysonge. 2020. "Using the consolidated framework for implementation research (CFIR) to assess the implementation context of a quality improvement program to reduce missed opportunities for vaccination in Kano, Nigeria: a mixed methods study." *Hum Vaccin Immunother* 16 (2):465-475. doi: 10.1080/21645515.2019.1654798.

For peer review only - http://bmjopen.bmj.com/site/about/guidelines.xhtml

Anwari, Z., M. Shukla, B. A. Maseed, G. F. Wardak, S. Sardar, J. Matin, G. S. Rashed, S. A. Hamedi, H. Sahak, A. H. Aziz, M. Boyd-Boffa, and R. Trasi. 2015. "Implementing people-centred health systems governance in 3 provinces and 11 districts of Afghanistan: a case study." *Confl Health* 9:2. doi: 10.1186/1752-1505-9-2.

Baba-Ari, F., E. A. Eboreime, and M. Hossain. 2018. "Conditional Cash Transfers for Maternal Health Interventions: Factors Influencing Uptake in North-Central Nigeria." *Int J Health Policy Manag* 7 (10):934-942. doi: 10.15171/ijhpm.2018.56.

Balarajan, Yarlini, and Michael R. Reich. 2016. "Political economy of child nutrition policy: A qualitative study of India's Integrated Child Development Services (ICDS) scheme." *Food Policy* 62:88-98. doi: https://doi.org/10.1016/j.foodpol.2016.05.001.

Billah, S. M., D. E. Hoque, M. Rahman, A. Christou, N. S. Mugo, K. Begum, T. Tahsina, Q. S. Rahman, E. K. Chowdhury, T. M. Haque, R. Khan, A. Siddik, J. Bryce, R. E. Black, and S. El Arifeen. 2018. "Feasibility of engaging "Village Doctors" in the Community-based Integrated Management of Childhood Illness (C-IMCI): experience from rural Bangladesh." *J Glob Health* 8 (2):020413. doi: 10.7189/jogh.08.020413.

Chimhutu, Victor. 2011. "Pay for Performance in Maternal Health in Tanzania: Perceptions, Expectations and Experiences in Mvomero district."

Chimhutu, Victor, Nils Gunnar Songstad, Marit Tjomsland, Mwifadhi Mrisho, and Karen Marie Moland. 2016. "The inescapable question of fairness in Pay-for-performance bonus distribution: a qualitative study of health workers' experiences in Tanzania." *Globalization and Health* 12 (1):77. doi: 10.1186/s12992-016-0213-5.

Chimhutu, Victor, Marit Tjomsland, and Mwifadhi Mrisho. 2019. "Experiences of care in the context of payment for performance (P4P) in Tanzania." *Globalization and Health* 15 (1):59. doi: 10.1186/s12992-019-0503-9.

Chimhutu, Victor, Marit Tjomsland, Nils Gunnar Songstad, Mwifadhi Mrisho, and Karen Marie Moland. 2015. "Introducing payment for performance in the health sector of Tanzania- the policy process." *Globalization and Health* 11 (1):38. doi: 10.1186/s12992-015-0125-9.

Doctor, H. V., S. E. Findley, A. Ager, G. Cometto, G. Y. Afenyadu, F. Adamu, and C. Green. 2012. "Using community-based research to shape the design and delivery of maternal health services in Northern Nigeria." *Reprod Health Matters* 20 (39):104-12. doi: 10.1016/s0968-8080(12)39615-8.

Grayman, Jesse Hession, Siti Ruhanawati, and Nelti Anggraini. 2014. "Opportunities and approaches for better nutrition outcomes through PNPM generasi : a qualitative study."

Henning, M. J., J. M. Zulu, C. Michelo, S. Simmons Zuilkowski, and C. Hubner. 2020. "Adolescent Mothers' Experiences With Community Health Assistants in Rural Zambia." *Int Q Community Health Educ* 40 (4):353-361. doi: 10.1177/0272684x19896737.

Khuzwayo, L. S., and M. Moshabela. 2017. "The perceived role of ward-based primary healthcare outreach teams in rural KwaZulu-Natal, South Africa." *Afr J Prim Health Care Fam Med* 9 (1):e1-e5. doi: 10.4102/phcfm.v9i1.1388.

Lignou, Sapfo, Sushmita Das, Jigna Mistry, Glyn Alcock, Neena Shah More, David Osrin, and Sarah J. L. Edwards. 2016. "Reconstructing communities in cluster trials?" *Trials* 17 (1):166. doi: 10.1186/s13063-016-1284-6.

Marcus, Tessa S., Jannie Hugo, and Champak C. Jinabhai. 2017. "Which primary care model? A qualitative analysis of ward-based outreach teams in South Africa." *African journal of primary health care & family medicine* 9 (1):e1-e8. doi: 10.4102/phcfm.v9i1.1252.

Moosa, S., A. Derese, and W. Peersman. 2017. "Insights of health district managers on the implementation of primary health care outreach teams in Johannesburg, South Africa: a descriptive study with focus group discussions." *Hum Resour Health* 15 (1):7. doi: 10.1186/s12960-017-0183-6.

Okeke, Edward N., Emma Pitchforth, Josephine Exley, Peter Glick, Isa Sadeeq Abubakar, Amalavoyal V. Chari, Usman Bashir, Kun Gu, and Obinna Onwujekwe. 2017. "Going to scale: design and implementation challenges of a program to increase access to skilled birth attendants in Nigeria." *BMC Health Services Research* 17 (1):356. doi: 10.1186/s12913-017-2284-2.

Oladepo, O., I. O. Dipeolu, and O. Oladunni. 2019. "Nigerian rural mothers' knowledge of routine childhood immunizations and attitudes about use of reminder text messages for promoting timely completion." *J Public Health Policy* 40 (4):459-477. doi: 10.1057/s41271-019-00180-7.

For peer review only - http://bmjopen.bmj.com/site/about/guidelines.xhtml

Oladepo, Oladimeji, Isaac Oluwafemi Dipeolu, and Opeyemi Oladunni. 2020. "Outcome of reminder text messages intervention on completion of routine immunization in rural areas, Nigeria." *Health Promotion International*. doi: 10.1093/heapro/daaa092.

Olafsdottir, Anna Elisabet, Iddy Mayumana, Irene Mashasi, Ikunda Njau, Masuma Mamdani, Edith Patouillard, Peter Binyaruka, Salim Abdulla, and Josephine Borghi. 2014. "Pay for performance: an analysis of the context of implementation in a pilot project in Tanzania." *BMC Health Services Research* 14 (1):392. doi: 10.1186/1472-6963-14-392.

Pérez, Myriam Cielo, Dinesh Chandra, Georges Koné, Rohit Singh, Valery Ridde, Marie-Pierre Sylvestre, Aaditeshwar Seth, and Mira Johri. 2020. "Implementation fidelity and acceptability of an intervention to improve vaccination uptake and child health in rural India: a mixed methods evaluation of a pilot cluster randomized controlled trial." *Implementation Science Communications* 1 (1):88. doi: 10.1186/s43058-020-00077-7.

Phiri, S. C., M. L. Prust, C. P. Chibawe, R. Misapa, J. W. van den Broek, and N. Wilmink. 2017. "An exploration of facilitators and challenges in the scale-up of a national, public sector community health worker cadre in Zambia: a qualitative study." *Hum Resour Health* 15 (1):40. doi: 10.1186/s12960-017-0214-3.

Rahayu;, Sri Kusumastuti, Nina Toyamah;, Stella Aleida Hutagalung;, Meuthia Rosfadhila; &, and Muhammad Syukri. 2008. Qualitative Baseline Study for PNPM Generasi and PKH : The Availability and Use of the Maternal and Child Health Services and Basic Education Services in the Provinces of West Java and East Nusa Tenggara. In *Development Economics Working Papers 22536*: East Asian Bureau of Economic Research.

Rahman, A. 2007. "Challenges and opportunities in developing a psychological intervention for perinatal depression in rural Pakistan--a multi-method study." *Archives of women's mental health* 10 (5):211-219. doi: 10.1007/s00737-007-0193-9.

Rahman, Atiya, Margaret Leppard, Sarawat Rashid, Nauruj Jahan, and Hashima E. Nasreen. 2016. "Community perceptions of behaviour change communication interventions of the maternal neonatal and child health programme in rural Bangladesh: an exploratory study." *BMC Health Services Research* 16 (1):389. doi: 10.1186/s12913-016-1632-y.

Robertson, Laura, Phyllis Mushati, Morten Skovdal, Jeffrey W. Eaton, Jeremiah C. Makoni, Tom Crea, Gideon Mavise, Lovemore Dumba, Christina Schumacher, Lorraine Sherr, Constance Nyamukapa, and Simon Gregson. 2014. "Involving Communities in the Targeting of Cash Transfer Programs for Vulnerable Children: Opportunities and Challenges." *World Development* 54:325-337. doi: https://doi.org/10.1016/j.worlddev.2013.09.002.

Sacks, Audrey and Grayman, Jesse Hession. 2018. Indonesia - Long-Term Generasi Qualitative Study (English)

Shah, P., N. Madhiwala, S. Shah, G. Desai, K. Dave, N. Dholakia, S. Patel, S. Desai, and D. Modi. 2019. "High uptake of an innovative mobile phone application among community health workers in rural India: An implementation study." *Natl Med J India* 32 (5):262-269. doi: 10.4103/0970-258x.295956.

Shelley, K. D., Y. W. Belete, S. C. Phiri, M. Musonda, E. C. Kawesha, E. M. Muleya, C. P. Chibawe, J. W. van den Broek, and K. B. Vosburg. 2016. "Implementation of the Community Health Assistant (CHA) Cadre in Zambia: A Process Evaluation to Guide Future Scale-Up Decisions." *J Community Health* 41 (2):398-408. doi: 10.1007/s10900-015-0110-5.

Skovdal, Morten, Phyllis Mushati, Laura Robertson, Shungu Munyati, Lorraine Sherr, Constance Nyamukapa, and Simon Gregson. 2013. "Social acceptability and perceived impact of a community-led cash transfer programme in Zimbabwe." *BMC Public Health* 13 (1):342. doi: 10.1186/1471-2458-13-342.

Skovdal, Morten, Laura Robertson, Phyllis Mushati, Lovemore Dumba, Lorraine Sherr, Constance Nyamukapa, and Simon Gregson. 2013. "Acceptability of conditions in a community-led cash transfer programme for orphaned and vulnerable children in Zimbabwe." *Health Policy and Planning* 29 (7):809-817. doi: 10.1093/heapol/czt060.

Vita, Febriany, Febriany Vita, Toyamah Nina, Toyamah Nina, Sodo Robert Justin, Sodo Robert Justin, Budiyati Sri, and Budiyati Sri. 2011. Qualitative Impact Study for PNPM Generasi and Pkh on the Provision and the

For peer review only - http://bmjopen.bmj.com/site/about/guidelines.xhtml

Utilization of Maternal and Child Health Services and Basic Education Services in the Provinces of West Java and East Nusa Tenggara. In *SMERU Research Institute*: SMERU Research Institute.

Zulu, J. M., J. Kinsman, C. Michelo, and A. K. Hurtig. 2013. "Developing the national community health assistant strategy in Zambia: a policy analysis." *Health Res Policy Syst* 11:24. doi: 10.1186/1478-4505-11-24.

Zulu, Joseph Mumba, John Kinsman, Charles Michelo, and Anna-Karin Hurtig. 2014. "Hope and despair: community health assistants' experiences of working in a rural district in Zambia." *Human Resources for Health* 12 (1):30. doi: 10.1186/1478-4491-12-30.

List of included other studies

Adamu, A. A., O. A. Uthman, M. A. Gadanya, O. O. Adetokunboh, and C. S. Wiysonge. 2019. "A multilevel analysis of the determinants of missed opportunities for vaccination among children attending primary healthcare facilities in Kano, Nigeria: Findings from the pre-implementation phase of a collaborative quality improvement programme." *PLoS One* 14 (7):e0218572. doi: 10.1371/journal.pone.0218572.

Adamu, Abdu Abdullahi. 2019. "Using quality improvement approach to address missed opportunities for vaccination in Kano Metropolis, Nigeria." PhD, Faculty of Medicine and Health Sciences, Stellenbosch University.

Alhassan, Robert Kaba. 2017. "Healthcare quality in Ghana: Improving healthcare quality and health worker motivation to promote sustainable health insurance." PhD, Faculty of Medicine (AMC-UvA), University of Amsterdam.

Banwat, ME., PW; Bupwatd, LA; Lar, AA; Apagu, and AI. Zoakah. 2014. "Effect of peer education on timeliness and completeness of routine immunization: An assessment in rural communities in North-Central Nigeria." *International Journal of Community Research* 3 (3):60-67.

Bekele, Abebe, Mengistu Kefale, and Mekonnen Tadesse. 2016. "Preliminary Assessment of the Implementation of the Health Services Extension Program: The case of Southern Ethiopia." *The Ethiopian Journal of Health Development* 22 (3).

Binyaruka, P., and J. Borghi. 2017. "Improving quality of care through payment for performance: examining effects on the availability and stock-out of essential medical commodities in Tanzania." *Trop Med Int Health* 22 (1):92-102. doi: 10.1111/tmi.12809.

Binyaruka, P., E. Patouillard, T. Powell-Jackson, G. Greco, O. Maestad, and J. Borghi. 2015. "Effect of Paying for Performance on Utilisation, Quality, and User Costs of Health Services in Tanzania: A Controlled Before and After Study." *PLoS One* 10 (8):e0135013. doi: 10.1371/journal.pone.0135013.

Binyaruka, P., B. Robberstad, G. Torsvik, and J. Borghi. 2018a. "Does payment for performance increase performance inequalities across health providers? A case study of Tanzania." *Health Policy Plan* 33 (9):1026-1036. doi: 10.1093/heapol/czy084.

Binyaruka, Peter, and Laura Anselmi. 2020. "Understanding efficiency and the effect of pay-for-performance across health facilities in Tanzania." *BMJ Global Health* 5 (5):e002326. doi: 10.1136/bmjgh-2020-002326.

Binyaruka, Peter, Bjarne Robberstad, Gaute Torsvik, and Josephine Borghi. 2018b. "Who benefits from increased service utilisation? Examining the distributional effects of payment for performance in Tanzania." *International Journal for Equity in Health* 17 (1):14. doi: 10.1186/s12939-018-0728-x.

Borghi, J., R. Little, P. Binyaruka, E. Patouillard, and A. Kuwawenaruwa. 2015. "In Tanzania, the many costs of pay-for-performance leave open to debate whether the strategy is cost-effective." *Health Aff (Millwood)* 34 (3):406-14. doi: 10.1377/hlthaff.2014.0608.

Borkum, Evan;, Anitha; Sivasankaran, Divya; Vohra, Swetha; Sridharan, Dana; Rotz, Lakshmi; Ramnakrishnan, Mercy; Manoranjini, Sukhmani; Sethi, and Anu. Rangarajan. 2015. "Evaluation of the Team-Based Goals and Performance-Based Incentives (TBGI) Intervention in Bihar: Findings from the 30-month Follow-up." *Mathematica Policy Research*.

Carmichael, Suzan L, Kala Mehta, Hina Raheel, Sridhar Srikantiah, Indrajit Chaudhuri, Shamik Trehan, Sunil Mohanty, Evan Borkum, Tanmay Mahapatra, Yingjie Weng, Rajani Kaimal, Anita Sivasankaran, Swetha Sridharan, Dana Rotz, Usha Kiran Tarigopula, Debarshi Bhattacharya, Yamini Atmavilas, Wolfgang Munar, Anu Rangarajan, and Gary L Darmstadt. 2019. "Effects of team-based goals and non-monetary incentives on front-

For peer review only - http://bmjopen.bmj.com/site/about/guidelines.xhtml

line health worker performance and maternal health behaviours: a cluster randomised controlled trial in Bihar, India." *BMJ Global Health* 4 (4):e001146. doi: 10.1136/bmjgh-2018-001146.

Cockcroft, A., N. Andersson, K. Omer, N. M. Ansari, A. Khan, U. U. Chaudhry, and U. Ansari. 2009. "One size does not fit all: local determinants of measles vaccination in four districts of Pakistan." *BMC Int Health Hum Rights* 9 Suppl 1 (Suppl 1):S4. doi: 10.1186/1472-698x-9-s1-s4.

Costa-i-Font, Joan and Parmar, Divya. 2017. Political Agency and Public Health Care: Evidence from India. In *CESifo Working Paper Series*. India: United Nationa University- UNU-WIDER.

Crea, Thomas M., Andrew D. Reynolds, Aakanksha Sinha, Jeffrey W. Eaton, Laura A. Robertson, Phyllis Mushati, Lovemore Dumba, Gideon Mavise, J. C. Makoni, Christina M. Schumacher, Constance A. Nyamukapa, and Simon Gregson. 2015. "Effects of cash transfers on Children's health and social protection in Sub-Saharan Africa: differences in outcomes based on orphan status and household assets." *BMC Public Health* 15 (1):511. doi: 10.1186/s12889-015-1857-4.

Doctor, H. V., and T. Dahiru. 2010. "Utilization of non-skilled birth attendants in northern Nigeria: a rough terrain to the health-related MDGs." *Afr J Reprod Health* 14 (2):37-45.

Doctor, H. V., S. E. Findley, G. Cometto, and G. Y. Afenyadu. 2013. "Awareness of critical danger signs of pregnancy and delivery, preparations for delivery, and utilization of skilled birth attendants in Nigeria." *J Health Care Poor Underserved* 24 (1):152-70. doi: 10.1353/hpu.2013.0032.

Doctor;, Henry V., Sally E. Findley;, Radheshyam Bairagi; and, and Tukur Dahiru. 2011. "Northern Nigeria Maternal, Newborn and Child Health Programme: Selected Analyses from Population-Based Baseline Survey." *The Open Demography Journal* 4:11-21.

Domek, G. J., I. L. Contreras-Roldan, E. J. Asturias, M. Bronsert, G. A. Bolaños Ventura, S. T. O'Leary, A. Kempe, and S. Bull. 2018. "Characteristics of mobile phone access and usage in rural and urban Guatemala: assessing feasibility of text message reminders to increase childhood immunizations." *Mhealth* 4:9. doi: 10.21037/mhealth.2018.03.05.

Domek, G. J., I. L. Contreras-Roldan, S. T. O'Leary, S. Bull, A. Furniss, A. Kempe, and E. J. Asturias. 2016. "SMS text message reminders to improve infant vaccination coverage in Guatemala: A pilot randomized controlled trial." *Vaccine* 34 (21):2437-2443. doi: 10.1016/j.vaccine.2016.03.065.

Domek, G. J., S. T. O'Leary, S. Bull, M. Bronsert, I. L. Contreras-Roldan, G. A. Bolaños Ventura, A. Kempe, and E. J. Asturias. 2018. "Measuring vaccine hesitancy: Field testing the WHO SAGE Working Group on Vaccine Hesitancy survey tool in Guatemala." *Vaccine* 36 (35):5273-5281. doi: 10.1016/j.vaccine.2018.07.046.

Findley, S. E., O. T. Uwemedimo, H. V. Doctor, C. Green, F. Adamu, and G. Y. Afenyadu. 2013. "Comparison of high- versus low-intensity community health worker intervention to promote newborn and child health in Northern Nigeria." *Int J Womens Health* 5:717-28. doi: 10.2147/ijwh.s49785.

Gibson, D. G., B. Ochieng, E. W. Kagucia, D. Obor, F. Odhiambo, K. L. O'Brien, and D. R. Feikin. 2015. "Individual level determinants for not receiving immunization, receiving immunization with delay, and being severely underimmunized among rural western Kenyan children." *Vaccine* 33 (48):6778-85. doi: 10.1016/j.vaccine.2015.10.021.

Grant, Carolyn, Dipty Nawal, Sai Mala Guntur, Manish Kumar, Indrajit Chaudhuri, Christine Galavotti, Tanmay Mahapatra, Kunal Ranjan, Gangesh Kumar, Sunil Mohanty, Mohammed Aftab Alam, Aritra Das, and Safia Jiwani. 2018. "'We pledge to improve the health of our entire community': Improving health worker motivation and performance in Bihar, India through teamwork, recognition, and non-financial incentives." *PLOS ONE* 13 (8):e0203265. doi: 10.1371/journal.pone.0203265.

Mirkuzie Woldie, Sudhakar Narayan Morankar, Garumma T. Feyissa, and Ronald Labonte, and David Sanders. 2015. "Coverage of child health services in rural districts of Ethiopia with the health services extension program." *Journal of Public Health and Epidemiology* 7 (7):223-231. doi: https://doi.org/10.5897/JPHE2015.0733.

Mitchell, Steven, Neil Andersson, Noor Mohammad Ansari, Khalid Omer, José Legorreta Soberanis, and Anne Cockcroft. 2009. "Equity and vaccine uptake: a cross-sectional study of measles vaccination in Lasbela District,

For peer review only - http://bmjopen.bmj.com/site/about/guidelines.xhtml

Pakistan." *BMC international health and human rights* 9 Suppl 1 (Suppl 1):S7-S7. doi: 10.1186/1472-698X-9-S1-S7.

Modi, D., R. Gopalan, S. Shah, S. Venkatraman, G. Desai, S. Desai, and P. Shah. 2015. "Development and formative evaluation of an innovative mHealth intervention for improving coverage of community-based maternal, newborn and child health services in rural areas of India." *Glob Health Action* 8:26769. doi: 10.3402/gha.v8.26769.

Modi, D., J. Patel, S. Desai, and P. Shah. 2016. "Accessing completeness of pregnancy, delivery, and death registration by Accredited Social Health Activists [ASHA] in an innovative mHealth project in the tribal areas of Gujarat: A cross-sectional study." *J Postgrad Med* 62 (3):170-2. doi: 10.4103/0022-3859.183168.

Modi, Dhiren, Somen Saha, Prakash Vaghela, Kapilkumar Dave, Ankit Anand, Shrey Desai, and Pankaj Shah. 2020. "Costing and Cost-Effectiveness of a Mobile Health Intervention (ImTeCHO) in Improving Infant Mortality in Tribal Areas of Gujarat, India: Cluster Randomized Controlled Trial." *JMIR Mhealth Uhealth* 8 (10):e17066. doi: 10.2196/17066.

Moore., Charity. 2008. Accessing Honduras' CCT Programme PRAF, Programa de Asignacion familiar: Expected and unexpected realities. Brazil: International Poverty Centre.

Nagar, Ruchit B. 2016. "Tying Community Engagement With Appropriate Technology At The Last Mile: A Cluster Randomized Trial To Determine The Effectiveness Of A Novel, Digital Pendant And Voice Reminder Platform On Increasing Infant Immunization Adherence Among Mothers In Rural Udaipur, India." School of Public Health, Yale University.

Negusse, H., E. McAuliffe, and M. MacLachlan. 2007. "Initial community perspectives on the Health Service Extension Programme in Welkait, Ethiopia." *Hum Resour Health* 5:21. doi: 10.1186/1478-4491-5-21.

Oduenyi, Chioma, Victor Ordu, and Ugo Okoli. 2019. "Assessing the operational effectiveness of a maternal and child health (MCH) conditional cash transfer pilot programme in Nigeria." *BMC Pregnancy and Childbirth* 19 (1):298. doi: 10.1186/s12884-019-2418-0.

Okeke, Edward, Peter Glick, Amalavoyal Chari, Isa Sadeeq Abubakar, Emma Pitchforth, Josephine Exley, Usman Bashir, Kun Gu, and Obinna Onwujekwe. 2016. "The effect of increasing the supply of skilled health providers on pregnancy and birth outcomes: evidence from the midwives service scheme in Nigeria." *BMC Health Services Research* 16 (1):425. doi: 10.1186/s12913-016-1688-8.

Onwujekwe, Obinna, Tim Ensor, Pamela Ogbozor, Chinyere Okeke, Uche Ezenwaka, Joseph P. Hicks, Enyi Etiaba, Benjamin Uzochukwu, Bassey Ebenso, and Tolib Mirzoev. 2020. "Was the Maternal Health Cash Transfer Programme in Nigeria Sustainable and Cost-Effective?" *Frontiers in public health* 8:582072-582072. doi: 10.3389/fpubh.2020.582072.

Padayachee, Thesandree;, Natasha; Chetty, Muzi; Matse, Tumelo; Mampe, and Helen Schneider. 2013. Progress in the establishment of ward based outreach teams: experiences in the North West Province. University of Western Cape.

Quayyum, Z., M. N. Khan, T. Quayyum, H. E. Nasreen, M. Chowdhury, and T. Ensor. 2013. ""Can community level interventions have an impact on equity and utilization of maternal health care" - evidence from rural Bangladesh." *Int J Equity Health* 12:22. doi: 10.1186/1475-9276-12-22.

Rahman, Mahfuzar, Fatema Tuz Jhohura, Sabuj Kanti Mistry, Tridib Roy Chowdhury, Tanveen Ishaque, Rasheduzzaman Shah, and Kaosar Afsana. 2015. "Assessing Community Based Improved Maternal Neonatal Child Survival (IMNCS) Program in Rural Bangladesh." *PLOS ONE* 10 (9):e0136898. doi: 10.1371/journal.pone.0136898.

Rangarajan;, Anu, Evan Borkum;, Swetha Sridharan;, Dana Rotz;, Mercy Manoranjini;, Seth Morgan;, Lalit Dandona;, Rakhi Dandona;, Priyanka Chaman;, and G. Anil Kumar. 2013. "Baseline Findings from the Ananya Evaluation." *Mathematica Policy Research Reports* 3e3c44fb11b84a07a3063c242.

Robertson, Laura, Phyllis Mushati, Jeffrey W. Eaton, Lorraine Sherr, Jeremiah C. Makoni, Morten Skovdal, Tom Crea, Gideon Mavise, Lovemore Dumba, Christina Schumacher, Shungu Munyati, Constance Nyamukapa, and Simon Gregson. 2012. "Household-based cash transfer targeting strategies in Zimbabwe: are we reaching the

For peer review only - http://bmjopen.bmj.com/site/about/guidelines.xhtml

most vulnerable children?" *Social science & medicine (1982)* 75 (12):2503-2508. doi: 10.1016/j.socscimed.2012.09.031.

Robinson, Victor C. 2006. Summary Report Women's TheRuralMaintenance Program: ACashforWorkProgram.

USAID. 2008. Essential Services for Health in Ethiopia: Final report November 2003- September 2008. Ethiopia: USAID.

UNICEF. 2010. Evaluation report of the community health strategy implementation in Kenya. Division of Community Health Services

Venkat, Preethi. 2016. "Intermediate Assessment Of The Khushi Baby Crct: Implementation Of A Novel Mhealth Solution For Vaccination Record Keeping In Rural Udaipur, Rajasthan, India."

Wakadha, H., S. Chandir, E. V. Were, A. Rubin, D. Obor, O. S. Levine, D. G. Gibson, F. Odhiambo, K. F. Laserson, and D. R. Feikin. 2013. "The feasibility of using mobile-phone based SMS reminders and conditional cash transfers to improve timely immunization in rural Kenya." *Vaccine* 31 (6):987-93. doi: 10.1016/j.vaccine.2012.11.093.

For peer review only

Appendix 6: Data extraction tools

Quantitative data extraction tool

1. VARIABLE LABEL	EXPLANATION
Study ID	This is the study ID - it should match the study ID from the Outcome Mapping Sheet (e.g., SC-SR_1)
Estimate ID	The estimate ID will provide a specific number for each effect size extracted and should include the original study number, underscore, then the unique ID number (e.g., SC-SR1_1, SC-SR1_2 and so on)
Author	For 1 author: leading author last name (e.g., Gomez) For 2 authors: both author last names with ampersand in between (e.g., Smith & Bahn) For 3 or more authors: leading author last name followed by et al. (e.g., Gupta et al.)
Year	Year published
Design	0=Experimental Design (e.g., RCT), 1=Quasi-Experimental Design
How Counterfactual is Chosen?	Free text (e.g., random control trial, propensity score matching, etc) - Multiple codes are ok
Analysis type for this effect size	Free text, what type of analysis was used (Regression, 2SLS, ANCOVA, etc.)- Multiple codes are ok
Country	Country of intervention
Region	Region/continent of intervention
Estimate Type	Type of data for this effect size: 1 = Continuous - means and SDs, 2 = Continuous - mean difference and SD, 3 = Dichotomous outcome - proportions, 4 = Regression data - dichotomous outcome (e.g., logistic regression) 5 = Regression data - continuous outcome (e.g., linear regression)
Comparison	1=No intervention (service delivery as usual), 2=Other intervention, 3=Pipeline (wait-list) control (still service delivery as usual)
Describe Comparison Group	If answer above is (1) no intervention, type N/A, if (2) Other Intervention, list what intervention the control group is receiving, if (3) Pipeline control, report when the control group will receive the intervention in relation to the treatment group (e.g., one year later)
Subgroup	Is this analysis of a subgroup? 0=no, 1=yes

For peer review only - http://bmjopen.bmj.com/site/about/guidelines.xhtml

If yes to subgroup, describe	Free text, describe the subgoup if applicable (e.g., boys, girls). If no subgroup, type N/A
Source	Note the page number, table number, column, and row you used to extract the data
Treatment Effect	1=Intention to Treat (ITT), 2=Average Treatment Effect on the Treated (ATET), 3=Average Treatment Effect (ATE) 4 = Local Average Treatment Effect (LATE)
Intervention	Free text, what is the intervention
Engagement as intervention	1=yes, 0=No
Engagement as intervention: developing community buy-in	1=yes, 0=No
Engagement as intervention: creation of new cadres or health committees	1=yes, 0=No
Engagement as intervention: both	1=yes, 0=No
Engagement in design	1=yes, 0=No
Engagement in design: community decision makinh	1=yes, 0=No
Engagement in design: community feedback	1=yes, 0=No
Engagement in implementation	1=yes, 0=No
Engagement in implementation: governance and decisions	1=yes, 0=No
Engagement in implementation: provision of resources	1=yes, 0=No
Multiple engagement types	1=yes, 0=No
Engagement in design and engagement as intervention	1=yes, 0=No
Engagement in design and implementation	1=yes, 0=No
Engagement in implementation and engagement as intervention	1=yes, 0=No
Exposure to intervention (in months)	How long is the intervention exposure itself?
Evaluation period (in months)	The total number of months elapsed between offering an intervention and the point at which an outcome measure is taken post intervention, or as a follow-up measurement. If less than one month, use decimals (e.g., one week would be .25)

For peer review only - http://bmjopen.bmj.com/site/about/guidelines.xhtml

Post-intervention or change from baseline?	0 = Post-intervention, 1 = Change from baseline
Source of the Outcome data (only use for outcomes in category JAA)	1=Immuniation Card, 2=Recall, 3=Combination of both immunisation card and recall, 4=Health Admin Data, 5=N/A
Author definition of outcome	Free text - How does the author define the outcome?
**OUTCOME CODES**	
**Thinking and Feeling**	Code 1 under any applicable columns. See OUTCOME GUIDANCE document for further explanations.
**Social Processes**	Code 1 under any applicable columns. See OUTCOME GUIDANCE document for further explanations.
**Readiness to Vaccinate**	Code 1 under any applicable columns. See OUTCOME GUIDANCE document for further explanations.
**Practical Factors**	Code 1 under any applicable columns. See OUTCOME GUIDANCE document for further explanations.
**Community Health Workers**	Code 1 under any applicable columns. See OUTCOME GUIDANCE document for further explanations.
**Vaccinators**	Code 1 under any applicable columns. See OUTCOME GUIDANCE document for further explanations.
**IAC Admins**	Code 1 under any applicable columns. See OUTCOME GUIDANCE document for further explanations.
**Health information systems**	Code 1 under any applicable columns. See OUTCOME GUIDANCE document for further explanations.
**Vaccine Availability**	Code 1 under any applicable columns. See OUTCOME GUIDANCE document for further explanations.
**Resources**	Code 1 under any applicable columns. See OUTCOME GUIDANCE document for further explanations.
**Vaccination coverage**	Code 1 under any applicable columns. See OUTCOME GUIDANCE document for further explanations.
**Health Outcomes**	Code 1 under any applicable columns. See OUTCOME GUIDANCE document for further explanations.
**EFFECT SIZE DATA EXTRACTION**	
Reverse Sign (i.e., decrease is good)	Record 0='no' if an increase is good, record 1='yes' if a decrease is good and the sign needs to be reversed.
Unit of analysis	What is the unit of analysis? UOA for this effect size: 1= Individual, 2= Household, 3= Group (e.g. community

For peer review only - http://bmjopen.bmj.com/site/about/guidelines.xhtml

	organisation), 4= Health Center, 5 = Village, 6 = Other, 7 = Not clear
mean_t	Outcome mean for the treatment group
sd_t	Outcome standard deviation for treatment group
mean_c	Outcome mean for the comparison group
sd_c	Outcome standard deviation for control group
mean_overall_diff	Overall mean difference (treatment - control)
diff se	Standard error of the overall mean difference
Diff _t	t-statistic of mean difference
Odds ratio	Odds ratio reported in the study
OR_se	Odds ratio standard error reported in the study
Risk ratio	Risk ratio reported in study
RR_se	Risk ratio standard error
reg_coeff	Report the regression coefficient of the treatment effect
reg_SE	Report the associated standard error of the regression coefficient.
reg_t	Report the associated t statistic of the effect size (coefficient/SE)
Exact p value	Exact p value if given, if not, record as written in the manuscript (e.g., p < .001, or p > .05)
clust_t	Number of clusters - treatment group
clust_c	Number of clusters - control group
clust_T	Number of clusters - total sample
n_t	Sample size - treatment group
n_c	Sample size - control group
n_T	Sample size - total sample
Does the sample size need to be adjusted (if so complete column CL)?	Code as ‘yes’ or no’
periods (1 if cross sectional)	Record how many periods of evaluation there are (e.g., cross section is 1, panel data with 3 measurements is 3)
Treatment Variable	Record the treatment variable as written in the model (e.g., the variable name the author uses, such as ("Intervention x Time")
dataset	Record if data comes from an identified dataset

For peer review only - http://bmjopen.bmj.com/site/about/guidelines.xhtml

coder	Record your name
Notes	Record any notes important for the team
n_T_revised	THIS IS FOR SENIOR QUANT LEAD TO FILL OUT
sp	THIS IS FOR SENIOR QUANT LEAD TO FILL OUT
d	THIS IS FOR SENIOR QUANT LEAD TO FILL OUT
g	THIS IS FOR SENIOR QUANT LEAD TO FILL OUT
var(d)	THIS IS FOR SENIOR QUANT LEAD TO FILL OUT
se(d)	THIS IS FOR SENIOR QUANT LEAD TO FILL OUT
CI_l	THIS IS FOR SENIOR QUANT LEAD TO FILL OUT
CI_u	THIS IS FOR SENIOR QUANT LEAD TO FILL OUT
remove	THIS IS FOR PROJECT MANAGER TO FILL OUT
Formula Used	THIS IS FOR SENIOR QUANT LEAD TO FILL OUT
yi_1	THIS IS FOR SENIOR QUANT LEAD TO FILL OUT
yi_rev	THIS IS FOR SENIOR QUANT LEAD TO FILL OUT
yi	THIS IS FOR SENIOR QUANT LEAD TO FILL OUT
vi	THIS IS FOR SENIOR QUANT LEAD TO FILL OUT
wi	THIS IS FOR SENIOR QUANT LEAD TO FILL OUT
ywi	THIS IS FOR SENIOR QUANT LEAD TO FILL OUT
95ci_lower	THIS IS FOR SENIOR QUANT LEAD TO FILL OUT
95ci_upper	THIS IS FOR SENIOR QUANT LEAD TO FILL OUT
cilow_3sf	THIS IS FOR SENIOR QUANT LEAD TO FILL OUT
cihigh_3sf	THIS IS FOR SENIOR QUANT LEAD TO FILL OUT
ci	THIS IS FOR SENIOR QUANT LEAD TO FILL OUT
wb_yi	THIS IS FOR SENIOR QUANT LEAD TO FILL OUT
Checked	THIS IS FOR EFFECT SIZE RELIABILITY CHECKER TO FILL OUT
ROB Category	THIS IS FOR SENIOR QUANT LEAD OR PM TO FILL OUT

For peer review only

For peer review only - http://bmjopen.bmj.com/site/about/guidelines.xhtml

Qualitative data extraction tool

Name	Description
Causal mechanism	This set of codes describes the causal mechanisms observed or hypothesized
Barriers to immunisation	Select any state reasons that people were not immunised. Try to use previously added codes to reduce possible redundancies, but add codes if needed.
Demand side barriers	Barriers related to low demand for immunisations
Fear	Low demand for immunisation due to fear of side effects or other factors
Financial	Select the financial reason that people were not immunised Add sub-codes as needed.
Cost of immunisation	People were not immunised due to the cost of the immunisation Do not add sub-codes
Cost of transport	People were not immunised due to the cost of transport.
Opportunity costs	People were not immunised due to opportunity costs
Knowledge of Immunisation Schedule	Low demand for immunisation due to limited knowledge of its importance. This includes instances when the person simply does not feel that immunisations are important (regardless of "book knowledge")
Logistics or distance	Barriers related to logistics or distance. For example, it is too far away, I need to arrange for XXX to happen while I am gone...
Self-efficacy	Issues related to not feeling empowered to get vaccinated. This includes not knowing how to go about getting vaccinations. For example, not knowing when or where to get vaccinated
Social norms	Low demand for immunisation due to social pressures and social norms
Understanding of importance	Low demand for immunisation due to limited knowledge of its importance. This includes instances when the person simply does not feel that immunisations are important (regardless of "book knowledge")
Supply side barriers	Limitations to supply that cause problems for immunisation
Availability of services	Services may not be physically present. This includes clinics being closed.
Human resources	Limited human resources may reduce the availability of immunisations. Ex: Appropriate staff were not present to provide the injection
Lack of Infrastructure or supplies	Use code if the lack of immunisation was because there were no drugs, vaccines, cold chain or other items related to physical infrastructure of the health facility

For peer review only - http://bmjopen.bmj.com/site/about/guidelines.xhtml

Poor quality services	Immunisations are not common because there is low quality of service. Providers may not be cooperative, slow, unprofessional/rude, unempathetic, abusive, or inconsistent.
Lack of accountability	Use code for text referring to issues related to accountability, corruption, governance and transparency in providing services.
Lack of Motivation	Use if for text indicating that the health workers are not motivated to perform their duties due to any reason like lack of monetary incentives, recognition, low job satisfaction and other issues.
Baseline conditions and descriptive characteristics	Discussions of the baseline conditions and descriptive characteristics of the region or population in which the intervention took place. This does not necessarily come from a baseline survey, but can be through other sources. Do not add sub-codes
Health systems context	Discussion of the health system functioning and context Example: The clinic served a population of 1,000 people Do not add sub-codes
Immunisation rates	Discussion of baseline immunisation rates. Example: Immunisation rates were low at baseline. Do not add sub-codes
Political context	Discussion of the political context Example: Political unrest made the intervention difficult to implement. Do not add sub-codes
Socioeconomic status and demographics	Discussions of SES and demographics at baseline. Example: The community was mostly farmers. Population size Do not add sub-codes
Facilitators of immunisation	Select any state reasons that people were immunised. Try to use previously added codes to reduce possible redundancies, but add codes if needed.
Demand Side Facilitators	
Favourable population SES characteristics	For instance those from a higher wealth quantile, urban areas or mothers with higher educational qualifications are more likely to get their children immunised. Do not add sub-codes.
Knowledge of immunisation schedule	Use this code if caregivers are aware of all immunisations in the schedule and know which vaccination is due when. Do not add sub-codes.
Lack of fear	Text indicating that there was no fear or vaccine hesitancy. Do not add sub-codes
Lack of financial constraints	Caregivers can afford the cost of immunisation, cost of transportation to visit healthcare facilities, taking children for immunisation does not come at a high opportunity cost for the caregiver. Do not add sub-codes.

For peer review only - http://bmjopen.bmj.com/site/about/guidelines.xhtml

	Lack of logistics or distance related challenges	For instance, caregivers were able to access health services because they lived within a 5km radius of a healthcare facility or they received outreach services regularly. Do not add sub-codes.
	Self-efficacy	Caregivers feel empowered to get their children vaccinated. For example, they know when and where to get vaccinated. Do not add sub-codes.
	Social norms	The social environment owing to prevailing social pressures and norms is encouraging towards child immunisation. For instance, the mother in law in the household or the traditional/religious leaders in the community support immunisation of children. Do not add sub-codes.
	Understanding of importance	Caregivers are aware about the importance of immunisation and are willing to get their children vaccinated. For example, caregivers are able to correctly identify vaccine preventable illness and therefore, more likely to get their children immunised. Do not add sub-codes.
	Supply-side facilitators	
	Availability of infrastructure or supplies	
	Good quality of services	
	High accountability	
	High motivation	
	Human resources	
	Availability of services	
Impacts		What impacts were ultimately achieved? Use this code for general impacts evaluated through the IE that do not fall into one of the other groups. Do not add sub-codes
	Descriptive impacts	Include authors' descriptions of impacts that were not directly measured through the IE. Ex: There was generally an increase over time in most sites
	Equity considerations	If impacts on marginalized groups are considered, select the type of marginalized group considered. Do not add sub-codes
	Ethnic minority	Impacts on ethnic minorities Do not add sub-codes
	Other	Impacts on other marginalized groups, like scheduled castes in India. Do not add sub-codes
	Religious minority	Impacts on religious minorities Do not add sub-codes
	Women	Impacts on women Do not add sub-codes

For peer review only - http://bmjopen.bmj.com/site/about/guidelines.xhtml

Long term impacts	Impact measured over a timeframe of more than 5 years from the end of the intervention Do not add additional sub-codes
Unintended impacts	Only use this sub-code if the impact is stated as unintended. Do not add sub-codes
Reasons for project success or failure	Select text in which authors discuss the reasons for the success or failure of the project. This could be related to intervention design, implementation, or the research approach. Example: The intervention was not successful due to a lack of political will. Do not add sub-codes
Failure	
Implementation or scale-up challenges	
Competing priorities of health workers	
Other delays, disruptions or implementation variability	
Payment delays	
Intervention	
Design - Engagement	
Design - other	
Duration, frequency and exposure or reach	
Not accounting for existing constraints or uncontrollable contextual trends	
Caregiver competing priorities	
Civil unrest or political instability or natural calamity	
Fear of AEFI	
Health system issues	
Health service access or availability	
Poor quality of service or infrastructure	Also includes issues related to health worker demotivation, recruitment or retention.

For peer review only
For peer review only - http://bmjopen.bmj.com/site/about/guidelines.xhtml

High baseline coverage	The interventions are not geared to/designed to achieve last mile coverage or breakthrough stagnating rates.
Lack of awareness or understanding	
Migration	
Resource constraints or scarcity	Infrastructure, electricity, cellular network, etc.
Social norms	
Wider socio-economic or health-related progress	
Study design	
Contamination	
Other methodological shortcomings	
Mixed results	
Success	
Existing or changing favourable factors or contextual trends	
Health system enablers	
Good quality services and infrastructure	Includes enablers like good outreach, service monitoring, etc.
Health service access and availability	
Self efficacy, social norms and awareness	
Socio-economic factors	
Implementation improvements	
Intervention features	
Community engagement	
Behaviour change communication	
Community dialogues	

For peer review only

For peer review only - http://bmjopen.bmj.com/site/about/guidelines.xhtml

	Community involvement in planning and implementation	
	Incentives	
	Needs assessments, pilots or stakeholder consultations	
	Other types of engagement	
	Customisation to local context	
	Health system integration and organisational structure	
	Health worker training	
	Intervention duration, dose and exposure	
	Leadership and supportive supervision	
	Other design features	
	Positive participant or beneficiary views of intervention	
Theory of change		Information related to the ToC. Do not code here, but use sub-codes. Do not add sub-codes.
Assumptions		when discussing a causal chain mechanism, we often make assumptions about what will happen. These assumptions are often implicit. We may print materials with the assumption that people will be able to read them. We may train people with the assumption they speak our language. When these assumptions are stated, use the sub-codes to indicate if these assumptions were validated or not. Do not add sub-codes
Incorrect assumption		This indicates that there was an assumption in the causal chain mechanism and the assumption was not correct. For example, a study assumed that increasing supply would increase immunisations. Here, we are assuming that people want immunisations and that low supply is the problem. If instead, we find that people did not want the immunisations to begin with, the assumption was incorrect and would be indicated here. Do not add sub-codes

For peer review only - http://bmjopen.bmj.com/site/about/guidelines.xhtml

Valid assumption	this indicates that there was an assumption in the causal chain mechanism and the assumption was valid. For example, a study assumed that increasing supply would increase immunisations. Here, we are assuming that people want immunisations and that low supply is the problem. If this proves to be true, the text should be selected here. Do not add sub-codes
Causal chain mechanisms	Descriptions of how change is expected to occur. These causal chains are often represented as flow charts, or at least they can be. Text that reflects a causal chain mechanism will discuss expected actions and reactions. “We did this and we expected that.” Do not add sub-codes
Conclusions	Authors conclusions. Any summary of the take-home message of the article. Tends to be "this worked," "this did not work," "in conclusion." Do not add sub-codes
Cost	Discussions of costs, including the costs of the intervention and any form of cost-analysis. Only select key "take-home message." Do not add sub-codes
Data source	For anything related to causal mechanisms, uptake, and uptake and fidelity challenges, indicate what data source provided this information. Do not add additional sub-codes
Author notes or experiences	
FGDs	
Implementer interpretations or experiences	
Individual interviews	
Literature Review	Use this code if the text alludes to existing evidence on the subject
Observations	If the data comes from direct observations. This must be enumerators looking at and noting the state of something. If it is simply "general impressions," use the notes/interpretations or experience codes. Do not add sub-codes
Intervention description	This set of codes describes the intervention
Beneficiary selection	Select relevant information regarding how beneficiaries were selected for the intervention. Note: This is how beneficiaries of the intervention were selected, NOT a research sampling design or how research participants were selected. Do not add sub-codes
Community involvement	Description of how the community was involved in beneficiary selection. Example: A community council decided who was eligible. Do not add sub-codes
Equity considerations	Descriptions of efforts to ensure beneficiary selection was fair and equitable. Example: There was a quota to ensure adequate representation for ethnic minorities Do not add sub-groups

For peer review only - http://bmjopen.bmj.com/site/about/guidelines.xhtml

Other selection	Other descriptions of the selection process and eligibility criteria Do not add sub-codes
Researcher involvement	Description of the researcher involvement in selection, including selection criteria designed for academic analysis Example: If only those who were involved in and RCT received the intervention, then the sampling frame for the RCT would also be how beneficiaries for the intervention were selected. Do not add sub-codes
Community engagement	This set of codes describes community engagement in the interventions
Community mobilization	Select the relevant reference to community mobilization, regardless of how this phrase is used Do not add sub-codes
Absent	Statements that there was no effort at community mobilisation Example: A challenge was that community members were not aware of the intervention activities. Do not add sub-codes
Definitions of community mobilization	The author's definition for community mobilisation Do not add sub-codes
Present - with trust building	Descriptions of community engagement that was done in such a way as to establish trust and buy-in. Example: A local organisation was recruited to inform community members of the intervention and explain its importance. Note: expect significant double coding with "Developing community buy-in, Present" Do not add sub-codes
Present - without trust building	Descriptions of community mobilization that was done without establishing trust and buy-in. Example: Simply informing the community of an intervention Do not add sub-codes
Inclusion criteria	Select the relevant reason this intervention / activity was included in the SR. Inclusion criteria into the research project should fall under "sampling frame." Do not use main code, only use sub-codes Do not add sub-codes
Community engagement as the intervention	Select the way in which community engagement was used as the intervention itself Do not add sub-codes
Developing community buy-in	Descriptions of activities whose primary purpose was the establishment of community buy-in / trust Do not add sub codes
Absent	Explicit statements that efforts to establish community buy-in / trust were not made should be assigned this code Example: Authors state that the intervention proceeded without engaging the community Do not add sub-codes
Present	If there were activities whose primary purpose was to establish community buy in or trust, select this code Example: Meetings with local leaders to get their support Note: expect significant double coding with "community mobilization, present - with trust building" Do not add sub-codes
New cadres	Description of the development of new cadres of community based structures or systems for health outreach Examples: Developing community health workers, health volunteers, or the establishment of committees Do not add sub-codes

For peer review only - http://bmjopen.bmj.com/site/about/guidelines.xhtml

Community engagement in designing the intervention	Select the type of community engagement that was sought before on the design of the intervention Do not add sub-codes
Absent	The community was not given the opportunity to provide feedback or make decisions on the design of the intervention Example: Explicit statements that the community was not consulted on the design of the intervention Do not add sub-codes
Community decision making	The community was given the opportunity to make decisions regarding the design of an intervention. This could vary from taking iterative feedback from the community to consensus building on the design of the intervention to the design being community led
Absent	The community did not have decision making power. Example: The intervention design was established a priori and not subject to change Do not add sub-codes
Community led	The community had the ultimate decision-making power and / or the intervention design was community led. Example: The community identified which barriers would be targeted and developed an action plan Do not add sub-codes
Partial	The community had some decision-making power Example: Decisions were made through collaboration between the community, implementers, and / or researchers Do not add sub-codes
Community feedback	The community was given the opportunity to provide feedback on the design of the intervention
Formative evaluation or stakeholder consultation	
Needs assessment	
Pilot	
There was a previous pilot	There was a pilot in which it is explicitly stated that community feedback was taken Do not add sub-codes
This was a pilot	The present study is a pilot in which community feedback is taken
Community engagement in implementation	Communities had some opportunity to affect the implementation of the intervention. For example, community is required to spend resources on building health infrastructure and decides whether the intervention will be implemented in their community or those where community members come together to form governance structures such as health committees . OR The community has some responsibility for making decisions regarding the delivery of an intervention. Do not add sub-codes

For peer review only - http://bmjopen.bmj.com/site/about/guidelines.xhtml

	Governance and decisions	
	Absent	Explicit statements that the community did not have decision making power. Example: Statements that the community was excluded from implementation. This may be stated as a problem during the discussion. Do not add sub-codes
	Present	The community has some form of decision making in the implementation of the intervention. This could include a community led governance structure. Example: There was a community group monitoring the implementation of the intervention. Do not add-sub codes
	Provision of resources	The community provides resources during the implementation Examples: Community provides building materials Do not add sub-codes
Component 1		Each unique component should be coded separately. A unique component is defined as the set of activates that are dependent on one another. Activities within separate components are not dependent on one another. Select the depth of engagement for the initial description of the component but code all subsequent activities to the main code. Example: A sticker based reminder and a digital reminder could each be implemented independently and would be separate components Do not add sub-codes
	Does not include engagement	Descriptions of interventions that involved no community engagement Example: The production of cold chain transport of vaccines Do not add sub-codes
	Includes some engagement	Description of activities that involved some community engagement Example: Health clinics holding vaccination days and posting fliers Do not add sub-codes
	Primary focus is engagement	Description of activities whose primary focus was community engagement Example: The establishment of community health councils Do not add sub-codes
Component 10		
	Does not include engagement	
	Includes some engagement	
	Primary focus is engagement	
Component 2		
	Does not include engagement	
	Includes some engagement	
	Primary focus is engagement	

For peer review only - http://bmjopen.bmj.com/site/about/guidelines.xhtml

Component 3

Does not include engagement

Includes some engagement

Primary focus is engagement

Component 4

Does not include engagement

Includes some engagement

Primary focus is engagement

Component 5

Does not include engagement

Includes some engagement

Primary focus is engagement

Component 6

Does not include engagement

Includes some engagement

Primary focus is engagement

Component 7

Does not include engagement

Includes some engagement

Primary focus is engagement

Component 8

Does not include engagement

Includes some engagement

Primary focus is engagement

Component 9

Does not include engagement

For peer review only - http://bmjopen.bmj.com/site/about/guidelines.xhtml

Includes some engagement	
Primary focus is engagement	
Future directions for implementation	Discussion of next steps, how the program could be re-designed, or suggestions for the future Do not add sub-codes
Intervention exposure or reach	Discussions of the proportion of the eligible population that the intervention tried to reach. For example, the number of people who received the video (regardless of whether it was actually watched). Do not add sub-codes
Objective	Statement of the goal, objective, or target of the intervention Do not add sub-codes
Participant views of the intervention	Text related to how participants perceived the intervention
Negative views	Participants did not view the intervention positively
Positive views	Participants viewed the intervention positively
Personnel implementing the program	Description of who implemented the program Do not add sub-codes
Target group	Description of the target group for this program Note: This could be different from beneficiary selection if (for example) fathers were provided with information to get them to have their wives immunise their children. Do not add additional sub-codes
Uptake	Program uptake is defined as initial engagement with the intervention. This could be attending the first meeting, or expressing interest. Use sub-codes when appropriate and only primary code when the text does not fall into the other two. Do not add additional sub-codes
Assessment of uptake	Description of how uptake was assessed Example: Attendance sheets were used to collect information on attendance at the first meeting. Do not add sub-codes Do not add sub-codes
Description of uptake	Description of uptake Example: Uptake was high, with 80% of eligible women participating in the intervention Do not add sub-codes
Research design	This set of codes describes the research design and provides information on internal and external validity. Do not add sub-codes
Author discussion of external validity	Any explicit discussion by authors Do not add sub-codes
Conflict of interest	Discussion of conflicts of interests. This could include discussions related to the independence of evaluators and data collectors from the implementers and donors. Select the relevant code indicating presence / absence of a conflict Do not add sub-codes Do not add sub-codes

For peer review only

For peer review only - http://bmjopen.bmj.com/site/about/guidelines.xhtml

No	Select any statement directly indicating that there was no conflict of interest Example: The data collection team was hired as external contractors Do not add sub-codes
Unclear	Select any text that makes a potential conflict of interest unclear Do not add sub-codes
Yes	Select any text that indicates a clear conflict of interest Example: The implementers were the data collectors Do not add sub-codes
Data collection	Discussion of data collection. Use main code for general discussions and only use sub-codes as appropriate Do not add sub-codes
Response accuracy	Any measure or discussion of response accuracy Example: We expect that some of our findings may be related to social desirability bias Do not add sub-codes
Retrospective	Text indicating that data collection was retrospective. Include discussion of challenges this may have caused Do not add additional sub-codes
Sampling frame	Descriptions of the sampling frame. Only select text related to how people were ultimately enrolled / selected. Text related to geographic distribution of villages, the method of randomisation (e.g. computer generated vs. paper based), and other details about the preparation for selection is not needed. Example: Women of child bearing age were selected when they presented at the clinic. Do not add sub-codes
Data limitations	Discussions of the limitations to the data that pose challenges (eg. we could not analyse X because of Y) Do not add additional sub-codes
Ethical approval	Statement of ethical approval Do not add sub-codes
Future directions for research	Descriptions of future directions for research Do not add sub-codes
Other limitations	Text related to limitations in the research design that are not data related. Example: Failure in randomisation Example: Failure in randomisation Do not add sub-codes
Uptake and fidelity challenges	This set of codes describes challenges with uptake and fidelity. Most include sub-codes titled "present" and "absent." In each case, present reflects a statement that this challenge occurred and absent reflects a statement that this challenge was not encountered. Add sub-codes if needed
Adherence	Discussion of people (not) completing the intervention activities (eg. stopped attending training sessions). Could involve discussions of variability in engagement with the intervention or discussions of continued engagement with the intervention. Do not add sub-codes
Absent	There were not challenges related to adherence. ie adherence was high

For peer review only - http://bmjopen.bmj.com/site/about/guidelines.xhtml

Present	There were problems with adherence. ie adherence was low
Administrative	Challenges related to record keeping, monitoring, and other administrative activities Do not add sub-codes
Absent	There were not administrative challenges. Example: All paperwork was conducted quickly and on time
Present	There were administrative challenges Ex: Paperwork was finished late and this delayed implementation
Attrition in the research	People did not complete the research study (eg. did not respond to all rounds of data collection Do not add sub-codes
Absent	There was not attrition in the research study. ie attrition was low
Present	There was significant attrition in the research study. ie attrition was high
Budget	Challenges due to budget limitations Do not add sub-codes
Budget limitations in the intervention	The intervention could not be implemented in a desired way due to budget limitations Do not add sub-codes
Absent	There were not budgetary limitations to the intervention. Ex: Due to the generous funding of our donor, we were able to.....
Present	There were budgetary limitations to the intervention Ex: We had planned X, but due to budget limitations has to Y
Budget limitations in the research study	The research study could not be implemented as desired due to budget limitations Do not add sub-codes
Absent	There were not budgetary limitations to the research study Ex: Due to the generous funding of our donor, we were able to ......
Present	There were budgetary constraints in the research study Ex: We were not able to collect X data due to limited budget
Contamination	Select the appropriate type of contamination described Do not add sub-codes
Contamination between intervention and control	The control group was incidentally exposed to the intervention. Example: Nearby villages were aware of education campaigns conducted.
Absent	Explicit statements that this contamination did not occur Do not add sub-codes
Present	Contamination between intervention and control occurred
Contamination by other programs	Other ongoing programs may have affected results Do not add additional sub-codes
Absent	There was not contamination by other programs Ex: No other similar programs were functioning in the area
Present	There was contamination by other programs Ex: The Red Cross was conducting a similar program in control villages at the time of our intervention

For peer review only - http://bmjopen.bmj.com/site/about/guidelines.xhtml

Hawthorn effects	Any description of the change in behaviour (real or reported) that is the result of people knowing they are being observed, without any real intent to adopt or maintain these behaviours. Example: Respondents in the UCT group may have been affected by the evaluation process: community awareness about the aims of the project could have affected actual or reported behaviours. Do not add sub-codes
Absent	Hawthorn effects were not observed or expected Ex: By using an enumeration team that was unaffiliated with the intervention, we expect to have reduced the chances that people adjusted their behaviour simply due to the enumerator's presence
Present	Hawthorn effects were observed or expected Ex: Due to significant marketing around the desirability of certain behaviours, people may have changed how they acted in front of enumerators without actually adopting certain behaviours when they were not observed.
Low implementation fidelity	Deviations in implementation of the intervention from what was planned. Note: this must be from what was planned, not simply what would be considered desirable Do not add sub-codes
Absent	Fidelity challenges were not present. The intervention was implemented as intended. Ex: The intervention was implemented as intended
Present	The intervention was not implemented as intended. Ex: Due to confusion among facilitators, some changed the order in which material was presented.
Mobilization	Challenges related to mobilizing people to participate Do not add sub-codes
Mobilizing in the intervention	People were not willing to engage with the intervention Do not add sub-codes
Absent	Low participation was not a challenge observed. ie participation was high.
Present	The challenge of low participation was encountered. ie participation was low
Mobilizing in the research	Challenges getting those who participated in the intervention to join the research project. (eg. refuse to be interviewed) Do not add sub-codes
Absent	Low participation was not a challenge observed. ie participation was high.
Present	The challenge of low participation was encountered. ie participation was low
Other Challenges	

For peer review only - http://bmjopen.bmj.com/site/about/guidelines.xhtml

Cost data extraction tool

Study Characteristics	Variable Label	Explanation	Source
**Coder**			
Study ID	Study ID	This is the study ID - it should match the study ID from the Outcome Mapping Sheet (e.g., SC-SR_1)	Data extraction tool_immunisation
	Study ID Component		
Year	Year	Year published	Data extraction tool_immunisation
Design	Design	0=Experimental Design (e.g., RCT), 1=Quasi-Experimental Design	Data extraction tool_immunisation
Country	Country	Country of intervention	Data extraction tool_immunisation
Study Link			
Which Vaccines were studied?			
How many vaccine doses were delivered?			
Source of info about vaccines			
Outcomes	[Source from CEA Inventory]		
Target population			
Implementing partners		What organization(s) carried out the intervention? [Please list names of organization(s) that implemented the program, e.g. IRC, Government of Uganda. Etc)]	Studies
Analytical perspective		Was the perspective of the costing stated explicitly? [Yes, No]. Analytical perspective is the choice of who has standing in the costing and determines whose costs and benefits will be counted. Perspective should be stated explicitly in the costing, so here it is ok to do a kw search for "perspective" to check	Studies
Analytical perspective		What is the analytical perspective of the costing (i.e. donor, financial cost, economic or social perspective).	Studies
Treatment arms		How many treatment arms were included in the study [# of treatment arms]	
Comparison	Comparison	1=No intervention (service delivery as usual), 2=Other intervention, 3=Pipeline (wait-list) control (still service delivery as usual)	Data extraction tool_immunisation
Describe Comparison Group	Describe Comparison Group	If answer above is (1) no intervention, type N/A, if (2) Other Intervention, list what intervention the control group is receiving, if (3) Pipeline control, report when the control group will receive the intervention in relation to the treatment group (e.g., one year later)	Data extraction tool_immunisation
Subgroup	Subgroup	Is this analysis of a subgroup? 0=no, 1=yes	Data extraction tool_immunisation

For peer review only - http://bmjopen.bmj.com/site/about/guidelines.xhtml

If yes to subgroup, describe	If yes to subgroup, describe	Free text, describe the subgoup if applicable (e.g., boys, girls). If no subgroup, type N/A	Data extraction tool_immunisation
Source	Source	Note the page number, table number, column, and row you used to extract the data	Data extraction tool_immunisation
Treatment Effect	Treatment Effect	1=Intention to Treat (ITT), 2=Average Treatment Effect on the Treated (ATET), 3=Average Treatment Effect (ATE) 4 = Local Average Treatment Effect (LATE)	Data extraction tool_immunisation
Summary of intervention	Summary of intervention	Free text, what is the intervention	Intervention_community engagement_dataset [col E]
Component #	Component #	List in numbers 1-6	Intervention_community engagement_dataset [col H]
Description	Description	Description [Col I in Intervention_community engagement_dataset]	Intervention_community engagement_dataset [col I]
Notes	Notes	Description [Col S in Intervention_community engagement_dataset]	Intervention_community engagement_dataset [col S]
Exposure to intervention (in months)	Exposure to intervention (in months)	How long is the intervention exposure itself?	Data extraction tool_immunisation
Evaluation period (in months)	Evaluation period (in months)	The total number of months elapsed between offering an intervention and the point at which an outcome measure is taken post intervention, or as a follow-up measurement. If less than one month, use decimals (e.g., one week would be .25)	Data extraction tool_immunisation
Post-intervention or change from baseline?	Post-intervention or change from baseline?	0 = Post-intervention, 1 = Change from baseline	Data extraction tool_immunisation
Source of the Outcome data (only use for outcomes in category JAA)	Source of the Outcome data (only use for outcomes in category JAA)	1=Immunisation Card, 2=Recall, 3=Combination of both immunisation card and recall, 4=Health Admin Data, 5=N/A	Data extraction tool_immunisation
Author definition of outcome	Author definition of outcome	Free text - How does the author define the outcome?	Data extraction tool_immunisation
Activities		What are the main (non-evaluation) activities undertaken by the project? (e.g. program design, targeting, community outreach, training of trainers, training of beneficiaries, M&E, etc.). Describe	Studies
Activities Data Source		Was the source of descriptive information about program activities taken from a table or taken from text? [Text, Table, Other(describe)]	Studies
Timeline		Is an intervention timeline included with the report? [Yes, No, If yes, page #]	Studies
Ingredients (unit cost of ingredients)		Is a list of cost ingredients or inputs for CEA, CBA, or CUA provided? [Yes, No]	Studies

For peer review only - http://bmjopen.bmj.com/site/about/guidelines.xhtml

		Ingredients will be items like "personnel", "staff", "travel", "office support", "program materials", etc. Note that searches for the unit costs of activities should include text searches as well as searches of tables.	
Are unit costs reported?		Look for a table with units or unit costs reported. Unit costs give the value of individual items that were used during the intervention, for example the unit cost of a staff person, e.g. a "health specialist" would be given in terms of monthly wages, or compensation or salary. Note that searches for the unit costs of activities should include text searches as well as searches of tables. [Yes, No, If Yes Page #]	Studies
Cost data sources		Was a source of cost data reported? (i.e. financial reports or accounting systems of implementing NGOs or donors (expenditures), NGO or donor (budgets), (market prices) Describe (or copy from text). Clarify - expenditure is what's actually spent, budget is what's planned for spending / not audited, often incorrect and not updated.	Studies
Costing methodology		Was a method of costing described? (i.e. key works: gross costing, micro-costing, ingredients method, activity-based costing method) [if yes, what was the method].	
Type of efficiency analysis (see glossary)		Indicate (CBA/CEA/CUA/cost-minimization, or innovative approaches: Social Return on Investment (SROI), Multi-Criteria Appraisal (MCA). Response options [CBA, CEA, CUA, SROI, MCA, or N/A if only "total cost", also ok to insert descriptive results that do not conform, e.g. “cost transfer ratio”]	Studies
Any cost-effectiveness analysis?	Source [from CEA Inventory]		
Any quant discussion of costs? (Full program)	Source [from CEA Inventory]		
If yes, what type? [Page number]	Source [from CEA Inventory]		
Currency of cost reporting		What is the currency in which costs were reported, i.e. USD, Rwandan Francs, etc.	Studies
Exchange rate		What exchange rate was used for currency adjustments? [e.g. 1.2 USD to 1 Euro]	
Exch rate year (estimated)			
Exchange rate date		What was the date, month, year of the exchange rate used? [If the exchange rate was calculated as an average over the implementation period, copy verbatim from text the definition of average exchange rate used]	

For peer review only

Was there discussion of a discount rate?		[Yes, No]	Studies
Discount rate		Which is the discount rate used in the analysis [this will be a % , please give the value]	Studies
If no discount rate is applied, was any justification provided?		Paste explanation provided in text. We consider any acknowledgement of the discount rate to be justification. For example, ok to report" "we did not discount costs or benefits" as justification	Studies
Inflation adjustment		Does the report mention if costs are given in "real" or "nominal" terms? Look also for "inflation"	Studies
Inflation adjustment of cost reported		If costs are reported in real terms, what inflation index was used to adjust for inflation?	Studies
Base year of the costing	base_year	Was a "base year" (or "start year") of the costing reported? [Yes, No] [If yes, What year?]	Studies
Average cost per number of participants		Was the average cost of the intervention reported per participant? [Yes, No] [If Yes, $ Amount per participant, and currency]	Studies
What is the denominator used in average intervention cost?	ac_ denominator	[Number, Description (please copy description verbatim from report)]	Studies
Average cost per DALY or QALY?		Is the average cost per DALY or QALY reported? [Yes, No, If Yes Page #], Also, add note if cost 'per capita' was given rather than average cost per number of participants.	Studies
Total intervention cost (excluding vaccine cost)	total_cost	Report total cost of the intervention, and page.	Studies
Did the total intervention cost reported above include the cost of vaccines?	vac_incl	A "No" indicates that cost ingredients were reported, but there is no reporting of vaccine costs. "Unclear" means we cannot tell from the information given if vaccines were included in total cost [Yes, No, Unclear]	Studies
Total cost description	total_cost_ desc	If there is a description of what is included in total cost, copy description from text here	Studies
Government contribution included in total cost?	tc_gov_ contb	[Yes, No]	
Is more than one total cost given in the report?		[Yes, No, If Yes, please describe additional "total cost" that is reported	
Number of immunisations			
Cost per vaccine delivered, cost per additional immunisation administered or cost per dose (of vaccine). Type of vaccine is given in col I			

For peer review only - http://bmjopen.bmj.com/site/about/guidelines.xhtml

Description and page number of cost per additional vaccine delivered.		Note if average costs per additional vaccine or marginal costs per additional vaccine is reported, where marginal costs should exclude fixed costs.	
Cost per additional child immunised_ All	cpc_all	[Yes, No, If Yes, please report cost per additional child immunised_all]	
Description and page number of cost per additional CHILD Immunised			
Cost per additional child immunised_DPT3	cpc_dpt3	[Yes, No, If Yes, please report cost per additional child immunised with DPT3]. Please make a note if only DPT1 was reported.	
Cost per additional child immunised_Measles	cpc_measles	[Yes, No, If Yes, please report cost per additional child immunised with measles]	
Baseline immunisation coverage of the target population_All		What was the reported baseline rate of immunisation (all immunisations) in the target population? [Percentage, or range]	Studies
Was baseline immunisation coverage of All immunisations reported for the target population, general population, or sample?		Was baseline immunisation coverage of All immunisations reported for the target population, general population, or sample? [target population, general population, sample]	Studies
Baseline immunisation coverage of the target population_DPT3		What was the reported baseline rate of immunisation (DPT3) in the target population? [Percentage, or range]	Studies
Was baseline immunisation coverage of DPT3 reported for the target population, general population, or sample?		Was baseline immunisation coverage of DPT3 reported for the target population, general population, or sample? [target population, general population, sample]	Studies
Baseline immunisation coverage of the target population_Measles		What was the reported baseline rate of immunisation (Measles) in the target population? [Percentage, or range]	Studies
Final immunisation coverage of the target population_All		What was the reported final rate of immunisation (all immunisations) in the target population? [Percentage, or range]	Studies
Final immunisation coverage of the target population_DPT3		What was the reported final rate of immunisation (DPT3) in the target population? [Percentage, or range]	Studies
Final immunisation coverage of the target population_Measles		What was the reported final rate of immunisation (Measles) in the target population? [Percentage, or range]	Studies
Non-compliance		Was there any evidence of non-compliance with treatment assignment. Non-compliance is where individuals assigned to treatment do not take the treatment, or the case where individuals assigned to control DO take the treatment. Ok to keyword search on "non-compliance", "non compliance", "noncompliance" [Yes, No] [If Yes, copy related text on non-compliance]	Studies

For peer review only - http://bmjopen.bmj.com/site/about/guidelines.xhtml

Spillover		Was there any evidence of spillover where individuals assigned to control receive a benefit or incur a 'cost' that results from the treatment intervention? [Yes, No] [If Yes, copy related text on spillovers detected]	Studies
Attrition or drop out		Was there evidence that participants dropped out of the study before follow-up could be completed? [Yes, No] [If Yes, copy related text on drop outs]	Studies
Vaccination coverage_All	JAA01. Full routine immunisation for children	Binary measure of whether or not children have received all routine vaccinations for the relevant country or region.	Data extraction tool_immunisation
Vaccination coverage_DPT3	JAA05. DPT3	Binary measure of whether or not children have received the third dose of the DPT or pentavalent vaccine. If the study does not specifically say "DPT3" (or "pentavalent 3"), but refers to "complete DPT/penta vaccination" or something similar, then use this code.	Data extraction tool_immunisation
Vaccination coverage_Measles	JAA11. Measles	Binary measure of whether or not children have received the measles vaccine	Data extraction tool_immunisation
Costs for significant outcomes only?		Does the report mention that costs will be reported only where significant outcomes are observed? [Yes, No]	

List of coded variables

VARIABLE LABEL	EXPLANATION
Study ID	This is the study ID - it should match the study ID from the Outcome Mapping Sheet (e.g., SC-SR_1)
Estimate ID	The estimate ID will provide a specific number for each effect size extracted and should include the original study number, underscore, then the unique ID number (e.g., SC-SR1_1, SC-SR1_2 and so on)
Author	For 1 author: leading author last name (e.g., Gomez)<br>For 2 authors: both author last names with ampersand in between (e.g., Smith & Bahn)<br>For 3 or more authors: leading author last name followed by et al. (e.g., Gupta et al.)
Year	Year published
Design	0=Experimental Design (e.g., RCT), 1=Quasi-Experimental Design
How Counterfactual is Chosen	Free text (e.g., random control trial, propensity score matching, etc) - Multiple codes are ok
Analysis type for this effect size	Free text, what type of analysis was used (Regression, 2SLS, ANCOVA, etc.)- Multiple codes are ok
Country	Country of intervention
Estimate Type	Type of data for this effect size: 1 = Continuous - means and SDs, 2 = Continuous - mean difference and SD, 3 = Dichotomous outcome - proportions, 4 = Regression data - dichotomous outcome (e.g., logistic regression)

For peer review only - http://bmjopen.bmj.com/site/about/guidelines.xhtml

	5 = Regression data - continuous outcome (e.g., linear regression)
Comparison	1=No intervention (service delivery as usual), 2=Other intervention, 3=Pipeline (wait-list) control (still service delivery as usual)
Describe Comparison Group	If answer above is (1) no intervention, type N/A, if (2) Other Intervention, list what intervention the control group is receiving, if (3) Pipeline control, report when the control group will receive the intervention in relation to the treatment group (e.g., one year later)
Subgroup	Is this analysis of a subgroup? 0=no, 1=yes
If yes to subgroup, describe	Free text, describe the subgoup if applicable (e.g., boys, girls). If no subgroup, type N/A
Source	Note the page number, table number, column, and row you used to extract the data
Treatment Effect	1=Intention to Treat (ITT), 2=Average Treatment Effect on the Treated (ATET), 3=Average Treatment Effect (ATE) 4 = Local Average Treatment Effect (LATE)
Intervention	Free text, what is the intervention
Exposure to intervention (in months)	How long is the intervention exposure itself?
Evaluation period (in months)	The total number of months elapsed between offering an intervention and the point at which an outcome measure is taken post intervention, or as a follow-up measurement. If less than one month, use decimals (e.g., one week would be .25)
Post-intervention or change from baseline?	0 = Post-intervention, 1 = Change from baseline
Source of the Outcome data (only use for outcomes in category JAA)	1=Immunization Card, 2=Recall, 3=Combination of both immunization card and recall, 4=Health Admin Data, 5=N/A, 6 = unclear
Author definition of outcome	Free text - How does the author define the outcome?
Intervention	Coded as engagement as intervention, engagement in the intervention design, engagement in implementation autonomy, or multiple engagement types (place a 1 in the appropriate column
If yes to multiple engagement types	Identify which engagement types are used.
Implemented by the government	0 = no, 1 = yes
Were new cadres of health workers formed?	0 = no, 1 = yes
Was vaccination hesitency identified as a barrier?	0 = no, 1 = yes
Baseline rates of full immunisation	record percentage vaccinated
Baseline rates of DPT3	record percentage vaccinated
**OUTCOME CODES**	
	Code 1 under any applicable columns.
**EFFECT SIZE DATA EXTRACTION**	
Reverse Sign (i.e., decrease is good)	Record 0='no' if an increase is good, record 1='yes' if a decrease is good and the sign needs to be reversed.
Unit of analysis	What is the unit of analysis? UOA for this effect size: 1= Individual, 2= Household, 3= Group (e.g.

For peer review only - http://bmjopen.bmj.com/site/about/guidelines.xhtml

	community organisation), 4= Health Center, 5 = Village, 6 = Other, 7 = Not clear
mean_t	Outcome mean for the treatment group
sd_t	Outcome standard deviation for treatment group
mean_c	Outcome mean for the comparison group
sd_c	Outcome standard deviation for control group
mean_overall_diff	Overall mean difference (treatment - control)
diff se	Standard error of the overall mean difference
Diff _t	t-statistic of mean difference
Odds ratio	Odds ratio reported in the study
OR_se	Odds ratio standard error reported in the study
Risk ratio	Risk ratio reported in study
RR_se	Risk ratio standard error
reg_coeff	Report the regression coefficient of the treatment effect
reg_SE	Report the associated standard error of the regression coefficient.
reg_t	Report the associated t statistic of the effect size (coefficient/SE)
Exact p value	Exact p value if given, if not, record as written in the manuscript (e.g., p < .001, or p > .05)
clust_t	Number of clusters - treatment group
clust_c	Number of clusters - control group
clust_T	Number of clusters - total sample
n_t	Sample size - treatment group
n_c	Sample size - control group
n_T	Sample size - total sample
periods (1 if cross sectional)	Record how many periods of evaluation there are (e.g., cross section is 1, panel data with 3 measurements is 3)
Treatment Variable	Record the treatment variable as written in the model (e.g., the variable name the author uses, such as ("Intervention x Time")
dataset	Record if data comes from an identified dataset
coder	Record your name
Notes	Record any notes important for the team
ROB Category	0 = low risk of bias, 1 = some concerns, 2 = high risk of bias

For peer review only

Appendix 7: Risk of bias assessment tools

Quantitative risk of bias tool

Code	Question	Coding format	Criteria
General	ID	EPPI ID	
Genera	Study first author	Open answer	
General	Time taken to complete assessment	Minutes	
General	Design type: What type of study design is used?	1= Randomised controlled trial (RCT) (random assignment to households/individuals) or quasi-RCT<br>2 = Cluster-RCT (quasi-RCT)<br>3 = Pseudo-RCT	-
General	Methods used for analysis: Which methods are used to control for selection bias and confounding?	1 = Statistical matching (PSM, CEM, covariate matching)<br>2 = Difference in differences (DID) estimation methods<br>3 = IV-regression (2-stage least squares or bivariate probit)<br>4 = Heckman selection model<br>5 = Fixed effects or random effects regression<br>6 = Covariate adjusted estimation<br>7 = Propensity weighted regression<br>8 = Comparison of means<br>9 = Other (please state)	-
General	Design and analysis method description	Open answer	Briefly describe the study design and analysis method undertaken by the authors.
General	Study population	Open answer	Provide any details in the paper that describe how the study population was selected, answering the question: what is the sampling strategy to recruit participants from that population into the evaluation?
General	Type of comparison group	1=No intervention (service delivery as usual)<br>2=Other intervention<br>3=Pipeline (wait-list) control (still service delivery as usual)	Indicate type of comparison group
General	Type of comparison group (if other)	Open answer	

For peer review only - http://bmjopen.bmj.com/site/about/guidelines.xhtml

General	Ethical clearance	Open answer	Provide any details of ethical research clearances granted. Report unclear if this information is not available.
General	Study registration	Open answer	Provide any details of study registration, including registry IDs, etc.
1: Bias arising from randomisation process	1.1 Was the allocation sequence random?	(1) Yes;<br>(2) Probably yes;<br>(3) Probably no;<br>(4) No;<br>(5) No information;	1.1 Was the allocation sequence random?<br>Answer 'Yes' if a random component was used in the sequence generation process. Examples include computer-generated random numbers; reference to a random number table; coin tossing; shuffling cards or envelopes; throwing dice; or drawing lots. Minimization is generally implemented with a random element (at least when the scores are equal), so an allocation sequence that is generated using minimization should generally be considered to be random.<br><br>Answer 'No' if no random element was used in generating the allocation sequence or the sequence is predictable. Examples include alternation; methods based on dates (of birth or admission); patient record numbers; allocation decisions made by clinicians or participants; allocation based on the availability of the intervention; or any other systematic or haphazard method.<br><br>Answer 'No information' if the only information about randomization methods is a statement that the study is randomized.<br><br>Note: In some situations a judgement may be made to answer 'Probably no' or 'Probably yes'. For example, if the study was large, conducted by an independent trials unit or carried out for regulatory purposes, it may be reasonable to assume that the sequence was random. Alternatively, if other (contemporary) trials by the same investigator team have clearly used non-random sequences, it might be reasonable to assume that the current study was done using similar methods.
1: Bias arising from randomisation process	Question 1.1 answer justification	Open answer	Justification for coding decision<br>(Include a brief summary of justification for rating, mentioning your response to all sub questions, cite relevant pages).
1: Bias arising from randomisation process	1.2 Did baseline differences between intervention groups suggest a problem with the randomisation process?	(1) Yes;<br>(2) Probably yes;<br>(3) Probably no;<br>(4) No;<br>(5) No information;	1.2 Did baseline differences between intervention groups suggest a problem with the randomization process?<br><br>Note that differences that are compatible with chance do not lead to a risk of bias.

For peer review only - http://bmjopen.bmj.com/site/about/guidelines.xhtml

			Answer 'No' if no imbalances are apparent or if any observed imbalances are compatible with chance.<br><br>Answer 'Yes' if there are imbalances that indicate problems with the randomization process, including:<br>(1) substantial differences between intervention group sizes, compared with the intended allocation ratio;<br>or<br>(2) a substantial excess in statistically significant differences in baseline characteristics between intervention groups, beyond that expected by chance (i.e., on more than 10% of the characteristics reported); or<br>(3) imbalance in one or more key prognostic factors, or baseline measures of outcome variables, that is very unlikely to be due to chance and for which the between-group difference is big enough to result in bias in the intervention effect estimate.<br>Also answer 'Yes' if there are other reasons to suspect that the randomization process was problematic, such as:<br>(4) excessive similarity in baseline characteristics that is not compatible with chance; or<br>(5) surprising absence of one or more key baseline characteristics that would be expected to be reported.<br><br>Answer 'No information' when there is no useful baseline information available (e.g. abstracts, or studies that reported only baseline characteristics of participants in the final analysis).<br><br>Note: Trialists may undertake analyses that attempt to deal with flawed randomization by controlling for imbalances in prognostic factors at baseline. To remove the risk of bias caused by problems in the randomization process, it would be necessary to know, and measure, all the prognostic factors that were imbalanced at baseline. It is unlikely that all important prognostic factors are known and measured, so such analyses will at best reduce the risk of bias.
1: Bias arising from randomisation process	Question 1.2 answer justification	Open answer	Justification for coding decision<br>(Include a brief summary of justification for rating, mentioning your response to all sub questions, cite relevant pages).
1: Bias arising from	Assignment mechanism: Was the allocation or identification	(0) Low risk of bias<br>(1) Medium risk of bias/Some concerns	Utliize your answers for questions 1.1 and 1.2 to determine the overall score.

For peer review only - http://bmjopen.bmj.com/site/about/guidelines.xhtml

randomisation process	mechanism random or as good as random?	(2) High risk of bias	
1: Assignment mechanism - Justification	Assignment justification	Open answer	Justification for coding decision<br>(Include a brief summary of justification for rating, mentioning your response to all sub questions, cite relevant pages).
2: Unit of analysis - Assessment	Unit of analysis: Is unit of analysis in cluster allocation addressed in standard error calculation?	1=Yes<br>2=No<br>3=Not reported/unclear<br>4=Not applicable	Score "Yes" if Unit of Analysis = Unit of Randomization OR if UoA ≠ UoR and standard errors are clustered at the UoR level OR data is collapsed to the UoR level<br><br>Score "Not reported/unclear" if not enough information is provided on the way the standard errors were calculated or what the unit of analysis is.<br><br>Score "Not applicable" if it is not a cluster RCT.<br><br>Score "No" otherwise.Score "Yes" if Unit of Analysis = Unit of Randomization OR if UoA ≠ UoR and standard errors are clustered at the UoR level OR data is collapsed to the UoR level<br><br>Score "Not reported/unclear" if not enough information is provided on the way the standard errors were calculated or what the unit of analysis is.<br><br>Score "Not applicable" if it is not a cluster RCT.<br><br>Score "No" otherwise.
2: Unit of analysis - Justification	Question 2 answer justification (provide page numbers)	Open answer	-
3. Bias due to missing outcome data (panel data)	3.1. Was there attrition, if yes, then what was the rate in treatment and control/comparison groups?	(1) Yes;<br>(2) Probably yes;<br>(3) Probably no;<br>(4) No;<br>(5) No information;	3.1. Was there attrition, if yes, then what was the rate in treatment and control/comparison groups?<br><br>In case of attrition, answer 'yes' or 'probably yes', otherwise answer 'no' or 'probably no'.<br><br>If there is no discussion on attrition then score 'no information'.<br><br>Specify the rate of attrition by the study arms.<br>Specify the attrition rate assumed for power calculations.

For peer review only - http://bmjopen.bmj.com/site/about/guidelines.xhtml

			Has the study discussed the ability of the follow-up study to detect the hypothesised outcome effect with the sample size attained?
3. Bias due to missing outcome data – Justification (panel data)	Question 3.1 answer justification	Open answer	Justification for coding decision<br>(Include a brief summary of justification for rating, mentioning your response to all sub questions, cite relevant pages).
3. Bias due to missing outcome data (panel data)	3.2. Does the study establish that attrition is randomly distributed (e.g. by examining correlation with determinants of outcomes, in both treatment and comparison groups or by presenting data showing balance on key characteristics across treatment and control)?	(1) Yes;<br>(2) Probably yes;<br>(3) Probably no;<br>(4) No;<br>(5) No information;	3.2. Does the study establish that attrition is randomly distributed (e.g. by examining correlation with determinants of outcomes, in both treatment and comparison groups or by presenting data showing balance on key characteristics across treatment and control)?<br><br>Score 'yes' or 'probably yes' if balance on key characteristics between attritors and non-attritors for each intervention group (including control) using baseline data has been presented and finds that there is no systematic difference on at least observable dimensions.<br><br>Score 'no' or 'probably no' if the study finds that there is a systematic difference on observables between attritors and non-attritors in any of the intervention groups.<br><br>If the baseline balance table between atritors and non-attritors is not presented or discussed then score 'no information'.
3. Bias due to missing outcome data – Justification (panel data)	Question 3.2 answer justification	Open answer	Justification for coding decision<br>(Include a brief summary of justification for rating, mentioning your response to all sub questions, cite relevant pages).
3. Bias due to missing outcome data – (panel data)	3.3. If there is non-random attrition, have authors used convincing statistical techniques to identify and adjust for the attrition bias?	(1) Yes;<br>(2) Probably yes;<br>(3) Probably no;<br>(4) No;<br>(5) No information;<br>(6) Not applicable	3.3. If there is non-random attrition, have authors used convicing statistical techniques to identify and adjust for the attrition bias?<br><br>If the study has used convincing parametric or non-parametric techniques (like ignorable maximum likelihood, Manski-Lee bounds) to identify and adjust for attrition bias, then score 'yes' or 'probably yes'. If the techniques used are not convincing then score 'no' or 'probably no'. If the study does not discuss using any statiscal techniques for identifying and adjusting for attrition bias then score 'no information'.

For peer review only - http://bmjopen.bmj.com/site/about/guidelines.xhtml

			If there is no non-random attrition, then this question does not apply and the score should be 'not applicable'
3. Bias due to missing outcome data – (panel data)	Question 3.3 answer justification	Open answer	Justification for coding decision<br>(Include a brief summary of justification for rating, mentioning your response to all sub questions, cite relevant pages).
3. Bias due to missing outcome data – Justification (panel data)	Bias due to missing outcome data justification	Open answer	Justification for coding decision<br>(Include a brief summary of justification for rating, mentioning your response to all sub questions, cite relevant pages).
3. Bias due to missing outcome data - Assessment		(0) Low risk of bias<br>(1) Medium risk of bias/Some concerns<br>(2) High risk of bias	Utliize your answers for questions 3.1 to 3.3 to determine the overall score.
3. Bias due to missing outcome data (repeated cross section)	3.1. Was there difference in sample size across baseline and follow-up surveys among treatment and control groups? If yes, what was the rate of difference among treatment and control/comparison groups? Was it lower or higher than baseline?	(1) Yes;<br>(2) Probably yes;<br>(3) Probably no;<br>(4) No;<br>(5) No information;	3.1. Was there difference in sample size across baseline and follow-up surveys among treatment and control groups? If yes, what was the rate of difference among treatment and control/comparison groups? Was it lower or higher than baseline?<br><br>In case of difference, answer 'yes' or 'probably yes', otherwise answer 'no' or 'probably no'.<br><br>If there is no information to assess the differences, then score 'no information'.<br><br>Specify the rate of difference by the study arms.<br>Specify the non-compliance and/or attrition rate assumed for power calculations.<br>Has the study discussed the ability of the follow-up study to detect the hypothesised outcome effect with the sample size attained?
3. Bias due to missing outcome data – Justification (repeated cross section)	Question 3.1 answer justification	Open answer	Justification for coding decision<br>(Include a brief summary of justification for rating, mentioning your response to all sub questions, cite relevant pages).
3. Bias due to missing outcome	3.2. Does the study establish that the sampling frame for baseline and follow-up	(1) Yes;<br>(2) Probably yes;<br>(3) Probably no;	3.2. Does the study establish that the sampling frame for baseline and follow-up surveys is same? Are the clusters (eg health center) and sub-clusters (eg. villages) the same across baseline and follow-up surveys?

For peer review only - http://bmjopen.bmj.com/site/about/guidelines.xhtml

data (repeated cross section)	surveys is same? Are the clusters (eg health center) and sub-clusters (eg. villages) the same across baseline and follow-up surveys?	(4) No;<br>(5) No information;	Score 'yes' or 'probably yes' if sampling frame (clusters and sub-clusters) are the same for baseline and follow-up surveys<br><br>Score 'no' or 'probably no' if the clusters or sub-clusters were different across baseline and follow-up surveys.<br><br>If there is no information on sampling frame across baseline or follow-up surveys, then score 'no information'.
3. Bias due to missing outcome data – Justification (repeated cross section)	Question 3.2 answer justification	Open answer	Justification for coding decision<br>(Include a brief summary of justification for rating, mentioning your response to all sub questions, cite relevant pages).
3. Bias due to missing outcome data –(repeated cross section)	3.3. Was the sample selection of respondents carried out in the same way as in the baseline? Was the intended number of respondents in the clusters/sub-clusters same across baseline and follow-up surveys?	(1) Yes;<br>(2) Probably yes;<br>(3) Probably no;<br>(4) No;<br>(5) No information;	3.3. Was the sample selection of respondents carried out in the same way as in the baseline? Was the intended number of respondents in the sub-clusters same across baseline and follow-up surveys?<br><br>Score 'yes' or 'probably yes' if the study used same listing process for selection of respondents and their intended number was same across baseline and follow-up surveys,<br><br>Score 'no' or 'probably no' if the study used different listing process for selection of respondents or their intended number was different across baseline and follow-up surveys.<br><br>If there is no information on the selection process of respondents then score 'no information'.
3. Bias due to missing outcome data - Justification (repeated cross section)	Question 3.3 answer justification	Open answer	Justification for coding decision<br>(Include a brief summary of justification for rating, mentioning your response to all sub questions, cite relevant pages).
3. Bias due to missing outcome data – Assessment	Bias due to missing outcome data	(0) Low risk of bias<br>(1) Medium risk of bias/Some concerns<br>(2) High risk of bias	Utliize your answers for questions 3.1 to 3.3 to determine the overall score using the criteria below

For peer review only - http://bmjopen.bmj.com/site/about/guidelines.xhtml

(repeated cross section)			
Bias due to missing outcome data – Justification (repeated cross section)	Bias due to missing outcome data justification	Open answer	Justification for coding decision<br>(Include a brief summary of justification for rating, mentioning your response to all sub questions, cite relevant pages).
3. Bias due to missing outcome data - Assessment	(0) Low risk of bias<br>(1) Medium risk of bias/Some concerns<br>(2) High risk of bias		Utliize your answers for questions 3.1 to 3.3 to determine the overall score.
3.Bias due to missing outcome data - Justification	Bias due to missing outcome data justification	Open answer	Justification for coding decision<br>(Include a brief summary of justification for rating, mentioning your response to all sub questions, cite relevant pages).
4: Bias due to deviations from intended interventions - Assessment	4.1. Was the study adequately protected against spill-overs/ contamination?	(1) Yes;<br>(2) Probably yes;<br>(3) Probably no;<br>(4) No;<br>(5) No information;	4.1. Was the study adequately protected against spill-overs/ contamination?<br><br>Score "Yes" if the intervention is unlikely to spill-over to comparisons (e.g. participants and non-participants are geographically and/or socially separated from one another and general equilibrium effects are not likely) and that the treatment and comparisons are isolated from other interventions which might explain changes in outcomes.<br><br>Score "No" if allocation was at the individual or classroom level and there are likely spill-overs within households and communities which are not controlled for (such as using trasferable vouchers), or other interventions likely to affect outcomes operating at the same time in either group.<br><br>Score "no information" if spill-overs and/or contamination are not discussed
4: Bias due to deviations from intended interventions - Justification	Question 4.1 answer justification	Open answer	Justification for coding decision<br>(Include a brief summary of justification for rating, mentioning your response to all sub questions, cite relevant pages).

For peer review only - http://bmjopen.bmj.com/site/about/guidelines.xhtml

4: Bias due to deviations from intended interventions - Assessment	4.2. Was the process of being monitored free from bias?	(1) Yes;<br>(2) Probably yes;<br>(3) Probably no;<br>(4) No;<br>(5) No information;	4.2. Was the process of being monitored free from bias?<br><br>Score "Yes" if the authors state explicitly that the process of monitoring the intervention is blinded, or argue convincingly why it is not likely that intervention delivery could affect the performance of participants in treatment and comparison groups in different ways (such as resulting in Hawthorne or John Henry effects). or there is nothing in the surveys that might have given the control participants an idea of what the other group might receive (e.g. provide cash transfer to the intervention area participants or reveal informmation that they did not have before or they did but there is no risk that this has changed their behaviours;<br><br>Score "No" if the the authors do not use an appropriate method to prevent Hawthorne and John Henry Effects (e.g. blinding of monitoring or other methods to ensure consistent monitoring across groups) and there is a risk that the intervention delivery or survey process could have changed the behaviours of treatment and comparison groups in different ways.<br><br>Score "no information" if the authors do not discuss potential bias due to monitoring of intervention.<br><br>Hawthorne effects may result where participants know that they are being observed and John Henry Effects may result from participant knowledge of being compared.
4: Bias due to deviations from intended interventions - Justification	Question 4.2 answer justification	Open answer	Justification for coding decision<br>(Include a brief summary of justification for rating, mentioning your response to all sub questions, cite relevant pages).
4: Bias due to deviations from intended interventions - Assessment	4.3. Was an appropriate analysis used to estimate the effect of assignment to intervention?	(1) Yes;<br>(2) Probably yes;<br>(3) Probably no;<br>(4) No;<br>(5) No information;	44.3. Was an appropriate analysis used to estimate the effect of assignment to intervention?<br><br>Both intention-to-treat (ITT) analyses and modified intention to treat (mITT) analyses excluding participants with missing outcome data should be considered appropriate. (Missing outcome data are addressed in a separate domain). Both 'as treated' analyses (in which trial participants are grouped according to the intervention that they received, rather than according to their assigned intervention) and

For peer review only - http://bmjopen.bmj.com/site/about/guidelines.xhtml

			naïve ‘per-protocol’ analyses (excluding trial participants who did not receive their assigned intervention) should be considered inappropriate. Analyses excluding eligible trial participants post-randomization should also be considered inappropriate, but post-randomization exclusions of ineligible participants (when eligibility was not confirmed until after randomization, and could not have been influenced by intervention group assignment) can be considered appropriate.<br><br>Score "Yes" if ITT or modified ITT analysis has been used, AND common analysis methods are used AND any covariates imbalanced at baseline are included as covariates in the analysis.<br>Score "No" otherwise. You may also score no if they use an uncommon analysis methods, such as 3SLS, or if they analyse dichotomous outcome using continuous methods (e.g., using linear regression instead of using the more appropriate logit or probit models) or if the is baseline imbalance but those covariates are not included in the model.<br><br>If the analytical specification is not provided score "no information"
4: Bias due to deviations from intended interventions - Justification	Question 4.3 answer justification	Open answer	Justification for coding decision<br>(Include a brief summary of justification for rating, mentioning your response to all sub questions, cite relevant pages).
4: Bias due to deviations from intended interventions - Assessment	Deviations from intended interventions: Spill-overs, cross-overs, contamination and performance bias: was the study adequately protected against spill-overs, cross-overs, contamination, and performance bias?	(0) Low risk of bias<br>(1) Medium risk of bias/Some concerns<br>(2) High risk of bias	Utliize your answers for questions 4.1 to 4.3 to determine the overall score.
4: Bias due to deviations from intended interventions - Justification	Deviations justification	Open answer	Justification for coding decision<br>(Include a brief summary of justification for rating, mentioning your response to all sub questions, cite relevant pages).<br><br>For example, intervention groups are geographically separated, authors use intention to treat estimation or instrumental variables to

For peer review only - http://bmjopen.bmj.com/site/about/guidelines.xhtml

			account for non-adherence, and survey questions are not likely to expose individuals in the control group to information about desirable behaviours ('survey effects').
5. Outcome measurement bias	5.1 Could the measurement of the outcome be different between the study arms?	(1) Yes;<br>(2) Probably yes;<br>(3) Probably no;<br>(4) No;<br>(5) No information;	5.1 Could the measurement of the outcome be different between the study arms?<br><br>Comparable methods of outcome measurement (data collection) involve the same measurement methods and thresholds, used at comparable time points. Differences between intervention groups may arise because of 'diagnostic detection bias' in the context of passive collection of outcome data, or if an intervention involves additional visits to a healthcare provider, leading to additional opportunities for outcome events to be identified.<br><br>Score 'yes' or 'probably yes' if the outcomes have been measured at different times periods for treatment and control arms or have used different methods of measurement.<br><br>Score 'no' or 'probably no' otherwise.<br><br>If no information is provided on the the time period and methods of data collection in treatment and control arms, then score 'no information'
5. Outcome measurement bias - Justification	Question 5.1 answer justification	Open answer	Justification for coding decision<br>(Include a brief summary of justification for rating, mentioning your response to all sub questions, cite relevant pages).
5. Outcome measurement bias	5.2. Were the outcome assessors (enumerators) blinded to the intervention assignment?	(1) Yes;<br>(2) Probably yes;<br>(3) Probably no;<br>(4) No;<br>(5) No information;	5.2. Were the outcome assessors (enumerators) blinded to the intervention assignment?<br><br>Score 'yes' or 'probably yes' if outcome assessors were blinded to intervention status and 'no' or 'probably no' otherwise.<br><br>If no information is provided on blinding of the outcome assessors, then score 'no information'.<br><br>Note that for participant-reported outcomes, the outcome assessor is the study participant.
5. Outcome measurement	Question 5.2 answer justification	Open answer	Justification for coding decision

For peer review only - http://bmjopen.bmj.com/site/about/guidelines.xhtml

bias - Justification			(Include a brief summary of justification for rating, mentioning your response to all sub questions, cite relevant pages).
5. Outcome measurement bias	5.3. If not blinded, could assessment of the outcome have been influenced by knowledge or administration of intervention received?	(1) Yes;<br>(2) Probably yes;<br>(3) Probably no;<br>(4) No;<br>(5) No information;	5.3. If not blinded, could assessment of the outcome have been influenced by knowledge or administration of intervention received?<br><br>Knowledge or administration of the assigned intervention could influence participant-reported outcomes (such as level of pain, immunization outcomes using recall), observer-reported outcomes involving some judgement, and intervention provider decision outcomes. They are unlikely to influence observer-reported outcomes that do not involve judgement, for example all-cause mortality.<br><br>For participant-reported outcomes, the assessment of outcome is potentially influenced by knowledge or administration of intervention received, leading to a judgement of at least ‘Some concerns’. Review authors will need to judge whether it is likely that participants’ reporting of the outcome was influenced by knowledge or administration of intervention received, in which case risk of bias is considered to be high.<br><br>Score 'yes' or 'probably yes' if assessment of outcome could have been influenced by knowledge of intervention received (e.g., if ANY of the immunization coverage outcomes are assessed through caregiver recall) and 'no' or 'probably no' otherwise (e.g., if immunization coverage outcome is assessed by immunization card).<br>If no information is available to make the assessment then score 'no information'.
5. Outcome measurement bias - Justification	Question 5.3 answer justification	Open answer	Justification for coding decision<br>(Include a brief summary of justification for rating, mentioning your response to all sub questions, cite relevant pages).
5. Outcome measurement bias - Assessment	Outcome measurement bias: Was the study free from biases in outcome measurement?	(0) Low risk of bias<br>(1) Medium risk of bias/Some concerns<br>(2) High risk of bias	Utliize your answers for questions 5.1 to 5.3 to determine the overall score.
5. Outcome measurement bias - Justification	Outcome measurement justification	Open answer	Justification for coding decision<br>(Include a brief summary of justification for rating, mentioning your response to all sub questions, cite relevant pages).

For peer review only - http://bmjopen.bmj.com/site/about/guidelines.xhtml

For peer review only

6. Reporting bias	6.1. Is a pre-analysis plan or protocol available which provides sufficient detail?	(1) Yes;<br>(2) Probably yes;<br>(3) Probably no;<br>(4) No;<br>(5) No information;	6.1. Is a pre-analysis plan or protocol available which provides sufficient detail?<br><br>Score 'yes' if they reference a preanalysis plan, and 'no' otherwise.
6. Reporting bias - Justification	Question 6.1 answer justification	Open answer	Justification for coding decision<br>(Include a brief summary of justification for rating, mentioning your response to all sub questions, cite relevant pages).
6. Reporting bias	6.2. Were all primary and secondary outcomes reported as per the pre-analysis plan/protocol?	(1) Yes;<br>(2) Probably yes;<br>(3) Probably no;<br>(4) No;<br>(5) No information;	6.2. Were all primary and secondary outcomes reported as per the pre-analysis plan/protocol?<br><br>Score "Yes" or "probably yes" if there is no evidence that outcomes were selectively reported (e.g. results for all relevant outcomes in the methods section are reported in the results section)<br><br>Score "No" or "probably no" if some important outcomes are subsequently omitted from the results or the significance and magnitude of important outcomes was not assessed or if multiple measurements of an outcome were made but only one or a subset is reported on the basis of the results (e.g. statistical significance)<br><br>Score "No information" if pre-analysis not available or the outcome intentions are not reported in sufficient detail to enable an assessment.
6. Reporting bias - Justification	Question 6.2 answer justification	Open answer	Justification for coding decision<br>(Include a brief summary of justification for rating, mentioning your response to all sub questions, cite relevant pages).
6. Reporting bias	6.3. Do reported results for the outcomes correspond to all intended analyses?	(1) Yes;<br>(2) Probably yes;<br>(3) Probably no;<br>(4) No;<br>(5) No information;	6.3. Do reported results for the outcomes correspond to all intended analyses?<br><br>A particular outcome domain may be analysed in multiple ways. Examples include: unadjusted and adjusted models; final value vs change from baseline vs analysis of covariance; transformations of variables; different definitions of composite outcomes (e.g. 'major adverse event'); conversion of continuously scaled outcome to categorical data with different cut-points; different sets of covariates for adjustment; and different strategies for dealing with missing data. Application of multiple methods generates multiple effect estimates for a specific outcome domain. If multiple estimates are generated but only one or a subset is reported on the basis of the results (e.g.

For peer review only - http://bmjopen.bmj.com/site/about/guidelines.xhtml

			statistical significance), there is a high risk of bias in the fully reported result.<br><br>Answer 'No' or 'Probably No' if:<br>There is clear evidence (usually through examination of a trial protocol or statistical analysis plan) that a domain was analysed in multiple ways, but data for only one or a subset of analyses is fully reported (without justification), and the fully reported result is likely to have been selected on the basis of the results. Selection on the basis of the results arises from a desire for findings to be newsworthy, sufficiently noteworthy to merit publication, or to confirm a prior hypothesis. For example, trialists who have a preconception or vested interest in showing that an experimental intervention is beneficial may be inclined to selectively report analyses that are favourable to the experimental intervention. Score probably no if authors do not report both adjusted and unadjusted models.<br><br>Answer 'Yes' or 'Probably Yes' if:<br>There is clear evidence (usually through examination of a trial protocol or statistical analysis plan) that all reported results for the outcome domain correspond to all intended analyses.<br>or<br>Not all intended analyses have been reported but authors have convincingly justified the reasons for not doing so.<br>or<br>There is only one possible way in which the outcome domain can be analysed (hence there is no opportunity to select from multiple analyses).<br>or<br>Analyses are inconsistent across different reports on the same trial, but the trialists have provided the reason for the inconsistency and it is not related to the nature of the results.<br><br>Answer 'No information' if:<br>Analysis intentions are not available, or the analysis intentions are not reported in sufficient detail to enable an assessment, and there is more than one way in which the outcome domain could have been analysed.
6. Reporting bias - Justification	Question 6.3 answer justification	Open answer	Justification for coding decision<br>(Include a brief summary of justification for rating, mentioning your response to all sub questions, cite relevant pages).

For peer review only - http://bmjopen.bmj.com/site/about/guidelines.xhtml

6. Reporting bias - Assessment	Analysis reporting: Was the study free from selective analysis reporting?	(0) Low risk of bias<br>(1) Medium risk of bias/Some concerns<br>(2) High risk of bias	Utliize your answers for questions 6.1 to 6.3 to determine the overall score.
6. Reporting bias - Justification	Analysis reporting justification	Open answer	Justification for coding decision<br>(Include a brief summary of justification for rating, mentioning your response to all sub questions, cite relevant pages).
7. Other bias - Assessment	Other risks of bias Is the study free from other sources of bias?	1= Yes,<br>4 = No	-
7. Other bias - Justification	Other bias justification	Open answer	Justification for coding decision<br>(Include a brief summary of justification for rating, mentioning your response to all sub questions, cite relevant pages). For example, information is collected using a different survey instrument in different intervention groups; measurement of the intervention received in unclear.
8. Blinding - observers - Assessment	Blinding of participants?	1 = Yes<br>2 = No<br>8 = unclear<br>9 = N/A	If there is no information, code NO. If there is information but it is ambiguous, code UNCLEAR.
8. Blinding - analysts - Assessment	Blinding of data analysts?	1 = Yes<br>2 = No<br>8 = unclear<br>9 = N/A	If there is no information, code NO. If there is information but it is ambiguous, code UNCLEAR.
8. Blinding - method(s)	Method(s) used to blind	Open answer (including describe method of placebo control) 9 = N/A	Describe method(s) used to blind
9. External validity - Random Sampling	Was random sampling used?	1 = Yes<br>2 = No	Was a random sampling method used (meaning everyone in the population had an equal chance of being selected for the study - note that this is different than random allocation to treatment versus control group)?<br>Score yes if: (a) there is a sampling frame. The sampling frame is the actual list of individuals that the sample will be drawn from. Ideally, it should include the entire target population (and nobody who is not part of that population). AND<br>(b) probability sampling is used (e.g., simple random, stratified random, cluster random, etc.)<br>Score no if: individuals are selected based on non-random criteria, and not every individual has a chance of being included (e.g., a convenience sample, a purposive sample, a snowball sample, a voluntary response sample, etc.).

For peer review only - http://bmjopen.bmj.com/site/about/guidelines.xhtml

9. External validity - Random Sampling Justification	Justification for answer to random sampling question	Open answer	Justification for coding decision (Include a brief summary of justification for rating, mentioning your response to all sub questions, cite relevant pages).Describe the sampling technique used.
9. External validity - Assessment	External validity	Open answer	a) What do authors say about external validity? Note any additional information related to generalizability.

For peer review only

For peer review only - http://bmjopen.bmj.com/site/about/guidelines.xhtml

Qualitative risk of bias tool

Critical appraisal of qualitative studies tool

This tool provides specific questions you should answer with regards to the study being appraised. Every question has three possible responses, which correspond to ‘strong’, ‘weak’, or ‘none’ – these are phrased slightly differently depending on the question. This process is subjective and relies on a basic level of familiarity with qualitative research methods. However, to further structure the assessment, each question is accompanied by a description of criteria you should consider before making your choice.

While attempting to answer a question, you may find that a study contains many of the relevant elements, but not all. Give this paper the benefit of the doubt – if the element being assessed is strong except for one minor element, choose the ‘strong’ option.

There is room for comments after each question. You are encouraged to utilize this space, though only the multiple answer response is mandatory. Use the space to document your thoughts if you feel uneasy about the choice made, or if you have any ideas which would help as we refine and revise the assessment tool. There is also room at the end for any additional notes on the paper as a whole.

The questions are arranged according to the order in which a standard academic article is arranged: Introduction, Methodology, Results, Discussion. This will allow you to answer the questions as you go over each section. That said, some documents will not follow this structure, and some academic articles are more fluid about where certain descriptions are located within the text. After you read through the article, go over all the questions once more to see whether any elements you thought were missing actually showed up in an unexpected place.

* Required

Assessor: *

Paper Title: *

Authors: *

Year: *

Introduction

*1. Is the research aim clearly stated? **

Look for this in either the abstract or the introduction. In the best studies, this will be an explicit formulation of the research aims/questions. There may be several. Other studies do not formally state the research aims as such, but they are clearly evident from the text. Note: you might find it useful to write down these research aims, as many of the other questions relate to them.

- Yes, a strong statement
- Yes, but an unclear or weak statement
- No

Question 1 – Notes:

*2. Is there a description of the context in which the study takes place? **

For peer review only - http://bmjopen.bmj.com/site/about/guidelines.xhtml

The study should make some reference to the geographical, temporal, or societal greater context within which the study topic is located. A study within a well-established area of research could likely be situated quite specifically. An exploratory study might cover a topic where there is little descriptive context to draw upon.

- Yes, a strong description
- Yes, but a weak description
- No

Question 2 – Notes:

*3. Is there a clear link to relevant literature? **

Literature should be cited not just in general, but in specific relation to the topic of the paper. In assessing whether the literature is relevant and sufficient, consider: Are most of the cited papers about the same area or topic as the study? Are both qualitative and quantitative studies cited? Are there at least some papers published within the last 10 years?

- Yes, a clear strong link
- Yes, but it could be improved
- No

Question 3 – Notes:

*4. Is there a clear link to theory? **

This can be achieved in many ways, and while it will often be in the introduction, it may show up elsewhere, so keep your eyes peeled. This requires a reference to a specific theory. There are two main ways this could happen: A reference could be made to an established theoretical framework (from the social sciences, from public health, etc.) which has been used to investigate similar questions, or which the authors think might apply in this case. The second possibility is that the authors describe a theoretical framework they have created or adapted themselves which they used in structuring their study, or that they refer to in considering their results.

- Yes, a strong one
- Yes, but a weak one
- No

Question 4 – Notes:

Methodology - Sampling

*5. Is there a description of the sampling procedure? **

Are there details about how sampling was actually conducted? A helpful way to consider this is whether you would be able to conduct this sampling yourself if you were to replicate the study. Every sampling approach has its own logic, but even for the more straightforward qualitative sampling, there should be some mention of this. Note: This

For peer review only - http://bmjopen.bmj.com/site/about/guidelines.xhtml

question does not evaluate whether this sampling is actually appropriate (see question 6). It only asks whether we have a clear idea of the process of sampling, at all.

- Yes, a strong description
- Yes, but a partial description
- No

Question 5 – Notes:

*6. Is the sampling strategy appropriate for the aims of the research? **

This question asks whether the sampling strategy was the right for one for choosing the participants/locations/etc. that could yield relevant information. If there is a strong claim of generalizability, was the sampling appropriate for achieving this? If the study attempts to present the views of a group, did sampling actually capture the people the study needed to hear from? Are there any groups which appear to be missing but clearly should have been spoken to in order to meet the research aims? Has saturation been achieved, and can we tell how this was established? Note that this is not a question of whether the method of collection (interview, focus group, etc.) would have yielded strong data – just whether the people recruited to the study were the right people.

- Yes, very appropriate
- Yes, but not completely appropriate
- No

Question 6 – Notes:

*7. Are sample characteristics sufficiently reported? **

The basic characteristics are often gender, location, ethnicity, and other demographic variables, but consider whether there are other sample characteristics specific to the question which should be reported. This might be clear by this point, but you might also want to look ahead and see whether the paper discusses any relevant characteristics that you would want to know about the sample in general (or you could return to this at the end).

- Yes, there's a sufficient description
- There's some description, but not enough
- No

Question 7 – Notes:

Methodology - Data Collection

*8. Is it clear how data were collected? **

Each method requires its own information. If interviews were used, is there detail on locations, presence of others, length of interview, questions asked, and other such details? For participant observation, is there a description of the

For peer review only - http://bmjopen.bmj.com/site/about/guidelines.xhtml

time spent in the field and the activities partaken in? For focus groups, is there detail on the number of participants, questions asked and setting?

- Yes, data collection is clearly described
- Yes, but more details are needed
- No

Question 8 – Notes:

*9. Are the methods of data recording reported? **

This could be as simple as 'the audio of interviews was taped', 'focus group summaries were written during the discussion', or 'interviews were transcribed, translated, and the translation was then verified'. Any of these would merit at least a 'weak' response, and more detail would be considered 'strong'.

- Yes, and reported well
- Yes, but only briefly mentioned
- No

Question 9 – Notes:

*10. Did the collection of the data address the research aims? **

Given the data needed to answer the question, is the method chosen actually well suited to obtaining such data? If we have knowledge of the questions in an interview guide or a focus group guide, are all the relevant subject areas covered? This isn't about whether there might be some problems in the collection (a bad interviewer could screw up even the best interview guide) but whether the method of data collection, if employed properly, would actually capture relevant data.

- Yes, completely
- Yes, but not entirely
- No

Question 10 – Notes:

Methodology - Analysis

*11. Are the methods of analysis explicitly stated? **

This should include a basic statement as to the approach used (thematic analysis, grounded theory, etc.) but also a breakdown of the steps taken (different rounds of coding, intermittent checks for quality, revisions, etc.)

- Yes, and stated well
- Yes, but more detail is needed
- No

For peer review only - http://bmjopen.bmj.com/site/about/guidelines.xhtml

For peer review only

Question 11 – Notes:

*12. Were there any inbuilt checks to assure the quality of the analysis? **

Some but not all of the following could be present. Was there more than one researcher involved in the analysis? Was there independent peer review during the analysis? Were inter-coder or intra-coder reliability checks conducted? Did the team meet regularly to discuss the analytic process? Did the researcher keep a journal of their thoughts and decisions?

- Yes, there were clear, strong checks throughout
- Yes, but they were weak checks
- No

Question 12 – Notes:

*13. Was there reflection on bias and positionality? **

Have the authors considered their potential biases in relation to the study? This should be a specific statement, and there should be mention of steps that were taken to address this, even if as basic as taking the time to write reflective notes or engaging in a group discussion of the issues.

- Yes, a clear reflection with detail provided
- Yes, but there's only a brief mention of this
- No

Question 13 – Notes:

Methodology - Researchers

14. Are there any details about the people who conducted the sampling, data collection, and analysis?

Not much is required, but look out for mentions of the training these people had, their positions/titles, their experience, etc.

- Yes, plenty
- Yes, a little
- No

Question 14 – Notes:

Results

*15. Is there enough data to support the claims? **

For peer review only - http://bmjopen.bmj.com/site/about/guidelines.xhtml

Variable, but consider whether major claims/themes are supported by more than a single quote or other type of original data. Look at whether the data presented is contextualised, and whether it demonstrates the claim that the authors suggest it does.

- Yes, there's plenty of clear, contextualised, relevant data
- There's some data, but it's weak
- No - there's little or no data presented that is supports the claims.

Question 15 – Notes:

*16. Are diverse viewpoints considered? **

At a basic level, we need to know who speakers are if they are quoted. This could be as minimal as ('22-year-old', or 'Mother, Group A'). Same goes for summaries with no direct quotes – it should be clear whose viewpoint the data comes from. The majority of the data presented will demonstrate the main themes/arguments, but no study is without some dissenting voices or unusual opinions. Are these ever mentioned? If there were many participants, but it appears that most quotes come from one or two individuals, there should be a reasonable explanation for why this is the case – and what it might mean for the study.

- Yes, thoroughly
- Yes, only occasionally
- No

Question 16 – Notes:

*17. Is there evidence which addresses every research aim? **

Look back to the research aims. Is there data relating to each one of them? If not, is there a clear reason for this? If a study ended up not finding data relevant to every aim, but acknowledges that this is the case and tries to account for the reason, this should still be marked as a yes. A 'null' result is still a result - sometimes collection and analysis can be excellent, yet no clear answer is found. If this appears to be the case, the study can still be considered 'strong'.

- Yes, every aim has some related evidence
- Yes, but some aims have distinctly less attending to them
- No

Question 17 – Notes:

Discussion

*18. Has the question been answered? **

A simple test is to go back to the research aims, formulate them as questions (if they're not already established as such) and see whether the authors have provided you with answers that are reasonable given the data presented. It should be clear how the data presented is appropriate and convincing evidence for the conclusion. In a strong paper,

For peer review only - http://bmjopen.bmj.com/site/about/guidelines.xhtml

the authors will make a clear and logical statement of how the results provide an answer to the question. A weaker paper might not cover all the questions, or might suggest questionable links between the data in the results and the conclusions reached.

- Yes, with a clear, logical, thorough answer
- Yes, but not well
- No

Question 18 – Notes:

*19. Are relevant literature, theory, or practice discussed in relation to the results? **

A good paper doesn't need to discuss all of these, but it should relate to at least one. Did it match up or challenge a theory? Does it have implications for practice? Are the results now situated within the broader literature?

- Yes, and discussed well
- Yes, but only briefly or generally discussed
- No

Question 19 – Notes:

*20. Has there been any triangulation? **

This takes many forms, but the basic question is – do the authors present any other sources of data which support or contradict their claims? These could be quantitative/qualitative/mixed studies, some form of evaluation of the conclusions by the participants themselves, comparison to other data recorded, etc. This could be in the results section or the discussion, so have another look.

- Yes, strong triangulation
- Yes, weak triangulation
- No

Question 20 – Notes:

*21. Are weaknesses considered? **

Mentioning them is a good step, but a strong paper will also argue how these weaknesses were attended to.

- Yes, major weaknesses considered and attended to
- Yes, but weaknesses are mentioned without much discussion
- No

Question 21 – Notes:

For peer review only - http://bmjopen.bmj.com/site/about/guidelines.xhtml

Ethics

22. Have ethical issues been taken into consideration? *

At the very basic level, there should be a description of informed consent. Also look for approval from an IRB (or other ethics committee as applicable), and whether issues of benefit/harm and confidentiality/secrecy were discussed with participants.

- Yes
- No

Question 22 – Notes:

General comments and thoughts:

Remember to look back at the questions before submitting.

Having read the paper as a whole, and having considered each question separately, you might now also have a clearer idea as to the answers to some questions. Have another look, particularly for questions where you noted some thoughts or concerns. You may want return to question 7 in particular, as you may now have a clearer idea of what sample characteristics should have been reported.

For peer review only

Cost evidence risk of bias tool

S.No.	Questions	Response
1a.	Is the form of economic evaluation clearly stated? Indicate (CBA/CEA/CUA/cost-minimization, or innovative approaches: Social Return on Investment (SROI), Multi-Criteria Appraisal (MCA). Response options [CBA, CEA, CUA, SROI, MCA, or N/A if only "total cost", also ok to insert descriptive results that do not conform, e.g. “cost transfer ratio”]	Yes, No
1b.	Is the perspective of the costing stated?	Yes, No
1d.	Cost data sources?	Yes, No
1e.1	Are unit costs reported (in Table)	Yes, No
1e.2	Are cost ingredients listed?	Yes, No
1e.3	Total cost description	Yes, No
1f.	Total cost reported?	not reported, Value if reported
1h.	Was cost per child immunised reported?	Yes, No, If yes, Cost
1j.	What exchange rate was used for currency adjustments? [e.g. 1.2 USD to 1 Euro]	value if given, Not reported
1j.	Exch rate year (estimated)	Year
1k.	Is time horizon for costs clearly stated? [Operationalise - was a base year of the costing reported?	Yes, No, If yes: year
7a.	Sensitivity analysis? [yes/ no]	n/a, Description if yes

For peer review only - http://bmjopen.bmj.com/site/about/guidelines.xhtml

Appendix 8: Risk of bias in the included studies

Quantitative risk of bias

Risk of bias in Randomised Controlled Trials

Of the included studies with experimental designs (k = 31) the majority (k = 23) have been identified as being at high risk of bias, and only two were rated as having a low risk of bias. Six studies were assessed as having some concerns. Of the six potential causes of bias that were analysed, outcome measurement bias and deviations from intended interventions were the most commonly documented issues. The most common pitfall related to outcome measurement bias was the use of caregiver reported (self-reported) measures of vaccinations received by a child in the absence and/or incompleteness of immunisation cards, which is common in L&MICs and is often influenced by the intervention itself. One such study stated that "The calculation of FIC coverage was based on data from the child's immunization card and/or the mother's recall. Mother's recall was taken in situations in which the card or specific data points on the card were missing.", (Gurley 2020). Relatedly, Demilew, (2021) reported that their outcome measures "...could be subject to recall bias or social desirability bias differentially by treatment group…". Deviations from intended interventions had to do with issues such as potential spillover related to geography (e.g. in Siddiqi 2020, centers were contiguously located) or transferable vouchers (e.g. Morris 2004). Other sources of bias in included RCT studies are reporting bias, and bias in the randomisation process. Supplementary Figure 2a illustrates the frequencies with which each cause of bias was identified across the 31 included RCTs, while Supplementary Figure 2b shows the risk of bias assessments for each individual study and each risk of bias category for RCT studies.

Supplmentary Figure 2a: Frequencies of Bias in RCTs

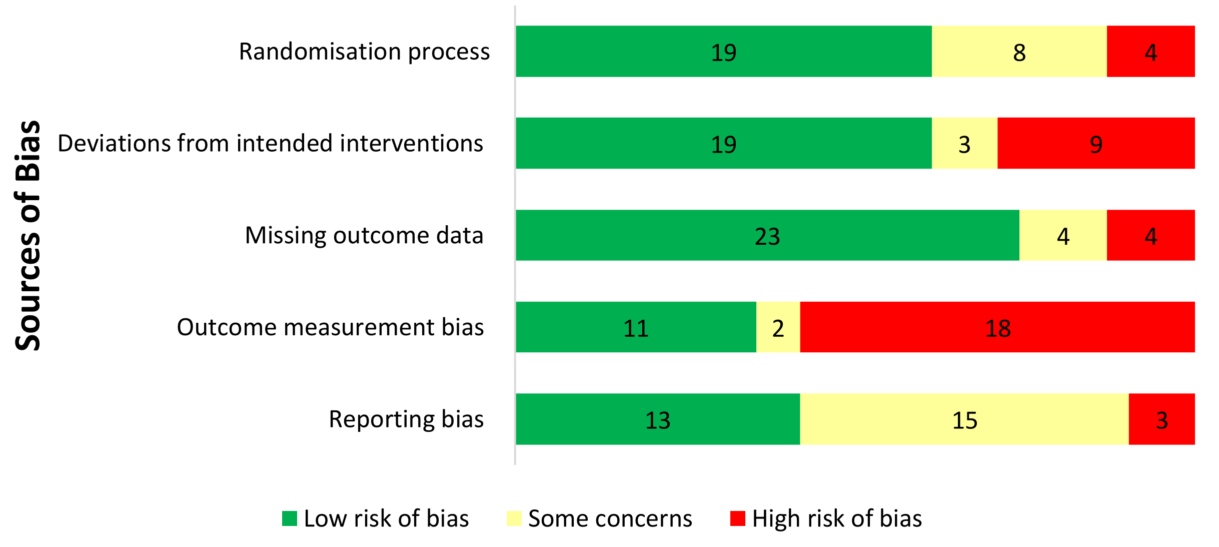

Supplementary Figure 2b: Risk of bias assessments for individual RCT studies

Study first author	Assignment mechanism: Was the allocation or identification mechanism random or as good as random?	Unit of analysis: Is unit of analysis in cluster allocation addressed in standard error calculation ?	Bias due to missing outcome data	Was the study adequately protected against spill-overs, cross-overs, contamination, and performance bias?	Outcome measurement bias: Was the study free from biases in outcome measurement?	Analysis reporting: Was the study free from selective analysis reporting?	Overall Risk of Bias Assessment
Alhassan, 2019							
Andersson, 2009							
Arifeen, 2009							
Banerjee, 2010							
Banerjee, 2020							
Björkman, 2009							
Bolam, 1998							
Borkum, 2014							
Demilew, 2020							
Domek, 2019							
Engineer, 2016							
Gibson, 2017							
Gurley, 2020							
Johri, 2018							
Lee, 2015							
Modi, 2019							
Mohanan, 2020							
More, 2012							
More, 2017							
Morris, 2004							
Murthy, 2019							
Nagar, 2018							
Nagar, 2020							
Olken, 2014							
Oyo-Ita, 2020							
Pramanik, 2020							
Rahman, 2008							
Robertson, 2013							
Seth, 2018							
Siddiqi, 2020							
Webster, 2019							

Note: Green cells signify low risk of bias, yellow cells signify some concerns related to risk of bias, and red cells signify high risk of bias.

For peer review only - http://bmjopen.bmj.com/site/about/guidelines.xhtml

Bias in Quasi-Experimental Designs

Of the 30 included quasi-experimental studies, the majority (k = 27) present a high risk for bias, two were assessed as low risk of bias and one study was assessed as having some concerns. Like the experimental studies the most common identified causes of bias amongst quasi-experimental designs were issues related to outcome measurement bias and deviations from intended interventions. This is no surprise as self-reporting was again a common concern (e.g. Admassie, 2009 and Findley, 2013). Supplementary Figure 3a illustrates the frequencies of other biases in our included QEDs, while Supplementary Figure 3b shows the risk of bias assessments for each individual study and each risk of bias category for QED studies.

Supplementary Figure 3a: Frequencies of Bias in QEDs

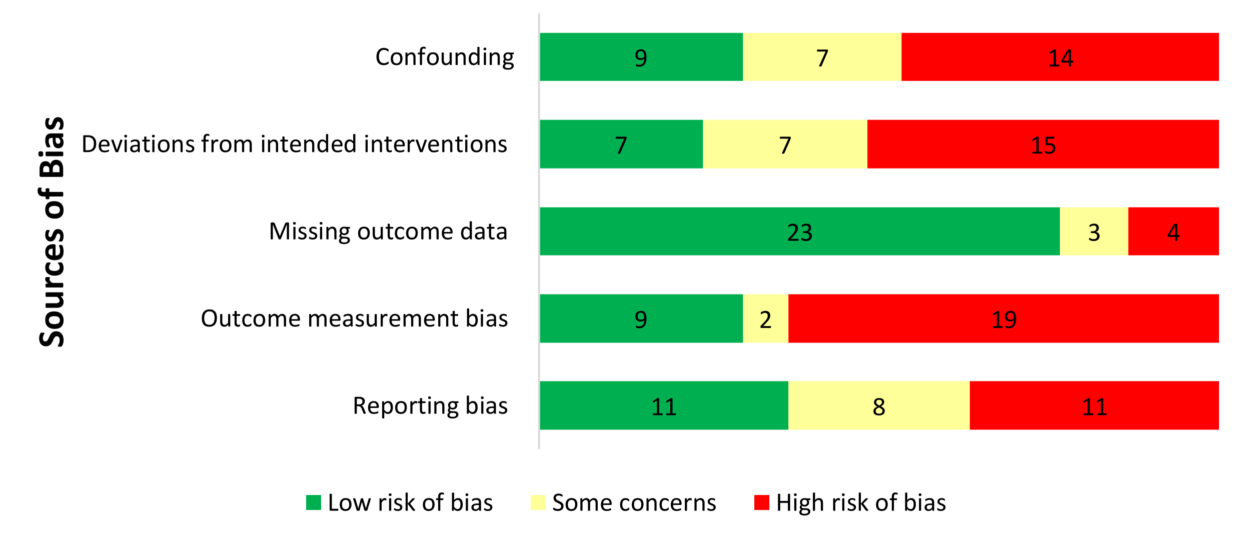

Supplelmentary Figure 3b: Risk of bias assessments for individual QED studies

Study first author	Was the identification method free from any sources of bias	Bias due to missing outcome data	Was the study adequately protected against spill-overs, cross-overs, contamination, and performance bias?	Outcome measurement bias: Was the study free from biases in outcome measurement?	Analysis reporting: Was the study free from selective analysis reporting?	Overall Risk of Bias Assessment
Adamu, 2019						
Admassie, 2009						
Assegaai, 2018						
Banwat, 2015						
Biemba, 2016						
Calderón-Ortiz, 1996						

For peer review only - http://bmjopen.bmj.com/site/about/guidelines.xhtml

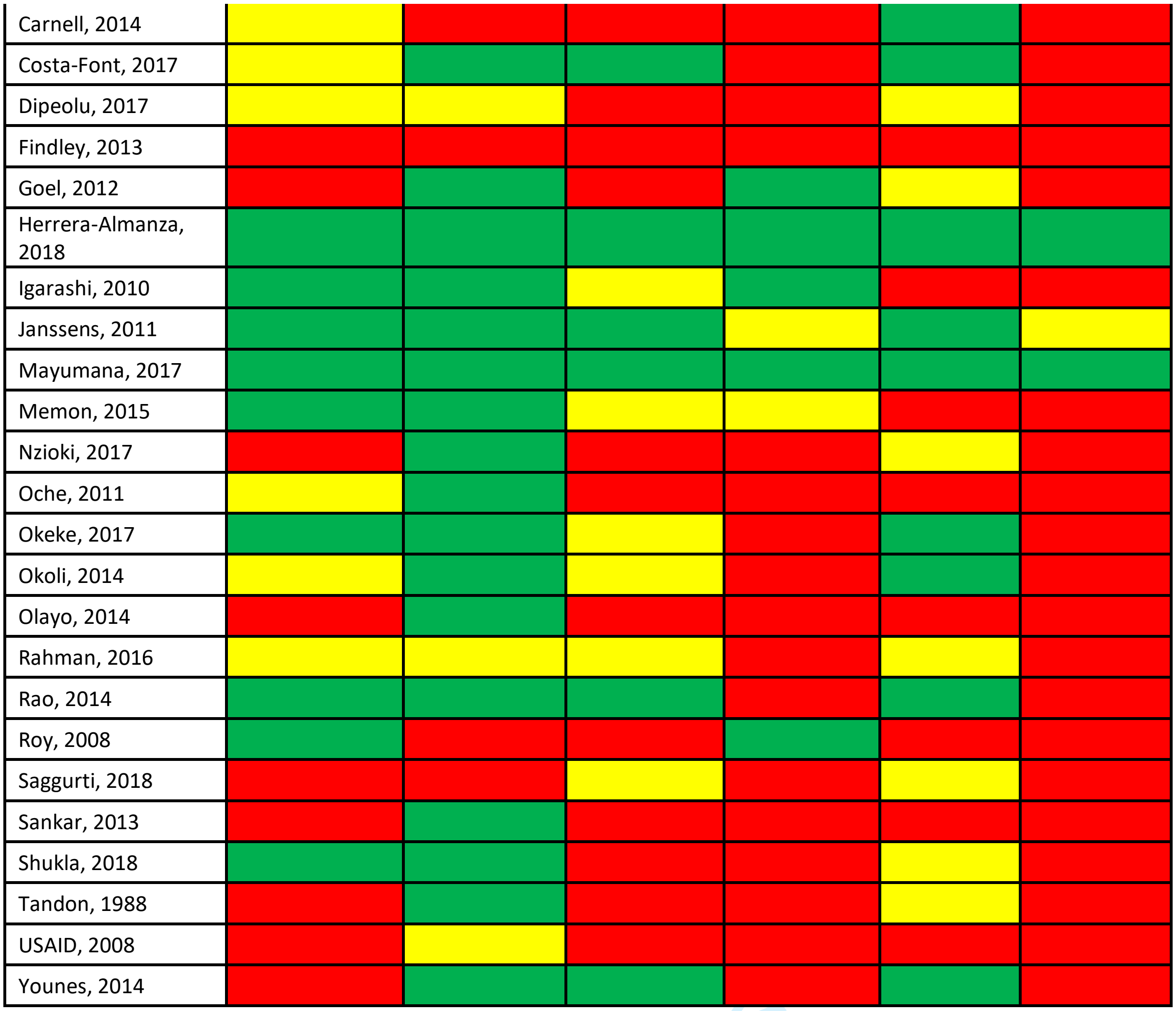

Qualitative risk of bias

Risk of bias assessments were conducted on 47 qualitative papers. Papers were scored as absent, weak, or present on 12 key elements and received corresponding scores of zero, one, or two for these ratings. Most (27) papers were missing at least some key elements, resulting in them receiving a zero value for these elements in their quality assessment scores (Supplementary Table 1; Supplementary Figure 4). However, 17 received quality assessment scores over 20, indicating that they received a value of two, or a "strong" rating, for most key elements (Supplementary Figure 5). The most common key elements to be missing were descriptions of sample characteristics and the analytic methods, with 17 studies failing to report on each of these (Supplementary Figure 5). The research aim was the most common key element to be stated strongly, with 39 papers clearly stating their aims.

Supplementary Table 1: Distribution of papers by strength of key elements

	Number
One or more key elements absent	27
One or more key elements weak	15

For peer review only - http://bmjopen.bmj.com/site/about/guidelines.xhtml

All key elements strong	5

Supplementary Figure 4: Distribution of papers by 12 key elements

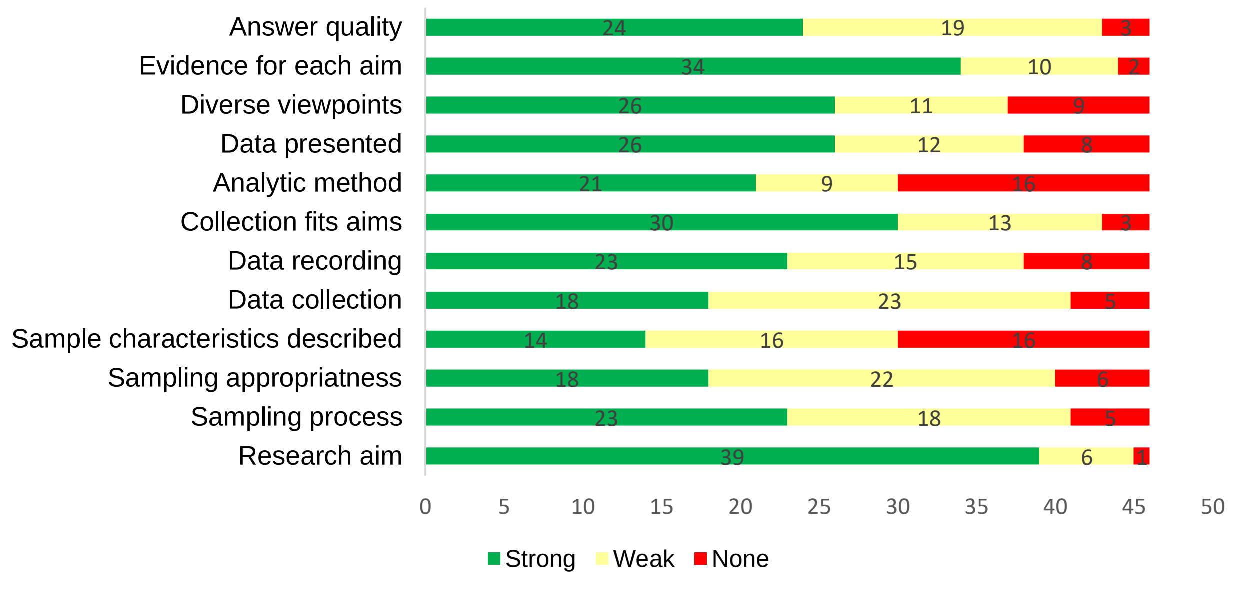

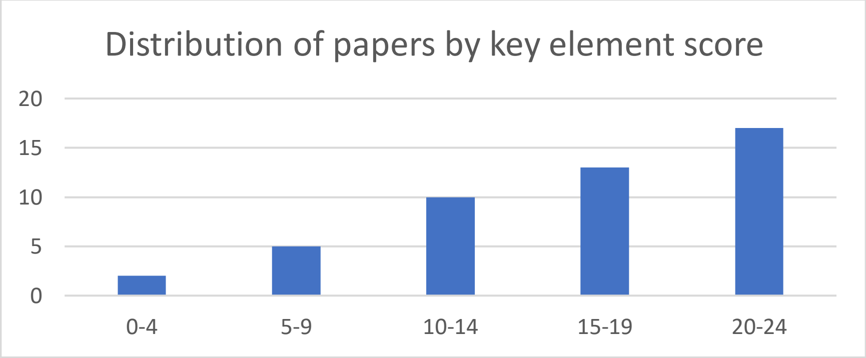

*Papers received scores of 0, 1 or 2 reflecting the strength of reporting on 12 key elements in the risk of bias assessment tool (Appendix E).

Supplementary Figure 5: Distribution of qualitative assessment scores*

Risk of bias in cost effectiveness estimates

The risk of bias analysis of the cost and cost-effectiveness analysis assessed the quality of underlying cost data, reporting, and analysis using information that was reported in the immunization studies. We assessed risk of bias along six primary dimensions which were adapted from a combination of tools, including: Doocy and Tappis (2017); Campbell Collaboration Economic Methods Policy Brief (Shemilt et al.,2008); and Methods for the Economic Evaluation of Health Care Programmes, (Drummond et al. 2015), that were adapted for cost analyses carried out in conjunction with impact evaluation studies of global development interventions.

For peer review only - http://bmjopen.bmj.com/site/about/guidelines.xhtml

This risk of bias tool specifically assesses the bias that arises from the collection and reporting of cost analysis in conjunction with impact evaluation studies of global development interventions. These studies often do not incorporate cost analyses. Indeed recent estimates suggest just 15-18% of impact analyses include any kind of cost analysis (Brown and Tanner 2019). This under-reporting of cost in conjunction with impact evaluations leads to very small samples from which to draw inferences about the cost, and cost-effectiveness of development interventions. Moreover, often when estimates are included with impact evaluations, the quality of the underlying data is low. Cost may have been added as an “after-thought”, rather than planned for in advance or treated as a research endeavor. Data sources for cost information may not be well-documented or may have been estimated using ‘back-of-the-envelope’ techniques (this phrase literally is used in write-ups of cost methods). There is often insufficient detail in reporting of cost to assess the quality of estimates and data and basic robustness checks of the analysis are very infrequently performed.

Finally, this tool specifically considers the elements of cost and effectiveness that were extracted for the analysis of incremental cost effectiveness. Our particular measure estimates the non-vaccine cost per dose of interventions to increase absolute immunization coverage by one percent, using available data extracted from the evaluations wherever possible, or by following the steps and calculations as outlined in Ozawa et al. (2018). The specific inputs to this analysis include estimates of: total intervention cost (net of vaccine cost); the number of vaccine doses provided to each child; the endline proportion of children that received immunisations in the treatment and control groups; and the cost per child immunised.

Dimensions of potential bias in costs reported and cost-effectiveness estimates

Planned, organized, cost analysis. A common challenge of cost analysis is a lack of planning which often can lead to poor underlying data quality. For example, cost estimates are subject to recall bias when assessed long after the program and evaluation are completed. Therefore, we examine three indicators of an organized or planned cost analysis. Specifically, we look for a clear description of the form of economic evaluation, and a description of the method used. We also look for a clear statement of analytical perspective--which is the choice of which actor has standing in the costing -- this may be the donor, the implementing partner, or perspective of the costing may be from a societal point of view. The analytical perspective is important because it determines whose costs and benefits will be counted in the costing. It may, for example, directly impact how the constituent components of total cost are counted. Although we considered a standard ROB question which asks: ‘Is a well-defined research question posed in answerable form?’, we find that impact evaluation studies do not pose research questions in the expected form. The three questions, given equal weight in the risk of bias assessment, were:

a. Is the form of economic evaluation clearly stated?
b. Is the perspective of the costing stated?
c. Was a method of costing described?

Quality data sources. The highest quality data for assessing the cost of interventions in low-and middle-income settings often are drawn from the expenditure reports or accounting statements of the program implementers. Expenditures are better quality because they represent actual, rather than planned expenses and they often are subject to audit, making them more reliable as compared with cost data taken from program budgets (Levin et al. 2018).

d. What is the quality of the primary data sources used for the cost estimates?

Descriptive, detailed cost information. The quality of descriptive detail on costs allows judgement into cost components and whether they align with intervention activities. Since the three criteria tend to be positively correlated, we give equal weight to the three indicators of detailed cost information. Specifically, we assessed the descriptive detail of reported costs:

e. Whether costs are reported by ingredients (or input or resource),
f. Whether unit costs are reported, and

For peer review only - http://bmjopen.bmj.com/site/about/guidelines.xhtml

g. Whether the information is presented in an organized cost table

We consider the **quality of cost estimates** that were key inputs to the cost synthesis, specifically

h. Total cost: What is the quality of the specific data components of total cost? Since total cost is a key input to the analysis we examine how it was reported. For example, some studies say simply, the total cost of the program was USD $3 Million. In the absence of descriptive information, i.e. the elements that comprise total cost, it is difficult to assess the quality or reliability of the total cost estimate.
i. Vaccine cost: Can we tell if vaccines were excluded? Since we need to exclude vaccine cost, it must be excluded or reported separately from total cost to be valid in this study.
j. Cost per child immunized: If cost per child immunized was not directly estimated and reported, we take total cost divided by the number of children in the treatment group multiplied by the proportion of children that were immunized. If the quality of underlying data are poor, this estimate may be biased – with unclear magnitude and direction.
k. Number of vaccines. Was the number of vaccines reported from observation of study participants, or was it reported per protocol? When we estimate cost per vaccine dose, the cost estimate will be biased downwards (less costly per dose) if the study does not report the actual number of doses administered. This is because some children may already have received a part of the vaccine protocol before enrolling in the study.

Are **key cost details** reported which allow us to adjust for time and currency differences? In this assessment of bias risk, the responses are given equal weight since we need both elements to accurately adjust for time and currency differences.

l. Are details of inflation and currency conversion clearly stated?
m. Is the time horizon for costs clearly stated?

We consider the **quality of cost analysis**, specifically:

n. Is sensitivity analysis conducted?

Results

We developed evaluation criteria and applied the protocol described above to the 22 immunisation evaluations that reported cost information. The results of this analysis are summarized below in Supplementary Figure 6, below.

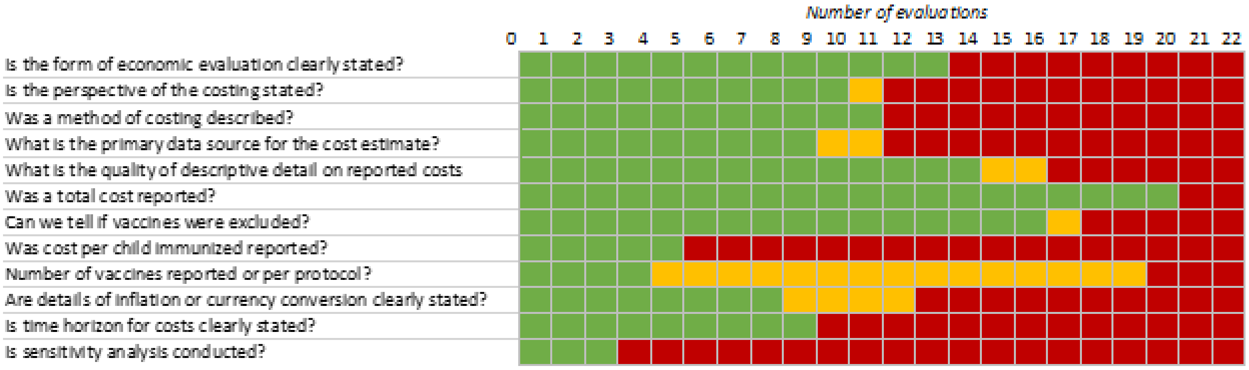

Note: green cells indicate that clear information was presented in the report to assess the risk of bias; yellow cells indicate incomplete information; and red cell indicate we found very little or no information to assess bias risk.

For peer review only - http://bmjopen.bmj.com/site/about/guidelines.xhtml

Supplementary Figure 6: Risk of bias assessment of cost and cost-effectiveness estimates used in the analysis

- *Just over half of the included evaluations appear to have carried out a planned, organized, cost analysis.* Thirteen of 22 evaluations clearly state the form of economic evaluation (i.e. cost-effectiveness analysis); ten studies report the perspective of the costing, which is key for judging the correct inclusion and exclusion criteria for the components of a total cost estimate; and eleven of 22 evaluations describe the method of costing that was used to collect cost data (i.e. the ingredients method).
- *Indications of the quality of underlying cost data are mixed.* Nine of the 22 evaluations used expenditure reports to generate cost estimates, two used budgets and the remaining 11 evaluations provided no information on the provenience of the underlying cost data that was used in the analysis.
- *The quality of the descriptive detail on reported costs was mixed. Just over half of all evaluations (14 of 22)* provided thorough, descriptive information on costs; two evaluations provided some descriptive information, e.g. a breakdown of key unit costs; and six evaluations gave very minimal or no descriptive information on costs.
- *In 17 of 22 evaluations, we have high confidence that total cost excludes vaccines costs.* A majority of evaluations reported an estimate of total cost (20 of 22 evaluations). In 16 of the 20 total cost estimates, there were clear indications that vaccine costs had been excluded from the estimate, and in one case, vaccine costs were included but reported separately so that we could subtract vaccine cost from total cost to derive the comparable total cost. It was not possible to tell if vaccines were excluded from the total cost estimates of five evaluations.
- *The cost per immunized child* – was only reported by the authors in five of 22 evaluations and for the remaining 16 was estimated by the authors to compile Supplementary Table 22.
- *The number of vaccine doses received per treated child was reported in only four evaluations;* in 15 studies, vaccine doses per child were estimated based on information "per protocol" which does not account for children's partial vaccination status at the point of enrollment in the study.
- *Fewer than half of the evaluations included the key information needed to adjust for time, currency, inflation or base year differences in the timing of expenditures.* This lack of reporting makes it very difficult to have complete confidence in the comparability of the cost estimates we generated.
- *Only three evaluations reported any kind of sensitivity analysis*, an indication of analytical robustness.

For peer review only - http://bmjopen.bmj.com/site/about/guidelines.xhtml

Appendix 9: Quantitative results – all community engagement interventions

Appendix A presents supplementary data for analyses presented in the main body of the manuscript for the primary outcomes of community engagement interventions, including funnel plots of publication bias and outlier analyses. It also includes a full presentation of the supplementary outcomes that were precluded from the main body of the manuscript due to word limitations.

Primary outcomes

Full immunisation

As a robustness check, we used robust variance analysis and included all dependent effects in the analysis, totaling 53 effects from the same 28 studies (*df* = 25.5). The overall average effect was slightly smaller but still significant ($\hat{\mu} = 0.11$ [95% CI: 0.04 to 0.18], *p* = .002). Sensitivity analyses show the effect to be sensitive to all values of Rho. The rank correlation test indicated funnel plot asymmetry ($p = 0.03$) but the regression test did not ($p = 0.57$; see Supplementary Figure 7). A trim and fill analysis indicated an identical effect size (see Supplementary Figure 7b)

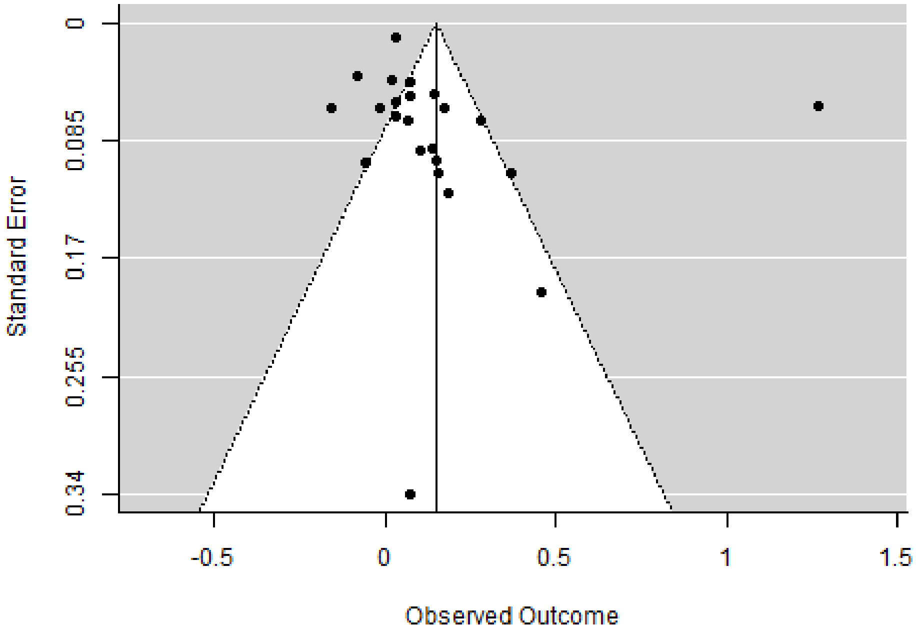

Supplementary Figure 7. Funnel plot for studies examining the effect of community engagement interventions on full immunisation

For peer review only - http://bmjopen.bmj.com/site/about/guidelines.xhtml

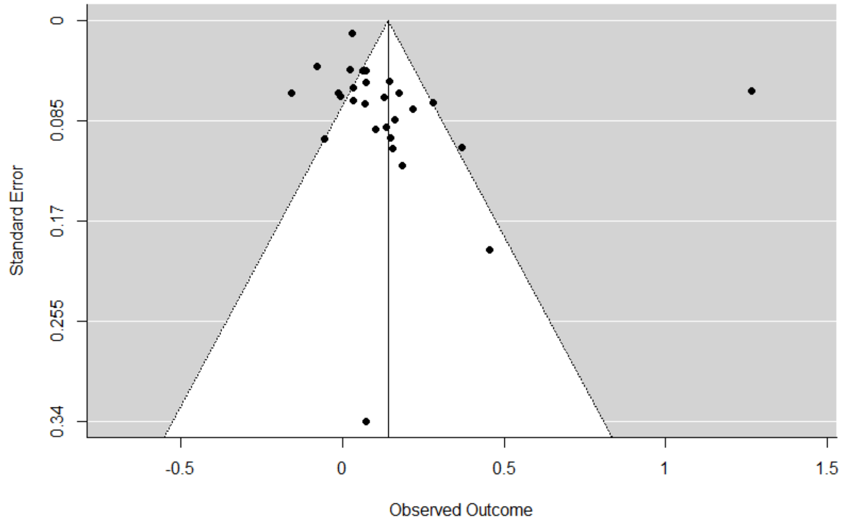

Supplementary Figure 7b. Trim and fill plot for studies examining the effect of community engagement interventions on full immunisation

DPT3

According to the Q-test, the true outcomes appear to be heterogeneous ($Q(21) = 90.43$, $p < 0.01$, $\hat{\tau}^2 = 0.01$, $I^2 = 76.78\%$). The rank correlation test indicated funnel plot asymmetry ($p = 0.04$) but not the regression test ($p = 0.06$), but trim and fill analyses indicated an identical effect size (Supplementary Figures 8 & 9). An examination of the studentized residuals revealed that none of the studies had a value larger than ± 3.05 and hence there was no indication of outliers in the context of this model. According to the Cook's distances, none of the studies could be considered to be overly influential. Leave one out analyses indicated that no single study was driving this result.

Publication year was a significant moderator such that each additional year reduced the size of the effect by .014 standard deviation units ($\hat{B} = -0.014, p = 0.019$ [95% CI: -0.03 to -0.002]). In other words, new studies have found smaller effects. There were no other significant moderators in the context of this model.

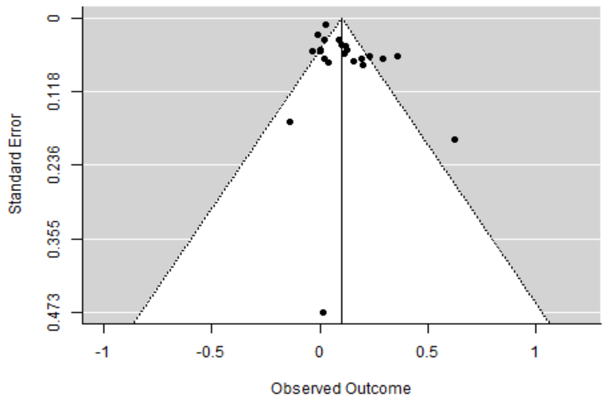

Supplementary Figure 8. Funnel plot for studies examining the effect of community engagement interventions on DPT3 vaccination

For peer review only - http://bmjopen.bmj.com/site/about/guidelines.xhtml

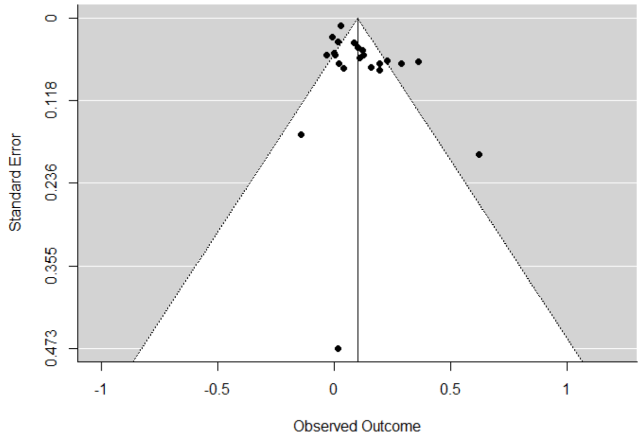

Supplementary Figure 9. Trim and fill plot for studies examining the effect of community engagement interventions on DPT3 vaccination

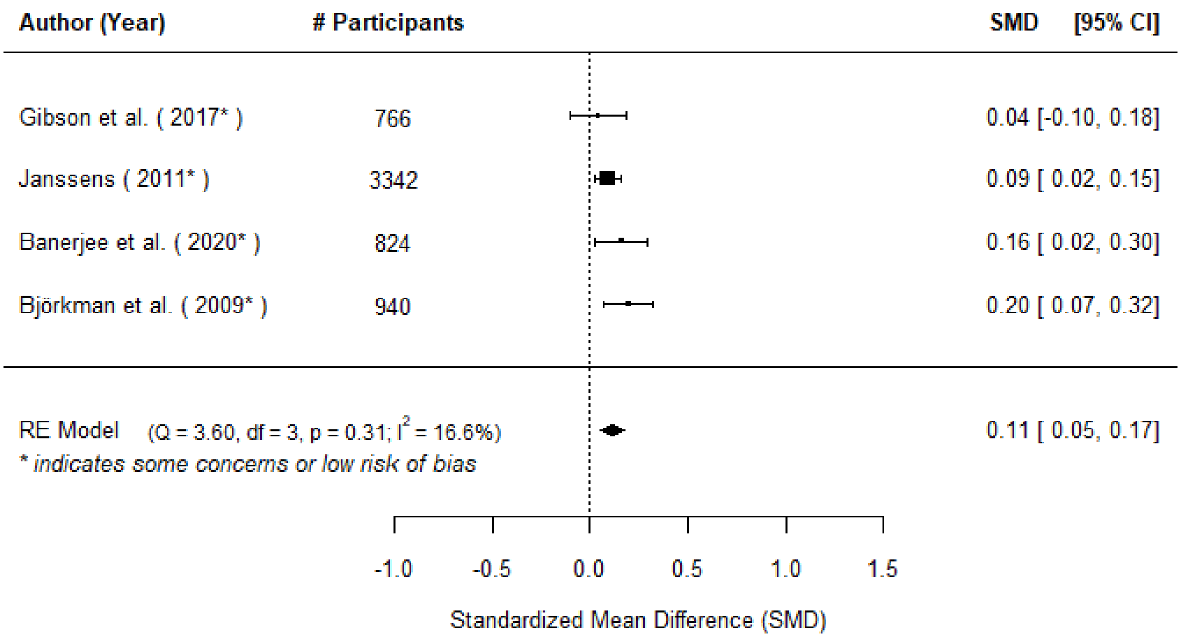

Supplementary Figure 10. *Forest plot showing the observed outcomes and the estimate of the random-effects model for the impact of community engagement interventions on DPT3 vaccination when low quality studies are removed*

As a robustness check, we used robust variance analysis and included all dependent effects in the analysis, totaling 36 effects from the same 22 studies (df = 18.2). The overall average effect was slightly smaller but still significant ($\hat{\mu}$ = 0.10 [95% CI: 0.05 to 0.15], p < .001). Sensitivity analyses show the effect to be sensitive to all values of Rho.

Measles

An examination of the studentized residuals revealed that one study (Sankar 2013) had a value larger than ±3.02 and may be a potential outlier in the context of this model. Indeed, sensitivity analysis leaving out Sankar (2013) would result in an increase in the average effect ($\hat{\mu}$ = 0.08 [95% CI: 0.05 to 0.12], and it was still statistically

For peer review only - http://bmjopen.bmj.com/site/about/guidelines.xhtml

significant ($z = 4.44$ $p < 0.001$). Leave one out analyses indicated that no other study was driving this result. According to the Cook's distances, none of the studies could be considered to be overly influential. A funnel plot of the estimates is shown in Figure A6. Both the rank correlation and the regression test indicated potential funnel plot asymmetry ($p = 0.05$ and $p = 0.02$, respectively). The trim and fill analysis (Supplementary Figure 13) estimates a smaller and non-significant effect ($\hat{\mu} = 0.02$ [95% CI: -0.02 to 0.06], $p =$ .36 Supplementary Figure 11 presents the results when low quality studies are removed.

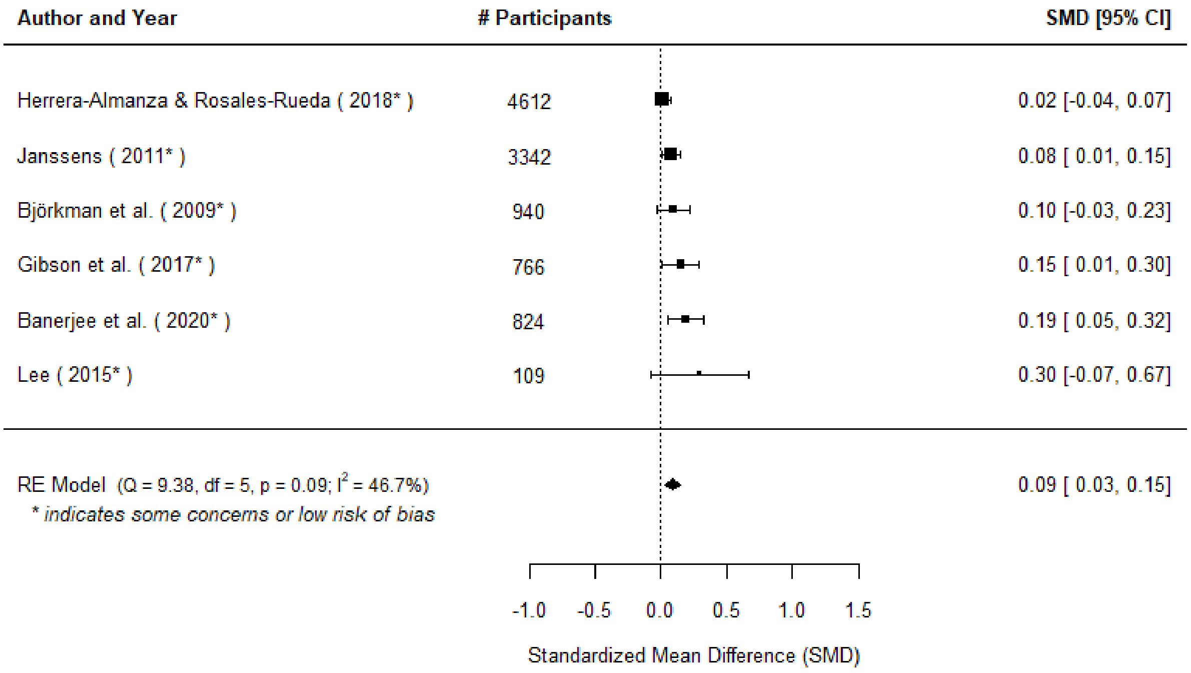

Supplementary Figure 11. Forest plot showing the observed outcomes and the estimate of the random-effects model for the impact of community engagement interventions on measles vaccination when low quality studies are removed

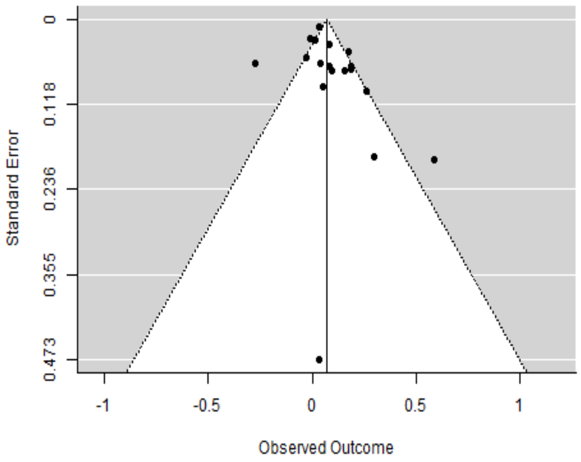

Supplementary Figure 12. Funnel plot for studies examining the effect of community engagement interventions on measles vaccination

For peer review only - http://bmjopen.bmj.com/site/about/guidelines.xhtml

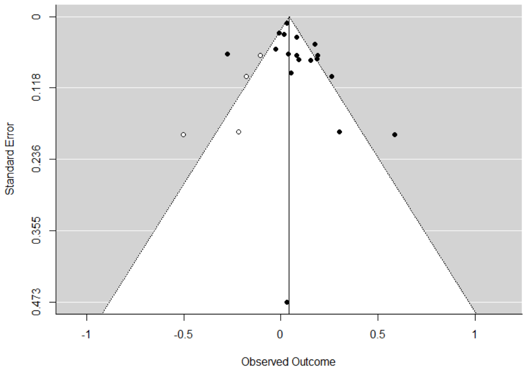

Supplementary Figure 13. Trim and fill funnel plot for studies examining the effect of community engagement interventions on measles vaccination

As a robustness check, we used robust variance analysis and included all dependent effects in the analysis, totaling 32 effects from the same 20 studies (*df* = 15.5). The overall average effect was slightly smaller but still significant ($\hat{\mu} = 0.06$ [95% CI: 0.01 to 0.11], p = .03). Sensitivity analyses show the effect to be sensitive to all values of Rho.

Vaccination timeliness: Full immunisation schedule

We included a total of $k = 5$ studies in the analysis. The estimated average outcome was $\hat{\mu} = 0.15$ ([95% CI: 0.07 to 0.24], $z = 3.41$, $p < 0.001$; $Supplemenyary\ Figure$ 14), indicating a small but significant benefit to the treated group compared to the control group. According to the Q-test, there was no significant amount of heterogeneity in the true outcomes ($Q(4) = 4.43$, $p = 0.35$, $\hat{\tau}^2 = 0.00$, $I^2 = 9.66\%$). An examination of the studentized residuals revealed that none of the studies had a value larger than ± 2.58 and hence there was no indication of outliers in the context of this model. This was confirmed by a leave-one-out analysis. According to the Cook's distances, none of the studies could be considered to be overly influential. With only one study not assessed as high risk of bias, we were unable to conduct sensitivity analyses by study quality. With no significant heterogeneity, we did not test for moderations. There were no dependent effects, so we could not use RVE as a robustness check in this case.

For peer review only - http://bmjopen.bmj.com/site/about/guidelines.xhtml

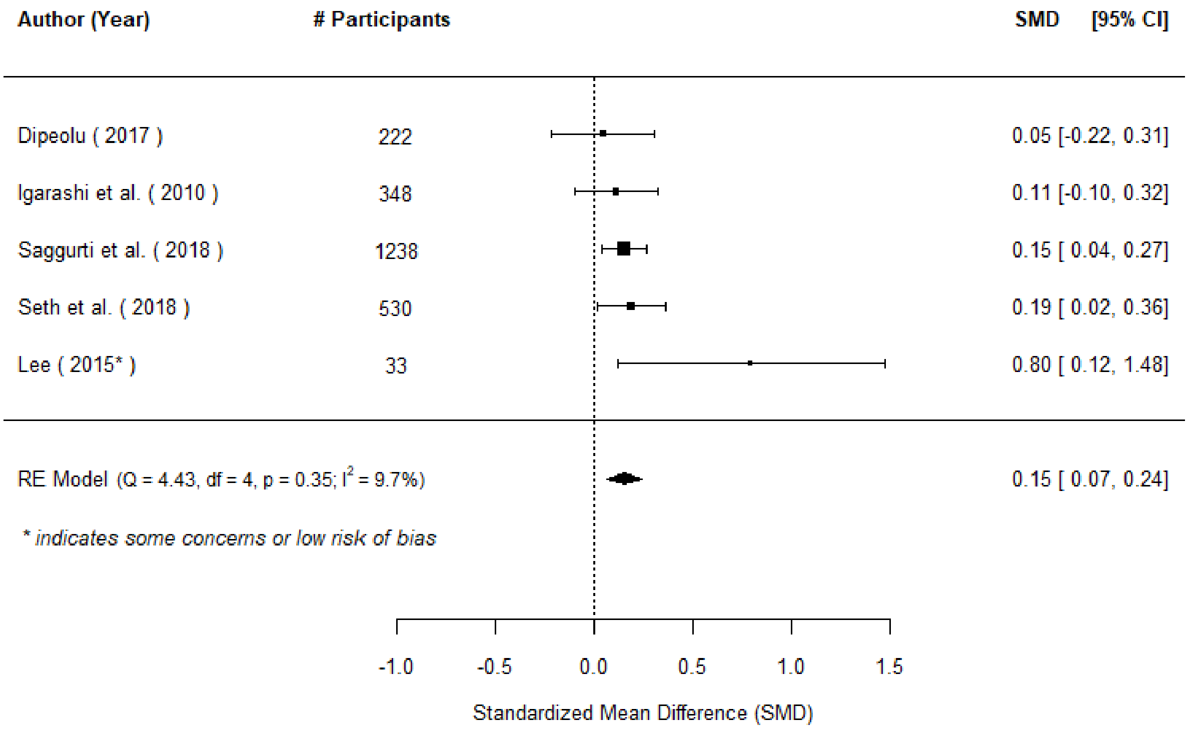

Supplementary Figure 14. Forest plot showing the observed outcomes and the estimate of the random-effects model for the impact of community engagement interventions on timeliness of full childhood immunisation

Vaccination timeliness: DPT3

A total of $k = 7$ studies examined the relationship between community engagement interventions and timeliness of DPT3 vaccination. The estimated average outcome was $\hat{\mu} = 0.09$ ([95% CI: 0.03 to 0.14], $z = 3.00$, $p < 0.01$), indicating a small but significant benefit to the intervention group compared to the control group (Supplementray Figure 15). According to the Q-test, there was no significant amount of heterogeneity in the true outcomes ($Q(6) = 5.09$, $p = 0.53$, $\hat{\tau}^2 = 0.00$, $I^2 = 0.00\%$). An examination of the studentized residuals revealed that none of the studies had a value larger than ± 2.69 and hence there was no indication of outliers in the context of this model. This was confirmed by a leave-one-out analysis. According to the Cook's distances, none of the studies could be considered to be overly influential. With no heterogeneity, moderator analyses were not appropriate. There were no dependent effects, so we could not use RVE as a robustness check in this case. When low quality studies are removed, only two studies remain, and the estimated average outcome increases to $\hat{\mu} = 0.11$ [95% CI: -0.05 to 0.27], but becomes non-significant $z = 1.32$, $p = 0.19$). However, with only two studies this should be interpreted with caution.

For peer review only - http://bmjopen.bmj.com/site/about/guidelines.xhtml

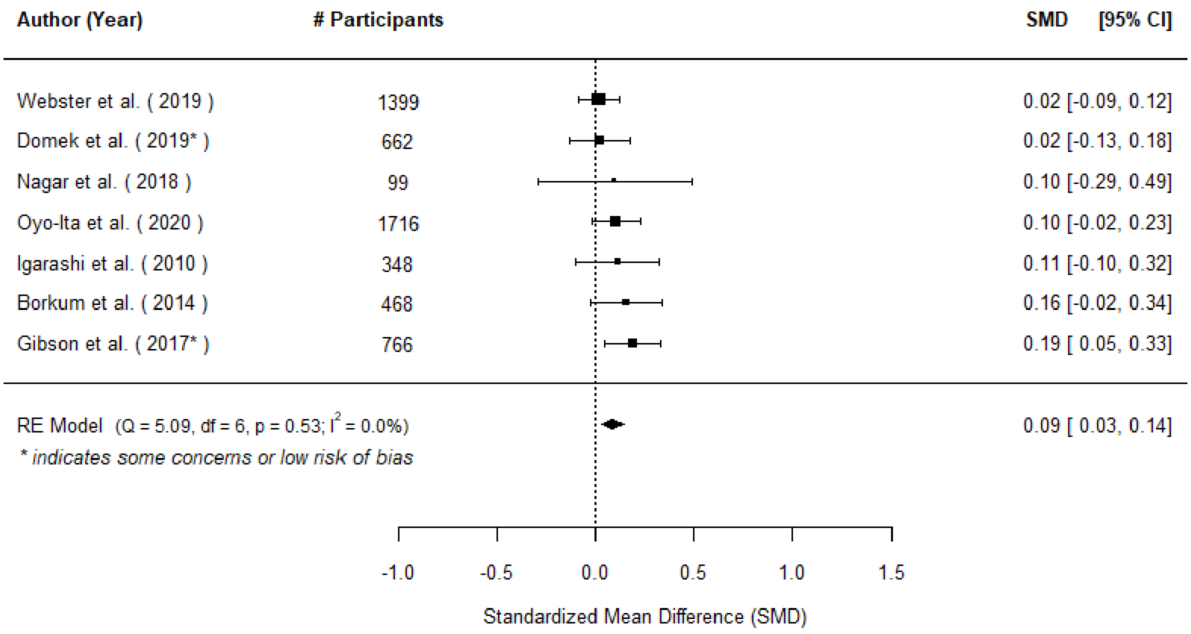

Supplementary Figure 15. *Forest plot showing the observed outcomes and the estimate of the random-effects model for the impact of community engagement interventions on the timeliness of DPT3 vaccination.*

Vaccination timeliness: Measles

Only $k = 2$ studies using community engagement interventions reported on the timeliness of measles vaccinations. The estimated average outcome was $\hat{\mu} = 0.23$ ([95% CI: 0.14 to 0.32]$, z = 5.06, p < 0.001; see$ Supplementary $Figure$ 16), indicating a small but significant benefit to the treated group compared to the control group. According to the Q-test, there was no significant amount of heterogeneity in the true outcomes ($(Q(1) = 0.23., p = 0.63, \hat{\tau}^2 = 0.00, I^2 = 0.00\%$). An examination of the studentized residuals revealed that none of the studies had a value larger than ± 2.69 and hence there was no indication of outliers in the context of this model. Leave-one-out analyses were not conducted as there were only two studies. According to the Cook's distances, none of the studies could be considered to be overly influential. With no heterogeneity and only two studies contributing effects, moderator analyses were not appropriate and we were unable to conduct sensitivity analyses by study quality or test for publication bias. There were no dependent effects, so we could not use RVE as a robustness check in this case.

For peer review only - http://bmjopen.bmj.com/site/about/guidelines.xhtml

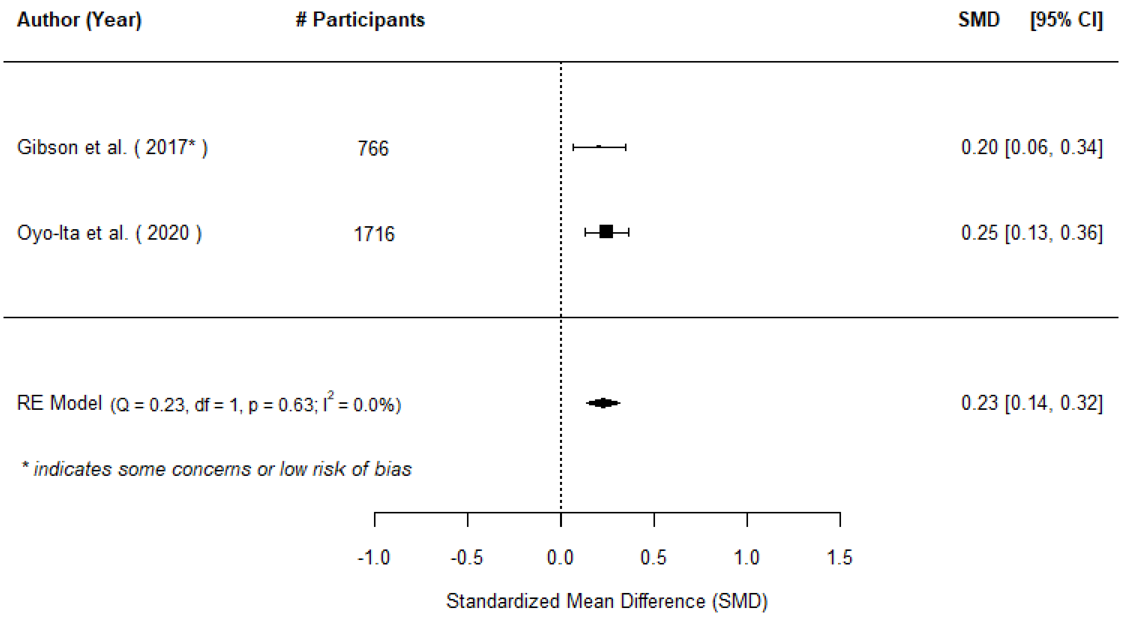

Supplementary Figure 16. *Forest plot showing the observed outcomes and the estimate of the random-effects model for the impact of community engagement interventions on the timeliness of measles vaccination.*

Secondary outcomes

Partial immunisation

We included a total of $k = 9$ studies the analysis. The estimated average outcome based on the random-effects model was $\hat{\mu} = 0.23$ ([95% CI: 0.09 to 0.37], $z = 3.15$, $p = 0.002$), indicating a benefit for the intervention group compared to the control group (see Supplementary Figure 17). A 95% credibility/prediction interval for the true outcomes is given by -0.20 to 0.66. Hence, although the average outcome is estimated to be positive, in some studies the true outcome may in fact be negative.

According to the Q-test, the true outcomes appear to be heterogeneous ($Q(8) = 219.11$, $p < 0.001$, $\hat{\tau}^2 = 0.04$, $I^2 = 96.351\%$). An examination of the studentized residuals revealed that none of the studies had a value larger than ± 2.77 and hence there was no indication of outliers in the context of this model. This was confirmed using a leave-one-out analysis. Likewise, according to the Cook's distances, none of the studies could be considered to be overly influential. With eight out of the nine studies being assessed as high risk of bias, we were unable to conduct sensitivity analysis by study quality.

For peer review only - http://bmjopen.bmj.com/site/about/guidelines.xhtml

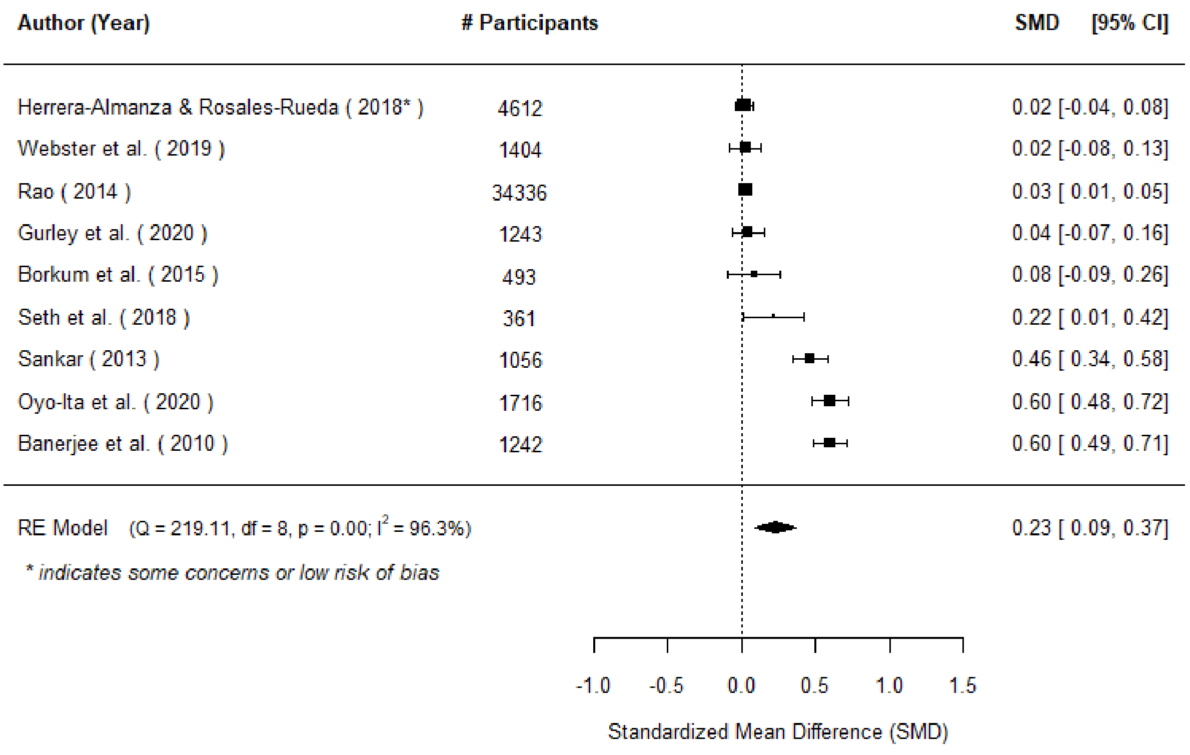

Supplementary Figure 17. Forest plot showing the observed outcomes and the estimate of the random-effects model for the impact of community engagement interventions on partial childhood immunisation

As a robustness check, we used robust variance analysis and included all dependent effects in the analysis, totaling 13 effects from the same 9 studies (df = 7.96). The overall average effect was slightly smaller but still significant ($\hat{\mu}$ = 0.21 [95% CI: 0.03 to 0.38], p = .03). Sensitivity analyses show the effect to be sensitive to all values of Rho.

BCG

We included a total of k = 12 studies in the analysis. The estimated average outcome based on the random-effects model was $\hat{\mu} = 0.06$ ([95% CI: 0.01 to 0.11], $z = 2.28$, $p = 0.02$), indicating a very small but significant benefit to the intervention participants compared to the control participants (see Supplementary Figure 18). According to the Q-test, the true outcomes appear to be heterogeneous ($Q(11) = 84.22$, $p < 0.01$, $\hat{\tau}^2 = 0.00$, $I^2 = 86.94\%$). A 95% credibility/prediction interval for the true outcomes is given by -0.08 to 0.19. Hence, although the average outcome is estimated to be positive, in some studies the true outcome may in fact be negative.

An examination of the studentized residuals revealed that one study (Banerjee 2010) had a value larger than ± 2.87 and may be a potential outlier in the context of this model. According to the Cook's distances, Banerjee (2010) could also be considered to be overly influential. Sensitivity analyses leaving each study out indicated that removing Banerjee (2010) would reduce the overall average effect ($\hat{\mu} = 0.02$ (95% CI: -0.005 to 0.04), and the resulting effect would be non-significant ($z = 1.51$, $p = 0.13$). According to the leave-one-out analysis, there are two additional studies whose removal would result in a non-significant effect; Costa-Font and Parmar (2017) and Rao (2014). Moderator analysis revealed that publication year was a significant predictor such that more recent studies find smaller effects that older studies, with each additional year reducing the size of the effect by .03 standard deviation units ($\beta = -0.03$ [95% CI: -0.05 to -0.001], $p = .04$). No other moderators were significant (see Supplementary Table 3). Only three studies were high or moderate quality. When those three studies were synthesised, the resulting effect was reduced ($\hat{\mu} = 0.01$ [95% CI: -0.08 to 0.10]) and no longer significant ($z = 0.22$, $p = 0.82$).

For peer review only - http://bmjopen.bmj.com/site/about/guidelines.xhtml

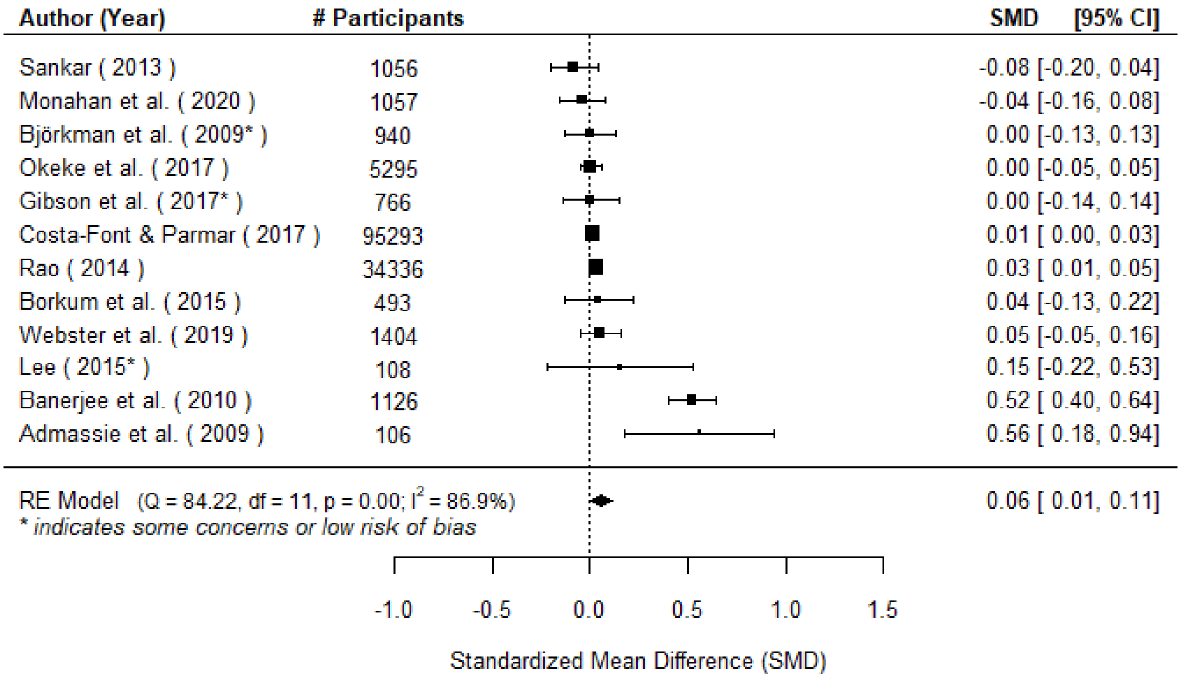

Supplementary Figure 18. Forest plot showing the observed outcomes and the estimate of the random-effects model for the impact of community engagement interventions on BCG vaccination.

A funnel plot of the estimates is shown in Supplementary Figure *18b*. The regression test indicated funnel plot asymmetry ($p = 0.04$) but not the rank correlation test ($p = 0.20$).

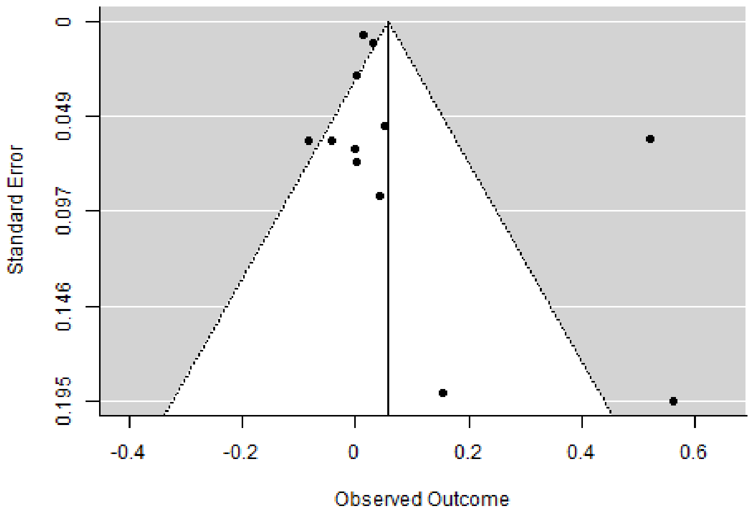

Supplementary Figure 18b: *Funnel plot*

As a robustness check, we used robust variance analysis and included all dependent effects in the analysis, totaling 16 effects from the same 12 studies (*df* = 7.4). The overall average effect was slightly smaller and non-significant ($\hat{\mu} = 0.04$ [95% CI: −0.02 to 0.10], *p* = .20). Sensitivity analyses show the effect to be sensitive to all values of Rho.

For peer review only - http://bmjopen.bmj.com/site/about/guidelines.xhtml

DPT1

We included a total of $k = 8$ studies in the analysis. The estimated average outcome based on the random-effects model was $\hat{\mu} = 0.04$ ([95% CI: -0.04 to 0.11], $z = 0.99$, $p = 0.32$), indicating no difference between the intervention group and the control group (see Supplementary Figure 19). A 95% credibility/prediction interval for the true outcomes is given by -0.14 to 0.21. Hence, although the average outcome is estimated to be positive, in some studies the true outcome may in fact be negative.

According to the Q-test, the true outcomes appear to be heterogeneous ($Q(7) = 30.18$, $p < 0.01$, $\hat{\tau}^2 = 0.01$, $I^2 = 76.81\%$). An examination of the studentized residuals revealed that none of the studies had a value larger than ± 2.73 and hence there was no indication of outliers in the context of this model. This was confirmed through a leave-one-out analysis. According to the Cook's distances, none of the studies could be considered to be overly influential. When we removed studies assessed as high risk of bias, two studies remained, and the average effect increased slightly ($\hat{\mu} = 0.04$ ([95% CI: -0.04 to 0.11]), but was still non-significant ($z = 0.99$, $p = 0.32$). Again, with only two studies contributing effects, this must be interpreted with caution.

We tested for potential sources of heterogeneity and found that study design was a significant predictor of the effect such that studies using quasi-experimental designs had smaller effects than RCTs by .15 standard deviation units ($\beta = -0.15$ [95% CI: -0.29 to -0.02], $p = .03$). We also found that there was a significant difference in the size of effects between programmes implemented by government agencies and those that were not, such that programmes implemented by government agencies (either alone or in tandem with another agency) had larger effects than programmes not implemented by a government agency by .17 standard deviation units ($\beta = 0.17$ [95% CI: 0.01 to 0.34], $p = .04$). No other moderators were significant (see Supplementary Table 3).

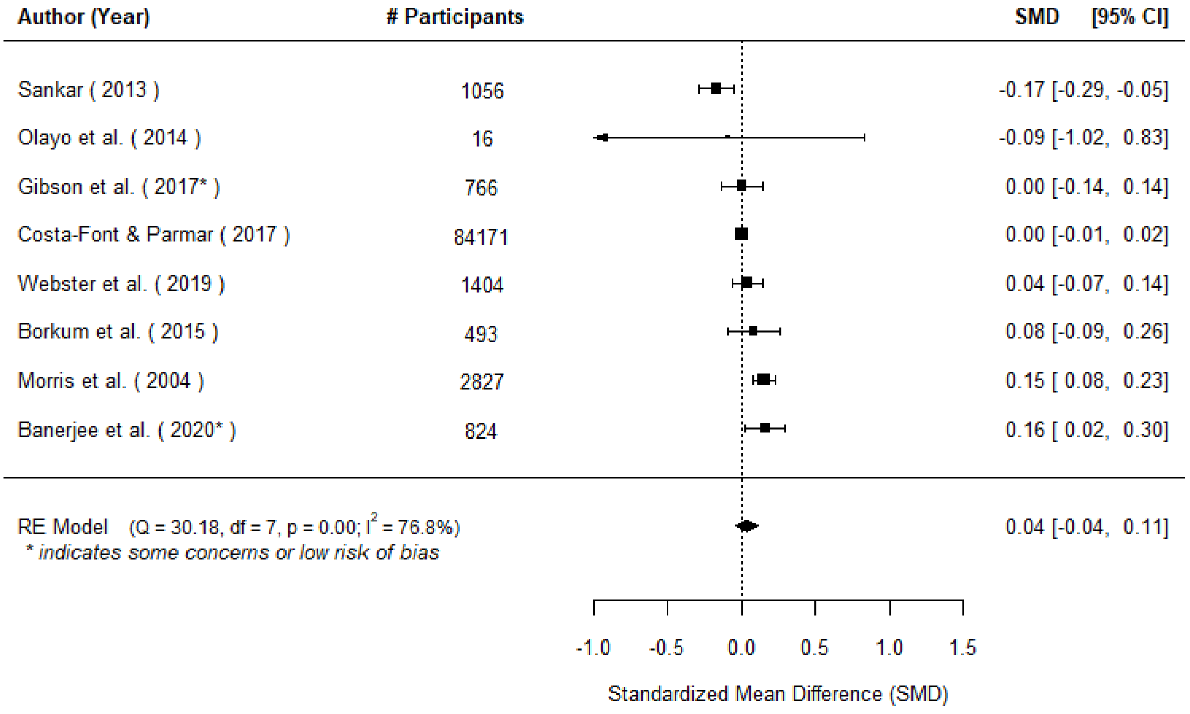

Supplementary Figure 19. Forest plot showing the observed outcomes and the estimate of the random-effects model for the impact of community engagement interventions on DPT1 vaccination.

For peer review only - http://bmjopen.bmj.com/site/about/guidelines.xhtml

As a robustness check, we used robust variance analysis and included all dependent effects in the analysis, totaling 21 effects from the same 8 studies (df = 5.24). The overall average effect was smaller and still non-significant ($\hat{\mu}$ = 0.01 [95% CI: −0.06 to 0.09], p = .66). Sensitivity analyses show the effect to be sensitive to all values of Rho.

DPT2

We included a total of $k = 5$ studies in the analysis. The estimated average outcome based on the random-effects model was $\hat{\mu} = 0.07$ (95% CI: 0.01 to 0.12). Therefore, the average outcome differed significantly from zero ($z = 2.23$, $p = 0.03$), indicating small but significant benefit to the treated group compared to the control group. A 95% credibility/prediction interval for the true outcomes is given by 0.01 to 0.12.

According to the Q-test, there was no significant amount of heterogeneity in the true outcomes ($Q(4) = 3.34$, $p = 0.50$, $\hat{\tau}^2 = 0.00$, $I^2 = 0.00\%$). An examination of the studentized residuals revealed that none of the studies had a value larger than ± 2.58 and hence there was no indication of outliers in the context of this model. According to the Cook's distances, none of the studies could be considered to be overly influential. A leave-one-out analyses indicated that the removal or either Banerjee and colleagues (2020) or Sankar (2013) would results in a non-significant average effect. With no heterogeneity present, we did not examine potential sources of variation. When high risk of bias studies were removed, the resulting average effect increased slightly ($\hat{\mu} = 0.11$ [95% CI: 0.01 to 0.20]) and was still significantly different from zero ($z = 2.12$, $p = 0.03$).

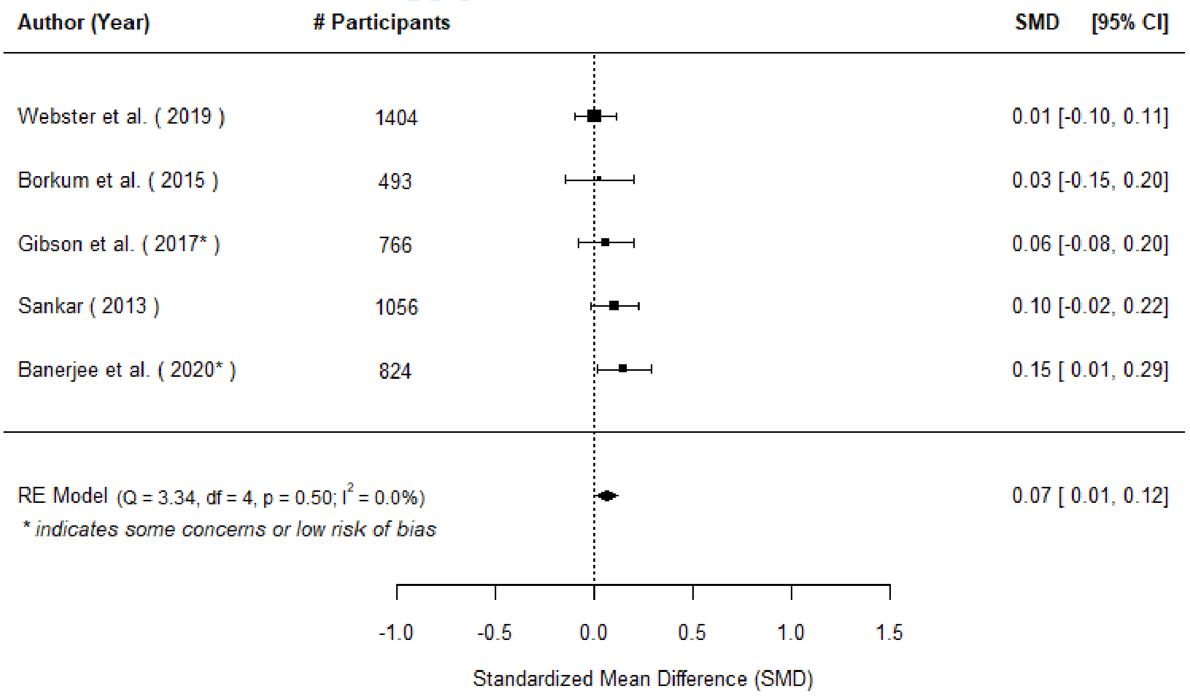

Supplementary Figure 20. Forest plot showing the observed outcomes and the estimate of the random-effects model for the impact of community engagement interventions on DPT2 vaccination.

This group of studies was not sufficiently powered for a robustness check using robust variance analysis (df = 3.51).

OPV0

We included a total of $k = 5$ studies in the analysis. The estimated average outcome based on the random-effects model was $\hat{\mu} = 0.10$ (95% CI: −0.06 to 0.26). Therefore, the average outcome did not differ significantly from zero ($z = 1.24$, $p = 0.22$), indicating no difference between the intervention and control groups (see Supplementary Figure 21). A 95% credibility/prediction interval for the true outcomes is given by −0.25 to 0.45. Hence, although the average outcome is estimated to be positive, in some studies the true outcome may in fact be negative.

For peer review only - http://bmjopen.bmj.com/site/about/guidelines.xhtml

According to the Q-test, the true outcomes appear to be heterogeneous ($Q(4) = 45.74$, $p < 0.01$, $\hat{\tau}^2 = 0.03$, $I^2 = 91.26\%$). An examination of the studentized residuals revealed that one study (Sankar 2013) had a value larger than ± 2.58 and may be a potential outlier in the context of this model. According to the Cook's distances, Sankar (2013) could also be considered to be overly influential. Indeed, sensitivity analyses leaving each study out indicated that removing Sankar (2013) would reduce the overall average effect ($\hat{\mu}$ = 0.01 [95% CI: -0.01 to 0.03]) which would become non-significant (z = 1.02, p = .31). Leave-one-out analyses confirmed that no other study unduly influenced the outcome.

Exposure to the intervention (in months) was a significant predictor of variation on OPV0 vaccinations such that each additional month of exposure increased the size of the effect by 0.02 standard deviation units (β = .02, [95% CI: 0.01 to 0.03]; p < .001). Publication year was also a significant predictor of variation on OPV0 vaccinations such that each additional year decreased the size of the effect by 0.07 standard deviation units (β = -0.07, [95% CI: −0.11 to −0.02]; p = 0.002), meaning more recent studies found smaller effects than older studies. With only one study that was not assessed as high risk of bias, we could not conduct a sensitivity analysis by study quality.

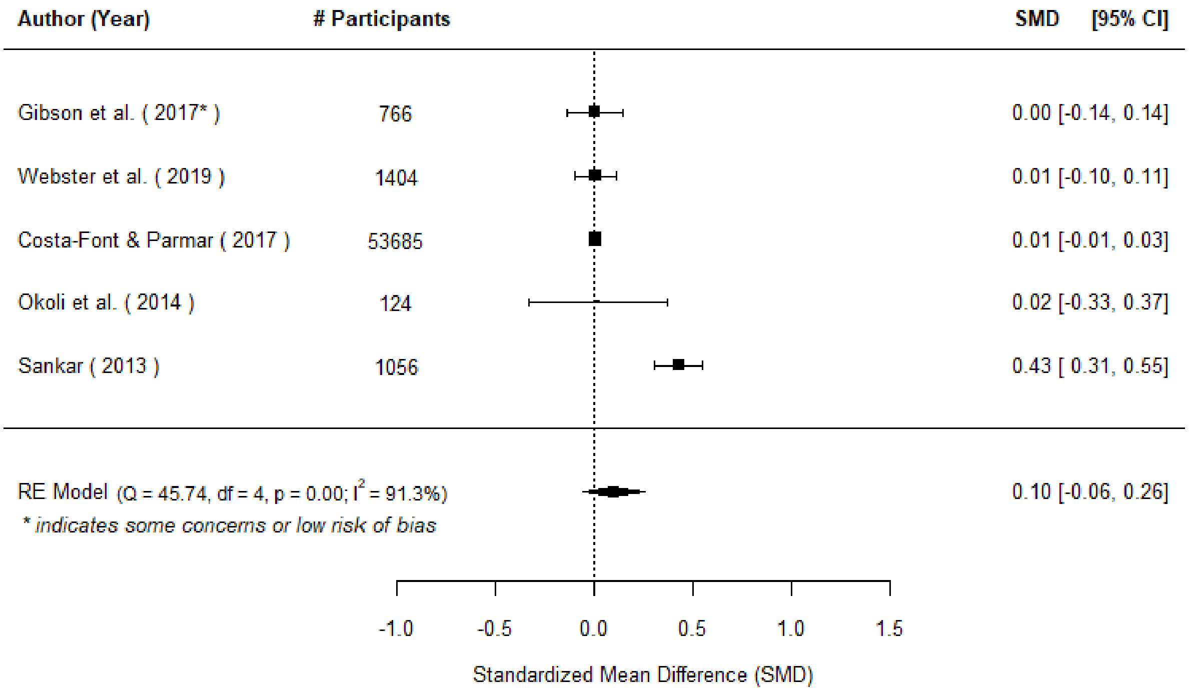

Supplementary Figure 21. Forest plot showing the observed outcomes and the estimate of the random-effects model for the impact of community engagement interventions on OPV0 vaccination.

This group of studies was not sufficiently powered for a robustness check using robust variance analysis (df = 3.76).

OPV1

We included a total of $k = 5$ studies in the analysis. The estimated average outcome based on the random-effects model was $\hat{\mu} = 0.08$ ([95% CI: 0.004 to 0.15], $z = 2.06$, $p = 0.04$), indicating a very small but significant benefit to the treated group compared to the control group (see Supplementary Figure 22). A 95% credibility/prediction interval for the true outcomes is given by -0.02 to 0.17. Hence, although the average outcome is estimated to be positive, in some studies the true outcome may in fact be negative.

For peer review only - http://bmjopen.bmj.com/site/about/guidelines.xhtml

According to the Q-test, there was no significant amount of heterogeneity in the true outcomes ($Q(4) = 4.77$, $p = 0.31$, $\hat{\tau}^2 = 0.00$, $I^2 = 16.17\%$), thus we did not test for sources of heterogeneity. An examination of the studentized residuals revealed that none of the studies had a value larger than ± 2.58 and hence there was no indication of outliers in the context of this model. According to the Cook's distances, none of the studies could be considered to be overly influential. Leave-one-out analyses indicated that the removal of any single study except for Webster et al. (2019) would result in a non-significant average effect.

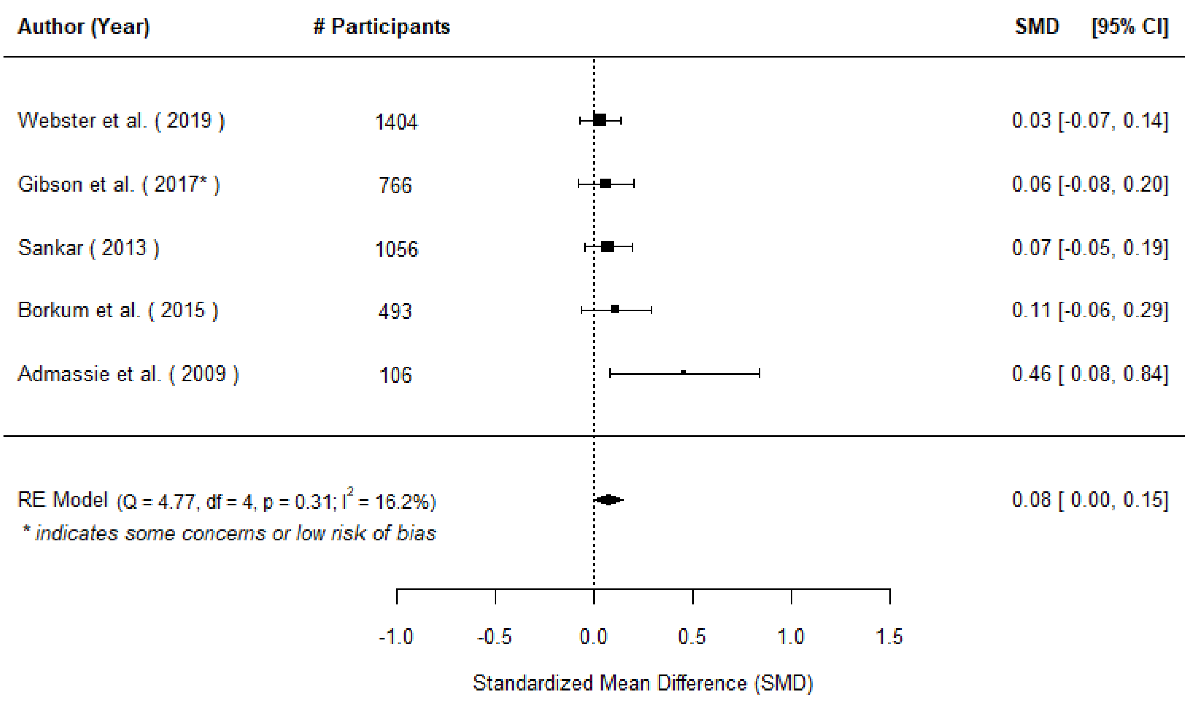

Supplementary Figure 22. Forest plot showing the observed outcomes and the estimate of the random-effects model for the impact of community engagement interventions on OPV1 vaccination.

This group of studies was not sufficiently powered for a robustness check using robust variance analysis (*df* = 3.02).

OPV2

We included a total of $k = 5$ studies in the analysis. The estimated average outcome based on the random-effects model was $\hat{\mu} = 0.24$ ([95% CI: 0.07 to 0.40], $z = 2.84$, $p < 0.01$), indicating a small but significant benefit to the treated group compared to the control group (see Supplementary Figure CEOPV2). A 95% credibility/prediction interval for the true outcomes is given by -0.12 to 0.59. Hence, although the average outcome is estimated to be positive, in some studies the true outcome may in fact be negative.

According to the Q-test, the true outcomes appear to be heterogeneous ($Q(4) = 22.32$, $p < 0.01$, $\hat{\tau}^2 = 0.03$, $I^2 = 82.08\%$). An examination of the studentized residuals revealed that none of the studies had a value larger than ± 2.58 and hence there was no indication of outliers in the context of this model. This was confirmed by a leave-one-out analysis. According to the Cook's distances, none of the studies could be considered to be overly influential. With only one study that was not assessed as high risk of bias, we could not conduct a sensitivity analysis by study quality.

We tested for potential sources of heterogeneity and found several significant moderators in the context of this model (see SupplementaryTable 3). Exposure to intervention was significant such that each additional month of exposure increased the average effect by .02 standard deviation units ($\beta = 0.02$ [95% CI: 0.01 to 0.02], p < .0001). In other words, longer interventions produced larger effects. Publication year was also significant such that older studies reported larger effects than more recent studies. Specifically, each additional year decreased the average effect by .05 standard deviation units ($\beta = -0.05$ [95% CI: -0.08 to -0.02], p < 0.001). Finally, region was a

For peer review only - http://bmjopen.bmj.com/site/about/guidelines.xhtml

significant predictor such that Sub-Saharan Africa had smaller effects that South Asia by .26 standard deviation units ($\beta = -0.26$ [95% CI: -0.39 to -0.13], $p < 0.001$).

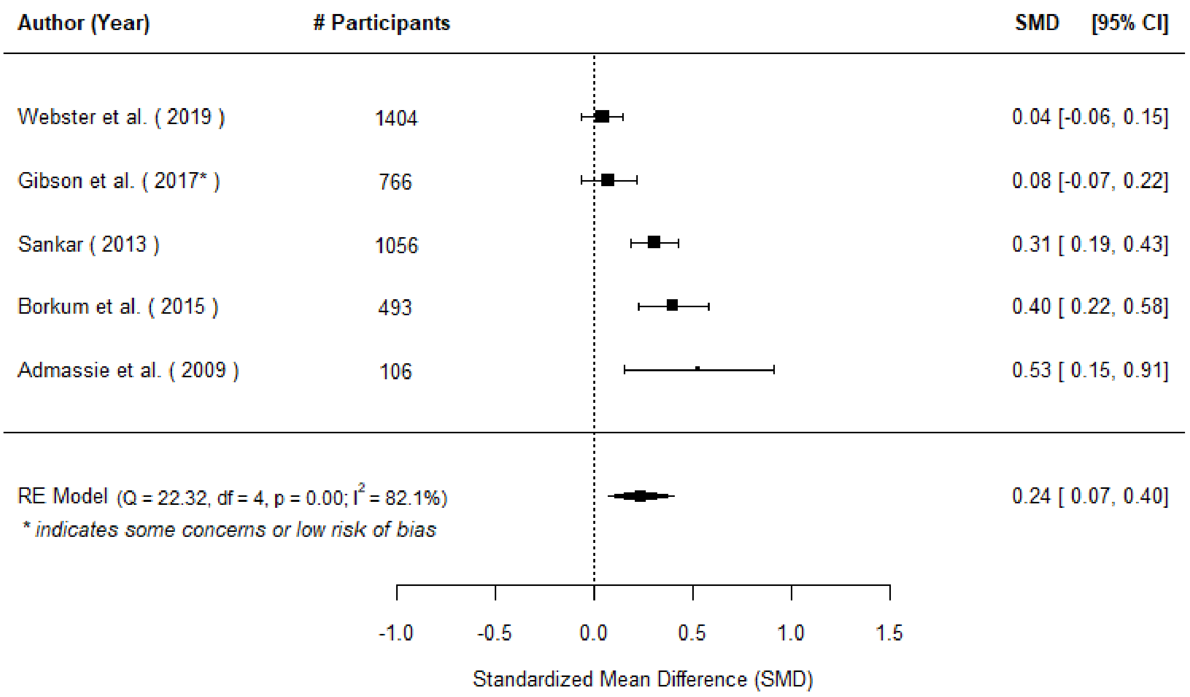

Supplementary Figure 23. Forest plot showing the observed outcomes and the estimate of the random-effects model for the impact of community engagement interventions on OPV2 vaccination.

This group of studies was not sufficiently powered for a robustness check using robust variance analysis (df = 3.75).

OPV3

We included total of $k = 9$ studies in the analysis. The estimated average outcome based on the random-effects model was $\hat{\mu} = 0.24$ ([95% CI: 0.09 to 0.40], $z = 3.06$, $p = 0.002$), indicating a moderate and significant benefit to the intervention group compared to the control group (see Supplementary Figure 24). A 95% credibility/prediction interval for the true outcomes is given by -0.22 to 0.70. Hence, although the average outcome is estimated to be positive, in some studies the true outcome may in fact be negative.

According to the Q-test, the true outcomes appear to be heterogeneous ($Q(8) = 222.99$, $p < 0.001$, $\hat{\tau}^2 = 0.05$, $I^2 = 96.41\%$). An examination of the studentized residuals revealed that one study (Sankar 2013) had a value larger than ± 2.77 and may be a potential outlier in the context of this model. According to the Cook's distances, one study (Sankar 2013) could be considered to be overly influential. Sensitivity analyses leaving each study out indicated that removing Sankar (2013) would substantially reduce the overall average effect ($\hat{\mu}$ = 0.08 [95% CI: 0.01 to 0.15]), but the effect would still be positive and significant (z = 2.39, p = .02). Leave-one-out analyses confirm that no other single study has undue influence on the estimated average effect. When low quality studies were removed, the resulting effect increased, but was no longer significant ($\hat{\mu} = 0.32$ [95% CI: -0.15 to 0.79], $z = 1.32$, $p = 0.19$). With only two studies of high or moderate quality, this result should be interpreted with caution. There were no significant sources of heterogeneity (see Supplementary Table 3).

For peer review only - http://bmjopen.bmj.com/site/about/guidelines.xhtml

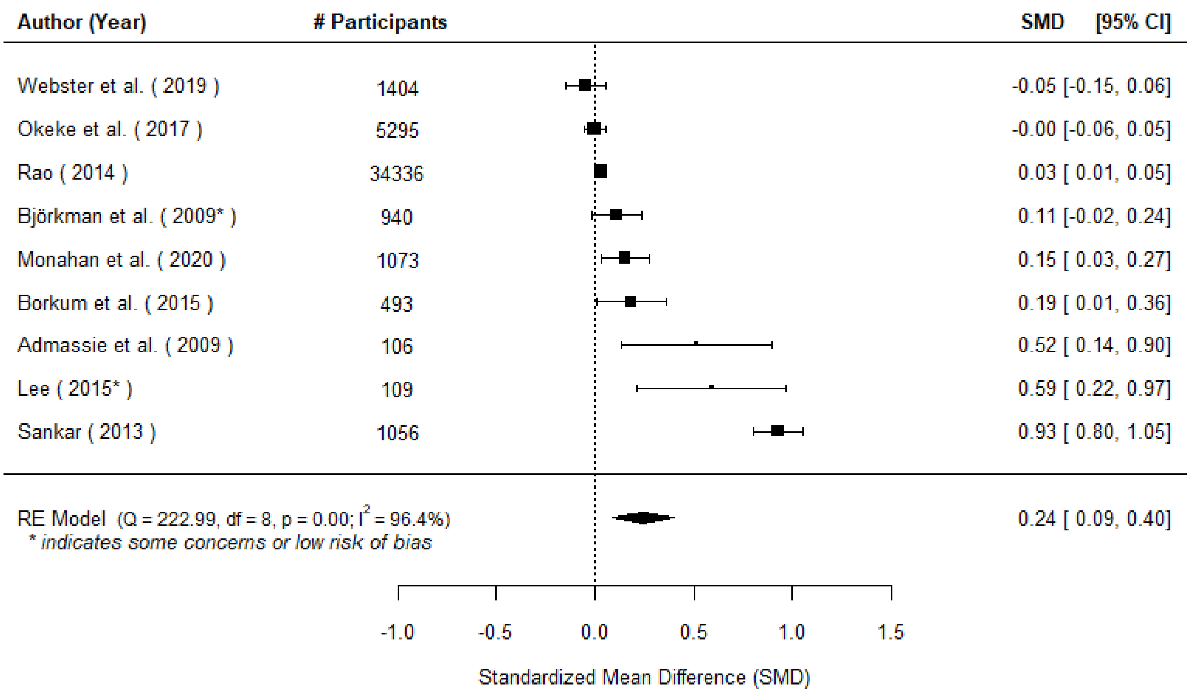

Supplementary Figure 24. Forest plot showing the observed outcomes and the estimate of the random-effects model for the impact of community engagement interventions on OPV3 vaccination.

As a robustness check, we used robust variance analysis and included all dependent effects in the analysis, totaling 10 effects from the same 9 studies (df = 6.74). The overall average effect was identical, but no longer significant ($\hat{\mu} = 0.24$ [95% CI: -0.02 to 0.51], p = .06). Sensitivity analyses show the effect to be sensitive to all values of Rho.

Dropouts

We included a total of $k = 5$ studies in the analysis. The estimated average outcome based on the random-effects model was $\hat{\mu} = 0.03$ ([95% CI: -0.11 to 0.16], $z = 0.36$, $p = 0.72$), indicating no significant difference between the intervention group and the control group (see Supplementary Figure 25). A 95% credibility/prediction interval for the true outcomes is given by -0.30 to 0.35. Hence, although the average outcome is estimated to be positive, in some studies the true outcome may in fact be negative.

According to the Q-test, the true outcomes appear to be heterogeneous ($Q(4) = 44.86$, $p < 0.001$, $\hat{\tau}^2 = 0.02$, $I^2 =$ 91.08%). An examination of the studentized residuals revealed that one study (Webster et al. 2019) had a value larger than ± 2.58 and may be a potential outlier in the context of this model. Indeed, sensitivity analyses indicated that removing Webster and colleagues (2019) would increase the average effect to $\hat{\mu} = 0.09$ (95% CI: -0.01 to 0.19), but the average outcome still did not differ significantly from zero ($z = 1.70 p = 0.09$). Leave-one-out analysis confirmed that no other single study had a disproportionate influence on the estimated average effect. According to the Cook's distances, none of the studies could be considered to be overly influential. With only one study assessed as having a low risk of bias (Banerjee et al. 2020) and the remaining studies at high risk of bias, we were unable to conduct sensitivity analysis by study quality. Only publication year was a significant predictor such that more recently published studies had higher average effects than older studies. Specifically, each additional year increased the average effect by .33 standard deviation units ($\beta = 0.33$ [95% CI: 0.10 to 0.57], $p = 0.01$).

For peer review only - http://bmjopen.bmj.com/site/about/guidelines.xhtml

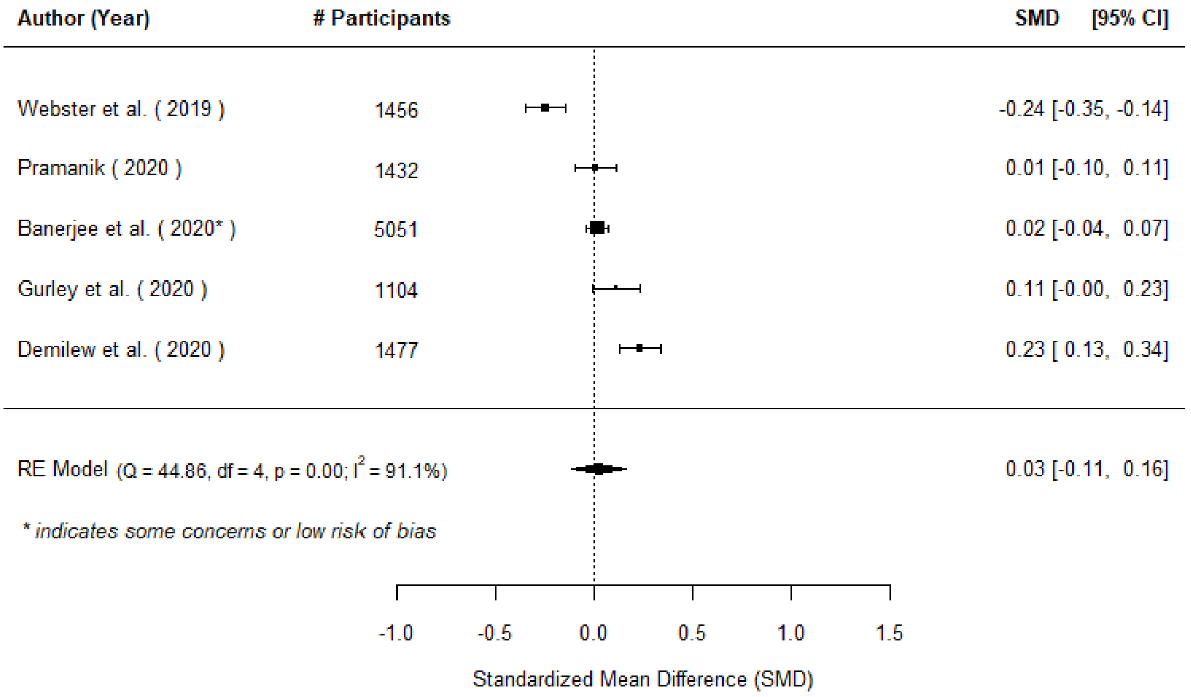

Supplementary Figure 25. Forest plot showing the observed outcomes and the estimate of the random-effects model for the impact of community engagement interventions on childhood morbidity.

This group of studies was not sufficiently powered for a robustness check using robust variance analysis (*df* = 3.98).

Morbidity

We used reports of diarrhea (most typically in the past two weeks) as a proxy for childhood morbidity. In all cases, effects were reverse coded such that positive effects always indicate a benefit to the treated group. Thus, a positive effect here would be interpreted as a reduction in diarrhea among treated participants compared to control participants. A total of $k = 10$ studies were included in the analysis. The estimated average outcome was $\hat{\mu} = 0.01$ ([95% CI: -0.06 to 0.08], $z = 0.34$, $p = 0.73$), indicating no significant difference between intervention group and control group (Supplementary Figure 26). A 95% credibility/prediction interval for the true outcomes is given by -0.19 to 0.21. Hence, although the average outcome is estimated to be positive, in some studies the true outcome may in fact be negative.

According to the Q-test, the true outcomes appear to be heterogeneous ($Q(9) = 52.37$, $p < 0.01$, $\hat{\tau}^2 = 0.01$, $I^2 = 82.82\%$). When high risk of bias studies were removed, only two studies remained. The resulting estimated average outcome was identical ($\hat{\mu} = 0.01$ [95% CI: -0.21 to 0.23]), and was still non-significant ($z = 0.09$, $p = 0.93$).

An examination of the studentized residuals revealed that one study (Assegaai et al. 2018) had a value larger than ± 2.81 and may be a potential outlier in the context of this model. According to the Cook's distances, Assegaai and colleagues (2018) could also be considered to be overly influential. Indeed, sensitivity analyses leaving each study out indicated that removing Assegaai and colleagues (2018) would reduce the overall average effect ($\hat{\mu}$ = -0.02 (95% CI: -0.08 to 0.05), making the effect negative but still non-significant (z = -0.47, *p* = .64). Leave-one-out analyses confirmed that no other single studies had a disproportionate effect on the estimated average effect size. A funnel plot of the estimates is shown in Supplementary Figure 26b. Neither the rank correlation nor the regression test indicated any funnel plot asymmetry ($p = 0.60$ and $p = 0.49$, respectively). We tested for sources of heterogeneity, but there were no significant moderators (see Supplementary Table 3)

For peer review only - http://bmjopen.bmj.com/site/about/guidelines.xhtml

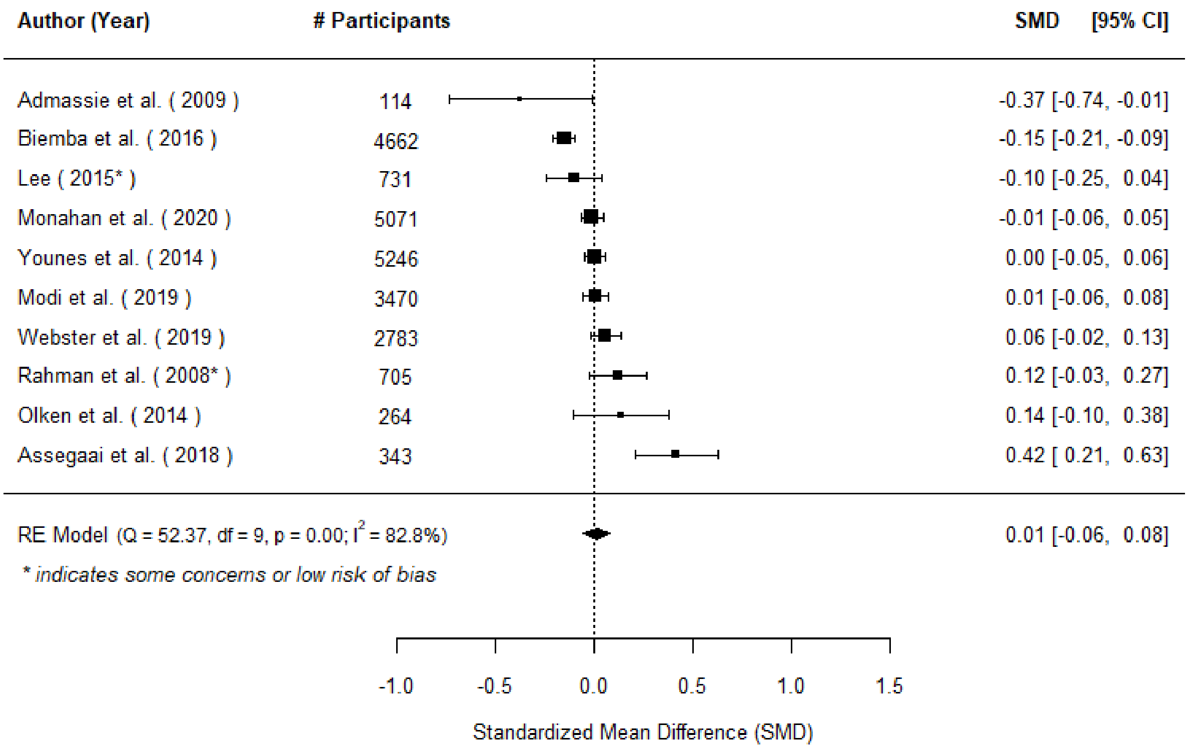

Supplementary Figure 26. Forest plot showing the observed outcomes and the estimate of the random-effects model for the impact of community engagement interventions on childhood morbidity.

review only

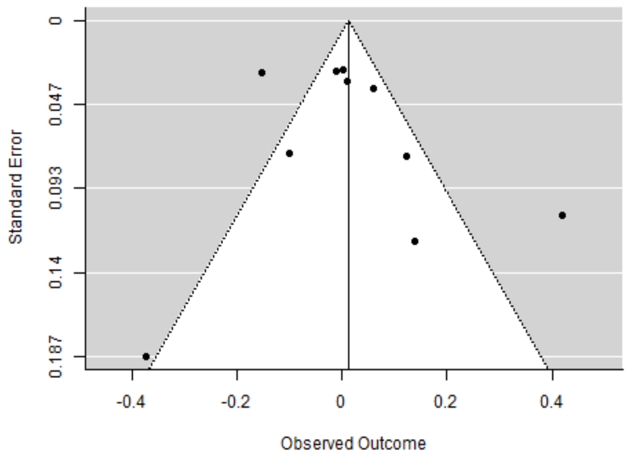

Supplementary Figure 26b. Funnel plot for studies examining the effect of community engagement interventions on childhood morbidity

As a robustness check, we used robust variance analysis and included all dependent effects in the analysis, totaling 26 effects from the same 10 studies (df = 7.69). The overall average effect was identical and still non-significant ($\hat{\mu}$ = 0.01 [95% CI: −0.09 to 0.10], p = .86). Sensitivity analyses show the effect to be sensitive to all values of Rho.

For peer review only - http://bmjopen.bmj.com/site/about/guidelines.xhtml

Mortality

We included total of $k = 6$ studies in the analysis. The estimated average outcome based on the random-effects model was $\hat{\mu} = -0.04$ (95% CI: -0.09 to 0.01; *see Supplementary Figure C27*). Therefore, the average outcome did not differ significantly from zero ($z = -1.66$, $p = 0.10$), indicating no difference in mortality between the treatment and control groups. A 95% credibility/prediction interval for the true outcomes is given by -0.14 to 0.06. Hence, although the average outcome is estimated to be negative, in some studies the true outcome may in fact be positive.

According to the Q-test, the true outcomes appear to be heterogeneous ($Q(5) = 19.28$, $p < 0.01$, $\hat{\tau}^2 = 0.00$, $I^2 = 74.07\%$). An examination of the studentized residuals revealed that none of the studies had a value larger than ± 2.64 and hence there was no indication of outliers in the context of this model. According to the Cook's distances, none of the studies could be considered to be overly influential. Leave-one-out analyses indicated that the removal of Monahan and colleagues (2020) would result in a statistically significant negative effect. When high risk of bias studies were removed, only two studies remained. The resulting estimated average outcome was identical ($\hat{\mu} = -0.04$ [95% CI: -0.12 to 0.04]), and was still non-significant ($z = -1.07$, $p = 0.29$). We tested for sources of heterogeneity, and only publication year was significant such that each additional year increased the effect by .01 standard deviation units ($\hat{B} = 0.01, p < 0.001$ [95% CI: 0.01 to 0.02]). There were no other significant moderators (see Supplementary Table 3).

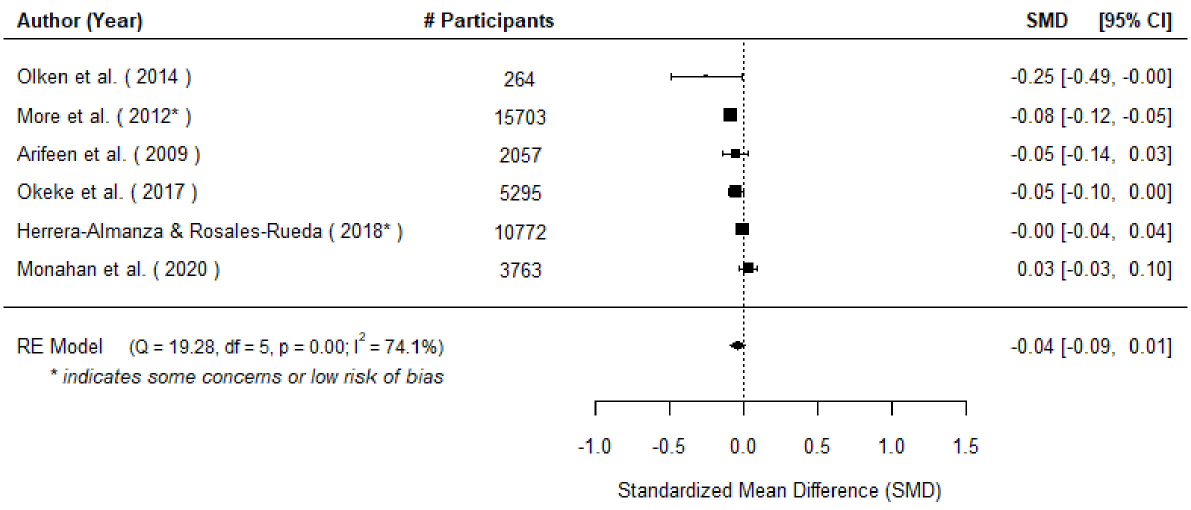

Supplementary Figure 27. Forest plot showing the observed outcomes and the estimate of the random-effects model for the impact of community engagement interventions on childhood morbidity.

As a robustness check, we used robust variance analysis and included all dependent effects in the analysis, totaling 25 effects from the same 6 studies ($df = 4.07$). The overall average effect was identical and still non-significant ($\hat{\mu} = -0.04$ [95% CI: -0.09 to 0.01], $p = .12$). Sensitivity analyses show the effect to be sensitive to all values of Rho.

Immunisation knowledge

We included a total of $k = 9$ studies in the analysis. The estimated average outcome based on the random-effects model was $\hat{\mu} = 0.19$ (95% CI: 0.07 to 0.31). Therefore, the average outcome differed significantly from zero ($z = 3.02$, $p < 0.01$), indicating a significant benefit to the treatment group compared to the control group (see Supplementary Figure 28). A 95% credibility/prediction interval for the true outcomes is given by -0.16 to 0.53. Hence, although the average outcome is estimated to be positive, in some studies the true outcome may in fact be negative.

For peer review only - http://bmjopen.bmj.com/site/about/guidelines.xhtml

According to the Q-test, the true outcomes appear to be heterogeneous ($Q(8) = 62.87$, $p < 0.01$, $\hat{\tau}^2 = 0.03$, $I^2 =$ 87.28%). An examination of the studentized residuals revealed that one study (Banwat et al. 2015) had a value larger than ± 2.77 and may be a potential outlier in the context of this model. Indeed, sensitivity analyses leaving each study out indicated that removing Banwat and colleagues (2015) would reduce the overall average effect ($\hat{\mu}$ = 0.11 (95% CI: -0.02 to 0.20), but the effect would still be positive and significant (z = 2.33, p = .02). Leave-one-out analyses confirmed no other single study was excessively influential on the average estimated effect. According to the Cook's distances, none of the studies could be considered to be overly influential.

We examined potential sources of heterogeneity, and study design was a significant predictor of immunisation knowledge such that studies using quasi-experimental designs had larger average effects than RCT designs by .44 standard deviation units ($\hat{\beta} = 0.44$ [95% CI: 0.23 to 0.64], p < 0.001). Region was also a significant predictor, but region was perfectly confounded with study design (e.g. all studies from South Asia were RCTs, while all studies from Sub-Saharan Africa were all quasi-experimental designs), so the effects were larger in Sub-Saharan Africa than from South Asia by the same .44 standard deviation units ($\beta = 0.44$ [95% CI: 0.23 to 0.64], p < 0.001). No other moderators were significant (see Supplementary Table 3)

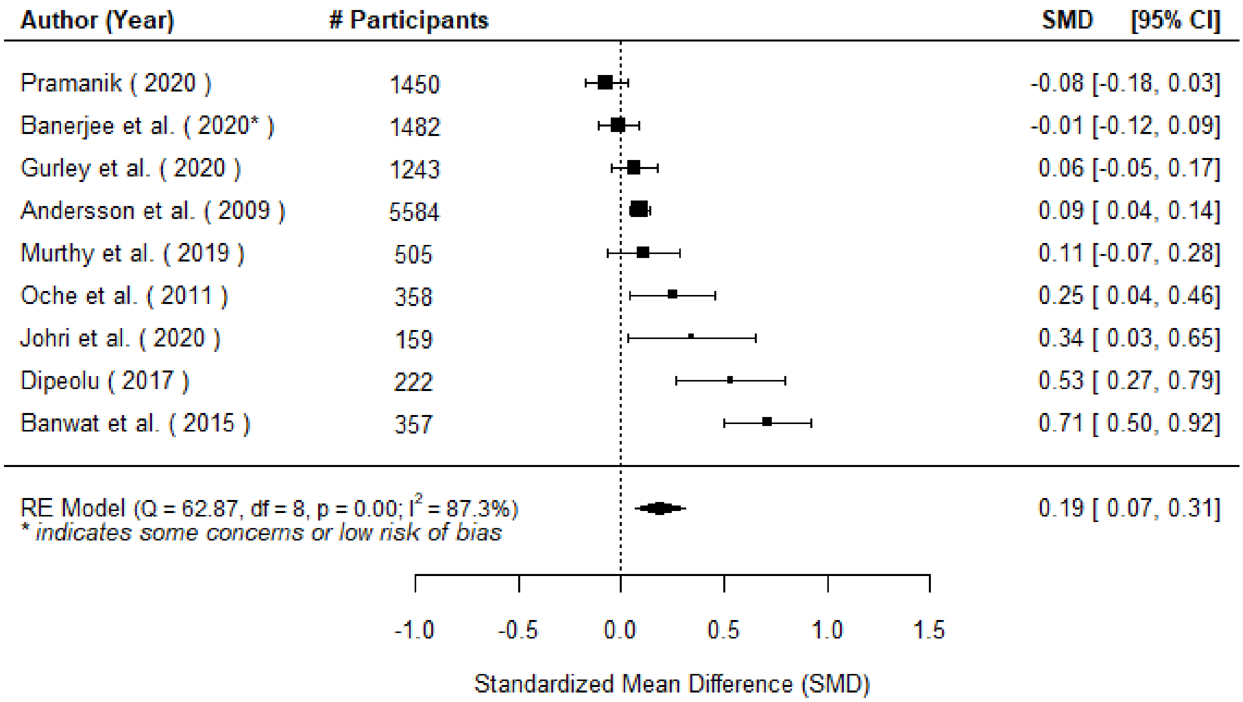

Supplementary Figure 28. Forest plot showing the observed outcomes and the estimate of the random-effects model for the impact of community engagement interventions on childhood morbidity.

As a robustness check, we used robust variance analysis and included all dependent effects in the analysis, totaling 13 effects from the same 9 studies (df = 7.69). The overall average effect slightly smaller and become non-significant ($\hat{\mu} = 0.17$ [95% CI: -0.02 to 0.37], p = .07). Sensitivity analyses show the effect to be sensitive to all values of Rho.

Immunisation attitudes

We included a total of $k = 6$ studies in the analysis. The estimated average outcome was $\hat{\mu} = 0.14$ ([95% CI: -0.03 to 0.31], $z = 1.60$, $p = 0.11$), indicating no significant different between the intervention and control groups (see Supplementary Figure 29). A 95% credibility/prediction interval for the true outcomes is given by -0.27 to 0.54.

For peer review only - http://bmjopen.bmj.com/site/about/guidelines.xhtml

Hence, although the average outcome is estimated to be positive, in some studies the true outcome may in fact be negative.

According to the Q-test, the true outcomes appear to be heterogeneous ($Q(5) = 50.43$, $p < 0.01$, $\hat{\tau}^2 = 0.04$, $I^2 = 90.09\%$). An examination of the studentized residuals revealed that one study (Banwat et al. 2015) had a value larger than ± 2.64 and may be a potential outlier in the context of this model. Indeed, sensitivity analyses leaving each study out indicated that removing Banwat and colleagues (2015) would reduce the overall average effect ($\hat{\mu}$ = 0.04 [95% CI: -0.05 to 0.14]), but the effect would still be positive and non-significant (z = 0.92, p = .36). Leave-one-out analyses also indicated that the removal of Dipeolu (2017) would result in a significant positive effect ($\hat{\mu}$ = 0.21 [95% CI: 0.04 to 0.38])According to the Cook's distances, none of the studies could be considered to be overly influential. With only one study not assessed as high risk of bias, we were unable to conduct a sensitivity analysis by study quality. We tested for sources of heterogeneity, but of the moderators we were able to test, none were significant (see Supplementary Table 3)

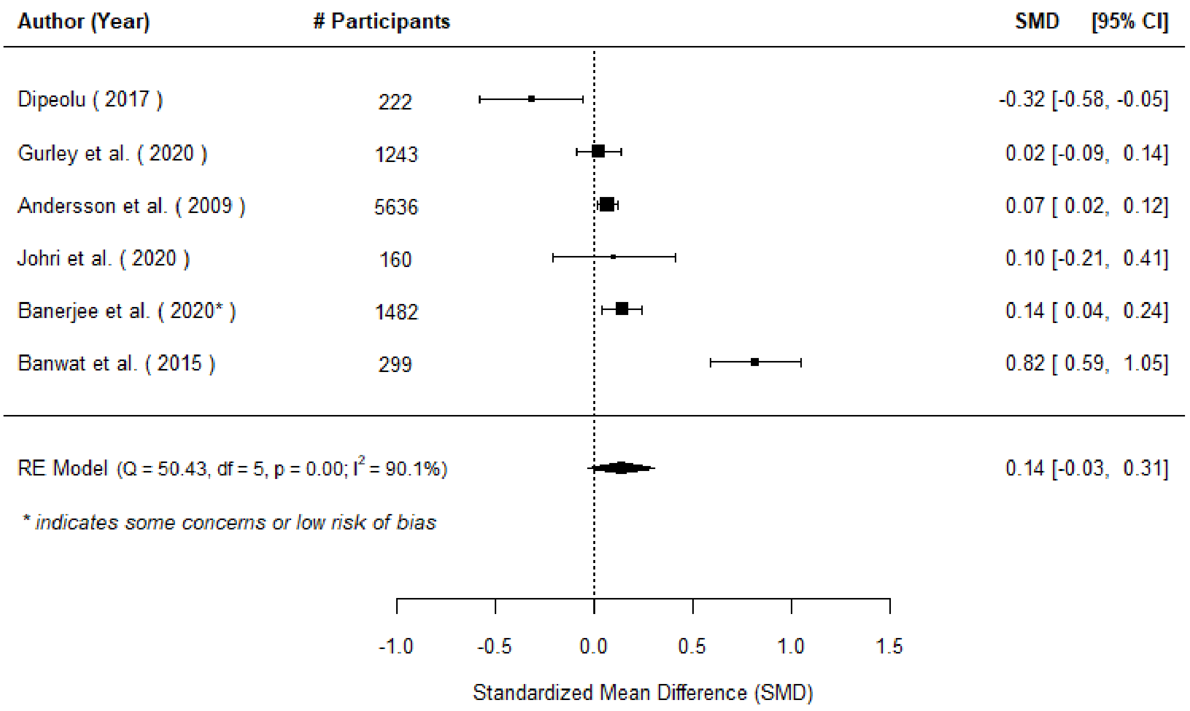

Supplementary Figure 29. Funnel plot for studies examining the effect of community engagement interventions on immunisation attitudes

There were no dependent effects, so we did not complete a robustness check using RVE for this analysis.

Vaccination card availability

We included total of $k = 4$ studies in the analysis. The estimated average outcome was $\hat{\mu} = -0.01$ (95% CI: -0.05 to 0.02). The average outcome did not differ significantly from zero ($z = -0.84$, $p = 0.40$), indicating no difference between the intervention group and the control group on vaccination card availability (see Supplementary Figure 30). A 95% credibility/prediction interval for the true outcomes is given by -0.05 to 0.02.

According to the Q-test, there was no significant amount of heterogeneity in the true outcomes ($Q(3) = 2.27$, $p = 0.52$, $\hat{\tau}^2 = 0.00$, $I^2 = 0.00\%$). One study (Herrera-Almanza & Rosales-Rueda 2018) had a relatively large weight compared to the rest of the studies (i.e., weight $\geq 3/k$, so a weight at least 3 times as large as having equal weights across studies). However, this was also the only study assessed as having a low risk of bias. The three other studies in the analysis were assessed as high risk of bias. An examination of the studentized residuals revealed that none of

For peer review only - http://bmjopen.bmj.com/site/about/guidelines.xhtml

the studies had a value larger than ± 2.50 and hence there was no indication of outliers in the context of this model. This was confirmed by a leave-one-out analysis. According to the Cook's distances, none of the studies could be considered to be overly influential. With no heterogeneity present, we did not test for moderation.

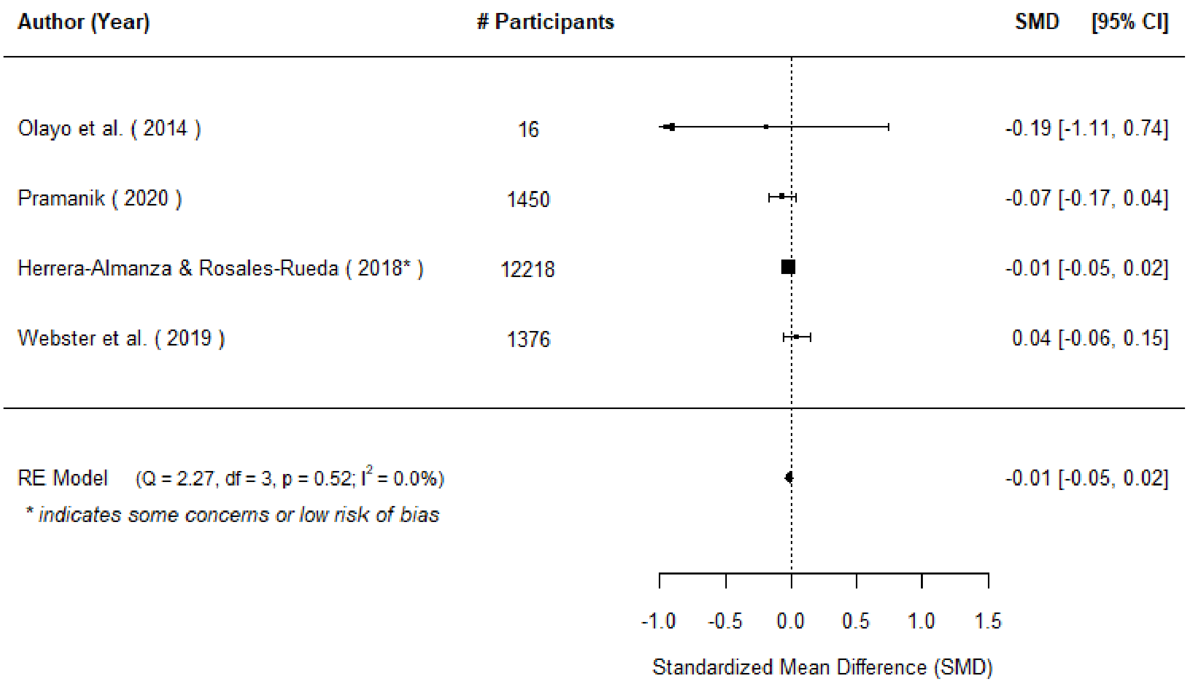

Supplementary Figure 30. Forest plot showing the observed outcomes and the estimate of the random-effects model for the impact of community engagement interventions on vaccination card availability.

There were no dependent effects, so we did not complete a robustness check using RVE for this analysis.

Experience and satisfaction with health services

Only two studies using community engagement interventions reported on experience and satisfaction with health services. The estimated average outcome based on the random-effects model was $\hat{\mu} = 0.04$ (95% CI: -0.15 to 0.23). Therefore, the average outcome did not differ significantly from zero ($z = 0.38$, $p = 0.70$, see Supplementary Figure 31). Given the small number of studies, this result should be interpreted with caution. A 95% credibility/prediction interval for the true outcomes is given by -0.13 to 0.31. According to the Q-test, the true outcomes appear to be homogeneous ($Q(1) = 1.93$, $p = 0.16$, $\hat{\tau}^2 = 0.01$, $I^2 = 48.22\%$; With only two studies and no heterogeneity, moderator analyses were not appropriate and tests of publication bias are not valid, and we were not powered for a robustness check using RVE. We also did not complete a leave-one-out analysis in this case.

For peer review only - http://bmjopen.bmj.com/site/about/guidelines.xhtml

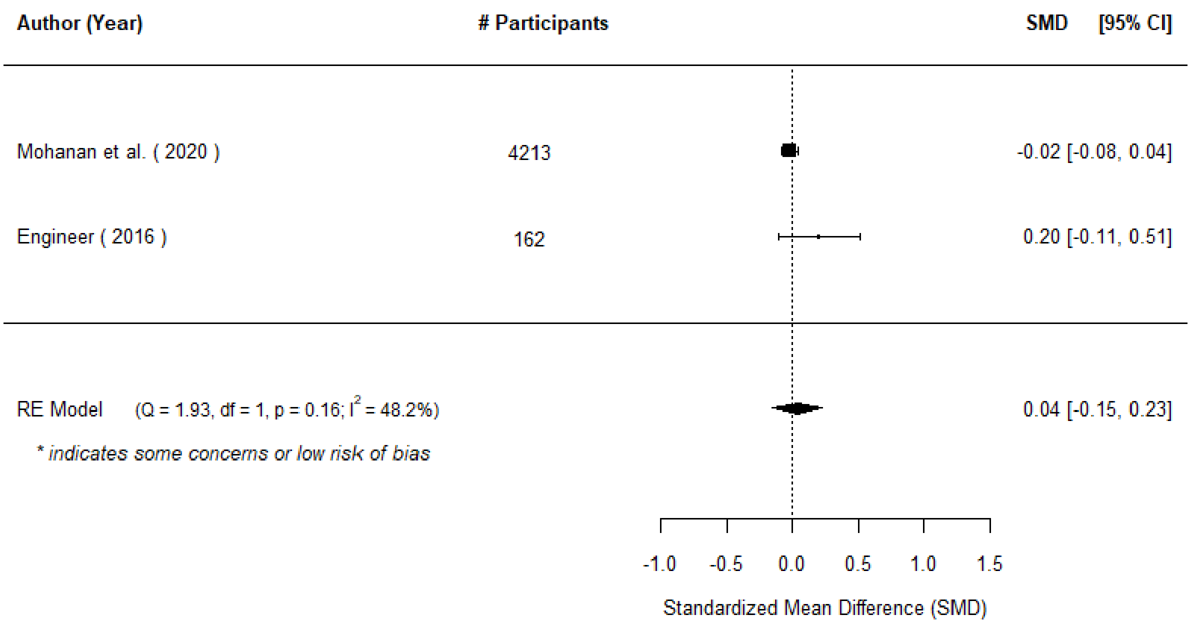

Supplementary Figure 31. Forest plot showing the observed outcomes and the estimate of the random-effects model for community engagement interventions on experience and satisfaction with health services.

Formal health worker's motivation, capacity and performance

Only two studies using community engagement interventions reported on formal health worker's motivation, capacity and performance. The estimated average outcome based on the random-effects model was $\hat{\mu} = 0.11$ (95% CI: -0.07 to 0.29). Therefore, the average outcome did not differ significantly from zero ($z = 0.38$, $p = 0.70$, see Supplementary Figure 32). Given the small number of studies, this result should be interpreted with caution. A 95% credibility/prediction interval for the true outcomes is given by -0.07 to 0.29. According to the Q-test, the true outcomes appear to be homogeneous ($Q(1) = 0.04$, $p = 0.84$, $\hat{\tau}^2 = 0.00$, $I^2 = 0.00\%$; With only two studies and no heterogeneity, moderator analyses were not appropriate and tests of publication bias are not valid, and we were not powered for a robustness check using RVE. We also did not complete a leave-one-out analysis in this case.

For peer review only - http://bmjopen.bmj.com/site/about/guidelines.xhtml

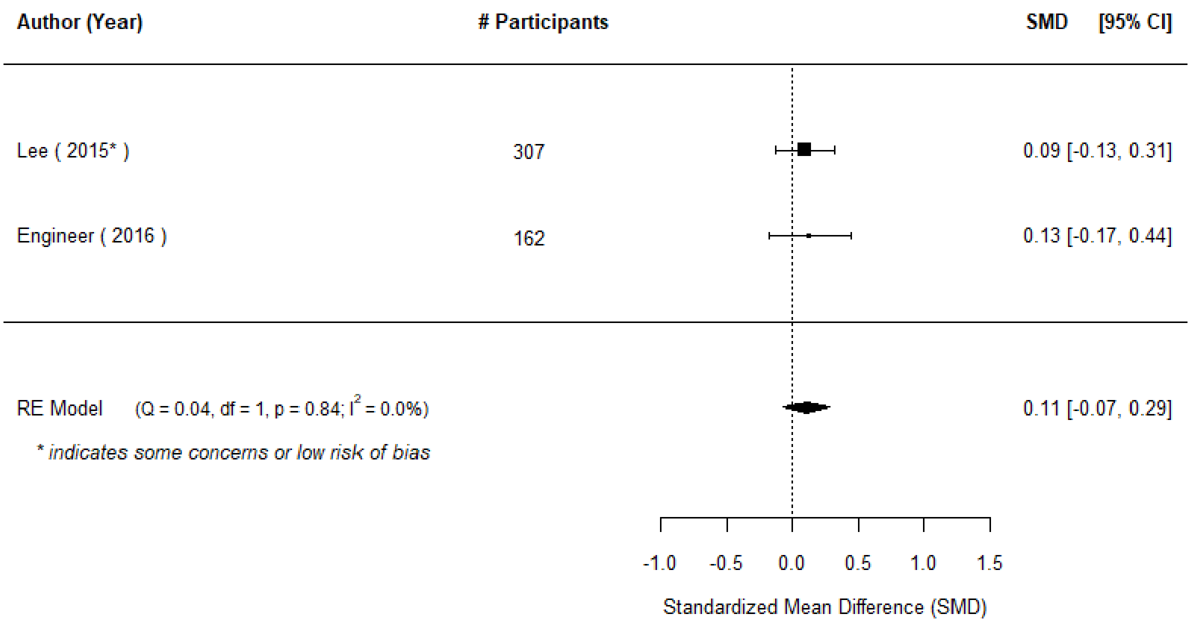

Supplementary Figure 32. Forest plot showing the observed outcomes and the estimate of the random-effects model for community engagement interventions on formal health worker's motivation, capacity and performance.

For peer review only - http://bmjopen.bmj.com/site/about/guidelines.xhtml

Supplementary Table 2: Summary of quantitative result

	TOTAL SAMPLE		ENGAGEMENT AS THE INTERVENTION	ENGAGEMENT IN THE DESIGN	ENGAGEMENT IN IMPLEMENTATION AUTONOMY	MULTIPLE ENGAGEMENT TYPES
	RVE (dependent effects)	RE (independent effects)	g, [95% CI], I^2, (k)	g, [95% CI], I^2, (k)	g, [95% CI], I^2, (k)	g, [95% CI], I^2, (k)
	g, [95% CI], I^2, (k; # of effects)	g, [95% CI], I^2, (k)				
**FULL IMMUNISATION**	**0.11**, [0.04, 0.18],** 87.27, (28; 53)	**0.14**, [0.06, 0.23],** 94.46, (28)	**0.08**, [0.03, 0.13],** 70.00, (12)	**010*, [0.02, 0.19],** 23.83, (5)	0.23, [-0.001, 0.47], 73.07, (2)	0.22, [-0.12, 0.56], 97.94, (9)
**PARTIAL IMMUNISATION**	**0.21*, [0.03, 0.38],** 95.90, (9; 13)	**0.23**, [0.09, 0.37],** 96.35, (9)	0.31, [-0.25, 0.87], 98.96, (2)	**0.14*, [0.01, 0.27],** 0.00, (2)	N/A	0.28, [-0.01, 0.56], 97.05, (4)
**MEASLES**	**0.06*, [0.01, 0.11],** 72.46, (20; 34)	**0.07**, [0.03, 0.11],** 73.64, (20)	**0.10***, [0.05, 0.15],** 60.29, (10)	**0.11*, [0.02, 0.21],** 0.00, (2)	0.03, [-0.09, 0.15], 54.84, (2)	0.03, [-0.10, 0.16], 86.76, (6)
**BCG**	0.04, [-0.02, 0.10], 79.17, (12; 16)	**0.06*, [0.01, 0.11],** 86.94, (12)	**0.02***, [0.01, 0.03],** 00.00, (4)	0.02, [-0.09, 0.13], 0.00, (2)	0.03, [-0.05, 0.11], 0.00, (2)	0.22, [-0.07, 0.52], 96.02, (4)
**DPT1**	0.01, [-0.06, 0.09], 62.55, (8; 21)	0.04, [-0.04, 0.11], 76.81, (8)	0.10, [-0.03, 0.22] 90.21, (3)	0.03, [-0.08.0.14], 0.00, (2)	N/A	**-0.17**, [-0.29, -0.05],** 0.00, (2)
**DPT2**	Not powered	**0.07*, [0.01, 0.12],** 0.00, (5)	N/A	0.05, [-0.06, 0.16], 0.00, (2)	N/A	N/A
**DPT3**	**0.10**, [0.05, 0.15],** 76.42, (22; 36)	**0.10***, [0.06, 0.14],** 76.78, (22)	**0.09**, [0.03, 0.15],** 73.17, (6)	0.04, [-0.01, 0.08], 0.00, (6)	0.11, [-0.05, 0.28], 80.12, (3)	**0.20**, [0.06, 0.34],** 87.93, (7)
**OPV0**	Not powered	0.10, [-0.06, 0.26], 91.26, (5)	N/A	0.01, [-0.13, 0.14], 0.00, (2)	N/A	N/A
**OPV1**	Not powered	**0.08*, [0.004, 0.15],** 16.17, (5)	N/A	0.08, [-0.03, 0.19], 0.00, (2)	N/A	0.22, [-0.15, 0.59], 72.32, (2)
**OPV2**	Not powered	**0.24**, [0.07, 0.40],** 82.08, (5)	N/A	0.23, [-0.09, 0.55], 87.44, (2)	N/A	**0.34***, [0.19, 0.50],** 15.51, (2)

For peer review only - http://bmjopen.bmj.com/site/about/guidelines.xhtml

**OPV3**	0.24, [-0.02, 0.51], 96.42, (9; 10)	**0.24**, [0.09, 0.40],** 96.41, (9)	0.16, [-0.02, 0.34], 83.83, (3)	N/A	0.03, [-0.12, 0.18], 70.38, (2)	0.48, [-0.24, 1.20], 98.92, (3)
**TIMELINESS (ALL)**	**0.11**, [0.06, 0.16],** 39.92, (11; 54)	----	----	----	----	----
**TIMELINESS (DTP3)**	No dependent effects	**0.09**, [0.03, 0.14],** 0.00, (7)	N/A	**0.12**, [0.03, 0.21],** 0.00, (4)	0.04, [-0.06, 0.13], 0.00, (2)	N/A
**TIMELINESS (MEASLES)**	No dependent effects	**0.23***, [0.14, 0.32],** 0.00, (2)	N/A	N/A	N/A	N/A
**TIMELINESS (COMPLETE IMMUNISATIONS)**	No dependent effects	**0.15***, [0.07, 0.24],** 9.66 (5)	N/A	**0.15*, [0.004, 0.29],** 0.00, (2)	0.38, [-0.28, 1.03], 72.02, (2)	N/A
**DROPOUTS**	Not powered	0.03, [-0.11, 0.16], 91.08, (5)	0.02, [-0.03, 0.06], 0.00, (2)	N/A	N/A	**0.18**, [0.06, 0.30],** 57.38, (2)
**MORBIDITY**	0.01, [-0.09, 0.10], 83.32, (10; 26)	0.01, [-0.06, 0.08], 82.82, (10)	-0.004, [-0.11, 0.10], 88.86, (5)	0.05, [-0.06, 0.15], 45.71, (2)	N/A	-0.10, [-0.60, 0.40], 80.88, (2)
**MORTALITY**	-0.04, [-0.09, 0.01], 69.91, (6; 25)	-0.04, [-0.09, 0.01], 74.07, (6)	-0.04, [-0.11, 0.04], 80.68, (3)	N/A	N/A	-0.04, [-0.10, 0.03], 62.75, (3)
**IMMUNISATION KNOWLEDGE**	0.17, [-0.02, 0.37], 87.30, (9; 13)	**0.19*, [0.07, 0.31],** 87.28, (9)	0.20, [-0.08, 0.49], 93.80, (4)	**0.31*, [0.04, 0.58],** 72.34, (3)	N/A	**0.09***, [0.04, 0.13],** 0.00, (2)
**IMMUNISATION ATTITUDES**	No dependent effects	0.14, [-0.03, 0.31], 90.09, (6)	0.47, [-0.19, 1.13], 96.36, (2)	-0.11, [-0.53, 0.30], 75.61, (2)	N/A	**0.06*, [0.01, 0.11],** 0.00, (2)
**VACCINATION CARD AVAILABILITY/RETENTION**	No dependent effects	-0.01, [-0.05, 0.02], 00.00, (4)	N/A	N/A	N/A	-0.01, [-0.05, 0.02], 00.00, (2)
**EXPERIENCE AND SATISFACTION WITH HEALTH SERVICES**	Not powered	0.04, [-0.015, .023], 48.22. (2)	N/A	N/A	N/A	N/A
**FORMAL HEALTH WORKER CAPACITY AND PERFORMANCE**	Not powered	0.11, [-0.07, 0.29], 0.00, (2)	N/A	N/A	N/A	N/A

For peer review only - http://bmjopen.bmj.com/site/about/guidelines.xhtml

Supplementary Table 3: Summary of all moderator analyses

For peer review only

For peer review only - http://bmjopen.bmj.com/site/about/guidelines.xhtml

	EXP INTMO	EVAL INMO	DESIGN	YEAR	REGION	CARD	POST INT V CFB	GOV IMPLEMENT	CADRES	VACCINE HESITENCY	BASELINE COVERAGE
	B (SE)	B (SE)	B (SE)	B (SE)	B (SE)	B (SE)	B (SE)	B (SE)	B (SE)	B (SE)	B (SE)
ALL EFFECTS RVE (K = 56)	-0.001 (0.003)	0.002 (0.006)	-0.10 (0.10)	-0.019* (0.007)	NP	0.06 (0.05)	-0.08 (0.05)	-0.08 (0.04)	-0.02 (0.05)	-0.04 (0.05)	-0.31 (0.15)
FULL IMMUNISATION RVE (K = 28)	0.0001 (0.002)	NP	-0.03 (0.09)	NP	NS (ALL COMPARISONS)	0.04 (0.11)	-0.16 (0.12)	-0.06 (0.10)	0.02 (0.09)	0.10 (0.19)	-0.13 (0.15)
FULL IMMUNISATION IND RE (K = 28)	-0.0002 (0.004)	-0.01 (0.02)	-0.05 (0.11)	-0.01 (0.01)	NS (ALL COMPARISONS)	0.04 (0.12)	-0.13 (0.10)	0.06 (0.12)	-0.06 (0.12)	0.17 (0.11)	-0.33 (0.25)
FULL IMMUNISATION IND EAI (K = 12)	-0.003* (0.002)	0.01 (0.01)	-0.06 (0.06)	0.01 (0.01)	0.02 (0.07)	-0.01 (0.06)	-0.04 (0.08)	0.08 (0.06)	-0.09 (0.06)	0.08 (0.07)	-0.06 (0.11)
FULL IMMUNISATION IND EID (K = 5)	N/A[C]	N/A[C]	N/A[C]	N/A[C]	N/A[C]	N/A[C]	N/A[C]	N/A[C]	N/A[C]	N/A[C]	N/A[C]
FULL IMMUNISATION IND EII (K = 2)	NP	NP	NP	NP	NP	NP	NP	NP	NP	NP	NP
FULL IMMUNISATION IND MET (K = 9)	0.005 (0.01)	-0.06 (0.06)	-0.12 (0.38)	-0.06 (0.03)	NS (ALL COMPARISONS)	N/A[B]	-0.49 (0.27)	0.04 (0.35)	N/A[B]	0.33 (0.31)	-0.66 (0.68)
PARTIAL IMMUNISATION RVE (K = 9)	NP	NP	NP	NP	NP	NP	0.10 (0.18)	NP	NP	0.27 (0.12)	N/A[E]
PARTIAL IMMUNISATION IND RE (K = 9)	-0.003 (0.006)	-0.01 (0.01)	-0.10 (0.19)	-0.03 (0.03)	-0.03 (0.19)	0.06 (0.21)	0.09 (0.18)	-0.30 (0.20)	-0.30 (0.19)	0.18 (0.19)	N/A[E]
PARTIAL IMMUNISATION IND EAI (K = 2)	NP	NP	NP	NP	NP	NP	NP	NP	NP	NP	N/A[E]
PARTIAL IMMUNISATION IND EID (K = 2)	NP	NP	NP	NP	NP	NP	NP	NP	NP	NP	N/A[E]
PARTIAL IMMUNISATION IND EII (K = 1)	NP	NP	NP	NP	NP	NP	NP	NP	NP	NP	N/A[E]
PARTIAL IMMUNISATION IND MET (K = 4)	0.01 (0.02)	-0.01 (0.01)	-0.10 (0.35)	-0.02 0.06)	0.05 (0.36)	N/A[B]	0.35 (0.33)	N/A[A]	N/A[B]	0.08 (0.35)	N/A[E]
MEASLES RVE (K = 20)	0.0001 (0.001)	NP	-0.03 (0.05)	-0.001 (0.004)	NP	0.01 (0.04)	-0.04 (0.03)	0.03 (0.03)	0.04 (0.04)	NP	N/A[E]

For peer review only

MEASLES IND RE (K = 20)	0.000 (0.001)	-0.003 (0.003)	-0.05 (0.06)	-0.003 (0.007)	NS (ALL COMPARISONS)	0.02 (0.07)	-0.04 (0.06)	0.06 (0.06)	0.05 (0.06)	0.13 (0.09)	N/A[D]
MEASLES: IND EAI (K = 10)	-0.001 (0.001)	-0.004 (0.006)	0.07 (0.05)	0.004 (0.01)	NS (ALL COMPARISONS)	0.02 (0.08)	0.05 (0.05)	-0.003 (0.05)	0.03 (0.05)	0.10 (0.09)	N/A[D]
MEASLES: IND EID (K = 2)	NP	NP	NP	NP	NP	NP	NP	NP	NP	NP	N/A[D]
MEASLES: IND EII (K = 2)	NP	NP	NP	NP	NP	NP	NP	NP	NP	NP	N/A[D]
MEASLES: IND MET (K = 6)	0.004 (0.01)	-0.004 (0.01)	-0.15 (0.31)	-0.04 (0.03)	0.18 (0.25)	0.07 (0.33)	-0.33 (0.19)	0.31 (0.20)	0.18 (0.25)	N/A[B]	N/A[D]
BCG RVE (K = 12)	NP	NP	-0.10 (0.10)	-0.03 (0.02)	-0.08 (0.12)	NP	-0.12 (0.10)	-0.10 (0.14)	NP	0.37*** (0.03)	N/A[D]
BCG IND RE (K = 12)	0.001 (0.004)	-0.01 (0.01)	-0.05 (0.11)	-0.03 ** (0.01)	0.004 (0.11)	-0.02 (0.04)	-0.12 (0.12)	-0.04 (0.13)	0.06 (0.13)	N/A[B]	N/A[D]
BCG: IND EAI (K = 4)	N/A[C]	N/A[C]	N/A[C]	N/A[C]	N/A[C]	N/A[C]	N/A[C]	N/A[C]	N/A[C]	N/A[C]	N/A[D]
BCG: IND EID (K = 2)	NP	NP	NP	NP	NP	NP	NP	NP	NP	NP	N/A[D]
BCG: IND EII (K = 2)	NP	NP	NP	NP	NP	NP	NP	NP	NP	NP	N/A[D]
BCG: IND MET (K = 4)	-0.002 (0.02)	N/A[B]	N/A[B]	-0.08 (0.04)	0.03 (0.41)	N/A[A]	-0.55*** (0.07)	0.03 (0.41)	0.03 (0.41)	N/A[B]	N/A[D]
DPT1: RVE (K = 8)	NP	NP	-0.10 (0.10)	0.001 (0.01)	NP	NP	NP	NP	NP	0.07 (0.03)	-0.13 (0.20)
DPT1: IND RE (K = 8)	-0.005 (0.01)	N/A[A]	-0.15* (0.07)	-0.004 (0.01)	NS (ALL COMPARISONS)	-0.08 (0.10)	0.02 (0.11)	0.17* (0.08)	N/A[B]	N/A[B]	N/A[E]
DPT1: IND EAI (K = 3)	NP	NP	NP	NP	NP	NP	NP	NP	NP	NP	NP
DPT1: IND EID (K = 2)	NP	NP	NP	NP	NP	NP	NP	NP	NP	NP	NP
DPT1: IND EII (K = 1)	NP	NP	NP	NP	NP	NP	NP	NP	NP	NP	NP
DPT1: IND MET (K = 2)	NP	NP	NP	NP	NP	NP	NP	NP	NP	NP	NP
DPT2 RVE (K = 5)	NP	NP	NP	NP	NP	NP	NP	NP	NP	NP	NP
DPT2 IND RE (K = 5)	N/A[C]	N/A[C]	N/A[C]	N/A[C]	N/A[C]	N/A[C]	N/A[C]	N/A[C]	N/A[C]	N/A[C]	N/A[C]
DPT2 IND EAI (K = 0)	NP	NP	NP	NP	NP	NP	NP	NP	NP	NP	NP
DPT2 IND EID (K = 2)	NP	NP	NP	NP	NP	NP	NP	NP	NP	NP	NP
DPT2 IND EII (K = 0)	NP	NP	NP	NP	NP	NP	NP	NP	NP	NP	NP
DPT2 IND MET (K = 3)	NP	NP	NP	NP	NP	NP	NP	NP	NP	NP	NP
DPT3 RVE (K = 22)	0.0003 (0.001)	NP	-0.03 (0.05)	-0.02 (0.01)	-0.01 (0.05)	NP	-0.01 (0.05)	-0.04 (0.05)	NP	NP	-0.50 (0.21)
DPT3 IND RE (K = 22)	-0.001 (0.001)	-0.002 (0.004)	0.05 (0.05)	-0.014* (0.006)	-0.02 (0.05)	0.14 (0.09)	-0.01 (0.05)	-0.01 (0.05)	-0.03 (0.06)	0.04 (0.06)	-0.40 (0.22)
DPT3 IND EAI (K = 6)	-0.001 (0.002)	0.01 (0.01)	-0.05 (0.07)	0.01 (0.01)	N/A[B]	N/A[A]	-0.08 (0.07)	0.05 (0.07)	-0.04 (0.07)	-0.03 (0.08)	-0.40 (0.22)
DPT3 IND EID (K = 6)	N/A[C]	N/A[C]	N/A[C]	N/A[C]	N/A[C]	N/A[C]	N/A[C]	N/A[C]	N/A[C]	N/A[C]	N/A[C]
DPT3 IND EII (K = 3)	NP	NP	NP	NP	NP	NP	NP	NP	NP	NP	NP

For peer review only

For peer review only - http://bmjopen.bmj.com/site/about/guidelines.xhtml

**DPT3 IND MET (K =7)**	0.003 (0.006)	-0.01** (0.004)	0.07 (0.16)	-0.03* (0.01)	-0.10 (0.15)	N/A[A]	-0.25 (0.15)	0.05 (0.17)	-0.04 (0.17)	-0.02 (0.17)	-0.56** (0.18)
OPV0 RVE (K = 5)	NP	NP	NP	NP	NP	NP	NP	NP	NP	NP	NP
**OPV0 IND RE (K = 5)**	0.02*** (0.004)	N/A[A]	0.16 (0.19)	-0.07** (0.02)	-0.20 (0.18)	0.16 (0.19)	N/A[B]	-0.21 (0.17)	N/A[A]	N/A[A]	N/A[D]
OPV0 IND EAI (K = 1)	NP	NP	NP	NP	NP	NP	NP	NP	NP	NP	N/A[D]
OPV0 IND EID (K = 2)	NP	NP	NP	NP	NP	NP	NP	NP	NP	NP	N/A[D]
OPV0 IND EII (K = 1)	NP	NP	NP	NP	NP	NP	NP	NP	NP	NP	N/A[D]
OPV0 IND MET (K = 1)	NP	NP	NP	NP	NP	NP	NP	NP	NP	NP	N/A[D]
OPV1 RVE (K = 5)	NP	NP	NP	NP	NP	NP	NP	NP	NP	NP	N/A[D]
**OPV1 IND RE (K = 5)**	N/A[C]	N/A[C]	N/A[C]	N/A[C]	N/A[C]	N/A[C]	N/A[C]	N/A[C]	N/A[C]	N/A[C]	N/A[D]
OPV01 IND EAI (K = 0)	NP	NP	NP	NP	NP	NP	NP	NP	NP	NP	N/A[D]
OPV1 IND EID (K = 2)	NP	NP	NP	NP	NP	NP	NP	NP	NP	NP	N/A[D]
OPV1 IND EII (K = 1)	NP	NP	NP	NP	NP	NP	NP	NP	NP	NP	N/A[D]
OPV1 IND MET (K = 2)	NP	NP	NP	NP	NP	NP	NP	NP	NP	NP	N/A[D]
OPV2 RVE (K = 5)	NP	NP	NP	NP	NP	NP	NP	NP	NP	NP	N/A[D]
**OPV2 IND RE (K = 5)**	0.02*** (0.003)	N/A[A]	0.22 (0.18)	-0.05** (0.02)	-0.26*** (0.07)	0.27 (0.07)	N/A[B]	0.03 (0.25)	N/A[B]	N/A[A]	N/A[D]
OPV02 IND EAI (K = 0)	NP	NP	NP	NP	NP	NP	NP	NP	NP	NP	N/A[D]
OPV2 IND EID (K = 2)	NP	NP	NP	NP	NP	NP	NP	NP	NP	NP	N/A[D]
OPV2 IND EII (K = 1)	NP	NP	NP	NP	NP	NP	NP	NP	NP	NP	N/A[D]
OPV2 IND MET (K = 2)	NP	NP	NP	NP	NP	NP	NP	NP	NP	NP	N/A[D]
**OPV3 RVE (K = 9)**	NP	NP	-0.03 (0.05)	NP	-0.24 (0.31)	NP	0.26 (0.30)	NP	-0.18 (0.33)	NP	N/A[D]
**OPV3 IND RE (K = 9)**	-0.001 (0.01)	-0.01 (0.01)	0.17 (0.23)	-0.03 (0.03)	-0.12 (0.23)	0.04 (0.32)	0.14 (0.25)	-0.34 (0.27)	-0.03 (0.27)	N/A[A]	N/A[D]
OPV3 IND EAI (K = 3)	NP	NP	NP	NP	NP	NP	NP	NP	NP	NP	N/A[D]
OPV3 IND EID (K = 1)	NP	NP	NP	NP	NP	NP	NP	NP	NP	NP	N/A[D]
OPV3 IND EII (K = 2)	NP	NP	NP	NP	NP	NP	NP	NP	NP	NP	N/A[D]
OPV3 IND MET (K = 3)	NP	NP	NP	NP	NP	NP	NP	NP	NP	NP	N/A[D]
**TIMELINESS RVE (K = 11)**	NP	NP	NP	NP	NP	NP	NP	NP	NP	NP	N/A[D]
**DPT3 TIMELINESS RE (K = 7)**	N/A[C]	N/A[C]	N/A[C]	N/A[C]	N/A[C]	N/A[C]	N/A[C]	N/A[C]	N/A[C]	N/A[C]	N/A[C]
DPT3 TIMELINESS EAI (K = 0)	--	--	--	--	--	--	--	--	--	--	--
**DPT3 TIMELINESS EID (K = 4)**	N/A[C]	N/A[C]	N/A[C]	N/A[C]	N/A[C]	N/A[C]	N/A[C]	N/A[C]	N/A[C]	N/A[C]	N/A[C]
DPT3 TIMELINESS EII (K =2)	NP	NP	NP	NP	NP	NP	NP	NP	NP	NP	NP
DPT3 TIMELINESS MET (K = 1)	NP	NP	NP	NP	NP	NP	NP	NP	NP	NP	NP
MEASLES TIMELINESS RE (K = 2)	NP	NP	NP	NP	NP	NP	NP	NP	NP	NP	NP
MEASLES TIMELINESS EAI (K = 0)	--	--	--	--	--	--	--	--	--	--	--
MEASLES TIMELINESS EID (K = 1)	NP	NP	NP	NP	NP	NP	NP	NP	NP	NP	NP
MEASLES TIMELINESS EII (K =0)	--	--	--	--	--	--	--	--	--	--	--
MEASLES TIMELINESS MET (K = 1)	NP	NP	NP	NP	NP	NP	NP	NP	NP	NP	NP
FI TIMELINESS RE (K =5)	N/A[C]	N/A[C]	N/A[C]	N/A[C]	N/A[C]	N/A[C]	N/A[C]	N/A[C]	N/A[C]	N/A[C]	N/A[C]
FI TIMELINESS EAI (K = 1)	NP	NP	NP	NP	NP	NP	NP	NP	NP	NP	NP

For peer review only - http://bmjopen.bmj.com/site/about/guidelines.xhtml

FI TIMELINESS EID (K = 2)	NP	NP	NP	NP	NP	NP	NP	NP	NP	NP	NP
FI TIMELINESS EII (K = 2)	NP	NP	NP	NP	NP	NP	NP	NP	NP	NP	NP
FI TIMELINESS MET (K = 0)	--	--	--	--	--	--	--	--	--	--	--
DROPOUT RVE (K = 5)	NP	NP	NP	NP	NP	NP	NP	NP	NP	NP	NP
DROPOUT RE (K = 5)	0.04 (0.04)	0.03 (0.03)	N/A[A]	0.33** (0.12)	-0.050 (0.183)	N/A[B]	N/A[A]	-0.23 (0.13)	N/A[A]	0.05 (0.18)	N/A[D]
DROPOUT EAI (K =2)	NP	NP	NP	NP	NP	NP	NP	NP	NP	NP	NP
DROPOUT EID (K = 0)	--	--	--	--	--	--	--	--	--	--	--
DROPOUT EII (K =1)	NP	NP	NP	NP	NP	NP	NP	NP	NP	NP	NP
DROPOUT MET (K = 2)	NP	NP	NP	NP	NP	NP	NP	NP	NP	NP	NP
MORBIDITY RVE (K = 10)	NP	NP	NP	0.01 (0.01)	NP	N/A[E]	0.02 (0.08)	NP	NP	N/A[A]	N/A[E]
MORBIDITY RE (K = 10)	0.003 (0.005)	-0.01 (0.01)	-0.04 0.11	0.01 (0.01)	NS (ALL COMPARI SONS)	-0.08 (0.10)	0.13 (0.15)	0.08 (0.11)	-0.05 (0.13)	N/A[A]	N/A[E]
MORBIDITY EAI (K = 5)	0.02** (0.01)	-0.01 (0.02)	N/A[B]	0.03 (0.05)	0.04 (0.22)	N/A[D]	0.12 (0.21)	0.26 (0.22)	0.12 (0.27)	N/A[A]	N/A[E]
MORBIDITY EID (K = 2)	NP	NP	NP	NP	NP	NP	NP	NP	NP	NP	NP
MORBIDITY EII (K = 1)	NP	NP	NP	NP	NP	NP	NP	NP	NP	NP	NP
MORBIDITY MET (K = 2)	NP	NP	NP	NP	NP	NP	NP	NP	NP	NP	NP
MORTALITY: RVE (K = 6)	NP	NP	NP	NP	NP	N/A[E]	NP	NP	NP	N/A[A]	N/A[D]
MORTALITY RE (6)	-0.002 (0.001)	0.002 (0.002)	0.03 (0.05)	0.01*** (0.003)	NS (ALL COMPARI SONS)	N/A[D]	-0.05 (0.05)	0.02 (0.05)	0.02 (0.05)	N/A[A]	N/A[D]
MORTALITY EAI (3)	NP	NP	NP	NP	NP	NP	NP	NP	NP	NP	NP
MORTALITY EID (0)	NP	NP	NP	NP	NP	NP	NP	NP	NP	NP	NP
MORTALITY EII (0)	NP	NP	NP	NP	NP	NP	NP	NP	NP	NP	NP
MORTALITY MET (3)	NP	NP	NP	NP	NP	NP	NP	NP	NP	NP	NP
KNOWLEDGE RVE (K = 7)	NP	NP	-0.10 (0.10)	NP	NP	NP	-0.06 (0.13)	NP	NP	NP	N/A[D]
KNOWLEDGE RE (K = 9)	-0.02 (0.01)	-0.01 (0.02)	0.44*** (0.11)	-0.01 (0.02)	0.44*** (0.11)	N/A[D]	-0.05 (0.18)	0.05 (0.23)	N/A[A]	-0.01 (0.23)	N/A[D]
KNOWLEDGE EAI (K = 4)	N/A[E]	N/A[E]	0.52* (0.21)	-0.05 (0.05)	0.52* (0.21)	N/A[E]	N/A[B]	N/A[B]	N/A[A]	N/A[A]	N/A[D]
KNOWLEDGE EID (K = 3)	NP	NP	NP	NP	NP	NP	NP	NP	NP	NP	NP
KNOWLEDGE EII (K = 0)	--	--	--	--	--	--	--	--	--	--	--
KNOWLEDGE MET (K = 2)	NP	NP	NP	NP	NP	NP	NP	NP	NP	NP	NP
ATTITUDES RVE (NO DEPENDENT EFFECTS)	N/A	N/A	N/A	N/A	N/A	N/A	N/A	N/A	N/A		
ATTITUDES RE (K = 6)	0.01 (0.02)	-0.004 (0.02)	0.17 (0.34)	-0.01 (0.04)	0.17 (0.34)	N/A[E]	0.38 (0.29)	-0.09 (0.43)	N/A[A]	N/A[B]	N/A[D]

For peer review only - http://bmjopen.bmj.com/site/about/guidelines.xhtml

ATTITUDES EAI (K = 2)	NP	NP	NP	NP	NP	NP	NP	NP	NP	NP	NP
ATTITUDES EID (K = 2)	NP	NP	NP	NP	NP	NP	NP	NP	NP	NP	NP
ATTITUDES EII (K = 0)	--	--	--	--	--	--	--	--	--	--	--
ATTITUDES MET (K = 2)	NP	NP	NP	NP	NP	NP	NP	NP	NP	NP	NP
CARD AVAIL/ RETENTION RVE (NO DEPENDENT EFFECTS)	N/A	N/A	N/A	N/A	N/A	N/A	N/A	N/A	N/A	N/A	N/A
CARD AVAIL/ RETENTION RE (K = 4)	N/A[C]	N/A[C]	N/A[C]	N/A[C]	N/A[C]	N/A[C]	N/A[C]	N/A[C]	N/A[C]	N/A[C]	N/A[D]
CARD AVAIL/ RETENTION EAI (K = 1)	NP	NP	NP	NP	NP	NP	NP	NP	NP	NP	NP
CARD AVAIL/ RETENTION EID (K = 0)	--	--	--	--	--	--	--	--	--	--	--
CARD AVAIL/ RETENTION EII (K = 1)	NP	NP	NP	NP	NP	NP	NP	NP	NP	NP	NP
CARD AVAIL/ RETENTION MET (K = 2)	NP	NP	NP	NP	NP	NP	NP	NP	NP	NP	NP
SATISFACTION W/ HEALTH WORKERS (K = 2)	NP	NP	NP	NP	NP	NP	NP	NP	NP	NP	NP
SATISFACTION W/ HEALTH WORKERS (K = 1)	NP	NP	NP	NP	NP	NP	NP	NP	NP	NP	NP
SATISFACTION W/ HEALTH WORKERS EAI (K = 1)	NP	NP	NP	NP	NP	NP	NP	NP	NP	NP	NP
SATISFACTION W/ HEALTH WORKERS EID (K = 1)	NP	NP	NP	NP	NP	NP	NP	NP	NP	NP	NP
SATISFACTION W/ HEALTH WORKERS EII (K = 0)	--	--	--	--	--	--	--	--	--	--	--
SATISFACTION W/ HEALTH WORKERS MET (K = 0)	--	--	--	--	--	--	--	--	--	--	--
FORMAL HEALTH WORKERS MOTIVATION, CAPACITY, AND PERFORMANCE (K = 2)	NP	NP	NP	NP	NP	NP	NP	NP	NP	NP	NP
FORMAL HEALTH WORKERS MOTIVATION, CAPACITY, AND PERFORMANCE (K = 1)	NP	NP	NP	NP	NP	NP	NP	NP	NP	NP	NP
FORMAL HEALTH WORKERS MOTIVATION, CAPACITY, AND PERFORMANCE EAI (K = 1)	NP	NP	NP	NP	NP	NP	NP	NP	NP	NP	NP
FORMAL HEALTH WORKERS MOTIVATION, CAPACITY, AND PERFORMANCE EID (K = 1)	NP	NP	NP	NP	NP	NP	NP	NP	NP	NP	NP

For peer review only - http://bmjopen.bmj.com/site/about/guidelines.xhtml

For peer review only

FORMAL HEALTH WORKERS MOTIVATION, CAPACITY, AND PERFORMANCE EII (K = 0)	--	--	--	--	--	--	--	--	--	--	--
FORMAL HEALTH WORKERS MOTIVATION, CAPACITY, AND PERFORMANCE MET (K = 0)	--	--	--	--	--	--	--	--	--	--	--

Note. NS = non-significant, NP = not powered, EAI = engagement as the intervention, EID = engagement in the intervention design, EII = engagement in implementation autonomy, MET = multiple engagement types, RVE = robust variance estimation, RE = random effects model using independent effects.

[a] Not applicable because all studies were in the same moderator group
[b] Not applicable because only one study was in a different moderator group
[c] Not applicable because there was no heterogeneity
[d] Not applicable because the moderator does not apply to the outcome category
[e] Not applicable because there was missing data
* $p < .05$, ** $p < .01$, *** $p < .001$

For peer review only - http://bmjopen.bmj.com/site/about/guidelines.xhtml

Appendix 10: Quantitative results – Engagement as intervention

Appendix 10 presents supplementary data for analyses presented in the main body of the manuscript for the primary outcomes of community engagement interventions using engagement as the intervention, including funnel plots of publication bias and outlier analyses. It also includes a full presentation of the supplementary outcomes that were precluded from the main body of the manuscript due to word limitations.

Full immunisation

Studies that used engagement as the intervention had a significant effect on full childhood immunisation ($\hat{\mu} = 0.08$ [95% CI: 0.03 to 0.13], $z = 3.02$, $p < 0.01$). A 95% credibility/prediction interval for the true outcomes is given by -0.06 to 0.22. Hence, although the average outcome is estimated to be positive, in some studies the true outcome may in fact be negative.

According to the Q-test, the true outcomes appear to be heterogeneous ($Q(11) = 36.67$, $p < 0.01$, $\hat{\tau}^2 = 0.005$, $I^2 = 70.00\%$). An examination of the studentized residuals revealed that none of the studies had a value larger than ± 2.87 and hence there was no indication of outliers in the context of this model. This was confirmed by a leave-one-out analysis. According to the Cook's distances, none of the studies could be considered to be overly influential. The forest plot is presented below (Supplemenary Figure 33). With only one high or moderate quality study, sensitivity analysis by study quality could not be completed for this body of evidence. A funnel plot of the estimates is shown in Figure B1B. Neither the rank correlation nor the regression test indicated any funnel plot asymmetry ($p = 0.20$ and $p = 0.07$, respectively). Exposure to the intervention (in months) was the only significant source of heterogeneity such that for each additional month of intervention exposure, effects decreased by .003 standard deviation units ($\hat{B} = -0.003, p = 0.049$ [95% CI: -0.01 to -0.00001]).

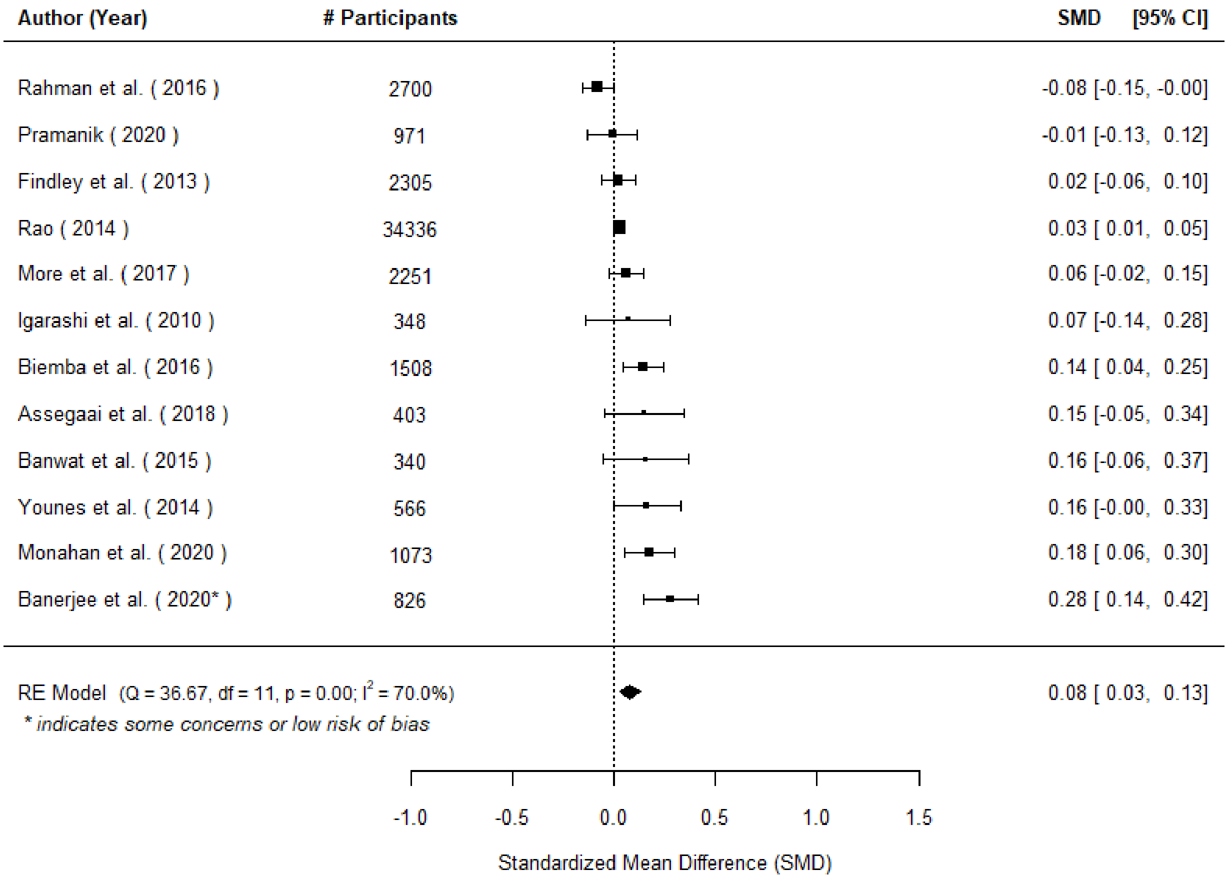

For peer review only - http://bmjopen.bmj.com/site/about/guidelines.xhtml

Supplementary Figure 33. Forest plot showing the observed outcomes and the estimate of the random-effects model for interventions with community engagement as the intervention on full immunisation

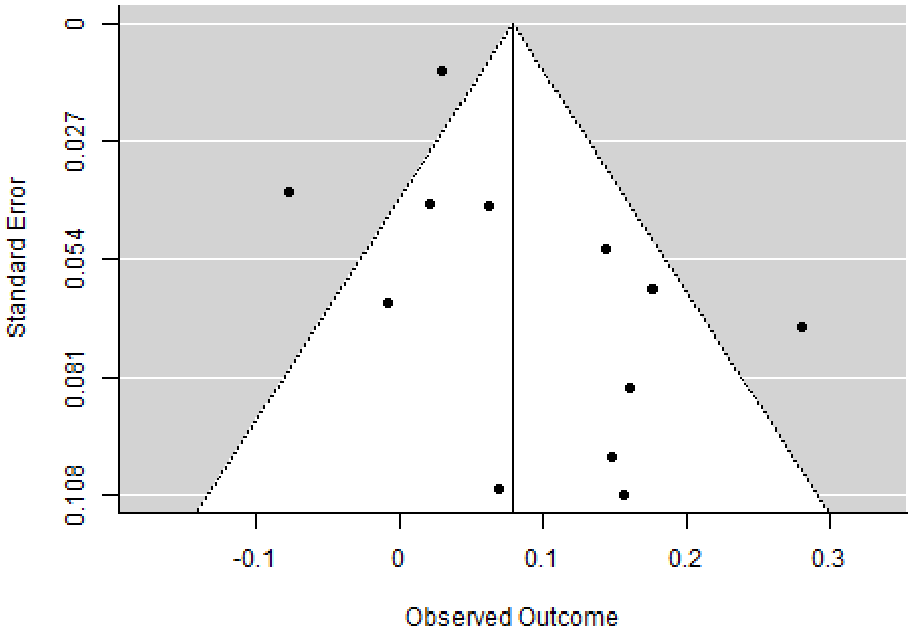

Supplementary Figure 33b. Forest plot showing the observed outcomes and the estimate of the random-effects model for interventions with community engagement as the intervention on full immunisation

DPT3

Studies that used engagement as the intervention also had a significant effect on DPT3 vaccination ($\hat{\mu} = 0.09$ [95% CI: 0.03 to 0.15], $z = 3.01$, $p < 0.01$). A 95% credibility/prediction interval for the true outcomes is given by -0.04 to 0.22. Hence, although the average outcome is estimated to be positive, in some studies the true outcome may in fact be negative.

According to the Q-test, the true outcomes appear to be heterogeneous ($\mathrm{Q}(5) = 18.64$, $\mathrm{p} < 0.01$, $\hat{\tau}^2 = 0.00$, $\mathrm{I}^2 = 73.17\%$). An examination of the studentized residuals revealed that none of the studies had a value larger than ± 2.64 and hence there was no indication of outliers in the context of this model. This was confirmed by a leave-one-out analysis. According to the Cook's distances, none of the studies could be considered to be overly influential. None of the moderators we tested were significant sources of heterogeneity (see Supplementary Table 3 in Appendix 9).

For peer review only - http://bmjopen.bmj.com/site/about/guidelines.xhtml

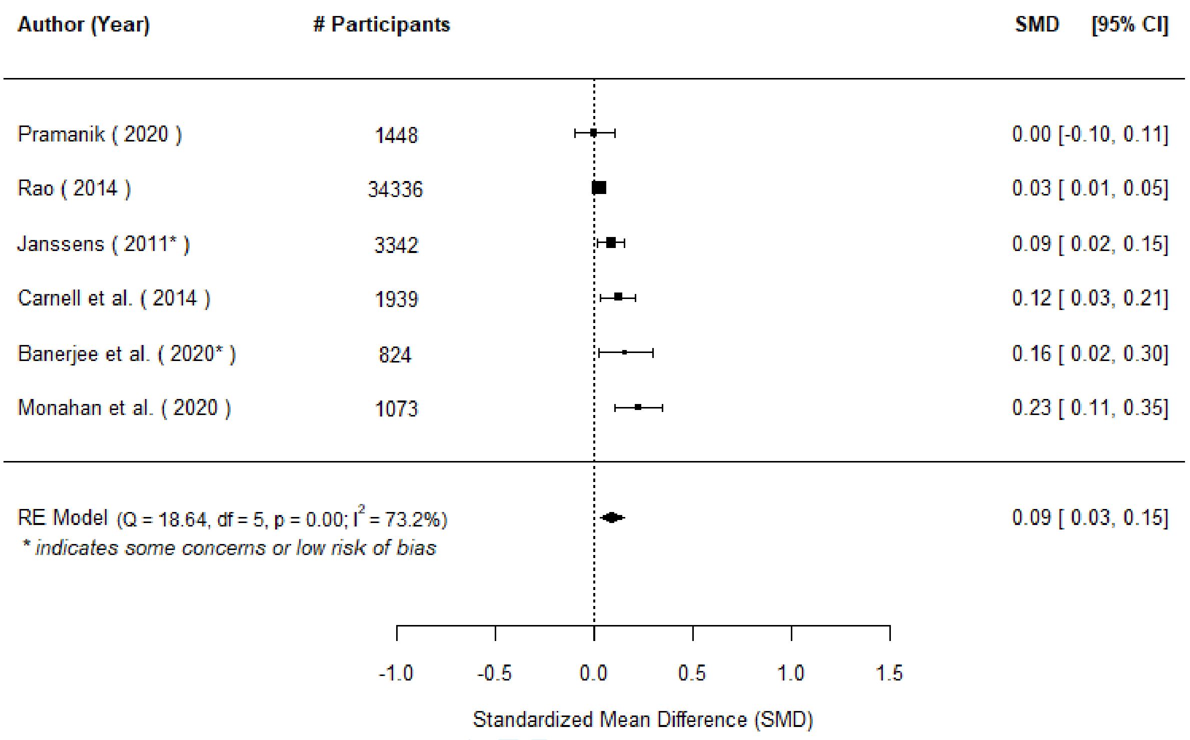

Supplementary Figure 34. Forest plot showing the observed outcomes and the estimate of the random-effects model for interventions with community engagement as the intervention on DPT3

Measles

Studies that used engagement as the intervention also had a significant effect on measles vaccination ($\hat{\mu} = 0.10$ [95% CI: 0.05 to 0.15], $z = 3.89$, $p < 0.001$). A 95% credibility/prediction interval for the true outcomes is given by -0.02 to 0.21. Hence, although the average outcome is estimated to be positive, in some studies the true outcome may in fact be negative.

According to the Q-test, the true outcomes appear to be heterogeneous ($Q(9) = 22.67$, $p = 0.007$, $\hat{\tau}^2 = 0.003$, $I^2 = 60.29\%$). An examination of the studentized residuals revealed that none of the studies had a value larger than ± 2.81 and hence there was no indication of outliers in the context of this model. This was confirmed by a leave-one-out analysis. According to the Cook's distances, none of the studies could be considered to be overly influential.

A funnel plot of the estimates is shown in Figure 35b. The regression test indicated funnel plot asymmetry ($p < 0.01$) but not the rank correlation test ($p = 0.38$). None of the moderators we tested were significant sources of heterogeneity (see Supplementary Table 3 in Appendix 9).

For peer review only - http://bmjopen.bmj.com/site/about/guidelines.xhtml

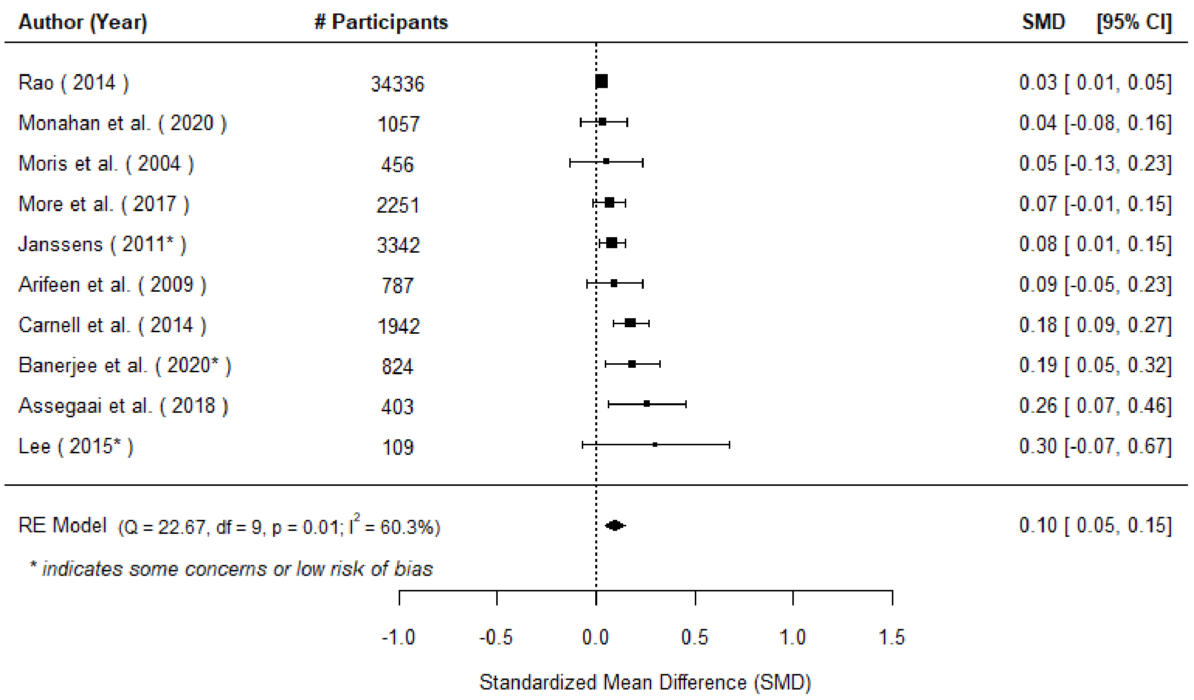

Supplementary Figure 35. Forest plot showing the observed outcomes and the estimate of the random-effects model for interventions with community engagement as the intervention on measles

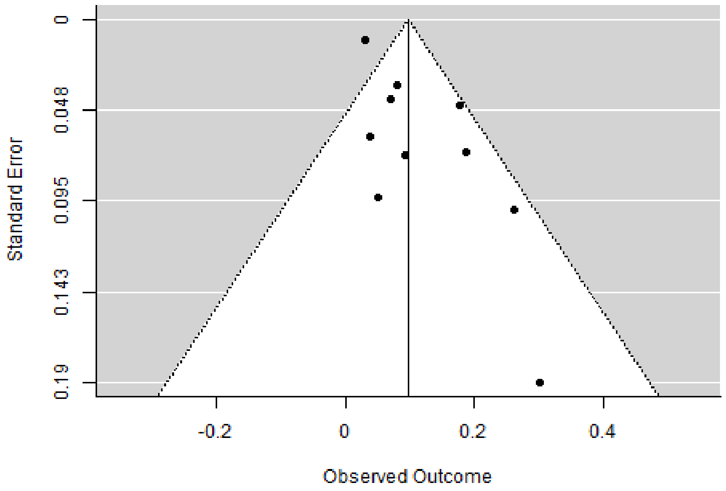

Supplementary Figure 35b. Funnel plot showing studies of community engagement as the intervention on measles

Timeliness of full childhood immunisation

Only one study (Saggurti 2018) fell into this intervention/outcome category, thus we were unable to perform a statistical synthesis. This quasi-experimental study from India found a small but significant impact of their programme on full childhood immunisation (g = 0.15 [95% CI: 0.04 to 0.27]), but like most studies, it was assessed as having a high risk of bias.

For peer review only - http://bmjopen.bmj.com/site/about/guidelines.xhtml

Timeliness of DPT3 vaccination

No studies examining timeliness of DPT3 vaccinations used engagement as the intervention.

Timeliness of measles vaccination

No studies examining timeliness of measles vaccinations used engagement as the intervention.

Secondary outcomes

Partial immunisation

Only two studies reporting on partial immunisation used interventions with community engagement as the intervention. The estimated average outcome based on the random-effects model was $\hat{\mu} = 0.31$ ([95% CI: -0.25 to 0.87], $z = 1.10, p = 0.27$), indicating no significant difference between the intervention group and the control group on partial immunisation (see Supplementary Figure 36). Given the small number of studies, this result should be interpreted with caution. A 95% credibility/prediction interval for the true outcomes is given by 0.01 to 0.71. According to the Q-test, the true outcomes appear to be heterogeneous ($Q(1) = 95.93$, $p < 0.001$, $\hat{\tau}^2 = 0.16$, $I^2 = 98.96\%$). With only two studies, moderator and leave-one-out analyses were not appropriate and tests of publication bias are not valid.

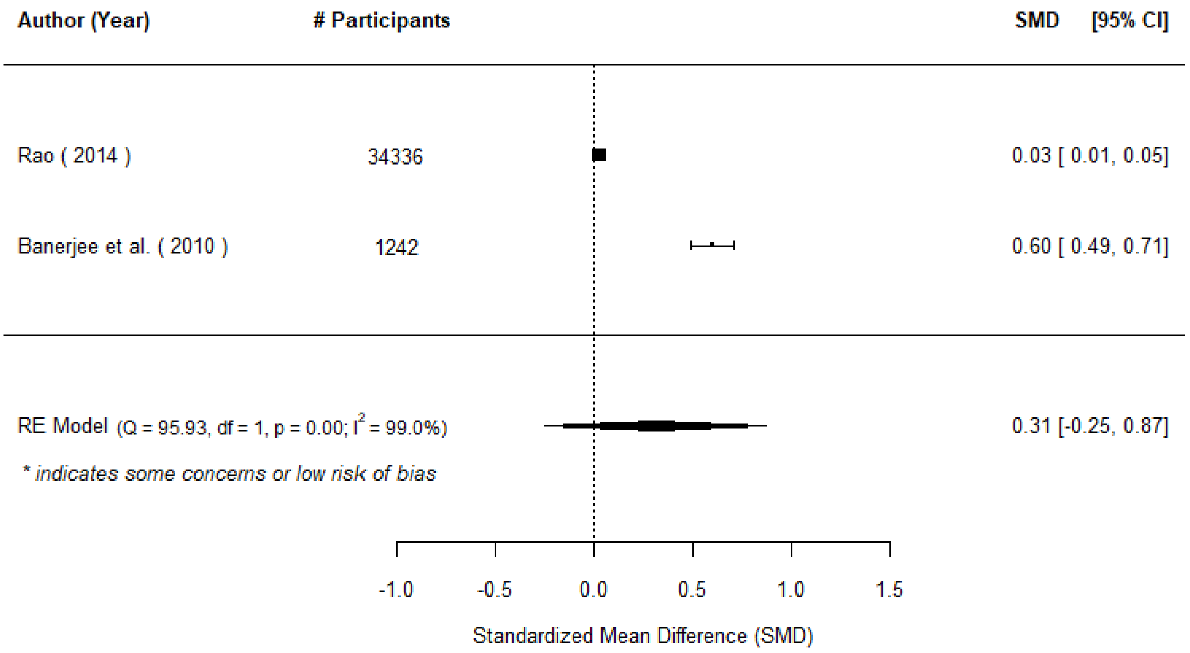

Supplementary Figure 36. Forest plot showing the observed outcomes and the estimate of the random-effects model for interventions with community engagement as the intervention on partial immunisation.

BCG

We included a total of $k = 4$ studies the analysis. The estimated average outcome based on the random-effects model was $\hat{\mu} = 0.02$ (95% CI: 0.01 to 0.03). Therefore, the average outcome differed significantly from zero ($z = 3.32$, $p < 0.01$), indicating a very small but significant benefit to the treated group compared to the control group (see Supplementary Figure 37). A 95% credibility/prediction interval for the true outcomes is given by 0.01 to 0.03. According to the Q-test, there was no significant amount of heterogeneity in the true outcomes ($Q(3) = 2.98$, $p = 0.39$, $\hat{\tau}^2 = 0.00$, $I^2 = 0.00\%$), thus we did not examine potential sources of heterogeneity for this model. An examination of the studentized residuals revealed that none of the studies had a value larger than ± 2.50 and hence there was no indication of outliers in the context of this model. This was confirmed by a leave one out analysis.According to the Cook's distances, none of the studies could be considered to be overly influential.

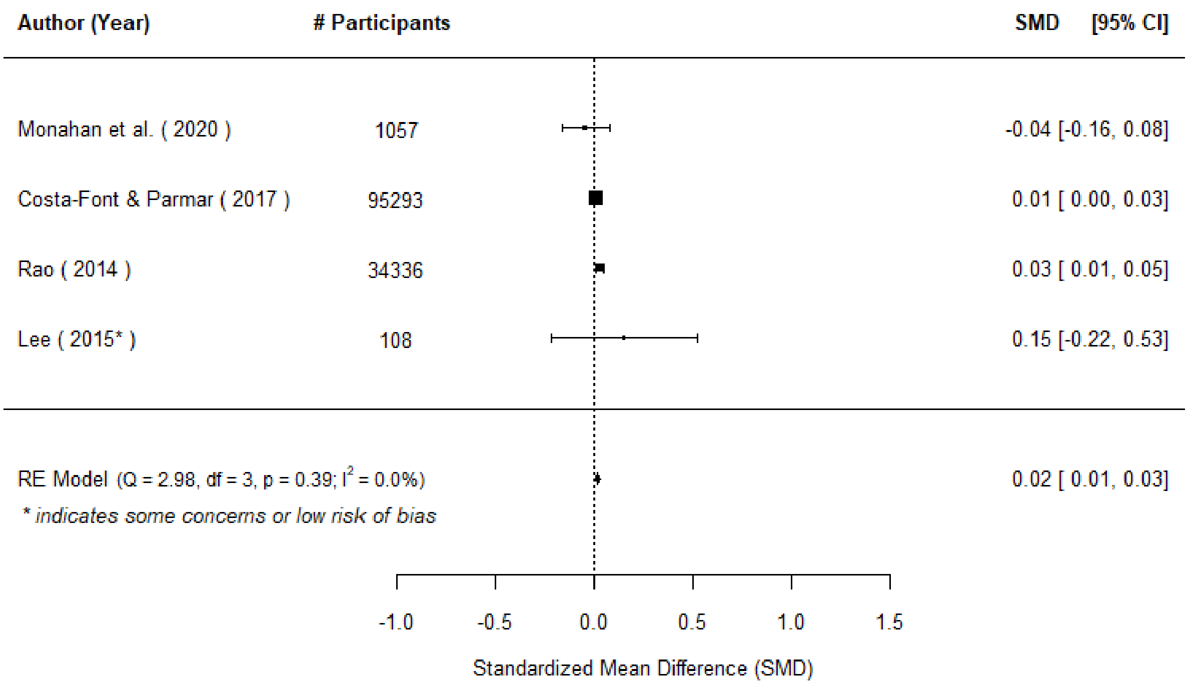

Supplementary Figure 37. Forest plot showing the observed outcomes and the estimate of the random-effects model for interventions with community engagement as the intervention on BCG vaccination.

DPT1

We included a total of $k = 3$ studies in the analysis. The estimated average outcome based on the random-effects model was $\hat{\mu} = 0.10$ ([95% CI: -0.03 to 0.22], $z = 1.53$, $p = 0.13$), indicating no difference between the treatment and control groups (see Supplementary Figure 38). A 95% credibility/prediction interval for the true outcomes is given by -0.14 to 0.33. Hence, although the average outcome is estimated to be positive, in some studies the true outcome may in fact be negative.

According to the Q-test, the true outcomes appear to be heterogeneous ($Q(2) = 20.42$, $p < 0.01$, $\hat{\tau}^2 = 0.01$, $I^2 = 90.21\%$). An examination of the studentized residuals revealed that one study (Costa-Font & Parmar 2017) had a value larger than ± 2.39 and may be a potential outlier in the context of this model. Indeed, sensitivity analyses leaving each study out indicated that removing Costa-Font & Parmar (2017) would increase the overall average effect ($\hat{\mu}$ = 0.16 (95% CI: 0.09 to 0.22), and the resulting effect would be positive and significant (z = 4.72, p < .001). Leave-one-out analyses indicated there were no other studies whose removal impacted the average effect estimate. According to the Cook's distances, none of the studies could be considered to be overly influential. With

For peer review only - http://bmjopen.bmj.com/site/about/guidelines.xhtml

only three studies, we were unable to conduct moderator analyses or test for publication bias. Only one study (Banerjee et al. 2020) was not assessed as high risk of bias.

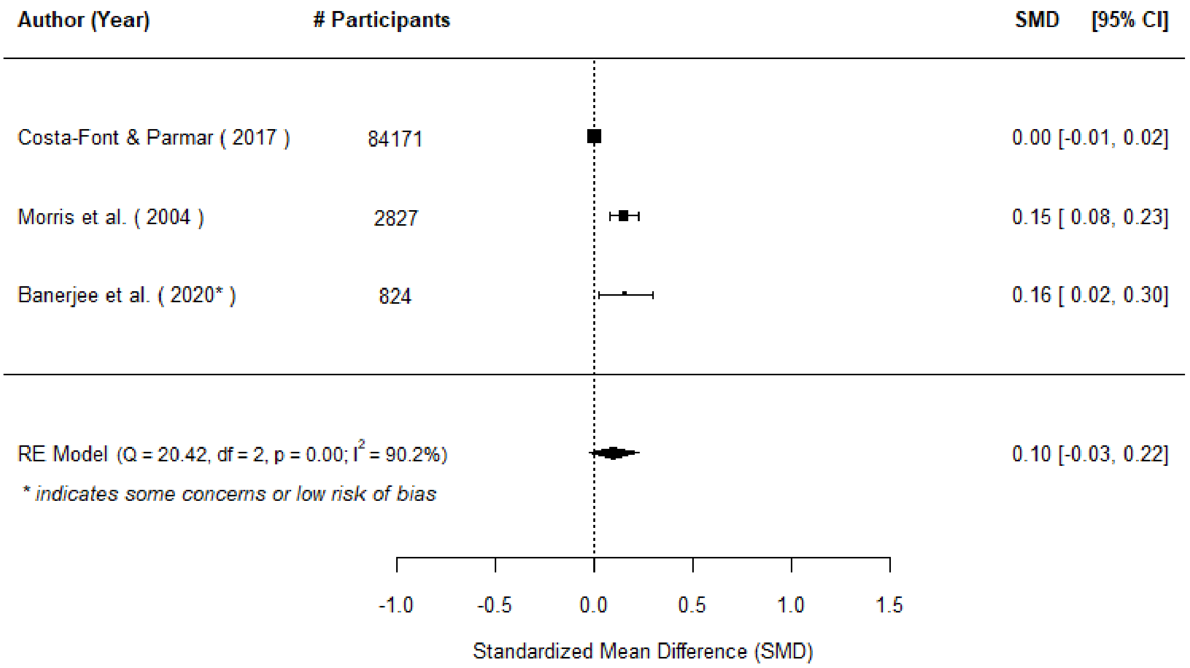

Supplementray Figure 38. *Forest plot showing the observed outcomes and the estimate of the random-effects model for interventions with community engagement as the intervention on DPT1 vaccination.*

DPT2

Only one study (Banerjee et al. 2020) fell into this intervention/outcome category, thus we were unable to perform a statistical synthesis. This cluster RCT from India found a small but significant positive effect of their programme on DPT2 vaccination (g = 0.15 [95% CI: 0.01 to 0.29]), but like most studies, it was assessed as having a high risk of bias.

OPV0

Only one study (Costa-Font & Parmar 2017) fell into this intervention/outcome category, thus we were unable to perform a statistical synthesis. This quasi-experimental study from India found a null effect of their programme on OPV0 vaccination (g = 0.01 [95% CI: -0.01 to 0.03]), but like most studies, it was assessed as having a high risk of bias.

OPV1

There were no studies reporting on OPV1 vaccination that used interventions with community engagement as the intervention.

OPV2

For peer review only - http://bmjopen.bmj.com/site/about/guidelines.xhtml

There were no studies reporting on OPV2 vaccination that used interventions with community engagement as the intervention.

OPV3

We included a total of $k = 3$ studies in the analysis. The estimated average outcome based on the random-effects model was $\hat{\mu} = 0.16$ ([95% CI: -0.02 to 0.34], $z = 1.78$, $p = 0.07$), indicating no significant difference between the intervention group and the control group on OPV3 vaccination (see Supplementary Figure 39). A 95% credibility/prediction interval for the true outcomes is given by -0.16 to 0.48. Hence, although the average outcome is estimated to be positive, in some studies the true outcome may in fact be negative.

According to the Q-test, the true outcomes appear to be heterogeneous ($Q(2) = 12.37$, $p = 0.002$, $\hat{\tau}^2 = 0.02$, $I^2 = 83.83\%$). An examination of the studentized residuals revealed that one study (Lee 2015) had a value larger than ± 2.39 and may be a potential outlier in the context of this model. Indeed, sensitivity analyses leaving each study out indicated that removing Lee (2015) would reduce the overall average effect ($\hat{\mu} = 0.08$ [95% CI: -0.04 to 0.19]), though in either case the effect is not significantly different from zero ($z = 1.29$, $p = 0.20$). Leave-one-out analyses indicated there were no other studies whose removal impacted the average effect estimate. According to the Cook's distances, none of the studies could be considered to be overly influential. With only three studies contributing effects, moderator analyses were not appropriate.

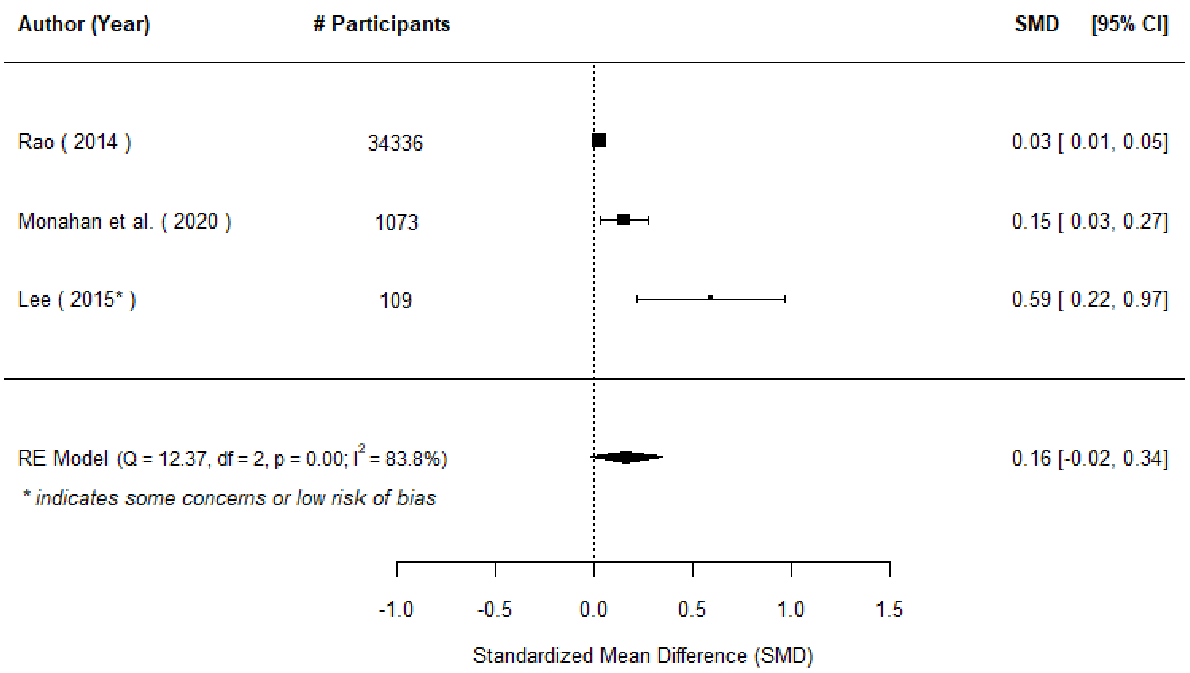

Supplementary Figure 39. Forest plot showing the observed outcomes and the estimate of the random-effects model for interventions with community engagement as the intervention on OPV3 vaccination.

Dropouts

Only two studies reporting on vaccination dropouts used interventions with community engagement as the intervention. The estimated average outcome based on the random-effects model was $\hat{\mu} = 0.02$ ([95% CI: -0.03 to 0.06], $z = 0.63$, $p = 0.53$), indicating no significant difference between the intervention group and the control group

For peer review only - http://bmjopen.bmj.com/site/about/guidelines.xhtml

on vaccination dropouts (see Supplementary Figure 40). Given the small number of studies, this result should be interpreted with caution. According to the Q-test, the true outcomes appear to be homogeneous ($Q(1) = 0.03$, $p = 0.86$, $\hat{\tau}^2 = 0.00$, $I^2 = 0.00\%$). With only two studies and no heterogeneity, moderator and leave-one-out analyses were not appropriate and tests of publication bias are not valid.

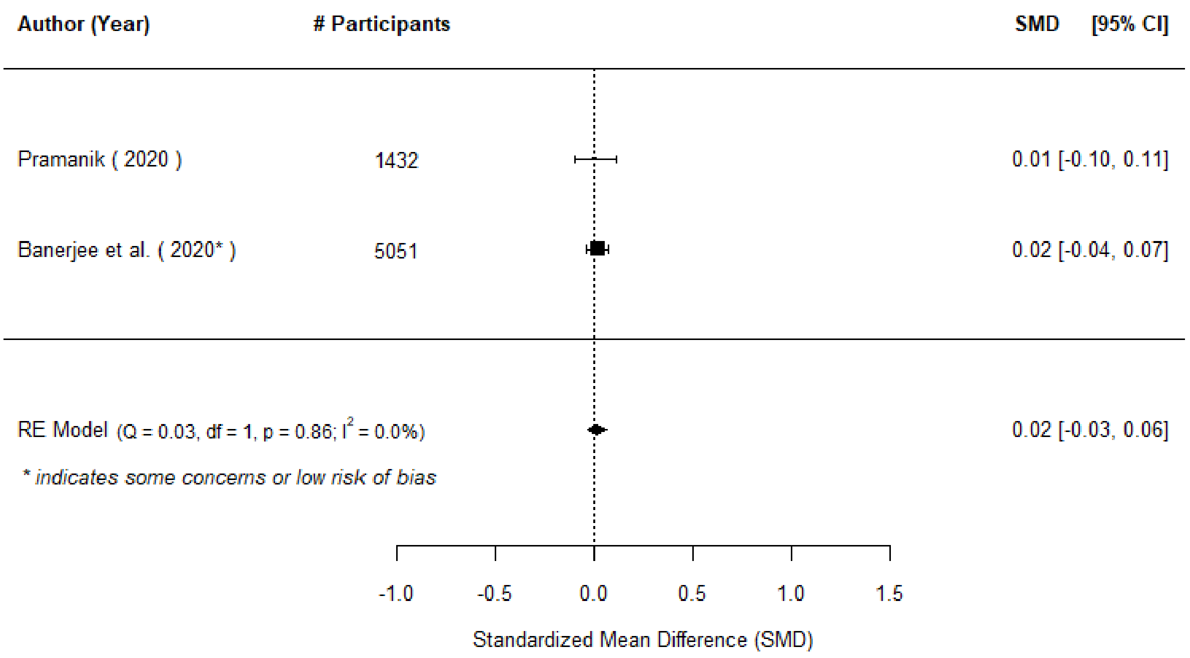

Supplementary Figure 40. Forest plot showing the observed outcomes and the estimate of the random-effects model for interventions with community engagement as the intervention on vaccination dropouts.

Morbidity

We used reports of diarrhea (most typically in the past two weeks) as a proxy for childhood morbidity. In all cases, effects were reverse coded such that positive effects always indicate a benefit to the treated group. Thus, a positive effect here would be interpreted as a reduction in diarrhea among treated participants compared to control participants. We included a total of $k = 5$ studies in the analysis. The estimated average outcome based on the random-effects model was $\hat{\mu} = -0.004$ (95% CI: -0.11 to 0.10). Therefore, the average outcome did not differ significantly from zero ($z = -0.08$, $p = 0.94$), indicating no difference between the treatment and control groups on childhood morbidity (see Supplementary Figure 41). A 95% credibility/prediction interval for the true outcomes is given by -0.24 to 0.23. Hence, although the average outcome is estimated to be negative, in some studies the true outcome may in fact be positive.

According to the Q-test, the true outcomes appear to be heterogeneous ($Q(4) = 35.91$, $p < 0.01$, $\hat{\tau}^2 = 0.01$, $I^2 = 88.86\%$). An examination of the studentized residuals revealed that one study (Assegaai et al. 2018) had a value larger than ± 2.58 and may be a potential outlier in the context of this model. Indeed, sensitivity analyses leaving each study out indicated that removing Assegaai and colleagues (2018) would reduce the overall average effect ($\hat{\mu}$ = -0.06 (95% CI: -0.14 to 0.02). While the resulting effect would be negative, the effect would still be non-significant (z = -1.50, p = .13). Leave-one-out analyses confirmed that no other studies exerted a disproportionate influence on the estimated average effect. According to the Cook's distances, none of the studies could be considered to be overly influential. There was only one study not assessed as high risk of bias, so we were unable to do a sensitivity analysis by study quality. Exposure to the intervention was a significant source of heterogeneity such that each additional

For peer review only - http://bmjopen.bmj.com/site/about/guidelines.xhtml

month of exposure increased the positive impact on morbidity by 0.02 standard deviation units $\hat{\mu} = 0.02$ (95% CI: 0.01 to 0.03, $p = 0.002$). No other moderators were significant (see Supplementary Table 3).

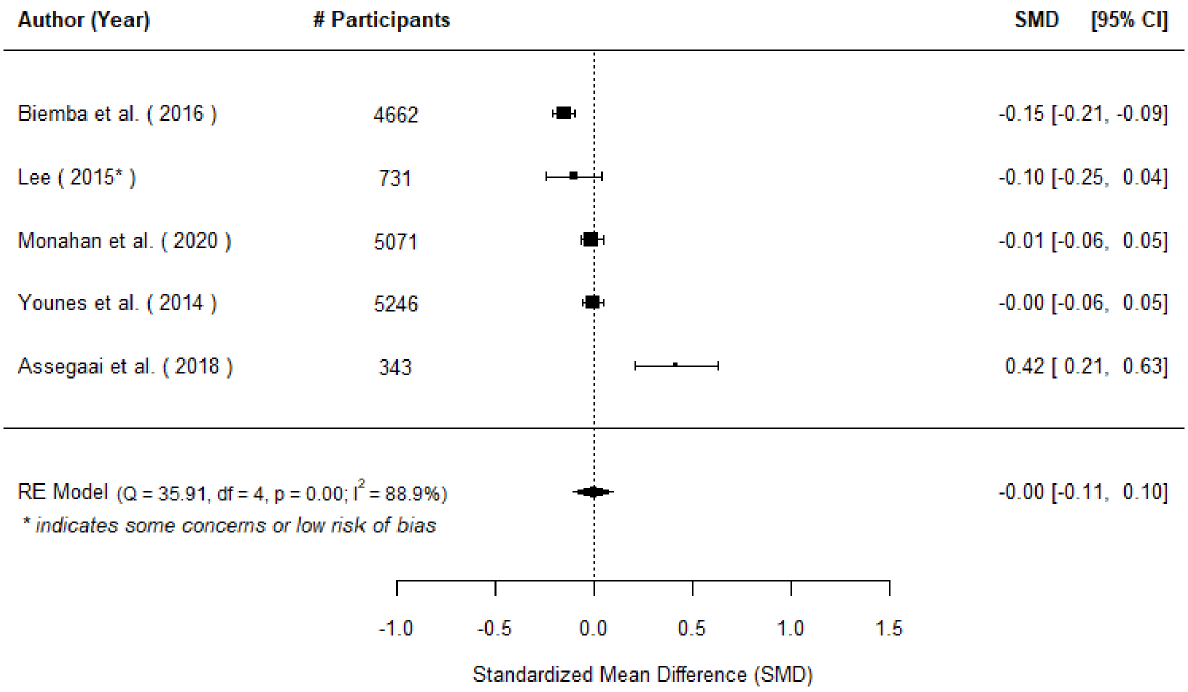

Supplementary Figure 41. Forest plot showing the observed outcomes and the estimate of the random-effects model for interventions with community engagement as the intervention on morbidity.

Mortality

We included a total of $k = 3$ studies in the analysis. The estimated average outcome based on the random-effects model was $\hat{\mu} = -0.04$ (95% CI: -0.11 to 0.04). Therefore, the average outcome did not differ significantly from zero ($z = -0.94$, $p = 0.34$), indicating no difference between the treatment and control group (see Supplementary Figure 42). A 95% credibility/prediction interval for the true outcomes is given by -0.18 to 0.10. Hence, although the average outcome is estimated to be negative, in some studies the true outcome may in fact be positive.

According to the Q-test, the true outcomes appear to be heterogeneous ($Q(2) = 10.35$, $p < 0.01$, $\hat{\tau}^2 = 0.00$, $I^2 = 80.68\%$). An examination of the studentized residuals revealed that one study (Monahan et al. 2020) had a value larger than ± 2.39 and may be a potential outlier in the context of this model. Indeed, sensitivity analyses leaving each study out indicated that removing Monahan and colleagues (2020) would reduce the overall average effect ($\hat{\mu}$ = 0.01 [95% CI: -0.14 to 0.16]), but the effect would still be positive and non-significant (z = 0.10, p = .92). Leave-one-out analyses confirmed that no other studies exerted a disproportionate influence on the estimated average effect. According to the Cook's distances, none of the studies could be considered to be overly influential. With only three studies contributing effects, we were unable to test for potential sources of heterogeneity.

For peer review only - http://bmjopen.bmj.com/site/about/guidelines.xhtml

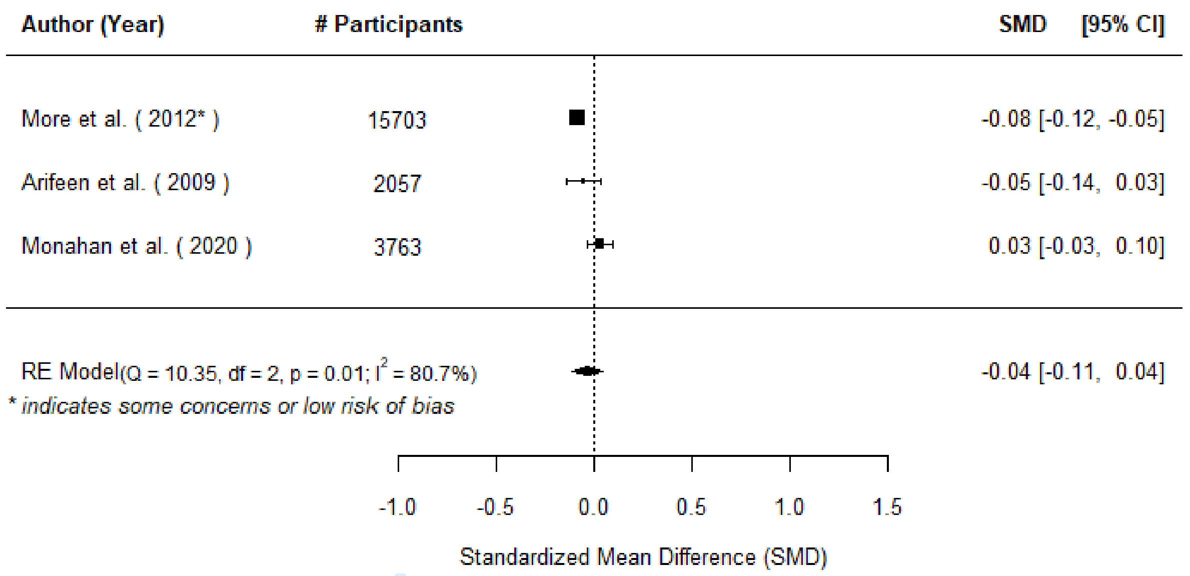

Supplementary Figure 42. Forest plot showing the observed outcomes and the estimate of the random-effects model for interventions with community engagement as the intervention on mortality.

Immunisation knowledge

We included a total of $k = 4$ studies in the analysis. The estimated average outcome based on the random-effects model was $\hat{\mu} = 0.20$ (95% CI: -0.08 to 0.49). Therefore, the average outcome did not differ significantly from zero ($z = 1.41$, $p = 0.16$), indicating no difference between the treatment and control groups. A 95% credibility/prediction interval for the true outcomes is given by -0.41 to 0.81. Hence, although the average outcome is estimated to be positive, in some studies the true outcome may in fact be negative.

According to the Q-test, the true outcomes appear to be heterogeneous ($Q(3) = 48.39$, $p < 0.01$, $\hat{\tau}^2 = 0.08$, $I^2 = 93.80\%$). An examination of the studentized residuals revealed that one study (Banwat et al. 2015) had a value larger than ± 2.50 and may be a potential outlier in the context of this model. Indeed, sensitivity analyses leaving each study out indicated that removing Banwat and colleagues (2015) would reduce the overall average effect ($\hat{\mu}$ = 0.02 (95% CI: -0.12 to 0.17), but the effect would still be positive and non-significant (z = 0.35, p = .73). Leave-one-out analyses confirmed that no other studies exerted a disproportionate influence on the estimated average effect. According to the Cook's distances, none of the studies could be considered to be overly influential.

We examined potential sources of heterogeneity, and study design was a significant predictor of immunisation knowledge such that studies using quasi-experimental designs had larger average effects than RCT designs by .52 standard deviation units ($\hat{\beta} = 0.52$ [95% CI: 0.11 to 0.94], p = 0.01). Region was also a significant predictor, but region was perfectly confounded with study design (e.g. all studies from South Asia were RCTs, while all studies from Sub-Saharan Africa were all quasi-experimental designs), so the effects were larger in Sub-Saharan Africa than from South Asia by the same .52 standard deviation units ($\beta = 0.52$ [95% CI: 0.11 to 0.94], p = 0.01). No other moderators were significant (see Supplementary Table 3 in Appendix 9)

For peer review only - http://bmjopen.bmj.com/site/about/guidelines.xhtml

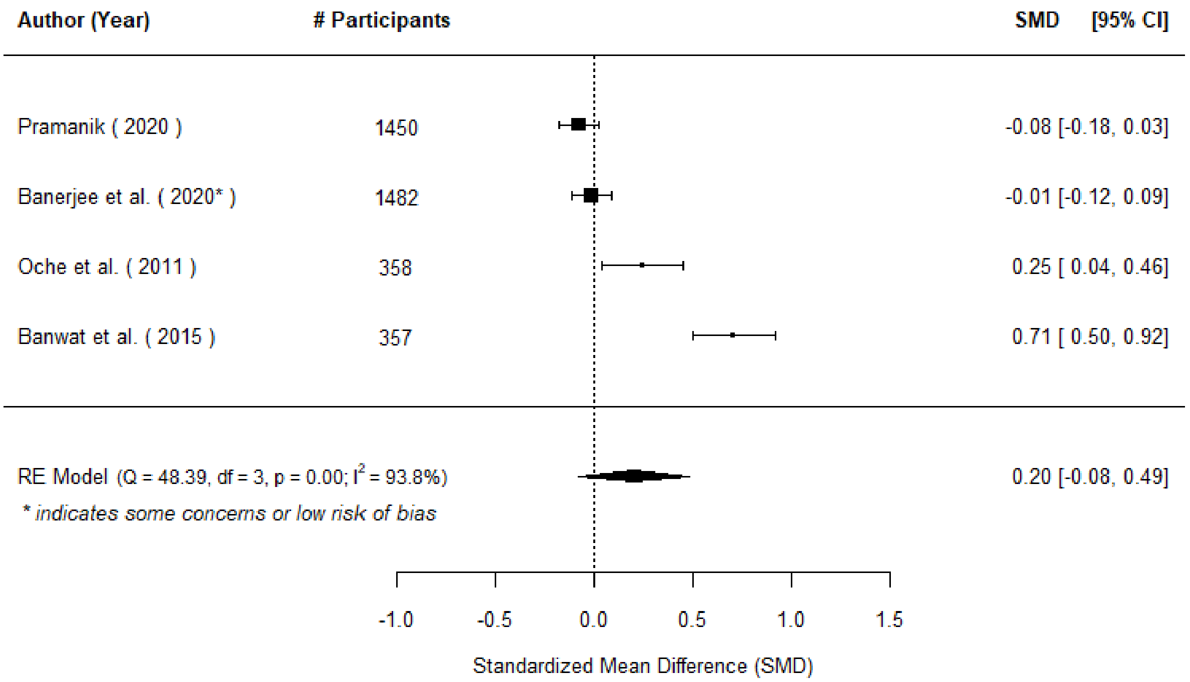

Supplementary Figure 43. Forest plot showing the observed outcomes and the estimate of the random-effects model for interventions with community engagement as the intervention on immunisation attitudes.

Immunisation attitudes

Only two studies reporting on immunisation attitudes used interventions with community engagement as the intervention. The estimated average outcome based on the random-effects model was $\hat{\mu} = 0.47$ ([95% CI: -0.19 to 1.13], $z = 1.40$, $p = 0.16$), indicating no significant difference between the intervention group and the control group (see Supplementary Figure 44). Given the small number of studies, this result should be interpreted with caution. According to the Q-test, the true outcomes appear to be heterogeneous ($Q(1) = 27.51$, $p < 0.001$, $\hat{\tau}^2 = 0.22$, $I^2 = 96.36\%$; With only two studies and no heterogeneity, moderator and leave-one-out analyses were not appropriate and tests of publication bias are not valid.

For peer review only - http://bmjopen.bmj.com/site/about/guidelines.xhtml

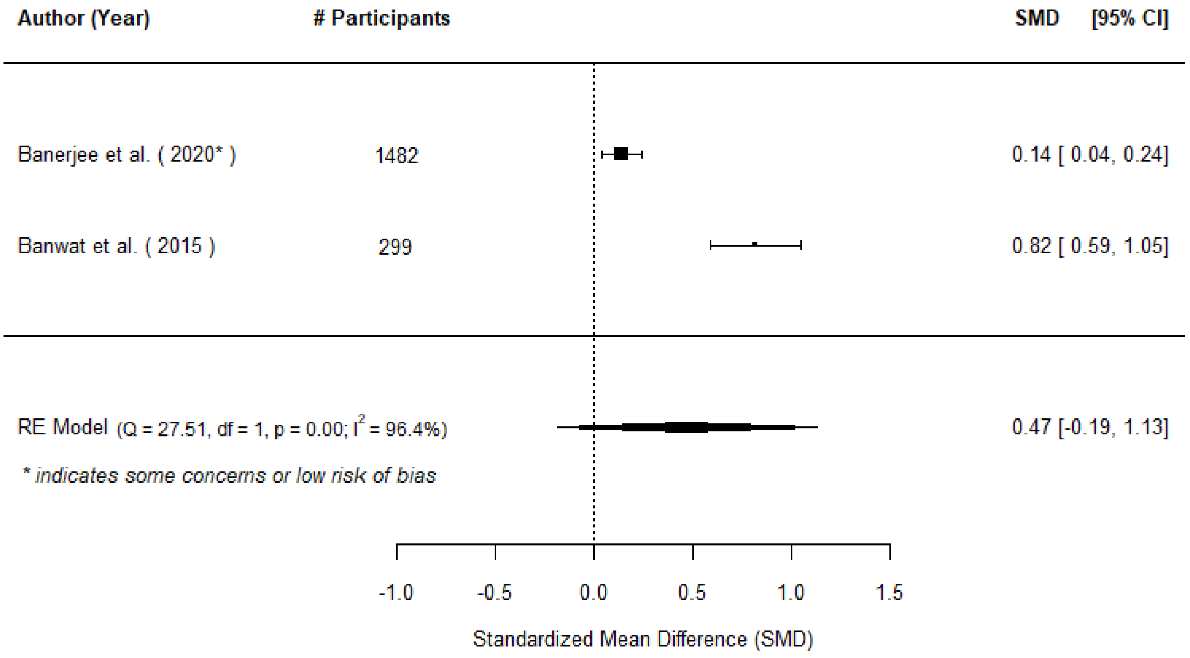

Supplementary Figure 44. Forest plot showing the observed outcomes and the estimate of the random-effects model for interventions with community engagement as the intervention on immunisation attitudes.

Vaccination card retention

Only one study (Pramanik 2020) fell into this intervention/outcome category, thus we were unable to perform a statistical synthesis. This cluster RCT from India found a null effect of their programme on vaccination card retention/availability (*g* = -0.07 [95% CI: -0.17 to 0.04]), but like most studies, it was assessed as having a high risk of bias.

Experience and satisfaction with health services

Only one study (Mohanan et al. 2020) fell into this intervention/outcome category, thus we were unable to perform a statistical synthesis. This cluster RCT from India did not find a significant effect of their programme on satisfaction with health services (*g* = -0.02 [95% CI: -0.08 to 0.04]), but like most studies, it was assessed as having a high risk of bias.

Formal health worker's motivation, capacity and performance

Only one study (Lee 2015) fell into this intervention/outcome category, thus we were unable to perform a statistical synthesis. This cluster RCT from Zambia did not find a significant effect of their programme on formal health worker's motivation, capacity, and performance (*g* = 0.09 [95% CI: -0.13 to 0.31]). This study was assessed as was assessed as having some concerns related to risk of bias.

For peer review only - http://bmjopen.bmj.com/site/about/guidelines.xhtml

Appendix 11: Quantitative results - community engagement in intervention design

Appendix 11 presents supplementary data for analyses presented in the main body of the manuscript for the primary outcomes of community engagement interventions using engagement in the intervention design, including funnel plots of publication bias and outlier analyses. It also includes a full presentation of the supplementary outcomes that were precluded from the main body of the manuscript due to word limitations.

Full immunisation

When studies used community engagement in the design, there was a significant effect on full childhood immunisation ($\hat{\mu} = 0.10$ [95% CI: 0.02 to 0.19], $z = 2.40$, $p = 0.02$). A 95% credibility/prediction interval for the true outcomes is given by -0.02 to 0.23. Hence, although the average outcome is estimated to be positive, in some studies the true outcome may in fact be negative.

According to the Q-test, there was no significant amount of heterogeneity in the true outcomes ($Q(4) = 5.25$, $p = 0.26$, $\hat{\tau}^2 = 0.00$, $I^2 = 23.83\%$). An examination of the studentized residuals revealed that none of the studies had a value larger than ± 2.58 and hence there was no indication of outliers in the context of this model. Leave-one-out analyses indicated that removal of Murthy et al. (2019), Borkum et al. (2015) or Rahman et al. (2008) would result in a non-significant effect estimate. According to the Cook's distances, none of the studies could be considered to be overly influential.

With no heterogeneity among effects, moderator analyses were not appropriate. When low quality studies were removed, two studies remained, and the summary effect increased ($\hat{\mu} = 0.14$ [95% CI: -0.01 to 0.29, but the effect was no longer significant, ($z = 1.89$, $p = 0.06$).

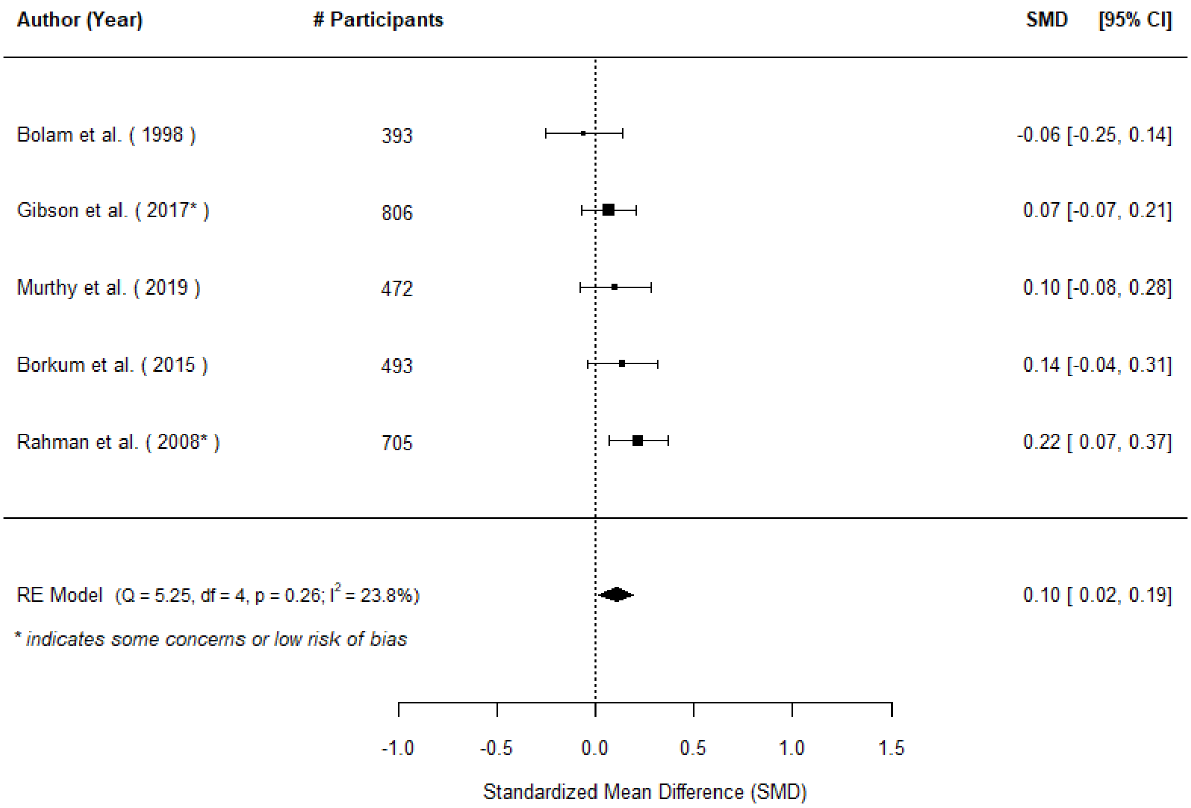

Supplementary Figure 45. Forest plot showing the observed outcomes and the estimate of the random-effects model for interventions with community engagement in the design on full immunisation

For peer review only - http://bmjopen.bmj.com/site/about/guidelines.xhtml

DPT3

When studies used community engagement in the design, there was not a significant effect on DPT3 vaccination ($\hat{\mu} = 0.04$ [95% CI: -0.01 to 0.08], $z = 1.69, p = 0.09$). According to the Q-test, there was no significant amount of heterogeneity in the true outcomes ($Q(5) = 4.36$, $p = 0.50$, $\hat{\tau}^2 = 0.00$, $I^2 = 0.00\%$). An examination of the studentized residuals revealed that none of the studies had a value larger than ± 2.64 and hence there was no indication of outliers in the context of this model. According to the Cook's distances, none of the studies could be considered to be overly influential. With no heterogeneity, we did not perform moderator or leave-one-out analyses, or compute a prediction interval. With only one study of low or medium quality, we were also unable to complete sensitivity analyses for this body of evidence.

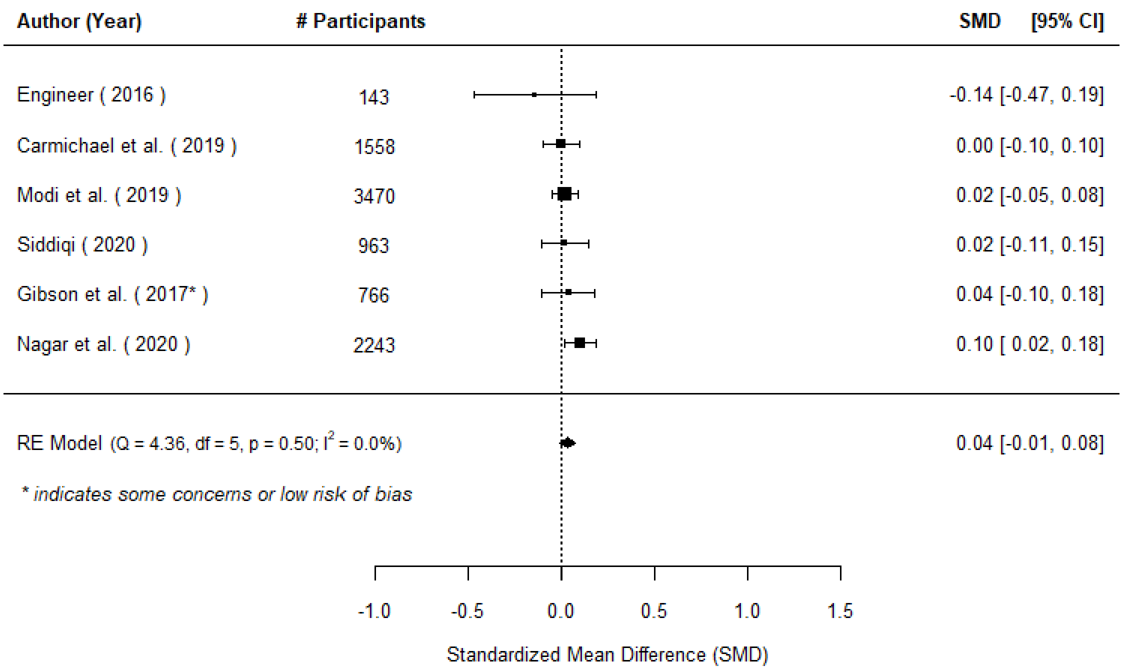

Supplementary Figure 46. Forest plot showing the observed outcomes and the estimate of the random-effects model for interventions with community engagement in the design on DPT3 vaccination

Measles

When studies used community engagement in the design, there was a significant effect on meales vaccination ($\hat{\mu} = 0.11$ [95% CI: 0.02 to 0.21], $z = 2.36$, $p = 0.02$). According to the Q-test, there was no significant amount of heterogeneity in the true outcomes ($Q(1) = 0.56$, $p = 0.46$, $\hat{\tau}^2 = 0.00$, $I^2 = 0.00\%$). With only two studies and no heterogeneity among effects, moderator and leave-one-out analyses were not appropriate.

For peer review only - http://bmjopen.bmj.com/site/about/guidelines.xhtml

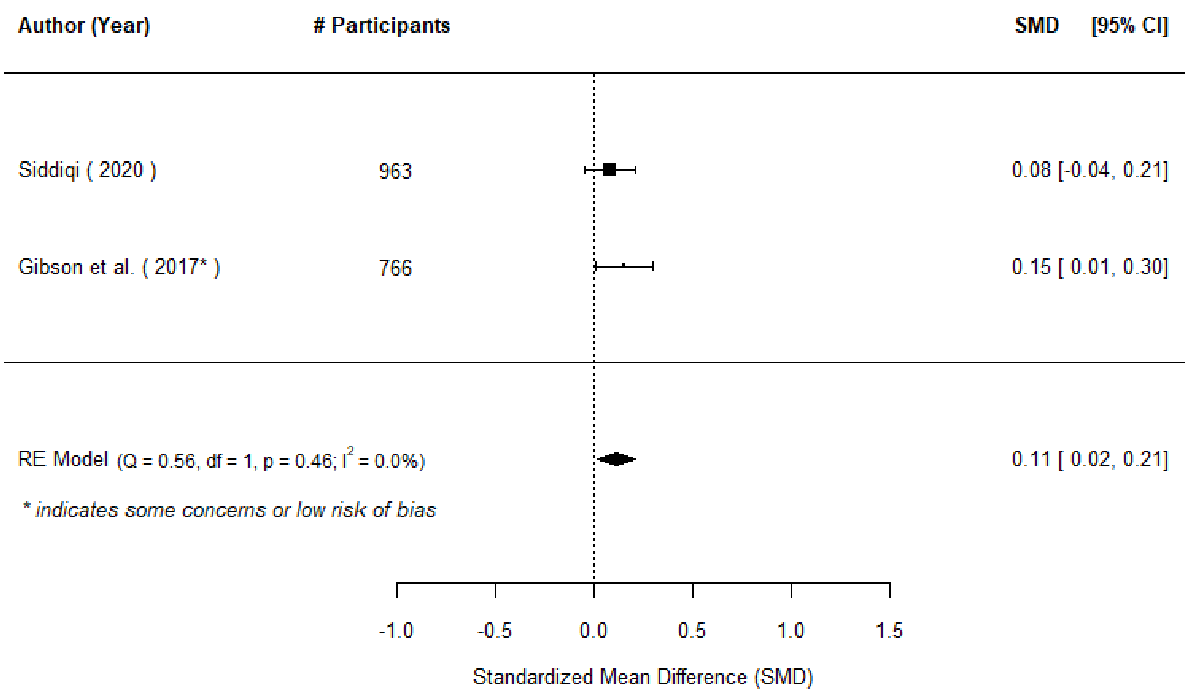

Supplementary Figure 47. Forest plot showing the observed outcomes and the estimate of the random-effects model for interventions with community engagement in the design on measles vaccination

Timeliness of full childhood immunisation

When studies used community engagement in the design, there was also a significant effect on timeliness full childhood immunisation $\hat{\mu} = 0.15$ ([95% CI: 0.004 to 0.29], $z = 2.01$, $p = 0.04$). According to the Q-test, there was no significant amount of heterogeneity in the true outcomes ($Q(1) = 0.81$, $p = 0.37$, $\hat{\tau}^2 = 0.00$, $I^2 = 0.00\%$). Both studies were assessed as high risk of bias. With only two studies and no heterogeneity, moderator and leave-one-out analyses were not appropriate.

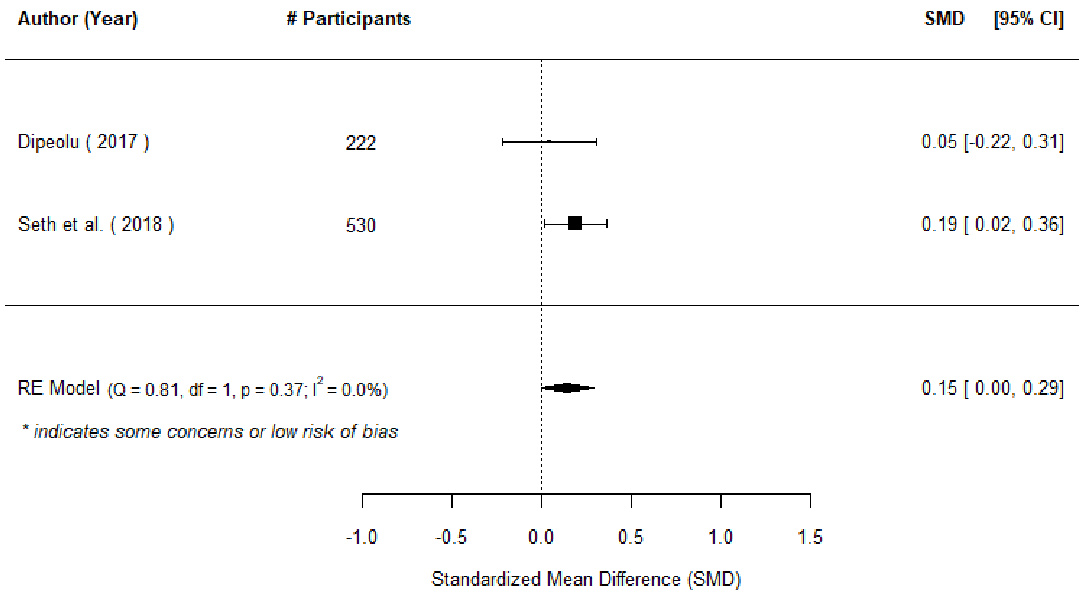

For peer review only - http://bmjopen.bmj.com/site/about/guidelines.xhtml

Supplementary Figure 48. Forest plot showing the observed outcomes and the estimate of the random-effects model for interventions with community engagement in the design on the timeliness of full childhood immunisation

Timeliness of DPT3 vaccination

When studies used community engagement in the design, there was also a significant effect on the timeliness of DPT3 vaccinations ($\hat{\mu} = 0.12$ [95% CI: 0.03 to 0.21], $z = 2.73$, $p < 0.01$). According to the Q-test, there was no significant amount of heterogeneity in the true outcomes ($Q(3) = 2.62$, $p = 0.45$, $\hat{\tau}^2 = 0.00$, $I^2 = 0.00\%$). An examination of the studentized residuals revealed that none of the studies had a value larger than ± 2.50 and hence there was no indication of outliers in the context of this model. A leave-one-out analysis indicated that removing Borkum et al. (2014) or Gibson et al. (2017) would result in a non-significant average estimated effect. According to the Cook's distances, none of the studies could be considered to be overly influential. With no heterogeneity indicated, we did not test for moderation.

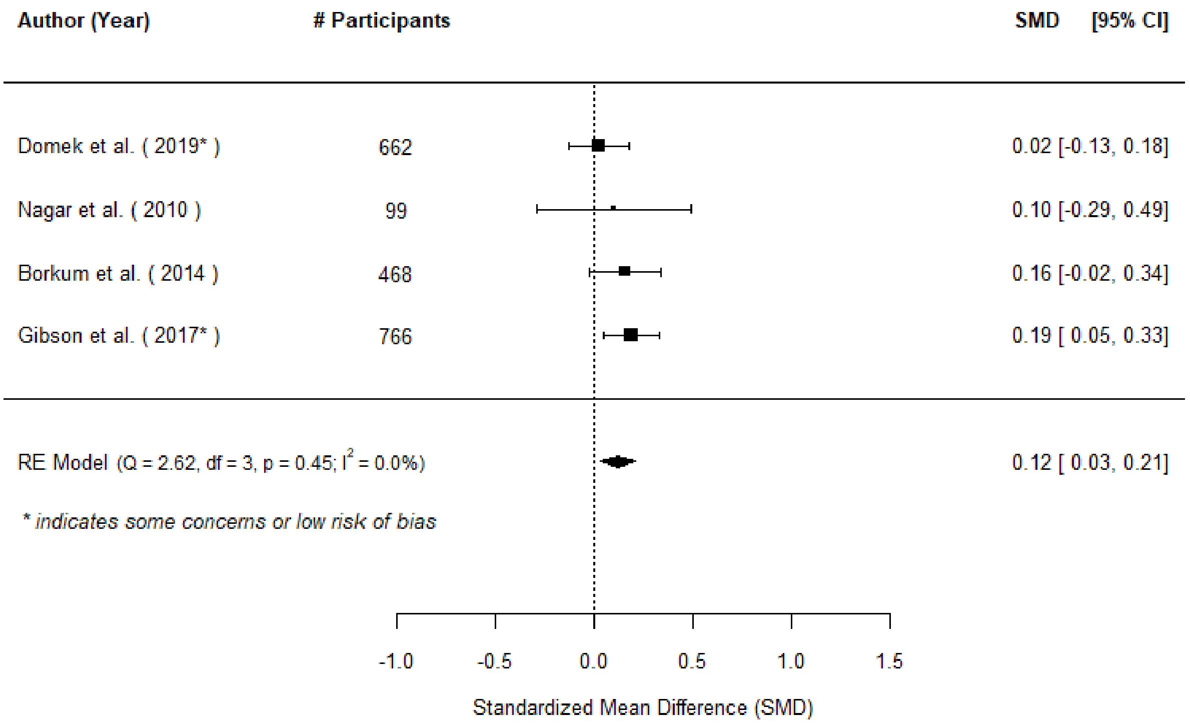

Supplementary Figure 49. Forest plot showing the observed outcomes and the estimate of the random-effects model for interventions with community engagement in the design of the intervention on the timeliness of DPT3 vaccinations.

Timeliness of measles vaccination

Only one study reporting on timeliness of measles vaccination used an intervention with engagement in the design (Gibson et al. 2017). This RCT conducted in Kenya found a small but significant positive effect (g = 0.20 [95% CI; 0.06 to 0.34]). This study was assessed as having some concerns of bias.

Secondary outcomes

Partial immunisation

Only two studies reporting on partial immunisation used interventions with community engagement in the intervention design. The estimated average outcome based on the random-effects model was $\hat{\mu} = 0.14$ ([95% CI: 0.01 to 0.27], $z = 2.05$, $p = 0.04$), indicating a small but significant benefit to the intervention group compared to the control group (see Supplementary Figure 50). Given the small number of studies, this result should be interpreted with caution. According to the Q-test, the true outcomes appear to be homogeneous ($Q(1) = 0.91$, $p = 0.34$, $\hat{\tau}^2 = 0.00$, $I^2 = 0.00\%$). With only two studies and no heterogeneity, moderator and leave-one-out analyses were not appropriate and tests of publication bias are not valid.

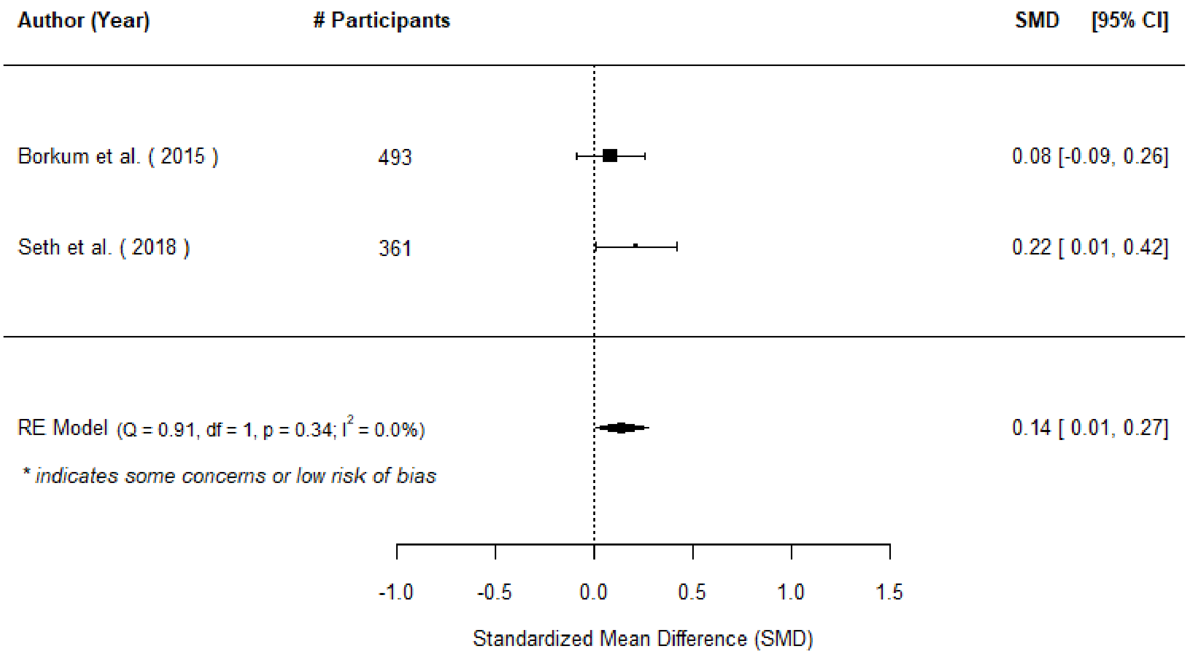

Supplementary Figure 50. Forest plot showing the observed outcomes and the estimate of the random-effects model for interventions with community engagement in the design of the intervention on partial childhood immunisation.

BCG

Only two studies reporting on BCG vaccination used interventions with community engagement in the intervention design. The estimated average outcome based on the random-effects model was $\hat{\mu} = 0.02$ ([95% CI: -0.09 to 0.13], $z = 0.32$, $p = 0.75$), indicating no difference between the intervention group and the control group (see Supplementary Figure 51). Given the small number of studies, this result should be interpreted with caution. According to the Q-test, the true outcomes appear to be homogeneous ($Q(1) = 0.11$, $p = 0.75$, $\hat{\tau}^2 = 0.00$, $I^2 = 0.00\%$). With only two studies and no heterogeneity, moderator and leave-one-out analyses were not appropriate and tests of publication bias are not valid.

For peer review only - http://bmjopen.bmj.com/site/about/guidelines.xhtml

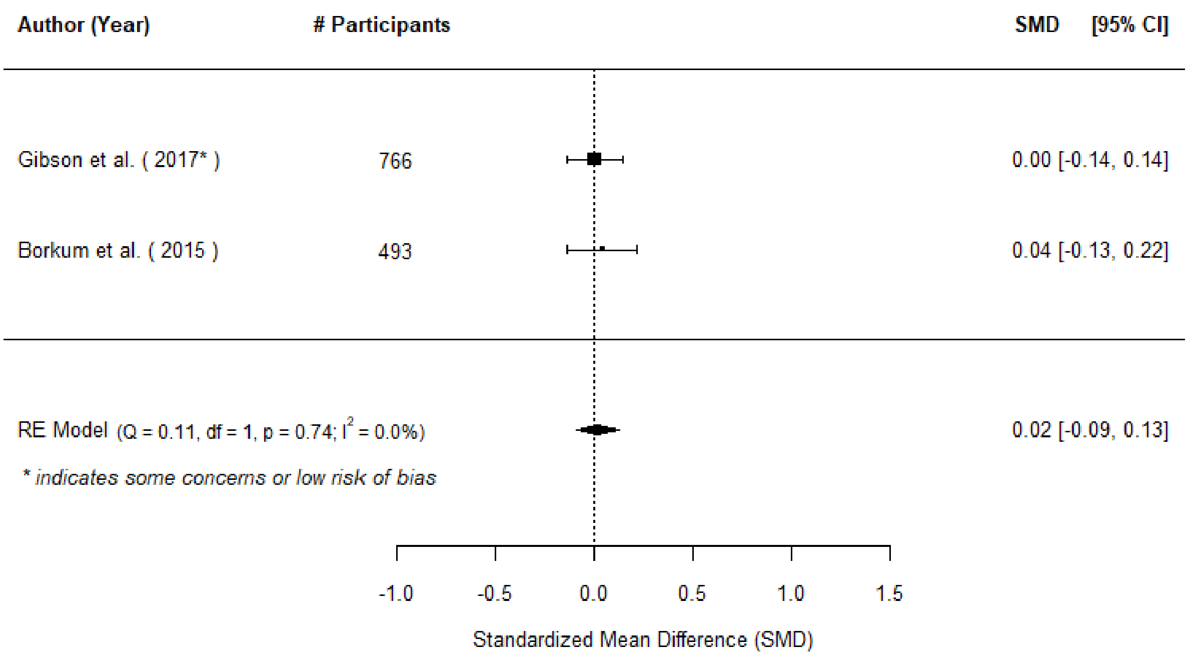

Supplementary Figure 51. Forest plot showing the observed outcomes and the estimate of the random-effects model for interventions with community engagement in the design of the intervention on BCG vaccination.

DPT1

Only two studies reporting on DPT1 vaccination used interventions with community engagement in the intervention design. The estimated average outcome based on the random-effects model was $\hat{\mu} = 0.03$ (95% CI: -0.08 to 0.14). Therefore, the average outcome did not differ significantly from zero ($z = 0.60$, $p = 0.55$, see Supplementary Figure 52), indicating no difference between the treatment group and the control group on DPT1 vaccination. Given the small number of studies, this result should be interpreted with caution. According to the Q-test, the true outcomes appear to be homogeneous ($\mathrm{Q}(1) = 0.48$, $\mathrm{p} = 0.49$, $\hat{\tau}^2 = 0.00$, $\mathrm{I}^2 = 0.00\%$). With only two studies and no heterogeneity, moderator and leave-one-out analyses were not appropriate and tests of publication bias are not valid.

For peer review only - http://bmjopen.bmj.com/site/about/guidelines.xhtml

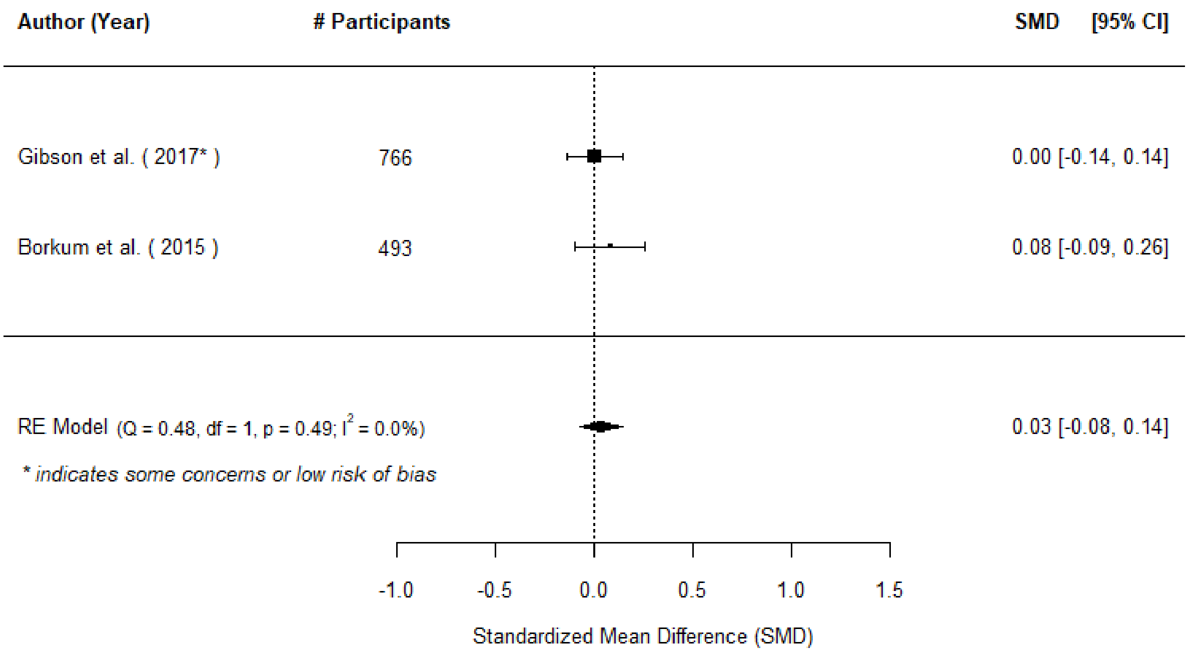

Supplementary Figure 52. Forest plot showing the observed outcomes and the estimate of the random-effects model for interventions with community engagement in the design on the intervention on DPT1 vaccination.

DPT2

Only two studies reporting on DPT2 vaccination used interventions with community engagement in the intervention design. The estimated average outcome based on the random-effects model was $\hat{\mu} = 0.05$ (95% CI: -0.06 to 0.16). Therefore, the average outcome did not differ significantly from zero ($z = 0.83$, $p = 0.41$, see Supplementary Figure 53), indicating no difference between the treatment group and the control group on DPT2 vaccination. Given the small number of studies, this result should be interpreted with caution. According to the Q-test, the true outcomes appear to be homogeneous ($Q(1) = 0.07$, $p = 0.41$, $\hat{\tau}^2 = 0.00$, $I^2 = 0.00\%$). With only two studies and no heterogeneity, moderator and leave-one-out analyses were not appropriate and tests of publication bias are not valid.

For peer review only - http://bmjopen.bmj.com/site/about/guidelines.xhtml

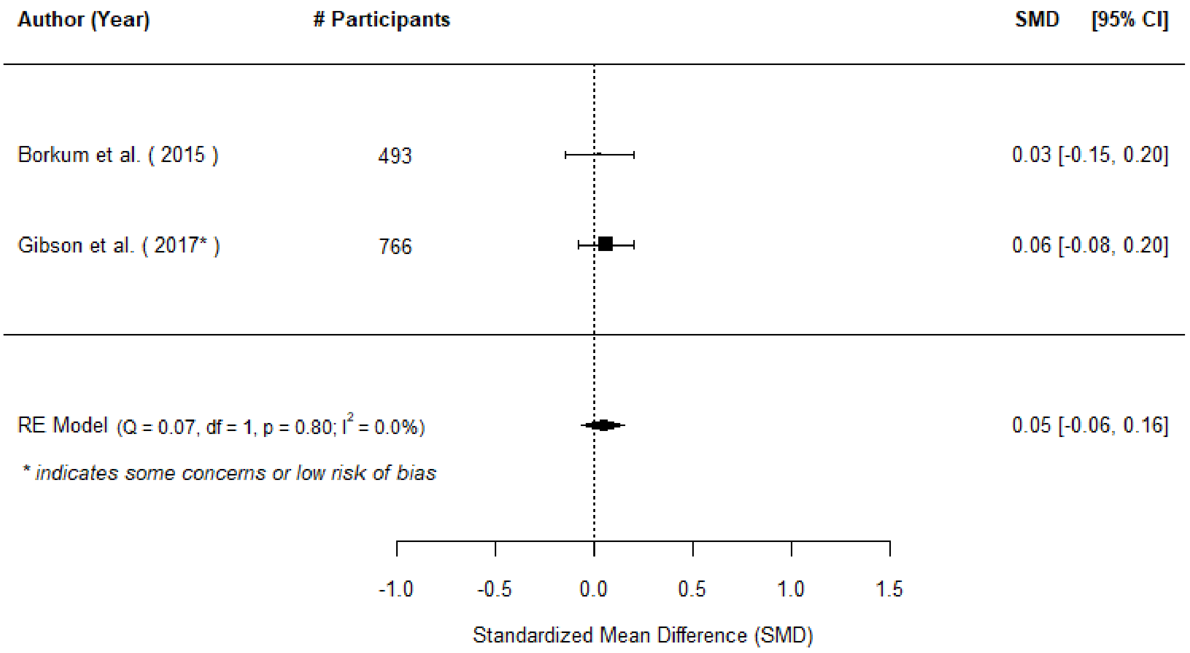

Supplementary Figure 53. Forest plot showing the observed outcomes and the estimate of the random-effects model for interventions with community engagement in the design on the intervention on DPT2 vaccination.

OPV0

Only two studies reporting on immunisation attitudes used interventions with community engagement in the intervention design. The estimated average outcome based on the random-effects model was $\hat{\mu} = 0.01$ (95% CI: -0.13 to 0.14). Therefore, the average outcome did not differ significantly from zero ($z = 0.08$, $p = 0.94$, see Supplementray Figure 54). Given the small number of studies, this result should be interpreted with caution. According to the Q-test, the true outcomes appear to be heterogeneous ($\mathrm{Q}(1) = 0.01$, $\mathrm{p} = 0.93$, $\hat{\tau}^2 = 0.00$, $\mathrm{I}^2 = 0.00\%$). With only two studies and no heterogeneity, moderator and leave-one-out analyses were not appropriate and tests of publication bias are not valid.

For peer review only - http://bmjopen.bmj.com/site/about/guidelines.xhtml

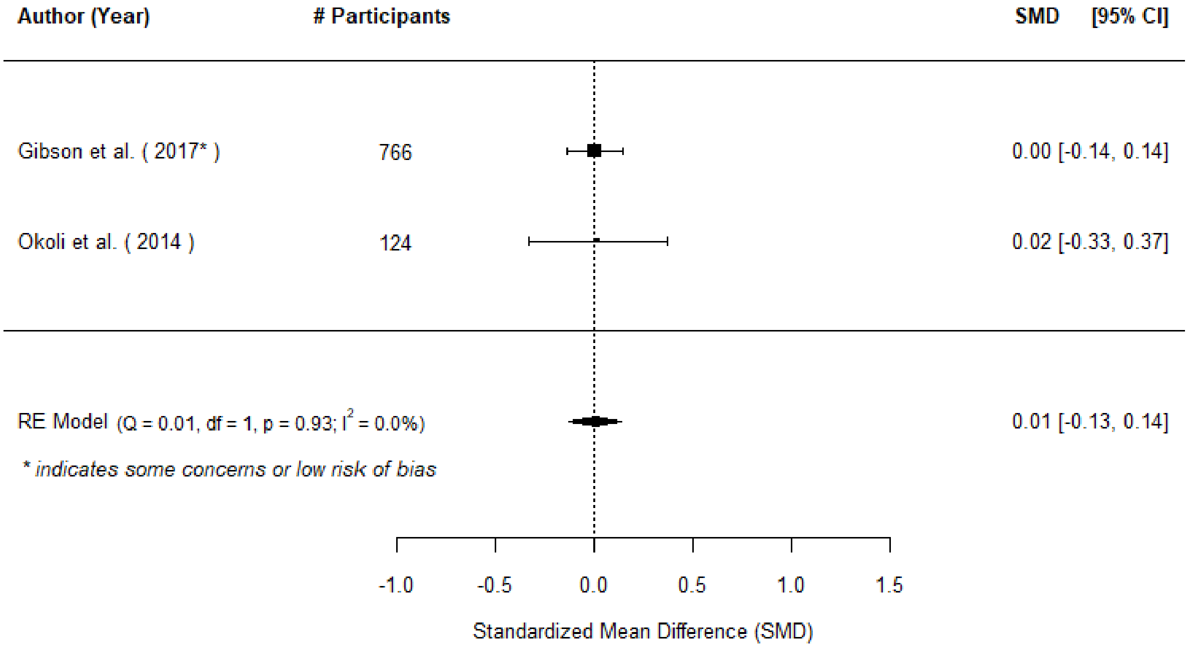

Supplementary Figure 54. Forest plot showing the observed outcomes and the estimate of the random-effects model for interventions with community engagement in the design on the intervention on OPV0 vaccination.

OPV1

Only two studies reporting on OPV1 vaccination used interventions with community engagement in the intervention design. The estimated average outcome based on the random-effects model was $\hat{\mu} =\ 0.08$ (95% CI: -0.03 to 0.19). Therefore, the average outcome did not differ significantly from zero ($z = 1.44, p = 0.15$, see Supplementary Figure 55). Given the small number of studies, this result should be interpreted with caution. According to the Q-test, the true outcomes appear to be homogeneous ($\mathrm{Q}(1) = 0.20, p = 0.65,\ \hat{\tau}^2 = 0.00,\ \mathrm{I}^2 = 0.00\%$). With only two studies, moderator and leave-one-out analyses were not appropriate and tests of publication bias are not valid.

For peer review only - http://bmjopen.bmj.com/site/about/guidelines.xhtml

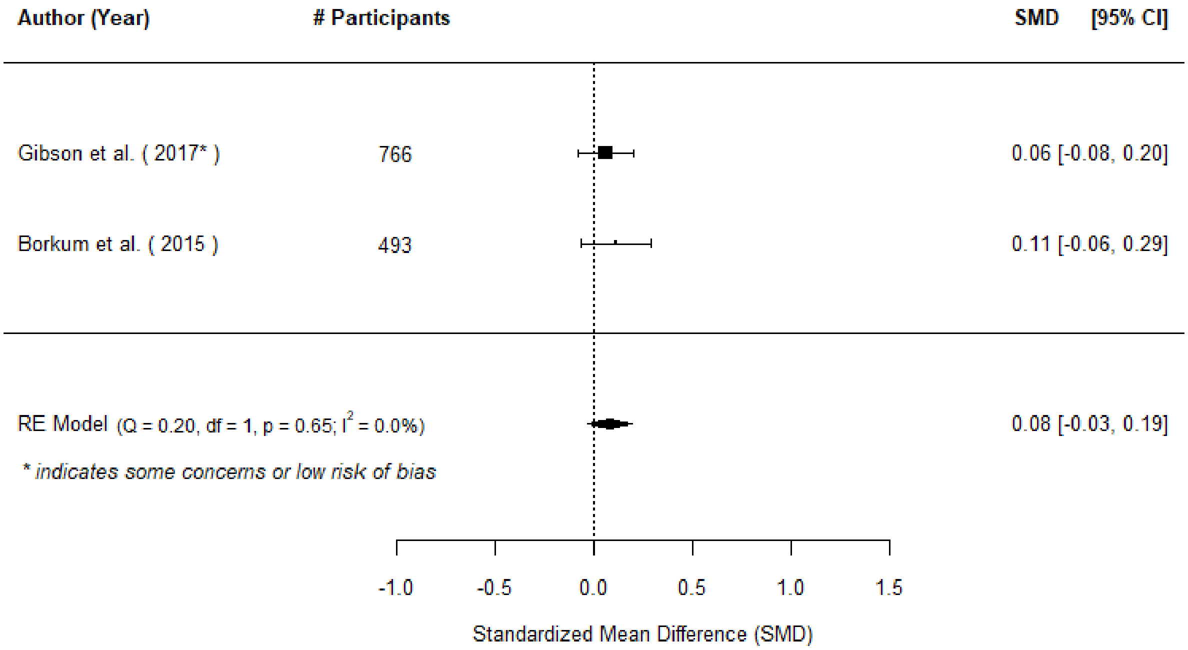

Supplementary Figure 55. Forest plot showing the observed outcomes and the estimate of the random-effects model for interventions with community engagement in the design on the intervention on OPV1 vaccination.

OPV2

Only two studies reporting on OPV2 vaccination used interventions with community engagement in the intervention design. The estimated average outcome based on the random-effects model was $\hat{\mu} = 0.23$ (95% CI: -0.09 to 0.55). Therefore, the average outcome did not differ significantly from zero ($z = 1.43$, $p = 0.15$, see Supplementray Figure 56), indicating no significant difference between the intervention group and the control group on OPV2 vaccinations. Given the small number of studies, this result should be interpreted with caution. According to the Q-test, the true outcomes appear to be heterogeneous ($Q(1) = 7.96, p = 0.004$, $\hat{\tau}^2 = 0.05$, $I^2 = 87.44\%$). With only two studies, moderator and leave-one-out analyses were not appropriate and tests of publication bias are not valid.

For peer review only - http://bmjopen.bmj.com/site/about/guidelines.xhtml

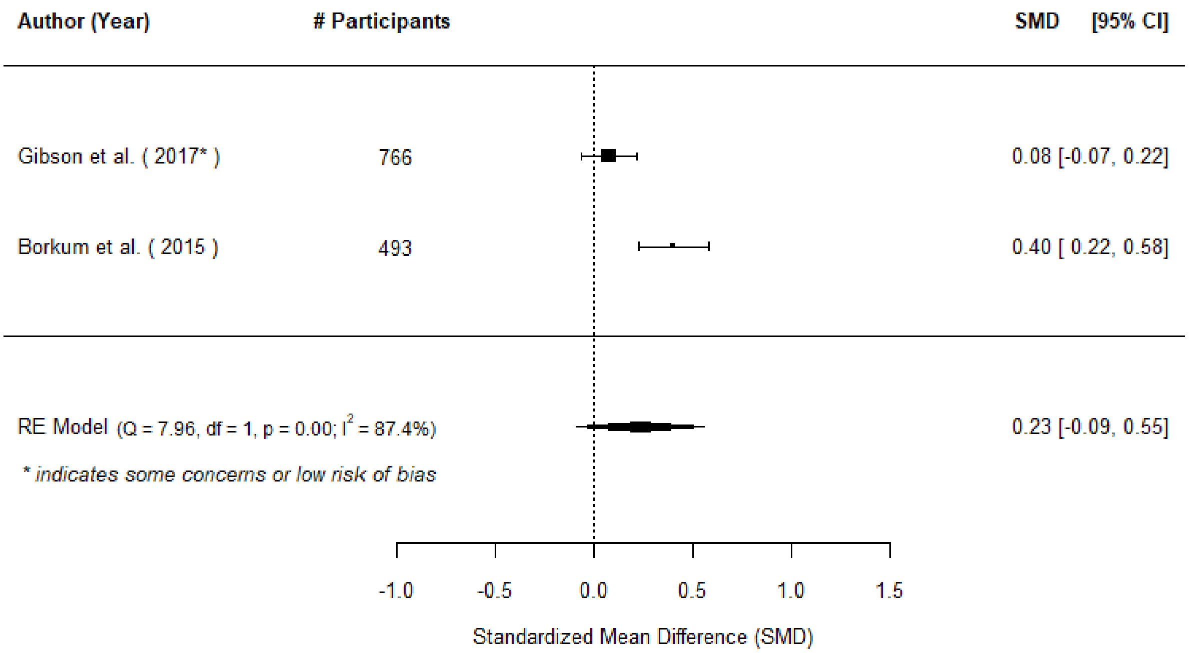

Supplementary Figure 56. Forest plot showing the observed outcomes and the estimate of the random-effects model for interventions with community engagement in the design on the intervention on OPV2 vaccination.

OPV3

Only one study (Borkum 2015) fell into this intervention/outcome category, thus we were unable to perform a statistical synthesis. This cluster RCT from India found a small but significant positive effect of their programme on OPV3 vaccination (g = 0.19 [95% CI: 0.01 to 0.36]), but again, it was assessed as having a high risk of bias.

Dropouts

There were no studies using engagement in the intervention design that reported on vaccination dropouts.

Morbidity

We used reports of diarrhea (most typically in the past two weeks) as a proxy for childhood morbidity. In all cases, effects were reverse coded such that positive effects always indicate a benefit to the treated group. Thus, a positive effect here would be interpreted as a reduction in diarrhea among treated participants compared to control participants. Only two studies reporting on childhood morbidity used interventions with community engagement in the intervention design. The estimated average outcome based on the random-effects model was $\hat{\mu} = 0.05$ (95% CI: -0.06 to 0.15). Therefore, the average outcome did not differ significantly from zero ($z = 0.88$, $p = 0.38$, see Supplementray Figure 57). Given the small number of studies, this result should be interpreted with caution. According to the Q-test, the true outcomes appear to be homogeneous ($\mathrm{Q}(1) = 1.84$, $\mathrm{p} = 0.17$, $\hat{\tau}^2 = 0.003$, $\mathrm{I}^2 = 45.71\%$). With only two studies, moderator and leave-one-out analyses were not appropriate and tests of publication bias are not valid.

For peer review only - http://bmjopen.bmj.com/site/about/guidelines.xhtml

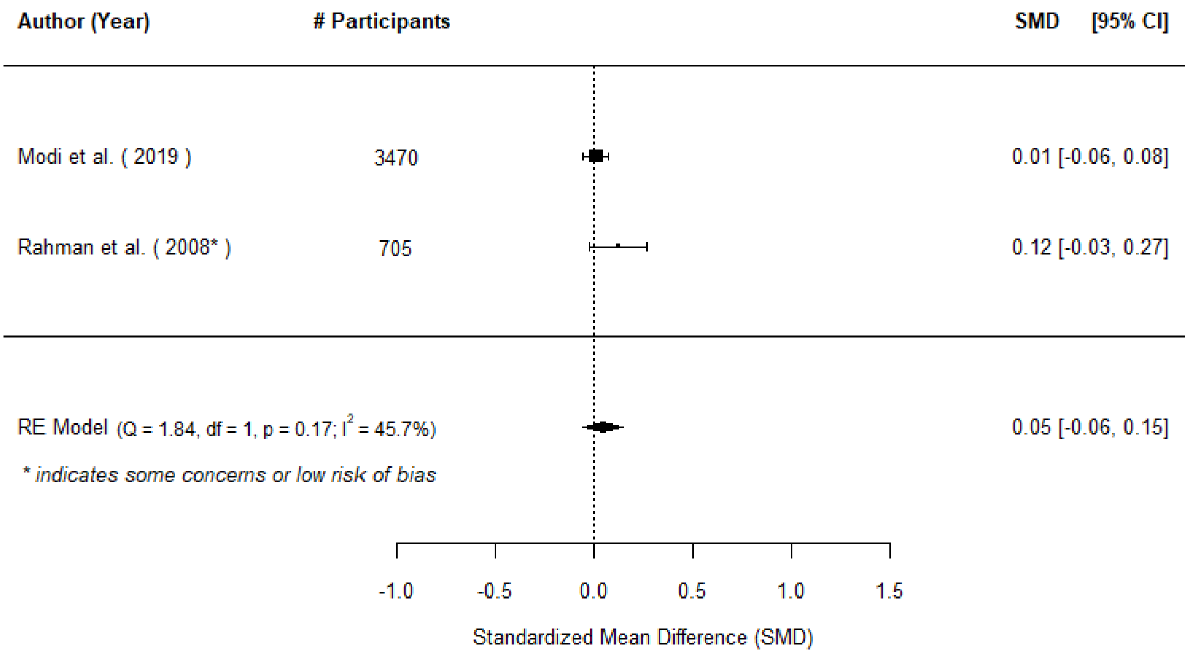

Supplementary Figure 57. Forest plot showing the observed outcomes and the estimate of the random-effects model for interventions with community engagement in the design on the intervention on childhood morbidity.

Mortality

There were no studies using engagement in the intervention design that reported on child mortality.

Immunisation knowledge

We included a total of $k = 3$ studies in the analysis. The estimated average outcome based on the random-effects model was $\hat{\mu} = 0.31$ (95% CI: 0.04 to 0.58). Therefore, the average outcome differed significantly from zero ($z = 2.26$, $p = 0.02$), indicating a moderate significant benefit to the intervention group compared to the control group (see Supplementary Figure 58). A 95% credibility/prediction interval for the true outcomes is given by -0.17 to 0.79. Hence, although the average outcome is estimated to be positive, in some studies the true outcome may in fact be negative.

According to the Q-test, the true outcomes appear to be heterogeneous ($\mathrm{Q}(2) = 7.23$, $\mathrm{p} = 0.03$, $\hat{\tau}^2 = 0.04$, $\mathrm{I}^2 = 72.34\%$). An examination of the studentized residuals revealed that one study (Murthy et al. 2019) had a value larger than ± 2.39 and may be a potential outlier in the context of this model. Indeed, sensitivity analyses leaving each study out indicated that removing Murthy and colleagues (2019) would increase the overall average effect ($\hat{\mu}$ = 0.45 (95% CI: 0.25 to 0.65), and the effect would still be positive and significant (z = 4.40, p < .001). Removal of either of the other studies results in a non-significant effect estimate. According to the Cook's distances, none of the studies could be considered to be overly influential. With only three studies, we were unable to test for sources of heterogeneity.

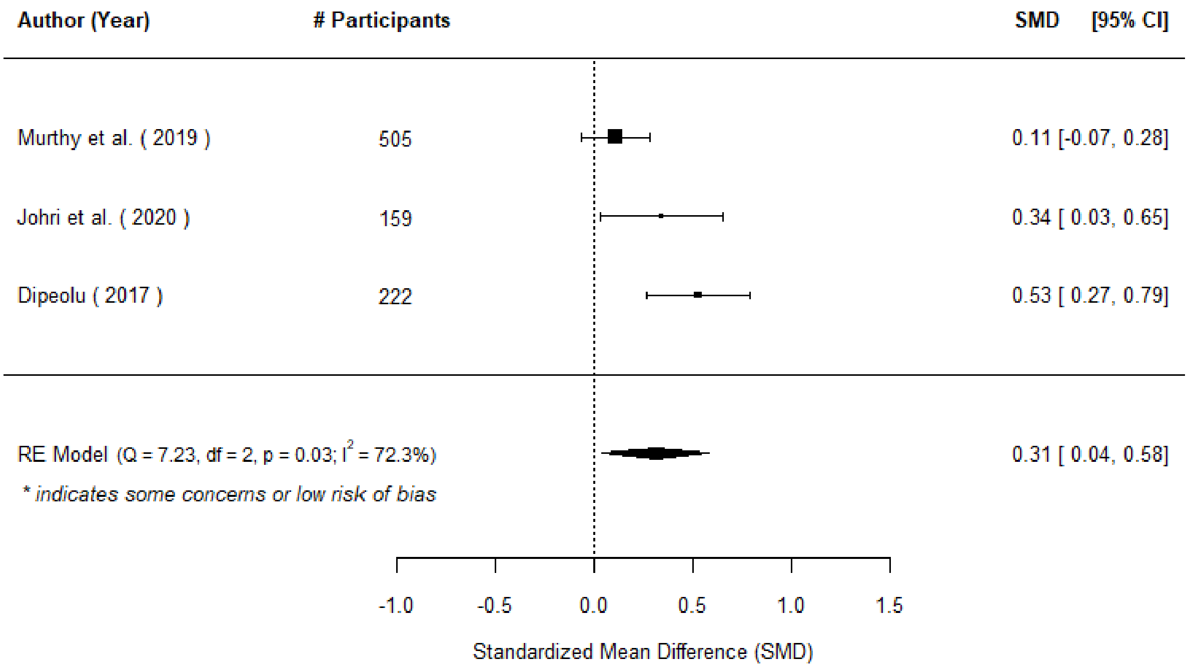

Supplementray Figure 58. Forest plot showing the observed outcomes and the estimate of the random-effects model for interventions with community engagement in the design on the intervention on childhood morbidity.

Immunisation attitudes

Only two studies reporting on immunisation attitudes used interventions with community engagement in the intervention design. The estimated average outcome based on the random-effects model was $\hat{\mu} = -0.11$ (95% CI: -0.53 to 0.30). Therefore, the average outcome did not differ significantly from zero ($z = -0.55$, $p = 0.58$, see Supplementary Figure 59). Given the small number of studies, this result should be interpreted with caution. According to the Q-test, the true outcomes appear to be heterogeneous ($\mathrm{Q}(1) = 4.10$, $\mathrm{p} = 0.04$, $\hat{\tau}^2 = 0.07$, $\mathrm{I}^2 = 75.61\%$). With only two studies, moderator and leave-one-out analyses were not appropriate and tests of publication bias are not valid.

For peer review only - http://bmjopen.bmj.com/site/about/guidelines.xhtml

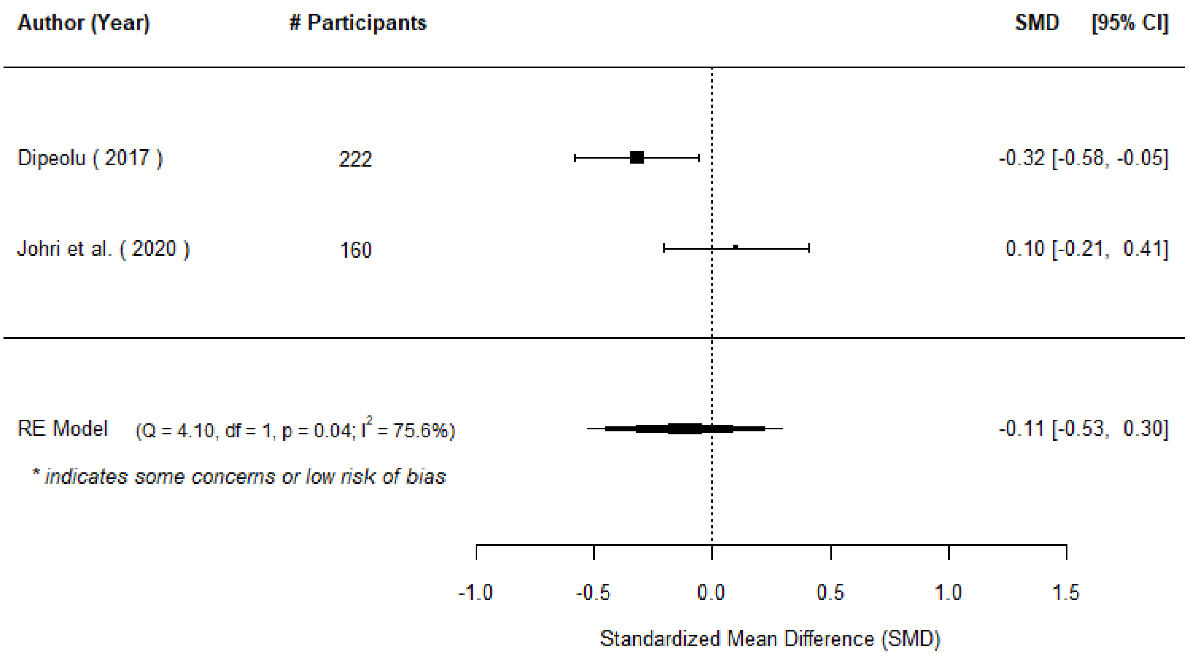

Supplementary Figure 59. Forest plot showing the observed outcomes and the estimate of the random-effects model for interventions with community engagement in the design on the intervention on immunisation attitudes.

Vaccination card retention

No studies reporting on vaccination card retention/availability used engagement in the intervention design.

Experience and satisfaction with health services

Only one study (Engineer 2016) fell into this intervention/outcome category, thus we were unable to perform a statistical synthesis. This cluster RCT from Afghanistan did not find a significant effect of their programme on satisfaction with health services (g = 0.20 [95% CI: -0.11 to 0.51]), but as with the other study reporting on this outcome, it was assessed as having a high risk of bias.

For peer review only - http://bmjopen.bmj.com/site/about/guidelines.xhtml

Appendix 12: Community engagement in implementation autonomy of interventions

Appendix 12 presents supplementary data for analyses presented in the main body of the manuscript for the primary outcomes of community engagement in implementation autonomy, including funnel plots of publication bias and outlier analyses. It also includes a full presentation of the supplementary outcomes that were precluded from the main body of the manuscript due to word limitations.

Full immunisation

Only k = 2 studies reported on the impact of interventions with community engagement in implementation autonomy on full childhood immunisation. There was no effect on full immunisation ($\hat{\mu} = 0.23$ [95% CI: -0.001 to $0.47], z = 1.95, p = 0.051$). According to the Q-test, the true outcomes appear to be homogeneous ($Q(1) = 3.71$, $p = 0.054$, $\hat{\tau}^2 = 0.02$, $I^2 = 73.07\%$, see Supplementray Figure 60). With only two studies, we could not test for moderation or publication bias, nor conduct sensitivity analysis by study quality or by leaving each study out.

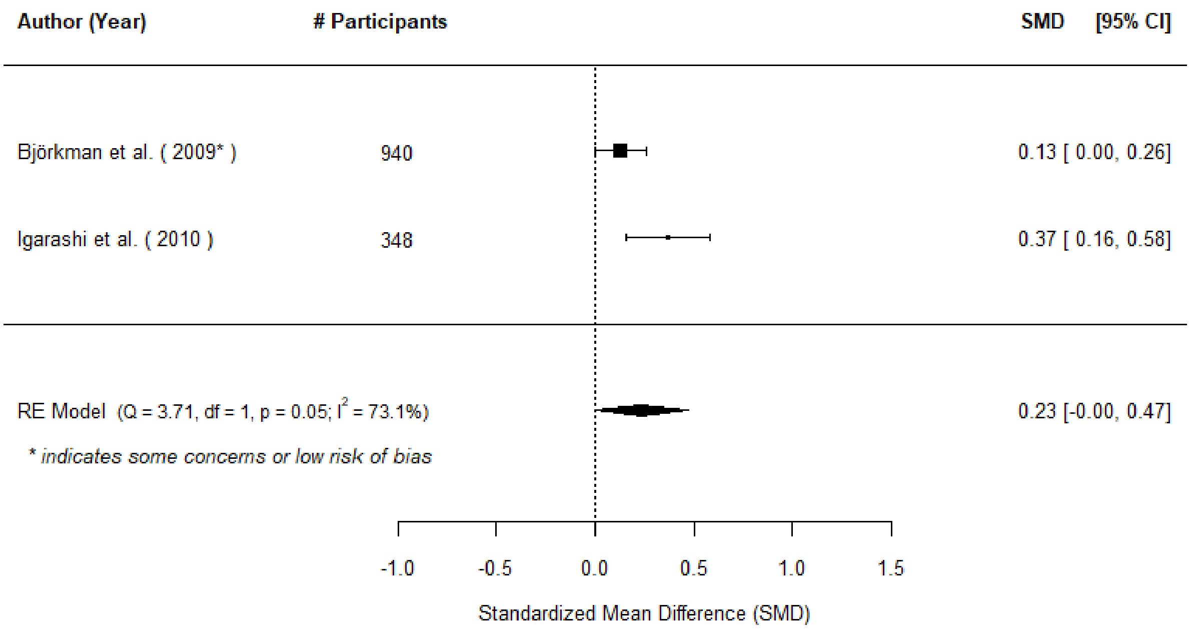

Supplementary Figure 60. Forest plot showing the observed outcomes and the estimate of the random-effects model for interventions with community engagement in implementation autonomy on full childhood immunisation.

DPT3

Only k = 3 studies examined the impact of interventions with community engagement in implementation autonomy on DPT3 vaccination. The average outcome based on the random-effects model indicated there was no effect on DPT3 vaccination ($\hat{\mu} = 0.11$ [95% CI: -0.05 to $0.28], z = 1.38, p = 0.17$). A 95% credibility/prediction interval for the true outcomes is given by -0.19 to 0.41. Hence, although the average outcome is estimated to be positive, in some studies the true outcome may in fact be negative.

According to the Q-test, the true outcomes appear to be heterogeneous ($Q(2) = 10.06$, $p < 0.01$, $\hat{\tau}^2 = 0.02$, $I^2 = 80.12\%$). An examination of the studentized residuals revealed that one study (Webster 2019) had a value larger than ± 2.39 and may be a potential outlier in the context of this model. Indeed, sensitivity analyses leaving each study out indicated that removing Webster (2019) would increase the overall average effect ($\hat{\mu}$= 0.20 [95% CI: 0.10 to 0.29]), with the effect still positive and significant (z = 3.99, p < .001). The leave-one-out analysis

For peer review only - http://bmjopen.bmj.com/site/about/guidelines.xhtml

confirmed that neither of the other studies significantly impact the average estimate when removed. According to the Cook's distances, none of the studies could be considered to be overly influential.

With only three studies, moderator analyses and tests of publication bias were not appropriate. With two of the three studies assessed as high risk of bias, we were also unable to conduct a sensitivity analysis by study quality.

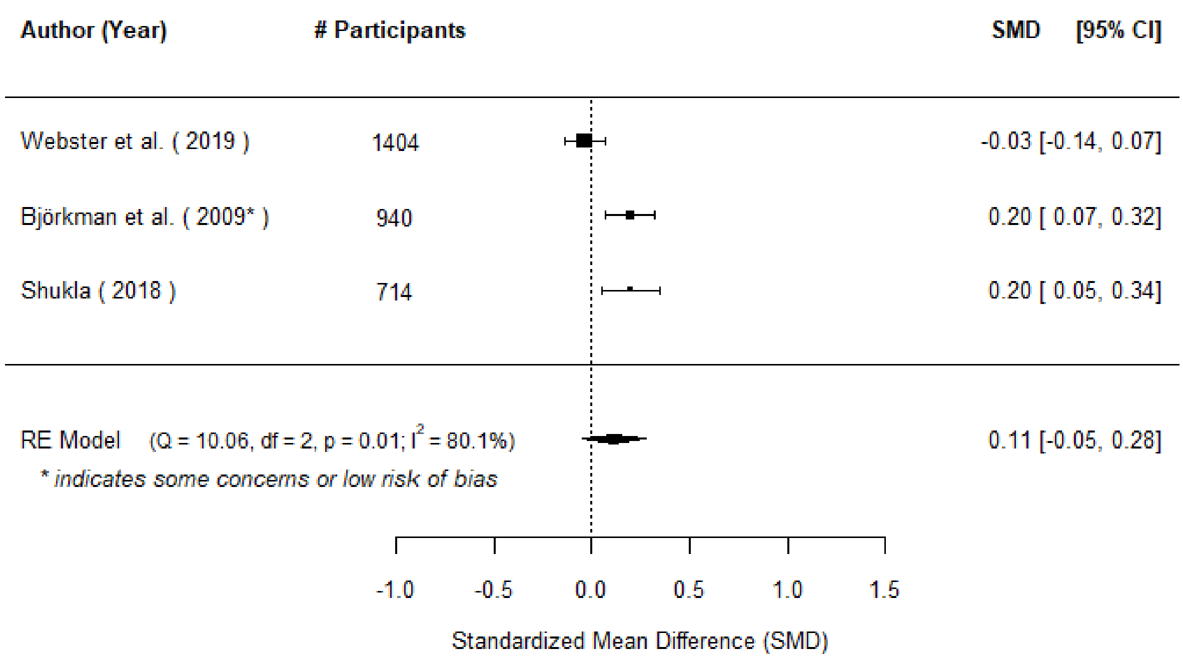

Supplementray Figure 61. Forest plot showing the observed outcomes and the estimate of the random-effects model for interventions with community engagement in implementation autonomy on DPT3 vaccination

Measles

Only $k = 2$ studies examined the impact of interventions with community engagement in implementation autonomy on measles vaccination. The average outcome based on the random-effects model indicated there was no effect on measles vaccination ($\hat{\mu} = 0.03$ [95% CI: -0.09 to 0.15], $z = 0.47$, $p = 0.64$). According to the Q-test, the true outcomes appear to be homogeneous ($Q(1) = 2.21$, $p = 0.14$, $\hat{\tau}^2 = 0.004$, $I^2 = 54.84\%$; see Supplementary Figure 62). With only two studies we were unable to test for publication bias, or complete moderator, leave-one-out, or sensitivity analyses.

For peer review only - http://bmjopen.bmj.com/site/about/guidelines.xhtml

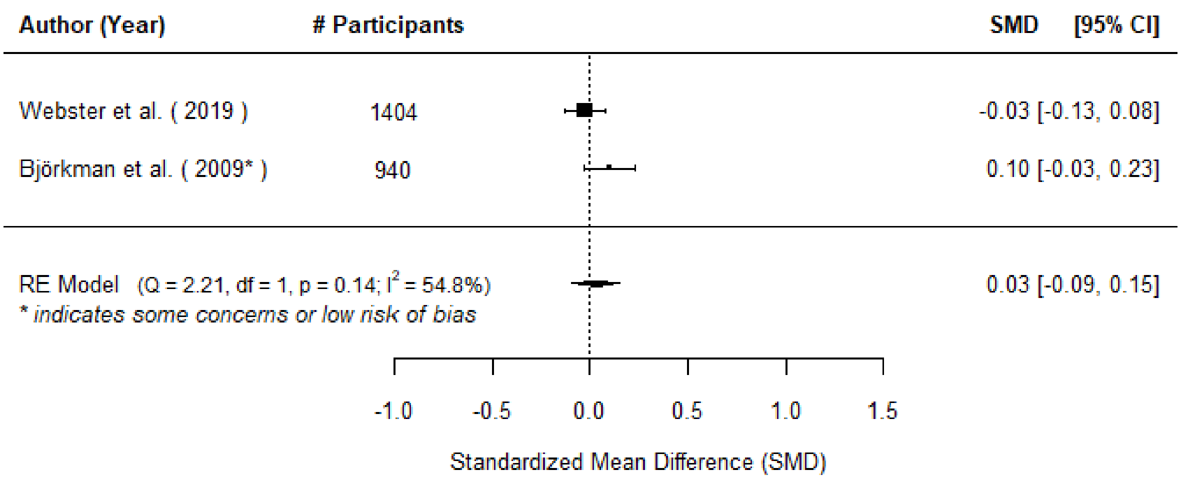

Supplementary Figure 62. Forest plot showing the observed outcomes and the estimate of the random-effects model for interventions with community engagement in implementation autonomy on measles vaccination

Timeliness of full childhood immunisation

Only k = 2 studies examined the impact of interventions with community engagement in implementation autonomy on the timeliness of full childhood immunisation. The average outcome based on the random-effects model indicated there was no effect on the timeliness of full childhood immunisation ($\hat{\mu} = 0.38$ [95% CI: -0.28 to $1.03], z = 1.13, p = 0.26$). According to the Q-test, the true outcomes appear to be homogeneous ($Q(1) = 3.57$, $p = 0.06$, $\hat{\tau}^2 = 0.17$, $I^2 = 72.02\%$). There was no indication of outliers in the context of this model, and with only two studies, we could not test for moderation or publication bias, or do a leave-one-out analysis. One study was assessed as having a high risk of bias (Igarashi et al. 2010) while the other was assessed as some concerns related to risk of bias (Lee 2015).

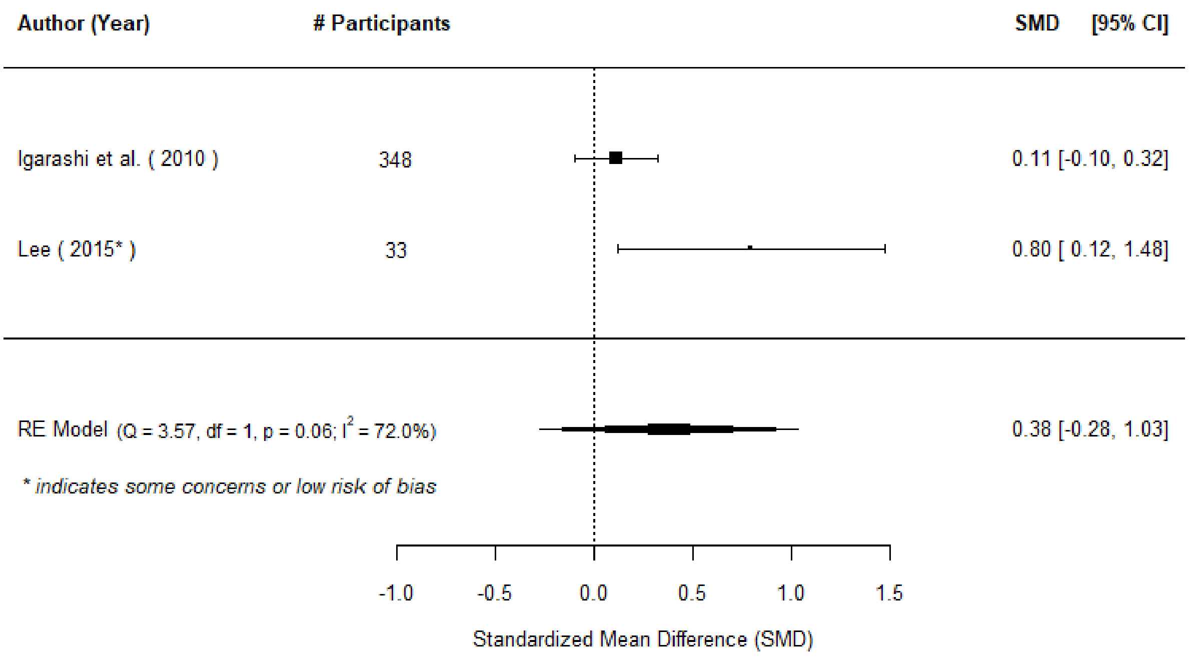

For peer review only - http://bmjopen.bmj.com/site/about/guidelines.xhtml

Supplementary Figure 63. Forest plot showing the observed outcomes and the estimate of the random-effects model for interventions with community engagement in implementation autonomy on DPT3 vaccination

DPT3 vaccination timeliness

Only k = 2 studies examined the impact of interventions with community engagement in implementation autonomy on DPT3 vaccination timeliness. The average outcome based on the random-effects model indicated there was no effect on the timeliness of DPT3 vaccination ($\hat{\mu} = 0.04$ [95% CI: -0.06 to 0.13], $z = 0.75$, $p = 0.45$). According to the Q-test, the true outcomes appear to be homogeneous ($Q(1) = 0.63$, $p = 0.45$, $\hat{\tau}^2 = 0.00$, $I^2 = 0.00\%$). There was no indication of outliers in the context of this model, and with only two studies, we could not test for moderation or publication bias, or conduct a leave-one-out analysis. Both studies were assessed as having a high risk of bias

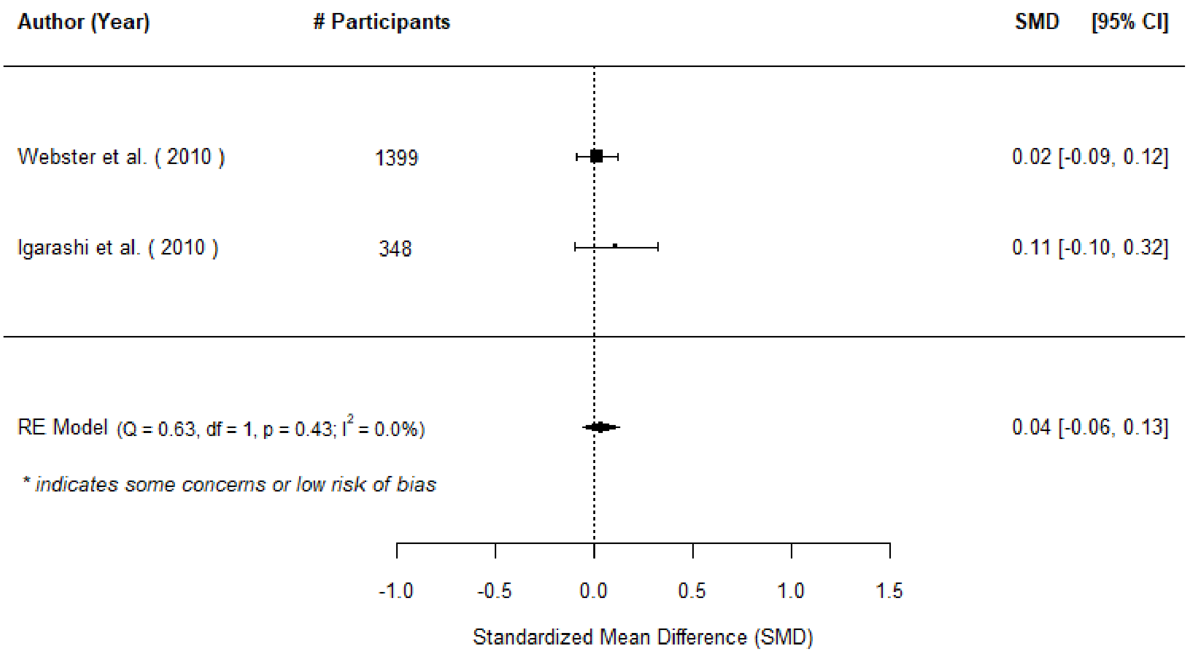

Supplementray Figure 64. Forest plot showing the observed outcomes and the estimate of the random-effects model for interventions with community engagement in implementation autonomy on DPT3 vaccination timeliness

Measles vaccination timeliness

No studies reporting on the timeliness of measles vaccinations used community engagement in implementation autonomy.

Secondary outcomes

Partial immunisation

For peer review only - http://bmjopen.bmj.com/site/about/guidelines.xhtml

Only one study (Webster et al. 2019) fell into this intervention/outcome category, thus we were unable to perform a statistical synthesis. This cluster RCT from Uganda found a null effect of their programme on partial childnood immunisation (*g* = 0.02 [95% CI: -0.08 to 0.13]), but like most studies, it was assessed as having a high risk of bias.

BCG

We included k = 2 studies in this analysis. The estimated average outcome was $\hat{\mu} = 0.03$ ([95% CI: -0.05 to 0.11], $z = 0.75$, $p = 0.46$) indicating no difference between the treated group and the untreated group (Supplementary Figure 62). According to the Q-test, the true outcomes appear to be homogeneous ($Q(1) = 0.37$, $p = 0.54$, $\hat{\tau}^2 = 0.00$, $I^2 = 00.00\%$). With only two studies and no heterogeneity, moderator and leave-one-out analyses were not appropriate and tests of publication bias are not valid.

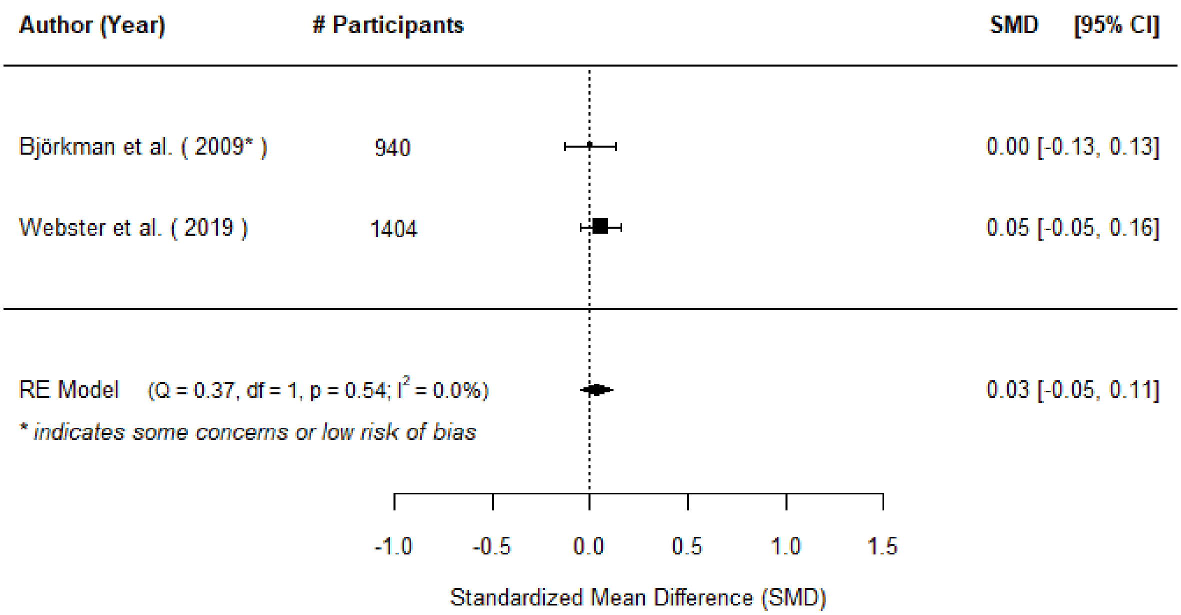

Supplementary Figure 65. Forest plot showing the observed outcomes and the estimate of the random-effects model for interventions with community engagement in implementation autonomy on BCG vaccination

DPT1

Only one study (Webster et al. 2019) fell into this intervention/outcome category, thus we were unable to perform a statistical synthesis. This cluster RCT from Uganda found a null effect of their programme on DPT1 vaccination (*g* = 0.04 [95% CI: -0.07 to 0.14]), but like most studies, it was assessed as having a high risk of bias.

DPT2

Only one study (Webster et al. 2019) fell into this intervention/outcome category, thus we were unable to perform a statistical synthesis. This cluster RCT from Uganda found a null effect of their programme on DPT1 vaccination (*g* = 0.01 [95% CI: -0.10 to 0.11]), but like most studies, it was assessed as having a high risk of bias.

For peer review only - http://bmjopen.bmj.com/site/about/guidelines.xhtml

OPV0

Only one study (Webster et al. 2019) fell into this intervention/outcome category, thus we were unable to perform a statistical synthesis. This cluster RCT from Uganda found a null effect of their programme on OPV0 vaccination (*g* = 0.01 [95% CI: -0.10 to 0.11]), but like most studies, it was assessed as having a high risk of bias.

OPV1

Only one study (Webster et al. 2019) fell into this intervention/outcome category, thus we were unable to perform a statistical synthesis. This cluster RCT from Uganda found a null effect of their programme on OPV1 vaccination (*g* = 0.03 [95% CI: -0.07 to 0.14]), but like most studies, it was assessed as having a high risk of bias.

OPV2

Only one study (Webster et al. 2019) fell into this intervention/outcome category, thus we were unable to perform a statistical synthesis. This cluster RCT from Uganda found a null effect of their programme on OPV2 vaccination (*g* = 0.04 [95% CI: -0.06 to 0.15]), but again, it was assessed as having a high risk of bias.

OPV3

Only two studies examined the impact of interventions with community engagement in implementation autonomy on OPV3 vaccination. The estimated average outcome was $\hat{\mu} = 0.03$ [95% CI: -0.12 to 0.18], which was not significant ($z = 0.35$, $p = 0.73$), indicating there was no difference between the intervention group and the control group. According to the Q-test, the true outcomes appear to be homogeneous ($Q(1) = 3.38$, $p = 0.07$, $\hat{\tau}^2 = 0.01$, $I^2 = 70.38\%$; see Supplementray Figure 66). With only two studies we were unable to test for publication bias or sources of heterogeneity or complete a leave-one-out analysis.

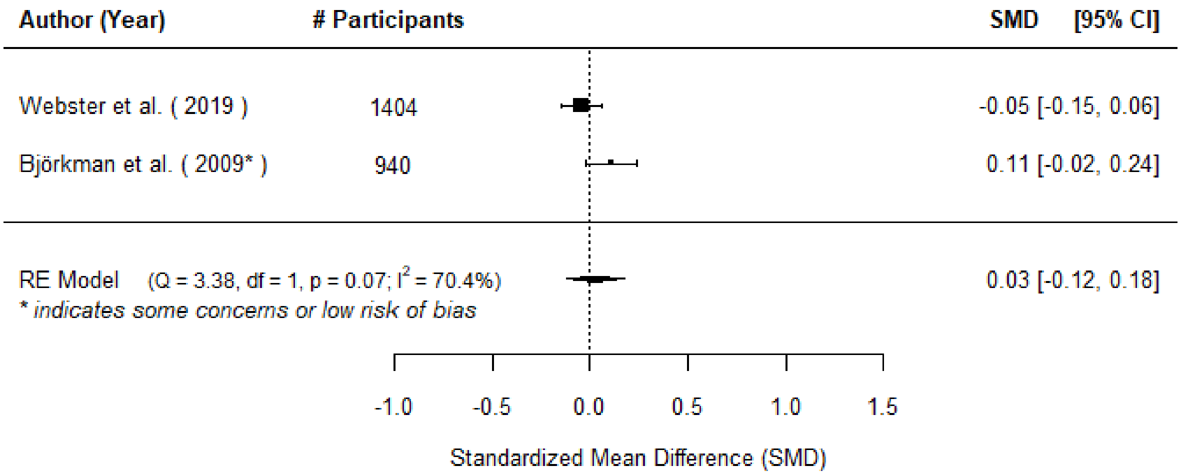

Supllementary Figure 66. Forest plot showing the observed outcomes and the estimate of the random-effects model for interventions with community engagement in implementation autonomy on OPV3 vaccinations.

For peer review only - http://bmjopen.bmj.com/site/about/guidelines.xhtml

Dropouts

Only one study (Webster et al. 2019) fell into this intervention/outcome category, thus we were unable to perform a statistical synthesis. This cluster RCT from Uganda found a small but significant negative impact of their programme on full childhood immunisation (g = -0.24 [95% CI: -0.35 to -0.14]), but like most studies, it was assessed as having a high risk of bias.

Morbidity

We used reports of diarrhea (most typically in the past two weeks) as a proxy for childhood morbidity. In all cases, effects were reverse coded such that positive effects always indicate a benefit to the treated group. Thus a positive effect here would be interpreted as a reduction in diarrhea among treated participants compared to control participants. Only one study (Webster et al. 2019) fell into this intervention/outcome category, thus we were unable to perform a statistical synthesis. This cluster RCT from Uganda found a null effect of their programme on morbidity (g = 0.06 [95% CI: -0.02 to 0.13]), but like most studies, it was assessed as having a high risk of bias.

Mortality

There were no studies using community engagement in implementation autonomy that reported on child mortality.

Immunisation knowledge

There were no studies reporting on immunisation knowledge that used community engagement in implementation autonomy.

Immunisation attitudes

There were no studies reporting on immunisation attitudes that used interventions with community engagement in implementation autonomy.

Vaccination card retention

Only one study (Webster et al. 2019) fell into this intervention/outcome category, thus we were unable to perform a statistical synthesis. This cluster RCT from Uganda found a very small positive but non-significant effect of their programme on full childhood immunisation (g = 0.04 [95% CI: -0.06 to -0.15]), but like most studies, it was assessed as having a high risk of bias.

Experience and satisfaction with health services

No studies reporting on experience and satisfaction with health services used engagement in implementation autonomy.

Formal health worker's motivation, capacity and performance

For peer review only - http://bmjopen.bmj.com/site/about/guidelines.xhtml

No studies reporting on formal health worker's motivation, capacity and performance used engagement in implementation autonomy.

For peer review only

For peer review only - http://bmjopen.bmj.com/site/about/guidelines.xhtml

Appendix 13: Multiple engagement types

Full immunisation

We included k = 9 studies examining the impact of interventions with multiple engagement types on full childhood immunisation. The average outcome based on the random-effects model indicated that there was no effect on full childhood immunisation ($\hat{\mu} = 0.22$ [95% CI: -0.12 to 0.56], $z = 1.27$, $p = 0.20$). A 95% credibility/prediction interval for the true outcomes is given by -0.81 to 1.25. Hence, although the average outcome is estimated to be positive, in some studies the true outcome may in fact be negative.

According to the Q-test, the true outcomes appear to be heterogeneous ($Q(8) = 388.56$, $p < 0.01$, $\hat{\tau}^2 = 0.25$, $I^2 = 97.94\%$, see Supplementary Figure 67). Leave-one-out analysis indicated that the removal of Banerjee et al. (2010) would reduce the average estimate to .03, but it would still be positive and non-significant. There were no high or moderate quality studies, so we were unable to perform a sensitivity analysis. None of the moderators were significant sources of heterogeneity in the context of this model, including whether the specific combination of engagement types led to different effects (see Supplementary Table 3), but for full childhood immunisation we found no differences by engagement packages ($\hat{\beta} = 0.36$, p $= 0.30$ [95% CI: -0.31 to 1.02]).

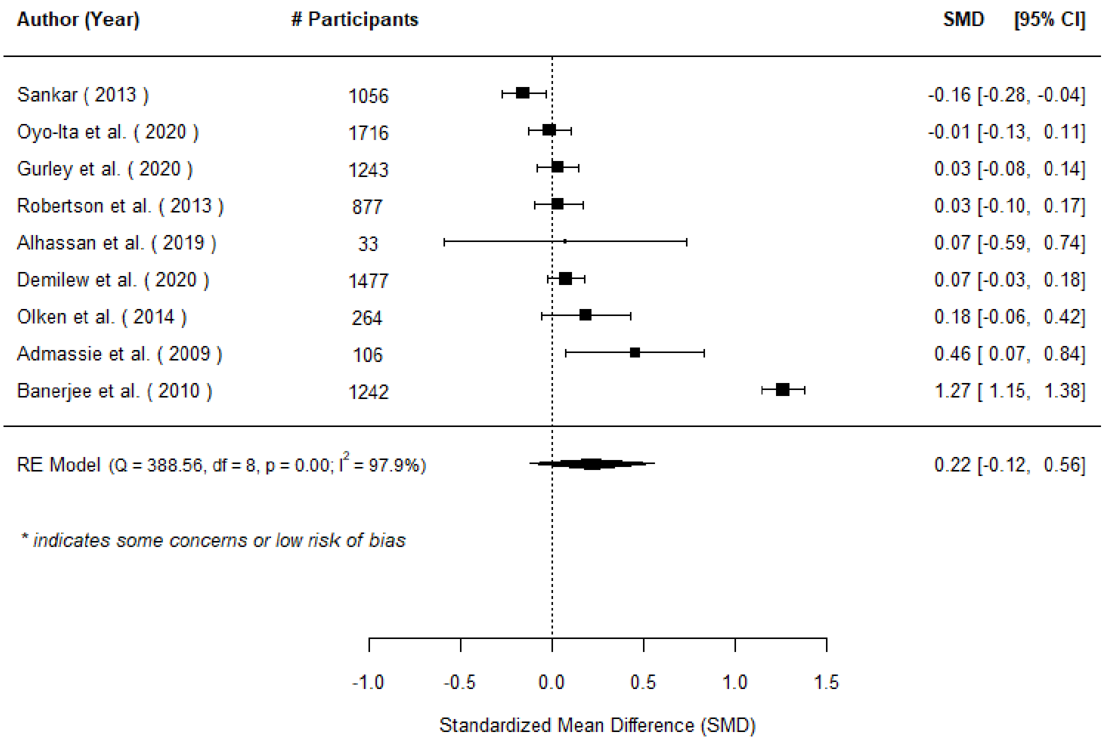

Supplementary Figure 67. Forest plot showing the observed outcomes and the estimate of the random-effects model for interventions with multiple engagement types on full immunisation

DPT3

We included k = 7 studies examining the impact of interventions with multiple engagement types on DPT3 vaccination. The average outcome based on the random-effects model indicated that there was a significant effect on DPT3 vaccination ($\hat{\mu} = 0.20$ *[95% CI:* 0.06 *to* 0.34], $z = 2.85$, $p < 0.01$). A 95% credibility/prediction interval for the true outcomes is given by -0.14 to 0.55. Hence, although the average outcome is estimated to be positive, in some studies the true outcome may in fact be negative.

For peer review only - http://bmjopen.bmj.com/site/about/guidelines.xhtml

According to the Q-test, the true outcomes appear to be heterogeneous ($Q(6) = 49.71$, $p < 0.01$, $\hat{\tau}^2 = 0.03$, $I^2 = 87.93\%$). An examination of the studentized residuals revealed that none of the studies had a value larger than ± 2.69 and hence there was no indication of outliers in the context of this model. This was confirmed with a leave-one-out analysis. According to the Cook's distances, none of the studies could be considered to be overly influential. With no high or moderate quality studies, we were unable to perform a sensitivity analysis. There were several moderators that were significant sources of heterogeneity. Evaluation period was significant such that each additional month between the end of the intervention and the collection of outcome data reduced the size of the effect by .01 standard deviation units ($\hat{\beta} = -0.01, p = 0.003$ [95% CI: -0.02 to -0.004]), suggesting smaller long term effect of the interventions. Publication year was also significant, such that each additional year reduced the size of the effect by .03 standard deviation units ($\hat{\beta} = -0.03, p = 0.03$ [95% CI: -0.05 to -0.003]). In other words, more recent studies have found smaller effects. Finally, baseline DPT3 coverage rates were significant such that a one unit increase in baseline DPT3 coverage was associated with a decrease of .56 in the effect of the programme ($\hat{\beta} = -0.56, p = 0.002$ [95% CI: -0.90 to -0.21]). In other words, the programmes were significantly more effective in areas with lower baseline coverage rates. We also tested whether the specific combination of engagement types led to different effects, but for DPT3 vaccination we found no differences by engagement packages ($\hat{\beta} = -0.07, p = 0.66$ [95% CI: -0.40 to -0.25]).

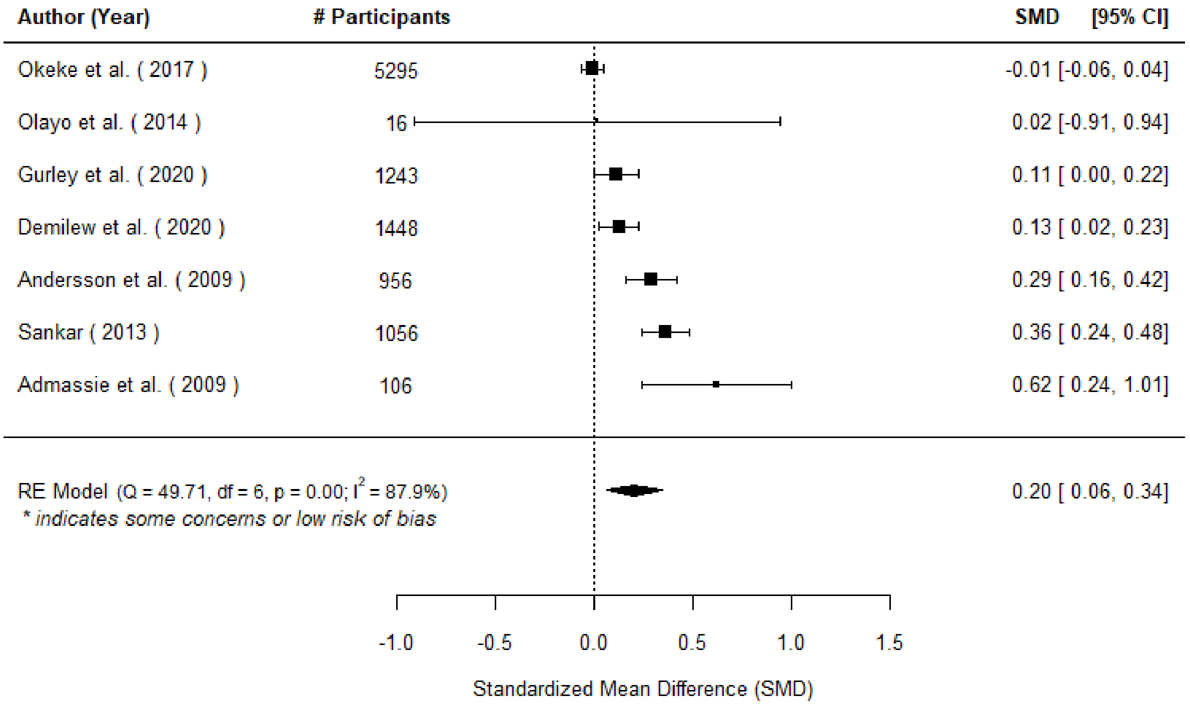

Supplementary Figure 68. Forest plot showing the observed outcomes and the estimate of the random-effects model for interventions with multiple engagement types on DPT3 vaccination

Measles

We included k = 6 studies examining the impact of interventions with multiple engagement types on measles vaccination. The average outcome based on the random-effects model indicated that there was no effect on measles coverage ($\hat{\mu} = 0.03$ [95% CI: -0.10 to 0.16], $z = 0.44$, $p = 0.66$). A 95% credibility/prediction interval for the true outcomes is given by -0.26 to 0.31. Hence, although the average outcome is estimated to be positive, in some studies the true outcome may in fact be negative.

For peer review only - http://bmjopen.bmj.com/site/about/guidelines.xhtml

According to the Q-test, the true outcomes appear to be heterogeneous ($Q(5) = 37.76$, $p < 0.01$, $\hat{\tau}^2 = 0.02$, $I^2 =$ 86.76%; see Supplementary Figure 69). An examination of the studentized residuals revealed that one study (Sankar 2013) had a value larger than ± 2.64 and may be a potential outlier in the context of this model. According to the Cook's distances, none of the studies could be considered to be overly influential. Indeed, sensitivity analyses leaving each study out indicated that removing Sankar (2013) would increase the overall average effect ($\hat{\mu} = 0.09$ (95% CI: -0.02 to 0.19), but the effect would still be non-significant ($z = 1.58$ $p = 0.11$), and that no other study's removal would significantly impact the average estimated effect. With only one high or medium quality study, we were unable to conduct sensitivity analysis by study quality. None of the moderators that could be tested were significant in the context of this model (see Supplementary Table 3). For measles vaccinations, we were unable to test whether different combinations of community engagement produced different results because all but one study used a combination of engagement in the implementation and engagement as the intervention.

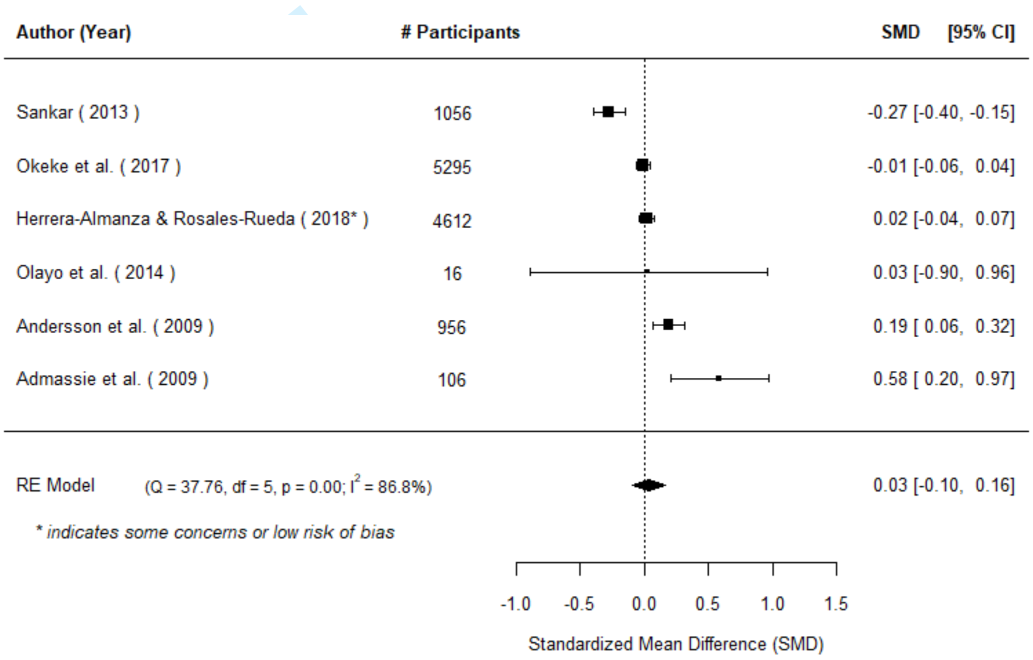

Supplementary Figure 69. Forest plot showing the observed outcomes and the estimate of the random-effects model for interventions with multiple engagement types on measles vaccination

Timeliness of full childhood immunisation

There were no studies using multiple engagement types that reported on the timeliness of full childhood immunisation.

Timeliness of DPT3 vaccination

Only one study reporting on timeliness of DPT3 vaccination used an intervention with multiple engagement types (Oyo-Ita et al. 2020). This RCT conducted in Nigeria found a small but not-significant positive effect (g = 0.10 [95%CI; -.0.2 to 0.23).This study was assessed as having a high risk of bias.

Timeliness of measles vaccination

Finally, only one study reporting on timeliness of measles vaccination used an intervention with multiple engagement types (Oyo-Ita et al. 2020). This RCT conducted in Nigeria found a moderate and significant positive

For peer review only - http://bmjopen.bmj.com/site/about/guidelines.xhtml

effect (g = 0.25 [95% CI; 0.13 to 0.36]). They used a combination of engagement in implementation autonomy and engagement as the intervention. This study was also assessed as having a high risk of bias.

Secondary outcomes

Partial immunisation

We included a total of $k = 4$ studies in the analysis. The estimated average outcome based on the random-effects model was $\hat{\mu} = 0.28$ (95% CI: -0.01 to 0.56). Therefore, the average outcome did not differ significantly from zero ($z = 1.91$, $p = 0.06$), indicating no difference between the intervention and control groups (see Supplementary Figure 70). A 95% credibility/prediction interval for the true outcomes is given by -0.35 to 0.91. Hence, although the average outcome is estimated to be positive, in some studies the true outcome may in fact be negative.

According to the Q-test, the true outcomes appear to be heterogeneous ($Q(3) = 101.75$, $p < 0.01$, $\hat{\tau}^2 = 0.08$, $I^2 = 97.05\%$). An examination of the studentized residuals revealed that none of the studies had a value larger than ± 2.50 and hence there was no indication of outliers in the context of this model. However, a leave-one-out analysis did indicate that removing Herrera-Almanza & Rosales-Rueda (2018) would result in a larger and statistically significant effect ($\hat{\mu} = 0.37$, [95% CI: -0.01 to 0.56], $p =$ .03)According to the Cook's distances, none of the studies could be considered to be overly influential. Of the moderators we were able to test, none were significant sources of heterogeneity (see Supplementary Table 3). We were unable to test for differences among engagement packages because only one study used a combination of engagement in the design and engagement as the intervention (Gurley et al. 2020) while the remaining studies used a combination of engagement in implementation autonomy and engagement as the intervention.

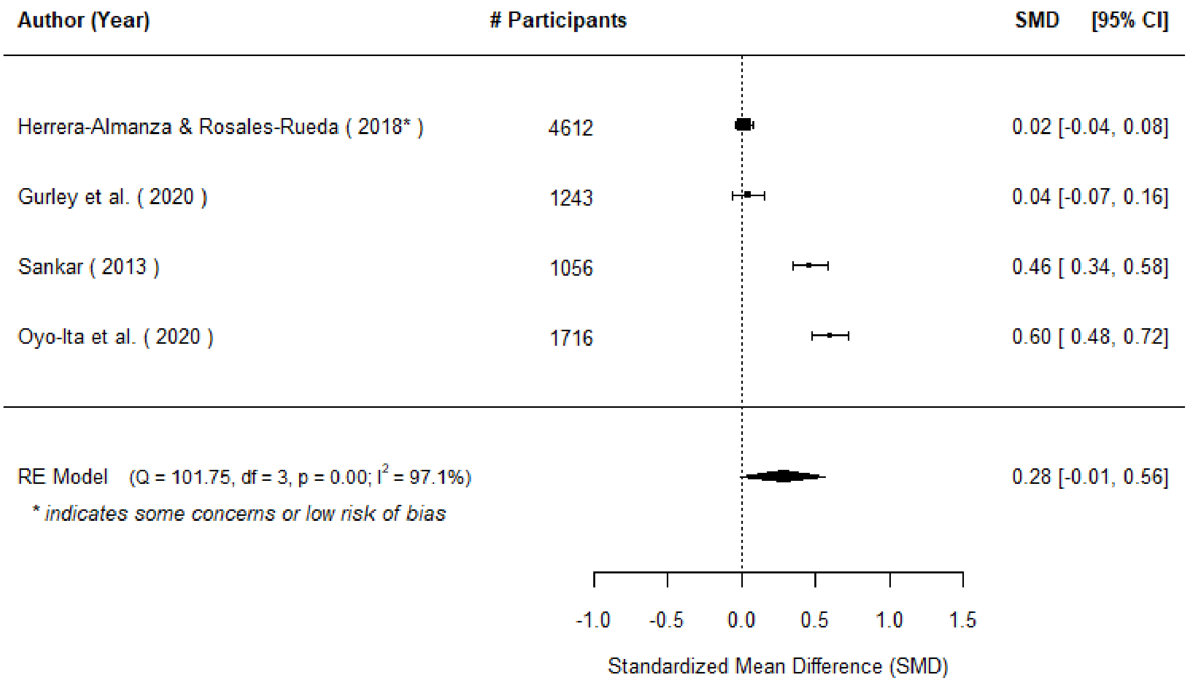

Supplementary Figure 70. Forest plot showing the observed outcomes and the estimate of the random-effects model for interventions with multiple engagement types on partial immunisation.

BCG

For peer review only - http://bmjopen.bmj.com/site/about/guidelines.xhtml

We included a total of $k = 4$ studies in the analysis. The estimated average outcome based on the random-effects model was $\hat{\mu} = 0.22$ (95% CI: -0.07 to 0.52). Therefore, the average outcome did not differ significantly from zero ($z = 1.48$, $p = 0.14$), indicating no differences between the treatment and control groups (see Supplementary Figure 71). A 95% credibility/prediction interval for the true outcomes is given by -0.41 to 0.86. Hence, although the average outcome is estimated to be positive, in some studies the true outcome may in fact be negative.

According to the Q-test, the true outcomes appear to be heterogeneous ($Q(3) = 75.36$, $p < 0.01$, $\hat{\tau}^2 = 0.08$, $I^2 =$ 96.02%). An examination of the studentized residuals revealed that one study (Banerjee 2010) had a value larger than ± 2.50 and may be a potential outlier in the context of this model. Indeed, sensitivity analyses leaving each study out indicated that removing Banerjee (2010) would reduce the overall average effect ($\hat{\mu} = 0.05$ (95% CI: -0.13 to 0.23), but the effect is still positive and non-significant ($z = 0.56$, $p = 0.58$). The leave-one-out analysis did not inticate that leaving any other studies out would significantly change the average estimated effect. According to the Cook's distances, none of the studies could be considered to be overly influential. With all studies assessed as high risk of bias, we were unable to conduct sensitivity analysis by study quality. We tested all moderators and only post-intervention versus change from baseline was a significant predictor of BCG vaccination, such that studies examining change from baseline had higher effects than studies examining post-intervention changes by .55 standard deviation units ($\hat{\beta} = -0.55$ [95% CI: -0.69 to -0.404], $p <$ .001). We could not test for moderation by engagement components because all but one study used a combination of engagement in implementation autonomy and engagement as the intervention.

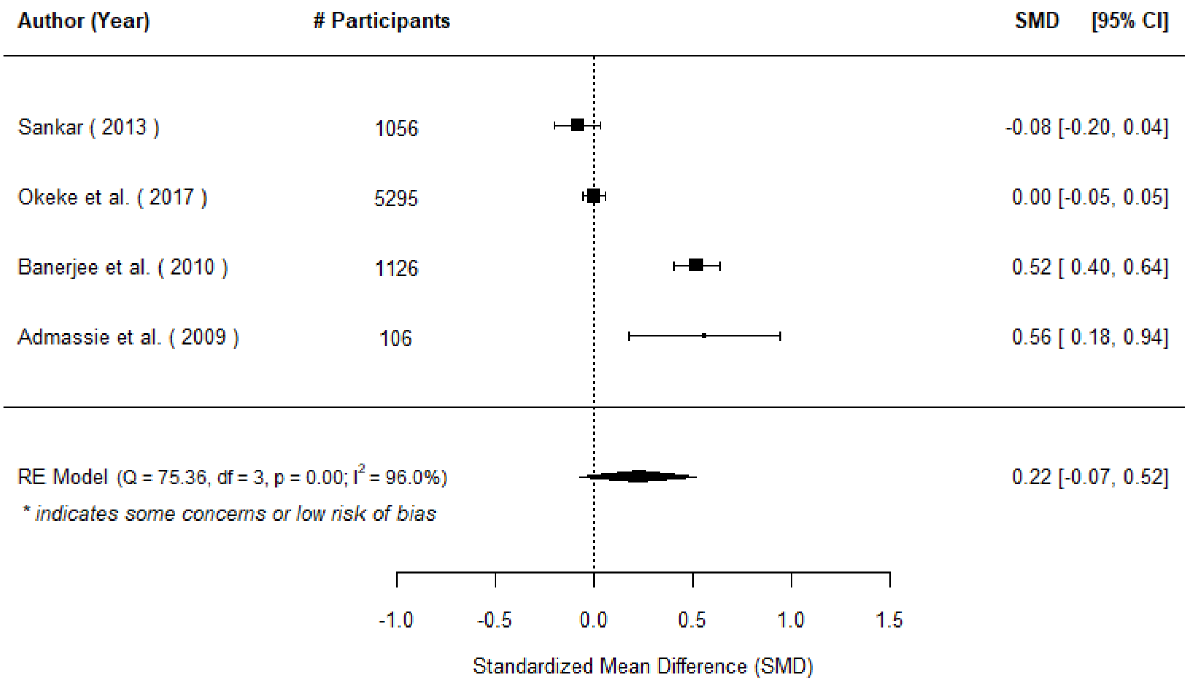

Supplemenatary Figure 71. Forest plot showing the observed outcomes and the estimate of the random-effects model for interventions with multiple engagement types on BCG vaccination.

DPT1

Only two studies reporting on DPT1 vaccination used interventions with multiple engagement types. The estimated average outcome based on the random-effects model was $\hat{\mu} = -0.17$ ([95% CI: -0.29 to -0.05], $z = -2.81$, $p =$ 0.005), thus there was a significant negative impact of the programmes on DPT1 vaccination, (see Supplementary

For peer review only - http://bmjopen.bmj.com/site/about/guidelines.xhtml

Figure 72). Given the small number of studies, this result should be interpreted with caution. According to the Q-test, the true outcomes appear to be homogeneous ($Q(1) = 0.03, p = 0.87, \hat{\tau}^2 = 0.00, I^2 = 0.00\%$). With only two studies, moderator and leave-one-out analyses were not appropriate and tests of publication bias are not valid.

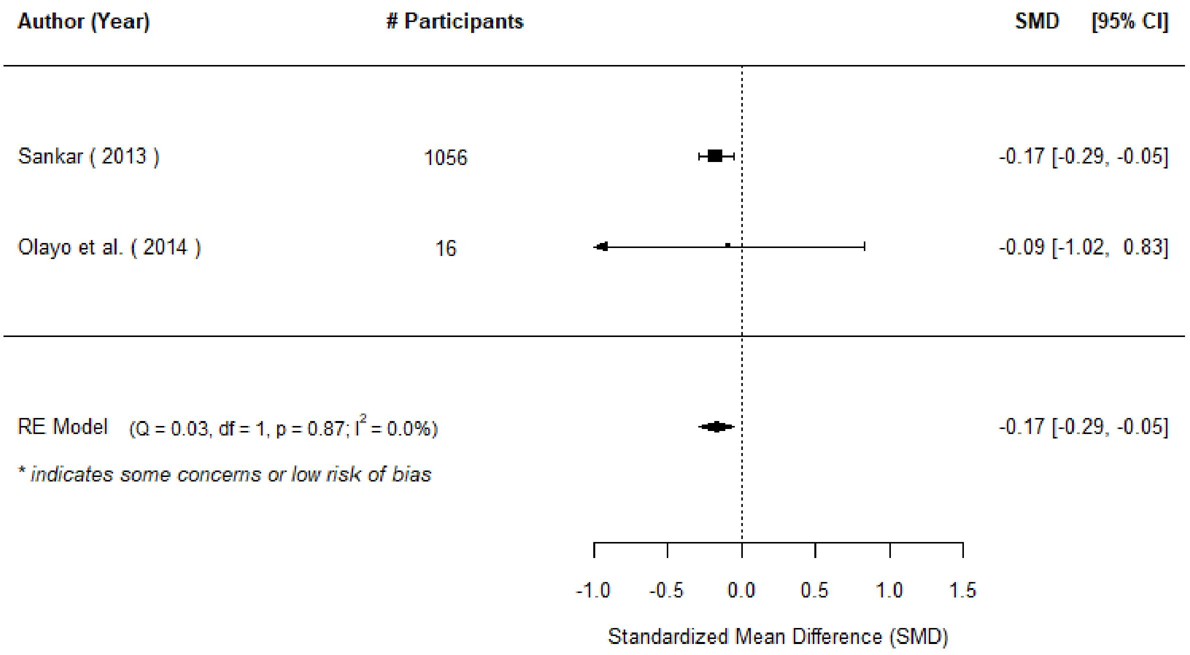

Supplementary Figure 72. Forest plot showing the observed outcomes and the estimate of the random-effects model for interventions with multiple engagement types on DPT1 vaccination.

DPT2

Only one study (Sankar 2013) fell into this intervention/outcome category, thus we were unable to perform a statistical synthesis. This quasi-experimental study from India found a null effect of their programme on OPV0 vaccination (g = 0.10 [95% CI: -0.02 to 0.22]), but like most studies, it was assessed as having a high risk of bias. Their programme included a combination of engagement in the implementation and engagement as the intervention.

OPV0

Only one study (Sankar 2013) fell into this intervention/outcome category, thus we were unable to perform a statistical synthesis. This quasi-experimental study from India found a significant positive effect of their programme on OPV0 vaccination (g = 0.43 [95% CI: 0.31 to 0.55]), but like most studies, it was assessed as having a high risk of bias. Their programme included a combination of engagement in implementation autonomy and engagement as the intervention.

OPV1

Only two studies reporting on OPV1 vaccination used interventions with multiple engagement types. The estimated average outcome based on the random-effects model was $\hat{\mu} =$ 0.22 (95% CI: -0.15 to 0.59). Therefore, the average

For peer review only - http://bmjopen.bmj.com/site/about/guidelines.xhtml

outcome did not differ significantly from zero ($z = 1.17$, $p = 0.24$, see Supplementary Figure 73). Given the small number of studies, this result should be interpreted with caution. According to the Q-test, the true outcomes appear to be homogeneous ($Q(1) = 3.61, p = 0.06, \hat{\tau}^2 = 0.05, I^2 = 72.32\%$). With only two studies, moderator and leave-one-out analyses were not appropriate and tests of publication bias are not valid.

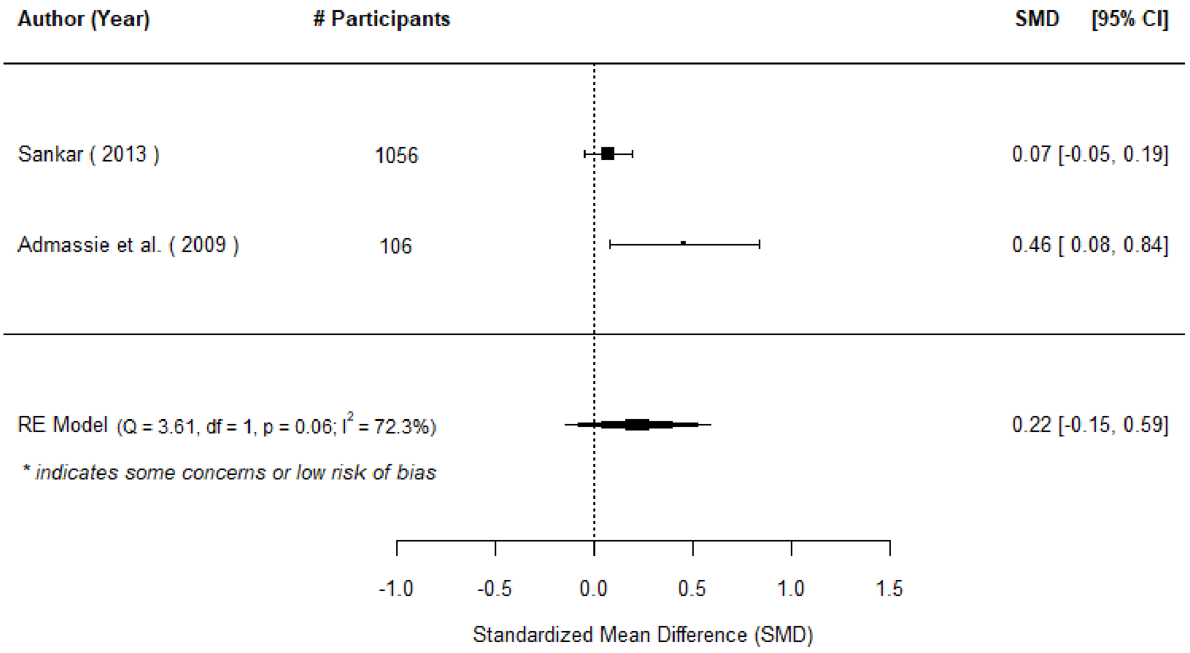

Supplementray Figure 73. Forest plot showing the observed outcomes and the estimate of the random-effects model for interventions with multiple engagement types on OPV1 vaccination.

OPV2

Only two studies reporting on OPV2 vaccination used interventions with multiple engagement types. The estimated average outcome based on the random-effects model was $\hat{\mu} = 0.34$ (95% CI: 0.19 to 0.50, $z = 4.28$, $p < 0.001$), indicating a small but significant benefit to the treated group compared to the control participants (see Supplementary Figure 74). Given the small number of studies, this result should be interpreted with caution. According to the Q-test, the true outcomes appear to be homogeneous ($Q(1) = 1.18, p = 0.28, \hat{\tau}^2 = 0.004, I^2 = 15.51\%$). With only two studies, moderator and leave-one-out analyses were not appropriate and tests of publication bias are not valid. Both studies used a combination of engagement in implementation autonomy and engagement as the intervention.

For peer review only - http://bmjopen.bmj.com/site/about/guidelines.xhtml

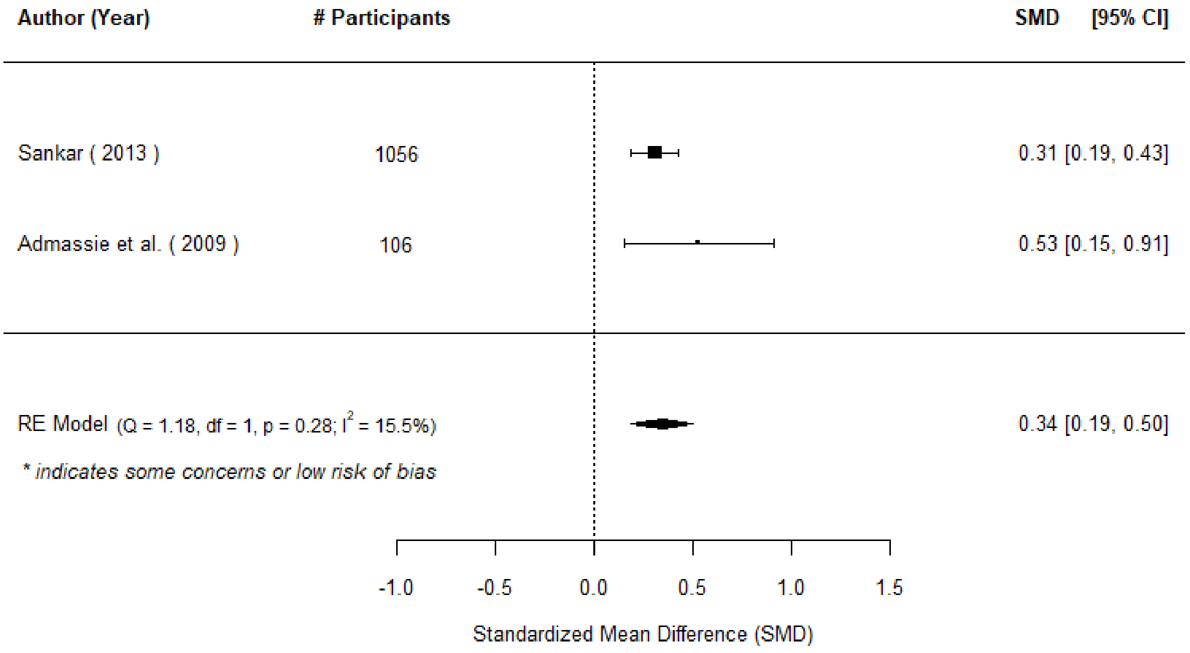

Supplementary Figure 74. Forest plot showing the observed outcomes and the estimate of the random-effects model for interventions with multiple engagement types on OPV2 vaccination.

OPV3

We included a total of $k = 3$ studies in the analysis. The estimated average outcome based on the random-effects model was $\hat{\mu} = 0.48$ ([95% CI: -0.24 to 1.20], $z = 1.30$, $p = 0.19$), indicating no difference between the intervention group and the control group (see Supplementary Figure 75). A 95% credibility/prediction interval for the true outcomes is given by -0.95 to 1.91. Hence, although the average outcome is estimated to be positive, in some studies the true outcome may in fact be negative.

According to the Q-test, the true outcomes appear to be heterogeneous ($Q(2) = 185.96$, $p < 0.001$, $\hat{\tau}^2 = 0.39$, $I^2 = 98.92\%$). An examination of the studentized residuals revealed that none of the studies had a value larger than ± 2.39 and hence there was no indication of outliers in the context of this model. However, the leave-one-out analysis indicates that removing Okeke et al. (2017) would result in a larger and statistically significant effect estimate ($\hat{\mu} = 0.76$ [95% CI: 0.37 to 1.16], $z = 3.80$, $p < 0.001$). According to the Cook's distances, none of the studies could be considered to be overly influential. With only three studies contributing effects, we were unable to examine sources of heterogeneity.

For peer review only - http://bmjopen.bmj.com/site/about/guidelines.xhtml

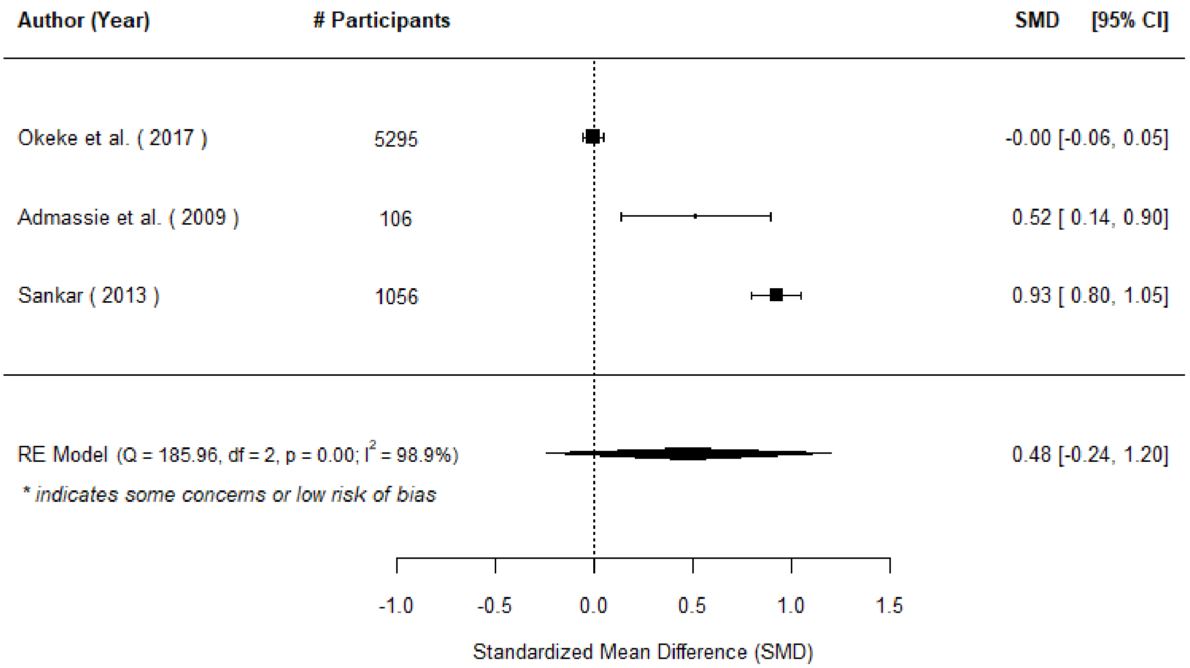

Supplementary Figure 75. Forest plot showing the observed outcomes and the estimate of the random-effects model for interventions with multiple engagement types on OPV3 vaccination.

Dropouts

Only two studies reporting on immunisation dropouts used interventions with multiple engagement types. All effects were computed such that a positive effect reflects a benefit to the treated group (e.g. a reduction in dropouts). The estimated average outcome based on the random-effects model was $\hat{\mu} =$ 0.18 (95% CI: 0.06 to 0.30). Therefore, the average outcome differed significantly from zero, indicating a small but significant benefit to the treated group compared to the control group ($z = 2.92$, $p = 0.004$, see Supplementary Figure 76). Given the small number of studies, this result should be interpreted with caution. According to the Q-test, the true outcomes appear to be homogeneous ($Q(1) = 2.35$, $p = 0.13$, $\hat{\tau}^2 = 0.004$, $I^2 = 57.38\%$). With only two studies, moderator and leave-one-out analyses were not appropriate and tests of publication bias are not valid.

For peer review only - http://bmjopen.bmj.com/site/about/guidelines.xhtml

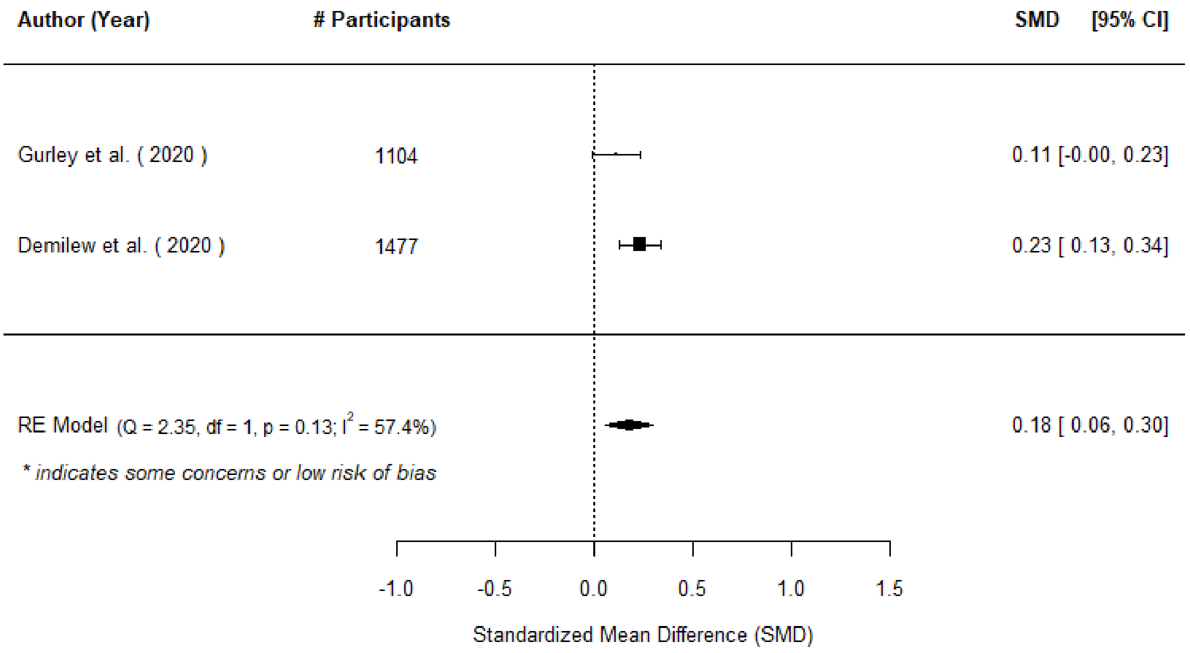

Supplementary Figure 76. Forest plot showing the observed outcomes and the estimate of the random-effects model for interventions with multiple engagement types on vaccination dropouts.

Morbidity

We used reports of diarrhea (most typically in the past two weeks) as a proxy for childhood morbidity. In all cases, effects were reverse coded such that positive effects always indicate a benefit to the treated group. Thus a positive effect here would be interpreted as a reduction in diarrhea among treated participants compared to control participants. Only two studies reporting on childhood morbidity used interventions with multiple engagement types. The estimated average outcome based on the random-effects model was $\hat{\mu} = -0.10$ (95% CI: -0.60 to 0.40). Therefore, the average outcome did not differ significantly from zero, indicating no difference between the treated group compared to the control group ($z = -0.38$, $p = 0.70$, see Supplementary Figure 77). Given the small number of studies, this result should be interpreted with caution. According to the Q-test, the true outcomes appear to be heterogeneous ($\mathrm{Q}(1) = 5.23$, $\mathrm{p} = 0.02$, $\hat{\tau}^2 = 0.11$, $\mathrm{I}^2 = 80.88\%$). With only two studies, moderator and leave-one-out analyses were not appropriate and tests of publication bias are not valid.

For peer review only - http://bmjopen.bmj.com/site/about/guidelines.xhtml

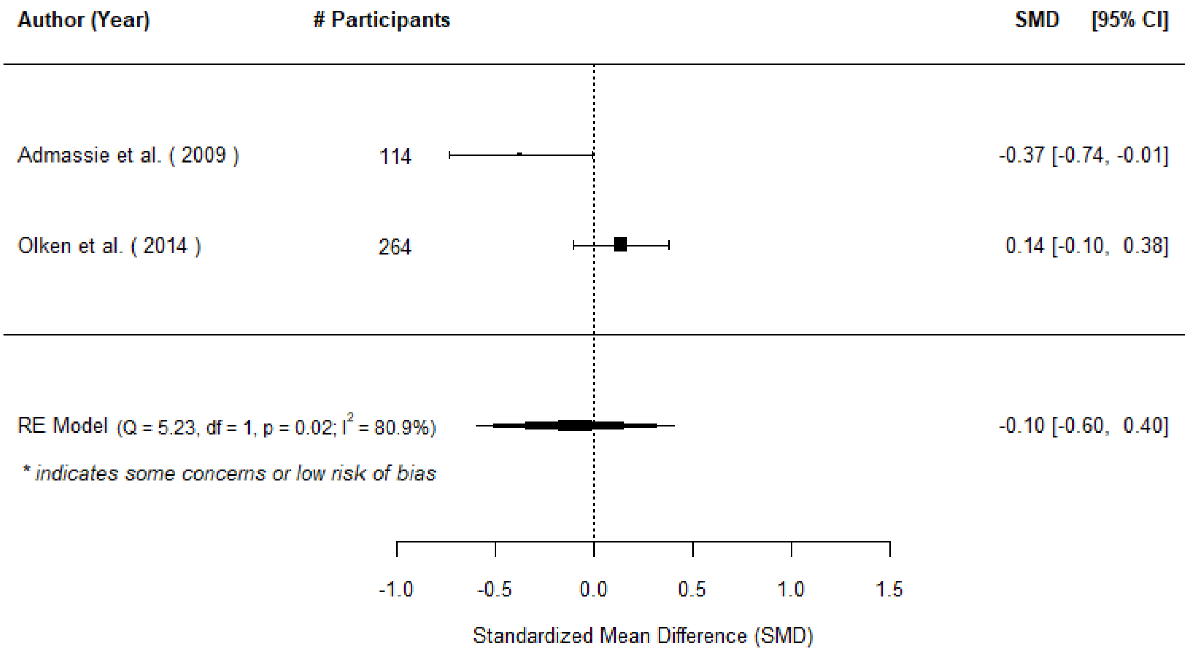

Supplementary Figure 77. Forest plot showing the observed outcomes and the estimate of the random-effects model for interventions with multiple engagement types on childhood morbidity.

Mortality

We included a total of $k = 3$ studies in the analysis. The observed outcomes ranged from -0.25 to -0.002. The estimated average outcome based on the random-effects model was $\hat{\mu} = -0.04$ (95% CI: -0.10 to 0.03, $see\ Supplementary\ Figure$ 78). Therefore, the average outcome did not differ significantly from zero ($z = -1.15$, $p = 0.25$), indicating no difference between the intervention participants and control participants on child mortality. A 95% credibility/prediction interval for the true outcomes is given by -0.14 to 0.07. Hence, although the average outcome is estimated to be negative, in some studies the true outcome may in fact be positive.

The Q-test for heterogeneity was not significant, but some heterogeneity may still be present in the true outcomes ($Q(2) = 5.37$, $p = 0.07$, $\hat{\tau}^2 = 0.0018$, $I^2 = 62.75\%$). An examination of the studentized residuals revealed that none of the studies had a value larger than ± 2.39 and hence there was no indication of outliers in the context of this model. This was confirmed by a leave-one-out analysis. According to the Cook's distances, none of the studies could be considered to be overly influential. With only three studies, moderator analyses were not appropriate and tests of publication bias are not valid.

For peer review only - http://bmjopen.bmj.com/site/about/guidelines.xhtml

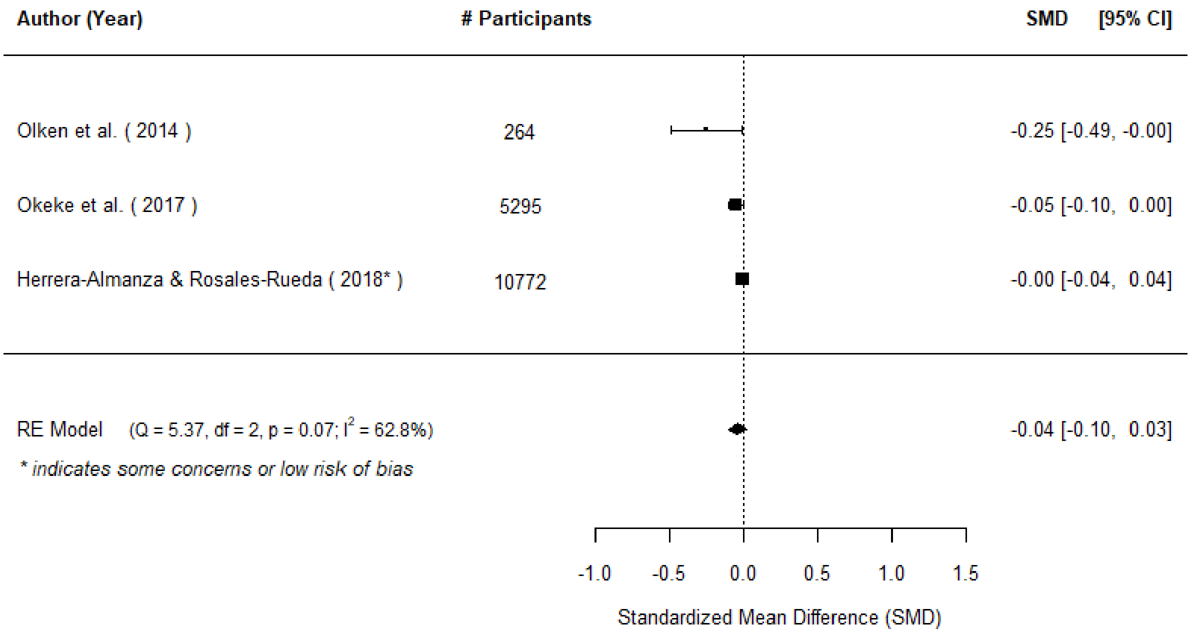

Supplementary Figure 78. Forest plot showing the observed outcomes and the estimate of the random-effects model for interventions with multiple engagement types on childhood morbidity.

Immunisation knowledge

Only two studies reporting on immunisation knowledge used interventions with multiple engagement types. The estimated average outcome based on the random-effects model was $\hat{\mu} = 0.09$ (95% CI: 0.04 to 0.13). Therefore, the average outcome differed significantly from zero, indicating a small but significant benefit to the treated group compared to the control group ($z = 3.55$, $p < 0.001$), see Supplementary Figure 79). Given the small number of studies, this result should be interpreted with caution. According to the Q-test, the true outcomes appear to be homogeneous ($\mathrm{Q}(1) = 0.19$, $\mathrm{p} = 0.66$, $\hat{\tau}^2 = 0.00$, $\mathrm{I}^2 = 0.00\%$). With only two studies, moderator and leave-one-out analyses were not appropriate and tests of publication bias are not valid.

For peer review only - http://bmjopen.bmj.com/site/about/guidelines.xhtml

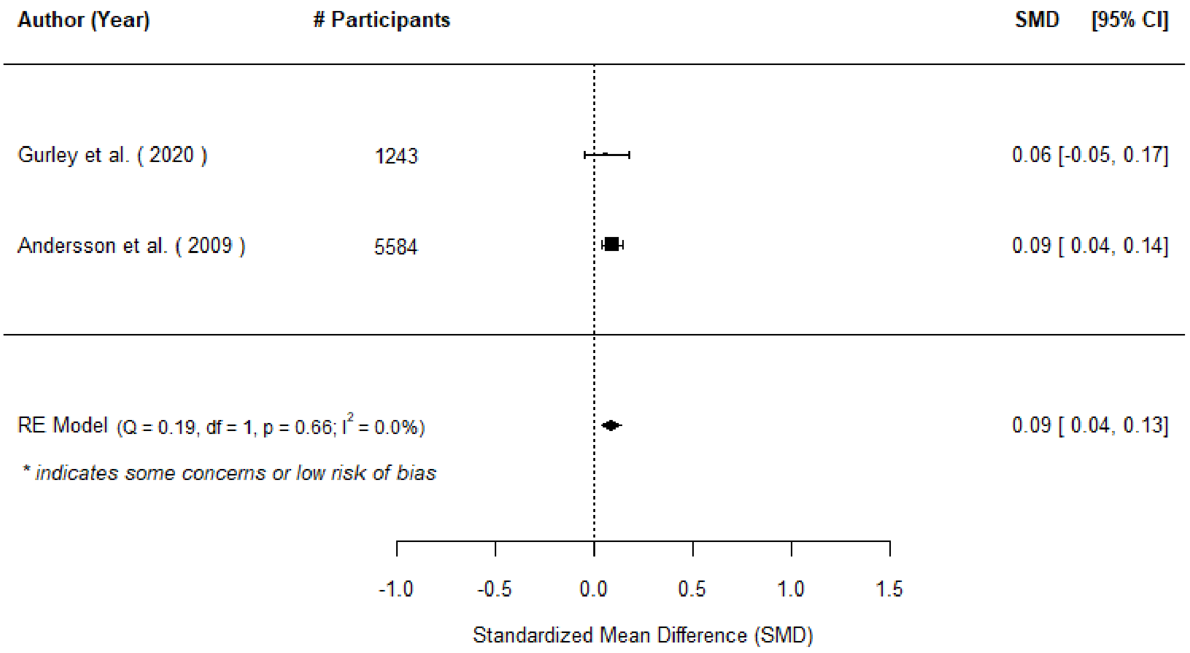

Supplementary Figure 79. Forest plot showing the observed outcomes and the estimate of the random-effects model for interventions with multiple engagement types on immunisation knowledge.

Immunisation attitudes

Only two studies reporting on immunisation attitudes used interventions with multiple engagement types. The estimated average outcome based on the random-effects model was $\hat{\mu} =$ 0.06 (95% CI: 0.01 to 0.11). Therefore, the average outcome differed significantly from zero, indicating a small but significant benefit to the treated group compared to the control group ($z = 2.50$, $p = 0.01$), see Supplemenatry Figure 80). Given the small number of studies, this result should be interpreted with caution. According to the Q-test, the true outcomes appear to be homogeneous ($\mathrm{Q}(1) = 0.50$, $\mathrm{p} = 0.48$, $\hat{\tau}^2 = 0.00$, $\mathrm{I}^2 = 0.00\%$). With only two studies, moderator and leave-one-out analyses were not appropriate and tests of publication bias are not valid.

For peer review only - http://bmjopen.bmj.com/site/about/guidelines.xhtml

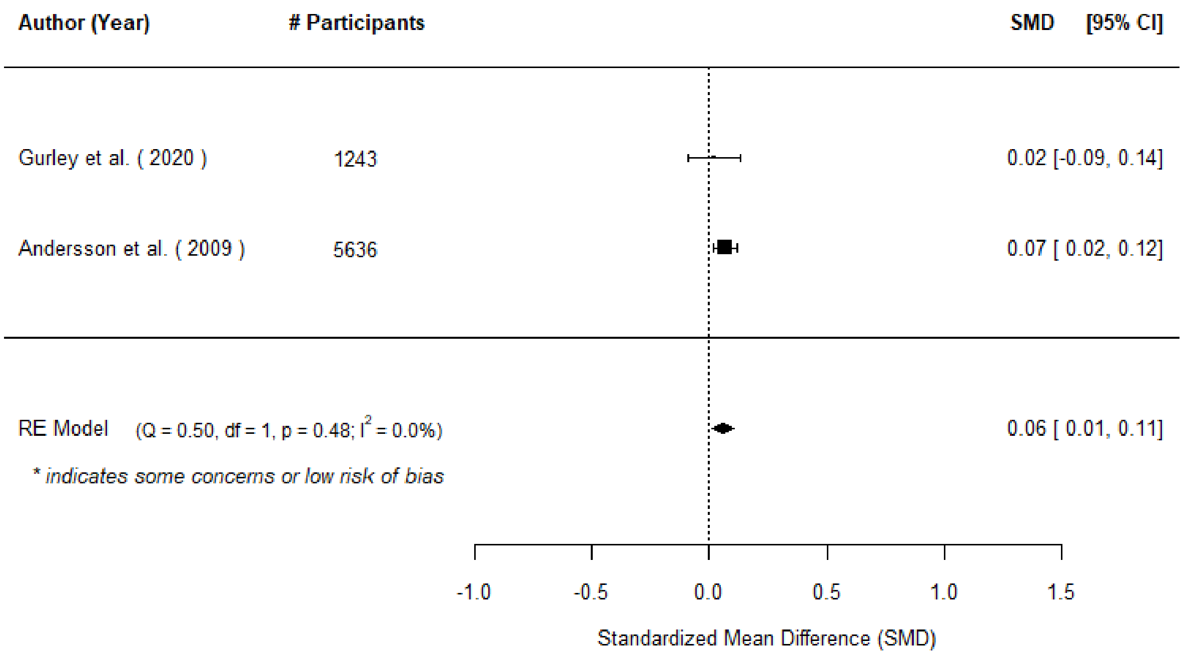

Supplementary Figure 80. Forest plot showing the observed outcomes and the estimate of the random-effects model for interventions with multiple engagement types on immunisation attitudes

Vaccination card availability

Only two studies reporting on vaccination card availability/retention used interventions with multiple engagement types. The estimated average outcome based on the random-effects model was $\hat{\mu} = -0.01$ (95% CI: -0.05 to 0.02). Therefore, the average outcome did not differ significantly from zero ($z = -0.78$, $p = 0.43$, see Supplementary Figure 81). Given the small number of studies, this result should be interpreted with caution. According to the Q-test, the true outcomes appear to be homogeneous ($Q(5) = 0.13$, $p = 0.43$, $\hat{\tau}^2 = 0.00$, $I^2 = 0.00\%$; With only two studies and no heterogeneity, moderator and leave-one-out analyses were not appropriate and tests of publication bias are not valid.

For peer review only - http://bmjopen.bmj.com/site/about/guidelines.xhtml

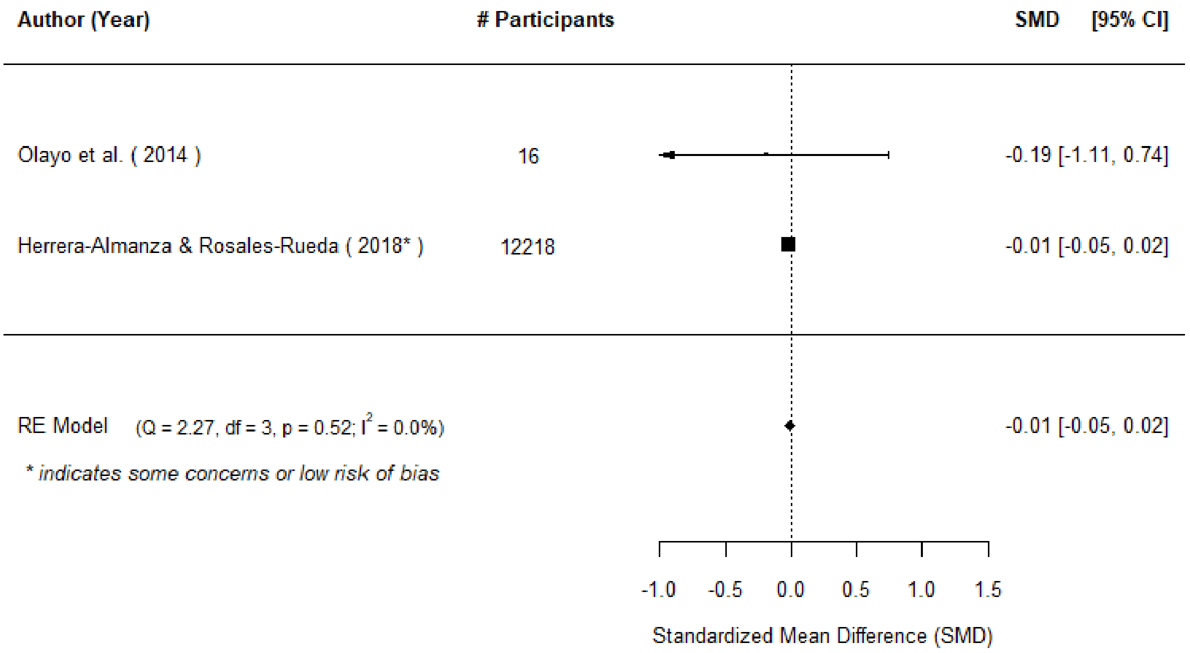

Supplementary Figure 81. Forest plot showing the observed outcomes and the estimate of the random-effects model for interventions with multiple engagement types on vaccination card availability

For peer review only - http://bmjopen.bmj.com/site/about/guidelines.xhtml

Appendix 14: Qualitative synthesis of results

Barriers to immunisation

Most information on barriers came from studies which used engagement as the intervention. Often, multiple barriers were cited in the same sentence. The vast majority of the barriers were identified through individual interviews and focus group discussions in the primary papers from which they were drawn. In addition, these were often supported by the authors' reflections, implementers' experiences, and observations. Sensitivity analysis, in which only qualitative papers that received an assessment score greater than 20 were included, indicated similar results. Therefore, we view the strength of the evidence to be high. Behavioural, social and practical barriers faced by caregivers were more consistently cited than direct constraints within the health system (Supplementary Figure 82, Supplemetary Table 4).

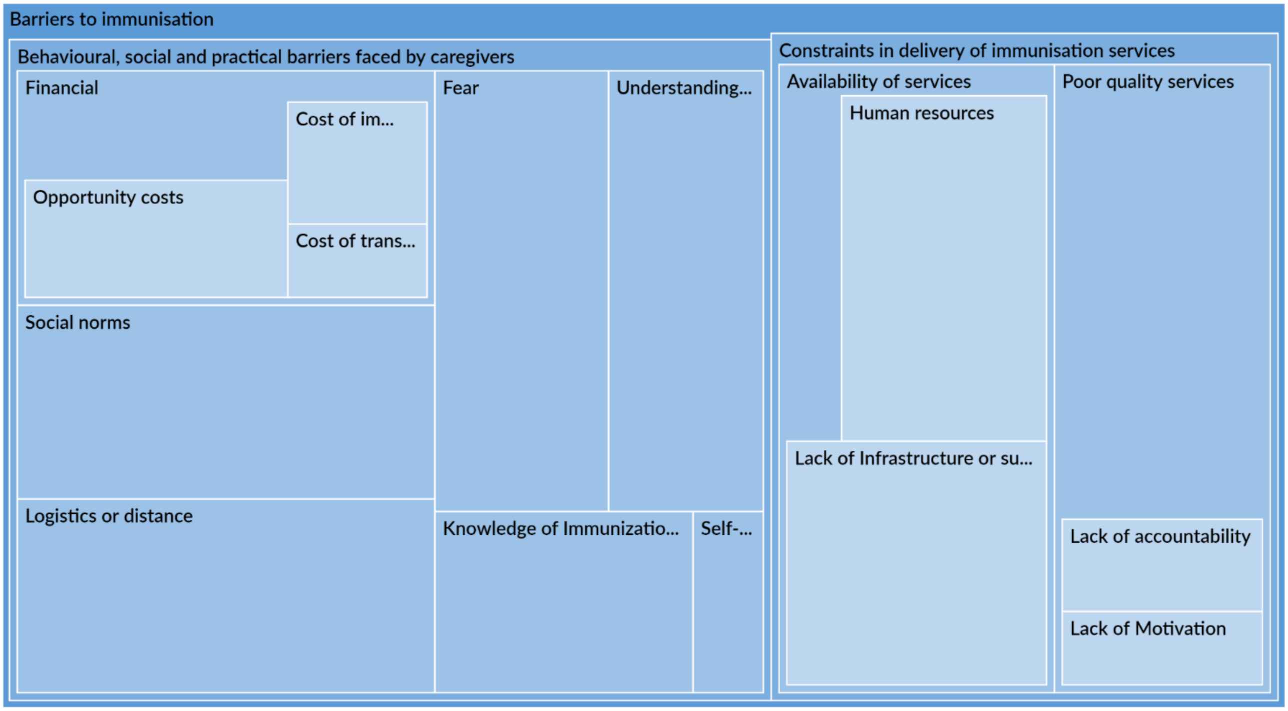

Supplementary Figure 82: Summary figure: Behavioural, social and practical barriers faced by caregivers were more consistently experienced than constraints in delivery of immunisation services[1]

The size of the rectangles reflects the number of times a unique theme was mentioned by an IE and its associated papers. Larger rectangles indicate that a theme was mentioned more times. Nested boxes reflect the proportion of times the main theme text also fell into the sub-them.

Supplementary Table 4: Comparison of the barriers to immunisation in communities in which community engagement activities were undertaken.

	**Community engagement as the intervention**	**Community engagement in the design of the intervention**	**Community engagement in the implementation autonomy of the intervention**

[1] The hierarchy charts showing barriers by type of community engagement are in Appendix H: barriers to immunisation

For peer review only - http://bmjopen.bmj.com/site/about/guidelines.xhtml

**Availability of resources**	Staff<br>Absenteeism<br>Stockouts<br>Building infrastructure	Staff<br>Stockouts	Staff<br>Post abandonment<br>Stockouts
**Quality of services**	Attitudes of health workers	Attitudes of health workers<br>Quality affected attendance	Attitudes of health workers<br>Services not implemented as intended
**Costs**	Other costs<br>Cost of the vaccine*	Other costs	Other costs<br>Cost of the vaccine
**Logistics or distance**	Distance	Convenience<br>Distance and access	Convenience<br>Transport
**Social norms**	Religious beliefs<br>Beliefs of others		Spousal consent<br>Religious beliefs
**Fear**	Side effects (and overcoming this fear)<br>Trust in the government or medicine	Side effects (from past experience)	
**Understanding of importance**	Cost and importance<br>Fear and importance		

Behavioural, social and practical barriers faced by caregivers

The most consistent behavioural, social or practical barrier faced by caregivers was cost. However, the kind of cost which posed a barrier was inconsistent, and mostly not that of the vaccination itself. Associated costs include opportunity costs, transport costs, and the costs of treating side effects. The cost of the immunisation itself was mentioned as a barrier by community members that received engagement as the intervention or engagement in the implementation of the intervention.

Logistics and distance were also consistent barriers to immunisation in settings where all three types of interventions occurred. The nature of the logistical or distance challenge was variable. Distance alone was reported as an issue in the communities with engagement as the intervention. However, Olken 2014 (World bank 2018) mention other logistical barriers such as wait times and language barriers. In settings where the community was involved in the design or implementation of the intervention, convenience was an issue in addition to distance. Community members in these areas reported issues related to wait times and the timing of clinic visits. Adamu 2019 (Adamu 2019 pre-implementation and Adamu 2019 Dissertation) mention demand being lower than it could be because women were unable to arrive at clinics at specified times due to competing demands.

Fear of side effects was common in communities in which engagement was the intervention or engagement was used in the design of the intervention. The concern was consistently related to known side effects and affected by past experience of side effect and trust in the government or medicine. The issue of vaccines causing sterilization was also mentioned. In one case, the intervention reduced these fears (Gurley 2020 (Gurley 2020)).

Social norms around immunisation posed consistent barriers; however, the source of the social restriction was variable. Spousal consent limited immunisations in areas where engagement was used in the implementation of interventions;

For peer review only - http://bmjopen.bmj.com/site/about/guidelines.xhtml

women could not vaccinate their children without the explicit approval of their husband, who may refuse. Religious beliefs sometimes resulted in the preference for non-Western medicine. In areas that received engagement as the intervention, religious beliefs and the influence of beliefs held by others sometimes inhibited immunisation. In particular, mother-in-laws and other family members who do not value immunisation may pose a barrier to immunisations.

Failure to *understand the importance of vaccination* was not consistently observed but reported in settings that received engagement as the intervention. When this barrier was mentioned, it was always paired with another, such as cost or fear. Authors implied that these other barriers were experienced and could not be overcome because there was a lack of understanding of the importance of vaccinations.

Constraints in delivery of immunisation services

Constraints in the delivery of immunisation services were widely reported but did not seem to be as common of a barrier as the behavioural, social and practical barriers faced by caregivers. The *availability of services* was the most consistently reported delivery barrier across all three types of engagement. Limited staff and stockouts were dominant delivery challenges. Communities that had engagement as the intervention or were engaged in implementation consistently have issues with staff being assigned but unavailable. Poor infrastructure, such as dilapidated buildings, was a challenge in communities that were involved in the design of the intervention. In areas where engagement was the intervention or the community was involved in the design of the intervention, the *quality of services* was also a more consistently reported barrier than any behavioural, social, or practical barrier faced by caregivers. Often, these were related to health worker attitudes, either a lack of motivation or conflict with caregivers.

Barriers to immunisation by type of community engagement

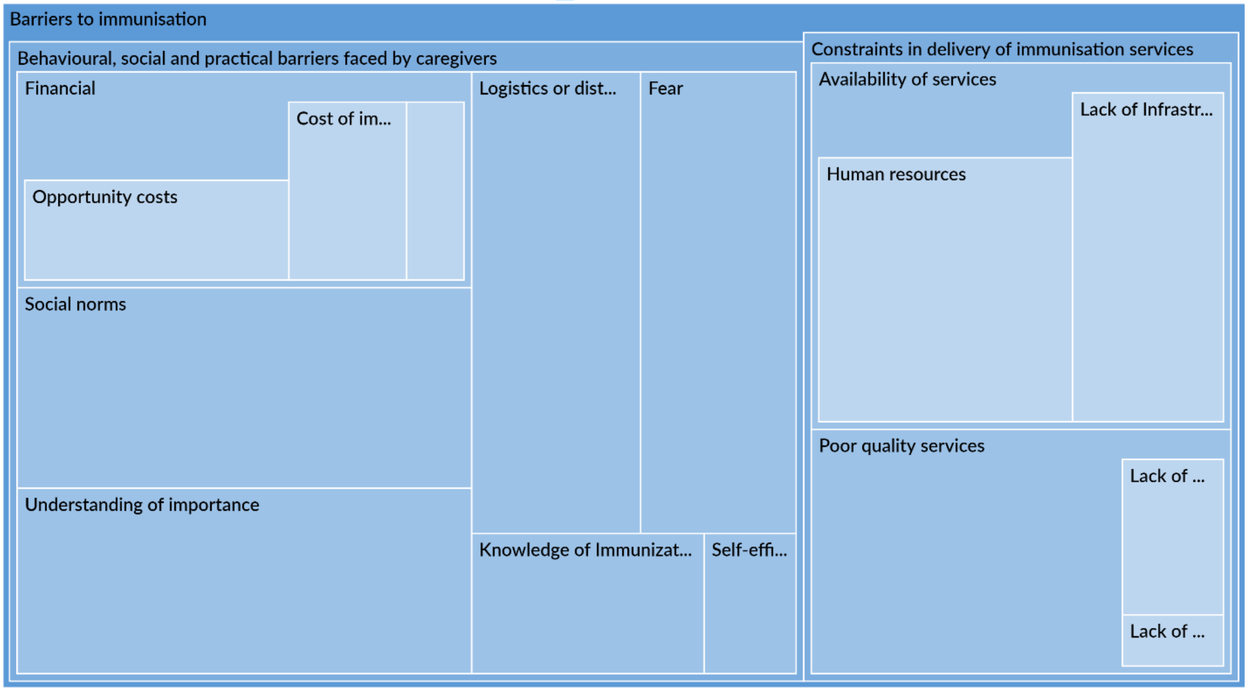

Figure 83: Engagement as intervention

For peer review only - http://bmjopen.bmj.com/site/about/guidelines.xhtml

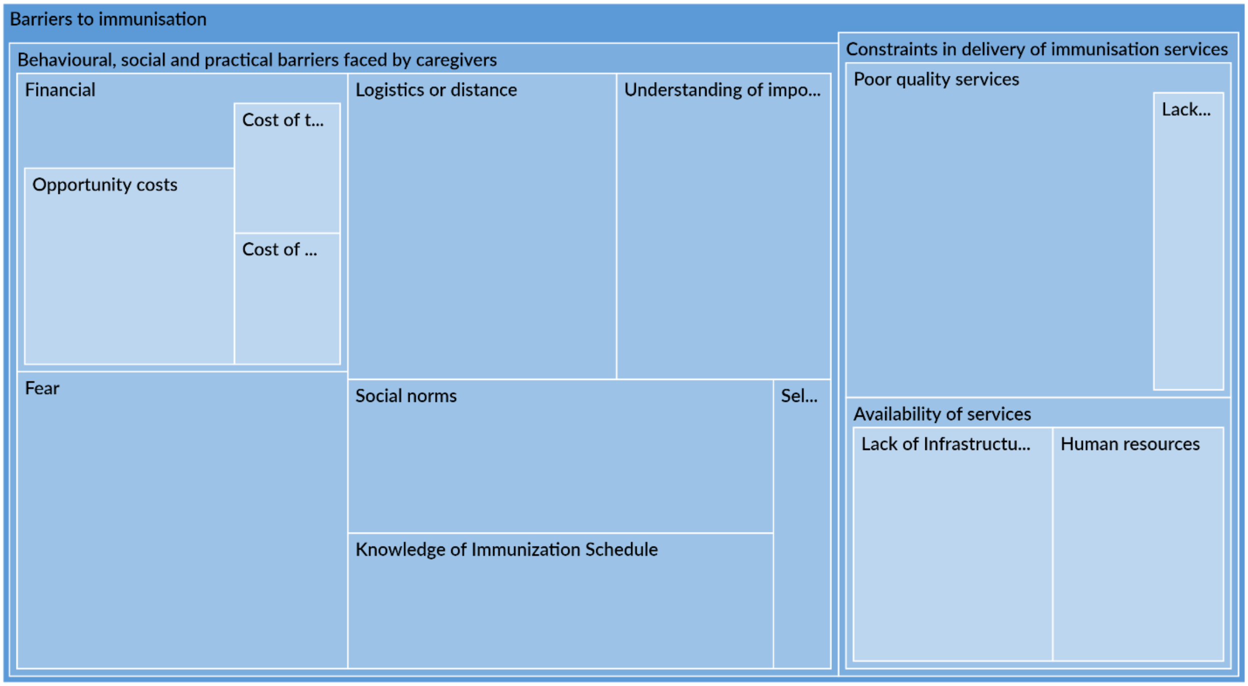

Figure 84: Engagement in design

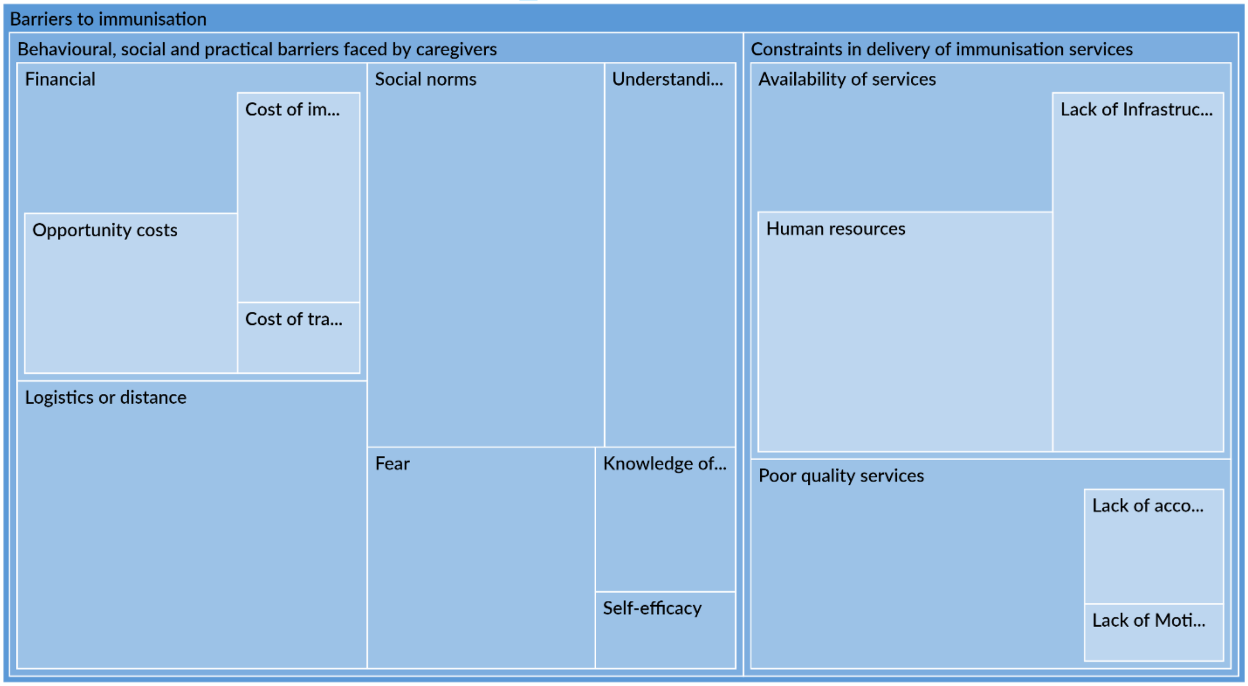

Figure 85: Engagement in implementation autonomy

Supplementary Table 5: Characteristic quotes of themes identified related to barriers to immunisation for interventions were community engagement.

For peer review only - http://bmjopen.bmj.com/site/about/guidelines.xhtml

Primary paper	Paper from which citation comes	Theme demonstrated	Quote	Page number
Financial				
Okeke et al 2017	Okeke et al 2017	Cost of vaccination	costs associated with use of services and lack of adequate transportation, hindered uptake.	54
Andersson 2009	Mitchell 2009	Other costs	Yet the groups noted that immediate financial and time costs associated with having a child vaccinated weigh very heavily in comparison with the potential costs associated with measles in the future.	7
Social norms				
Banerjee et al 2020	Appendix K	Religion	Religion emerged as an implicit theme. Only two respondents mentioned explicitly that religious belief might inhibit immunisation: that God would decide the fate of their child.	7
Andersson 2009	Cockroft 2009	Beliefs of others	"My mother-in-law says 'What kind of children have you produced that they ought to be vaccinated? We were never vaccinated, so why are you behaving so delicately?'" (female group, Khanewal)	8
Understanding of importance				
Olken 2014	Grayman 2013	Costs and importance	Typically the answer is that the parents are too busy working on their farms, or they feel that posyandu is no longer necessary after they are older than two years of age.	66
Paramanik 2018	Paramanik 2020	Fear and importance	As reported by ASHAs the mothers-in-law were not able to accept the concept of vaccination. They apparently argued on the ground that they had children too and that they never took them for any vaccination, that post vaccination the child gets fever which causes sleep disturbance for the child and in turn negatively affects the whole family.	84

For peer review only - http://bmjopen.bmj.com/site/about/guidelines.xhtml

Logistics or distance

Alhassan 2019	Alhassan 2019	Distance	In Ghana, access to maternal and child health services is impeded by longer travel times to health facilities.	2
Oyo-Ita 2020	Oyo-Ita 2020	Distance	children whose mothers found it difficult to reach the health facility	

Fear

Banerjee et al 2020	Appendix K	Trust	A couple of respondents suggested this could be interacting with a lack of trust of front line health workers.	20
Oyo-Ita 2020	Oyo-Ita 2020	Side effects	She said she doesn't want anybody to give her child injection so that the child will not become sick	31
Gurley 2020	Gurley 2020	Overcoming fear of side effects	We observed changes in how FGD respondents discussed side effects over the course of the study, from a perception that they were severe and not worth the risk of vaccination, to a sense they could be handled and are not a reason to forgo vaccination. I also took my child for vaccination. She cried for two days and I said let her cry; at least she will be safe in future from diseases. Mothers FGD, September 2017, intervention village	35

Human resources

Gurley 2020	Gurley 2020	Staff	In addition to suboptimal ANM clinical quality, nine ANM posts were vacant during the study period, resulting in suboptimal availability of services in some communities, which was beyond our influence	38
Olken 2014	Rahayu 2008	Absenteeism	the community is often disappointed with the services of puskesmas officers who do not attend posyandu services as this means that immunisations must be postponed until the posyandu session scheduled for the next month.	33

For peer review only - http://bmjopen.bmj.com/site/about/guidelines.xhtml

Infrastructure and supplies				
Okeke et al 2017	Okeke et al 2017	Building infrastructure	That is supposed to be our office. If you get there now, half of it is just sand; even the doors to this clinic are not closing, they are eating up by termite. the condition of two of the facilities was perceived to be detrimental to their ability to deliver care.	43
Findley et al 2013	Doctor et al 2011	Vaccine stockout and long wait times	67% of parents were unable to receive all immunisations reported lack of vaccine as a problem, and 13% had difficulties with the long wait.	17
Multiple barriers				
Okeke et al 2017	Okeke et al 2017	Cost of vaccination and logistics	costs associated with use of services and lack of adequate transportation, hindered uptake.	
Arifeen 2019	Billah 2018	Many simultaneous barriers	Furthermore, the lack of trust in formal health care providers, high out of pocket costs of health care in the public sector, and challenges with accessibility has led to the rapid proliferation of informal health providers to fill the gap between supply and demand across both rural and urban areas	2
Tandon 1988	Islam 2013	Many simultaneous barriers	low capacity to supervise monitor and implement micro plans at district level, lack of effective vaccine distribution to immunisation sites, ageing and poorly maintained Cold Chain, lack of adequately trained human resources, low managerial and support capacity at the state and district immunisation units and weak management of fund flows.	10

Supplementary Table 6: Characteristic quotes of themes identified related to barriers to immunisation for interventions that used community engagement in their design.

For peer review only - http://bmjopen.bmj.com/site/about/guidelines.xhtml

Primary paper	Paper from which citation comes	Theme demonstrated	Quote	Page number
Financial				
Anderson 2009	Andersson 2009	Other costs	Discussions in our focus groups confirmed the importance of poverty as a barrier to vaccination in many cases, as parents described being unable to afford the costs of the supposedly "free" immunisations: travel costs, opportunity costs, and demands for unofficial payments.	10
Fear				
Dipeolu 2017	Oladepo 2019	Fear of side effects	Prominent factors perceived as affecting timely and full completion of routine immunisations for children aged below 12 months include lack of awareness of immunisation (61.6%) and of subsequent doses (58.4%), and fear of side effects (59.7%). Others include rumours (for example, an association with future infertility)	464
Adamu 2019	Adamu 2019 Dissertation	Past experience	Experiences with the side effects of vaccines can influence the behaviour of caregivers and reduce their motivation to immunize eligible children.	134
Logistics				
Andersson 2009	Mitchell 2009	Physical access to facilities	In both urban and rural areas, access to a government facility providing vaccinations, a key equity factor, was a determining factor for uptake.	8
Nagar 2018	Nagar 2016	Convenience	not knowing where to go, not having time or mutually convenient time, facing long wait times at the camp, having fear of side effects, and acting under misguided advice	5
Human resources				

For peer review only

Borkum 2014	Rangarajan 2013	Staff	Staff vacancies are common, especially for lady health visitors and pharmacists. Few of the PHCs surveyed are fully staffed.	
Infrastructure or supplies				
Domek 2019	Domek et al 2018 Vaccine hesitancy	Stock outs	Of note, Guatemala experienced significant political instability during our study period, which led to considerable vaccine shortages experienced by all of our clinics.	3
Poor quality				
Dipeolu 2017	Dipeolu 2017	Attitudes of health workers	Attitudes and behaviours of healthcare workers such as treating mothers in an unfriendly, disrespectful, or even abusive manner are frequently cited as discouraging children's vaccination. Healthcare workers reportedly screamed at mothers who forgot the child's card, missed a scheduled vaccination	49
Gurely 2020	Gurley 2020	Quality affected attendance	For example, the existence of ANM vaccinators did not ensure their quality, and we observed that suboptimal clinical ANM quality discouraged retention across the vaccine schedule and perhaps negatively impacted beneficiaries' trust in the health system.	37

Supplementary Table 7: Characteristic quotes of themes identified related to barriers to immunisation for interventions that used community engagement in their implementation

**Primary paper**	**Paper from which citation comes**	**Theme demonstrated**	**Quote**	**Page number**
Financial				
Okeke et al 2017	Okeke et al 2017	Cost of the vaccine itself	costs associated with use of services and lack of adequate transportation, hindered uptake.	54

For peer review only - http://bmjopen.bmj.com/site/about/guidelines.xhtml

Adamu 2019	Adamu 2019 Dissertation	Other costs associated with vaccination	However, the cost of treating vaccine reactions like fever that might occur following immunisation are borne for the caregivers, which can result in out-of-pocket expenditure.	133
Social norms				
Adamu 2019	Adamu 2019 Dissertation	Spousal consent	In addition, we found that the high level of social control that men have over women in this area could also cause MOV among children. Caregivers reported that without their husband's consent, they still cannot vaccinate their children, even if they're in a health facility for other preventive or curative services.	133
Olken 2014	Rahayu 2008	Religious beliefs	The community trusts and believes in the choice that their parents made to use the dukun beranak, to the point where it becomes the norm.	34
Logistics or distance				
Webster 2019	Webster et al 2019	Convenience	Key reasons cited for non-immunisation were related to lack of convenience; the aforementioned distance between homes and health facilities, moving to the fields during farming seasons,	42
Alhassan 2019	Alhassan 2019	Transport	In Ghana, access to maternal and child health services is impeded by longer travel times to health facilities.	2
Human resources				
Olken 2014	Febriany 2011	Post abandonment	Poor facilities adversely affected the availability and utilization of MCH and basic education services. The midwife did not want to live in the village because here there is no electricity and no water (FGD Female-NTT).	9

For peer review only - http://bmjopen.bmj.com/site/about/guidelines.xhtml

Herrara-Almanza 2018	Herrara-Almanza & Rossales-Rueda 2018	Lack of personnel	Poor transportation infrastructure and a shortage of medical personnel limit the basic health access to the population living in remote areas	7
Infrastructure or supplies				
Webster 2019	Webster et al 2019	Vaccine stockouts	Furthermore, vaccine stock-outs at health facilities discouraged caregivers from attending vaccination services.	42
Multiple barriers				
Webster 2019	Webster et al 2019	Demand and supply side	Health system factors affecting immunisation service delivery and uptake in northern Uganda include interruptions in the vaccine cold chain due to poor management of equipment and supplies, limited supervision of health teams by the district health teams, low staffing levels, long distances to the health facilities especially in the hard-to-reach areas, and limited resources to support outreach services. There is also a lack of good quality data to support decision-making.	1
Olken 2014	Rahayu 2008	Poor quality and post abandonment	Dissatisfaction is often related to the midwife's character, ineffective medicine, minimal experience of the midwife, difficulties in reaching the midwife, and a midwife's absence from the post.	68
Herrara-Almanza 2018	Herrara-Almanza & Rossales-Rueda 2018	Transport and personnel	Poor transportation infrastructure and a shortage of medical personnel limit the basic health access to the population living in remote areas	7

Facilitators of immunisation

Fewer studies discussed facilitators of immunisation as compared to barriers. Much of the information coded came from studies in which the intervention was engagement. To a substantial extent, the sources of this information were key individual interviews and focus group discussions. These were often supported by surveyor or enumerator's observations. In some instances, discussion on facilitators in the primary papers was informed by existing literature.

For peer review only - http://bmjopen.bmj.com/site/about/guidelines.xhtml

A sensitivity analysis, comprising 19 qualitative papers with a risk of bias assessment score of 20 or higher, indicated similar results. Therefore, we view the evidence to be of high strength. Facilitators related to behavioural, social and practical factors faced by caregivers were more common than the facilitators related to the delivery of immunisation services (Supplementary Figure 86). A segregated analysis of the facilitators by the engagement type was not possible due to the limited information coded on this theme.

Behavioural, social and practical barriers faced by caregivers

The most consistent facilitator across all intervention types was the caregiver's *understanding of importance* of immunisation. In general, studies associated caregivers' awareness and perception of the benefits of vaccination to an enabling environment for immunisation uptake (Adamu 2019; Banerjee 2020). In most instances caregivers were able to link benefits of vaccines to the diseases it prevented (Anderson (Cockroft 2009); Pramanik 2020).

Lack of logistics or distance related issues and enabling *social norms* were also consistently sighted as facilitators of immunisation. When it came to logistics, easy access either in the form of proximity to a health care facility (Anderson (Cockroft 2009)) or provision of transportation to an immunisation camp (Olken 2014; Rahayu 2008) were cited as enabling factors. *Social norms* were mentioned as a facilitator in instances where mothers had a significant influence of over decisions regarding her child's health. In addition, peer influence was also mentioned as a factor (Banerjee 2020). Though less consistently cited than the other factors, f*avourable socio-economic characteristics* such as maternal education (Admassie 2009) were also cited as facilitators of immunisation.

Constraints in delivery of immunisation services

Delivery of immunisation services was less consistently reported as a facilitator as compared to behavioural, social and practical factors. The most consistentdelivery related facilitator was the *availability of infrastructure or supplies* which included provision of good quality of services, availability of adequate healthcare providers at the point of care and availability of vaccine supplies.

For peer review only - http://bmjopen.bmj.com/site/about/guidelines.xhtml

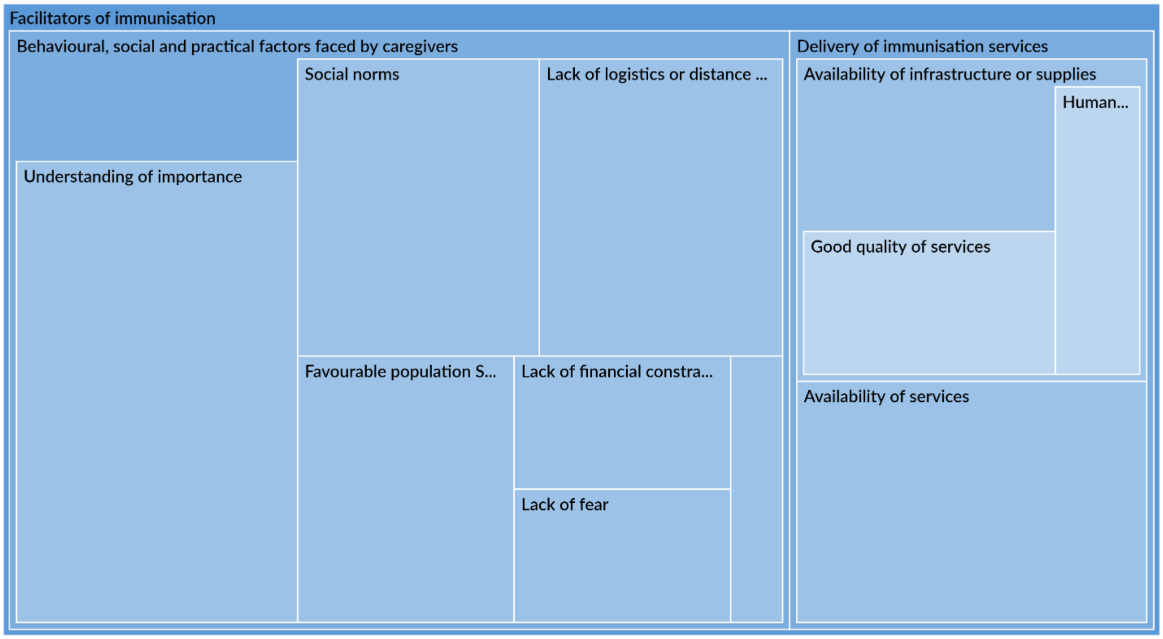

Supplementary Figure 86: Summary figure: Facilitators related to behavioural, social and practical factors faced by caregivers were more common than those related to delivery of immunisation services

The size of the rectangles reflects the number of times a unique theme was mentioned by an IE and its associated papers. Larger rectangles indicate that a theme was mentioned more times. Nested boxes reflect the proportion of times the main theme text also fell into the sub-them.

Facilitators of immunisation by type of community engagement

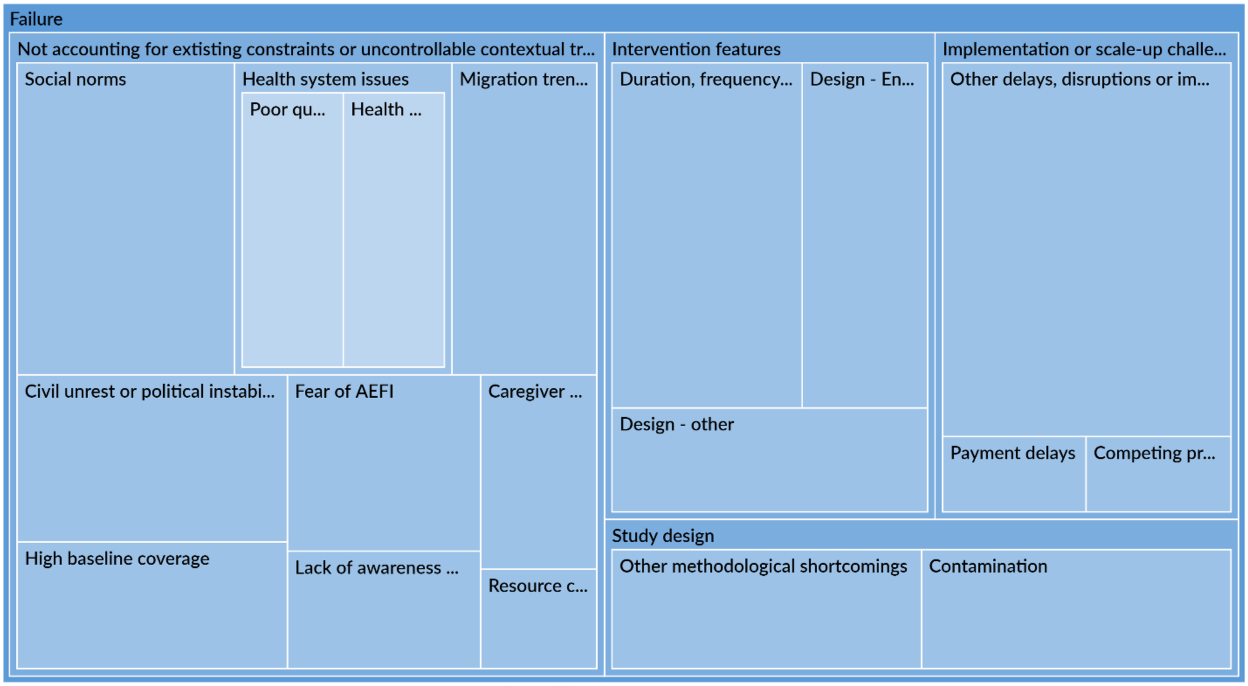

Supplementary Figure 87: Engagement as intervention

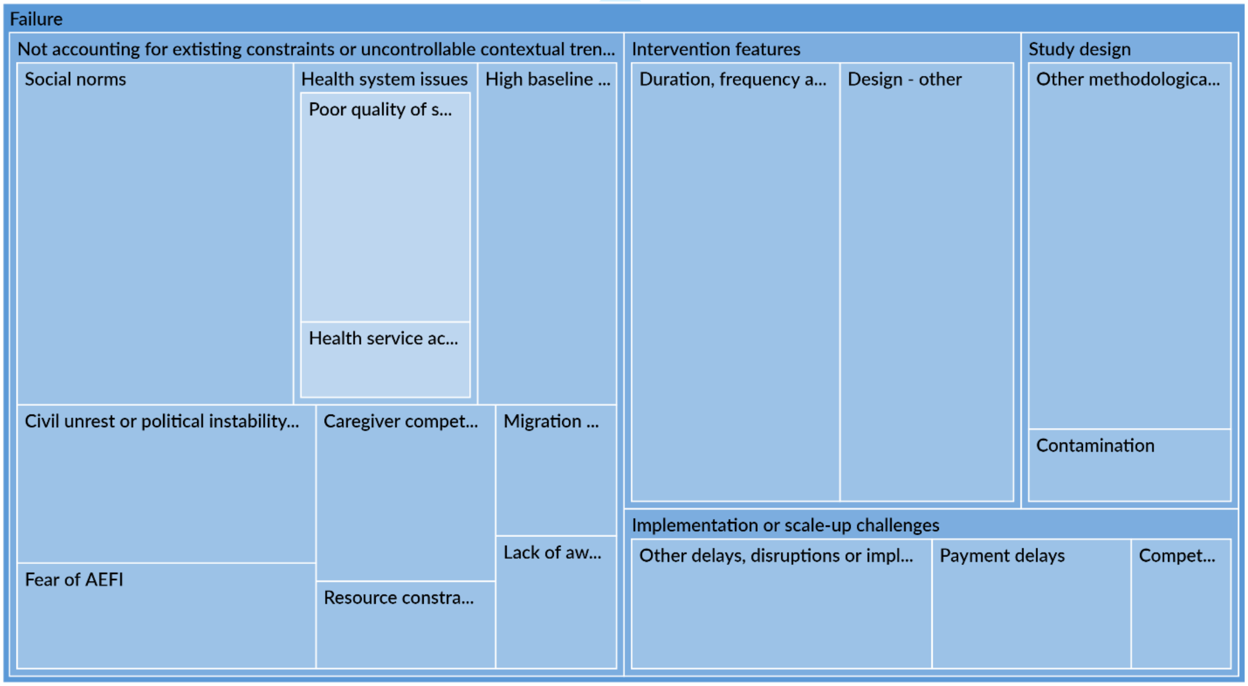

Supplementary Figure 88: Engagement in design

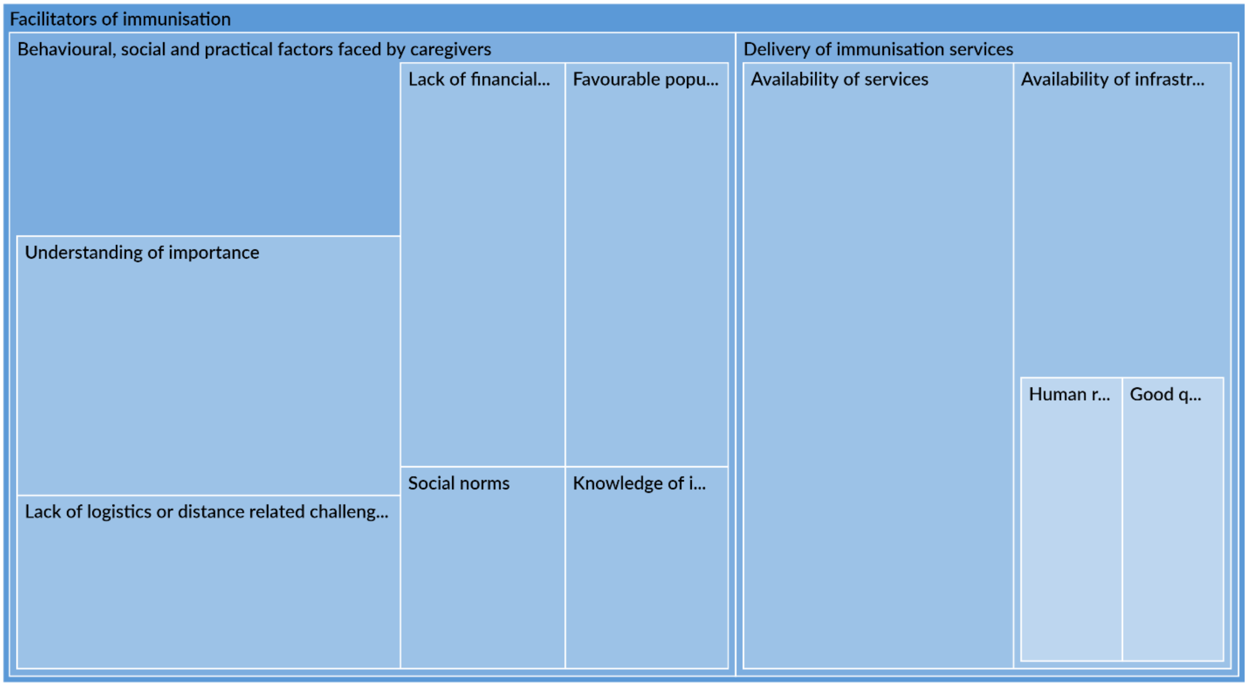

Supplementary Figure 89: Engagement in implementation autonomy

Supplementary Table 8: Characteristic quotes of themes identified related to facilitators of immunisation for community engagement interventions

**Primary paper**	**Paper from which citation comes**	**Theme demonstrated**	**Quote**	**Page number**
Understanding of importance				
Adamu et al. 2019	Adamu 2019 Dissertation	Awareness of benefits	In addition, caregiver beliefs about the capabilities of immunization was overwhelmingly strong: "… this vaccination is very important because it prevents infection from measles, cough, hepatitis, fever, pneumonia and yellow fever.	129
Oyo-Ita et al. 2020	Oyo-Ita et al. 2020	Positive perception of vaccination	It was found from the baseline qualitative study that respondents were generally knowledgeable about and had a positive attitude towards vaccination. They believed vaccines prevented their children from acquiring deadly infections and attributed low numbers of deaths of children to vaccination	31
Socio-economic characteristics				

For peer review only - http://bmjopen.bmj.com/site/about/guidelines.xhtml

Andersson et al. 2009	Cockroft 2009	Importance of maternal education	Mother's education was related to measles vaccination in all four districts, in urban and rural sites.	10
Social norms				
Pramanik 2020	Pramanik 2020	Household decision-making	Key informants further mentioned that in rare cases, it was seen that presence of a knowledgeable daughter in law in the family compelled families to vaccinate their children	85
Availability of infrastructure, supplies or services				
Olken et al. 2014	Rahayu 2008	Availability of medical supplies	The midwife has comprehensive equipment. She has infusion equipment, blood pressure monitor, injections, medicines, scales for infants. The midwife also provides a room in the polindes for mothers who have just given birth. (Women's FGD, Kuanek, East Miomaffo, TTU, NTT)	25
Andersson et al. 2009	Cockroft 2009	Outreach by healthcare workers	in rural sites a vaccination team visiting the community also increased the likelihood that the child had received measles vaccine; this effect was much stronger in rural Khanewal.	5
Adamu et al. 2019	Adamu 2019 Dissertation	Availability of vaccination services	In this study, some caregivers indicated that recommended birth doses of vaccines are provided in the labor room as soon as the child is born. This practice saves time and reduces the chance of missed opportunities in this service delivery point.	134

For peer review only - http://bmjopen.bmj.com/site/about/guidelines.xhtml

Reasons for intervention success

Most studies with significant positive quantitative impacts provide some plausible explanation for why an intervention succeeded. These were often based on authors' notes, experiences and impressions of *why* their hypothesis was proven true. We expect the authors to be a good source of this information because of their knowledge of causal linkages and assumptions made in the intervention theory of change. Individual interviews and focus group discussions supported the authors' conclusions. The sensitivity analysis, which included qualitative papers with an assessment of score of 20 or higher, showed similar results. Given this, we consider the evidence to be of high strength.

Overall, reasons for intervention success were consistently attributed to specific *intervention features* across all engagement types. These included community engagement, leadership and supportive supervision, customisation to local context, incentives, health system integration and other design features. Though not as common, success was also attributed to *existing or changing favourable characteristics* within a given context such as availability of and access to good quality health services as well as positive participant views with respect to the intervention (Supplementary Figure 90; Supplementary Table 9).

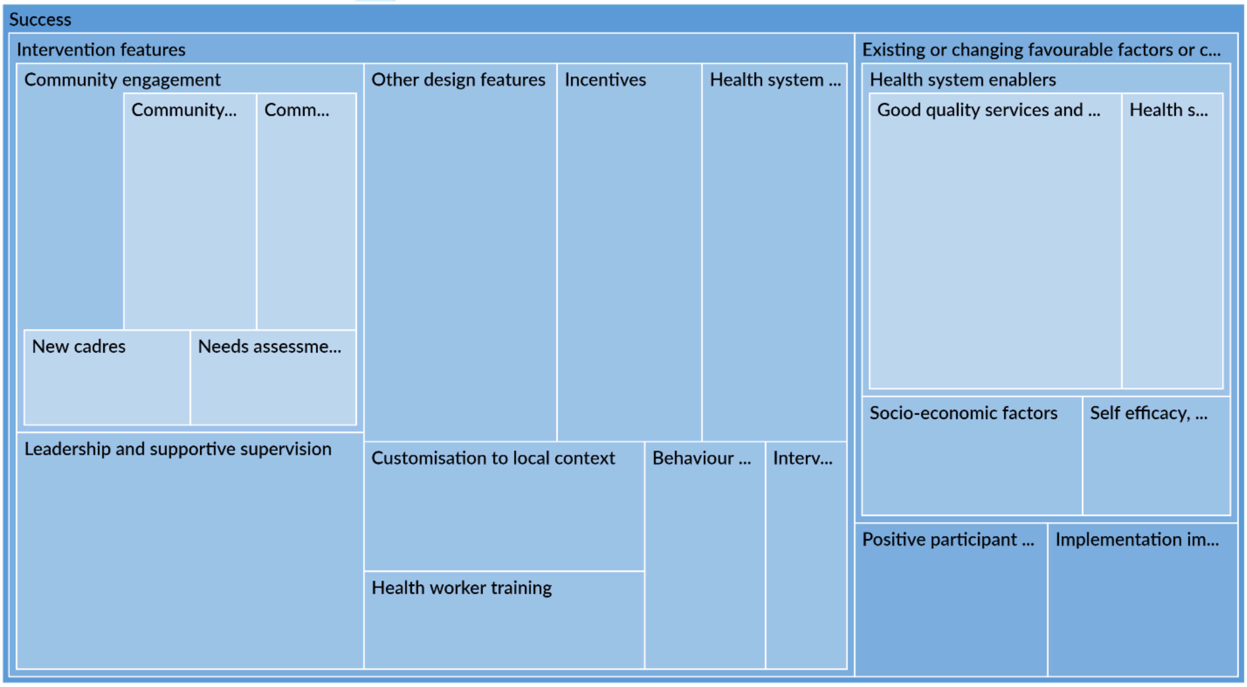

Supplementary Figure 90: Reasons for success: a summary figure

The size of the rectangles reflects the number of times a unique theme was mentioned by an IE and its associated papers. Larger rectangles indicate that a theme was mentioned more times. Nested boxes reflect the proportion of times the main theme text also fell into the sub-theme.

Supplementary Table 9: Comparison of the reasons for intervention success by type of community engagement.

For peer review only - http://bmjopen.bmj.com/site/about/guidelines.xhtml

	**Community engagement as the intervention**	**Community engagement in design**	**Community engagement in implementation autonomy of interventions**
**Intervention features –** *community engagement*	Community dialogues<br>Creation of new cadres	Community dialogues<br>Needs assessments	Community involvement in planning & implementation
**Intervention features –** *Leadership & supportive supervision*	Encouragement from leaders	Supportive supervision	Involvement of leadership<br>Health worker autonomy
**Intervention features –** *Health system integration*	Involvement of government in implementation	Separate workforce dedicated to the intervention	Intersectoral integration of health programmes
**Intervention features –** *Health worker training*	Training on monitoring health cards of children	Training of all health personnel involved in the intervention	Training of all health personnel involved in the intervention
**Favourable contextual factors –** *Health system enablers*	Good quality of services<br>Health service access	Good quality of services	Good quality of services
**Favourable contextual factors –** *Socio-economic factors*	Female literacy	General literacy levels<br>High maternal education	Primary schooling of girls and women
**Improvements in intervention Implementation over time**	-	Health worker performance improvement over time	Resolution of fidelity/adherence issues
**Positive participant/ beneficiary views**	Caregiver perception	Health worker perception	Health worker perception

.

For peer review only - http://bmjopen.bmj.com/site/about/guidelines.xhtml

Community engagement as the intervention

Studies in which engagement was the intervention consistently attributed success to characteristics associated with intervention features and relatively less consistently to existing or changing favourable characteristics within a given context.

Notably, success was attributed to *intervention features* associated with community engagement such as community dialogues which were participatory in nature and improved relationship between the health system and communities (Assegaai 2018 (Padayachee 2013); Findley 2013). Involvement of community members such as traditional and religious leaders in planning and implementation of an intervention also came up as a reason for success (Oyo-Ita 2020). In a few instances, success was also attributed to acceptance of health worker cadres by the communities as long as these health workers belonged to the communities they served (Biemba 2016).

Studies also attributed intervention success to non-engagement *intervention features* including incentives given to caregivers, leadership and supportive supervision which improved overall health service delivery and health worker performance. As noted above, some studies also cited *existing or changing favourable characteristics within a given context* as reasons for success. Among these, those related to the health system, particularly, good service quality (Rao 2016) and, to a lesser extent, access to services (Banwat 2015 (Banwat 2014)) were consistently reported. Interestingly, none of the studies in this engagement category mentioned *implementation improvements* as a reason for intervention success.

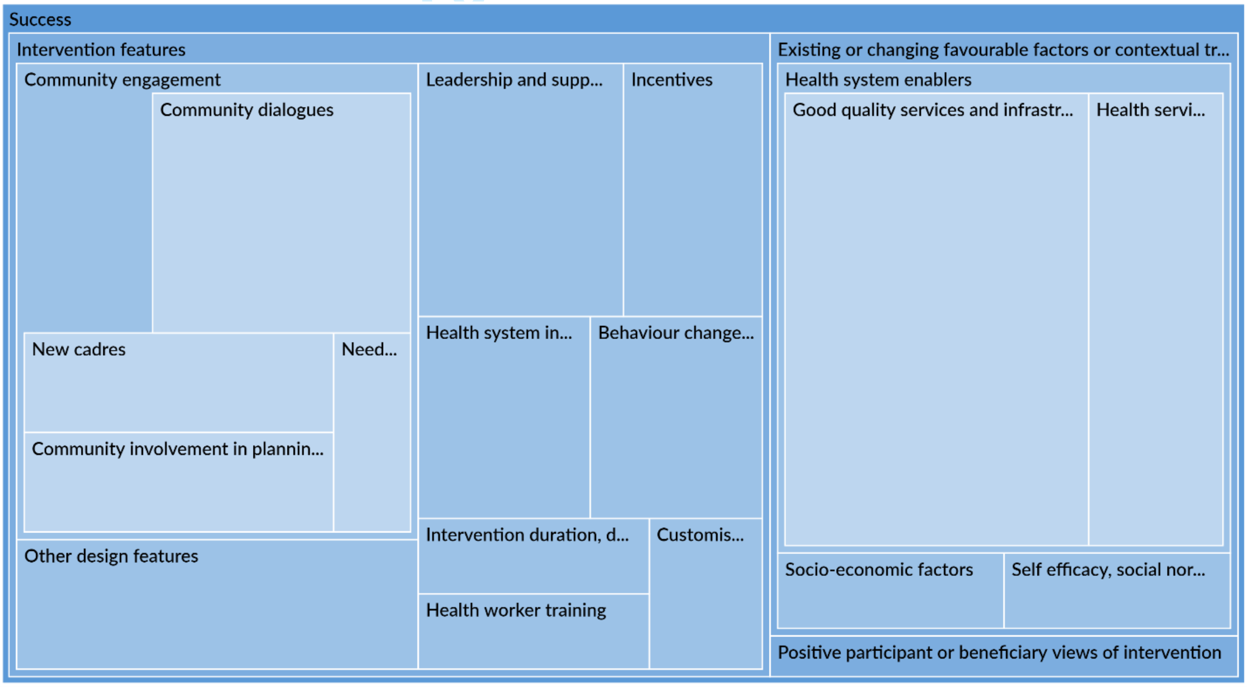

Supplementary Figure 91: Reasons for success of interventions with engagement as intervention

Supplementary Table 10: Characteristic quotes of themes identified related to reasons for success for interventions were community engagement.

Primary paper	Paper from which citation comes	Sub-theme demonstrated	Quote	Page number

For peer review only - http://bmjopen.bmj.com/site/about/guidelines.xhtml

Intervention features

Assegaai 2018	Padayachee 2013	*Community dialogue*	Interviewees spoke of the tangible benefits of the community dialogues and proactive household approaches as expanding access, improving relationships with communities, and increasing knowledge and uptake of services.	76
Oyo-Ita 2020	-	Community involvement in planning and implementation	Inclusion of TRLs in the planning, implementation and evaluation of an intervention is useful in ensuring support from the community.	42
Biemba 2016	Phiri 2017	Leadership and supportive supervision	CHAs reported that supervision by experienced health workers was valuable to them because it reinforced skills they learned in training and provided general encouragement. This study finds that the quality and frequency of supervision is heavily influenced by the proximity of supervisors' work station to that of the CHAs.	5
Banerjee 2020	-	Incentives	Most primary caregivers expressed positive opinions about recharges. Some admitted to finding them motivating or influencing their decision to immunise their child (whether or not they actually received them). This was linked to generally liking free things ('What's not to like?'), their self-assessed poverty level, or their trust in the government 'investing in them'.	53

For peer review only - http://bmjopen.bmj.com/site/about/guidelines.xhtml

Existing or changing favourable factors or contextual trends

Rao et al. 2014	Rao et al. 2016	Good quality of services and infrastructure	Moreover, the ASHA worker is envisioned not to work in isolation but as an integral part of the public health system, working together with other health personnel to both ease their work-pressures and assist them in performing their duties more efficiently. For instance, the ASHA worker works with the ANM and the anganwadi worker to organize monthly village health days. Therefore, the extent to which the increase in information provision via other health workers is attributable to the ASHA worker is not quantifiable in this context.	25
Findley et al. 2013	-	Good quality of services and infrastructure	simultaneous improvement in the quality of care provided by the CHW and nurse-midwives at the health post gave women the confidence that they could go to the health post to seek advice and care.	10
Banwat 2015	Banwat 2014-	Health service access and availability	A child was also found to be most likely to be fully and timely immunized if he/she lived within 30 minutes walking distance from the health facility.	66

Community engagement in design

The most consistentreasons for success were related to *intervention features* with the most reported characteristics being community engagement and provision of incentives to caregivers. Within community engagement, participatory community dialogues (Andersson 2009), stakeholder consultations on intervention design (Modi 2019) and community involvement in planning and implementation (Adamu 2019) were associated with intervention success. Receiving incentives was perceived to be motivating and empowering by the intended beneficiaries (Banerjee 2020; Nagar 2020).

For peer review only - http://bmjopen.bmj.com/site/about/guidelines.xhtml

Besides community engagement and incentives, o*ther intervention features* that were consistently reported included leadership and supportive supervision, health worker training and customisation to local context.

Very few studies discussed or attributed success to reasons other than intervention features. Nevertheless, the few studies that did, referred to *existing or changing favourable contextual factors* such as a high prevalence of maternal education (Johri 2020) or *improved implementation* by resolution of issues related to adherence (Modi 2019). A few studies also discussed *positive participant or beneficiary views* that could have potentially contributed to intervention success.

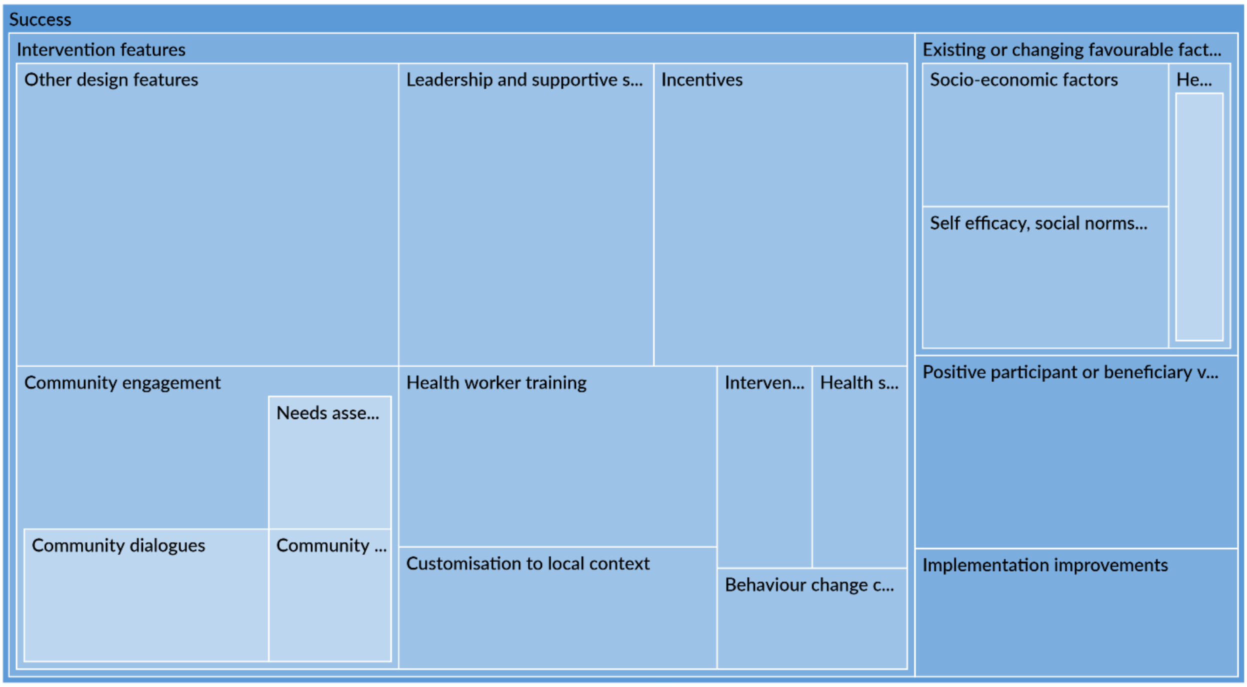

Supplementray Figure 92: Reasons for success of interventions with engagement in design

Supplementary Table 11: Characteristic quotes of themes identified related to reasons for success for interventions that used community engagement in their design.

**Primary paper**	**Paper from which citation comes**	**Sub-theme demonstrated**	**Quote**	**Page number**
Intervention features				
Andersson 2009	-	Community dialogue	The structured discussion rounds sometimes led to action plans in the intervention communities beyond stimulating discussion about vaccinations within households. Particularly in those villages with poor access to vaccination services, plans included sharing transport to vaccination points and providing care for some children while parents took others to be vaccinated. These community initiatives may have helped to maintain vaccination levels in the face of generally falling levels.	8

For peer review only - http://bmjopen.bmj.com/site/about/guidelines.xhtml

Adamu 2019	Adamu 2019 CFIR	Leadership and supportive supervision	Participants expressed satisfaction with the supervisory plan that was put in place for the quality improvement program as it enabled quick feedback. Supervisory visits were conducted by different stakeholders that are higher-ranking officials within the health systems. These include local government and zonal primary health-care management board officials. "We receive supervision from local government, they use to come and supervised us to check how we conduct our duties."– PHC 3	468
Assegaai 2018	Khuzwayo 2017	Referral to caregivers	Referrals to clinics by the teams were regarded as an important aspect of bringing services closer to the communities. Respondents said that they would not have gone to the clinic had they not been referred by the WBOT, for example for deworming of children, or continuation of care for complicated cases.	3
Existing or changing favourable factors or contextual trends				
Banerjee 2020	-	Self efficacy, social norms and awareness	By and large the main, explicitly stated driver for primary caregivers to get their child vaccinated is their positive perception or attitude toward immunization: that immunization will benefit their child. This acts as a push even for primary caregivers who have limited knowledge, who don't understand exactly how or why immunization is beneficial. Some of these primary caregivers hold strong personal conviction of the importance of immunization, despite their lack of knowledge, while others are undecided or unsure about the importance of immunization, but are still overall positive. In both cases, it seems that the positive perception or attitude is influenced by others: what others say and do. This is often mediated by trust: either trust in government and authority (e.g. ANM), household or family members, other primary caregivers in the community, or people in positions of power in the community (e.g. religious leaders in Mewat). Interestingly, improved knowledge and awareness was stated by a number of respondents as a key factor to convince primary caregivers (themselves, or others) to attend immunization camps. This demonstrates, as was mentioned in response to ANM performance, that	Appendix K – qualitative findings

For peer review only - http://bmjopen.bmj.com/site/about/guidelines.xhtml

			primary caregivers value information about vaccines	
Gibson et al. 2017	-	Socio-economic factors	The success of SMS reminders to elicit a behaviour is multifactorial; the content of the message, the type of behaviour being reminded, indirect and direct costs incurred, literacy level, and other contextual factors all being potential explanatory factors.	e436
Implementation improvements				
Modi 2019	-	Operational improvements	Finally, we identified critical operational requirements that improved adherence to the intervention; this included supportive supervision, timely resolution of technology problems, and change management, including monetary and/ or nonmonetary incentives depending on the context.	20

Community engagement in implementation autonomy of intervention

Similar to the previous two engagement categories, reasons for success in interventions with engagement in implementation were also consistently attributed to certain *intervention features*. These characteristics were associated with certain aspects of *community engagement* itself such as conducting stakeholder consultations (Modi 2019) or involvement of community members such as health workers in intervention planning and implementation (Adamu 2019). Other aspects of intervention features such as *integration with the health system, customisation to the local context* and *leadership and supportive supervision* were also consistently cited.

Like the engagement in design category, crediting intervention success to *existing or changing favourable contextual factors* or *improved implementation* was less consistently reported among studies in which community engagement was involved in the implementation of the intervention. Nevertheless, *health system enablers* such as good quality of services and access to health services were mentioned as factors which could have contributed to intervention success.

For peer review only - http://bmjopen.bmj.com/site/about/guidelines.xhtml

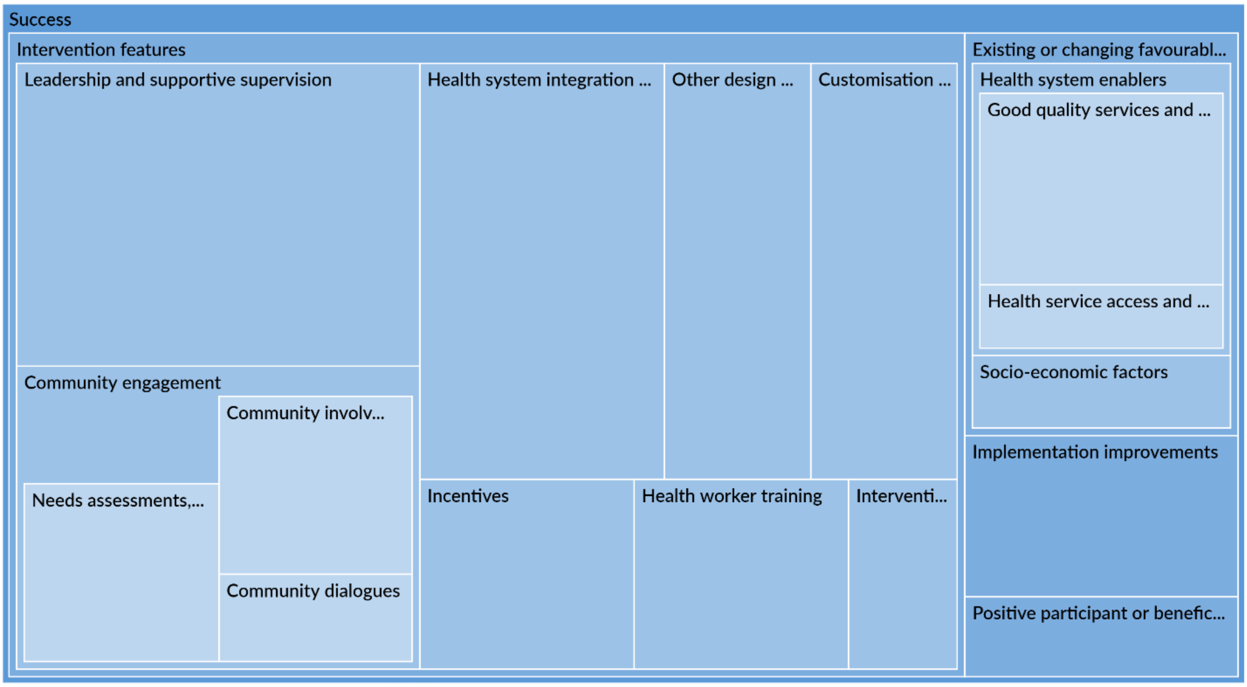

Supplementary Figure 93: Reasons for success of interventions with engagement in implementation autonomy

Supplementary Table 12: Characteristic quotes of themes identified related to reasons for success for interventions that used community engagement in implementation.

**Primary paper**	**Paper from which citation comes**	**Sub-theme demonstrated**	**Quote**	**Page number**
Intervention features				
Adamu 2019	-	Community involvement in planning and implementation	Stakeholders were systematically involved in the planning and execution of the QI program. The change ideas were selected by frontline health workers and they tailored them to their local context. Also, change ideas are multi-faceted and multimodal, and this is suitable for addressing complex problems in complex health systems.	9
Sankar 2013	-	Needs assessments, pilots or stakeholder consultations	The project interventions were developed in keeping with the especial needs of the population, and rooted in the conditions and circumstances of the area. It drew heavily from the understanding and opinions of local people. The results reflect the fruits of a highly contextualised program.	40

For peer review only - http://bmjopen.bmj.com/site/about/guidelines.xhtml

Shukla 2018	Anwari 2015	Leadership and supportive supervision	District Health Offices and DHCCs were less well established compared to the Provincial Public Health Directorates and PPHCCs; the ministry did not have adequate resources to equip them well. Despite these challenges, the leadership and involvement of the ministry in the intervention mattered. The provincial and district health governance leaders were inspired to improve their governance because the ministry leaders were interested in the pilot intervention.	14
Existing or changing favourable factors or contextual trends				
Admassie 2009	-	Good quality services and infrastructure	The impact of the programme on the proportion of children who got vaccines against<br><br>major childhood illnesses also varies according to supply side variables (see Table 6). The programme has larger effects in villages with one HEW and with better quality health posts.	441
Herrera-Almanza and Rosales-Rueda 2018	-	Health service access and availability	However, the fact that we find results on vaccination uptake is an indication that the health workers were somehow present in remote areas.	29
Admassie 2009	-	Socio-economic factors	This implies that encouraging and supporting primary schooling for girls and women will enhance the programme's impact on proportion of children vaccinated against major childhood illnesses.	441
Implementation improvements				
Mayumana 2017	Binyaruka 2017	Managing vaccine stockouts	The verification system under P4P also meant that district supervision was intensified, providing more opportunities for district managers to identify and address stock-outs of a wider range of drugs.	97

For peer review only - http://bmjopen.bmj.com/site/about/guidelines.xhtml

Reasons for intervention failure

Most studies that did not find a significant positive impact attributed the failure of the intervention to a specific cause. The most consistently reported reason for failure across all engagement types was *not accounting for contextual constraints*, though the exact nature of these reasons varied. Social norms such as misconception regarding immunisation and health worker attrition were reported as common challenges in interventions that were community engagement. Interventions that used engagement in design consistently reported poor access to health services and caregivers' skepticism regarding benefits of immunisation as the reasons for failure. Poor quality of health services was noted as an issue in interventions where engagement occurred in implementation.

The other consistently reported reasons for failure were attributed to inadequate *intervention features* and *implementation challenges.* Among the intervention features, inadequate duration, frequency or exposure to the intervention were the most notable reasons for failure across all engagement types. Though relatively less common, the nature of the community engagement itself was also attributed to failure in interventions that used engagement in implementation or as intervention. Among implementation challenges, disruption due to inadequate implementation instructions, difficulty in accessing the implementation sites due to geographic proximity and competing priorities of health workers were noted as some of the prevalent challenges (Supplementary Figure 94; Supplementary Table 13).

Another less consistently reported reason for failure was study design issues such as contamination between treatment and control other methodological challenges such as biased sampling frame and issues with enrolling study populations. The reasons for failure were largely based on authors' notes or experiences and individual interviews. We expect the authors to be a good source of this information because of their knowledge of causal linkages and assumptions made in the intervention theory of change. Focus group discussions and implementers' experiences supported these findings. The sensitivity analysis, which included qualitative papers with an assessment of score of 20 or higher, showed similar results. Except for one sub-theme which was dominated by one or two studies, other sub-themes were consistently distributed across the evidence base (indicated in Supplementary Table 13). Therefore, we consider the evidence to be of high strength.

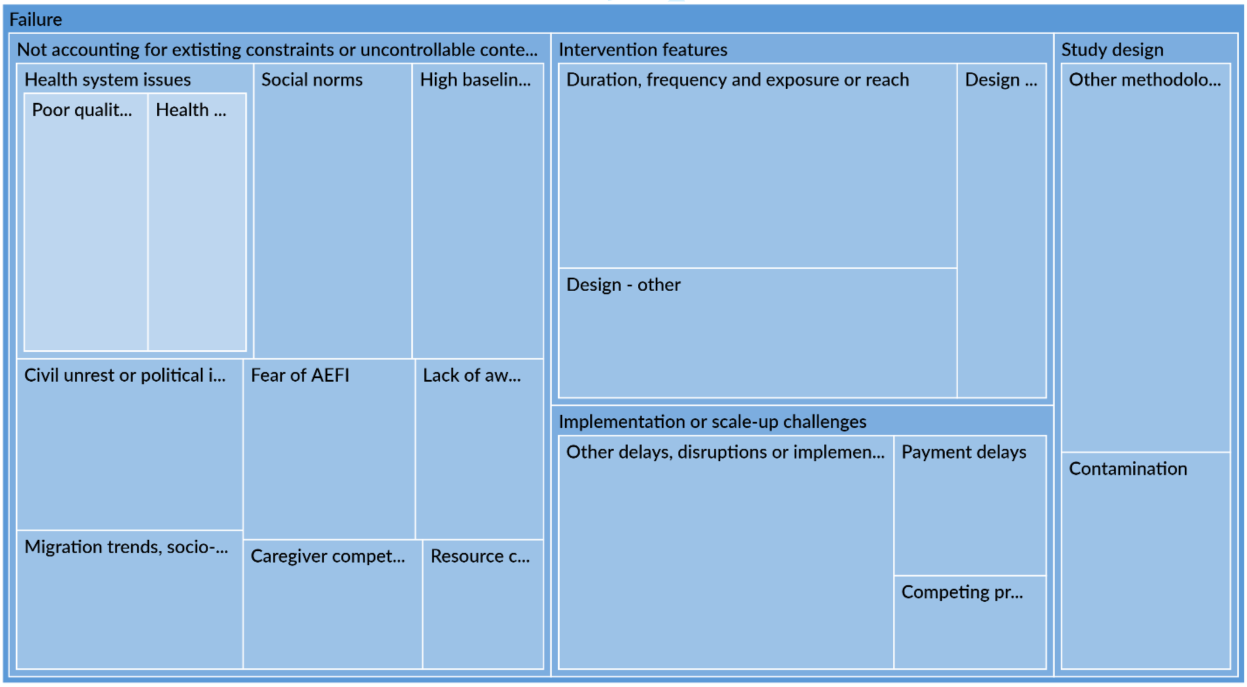

Supplementary Figure 94: Summary figure: reasons for failure

Supplementary Table 13: Comparison of the reasons for intervention failure by type of community engagement**.**

	**Community engagement as the intervention**	**Community engagement in design**	**Community engagement in implementation autonomy of the intervention**
**Not accounting for contextual constraints –** *health system issues*	Health worker attrition	Issues with accessing health services	Poor quality of services
**Not accounting for contextual constraints –** *prevalent social norms*	Fear of sterilization	Skepticism towards perceived value of immunisation	-
**Not accounting for contextual constraints –** *baseline immunisation coverage*	High baseline coverage	High baseline coverage	High BCG coverage
**Not accounting for contextual constraints –** *unrest, insecurity or other*	Civil unrest<br>Natural calamity	Political instability	-
**Intervention features –** *Duration, dose or frequency of intervention*	Inadequate intervention duration or exposure	Inadequate intervention frequency	Inadequate intervention duration or exposure
**Intervention feature –** *issue in design of engagement*	Lack of collective action	-	Elite capture
**Implementation challenges –** *disruptions or delays*	Resource constraint due to multiple programmes running at the same time	Fidelity issues	Staff turnover*
**Study design –**	Contamination due to similar programmes	Contamination due to similar programmes	Contamination between treatment and control

For peer review only - http://bmjopen.bmj.com/site/about/guidelines.xhtml

Contamination			

*Most of the references coded under this theme were from Olken 2014 and associated papers.

Community engagement as the intervention

Not accounting for contextual trends were the most consistently reported reason for failure. Social norms, health system issues, migration, civil unrest and fear of adverse events following immunisation were reported consistently within existing constraints. Most of the challenges related to social norms mentioned misconception about immunisation (Banerjee 2020) and limited decision-making powers of mothers (Gurley 2020) as the reason for low immunisation uptake. Civil unrest, political instability and natural calamities also hindered a few interventions from achieving intended impacts (Pramanik 2020; Morris 2004).

The other reasons for failure comprised issues related to *intervention features, implementation issues* and, to a lesser extent, *study design.* Issues with intervention feature ranged from inadequate duration or frequency of the intervention to certain aspects of community engagement failing to elicit a positive response among study participants (Pramanik 2020; More 2012). Implementation issues were consistently reported as reasons for failure. Some of the challenges mentioned were competing demands by different programmes running simultaneously (Carnell 2014), high staff turnover (Gurley 2020; Olken 2014) and low implementation fidelity (Oyo-Ita 2020). These issues are also discussed in detail under the 'uptake and fidelity challenges theme'.

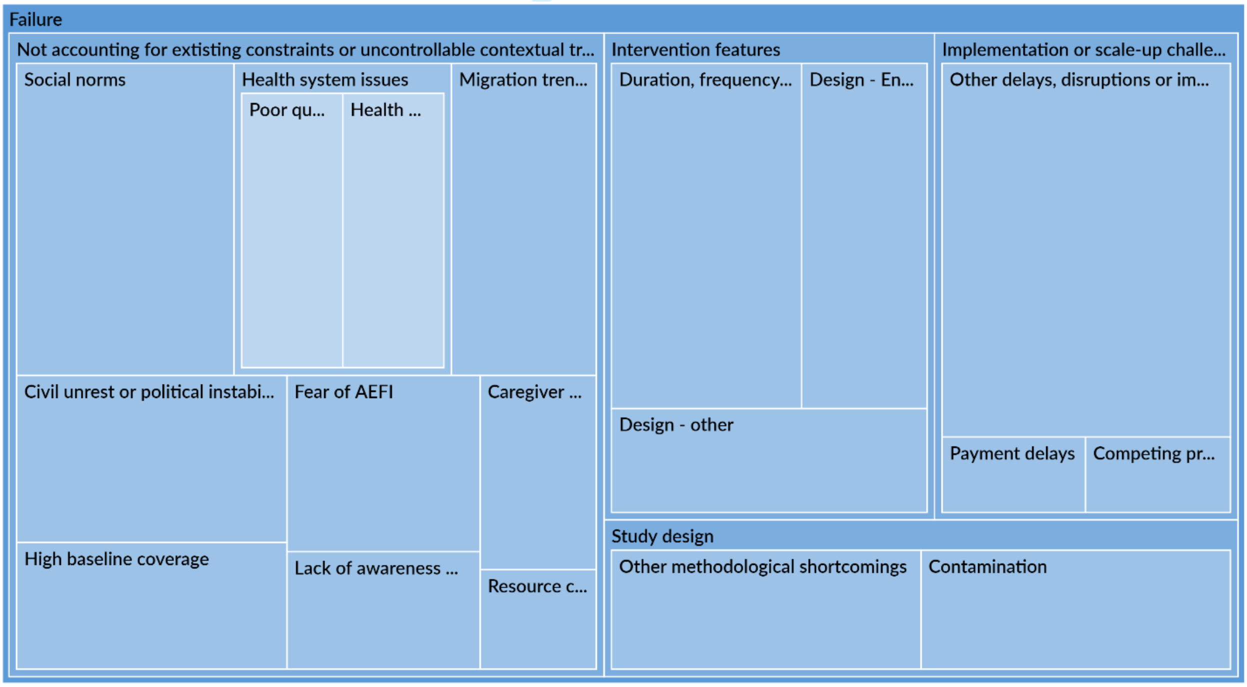

Supplementary Figure 95: Reasons for failure of interventions with engagement as intervention

Supplementray Table 14: Characteristic quotes of themes identified related to reasons for failure for interventions were community engagement.

For peer review only - http://bmjopen.bmj.com/site/about/guidelines.xhtml

Primary paper	Paper from which citation comes	Sub-theme demonstrated	Quote	Page number
Implementation or scale-up challenges				
Carnell 2014	-	Attrition of implementation personnel	The ESHE and RHB teams both faced personnel challenges in Amhara, which may have affected management and performance.	559
Gurley 2020	-	Infrastructure constraints	Community constraints around screening spaces and economic activities also introduced challenges.	73
Not accounting for existing constraints or uncontrollable trends				
More 2012	-	Lack of access to health care	The third issue was the complexity of urban health care. Antenatal care was the norm and the nadir for institutional delivery in trial clusters was 75%. Around 57% of antenatal care and 30% of deliveries were in the private sector (this in a slumdwelling population). Open access to private providers, and to institutions at all levels of the public sector hierarchy, is a challenge to systematic health care delivery. Our findings confirmed the tendency to bypass public maternity homes, which should handle uncomplicated deliveries, in favour of tertiary institutions.	E1001257
Pramanik 2020	-	Natural calamity	Assam has a wonderful collection of festivals that are of the utmost importance. And more tragically when the Brahmaputra River floods the priority is survival. The project simply has to adapt to these ebbs and flows, but there is a pressure to deliver to a schedule that inevitably leads to a box ticking approach that is inconsistent with the development of ownership by the community.	68

For peer review only - http://bmjopen.bmj.com/site/about/guidelines.xhtml

Oyo-Ita 2020	-	Fear of AEFI	These responses are indications that fear of side effects can hinder vaccine uptake. This may have contributed to non-impact on the proportion of fully vaccinated children in this study, as the TRLs in the post-intervention qualitative study still mentioned this as a common reason for poor uptake of vaccines.	32
Intervention features				
Olken 2014	Grayman 2013	Engagement failure due to elite capture	In general, researchers discovered that Generasi provides opportunities for elites to retain and fortify their social standing. As brokers delivering CDD resources into their communities, local elites leverage Generasi to accumulate additional status for themselves. Without exception, every Generasi actor that the researchers met held other leadership roles in their community and maintained close ties with the local structures of village and sub-district governance.	48
Robertson 2013	-	Inadequate exposure to the intervention	Our study was limited by the short intervention period. Whether the effects of the programmes would change with time is unclear. The follow-up survey was done 2 months after interventions had finished, so fear of penalties should not have biased responses from CCT households, although the effects of the programmes could have attenuated by the time of the survey.	8
Study design				
More 2017	-	Contamination due to other programmes	although we found no evidence of contamination of control clusters by the intervention, government schemes and the activities of municipal and non-governmental providers might have improved health in control clusters.	E347

Community engagement in design

Most consistently reported reasons for failure of interventions in which communities were engaged in the design broadly fell under *not accounting for contextual constraints.* Among these, social norms were the most consistently reported reason with vaccine hesitancy being one of the driving forces behind immunisation refusal

For peer review only - http://bmjopen.bmj.com/site/about/guidelines.xhtml
For peer review only

(Banerjee 2020; Gurley 2020). Other reasons for failure within this theme were high baseline immunisation coverage, health system issues related to quality of services, and civil unrest or political instability. Prevailing socio-economic trends, lack of awareness, fear of AEFI and caregiver competing priorities were also attributed to intervention failure, though less consistently reported as compared to other factors.

Besides existing constraints, a few studies also attributed failure *intervention features* with the insufficient duration, frequency and exposure of intervention as a possible reason for failure. A much smaller subset of studies also referred to issues related to study design and implementation or scale-up challenges.

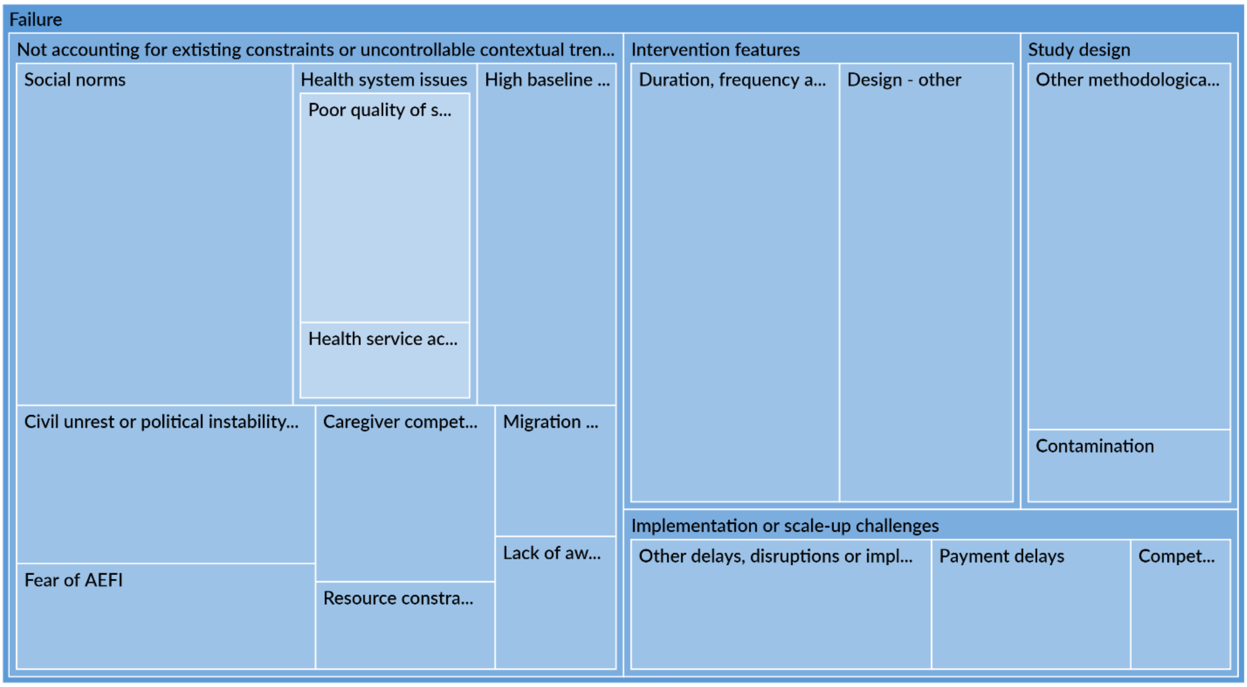

Supplementary Figure 96: Reasons for failure of interventions with engagement in design

Supplementary Table 15: Characteristic quotes of themes identified related to reasons for failure for interventions that used community engagement in their design.

**Primary paper**	**Paper from which citation comes**	**Sub-theme demonstrated**	**Quote**	**Page number**
Implementation or scale-up challenges				
Gurley 2020	-	Staff turnover	On the implementer side, staff turnover at NYST and research activities introduced delays in video production.	73
Engineer 2016	-	Payment delays	Problems with implementation likely dampened any potential effect. The scheme was rolled out in phases, but there were some delays, particularly with the initial payments.	456

For peer review only - http://bmjopen.bmj.com/site/about/guidelines.xhtml

Not accounting for existing constraints or uncontrollable trends

Gibson 2017	-	High baseline immunisation coverage	SMS reminders were probably not effective at improving full immunisation and vaccine-specific coverages in this study because of high baseline coverage levels and because SMS reminders might not have addressed the demand side deficiencies in this study area.	e436
Banerjee 2020	-	Caregiver competing priorities	Across districts, there are 'hard-to-convince' populations who are not affected by incentives. Some ANMs listed specific populations groups they felt continued to be unaffected by the programme, and the incentives specifically, such as migrant workers, daily wage workers and the Muslim community. In Panipat, the top reason given by ANMs for resistance to immunisation by the few still unconvinced (migrants, Muslims) was the fever brought on as an after-effect by a certain vaccine (penta)30 and direct costs involved for daily wage workers.	92
Gurley 2020	-	Social norms	It is worth noting that in contrast to the high intent recorded through the household survey, the process evaluation indicated there were individuals who actively chose to not vaccinate their children. This may be a function of social desirability bias in the household survey, though we posit that it may also reflect family constraints or logistical barriers that prevent a mother from taking a vaccine decision regardless of own personal intention to vaccinate. As noted elsewhere, mothers are not the sole or primary decision-makers and other family members reported lower intent to vaccinate.	75

Intervention features

Siddiqi 2020	-	Inadequate exposure to the intervention	Our findings also provide evidence of the strong reliance on the immunization card being the established immunization recall method and we may also postulate that the short duration of the study did not provide enough time to 'institutionalize' the use of the bracelets.	12

For peer review only - http://bmjopen.bmj.com/site/about/guidelines.xhtml

Okoli 2014	Baba-Ari 2018	Communication failure	Experiences of the beneficiaries with the programme point to problems with programme communication as they had a poor understanding of the aims of the CCT programme aside from receiving its immediate cash benefits and as such might revert to their original behaviours after the programme ends.	940
Study design				
Domek 2019	-	Selection bias in study enrollment	This may have been partly because our study population had higher baseline immunization coverage than we were adequately powered to assess. These higher than expected completion rates were likely due in part to a selection bias in enrolling children as they presented for their first immunization visit, which would have unintentionally excluded children who either presented significantly delayed or not at all for vaccines.	6197

Community engagement in implementation autonomy of interventions

Fewer studies under this engagement classification reported reasons for failure as compared to success. Among the reasons cited for failure, those related to *not accounting for contextual constraints* were the most consistently reported followed by *implementation challenges*. Most of the references to implementation challenges or failures were made by Olken 2014 and its associated studies which discussed multiple implementation barriers to a block-level grant intervention. Though less consistently reported, some studies also mentioned issues with intervention features or study design as the reasons for intervention failure. Highlighting the failure of community engagement, Olken 2014 (Grayman 2013) discussed how elite capture wherein local community leaderships and elites leveraged the intervention to retain social standing became an issue for the programme.

For peer review only - http://bmjopen.bmj.com/site/about/guidelines.xhtml

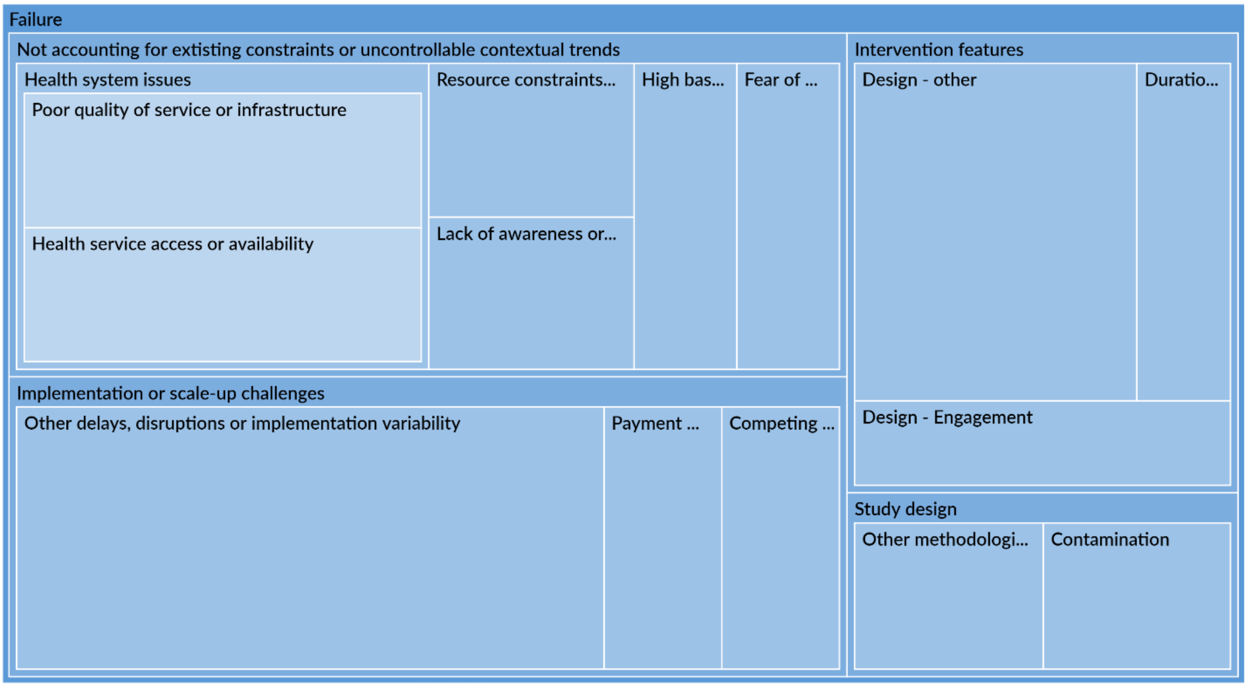

Supplementary Figure 97: Reasons for failure of interventions with engagement in implementation autonomy

Supplementary Table 16: Characteristic quotes of themes identified related to reasons for failure for interventions that used community engagement in implementation.

**Primary paper**	**Paper from which citation comes**	**Sub-theme demonstrated**	**Quote**	**Page number**
Implementation or scale-up challenges				
Oyo-Ita 2020	-	Low implementation fidelity	The non-impact on up-to-date vaccination could have been accounted for by the weak link in the intervention caused by not sharing data directly with community members as planned.	38
Olken 2014	World Bank 2018	Staff turnover	High village-level staff turnover may partially explain why Generasi staff in the villages are so unfamiliar with the bonus system. The entire Generasi team in Desa Lelaok (Petis subdistrict, Pamekasan district) and the PK in Desa Rampe (Nelle subdistrict, Lembata) had never heard of the bonus system, but all of them had started within the past year.	38

For peer review only - http://bmjopen.bmj.com/site/about/guidelines.xhtml

Webster 2019	-	Competing priorities of health workers	Although the proportion of respondents reporting a home visit by a VHT decreased from baseline to endline in both control and intervention clusters the proportion of visits where immunization was the reason for the visit increased. The decrease in VHT home visits may be due in part to observations that in the latter months of the intervention, VHT services were sought by other organisations involved in the refugee response in some of the project areas, who paid VHTs higher allowances.	47
Not accounting for existing constraints or uncontrollable trends				
Okeke 2017	-	Resource constraints or scarcity	Lack of electricity and water were also frequently cited as problems.	43
Okeke 2017	-	Poor quality of services or infrastructure	The data suggest that part of the reason why the program did not have larger impacts is that other dimensions of quality did not improve. For example, clinic infrastructure in many cases remained poor, as did availability of drugs and supplies.	49
Intervention features				
Webster 2019	-	Intervention design failure	The main mechanisms of change identified were improved accessibility to immunization services through increased numbers of vaccination outreaches, increased VHT motivation through monthly allowances together with social motivation in outdoing each other in defaulter tracing, use of community resources to achieve outcomes that is the support of local community leadership; and increased interaction between health workers and HCWs and VHTs at monthly VHT/HCW meetings. These identified mechanisms of change were predominantly due to supplementary activities that were implemented in both intervention and control clusters.	49
Study design				

For peer review only - http://bmjopen.bmj.com/site/about/guidelines.xhtml

Webster 2019	-	Contamination between treatment and control	There was some contamination of control clusters, largely due to transfer of health workers from intervention to control sites. At two control sites it was evident that the health workers sought to set up a similar defaulter tracing approach.	49

Reasons for heterogenous impacts

A small subset of studies is also coded under the theme *heterogeneous impacts*. Even though they reported overall significant positive quantitative findings, these studies discussed their heterogenous or non-uniform results with respect to not achieving the intended impact among certain subgroups, study sites or reported null results for some of the immunisation-related outcomes. Each of these studies offer a slightly different perspective on their heterogeneous results. For instance, Adamu et al. 2019 and Carnell et al. 2014 postulate that the characteristics of individuals involved in the intervention implementation, contextual factors or implementation processes could have led to partial intervention success or measurable positive gain in only select study sites. Interestingly, both Banerjee et al. 2020 and Gibson et al. 2017 highlight very different challenges with respect to their interventions comprising incentives to caregivers. Banerjee et al. describe how incentives designed to motivate caregivers did not work on Muslim minorities due to high vaccine hesitancy which stemmed from the belief that vaccination causes sterlisation. On the other hand, the Gibson study reported that their SMS reminders did not work in the absence of incentives, implying that reminders alone were insufficient to encourage behaviour change among caregivers and result in immunisation uptake. This shows that the benefit of providing incentives may not be consistent across different contexts and uptake may be influenced by other socio-economic factors. Both studies further elaborate the limits to which incentives can address deep rooted mistrust of vaccination among certain subgroups. Overall, the discussion on these studies provide important insights about how external factors, implementation variability or invalid theory of change assumptions may contribute to partial intervention success or failure. We also looked through the literature for *unintended impacts* but there was insufficient information to draw any broader conclusions.

Uptake and fidelity challenges

Uptake and fidelity challenges were generally identified by authors through primary data or personal experience and knowledge of the intervention. Because this theme relates to how the project was implemented, we expect that authors are a good source of this information. Individual interviews and focus group discussions supported the findings. Themes were broadly consistent in the full analysis and in sensitivity analysis in which on the qualitative papers with a quality assessment score greater than 20 were included. However, there are several instances in which a single sub-theme is dominated by one or two studies. These are indicated in Supplementary Table 17 and discussed in the text. Given this, we believe that the evidence on uptake and fidelity challenges has only moderate strength.

The most consistently reported challenges were related to administration, mobilization, fidelity, and contamination (Supplementary Figure 98; Supplementray Table 17). Administrative challenges were cited consistently across all three types of interventions, but their nature varied. Although technical issues and politics plagued interventions that used community engagement in the design of interventions or as the intervention, these issues were not common in interventions that used engagement in their implementation. Staffing was a challenge in interventions that used engagement in implementation or as the intervention. Implementation fidelity was mentioned as a challenge by all three types of interventions. In all cases, the most consistent issue was that realities on the ground forced changes to the intervention. The issue of mobilizing people into the intervention appears to be common; however, this category is dominated by two papers (Pereze 2020 and Olken 2014) which cite a variety of mobilization challenges. As such, the evidence on this theme is somewhat weak. Contamination was also consistently reported. For the most part, it was mentioned due to its absence. Interventions whose goal was

For peer review only - http://bmjopen.bmj.com/site/about/guidelines.xhtml

engagement discussed this topic much more than interventions with engagement in their design or implementation.

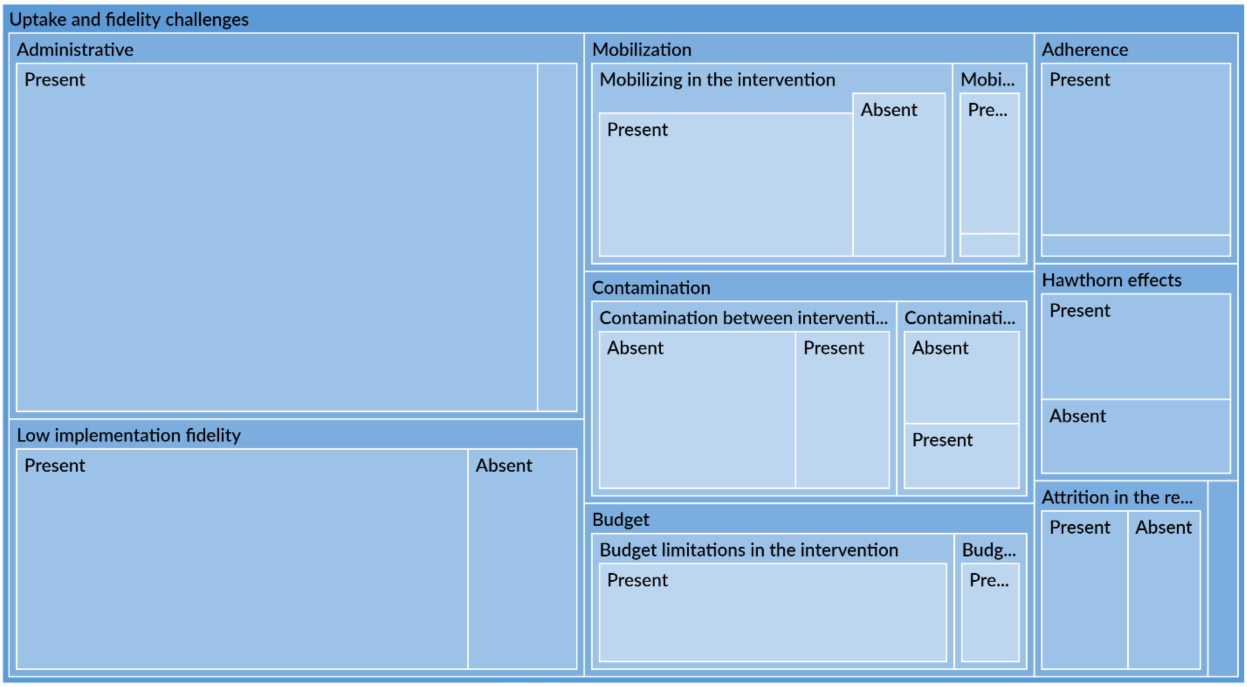

Supplementary Figure 98: Summary figure: uptake and fidelity challenges[2]

Supplementary Table 17: Comparison of the challenges faced by interventions using different types of community engagement. Numbers reflect relative frequency.

		Community engagement as the intervention	Community engagement in the design of the intervention		Community engagement in the implementation of the intervention
**Administrative challenges**		Technical issues<br>Politics<br>Coordination<br>Staffing	Technical issues<br>Politics<br>Bureaucracy<br>Security		Funding disbursement<br>Communication across stakeholders<br>Staffing
**Mobilization challenges**		Gender based religious and cultural norms prevented participation***	Recruiting people into the intervention due to work, distance, and competing priorities*		Recruiting facilitators and participants into the intervention due to other opportunities, miscommunication of intervention expectations, declines in incentives, caregiving obligations, and more**
**Fidelity challenges**		Failure to account for on the ground realities resulted in changes or failures in intervention	Failure to account for on the ground realities resulted in changes or failures in intervention implementation		Failure to account for on the ground realities resulted in changes or failures in intervention implementation

[2] Coding is presented in a present/absent format because, in some cases, authors explicitly stated that a challenge was not encountered.

For peer review only - http://bmjopen.bmj.com/site/about/guidelines.xhtml

		implementation. Improper or insufficient supervision was also common.			
**Contamination**		Authors were more likely to discuss the absence of this issue than to report upon its presence.	Authors were more likely to discuss the absence of this issue than to report upon its presence.		
**Budget restrictions**					Inadequate or delayed pay resulted in activities not being implemented.

*Most of this evidence came from a Pereze 2020 which cited multiple reasons for mobilisation challenges
**All evidence on challenges came from Olken 2014
***All evidence from Olken 2014 also applies to this group as Olken 2014 falls into both categories

Community engagement as the intervention

Discussions of *administrative challenges* consistently related to coordination, technical issues, staffing, and politics. The single study that reported on the absence of certain administrative issues also reported on the presence of many others. This is likely because it was a 3ie report, which tend to be quite detailed and include significant qualitative components.

Issues with the faithful *implementation* of these interventions were consistently the result of improper supervision and management or a failure to account for practicalities on the ground. We found one case where the administrative challenges directly led to low implementation fidelity. However, a non-trivial proportion of studies reported statistics indicating high fidelity.

The studies that discussed contamination largely did so in order to demonstrate that contamination was not a concern. Authors consistently focused on the issue of contamination between intervention and control sites. This was often addressed with vague statements. Distance was largely used to avoid contamination.

Discussion of challenges in mobilization interventions was dominated by a single source (Olken et al 2014 also discussed the engagement in implementation section). However, Rahman 2016 also discussed several mobilization issues that resulted from religious and cultural norms, largely around gender, preventing participation.

For peer review only

For peer review only - http://bmjopen.bmj.com/site/about/guidelines.xhtml

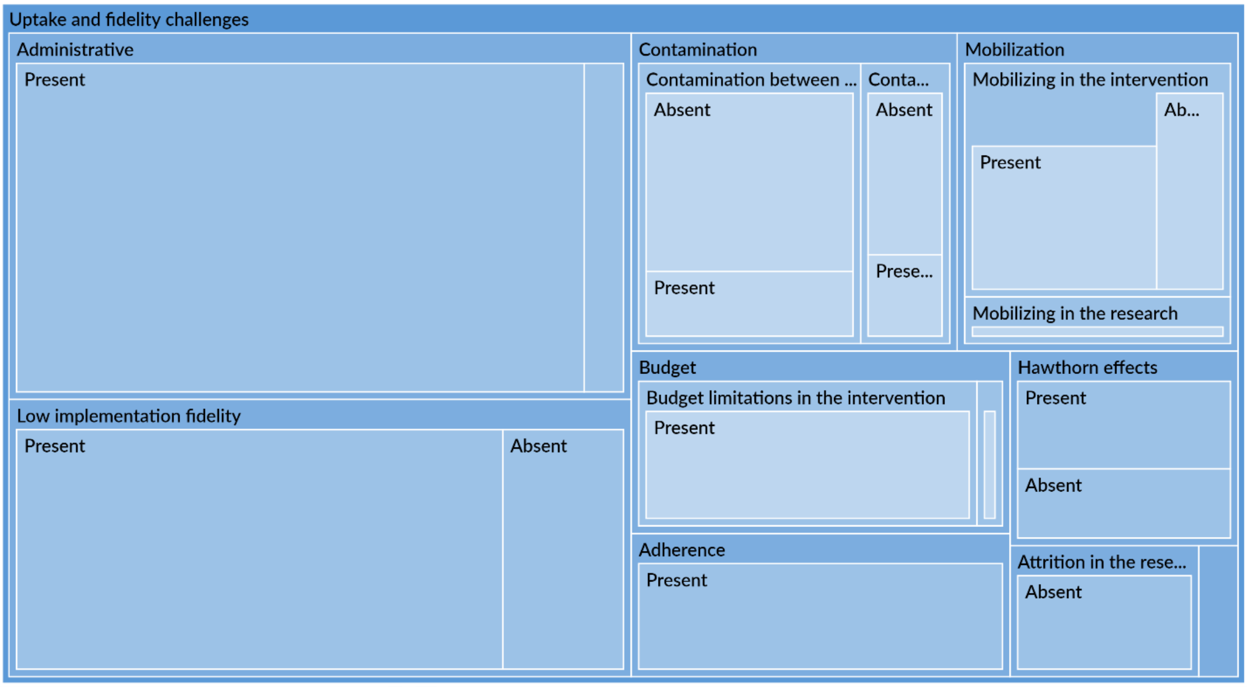

Supplementary Figure 99: Engagement as intervention

Supplementary Table 18: Characteristic quotes of themes identified related to uptake and fidelity challenges for interventions were community engagement.

Primary paper	Paper from which citation comes	Sub-theme demonstrated	Quote	Page number
*Administrative challenges**				
Assengai et al 2018	Khuzwayo et al 2017	Coordination challenges	Respondents report a lack of communication about visit scheduling.	3
			This is compounded by dual reporting lines in many parts of the country, where CHWs remain linked to and receive stipends through NGO intermediaries, while being accountable to PHC facility managers.	
Demilew et al 2019	Demilew et al 2020	Technical and political challenges	The Mobile Intervention was left from being implemented because of Internet service inaccessibility that resulted from the state of emergency declared by the Ethiopian government.	23

For peer review only - http://bmjopen.bmj.com/site/about/guidelines.xhtml

Banerjee et al 2020	Banerjee et al 2020	Political challenges	This study has faced various degrees of support from our primary government stakeholder since its inception, and this is the main reason why the timeline has been extended multiple times over the past seven years (when discussions with the Haryana government first began). We faced issues in continuity and buy-in especially when higher-ranking officers were transferred.	69
		Absence of administrative barriers	Given that J-PAL South Asia was also responsible for implementation, we recruited a dedicated team with relevant experience to do this. As a result, we were acutely aware of what was happening in the field, and able to identify and resolve issues quickly.	71
Implementation fidelity				
Biemba et al 2016	Phiri et al 2017	Inadequate supervision	Supervision and mentorship is crucial to optimizing the skills of CHAs, but this study outlines that supervision is not always implemented as intended and that in the absence of regular supervision	7
Morris et al 2004	Morris et al 2004	Expectations did not match realities on the ground	The service-level package was not implemented in accordance with protocol because no legal means could be identified of transferring resources	2034
Demilew et al 2019	Demilew et al 2020	Administrative challenges caused implementation challenges	This intervention was designed on the expectation that the text message portion could be rolled out in tandem with the ECIIN, an immunisation tracking program that planned to have HEWs report immunisation dates, mother's data, child's data, using text messages. Due to data issues and conflict in the region ECIIN was discontinued.	34
Gurley et al 2020	Gurley et al 2020	High fidelity	The types of screenings were conducted as planned, with 446 mothers group screenings, 445 VHND screenings, 223 men's screenings, and 444 HTR screenings (Table 6). Within a single village, this translated to an average of 12 sessions held in mothers groups, 12 held in VHNDs, 12 held in HTR areas, and 6 held in men's groups.	23
Contamination				
Moore et al 2017	Moore et al 2017	Absence of contamination	Our intervention and control groups were generally similar, with high coverage and fidelity to planned activities and negligible contamination.	e347

For peer review only - http://bmjopen.bmj.com/site/about/guidelines.xhtml

Paraminik et al 2018	Paraminik et al 2020	Distance reduced contamination	To mitigate potential contamination between intervention and control villages, we attempted to ensure that the intervention and control villages are sufficiently far apart from one another.	25

*Challenges related to mobilisation are not presented because this information largely came from a single paper making the identification of characteristic issues *across* papers impossible.

Community engagement in the design of the intervention

Among interventions that used community engagement in their design, the most consistently reported *administrative challenge* related to technical issues, largely surrounding phones and internet connections (Supplementary Table 17). However, other administrative challenges were related to combinations of politics, bureaucracy, and security concerns. The single paper that indicated a lack of administrative challenges reported successful syncing to the server, but also reported on other technological issues.

Mobilization challenges largely related to recruiting people into the intervention. Most of this information came from a 2020 (Pereze et al 2020) paper which presented information on the implementation fidelity and acceptability of an intervention that combined in-person and digital education to promote vaccination (Johri 2018). Authors describe challenges with participants being unable to attend due to work, distance, and competing priorities. Limited access to phones made mobilization difficult as well.

Acknowledgement of *low implementation fidelity* was consistently accompanied by discussions of why changes were made or the implications of these changes for results. Generally, changes in the intervention plan were forced due to practical constraints that had not been accounted for and were expected to result in an attenuation of impacts. Reports of high fidelity were succinct and responded to concerns a reader may be expected to have.

Contamination can occur between the intervention and control or due to ongoing activities of other programs. The former of these was reported more consistently than the latter. Authors were more likely to discuss the absence of this issue than to report upon its presence. Some offered explanations as to why contamination was not a concern or steps taken to avoid contamination. Others reported on results related to measures of contamination.

For peer review only - http://bmjopen.bmj.com/site/about/guidelines.xhtml

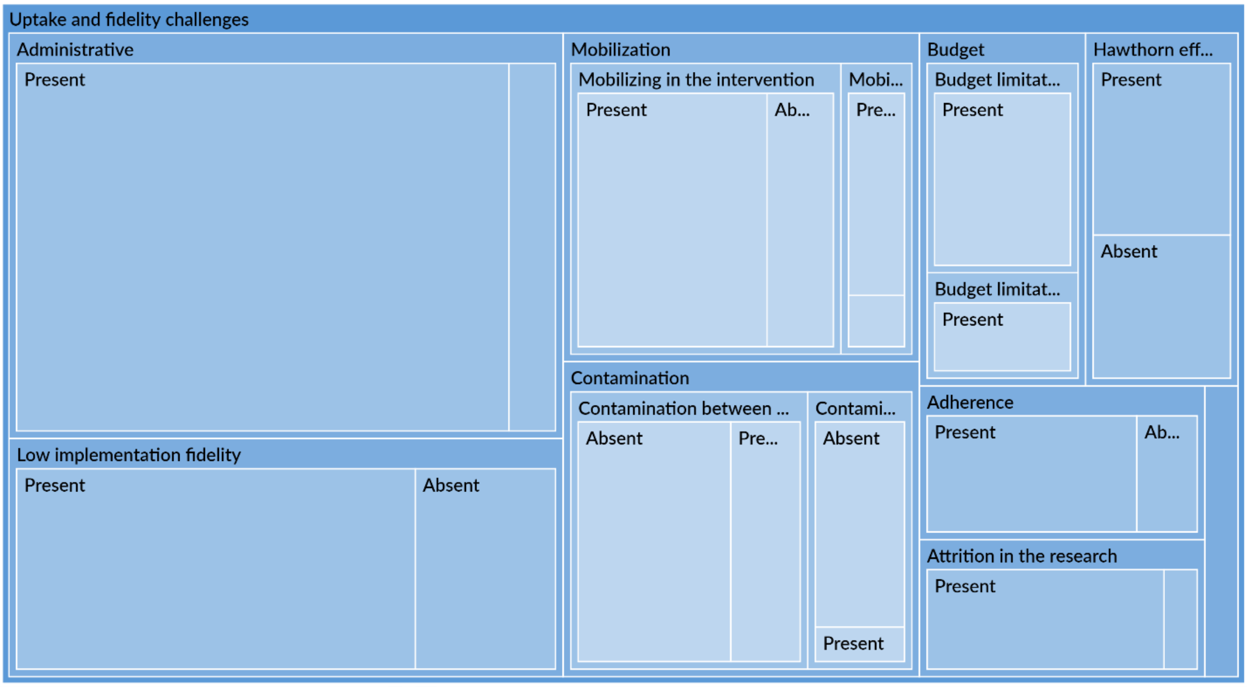

Supplementary Figure 100: Engagement in design

Supplementary Table 19: Characteristic quotes of themes identified related to uptake and fidelity challenges for interventions that used community engagement in their design

**Primary paper**	**Paper from which citation comes**	**Sub-theme demonstrated**	**Quote**	**Page number**
*Administrative challenges**				
Nagar et al 2020	Nagar et al 2020	Technological problems	The most prevalent issues reported by ANMs using the KB App included having to reenter data after an app crash (60.9%), having issues with scanning the pendant (65.2%), and issues with the application crashing (56.5%), and time required to enter data (33.3%).	138
Modi et al 2019	Modi et al 2015	Technological problems	Limited phone memory occasionally created technology-related issues, with the increasing requirement to store data on the mobile phone.	10
		Absence of technological problems	The failure rate, defined as the proportion of forms that failed to get synced with the server, was <1%.	266
Implementation fidelity				

For peer review only - http://bmjopen.bmj.com/site/about/guidelines.xhtml

Gruley et al 2020	Gruley et al 2020	Reason changes were implemented	Last, due to resource constraints, the project was not able to implement the intervention component for sharing videos from phone to phone using Bluetooth technology.	22
Engineer et al 2019	Engineer et al 2016	Implications of low fidelity for the research	Problems with implementation likely dampened any potential effect. The scheme was rolled out in phases, but there were some delays, particularly with the initial payments.	456
Banerjee et al 2010	Banerjee et al 2010	High implementation fidelity	Review of records showed that of 1336 planned camps, 95% (1269) took place.	2
Contamination				
Borkum et al 2014	Borkum et al 2014	Absence of contamination with other interventions	Because FLWs and households in the treatment and control groups received non-TBGI Ananya program interventions that were being implemented simultaneously across Begusarai, the RCT was designed to measure the value-added of TBGI beyond these other Ananya interventions. It was not designed to measure the impact of TBGI introduced in isolation.	6
Nagar et al 2018	Nagar et al 2016	Steps to avoid contamination	a cluster randomised approach further allowed non-contamination of social signaling interventions within the contacts of a given village	34
Johri et al 2018	Pereze et al 2020	Little contamination observed	According to the analysis of the records, only people who belonged to the intervention group attended the community meetings. The people who communicated with or received calls from the IVR platform were from the intervention group, except for one (1/166) control group member.	15

*Challenges related to mobilisation are not presented because this information largely came from a single paper making the identification of characteristic issues *across* papers impossible.

Community engagement in the implementation autonomy of the intervention

Administrative challenges in interventions that used engagement in their implementation largely related to funding disbursement, communication across stakeholders, staffing issues (Supplementary Table 17). One study (Bjorkman et al 2009) reported that they did not believe administrative differences affected results. This was

For peer review only - http://bmjopen.bmj.com/site/about/guidelines.xhtml

coded as the absence of an administrative challenge,but does not mean that administrative issues were not encountered.

Implementation fidelity appears to have been more of a challenge for these interventions than for those that leveraged community engagement in their design. These issues were consistently related to the intervention not functioning as expected. In some cases, this was because the reality on the ground did not reflect expectations. However, two studies that reference challenges with implementation fidelity also report upon successes in implementing according to plan.

All reports of challenges *mobilising* participants into interventions that were implemented by the community came from a paper related to an intervention that used block grants to support maternal and child health and education (Olken 2014). Challenges were encountered in the recruitment of both facilitators and participants. These related to other opportunities, miscommunication of intervention expectations, declines in incentives, caregiving obligations, and more. However, Herrara-Almanza 2018 report that community health workers felt strong responsibility for the wellbeing of their communities and attrition was low.

Interventions that used engagement in their implementation also experienced significant *budgetary constraints*. Almost universally, these constraints were the result of inadequate or delayed pay. Often, this resulted in activities not being implemented.

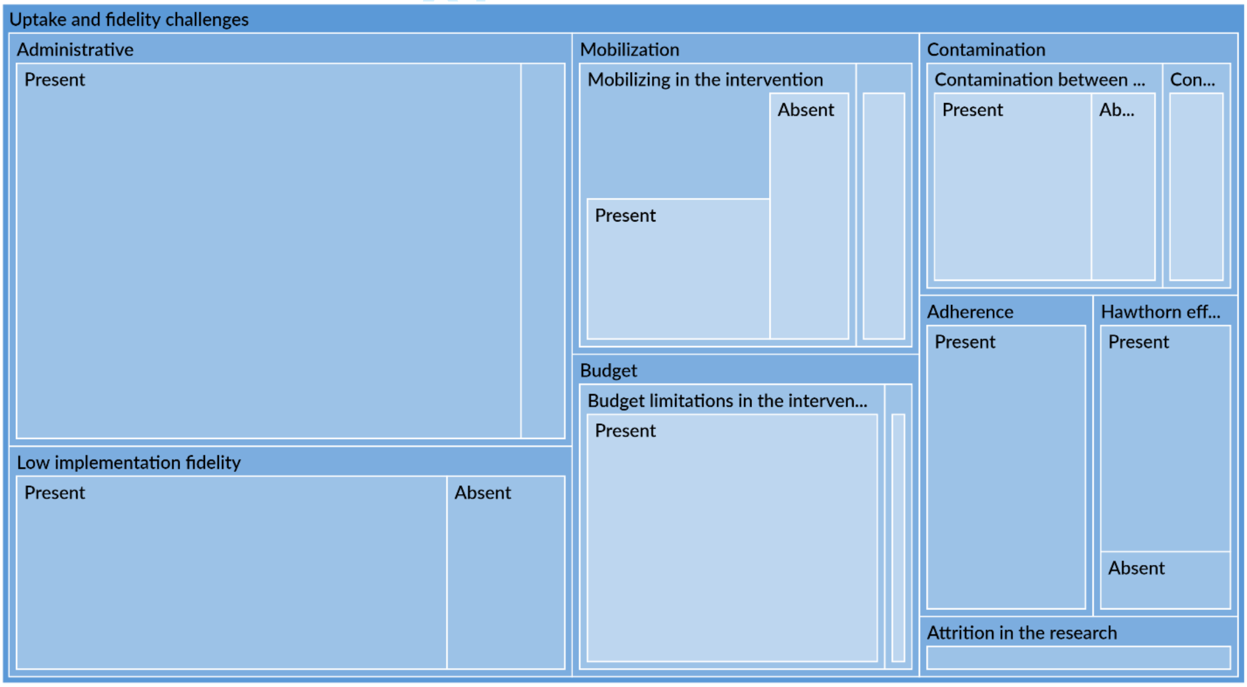

Supplementary Figure 101: Engagement in implementation autonomy of the intervention

Supplementary Table 20: Characteristic quotes of themes identified related to uptake and fidelity challenges for interventions that used community engagement in implementing the intervention.

**Primary paper**	**Paper from which citation comes**	**Sub-theme demonstrated**	**Quote**	**Page number**
Administrative challenges*				
Olken 2014	Febriany et al	Delays in funding	The most frequent complaint by FDs	14

For peer review only - http://bmjopen.bmj.com/site/about/guidelines.xhtml

	2011		regarding barriers to program implementation was the delays in aid disbursement.	
Mayumana et al 2017	Chimbutu et al 2011	Stakeholder communication problem	The NTPI"s programme, however, did not start in 2007 as planned… The major reason for this is on the implementation challenges between the two partners. The MOHSW preferred an accelerated approach while NORAD preferred an implementation framework to be designed by the Ifakara Health Institute (IHI).	16
Oyo-Ita 2020	Oyo-Ita 2020	Staffing and bureaucracy challenges	Low staffing of facilities constrained health staff from deploying the defaulters' register. Some complained that they had many registers other than the defaulters' register to fill.	18
Implementation fidelity				
Oyo-Ita 2020	Oyo-Ita 2020	Inconsistent implementation	The community engagement did not use town hall meetings as planned. Only one town hall meeting was held.	18
			The TRL training intervention was carried out as planned.	17
Okeke et al 2017	Okeke et al 2017	Expectations did not match realities on the ground	Contrary to the role of the states outlined in the MOU, policymakers in Enugu state reported playing no role in the implementation of the scheme. One suggested that the division of responsibility drawn up in the MOU did not align with the setup of health care in Nigeria given that the state government has responsibility for secondary care only.	50
Mayumana et al 2017	Olfasdottir et al 2014	Intervention did not function as expected	Firstly, the work load on staff is considerably higher than they are contracted for, which is not reflected in the remuneration; payments are delayed and overtime and eligible allowances are not always paid causing demotivation among staff.	7
		Intervention was implemented according to clear standards	The direct and transparent payment of funds, as well as more frequent contact with their managers was reported to enhance trust and improve the relationship between health workers and	64

For peer review only - http://bmjopen.bmj.com/site/about/guidelines.xhtml

			their managers.	
Budgetary constraints				
Okeke et al 2017	Okeke et al 2017	Pay was severely delayed	Some local government don't have the money to pay and they have not paid for a period of time. Most of them [midwives] have even finished their service and gone out without receiving any penny from the local government.	41
Mayumana et al 2017	Mayumana et al 2017	Activities were suspended	According to the qualitative data one of the obstacles for providing more frequent supervision visits was a lack of financial resources: We do supervision every quarter. Our plan is to do supervision monthly but due to limited budget we haven't yet done this.	6

*Challenges related to mobilization are not presented because this information came from a single paper making the identification of characteristic issues *across* papers impossible.

Sensitivity analysis for qualitative evidence

We carried out a sensitivity analysis in which we considered only the 17 qualitative studies associated with 12 impact evaluations that had a risk of bias assessment score of 20 or higher. Broadly, the themes that emerged from this analysis were consistent with those of the full analysis. We did not have enough qualitative evidence to conduct a sensitivity analysis by engagement type. An important caveat of this analysis is that nearly half of the papers rated as high "quality" were associated with three impact evaluations. The panel figures below provide comparison of the emerging themes in the full analysis versus the sensitivity analysis by comparing hierarchy charts related to barriers, facilitators, reasons for project success or failure, and uptake and fidelity challenges.

Barriers to immunisation

The most common barriers were broadly similar across the full analysis and the sensitivity analysis. *Behavioural, social and practical barriers faced by caregivers* were the most consistently reported barriers followed by *constraints in delivery of immunisation services* (Supplementary Figure 102). The most common barriers faced by caregivers were unavailability of immunisation services, financial constraints and social norms.

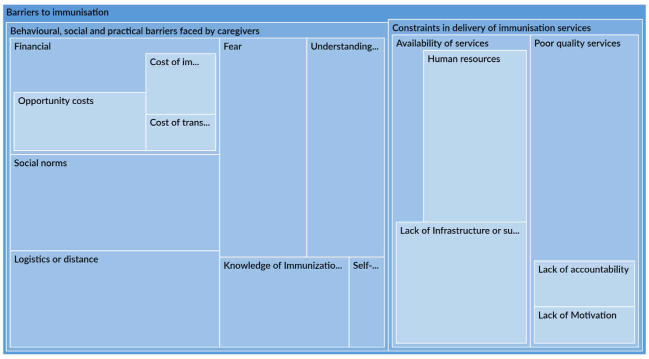

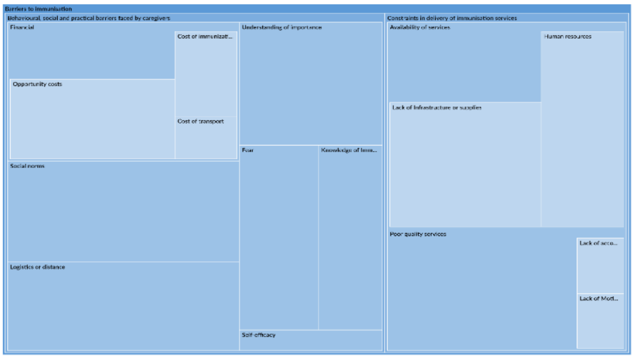

Supplementary Figure 102 (a): Full analysis *(b): Sensitivity analysis*

For peer review only - http://bmjopen.bmj.com/site/about/guidelines.xhtml

Facilitators of immunisation

The most common facilitators of immunisation were broadly similar across the full analysis and the sensitivity analysis. *Behavioural, social and practical factors faced by caregivers* were the most consistently reported facilitators of immunisation in a given context followed by good *delivery of immunisation services* (Supplementray Figure 103). The most consistently reported facilitators were availability of immunisation services, enabling social norms and caregivers' understanding of importance of immunisation.

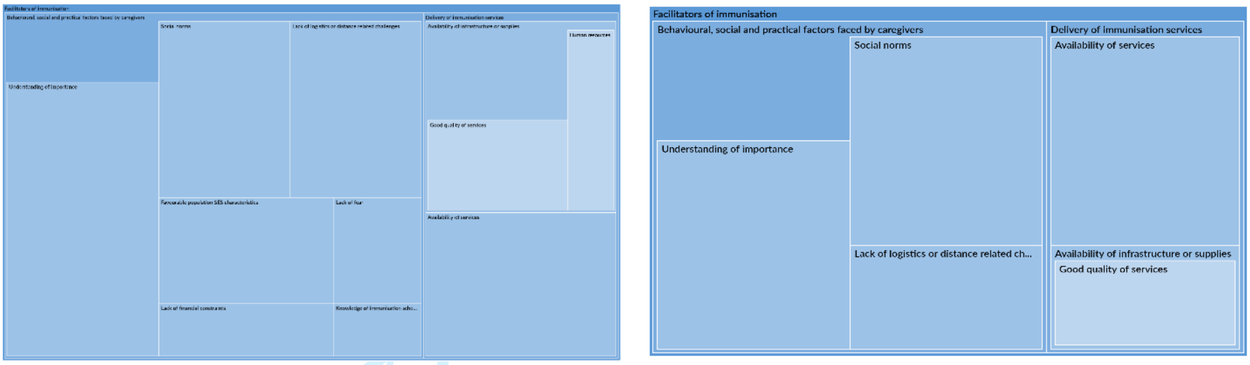

Supplementary Figure 103 (a): Full analysis *(b): Sensitivity analysis*

Reasons for intervention success

The most consistently reported reasons for success were broadly similar across the full analysis and the sensitivity analysis. Success was consistently attributed to *intervention features* including leadership and supportive supervision, community engagement, health worker training, incentives and customisation to local context. Though not as common, success was also attributed to *existing or changing favourable characteristics* within a given context such as positive participant views, enabling social norms and availability of and access to good quality health services. Given that a very small subset of papers were included in the sensitivity analysis, a few themes that emerged in the full analysis were missing in the sensitivity analysis. Supplementary Figure 104 provides the hierarchy charts comparing the two analyses.

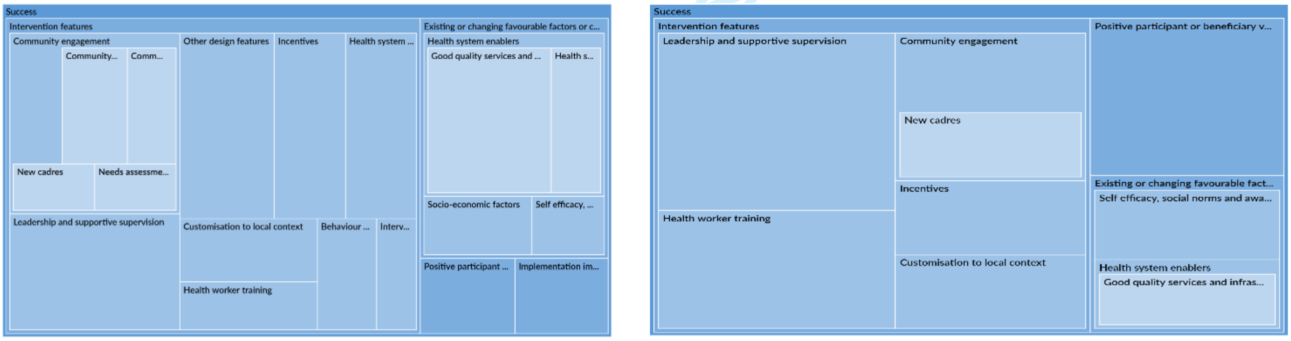

Supplementary Figure 104 (a): Full analysis *(b): Sensitivity analysis*

Reasons for intervention failure

The most consistently reported reasons for failure were broadly similar across the full analysis and the sensitivity analysis. The most consistent reasons for failure were attributed to *accounting for contextual constraints* and inadequate *intervention features*. Social norms, health system related issues, political or civil unrest and high baseline coverage were some of the most common contextual reasons for failure. Among the intervention features, inadequate duration, frequency or exposure to the intervention were the most notable reasons for failure. Though not as prominently reported as compared to the full analysis, *implementation challenges* were also noted to have caused intervention failure in the sensitivity analysis. Among these,

For peer review only - http://bmjopen.bmj.com/site/about/guidelines.xhtml

disruption due to inadequate implementation instructions and competing priorities of health workers were noted as some of the prevalent challenges.

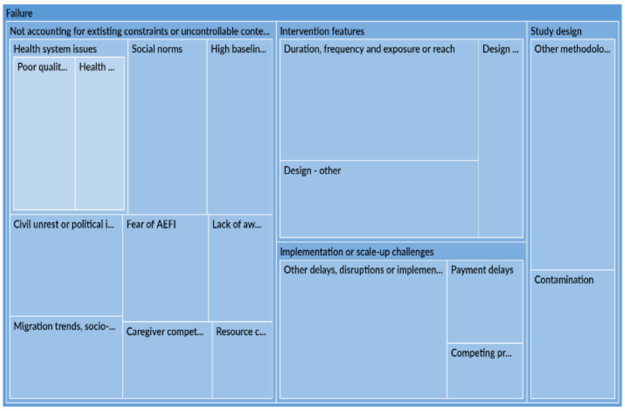

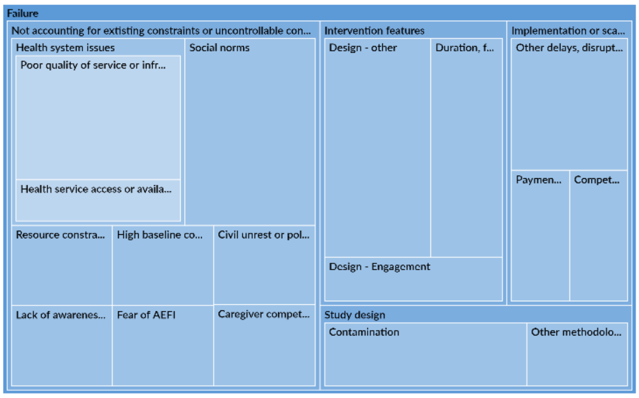

Supplementary Figure 105 (a): Full analysis *(b): Sensitivity analysis*

Uptake and fidelity challenges

The most consistently reported uptake and fidelity issues were broadly similar across the full analysis and the sensitivity analysis. The most common challenges consistently reported challenges were related to administration, mobilization, fidelity, contamination and budget constraints. Administrative challenges were cited consistently. Similar to the full analysis, the primary most consistent issue was that realities on the ground forced changes to the intervention.

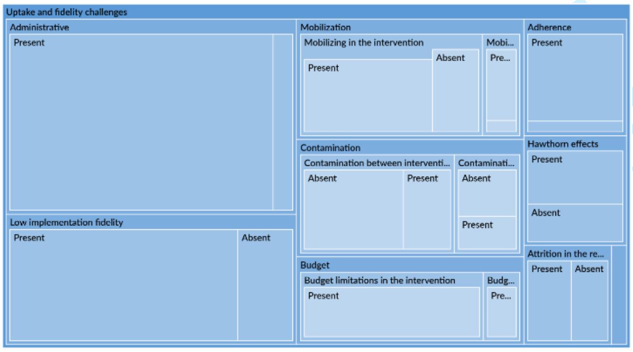

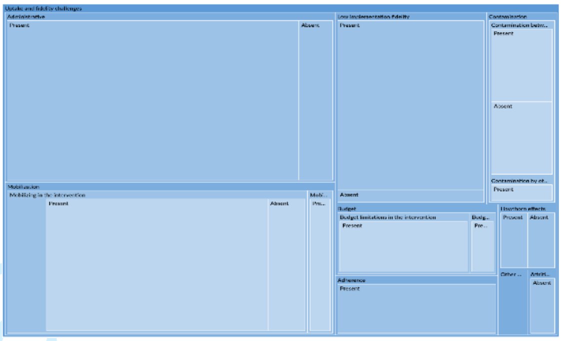

Supplementary Figure 106 (a): Full analysis *(b): Sensitivity analysis*

For peer review only - http://bmjopen.bmj.com/site/about/guidelines.xhtml

Appendix 15: Synthesis of cost evidence

This section reports on the results of the analysis of the inventory of included studies (see Table 21) and of the estimates of the non-vaccine cost per dose of interventions to increase absolute immunisation coverage by one percent (see Table 22).

Characteristics of included studies

Twenty-two of the evaluations selected for inclusion in the systematic review reported some type of cost analysis. Of these, 18 used an experimental design and the remaining 4 used quasi-experimental methods to identify the impact of treatment.

Of the 22 studies: 20 studies reported a total cost of the intervention; 12 included a cost-effectiveness analysis; three included cost-efficiency analysis; two studies included a cost-benefit analysis and two analysed cost per quality-adjusted life year (QALY).

Table 21: Characteristics of studies including any type of cost analysis.

					Type of Cost Analysis			
Author, Date	Country	Evaluation design	Intervention exposure (months)	Total Cost	Cost-efficiency Analysis	Cost-effectiveness Analysis	Cost-Benefit Analysis	Cost per QALY or DALY
Andersson, 2013	Pakistan	Experimental	8	✓				
Banerjee, 2010	India	Experimental	18	✓		✓		
Banerjee, 2020	India	Experimental	14	✓		✓		
Björkman, 2009	Uganda	Experimental	0.17	✓				
Borkum, 2020	India	Experimental	12	✓	✓	✓		
Demilew, 2020	Ethiopia	Experimental	17	✓				
Gurley, 2020	India	Experimental	11	✓		✓		
Johri, 2009	India	Experimental	3	✓				
Manoj, 2020	India	Experimental	4	✓				
Modi, 2019	India	Experimental	12	✓	✓	✓	✓	✓
More, 2017	India	Experimental	24	✓				
Morris, 2020	Honduras	Experimental	24	✓		✓		
Nagar, 2020	India	Experimental	20	✓		✓		
Olken, 2014	Indonesia	Experimental	24	✓		✓		
Oyo-Ita, 2020	Nigeria	Experimental	18	✓		✓		
Pramanik, 2018	India	Experimental	13	✓				
Seth, 2010	India	Experimental	9.7			✓		
Webster, 2019	Uganda	Experimental	12	✓			✓	
Admassie, 2017	Ethiopia	Quasi-experimental	48		✓			
Carnell, 2014	Ethiopia	Quasi-experimental	48	✓				
Findley, 2004	Nigeria	Quasi-experimental	24	✓		✓		
Saggurti, 2009	India	Quasi-experimental	2	✓		✓		✓

By country. Fourteen of the community participation interventions to improve child immunisation studies were conducted in Asian countries: of these, 12 studies took place in India; and one each in Pakistan and Indonesia. One study was conducted in a Central American country, Honduras and seven studies were conducted in African countries: three in Ethiopia; and two each in Nigeria and Uganda

For peer review only - http://bmjopen.bmj.com/site/about/guidelines.xhtml

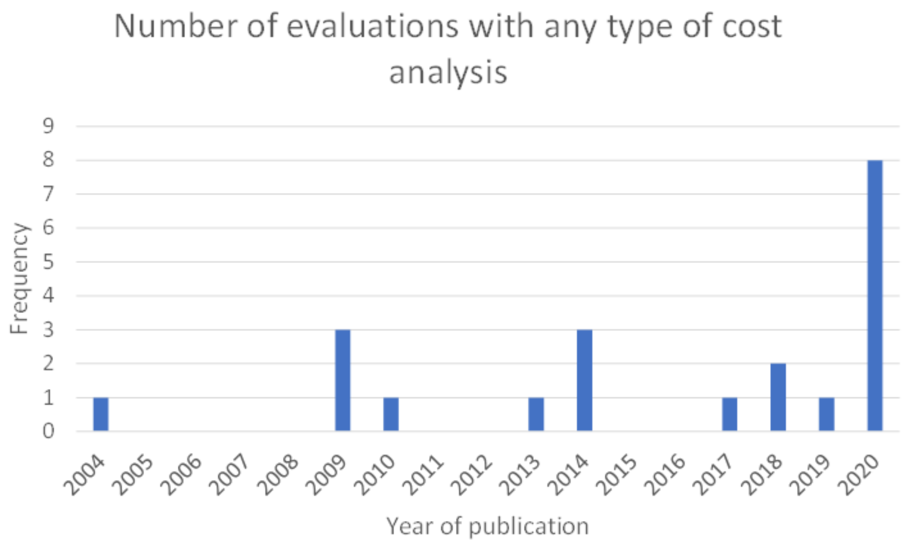

Supplemenary Figure 107: Number of evaluations with any type of cost analysis
The production of cost evidence in published evaluations of community participation interventions to improve child immunisation in low- and middle-income countries was uneven over the years. The first recorded evaluation with any cost analysis was published in 2004. Between 2009 and 2019, no more than three of the identified evaluations included any type of cost analysis in any given year. In 2020, the largest number of evaluations, eight, were published in a single year. The eight evaluations published in 2020 were required to generate cost analysis in partial fulfillment of the grant requirements in 3ie's Innovations in Increasing Immunisation Evidence Programme. Funding for these studies was provided by the Bill and Melinda Gates Foundation.

Non-vaccine cost per dose of intervention to increase immunisation coverage by one percent

Of the 22 included studies reporting cost and impact information, we estimate the non-vaccine cost per dose of intervention to increase immunisation coverage by one percent for 14. Because several studies report results by treatment arm, we report results for a total of 17 treatment arms. These results are summarised in detail in Table 22.

Three studies and one treatment arm were dropped from the analysis. In the case of Johri, 2020 this was because the proportion of children vaccinated was not reported as an outcome of this evaluation. Morris 2020 was dropped because it is unclear how to combine the PRAF II costs reported in a separate paper with intervention as described in Moris, 2020, in addition, the evaluation found a null result on the immunisation outcomes. Findley, 2013 was dropped because the reported cost information is insufficient for generating a total cost estimate or cost per vaccine dose, and "immunisation status" is not clearly defined. In the case of Banerjee, 2020, no coverage change information was reported for the Gossip seeds treatment arm which was dropped from the analysis as a result.

Six studies, Findley, 2004; Pramanik, 2018; Webster, 2019; Oyo-Ita, 2020, Modi, 2019; and Demilew, 2020 reported negative or null results and were excluded from the analysis of non-vaccine cost per dose of intervention to increase immunisation coverage by one percent.

The median non-vaccine cost per dose of intervention to increase immunisation coverage by one percent for the 14 estimates was US $3.68 (all costs are reported in 2019 US dollars). In comparison, the average was US $ 46.60 which is driven by three observations, Olken 2014 (US $23.10); Carnell, 2014 (US $641.08); and Gurley, 2020 (US $29.98).

For peer review only - http://bmjopen.bmj.com/site/about/guidelines.xhtml

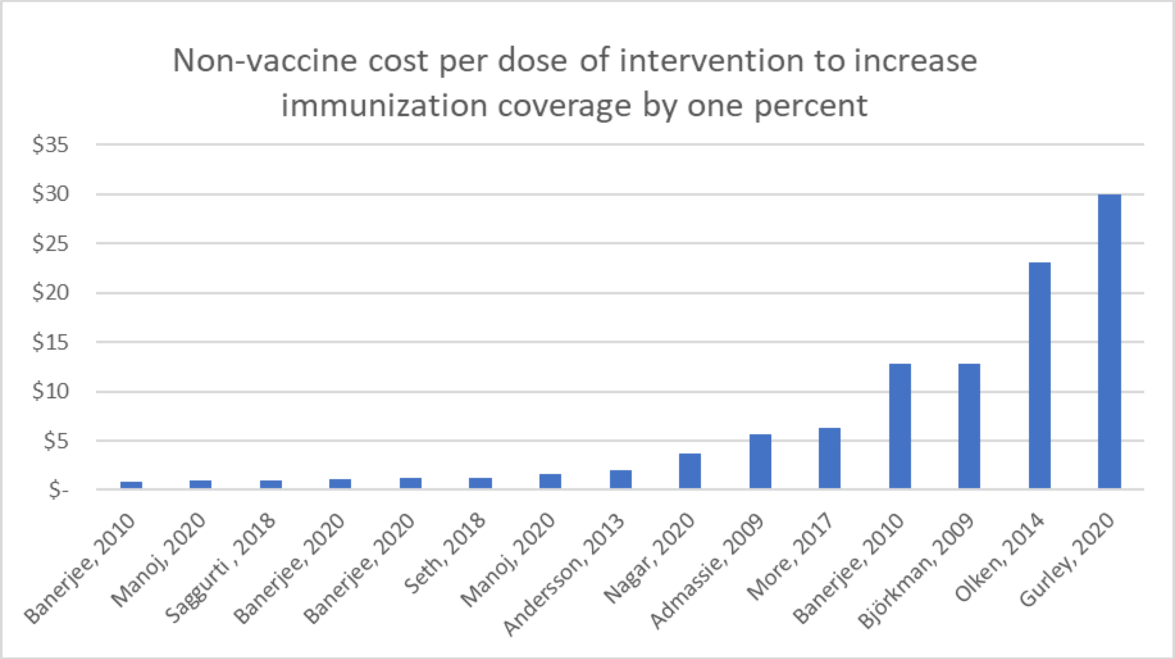

Supplementary Figure 108: Non-vaccine cost per dose of intervention to increase immunisation coverage by one percent

Supplementray Figure 108 illustrates that the range of estimates varied from a minimum of US $0.88 to a maximum of US $29.98. The lowest cost per vaccine dose was observed in Banerjee, 2010 for the "camps with incentives" intervention at US $0.88 per vaccine dose to increase immunisation coverage by one percent. The highest cost was observed in Carnell, 2014 at $641.08 - where the five-year intervention included system-building activities such as building and staffing a health post. In addition, the transparency of cost reporting in Carnell is very low, which further contributes to uncertainty in this cost estimate Indeed, Munk et. Al (2019) dropped this study from their systematic review of costs because they judged the available cost information to be insufficient to calculate the cost per vaccine dose. Since Carnell is an outlier observation, we take the intervention with the second-highest cost per incremental outcome which was reported in Gurley, 2020 at US $29.98.

For peer review only - http://bmjopen.bmj.com/site/about/guidelines.xhtml

Supplementary Table 22: Detailed estimates of non-vaccine intervention cost per dose and absolute coverage change

Author, Date	Country	Total Cost USD $2019	Treatment	Outcome	Vaccines Reported (list)	Number of doses	Non-vaccine intervention cost per vaccine dose ($ 2019 USD)	Absolute coverage change (Intervention - Control)	Non-vaccine cost per dose of intervention to increase immunization coverage by one percent	Impact notes	Cost notes	Immunization notes. Basel
Oyo-Ita, 2020	Nigeria	$15,158	Training of traditional and religious leaders; health workers; and community mobilization to improve vaccination	Proportion of children with up-to-date vaccination	Pentavalent3, Measles	5 per protocol	$4.59	Null effect	negative / null result	Unadjusted proportion, endline (intervention - control)	Incremental to no intervention control group, excludes healthcare system-building costs	46% is baseline up to date vaccination in intervention, 48% control
Demilew, 2020	Ethiopia	$224,269	A system of tracking children's immunisation status using a poster and stamp system	Probability of receiving all 3 doses of DPT/PCV	DPT - Diphtheria, pertussis, and tetanus / PCV - Pneumococcal Conjugate Vaccine	6 per protocol	$23	Null effect	negative / null result	Unadjusted proportion, endline (intervention - control)	Incremental to no intervention control group, excludes healthcare system-building costs	Baseline immunization not reported
Seth, 2018	India	$2,178	Reminders + Incentives	Proportion of total number of immunizations received by a child divided by the total number of immunizations required	BCG, DPT, H influenzae type B, Polio, Hepatitis B, Rotavirus, Measles	3.21 per child, 574 doses	$4	3.1	$1.252	Unadjusted proportion, (end of study - baseline)	Incremental to no intervention control group, excludes healthcare system-building costs	40% is baseline immunization coverage in treatment and 39% in control
Banerjee, 2010	India	$24,599.27	Camps without incentives	Proportion of children fully immunised	Full immunisation is one dose of BCG vaccine, three doses of DPT (diphtheria-pertussis,	2.35 per child	$153.44	12.0	$12.79	Unadjusted proportion, endline (intervention - control)	Incremental to no intervention control group, excludes healthcare system-building costs	2% is baseline full immunsation rate in the study area
Banerjee, 2010		$12,408.09	Camps with incentives	Proportion of children fully immunised	tetanus) vaccine, three doses of oral polio vaccine, and one dose of measles vaccine.	2.85 per child	$29.22	33.0	$0.89	Unadjusted proportion, endline (intervention - control)	Incremental to no intervention control group, excludes healthcare system-building costs	2% is baseline full immunsation rate in the study area
Johri, 2009	India	-	-		-	-	-	-	-	-	-	-
Andersson, 2013	Pakistan	$70,981.46	Three structured community discussions of of local vaccination rates; costs and benefits of childhood vaccination; and local action plans.	Uptake of full DPT-3 and Measles vaccination in children 12-23 months	DPT-3, Measles	5 per protocol	$50.16	24	$2.07	Unadjusted proportion, endline (intervention - control)	Incremental to no intervention control group, excludes healthcare system-building costs	Baseline for DPT3 is 0.51 in treatment and 0.45 in control
Findley, 2004	Nigeria	-	-		-	-	-	-	negative / null result	-	-	-
Morris, 2020	Honduras	-	-		-	-	-	-	-	-	-	
Pramanik, 2018	India	$101,636.61	Community-based learning cycles where a community identifies a problem, makes an action plan, takes action and learns from the process	Full immunization take-up	1 BCG dose; 3 doses OPV; 3 doses DPT or Pentavalent vaccine, and one measles vaccine	8 per child 12-23 months	$17.20	RC = -1%	negative / null result	Unadjusted proportion, endline (intervention - control)	Incremental to no intervention control group, excludes healthcare system-building costs	75% is baseline full immunization coverage in treatment and 76% in control

For peer review only

234

Author, Date	Country	Total Cost USD $2019	Treatment	Outcome	Vaccines Reported (list)	Number of doses	Non-vaccine intervention cost per vaccine dose ($ 2019 USD)	Absolute coverage change (Intervention - Control)	Non-vaccine cost per dose of intervention to increase immunization coverage by one percent	Impact notes	Cost notes	Immunization notes
Saggurti , 2018	India	$39,597.45	Participatory behavior communcation on maternal, neonatal, child health and promoting collectivization processes through women's self-help groups (SHGs)	Age-appropriate immunization among children under one year	9 doses - 1 BCG, 1 OPV, 3 OPV, 1,2,3,; 3 DPT, 1 Measles	9 per child under 1 year	$9.37	9	$1.03	Quasi-experimental. DID estimate. Intervention and control proportions were adjusted for baseline covariates	Incremental to no intervention control group, excludes healthcare system-building costs	Baseline full immunization was 42.9% in treatment and 52.5% in control
Admassie, 2009	Ethiopia	$1,008,991.46	Received Measles vaccine for children 9 to 11 months	Proportion of children 12–60 months old vaccinated against major diseases. tuberculosis, diphtheria, whooping cough (pertussis), tetanus, polio and measles	8 doses: tuberculosis, polio, diphtheria–pertussis–tetanus, and measles	8 per child 12 - 60 months	$56.19	9.9	$5.68	Quasi-experimental. Intervention and control proportions were adjusted for baseline covariates	Incremental cost of running Health Services Extension Programme (HSEP) in the treatment areas, includes system-building cost to construct and staff a health post	Baseline immunization not reported
More, 2017	India	$213,420.20	Community resource centres	Proportion of children aged 12–23 months fully immunised	BCG, diphtheria, pertussis, and tetanus [three doses], polio, hepatitis B virus [three doses], and measles	8 per protocol	$37.02	5.8	$6.34	Unadjusted proportion endline, (intervention - control). Change was positive but not statistically significantly different from zero	Incremental to no intervention control group,excludes healthcare system-building costs	65% is baseline full immunization coverage in treatment and 63% in control
Olken, 2014	Indonesia	$12,264,782.40	Village-level block grants for maternal and child health and education that incorporated relative performance incentives	Proportion of children under two years of age with BCG vaccine	1 dose, BCG	1.00	$36.97	1.60	$23.11	Adjusted average standardized effect (absolute coverage change) was positive but not statistically significantly different from zero.	Incremental cost of preference-weighted incentive block grant program for vaccines relative to the cost of the program of community block grants without incentives	65.3% is baseline full immunization coverage
Borkum, 2020	India	$39,571.93	A program that offers non-monetary incentives for teamwork and goal-setting activities to improve maternal and child health at health subscenters in India	Proportion of children ages 6-11 months who recieved DPT-3 by the age of 6 months	DPT-3	585	$22.54	5.4	$4.17	Adjusted proportion (intervention - control). Difference is positive but not significantly different from zero	Incremental to no intervention control group, excludes healthcare system-building costs	45.5% is baseline mean for a child fully immunized except measles in the treatment group, and 39.9% in the control group
Manoj, 2020	India	$61,344.88	Information treatment	Full immunization of children 12-23 months	8 doses: BCG, measles, and 3 doses each for Polio and DPT	8 per protocol	$14.10	8.8	$1.60	Unadjusted increase in proportion immunised in intervention relative to control. We report separately by treatment arm because there is corresponding cost info.	Incremental to no intervention control group, excludes healthcare system-building costs	Baseline immunization not reported
Manoj, 2020	India	$61,344.88	Information + Facilitation treatment	Full immunization of children 12-23 months	8 doses: BCG, measles, and 3 doses each for Polio and DPT	8 per protocol	$12.63	13.4	$0.94	Unadjusted increase in proportion immunised in intervention relative to control. We report separately by treatment arm because there is corresponding cost info.	Incremental to no intervention control group, excludes healthcare system-building costs	Baseline immunization not reported
Björkman, 2009	Uganda	$182,003.09	Community-based monitoring of public primary health care providers	Proportion of children ages 13-24 months who received required doses of DPT	3 doses: DPT	3	$76.83	6.0	$12.81	Adjusted percent increase in 1-year old immunisation rate	Back of the envelope total cost, incremental intervention cost relaitve to control	Baseline immunization not reported
Banerjee, 2020	India	$342,595.94	High slope incentives			12 per pro	$13.81	11	$1.21	Adjusted percent change in fully immunised	Incremental to no intervention control group, excludes healthcare system-building costs	20.8% - 50.05% is the range of baseline means, depending on the district for a child fully vaccinated
		$367,078.25	Low slope incentives			12 per pro	$13.85	12	$1.14	Adjusted percent change in fully immunised	Incremental to no intervention control group, excludes healthcare system-building costs	20.8% - 50.05% is the range of baseline means, depending on the district for a child fully vaccinated
		$78,072.73	Gossip seeds	Proportion of children under 12 months who received 1 dose of BCG, 4 doses of the oral polio vaccine, 3 doses of penta, 3 doses of rotavirus and 1 dose of measles	BCG, penta doses 1–3 and measles-1	12 per pro	NA	-	-	No coverage change reported for the Gossip seeds treatment.	Incremental to no intervention control group, excludes healthcare system-building costs	20.8% - 50.05% is the range of baseline means, depending on the district for a child fully vaccinated
Nagar, 2020	India	$275,702.59	Tracking system: The Khushi Baby platform, a wearable digital health record (pendant) and with patient-specific, dialect-specific voice reminders	Full and timely Immunization as verified by MAMTA card for children 12-23 months	8 doses: Polio 1-3, BCG, Pentavalent 1-3, Measles	6 per protocol	$41.17	11.2	$3.68	Unadjusted increase in proportion immunised in intervention relative to control	Incremental to no intervention control group, excludes healthcare system-building costs	25.4% is intervention baseline, full immunisation without birth doses and 22.5% is control
Gurley, 2020	India	$175,603.81	PATH - community-led videos	Proportion of children 6 to 17 months of age who had received all age-appropriate vaccines in India's vaccine schedule	13 doses: Birth doses (OPV, BCG, HepB); DPT1-3; HepB1-3; MR; OPV1-3	13 per protocol	$26.39	0.88	$29.98	Unadjusted proportion, endline (intervention - control). Difference is positive but not signficantly different from zero	Incremental to no intervention control group, excludes healthcare system-building costs	51.94% in treatment and 53.85 in control for full immunization
Carnell , 2014	Ethiopia	$27,316,577.57	Three pillars approach Eshe project strengthening health systems, improving health workers' performance, and engaging the community	Percentage of children aged 12-23 months, who received DPT3 vaccination, or Measles	2 doses: DPT3, Measles	2 per protocol	$5,449.19	8.5	$641.08	Coverage change is the average for DPT3 and Measles. Cost data very low transparency, making cost estimate unreliable	System-building' activities are evident in this intervention. However, cost data have very low transparency. To calculate total cost for treating vaccinated	49.3% in treatment and 33.87% in control for DPT3
Modi, 2019	India	$160,881.37	A mobile-phone app to assist ASHAs and health providers to schedule and track home health visits and follow-on care	Proportion of infants (6-8 months) who received all three doses of DPT or Pentavalent vaccine	3 doses: DPT or Pentavalent vaccine	3 per protocol		null result	negative / null result	Unadjusted proportion, endline (intervention - control)	Incremental to no intervention control group, excludes healthcare system-building costs	71.1% in treatment and 75.5% in control for DPT3
Webster, 2019	Uganda	$224,224.18	mReach data platform and the 'Fifth Child', a community engagement strategy to increase immunization	Proportion of children ages 12-23 months with DPT3 and MCV combined immunization coverage	2 doses: DPT3, Measles	2 per protocol	$204.63	null result	negative / null result	Unadjusted proportion, endline (intervention - control)	Incremental to no intervention control group, excludes healthcare system-building costs	[illegible] 80.3 % in control for DPT3

For peer review only - http://bmjopen.bmj.com/site/about/guidelines.xhtml

For peer review only

For peer review only - http://bmjopen.bmj.com/site/about/guidelines.xhtml

For peer review only

237

Björkman, 2009	Uganda	$182,003.09	Community-based monitoring of public primary health care providers	Proportion of children ages 13-24 months who received required doses of DPT	3 doses: DPT	3	$76.83	6.0	$12.81	Adjusted percent increase in 1-year old immunisation rate	Back of the envelope total cost, incremental intervention cost relaitve to control	Baseline immunization not reported
Banerjee, 2020	India	$342,595.94	High slope incentives			12 per pro	$13.81	11	$1.21	Adjusted percent change in fully immunised	Incremental to no intervention control group, excludes healthcare system-building costs	20.8% - 50.05% is the range of baseline means, depending on the district for a child fully vaccinated
		$367,078.25	Low slope incentives	Proportion of children under 12 months who received 1 dose of BCG, 4 doses of the oral polio vaccine, 3 doses of penta, 3 doses of rotavirus and 1 dose of measles		12 per pro	$13.85	12	$1.14	Adjusted percent change in fully immunised	Incremental to no intervention control group, excludes healthcare system-building costs	20.8% - 50.05% is the range of baseline means, depending on the district for a child fully vaccinated
		$78,072.73	Gossip seeds		BCG, penta doses 1–3 and measles-1	12 per pro	NA	-	-	No coverage change reported for the Gossip seeds treatment.	Incremental to no intervention control group, excludes healthcare system-building costs	20.8% - 50.05% is the range of baseline means, depending on the district for a child fully vaccinated
Nagar, 2020	India	$275,702.59	Tracking system: The Khushi Baby platform, a wearable digital health record (pendant) and with patient-specific, dialect-specific voice reminders	Full and timely Immunization as verified by MAMTA card for children 12-23 months	8 doses: Polio 1-3, BCG, Pentavalent 1-3, Measles	6 per protocol	$41.17	11.2	$3.68	Unadjusted increase in proportion immunised in intervention relative to control	Incremental to no intervention control group, excludes healthcare system-building costs	25.4% is intervention baseline, full immunisation without birth doses and 22.5% is control
Gurley, 2020	India	$175,603.81	PATH - community-led videos	Proportion of children 6 to 17 months of age who had received all age-appropriate vaccines in India's vaccine schedule	13 doses: Brith doses (OPV, BCG, HepB); DPT1-3; HepB1-3; MR; OPV1-3	13 per protcol	$26.39	0.88	$29.98	Unadjusted proportion, endline (intervention - control). Difference is positive but not signficantly different from zero	Incremental to no intervention control group, excludes healthcare system-building costs	52.88% is baseline percentage of children in the State of Uttar Pradesh who received all necessary vaccines in 2012–2013
Carnell , 2014	Ethiopia	$27,316,577.57	Three pillars approach Eshe project: strengthening health systems, improving health workers' performance, and engaging the community	Percentage of children aged 12-23 months, who received DPT3 vaccination, or Measles	2 doses: DPT3, Measles	2 per protocol	$5,449.19	8.5	$641.08	Coverage change is the average for DPT3 and Measles. Cost data very low transparency, making cost estimate unreliable	System-building' activities are evident in this intervention. However, cost data hvae very low transparency. To calculate total cost for treating vaccinated	Baseline immunization not reported
Modi, 2019	India	$160,881.37	A mobile-phone app to assist ASHAs and health providers to schedule and track home health visits and follow-on care	Proportion of infants (6-8 months) who received all three doses of DPT or Pentavalent vaccine	3 doses: DPT or Pentavalent vaccine	3 per protocol		null result	negative / null result	Unadjusted proportion, endline (intervention - control)	Incremental to no intervention control group, excludes healthcare system-building costs	Baseline immunization not reported
Webster, 2019	Uganda	$224,224.18	mReach data platform and the 'Fifth Child', a community engagement strategy to increase immunisation	Proportion of children ages 12-23 months with DPT3 and MCV combined immunization coverage	2 doses: DPT3, Measles	2 per protocol	$204.63	null result	negative / null result	Unadjusted proportion, endline (intervention - control)	Incremental to no intervention control group, excludes healthcare system-building costs	Baseline immunization not reported

PRISMA 2020 Checklist

Section and Topic	Item #	Checklist item	Location where item is reported #page no
**TITLE**			
Title	1	Identify the report as a systematic review.	p.1
**ABSTRACT**			
Abstract	2	See the PRISMA 2020 for Abstracts checklist.	pp. 2-3
**INTRODUCTION**			
Rationale	3	Describe the rationale for the review in the context of existing knowledge.	pp. 4-5
Objectives	4	Provide an explicit statement of the objective(s) or question(s) the review addresses.	p. 4
**METHODS**			
Eligibility criteria	5	Specify the inclusion and exclusion criteria for the review and how studies were grouped for the syntheses.	pp. 5-6, appendix 1
Information sources	6	Specify all databases, registers, websites, organisations, reference lists and other sources searched or consulted to identify studies. Specify the date when each source was last searched or consulted.	pp. 5-6, appendix 3
Search strategy	7	Present the full search strategies for all databases, registers and websites, including any filters and limits used.	p. 5, appendix 3, 4
Selection process	8	Specify the methods used to decide whether a study met the inclusion criteria of the review, including how many reviewers screened each record and each report retrieved, whether they worked independently, and if applicable, details of automation tools used in the process.	p. 5
Data collection process	9	Specify the methods used to collect data from reports, including how many reviewers collected data from each report, whether they worked independently, any processes for obtaining or confirming data from study investigators, and if applicable, details of automation tools used in the process.	p. 6
Data items	10a	List and define all outcomes for which data were sought. Specify whether all results that were compatible with each outcome domain in each study were sought (e.g. for all measures, time points, analyses), and if not, the methods used to decide which results to collect.	p. 6, appendix 6
	10b	List and define all other variables for which data were sought (e.g. participant and intervention characteristics, funding sources). Describe any assumptions made about any missing or unclear information.	p. 6, appendix 6
Study risk of bias assessment	11	Specify the methods used to assess risk of bias in the included studies, including details of the tool(s) used, how many reviewers assessed each study and whether they worked independently, and if applicable, details of automation tools used in the process.	p. 7, appendix 7
Effect measures	12	Specify for each outcome the effect measure(s) (e.g. risk ratio, mean difference) used in the synthesis or presentation of results.	p. 7
Synthesis methods	13a	Describe the processes used to decide which studies were eligible for each synthesis (e.g. tabulating the study intervention characteristics and comparing against the planned groups for each synthesis (item #5)).	p. 7
	13b	Describe any methods required to prepare the data for presentation or synthesis, such as handling of missing summary statistics, or data conversions.	p. 7
	13c	Describe any methods used to tabulate or visually display results of individual studies and syntheses.	p. 7
	13d	Describe any methods used to synthesize results and provide a rationale for the choice(s). If meta-analysis was performed, describe the model(s), method(s) to identify the presence and extent of statistical heterogeneity, and software package(s) used.	p. 7
	13e	Describe any methods used to explore possible causes of heterogeneity among study results (e.g. subgroup analysis, meta-regression).	p. 7
	13f	Describe any sensitivity analyses conducted to assess robustness of the synthesized results.	p. 7

For peer review only

PRISMA 2020 Checklist

Section and Topic	Item #	Checklist item	Location where item is reported #page no
Reporting bias assessment	14	Describe any methods used to assess risk of bias due to missing results in a synthesis (arising from reporting biases).	p. 7
Certainty assessment	15	Describe any methods used to assess certainty (or confidence) in the body of evidence for an outcome.	NA
**RESULTS**			
Study selection	16a	Describe the results of the search and selection process, from the number of records identified in the search to the number of studies included in the review, ideally using a flow diagram.	p. 8, figure 1
	16b	Cite studies that might appear to meet the inclusion criteria, but which were excluded, and explain why they were excluded.	pp. 6, 8, appendix 4
Study characteristics	17	Cite each included study and present its characteristics.	p. 8, table 1
Risk of bias in studies	18	Present assessments of risk of bias for each included study.	p. 21, appendix 8
Results of individual studies	19	For all outcomes, present, for each study: (a) summary statistics for each group (where appropriate) and (b) an effect estimate and its precision (e.g. confidence/credible interval), ideally using structured tables or plots.	p. 8
Results of syntheses	20a	For each synthesis, briefly summarise the characteristics and risk of bias among contributing studies.	pp. 9, 21
	20b	Present results of all statistical syntheses conducted. If meta-analysis was done, present for each the summary estimate and its precision (e.g. confidence/credible interval) and measures of statistical heterogeneity. If comparing groups, describe the direction of the effect.	pp. 21-26, appendix 9-13
	20c	Present results of all investigations of possible causes of heterogeneity among study results.	pp. 21-26, appendix 9-13
	20d	Present results of all sensitivity analyses conducted to assess the robustness of the synthesized results.	pp. 21-26, appendix 9-13
Reporting biases	21	Present assessments of risk of bias due to missing results (arising from reporting biases) for each synthesis assessed.	pp. 21-26, appendix 9-13
Certainty of evidence	22	Present assessments of certainty (or confidence) in the body of evidence for each outcome assessed.	NA
**DISCUSSION**			
Discussion	23a	Provide a general interpretation of the results in the context of other evidence.	p. 26
	23b	Discuss any limitations of the evidence included in the review.	pp. 3, 26
	23c	Discuss any limitations of the review processes used.	p. 3
	23d	Discuss implications of the results for practice, policy, and future research.	p. 28
**OTHER INFORMATION**			

For peer review only

PRISMA 2020 Checklist

Section and Topic	Item #	Checklist item	Location where item is reported #page no
Registration and protocol	24a	Provide registration information for the review, including register name and registration number, or state that the review was not registered.	p. 3
	24b	Indicate where the review protocol can be accessed, or state that a protocol was not prepared.	p. 3
	24c	Describe and explain any amendments to information provided at registration or in the protocol.	Appendix 2
Support	25	Describe sources of financial or non-financial support for the review, and the role of the funders or sponsors in the review.	p. 29
Competing interests	26	Declare any competing interests of review authors.	p. 29
7Availability of data, code and other materials	27	Report which of the following are publicly available and where they can be found: template data collection forms; data extracted from included studies; data used for all analyses; analytic code; any other materials used in the review.	p. 29

From: Page MJ, McKenzie JE, Bossuyt PM, Boutron I, Hoffmann TC, Mulrow CD, et al. The PRISMA 2020 statement: an updated guideline for reporting systematic reviews. BMJ 2021;372:n71. doi: 10.1136/bmj.n71

For more information, visit: http://www.prisma-statement.org/

For peer review only
